# A bio-inspired synthesis of oxindoles by catalytic aerobic dual C–H functionalization of phenols†
†Electronic supplementary information (ESI) available: Synthetic procedures, complete characterization data. Crystallographic data for **Q3**, **SQ3**, **13**, **23** and **29**. CCDC 1406066-1406069 and 1421991 For ESI and crystallographic data in CIF or other electronic format see DOI: 10.1039/c5sc02395e


**DOI:** 10.1039/c5sc02395e

**Published:** 2015-10-06

**Authors:** Zheng Huang, Mohammad S. Askari, Kenneth Virgel N. Esguerra, Tian-Yang Dai, Ohhyeon Kwon, Xavier Ottenwaelder, Jean-Philip Lumb

**Affiliations:** a Department of Chemistry , McGill University , Montreal , QC H3A 0B8 , Canada . Email: jean-philip.lumb@mcgill.ca; b Department of Chemistry and Biochemistry , Concordia University , Montreal , QC H4B 1R6 , Canada . Email: dr.x@concordia.ca

## Abstract

Nitrogen-containing heterocycles are fundamentally important to the function of pharmaceuticals, agrochemicals and materials. Herein, we report a bio-inspired approach to the synthesis of oxindoles, which couples the energetic requirements of dehydrogenative C–N bond formation to the reduction of molecular oxygen (O_2_). Our method is inspired by the biosynthesis of melanin pigments (melanogenesis), but diverges from the biosynthetic polymerization. Mechanistic analysis reveals the involvement of Cu^II^-semiquinone radical intermediates, which enable dehydrogenative carbon–heteroatom bond formation that avoids a catechol/quinone redox couple. This mitagates the deleterious polarity reversal that results from phenolic dearomatization, and enables a high-yielding phenolic C–H functionalization under catalytic aerobic conditions. Our work highlights the broad synthetic utility and efficiency of forming C–N bonds *via* a catalytic aerobic dearomatization of phenols, which is currently an underdeveloped transformation.

## Abstract 2

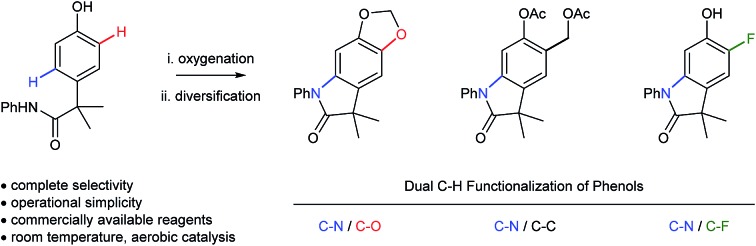
We report a bio-inspired approach to the synthesis of oxindoles, which couples the energetic requirements of dehydrogenative C–N bond formation to the reduction of oxygen.

## Introduction

**Scheme 1:**
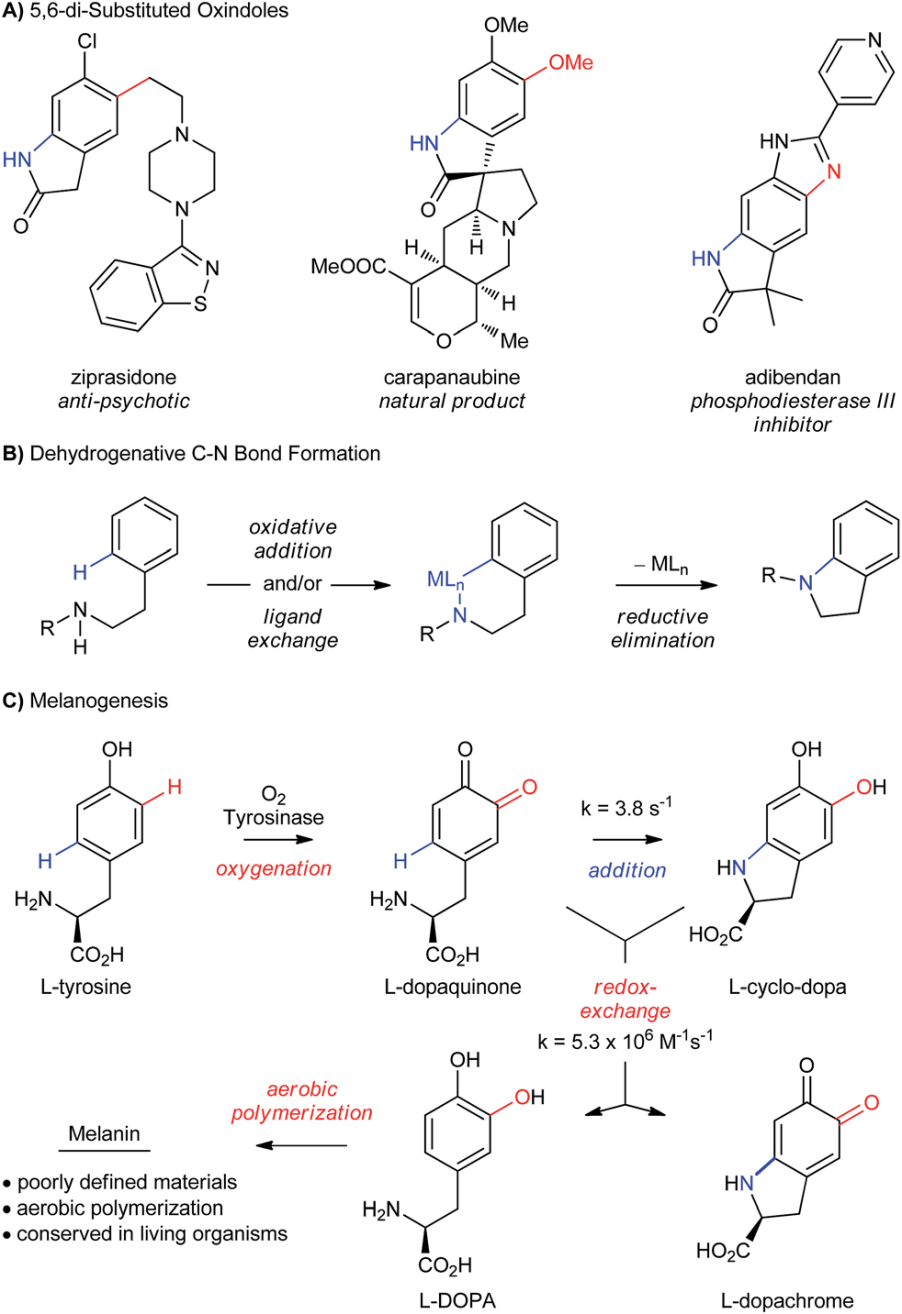
(A) Biologically active 4,5-disubstituted oxindoles. (B) Traditional C–N bond formation by cross-coupling. (C) Proposed mechanism for the biosynthesis of melanin pigments.

The selective oxidation of aromatic C–H bonds is critically important for the valorization of feedstock chemicals, since heteroatoms impart many desirable properties to small molecules and materials.^1^ Functional molecules generally contain more than one heteroatom–carbon bond (Scheme 1A), but the overwhelming majority of C–H oxidations functionalize just one bond at a time (Scheme 1B).^2^,^3^ Even with their increasing sophistication, C–H functionalizations used in sequence^4^ negatively impacts synthetic efficiency,^5^ which is compounded by increasing challenges of chemoselectivity. Thus, existing strategies to install the C–N bond of 5,6-di-substituted oxindoles (Scheme 1A) by dehydrogenative coupling would require selective functionalization of the product at C5 and C6, or would need to be chemoselective to existing functionalities at these positions.

An alternative approach to aromatic C–H functionalization occurs during the biosynthesis of melanin pigments,^6^ whereby the aromatic C–O and C–N bonds of l-cyclodopa are installed by a phenol-directed dual C–H functionalization (Scheme 1C). Such high levels of efficiency and simplicity for dehydrogenative heteroatom–carbon bond formation are attractive,2a,^7^–^9^ but melanin is a complex, irregular bio-material, whose heterogeneity reflects an inherent complication of installing heteroatoms by this mechanism. l-Cyclodopa is significantly more electron-rich than l-tyrosine, and its oxidation to l-dopachrome is facile. This can occur by autoxidation or by redox exchange with l-dopaquinone, which is competitive with C–N cyclization at millimolar concentrations (Scheme 1C).^10^ This affords a complex mixture of redox-active intermediates that is ultimately translated into the bio-material. In a laboratory setting, this contributes to poor selectivity, which is a well-known challenge when oxidizing phenols with O_2_.^11^ With the exception of Patureau's recent work^12^ and Hay's industrial aerobic polymerization of 2,6-dimethyl phenol,^13^ functionalizing the C–H bonds of phenols with heteroatoms requires stoichiometric quantities of an external oxidant11a,^14^,^15^ or oxidation of the heteroatom prior to coupling.^16^ And while the merits of developing catalytic aerobic alternatives are clear,^17^ progress has been slow in the case of phenols, due in part to their facile oxidation to phenoxyl radicals.^11^,^18^

**Scheme 2:**
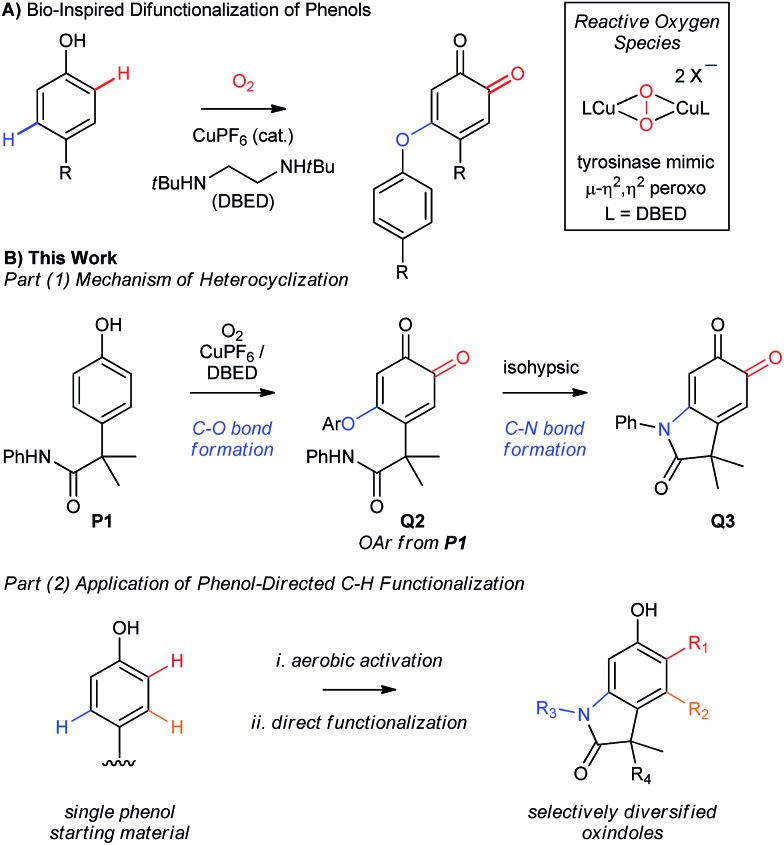
(A) Previous work from our groups. (B) This work: development of a catalytic aerobic functionalization of phenols.

The potential efficiency of a controlled “melanogenic” functionalization inspired one of our groups to develop a catalytic aerobic transformation that uses a small-molecule mimic of the enzyme tyrosinase (Scheme 2A).^19^ Tyrosinase is a type-III Cu-enzyme responsible for triggering melanogenesis.11b,^20^ It avoids radical-based oxidations of phenols by confining O_2_-activation and oxygen atom transfer to the inner coordination sphere of its dinuclear Cu active site. This is a well-accepted strategy for avoiding radical oxidations,11c,^18^,^21^ but it remains difficult to implement in the absence of the protein matrix.11*b* Inspired by the work of Stack^22^ and others,^23^ we developed conditions for the selective *ortho*-oxygenation of phenols that employ catalytic amounts of [Cu^I^(CH_3_CN)_4_](PF_6_) (abbreviated CuPF_6_), *N*,*N*′-di-*tert*-butylethylenediamine (DBED) and O_2_ at room temperature.^19^,^24^,^25^ Unlike the enzyme, however, which is selective for *ortho*-oxygenation, our conditions return coupled *ortho*-quinones that have undergone an additional C–H bond oxidation. This highlights important differences between the mechanism of our catalytic transformation and the mechanism of the enzyme, which we explore in the present work.

In Part 1 of this manuscript, we demonstrate that the heterocyclization of phenol **P1** to oxindoloquinone **Q3** occurs by a mechanism of homo-coupling and substitution, such that C–N bond formation is isohypsic5d–f (Scheme 2B). This is distinct from the commonly accepted mechanism of melanogenesis (Scheme 1C),6*a* and it provides important benefits to the efficiency and selectivity of heterocyclization by circumventing redox exchange. In Part 2, we apply our dual C–H functionalization to a synthesis of oxindoles, which highlights the versatility of activating phenols as their *ortho*-quinones.^26^ Unlike traditional cross-coupling reactions, which afford stable products following a single C–N coupling, the *ortho*-quinone obtained by our transformation is an activated precursor to the aromatic heterocycle. *ortho*-Quinones participate in a range of complexity-generating transformations,6a,^26^ allowing us to rapidly diversify the oxindole product from a single phenol starting material. This strategy is unique amongst dehydrogenative couplings, in that the energy stored in O_2_ is not only used to install one aromatic C–N bond, but also creates an activated product that is readily amenable to further functionalization. While this is a general feature of reactions that de-aromatize phenols,14i,^27^ our case marks a rare example where de-aromatization is conducted by aerobic catalysis.^17^,^28^

## Results and discussion

### Part 1. Mechanistic investigation

#### Reaction optimization and control experiments

**Scheme 3:**
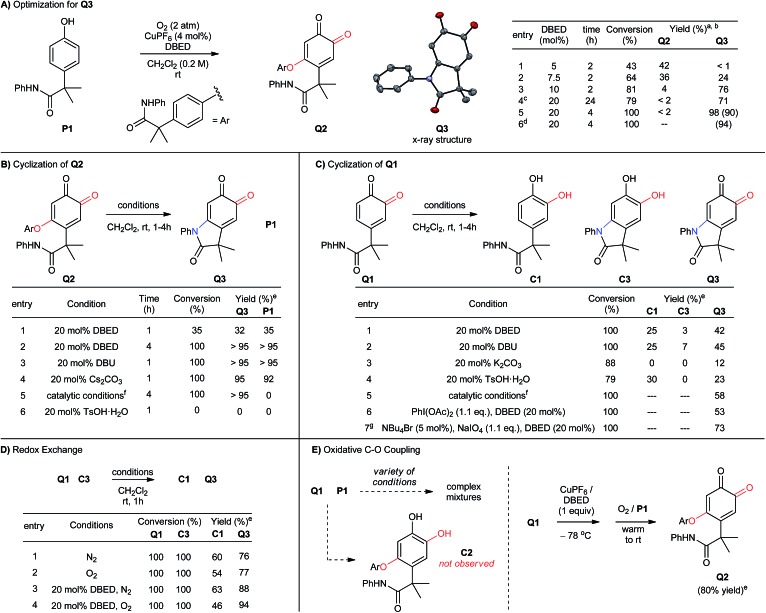
(A-E) Optimization of reaction conditions and control experiments. (a) Reactions performed with 0.5 mmol of **P1**. Work-up: 10% aqueous NaHSO_4_. (b) Product yield determined by ^1^H-NMR using hexamethylbenzene as an internal standard. Isolated yield reported in parenthesis. (c) Reaction performed under open-flask conditions. (d) Reaction performed using 5 g of **P1**. (e) Reactions were performed with 0.1 mmol of starting material, and analyzed by ^1^H-NMR following acidic work-up using hexamethylbenzene as an internal standard. (f) Catalytic conditions: [Cu(MeCN)_4_](PF_6_) (4 mol%), DBED (20 mol%), O_2_ (2 atm). (g) Reaction performed in a biphasic mixture of CH_2_Cl_2_ (10 mL) and water (2 mL).

When we examined the oxidative functionalization of acetanilide **P1** under our standard reaction conditions,^19^ we discovered a surprising mixture of O-coupled *ortho*-quinone **Q2** and oxindoloquinone **Q3** (Scheme 3A). The ratio of these products is sensitive to the quantity of DBED relative to CuPF_6_, so that a [DBED]/[Cu] ratio of 5/4 is completely selective for **Q2**, 7.5/4 affords a mixture and 10/4 favours **Q3** after 2 h (entries 1–3). This dependence on DBED suggests a base-promoted cyclization of **Q2** as a key step in the formation of **Q3** (see below), which is surprising since the conventional mechanism of melanogenesis does not involve phenolic C–O coupling prior to C–N coupling. While open-flask conditions are tolerated (entry 4), incomplete conversion after prolonged reaction times led us to use an overpressure of 1 atm of pure O_2_ for our optimized conditions. Thus, oxygenation of **P1** for 4h at room temperature in the presence of 4 mol% CuPF_6_ and 20 mol% DBED leads to a 90% yield of isolated oxindoloquinone **Q3** at complete conversion (entry 5). Reaction efficiency is maintained on 5 g scale, wherein **Q3** is isolated in 94% yield (entry 6).

The cyclization of **Q2** can be promoted by a variety of bases and cleanly returns **Q3** and **P1** (Scheme 3B, entries 1–4).^29^ Oxindoloquinone **Q3** is the only observed product if **Q2** is re-subjected to the standard catalytic conditions (entry 5), demonstrating that **Q2** is a competent reaction intermediate in the transformation of **P1** into **Q3**, and that upon its release from **Q2**, **P1** can re-enter the catalytic cycle for conversion to **Q3**. Cyclization of **Q2** to **Q3** is not promoted by acid (entry 6), suggesting that **Q2** does not cyclize to an appreciable extent during the acidic work-up.

C–N bond formation by substitution is significantly more efficient than intramolecular cyclization of the acetanilide within *ortho*-quinone **Q1** (Scheme 3C). To evaluate this transformation, we synthesized **Q1** from **P1** by using Pettus' *ortho*-oxygenation method with IBX.16*f* In the absence of an oxidant, exposure of **Q1** to 20 mol% DBED affords a 42% yield of **Q3**, along with a 25% yield of **C1** and trace amounts (<5%) of **C3** at complete conversion of **Q1** (entry 1). Selectivity for **Q3** improves if the cyclization is performed under oxidizing conditions (entries 2–4), but never as cleanly as catalytic conditions directly from **P1** or base-promoted cyclization of **Q2**. The results of entry 1 are consistent with a redox exchange between **C3** and **Q1**, whose feasibility was confirmed independently by mixing equimolar amounts of **Q1** and **C3** under a variety of conditions (Scheme 3D, entries 1–4). In each case, redox exchange is accompanied by a significant loss of mass balance (up to 69%), making the **Q1**-to-**C3**-to-**Q3** pathway inconsistent with the high selectivity observed under our catalytic conditions.

The cyclization of **Q2** is said to be isohypsic,5d–f in that additional oxidation is not required to arrive at target quinone **Q3**. This avoids the possibility of a redox exchange following C–N bond formation between **Q1** and **C3**. While this may account for the efficiency of C–N bond formation, it cannot account for the formation of **Q2**, which represents an oxidative coupling between **Q1** and **P1** (Scheme 3E). This coupling reaction should be equally sensitive to the formation of electron-rich catechol **C2**, which would be capable of redox exchange with **Q1**. In all of our attempts to perform the addition of **P1** to **Q1** in the absence of Cu and O_2_, we observed complex reaction mixtures (see Table S5 in the ESI†). This highlights well-known difficulties of performing a nucleophilic addition to *ortho*-quinones *via* a straightforward 2-electron process.^30^ If, however, **Q1** is premixed with equimolar quantities of CuPF_6_ and DBED prior to the addition of **P1** under O_2_, **Q2** is obtained in yields that range from 80 to 95% (Scheme 3E). This is consistent with a Cu-mediated dehydrogenative coupling between **P1** and **Q1** to afford **Q2** that does not generate catechol **C2** as an intermediate. Additional support for this hypothesis is provided by the complete absence of **Q3** if **C2**, or if a 1 : 1 mixture of **C2** and **P1**, is subjected to the standard catalytic conditions. These results are surprising since catechols are considerably more electron-rich than phenols and are generally easier to oxidize to the *ortho*-quinone. Nevertheless, a catalyst system composed of 4% CuPF_6_ and 20% DBED does not promote an efficient oxidation of **C2**, nor of **C3** (see ESI†). The absence of catechols under our catalytic conditions contrasts with the previous work of Maumy and Capdevielle, who proposed catechols as viable products of oxygenation under their stoichiometric Cu-mediated conditions (so-called “corrosion method”).^31^ We suspect that in both cases, the immediate product of oxygenation is a quinone–Cu complex (see below),^25^,^32^ and that the fate of this intermediate rests in the precise nature of the reaction conditions.

#### Spectroscopic investigation

**Scheme 4:**
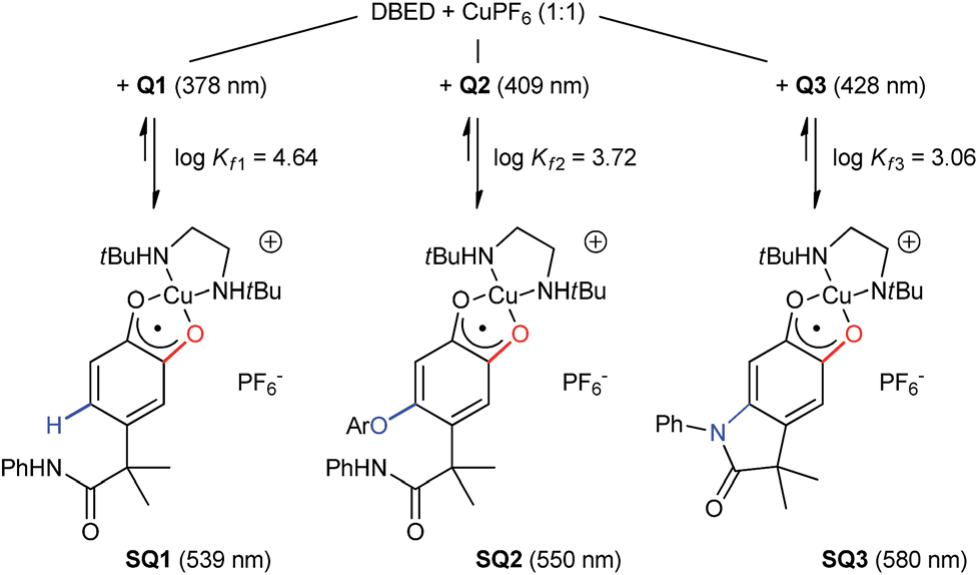
Independent synthesis of **SQ1–3** by coordination and electron-transfer of DBED-Cu(i) to **Q1–3**, with binding constants and main spectral features. Details in Fig. S3–S6.†

**Fig. 1:**
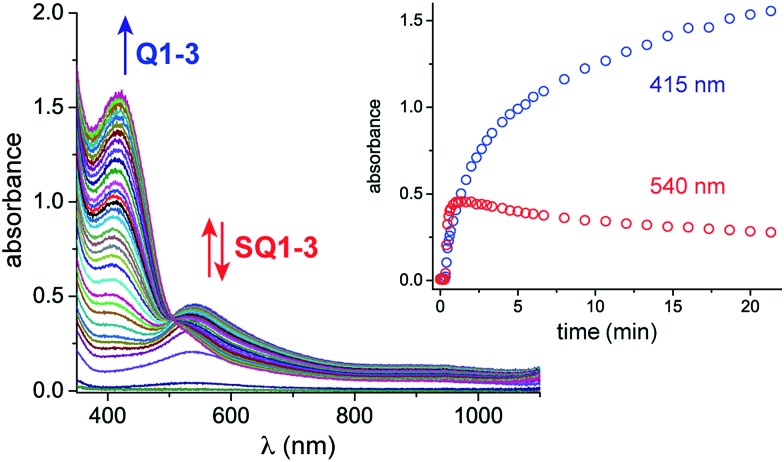
*In situ* UV-vis spectroscopic monitoring under catalytic conditions: CH_2_Cl_2_, 25 °C, 15.67 mM **P1**, 9% CuPF_6_, 20% DBED, O_2_ (2 atm), 1.0 mm pathlength. Inset: absorbance profile at 415 and 540 nm. To ensure homogeneous mixtures of **P1**, UV-vis experiments were conducted at a low concentration of **P1**. To improve the rate of conversion, the Cu loading was increased to 8–9%.

**Fig. 2:**
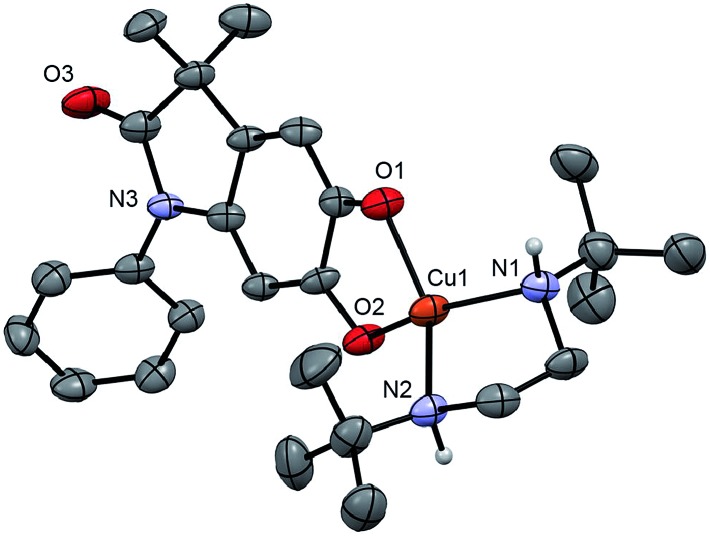
ORTEP at 50% ellipsoid probability of one molecule of **SQ3**, a cation, in the X-ray structure of (**SQ3**)_2_(SbF_6_)_2_·2.5 CH_2_Cl_2_. H atoms except those on N1 and N2 are omitted for clarity. Selected bond lengths (Å): Cu–N1 1.992(5), Cu1–N2 1.994(4), Cu1–O1 1.955(4), Cu1–O2 1.946(4), C1–O1 1.284(6), C2–O2 1.291(6). Dihedral angle between N1Cu1N2 and O1Cu1O2 planes: 50.34°.

Monitoring the conversion of **P1** into **Q3** by *in situ* UV-visible spectroscopy reveals the intermediacy of Cu(ii)-semiquinone radicals **SQ1–3** (Scheme 4), which possess characteristic absorption bands at ∼550 nm (Fig. 1a, Fig. S6†). These structural assignments are supported by previous work from us^24^,^25^ and Stack,^22^ ESI-MS characterization of the reaction mixture at short reaction times (Fig. S1–S2†), and the independent synthesis of **SQ1–3** from **Q1–3**, CuPF_6_ and DBED (Scheme 4). Thus, coordination of **Q1–3** with DBED-Cu(i) is thermodynamically favoured, and elicits the transfer of one electron from the metal to the quinone (Scheme 4). Stronger binding is observed with the more electron-poor **Q1** than **Q2** or **Q3**, spanning more than one order of magnitude. The viability of **SQ1–3** as reaction intermediates was confirmed in experiments using them as the sole source of Cu (Section 4e of the ESI†). Thus, the efficiency and selectivity in the transformation of **P1** to **Q3** are not affected when **SQ1–3** are prepared independently, and then employed as pre-catalysts under otherwise identical conditions. The solid-state structure of **SQ3**, obtained by single-crystal X-ray analysis of its SbF_6_^–^ salt (Fig. 2), reveals a pseudo-tetrahedral coordination environment around Cu(ii) with a 50.3° dihedral angle between the NCuN and OCuO planes, and C–O bond lengths within 1.278–1.291 Å, consistent with previously reported Cu(ii)-semiquinones.^25^,^32^,^33^ Finally, the correspondence between the electronic structures of the intensely purple **SQ1**, **SQ2** and **SQ3** (Fig. S6†) and those previously reported in the literature provides additional support for their assignment as DBED-ligated Cu(ii)-semiquinone complexes.^25^,^32^

**Fig. 3:**
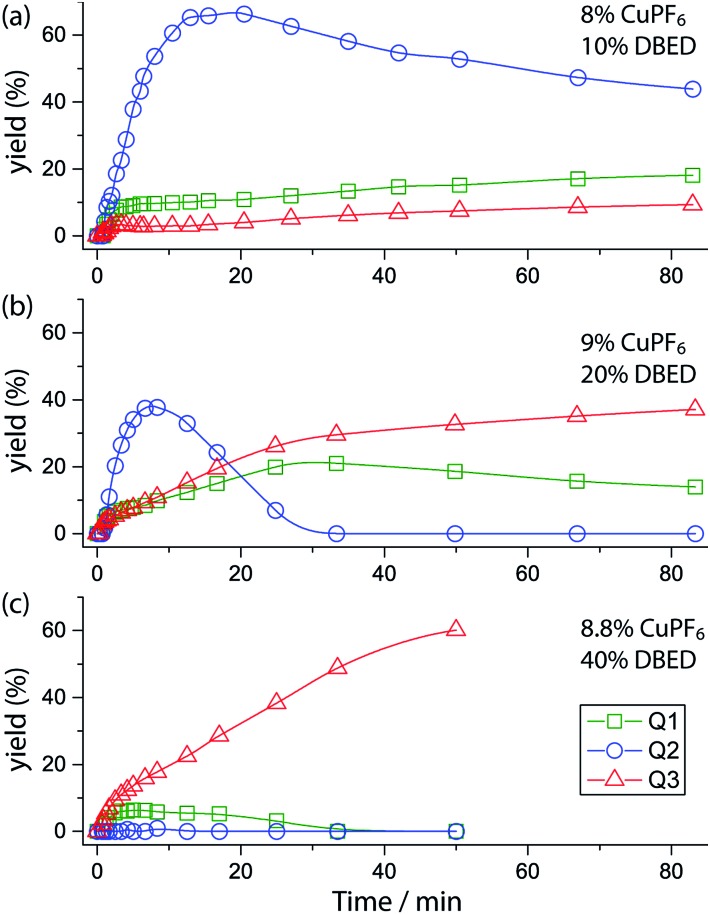
Yields of **Q1** (green squares), **Q2** (blue circles), and **Q3** (red triangles) during the reaction under three sets of conditions, deduced by fitting UV-vis spectra at various time points (see Fig. S7–S9† for full data including **SQ1–3**). Conditions: CH_2_Cl_2_, 25 °C CH_2_Cl_2_, O_2_ (1 atm), 15.67 mM **P1** and (a) 8% CuPF_6_, 10% DBED; (b) 9% CuPF_6_, 20% DBED (as in Fig. 1); (c) 8.8% CuPF_6_, 40% DBED. The *y*-axis is scaled to the maximum concentration of each species, *i.e.* [**Q1**]_max_ = [**Q3**]_max_ = [**P1**]_0_ and [**Q2**]_max_ = 0.5 [**P1**]_0_. Thus each point in the graph gives the yield of each species.

Time profiling of all visible species (**Q1–Q3** and **SQ1–SQ3**) at different DBED/CuPF_6_ ratios reveals how DBED in excess of CuPF_6_ influences the ratio of **Q2** and **Q3** (Fig. 3), as well as the speciation of **SQ1–3** (Fig. S7–S9†). UV-vis monitoring of the catalytic oxygenation of **P1** with a small excess of DBED relative to CuPF_6_ (10% DBED and 8% CuPF_6_ per **P1**; 1.25 ratio) shows the fast formation of **SQ1** as a ∼1 : 1 mixture with **SQ2**, which gradually converts to **Q2** as the reaction progresses (Fig. 3a and S7†). Appreciable amounts of **Q3** (>10%) are only observed after 1 h, consistent with the results of entry 1 in Scheme 3A, which return **Q2** as the major product when the DBED/CuPF_6_ ratio is 1.25. When the concentration of DBED is increased to a ∼2-fold excess relative to CuPF_6_, **SQ1–3** form rapidly, and gradually convert to **Q3** over the course of 1 h (Fig. 3b and S1, S2 and S8†). Importantly, the quantity of **Q2** goes through a maximum, strongly suggesting that it is an intermediate in the formation of the final product, **Q3**. The use of a ∼4-fold excess of DBED results in the most rapid formation of **Q3**, supporting our hypothesis that **Q3** forms by a DBED-promoted cyclization of **Q2** (Fig. 3c and S9†). A notable feature of the reaction speciation is the consistently low concentration of **Q1**. Of all the quinones, **Q1** is the least substituted and most electron-deficient, making it the most reactive towards non-discriminant nucleophilic attack. By remaining bound to Cu as **SQ1**, the most prevalent semi-quinone intermediate in all experiments, **Q1** is effectively protected as a partially reduced, Cu-ligated radical anion, whose stability is enhanced by steric shielding of the ligand environment.

#### Proposed catalytic cycle

**Scheme 5:**
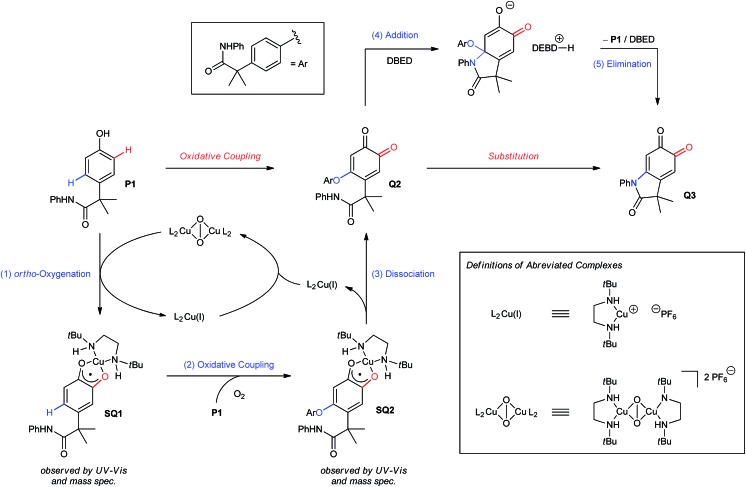
Proposed mechanism.

The results from our mechanistic studies lead us to propose the catalytic cycle illustrated in Scheme 5. *ortho*-Oxygenation of **P1** with a DBED_2_Cu_2_O_2_ peroxo species^25^ affords a mixture of **Q1** and **SQ1**, which we observe by UV-vis and ESI-MS. The feasibility of an oxidative coupling between **SQ1** and **P1** in the presence of O_2_ was demonstrated in Scheme 3E, and results in the formation of **SQ2**, which is also observed by UV-vis and ESI-MS. Dissociation of **SQ2** releases Cu(i) and closes the catalytic cycle of *ortho*-oxygenation, setting the stage for a base-promoted cyclization of **Q2** to generate **Q3**. Addition of the pendant amide triggers the elimination of **P1**, which re-enters the catalytic cycle, consistent with entry 6 of Scheme 3B. Under these conditions, heteroatom–carbon bond formation is either oxidative (step 2) or isohypsic (steps 4 and 5), enabling C–H functionalization without the formation of an electron-rich catechol. This is an important feature of the transformation, since it provides a mechanistic framework for the functionalization of *ortho*-quinones that avoids problems of selectivity that can be associated with redox-exchange.

The involvement of Cu beyond *ortho*-oxygenation creates an important distinction with melanogenesis, wherein Cu remains bound within the active site of tyrosinase.^20^ In melanogenesis, spontaneous cyclization of dopaquinone into cyclodopa outside of the enzyme's active site produces a quinone/catechol redox-couple that can engage in redox exchange (Scheme 1B).^10^ To what extent redox exchange contributes to the complexity of the melanin polymer remains unclear, but its negative impact on the cyclization of **Q1** is clear (Scheme 3C). Thus, we attribute the high degrees of selectivity under our catalytic conditions to the intimate involvement of Cu during and following *ortho*-oxygenation, which sequesters **Q1** as the **SQ1** complex, and promotes an oxidative coupling with **P1** that avoids the formation of an intermediate catechol, and thus redox-exchange. *ortho*-Quinones remain underutilized in organic synthesis, since they are largely viewed as reactive intermediates that are prone to spontaneous polymerization. We demonstrate here that many of these complications can be avoided by metal complexation, which should become a general strategy for exploiting the broad reactivity of these intermediates.

### Part 2. Synthetic utility

#### Scope of substituents on nitrogen

Cross-coupling reactions that form C–N bonds by reductive elimination are sensitive to the steric and electronic properties of the nitrogen atom undergoing bond formation.1a,^34^ This is particularly evident in dehydrogenative C–N bond forming reactions, which are typically restricted to a single substituent on nitrogen for a given set of conditions.7d,^35^ This is not the case under our conditions, where C–N bond formation is a base-promoted process that does not require a transition metal. As a result, a broad range of nitrogen substituents possessing very different p*K*_a_ values are accommodated by making only slight adjustments to the reaction pH (Table 1). For example, when our standard conditions are applied to the cyclization of benzyl amide **1a**, coupled *ortho*-quinone **2a** is formed selectively (entry 2), due to the decreased acidity of the amide N–H bond relative to **P1** (entry 1). Correspondingly, selectivity for the oxindoloquinone is restored by adding 30 mol% of the more basic 1,8-diazabicyclo[5.4.0]undec-7-ene (DBU) (entry 3). Alternatively, cyclization of an *O*-methyl hydroxamic ether under standard conditions (entry 4) returns significant decomposition that we attribute to the increased acidity of the N–H bond and the potential lability of the N–O bond. Therefore, to restore selectivity for the oxindoloquinone, we simply decrease the amount of DBED to 5 mol% (entry 5). Finally, the successful cyclization of a Boc-imide (entry 6) highlights the breadth of substituents that are tolerated on nitrogen, which include aryl, alkyl, methoxy or carbonyl. To our knowledge, there are no other examples of a dehydrogenative amination reaction that tolerate comparably diverse substituents on the nitrogen nucleophile.^2^

**Table 1:** Scope of substituents on nitrogen^*a*^

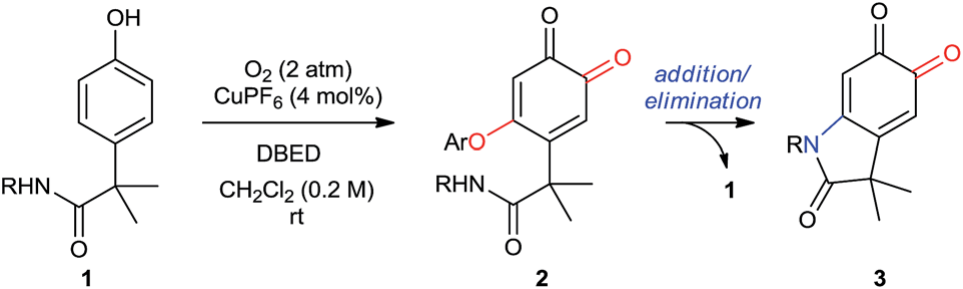							
Entry	R	Approximate p*K*_a_ (DMSO)^*b*^	DBED (mol%)	Time (h)	Conversion (%)	Yield of **2**^*c*^ (%)	Yield of **3**^*c*^ ^,^^*d*^ (%)
1	Ph (**P1**)	22	20	4	100	—	98 (90)
2^*e*^	Bn (**1a**)	26	20	4	80	56	4
3	Bn (**1a**)		20	8	78	<2	67 (61)
4	OMe (**1b**)	17	20	4	74	—	37
5	OMe (**1b**)		5	4	86	—	72 (59)
6^*f*^	Boc (**1c**)	18	20	4	93	—	86 (84)

^*a*^Reactions performed with 0.5 mmol of **1**.

^*b*^These approximate p*K*_a_ values for the amide N–H are derived from Bordwell's or Evans' p*K*_a_ tables.

^*c*^Product yield determined by ^1^H-NMR using hexamethylbenzene as an internal standard.

^*d*^Isolated yield in parenthesis using 1 mmol of **1**.

^*e*^DBU (30 mol%) was used as additive.

^*f*^Due to poor substrate solubility, the reaction was performed at 45 °C.

#### Aryl substitution on nitrogen

The particular importance of *N*-aryl^36^ linkages in pharmaceuticals and agrochemicals1a,b,36d prompted us to investigate the scope of aryl substitution on the acetanilide (Table 2). Both donating and withdrawing groups are tolerated on the aryl ring (entries 1–16), including the strongly electron-donating and redox-sensitive *N*,*N*-dimethylamine (entry 5). Primary benzylic hydrogen atoms are also tolerated (entries 6–8), as are halogen substituents (entries 10–14), demonstrating compatibility with C–H bonds that are susceptible to aerobic oxidation,11c,^37^ as well as functional groups classically employed in cross-coupling reactions. A particular advantage of using Cu to oxidize C–H bonds is its resistance to heteroatom poisoning, which frequently limits related transformations catalyzed by precious metals.^38^ Under our conditions, a broad range of heteroaromatic rings are tolerated, affording pyridine-, pyrazine-, pyrazole- and quinoline-substituted oxindoloquinones in good yields (entries 18 to 24). These examples also highlight the compatibility of our method with directing groups commonly used for directed C–H functionalization (entries 18 and 23),2g,^39^ and sets the stage for substrate diversification by orthogonal amide-directed C–H oxidations (see Scheme 7 below).

**Table 2:** Scope of aryl and hetero-aryl substituents on nitrogen^*a*^

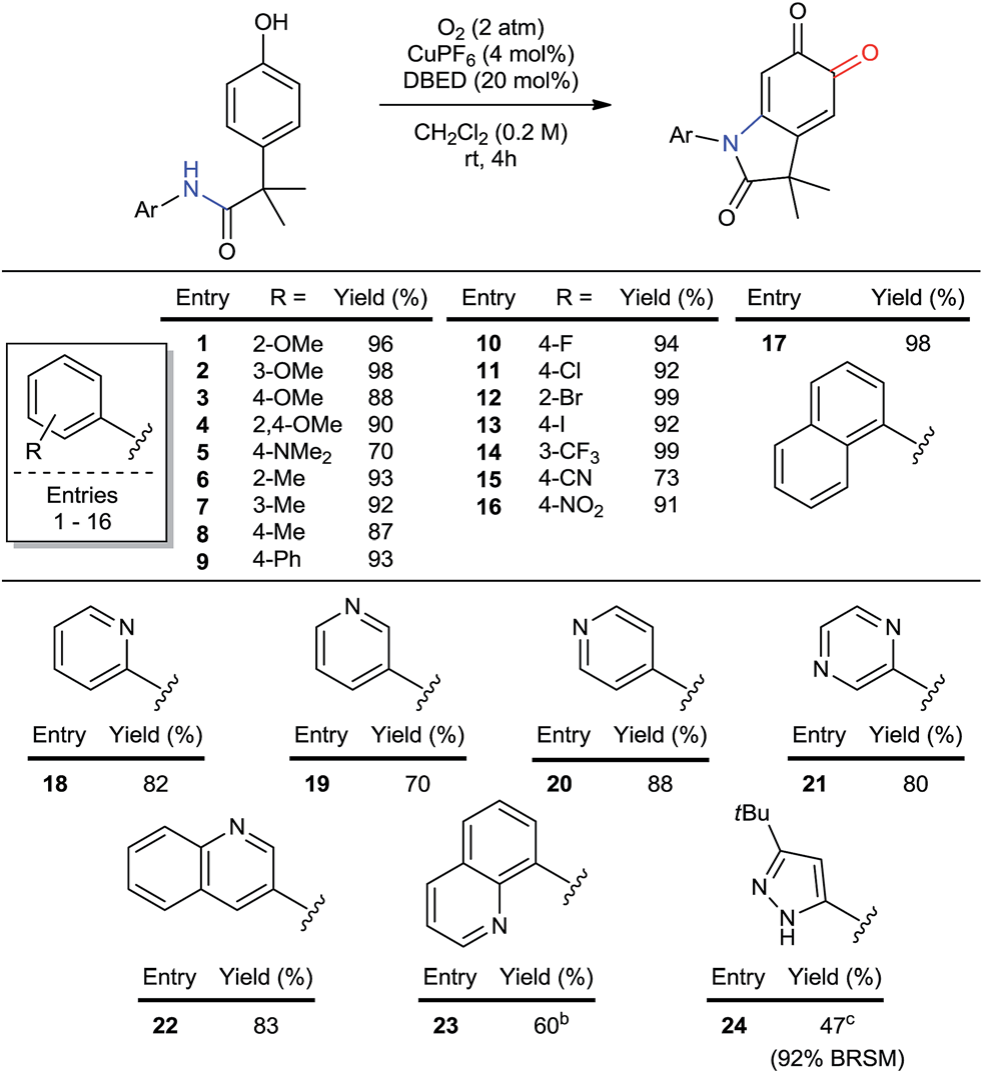

^*a*^Reactions performed on 1 mmol scale and reported yields are of the purified oxindoloquinone.

^*b*^30 mol% DBED, 6 h.

^*c*^A 20%-by-volume mixture of THF in CH_2_Cl_2_ was used due to issues of solubility.

#### Scope of benzylic substituents

Our method remains chemoselective for *ortho*-oxygenation of phenols bearing a variety of benzylic substituents (Table 3). These include allyl, prenyl and cinnamyl groups (entries 1–3), as well as a silyl-protected alkyne (entry 4), demonstrating compatibility with π–bonds that provide versatile synthetic handles for additional transformations. Likewise, halogenated benzyl substituents are tolerated (entries 5–8), demonstrating chemoselectivity for phenolic oxidation in the presence of 2° benzylic hydrogen atoms. In addition to a 5-membered spirocycle (entry 9), sensitive functionalities, including a 1° alcohol, a 1° tosylate, an azide, and a nitrile are tolerated under our standard reaction conditions (entries 10–13).

**Table 3:** Scope of benzylic substituents^*a*^

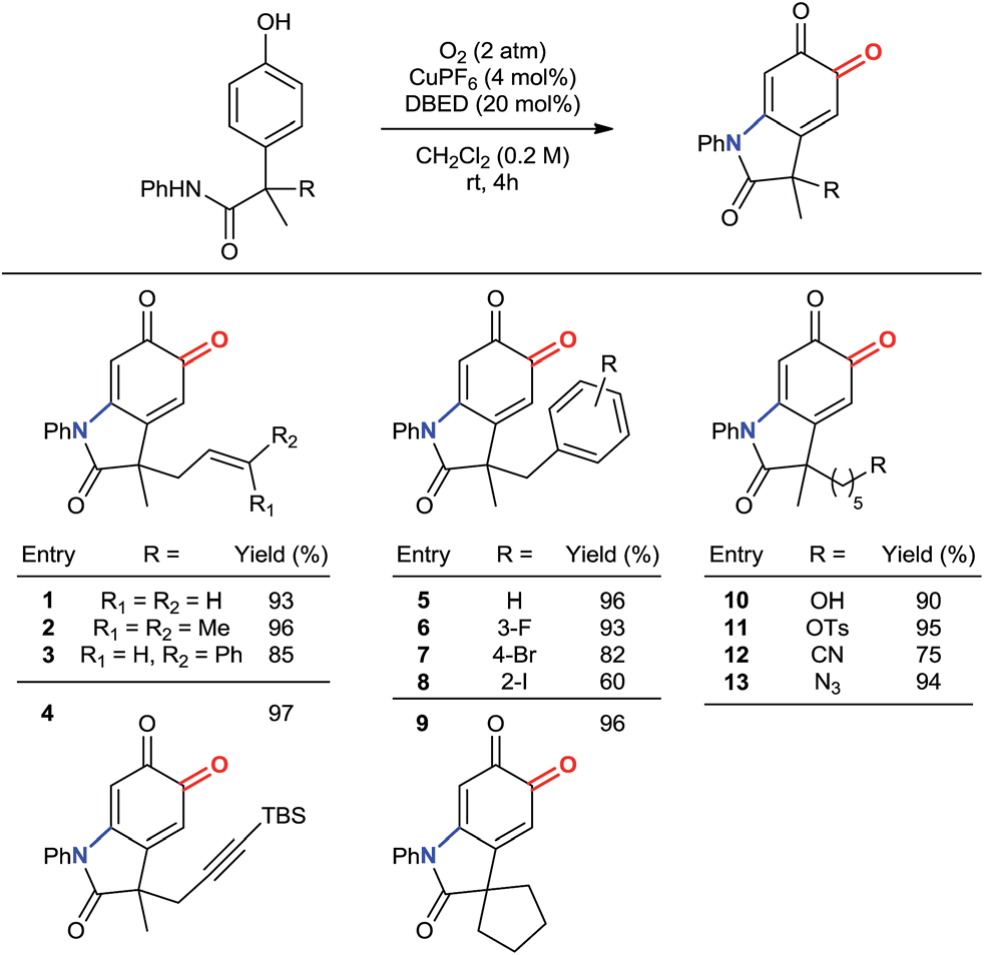

^*a*^Reactions performed on 1 mmol scale and reported yields are of the purified oxindoloquinone.

**Scheme 6:**
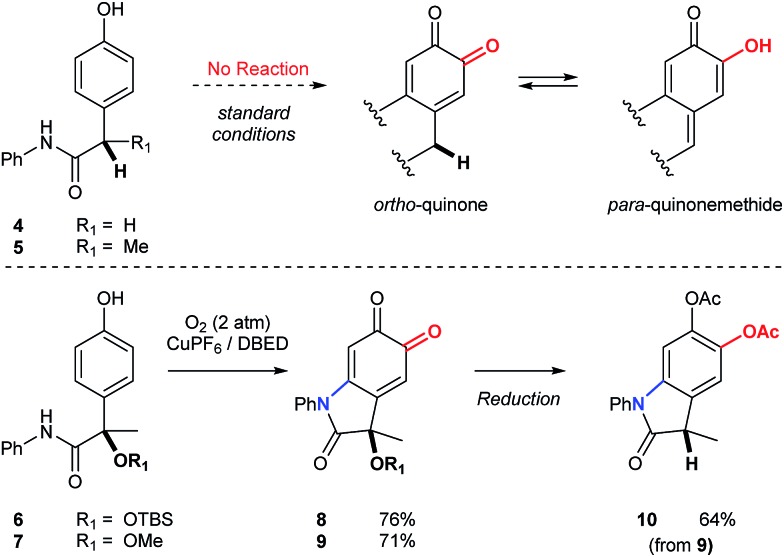
Synthesis of oxindoles possessing 3° benzylic centers. Condition for the synthesis of **8** and **9**: [Cu(MeCN)_4_](PF_6_) (10 mol%), DBED (50 mol%), O_2_ (2 atm), CH_2_Cl_2_ (0.2 M), rt, 12 h. Condition for the reduction of **9** to **10**: (1) Na_2_S_2_O_4_(aq), then Ac_2_O, NEt_3_. (2) BF_3_·Et_2_O, Et_3_SiH. See ESI† for detailed experimental procedures.

Substrates possessing hydrogen atoms α- to the amide constitute a current limitation, since tautomerization of the corresponding *ortho*-quinone to the *para*-quinone methide is problematic (Scheme 6). We have previously demonstrated that α-hydroxyketones, structurally related to the *para*-quinone methide, exhibit a dose-dependent inhibition of catalysis.^19^,^40^ To address this limitation in the context of an oxindole synthesis, we investigated benzylic ethers (**6** and **7**) as hydrogen atom surrogates, since their deoxygenation with triethylsilane occurs smoothly, following reduction and protection of oxindoloquinone **9** (Scheme 6). This provides a concise synthesis of polyfunctional oxindole **10**, possessing a 3° benzylic center.

#### Diversely substituted phenols

**Scheme 7:**
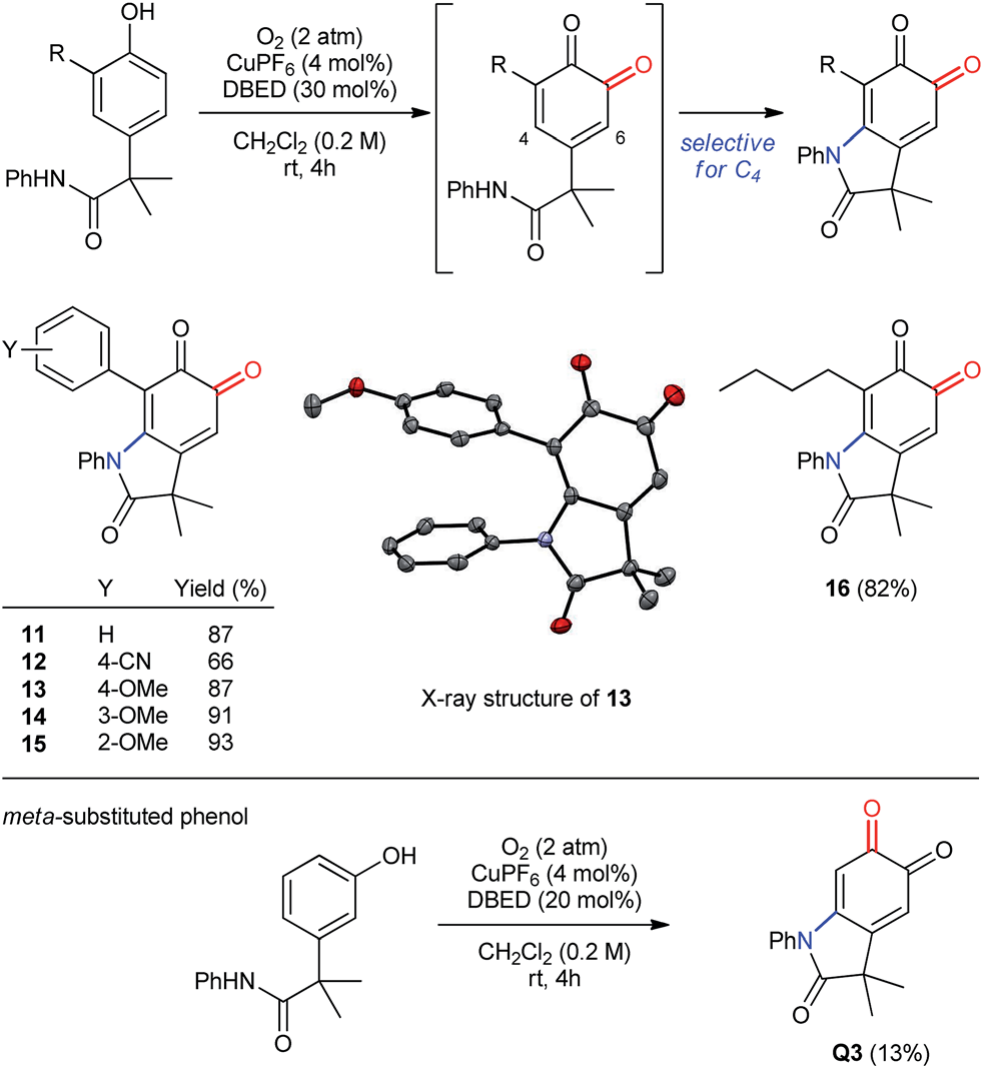
Oxidation of 2,4-disubstituted and *meta*-substituted phenols.

The oxygenation of more sterically encumbered 2,4-di-substituted phenols raises important questions regarding the chemo- and regioselectivity of our methodology (Scheme 7). In previous work,18*a* we have observed poor selectivity for *ortho*-oxygenation of phenols bearing *tert*-butyl substituents at **C2**. Therefore, we were pleased to observe clean oxygenation of phenols possessing less sterically demanding substituents, including aryl or alkyl groups (entries 1–6), which provide the corresponding oxindoloquinone as a single regioisomer resulting from selective 1,4-addition at C4 rather than 1,6-addition at C6. Both electron-donating and withdrawing substituents are tolerated in the *para*-position of the 2-aryl ring, as are methoxy groups in either *ortho*- or *meta*-positions. In addition to aryl rings, an *n*-butyl group is also tolerated in the *ortho*-position, which demonstrates compatibility for benzylic hydrogen atoms that are not acidic (entry 6). Finally, oxygenative cyclization of the *meta*-isomer of **P1** affords an identical oxindoloquinone **Q3** as is obtained from the *para*-isomer **P1**, albeit with diminished efficiency due to the formation of unidentifiable by-products.

#### Diversification by orthogonal C–H functionalization

**Scheme 8:**
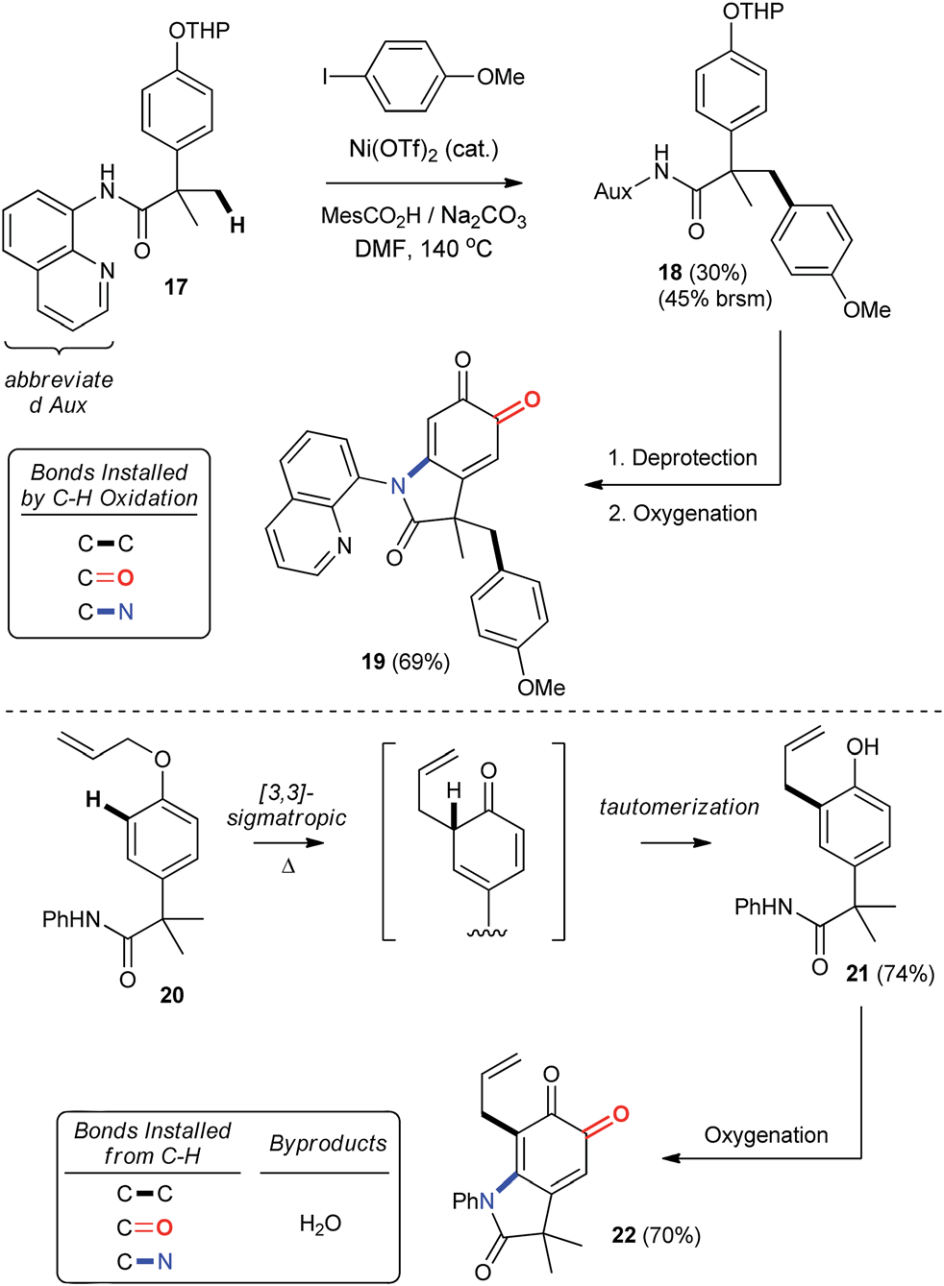
Product diversification by orthogonal C–H functionalization. See ESI† for detailed experimental procedures.

The importance of C–H functionalization to synthetic efficiency has motivated numerous strategies aimed at diversifying these relatively inert and yet omnipresent bonds.^2^ In this context, the 8-aminoquinoline directing group developed by Daugulis has received considerable attention for directing site-selective functionalization of both sp^2^ and sp^3^ hybridized C–H bonds.2g,^39^ Our chemistry offers an important complement to these previously reported methodologies, since phenolic oxygenation is not disrupted by the presence of Daugulis' group (entry 23, Table 2). This creates an opportunity to streamline substrate diversification through orthogonal C–H functionalization reactions, which we illustrate in Scheme 8 by using Chatani's Ni-catalyzed arylation of Csp^3^–H bonds.^41^ This provides THP-protected ether **18**, which is readily converted into oxindoloquinone **19** following removal of the THP group and aerobic functionalization. Alternatively, a more traditional C–H functionalization by way of a 3,3-sigmatropic rearrangement can be used to synthesize 2,4-di-substituted phenol **21**, which is then functionalized under our standard conditions. Thus, the syntheses of **19** and **22** highlight a unique approach to the selective functionalization of the *ortho*- and *meta*-positions of *para*-substituted phenols that hinges on catalytic aerobic dearomatization. This gives rise to regioselective C–C, C–O or C–N bond formation by direct C–H bond functionalization.

#### Diversification of the oxindoloquinone

**Scheme 9:**
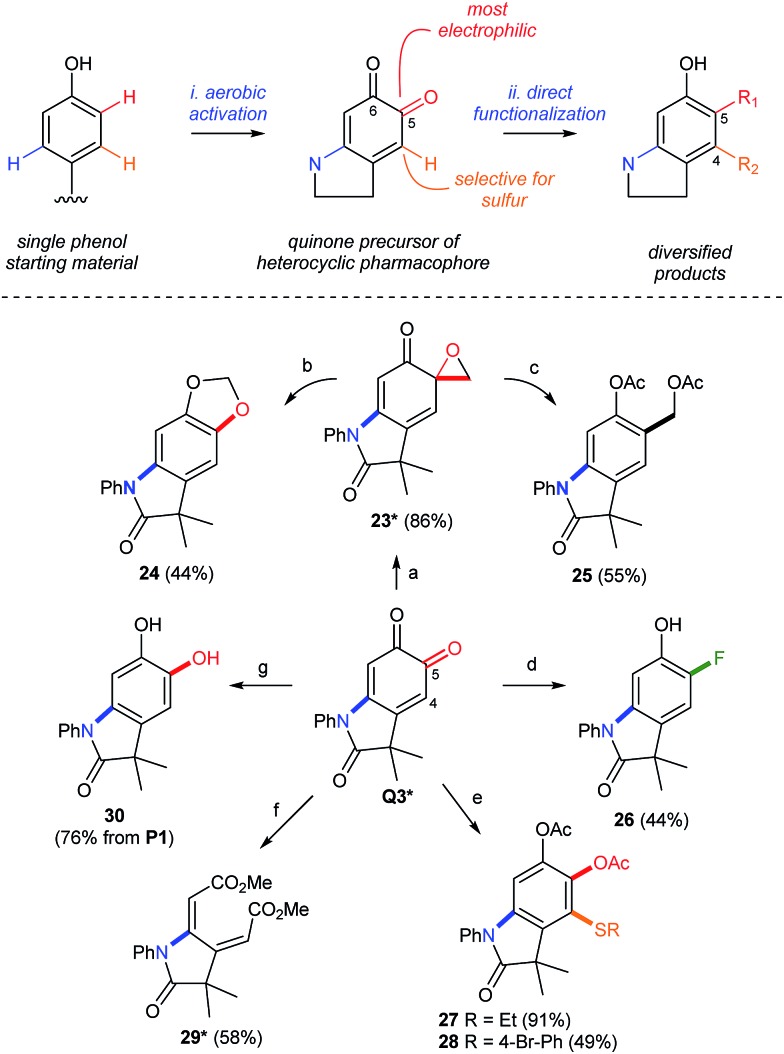
Synthetic Diversification of **Q3**. Yields in parenthesis are based on the amount of **Q3** without other notification. (a) [Cu(MeCN)_4_](PF_6_), CH_2_N_2_, THF. (b) TBSCl, NEt_3_, PhMe. (c) CeCl_3_, NaBH_4_, then Ac_2_O. (d) Deoxofluor®, CHCl_3_, then NaBH_4_, DBU, MeOH. (e) R–SH, DIPEA, CH_2_Cl_2_, then Ac_2_O. (f) Pb(OAc)_4_, PhMe/MeOH. (g) Na_2_S_2_O_4_ workup. *Characterized by X-ray crystallography. See the ESI† for details of the crystallographic characterization and experimental procedures.

The re-aromatization of *ortho*-quinones provides a strong driving force to selectively diversify substituents on the corresponding oxindole. This creates a 1–3 step sequence that functionalizes the *meta*- and *ortho*-positions of the starting phenol by dual C–N/C–O, C–N/C–C, or C–N/C–F bond formation (Scheme 9). Regioselectivity is governed by the electronic differentiation of the quinone carbonyls (see top of Scheme 9). This enables selective manipulation of the more electrophilic carbonyl in **Q3** by regioselective cycloproponation under the conditions of Pettus.^42^ The resulting α-epoxyketone **23**, whose structure was confirmed by single crystal X-ray analysis (see the ESI†), is then isomerized to dioxalane **24** (ref. 43) or reduced to acetoxy methyl derivative **25** (ref. 44) by selective C–C or C–O bond cleavage, respectively. Deoxyfluorination^45^ is also selective for the C5 carbonyl, enabling a *meta*-C–N, *ortho*-C–F dual-functionalization of **P1** over a short synthetic sequence. Differentiation of the 1,3-cyclohexadiene in **Q3** is also possible by the regioselective addition of sulfur to C4, which highlights a convenient method for the formation of aromatic C–S bonds by a simple addition of the thiol at room temperature. While these examples modify the periphery of the oxindole by aromatization of **Q3**, cleavage of C4–C5 by lead tetraacetate^46^ affords the corresponding muconic ester **29** as a single geometric isomer, as determined by single crystal X-ray analysis (see the ESI†), and demonstrates that either aromatic or non-aromatic lactams, with very different three-dimensional structures, can be produced from the same starting phenol in under 2 steps. This highlights an unappreciated property of *ortho*-quinones, which could be attractive for applications in drug discovery where rapid diversification of a biologically active pharmacophore is required.^47^ We note that phenols are widely distributed in pharmaceuticals^48^ and natural products, making them ideal functional handles for late-stage modifications by directed C–H functionalization.

## Conclusion

The chemistry of melanogenesis offers a wealth of inspiration for developing low-energy and practical solutions to chemical challenges.6*a* This has already been appreciated in materials science, where there are countless applications of bio-inspired resins or adhesives that are synthesized by aerobic catechol or phenol polymerization reactions.^13^,^21^,^49^ While the value of these melanin- like materials is clear, very little is known about the precise nature of their assembly, and controlling or predictably influencing their properties remains difficult. In this article, we highlight two important considerations when approaching the chemistry of melanin. The first is the non-innocent role of transition metals following oxygenation, which influences the reactivity of *ortho*-quinones under our catalytic conditions. The second concerns the nucleophilic attack onto the *ortho*-quinone, which can trigger redox exchange. By avoiding redox exchange, our method introduces C-heteroatom bonds under exceptionally mild and selective conditions when compared to dehydrogenative coupling reactions in general.^2^ Finally, by generating an *ortho*-quinone, our method is unique amongst C–H functionalization reactions, in that the product of C–N bond formation is more reactive than the starting phenol, and is readily diversified through a series of regioselective transformations. This allows the rapid diversification of chemical space from a readily accessible starting material in a process that is solely driven by the favourable reduction of O_2_ to H_2_O.

## Supplementary Material

Supplementary information

Supplementary information

Crystal structure data
