# Supplementary material for: A bio-inspired synthesis of oxindoles by catalytic aerobic dual C–H functionalization of phenols
Source: Chem Sci. 2015 Oct 6;7(1):358–69. doi: 10.1039/c5sc02395e (PMC5952266; doi:10.1039/c5sc02395e)

Electronic Supplementary Material (ESI) for Chemical Science.
This journal is © The Royal Society of Chemistry 2015

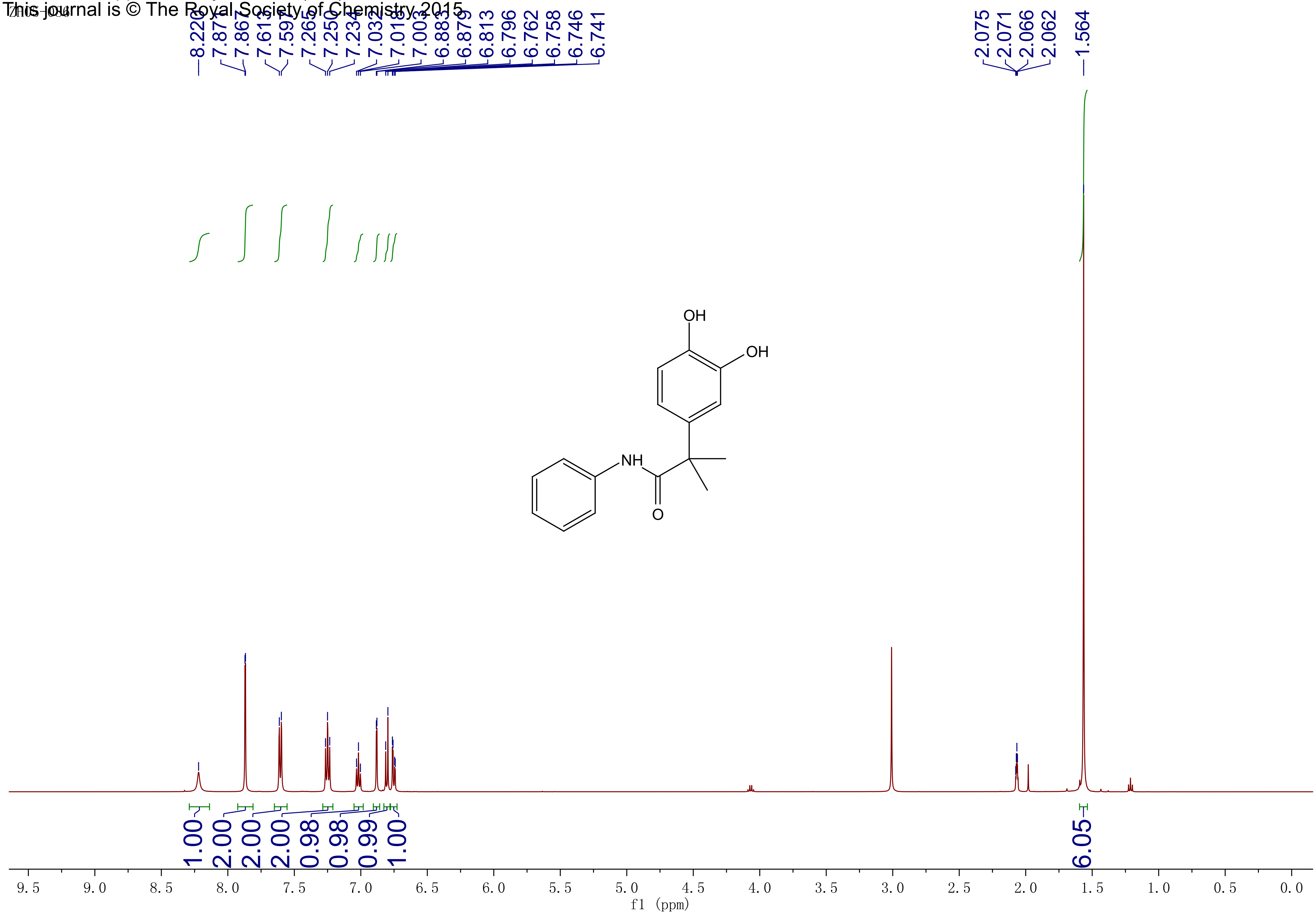

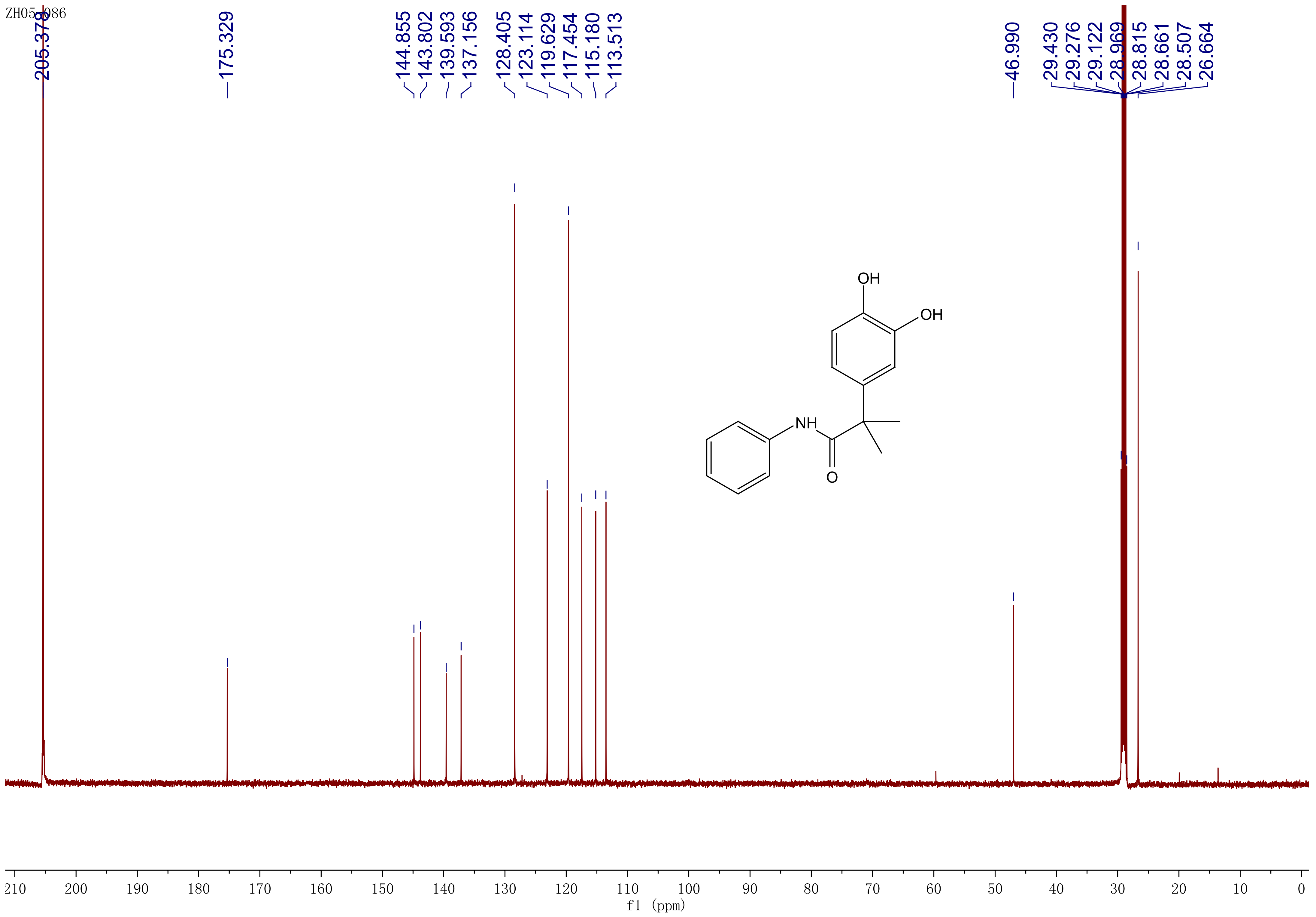
ZH05-086
205.378
175.329
144.855
143.802
139.593
137.156
128.405
123.114
119.629
117.454
115.180
113.513
46.990
29.430
29.276
29.122
28.969
28.815
28.661
28.507
26.664
OH
OH
NH
O
f1 (ppm)

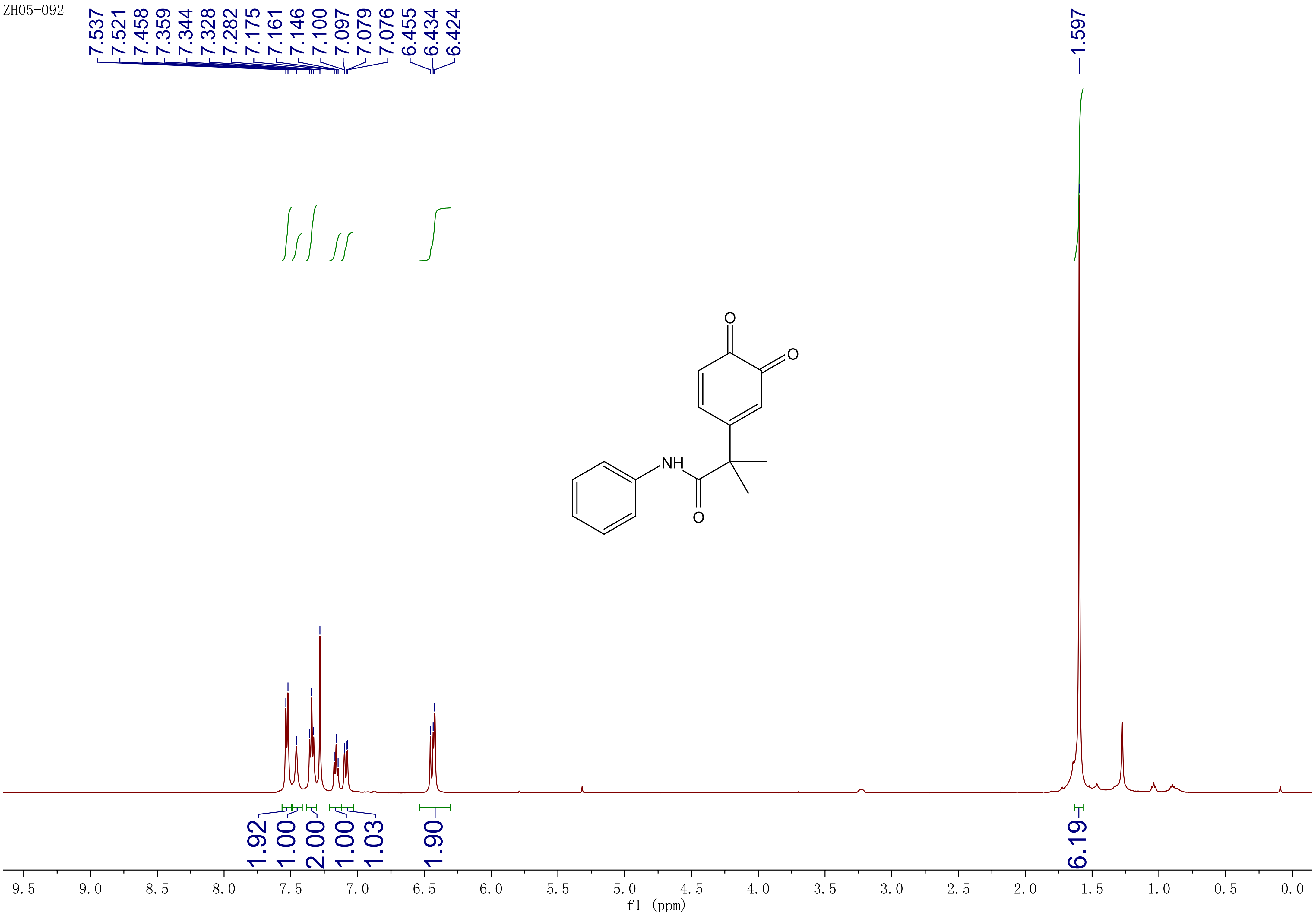
ZH05-092
7.537
7.521
7.458
7.359
7.344
7.328
7.282
7.175
7.161
7.146
7.100
7.097
7.079
7.076
6.455
6.434
6.424
1.597
O
O
NH
O
1.92
1.00
2.00
1.00
1.03
1.90
6.19
9.5 9.0 8.5 8.0 7.5 7.0 6.5 6.0 5.5 5.0 4.5 4.0 3.5 3.0 2.5 2.0 1.5 1.0 0.5 0.0
f1 (ppm)

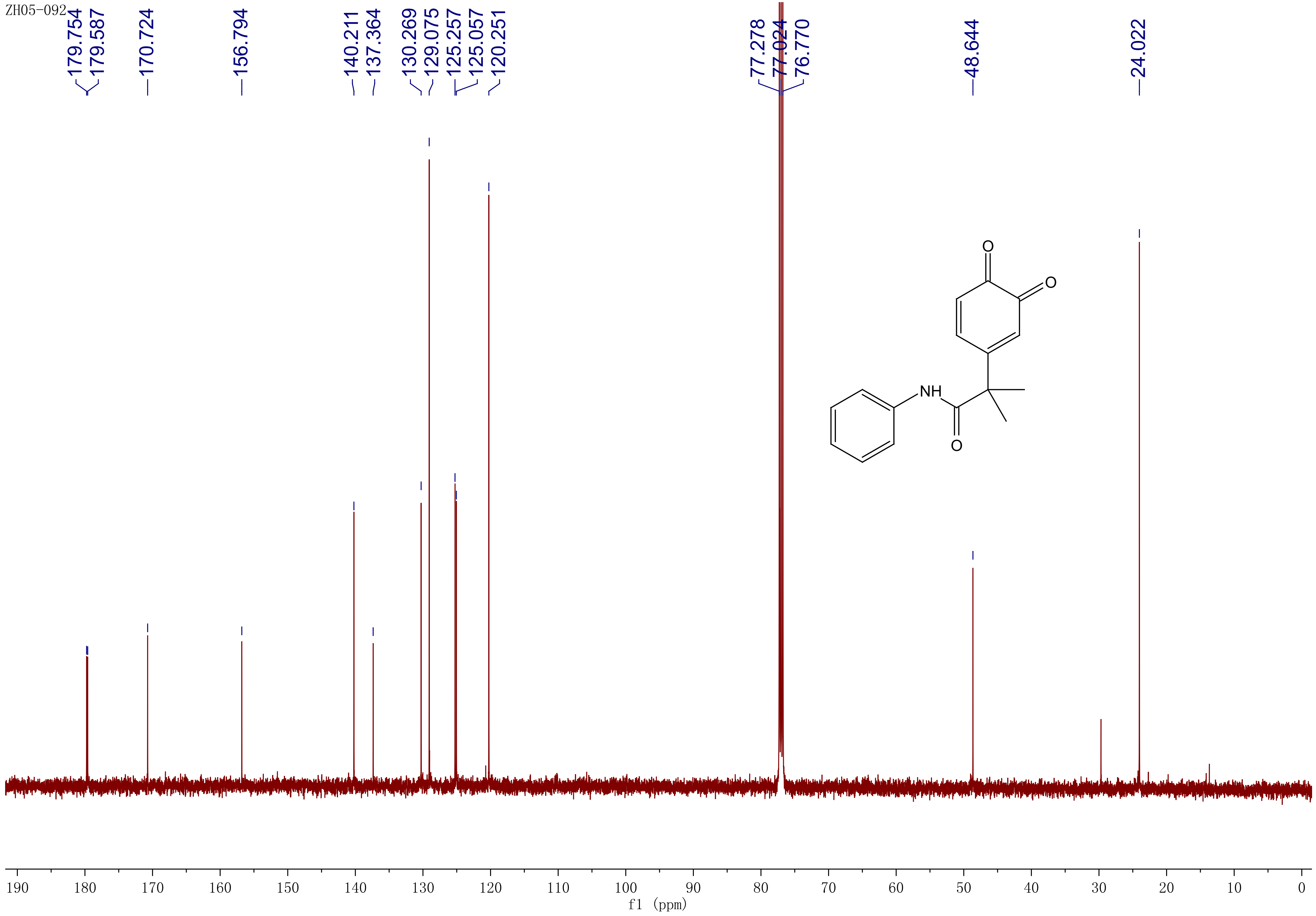
ZH05-092
179.754
179.587
170.724
156.794
140.211
137.364
130.269
129.075
125.257
125.057
120.251
77.278
77.024
76.770
48.644
24.022
O
O
NH
O
190
180
170
160
150
140
130
120
110
100
90
80
70
60
50
40
30
20
10
0
f1 (ppm)

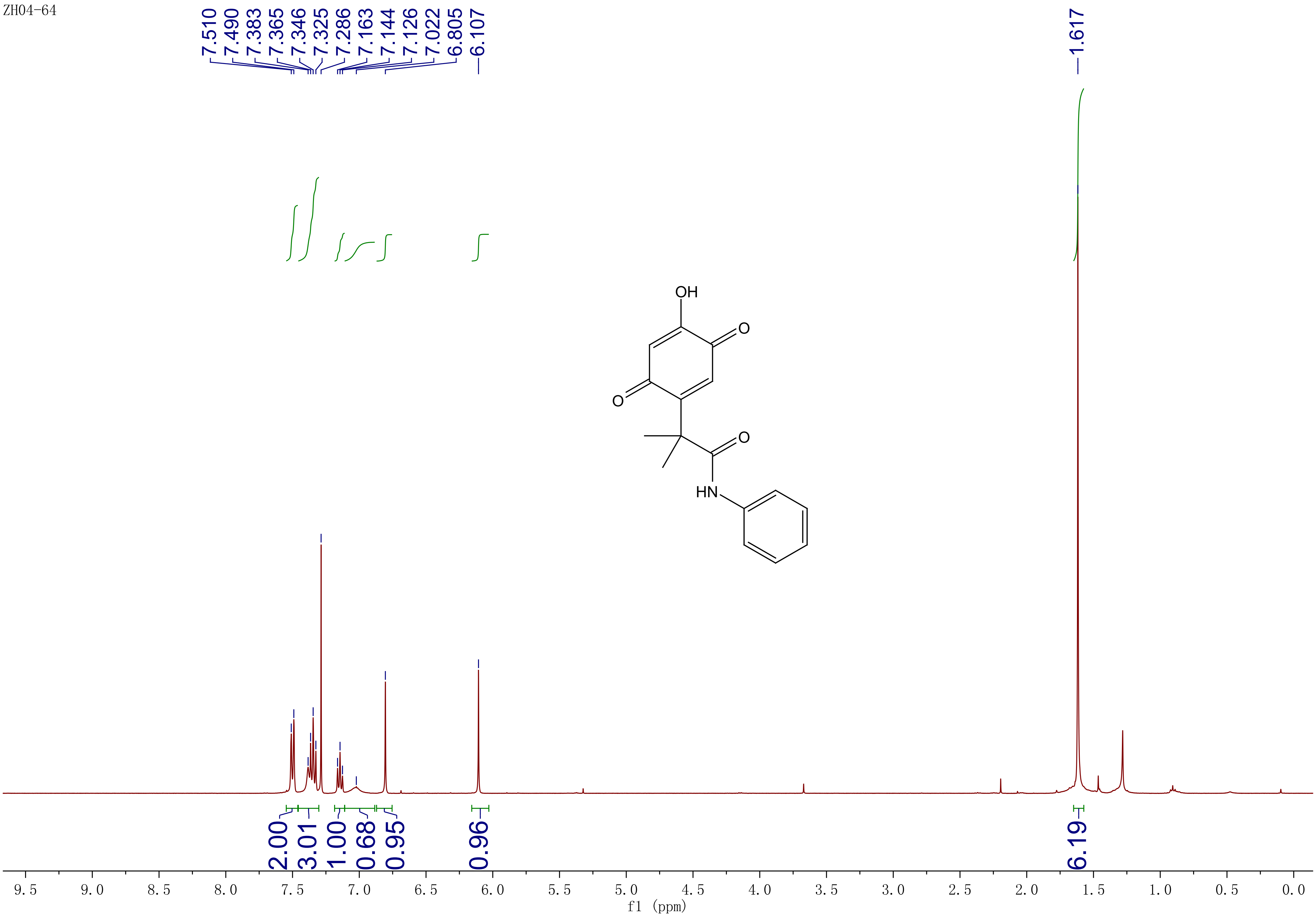
ZH04-64
7.510
7.490
7.383
7.365
7.346
7.325
7.286
7.163
7.144
7.126
7.022
6.805
6.107
1.617
OH
O
O
O
HN
2.00
3.01
1.00
0.68
0.95
0.96
6.19
9.5 9.0 8.5 8.0 7.5 7.0 6.5 6.0 5.5 5.0 4.5 4.0 3.5 3.0 2.5 2.0 1.5 1.0 0.5 0.0
f1 (ppm)

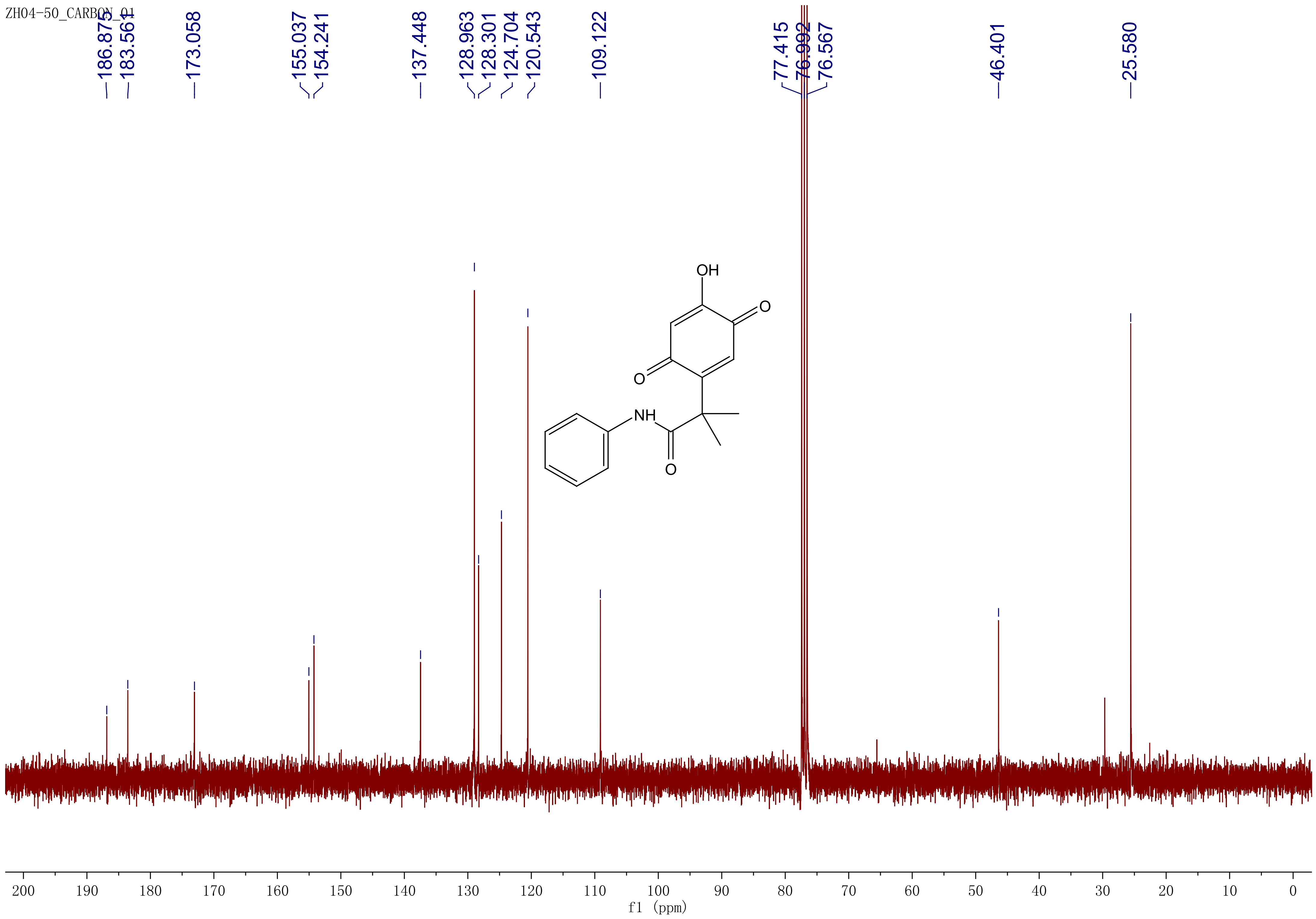
ZH04-50_CARBON_01
186.875
183.561
173.058
155.037
154.241
137.448
128.963
128.301
124.704
120.543
109.122
77.415
76.992
76.567
46.401
25.580
OH
O
O
NH
O
200 190 180 170 160 150 140 130 120 110 100 90 80 70 60 50 40 30 20 10 0
f1 (ppm)

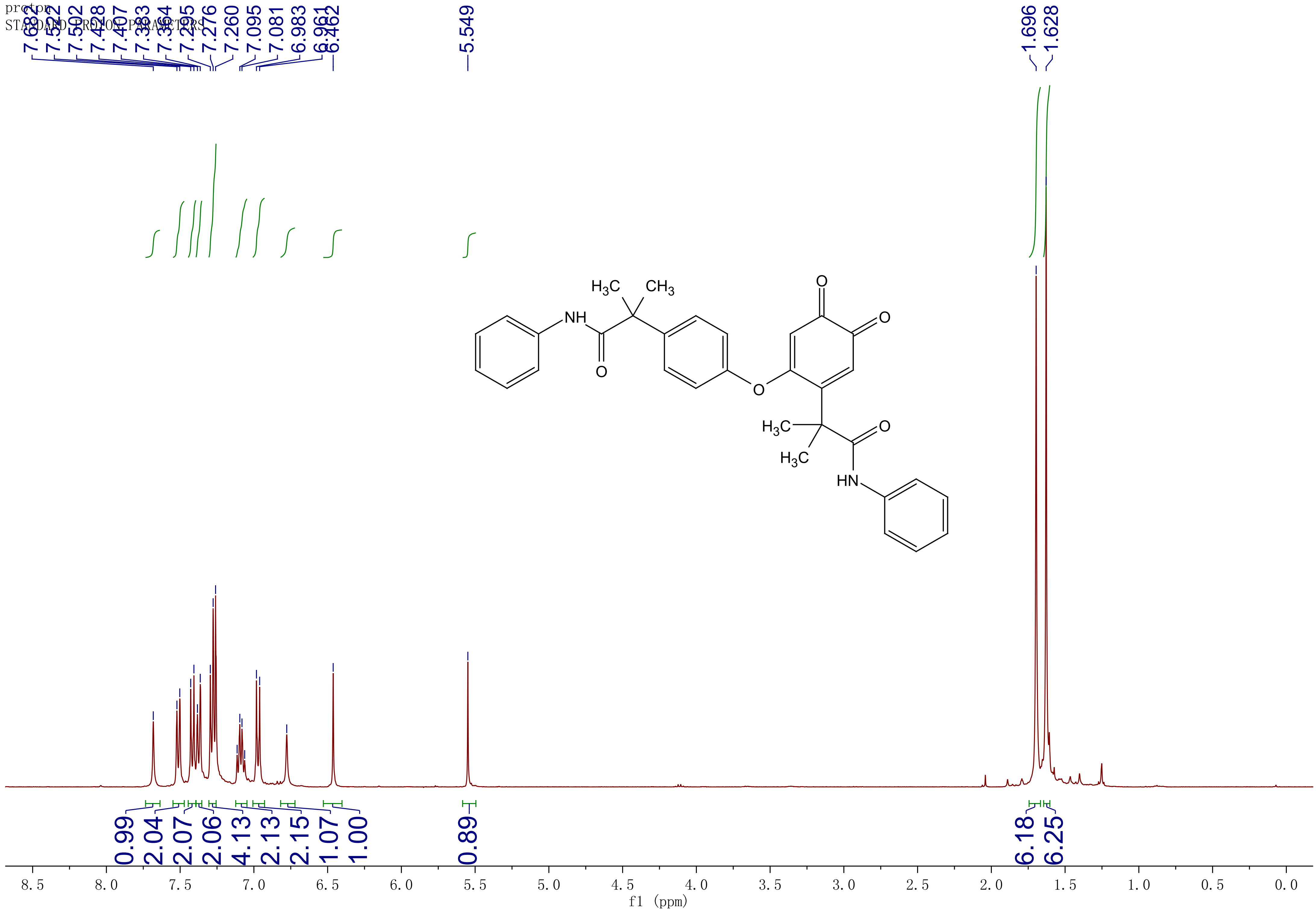

proton
STANDARD PROTON PARAMETERS
7.682
7.522
7.502
7.428
7.407
7.383
7.364
7.295
7.276
7.260
7.095
7.081
6.983
6.961
6.462
5.549
1.696
1.628
H3C
CH3
NH
O
O
O
O
H3C
H3C
O
HN
0.99
2.04
2.07
2.06
4.13
2.13
2.15
1.07
1.00
0.89
6.18
6.25
8.5
8.0
7.5
7.0
6.5
6.0
5.5
5.0
4.5
4.0
3.5
3.0
2.5
2.0
1.5
1.0
0.5
0.0
f1 (ppm)

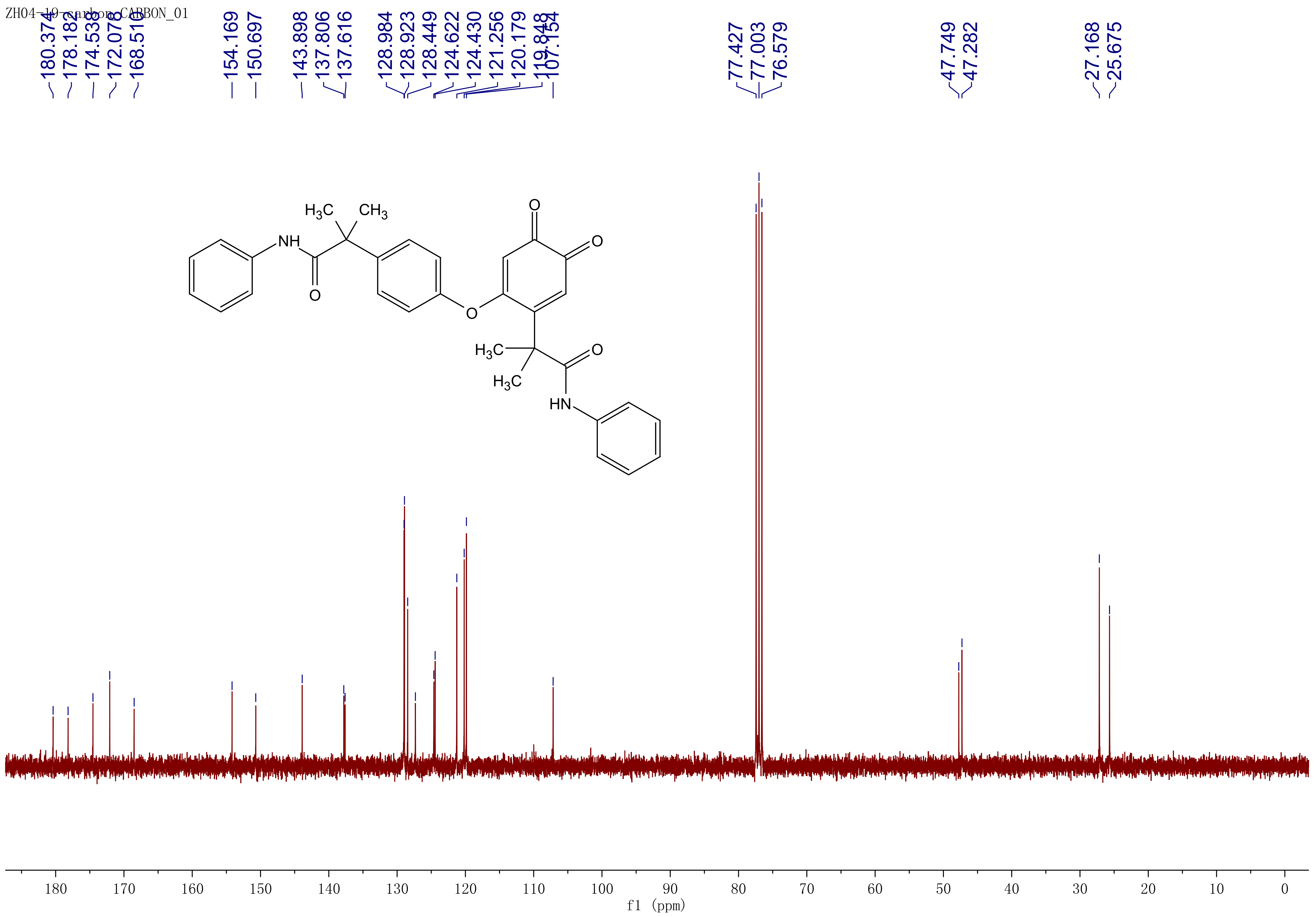

ZH04-19-carbon_CARBON_01
180.374
178.182
174.538
172.078
168.510
154.169
150.697
143.898
137.806
137.616
128.984
128.923
128.449
124.622
124.430
121.256
120.179
119.848
107.154
77.427
77.003
76.579
47.749
47.282
27.168
25.675
H3C
CH3
NH
O
O
O
O
H3C
H3C
O
HN
O
f1 (ppm)

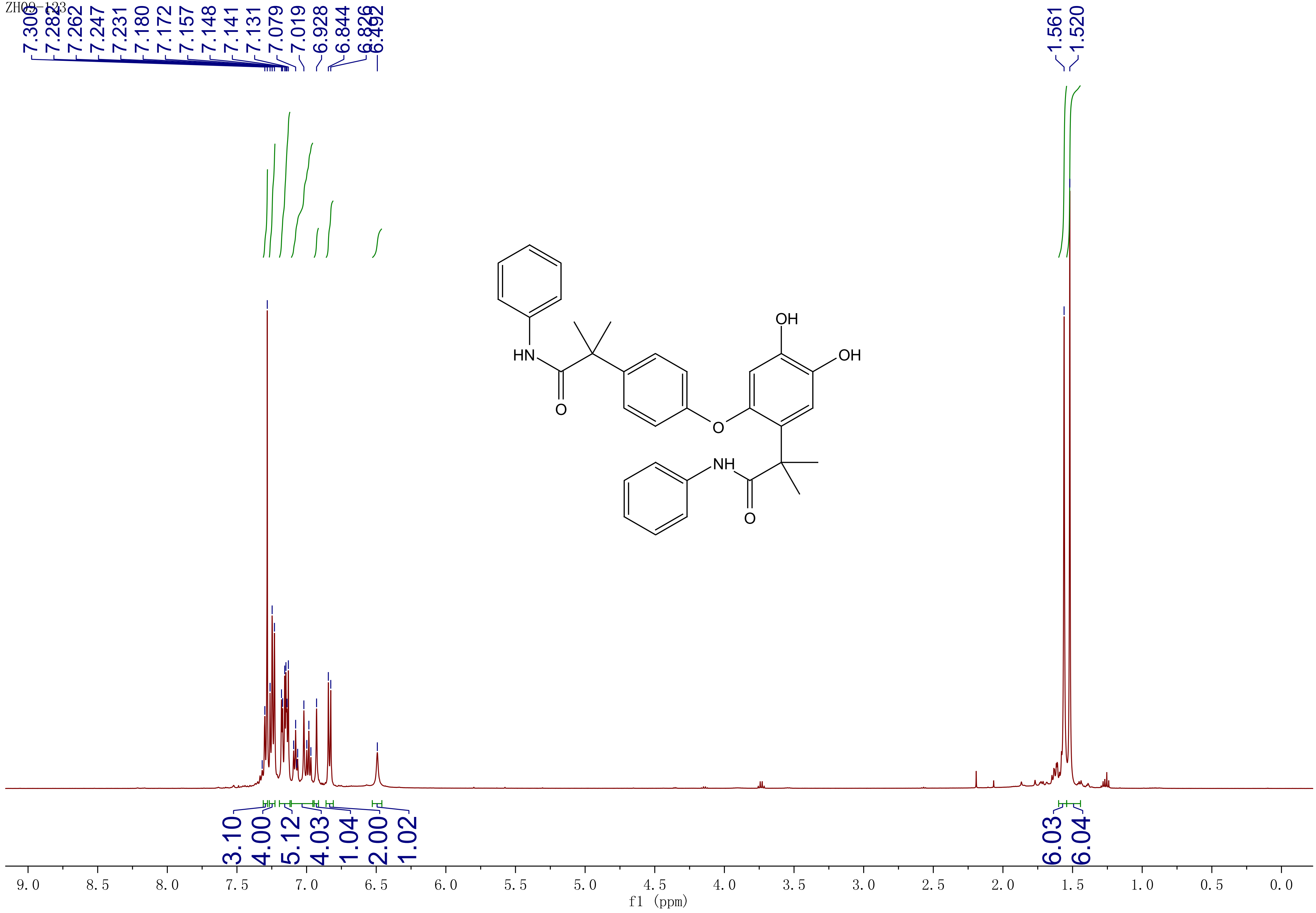
ZH09-123
7.300
7.282
7.262
7.247
7.231
7.180
7.172
7.157
7.148
7.141
7.131
7.079
7.019
6.928
6.844
6.826
6.492
1.561
1.520
OH
OH
HN
O
O
NH
O
3.10
4.00
5.12
4.03
1.04
2.00
1.02
6.03
6.04
9.0
8.5
8.0
7.5
7.0
6.5
6.0
5.5
5.0
4.5
4.0
3.5
3.0
2.5
2.0
1.5
1.0
0.5
0.0
f1 (ppm)

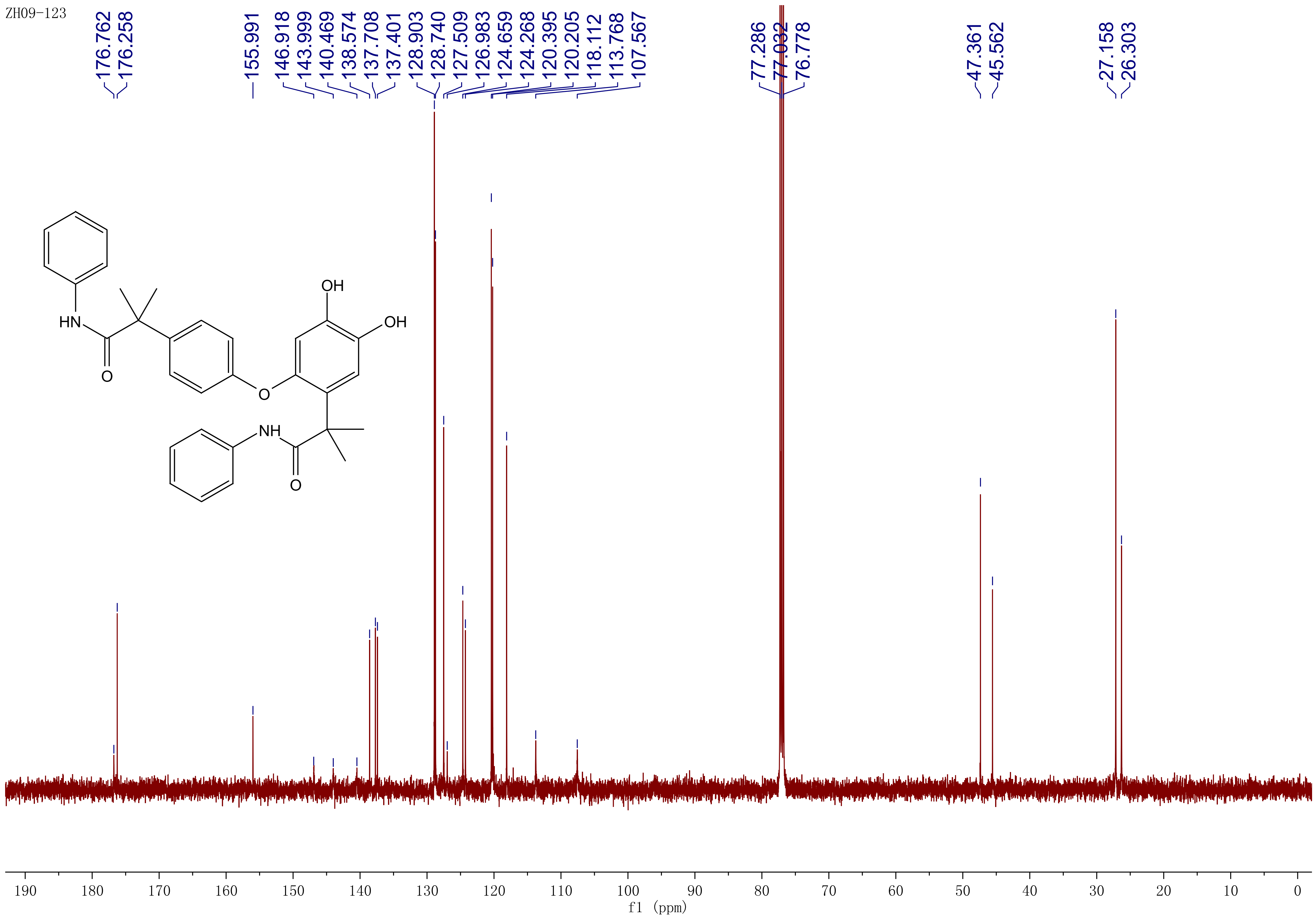
ZH09-123
176.762
176.258
155.991
146.918
143.999
140.469
138.574
137.708
137.401
128.903
128.740
127.509
126.983
124.659
124.268
120.395
120.205
118.112
113.768
107.567
77.286
77.032
76.778
47.361
45.562
27.158
26.303
OH
OH
HN
O
O
NH
O
190
180
170
160
150
140
130
120
110
100
90
80
70
60
50
40
30
20
10
0
f1 (ppm)

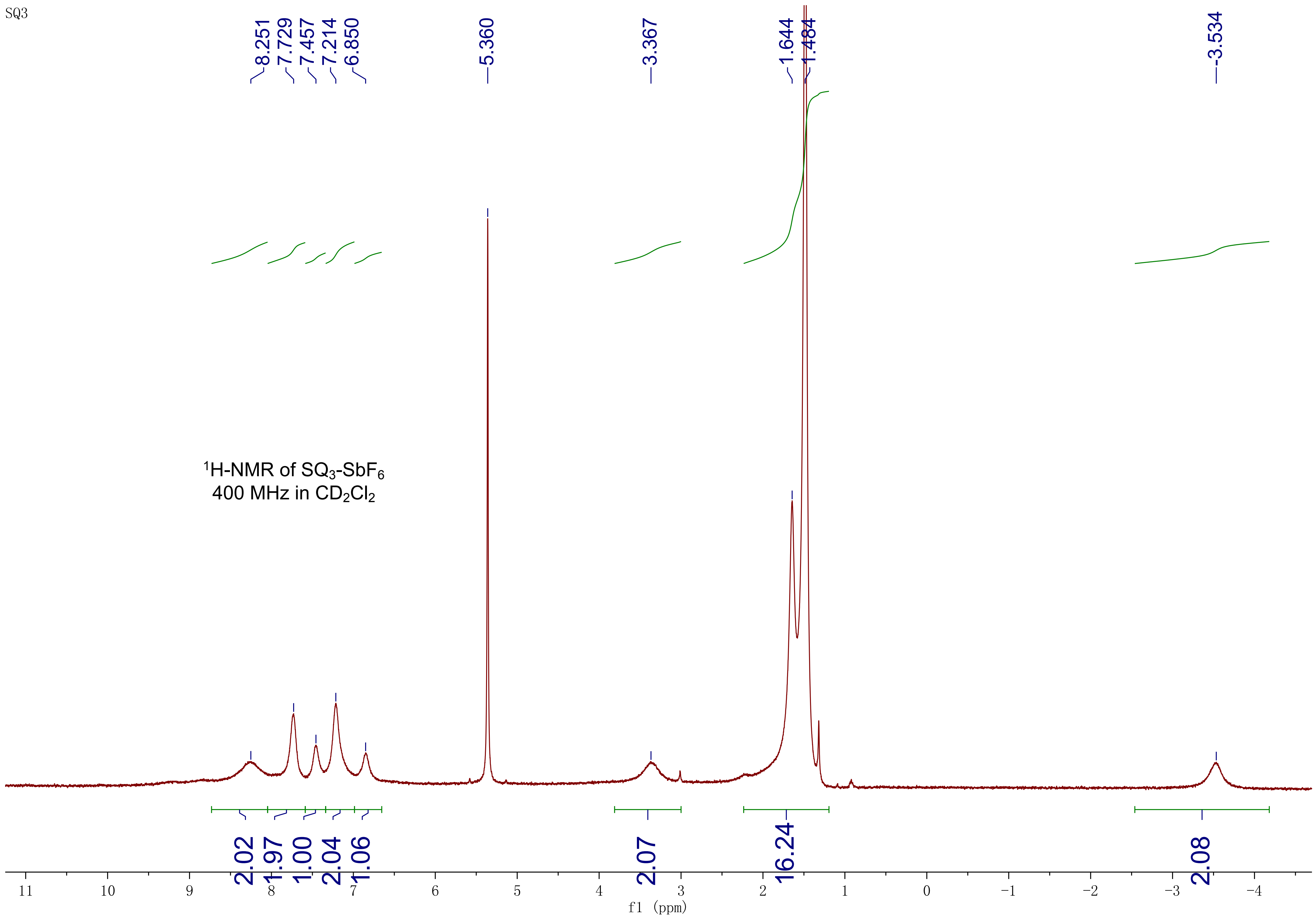
SQ3
8.251
7.729
7.457
7.214
6.850
5.360
3.367
1.644
1.484
-3.534
$^1$H-NMR of $SQ_3$-$SbF_6$
400 MHz in $CD_2Cl_2$
2.02
1.97
1.00
2.04
1.06
2.07
16.24
2.08
f1 (ppm)

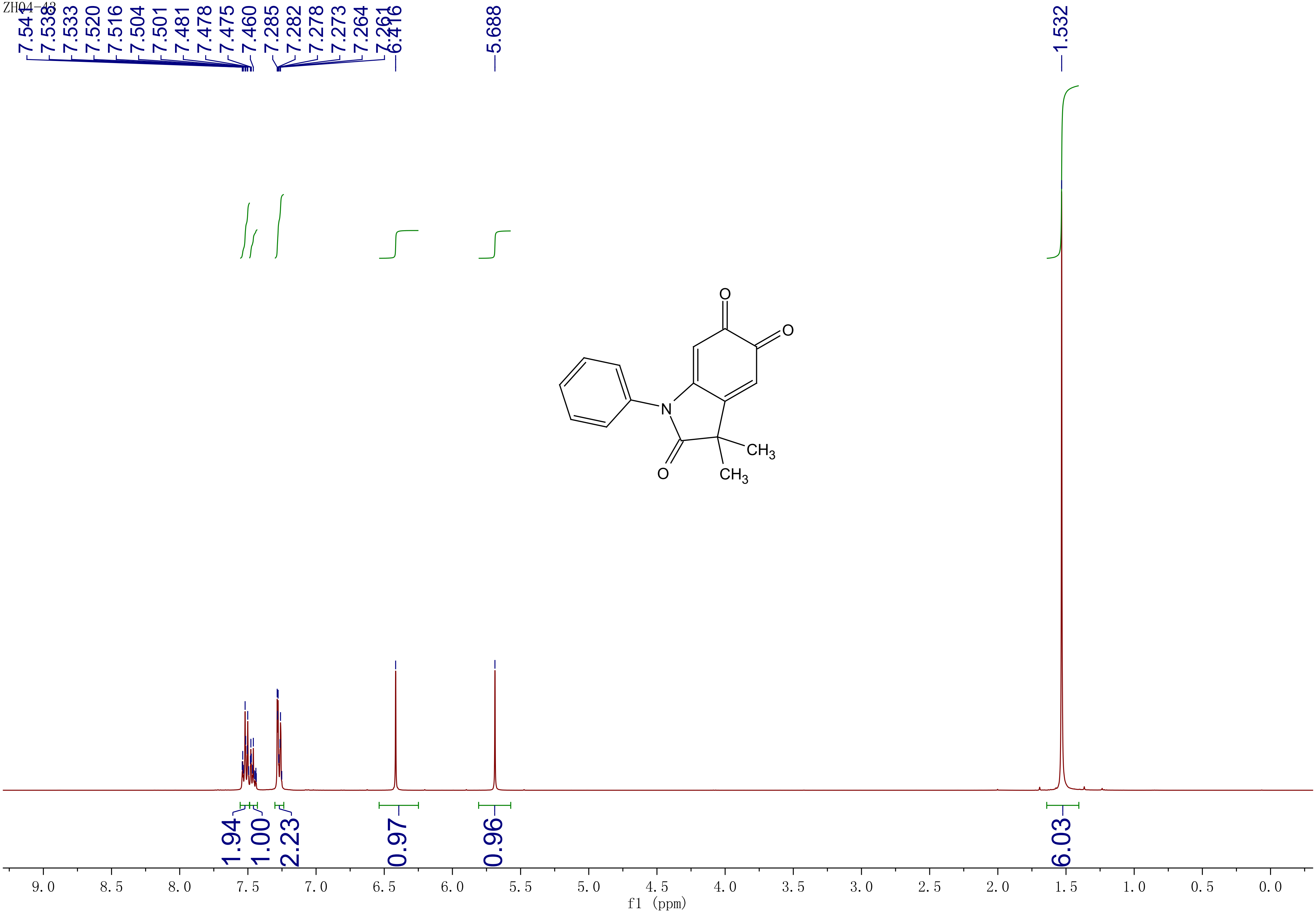

ZH04-43
7.541
7.538
7.533
7.520
7.516
7.504
7.501
7.481
7.478
7.475
7.460
7.285
7.282
7.278
7.273
7.264
7.261
6.416
5.688
1.532
1.94
1.00
2.23
0.97
0.96
6.03
O
O
N
O
CH3
CH3
9.0
8.5
8.0
7.5
7.0
6.5
6.0
5.5
5.0
4.5
4.0
3.5
3.0
2.5
2.0
1.5
1.0
0.5
0.0
f1 (ppm)

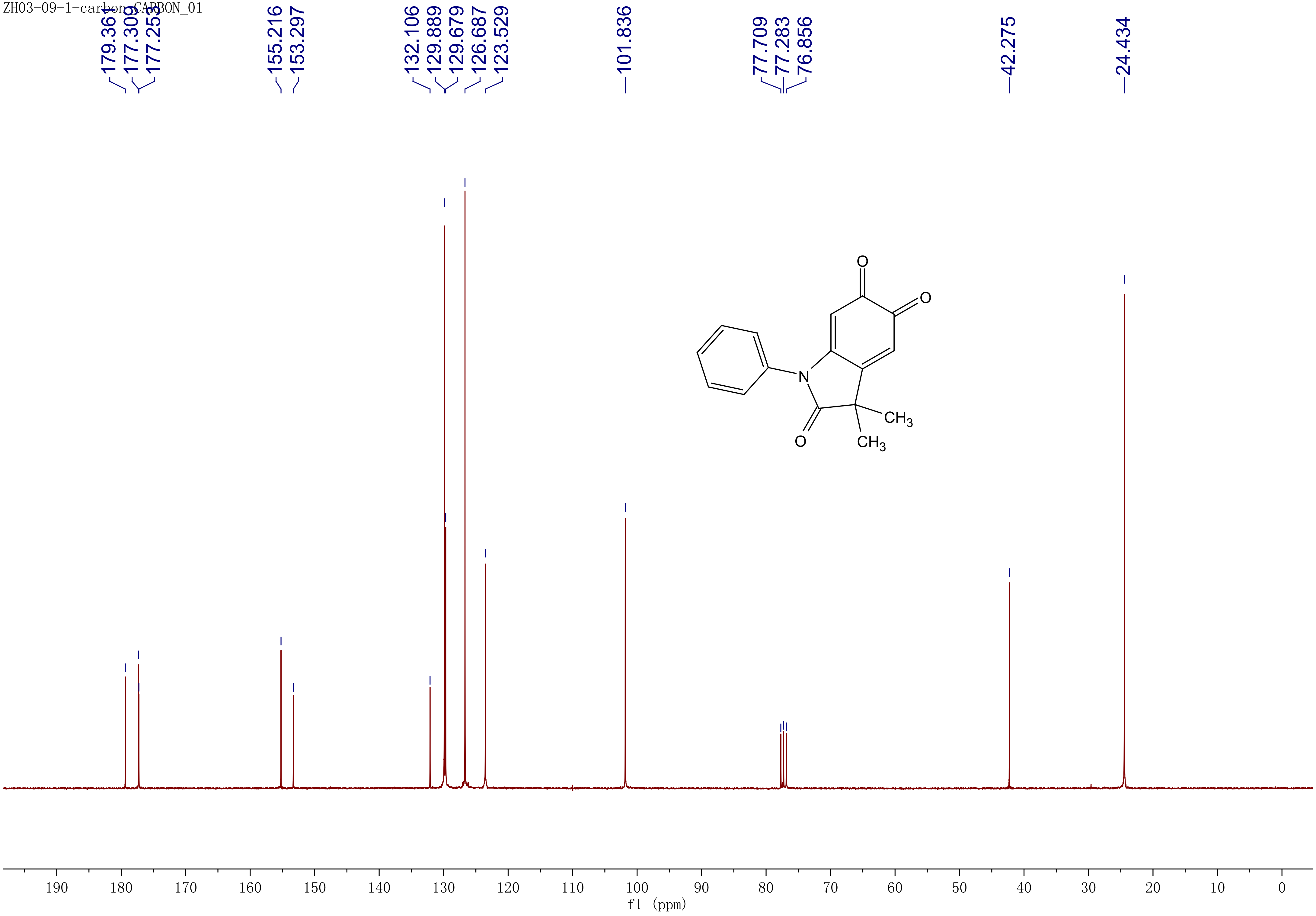
ZH03-09-1-carbon_CARBON_01
179.361
177.309
177.253
155.216
153.297
132.106
129.889
129.679
126.687
123.529
101.836
77.709
77.283
76.856
42.275
24.434
O
O
N
O
CH3
CH3
190 180 170 160 150 140 130 120 110 100 90 80 70 60 50 40 30 20 10 0
f1 (ppm)

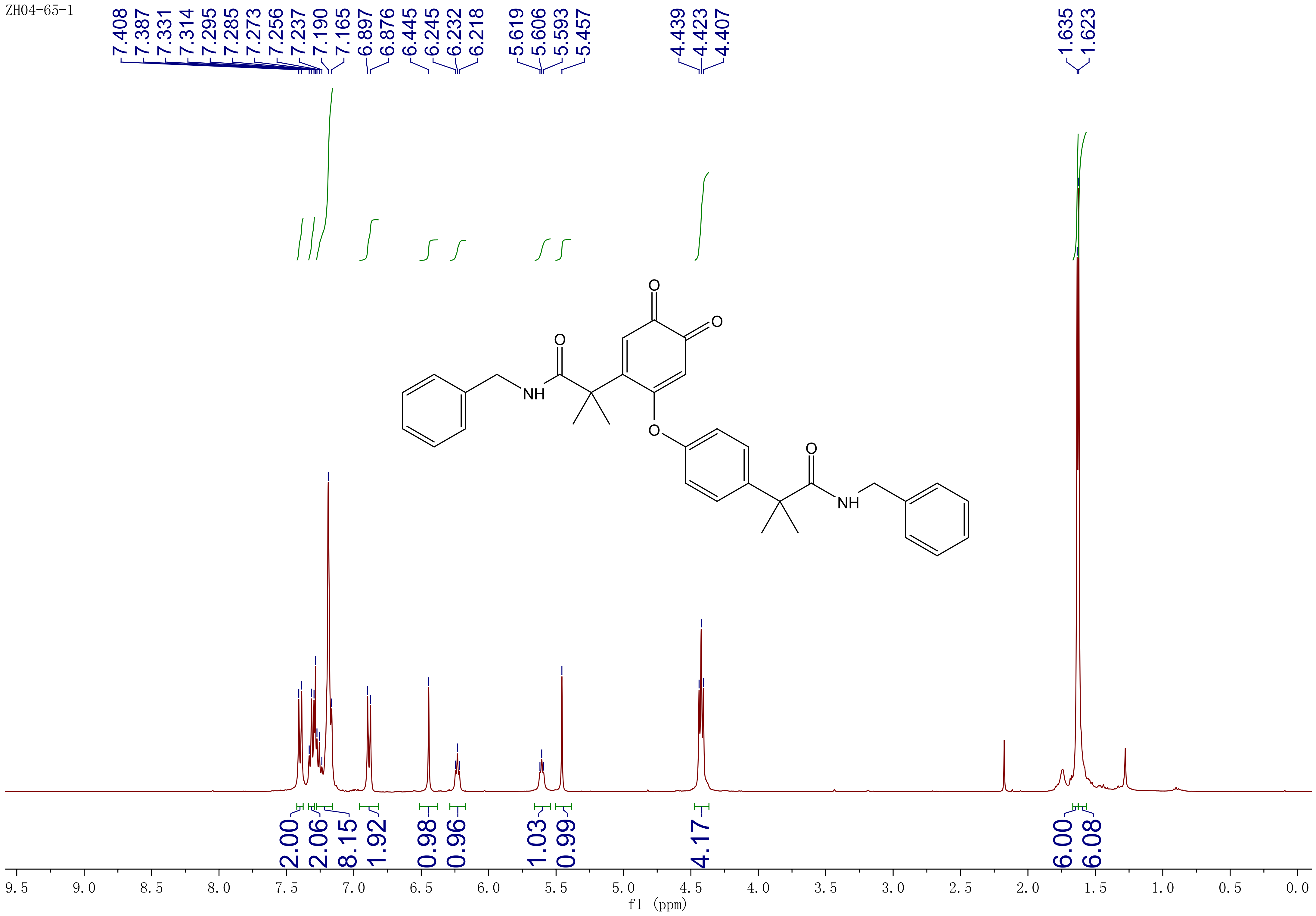

ZH04-65-1
7.408
7.387
7.331
7.314
7.295
7.285
7.273
7.256
7.237
7.190
7.165
6.897
6.876
6.445
6.245
6.232
6.218
5.619
5.606
5.593
5.457
4.439
4.423
4.407
1.635
1.623
O
O
O
NH
O
O
NH
2.00
2.06
8.15
1.92
0.98
0.96
1.03
0.99
4.17
6.00
6.08
9.5
9.0
8.5
8.0
7.5
7.0
6.5
6.0
5.5
5.0
4.5
4.0
3.5
3.0
2.5
2.0
1.5
1.0
0.5
0.0
f1 (ppm)

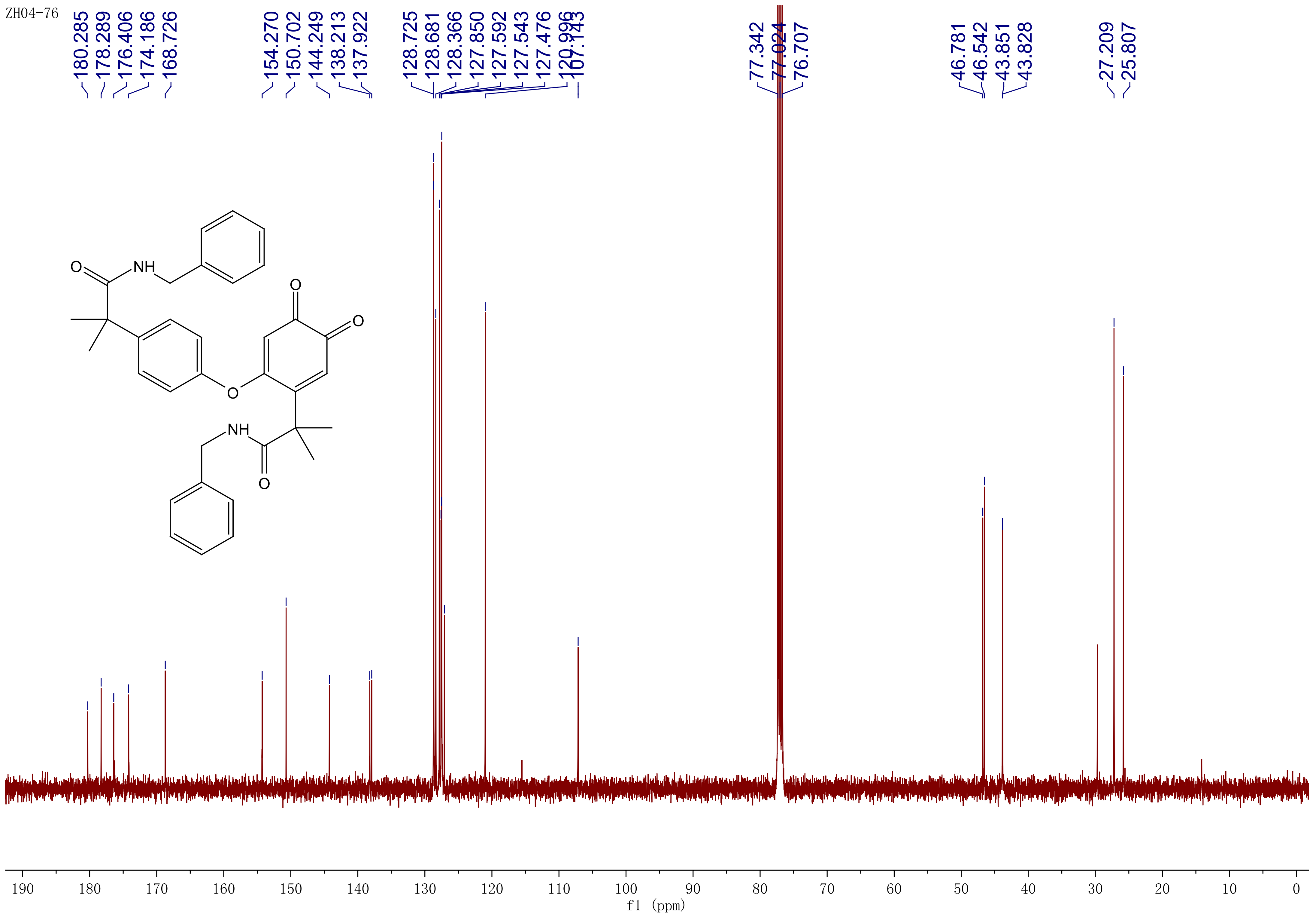
ZH04-76
180.285
178.289
176.406
174.186
168.726
154.270
150.702
144.249
138.213
137.922
128.725
128.681
128.366
127.850
127.592
127.543
127.476
120.996
107.143
77.342
77.024
76.707
46.781
46.542
43.851
43.828
27.209
25.807
O
NH
O
O
O
NH
O
190 180 170 160 150 140 130 120 110 100 90 80 70 60 50 40 30 20 10 0
f1 (ppm)

ZH04-60

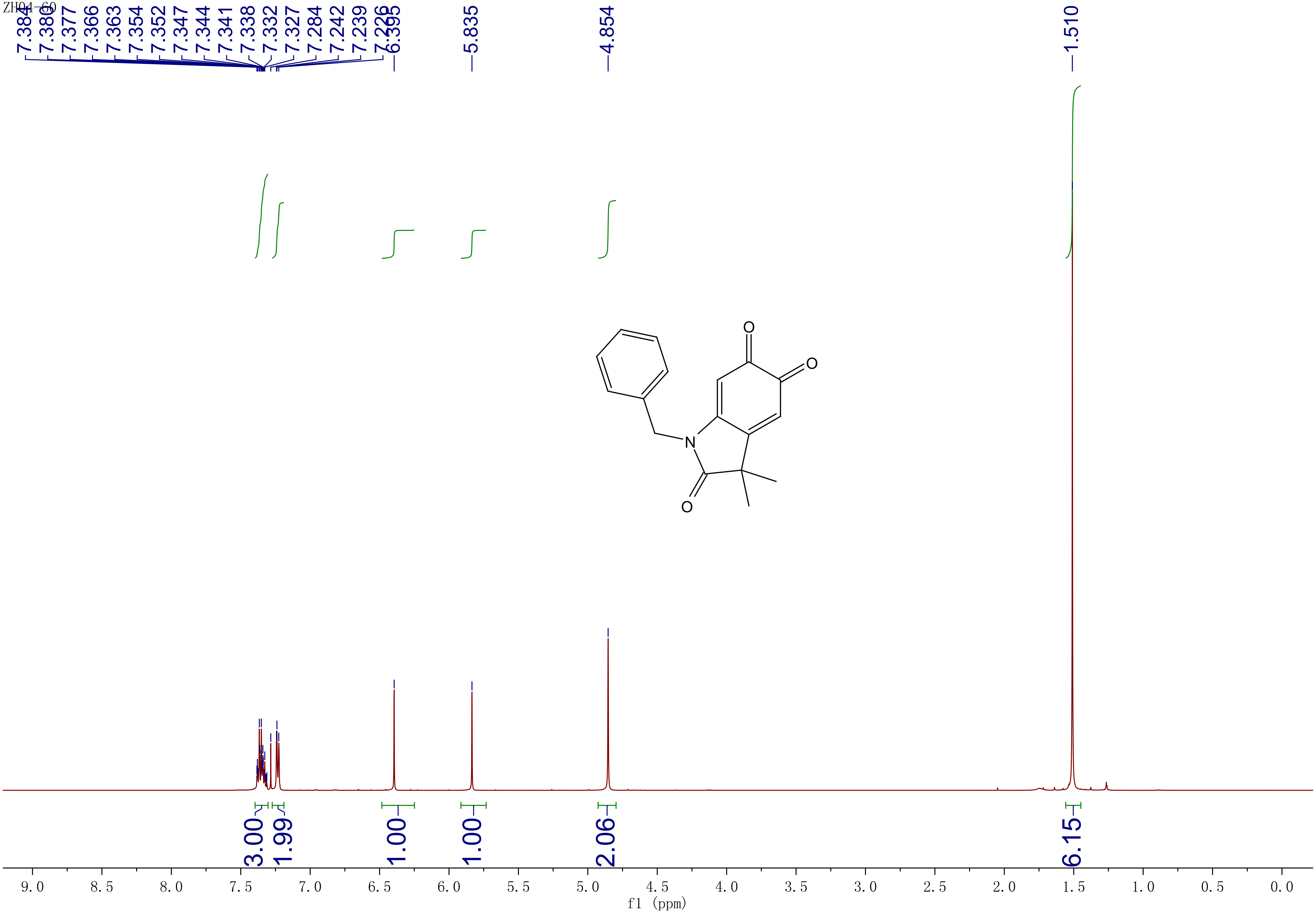

7.384
7.380
7.377
7.366
7.363
7.354
7.352
7.347
7.344
7.341
7.338
7.332
7.327
7.284
7.242
7.239
7.226
6.395
5.835
4.854
1.510
3.00
1.99
1.00
1.00
2.06
6.15
9.0
8.5
8.0
7.5
7.0
6.5
6.0
5.5
5.0
4.5
4.0
3.5
3.0
2.5
2.0
1.5
1.0
0.5
0.0
f1 (ppm)
O
O
N
O

ZH04-60

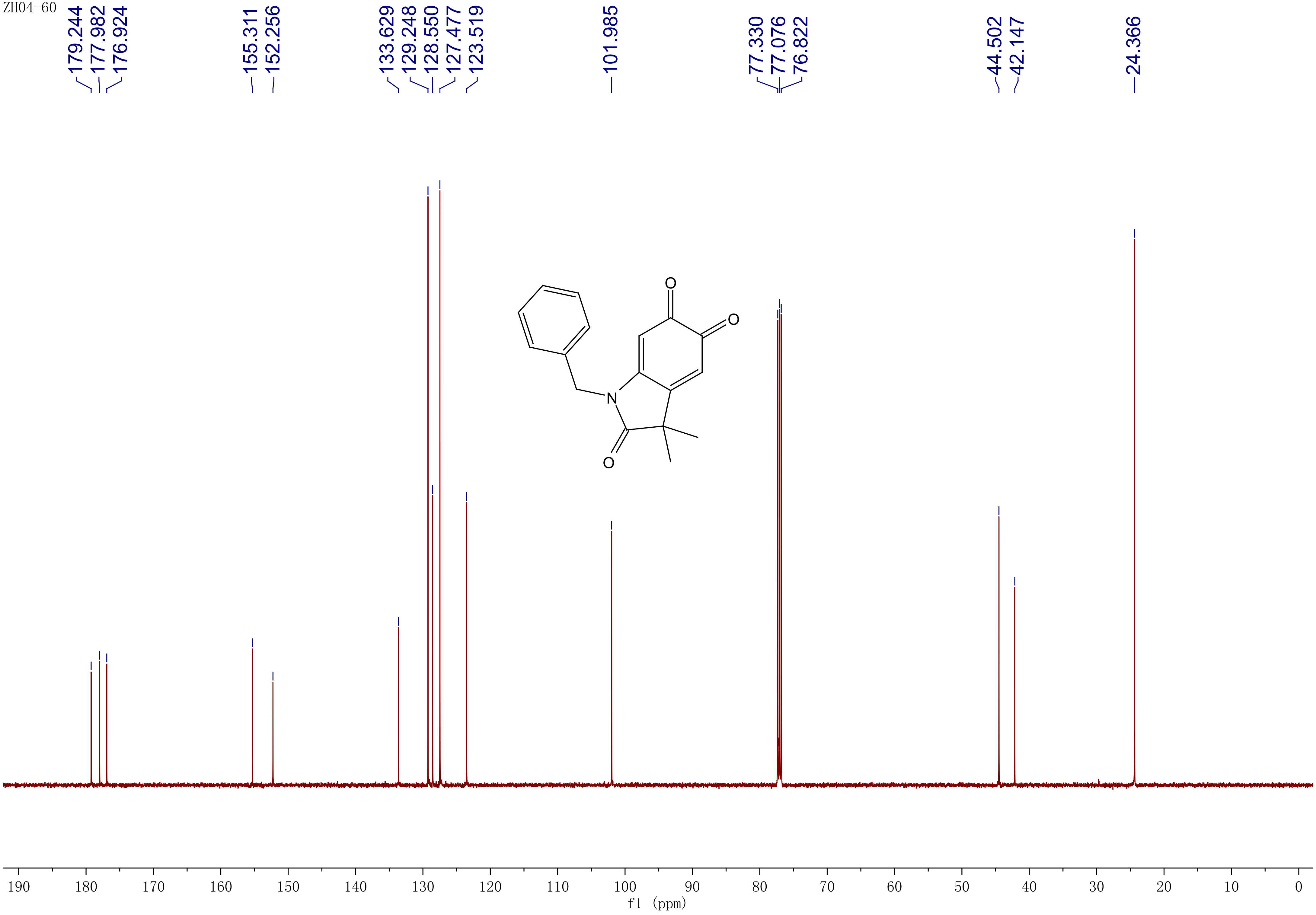

179.244
177.982
176.924
155.311
152.256
133.629
129.248
128.550
127.477
123.519
101.985
77.330
77.076
76.822
44.502
42.147
24.366
O
O
N
O
190 180 170 160 150 140 130 120 110 100 90 80 70 60 50 40 30 20 10 0
f1 (ppm)

proton
STANDARD PROTON PARAMETERS

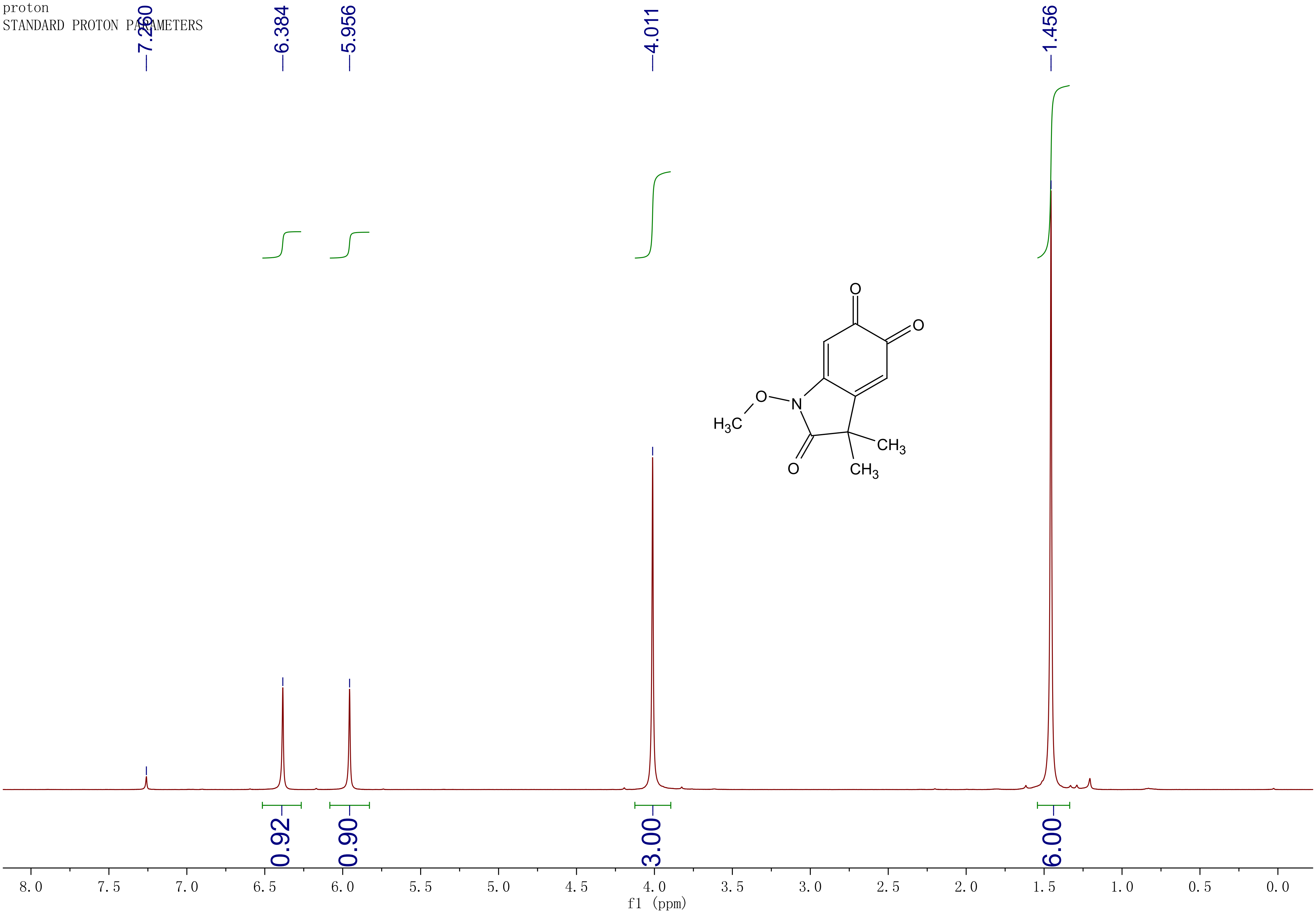

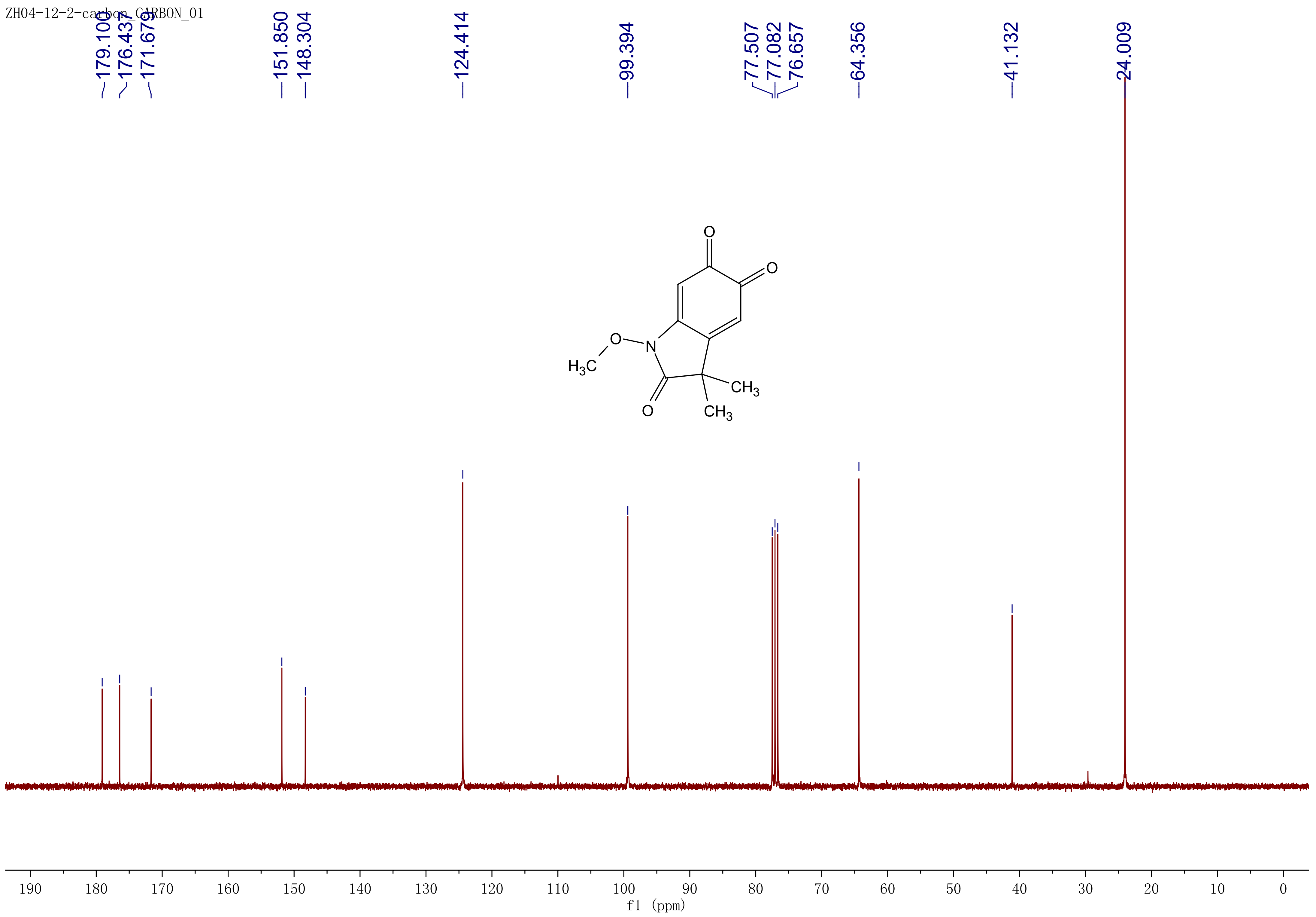

ZH04-12-2-carbon_CARBON_01
179.100
176.437
171.679
151.850
148.304
124.414
99.394
77.507
77.082
76.657
64.356
41.132
24.009
O
O
O
N
O
H3C
CH3
CH3
190 180 170 160 150 140 130 120 110 100 90 80 70 60 50 40 30 20 10 0
f1 (ppm)

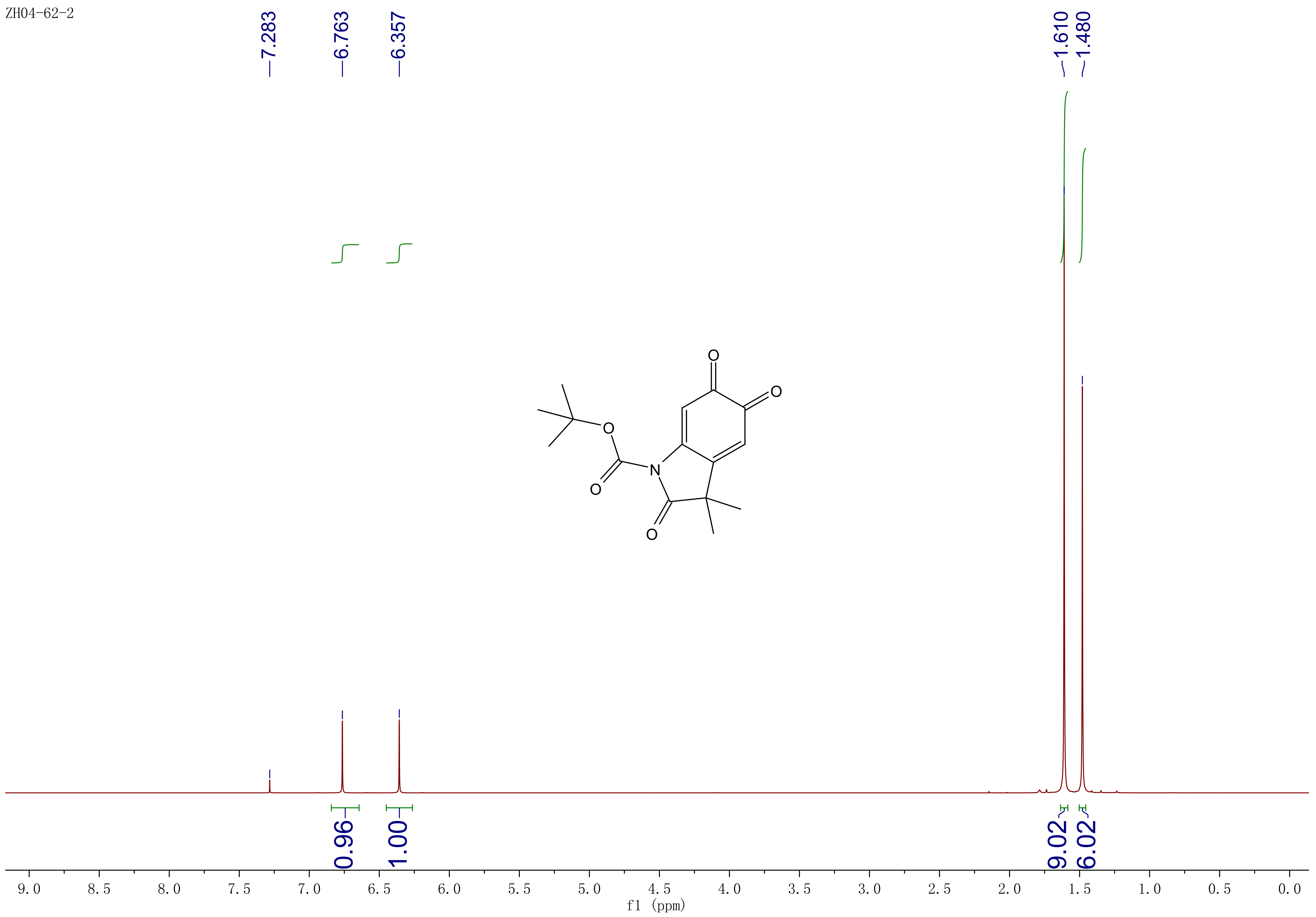
ZH04-62-2
7.283
6.763
6.357
1.610
1.480
0.96
1.00
9.02
6.02
9.0 8.5 8.0 7.5 7.0 6.5 6.0 5.5 5.0 4.5 4.0 3.5 3.0 2.5 2.0 1.5 1.0 0.5 0.0
f1 (ppm)
O
O
O
O
O
N
O

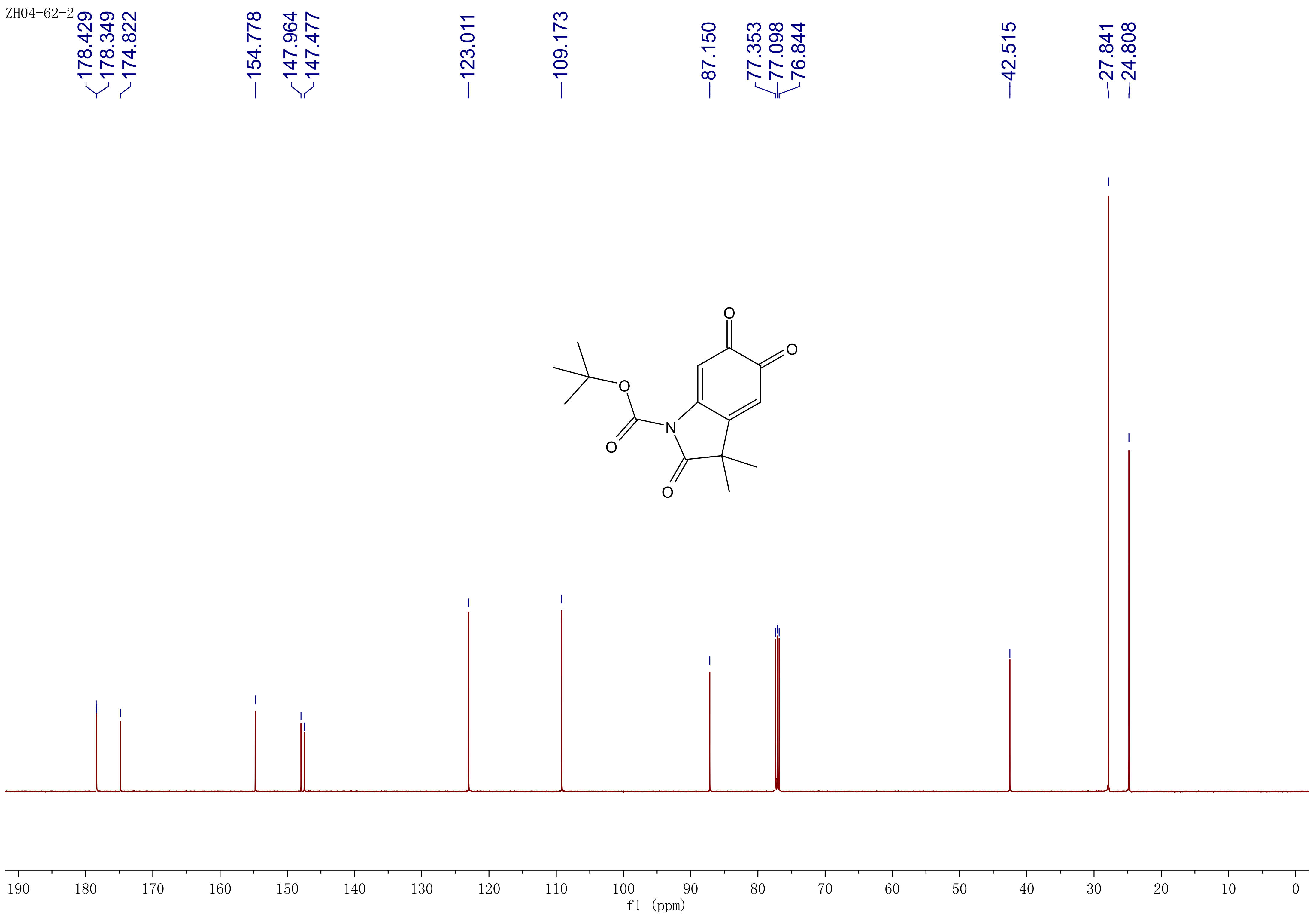
ZH04-62-2
178.429
178.349
174.822
154.778
147.964
147.477
123.011
109.173
87.150
77.353
77.098
76.844
42.515
27.841
24.808
O
O
O
O
O
N
190
180
170
160
150
140
130
120
110
100
90
80
70
60
50
40
30
20
10
0
f1 (ppm)

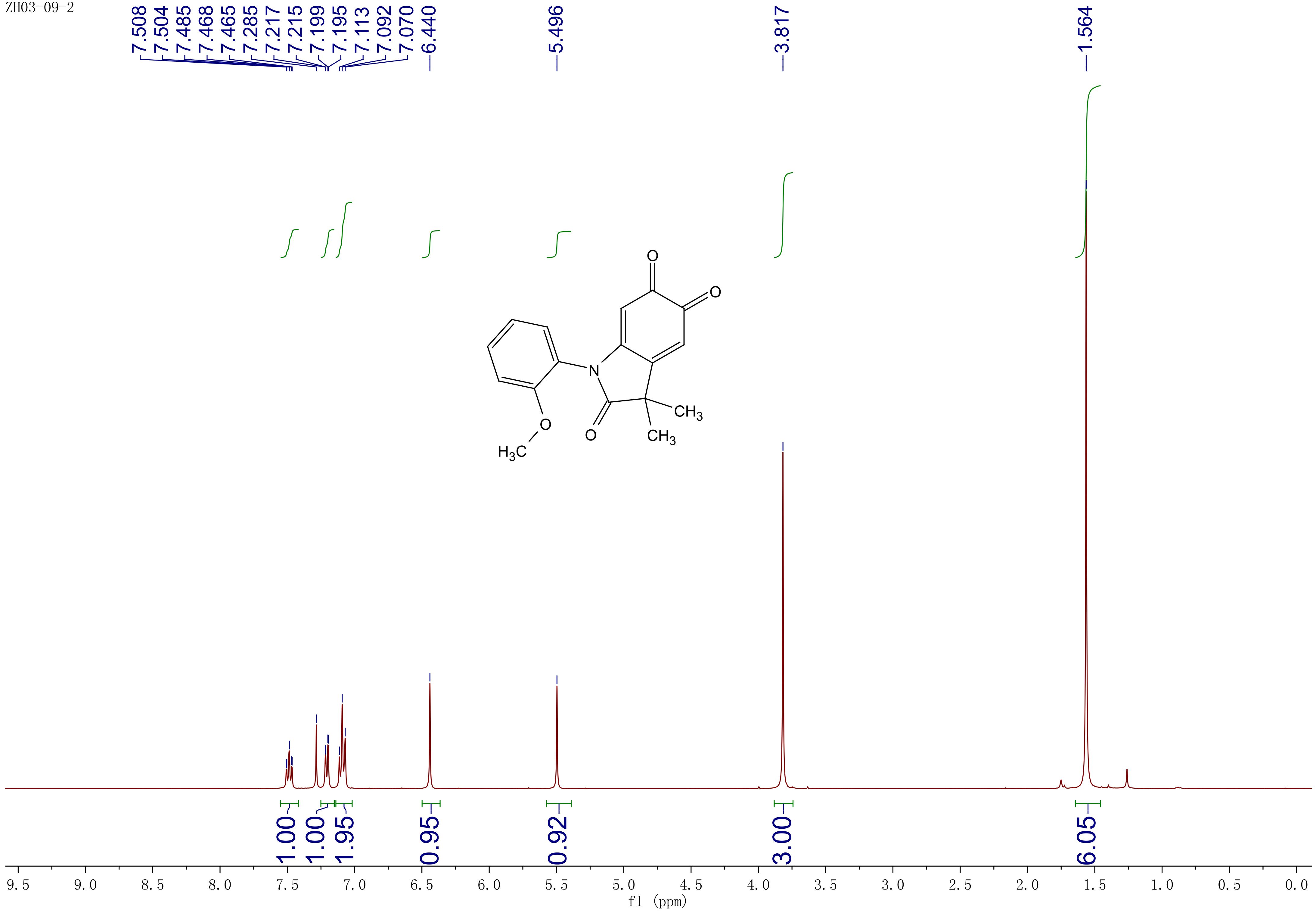

ZH03-09-2
7.508
7.504
7.485
7.468
7.465
7.285
7.217
7.215
7.199
7.195
7.113
7.092
7.070
6.440
5.496
3.817
1.564
1.00
1.00
1.95
0.95
0.92
3.00
6.05
9.5
9.0
8.5
8.0
7.5
7.0
6.5
6.0
5.5
5.0
4.5
4.0
3.5
3.0
2.5
2.0
1.5
1.0
0.5
0.0
f1 (ppm)

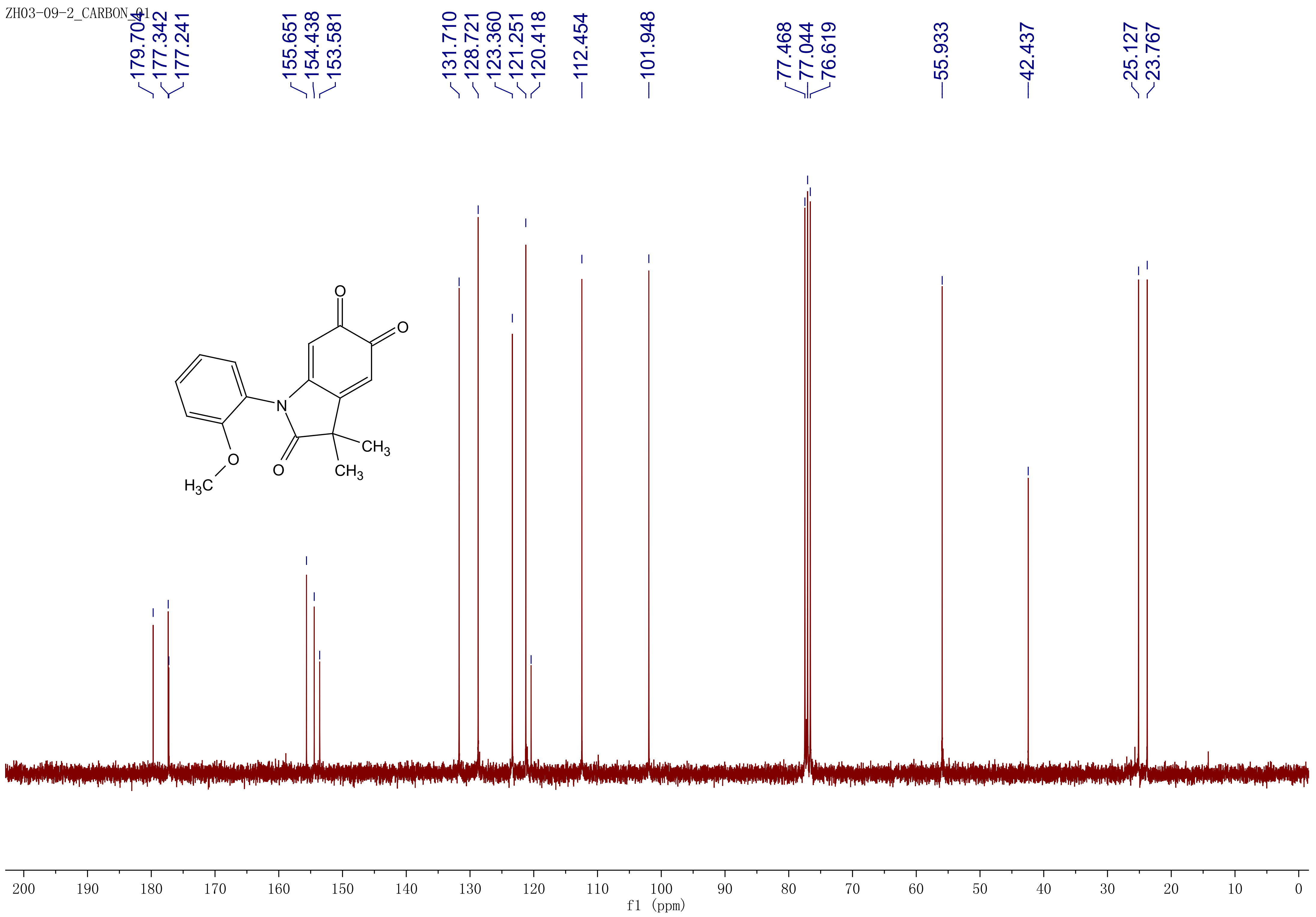
ZH03-09-2_CARBON_01
179.704
177.342
177.241
155.651
154.438
153.581
131.710
128.721
123.360
121.251
120.418
112.454
101.948
77.468
77.044
76.619
55.933
42.437
25.127
23.767
O
O
N
CH3
CH3
O
O
H3C
200 190 180 170 160 150 140 130 120 110 100 90 80 70 60 50 40 30 20 10 0
f1 (ppm)

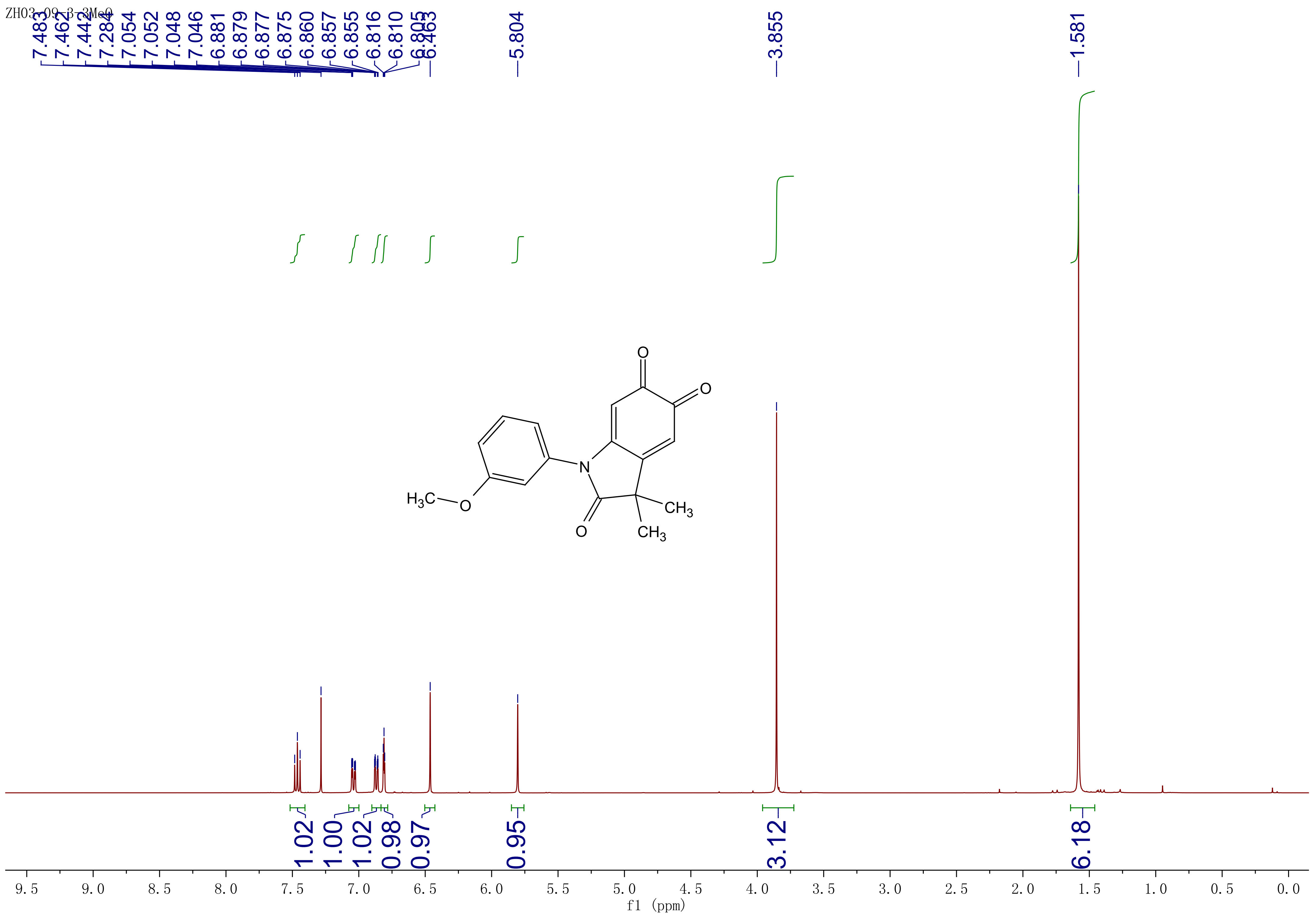
ZH03-09-3-3MeO
7.483
7.462
7.442
7.284
7.054
7.052
7.048
7.046
6.881
6.879
6.877
6.875
6.860
6.857
6.855
6.816
6.810
6.805
6.463
5.804
3.855
1.581
H3C
O
N
O
O
O
CH3
CH3
1.02
1.00
1.02
0.98
0.97
0.95
3.12
6.18
9.5
9.0
8.5
8.0
7.5
7.0
6.5
6.0
5.5
5.0
4.5
4.0
3.5
3.0
2.5
2.0
1.5
1.0
0.5
0.0
f1 (ppm)

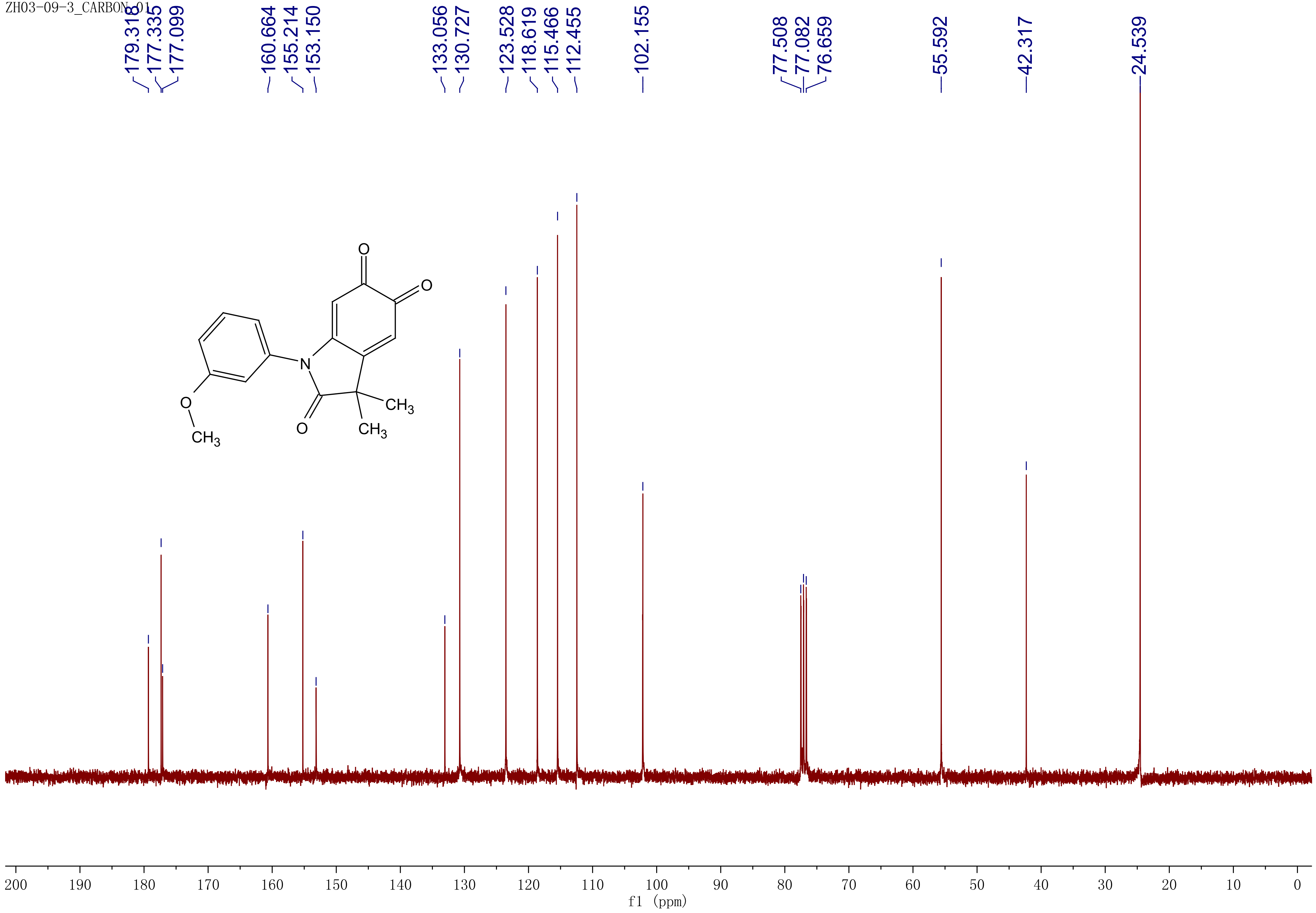
ZH03-09-3_CARBON_01
179.318
177.335
177.099
160.664
155.214
153.150
133.056
130.727
123.528
118.619
115.466
112.455
102.155
77.508
77.082
76.659
55.592
42.317
24.539
O
O
N
O
CH3
CH3
O
CH3
200 190 180 170 160 150 140 130 120 110 100 90 80 70 60 50 40 30 20 10 0
f1 (ppm)

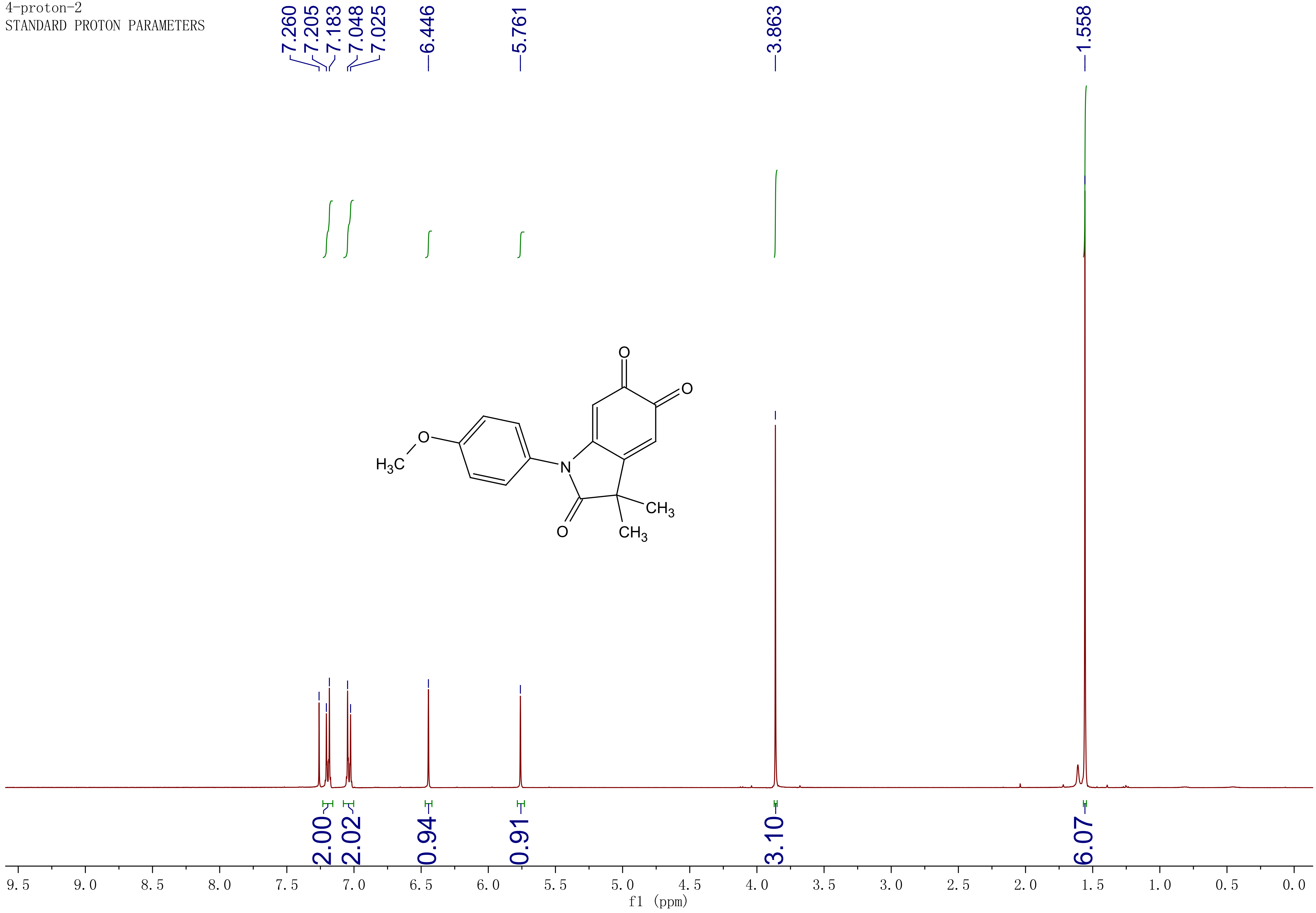
4-proton-2
STANDARD PROTON PARAMETERS
7.260
7.205
7.183
7.048
7.025
6.446
5.761
3.863
1.558
O
O
H3C
O
N
CH3
CH3
O
2.00
2.02
0.94
0.91
3.10
6.07
9.5
9.0
8.5
8.0
7.5
7.0
6.5
6.0
5.5
5.0
4.5
4.0
3.5
3.0
2.5
2.0
1.5
1.0
0.5
0.0
f1 (ppm)

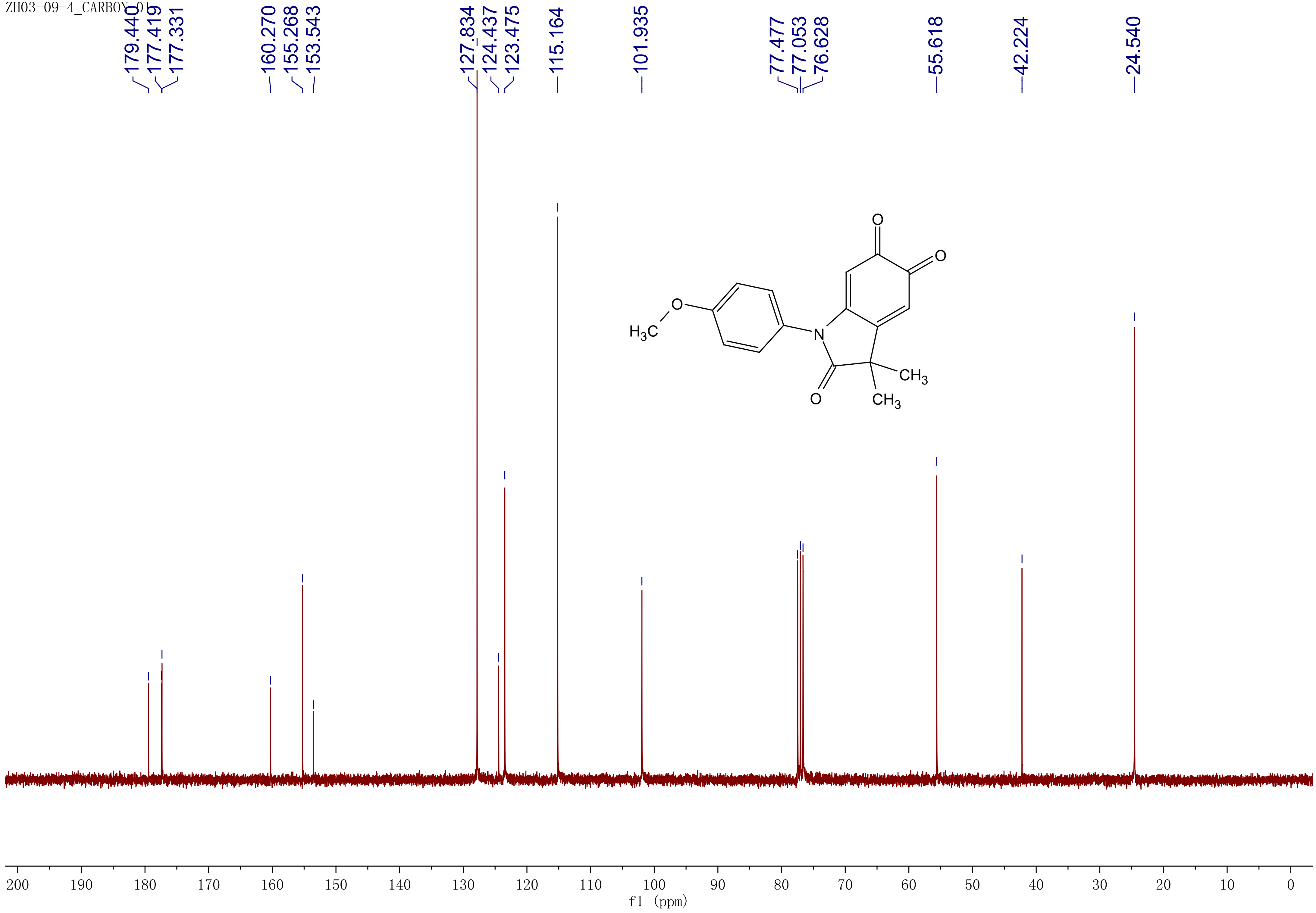
ZH03-09-4_CARBON_01
179.440
177.419
177.331
160.270
155.268
153.543
127.834
124.437
123.475
115.164
101.935
77.477
77.053
76.628
55.618
42.224
24.540
H3C
O
N
O
O
O
CH3
CH3
200 190 180 170 160 150 140 130 120 110 100 90 80 70 60 50 40 30 20 10 0
f1 (ppm)

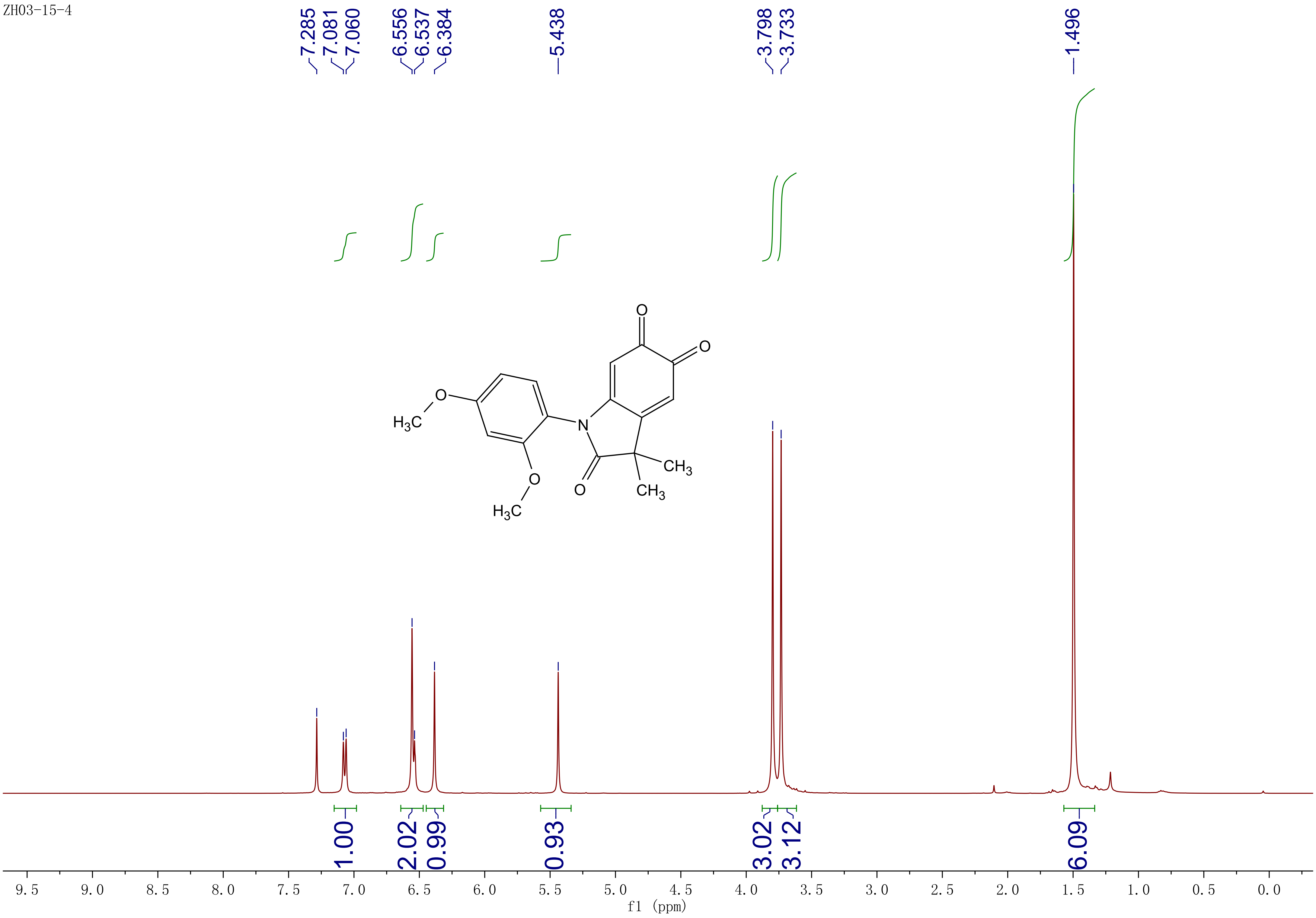
ZH03-15-4
7.285
7.081
7.060
6.556
6.537
6.384
5.438
3.798
3.733
1.496
O
O
H3C
O
N
CH3
CH3
O
O
H3C
1.00
2.02
0.99
0.93
3.02
3.12
6.09
9.5 9.0 8.5 8.0 7.5 7.0 6.5 6.0 5.5 5.0 4.5 4.0 3.5 3.0 2.5 2.0 1.5 1.0 0.5 0.0
f1 (ppm)

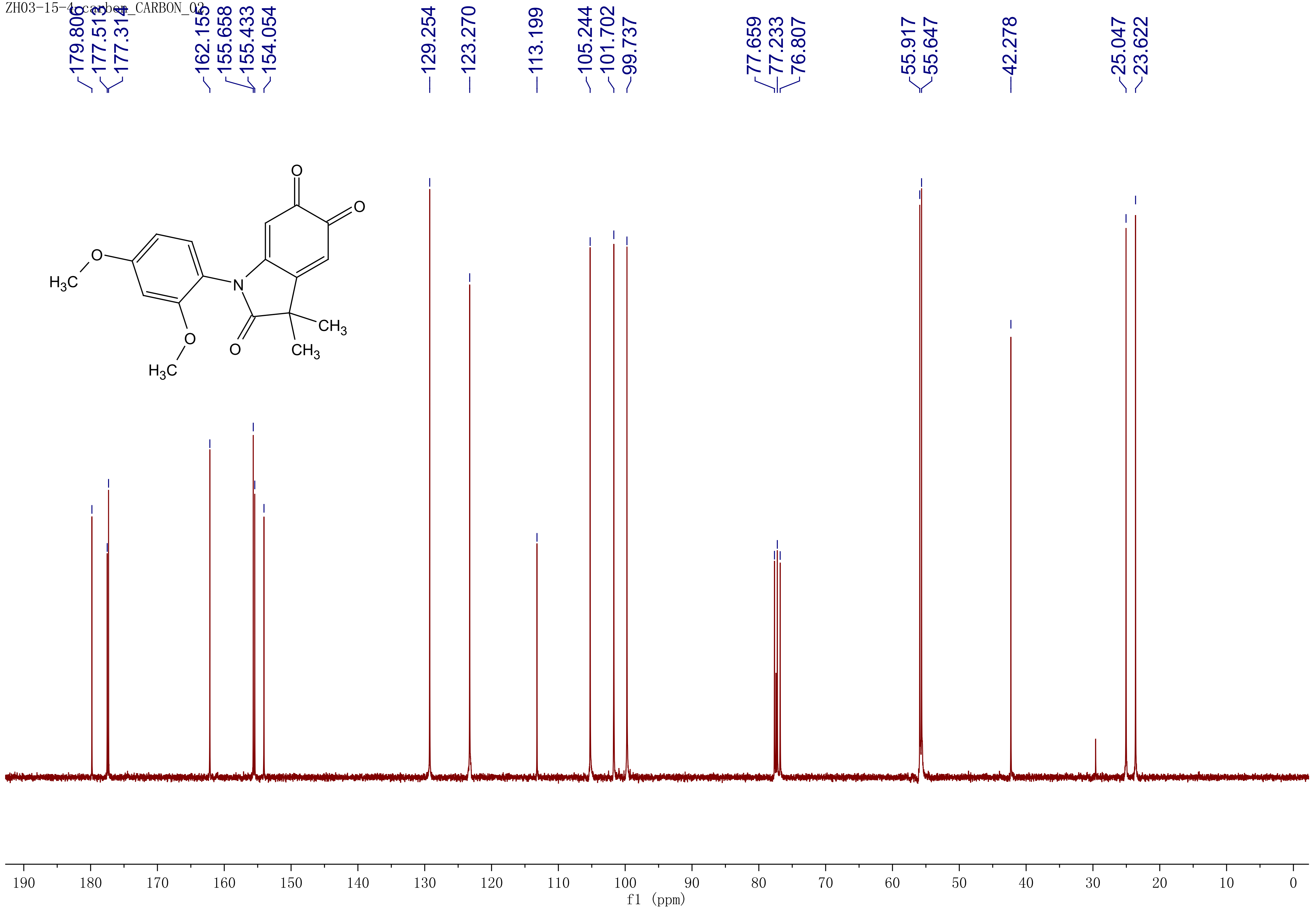
ZH03-15-4-carbon_CARBON_02
179.806
177.51
177.314
162.155
155.658
155.433
154.054
129.254
123.270
113.199
105.244
101.702
99.737
77.659
77.233
76.807
55.917
55.647
42.278
25.047
23.622
O
O
O
H3C
N
CH3
CH3
O
O
H3C
190 180 170 160 150 140 130 120 110 100 90 80 70 60 50 40 30 20 10 0
f1 (ppm)

1-proton
STANDARD PROTON PARAMETERS

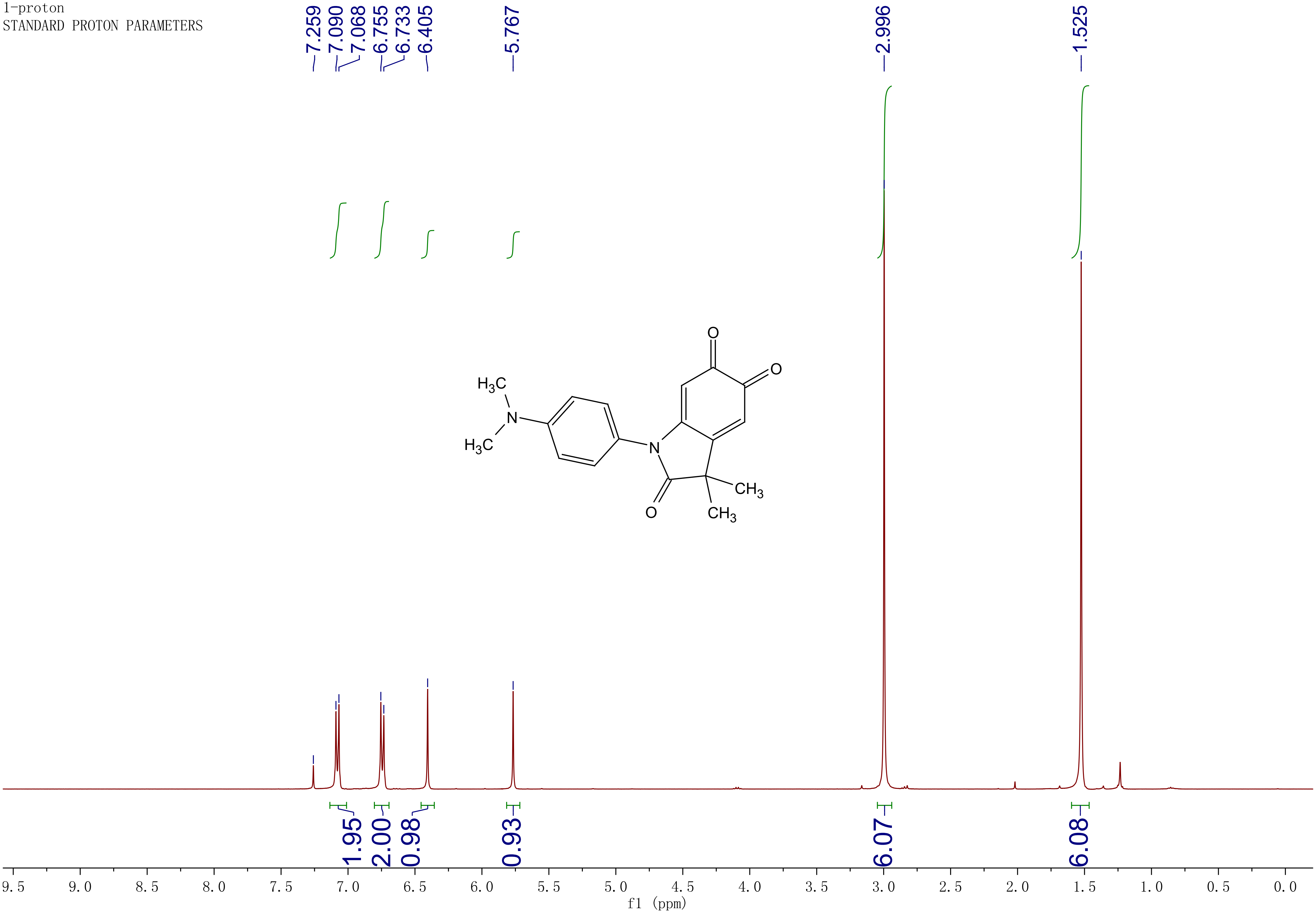

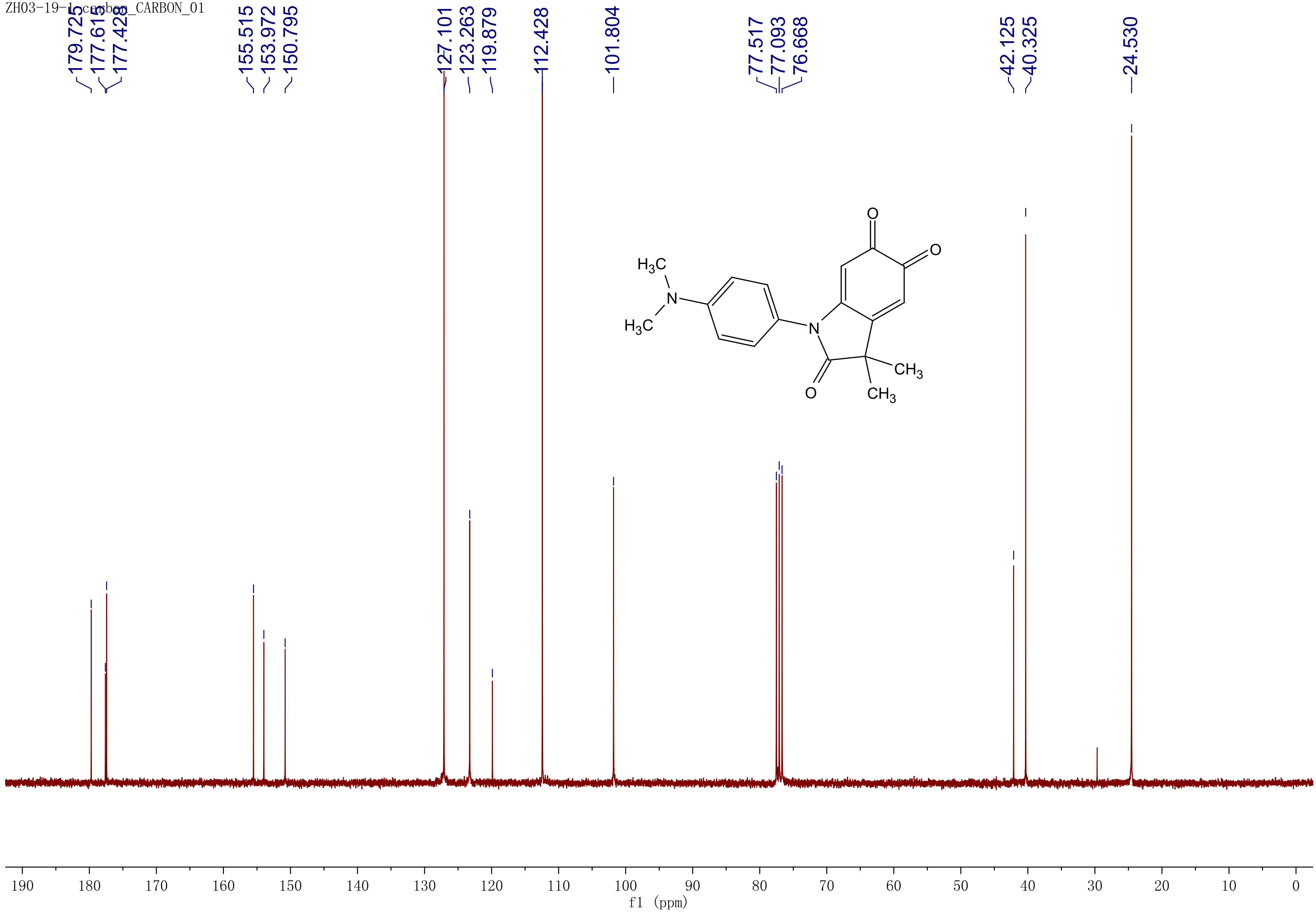
ZH03-19-1_carbon_CARBON_01
179.725
177.615
177.428
155.515
153.972
150.795
127.101
123.263
119.879
112.428
101.804
77.517
77.093
76.668
42.125
40.325
24.530
H3C
N
H3C
N
O
O
O
CH3
CH3
190 180 170 160 150 140 130 120 110 100 90 80 70 60 50 40 30 20 10 0
f1 (ppm)

1-proton
STANDARD PROTON PARAMETERS

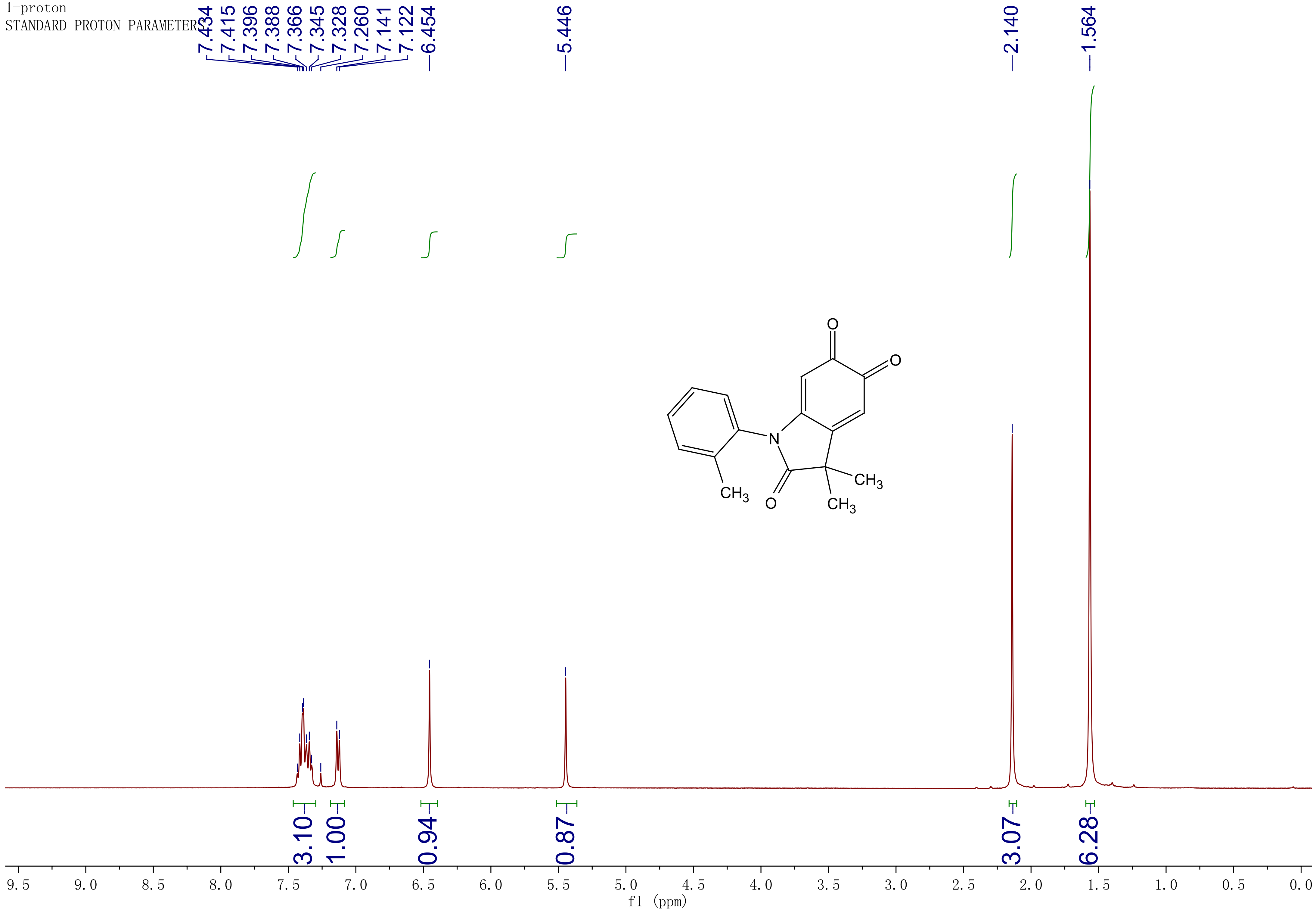

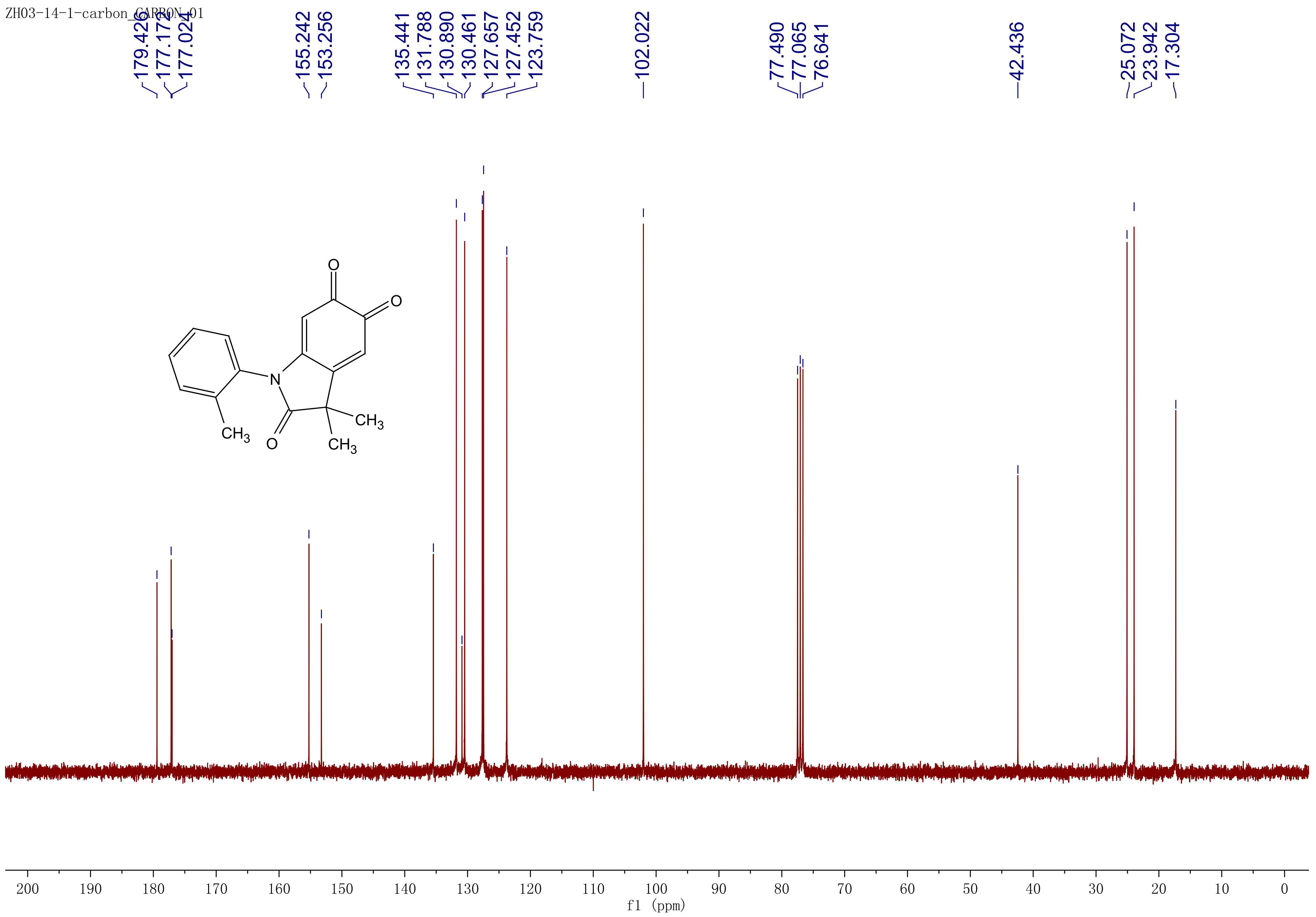
ZH03-14-1-carbon_CARBON_01
179.426
177.172
177.024
155.242
153.256
135.441
131.788
130.890
130.461
127.657
127.452
123.759
102.022
77.490
77.065
76.641
42.436
25.072
23.942
17.304
O
O
N
CH3
CH3
CH3
O
200 190 180 170 160 150 140 130 120 110 100 90 80 70 60 50 40 30 20 10 0
f1 (ppm)

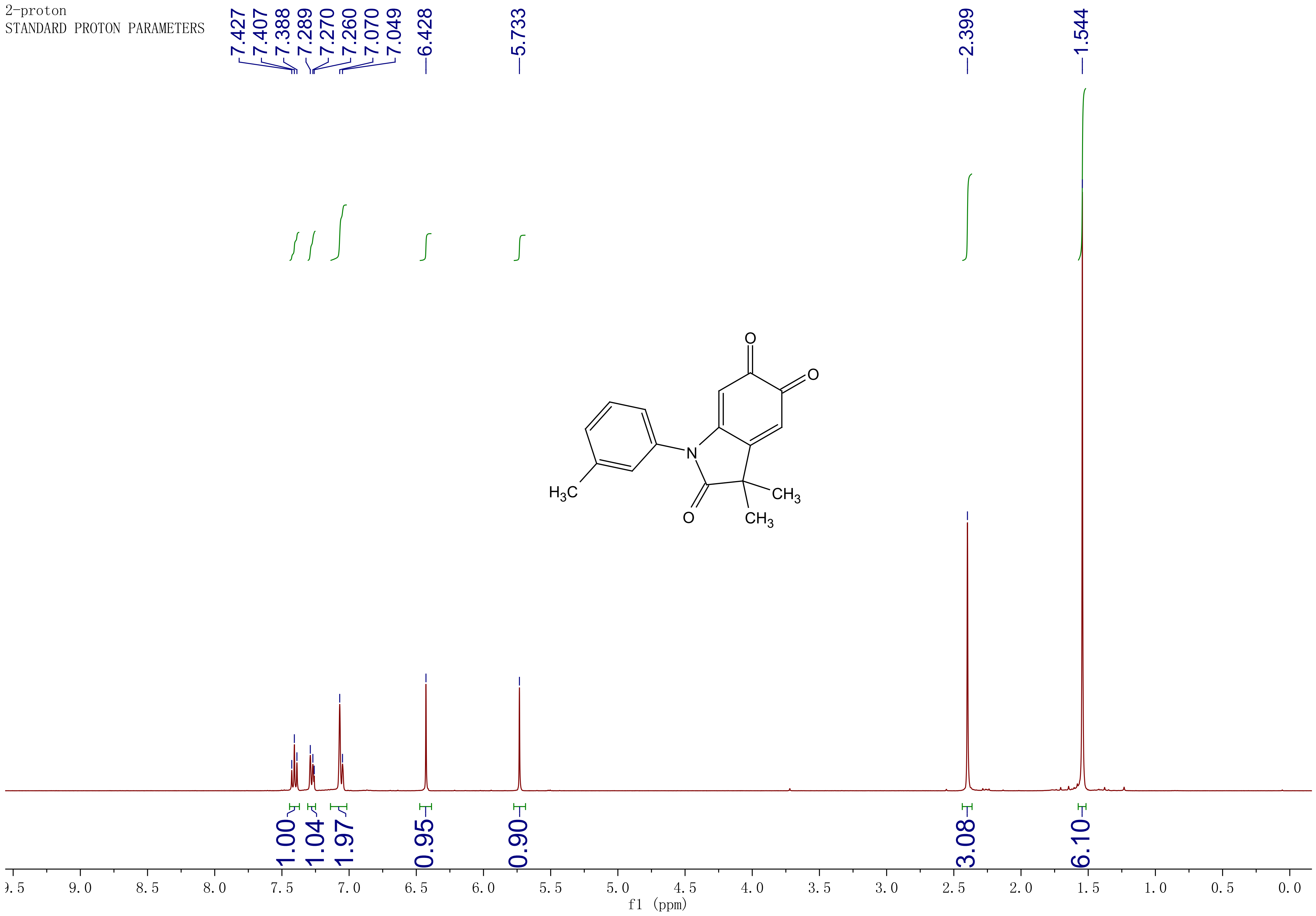
2-proton
STANDARD PROTON PARAMETERS
7.427
7.407
7.388
7.289
7.270
7.260
7.070
7.049
6.428
5.733
2.399
1.544
1.00
1.04
1.97
0.95
0.90
3.08
6.10
O
O
N
O
H3C
CH3
CH3
9.0
8.5
8.0
7.5
7.0
6.5
6.0
5.5
5.0
4.5
4.0
3.5
3.0
2.5
2.0
1.5
1.0
0.5
0.0
f1 (ppm)

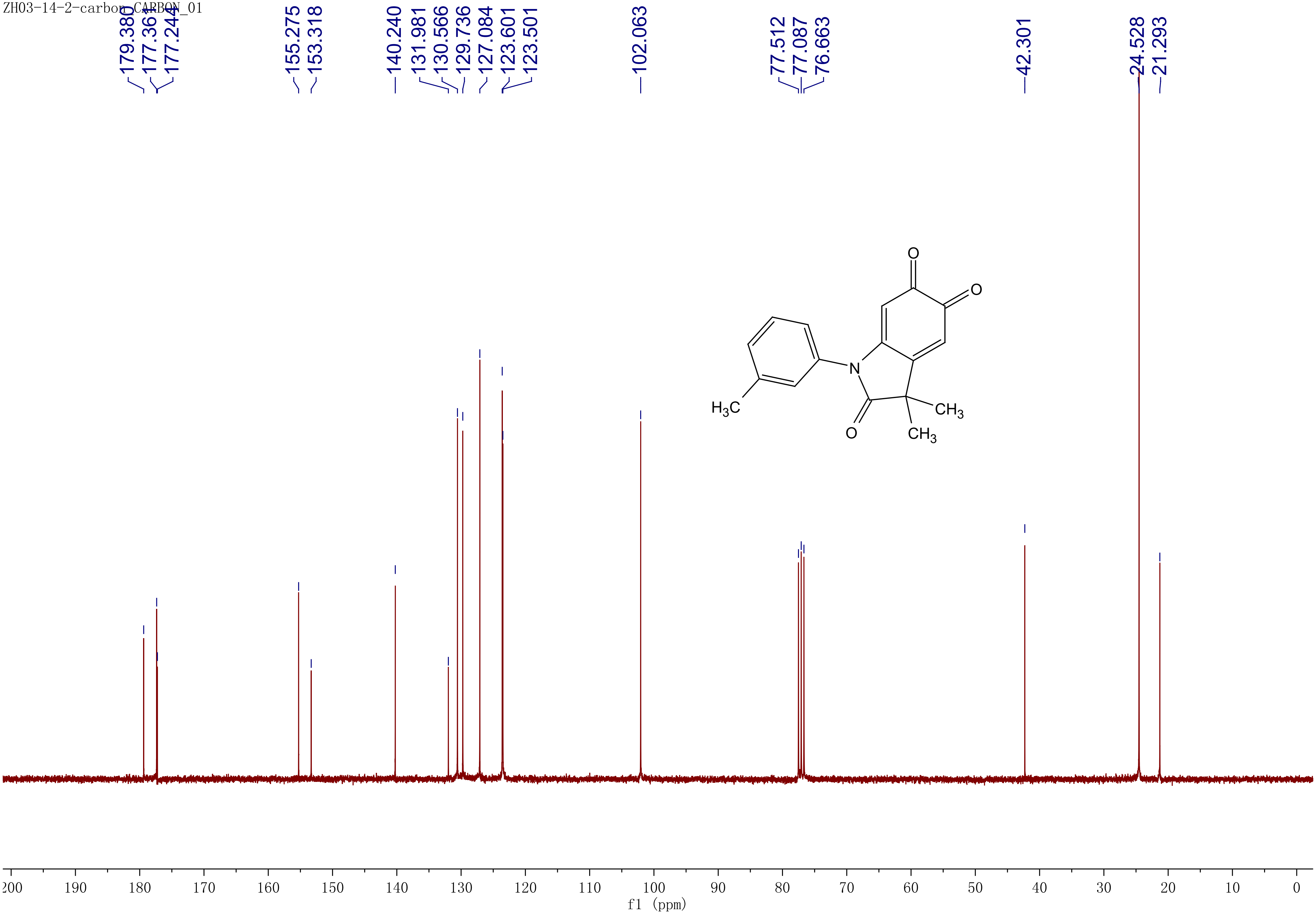
ZH03-14-2-carbon_CARBON_01
179.380
177.361
177.244
155.275
153.318
140.240
131.981
130.566
129.736
127.084
123.601
123.501
102.063
77.512
77.087
76.663
42.301
24.528
21.293
O
O
N
H3C
O
CH3
CH3
200 190 180 170 160 150 140 130 120 110 100 90 80 70 60 50 40 30 20 10 0
f1 (ppm)

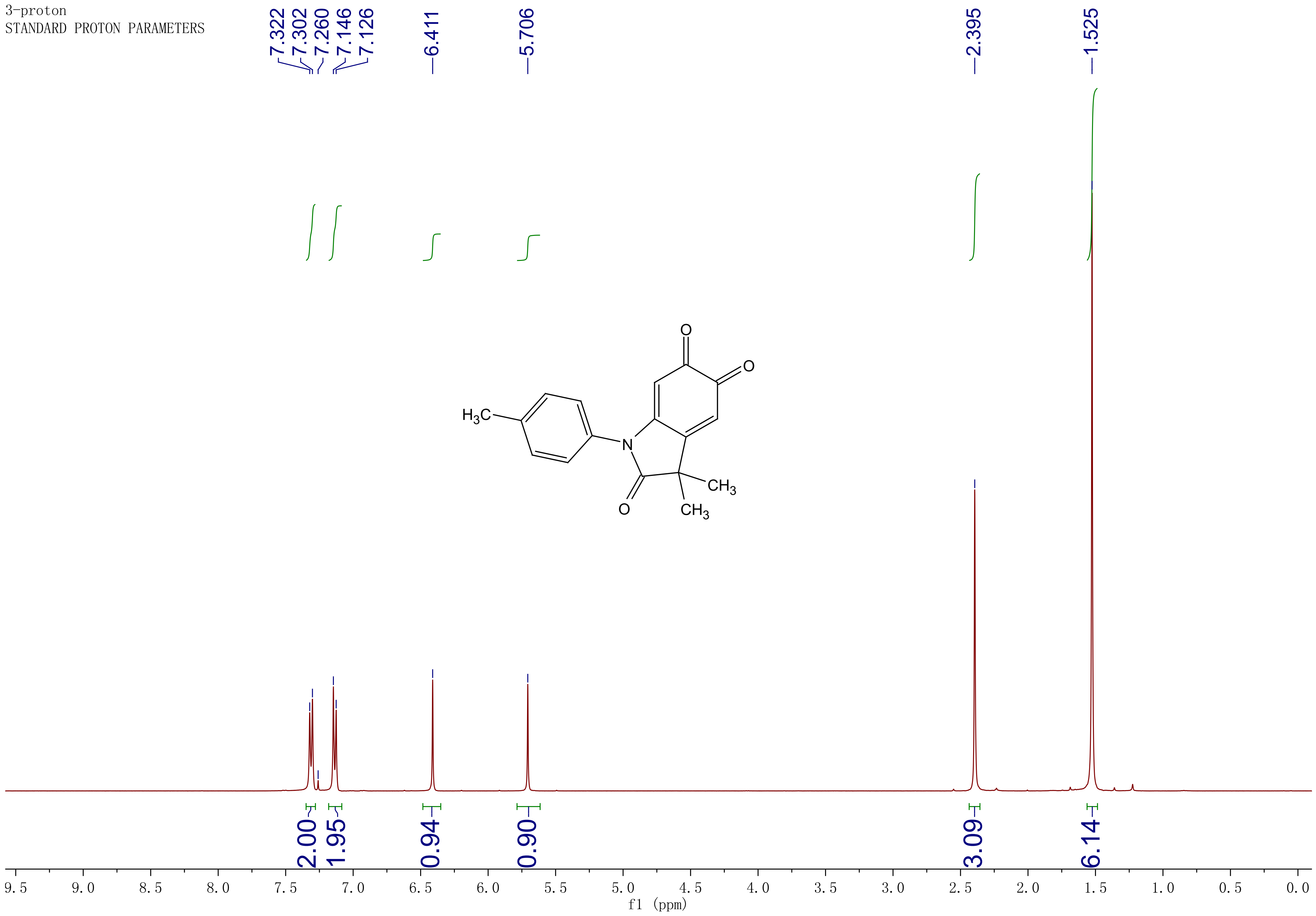
3-proton
STANDARD PROTON PARAMETERS
7.322
7.302
7.260
7.146
7.126
6.411
5.706
2.395
1.525
H3C
N
O
O
O
CH3
CH3
2.00
1.95
0.94
0.90
3.09
6.14
9.5
9.0
8.5
8.0
7.5
7.0
6.5
6.0
5.5
5.0
4.5
4.0
3.5
3.0
2.5
2.0
1.5
1.0
0.5
0.0
f1 (ppm)

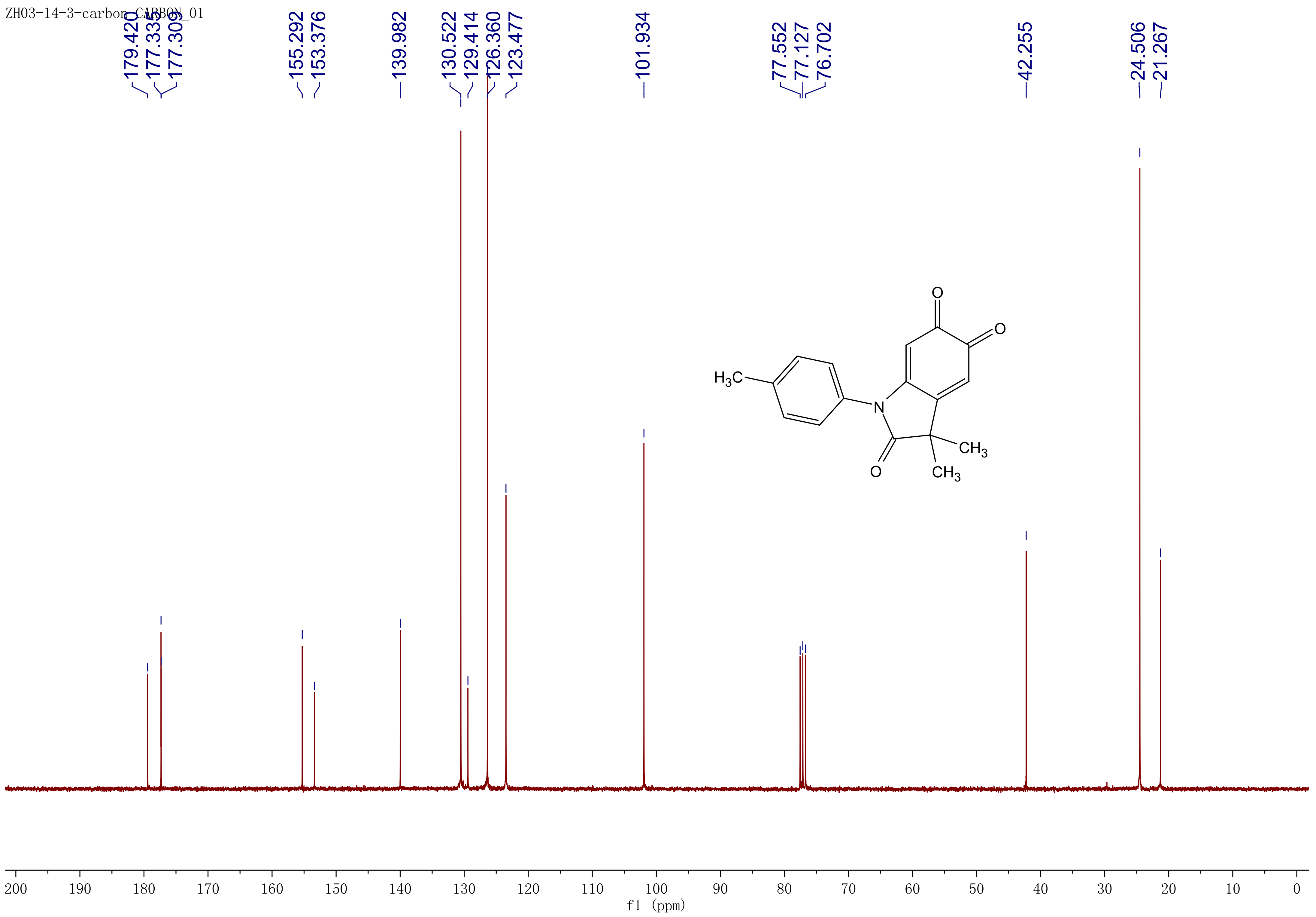
ZH03-14-3-carbon_CARBON_01
179.420
177.335
177.309
155.292
153.376
139.982
130.522
129.414
126.360
123.477
101.934
77.552
77.127
76.702
42.255
24.506
21.267
O
O
$H_3C$
N
$CH_3$
O
$CH_3$
200 190 180 170 160 150 140 130 120 110 100 90 80 70 60 50 40 30 20 10 0
f1 (ppm)

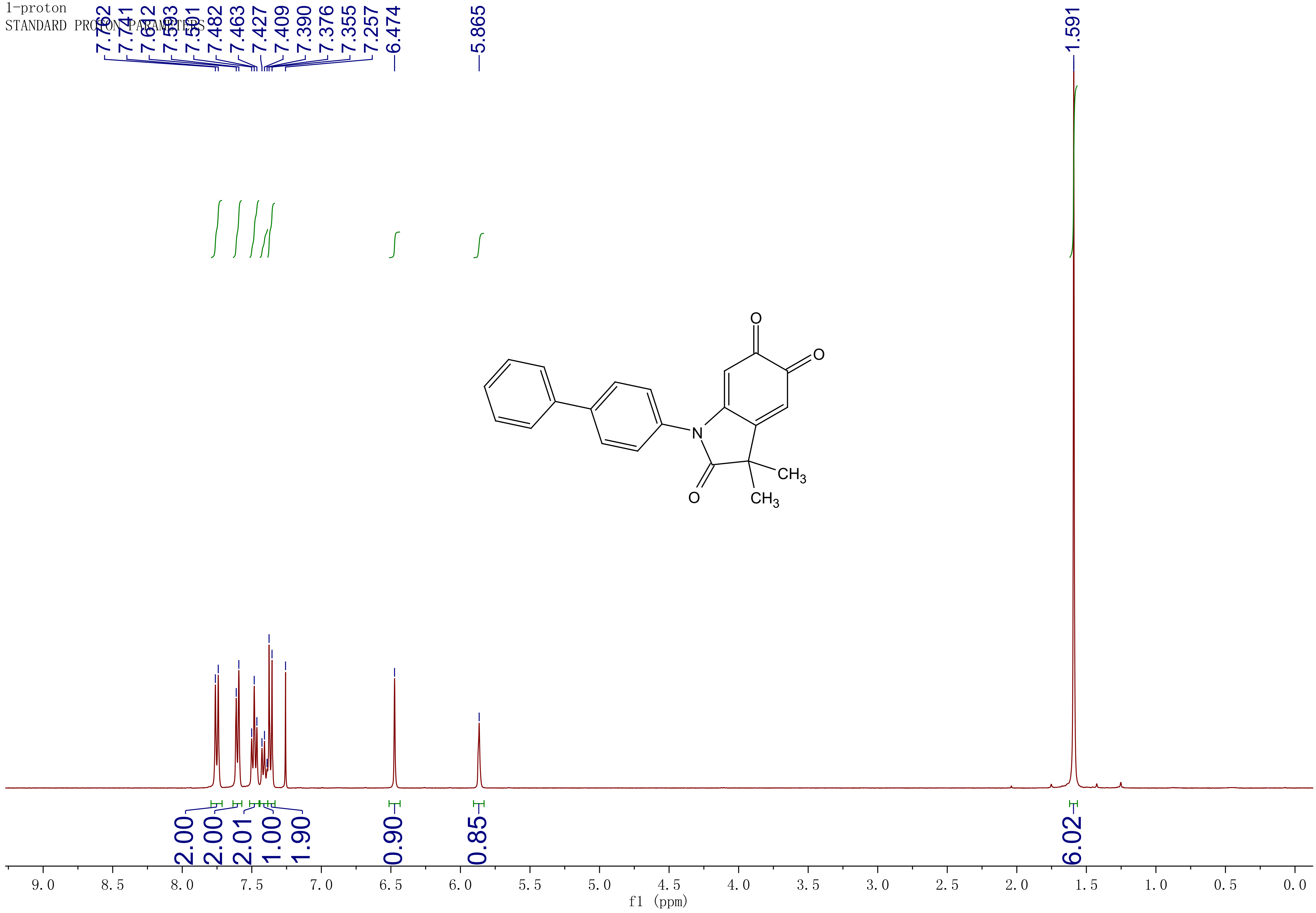
1-proton
STANDARD PROTON PARAMETERS
7.762
7.741
7.612
7.593
7.501
7.482
7.463
7.427
7.409
7.390
7.376
7.355
7.257
6.474
5.865
1.591
O
O
N
O
CH3
CH3
2.00
2.00
2.01
1.00
1.90
0.90
0.85
6.02
9.0 8.5 8.0 7.5 7.0 6.5 6.0 5.5 5.0 4.5 4.0 3.5 3.0 2.5 2.0 1.5 1.0 0.5 0.0
f1 (ppm)

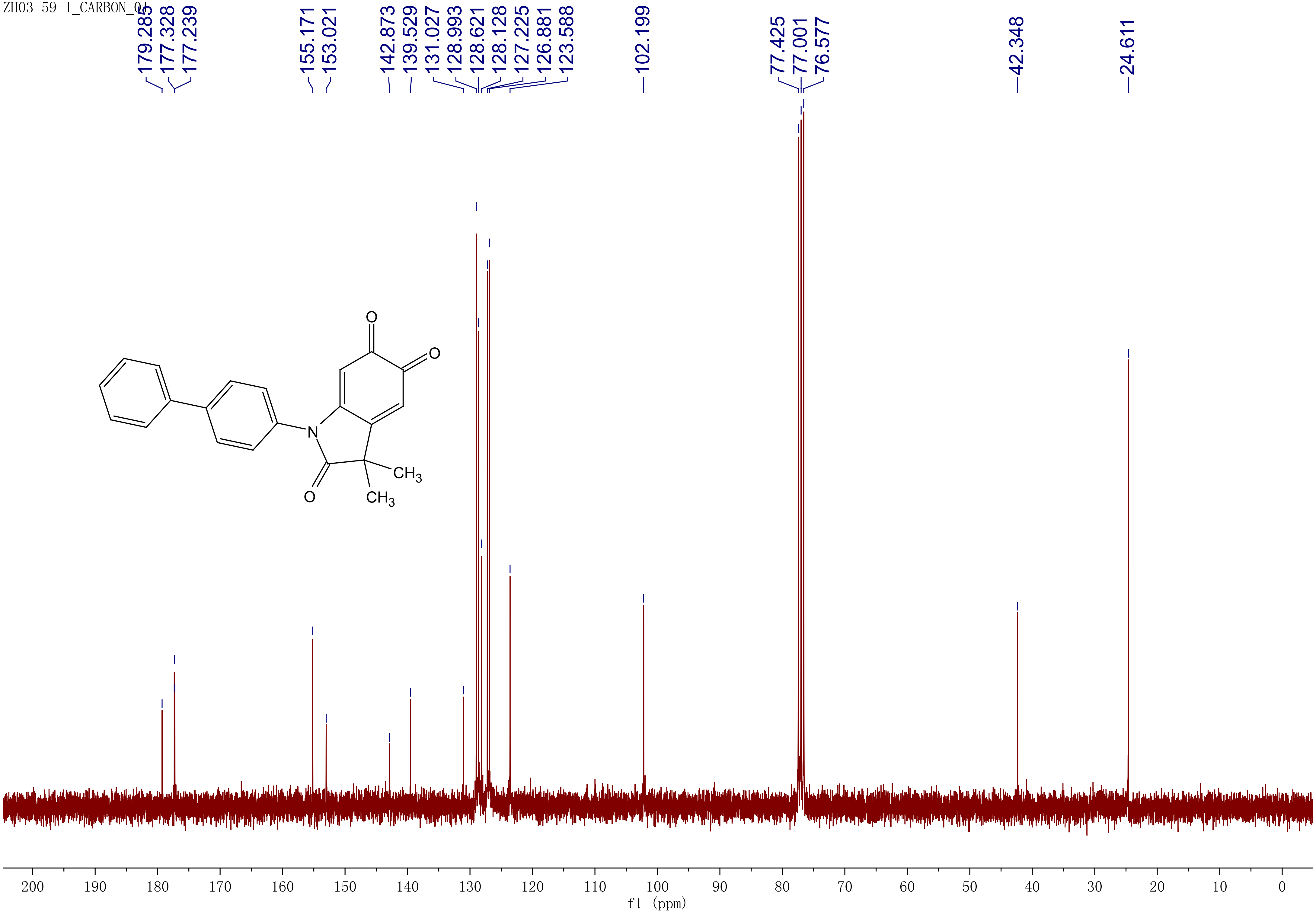
ZH03-59-1_CARBON_01
179.285
177.328
177.239
155.171
153.021
142.873
139.529
131.027
128.993
128.621
128.128
127.225
126.881
123.588
102.199
77.425
77.001
76.577
42.348
24.611
O
O
N
O
CH3
CH3
200 190 180 170 160 150 140 130 120 110 100 90 80 70 60 50 40 30 20 10 0
f1 (ppm)

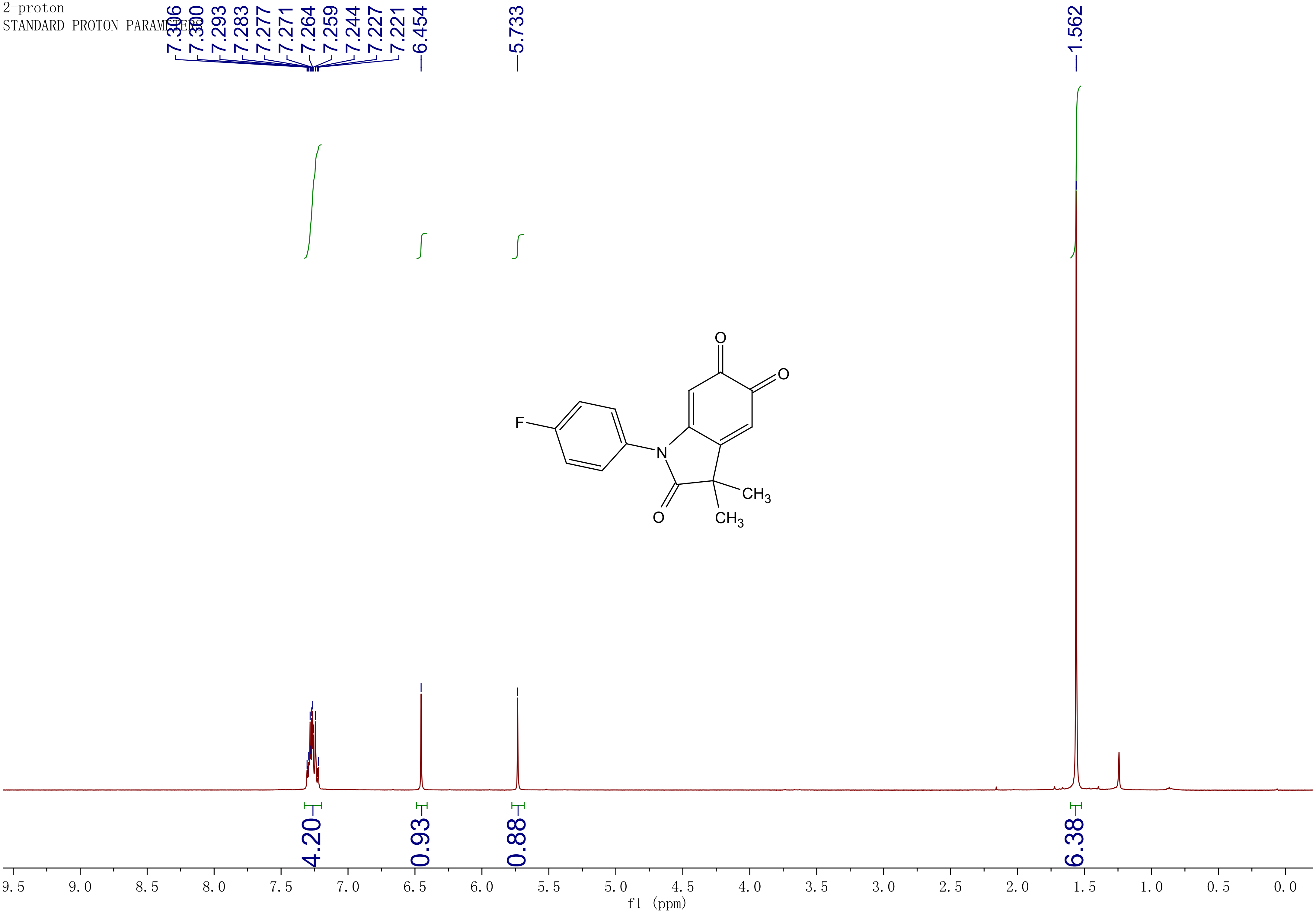

2-proton
STANDARD PROTON PARAMETERS
7.306
7.300
7.293
7.283
7.277
7.271
7.264
7.259
7.244
7.227
7.221
6.454
5.733
1.562
F
N
O
O
O
CH3
CH3
4.20
0.93
0.88
6.38
9.5 9.0 8.5 8.0 7.5 7.0 6.5 6.0 5.5 5.0 4.5 4.0 3.5 3.0 2.5 2.0 1.5 1.0 0.5 0.0
f1 (ppm)

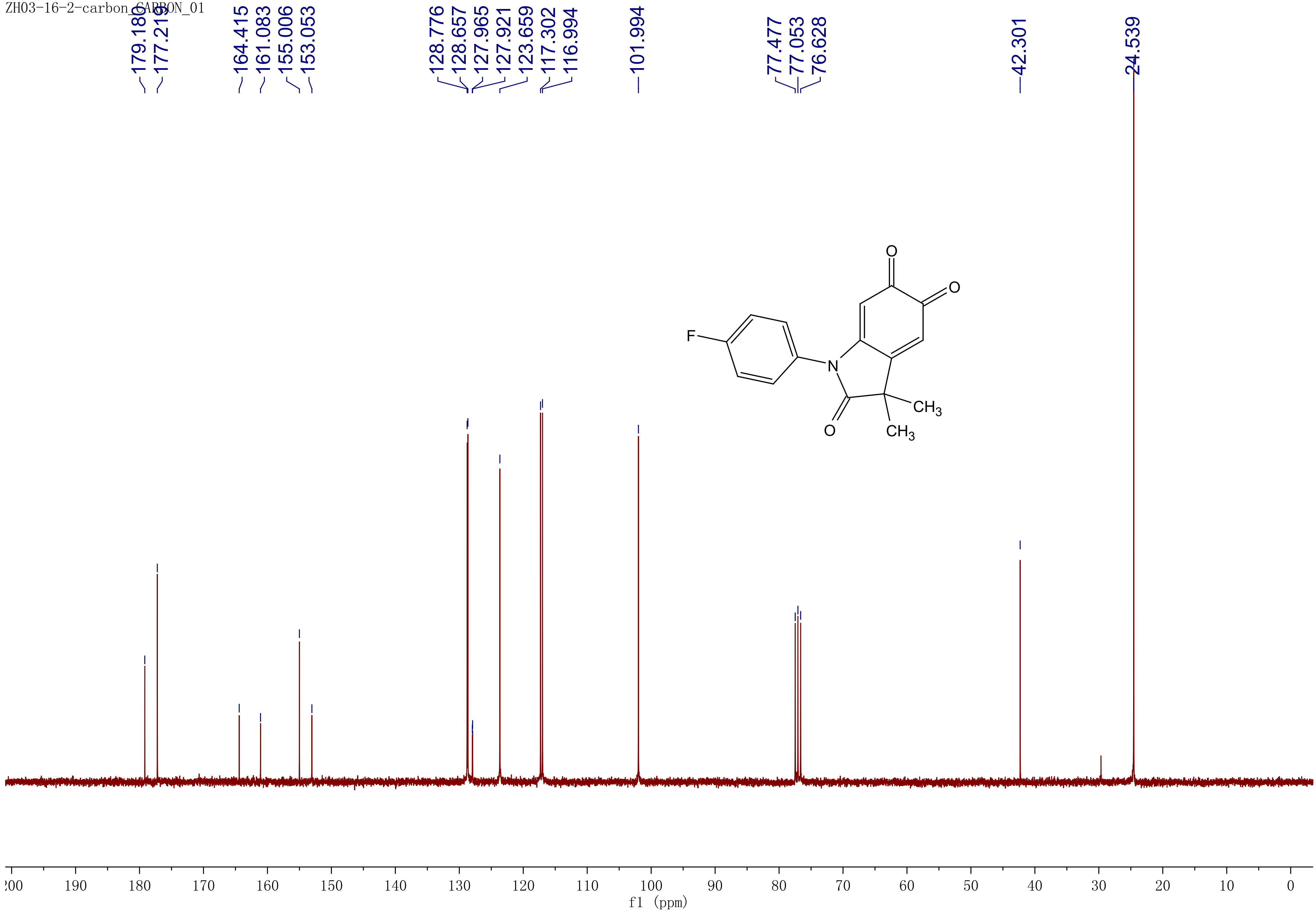
ZH03-16-2-carbon_CARBON_01
179.180
177.219
164.415
161.083
155.006
153.053
128.776
128.657
127.965
127.921
123.659
117.302
116.994
101.994
77.477
77.053
76.628
42.301
24.539
F
N
O
O
O
CH3
CH3
200
190
180
170
160
150
140
130
120
110
100
90
80
70
60
50
40
30
20
10
0
f1 (ppm)

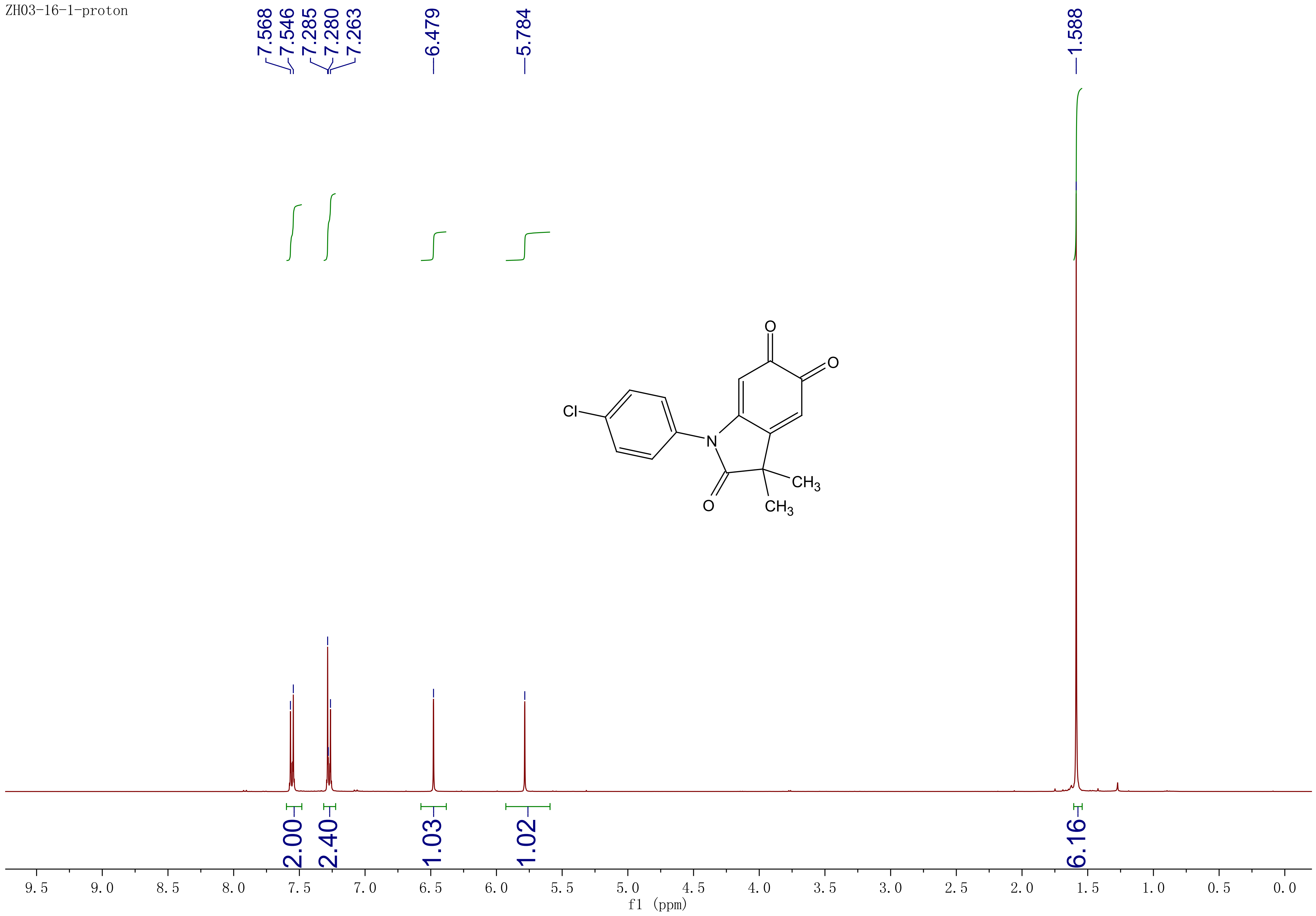

ZH03-16-1-proton
7.568
7.546
7.285
7.280
7.263
6.479
5.784
1.588
O
O
Cl
N
CH3
O
CH3
2.00
2.40
1.03
1.02
6.16
9.5
9.0
8.5
8.0
7.5
7.0
6.5
6.0
5.5
5.0
4.5
4.0
3.5
3.0
2.5
2.0
1.5
1.0
0.5
0.0
f1 (ppm)

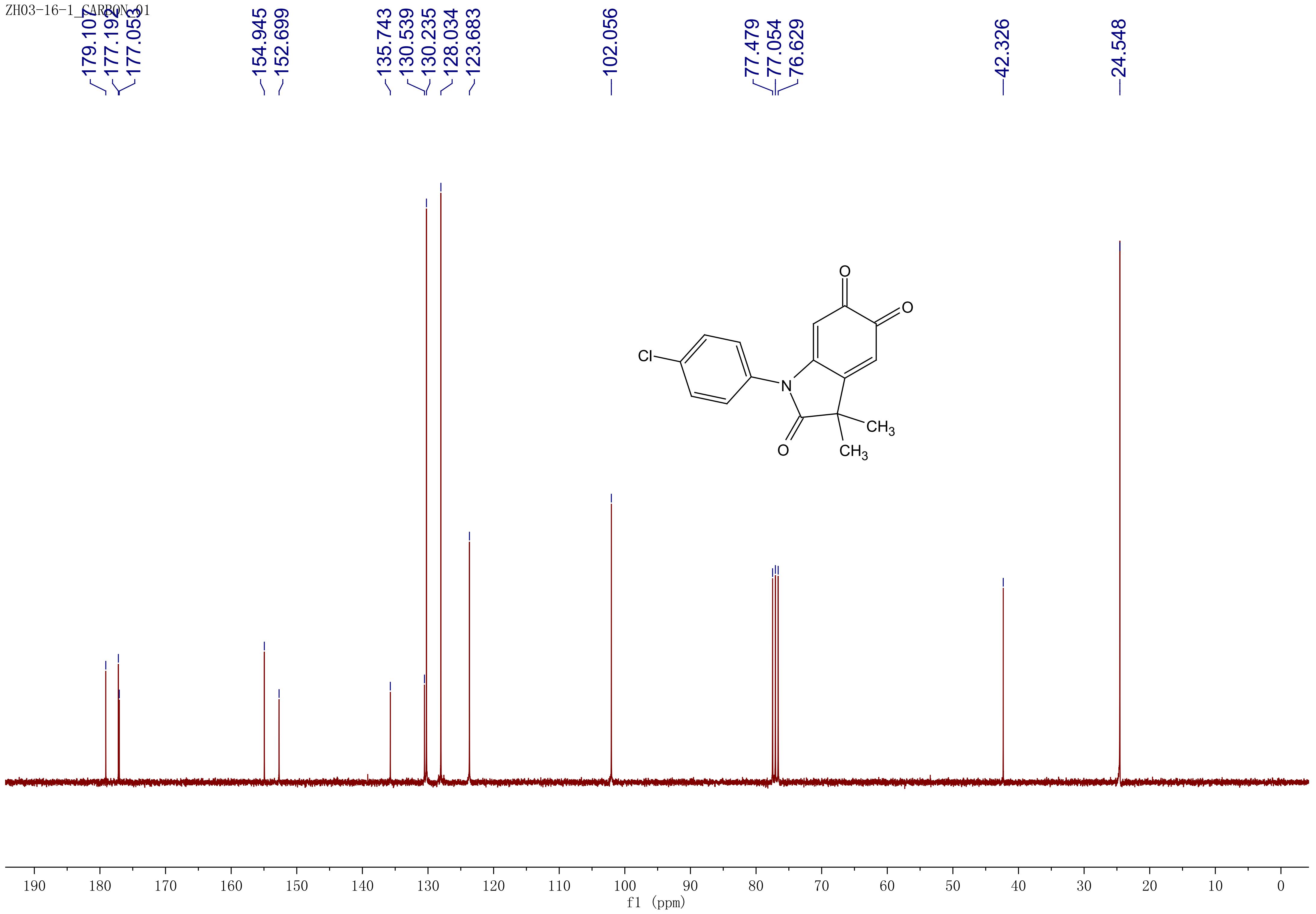
ZH03-16-1_CARBON_01
179.107
177.192
177.053
154.945
152.699
135.743
130.539
130.235
128.034
123.683
102.056
77.479
77.054
76.629
42.326
24.548
O
O
Cl
N
CH3
O
CH3
190 180 170 160 150 140 130 120 110 100 90 80 70 60 50 40 30 20 10 0
f1 (ppm)

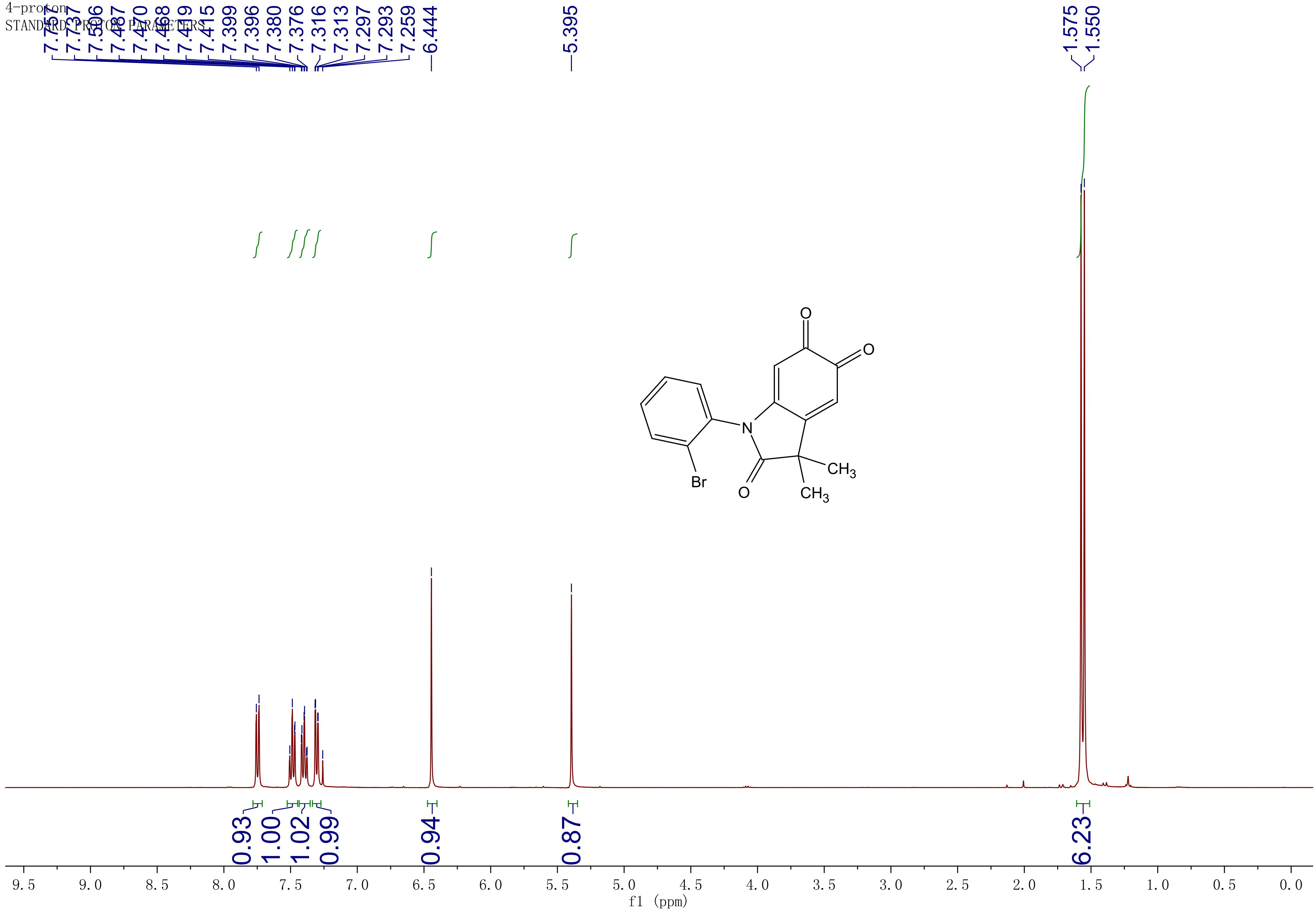
4-proton
STANDARD PROTON PARAMETERS
7.757
7.737
7.506
7.487
7.470
7.468
7.419
7.415
7.399
7.396
7.380
7.376
7.316
7.313
7.297
7.293
7.259
6.444
5.395
1.575
1.550
O
O
N
O
Br
CH3
CH3
0.93
1.00
1.02
0.99
0.94
0.87
6.23
9.5
9.0
8.5
8.0
7.5
7.0
6.5
6.0
5.5
5.0
4.5
4.0
3.5
3.0
2.5
2.0
1.5
1.0
0.5
0.0
f1 (ppm)

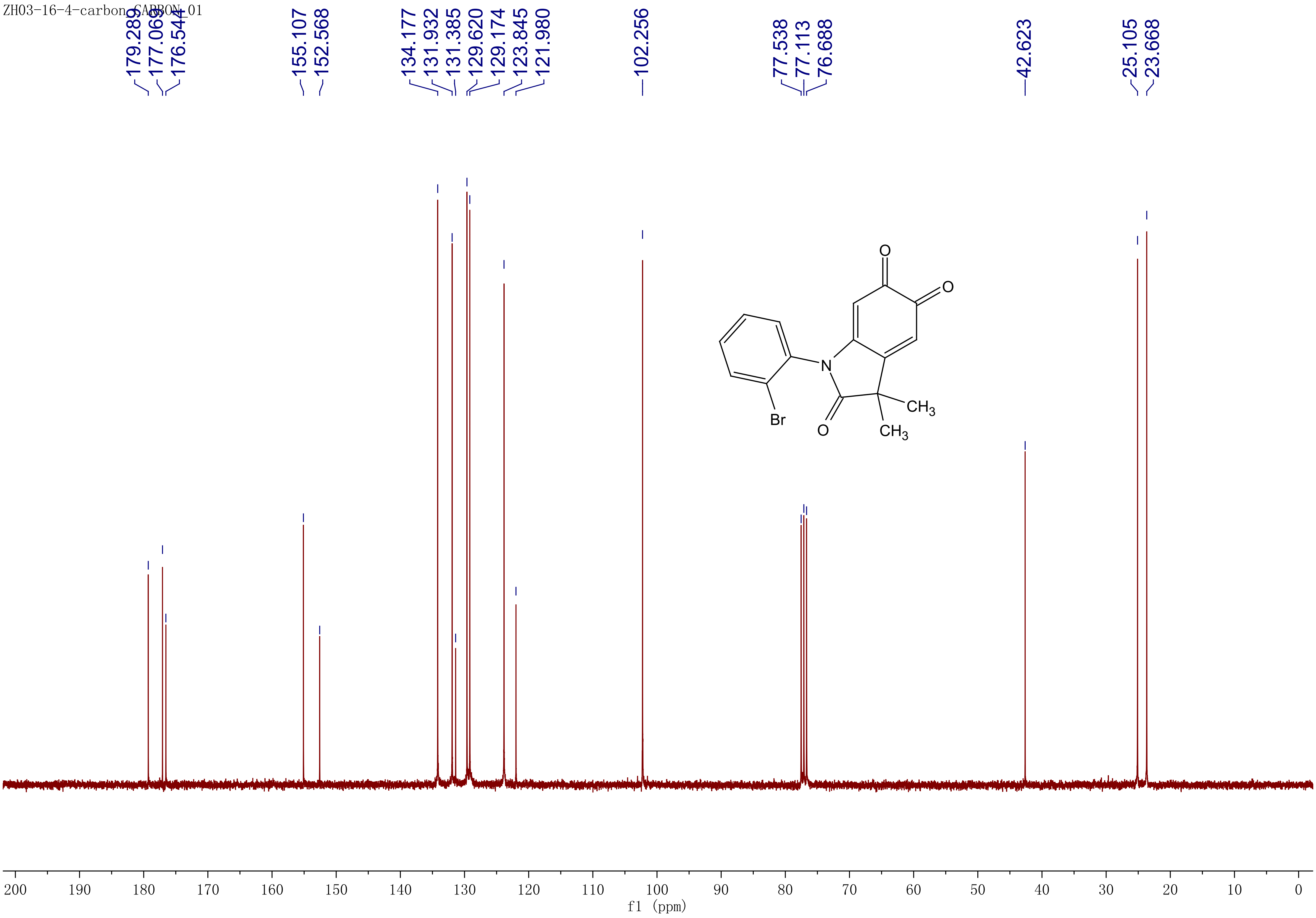
ZH03-16-4-carbon_CARBON_01
179.289
177.069
176.544
155.107
152.568
134.177
131.932
131.385
129.620
129.174
123.845
121.980
102.256
77.538
77.113
76.688
42.623
25.105
23.668
O
O
N
Br
O
CH3
CH3
200 190 180 170 160 150 140 130 120 110 100 90 80 70 60 50 40 30 20 10 0
f1 (ppm)

ZH03-11-3

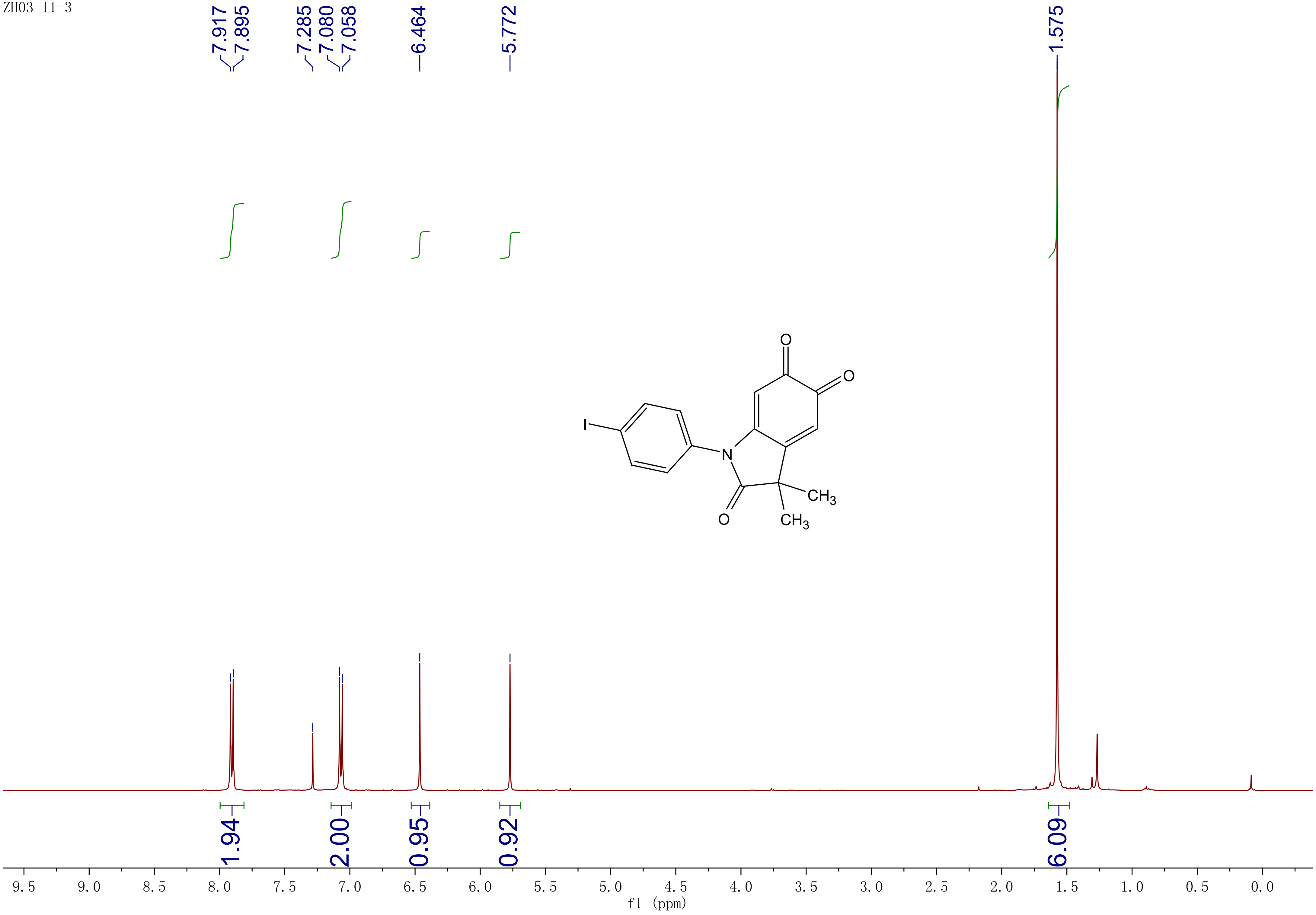
7.917
7.895
7.285
7.080
7.058
6.464
5.772
1.575
1.94
2.00
0.95
0.92
6.09
9.5 9.0 8.5 8.0 7.5 7.0 6.5 6.0 5.5 5.0 4.5 4.0 3.5 3.0 2.5 2.0 1.5 1.0 0.5 0.0
f1 (ppm)
O
O
I
N
O
CH3
CH3

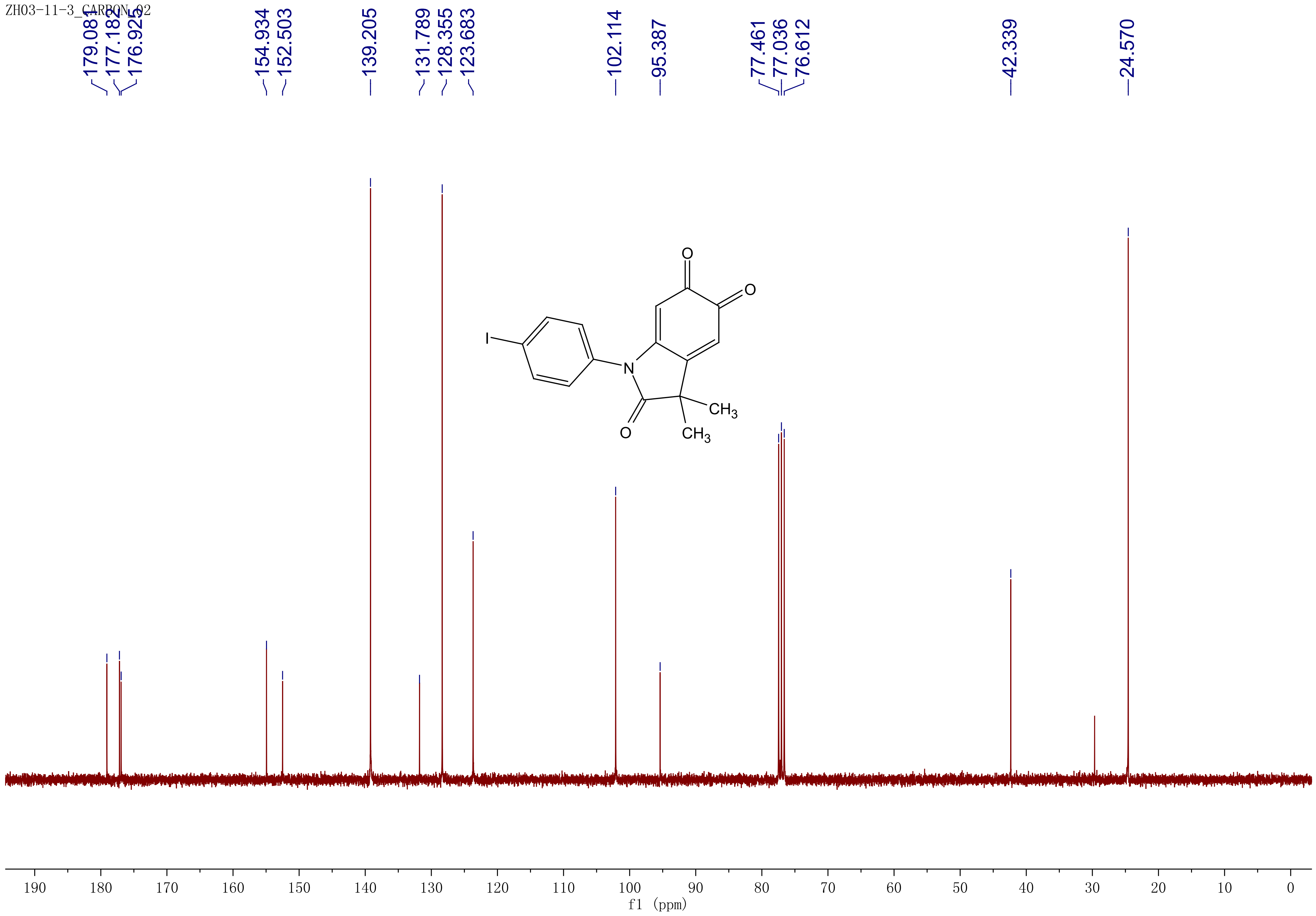
ZH03-11-3_CARBON_02
179.081
177.182
176.925
154.934
152.503
139.205
131.789
128.355
123.683
102.114
95.387
77.461
77.036
76.612
42.339
24.570
O
O
I
N
CH3
O
CH3
190 180 170 160 150 140 130 120 110 100 90 80 70 60 50 40 30 20 10 0
f1 (ppm)

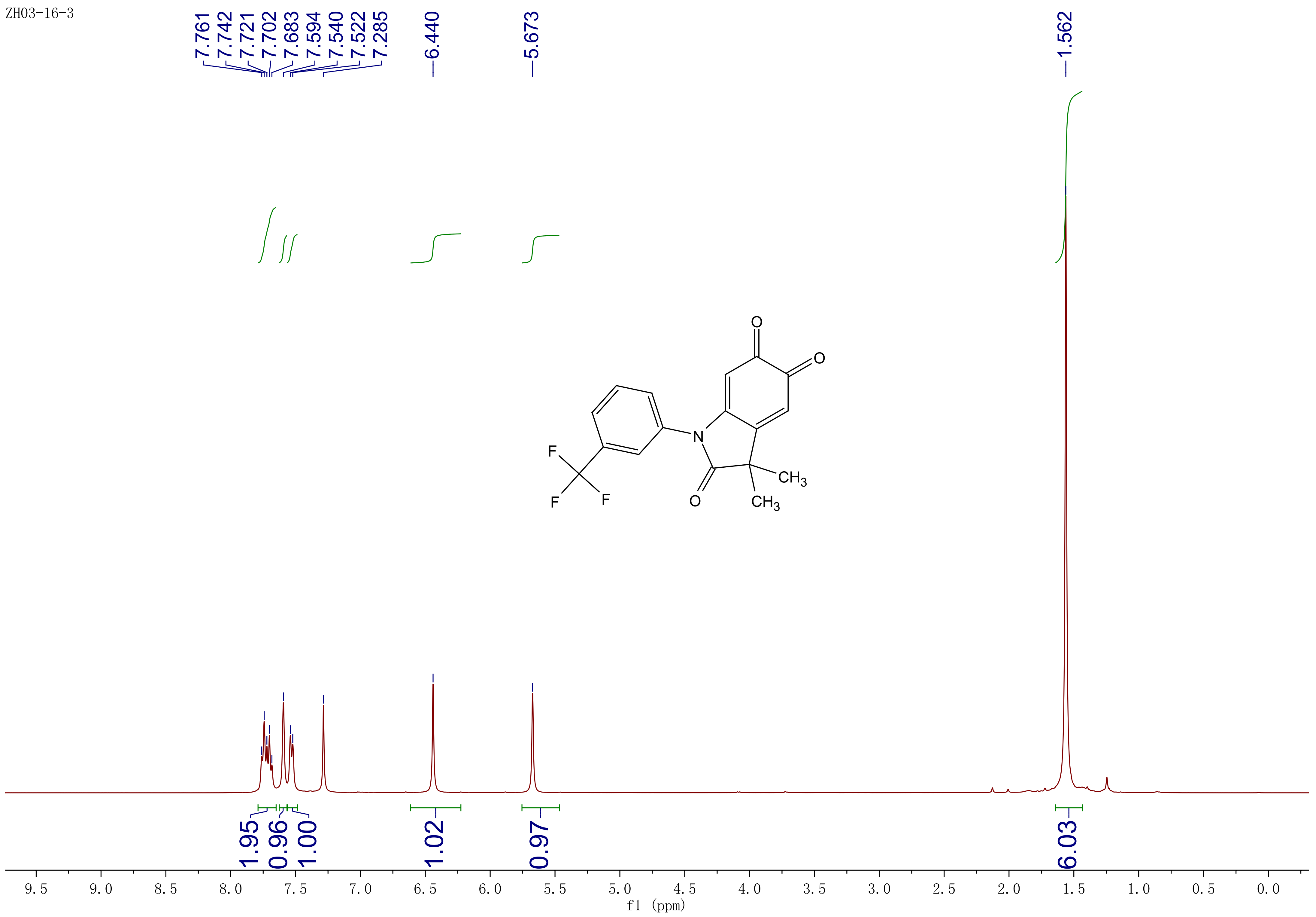

ZH03-16-3
7.761
7.742
7.721
7.702
7.683
7.594
7.540
7.522
7.285
6.440
5.673
1.562
O
O
N
F
F
F
O
CH3
CH3
1.95
0.96
1.00
1.02
0.97
6.03
9.5
9.0
8.5
8.0
7.5
7.0
6.5
6.0
5.5
5.0
4.5
4.0
3.5
3.0
2.5
2.0
1.5
1.0
0.5
0.0
f1 (ppm)

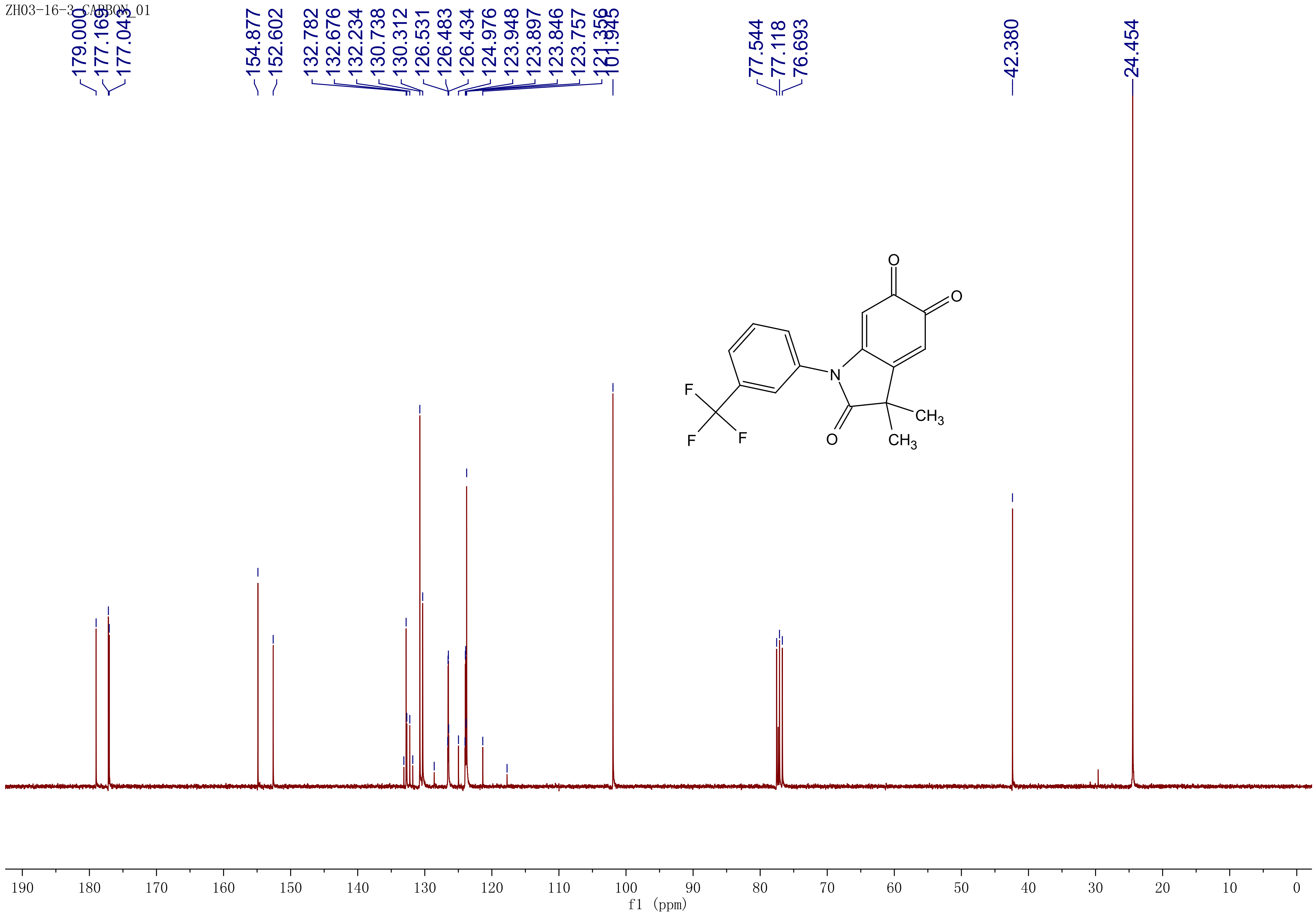
ZH03-16-3_CARBON_01
179.000
177.169
177.043
154.877
152.602
132.782
132.676
132.234
130.738
130.312
126.531
126.483
126.434
124.976
123.948
123.897
123.846
123.757
121.356
101.945
77.544
77.118
76.693
42.380
24.454
O
O
N
F
F
F
O
CH3
CH3
190
180
170
160
150
140
130
120
110
100
90
80
70
60
50
40
30
20
10
0
f1 (ppm)

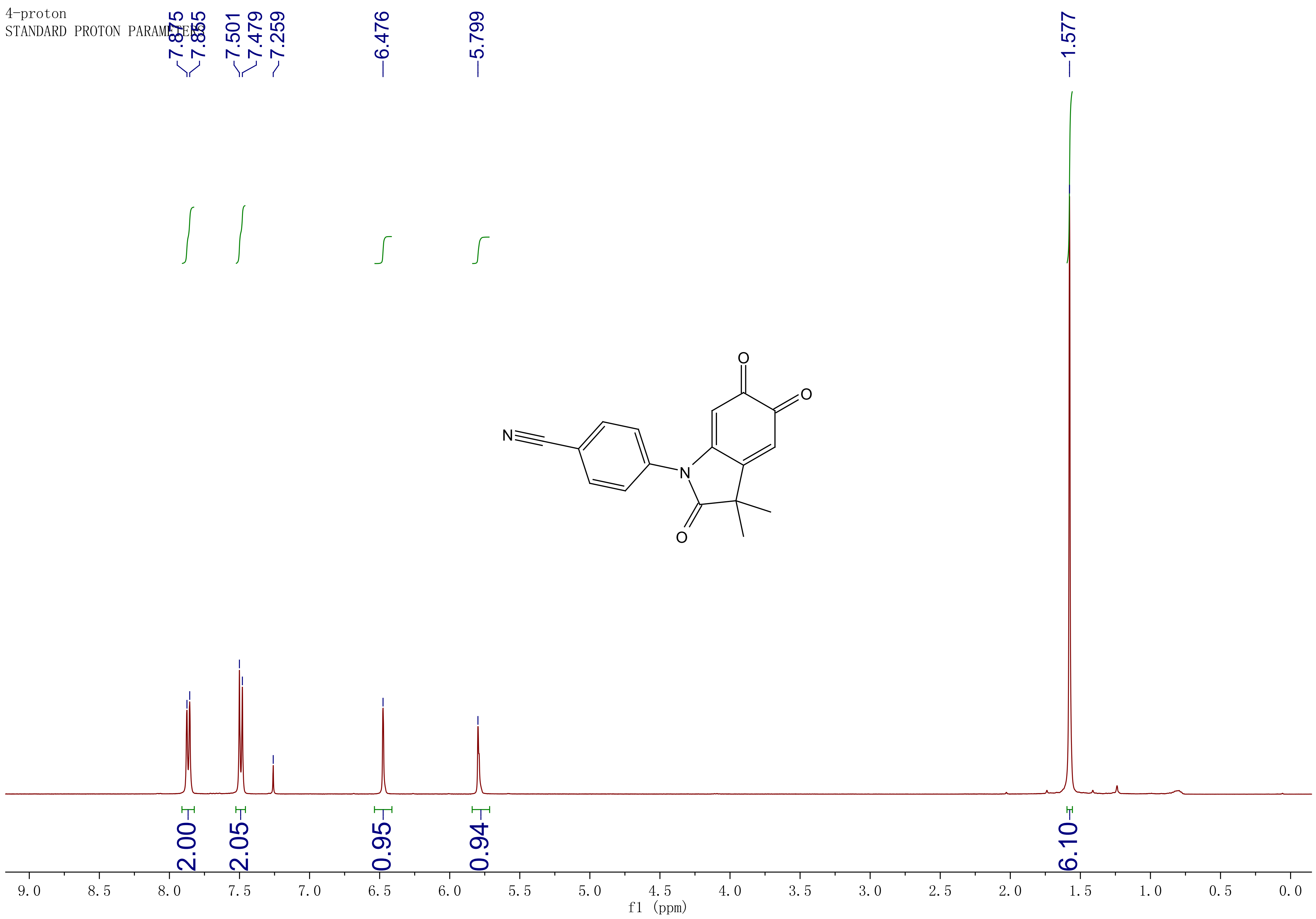
4-proton
STANDARD PROTON PARAMETERS
7.875
7.855
7.501
7.479
7.259
6.476
5.799
1.577
2.00
2.05
0.95
0.94
6.10
9.0
8.5
8.0
7.5
7.0
6.5
6.0
5.5
5.0
4.5
4.0
3.5
3.0
2.5
2.0
1.5
1.0
0.5
0.0
f1 (ppm)
N
N
O
O
O

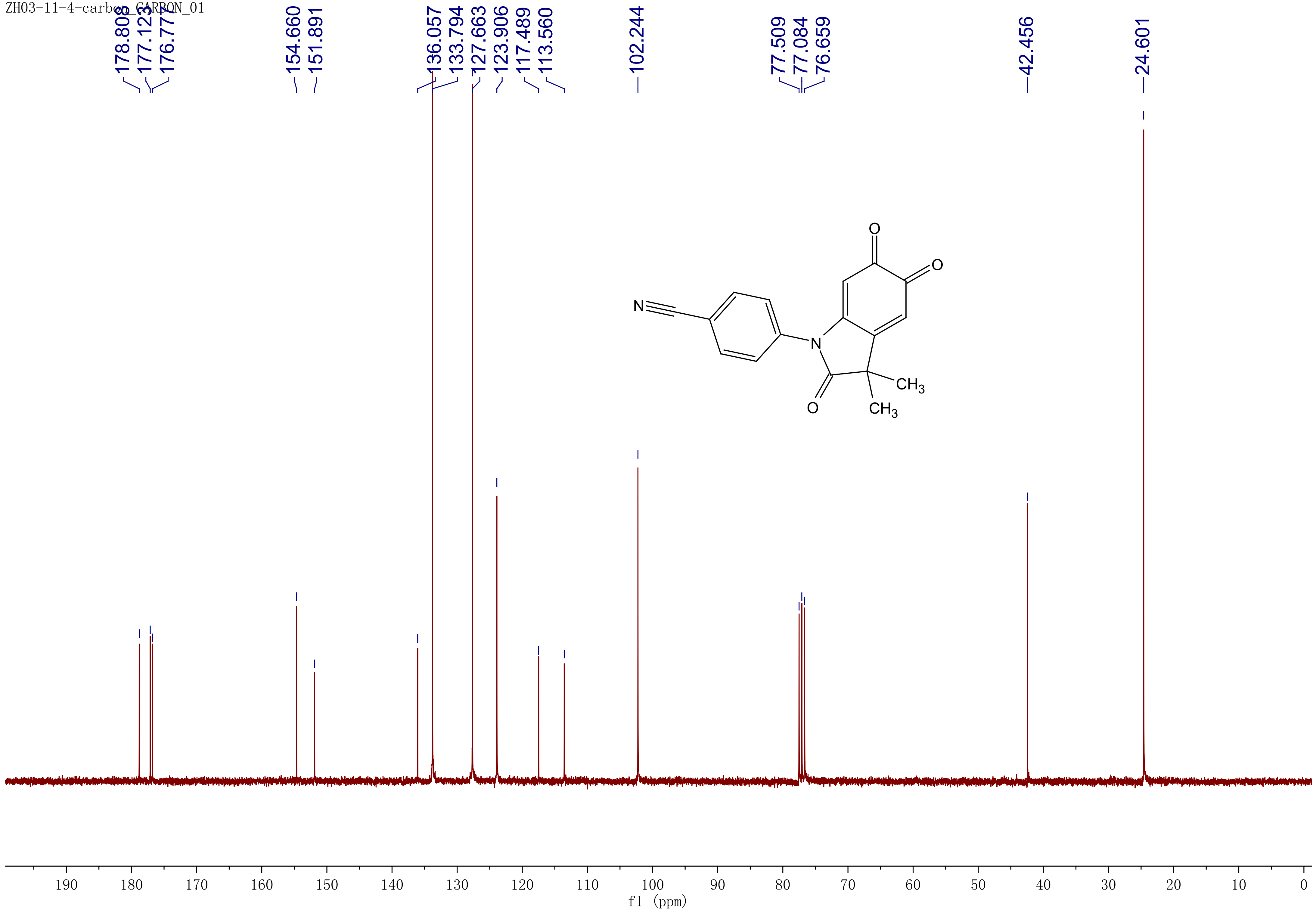

ZH03-11-4-carbon_CARBON_01
178.808
177.123
176.777
154.660
151.891
136.057
133.794
127.663
123.906
117.489
113.560
102.244
77.509
77.084
76.659
42.456
24.601
N
N
O
O
O
CH3
CH3
190 180 170 160 150 140 130 120 110 100 90 80 70 60 50 40 30 20 10 0
f1 (ppm)

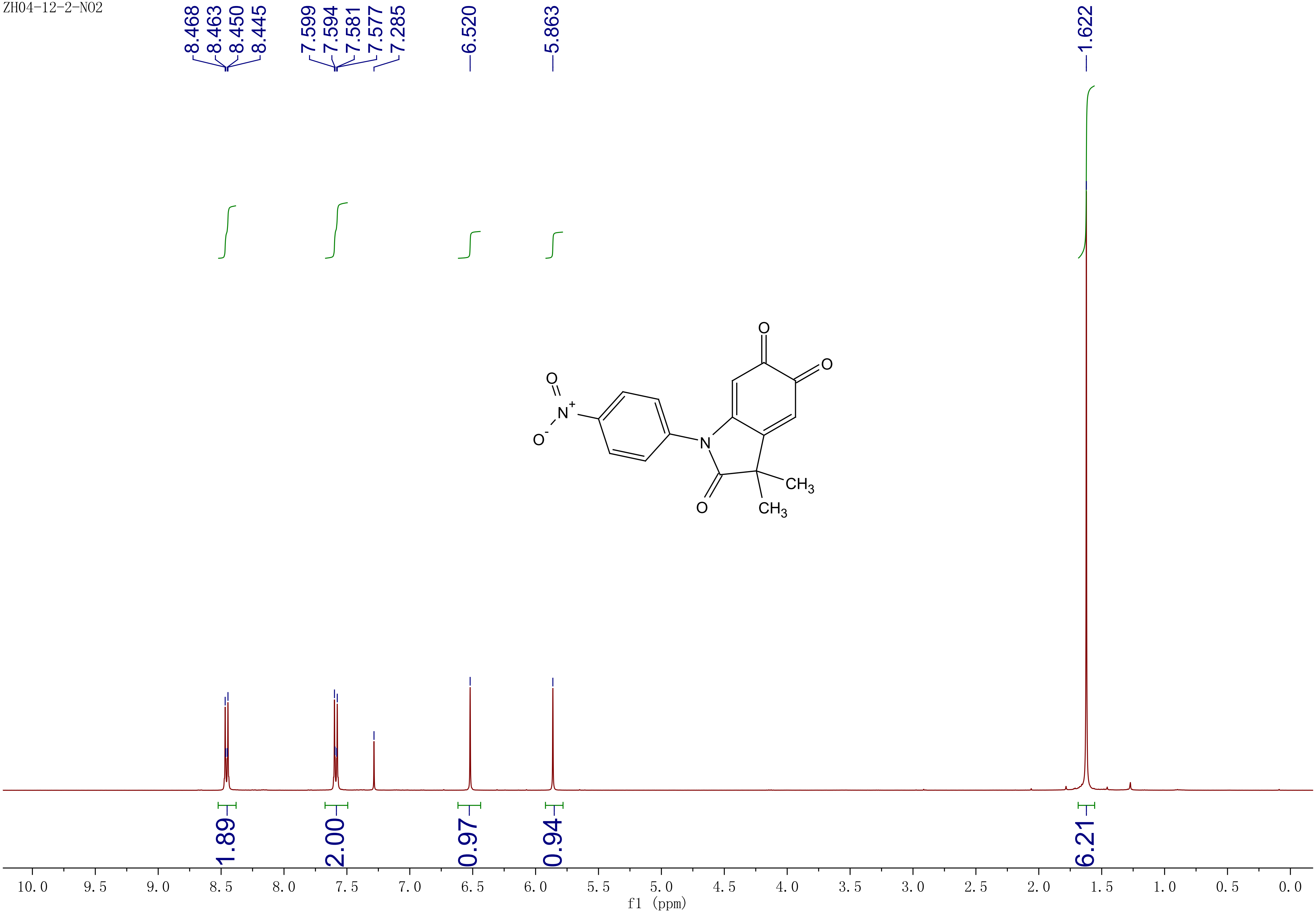
ZH04-12-2-NO2
8.468
8.463
8.450
8.445
7.599
7.594
7.581
7.577
7.285
6.520
5.863
1.622
1.89
2.00
0.97
0.94
6.21
10.0 9.5 9.0 8.5 8.0 7.5 7.0 6.5 6.0 5.5 5.0 4.5 4.0 3.5 3.0 2.5 2.0 1.5 1.0 0.5 0.0
f1 (ppm)
O
N+
O-
N
O
O
O
CH3
CH3

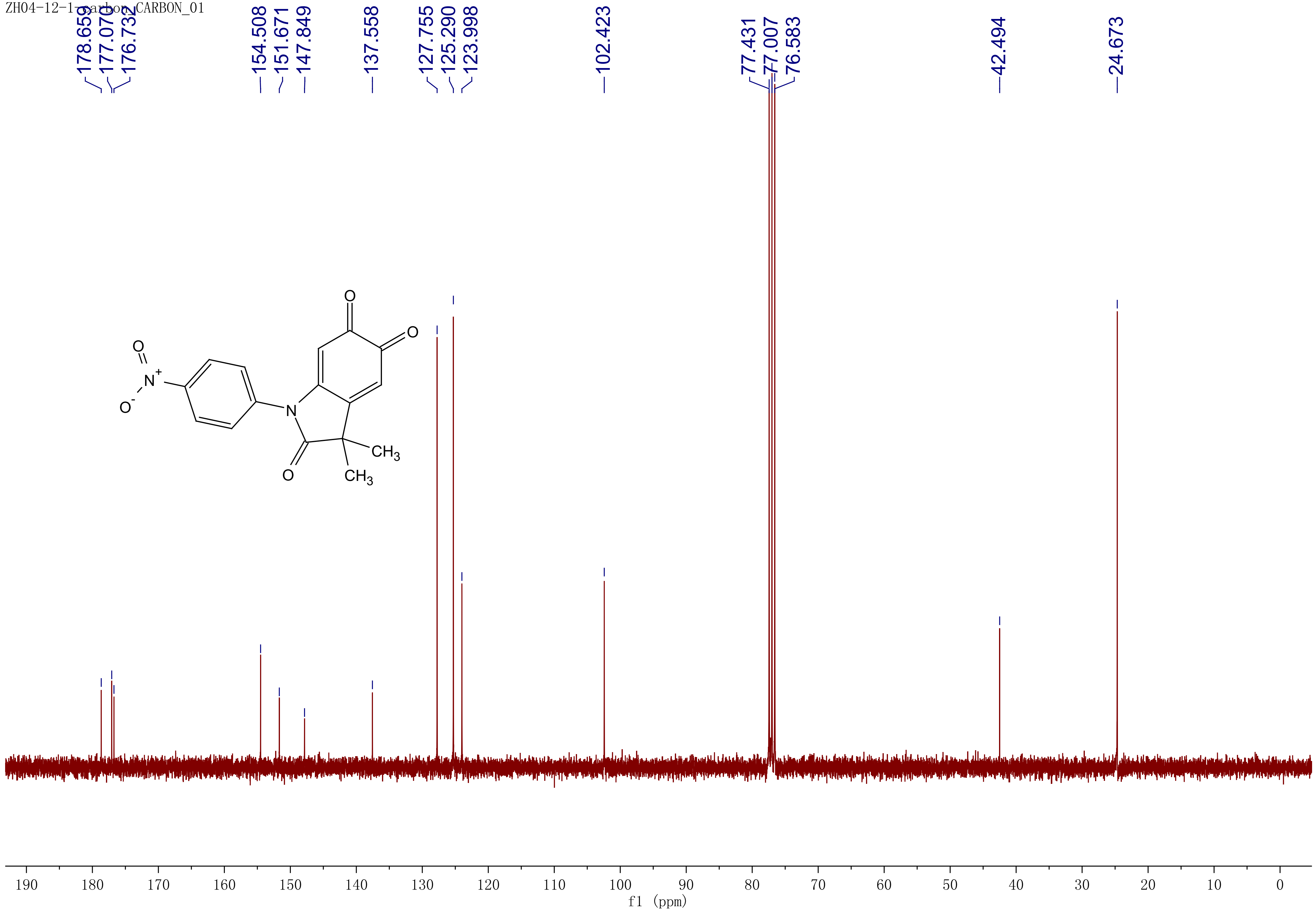
ZH04-12-1-carbon_CARBON_01
178.659
177.070
176.732
154.508
151.671
147.849
137.558
127.755
125.290
123.998
102.423
77.431
77.007
76.583
42.494
24.673
190 180 170 160 150 140 130 120 110 100 90 80 70 60 50 40 30 20 10 0
f1 (ppm)
O
O
O
N+
O-
N
O
CH3
CH3

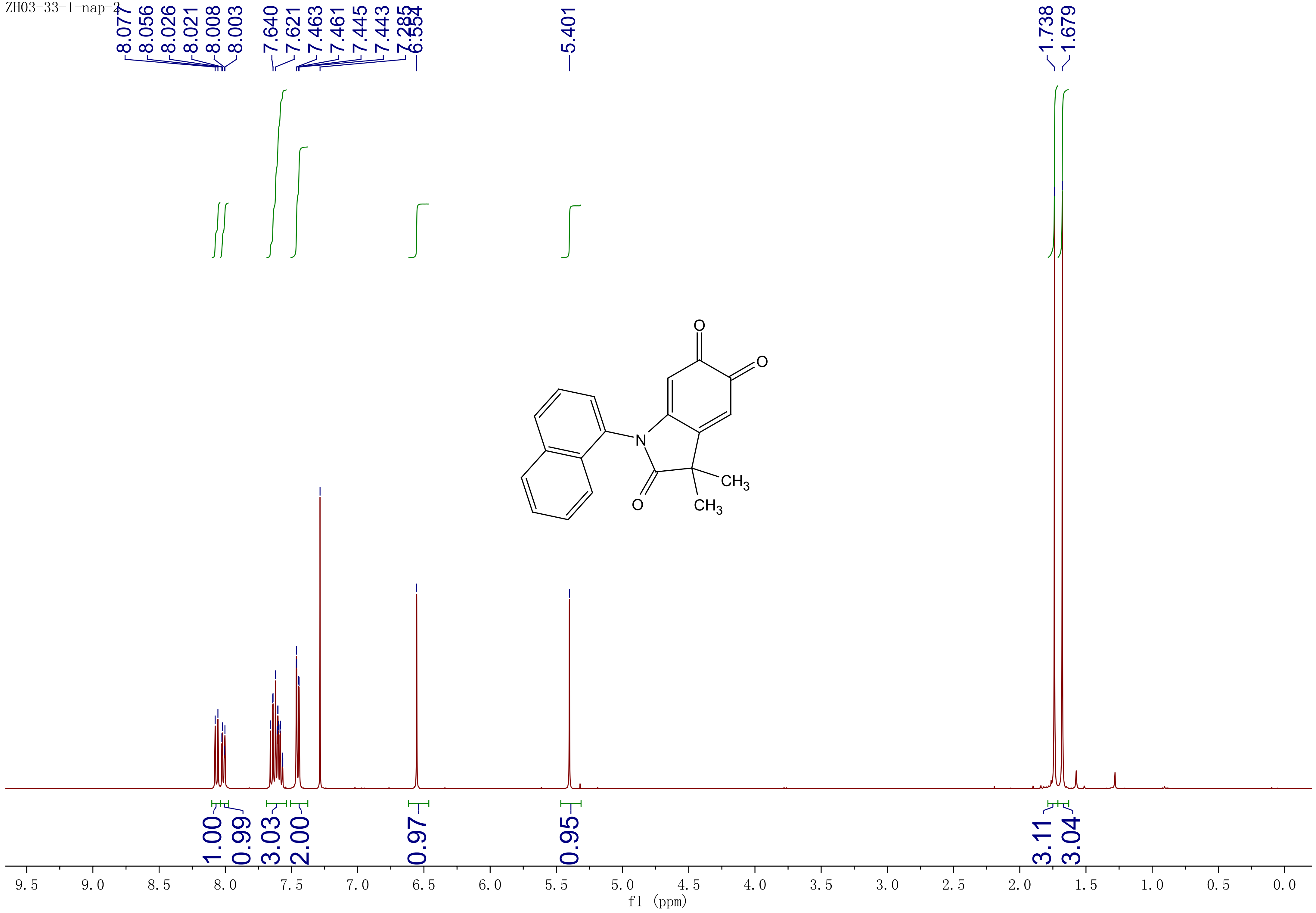
ZH03-33-1-nap-2
8.077
8.056
8.026
8.021
8.008
8.003
7.640
7.621
7.463
7.461
7.445
7.443
7.285
6.554
5.401
1.738
1.679
1.00
0.99
3.03
2.00
0.97
0.95
3.11
3.04
O
O
N
O
CH3
CH3
9.5
9.0
8.5
8.0
7.5
7.0
6.5
6.0
5.5
5.0
4.5
4.0
3.5
3.0
2.5
2.0
1.5
1.0
0.5
0.0
f1 (ppm)

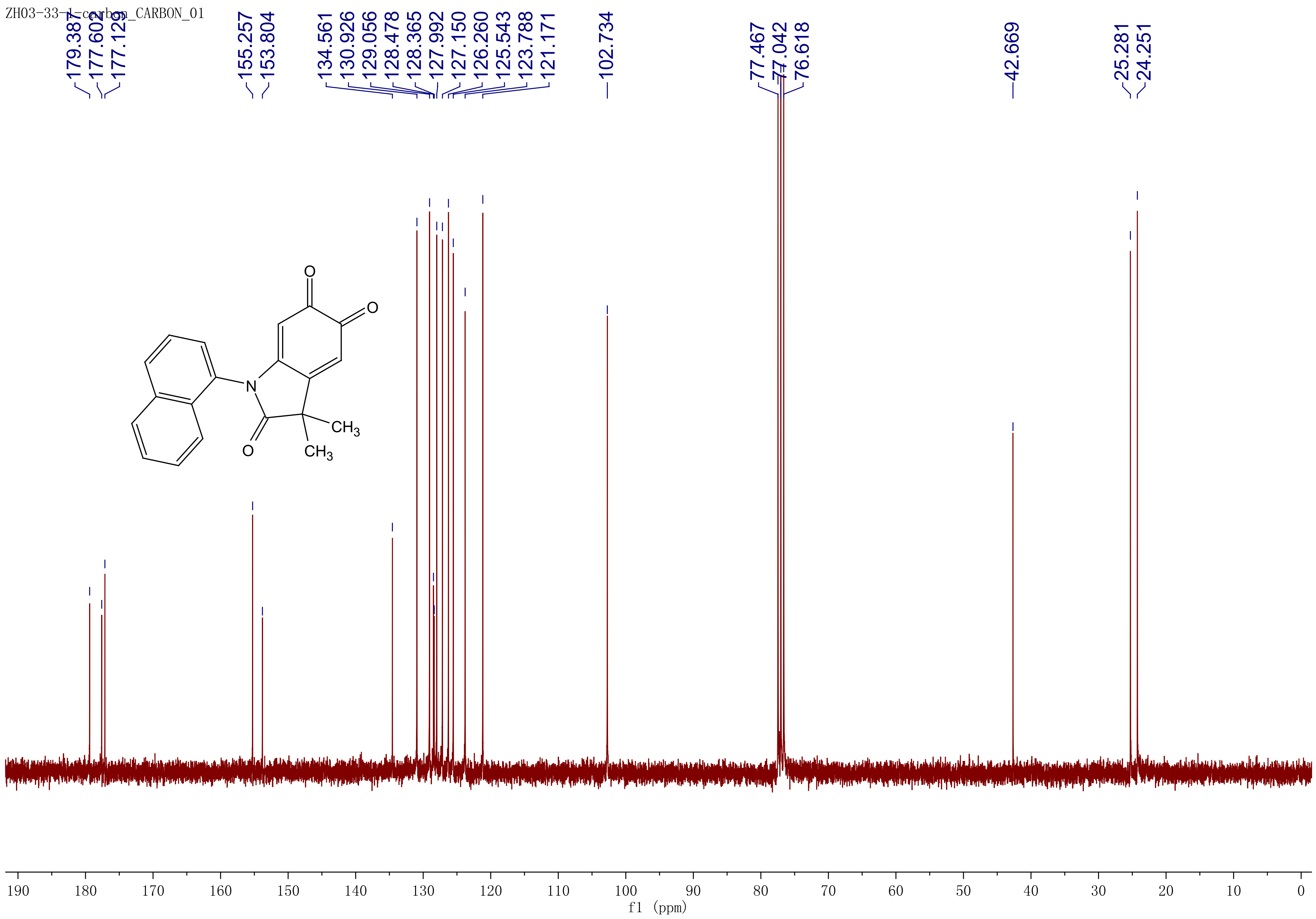
ZH03-33-1-carbon_CARBON_01
179.387
177.602
177.129
155.257
153.804
134.561
130.926
129.056
128.478
128.365
127.992
127.150
126.260
125.543
123.788
121.171
102.734
77.467
77.042
76.618
42.669
25.281
24.251
O
O
N
O
CH3
CH3
190 180 170 160 150 140 130 120 110 100 90 80 70 60 50 40 30 20 10 0
f1 (ppm)

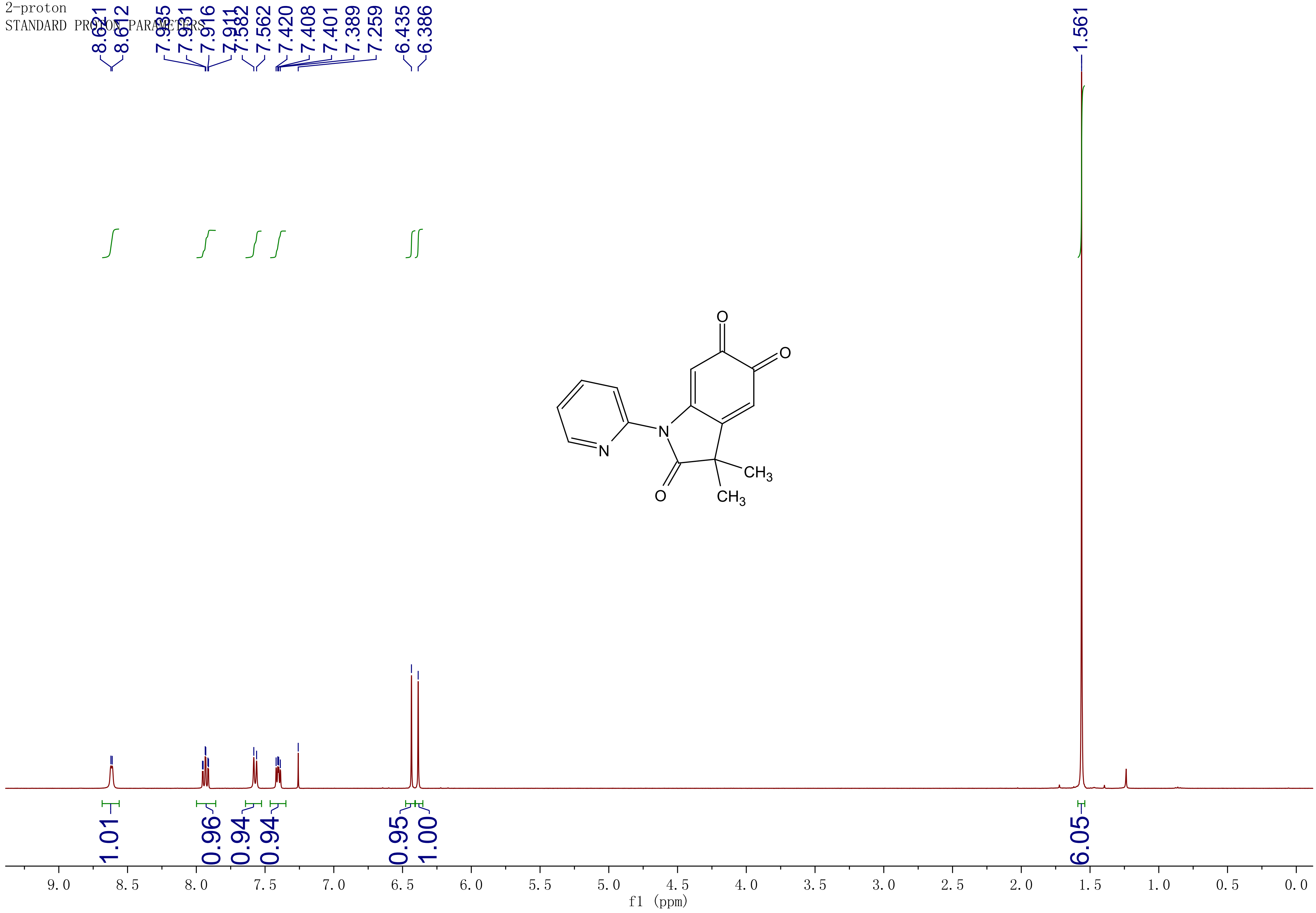
2-proton
STANDARD PROTON PARAMETERS
8.621
8.612
7.935
7.931
7.916
7.911
7.582
7.562
7.420
7.408
7.401
7.389
7.259
6.435
6.386
1.561
1.01
0.96
0.94
0.94
0.95
1.00
6.05
O
O
N
N
O
CH3
CH3
9.0 8.5 8.0 7.5 7.0 6.5 6.0 5.5 5.0 4.5 4.0 3.5 3.0 2.5 2.0 1.5 1.0 0.5 0.0
f1 (ppm)

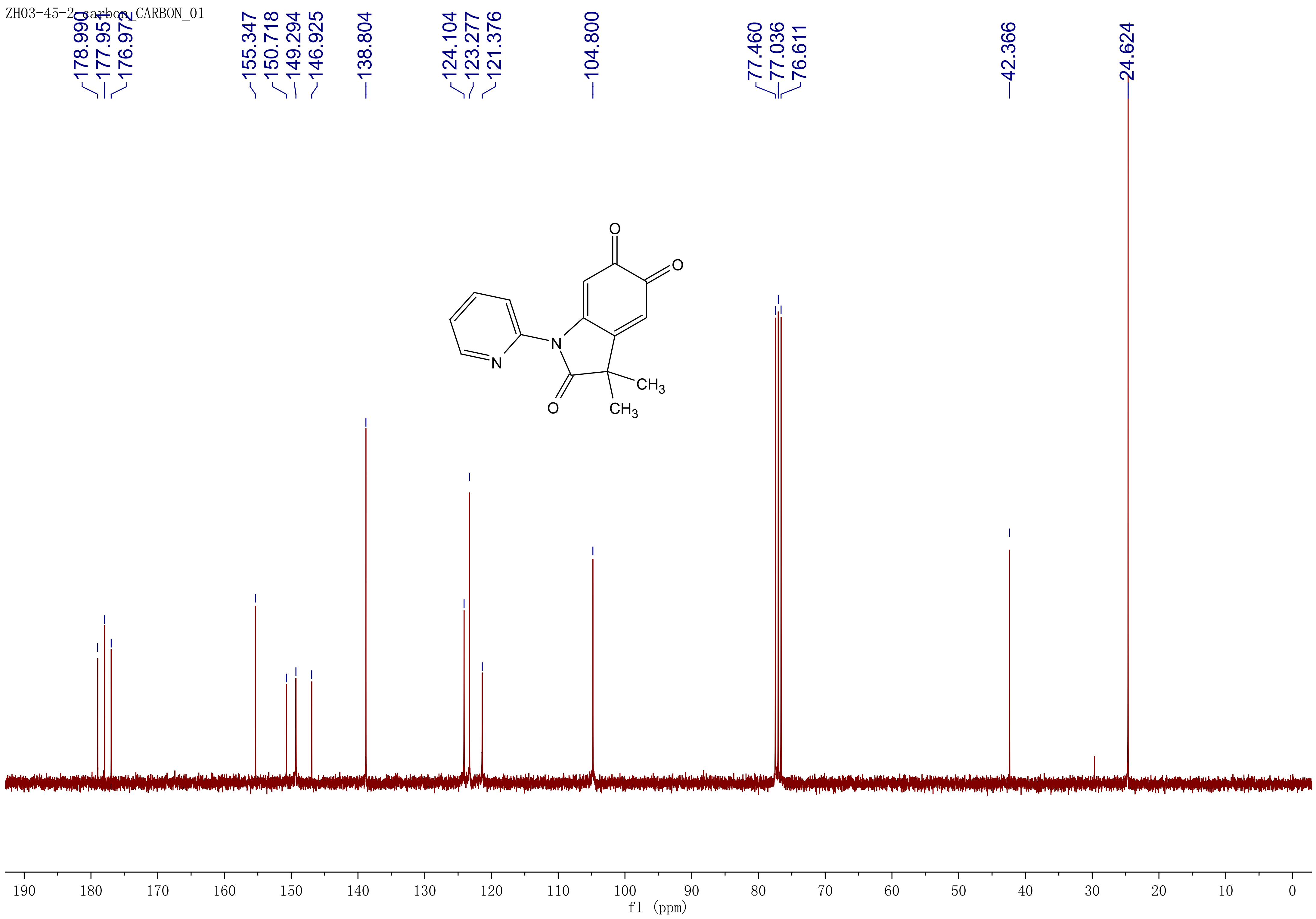

ZH03-45-2-carbon,CARBON_01
178.990
177.951
176.972
155.347
150.718
149.294
146.925
138.804
124.104
123.277
121.376
104.800
77.460
77.036
76.611
42.366
24.624
O
O
N
N
O
CH3
CH3
190 180 170 160 150 140 130 120 110 100 90 80 70 60 50 40 30 20 10 0
f1 (ppm)

ZH03-45-1

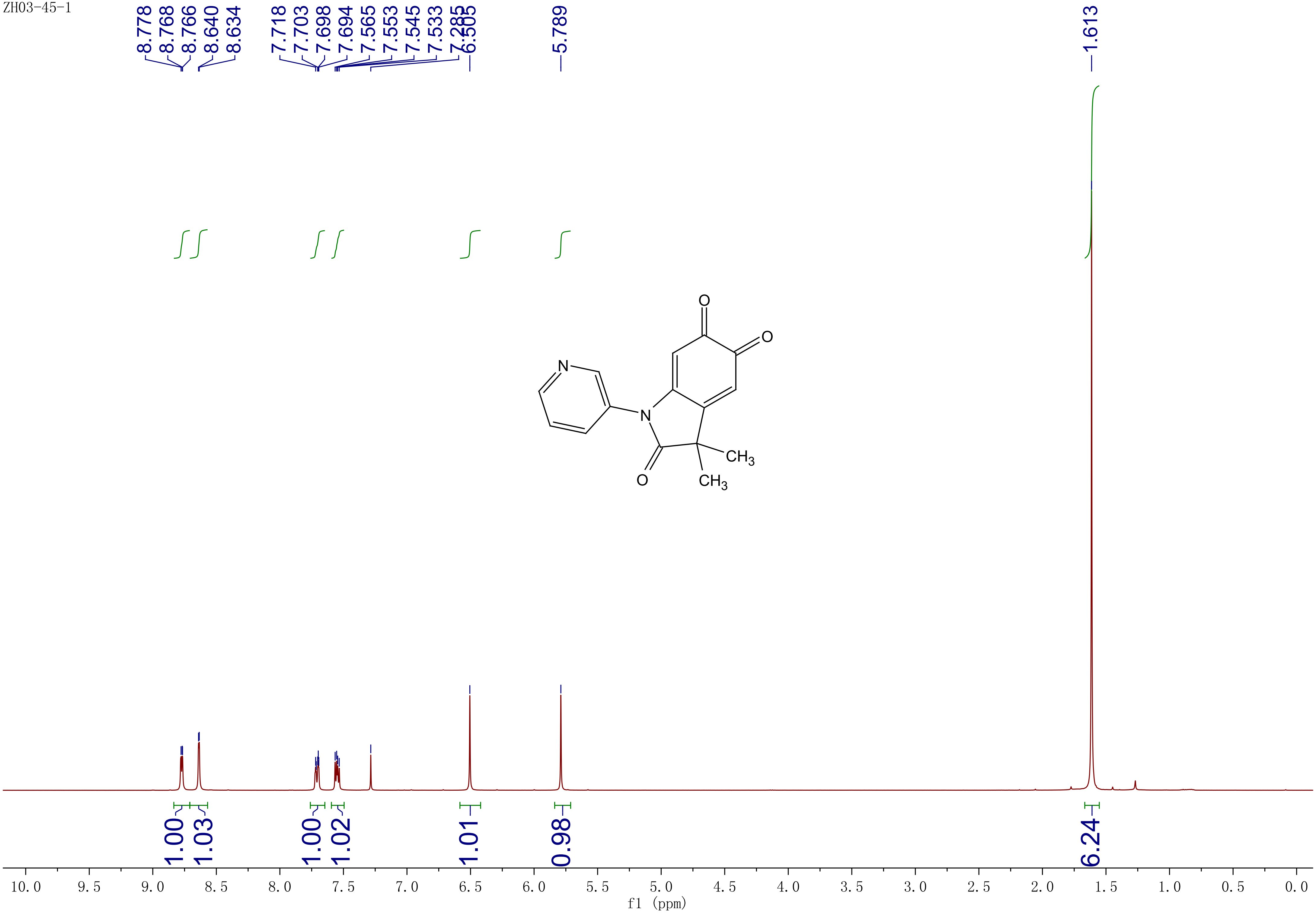
8.778
8.768
8.766
8.640
8.634
7.718
7.703
7.698
7.694
7.565
7.553
7.545
7.533
7.285
6.505
5.789
1.613
O
O
N
N
CH3
O
CH3
1.00
1.03
1.00
1.02
1.01
0.98
6.24
10.0 9.5 9.0 8.5 8.0 7.5 7.0 6.5 6.0 5.5 5.0 4.5 4.0 3.5 3.0 2.5 2.0 1.5 1.0 0.5 0.0
f1 (ppm)

ZH03-45-1

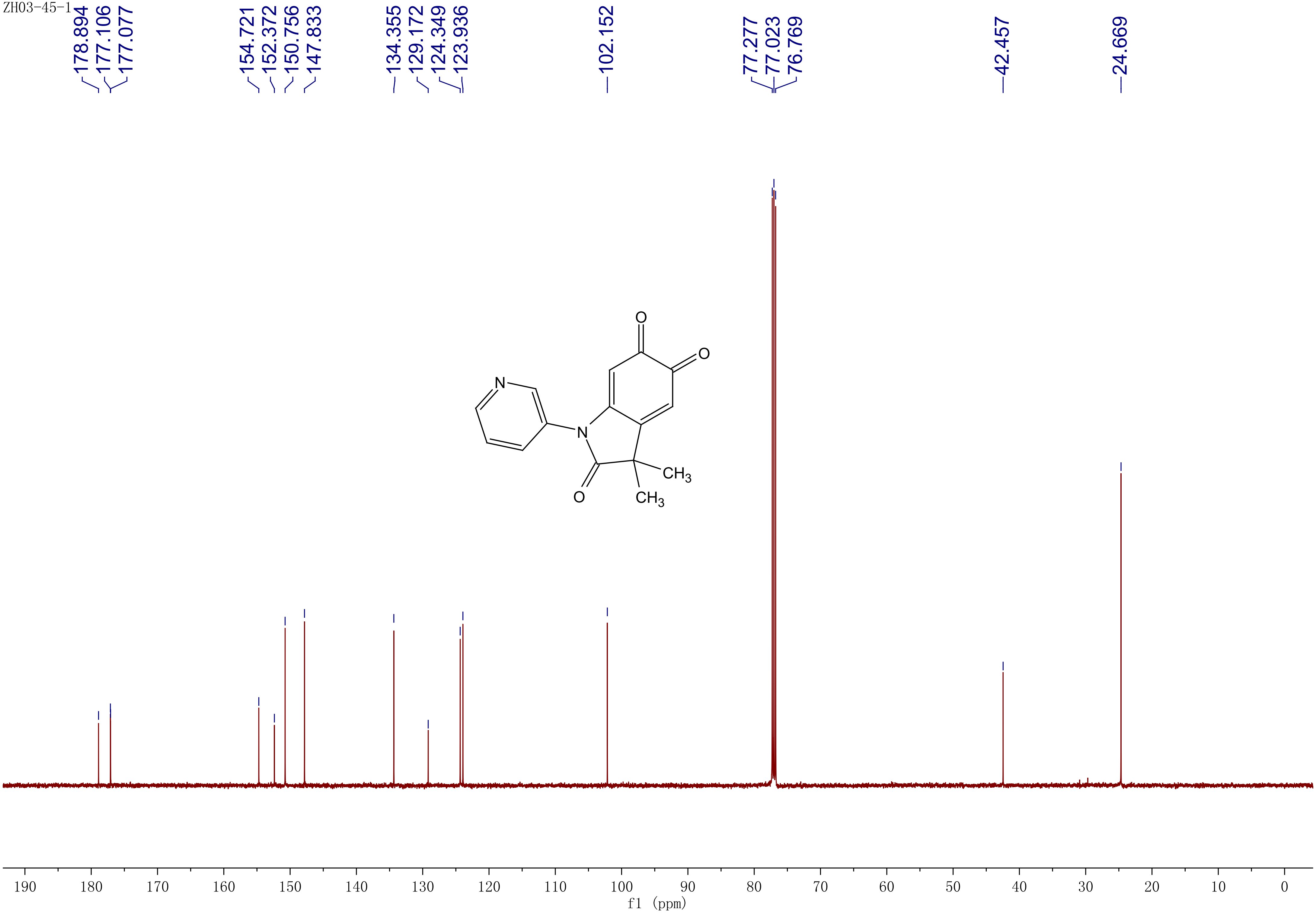

178.894
177.106
177.077
154.721
152.372
150.756
147.833
134.355
129.172
124.349
123.936
102.152
77.277
77.023
76.769
42.457
24.669
N
N
O
O
O
CH3
CH3
190 180 170 160 150 140 130 120 110 100 90 80 70 60 50 40 30 20 10 0
f1 (ppm)

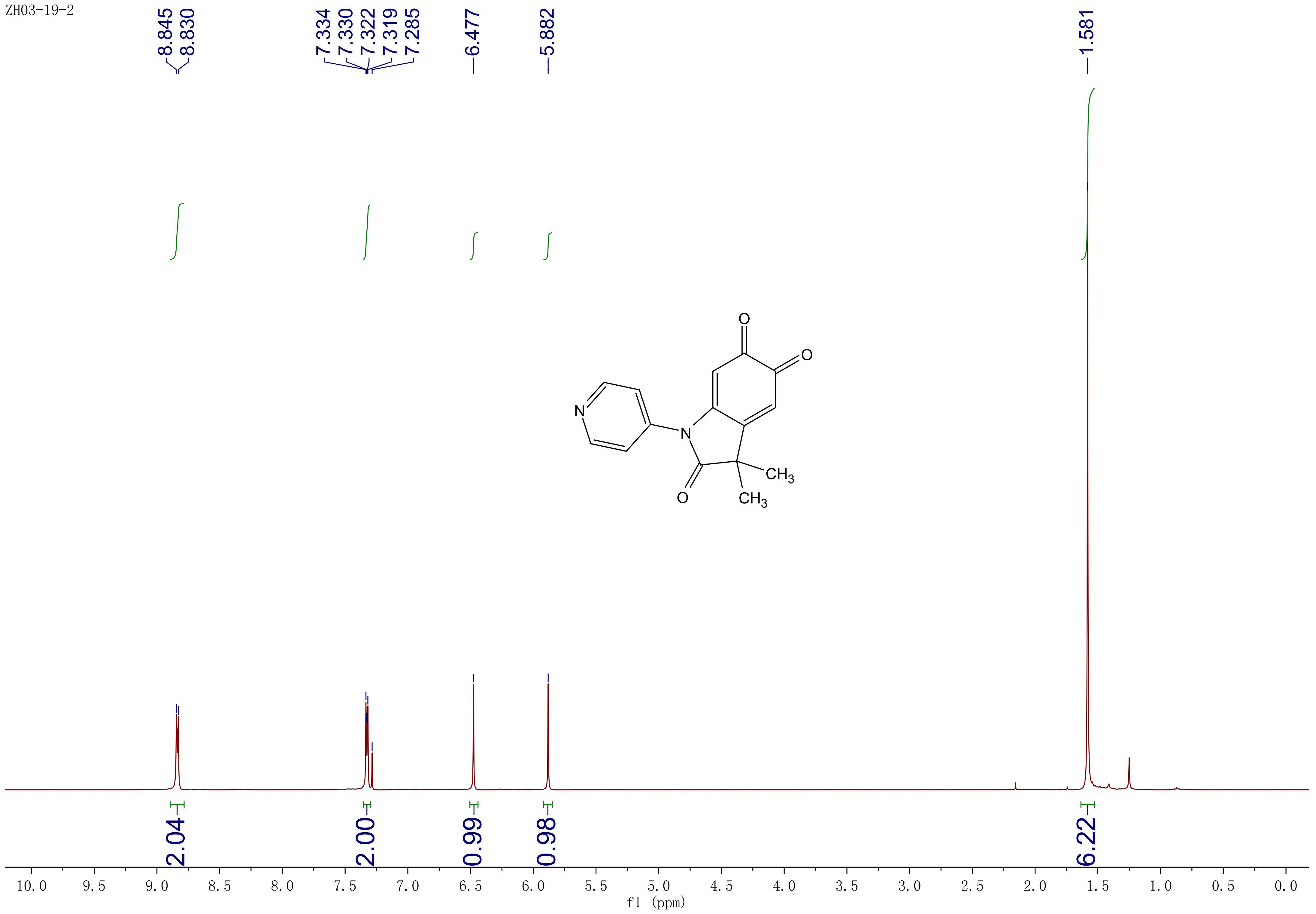
ZH03-19-2
8.845
8.830
7.334
7.330
7.322
7.319
7.285
6.477
5.882
1.581
O
O
N
N
O
CH3
CH3
2.04
2.00
0.99
0.98
6.22
10.0 9.5 9.0 8.5 8.0 7.5 7.0 6.5 6.0 5.5 5.0 4.5 4.0 3.5 3.0 2.5 2.0 1.5 1.0 0.5 0.0
f1 (ppm)

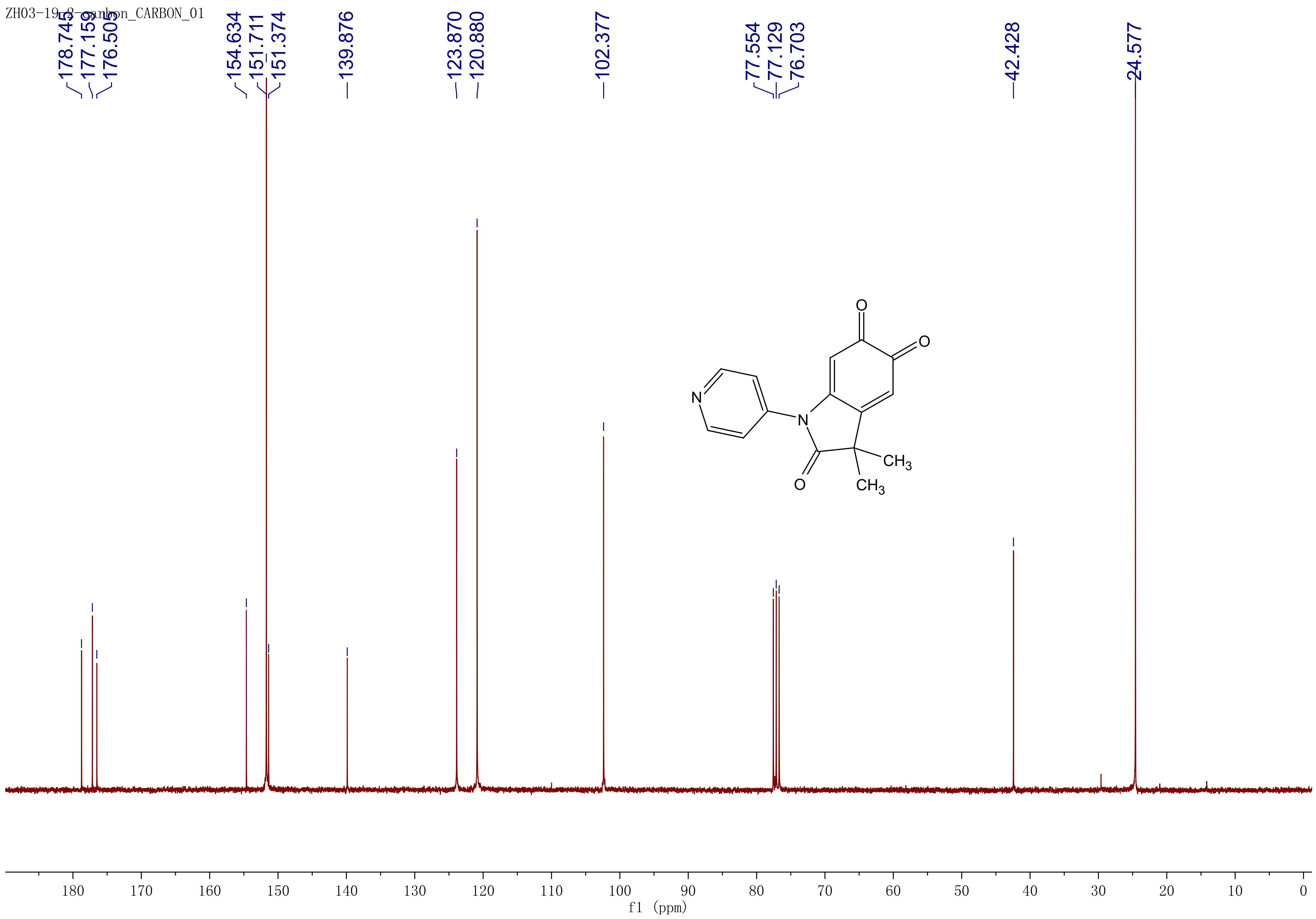

ZH03-19-2-carbon_CARBON_01
178.745
177.159
176.505
154.634
151.711
151.374
139.876
123.870
120.880
102.377
77.554
77.129
76.703
42.428
24.577
N
N
O
O
O
CH3
CH3
180
170
160
150
140
130
120
110
100
90
80
70
60
50
40
30
20
10
0
f1 (ppm)

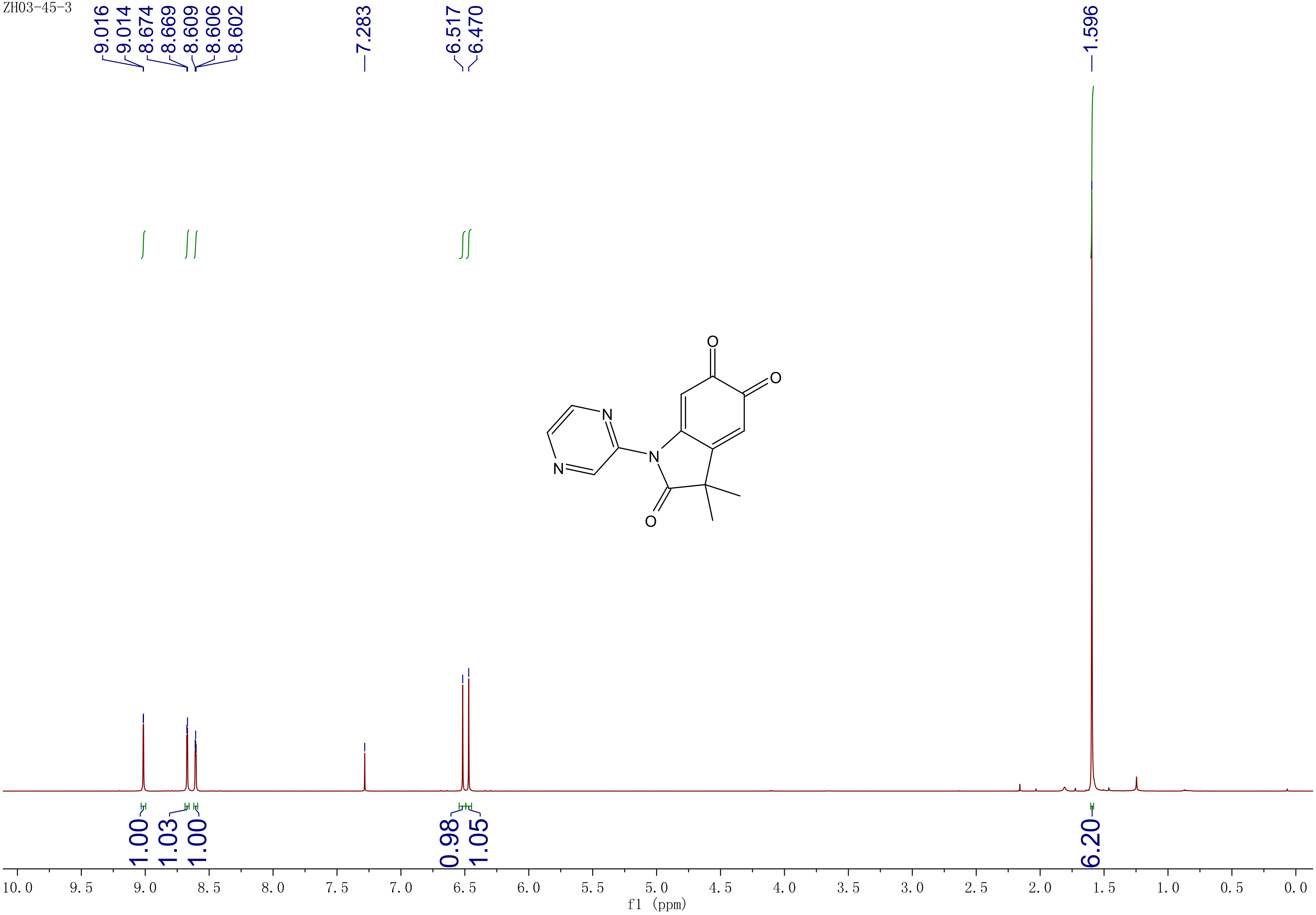
ZH03-45-3
9.016
9.014
8.674
8.669
8.609
8.606
8.602
7.283
6.517
6.470
1.596
1.00
1.03
1.00
0.98
1.05
6.20
10.0
9.5
9.0
8.5
8.0
7.5
7.0
6.5
6.0
5.5
5.0
4.5
4.0
3.5
3.0
2.5
2.0
1.5
1.0
0.5
0.0
f1 (ppm)
O
O
N
N
N
O

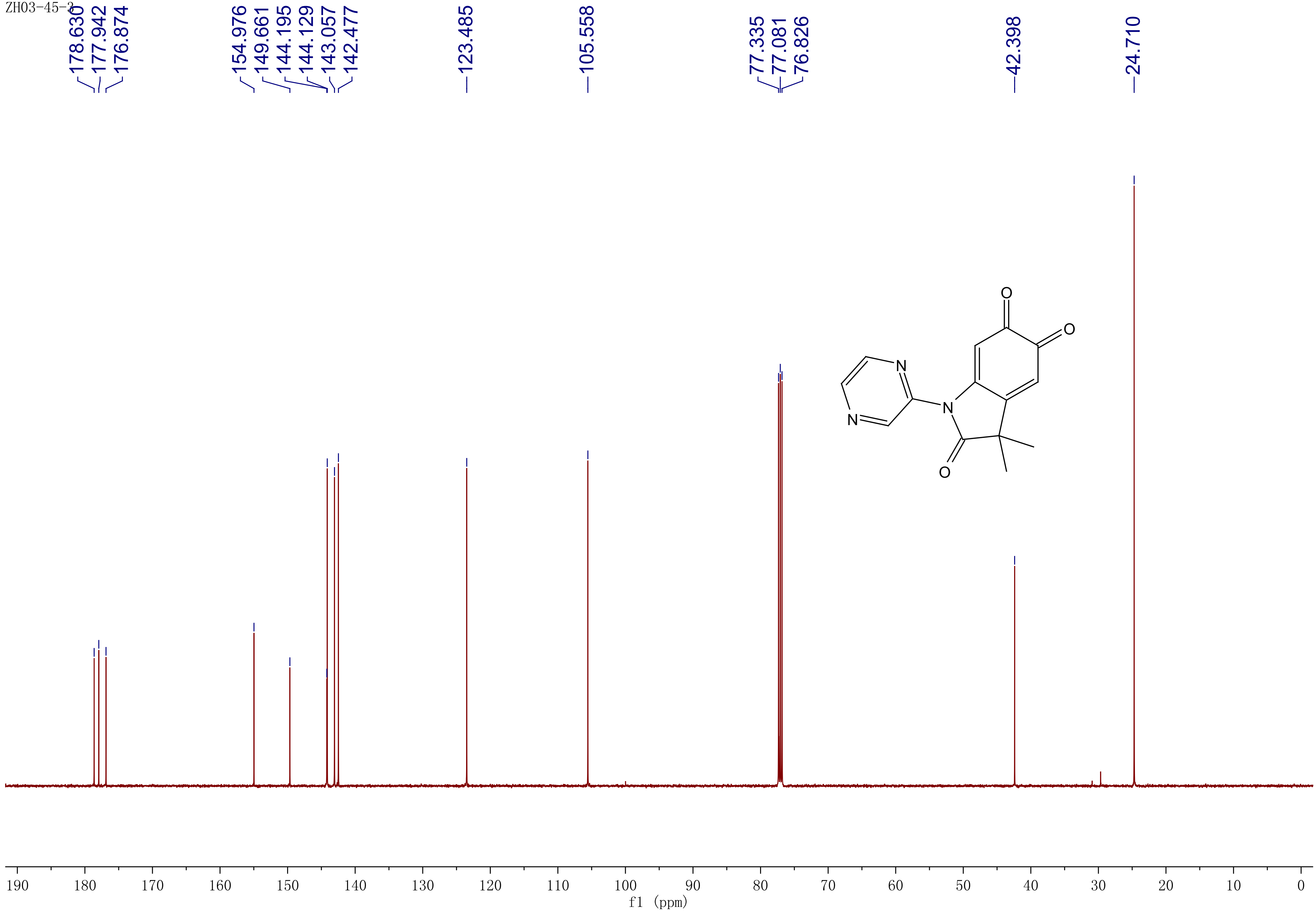

ZH03-45-3
178.630
177.942
176.874
154.976
149.661
144.195
144.129
143.057
142.477
123.485
105.558
77.335
77.081
76.826
42.398
24.710
O
O
N
N
N
O
190
180
170
160
150
140
130
120
110
100
90
80
70
60
50
40
30
20
10
0
f1 (ppm)

ZH03-45-4

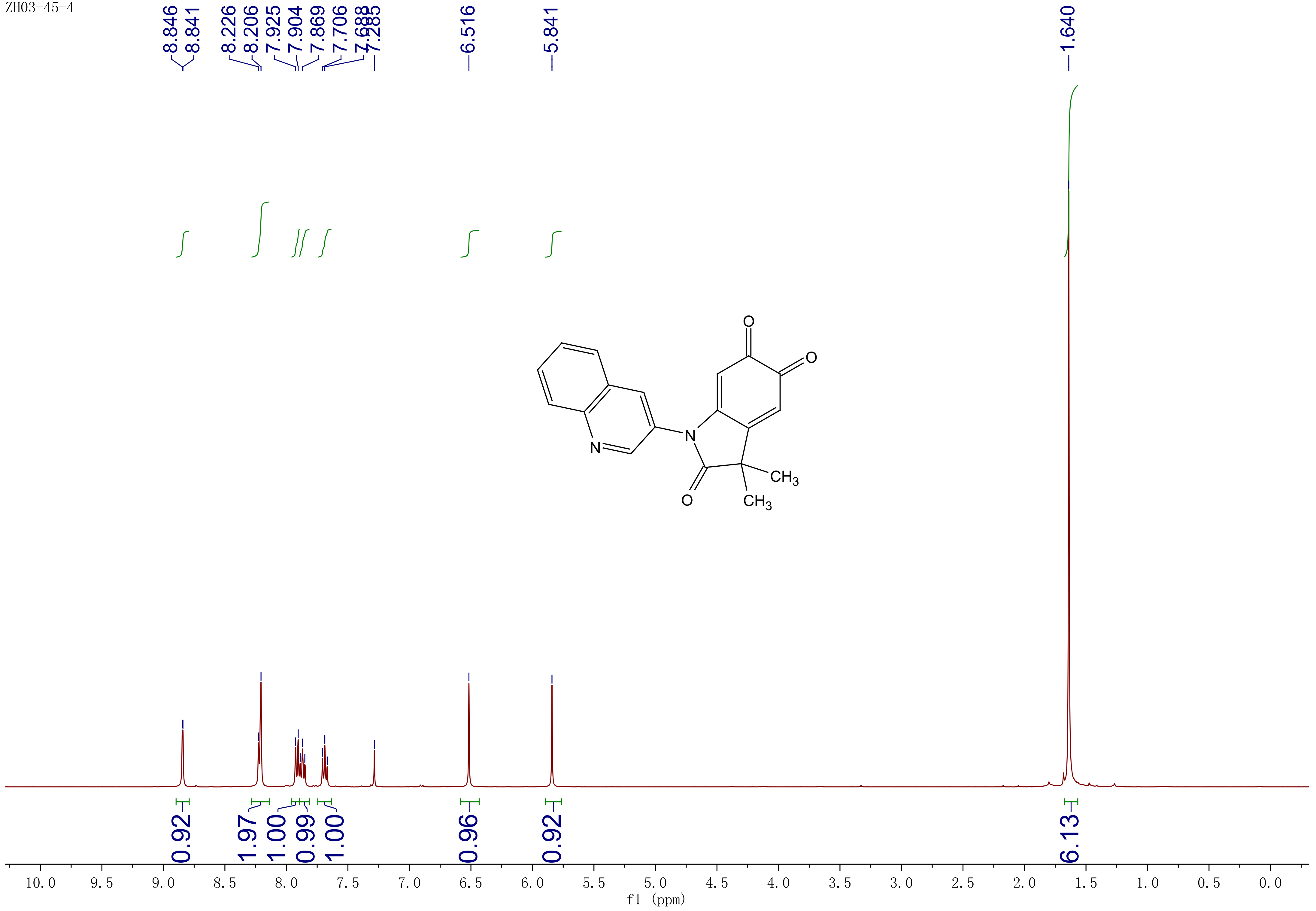

8.846
8.841
8.226
8.206
7.925
7.904
7.869
7.706
7.688
7.285
6.516
5.841
1.640
O
O
N
N
O
CH3
CH3
0.92
1.97
1.00
0.99
1.00
0.96
0.92
6.13
10.0
9.5
9.0
8.5
8.0
7.5
7.0
6.5
6.0
5.5
5.0
4.5
4.0
3.5
3.0
2.5
2.0
1.5
1.0
0.5
0.0
f1 (ppm)

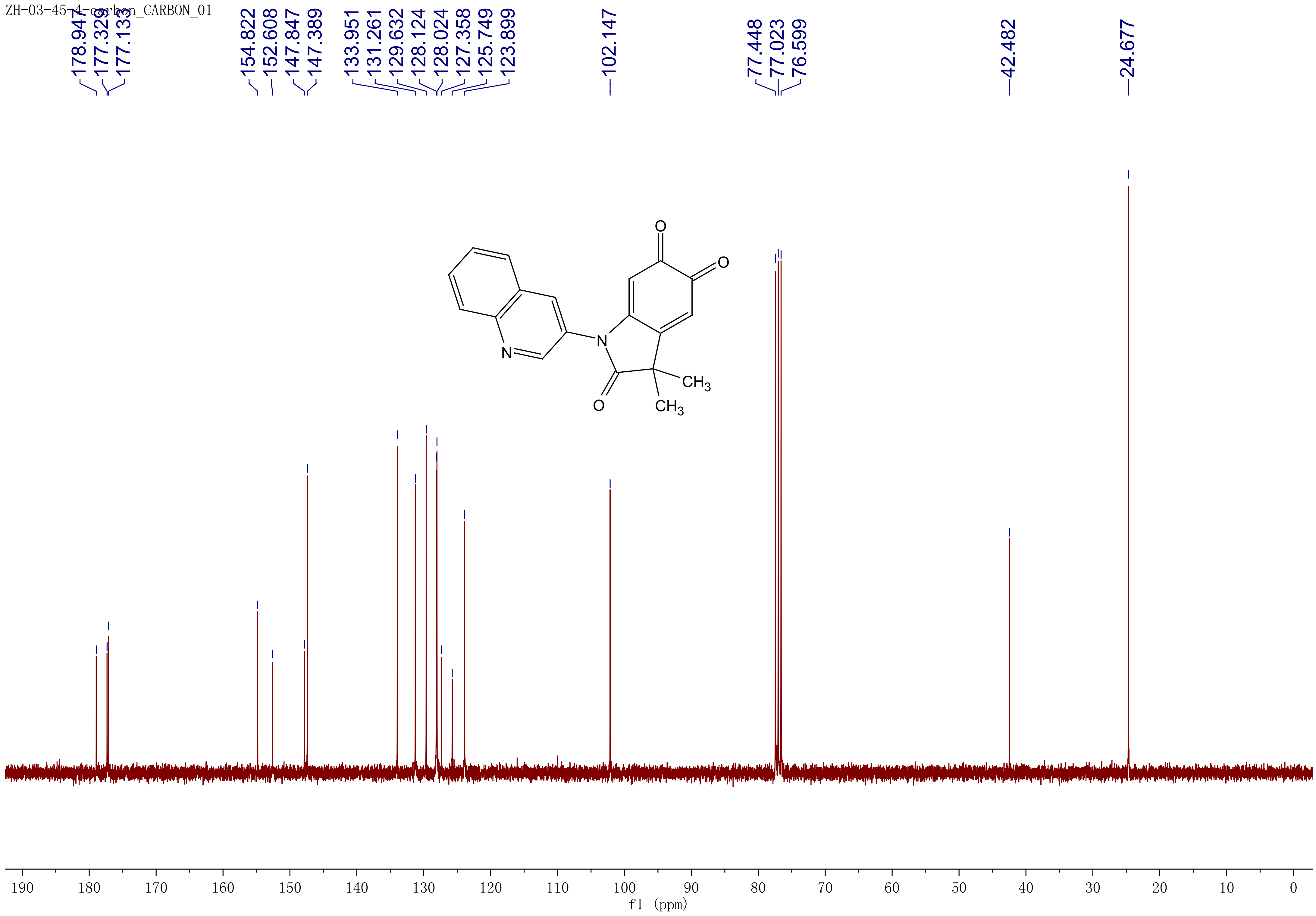

ZH-03-45-4-carbon_CARBON_01
178.947
177.329
177.133
154.822
152.608
147.847
147.389
133.951
131.261
129.632
128.124
128.024
127.358
125.749
123.899
102.147
77.448
77.023
76.599
42.482
24.677
O
O
N
N
O
CH3
CH3
190 180 170 160 150 140 130 120 110 100 90 80 70 60 50 40 30 20 10 0
f1 (ppm)

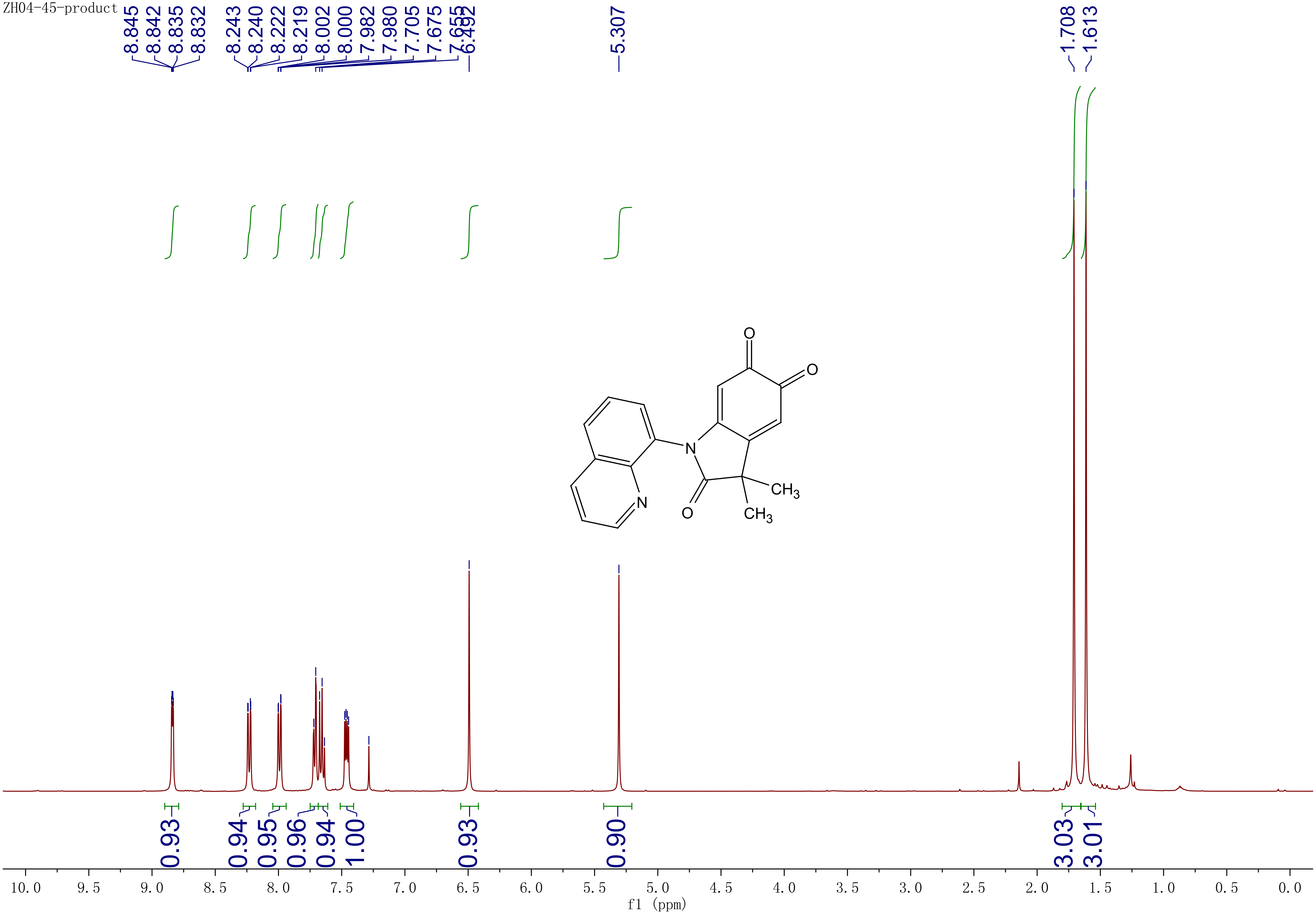
ZH04-45-product
8.845
8.842
8.835
8.832
8.243
8.240
8.222
8.219
8.002
8.000
7.982
7.980
7.705
7.675
7.655
6.492
5.307
1.708
1.613
0.93
0.94
0.95
0.96
0.94
1.00
0.93
0.90
3.03
3.01
O
O
N
O
N
CH3
CH3
10.0 9.5 9.0 8.5 8.0 7.5 7.0 6.5 6.0 5.5 5.0 4.5 4.0 3.5 3.0 2.5 2.0 1.5 1.0 0.5 0.0
f1 (ppm)

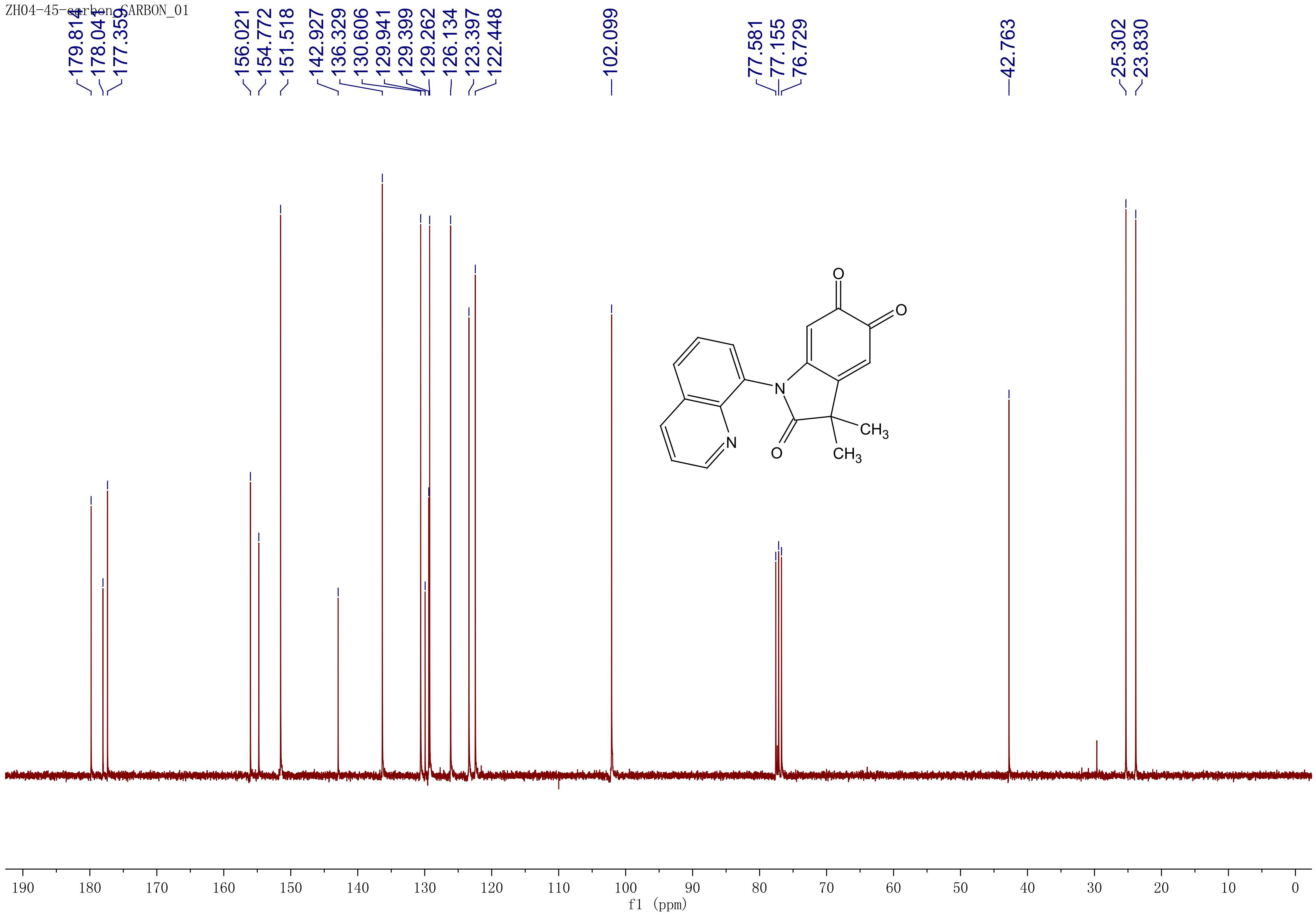
ZH04-45-carbon CARBON_01
179.814
178.041
177.359
156.021
154.772
151.518
142.927
136.329
130.606
129.941
129.399
129.262
126.134
123.397
122.448
102.099
77.581
77.155
76.729
42.763
25.302
23.830
O
O
N
N
O
CH3
CH3
190 180 170 160 150 140 130 120 110 100 90 80 70 60 50 40 30 20 10 0
f1 (ppm)

ZH04-15-2
proton-acetone

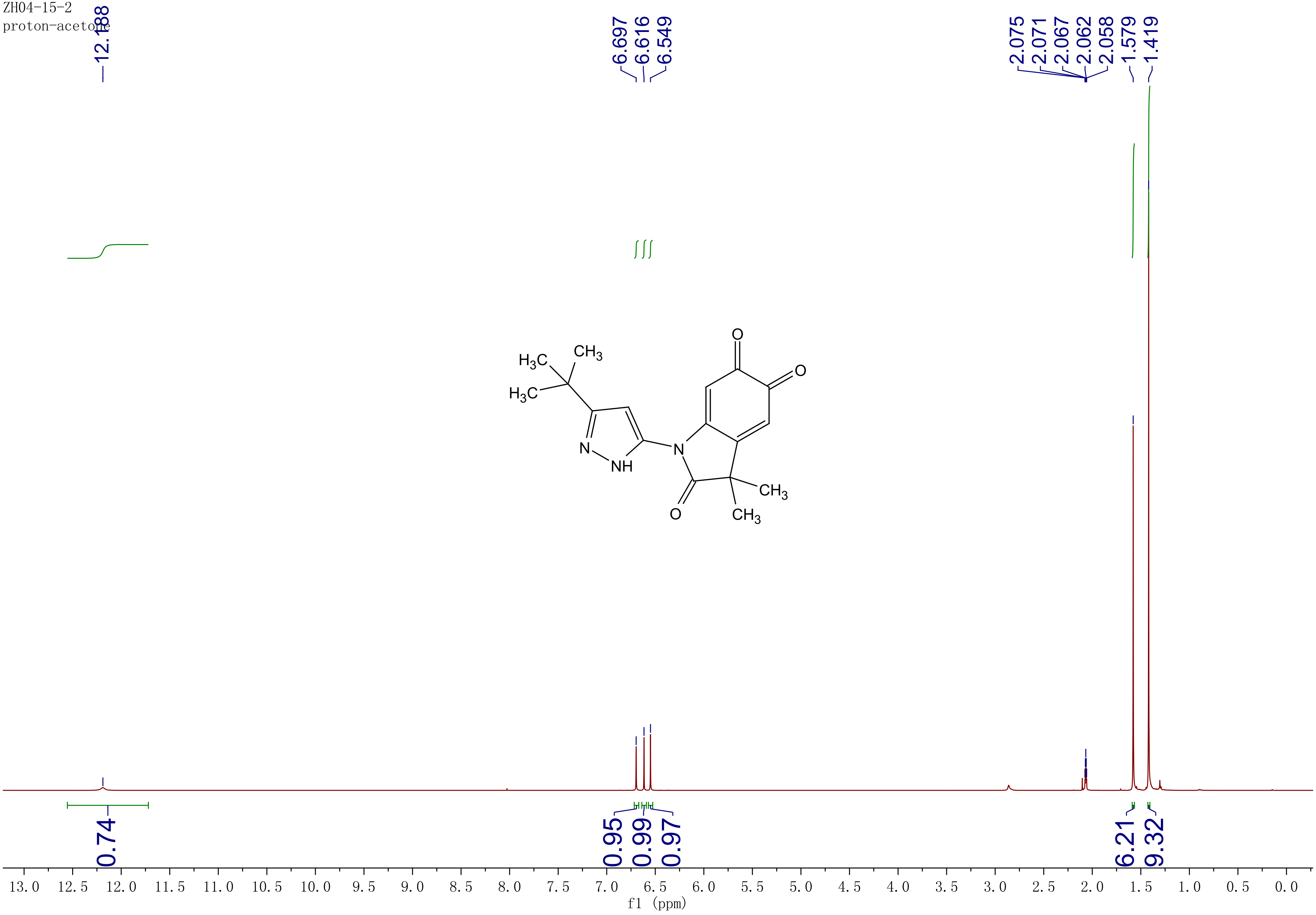

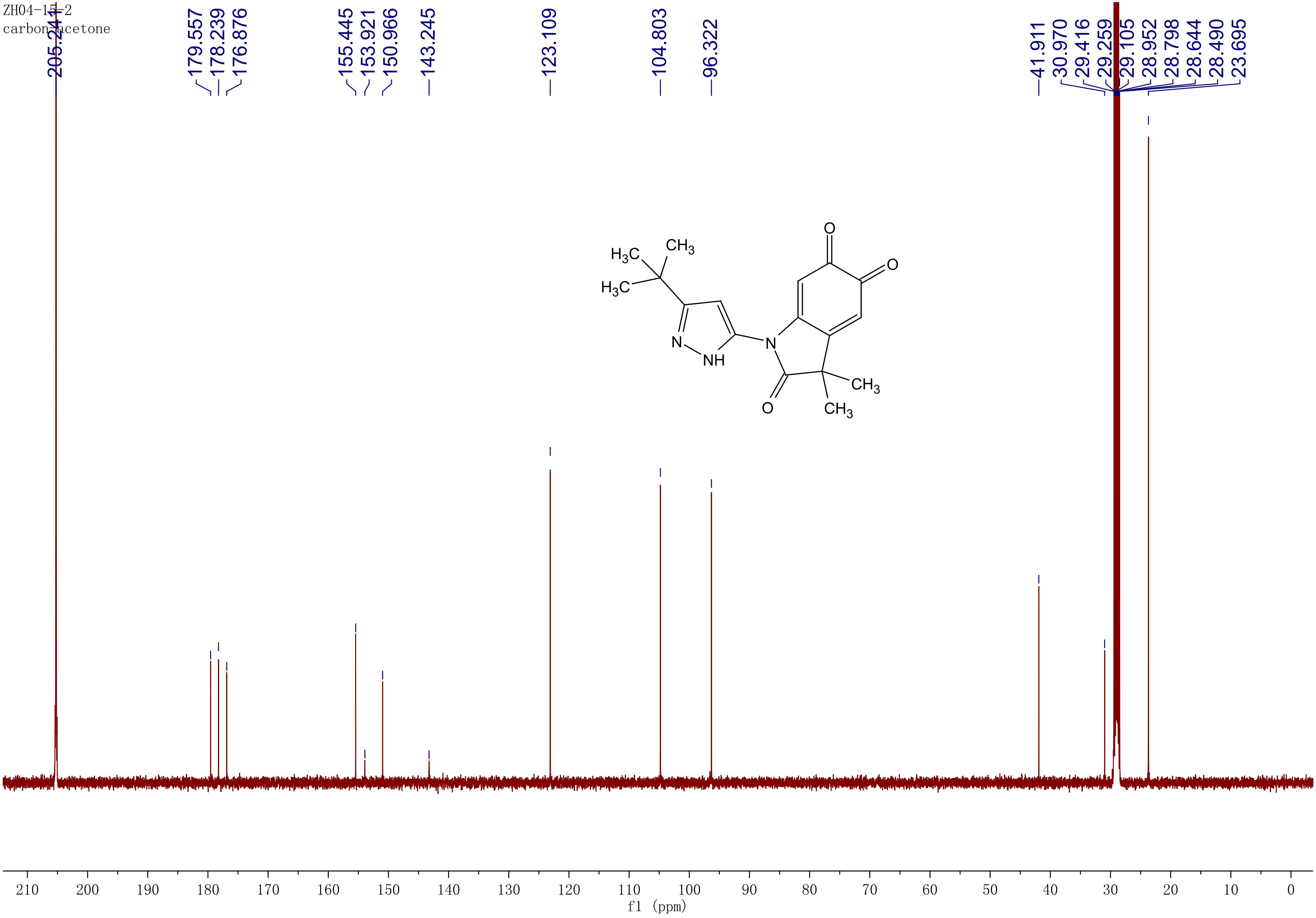
ZH04-15-2
carbon-acetone
205.241
179.557
178.239
176.876
155.445
153.921
150.966
143.245
123.109
104.803
96.322
41.911
30.970
29.416
29.259
29.105
28.952
28.798
28.644
28.490
23.695
H3C
CH3
H3C
O
O
N
NH
N
O
CH3
CH3
210 200 190 180 170 160 150 140 130 120 110 100 90 80 70 60 50 40 30 20 10 0
f1 (ppm)

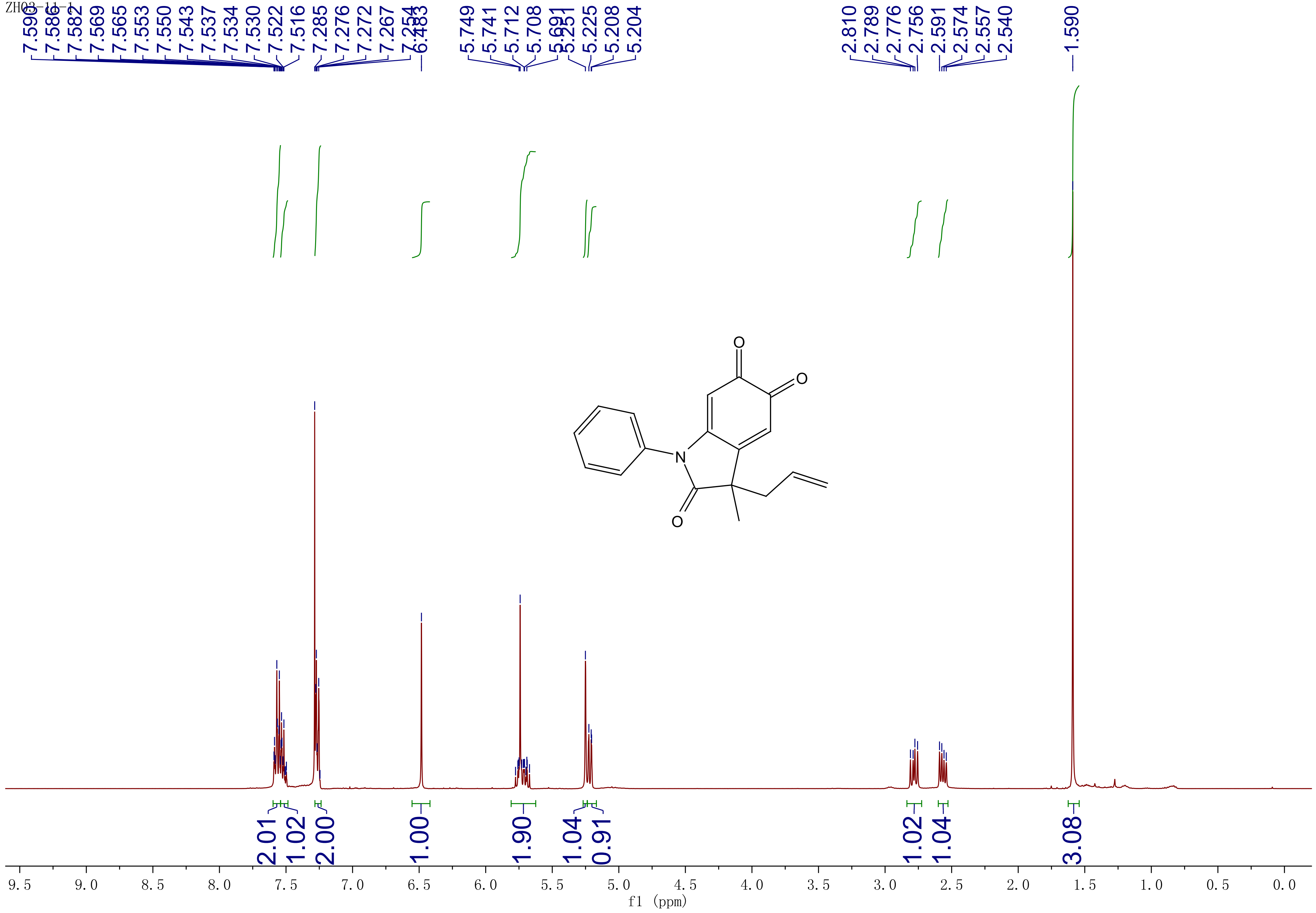
ZH03-11-1
7.590 7.586 7.582 7.569 7.565 7.553 7.550 7.543 7.537 7.534 7.530 7.522 7.516 7.285 7.276 7.272 7.267 7.254 6.483
5.749 5.741 5.712 5.708 5.691 5.251 5.225 5.208 5.204
2.810 2.789 2.776 2.756 2.591 2.574 2.557 2.540
1.590
2.01 1.02 2.00 1.00 1.90 1.04 0.91 1.02 1.04 3.08
O
O
N
O
9.5 9.0 8.5 8.0 7.5 7.0 6.5 6.0 5.5 5.0 4.5 4.0 3.5 3.0 2.5 2.0 1.5 1.0 0.5 0.0
f1 (ppm)

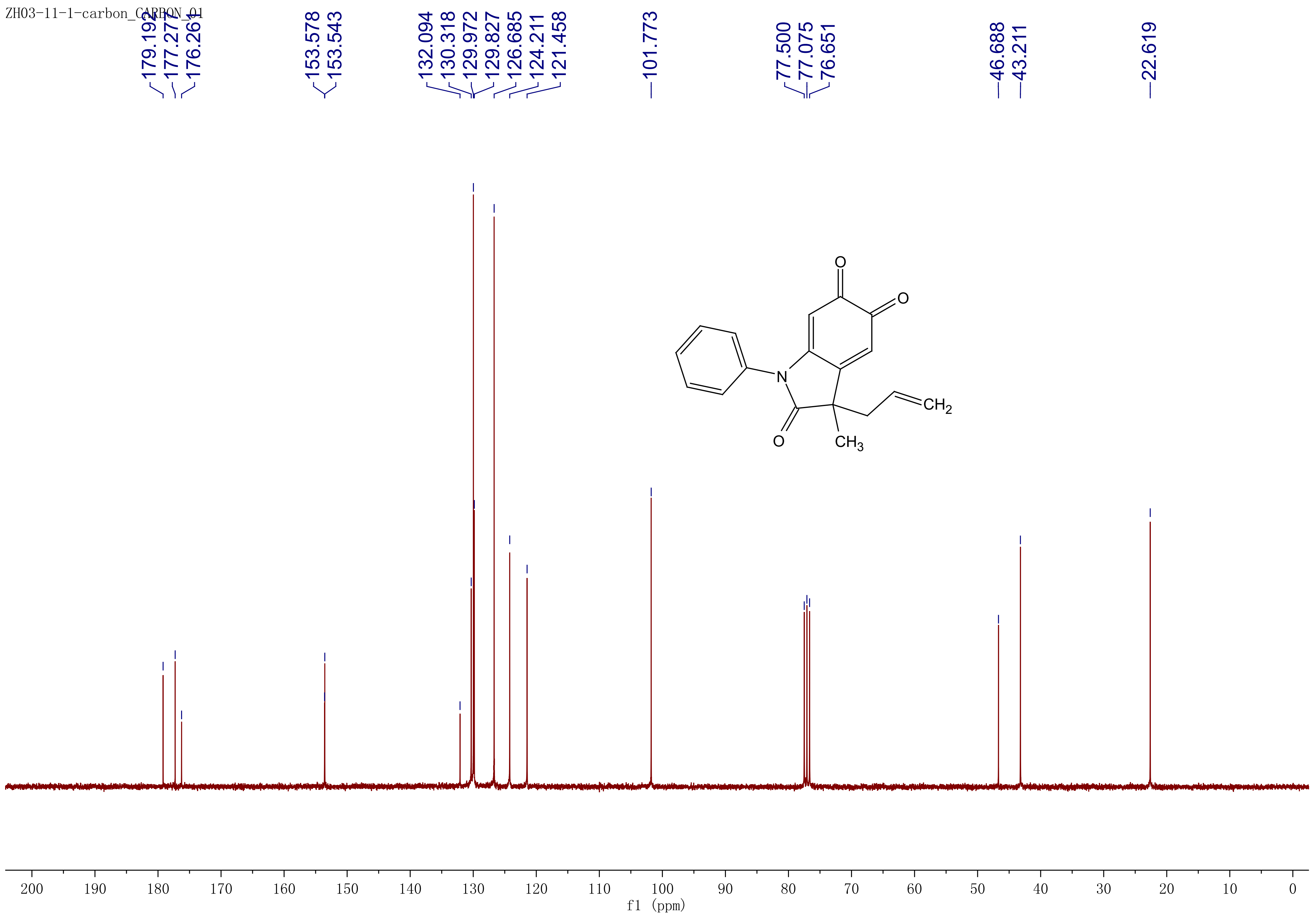

ZH03-11-1-carbon_CARBON_01
179.192
177.277
176.261
153.578
153.543
132.094
130.318
129.972
129.827
126.685
124.211
121.458
101.773
77.500
77.075
76.651
46.688
43.211
22.619
O
O
N
CH2
O
CH3
200
190
180
170
160
150
140
130
120
110
100
90
80
70
60
50
40
30
20
10
0
f1 (ppm)

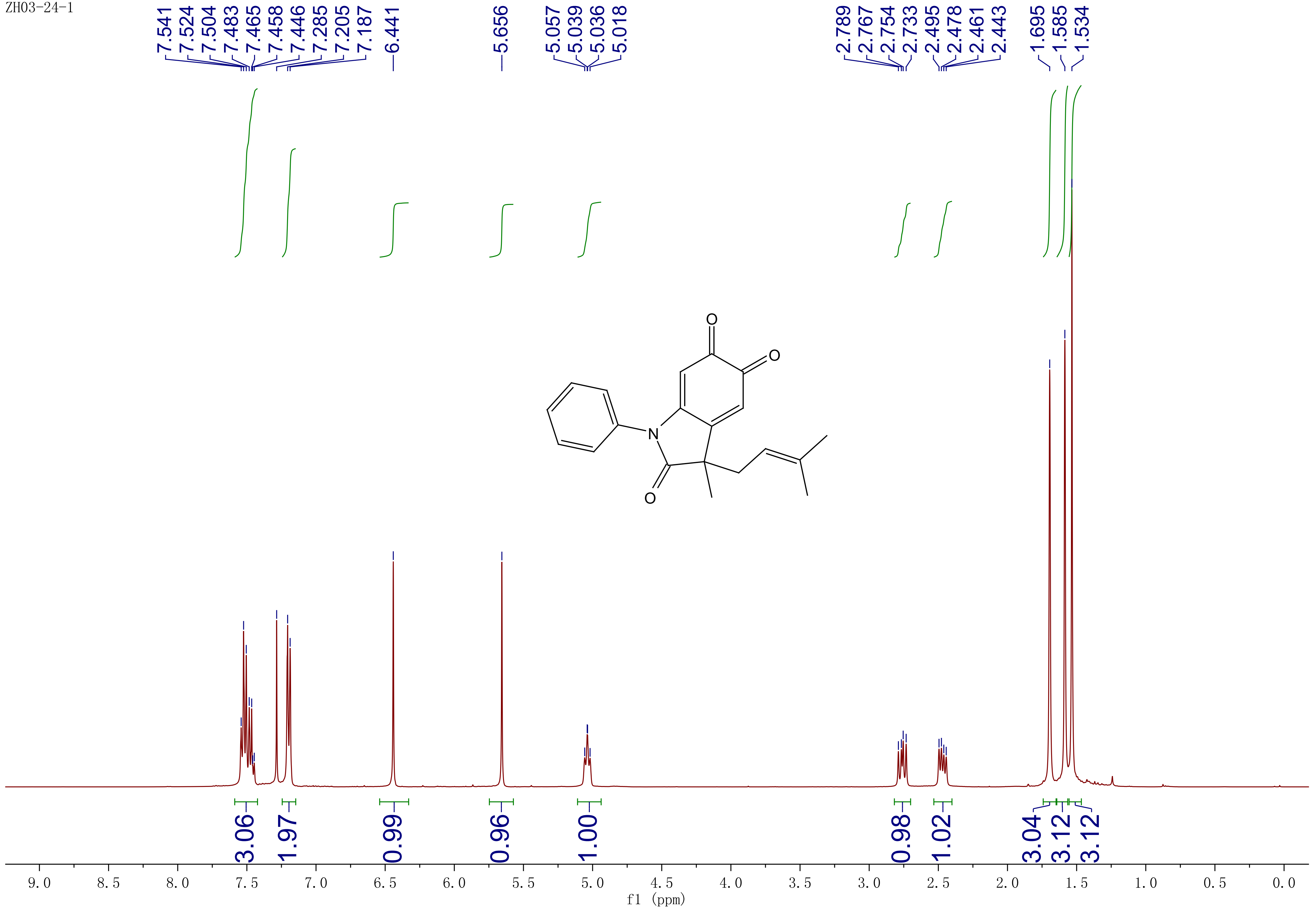
ZH03-24-1
7.541
7.524
7.504
7.483
7.465
7.458
7.446
7.285
7.205
7.187
6.441
5.656
5.057
5.039
5.036
5.018
2.789
2.767
2.754
2.733
2.495
2.478
2.461
2.443
1.695
1.585
1.534
3.06
1.97
0.99
0.96
1.00
0.98
1.02
3.04
3.12
3.12
O
O
N
O
9.0 8.5 8.0 7.5 7.0 6.5 6.0 5.5 5.0 4.5 4.0 3.5 3.0 2.5 2.0 1.5 1.0 0.5 0.0
f1 (ppm)

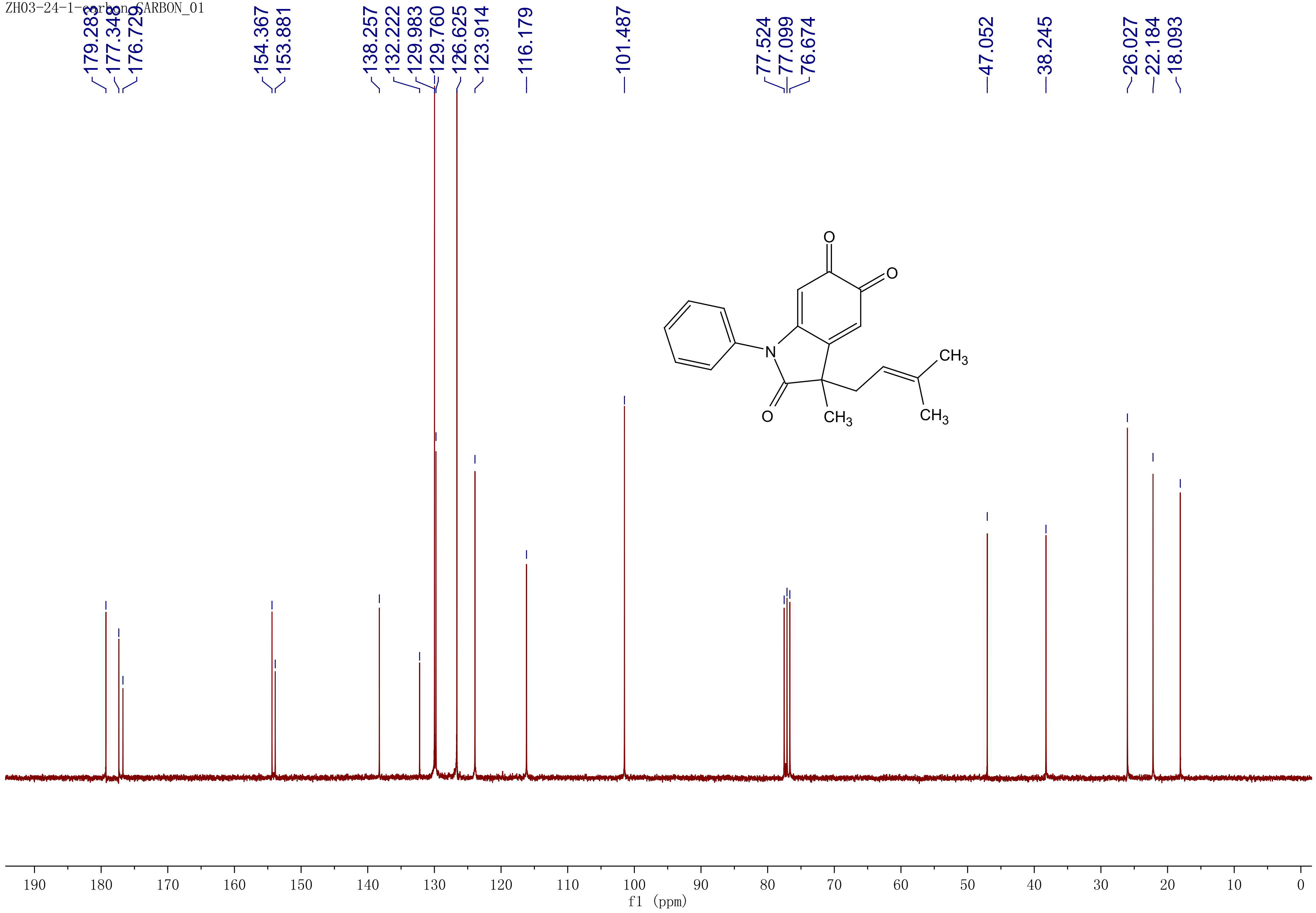
ZH03-24-1-carbon_CARBON_01
179.283
177.348
176.729
154.367
153.881
138.257
132.222
129.983
129.760
126.625
123.914
116.179
101.487
77.524
77.099
76.674
47.052
38.245
26.027
22.184
18.093
O
O
N
O
CH3
CH3
CH3
190 180 170 160 150 140 130 120 110 100 90 80 70 60 50 40 30 20 10 0
f1 (ppm)

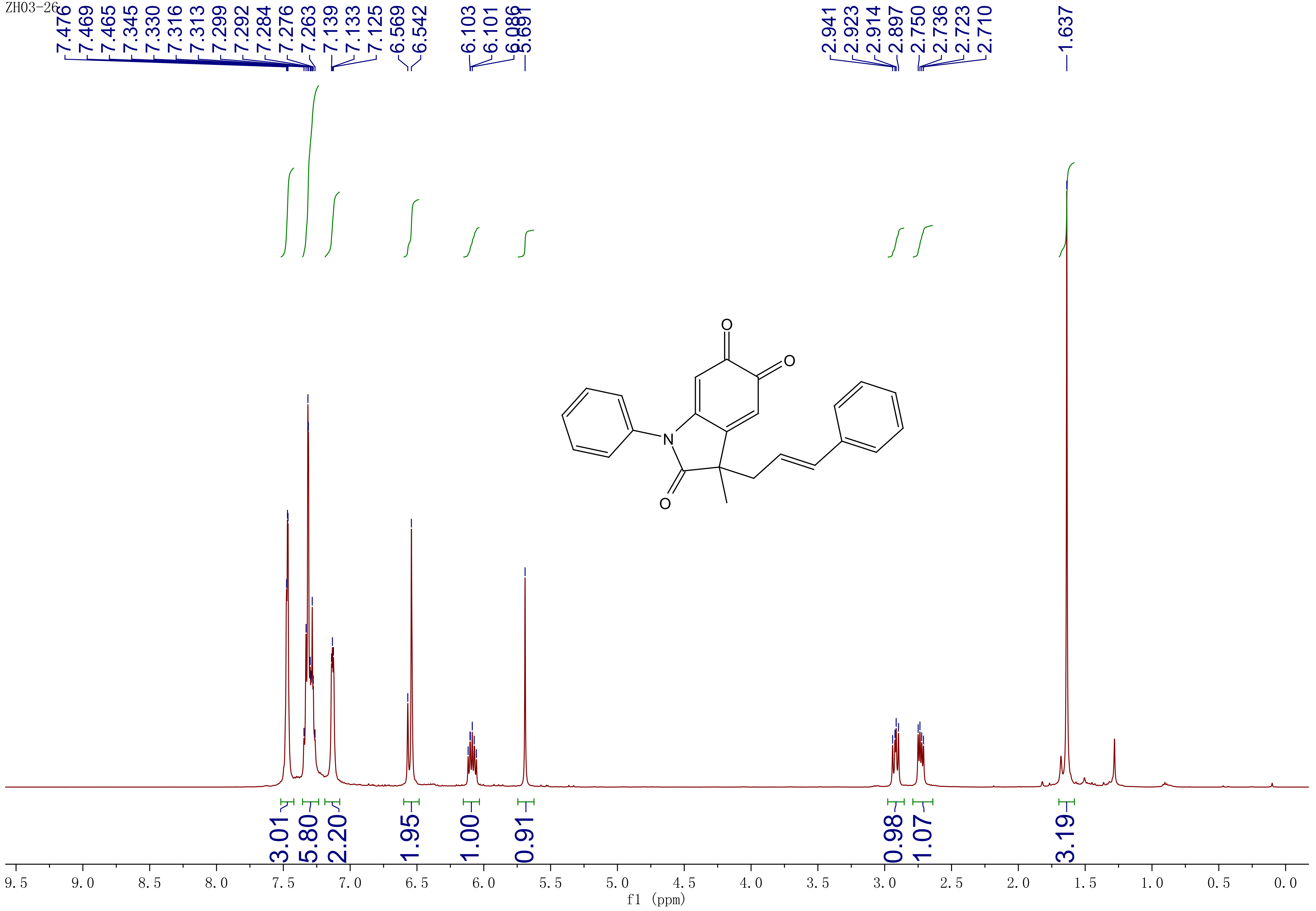
ZH03-26
7.476
7.469
7.465
7.345
7.330
7.316
7.313
7.299
7.292
7.284
7.276
7.263
7.139
7.133
7.125
6.569
6.542
6.103
6.101
6.086
5.691
2.941
2.923
2.914
2.897
2.750
2.736
2.723
2.710
1.637
O
O
N
O
3.01
5.80
2.20
1.95
1.00
0.91
0.98
1.07
3.19
9.5
9.0
8.5
8.0
7.5
7.0
6.5
6.0
5.5
5.0
4.5
4.0
3.5
3.0
2.5
2.0
1.5
1.0
0.5
0.0
f1 (ppm)

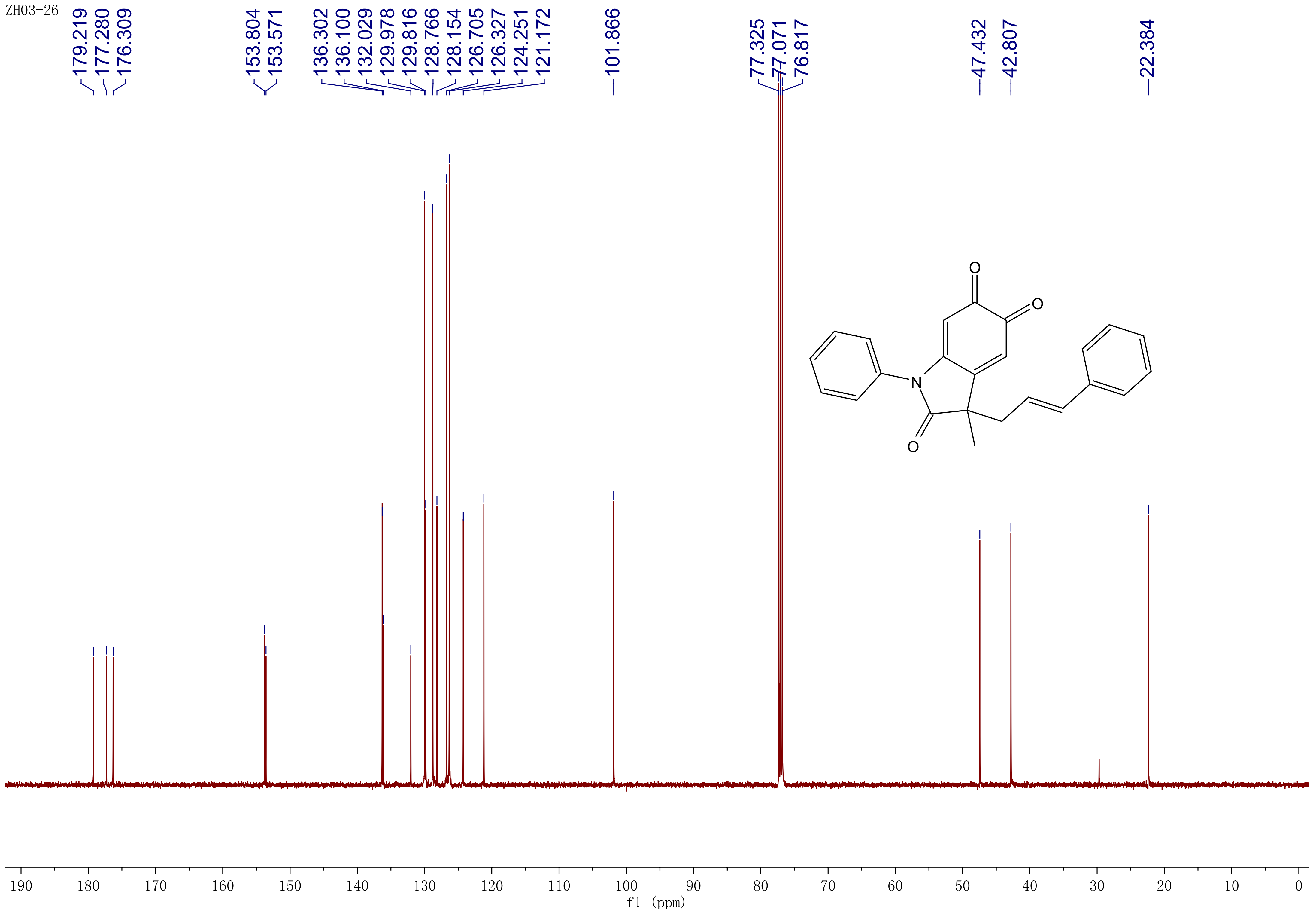
ZH03-26
179.219
177.280
176.309
153.804
153.571
136.302
136.100
132.029
129.978
129.816
128.766
128.154
126.705
126.327
124.251
121.172
101.866
77.325
77.071
76.817
47.432
42.807
22.384
190 180 170 160 150 140 130 120 110 100 90 80 70 60 50 40 30 20 10 0
f1 (ppm)
O
O
N
O

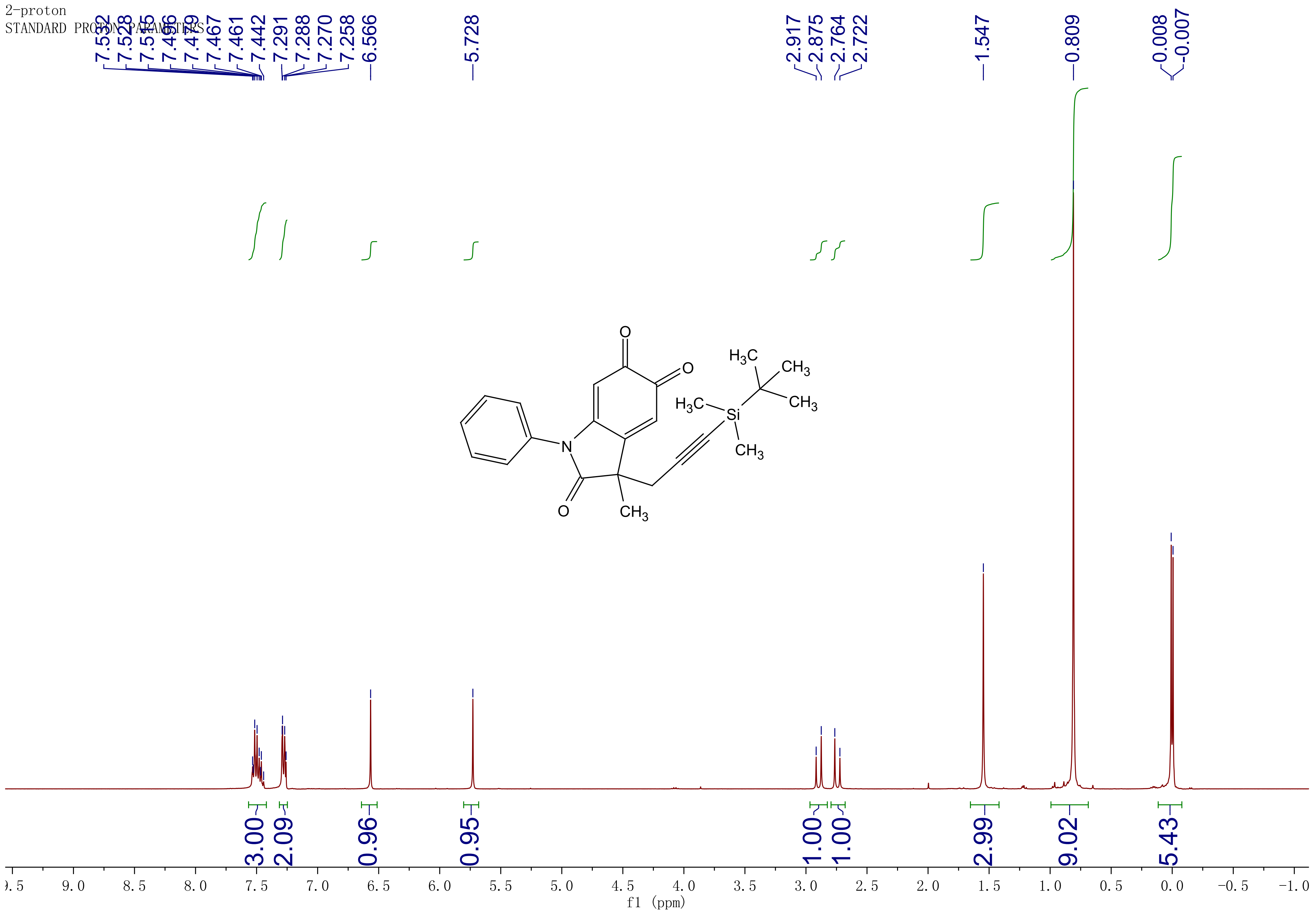
2-proton
STANDARD PROTON PARAMETERS
7.532
7.528
7.515
7.496
7.479
7.467
7.461
7.442
7.291
7.288
7.270
7.258
6.566
5.728
2.917
2.875
2.764
2.722
1.547
0.809
0.008
-0.007
O
O
H3C
CH3
CH3
H3C
Si
N
CH3
O
CH3
3.00
2.09
0.96
0.95
1.00
1.00
2.99
9.02
5.43
f1 (ppm)

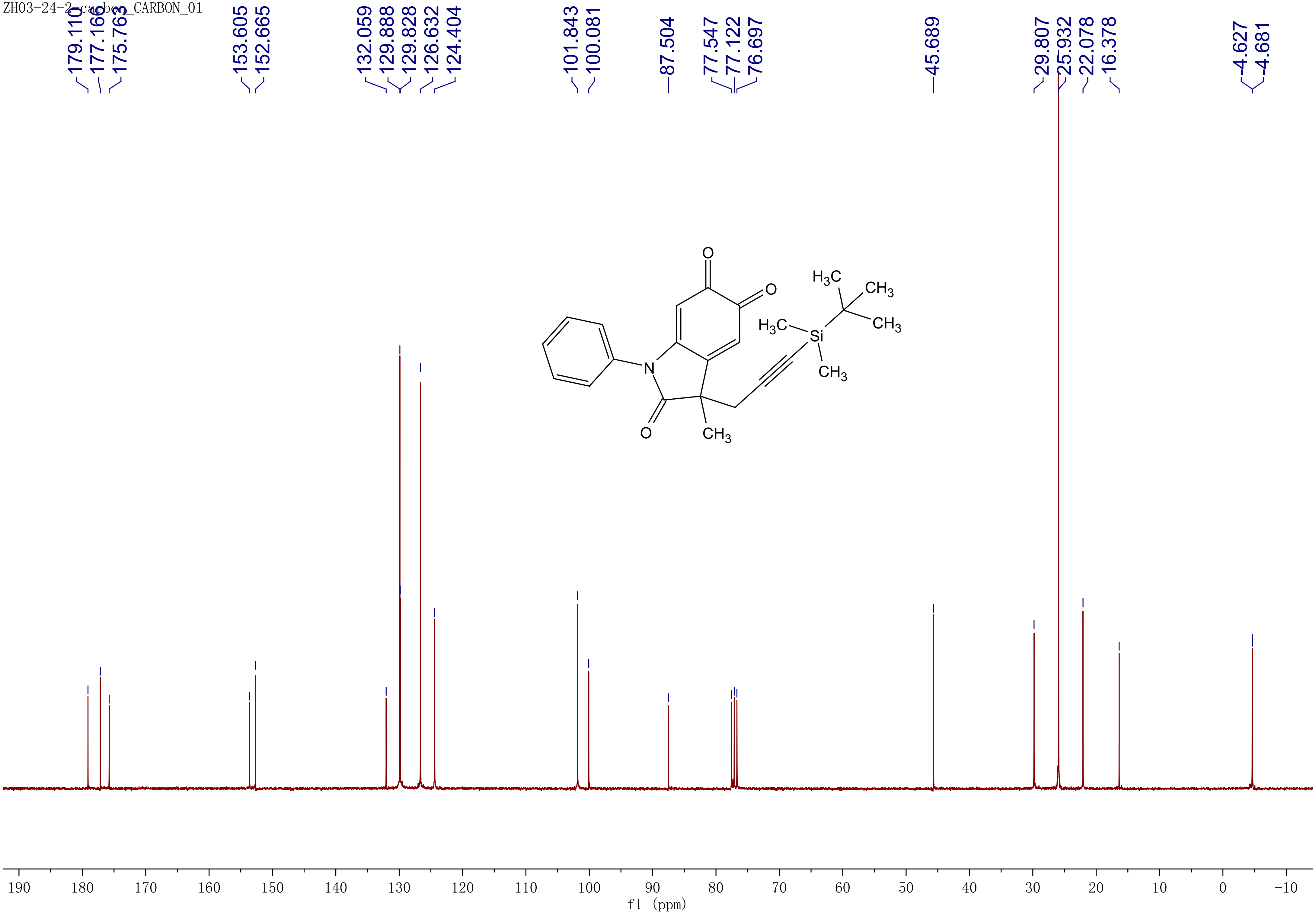

ZH03-24-2-carbon_CARBON_01
179.110
177.166
175.763
153.605
152.665
132.059
129.888
129.828
126.632
124.404
101.843
100.081
87.504
77.547
77.122
76.697
45.689
29.807
25.932
22.078
16.378
-4.627
-4.681
O
O
H3C
CH3
H3C
Si
CH3
CH3
N
O
CH3
190 180 170 160 150 140 130 120 110 100 90 80 70 60 50 40 30 20 10 0 -10
f1 (ppm)

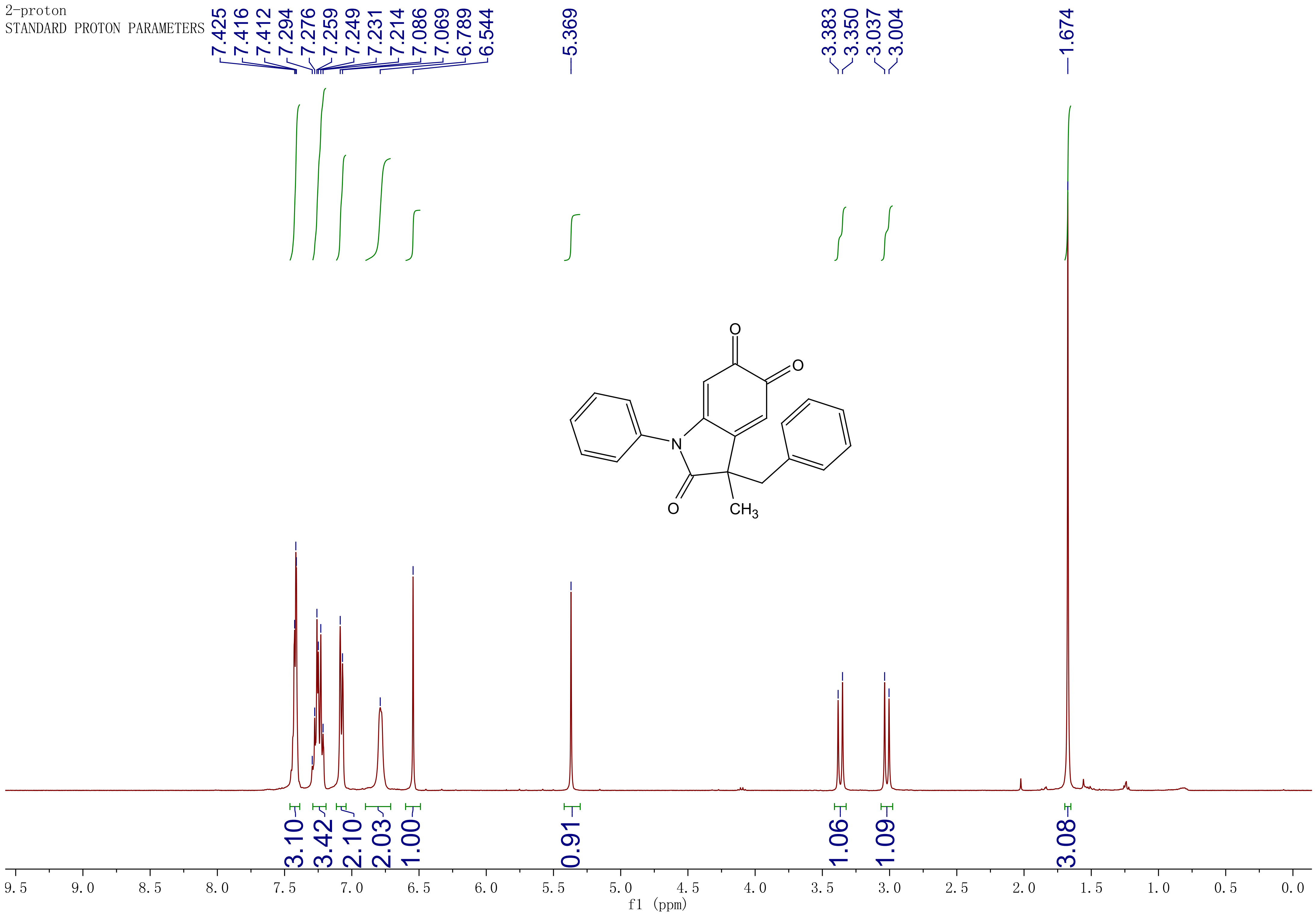
2-proton
STANDARD PROTON PARAMETERS
7.425
7.416
7.412
7.294
7.276
7.259
7.249
7.231
7.214
7.086
7.069
6.789
6.544
5.369
3.383
3.350
3.037
3.004
1.674
O
O
N
O
CH3
3.10
3.42
2.10
2.03
1.00
0.91
1.06
1.09
3.08
9.5
9.0
8.5
8.0
7.5
7.0
6.5
6.0
5.5
5.0
4.5
4.0
3.5
3.0
2.5
2.0
1.5
1.0
0.5
0.0
f1 (ppm)

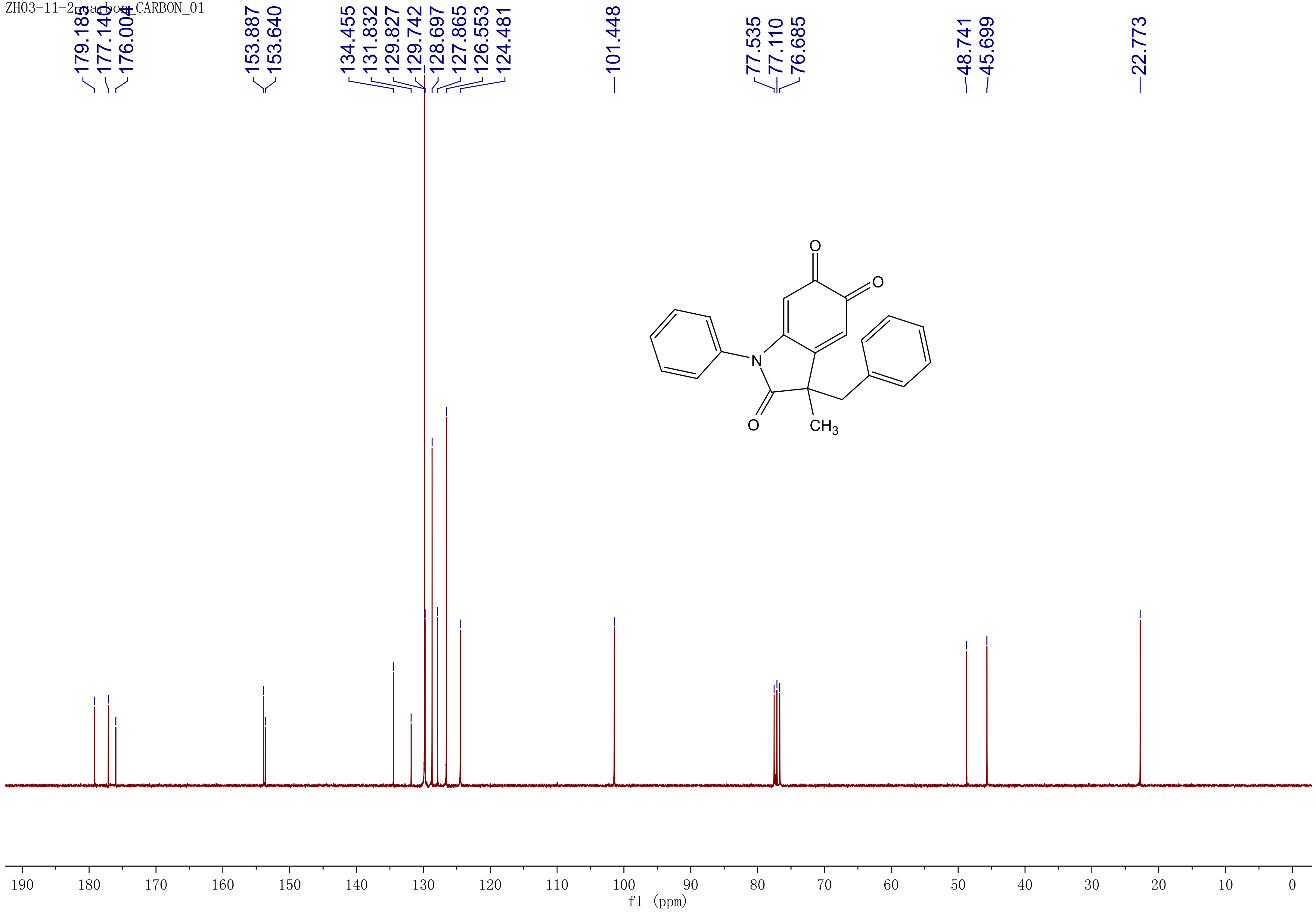
ZH03-11-2-carbon-CARBON_01
179.185
177.140
176.004
153.887
153.640
134.455
131.832
129.827
129.742
128.697
127.865
126.553
124.481
101.448
77.535
77.110
76.685
48.741
45.699
22.773
O
O
N
O
CH3
190 180 170 160 150 140 130 120 110 100 90 80 70 60 50 40 30 20 10 0
f1 (ppm)

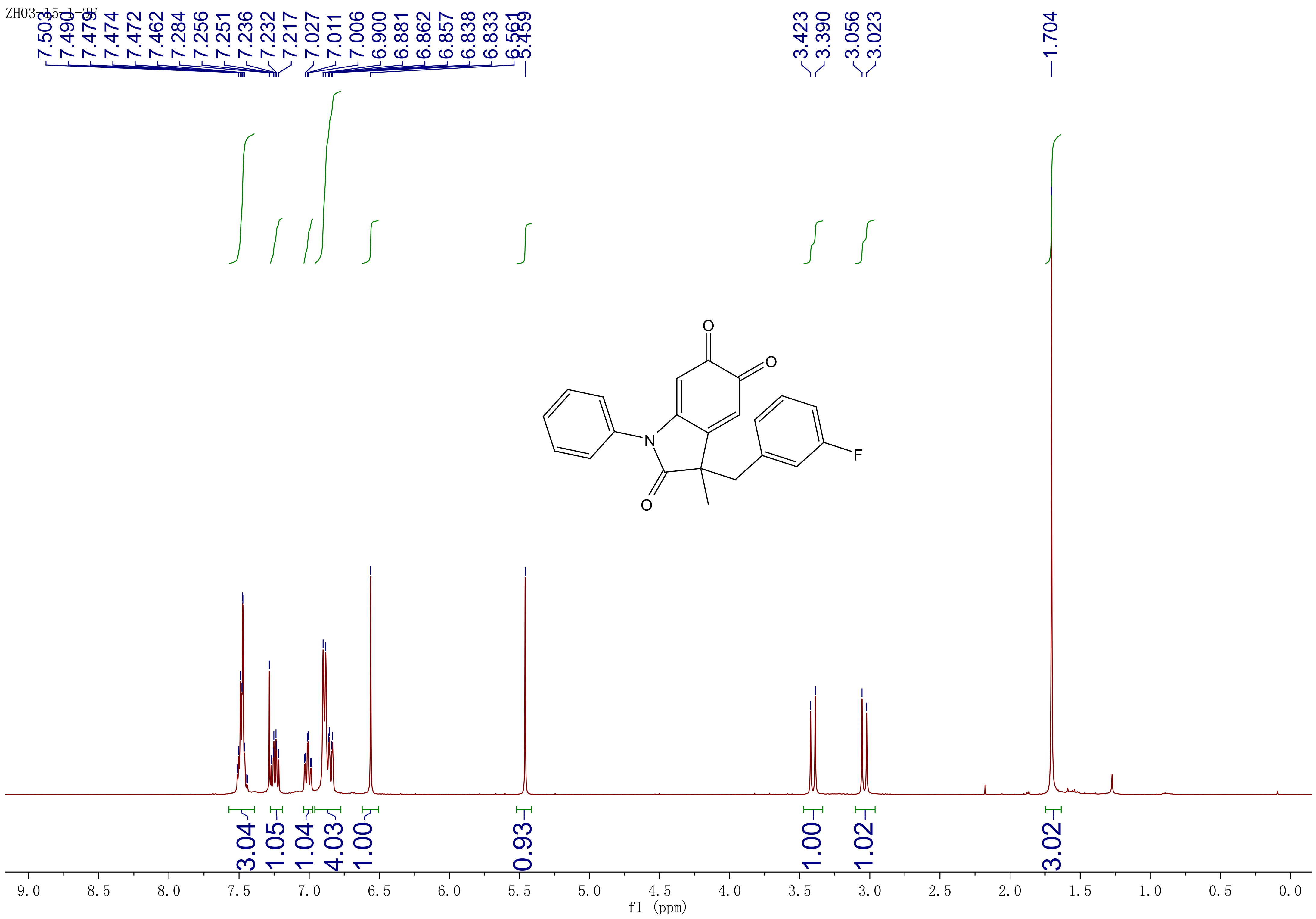

ZH03-45-1-3F
7.503
7.490
7.479
7.474
7.472
7.462
7.284
7.256
7.251
7.236
7.232
7.217
7.027
7.011
7.006
6.900
6.881
6.862
6.857
6.838
6.833
6.561
5.459
3.423
3.390
3.056
3.023
1.704
O
O
N
F
O
3.04
1.05
1.04
4.03
1.00
0.93
1.00
1.02
3.02
9.0
8.5
8.0
7.5
7.0
6.5
6.0
5.5
5.0
4.5
4.0
3.5
3.0
2.5
2.0
1.5
1.0
0.5
0.0
f1 (ppm)

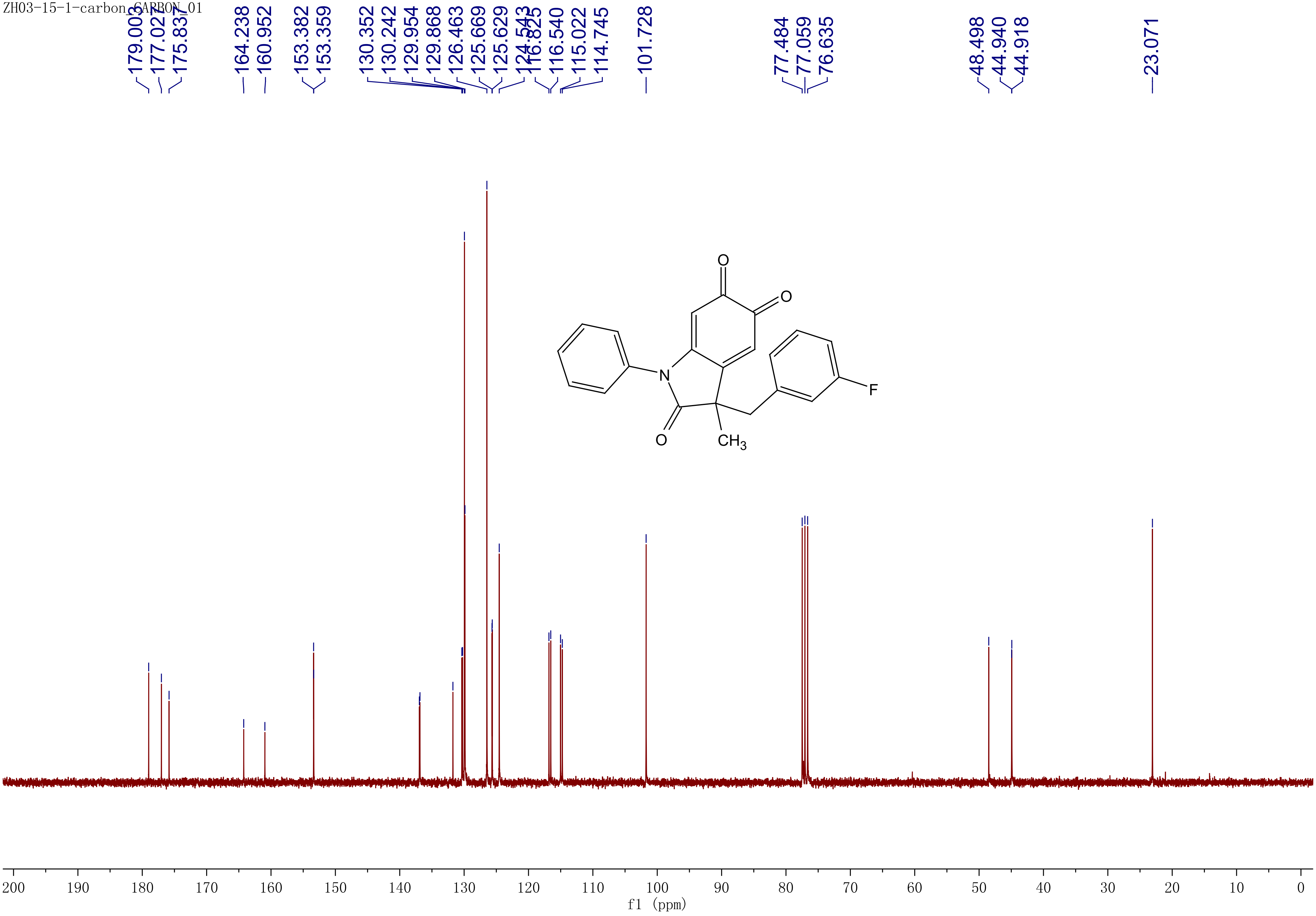
ZH03-15-1-carbon_CARBON_01
179.003
177.027
175.837
164.238
160.952
153.382
153.359
130.352
130.242
129.954
129.868
126.463
125.669
125.629
124.543
116.825
116.540
115.022
114.745
101.728
77.484
77.059
76.635
48.498
44.940
44.918
23.071
O
O
N
F
O
CH3
200 190 180 170 160 150 140 130 120 110 100 90 80 70 60 50 40 30 20 10 0
f1 (ppm)

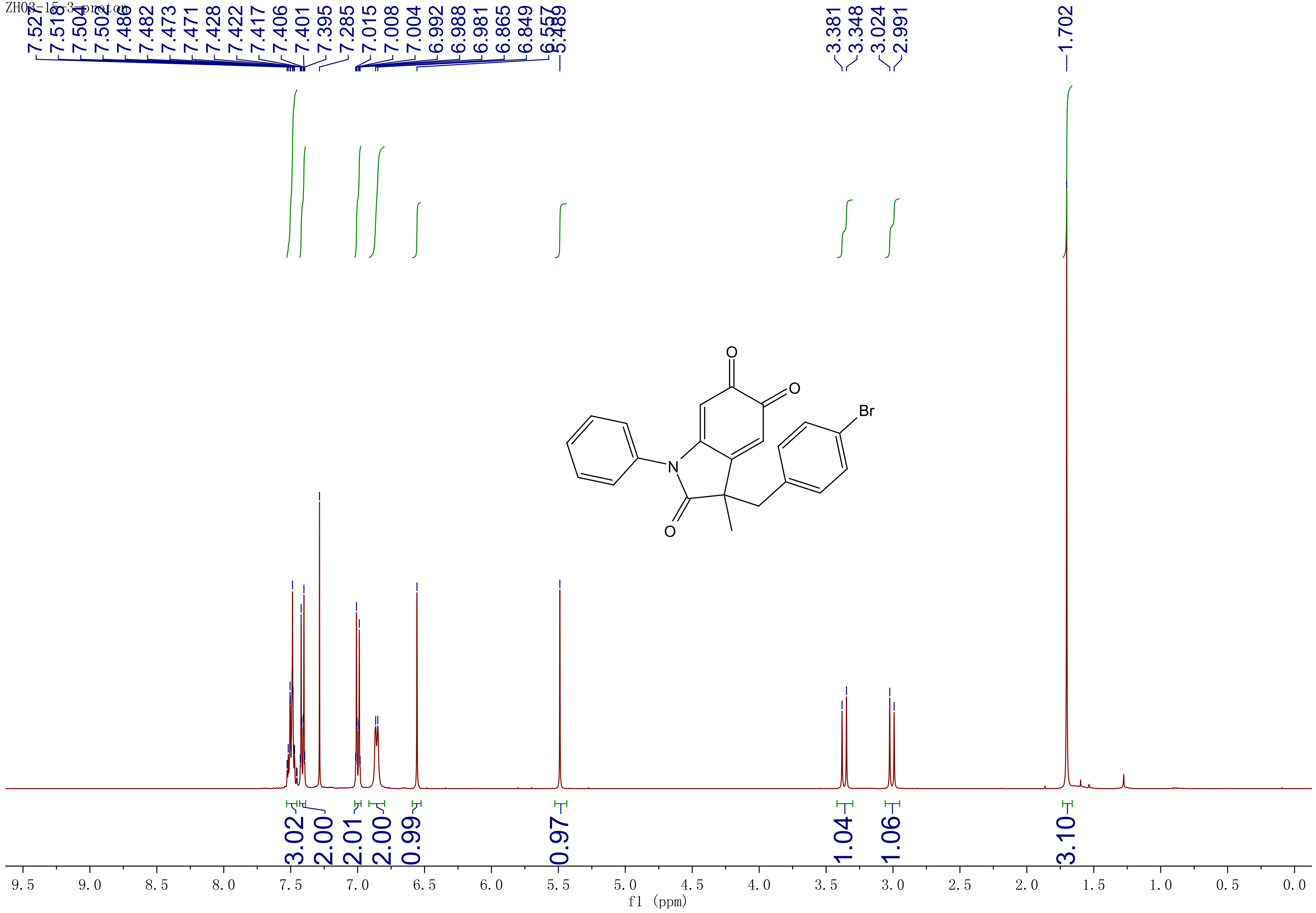
ZH03-15-3-proton
7.527
7.518
7.504
7.502
7.486
7.482
7.473
7.471
7.428
7.422
7.417
7.406
7.401
7.395
7.285
7.015
7.008
7.004
6.992
6.988
6.981
6.865
6.849
6.557
5.489
3.381
3.348
3.024
2.991
1.702
O
O
Br
N
O
3.02
2.00
2.01
2.00
0.99
0.97
1.04
1.06
3.10
9.5 9.0 8.5 8.0 7.5 7.0 6.5 6.0 5.5 5.0 4.5 4.0 3.5 3.0 2.5 2.0 1.5 1.0 0.5 0.0
f1 (ppm)

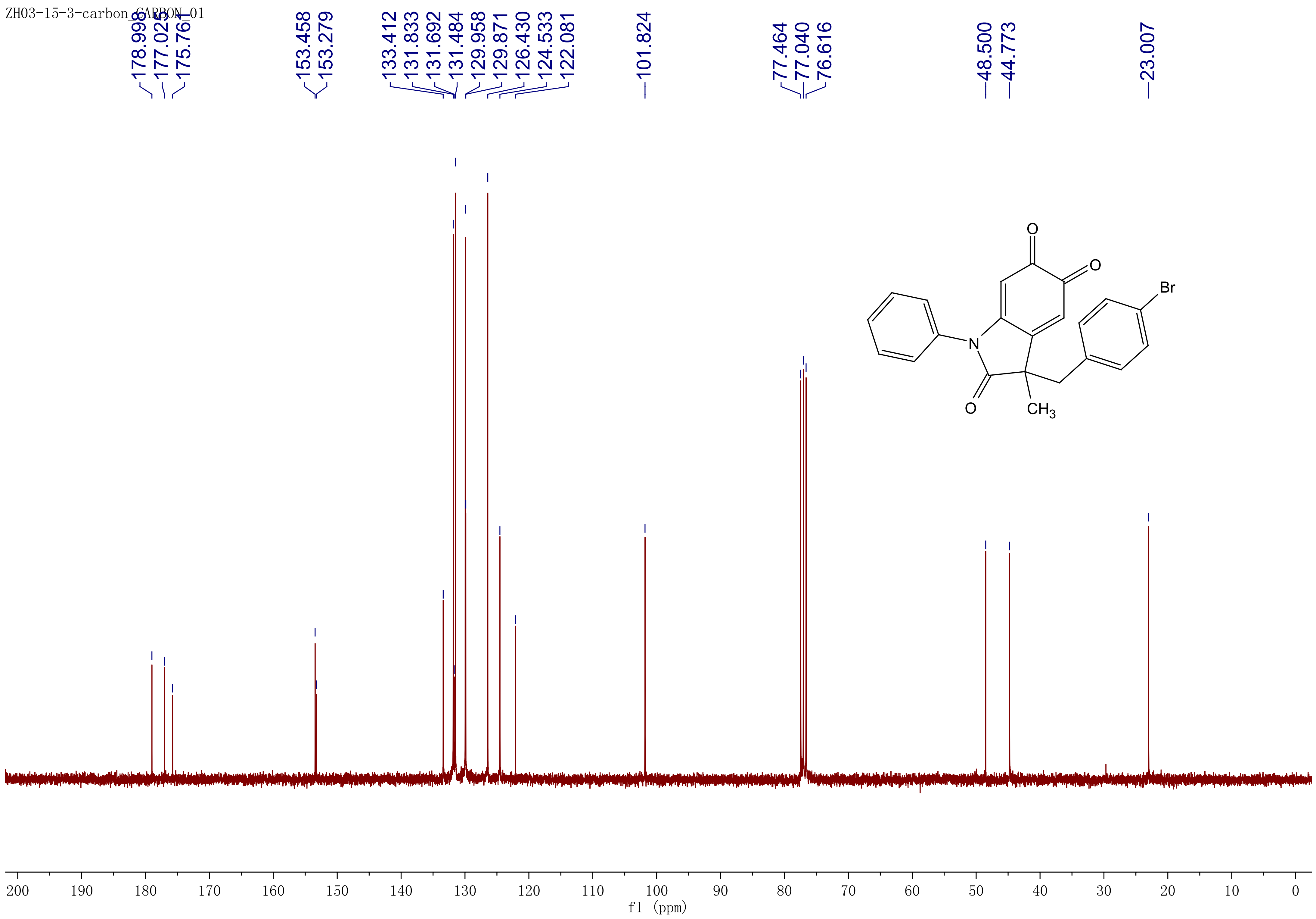

ZH03-15-3-carbon_CARBON_01
178.998
177.025
175.761
153.458
153.279
133.412
131.833
131.692
131.484
129.958
129.871
126.430
124.533
122.081
101.824
77.464
77.040
76.616
48.500
44.773
23.007
O
O
Br
N
O
CH3
200 190 180 170 160 150 140 130 120 110 100 90 80 70 60 50 40 30 20 10 0
f1 (ppm)

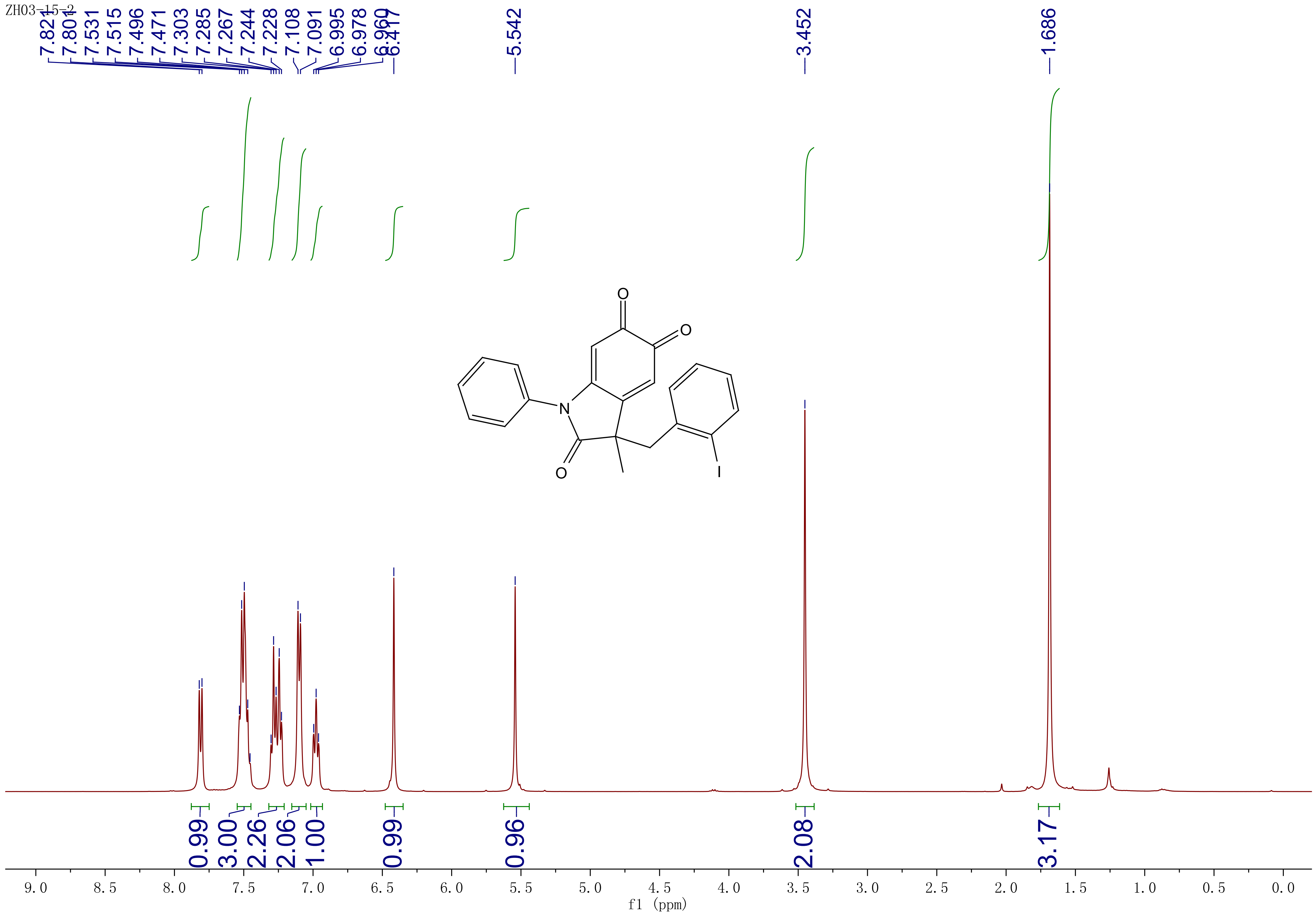

ZH03-15-2
7.821
7.801
7.531
7.515
7.496
7.471
7.303
7.285
7.267
7.244
7.228
7.108
7.091
6.995
6.978
6.960
6.417
5.542
3.452
1.686
0.99
3.00
2.26
2.06
1.00
0.99
0.96
2.08
3.17
9.0 8.5 8.0 7.5 7.0 6.5 6.0 5.5 5.0 4.5 4.0 3.5 3.0 2.5 2.0 1.5 1.0 0.5 0.0
f1 (ppm)
O
O
N
O
I

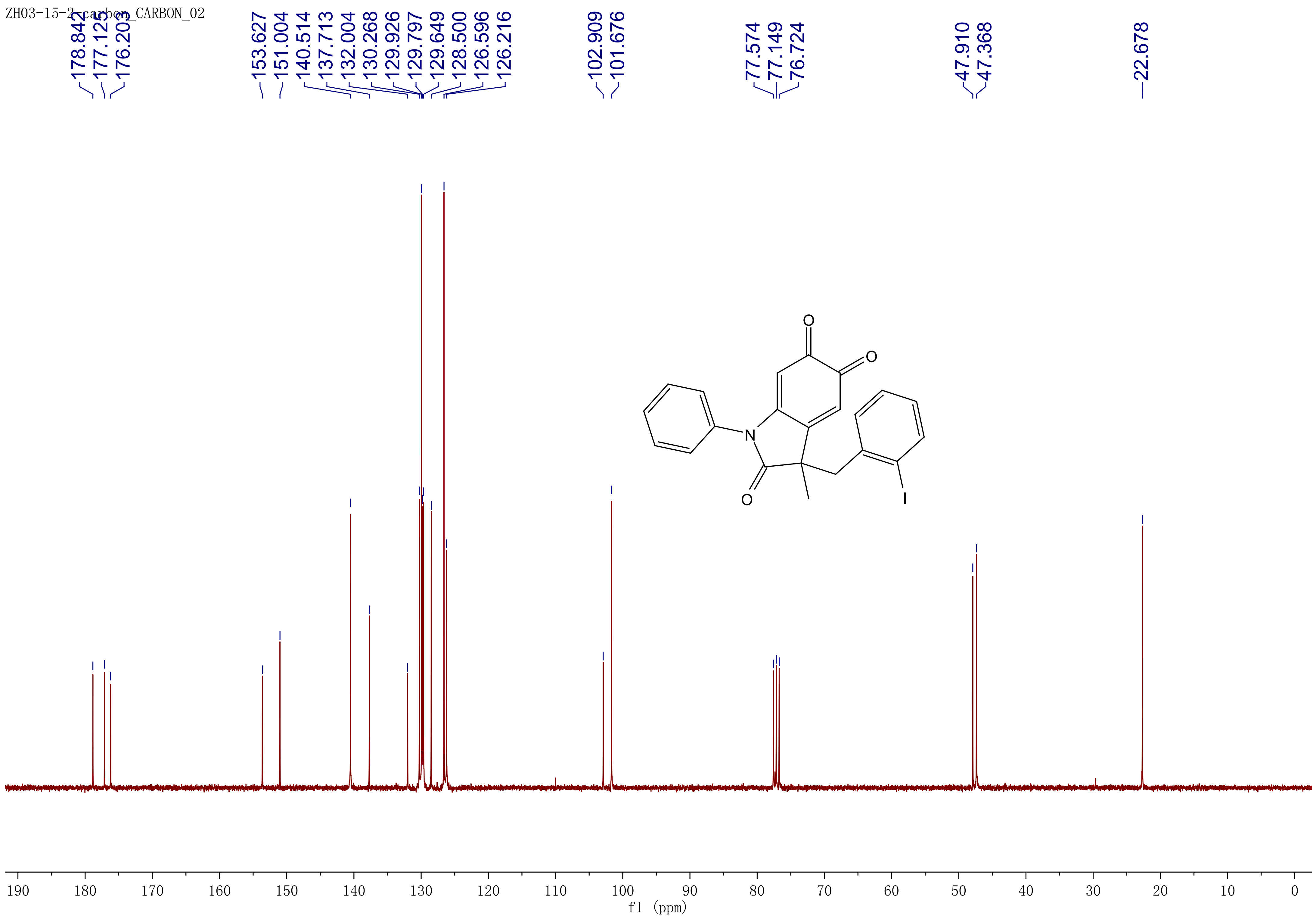
ZH03-15-2-carbon_CARBON_02
178.842
177.125
176.203
153.627
151.004
140.514
137.713
132.004
130.268
129.926
129.797
129.649
128.500
126.596
126.216
102.909
101.676
77.574
77.149
76.724
47.910
47.368
22.678
O
O
N
O
I
190
180
170
160
150
140
130
120
110
100
90
80
70
60
50
40
30
20
10
0
f1 (ppm)

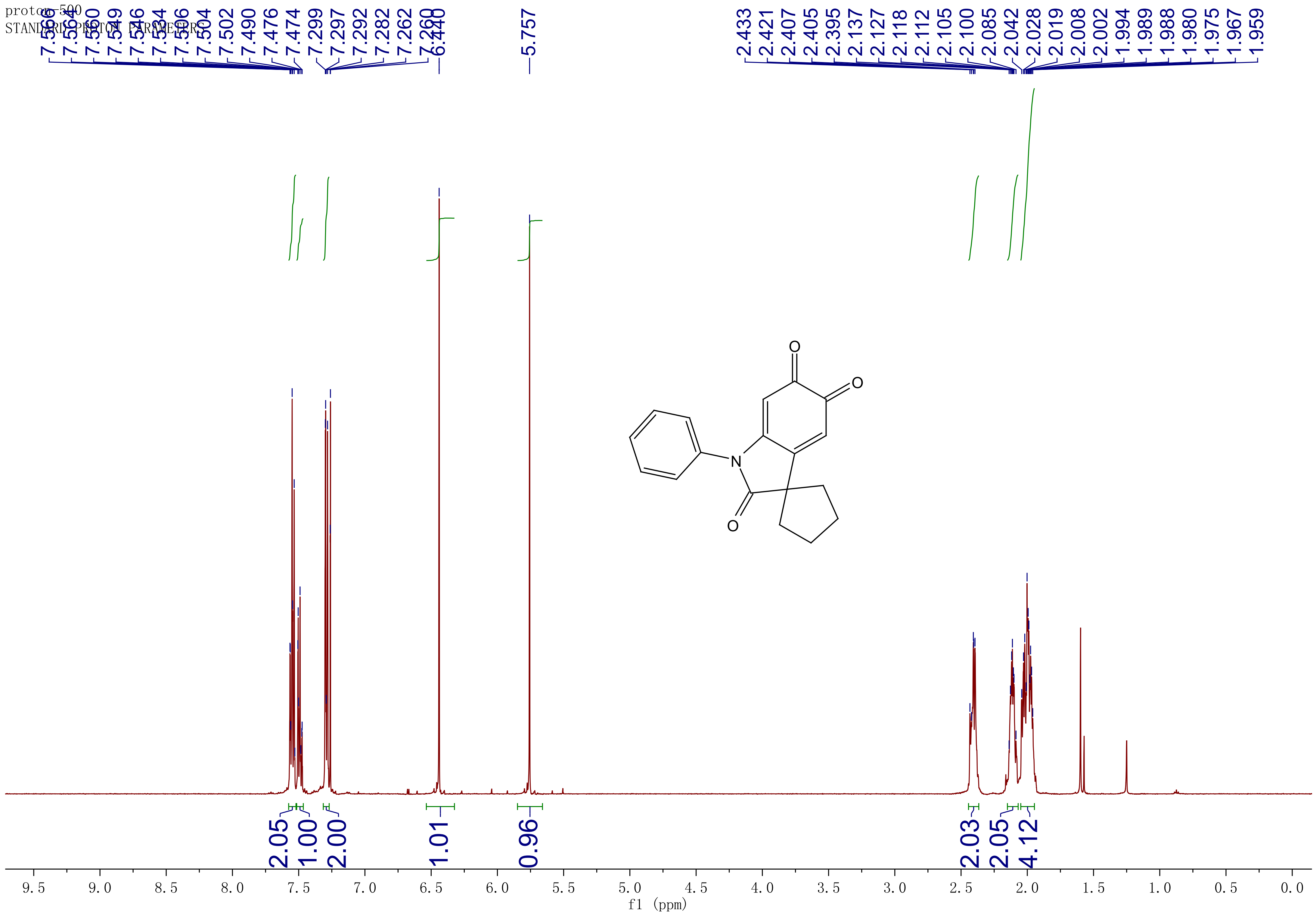

proton-500
STANDARD PROTON PARAMETERS
7.560
7.549
7.546
7.534
7.506
7.504
7.502
7.490
7.476
7.474
7.299
7.297
7.292
7.282
7.262
7.260
6.440
5.757
2.433
2.421
2.407
2.405
2.395
2.137
2.127
2.118
2.112
2.105
2.100
2.085
2.042
2.028
2.019
2.008
2.002
1.994
1.989
1.988
1.980
1.975
1.967
1.959
O
O
N
O
2.05
1.00
2.00
1.01
0.96
2.03
2.05
4.12
9.5
9.0
8.5
8.0
7.5
7.0
6.5
6.0
5.5
5.0
4.5
4.0
3.5
3.0
2.5
2.0
1.5
1.0
0.5
0.0
f1 (ppm)

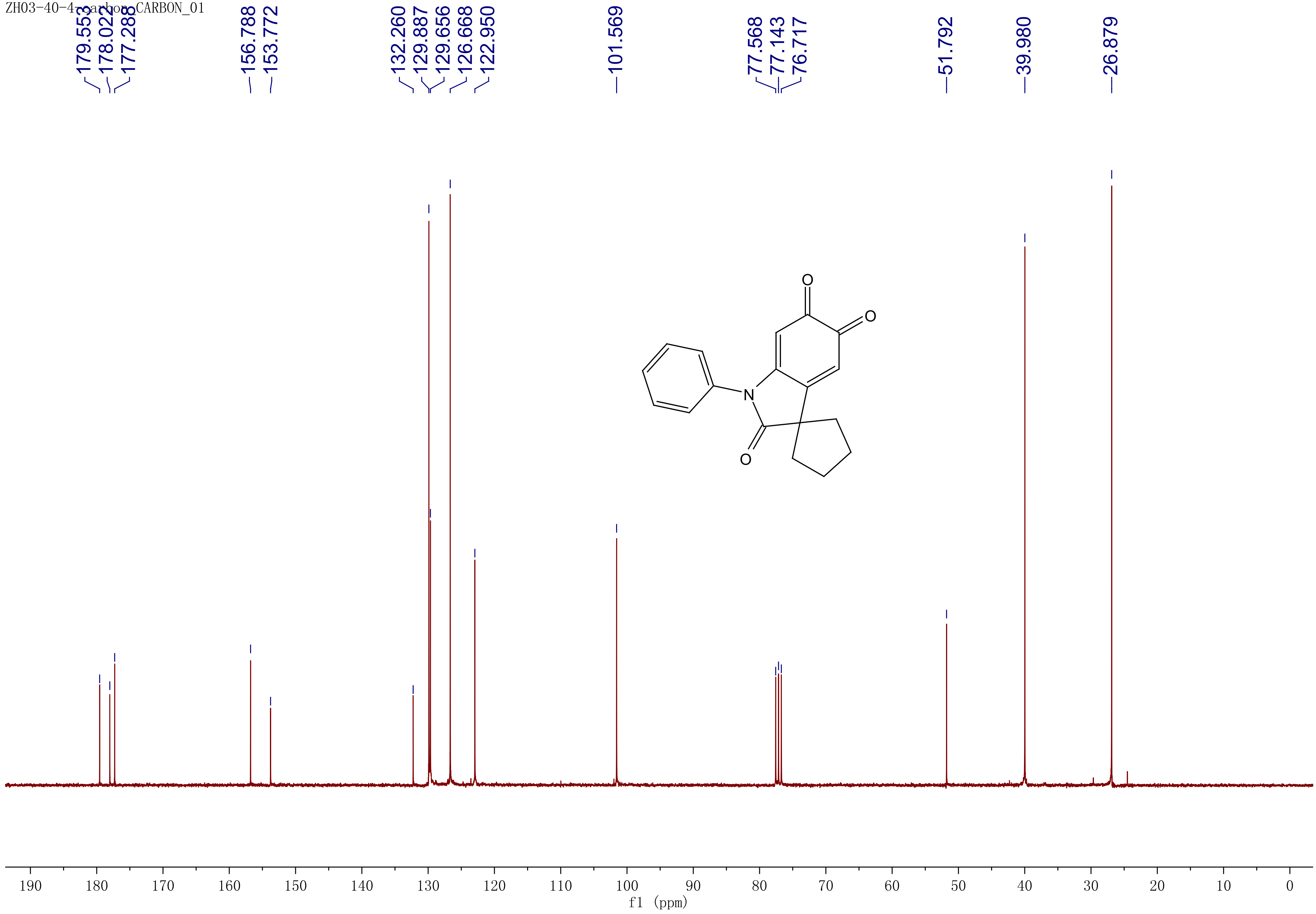
ZH03-40-4-carbon CARBON_01
179.553
178.022
177.288
156.788
153.772
132.260
129.887
129.656
126.668
122.950
101.569
77.568
77.143
76.717
51.792
39.980
26.879
O
O
N
O
190 180 170 160 150 140 130 120 110 100 90 80 70 60 50 40 30 20 10 0
f1 (ppm)

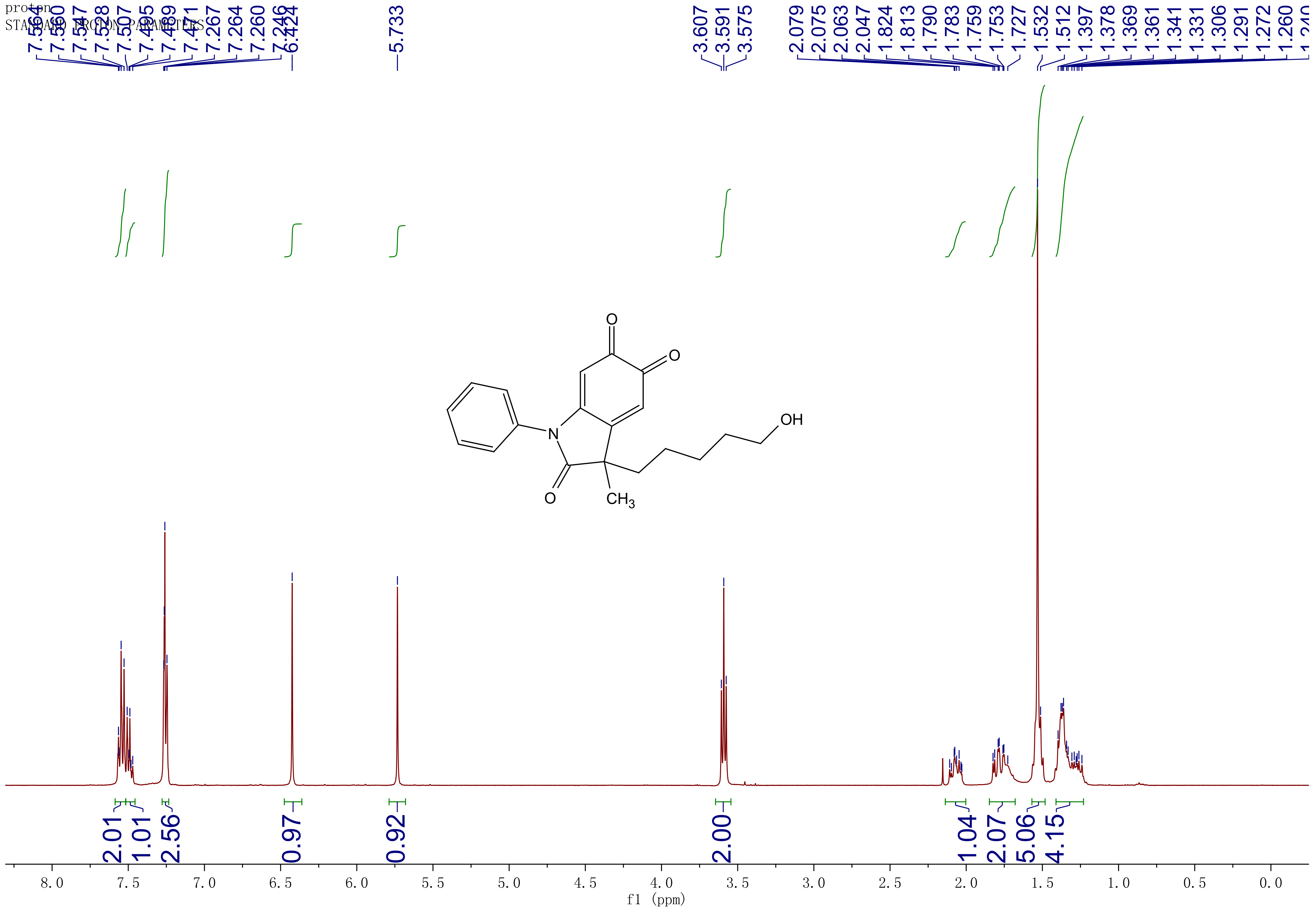
proton
STANDARD PROTON PARAMETERS
7.564 7.560 7.547 7.528 7.507 7.495 7.489 7.471 7.267 7.264 7.260 7.246 6.424
5.733
3.607 3.591 3.575
2.079 2.075 2.063 2.047 1.824 1.813 1.790 1.783 1.759 1.753 1.727 1.532 1.512 1.397 1.378 1.369 1.361 1.341 1.331 1.306 1.291 1.272 1.260
O
O
OH
N
O
CH3
2.01 1.01 2.56 0.97 0.92 2.00 1.04 2.07 5.06 4.15
8.0 7.5 7.0 6.5 6.0 5.5 5.0 4.5 4.0 3.5 3.0 2.5 2.0 1.5 1.0 0.5 0.0
f1 (ppm)

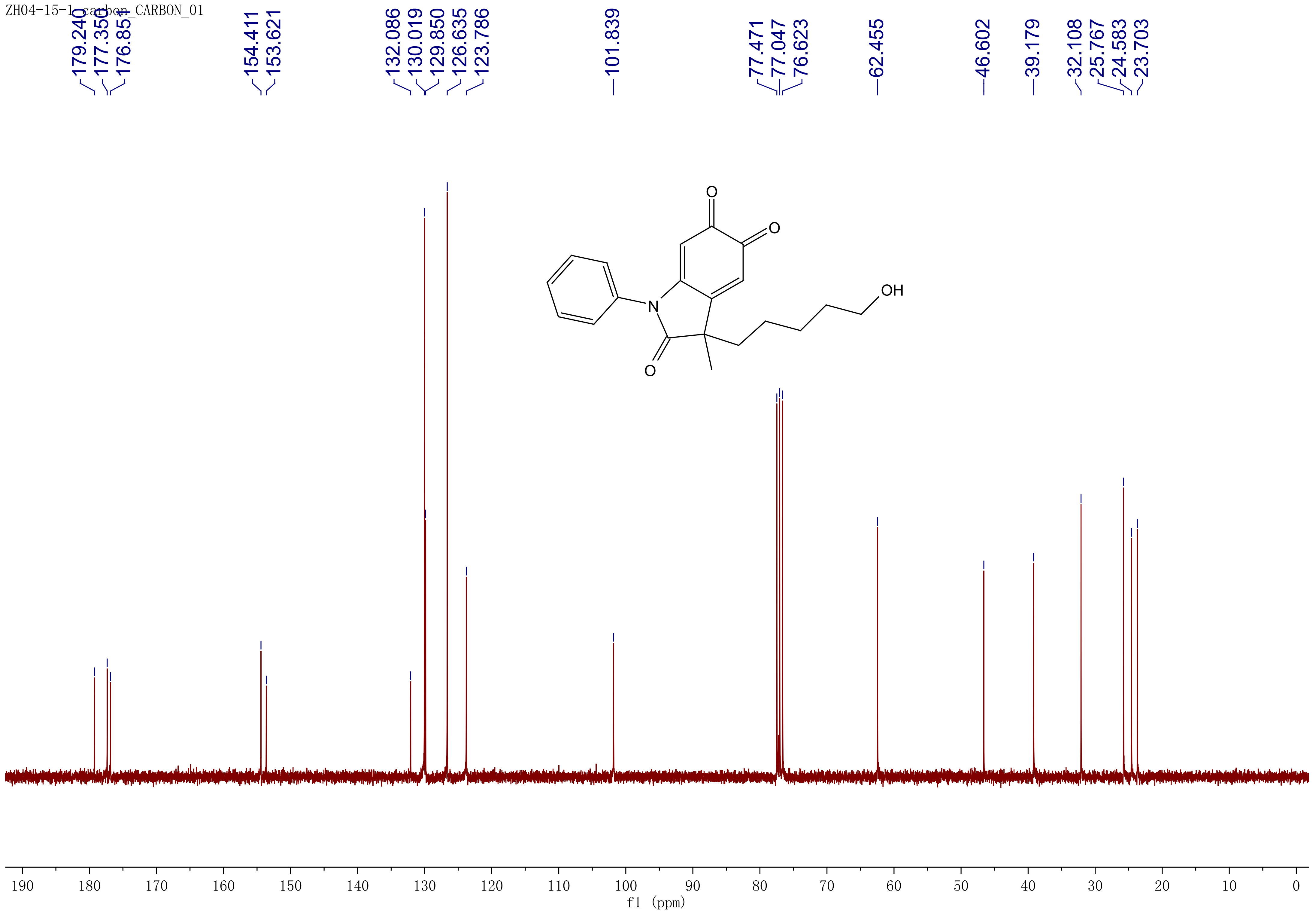

ZH04-15-1_carbon_CARBON_01
179.240
177.350
176.851
154.411
153.621
132.086
130.019
129.850
126.635
123.786
101.839
77.471
77.047
76.623
62.455
46.602
39.179
32.108
25.767
24.583
23.703
O
O
OH
N
O
190
180
170
160
150
140
130
120
110
100
90
80
70
60
50
40
30
20
10
0
f1 (ppm)

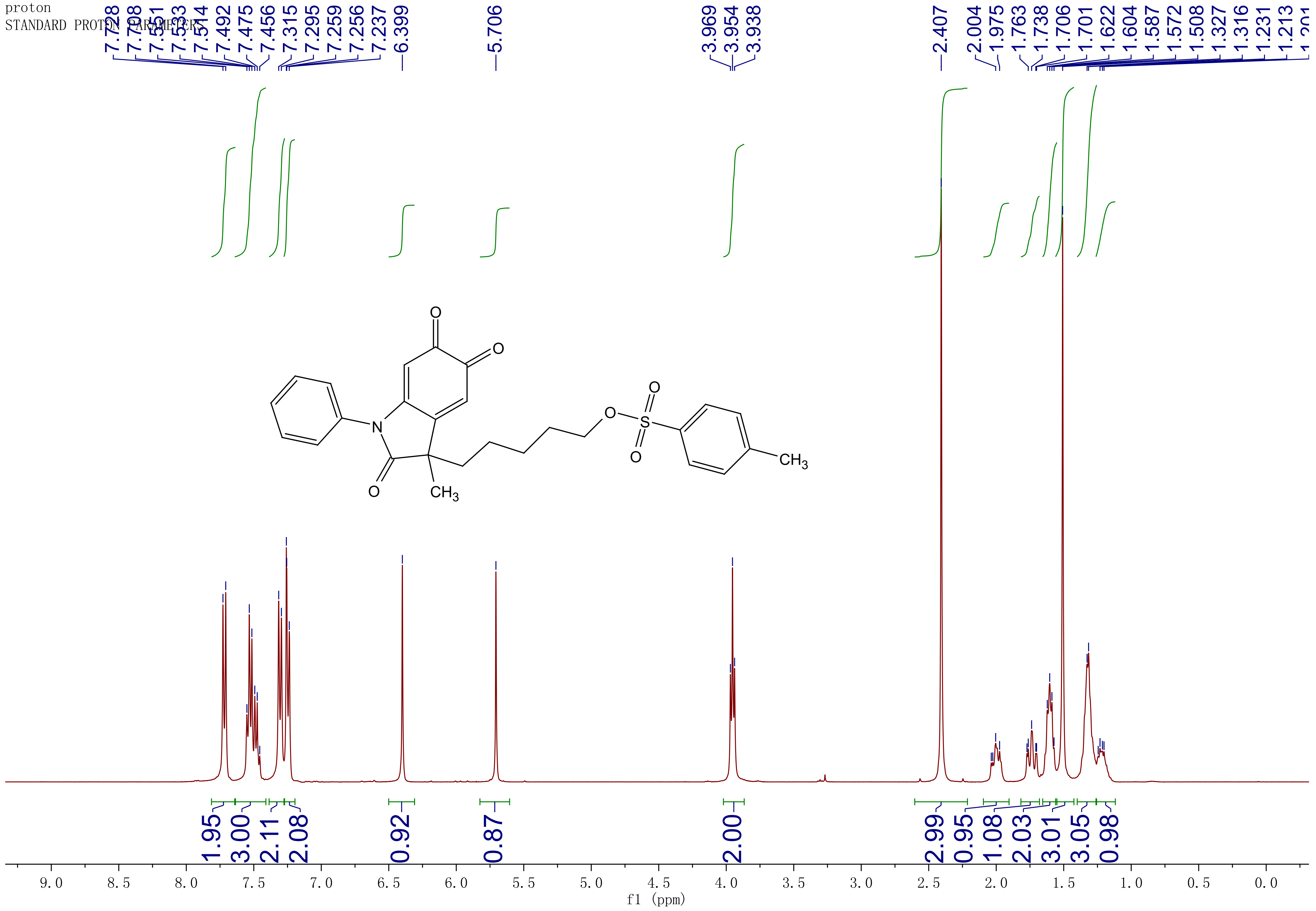
proton
STANDARD PROTON PARAMETERS
7.728
7.708
7.551
7.533
7.514
7.492
7.475
7.456
7.315
7.295
7.259
7.256
7.237
6.399
5.706
3.969
3.954
3.938
2.407
2.004
1.975
1.763
1.738
1.706
1.701
1.622
1.604
1.587
1.572
1.508
1.327
1.316
1.231
1.213
O
O
O
O
N
S
O
O
CH3
CH3
1.95
3.00
2.11
2.08
0.92
0.87
2.00
2.99
0.95
1.08
2.03
3.01
3.05
0.98
9.0
8.5
8.0
7.5
7.0
6.5
6.0
5.5
5.0
4.5
4.0
3.5
3.0
2.5
2.0
1.5
1.0
0.5
0.0
f1 (ppm)

ZH04-23-2-carbon_CARBON_01

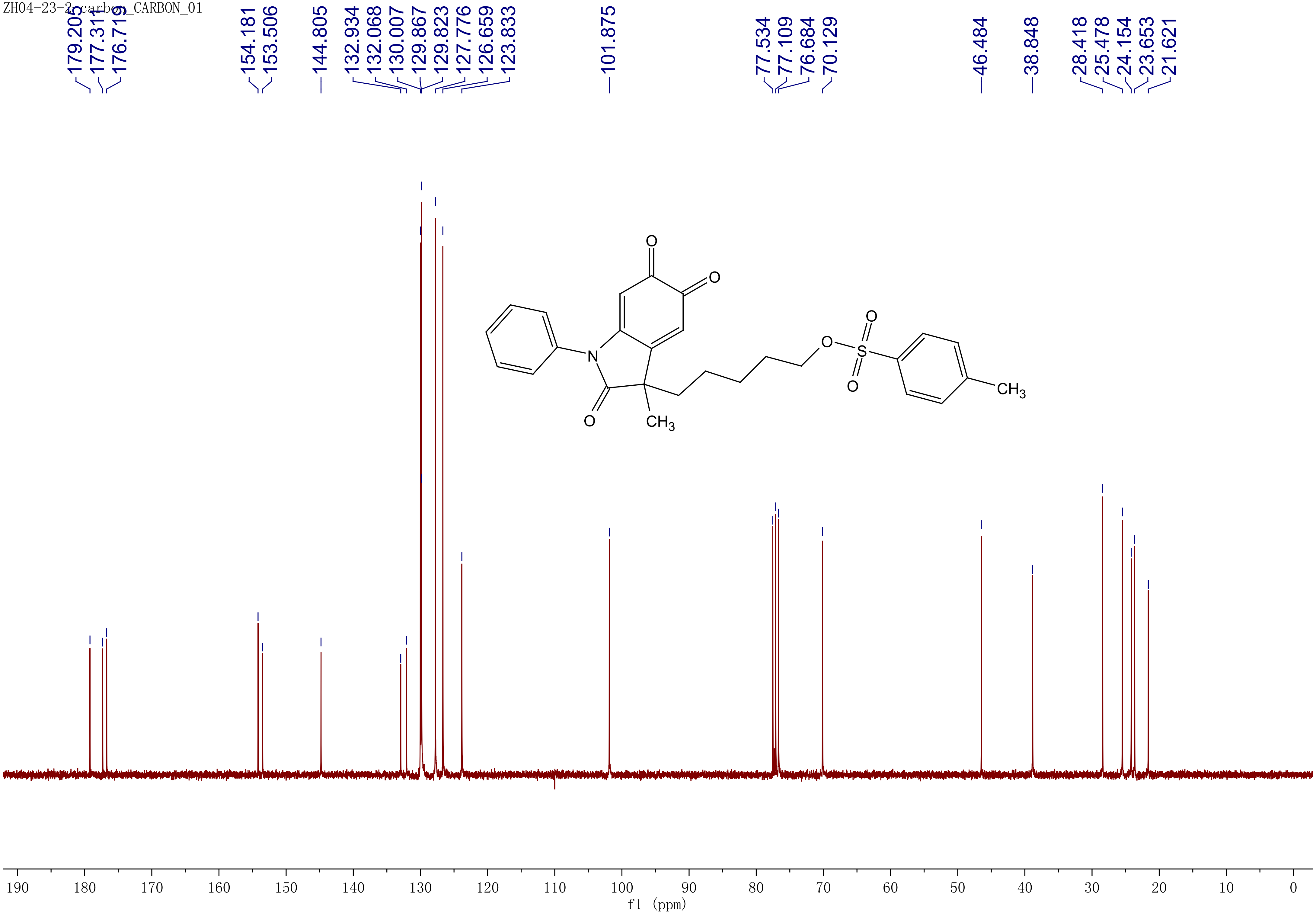

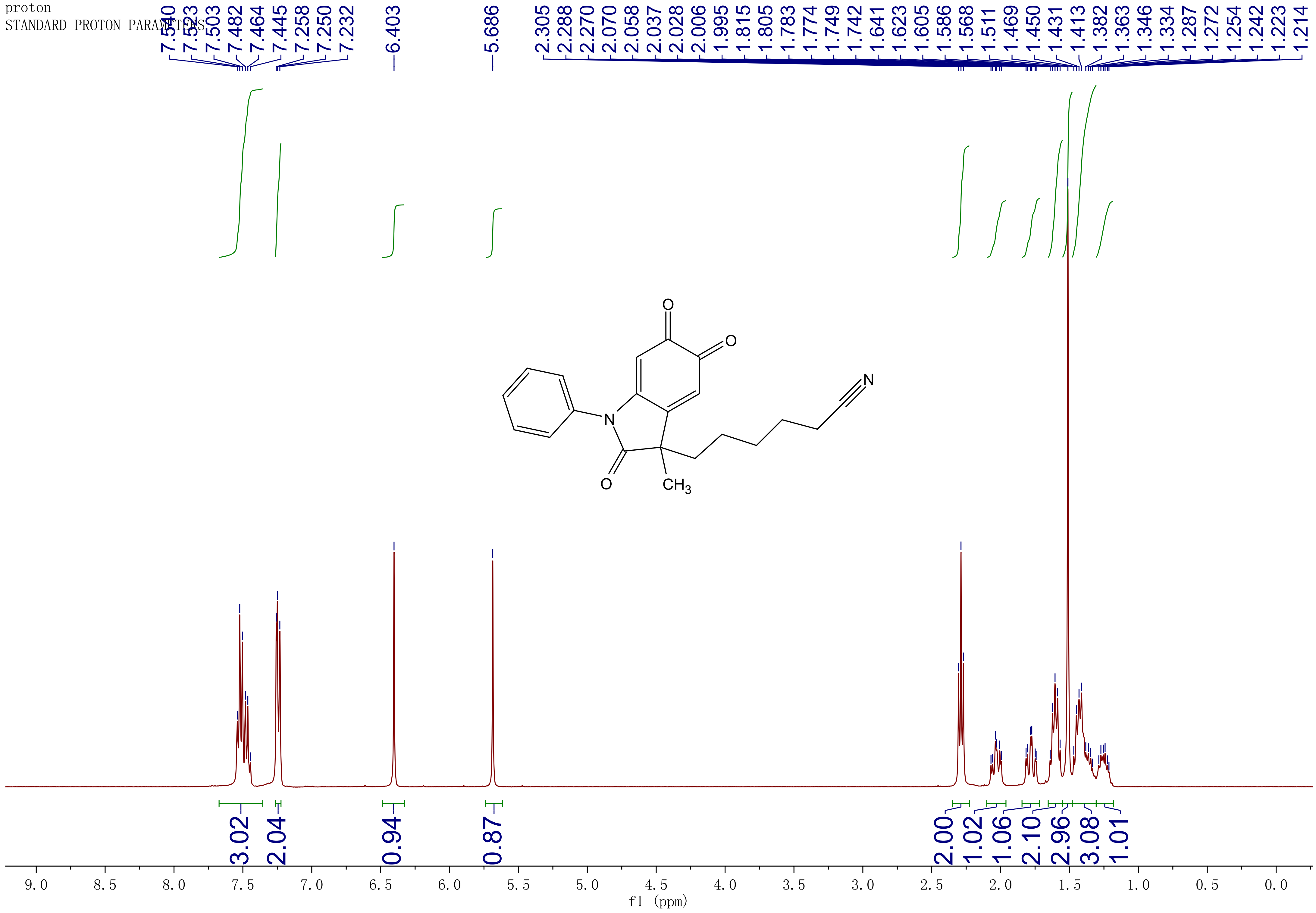
proton
STANDARD PROTON PARAMETERS
7.540
7.523
7.503
7.482
7.464
7.445
7.258
7.250
7.232
6.403
5.686
2.305
2.288
2.270
2.070
2.058
2.037
2.028
2.006
1.995
1.815
1.805
1.783
1.774
1.749
1.742
1.641
1.623
1.605
1.586
1.568
1.511
1.469
1.450
1.431
1.413
1.382
1.363
1.346
1.334
1.287
1.272
1.254
1.242
1.223
1.214
O
O
N
N
O
CH3
3.02
2.04
0.94
0.87
2.00
1.02
1.06
2.10
2.96
3.08
1.01
9.0
8.5
8.0
7.5
7.0
6.5
6.0
5.5
5.0
4.5
4.0
3.5
3.0
2.5
2.0
1.5
1.0
0.5
0.0
f1 (ppm)

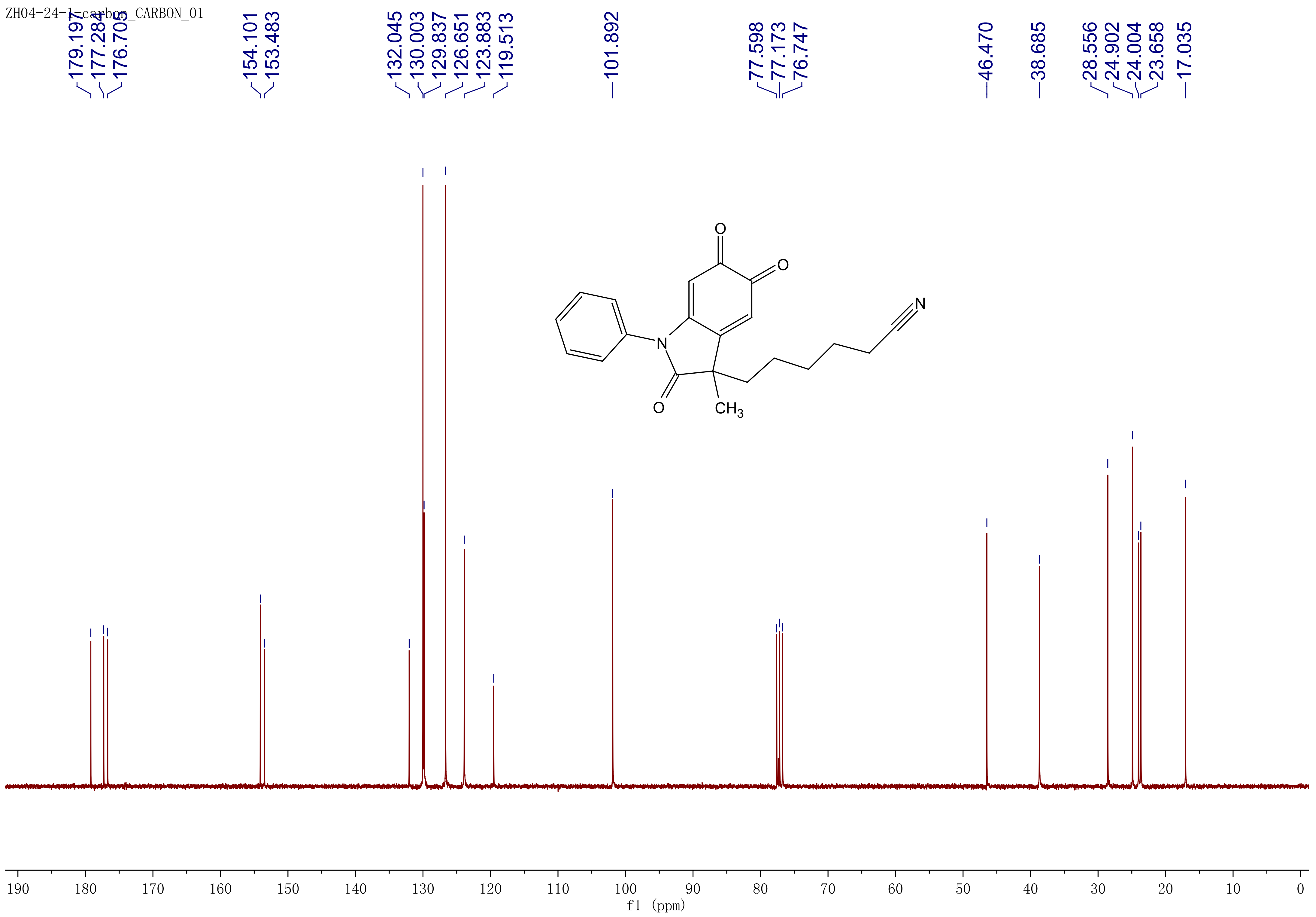
ZH04-24-1-carbon_CARBON_01
179.197
177.284
176.705
154.101
153.483
132.045
130.003
129.837
126.651
123.883
119.513
101.892
77.598
77.173
76.747
46.470
38.685
28.556
24.902
24.004
23.658
17.035
O
O
N
N
O
$CH_3$
190 180 170 160 150 140 130 120 110 100 90 80 70 60 50 40 30 20 10 0
f1 (ppm)

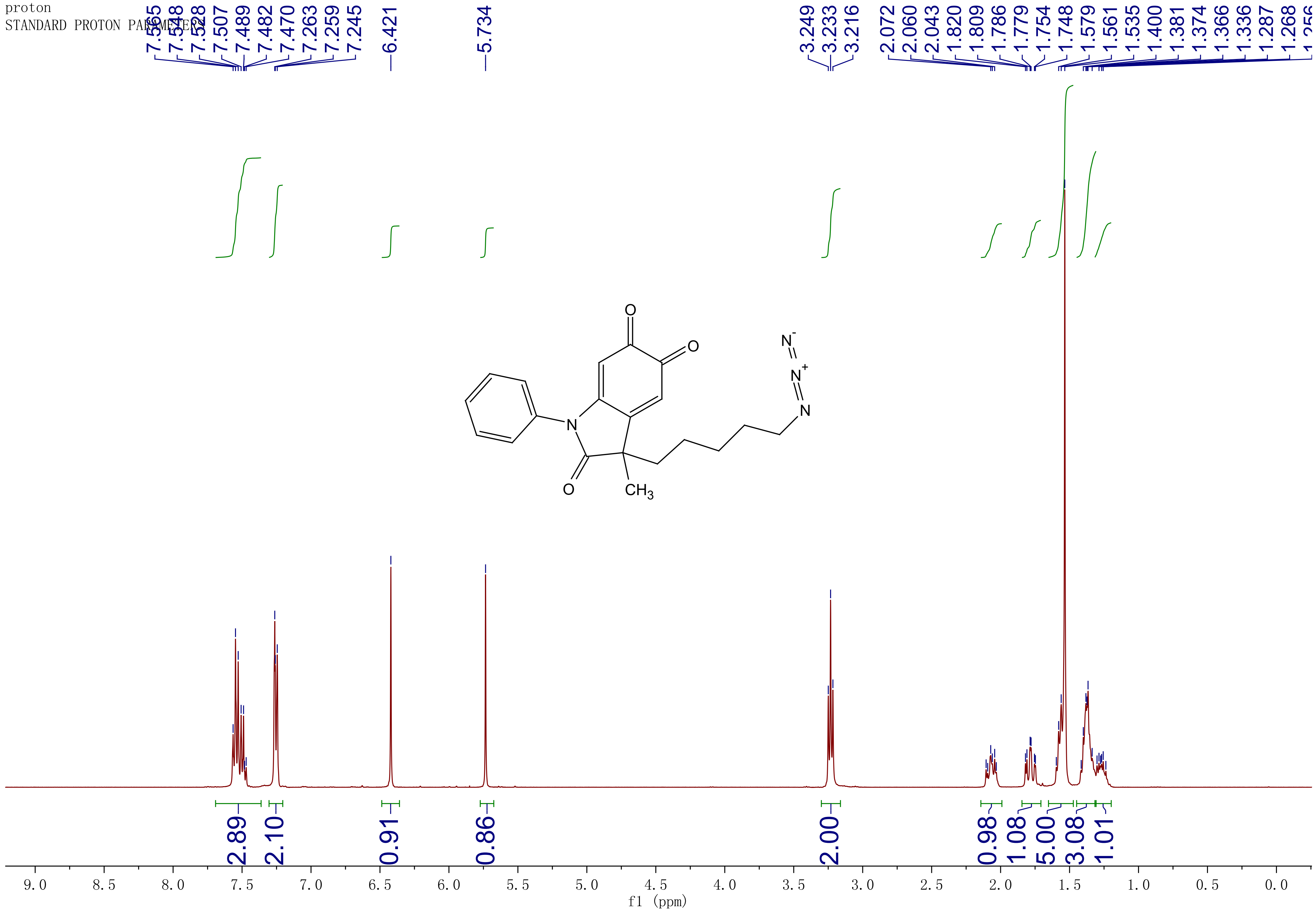

proton
STANDARD PROTON PARAMETERS
7.555
7.548
7.528
7.507
7.489
7.482
7.470
7.263
7.259
7.245
6.421
5.734
3.249
3.233
3.216
2.072
2.060
2.043
1.820
1.809
1.786
1.779
1.754
1.748
1.579
1.561
1.535
1.400
1.381
1.374
1.366
1.336
1.287
1.268
O
O
N
O
CH3
N
N+
N-
2.89
2.10
0.91
0.86
2.00
0.98
1.08
5.00
3.08
1.01
9.0
8.5
8.0
7.5
7.0
6.5
6.0
5.5
5.0
4.5
4.0
3.5
3.0
2.5
2.0
1.5
1.0
0.5
0.0
f1 (ppm)

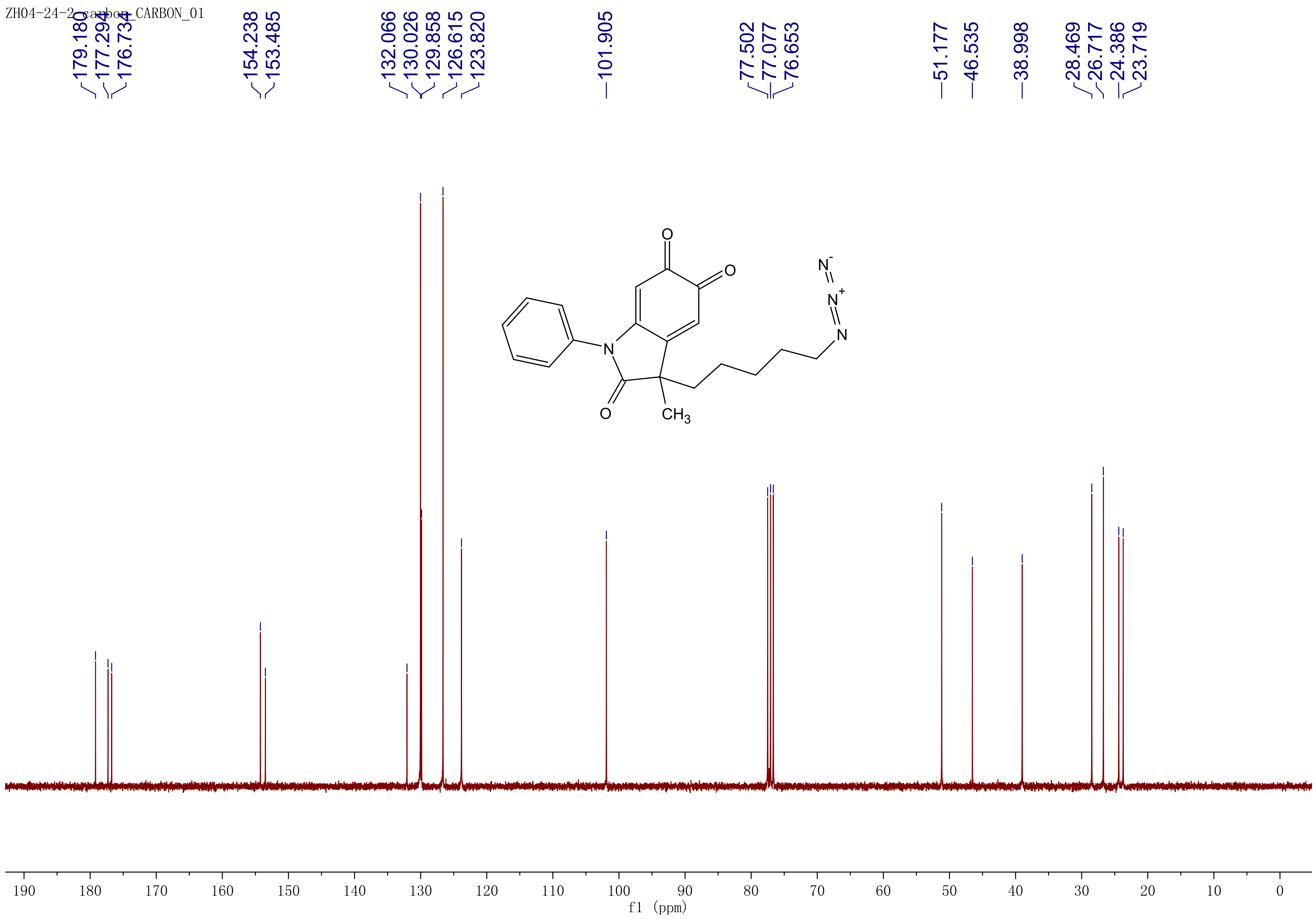
ZH04-24-2_carbon_CARBON_01
179.180
177.294
176.734
154.238
153.485
132.066
130.026
129.858
126.615
123.820
101.905
77.502
77.077
76.653
51.177
46.535
38.998
28.469
26.717
24.386
23.719
f1 (ppm)

ZH06-030

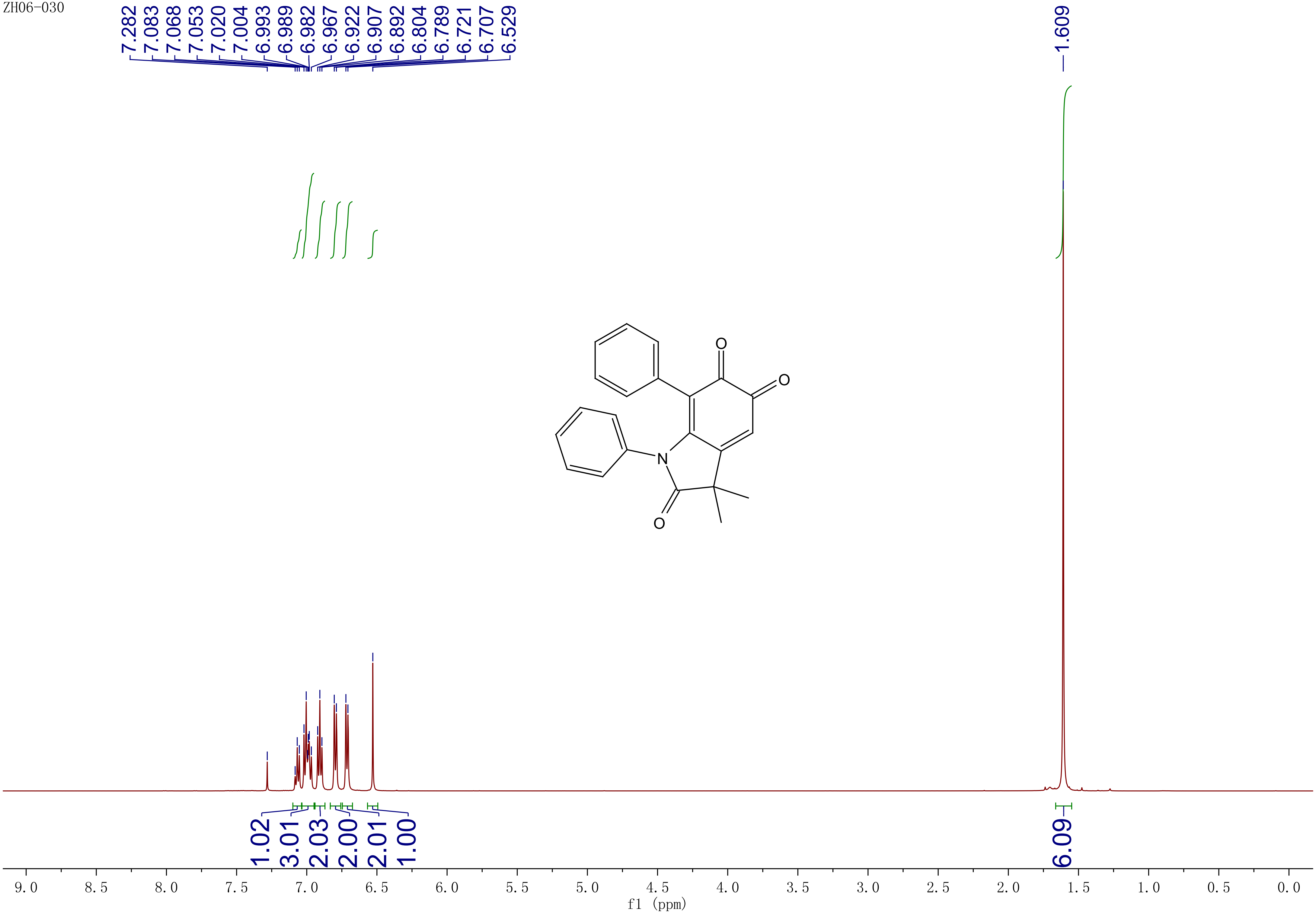

7.282
7.083
7.068
7.053
7.020
7.004
6.993
6.989
6.982
6.967
6.922
6.907
6.892
6.804
6.789
6.721
6.707
6.529
1.609
1.02
3.01
2.03
2.00
2.01
1.00
6.09
9.0
8.5
8.0
7.5
7.0
6.5
6.0
5.5
5.0
4.5
4.0
3.5
3.0
2.5
2.0
1.5
1.0
0.5
0.0
f1 (ppm)
O
O
N
O

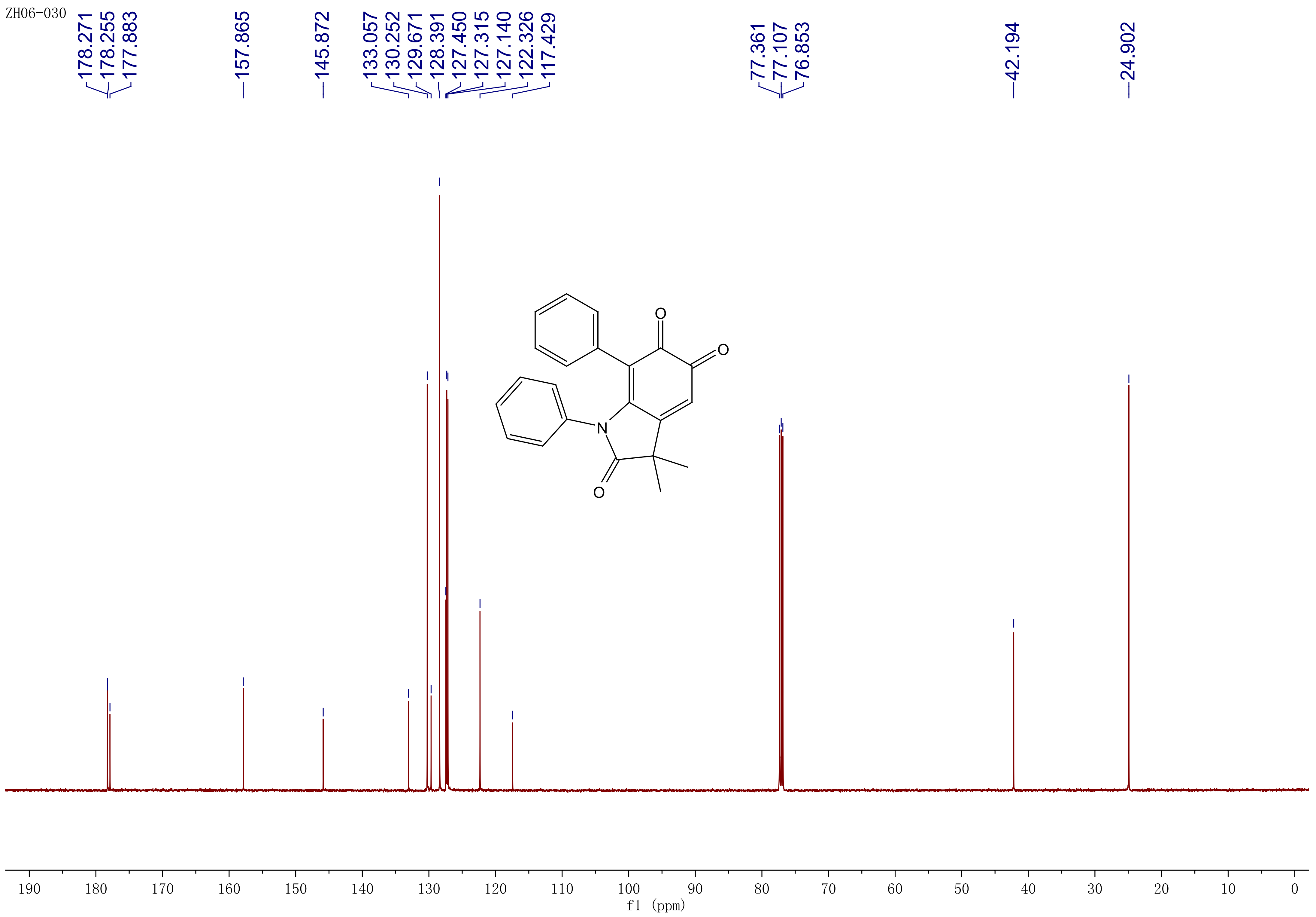
ZH06-030
178.271
178.255
177.883
157.865
145.872
133.057
130.252
129.671
128.391
127.450
127.315
127.140
122.326
117.429
77.361
77.107
76.853
42.194
24.902
O
O
N
O
190 180 170 160 150 140 130 120 110 100 90 80 70 60 50 40 30 20 10 0
f1 (ppm)

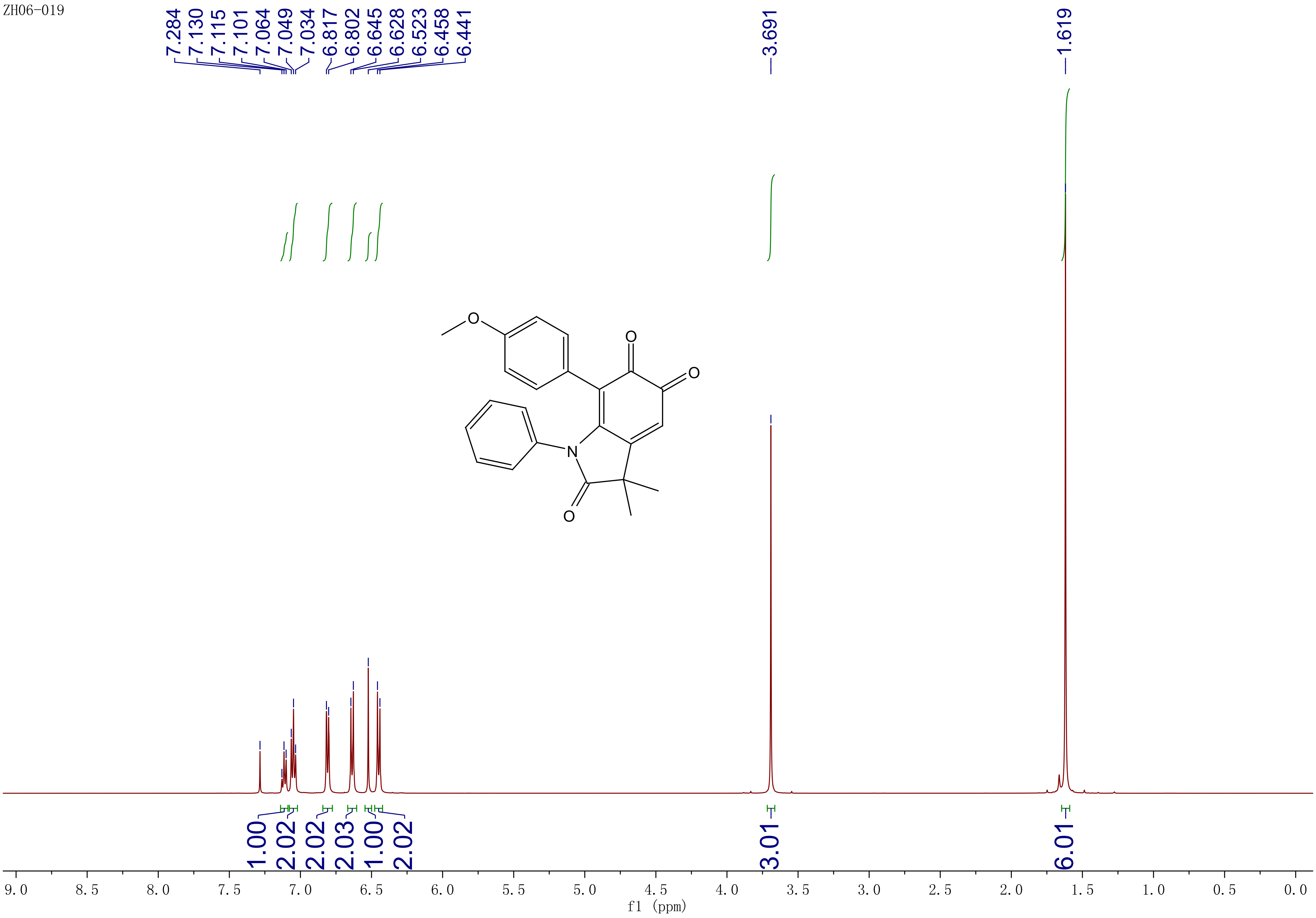
ZH06-019
7.284
7.130
7.115
7.101
7.064
7.049
7.034
6.817
6.802
6.645
6.628
6.523
6.458
6.441
3.691
1.619
O
N
1.00
2.02
2.02
2.03
1.00
2.02
3.01
6.01
9.0 8.5 8.0 7.5 7.0 6.5 6.0 5.5 5.0 4.5 4.0 3.5 3.0 2.5 2.0 1.5 1.0 0.5 0.0
f1 (ppm)

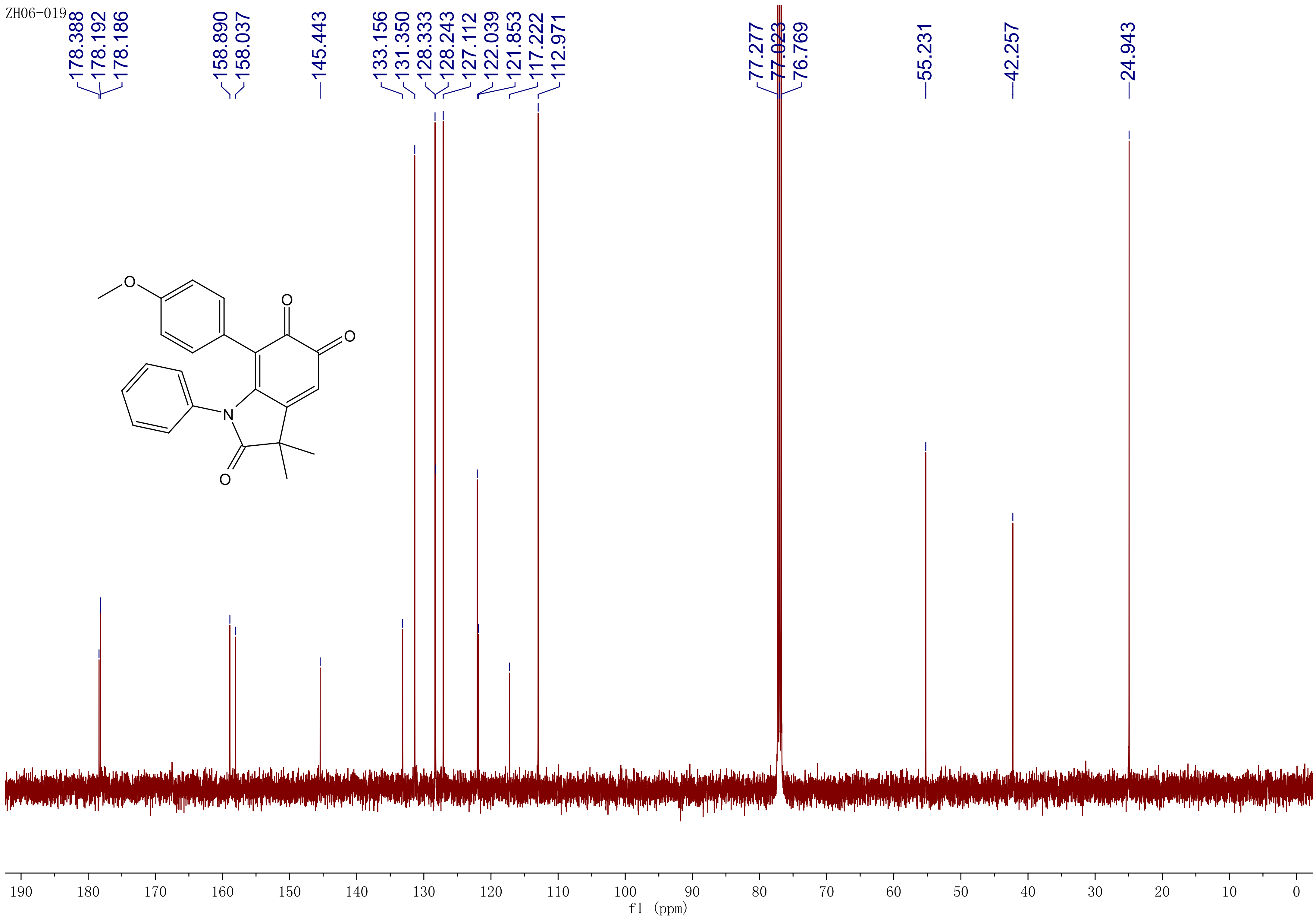
ZH06-019
178.388
178.192
178.186
158.890
158.037
145.443
133.156
131.350
128.333
128.243
127.112
122.039
121.853
117.222
112.971
77.277
77.023
76.769
55.231
42.257
24.943
O
O
O
N
O
190 180 170 160 150 140 130 120 110 100 90 80 70 60 50 40 30 20 10 0
f1 (ppm)

ZH06-047

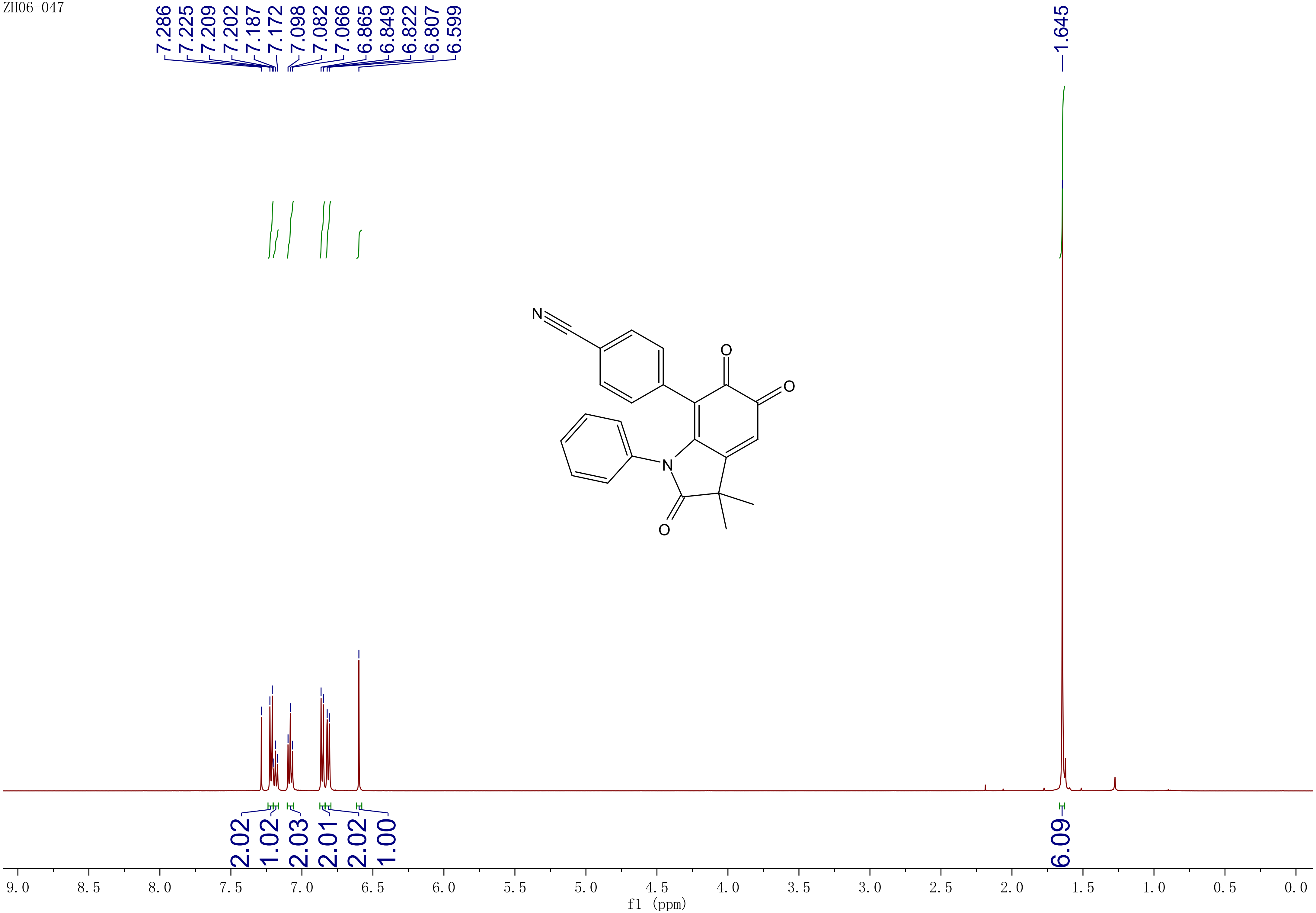

7.286
7.225
7.209
7.202
7.187
7.172
7.098
7.082
7.066
6.865
6.849
6.822
6.807
6.599
1.645
N
O
O
N
O
2.02
1.02
2.03
2.01
2.02
1.00
6.09
9.0
8.5
8.0
7.5
7.0
6.5
6.0
5.5
5.0
4.5
4.0
3.5
3.0
2.5
2.0
1.5
1.0
0.5
0.0
f1 (ppm)

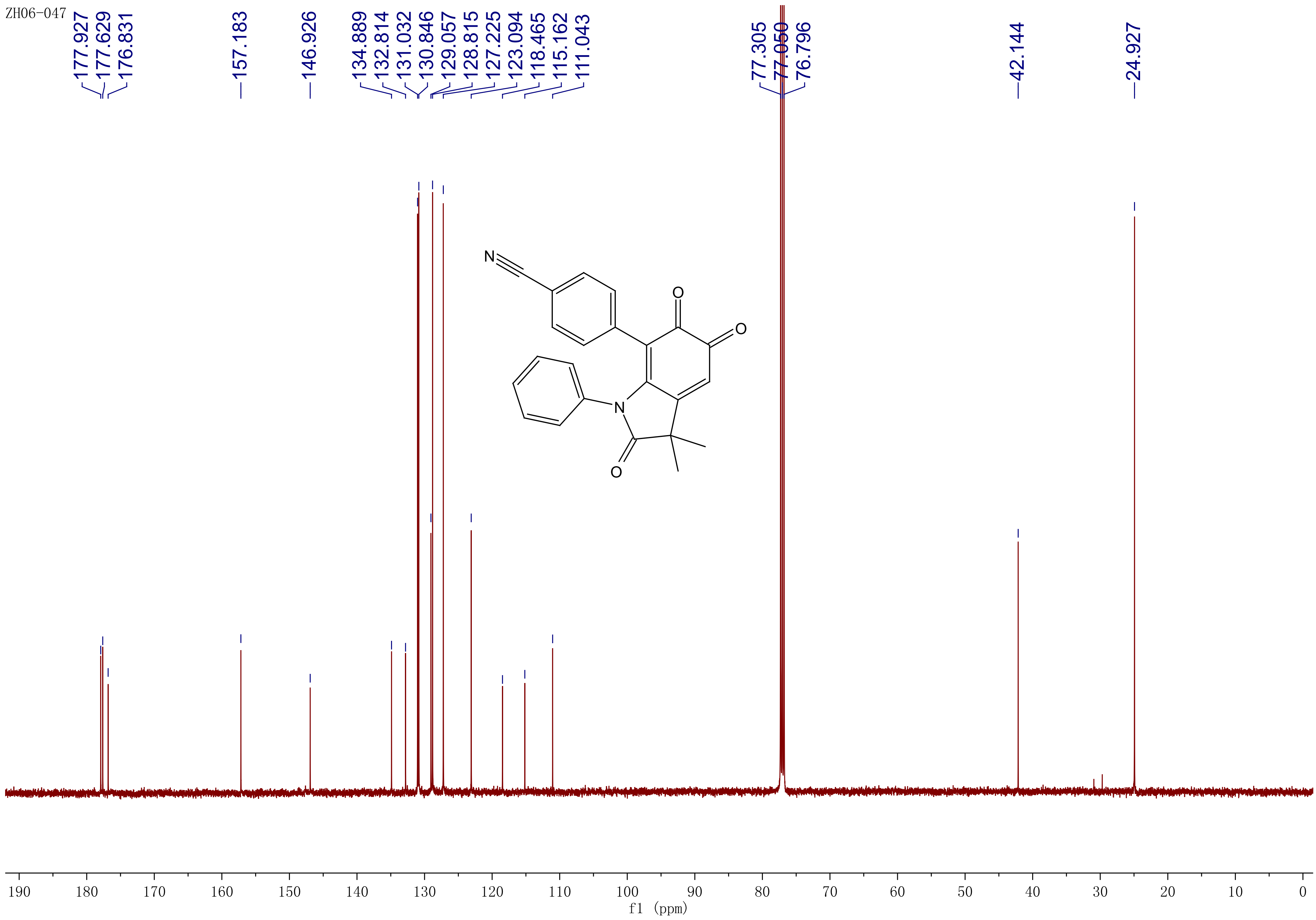

ZH06-047
177.927
177.629
176.831
157.183
146.926
134.889
132.814
131.032
130.846
129.057
128.815
127.225
123.094
118.465
115.162
111.043
77.305
77.050
76.796
42.144
24.927
N
O
O
N
O
190 180 170 160 150 140 130 120 110 100 90 80 70 60 50 40 30 20 10 0
f1 (ppm)

ZH06-032

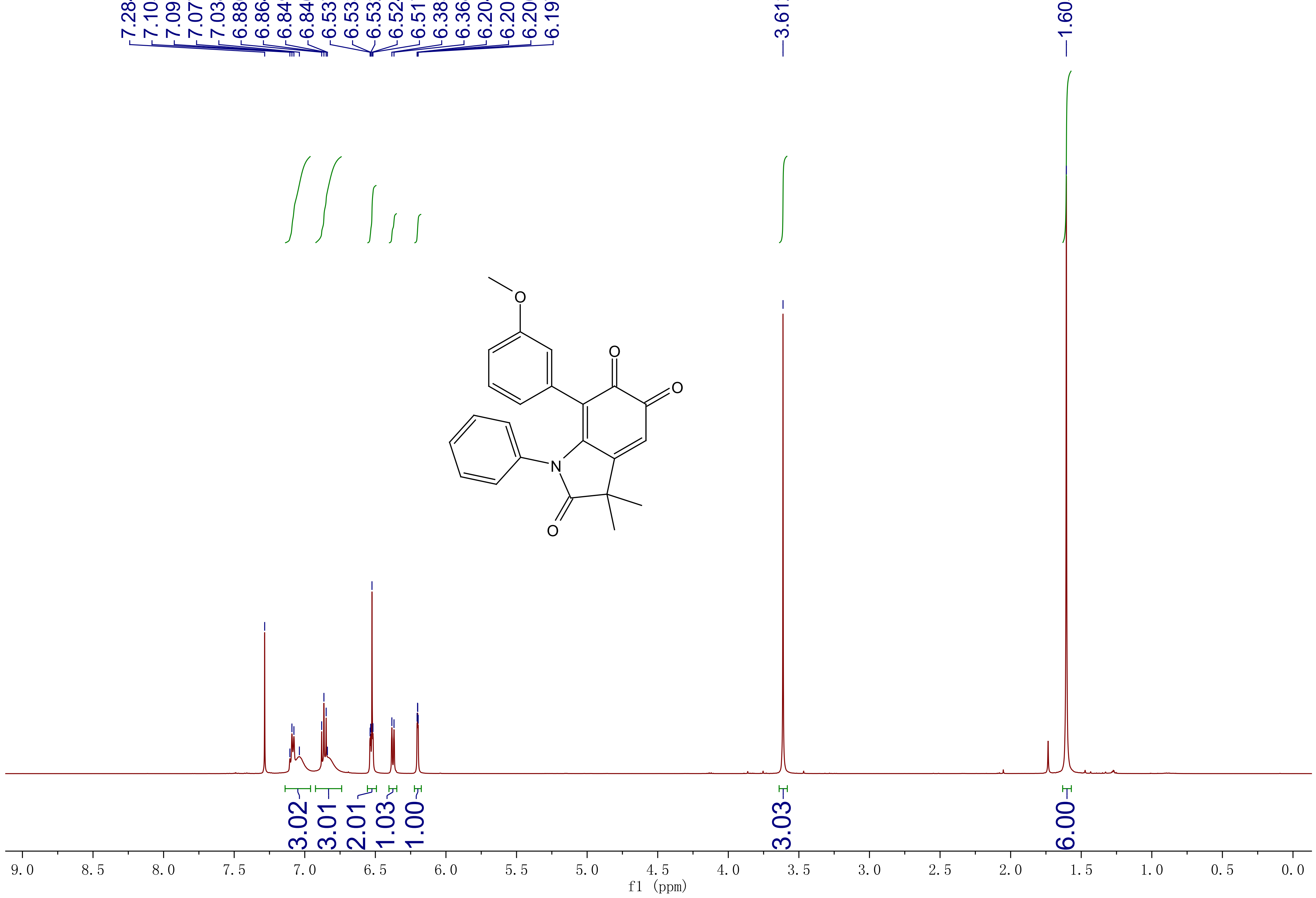
7.284
7.105
7.091
7.077
7.038
6.880
6.864
6.849
6.840
6.537
6.533
6.532
6.524
6.517
6.383
6.368
6.204
6.201
6.200
6.197
3.612
1.605
3.02
3.01
2.01
1.03
1.00
3.03
6.00
O
O
O
N
O
9.0
8.5
8.0
7.5
7.0
6.5
6.0
5.5
5.0
4.5
4.0
3.5
3.0
2.5
2.0
1.5
1.0
0.5
0.0
f1 (ppm)

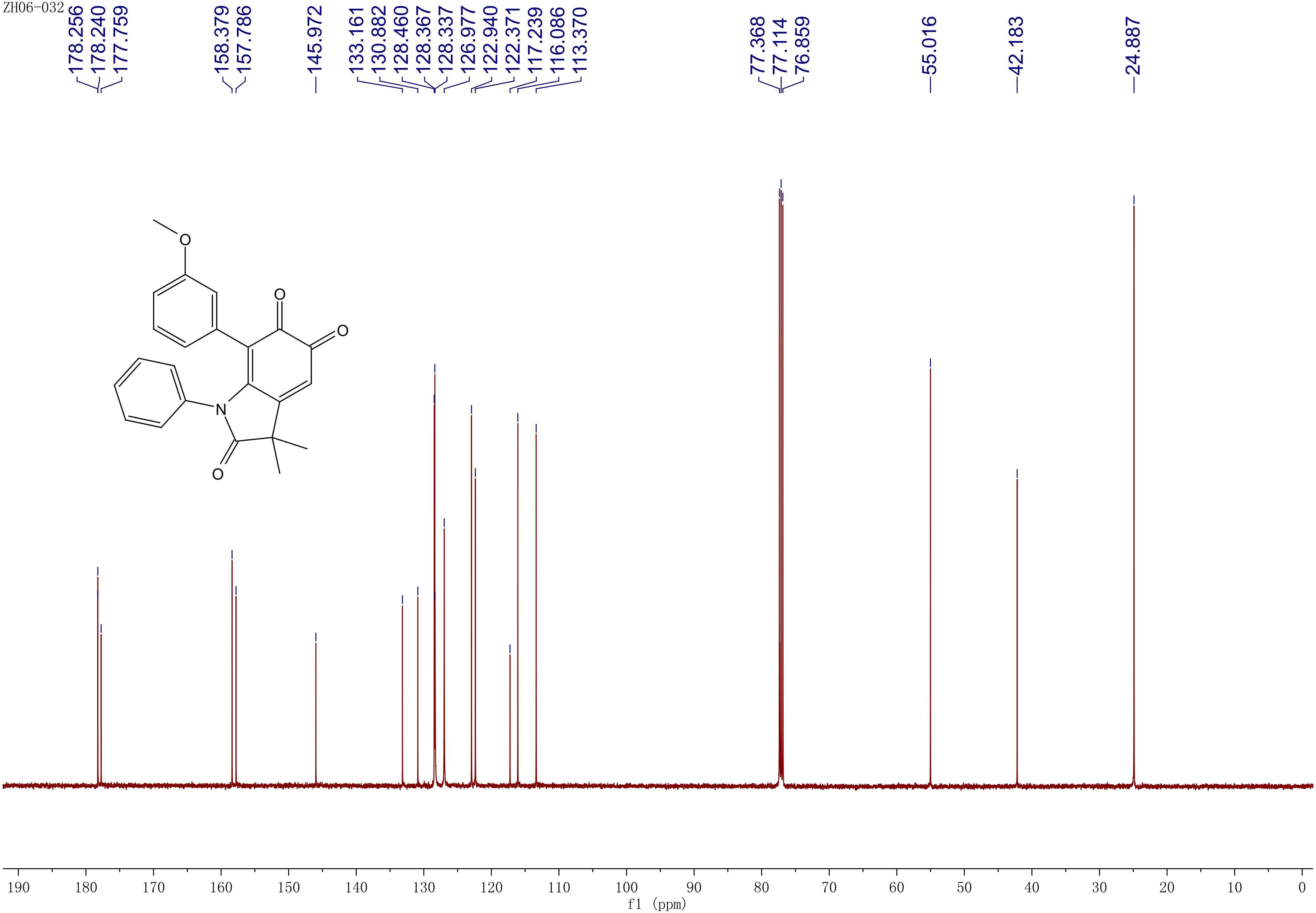
ZH06-032
178.256
178.240
177.759
158.379
157.786
145.972
133.161
130.882
128.460
128.367
128.337
126.977
122.940
122.371
117.239
116.086
113.370
77.368
77.114
76.859
55.016
42.183
24.887
O
O
O
N
O
O
190 180 170 160 150 140 130 120 110 100 90 80 70 60 50 40 30 20 10 0
f1 (ppm)

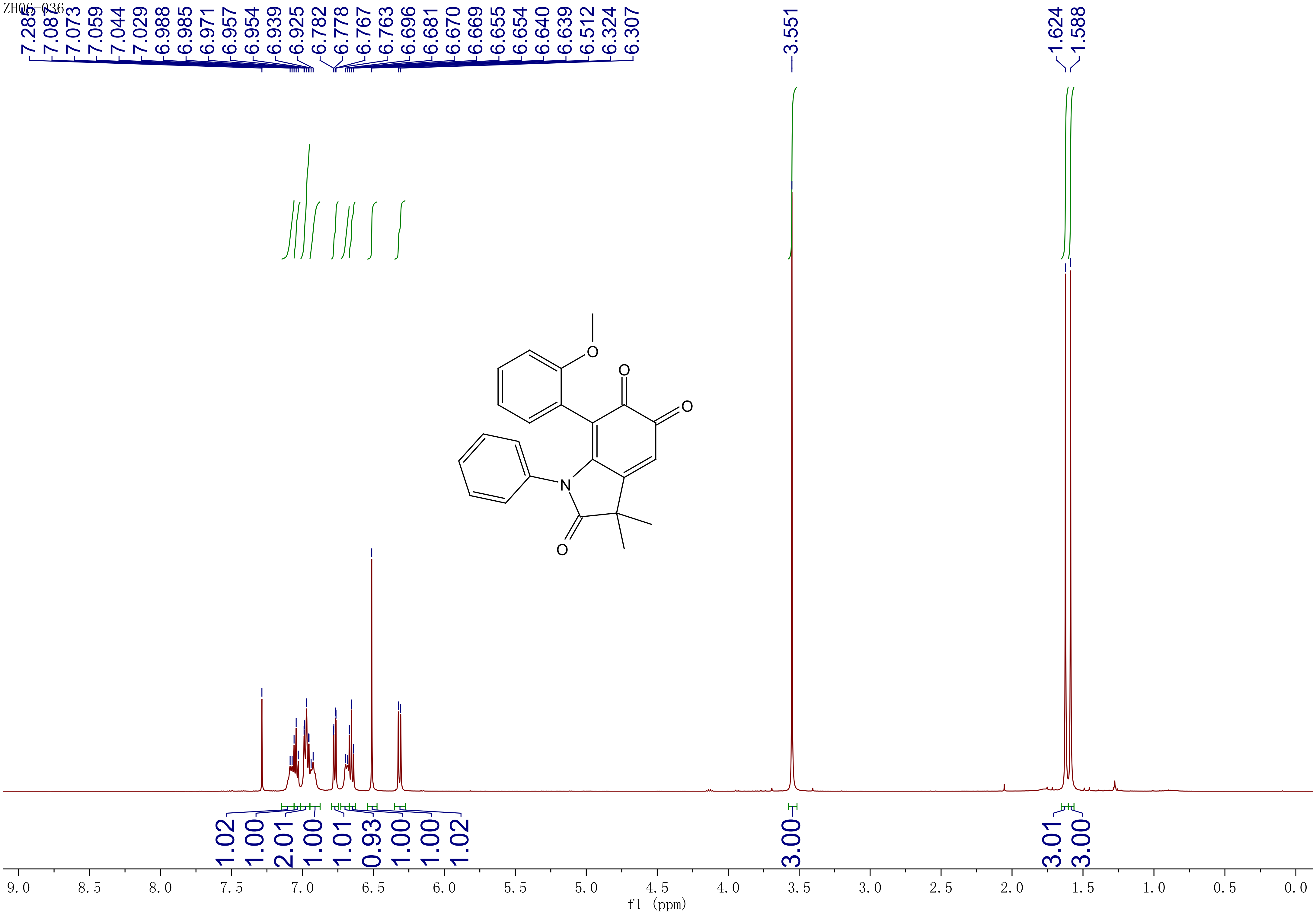
ZH06-036
7.285
7.087
7.073
7.059
7.044
7.029
6.988
6.985
6.971
6.957
6.954
6.939
6.925
6.782
6.778
6.767
6.763
6.696
6.681
6.670
6.669
6.655
6.654
6.640
6.639
6.512
6.324
6.307
3.551
1.624
1.588
O
O
O
N
O
1.02
1.00
2.01
1.00
1.01
0.93
1.00
1.00
1.02
3.00
3.01
3.00
9.0
8.5
8.0
7.5
7.0
6.5
6.0
5.5
5.0
4.5
4.0
3.5
3.0
2.5
2.0
1.5
1.0
0.5
0.0
f1 (ppm)

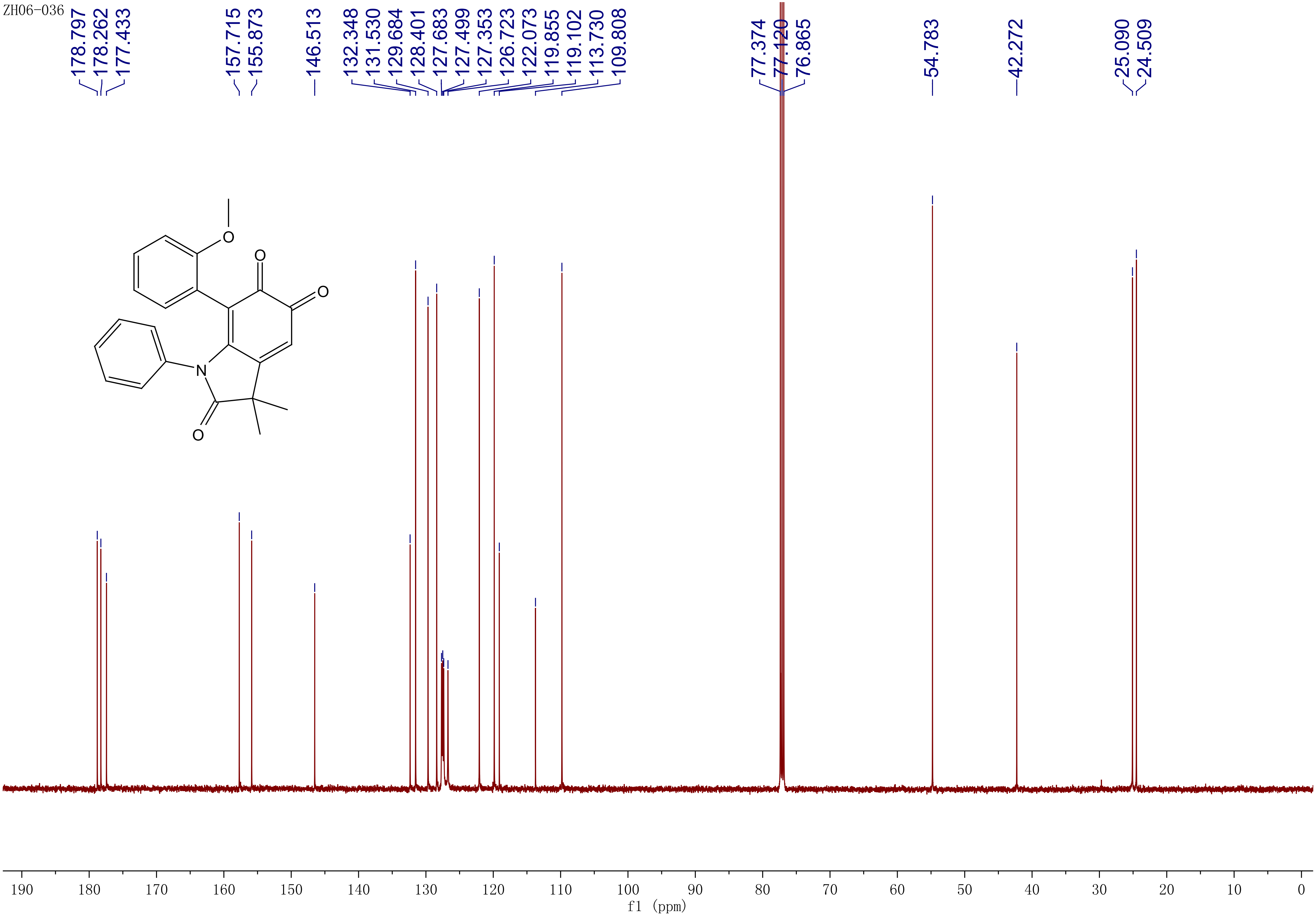
ZH06-036
178.797
178.262
177.433
157.715
155.873
146.513
132.348
131.530
129.684
128.401
127.683
127.499
127.353
126.723
122.073
119.855
119.102
113.730
109.808
77.374
77.120
76.865
54.783
42.272
25.090
24.509
O
O
O
N
O
190 180 170 160 150 140 130 120 110 100 90 80 70 60 50 40 30 20 10 0
f1 (ppm)

ZH06-051

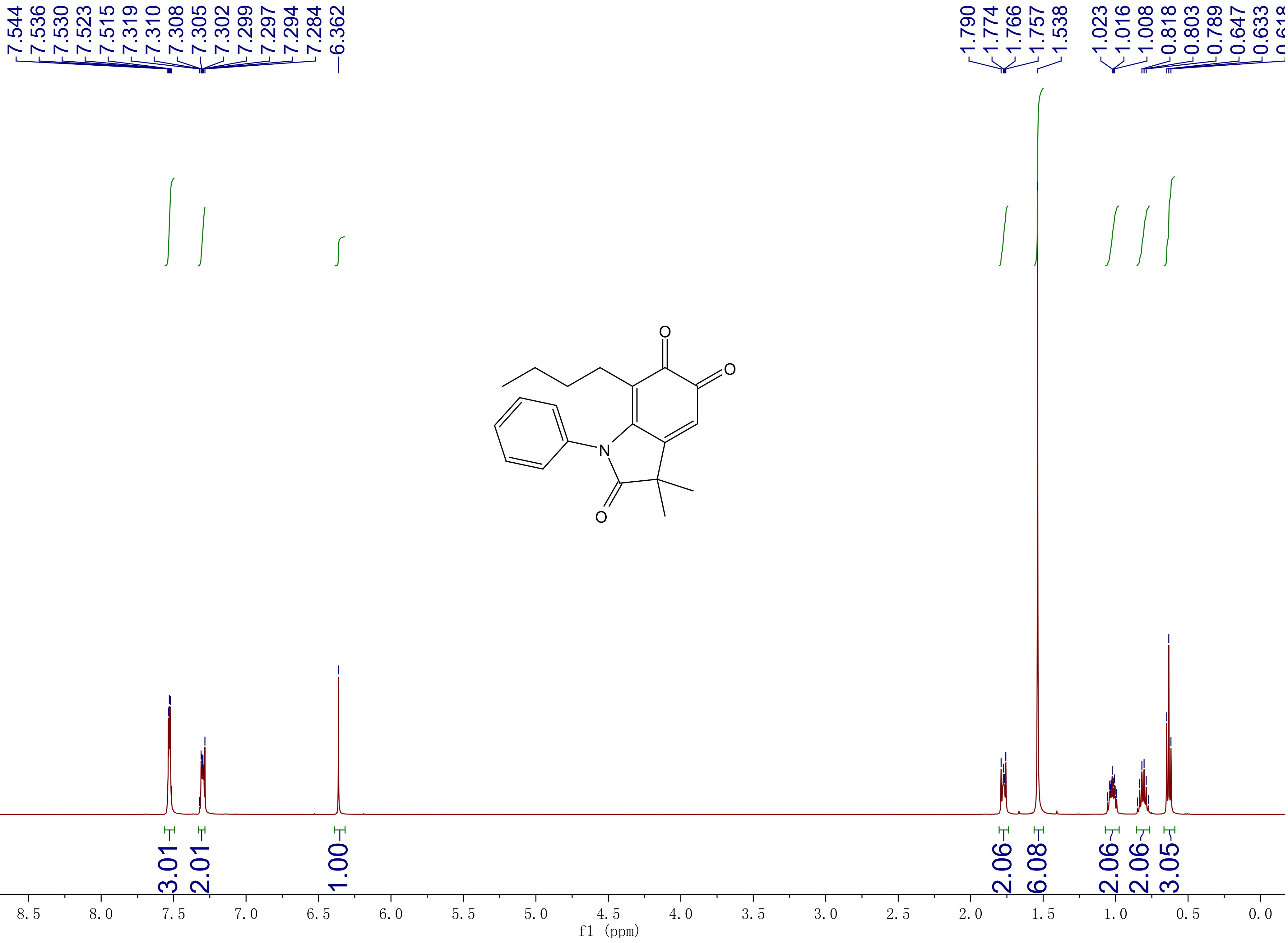
7.544
7.536
7.530
7.523
7.515
7.319
7.310
7.308
7.305
7.302
7.299
7.297
7.294
7.284
6.362
1.790
1.774
1.766
1.757
1.538
1.023
1.016
1.008
0.818
0.803
0.789
0.647
0.633
O
O
N
O
3.01
2.01
1.00
2.06
6.08
2.06
2.06
3.05
9.0
8.5
8.0
7.5
7.0
6.5
6.0
5.5
5.0
4.5
4.0
3.5
3.0
2.5
2.0
1.5
1.0
0.5
0.0
f1 (ppm)

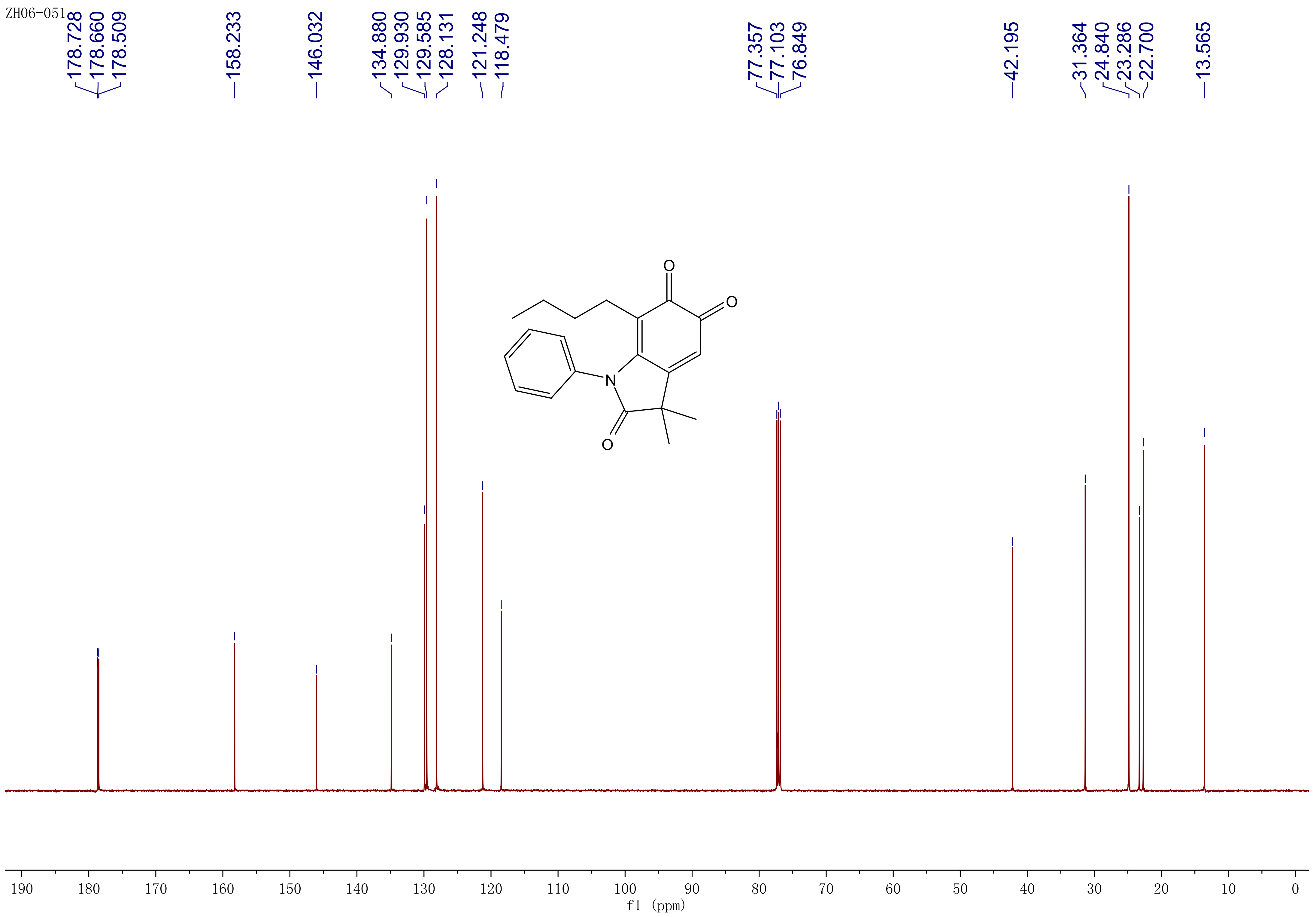

ZH06-051
178.728
178.660
178.509
158.233
146.032
134.880
129.930
129.585
128.131
121.248
118.479
77.357
77.103
76.849
42.195
31.364
24.840
23.286
22.700
13.565
O
O
N
O
190
180
170
160
150
140
130
120
110
100
90
80
70
60
50
40
30
20
10
0
f1 (ppm)

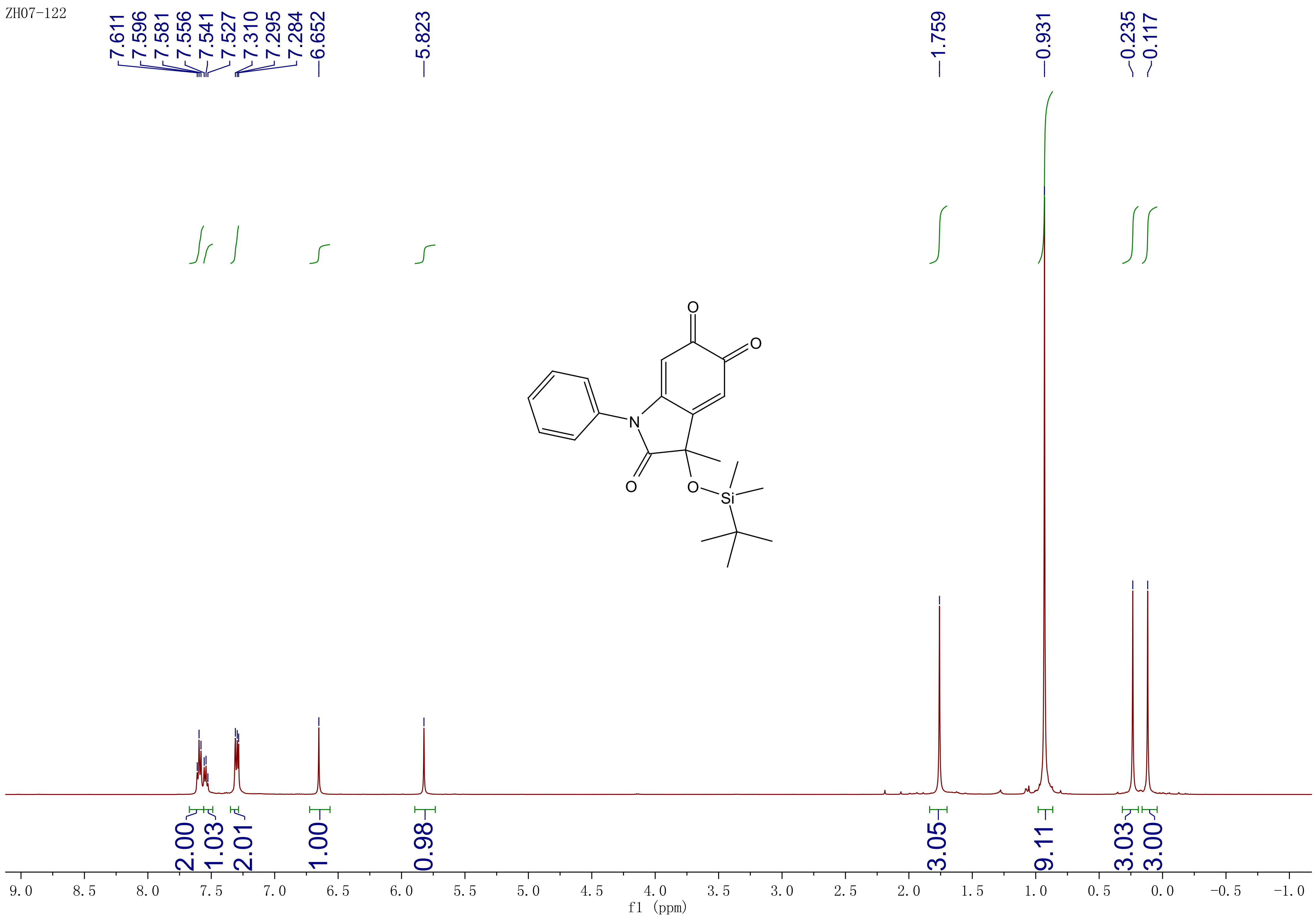
ZH07-122
7.611
7.596
7.581
7.556
7.541
7.527
7.310
7.295
7.284
6.652
5.823
1.759
0.931
0.235
0.117
O
O
N
O
O
Si
2.00
1.03
2.01
1.00
0.98
3.05
9.11
3.03
3.00
9.0
8.5
8.0
7.5
7.0
6.5
6.0
5.5
5.0
4.5
4.0
3.5
3.0
2.5
2.0
1.5
1.0
0.5
0.0
-0.5
-1.0
f1 (ppm)

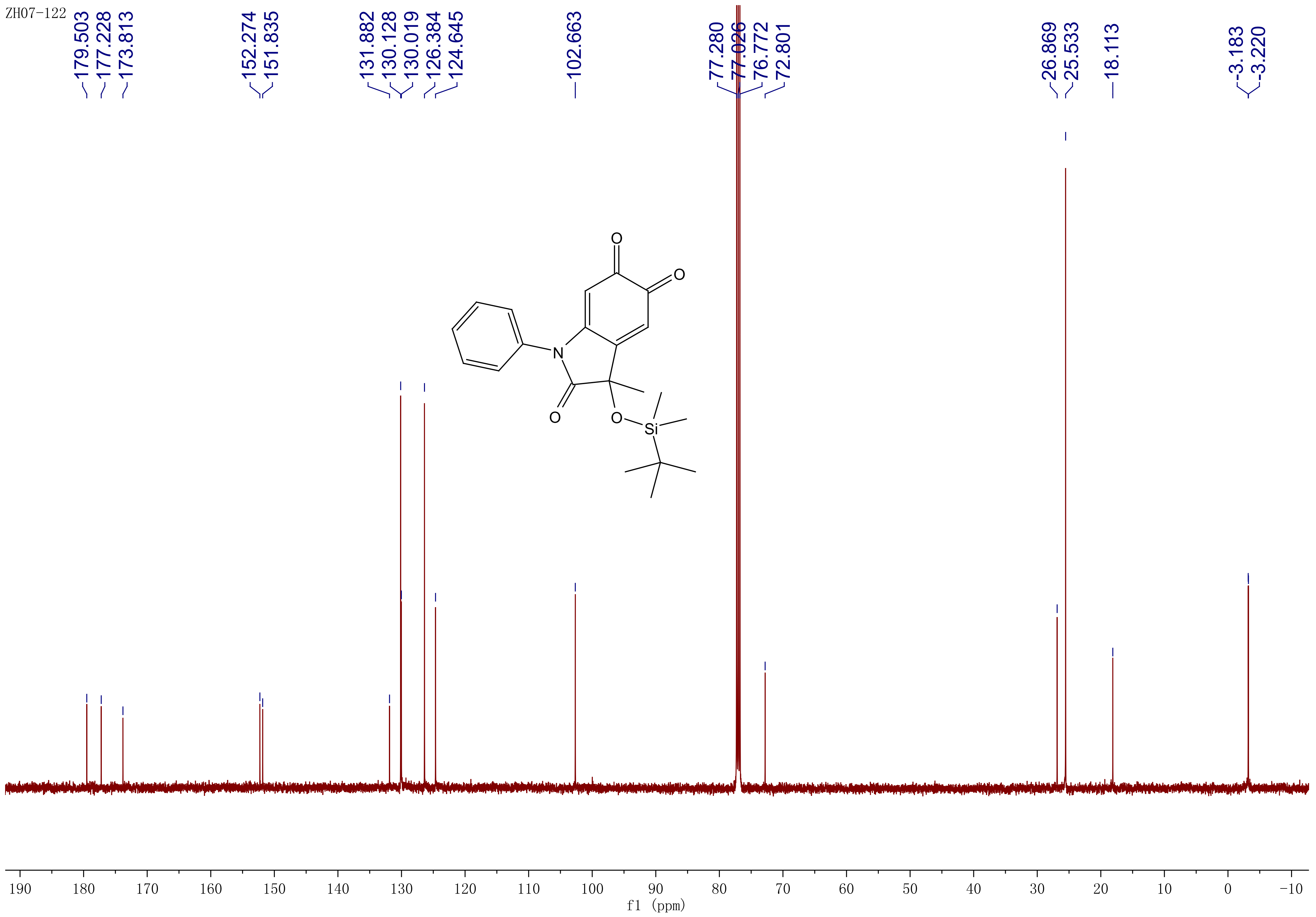
ZH07-122
179.503
177.228
173.813
152.274
151.835
131.882
130.128
130.019
126.384
124.645
102.663
77.280
77.026
76.772
72.801
26.869
25.533
18.113
-3.183
-3.220
O
O
N
O
O
Si
190 180 170 160 150 140 130 120 110 100 90 80 70 60 50 40 30 20 10 0 -10
f1 (ppm)

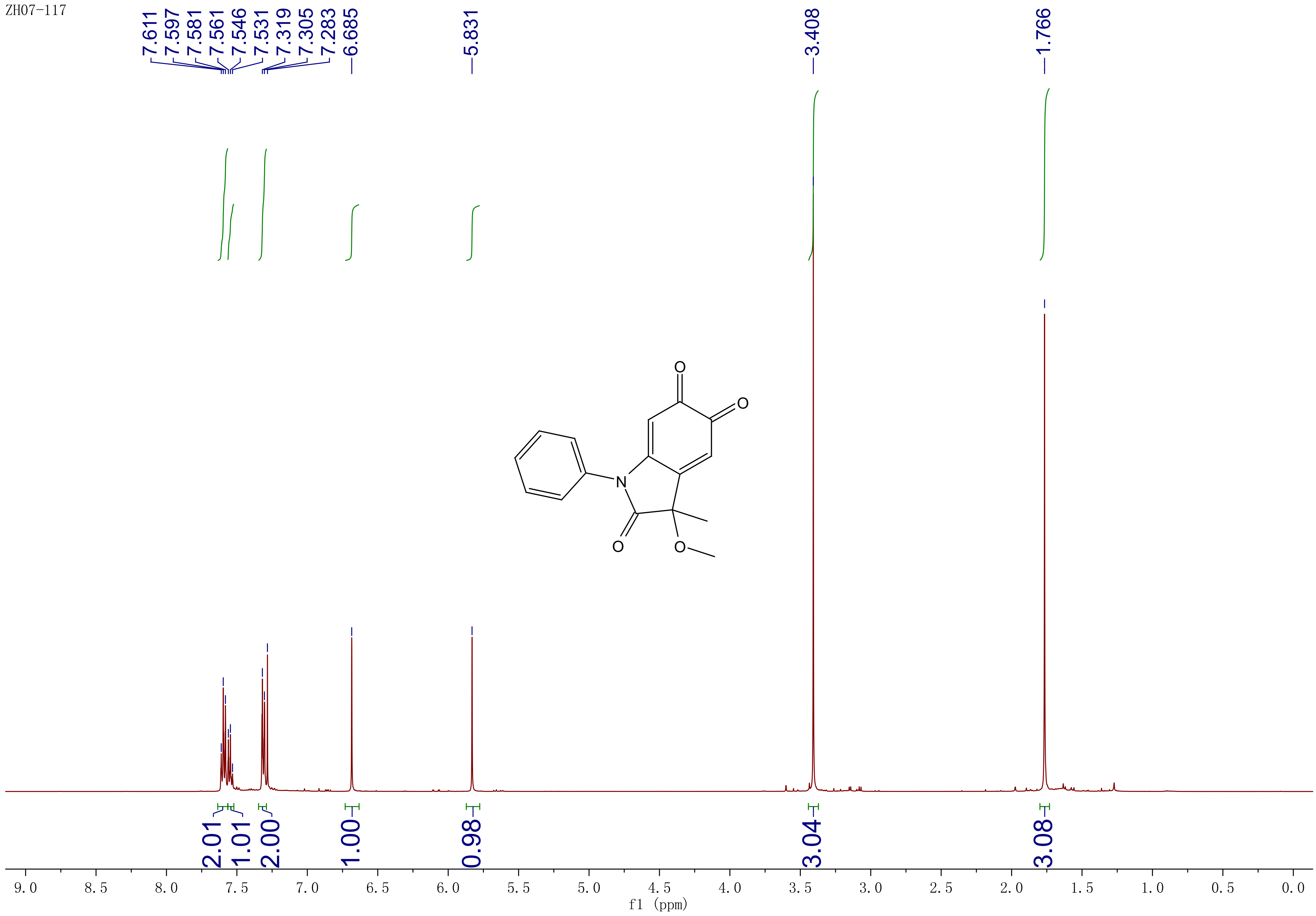
ZH07-117
7.611
7.597
7.581
7.561
7.546
7.531
7.319
7.305
7.283
6.685
5.831
3.408
1.766
2.01
1.01
2.00
1.00
0.98
3.04
3.08
O
O
N
O
O
9.0 8.5 8.0 7.5 7.0 6.5 6.0 5.5 5.0 4.5 4.0 3.5 3.0 2.5 2.0 1.5 1.0 0.5 0.0
f1 (ppm)

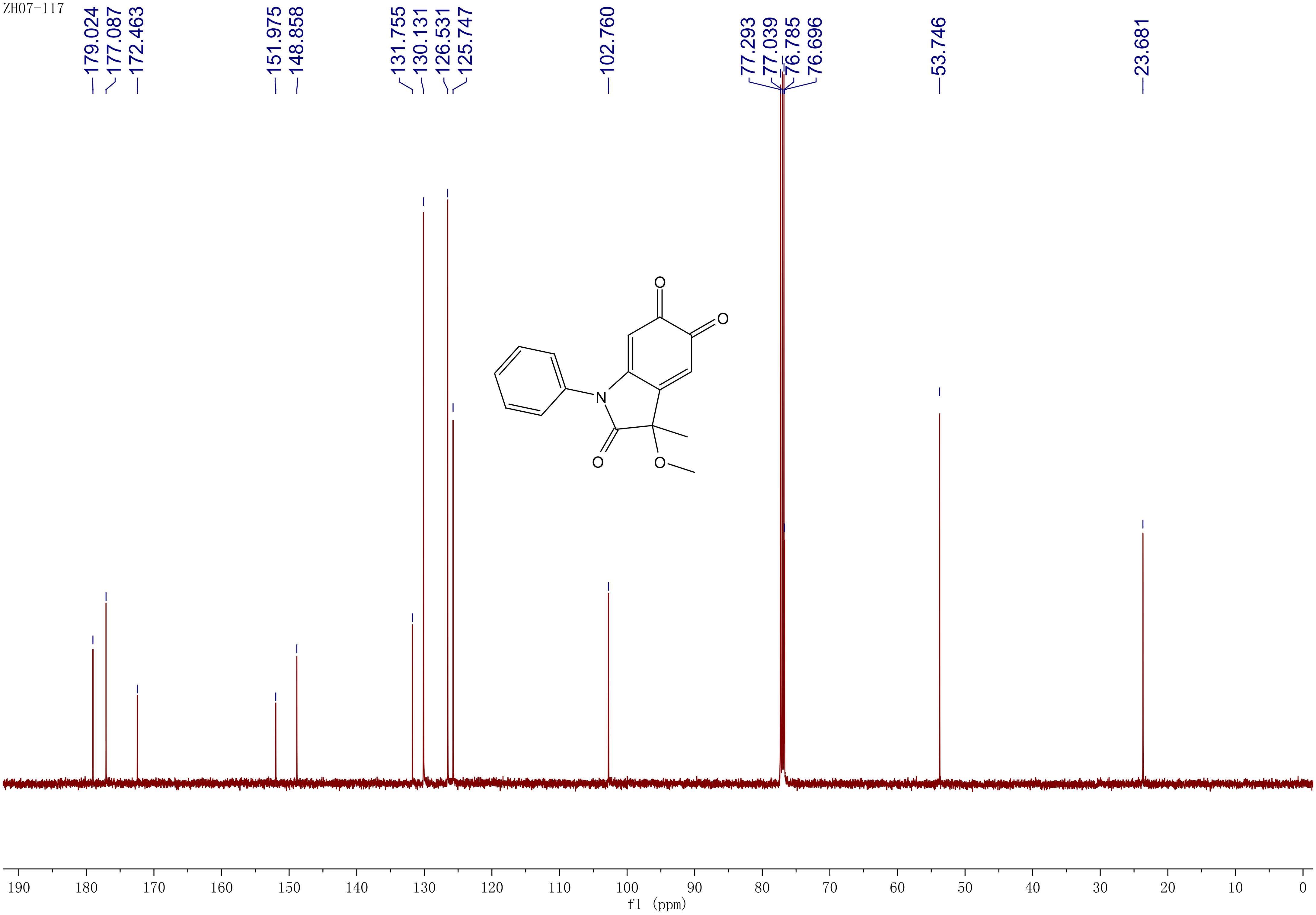
ZH07-117
179.024
177.087
172.463
151.975
148.858
131.755
130.131
126.531
125.747
102.760
77.293
77.039
76.785
76.696
53.746
23.681
O
O
N
O
O
190 180 170 160 150 140 130 120 110 100 90 80 70 60 50 40 30 20 10 0
f1 (ppm)

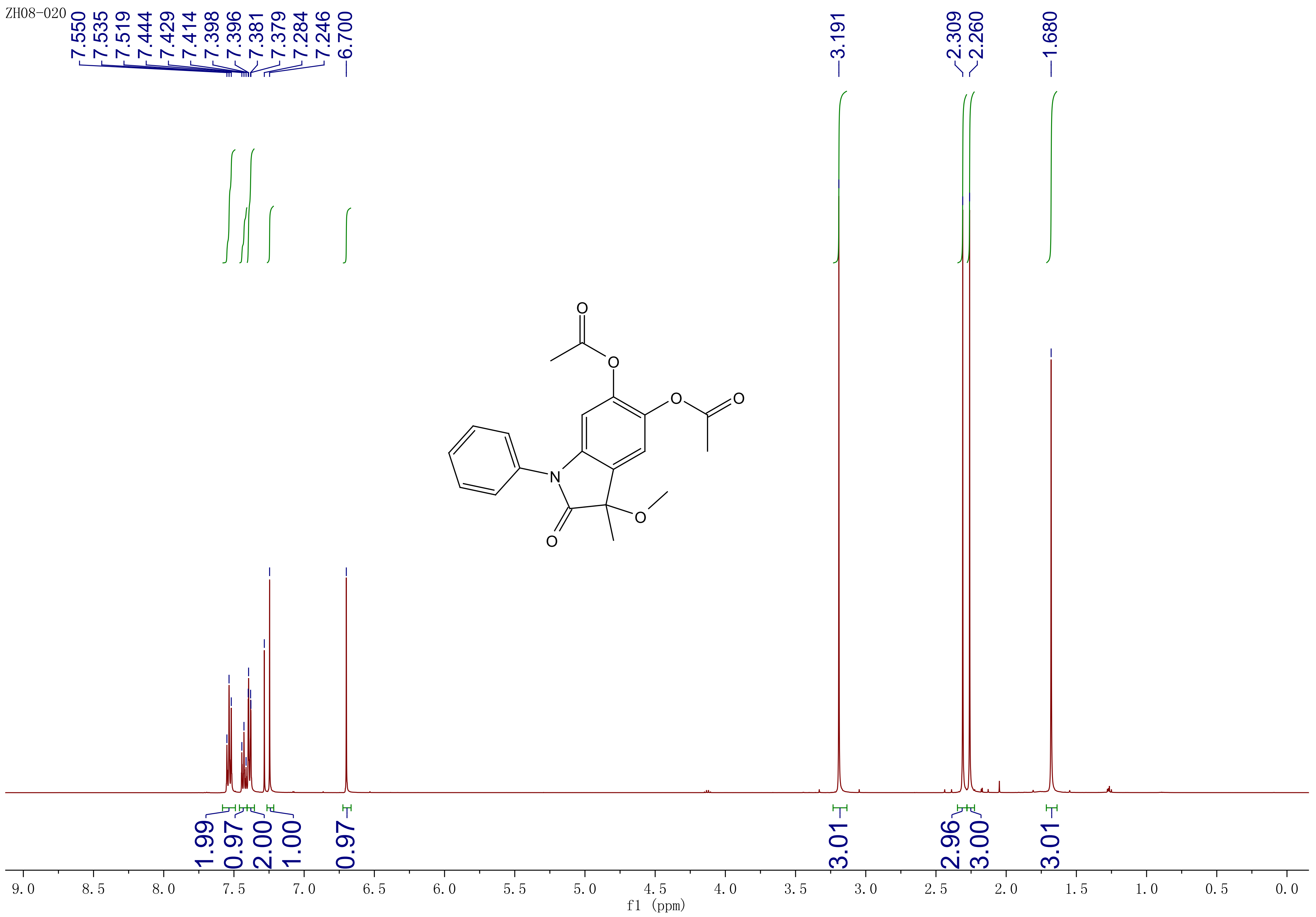
ZH08-020
7.550
7.535
7.519
7.444
7.429
7.414
7.398
7.396
7.381
7.379
7.284
7.246
6.700
3.191
2.309
2.260
1.680
1.99
0.97
2.00
1.00
0.97
3.01
2.96
3.00
3.01
9.0
8.5
8.0
7.5
7.0
6.5
6.0
5.5
5.0
4.5
4.0
3.5
3.0
2.5
2.0
1.5
1.0
0.5
0.0
f1 (ppm)
O
O
O
O
N
O
O

ZH08-020

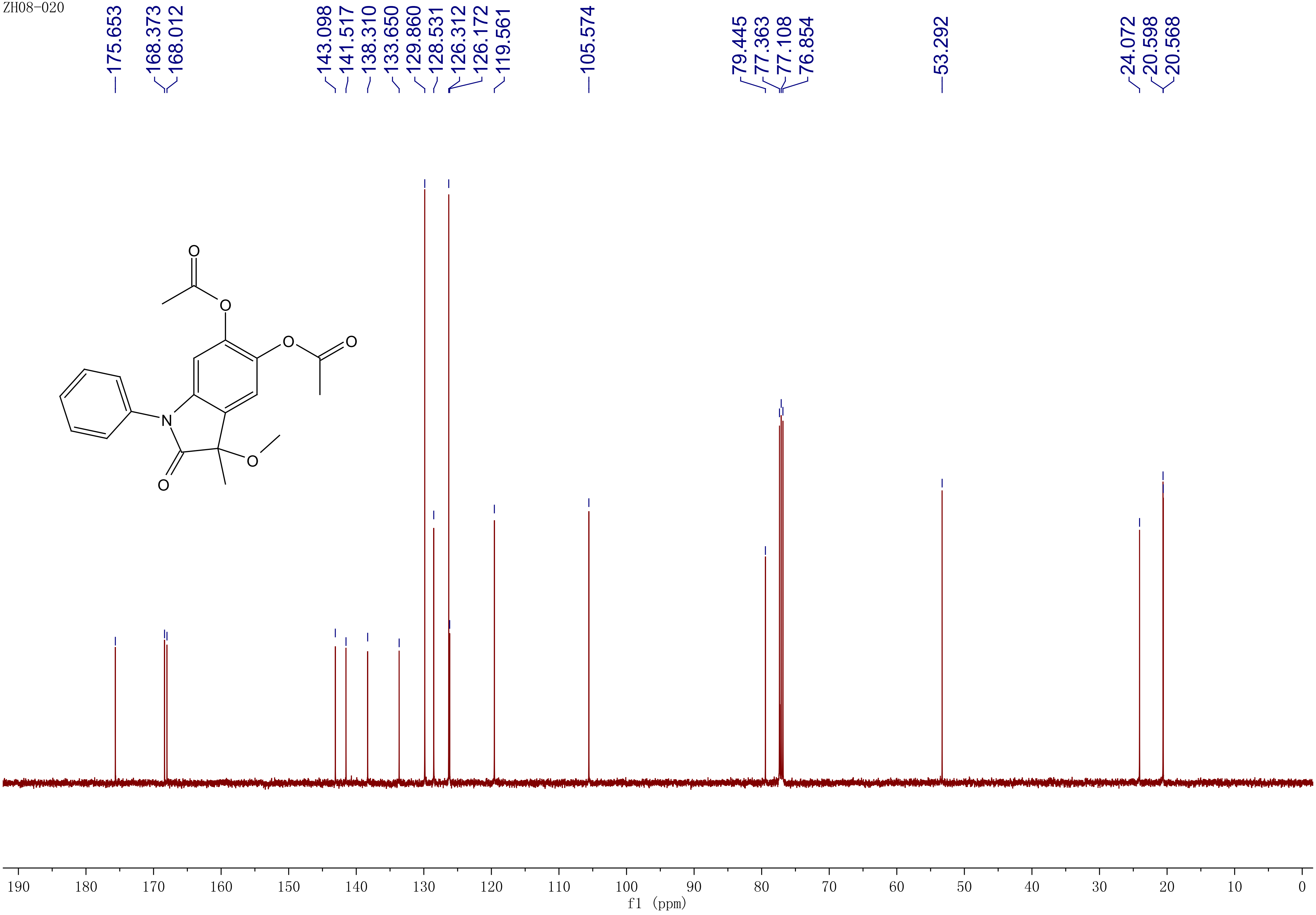
175.653
168.373
168.012
143.098
141.517
138.310
133.650
129.860
128.531
126.312
126.172
119.561
105.574
79.445
77.363
77.108
76.854
53.292
24.072
20.598
20.568
190 180 170 160 150 140 130 120 110 100 90 80 70 60 50 40 30 20 10 0
f1 (ppm)

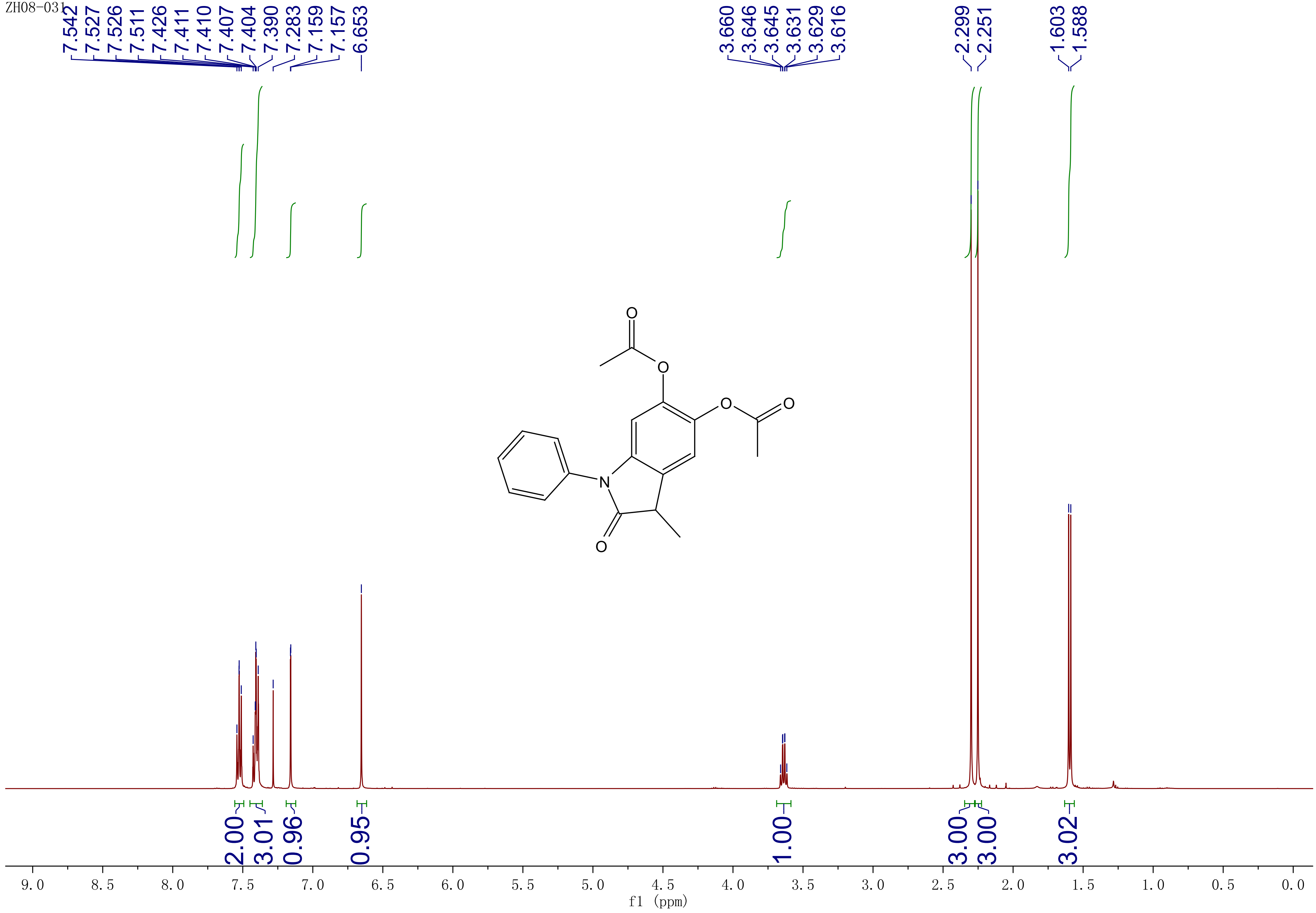

ZH08-031
7.542
7.527
7.526
7.511
7.426
7.411
7.410
7.407
7.404
7.390
7.283
7.159
7.157
6.653
3.660
3.646
3.645
3.631
3.629
3.616
2.299
2.251
1.603
1.588
2.00
3.01
0.96
0.95
1.00
3.00
3.00
3.02
9.0
8.5
8.0
7.5
7.0
6.5
6.0
5.5
5.0
4.5
4.0
3.5
3.0
2.5
2.0
1.5
1.0
0.5
0.0
f1 (ppm)

ZH08-031

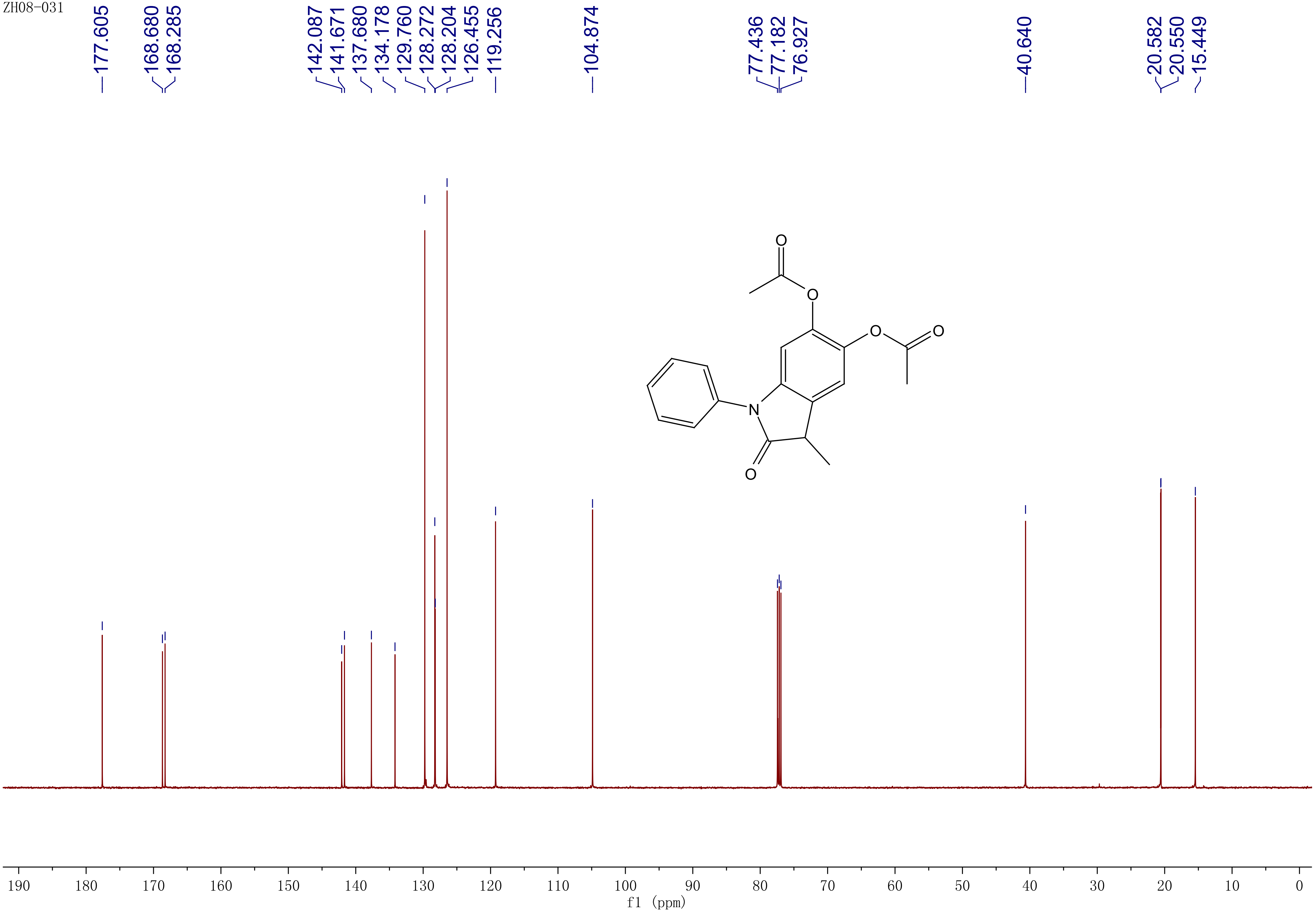
177.605
168.680
168.285
142.087
141.671
137.680
134.178
129.760
128.272
128.204
126.455
119.256
104.874
77.436
77.182
76.927
40.640
20.582
20.550
15.449
O
O
O
O
N
O
190 180 170 160 150 140 130 120 110 100 90 80 70 60 50 40 30 20 10 0
f1 (ppm)

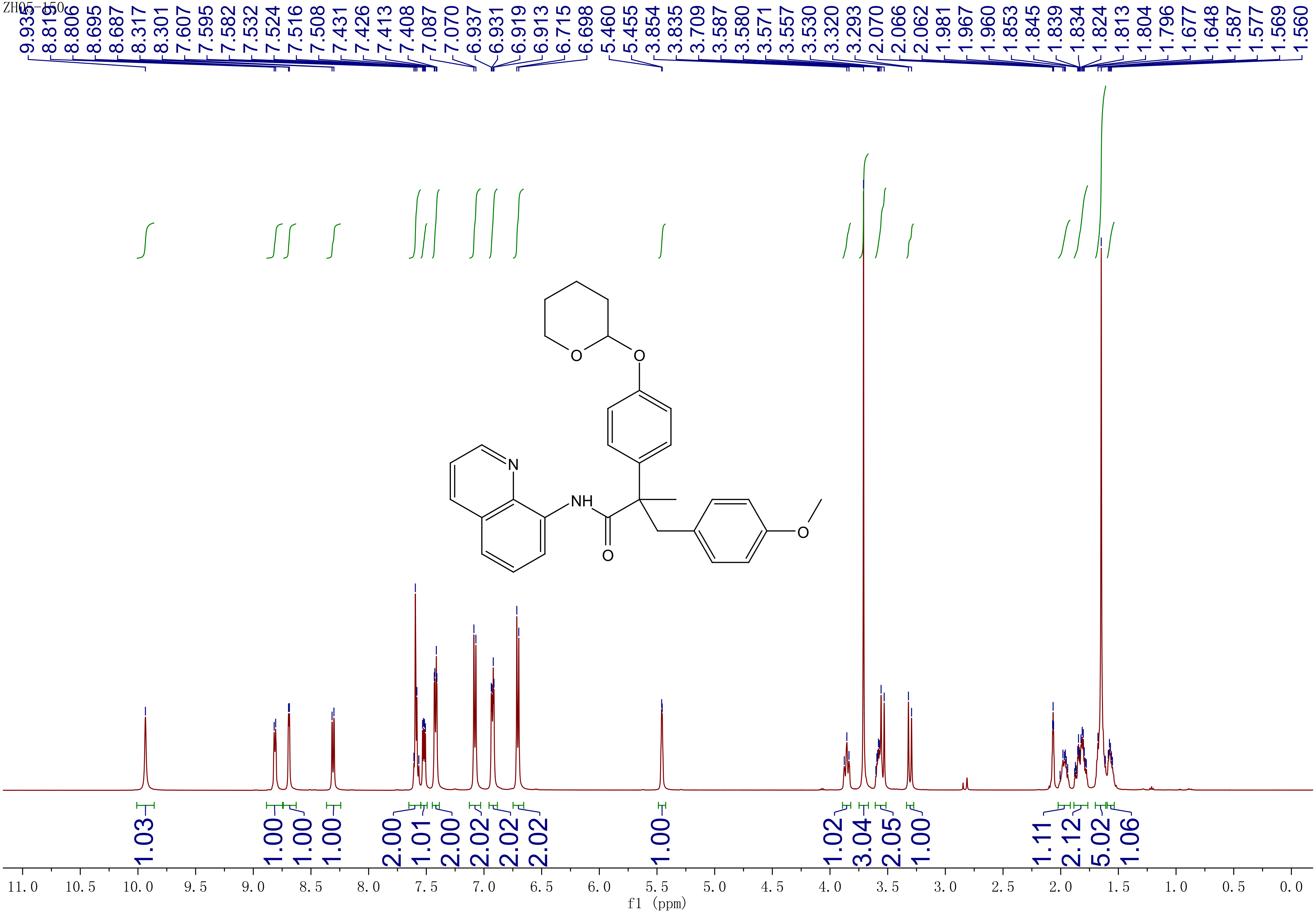
ZH05-150
9.935
8.819
8.806
8.695
8.687
8.317
8.301
7.607
7.595
7.582
7.532
7.524
7.516
7.508
7.431
7.426
7.413
7.408
7.087
7.070
6.937
6.931
6.919
6.913
6.715
6.698
5.460
5.455
3.854
3.835
3.709
3.587
3.580
3.571
3.557
3.530
3.320
3.293
2.070
2.066
2.062
1.981
1.967
1.960
1.853
1.845
1.839
1.834
1.824
1.813
1.804
1.796
1.677
1.648
1.587
1.577
1.569
1.560
N
NH
O
O
O
O
1.03
1.00
1.00
1.00
2.00
1.01
2.00
2.02
2.02
2.02
1.00
1.02
3.04
2.05
1.00
1.11
2.12
5.02
1.06
11.0 10.5 10.0 9.5 9.0 8.5 8.0 7.5 7.0 6.5 6.0 5.5 5.0 4.5 4.0 3.5 3.0 2.5 2.0 1.5 1.0 0.5 0.0
f1 (ppm)

ZH05-150

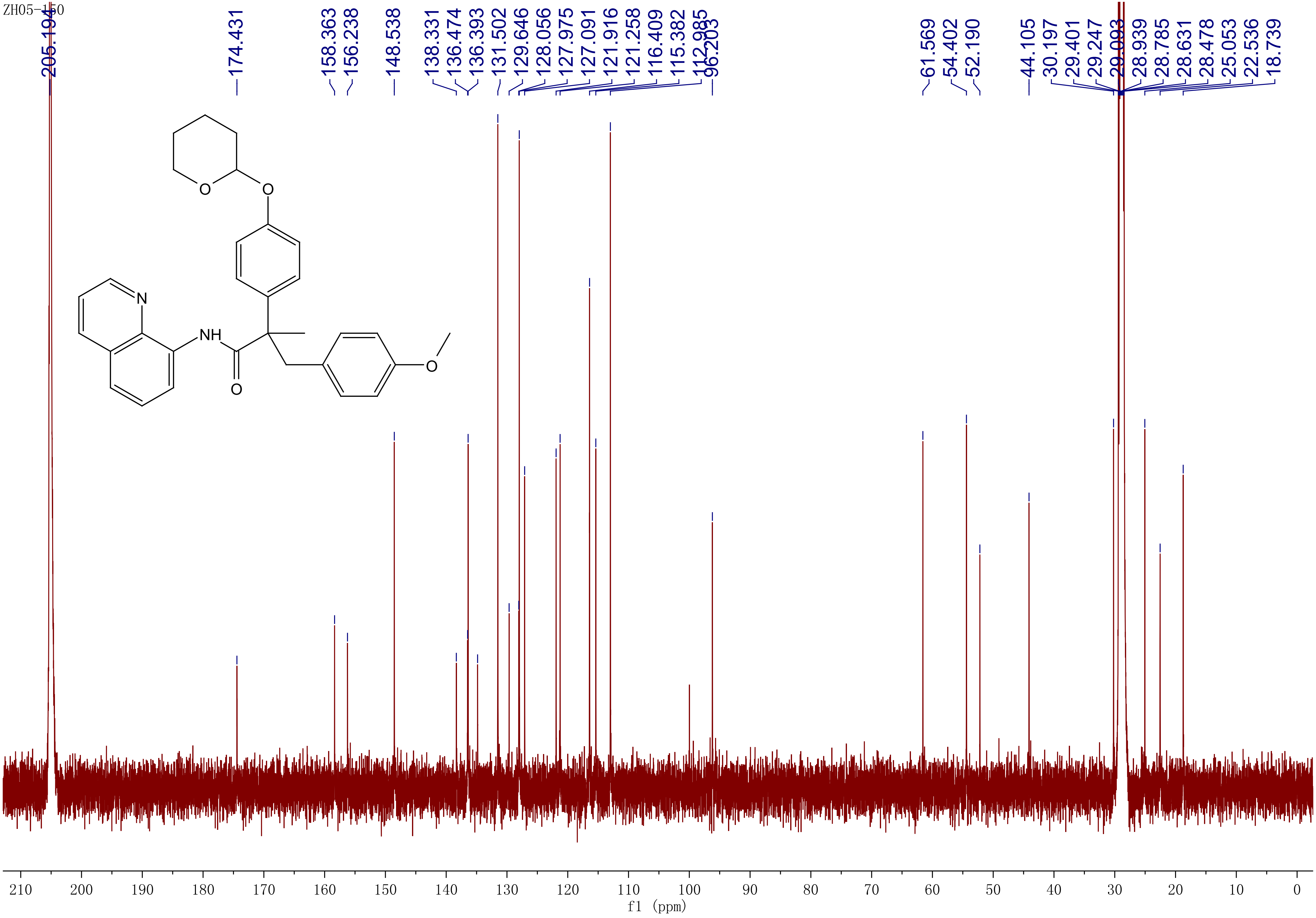
205.194
174.431
158.363
156.238
148.538
138.331
136.474
136.393
131.502
129.646
128.056
127.975
127.091
121.916
121.258
116.409
115.382
112.985
96.203
61.569
54.402
52.190
44.105
30.197
29.401
29.247
29.093
28.939
28.785
28.631
28.478
25.053
22.536
18.739
O
O
N
NH
O
O
210 200 190 180 170 160 150 140 130 120 110 100 90 80 70 60 50 40 30 20 10 0
f1 (ppm)

ZH06-009

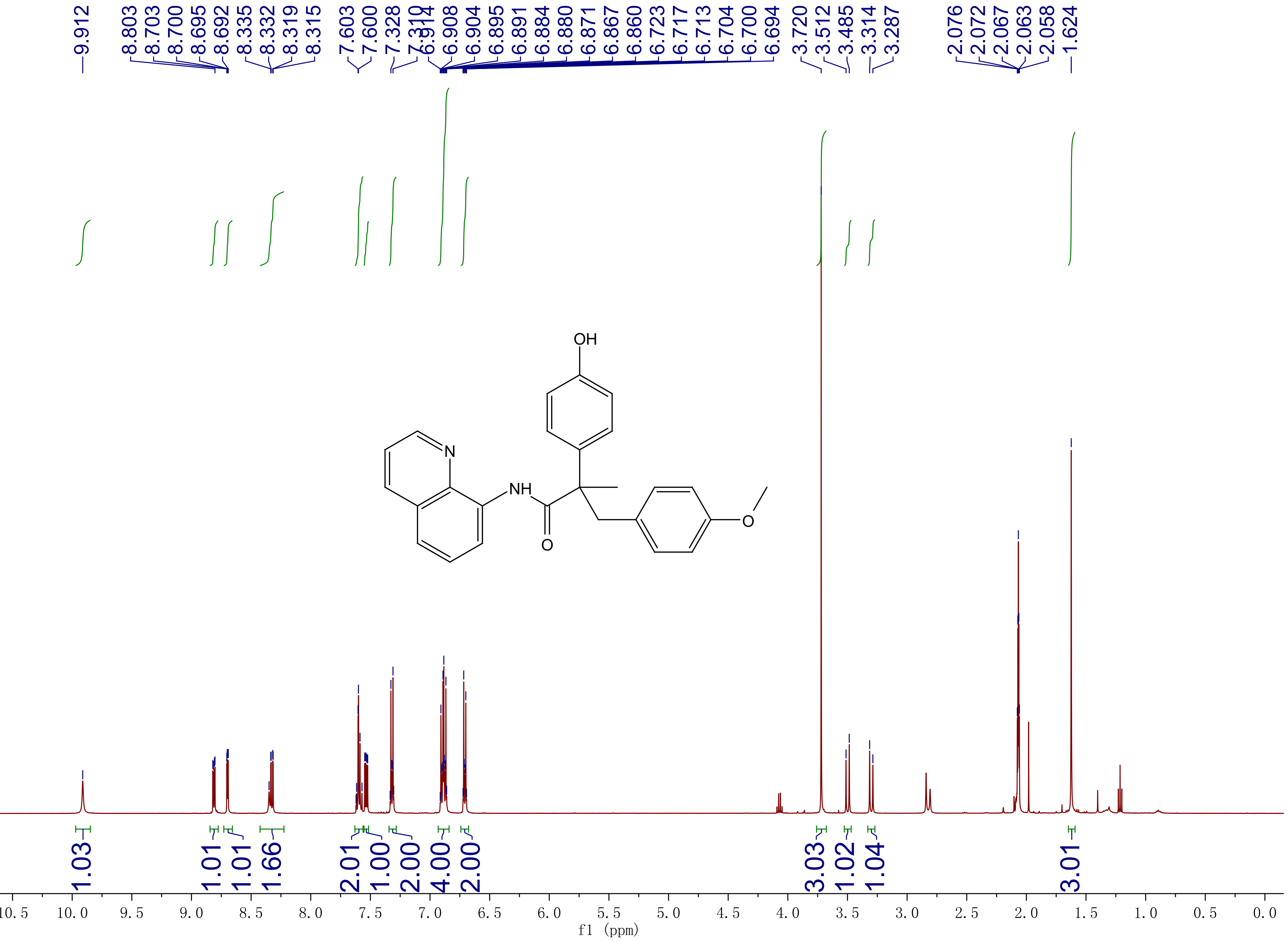

9.912
8.803
8.703
8.700
8.695
8.692
8.335
8.332
8.319
8.315
7.603
7.600
7.328
7.310
6.914
6.908
6.904
6.895
6.891
6.884
6.880
6.871
6.867
6.860
6.723
6.717
6.713
6.704
6.700
6.694
3.720
3.512
3.485
3.314
3.287
2.076
2.072
2.067
2.063
2.058
1.624
OH
N
NH
O
O
1.03
1.01
1.01
1.66
2.01
1.00
2.00
4.00
2.00
3.03
1.02
1.04
3.01
11.0
10.5
10.0
9.5
9.0
8.5
8.0
7.5
7.0
6.5
6.0
5.5
5.0
4.5
4.0
3.5
3.0
2.5
2.0
1.5
1.0
0.5
0.0
f1 (ppm)

ZH06-009

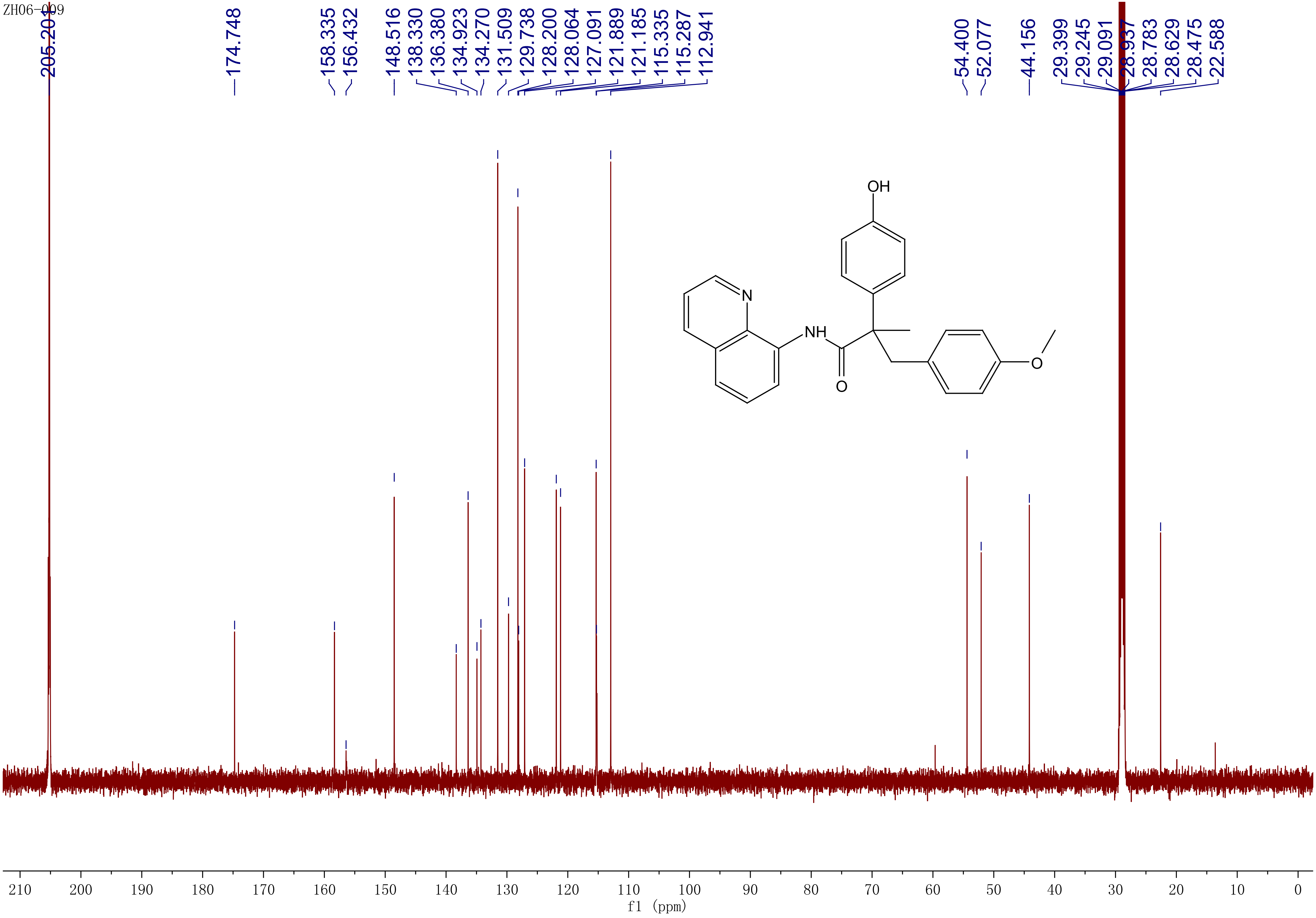
205.201
174.748
158.335
156.432
148.516
138.330
136.380
134.923
134.270
131.509
129.738
128.200
128.064
127.091
121.889
121.185
115.335
115.287
112.941
54.400
52.077
44.156
29.399
29.245
29.091
28.937
28.783
28.629
28.475
22.588
OH
N
NH
O
O
210 200 190 180 170 160 150 140 130 120 110 100 90 80 70 60 50 40 30 20 10 0
f1 (ppm)

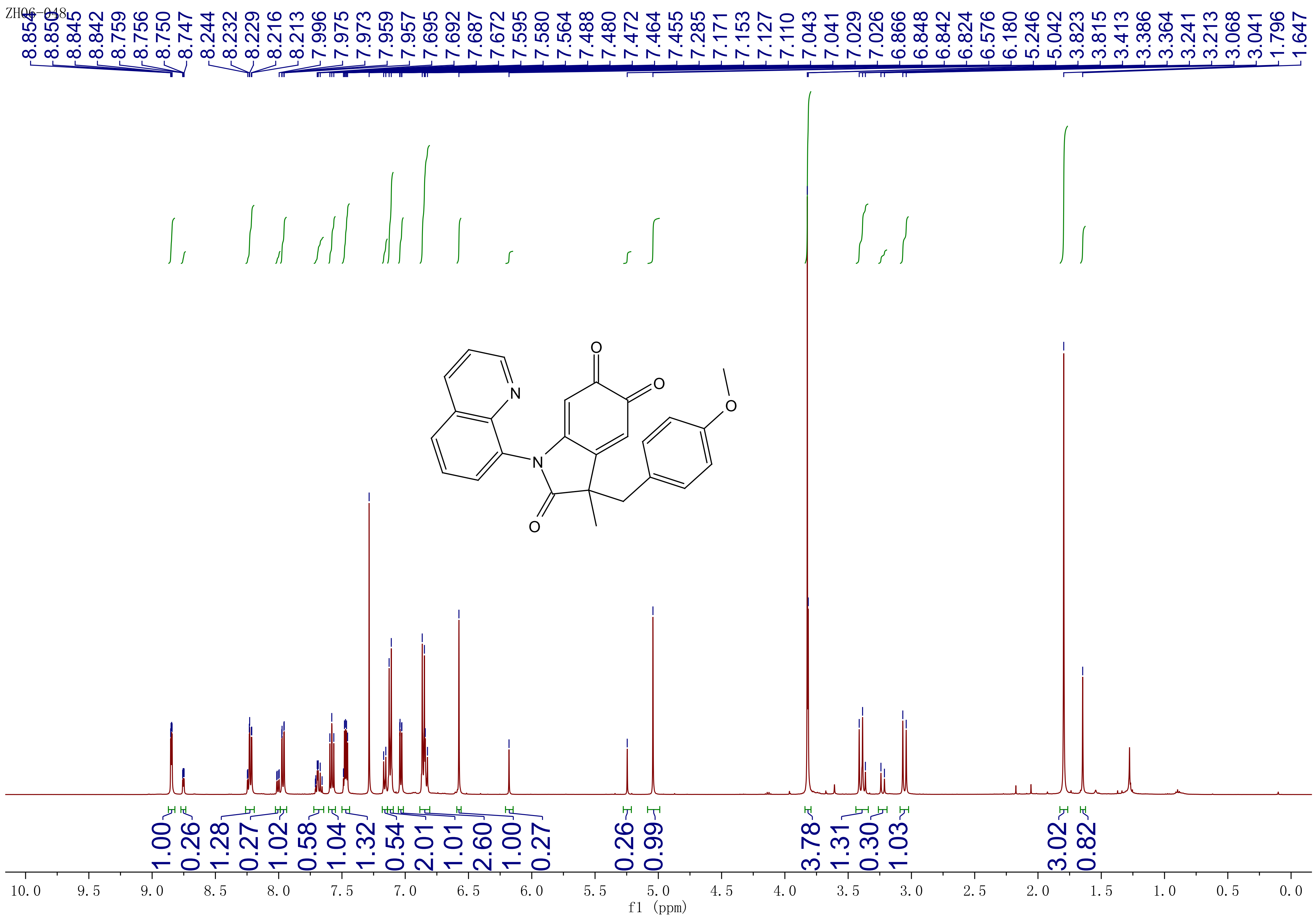
ZH06-048
8.854
8.850
8.845
8.842
8.759
8.756
8.750
8.747
8.244
8.232
8.229
8.216
8.213
7.996
7.975
7.973
7.959
7.957
7.695
7.692
7.687
7.672
7.595
7.580
7.564
7.488
7.480
7.472
7.464
7.455
7.285
7.171
7.153
7.127
7.110
7.043
7.041
7.029
7.026
6.866
6.848
6.842
6.824
6.576
6.180
5.246
5.042
3.823
3.815
3.413
3.386
3.364
3.241
3.213
3.068
3.041
1.796
1.647
N
N
O
O
O
O
1.00
0.26
1.28
0.27
1.02
0.58
1.04
1.32
0.54
2.01
1.01
2.60
1.00
0.27
0.26
0.99
3.78
1.31
0.30
1.03
3.02
0.82
10.0
9.5
9.0
8.5
8.0
7.5
7.0
6.5
6.0
5.5
5.0
4.5
4.0
3.5
3.0
2.5
2.0
1.5
1.0
0.5
0.0
f1 (ppm)

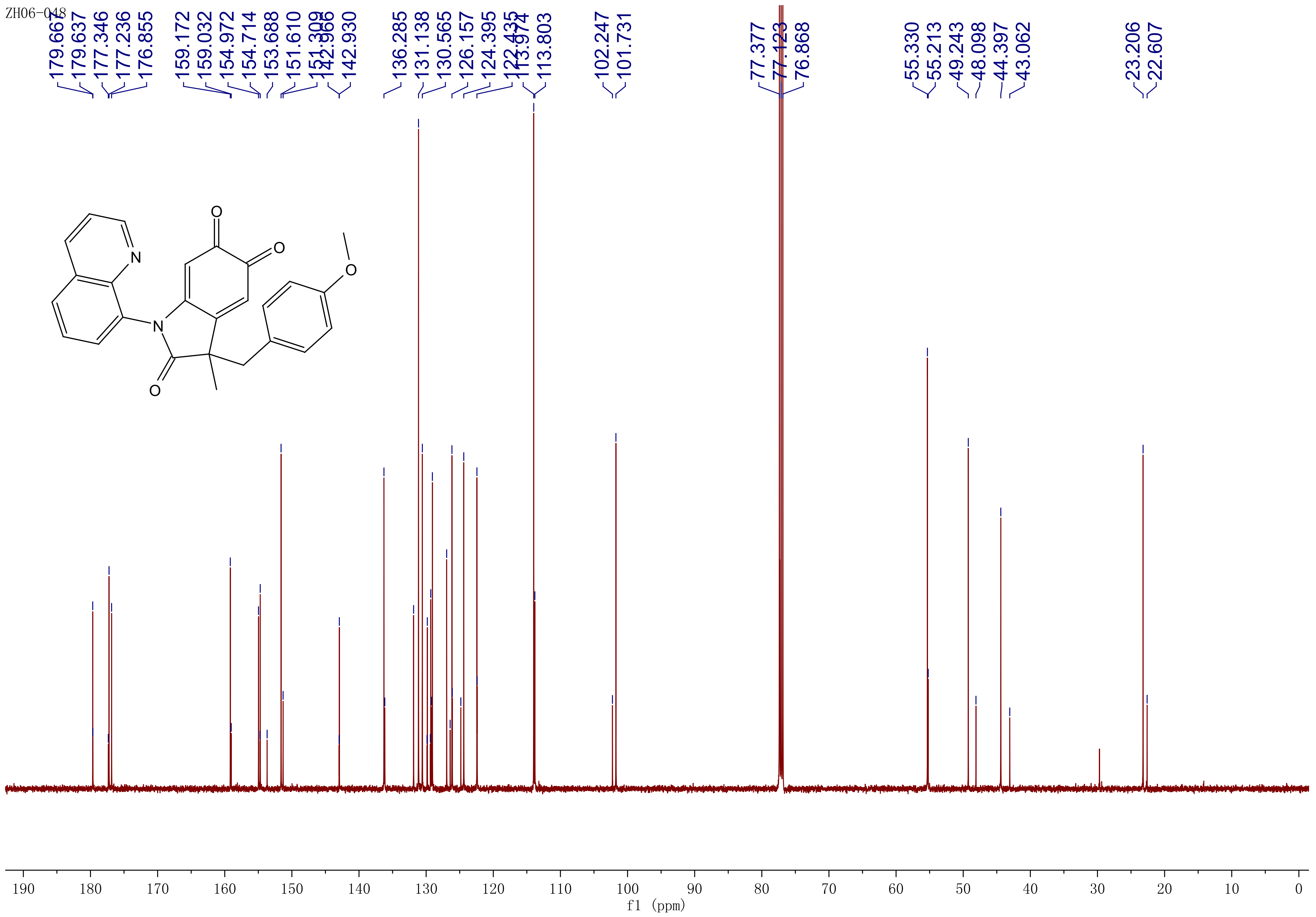
ZH06-048
179.667
179.637
177.346
177.236
176.855
159.172
159.032
154.972
154.714
153.688
151.610
151.309
142.930
136.285
131.138
130.565
126.157
124.395
122.435
113.974
113.803
102.247
101.731
77.377
77.123
76.868
55.330
55.213
49.243
48.098
44.397
43.062
23.206
22.607
N
O
190 180 170 160 150 140 130 120 110 100 90 80 70 60 50 40 30 20 10 0
f1 (ppm)

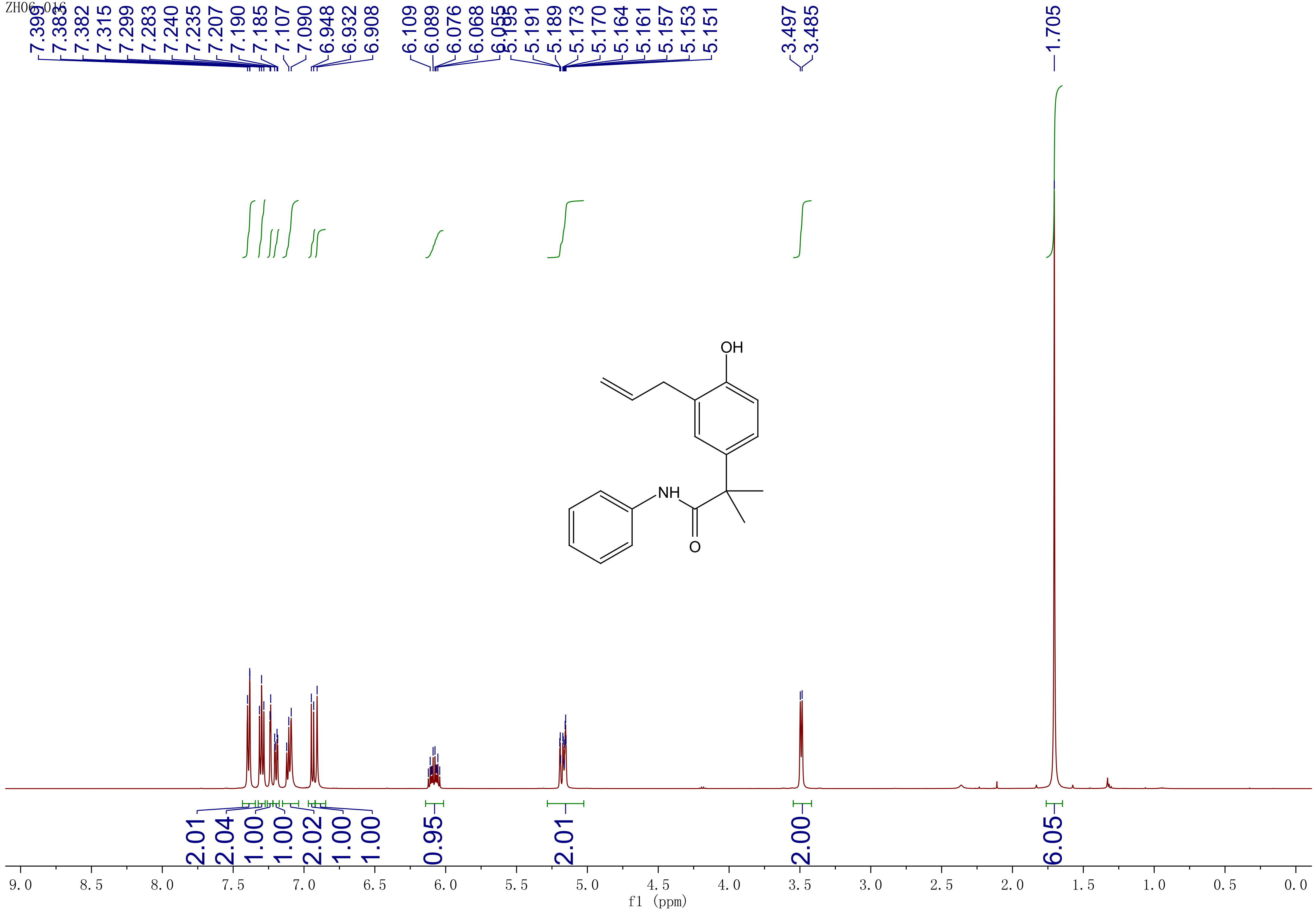

ZH06-016
7.399
7.383
7.382
7.315
7.299
7.283
7.240
7.235
7.207
7.190
7.185
7.107
7.090
6.948
6.932
6.908
6.109
6.089
6.076
6.068
6.055
5.195
5.191
5.189
5.173
5.170
5.164
5.161
5.157
5.153
5.151
3.497
3.485
1.705
OH
NH
O
2.01
2.04
1.00
1.00
2.02
1.00
1.00
0.95
2.01
2.00
6.05
9.0
8.5
8.0
7.5
7.0
6.5
6.0
5.5
5.0
4.5
4.0
3.5
3.0
2.5
2.0
1.5
1.0
0.5
0.0
f1 (ppm)

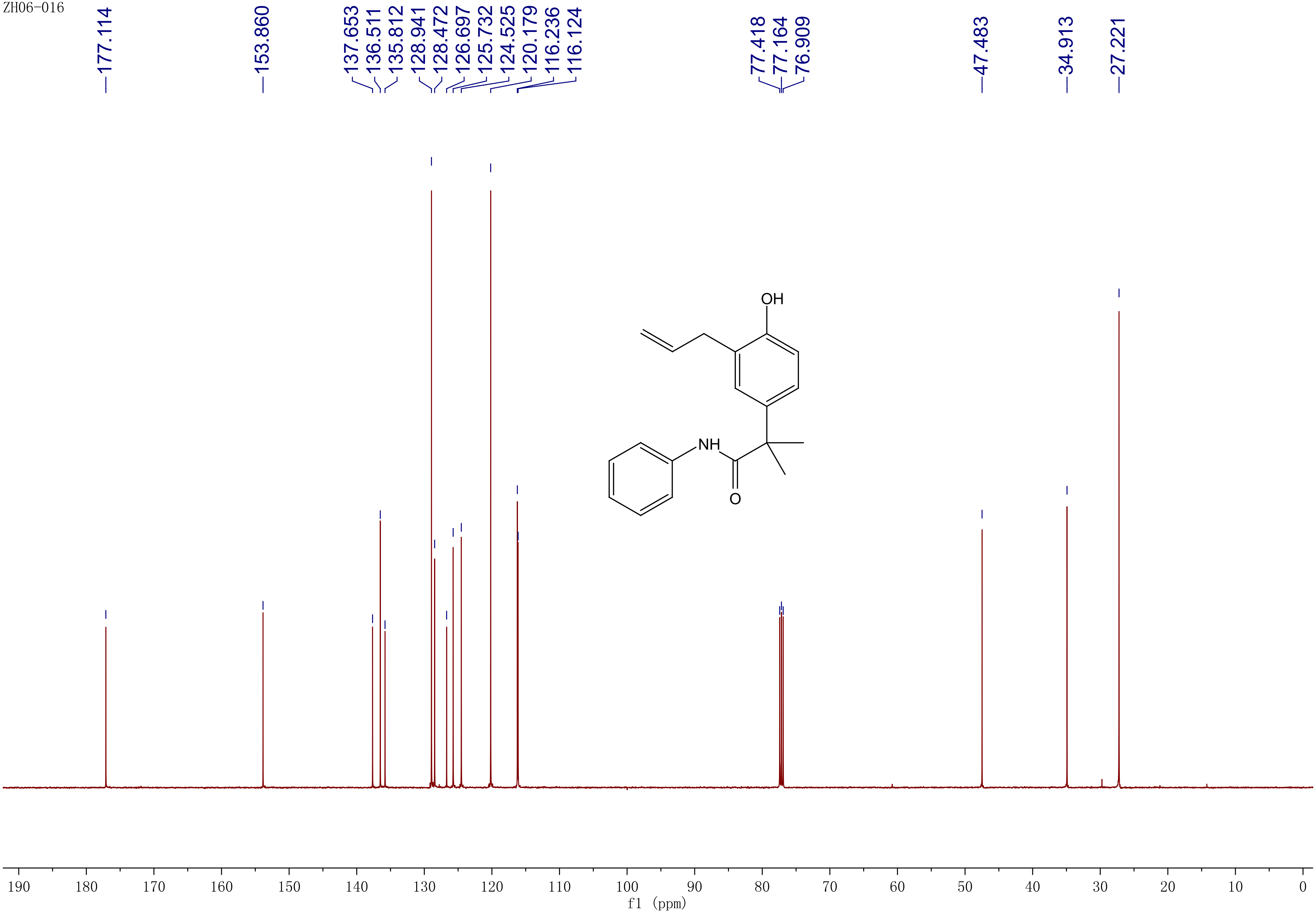
ZH06-016
177.114
153.860
137.653
136.511
135.812
128.941
128.472
126.697
125.732
124.525
120.179
116.236
116.124
77.418
77.164
76.909
47.483
34.913
27.221
OH
NH
O
190 180 170 160 150 140 130 120 110 100 90 80 70 60 50 40 30 20 10 0
f1 (ppm)

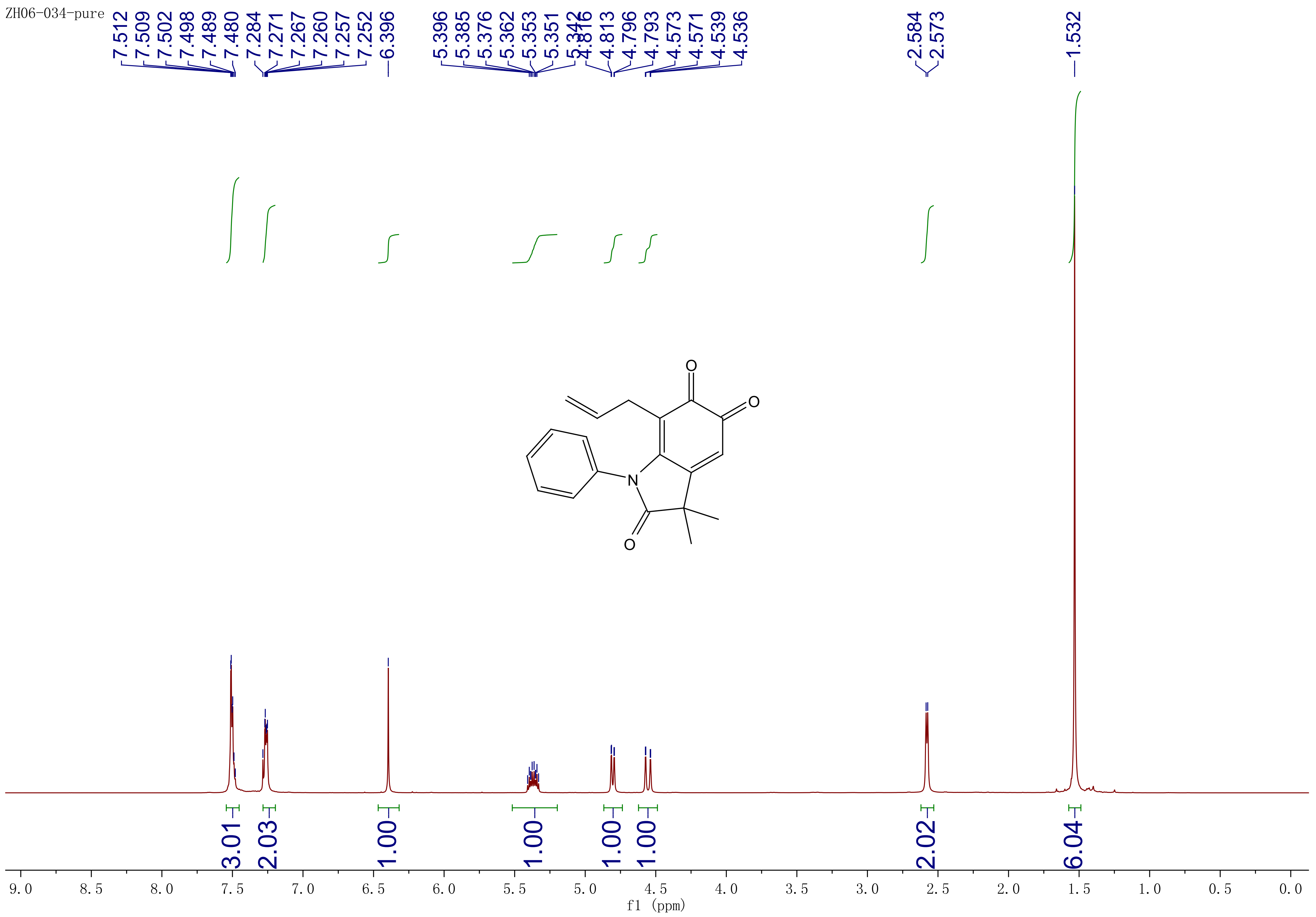
ZH06-034-pure
7.512
7.509
7.502
7.498
7.489
7.480
7.284
7.271
7.267
7.260
7.257
7.252
6.396
5.396
5.385
5.376
5.362
5.353
5.351
5.342
4.816
4.813
4.796
4.793
4.573
4.571
4.539
4.536
2.584
2.573
1.532
O
O
N
O
3.01
2.03
1.00
1.00
1.00
1.00
2.02
6.04
9.0
8.5
8.0
7.5
7.0
6.5
6.0
5.5
5.0
4.5
4.0
3.5
3.0
2.5
2.0
1.5
1.0
0.5
0.0
f1 (ppm)

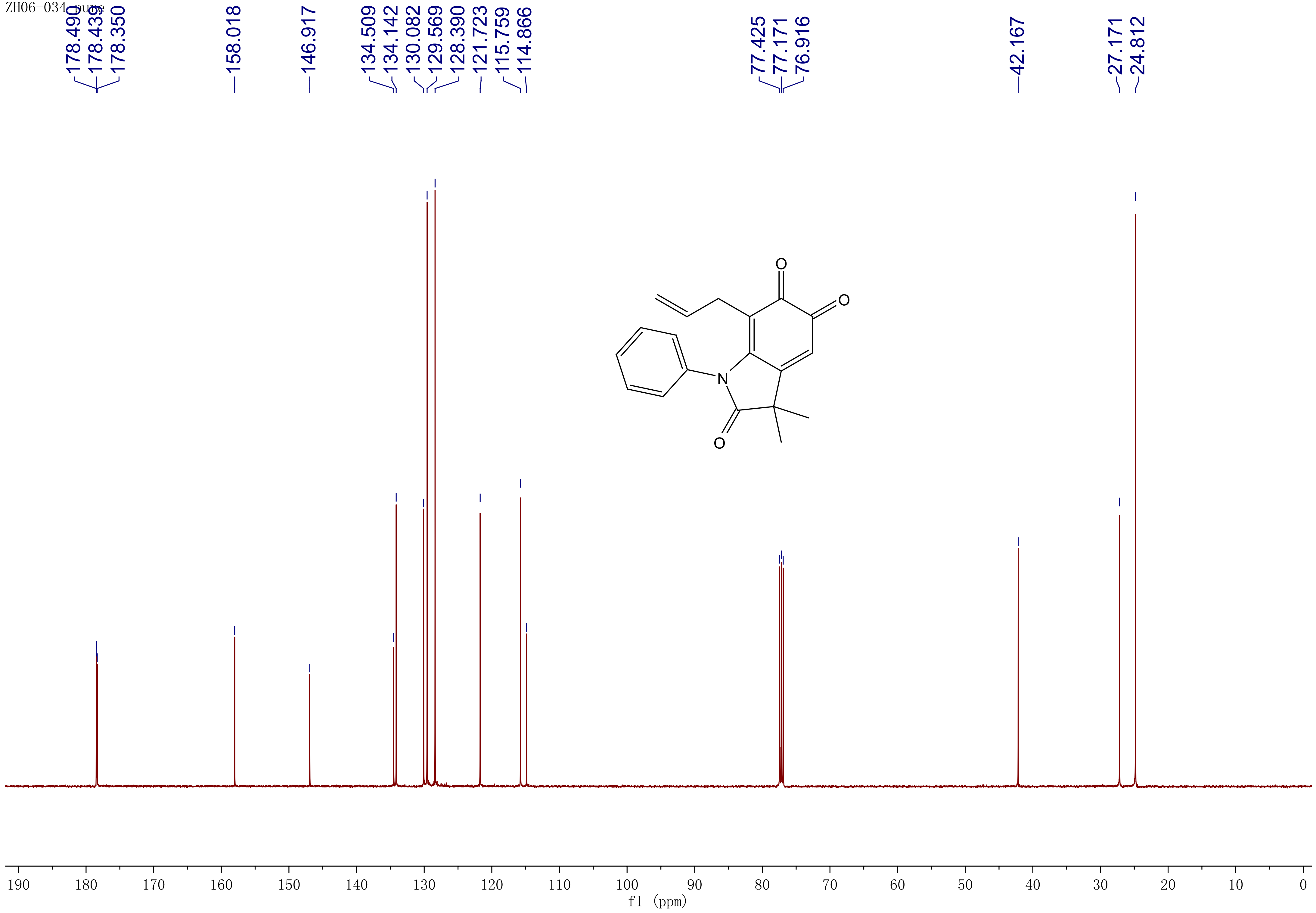
ZH06-034-pure
178.490
178.439
178.350
158.018
146.917
134.509
134.142
130.082
129.569
128.390
121.723
115.759
114.866
77.425
77.171
76.916
42.167
27.171
24.812
O
O
N
O
190 180 170 160 150 140 130 120 110 100 90 80 70 60 50 40 30 20 10 0
f1 (ppm)

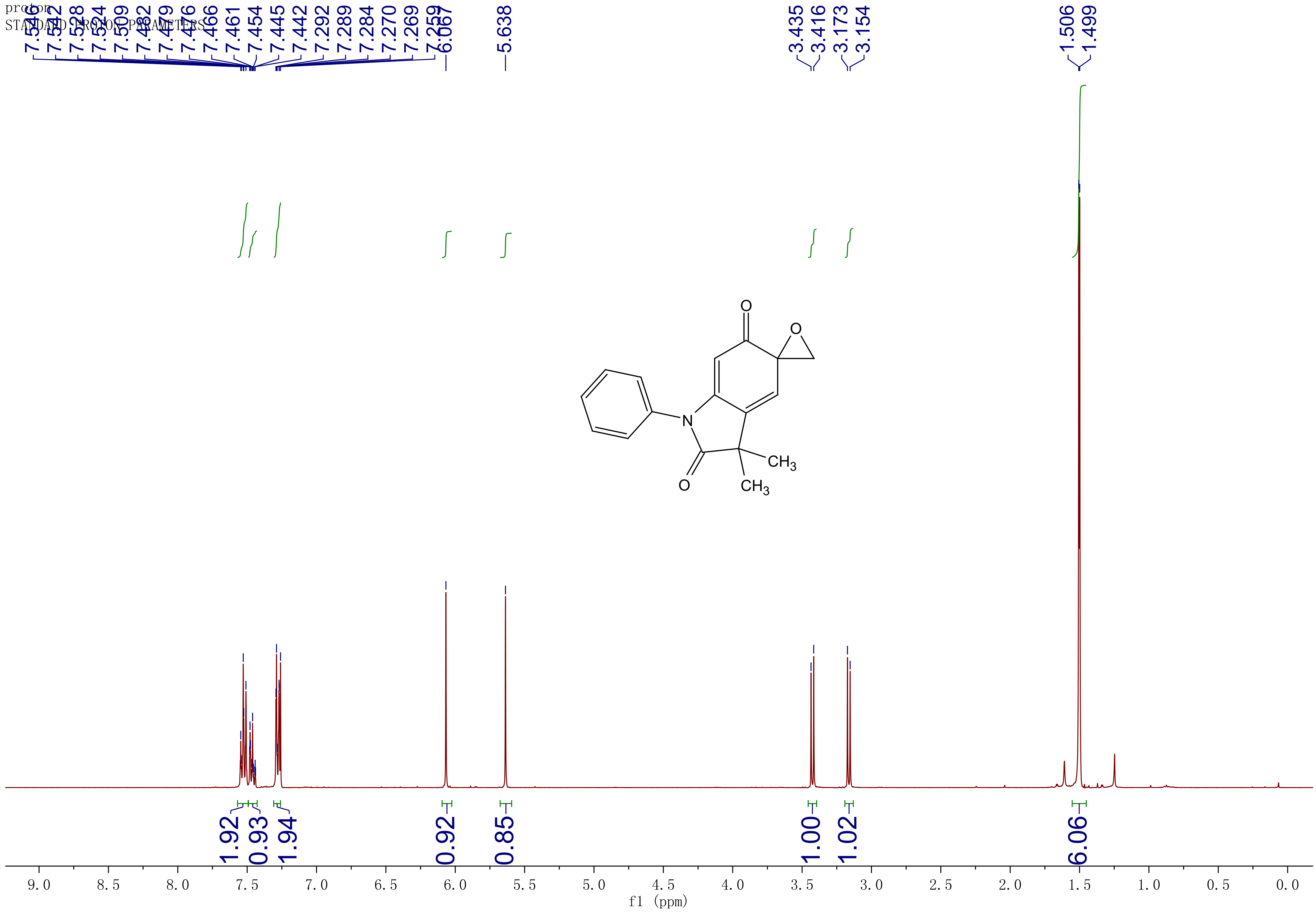

proton
STANDARD PROTON PARAMETERS
7.546
7.542
7.528
7.524
7.509
7.482
7.479
7.476
7.466
7.461
7.454
7.445
7.442
7.292
7.289
7.284
7.270
7.269
7.259
6.067
5.638
3.435
3.416
3.173
3.154
1.506
1.499
O
O
N
O
CH3
CH3
1.92
0.93
1.94
0.92
0.85
1.00
1.02
6.06
9.0
8.5
8.0
7.5
7.0
6.5
6.0
5.5
5.0
4.5
4.0
3.5
3.0
2.5
2.0
1.5
1.0
0.5
0.0
f1 (ppm)

carbon

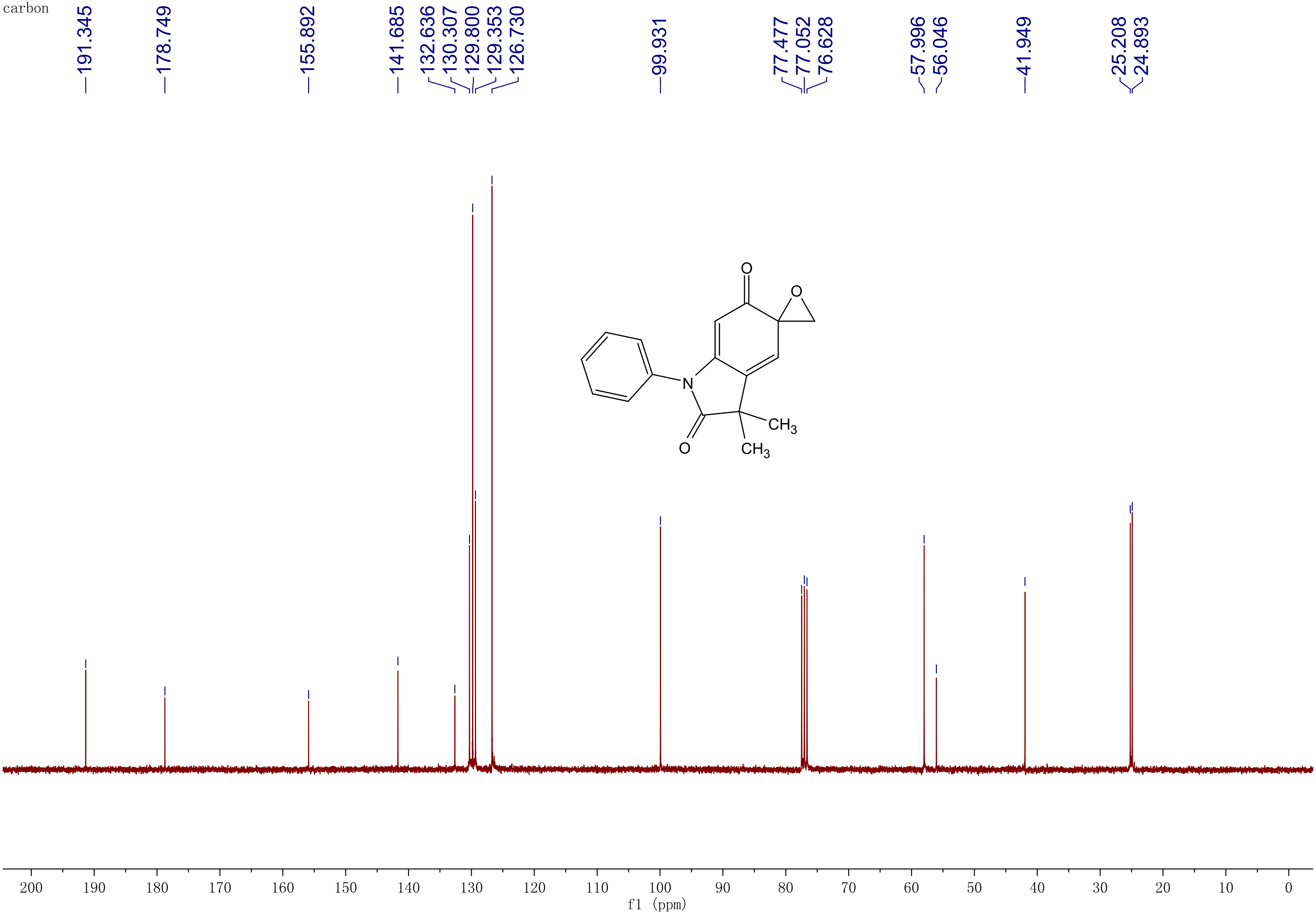
191.345
178.749
155.892
141.685
132.636
130.307
129.800
129.353
126.730
99.931
77.477
77.052
76.628
57.996
56.046
41.949
25.208
24.893
O
O
N
O
CH3
CH3
200 190 180 170 160 150 140 130 120 110 100 90 80 70 60 50 40 30 20 10 0
f1 (ppm)

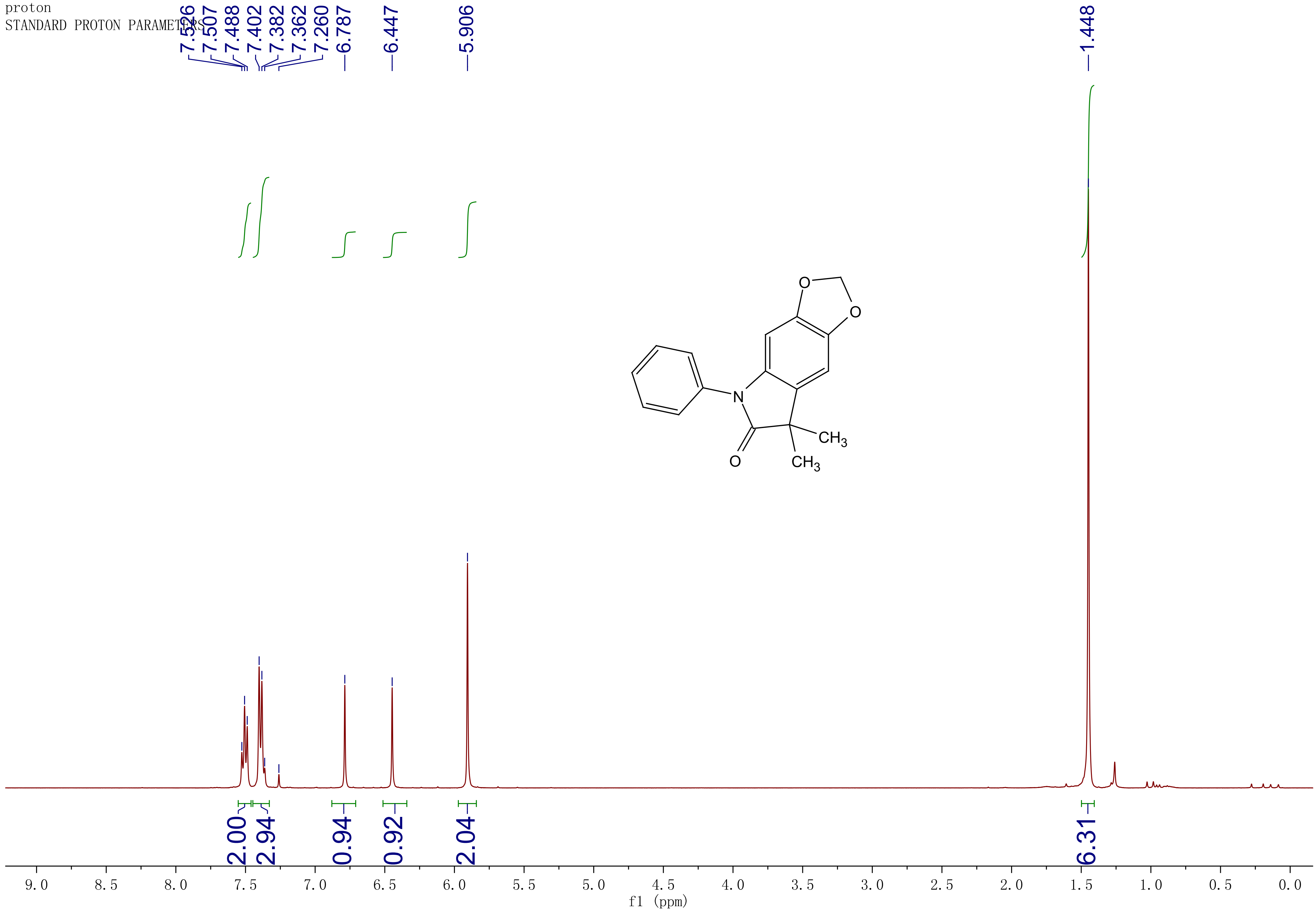
proton
STANDARD PROTON PARAMETERS
7.526
7.507
7.488
7.402
7.382
7.362
7.260
6.787
6.447
5.906
1.448
2.00
2.94
0.94
0.92
2.04
6.31
N
O
O
O
CH3
CH3
9.0
8.5
8.0
7.5
7.0
6.5
6.0
5.5
5.0
4.5
4.0
3.5
3.0
2.5
2.0
1.5
1.0
0.5
0.0
f1 (ppm)

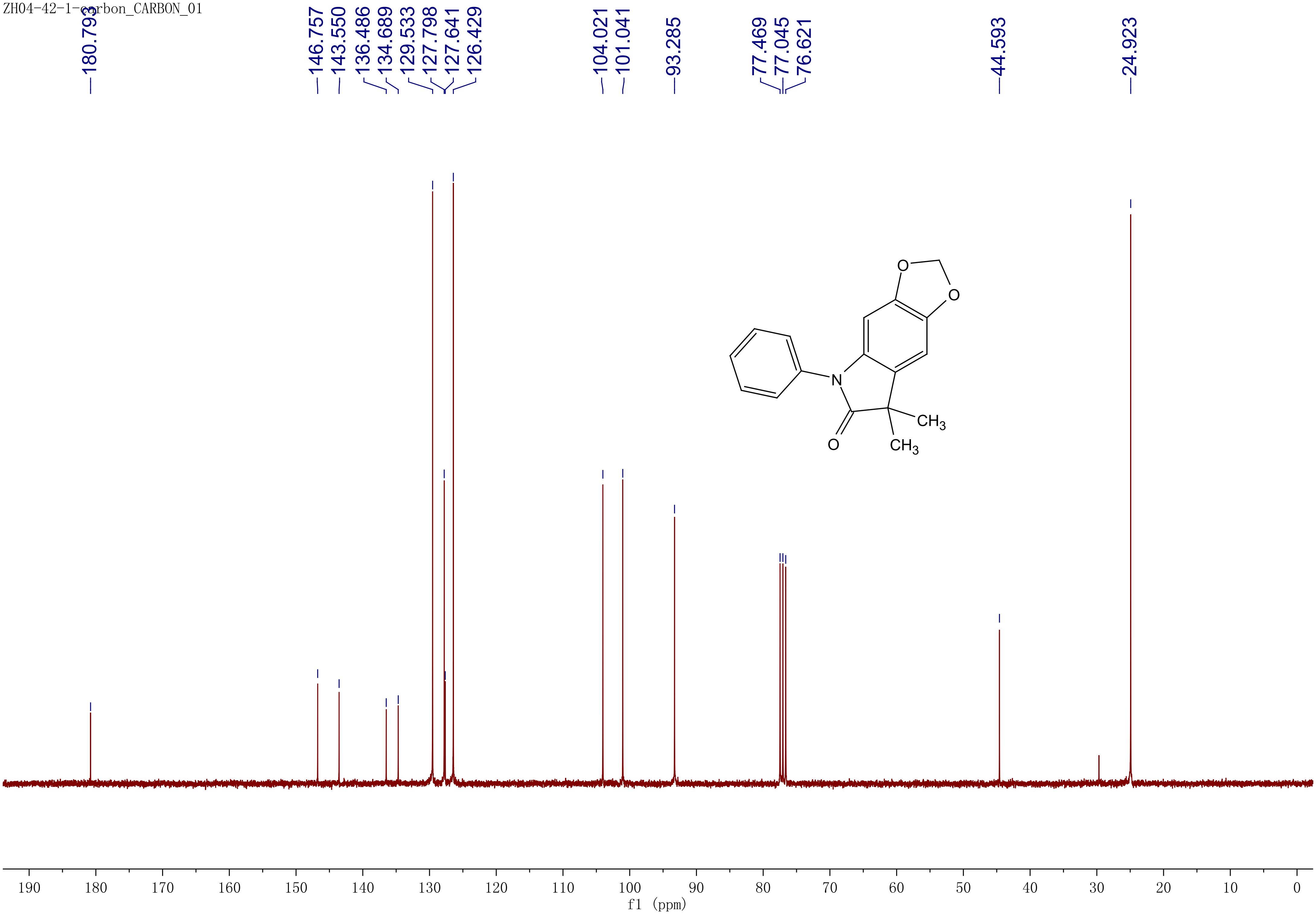
ZH04-42-1-carbon_CARBON_01
180.793
146.757
143.550
136.486
134.689
129.533
127.798
127.641
126.429
104.021
101.041
93.285
77.469
77.045
76.621
44.593
24.923
O
O
N
O
CH3
CH3
190 180 170 160 150 140 130 120 110 100 90 80 70 60 50 40 30 20 10 0
f1 (ppm)

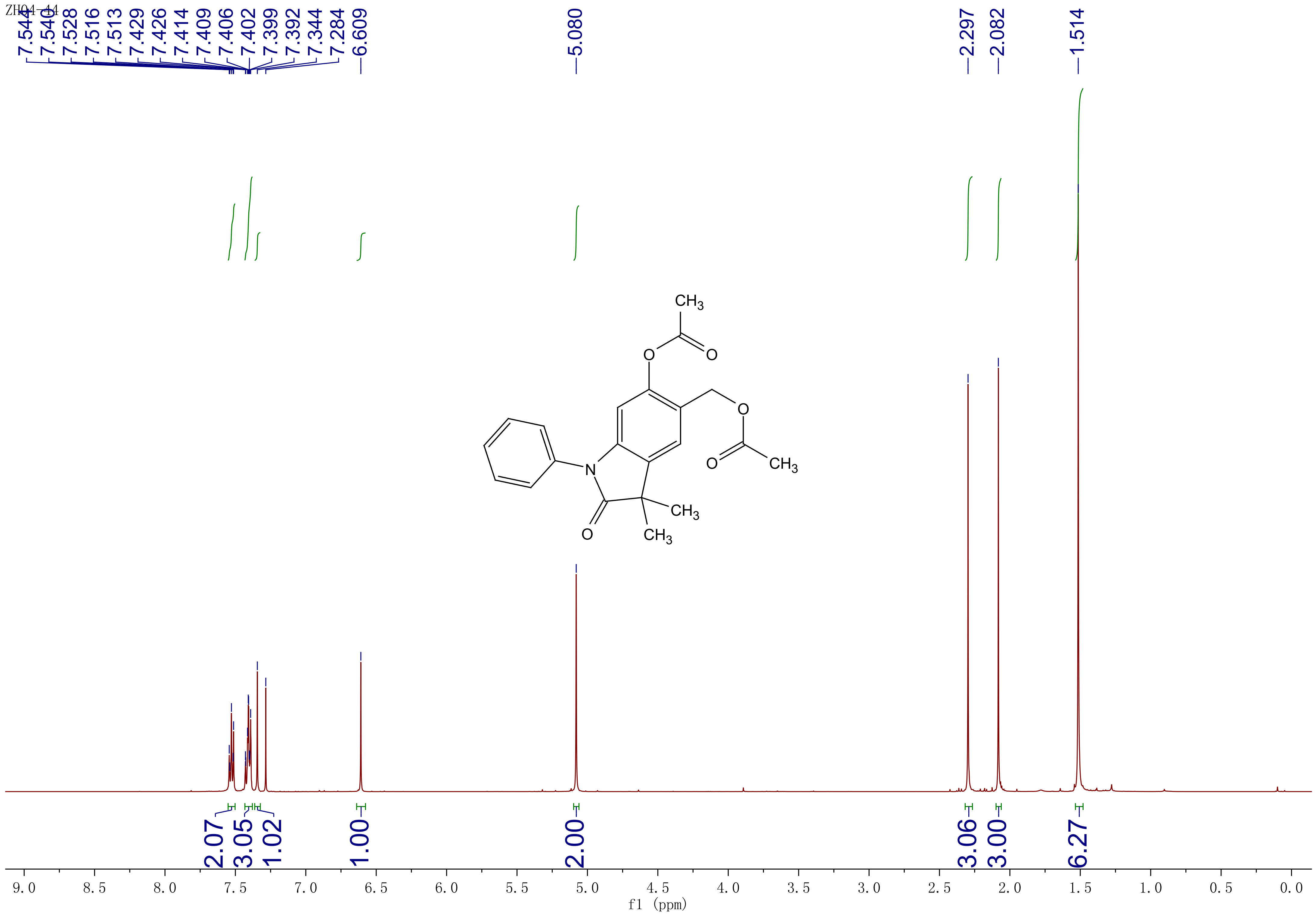
ZH04-44
7.544
7.540
7.528
7.516
7.513
7.429
7.426
7.414
7.409
7.406
7.402
7.399
7.392
7.344
7.284
6.609
5.080
2.297
2.082
1.514
CH3
O
O
O
O
CH3
N
CH3
CH3
O
2.07
3.05
1.02
1.00
2.00
3.06
3.00
6.27
9.0
8.5
8.0
7.5
7.0
6.5
6.0
5.5
5.0
4.5
4.0
3.5
3.0
2.5
2.0
1.5
1.0
0.5
0.0
f1 (ppm)

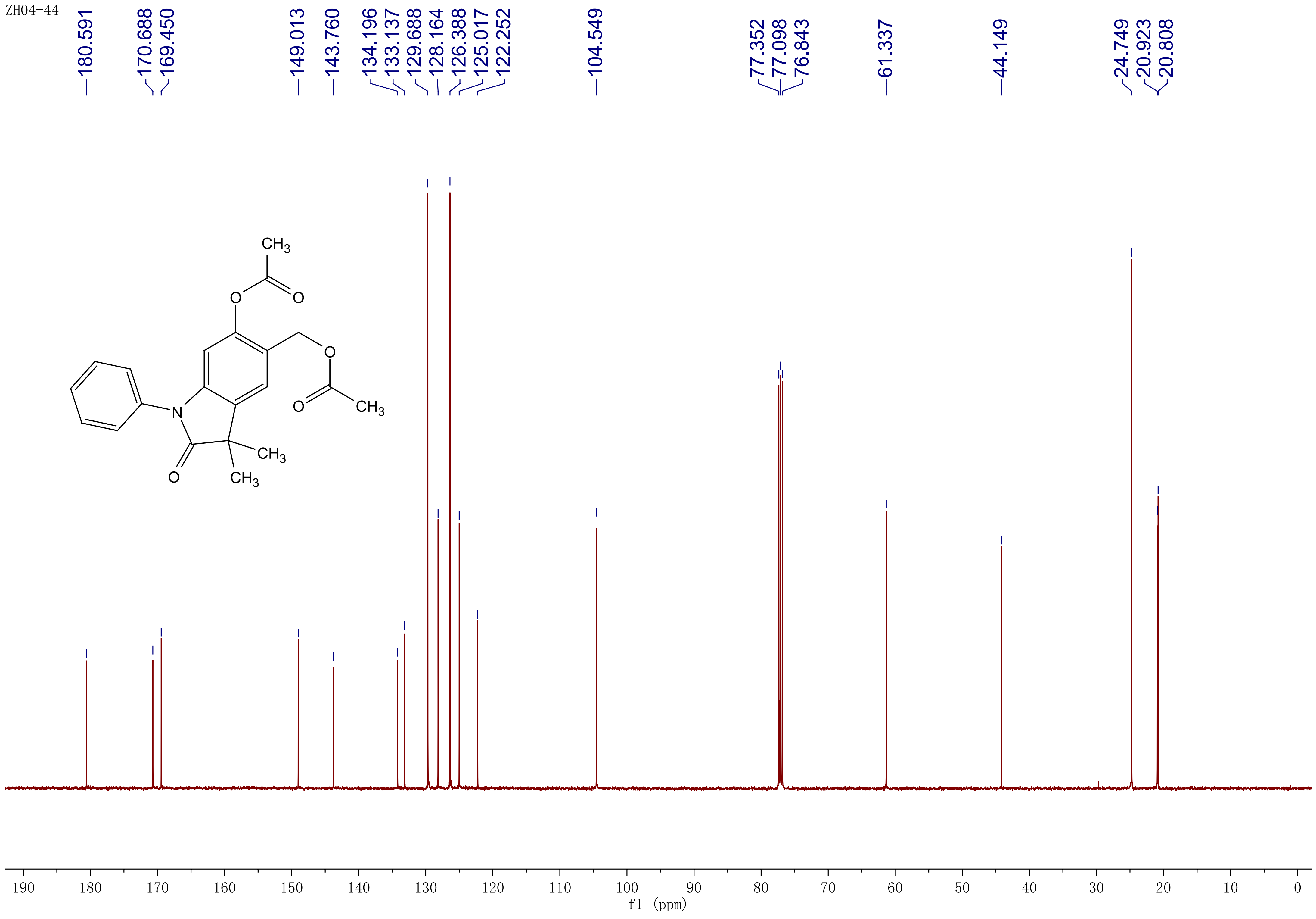
ZH04-44
180.591
170.688
169.450
149.013
143.760
134.196
133.137
129.688
128.164
126.388
125.017
122.252
104.549
77.352
77.098
76.843
61.337
44.149
24.749
20.923
20.808
CH3
O
O
O
O
N
O
CH3
CH3
CH3
190 180 170 160 150 140 130 120 110 100 90 80 70 60 50 40 30 20 10 0
f1 (ppm)

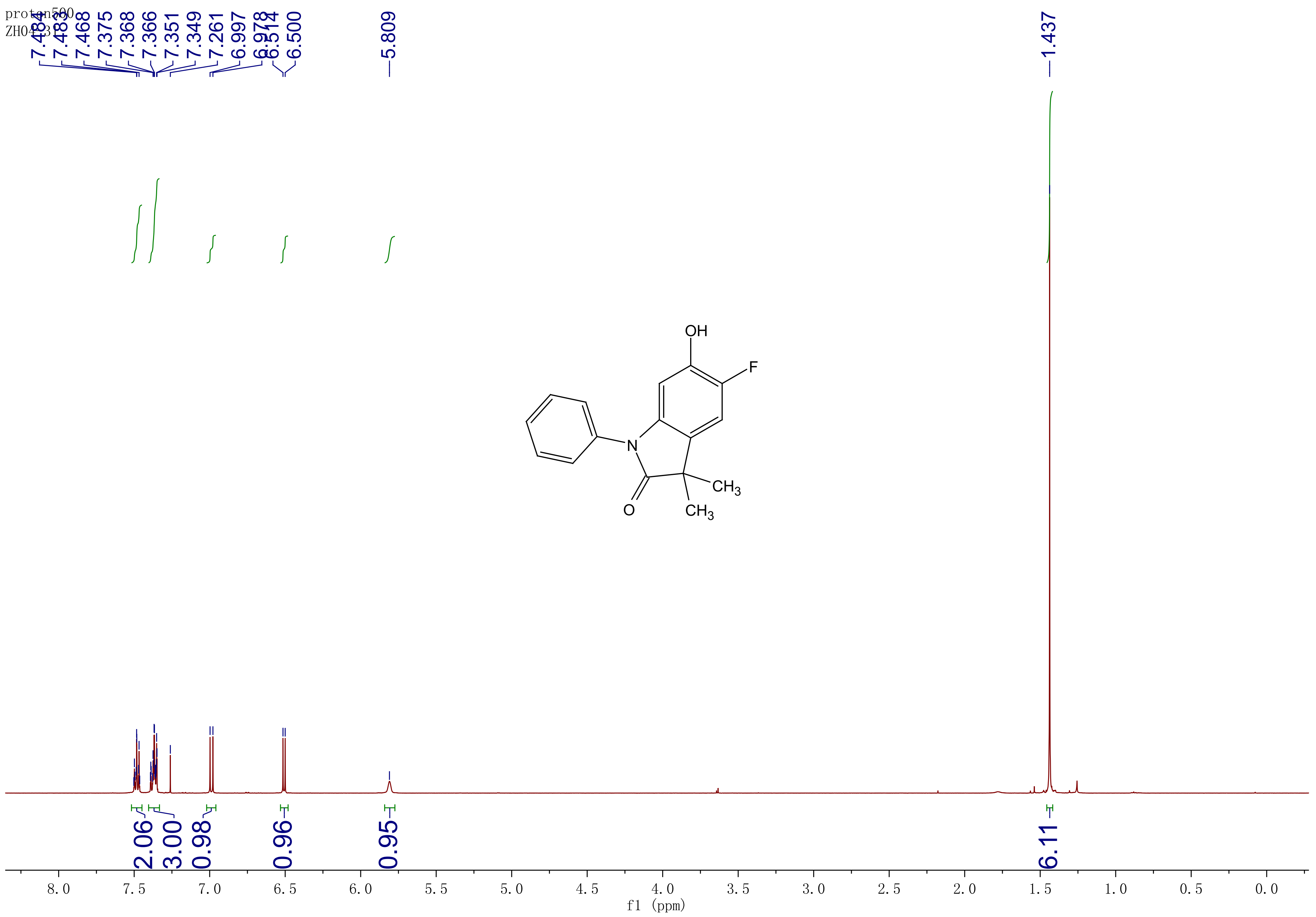

proton500
7.484
7.483
7.468
7.375
7.368
7.366
7.351
7.349
7.261
6.997
6.978
6.514
6.500
5.809
1.437
OH
F
N
O
CH3
CH3
2.06
3.00
0.98
0.96
0.95
6.11
8.0
7.5
7.0
6.5
6.0
5.5
5.0
4.5
4.0
3.5
3.0
2.5
2.0
1.5
1.0
0.5
0.0
f1 (ppm)

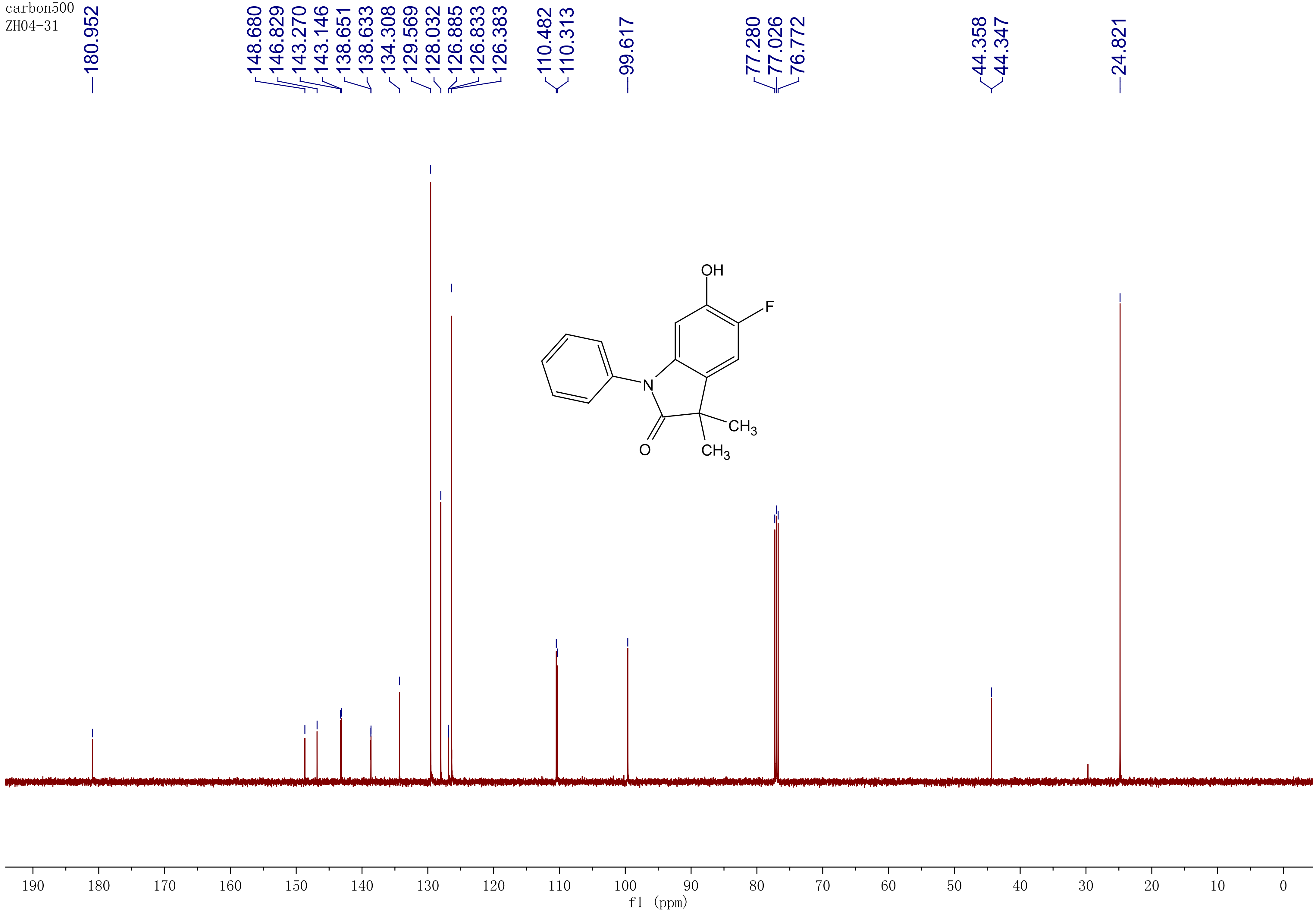
carbon500
ZH04-31
180.952
148.680
146.829
143.270
143.146
138.651
138.633
134.308
129.569
128.032
126.885
126.833
126.383
110.482
110.313
99.617
77.280
77.026
76.772
44.358
44.347
24.821
OH
F
N
O
CH3
CH3
190 180 170 160 150 140 130 120 110 100 90 80 70 60 50 40 30 20 10 0
f1 (ppm)

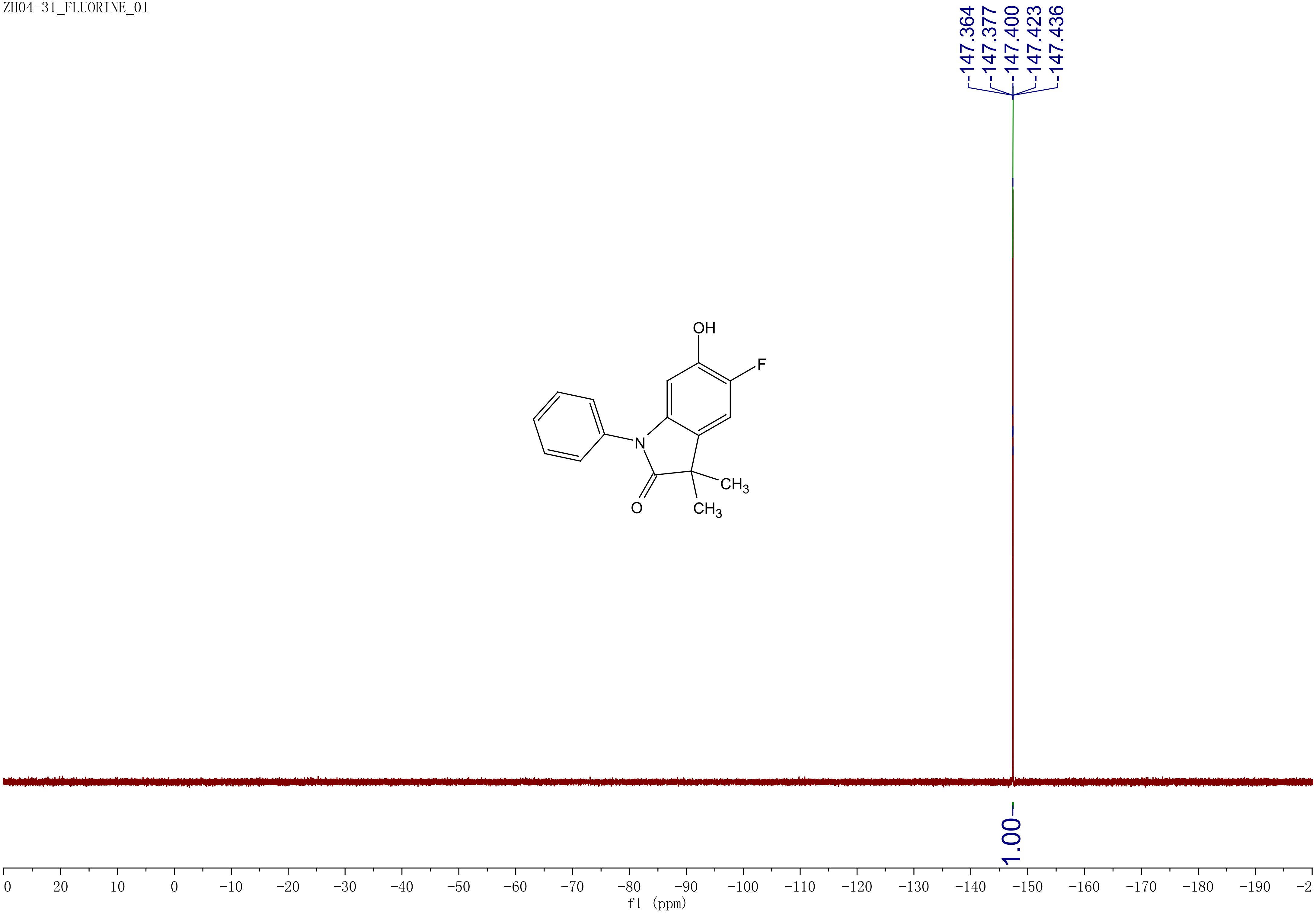
ZH04-31_FLUORINE_01
-147.364
-147.377
-147.400
-147.423
-147.436
OH
F
N
O
CH3
CH3
1.00
f1 (ppm)

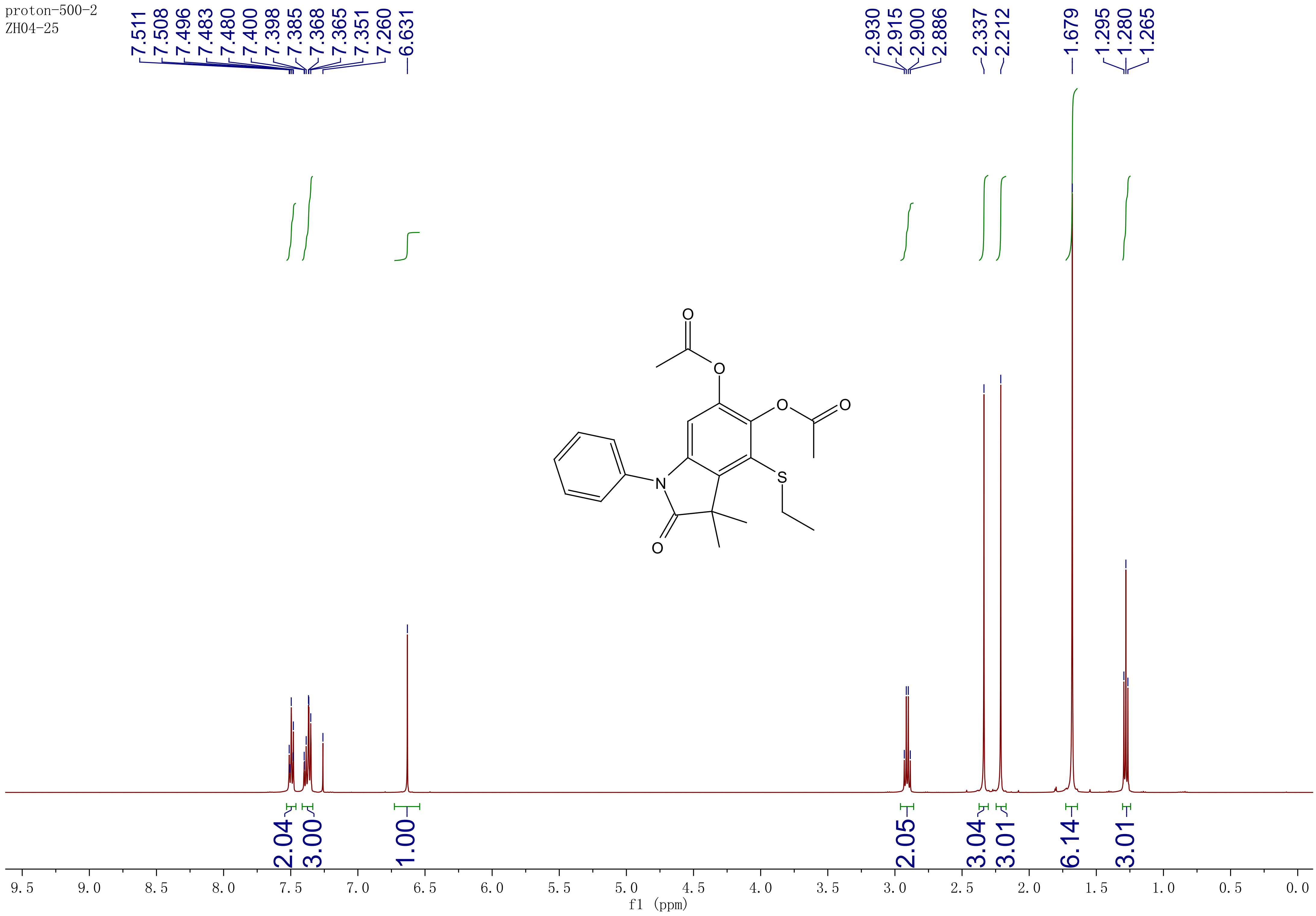
proton-500-2
ZH04-25
7.511
7.508
7.496
7.483
7.480
7.400
7.398
7.385
7.368
7.365
7.351
7.260
6.631
2.930
2.915
2.900
2.886
2.337
2.212
1.679
1.295
1.280
1.265
2.04
3.00
1.00
2.05
3.04
3.01
6.14
3.01
O
N
S
9.5
9.0
8.5
8.0
7.5
7.0
6.5
6.0
5.5
5.0
4.5
4.0
3.5
3.0
2.5
2.0
1.5
1.0
0.5
0.0
f1 (ppm)

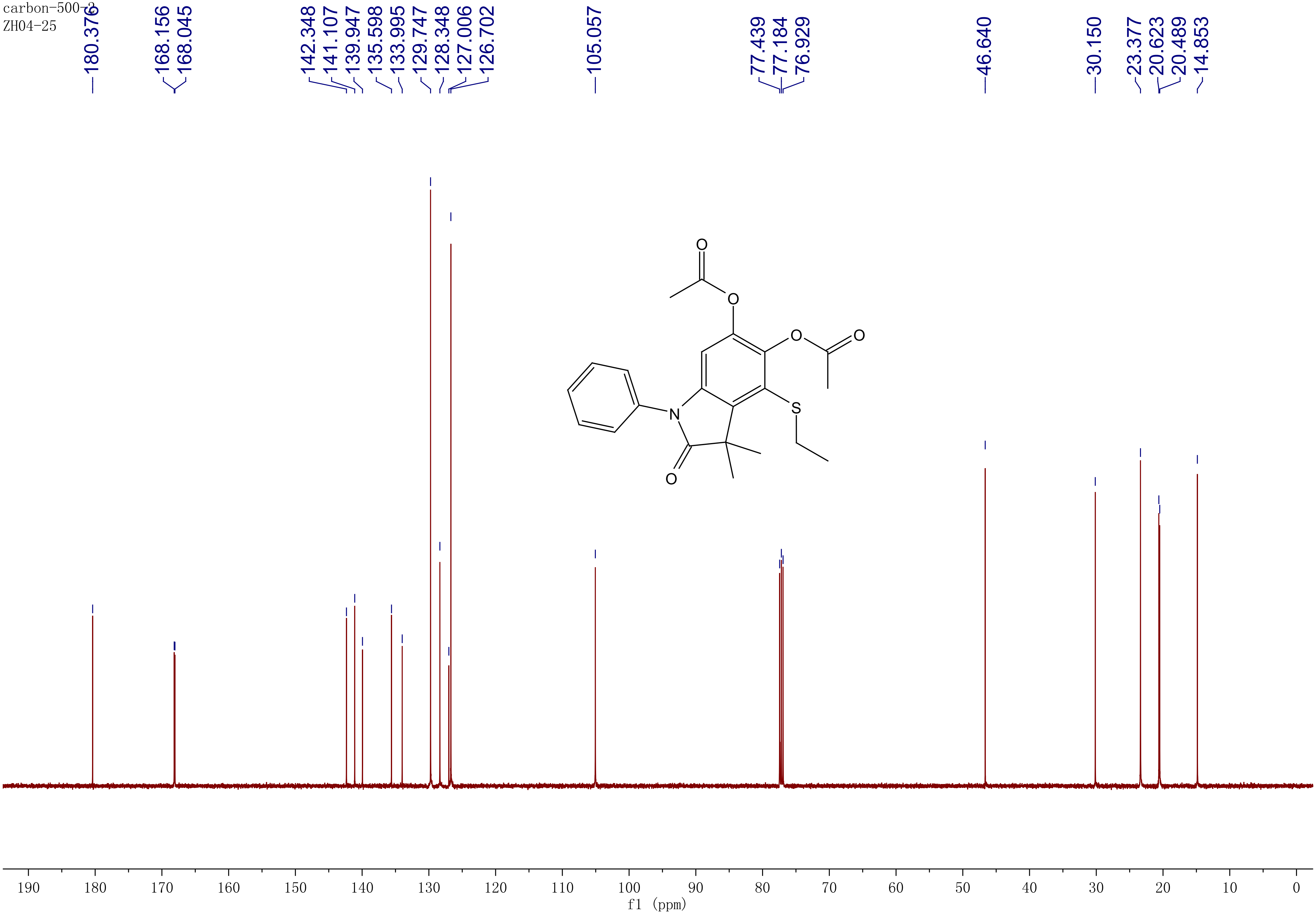
carbon-500-2
ZH04-25
180.376
168.156
168.045
142.348
141.107
139.947
135.598
133.995
129.747
128.348
127.006
126.702
105.057
77.439
77.184
76.929
46.640
30.150
23.377
20.623
20.489
14.853
O
O
O
O
N
S
O
190
180
170
160
150
140
130
120
110
100
90
80
70
60
50
40
30
20
10
0
f1 (ppm)

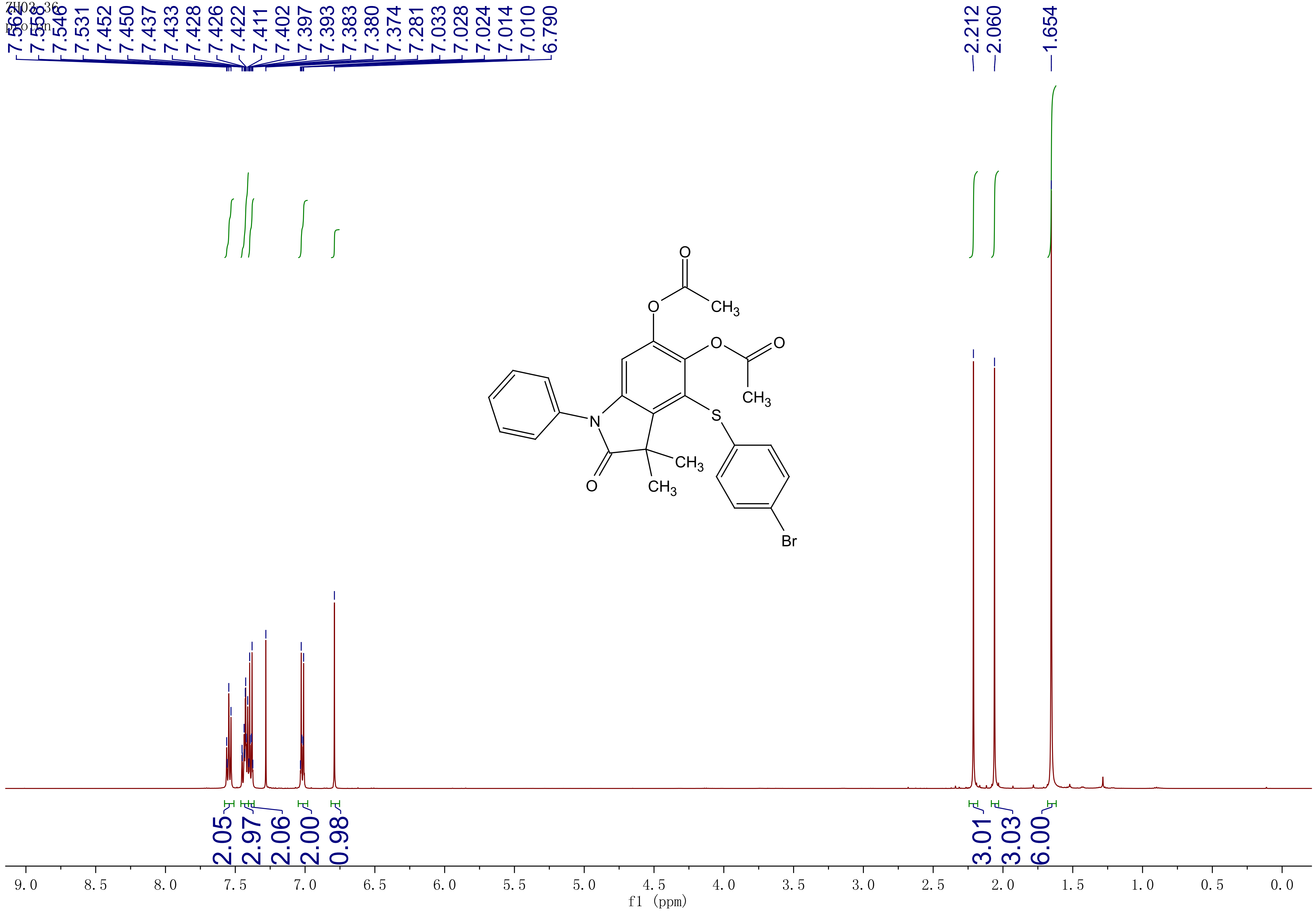

ZH02-36
proton
7.562
7.558
7.546
7.531
7.452
7.450
7.437
7.433
7.428
7.426
7.422
7.411
7.402
7.397
7.393
7.383
7.380
7.374
7.281
7.033
7.028
7.024
7.014
7.010
6.790
2.212
2.060
1.654
O
CH3
O
O
O
CH3
N
S
CH3
CH3
O
Br
2.05
2.97
2.06
2.00
0.98
3.01
3.03
6.00
9.0 8.5 8.0 7.5 7.0 6.5 6.0 5.5 5.0 4.5 4.0 3.5 3.0 2.5 2.0 1.5 1.0 0.5 0.0
f1 (ppm)

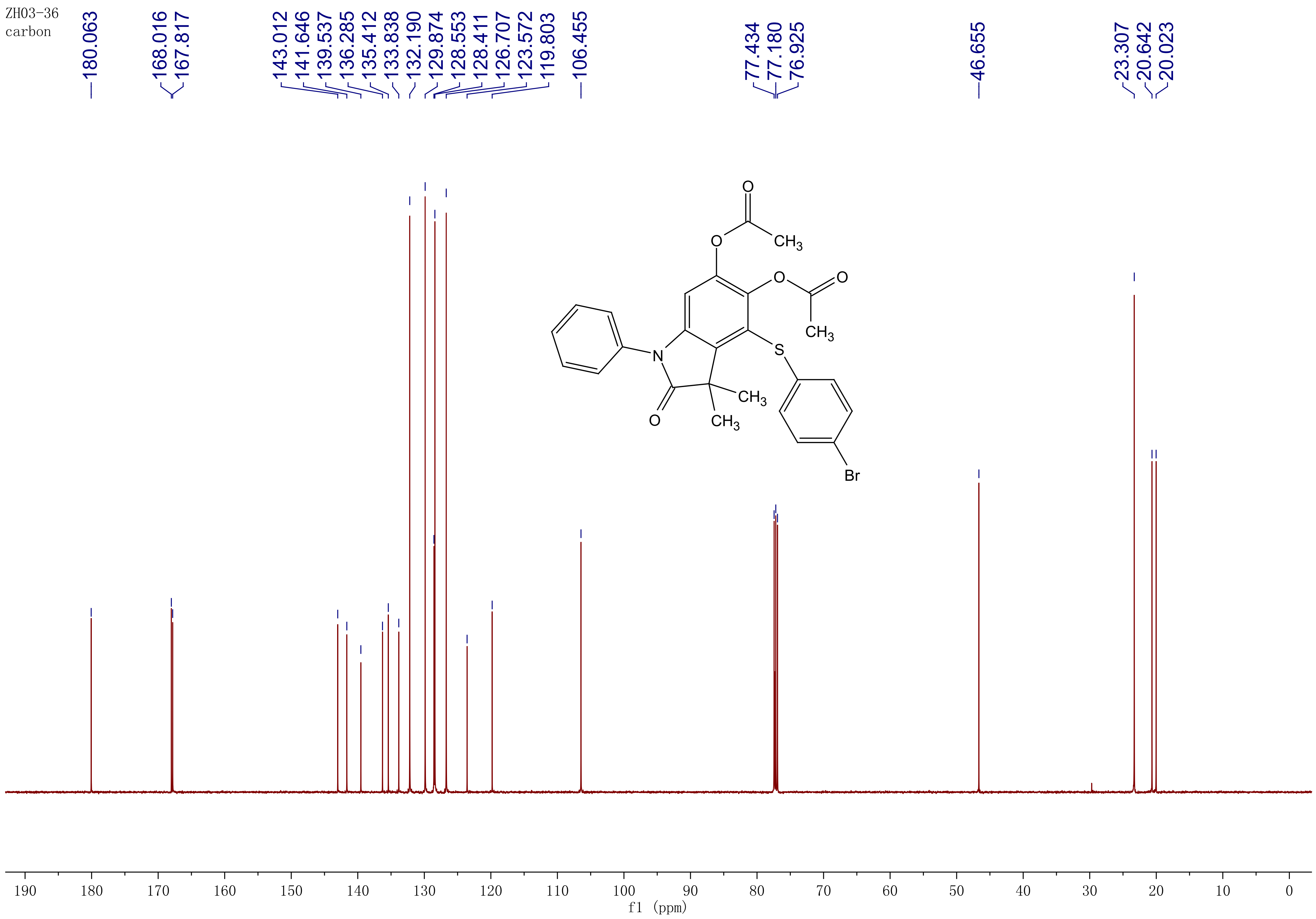
ZH03-36
carbon
180.063
168.016
167.817
143.012
141.646
139.537
136.285
135.412
133.838
132.190
129.874
128.553
128.411
126.707
123.572
119.803
106.455
77.434
77.180
76.925
46.655
23.307
20.642
20.023
O
O
CH3
O
O
CH3
N
S
CH3
CH3
O
Br
190 180 170 160 150 140 130 120 110 100 90 80 70 60 50 40 30 20 10 0
f1 (ppm)

ZH04-14
proton

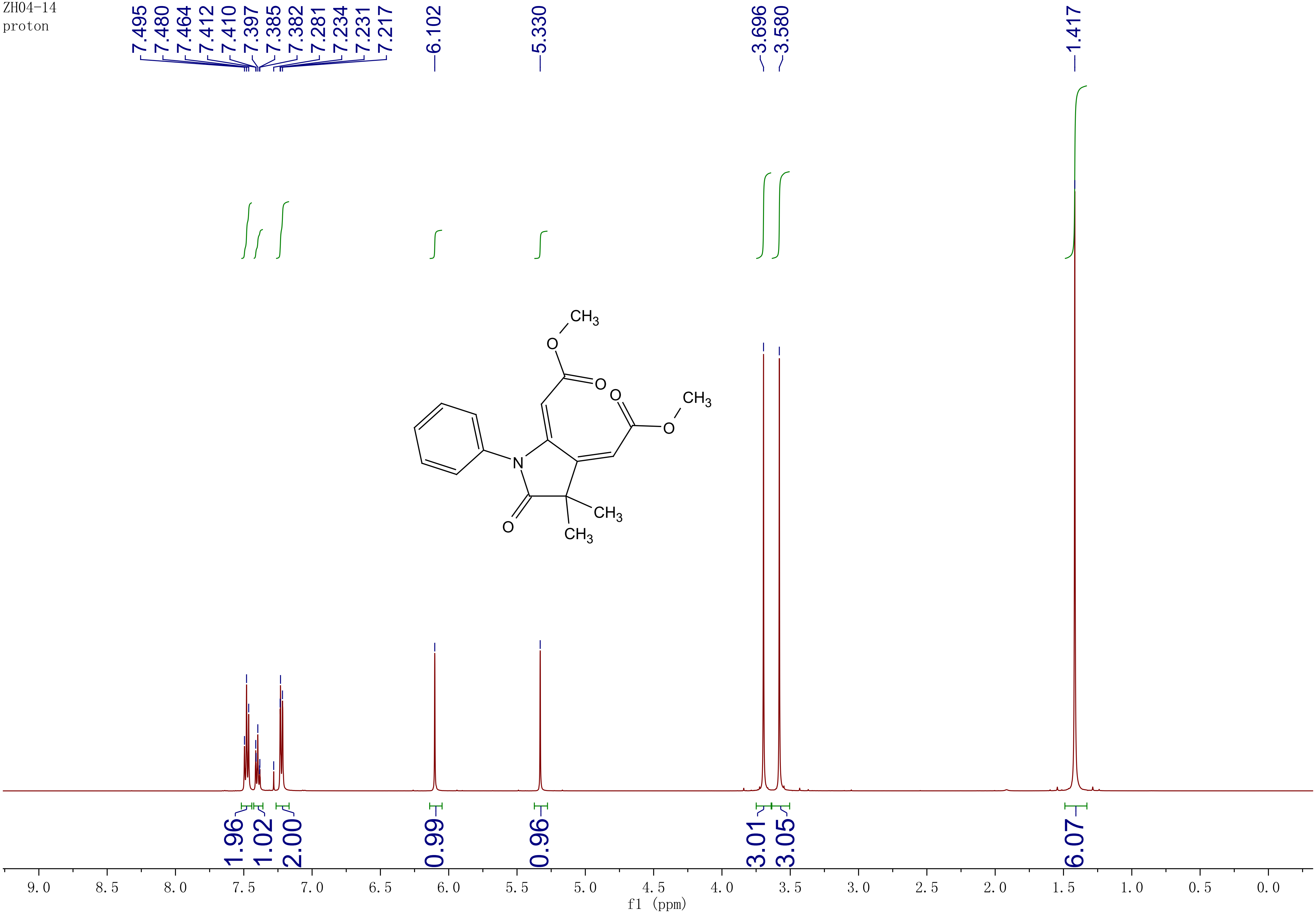

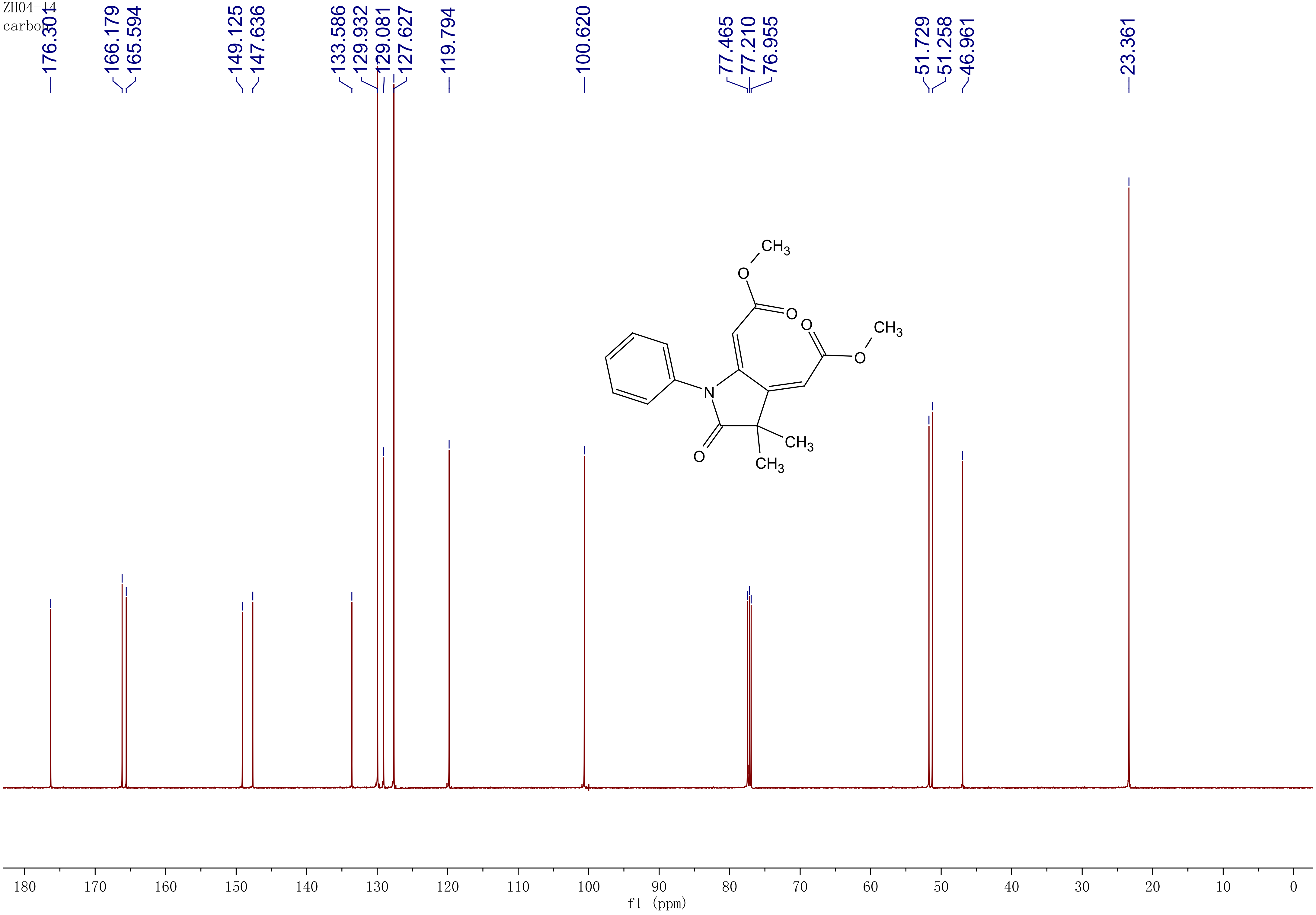

ZH04-14
carbon
176.301
166.179
165.594
149.125
147.636
133.586
129.932
129.081
127.627
119.794
100.620
77.465
77.210
76.955
51.729
51.258
46.961
23.361
CH3
O
O
O
CH3
O
N
O
CH3
CH3
180
170
160
150
140
130
120
110
100
90
80
70
60
50
40
30
20
10
0
f1 (ppm)

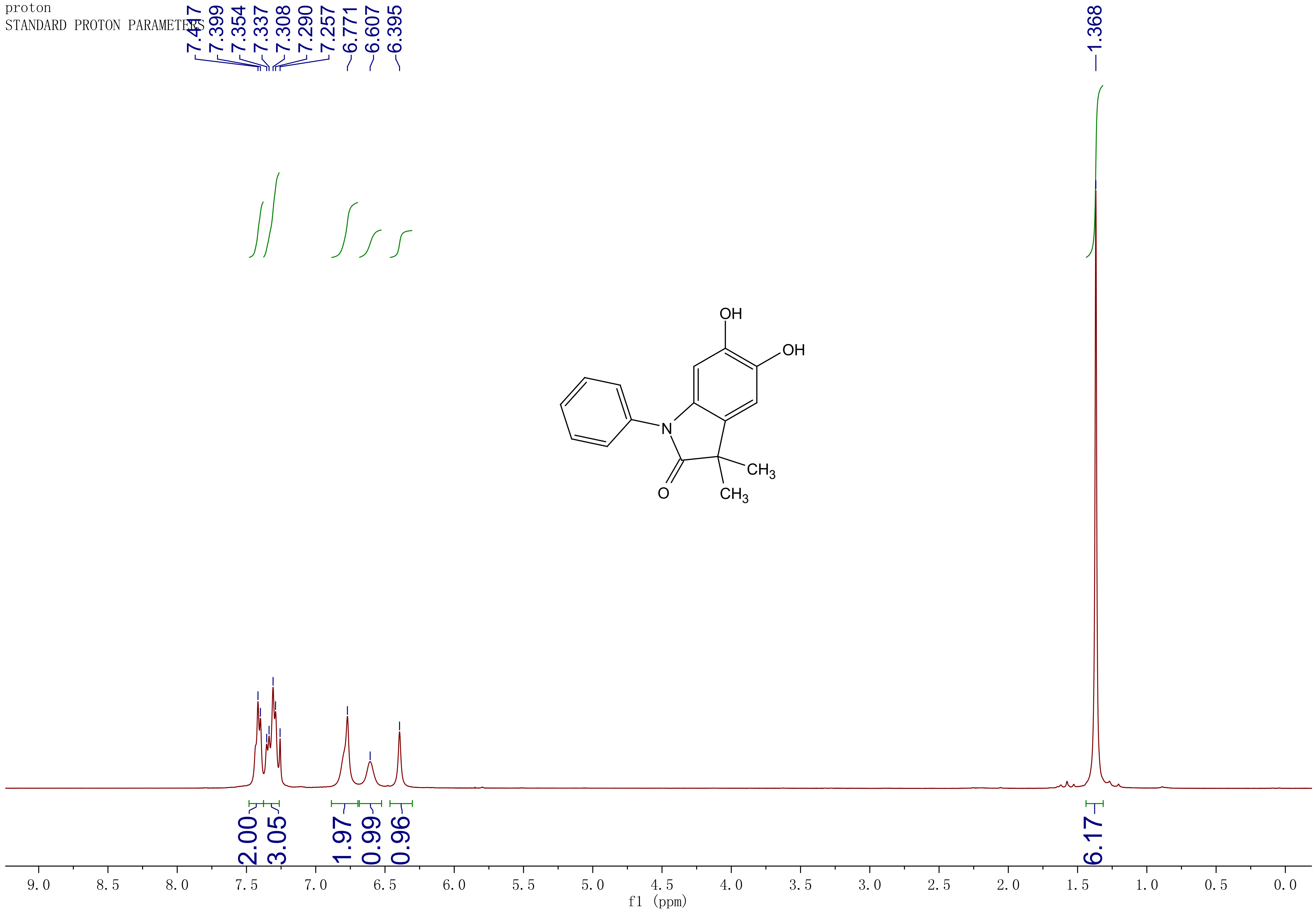
proton
STANDARD PROTON PARAMETERS
7.417
7.399
7.354
7.337
7.308
7.290
7.257
6.771
6.607
6.395
1.368
OH
OH
N
O
CH3
CH3
2.00
3.05
1.97
0.99
0.96
6.17
9.0
8.5
8.0
7.5
7.0
6.5
6.0
5.5
5.0
4.5
4.0
3.5
3.0
2.5
2.0
1.5
1.0
0.5
0.0
f1 (ppm)

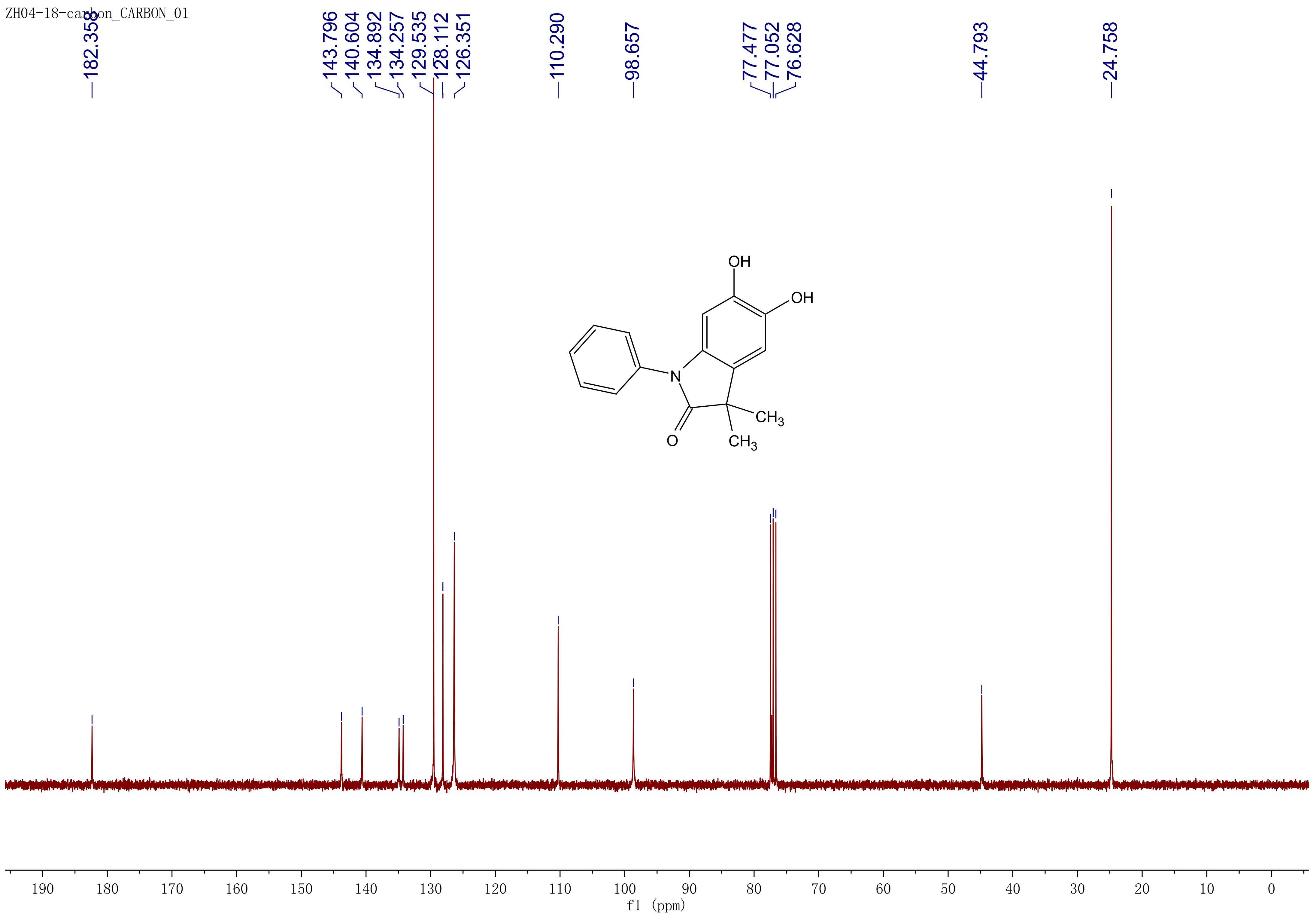

ZH04-18-carbon_CARBON_01
182.358
143.796
140.604
134.892
134.257
129.535
128.112
126.351
110.290
98.657
77.477
77.052
76.628
44.793
24.758
OH
OH
N
O
CH3
CH3
190 180 170 160 150 140 130 120 110 100 90 80 70 60 50 40 30 20 10 0
f1 (ppm)

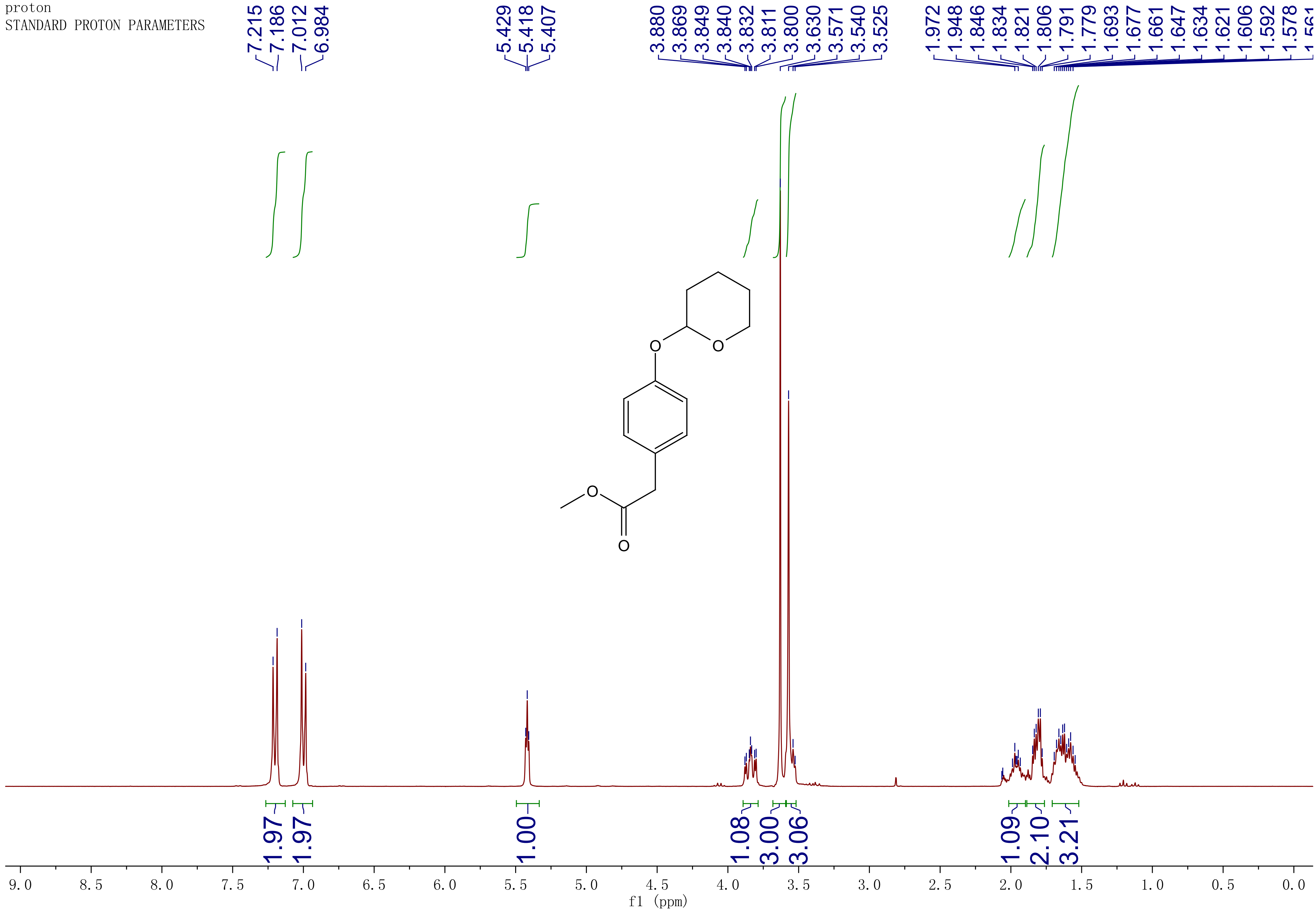
proton
STANDARD PROTON PARAMETERS
7.215
7.186
7.012
6.984
5.429
5.418
5.407
3.880
3.869
3.849
3.840
3.832
3.811
3.800
3.630
3.571
3.540
3.525
1.972
1.948
1.846
1.834
1.821
1.806
1.791
1.779
1.693
1.677
1.661
1.647
1.634
1.621
1.606
1.592
1.578
O
O
O
O
1.97
1.97
1.00
1.08
3.00
3.06
1.09
2.10
3.21
9.0
8.5
8.0
7.5
7.0
6.5
6.0
5.5
5.0
4.5
4.0
3.5
3.0
2.5
2.0
1.5
1.0
0.5
0.0
f1 (ppm)

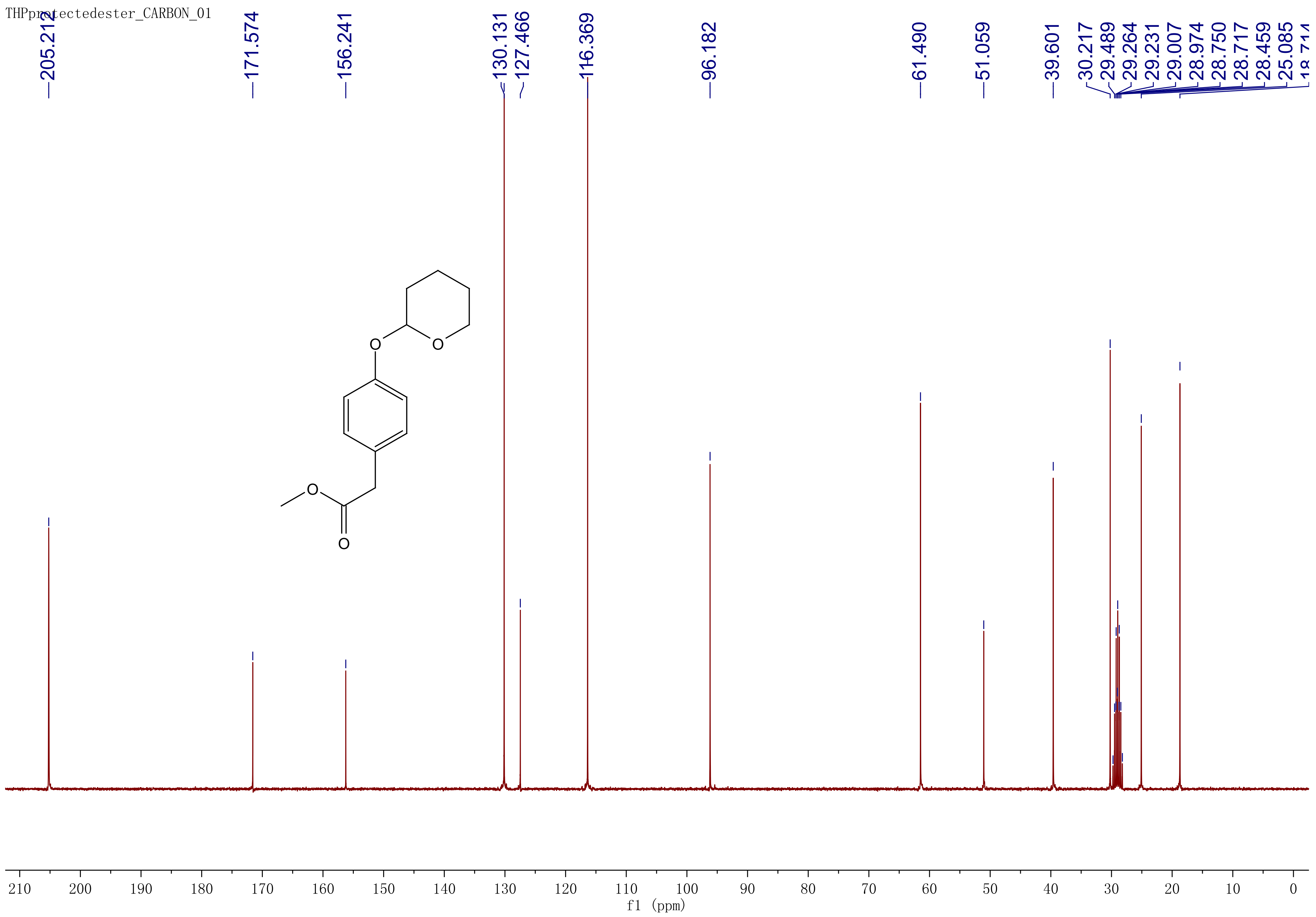
THPprotectedester_CARBON_01
205.212
171.574
156.241
130.131
127.466
116.369
96.182
61.490
51.059
39.601
30.217
29.489
29.264
29.231
29.007
28.974
28.750
28.717
28.459
25.085
O
O
O
O
210 200 190 180 170 160 150 140 130 120 110 100 90 80 70 60 50 40 30 20 10 0
f1 (ppm)

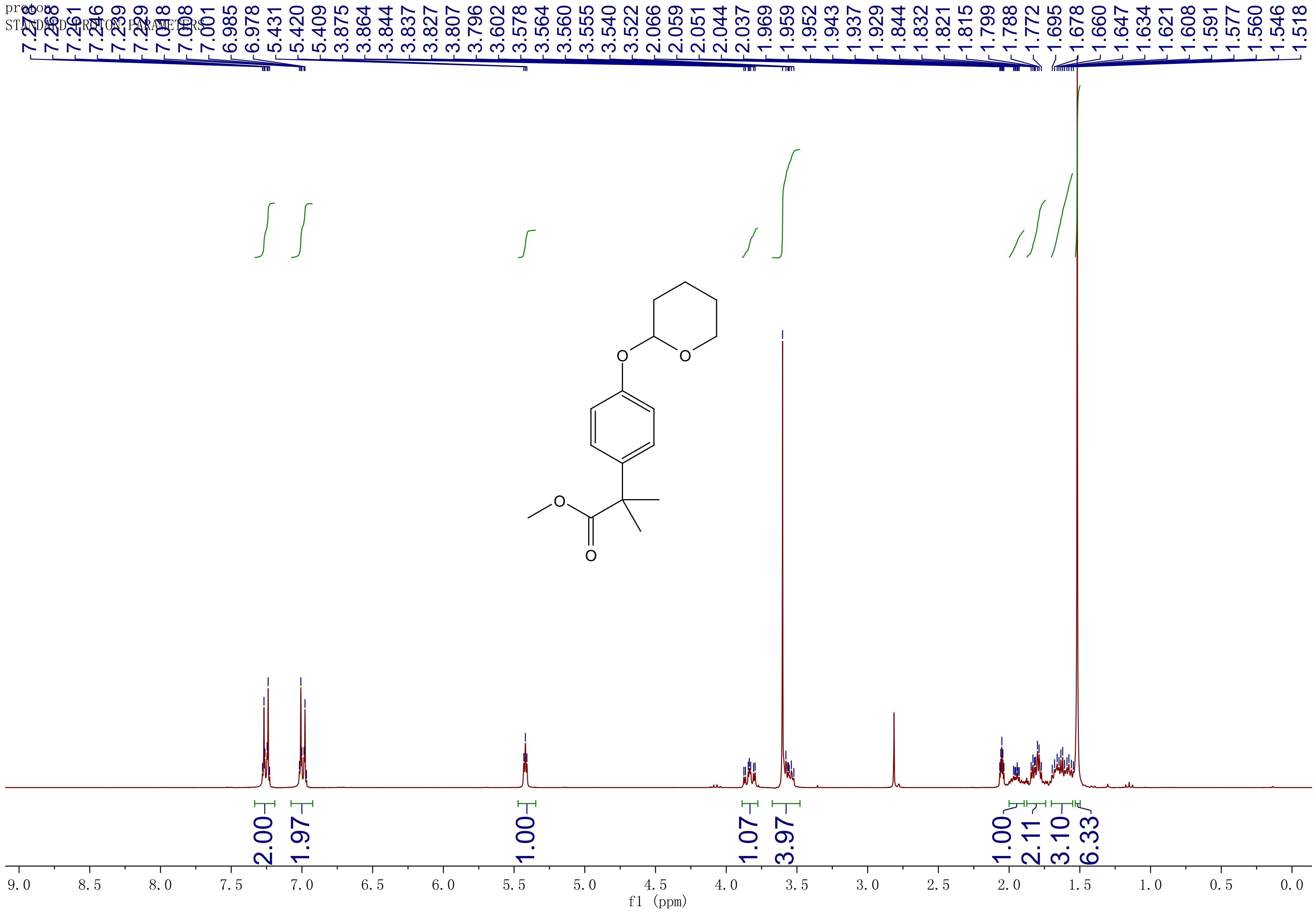

proton
STANDARD PROTON PARAMETERS
7.278
7.268
7.261
7.246
7.239
7.229
7.018
7.008
7.001
6.985
6.978
5.431
5.420
5.409
3.875
3.864
3.844
3.837
3.827
3.807
3.796
3.602
3.578
3.564
3.560
3.555
3.540
3.522
2.066
2.059
2.051
2.044
2.037
1.969
1.959
1.952
1.943
1.937
1.929
1.844
1.832
1.821
1.815
1.799
1.788
1.772
1.695
1.678
1.660
1.647
1.634
1.621
1.608
1.591
1.577
1.560
1.546
1.518
O
O
O
O
O
2.00
1.97
1.00
1.07
3.97
1.00
2.11
3.10
6.33
9.0
8.5
8.0
7.5
7.0
6.5
6.0
5.5
5.0
4.5
4.0
3.5
3.0
2.5
2.0
1.5
1.0
0.5
0.0
f1 (ppm)

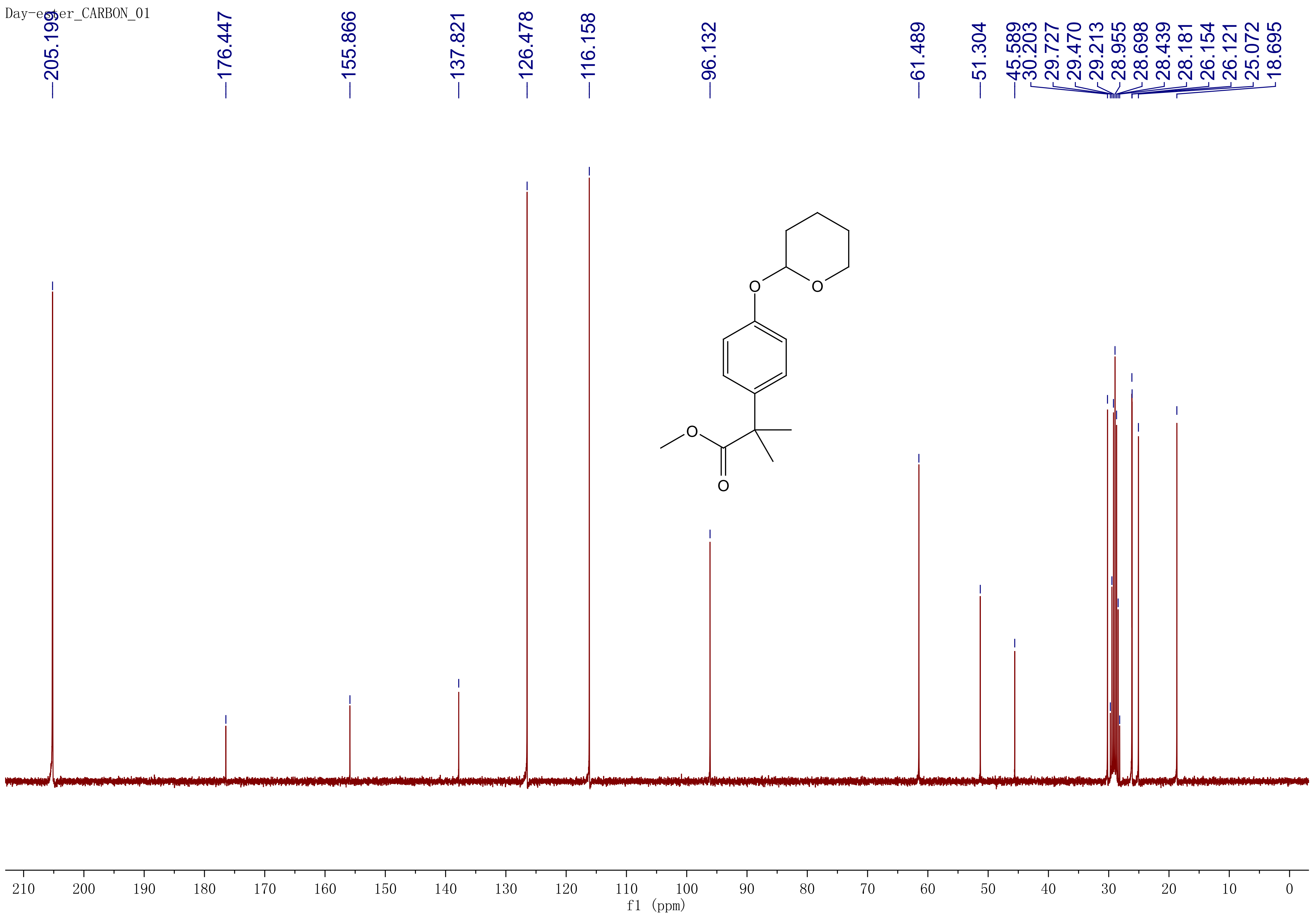
Day-ester_CARBON_01
205.199
176.447
155.866
137.821
126.478
116.158
96.132
61.489
51.304
45.589
30.203
29.727
29.470
29.213
28.955
28.698
28.439
28.181
26.154
26.121
25.072
18.695
O
O
O
O
210 200 190 180 170 160 150 140 130 120 110 100 90 80 70 60 50 40 30 20 10 0
f1 (ppm)

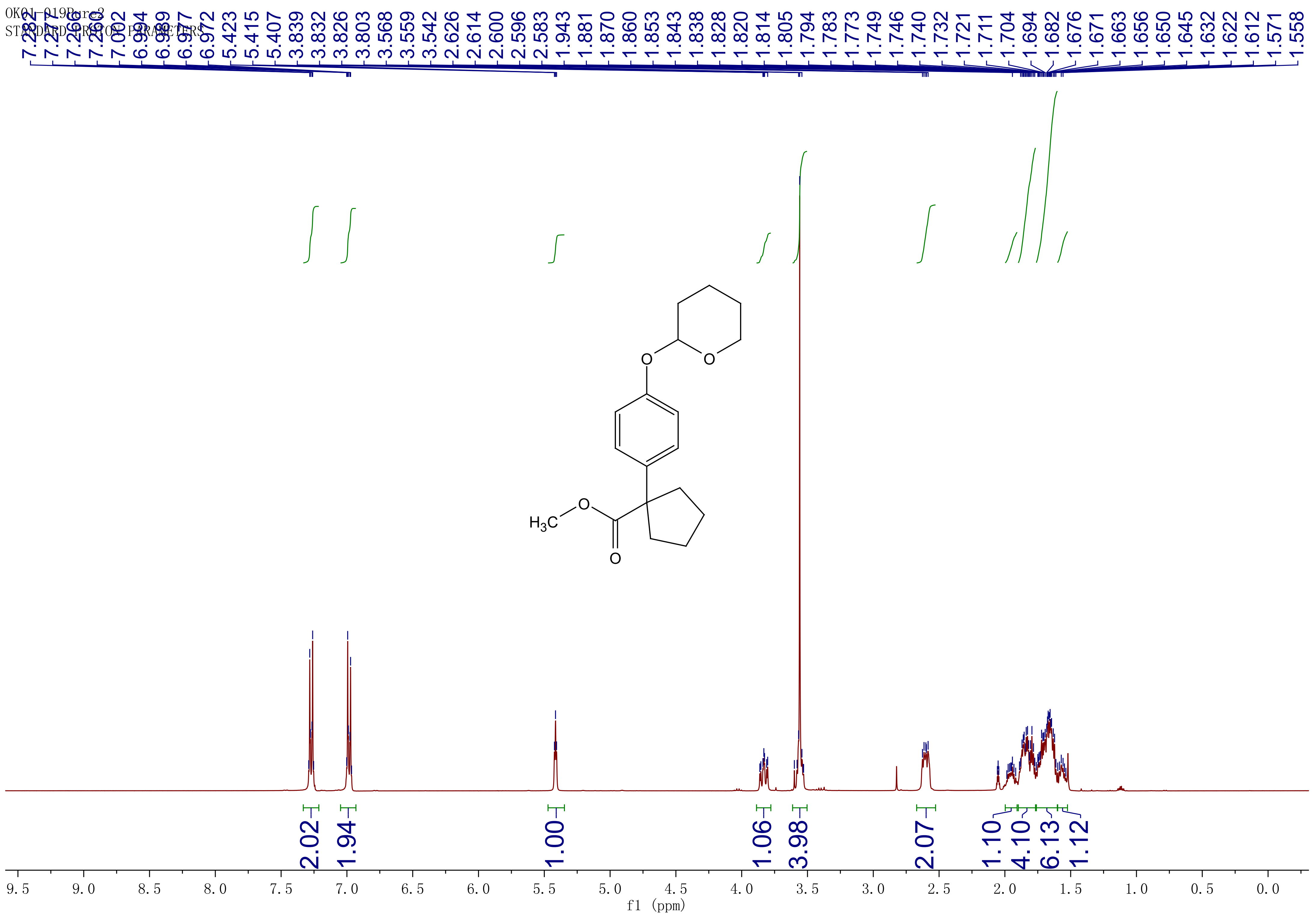
OK01-019Pure2
STANDARD PROTON PARAMETERS
H3C
O
O
O
O
2.02
1.94
1.00
1.06
3.98
2.07
1.10
4.10
6.13
1.12
f1 (ppm)

OK01-01[illegible]_CARBON_01

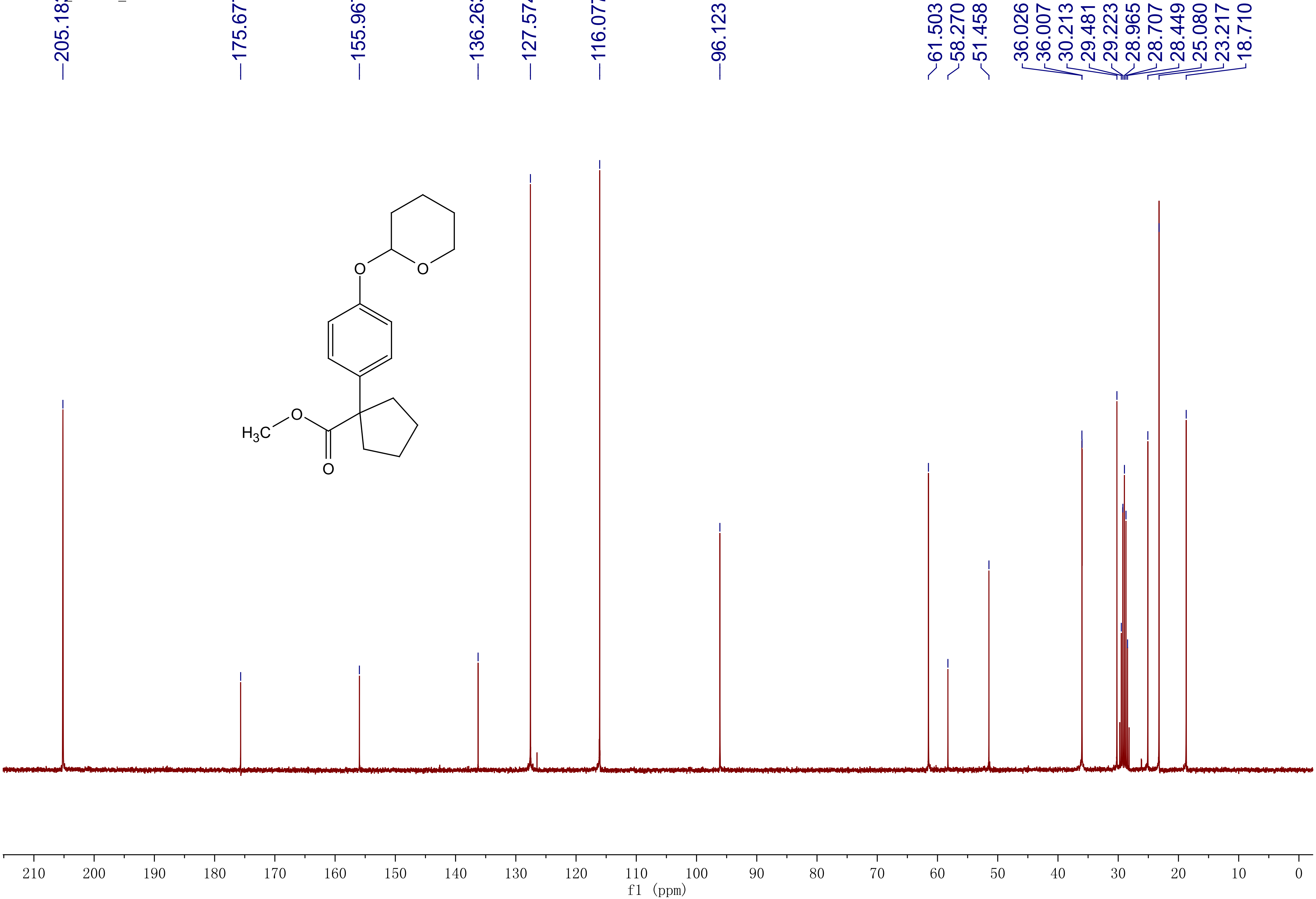

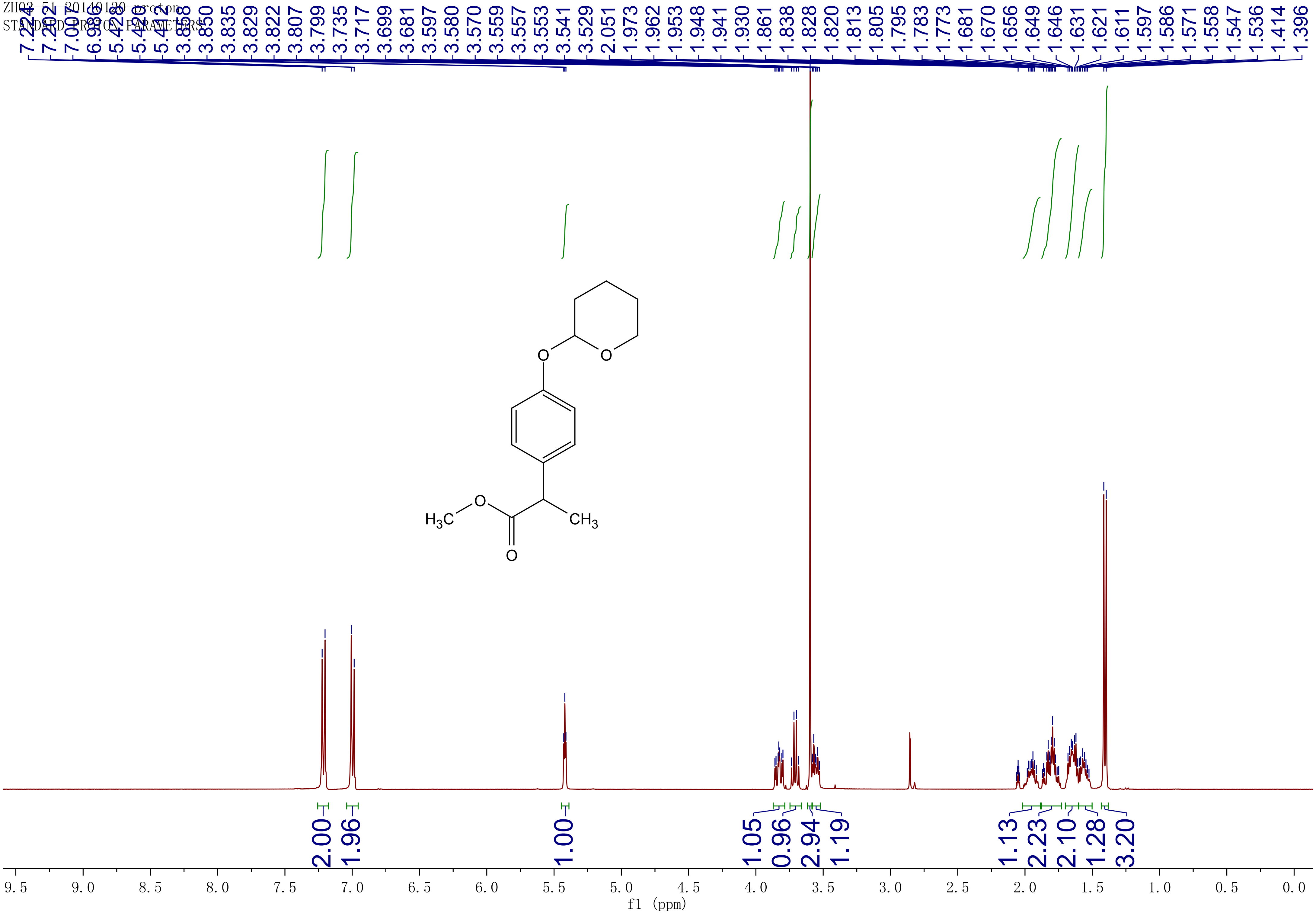
ZH02-51-20140120-proton
STANDARD PROTON PARAMETERS
3.850
3.835
3.829
3.822
3.807
3.799
3.735
3.717
3.699
3.681
3.597
3.580
3.570
3.559
3.557
3.553
3.541
3.529
2.051
1.973
1.962
1.953
1.948
1.941
1.930
1.861
1.838
1.828
1.820
1.813
1.805
1.795
1.783
1.773
1.681
1.670
1.656
1.649
1.646
1.631
1.621
1.611
1.597
1.586
1.571
1.558
1.547
1.536
1.414
1.396
O
O
O
O
H3C
CH3
O
2.00
1.96
1.00
1.05
0.96
2.94
1.19
1.13
2.23
2.10
1.28
3.20
9.5
9.0
8.5
8.0
7.5
7.0
6.5
6.0
5.5
5.0
4.5
4.0
3.5
3.0
2.5
2.0
1.5
1.0
0.5
0.0
f1 (ppm)

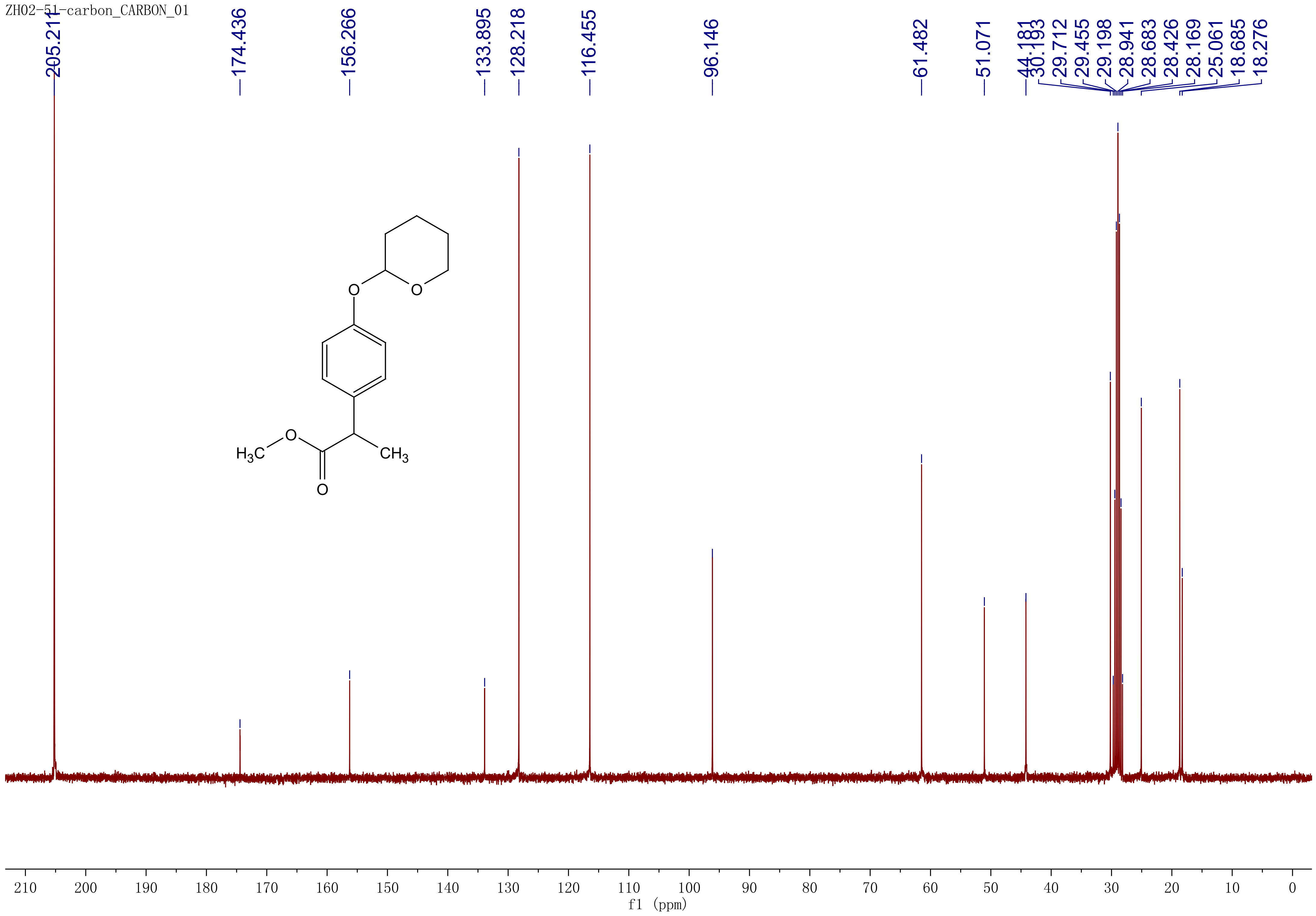
ZH02-51-carbon_CARBON_01
205.211
174.436
156.266
133.895
128.218
116.455
96.146
61.482
51.071
44.181
30.193
29.712
29.455
29.198
28.941
28.683
28.426
28.169
25.061
18.685
18.276
O
O
O
H3C
CH3
O
210 200 190 180 170 160 150 140 130 120 110 100 90 80 70 60 50 40 30 20 10 0
f1 (ppm)

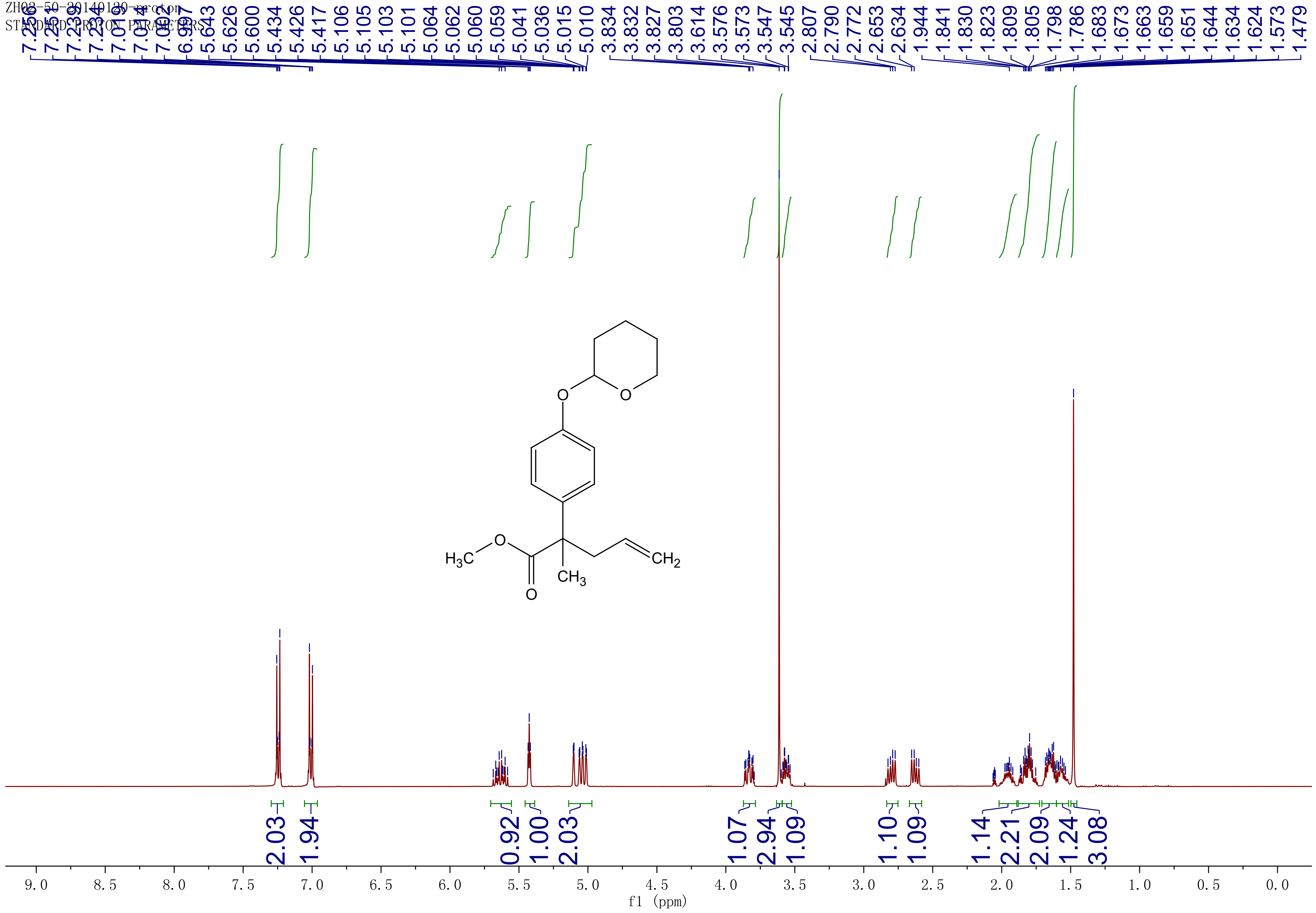
ZH02-50-20140120-proton
STANDARD PROTON PARAMETERS
5.643 5.626 5.600 5.434 5.426 5.417 5.106 5.105 5.103 5.101 5.064 5.062 5.060 5.059 5.041 5.036 5.015 5.010 3.834 3.832 3.827 3.803 3.614 3.576 3.573 3.547 3.545 2.807 2.790 2.772 2.653 2.634 1.944 1.841 1.830 1.823 1.809 1.805 1.798 1.786 1.683 1.673 1.663 1.659 1.651 1.644 1.634 1.624 1.573 1.479
O
O
H3C
O
CH3
CH2
O
2.03 1.94 0.92 1.00 2.03 1.07 2.94 1.09 1.10 1.09 1.14 2.21 2.09 1.24 3.08
9.0 8.5 8.0 7.5 7.0 6.5 6.0 5.5 5.0 4.5 4.0 3.5 3.0 2.5 2.0 1.5 1.0 0.5 0.0
f1 (ppm)

ZH02_50-carbon_CARBON_01

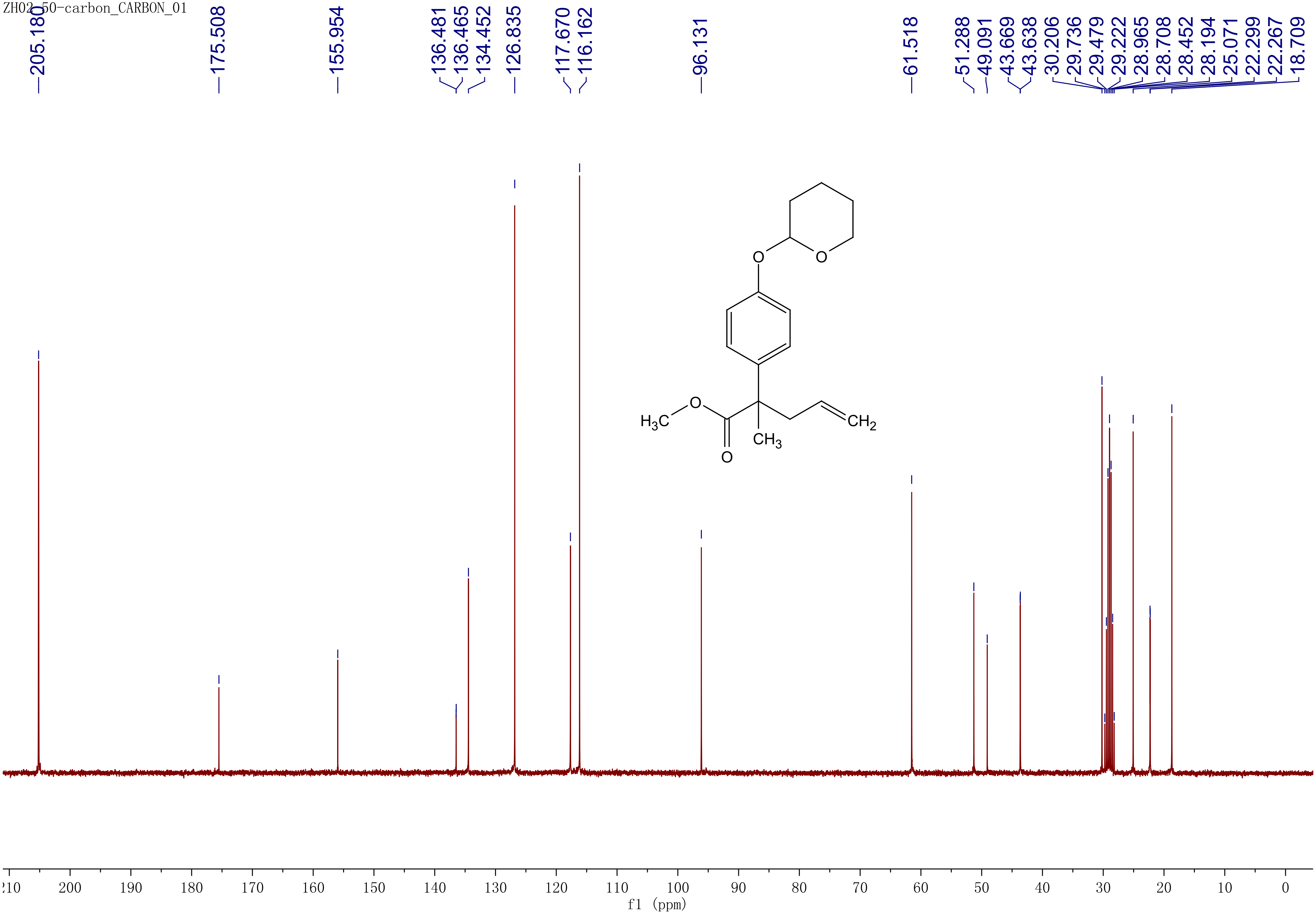

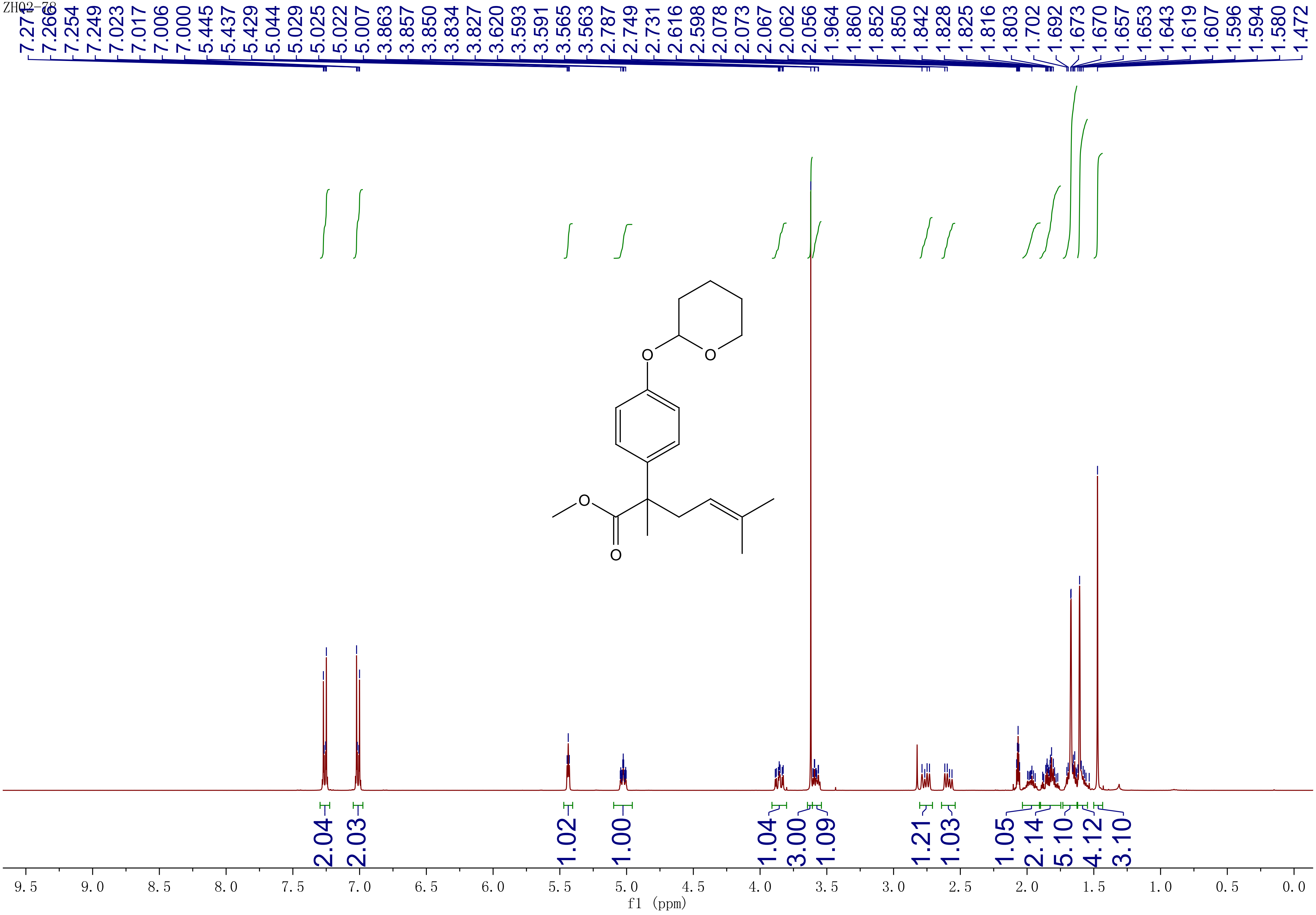
ZH02-78
7.266 7.254 7.249 7.023 7.017 7.006 7.000 5.445 5.437 5.429 5.044 5.029 5.025 5.022 5.007 3.863 3.857 3.850 3.834 3.827 3.620 3.593 3.591 3.565 3.563 2.787 2.749 2.731 2.616 2.598 2.078 2.073 2.067 2.062 2.056 1.964 1.860 1.852 1.850 1.842 1.828 1.825 1.816 1.803 1.702 1.692 1.673 1.670 1.657 1.653 1.643 1.619 1.607 1.596 1.594 1.580 1.472
O
O
O
O
2.04 2.03 1.02 1.00 1.04 3.00 1.09 1.21 1.03 1.05 2.14 5.10 4.12 3.10
9.5 9.0 8.5 8.0 7.5 7.0 6.5 6.0 5.5 5.0 4.5 4.0 3.5 3.0 2.5 2.0 1.5 1.0 0.5 0.0
f1 (ppm)

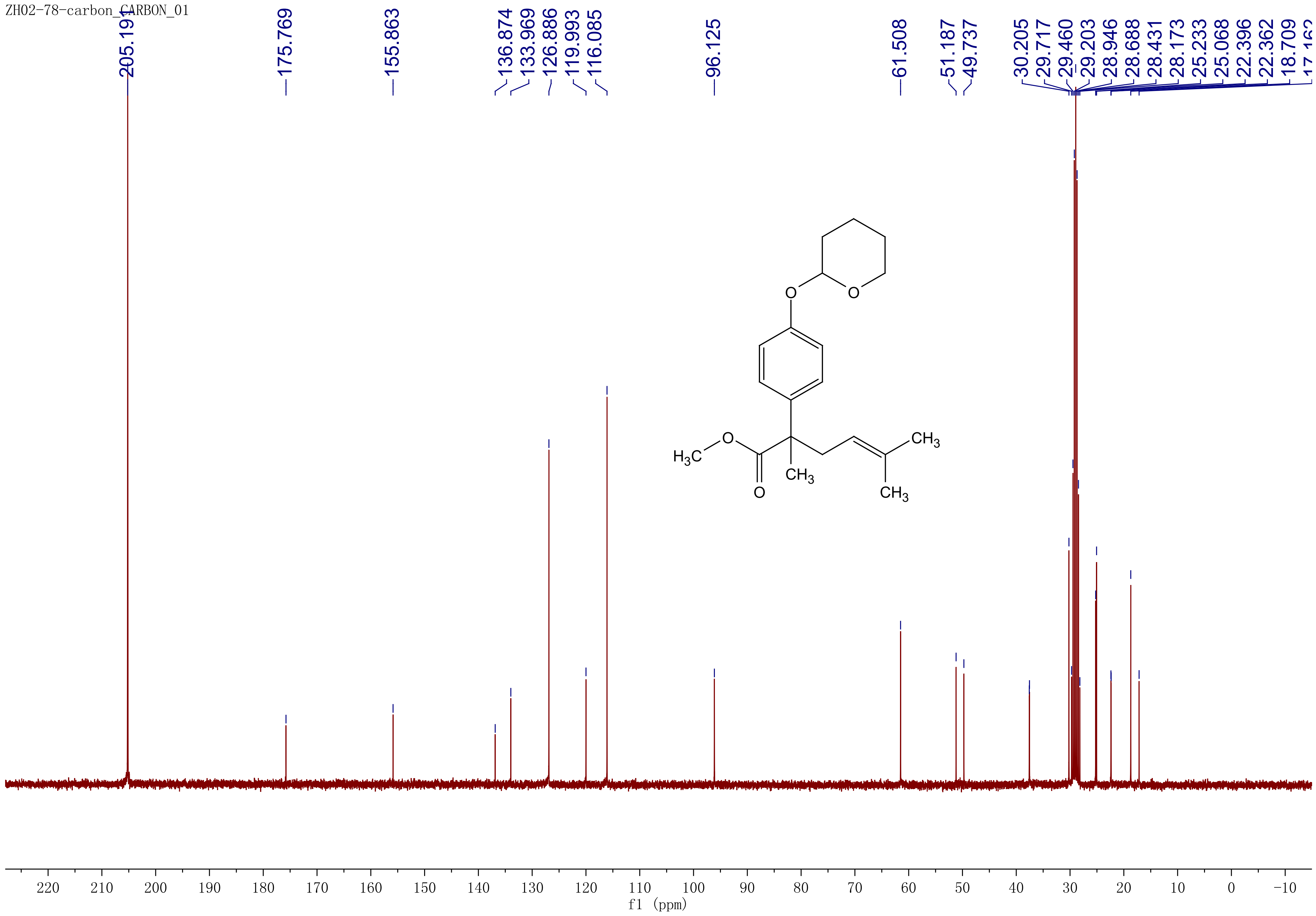
ZH02-78-carbon_CARBON_01
205.19
175.769
155.863
136.874
133.969
126.886
119.993
116.085
96.125
61.508
51.187
49.737
30.205
29.717
29.460
29.203
28.946
28.688
28.431
28.173
25.233
25.068
22.396
22.362
18.709
O
O
O
H3C
O
CH3
O
CH3
CH3
220 210 200 190 180 170 160 150 140 130 120 110 100 90 80 70 60 50 40 30 20 10 0 -10
f1 (ppm)

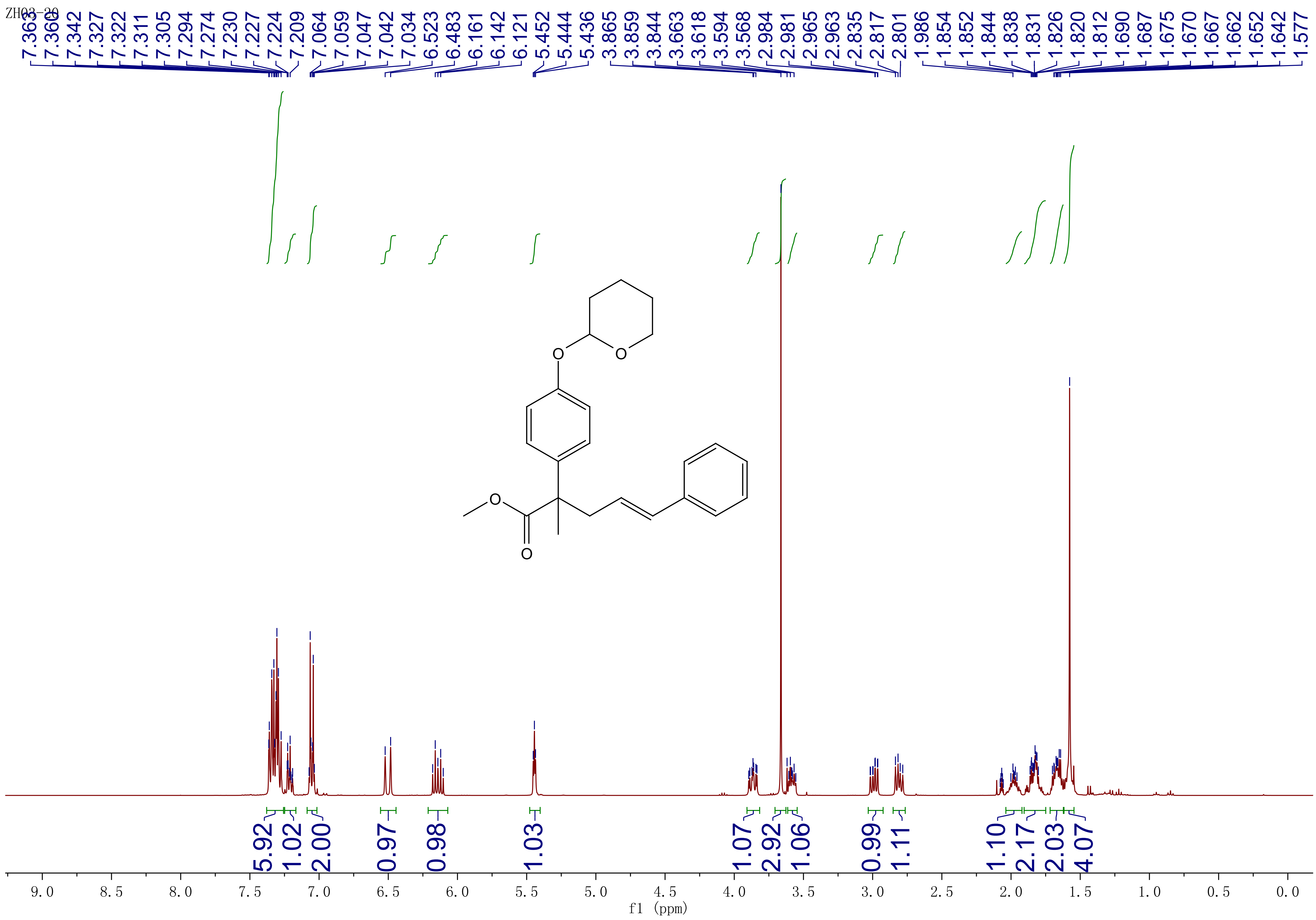

ZH03-20
7.363 7.360 7.342 7.327 7.322 7.311 7.305 7.294 7.274 7.230 7.227 7.224 7.209 7.064 7.059 7.047 7.042 7.034 6.523 6.483 6.161 6.142 6.121 5.452 5.444 5.436 3.865 3.859 3.844 3.663 3.618 3.594 3.568 2.984 2.981 2.965 2.963 2.835 2.817 2.801 1.986 1.854 1.852 1.844 1.838 1.831 1.826 1.820 1.812 1.690 1.687 1.675 1.670 1.667 1.662 1.652 1.642 1.577
O
O
O
O
5.92 1.02 2.00 0.97 0.98 1.03 1.07 2.92 1.06 0.99 1.11 1.10 2.17 2.03 4.07
9.0 8.5 8.0 7.5 7.0 6.5 6.0 5.5 5.0 4.5 4.0 3.5 3.0 2.5 2.0 1.5 1.0 0.5 0.0
f1 (ppm)

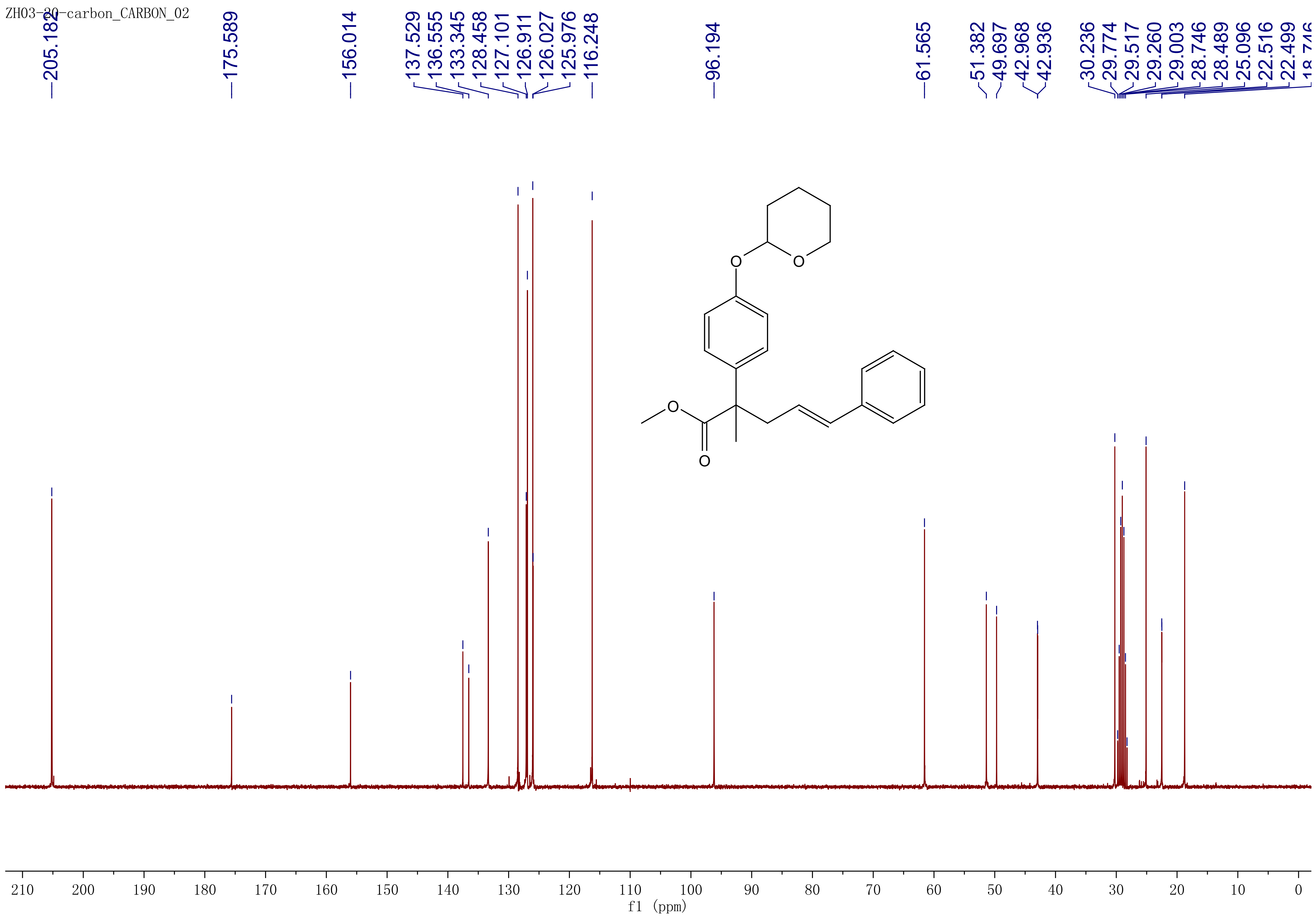
ZH03-20-carbon_CARBON_02
205.182
175.589
156.014
137.529
136.555
133.345
128.458
127.101
126.911
126.027
125.976
116.248
96.194
61.565
51.382
49.697
42.968
42.936
30.236
29.774
29.517
29.260
29.003
28.746
28.489
25.096
22.516
22.499
O
O
O
O
210 200 190 180 170 160 150 140 130 120 110 100 90 80 70 60 50 40 30 20 10 0
f1 (ppm)

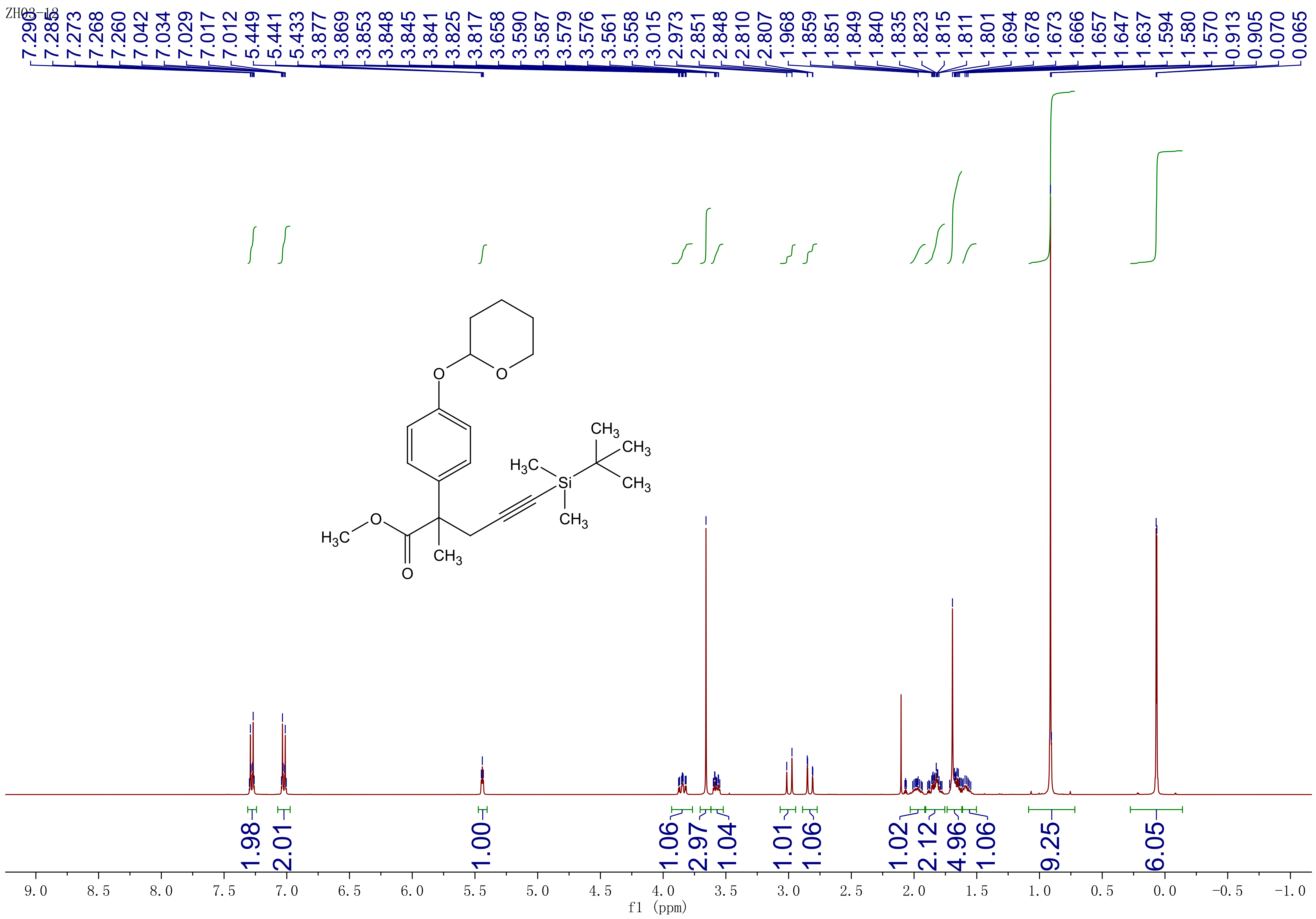

ZH03-12
7.290 7.285 7.273 7.268 7.260 7.042 7.034 7.029 7.017 7.012 5.449 5.441 5.433 3.877 3.869 3.853 3.848 3.845 3.841 3.825 3.817 3.658 3.590 3.587 3.579 3.576 3.561 3.558 3.015 2.973 2.851 2.848 2.810 2.807 1.968 1.859 1.851 1.849 1.840 1.835 1.823 1.815 1.811 1.801 1.694 1.678 1.673 1.666 1.657 1.647 1.637 1.594 1.580 1.570 0.913 0.905 0.070 0.065
O
O
O
H3C
CH3
CH3
CH3
Si
CH3
H3C
O
O
CH3
1.98
2.01
1.00
1.06
2.97
1.04
1.01
1.06
1.02
2.12
4.96
1.06
9.25
6.05
9.0 8.5 8.0 7.5 7.0 6.5 6.0 5.5 5.0 4.5 4.0 3.5 3.0 2.5 2.0 1.5 1.0 0.5 0.0 −0.5 −1.0
f1 (ppm)

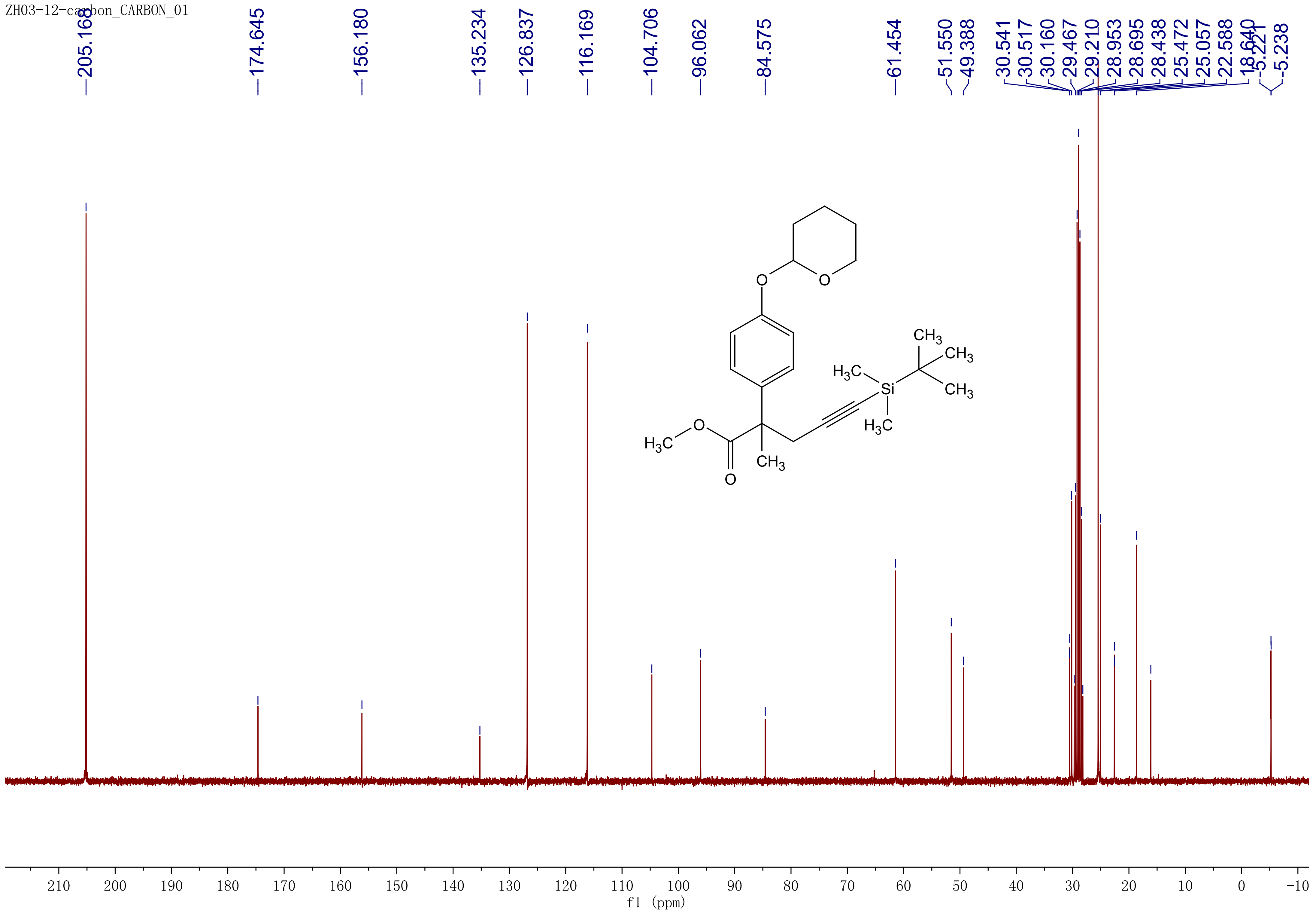
ZH03-12-carbon_CARBON_01
205.168
174.645
156.180
135.234
126.837
116.169
104.706
96.062
84.575
61.454
51.550
49.388
30.541
30.517
30.160
29.467
29.210
28.953
28.695
28.438
25.472
25.057
22.588
18.640
-5.221
-5.238
O
O
CH3
CH3
H3C
Si
CH3
H3C
H3C
O
CH3
O
210
200
190
180
170
160
150
140
130
120
110
100
90
80
70
60
50
40
30
20
10
0
-10
f1 (ppm)

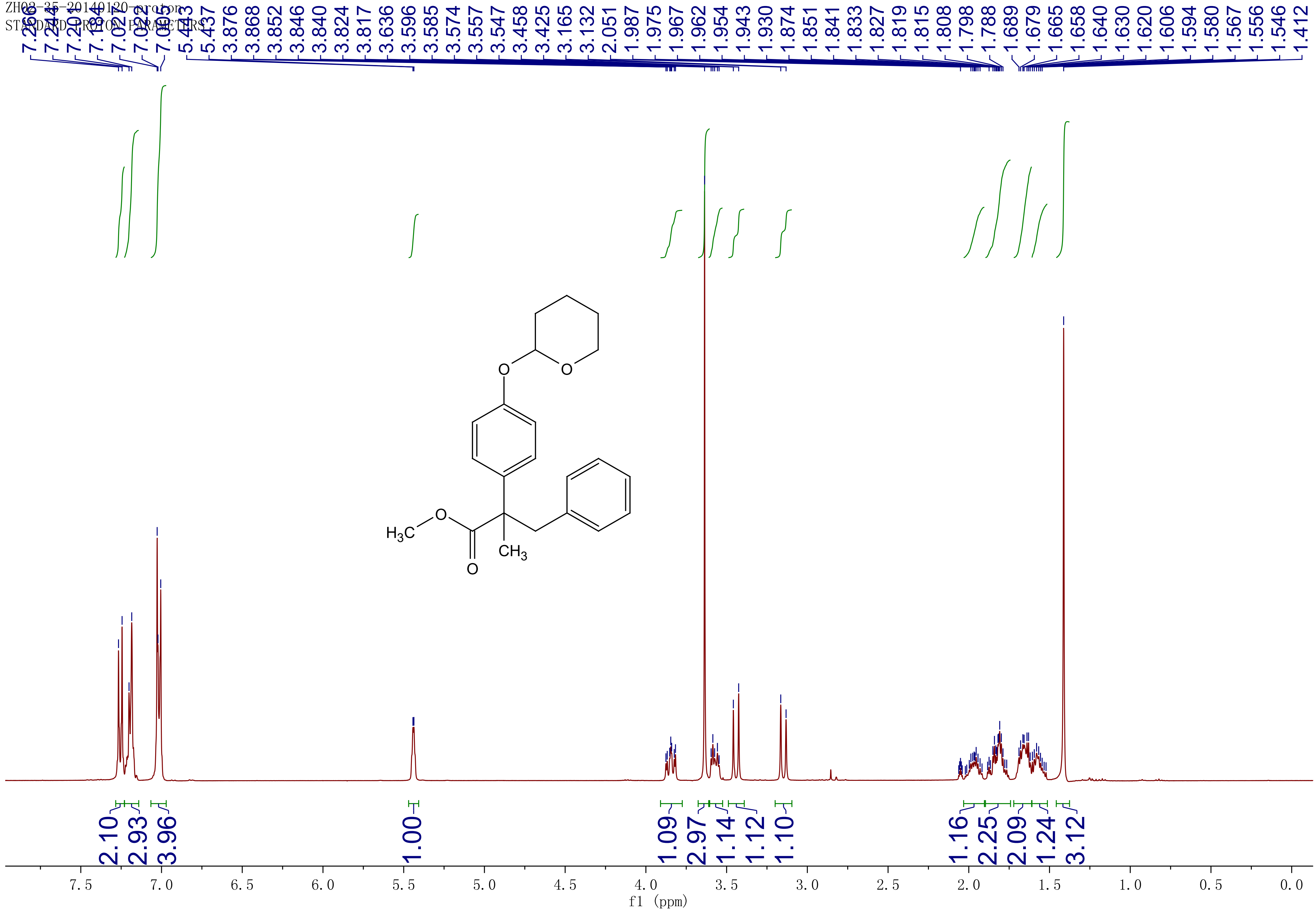
ZH02-35-20140120-proton
STANDARD PROTON PARAMETERS
7.266
7.244
7.201
7.184
7.027
7.022
7.005
5.443
5.437
3.876
3.868
3.852
3.846
3.840
3.824
3.817
3.636
3.596
3.585
3.574
3.557
3.547
3.458
3.425
3.165
3.132
2.051
1.987
1.975
1.967
1.962
1.954
1.943
1.930
1.874
1.851
1.841
1.833
1.827
1.819
1.815
1.808
1.798
1.788
1.689
1.679
1.665
1.658
1.640
1.630
1.620
1.606
1.594
1.580
1.567
1.556
1.546
1.412
O
O
O
$H_3C$
O
$CH_3$
2.10
2.93
3.96
1.00
1.09
2.97
1.14
1.12
1.10
1.16
2.25
2.09
1.24
3.12
7.5
7.0
6.5
6.0
5.5
5.0
4.5
4.0
3.5
3.0
2.5
2.0
1.5
1.0
0.5
0.0
f1 (ppm)

ZH02-35-carbon_CARBON_02

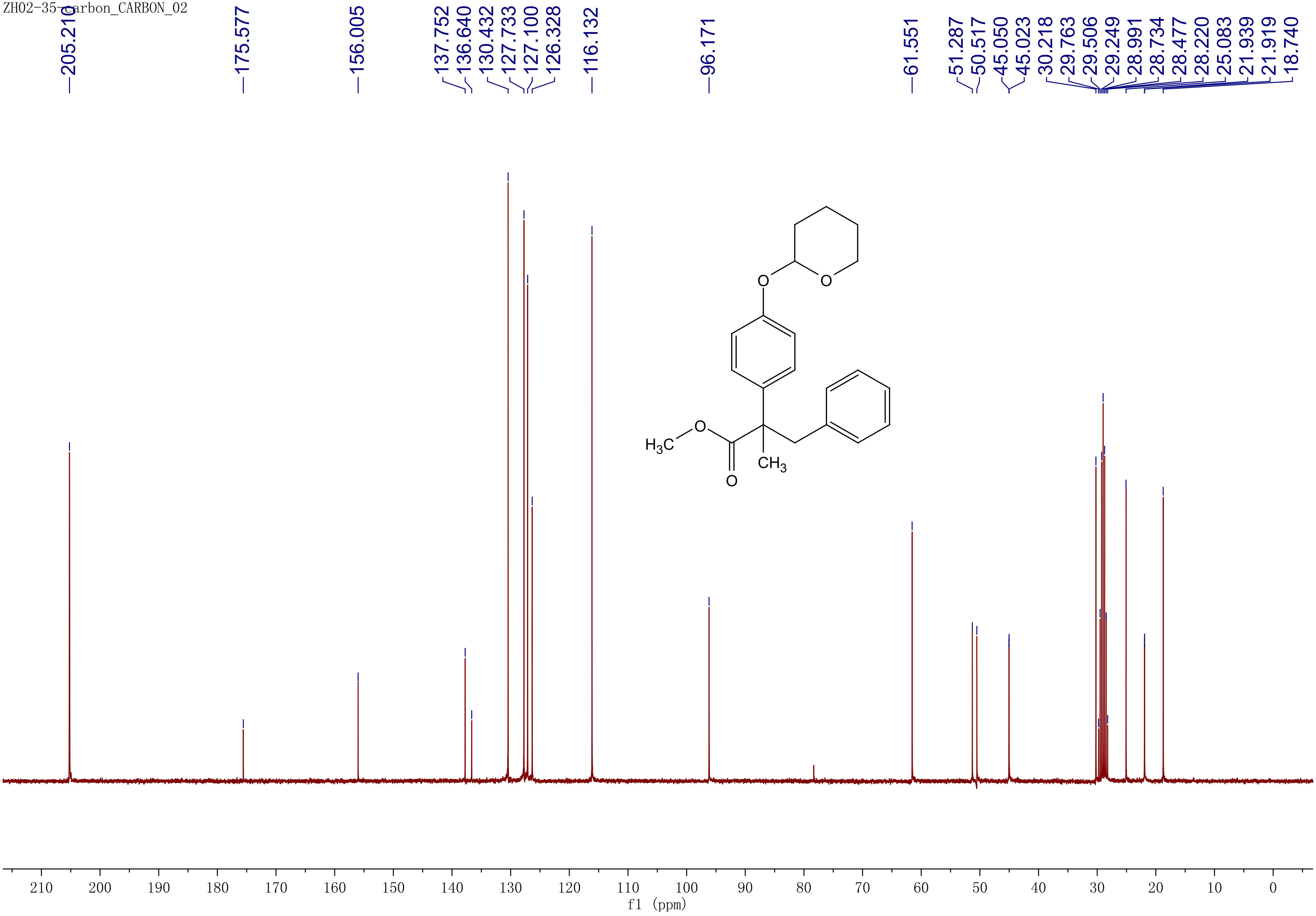

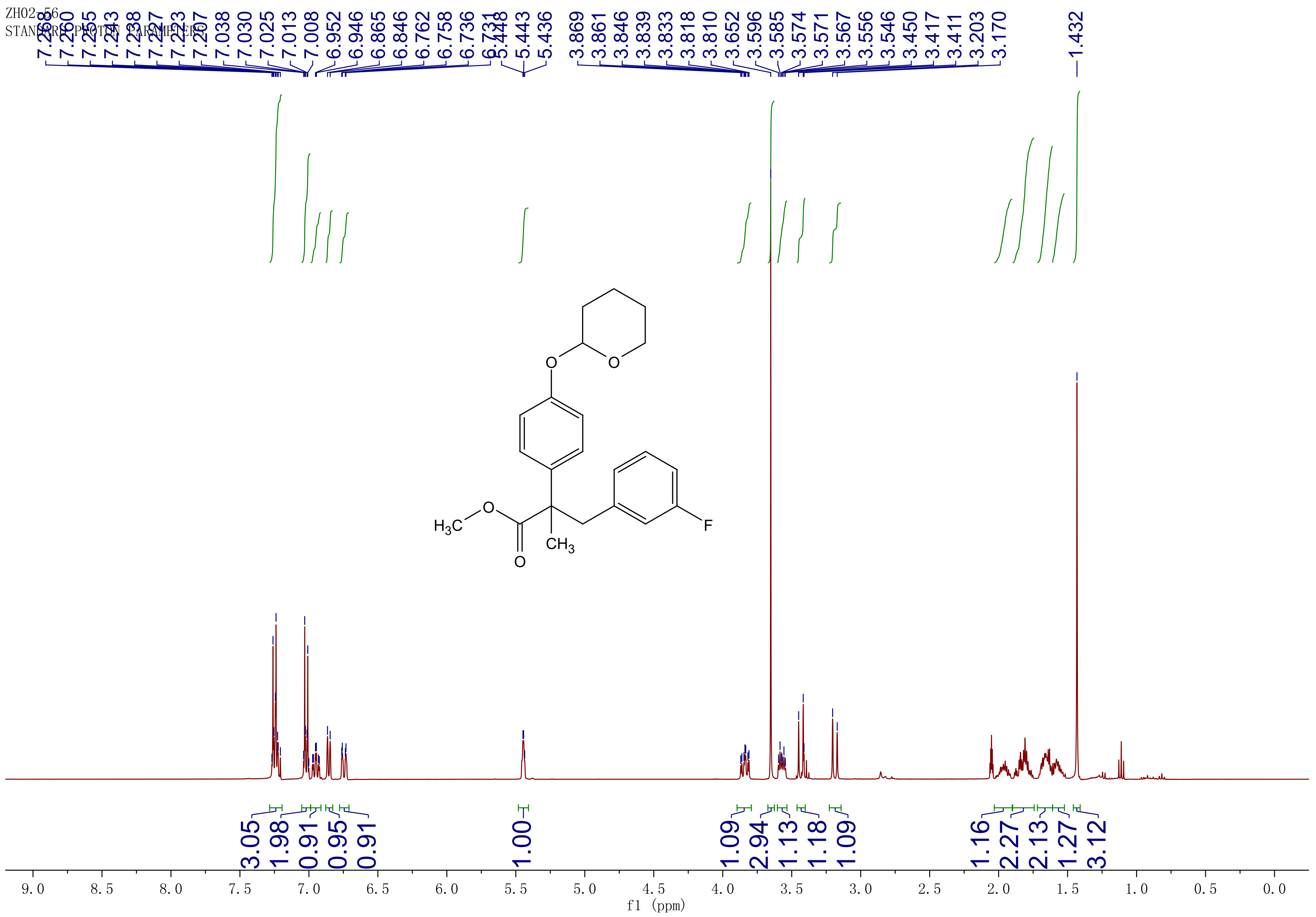

ZH02-56
STANDARD PROTON PARAMETERS
7.260
7.255
7.243
7.238
7.227
7.223
7.207
7.038
7.030
7.025
7.013
7.008
6.952
6.946
6.865
6.846
6.762
6.758
6.736
6.731
5.448
5.443
5.436
3.869
3.861
3.846
3.839
3.833
3.818
3.810
3.652
3.596
3.585
3.574
3.571
3.567
3.556
3.546
3.450
3.417
3.411
3.203
3.170
1.432
O
O
H3C
O
O
CH3
F
3.05
1.98
0.91
0.95
0.91
1.00
1.09
2.94
1.13
1.18
1.09
1.16
2.27
2.13
1.27
3.12
9.0
8.5
8.0
7.5
7.0
6.5
6.0
5.5
5.0
4.5
4.0
3.5
3.0
2.5
2.0
1.5
1.0
0.5
0.0
f1 (ppm)

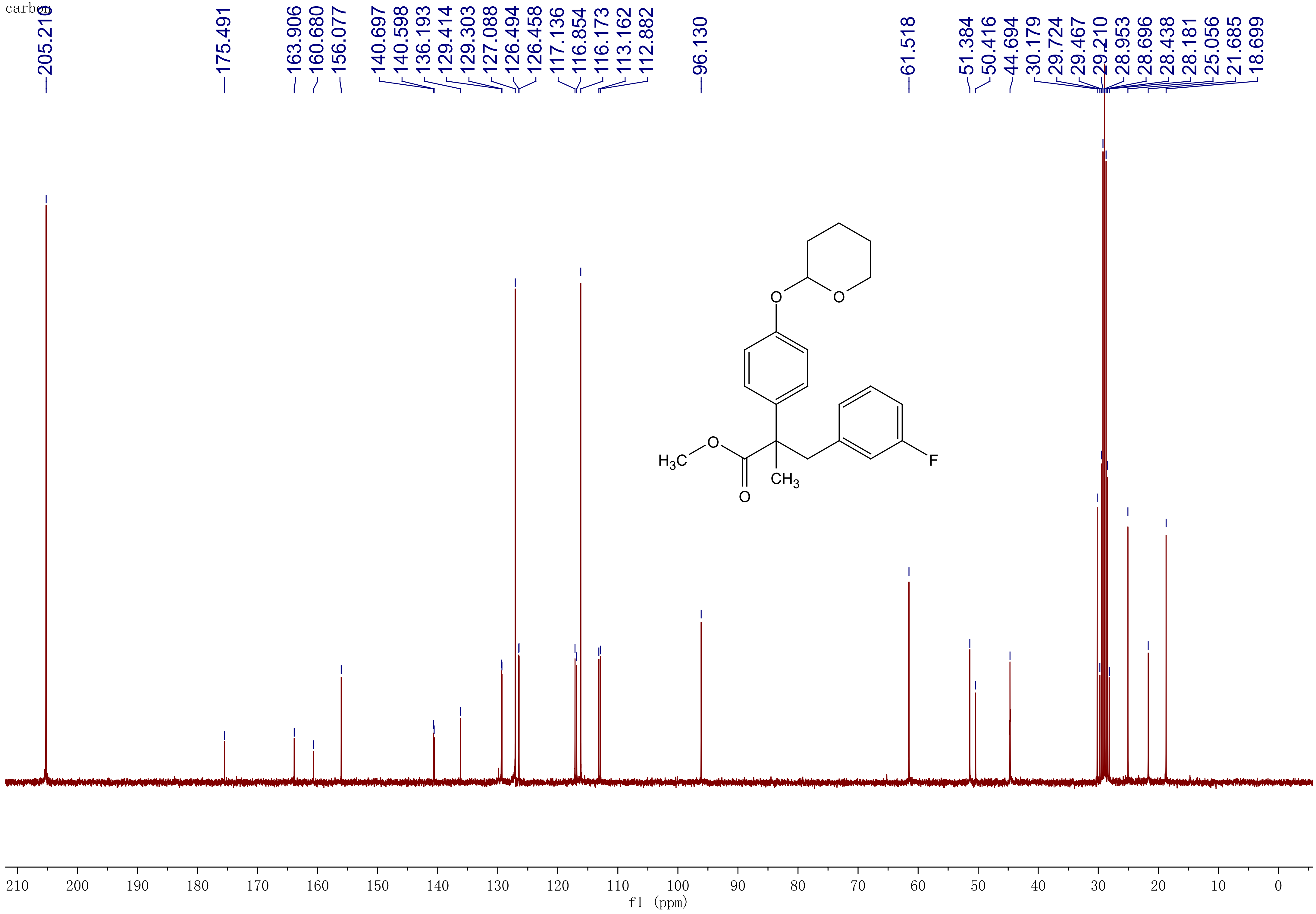

carbon
205.210
175.491
163.906
160.680
156.077
140.697
140.598
136.193
129.414
129.303
127.088
126.494
126.458
117.136
116.854
116.173
113.162
112.882
96.130
61.518
51.384
50.416
44.694
30.179
29.724
29.467
29.210
28.953
28.696
28.438
28.181
25.056
21.685
18.699
O
O
$H_3C$
O
O
$CH_3$
F
210
200
190
180
170
160
150
140
130
120
110
100
90
80
70
60
50
40
30
20
10
0
f1 (ppm)

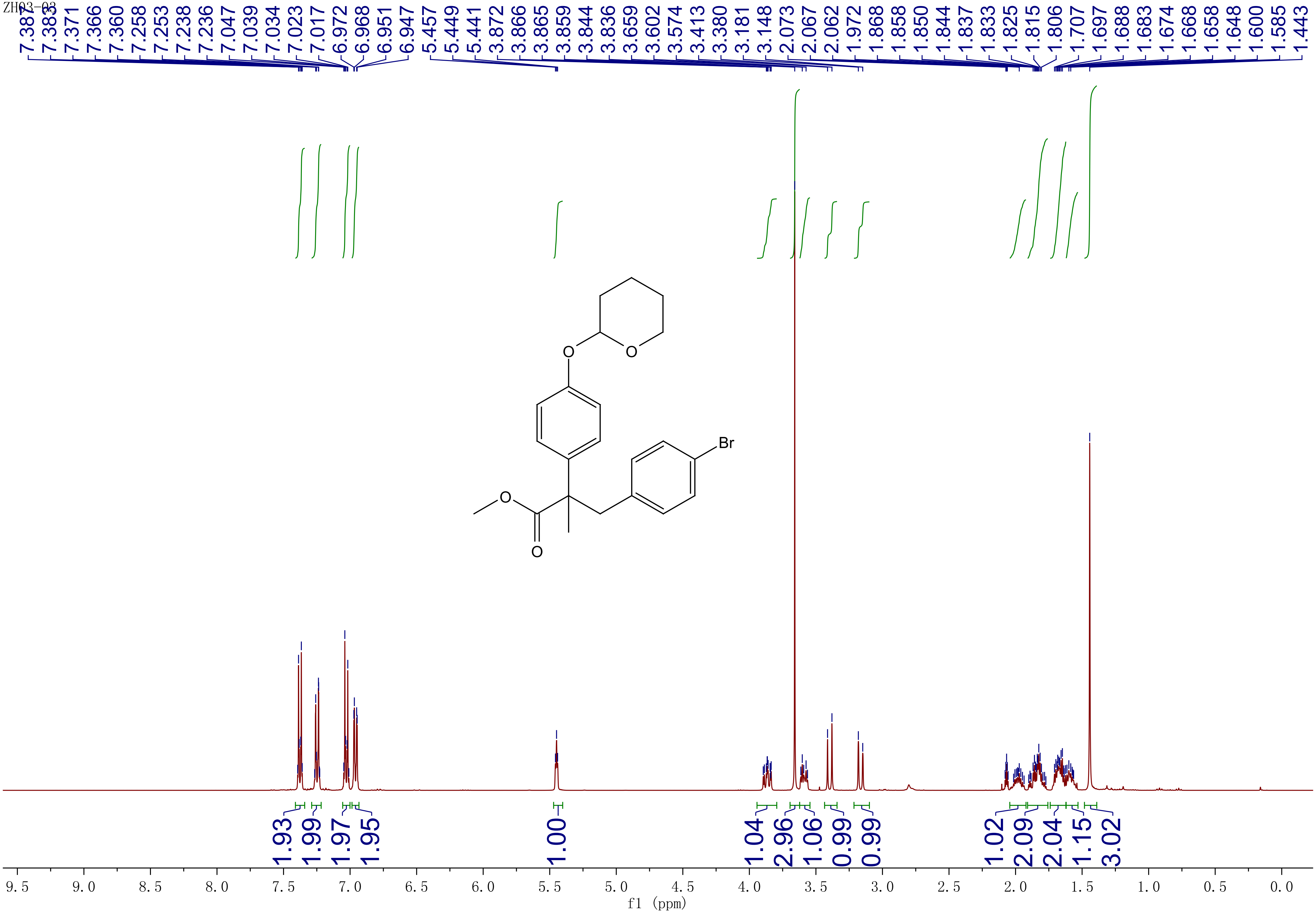

ZH03-03
7.387
7.383
7.371
7.366
7.360
7.258
7.253
7.238
7.236
7.047
7.039
7.034
7.023
7.017
6.972
6.968
6.951
6.947
5.457
5.449
5.441
3.872
3.866
3.865
3.859
3.844
3.836
3.659
3.602
3.574
3.413
3.380
3.181
3.148
2.073
2.067
2.062
1.972
1.868
1.858
1.850
1.844
1.837
1.833
1.825
1.815
1.806
1.707
1.697
1.688
1.683
1.674
1.668
1.658
1.648
1.600
1.585
1.443
O
O
Br
O
O
1.93
1.99
1.97
1.95
1.00
1.04
2.96
1.06
0.99
0.99
1.02
2.09
2.04
1.15
3.02
9.5
9.0
8.5
8.0
7.5
7.0
6.5
6.0
5.5
5.0
4.5
4.0
3.5
3.0
2.5
2.0
1.5
1.0
0.5
0.0
f1 (ppm)

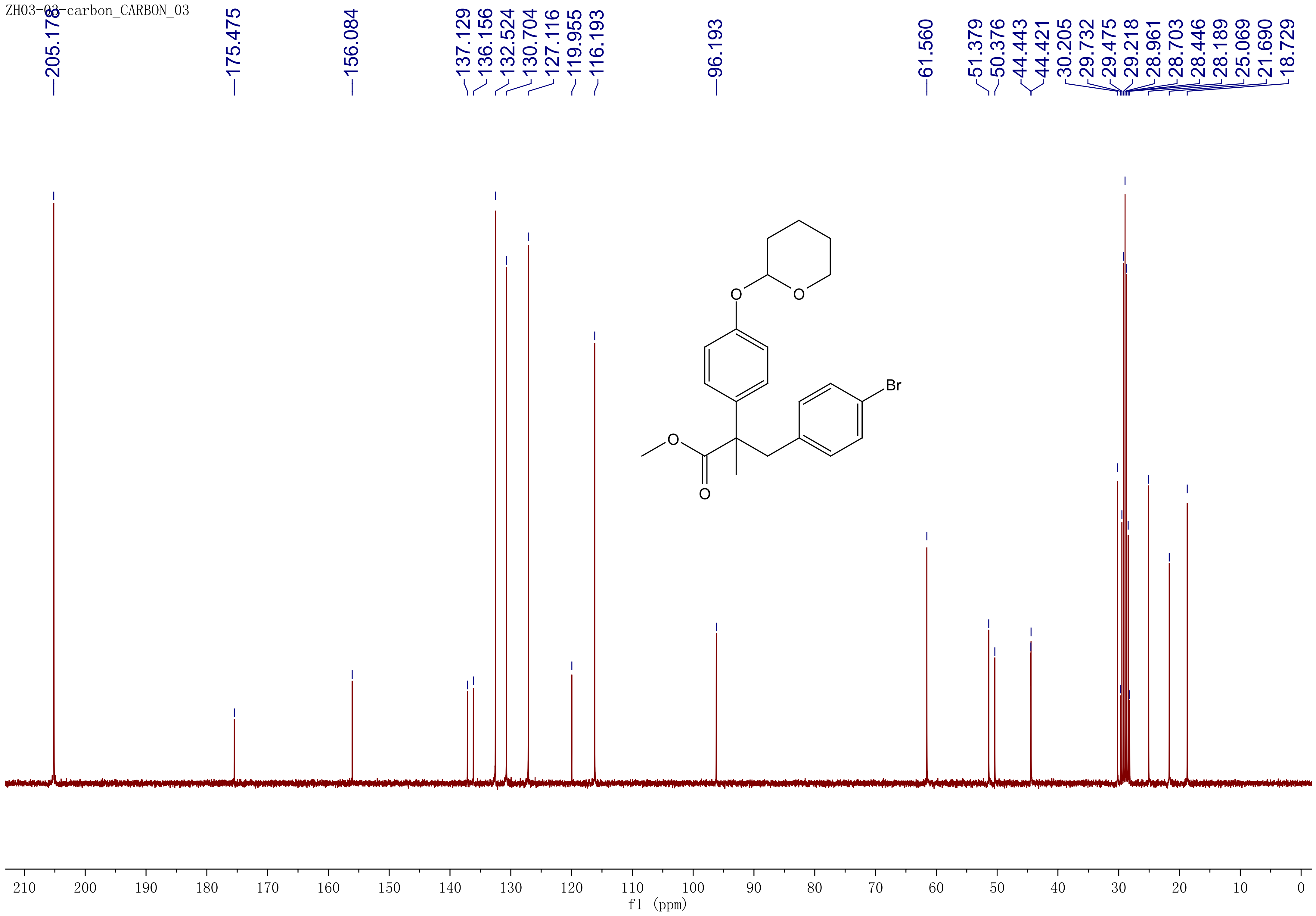

ZH03-03-carbon_CARBON_03
205.178
175.475
156.084
137.129
136.156
132.524
130.704
127.116
119.955
116.193
96.193
61.560
51.379
50.376
44.443
44.421
30.205
29.732
29.475
29.218
28.961
28.703
28.446
28.189
25.069
21.690
18.729
O
O
Br
O
O
210 200 190 180 170 160 150 140 130 120 110 100 90 80 70 60 50 40 30 20 10 0
f1 (ppm)

ZH02-71

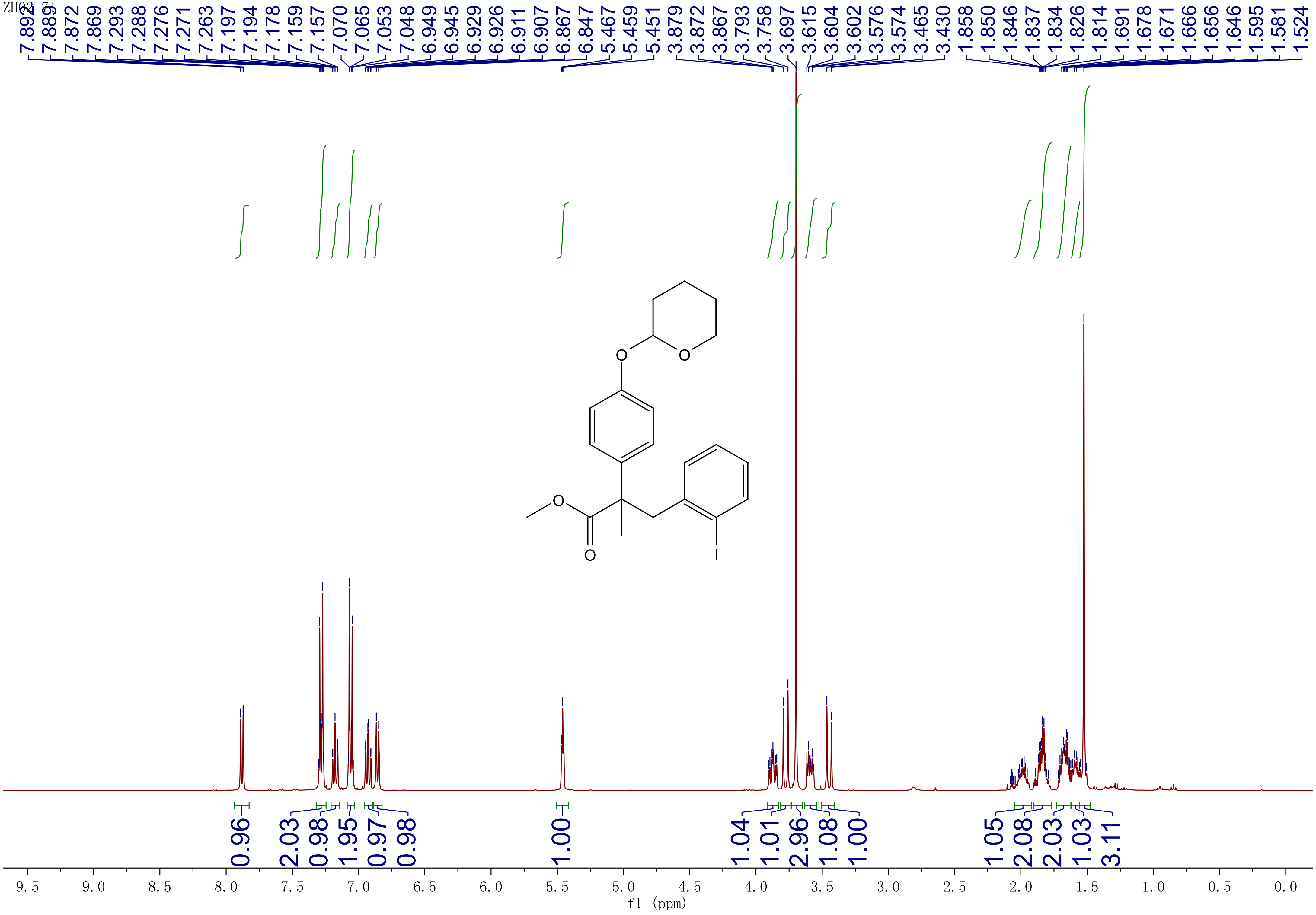

7.892 7.889 7.872 7.869 7.293 7.288 7.276 7.271 7.263 7.197 7.194 7.178 7.159 7.157 7.070 7.065 7.053 7.048 6.949 6.945 6.929 6.926 6.911 6.907 6.867 6.847 5.467 5.459 5.451 3.879 3.872 3.867 3.793 3.758 3.697 3.615 3.604 3.602 3.576 3.574 3.465 3.430 1.858 1.850 1.846 1.837 1.834 1.826 1.814 1.691 1.678 1.671 1.666 1.656 1.646 1.595 1.581 1.524
O
O
O
O
I
0.96
2.03
0.98
1.95
0.97
0.98
1.00
1.04
1.01
2.96
1.08
1.00
1.05
2.08
2.03
1.03
3.11
9.5 9.0 8.5 8.0 7.5 7.0 6.5 6.0 5.5 5.0 4.5 4.0 3.5 3.0 2.5 2.0 1.5 1.0 0.5 0.0
f1 (ppm)

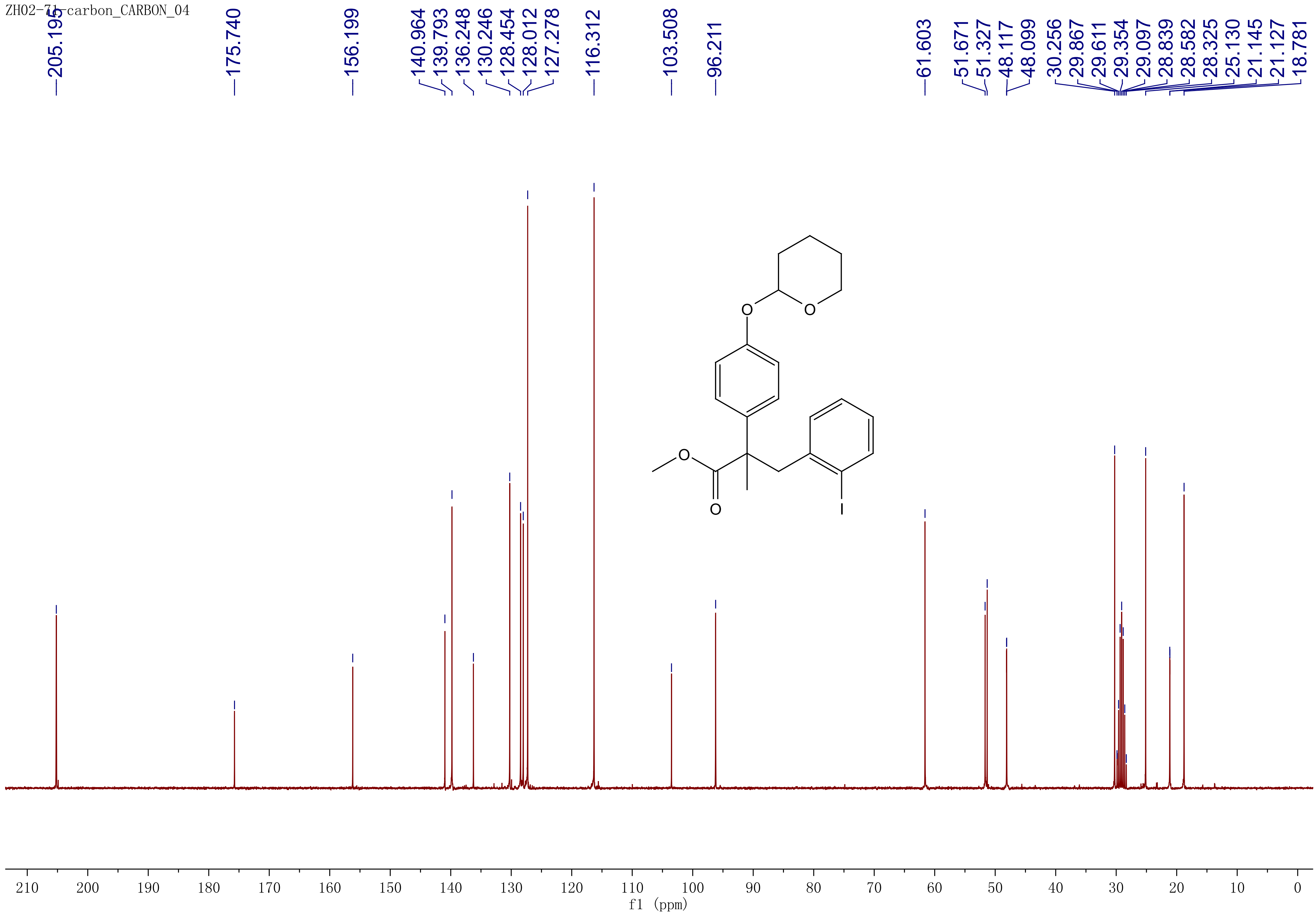
ZH02-71-carbon_CARBON_04
205.195
175.740
156.199
140.964
139.793
136.248
130.246
128.454
128.012
127.278
116.312
103.508
96.211
61.603
51.671
51.327
48.117
48.099
30.256
29.867
29.611
29.354
29.097
28.839
28.582
28.325
25.130
21.145
21.127
18.781
O
O
O
O
I
210
200
190
180
170
160
150
140
130
120
110
100
90
80
70
60
50
40
30
20
10
0
f1 (ppm)

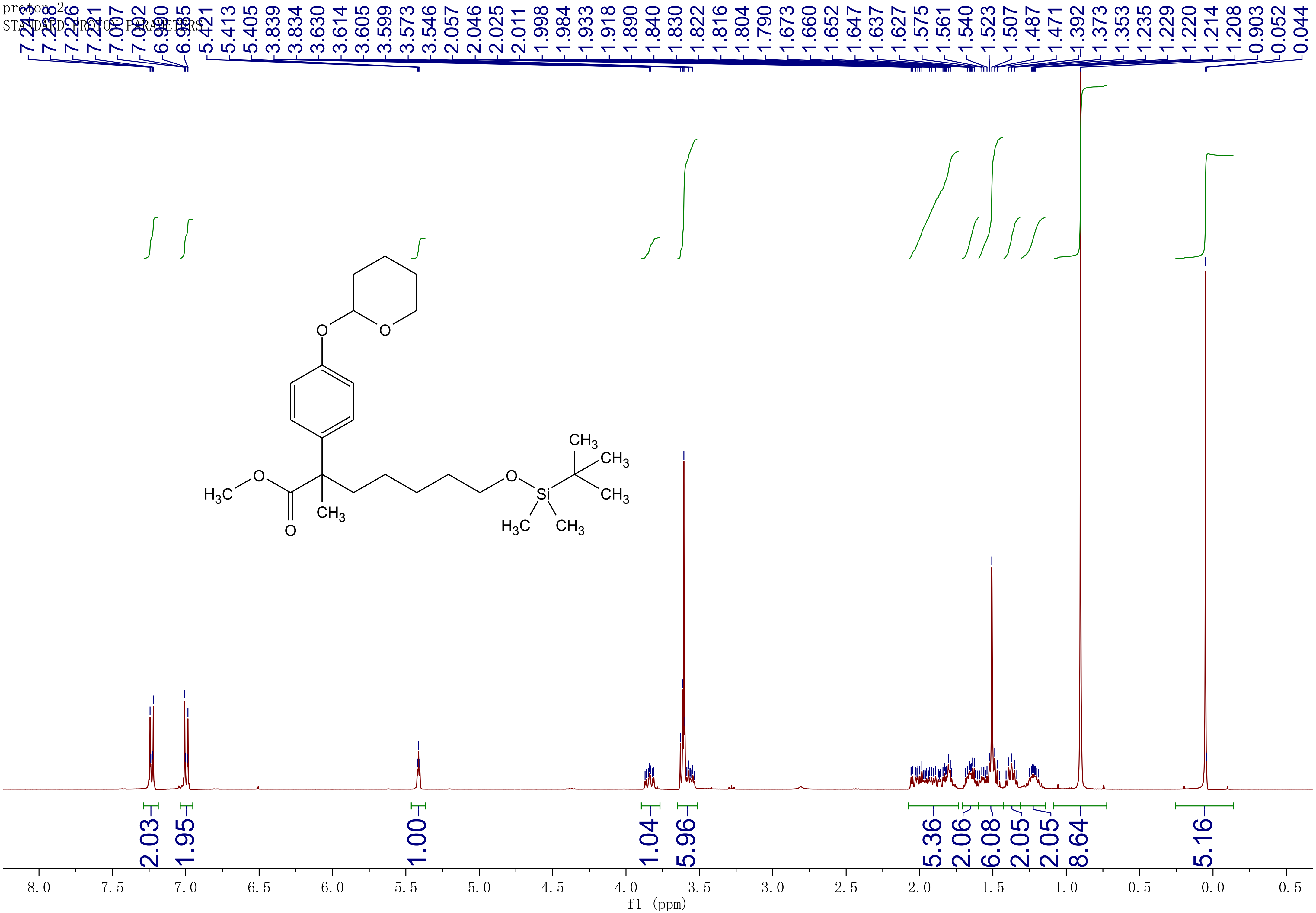
proton-2
STANDARD PROTON PARAMETERS
7.243 7.238 7.226 7.221 7.007 7.002 6.990 6.985 5.421 5.413 5.405 3.839 3.834 3.630 3.614 3.605 3.599 3.573 3.546 2.057 2.046 2.025 2.011 1.998 1.984 1.933 1.918 1.890 1.840 1.830 1.822 1.816 1.804 1.790 1.673 1.660 1.652 1.647 1.637 1.627 1.575 1.561 1.540 1.523 1.507 1.487 1.471 1.392 1.373 1.353 1.235 1.229 1.220 1.214 1.208 0.903 0.052 0.044
2.03 1.95 1.00 1.04 5.96 5.36 2.06 6.08 2.05 2.05 8.64 5.16
8.0 7.5 7.0 6.5 6.0 5.5 5.0 4.5 4.0 3.5 3.0 2.5 2.0 1.5 1.0 0.5 0.0 -0.5
f1 (ppm)
O
O
O
$H_3C$
O
O
$CH_3$
O
Si
$CH_3$
$CH_3$
$CH_3$
$H_3C$
$CH_3$

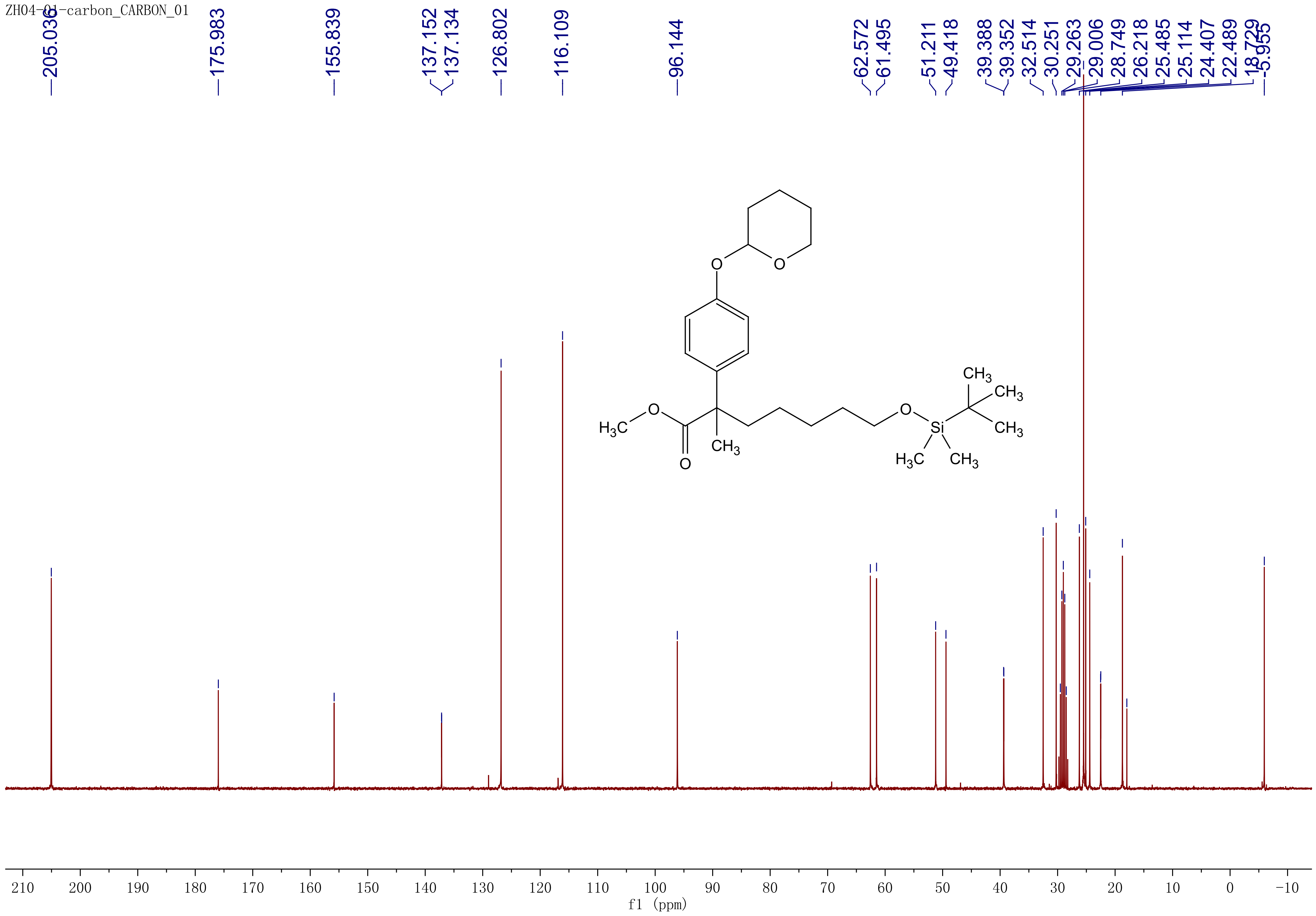
ZH04-01-carbon_CARBON_01
205.036
175.983
155.839
137.152
137.134
126.802
116.109
96.144
62.572
61.495
51.211
49.418
39.388
39.352
32.514
30.251
29.263
29.006
28.749
26.218
25.485
25.114
24.407
22.489
18.729
-5.955
O
O
O
$H_3C$
O
$CH_3$
O
$CH_3$
$CH_3$
Si
$CH_3$
$H_3C$
$CH_3$
210 200 190 180 170 160 150 140 130 120 110 100 90 80 70 60 50 40 30 20 10 0 -10
f1 (ppm)

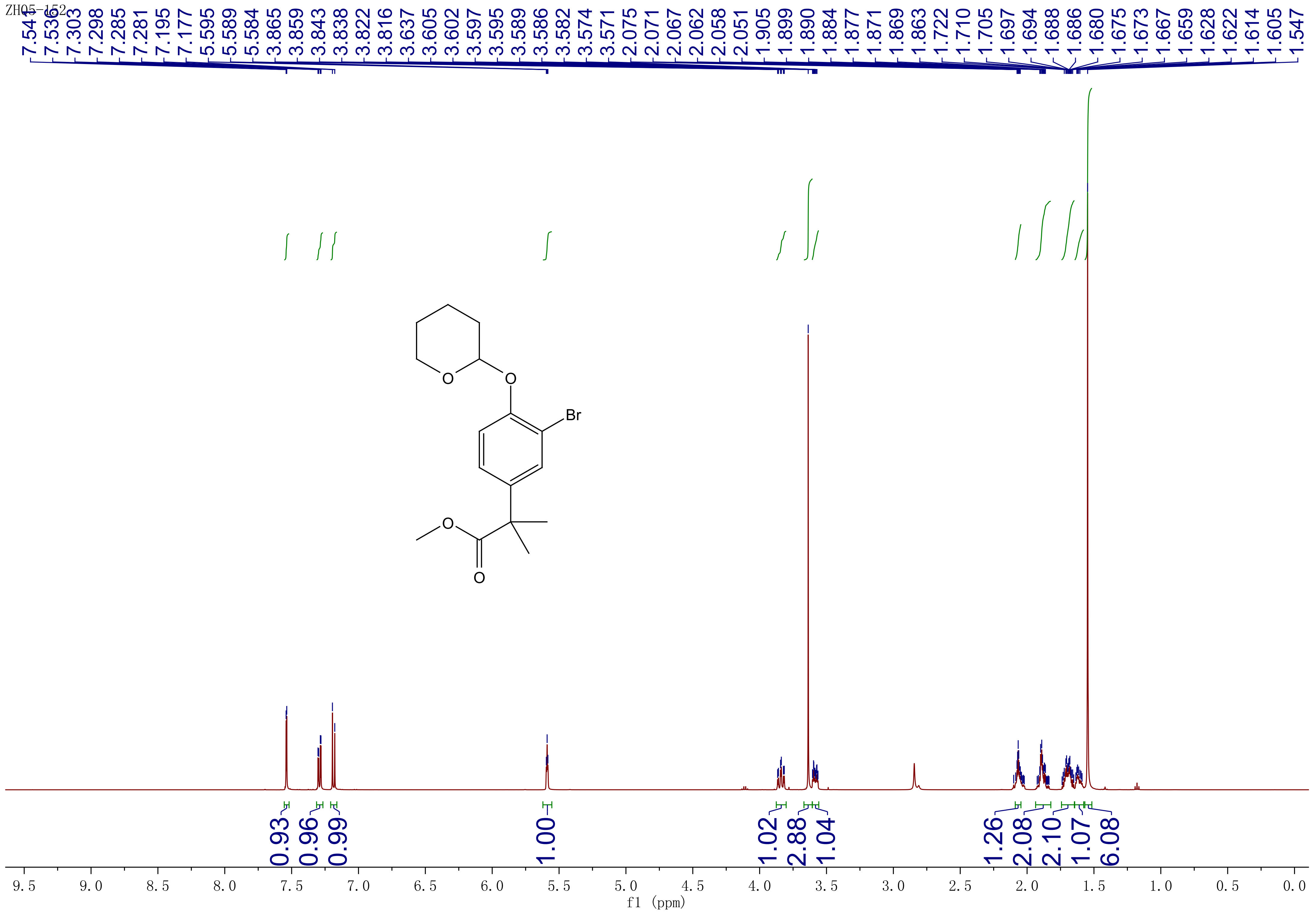
ZH05-152
7.54[illegible]
7.536
7.303
7.298
7.285
7.281
7.195
7.177
5.595
5.589
5.584
3.865
3.859
3.843
3.838
3.822
3.816
3.637
3.605
3.602
3.597
3.595
3.589
3.586
3.582
3.574
3.571
2.075
2.071
2.067
2.062
2.058
2.051
1.905
1.899
1.890
1.884
1.877
1.871
1.869
1.863
1.722
1.710
1.705
1.697
1.694
1.688
1.686
1.680
1.675
1.673
1.667
1.659
1.628
1.622
1.614
1.605
1.547
O
O
Br
O
O
0.93
0.96
0.99
1.00
1.02
2.88
1.04
1.26
2.08
2.10
1.07
6.08
9.5 9.0 8.5 8.0 7.5 7.0 6.5 6.0 5.5 5.0 4.5 4.0 3.5 3.0 2.5 2.0 1.5 1.0 0.5 0.0
f1 (ppm)

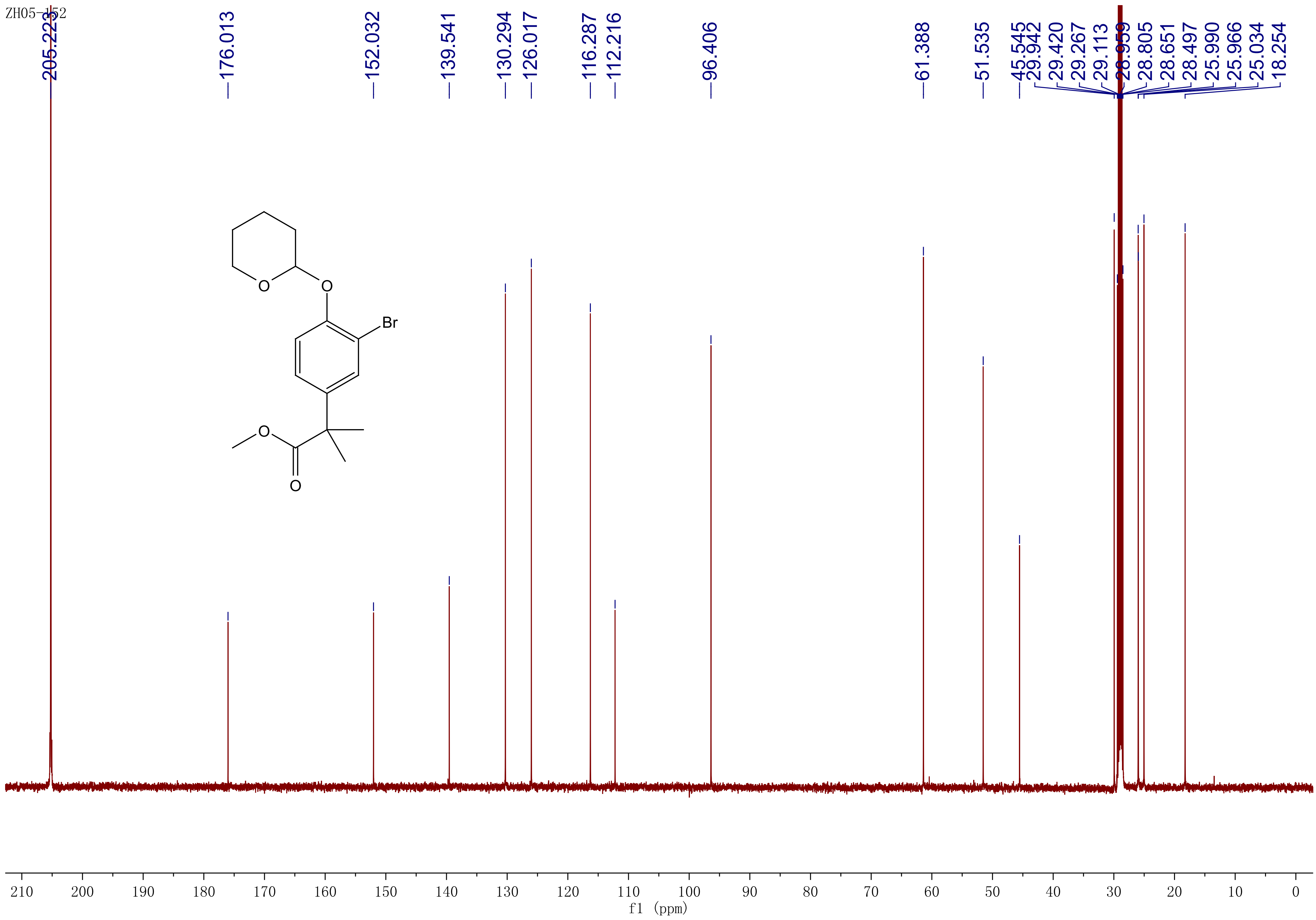
ZH05-152
205.223
176.013
152.032
139.541
130.294
126.017
116.287
112.216
96.406
61.388
51.535
45.545
29.942
29.420
29.267
29.113
28.959
28.805
28.651
28.497
25.990
25.966
25.034
18.254
O
O
Br
O
O
210 200 190 180 170 160 150 140 130 120 110 100 90 80 70 60 50 40 30 20 10 0
f1 (ppm)

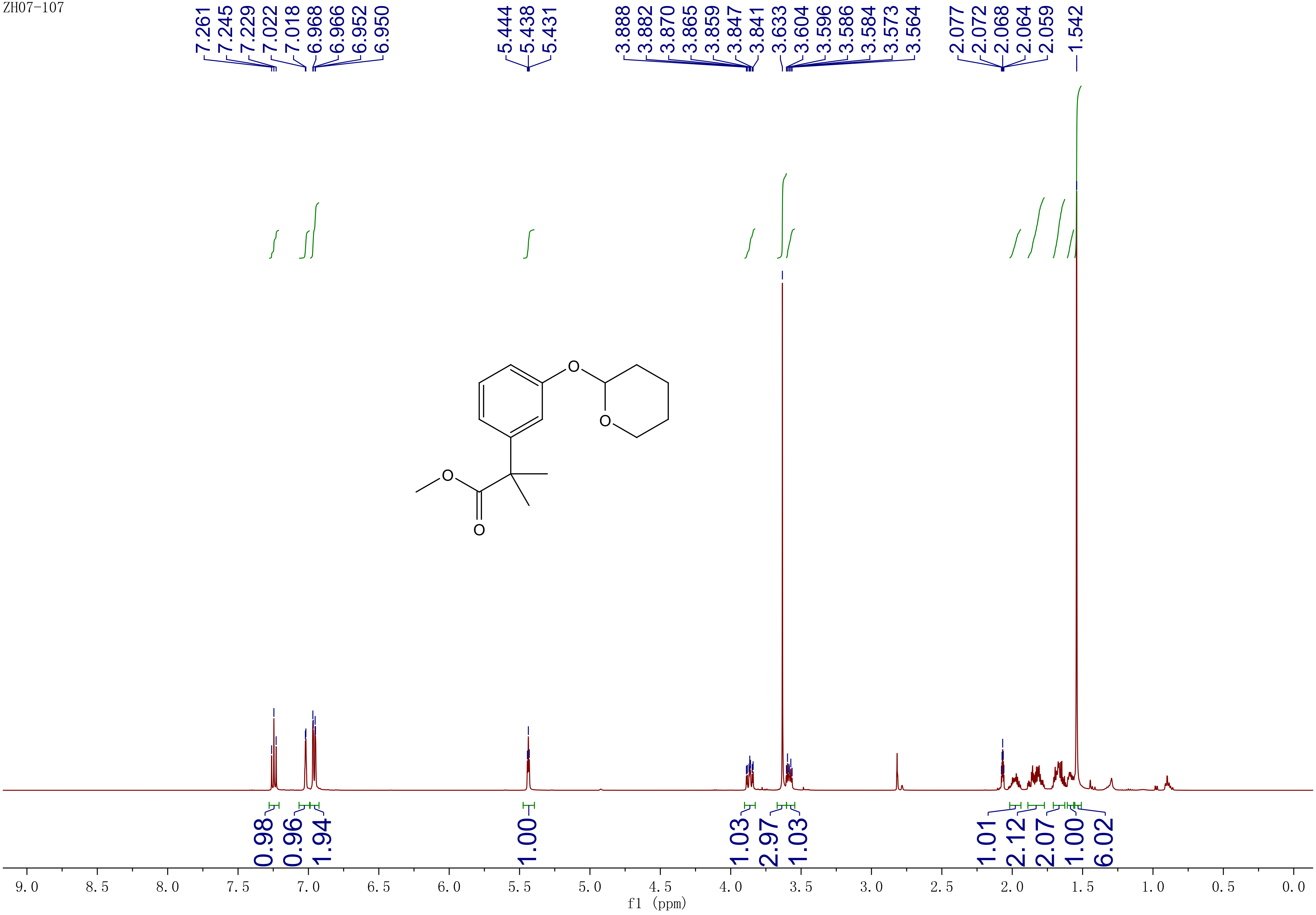
ZH07-107
7.261
7.245
7.229
7.022
7.018
6.968
6.966
6.952
6.950
5.444
5.438
5.431
3.888
3.882
3.870
3.865
3.859
3.847
3.841
3.633
3.604
3.596
3.586
3.584
3.573
3.564
2.077
2.072
2.068
2.064
2.059
1.542
0.98
0.96
1.94
1.00
1.03
2.97
1.03
1.01
2.12
2.07
1.00
6.02
9.0 8.5 8.0 7.5 7.0 6.5 6.0 5.5 5.0 4.5 4.0 3.5 3.0 2.5 2.0 1.5 1.0 0.5 0.0
f1 (ppm)

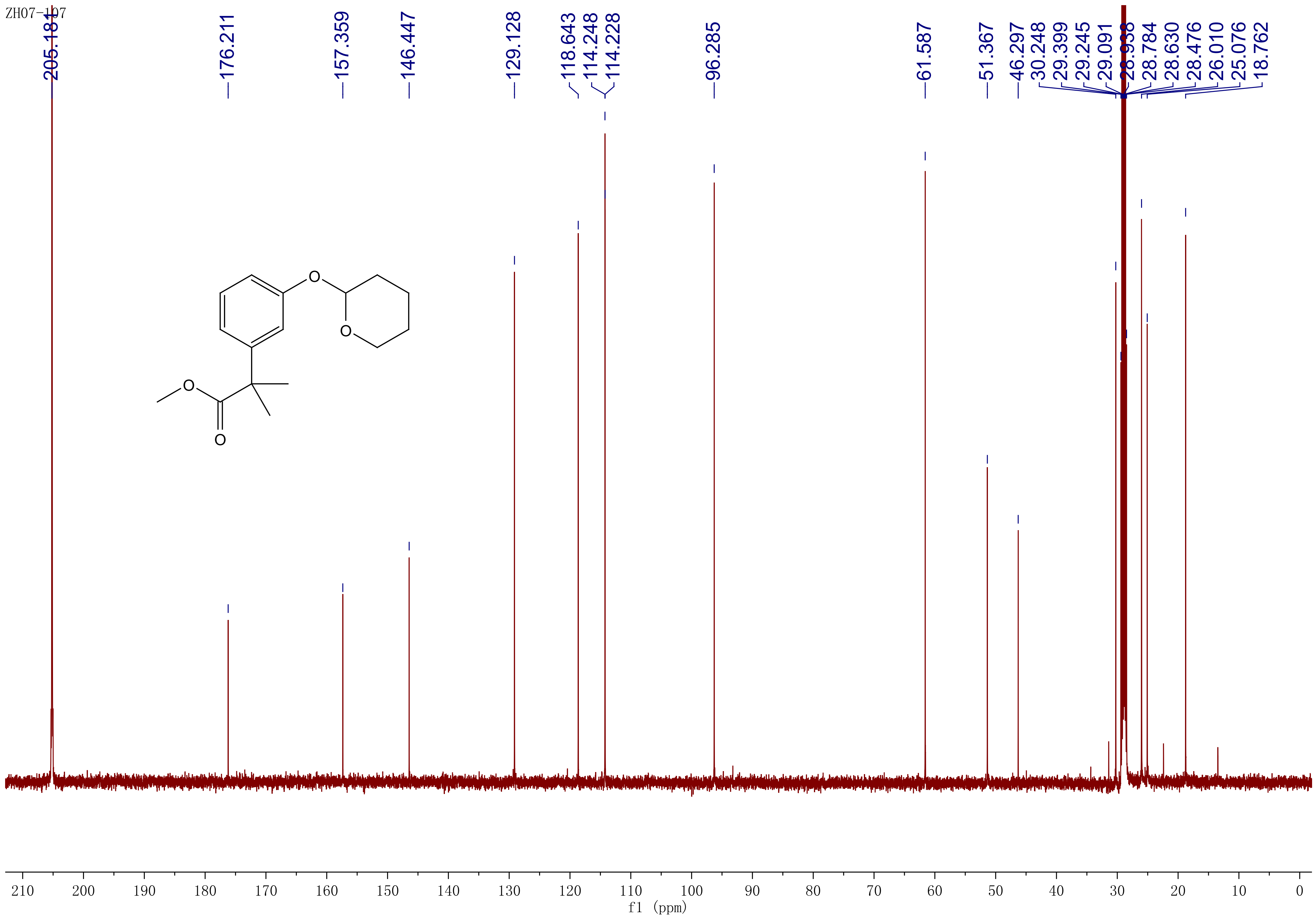
ZH07-107
205.181
176.211
157.359
146.447
129.128
118.643
114.248
114.228
96.285
61.587
51.367
46.297
30.248
29.399
29.245
29.091
28.938
28.784
28.630
28.476
26.010
25.076
18.762
O
O
O
O
210 200 190 180 170 160 150 140 130 120 110 100 90 80 70 60 50 40 30 20 10 0
f1 (ppm)

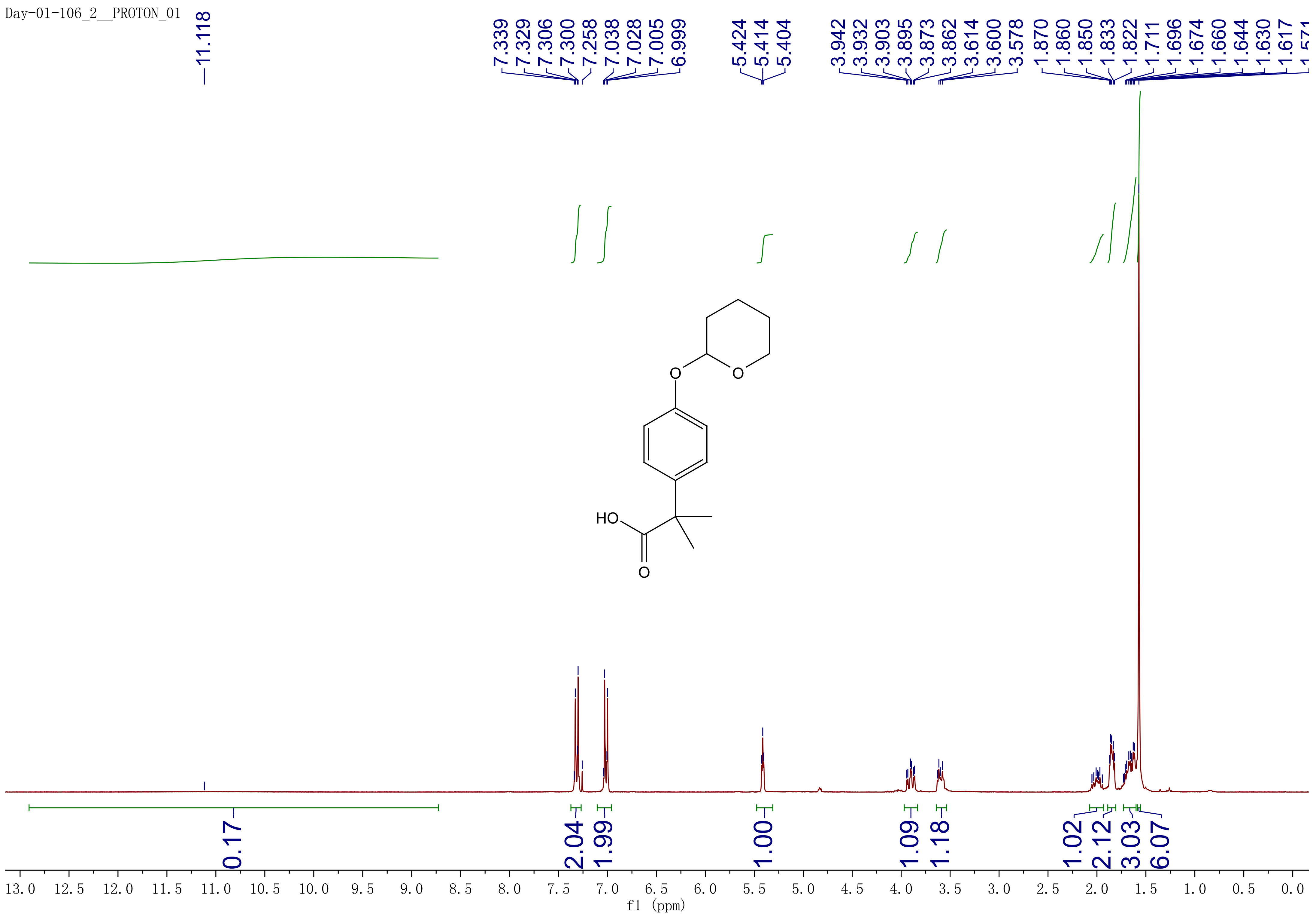

Day-01-106_2__PROTON_01
f1 (ppm)

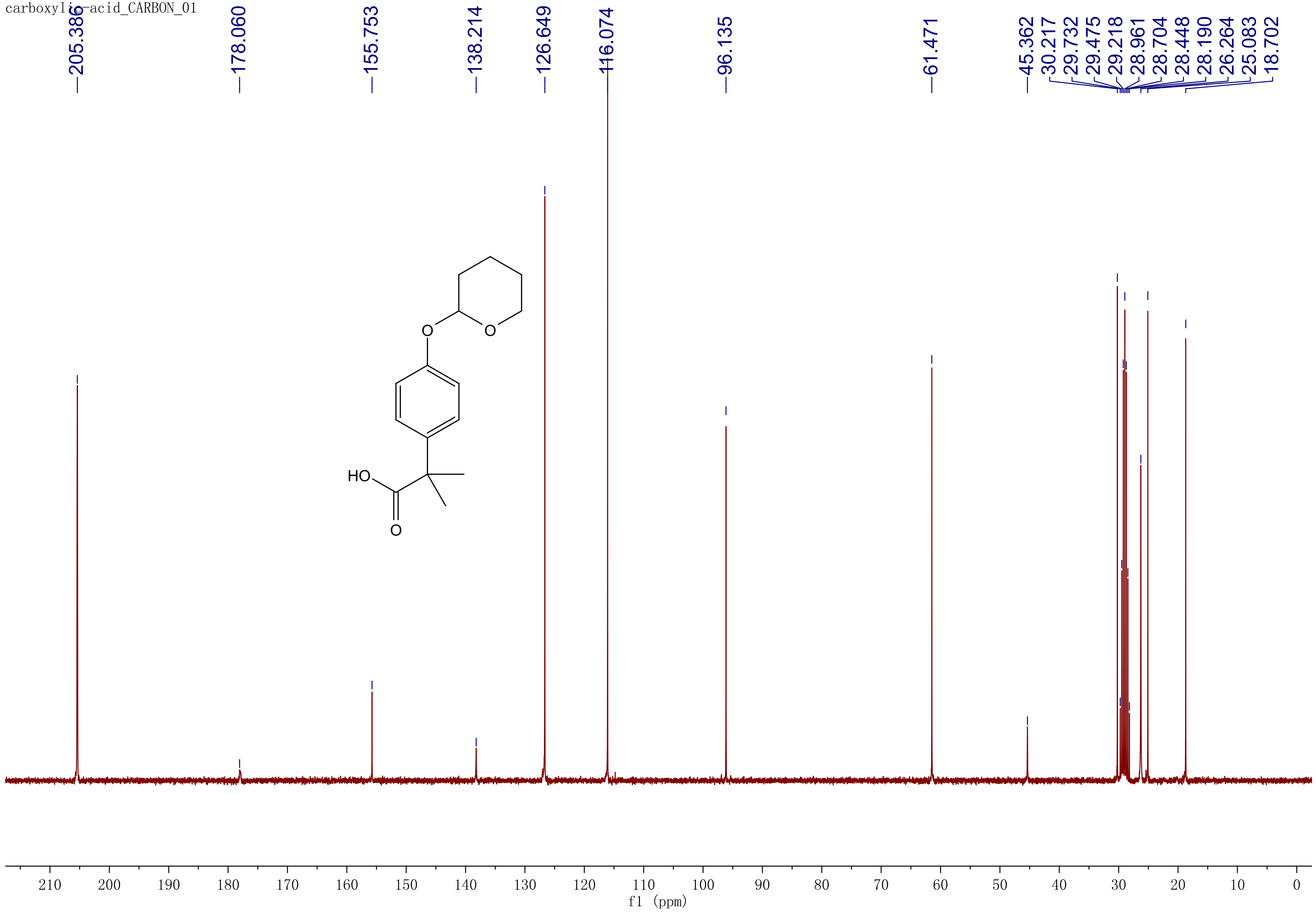
carboxylic-acid_CARBON_01
205.386
178.060
155.753
138.214
126.649
116.074
96.135
61.471
45.362
30.217
29.732
29.475
29.218
28.961
28.704
28.448
28.190
26.264
25.083
18.702
O
O
HO
O
210 200 190 180 170 160 150 140 130 120 110 100 90 80 70 60 50 40 30 20 10 0
f1 (ppm)

ZH03-56
STANDARD PROTON PARAMETERS

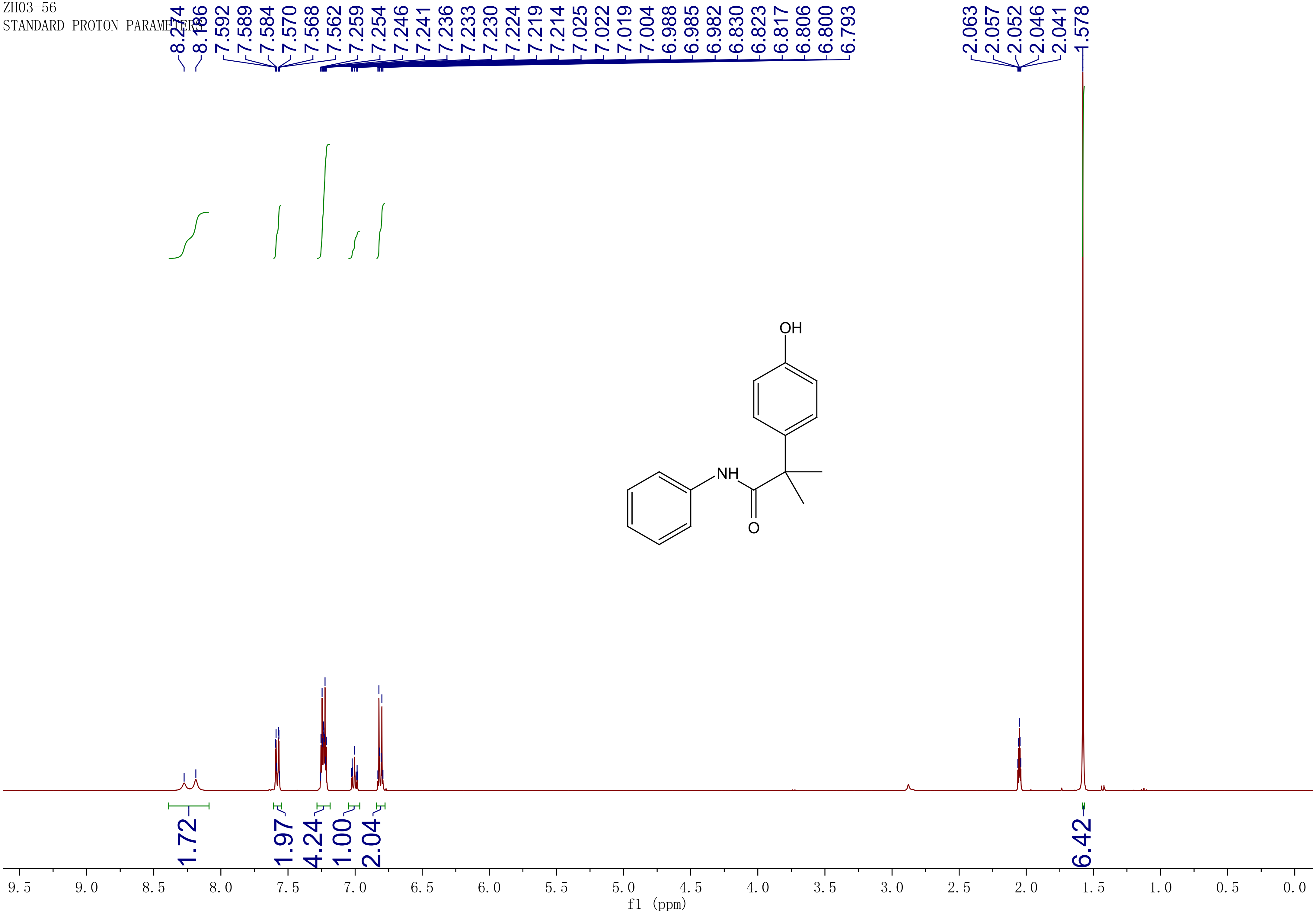

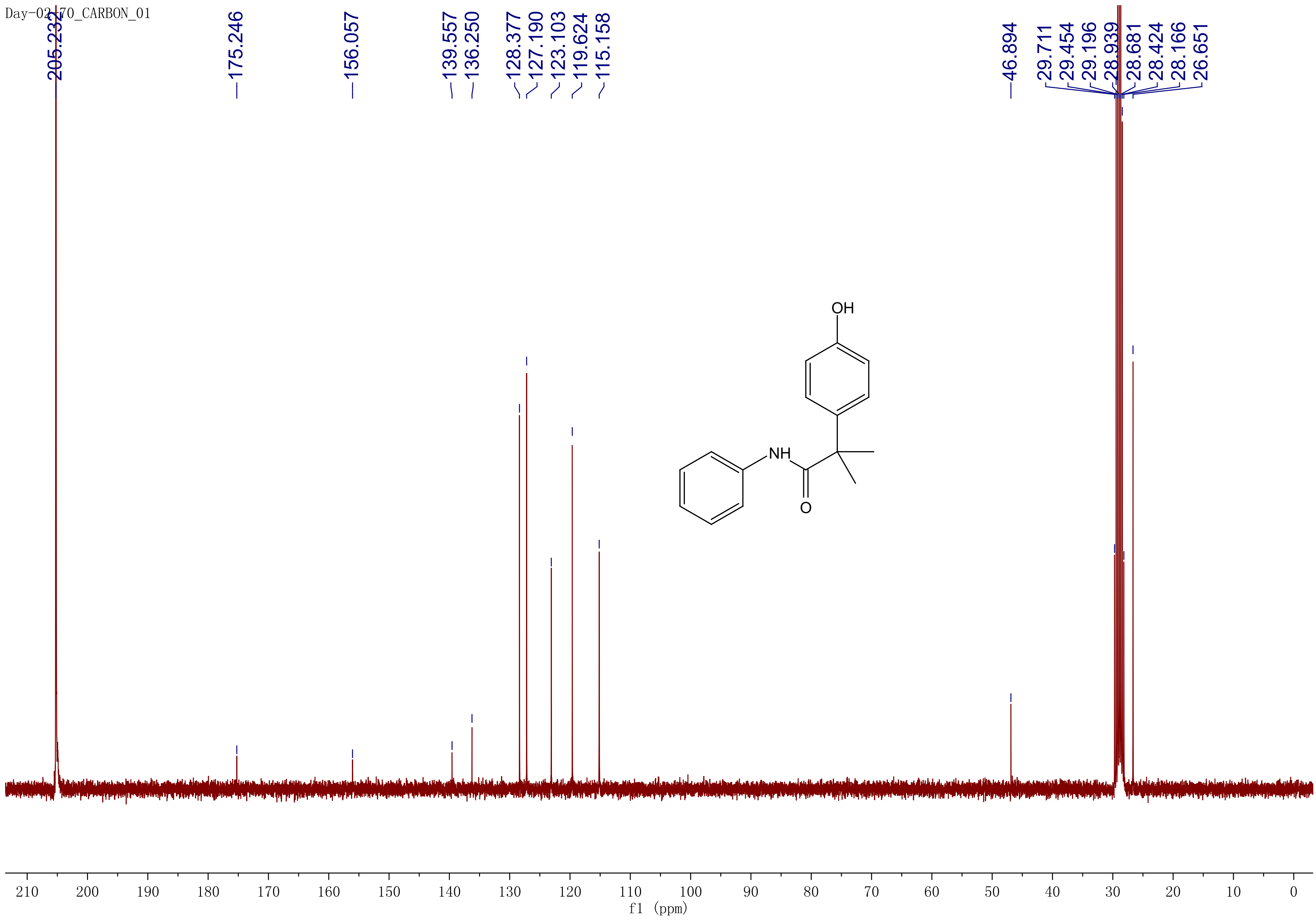

Day-02-70_CARBON_01
205.232
175.246
156.057
139.557
136.250
128.377
127.190
123.103
119.624
115.158
46.894
29.711
29.454
29.196
28.939
28.681
28.424
28.166
26.651
OH
NH
O
210 200 190 180 170 160 150 140 130 120 110 100 90 80 70 60 50 40 30 20 10 0
f1 (ppm)

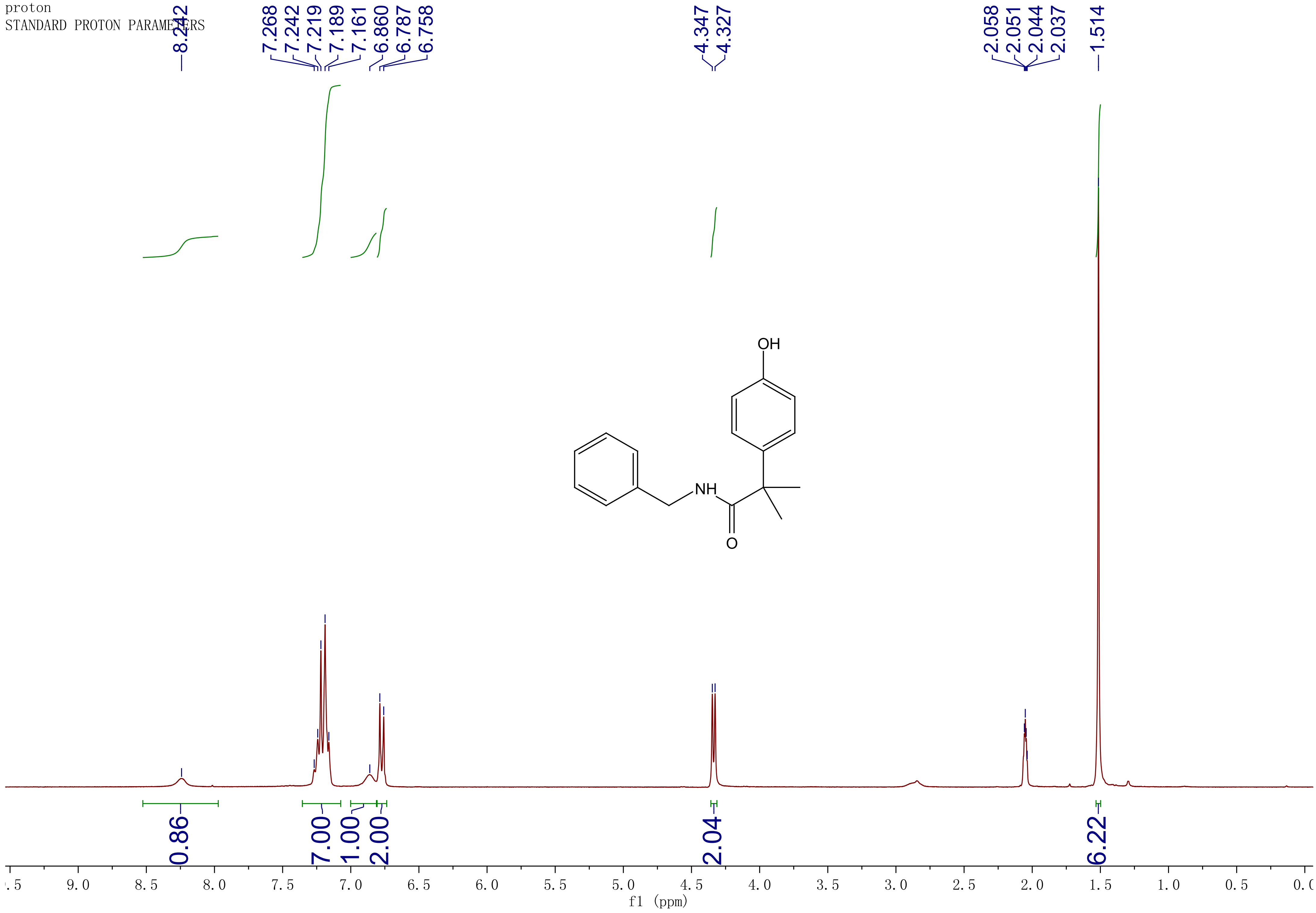
proton
STANDARD PROTON PARAMETERS
8.242
7.268
7.242
7.219
7.189
7.161
6.860
6.787
6.758
4.347
4.327
2.058
2.051
2.044
2.037
1.514
OH
NH
O
0.86
7.00
1.00
2.00
2.04
6.22
9.0
8.5
8.0
7.5
7.0
6.5
6.0
5.5
5.0
4.5
4.0
3.5
3.0
2.5
2.0
1.5
1.0
0.5
f1 (ppm)

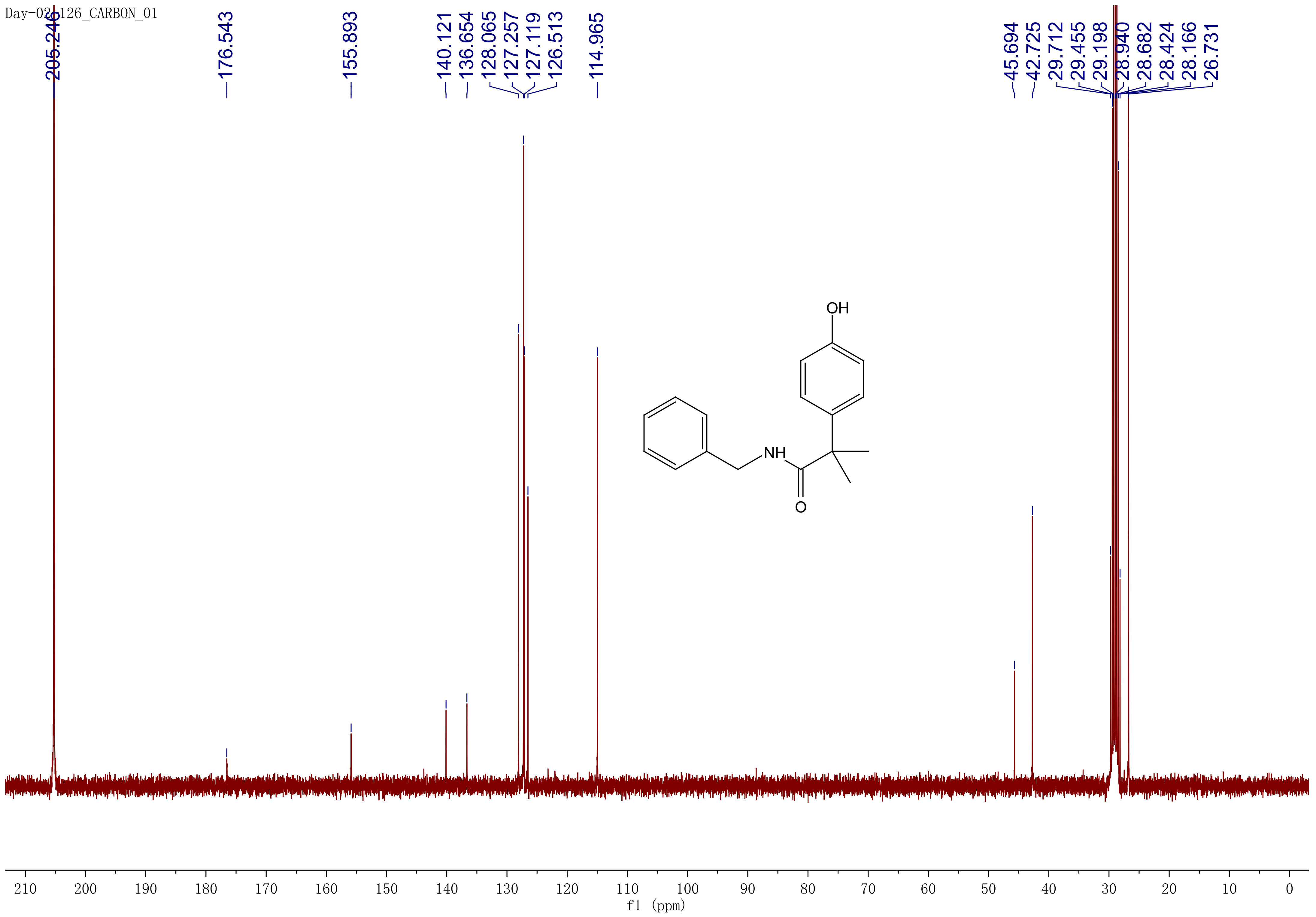

Day-02-126_CARBON_01
205.246
176.543
155.893
140.121
136.654
128.065
127.257
127.119
126.513
114.965
45.694
42.725
29.712
29.455
29.198
28.940
28.682
28.424
28.166
26.731
OH
NH
O
210 200 190 180 170 160 150 140 130 120 110 100 90 80 70 60 50 40 30 20 10 0
f1 (ppm)

Day-02-134
STANDARD PROTON PARAMETERS

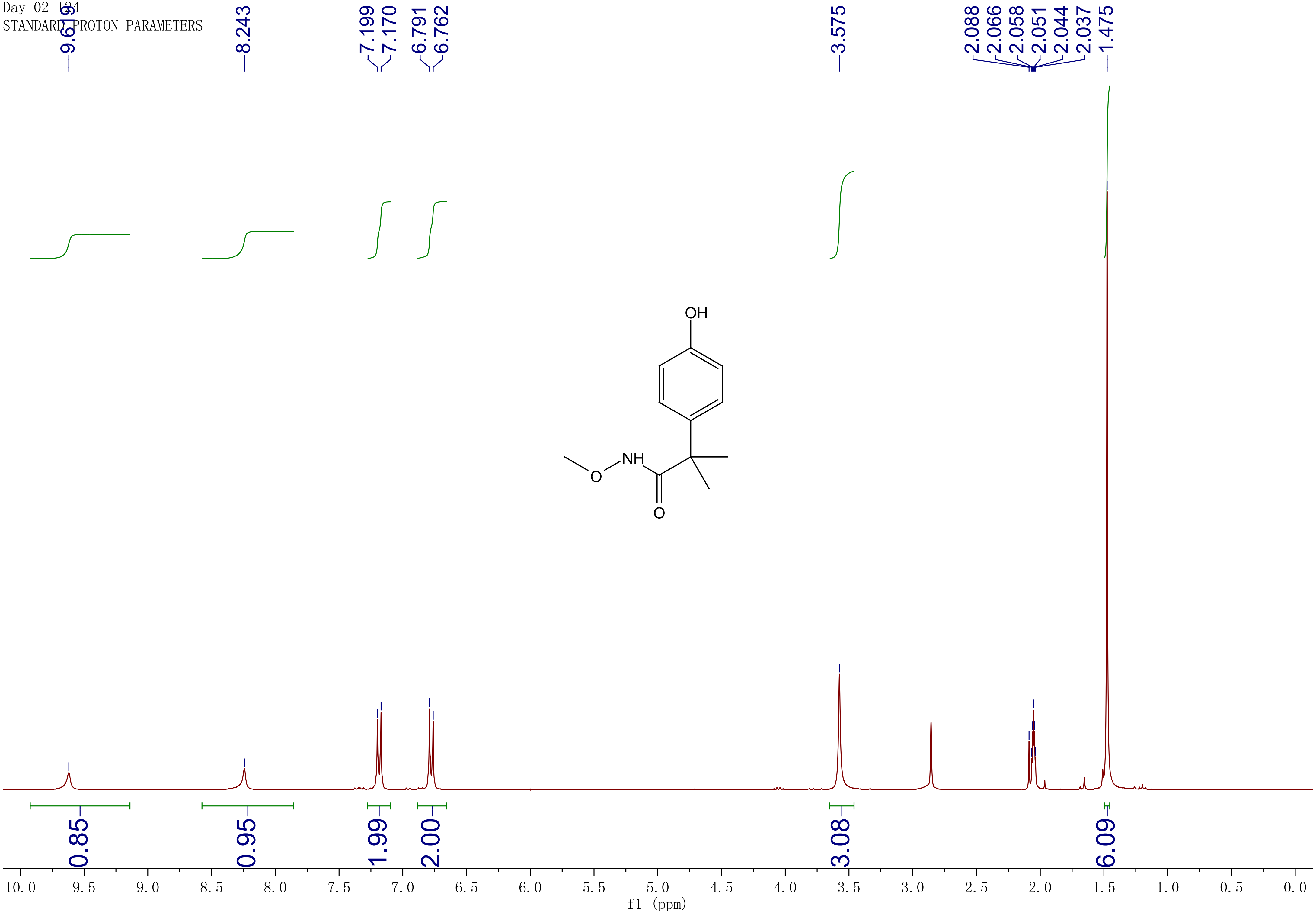

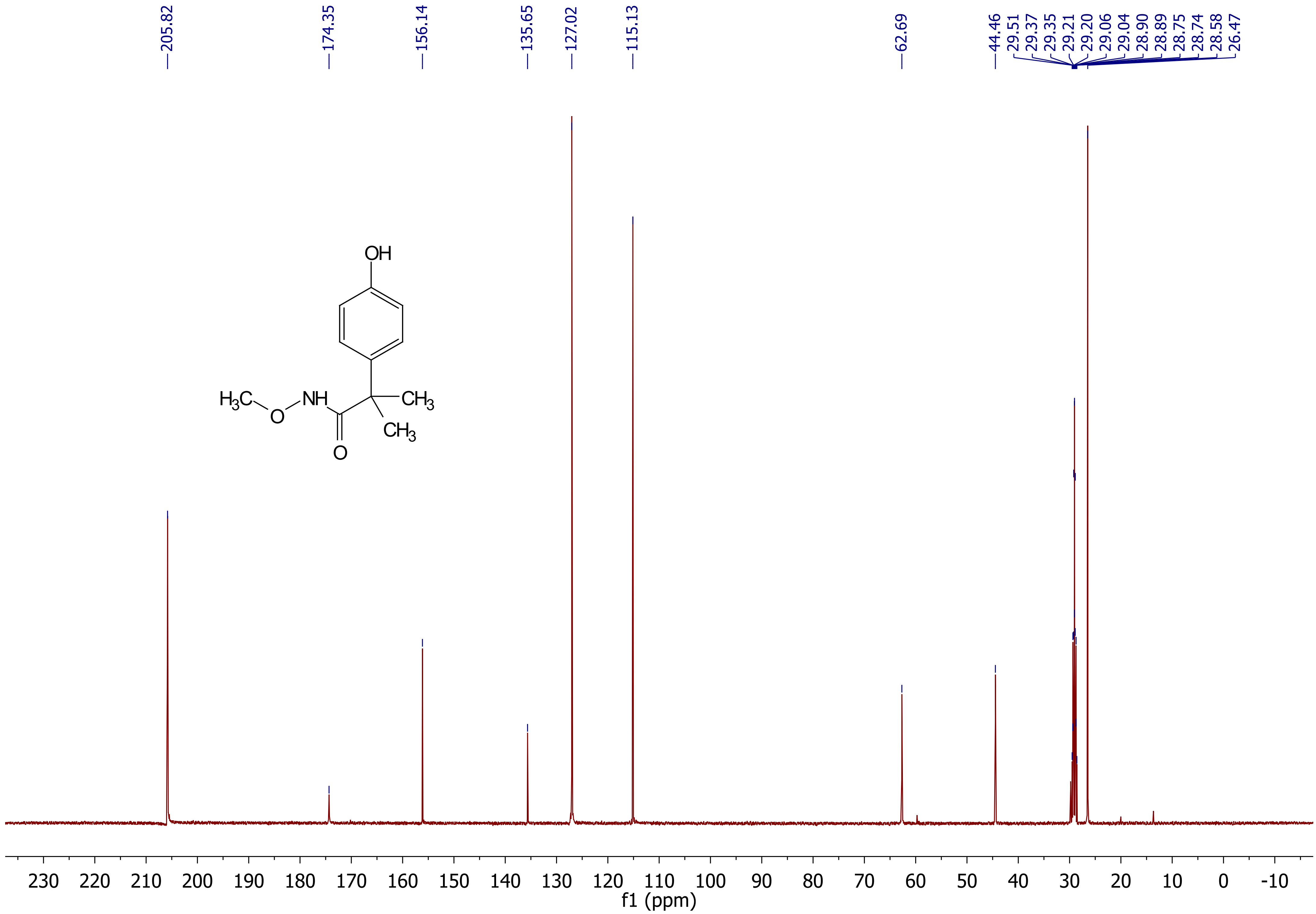

205.82
174.35
156.14
135.65
127.02
115.13
62.69
44.46
29.51
29.37
29.35
29.21
29.20
29.06
29.04
28.90
28.89
28.75
28.74
28.58
26.47
OH
$H_3C$
O
NH
$CH_3$
$CH_3$
O
230 220 210 200 190 180 170 160 150 140 130 120 110 100 90 80 70 60 50 40 30 20 10 0 -10
f1 (ppm)

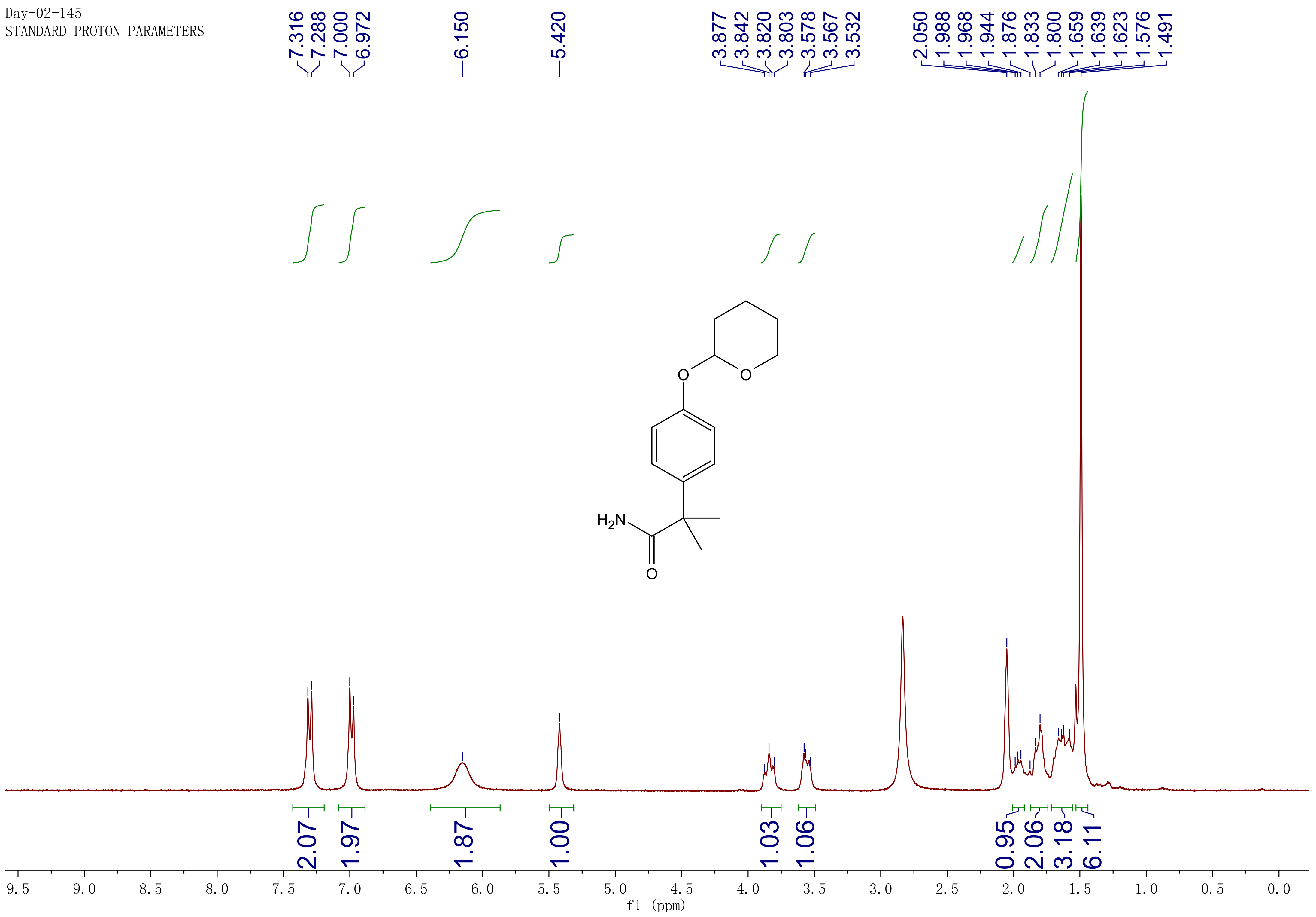
Day-02-145
STANDARD PROTON PARAMETERS
7.316
7.288
7.000
6.972
6.150
5.420
3.877
3.842
3.820
3.803
3.578
3.567
3.532
2.050
1.988
1.968
1.944
1.876
1.833
1.800
1.659
1.639
1.623
1.576
1.491
O
O
$H_2N$
O
2.07
1.97
1.87
1.00
1.03
1.06
0.95
2.06
3.18
6.11
9.5
9.0
8.5
8.0
7.5
7.0
6.5
6.0
5.5
5.0
4.5
4.0
3.5
3.0
2.5
2.0
1.5
1.0
0.5
0.0
f1 (ppm)

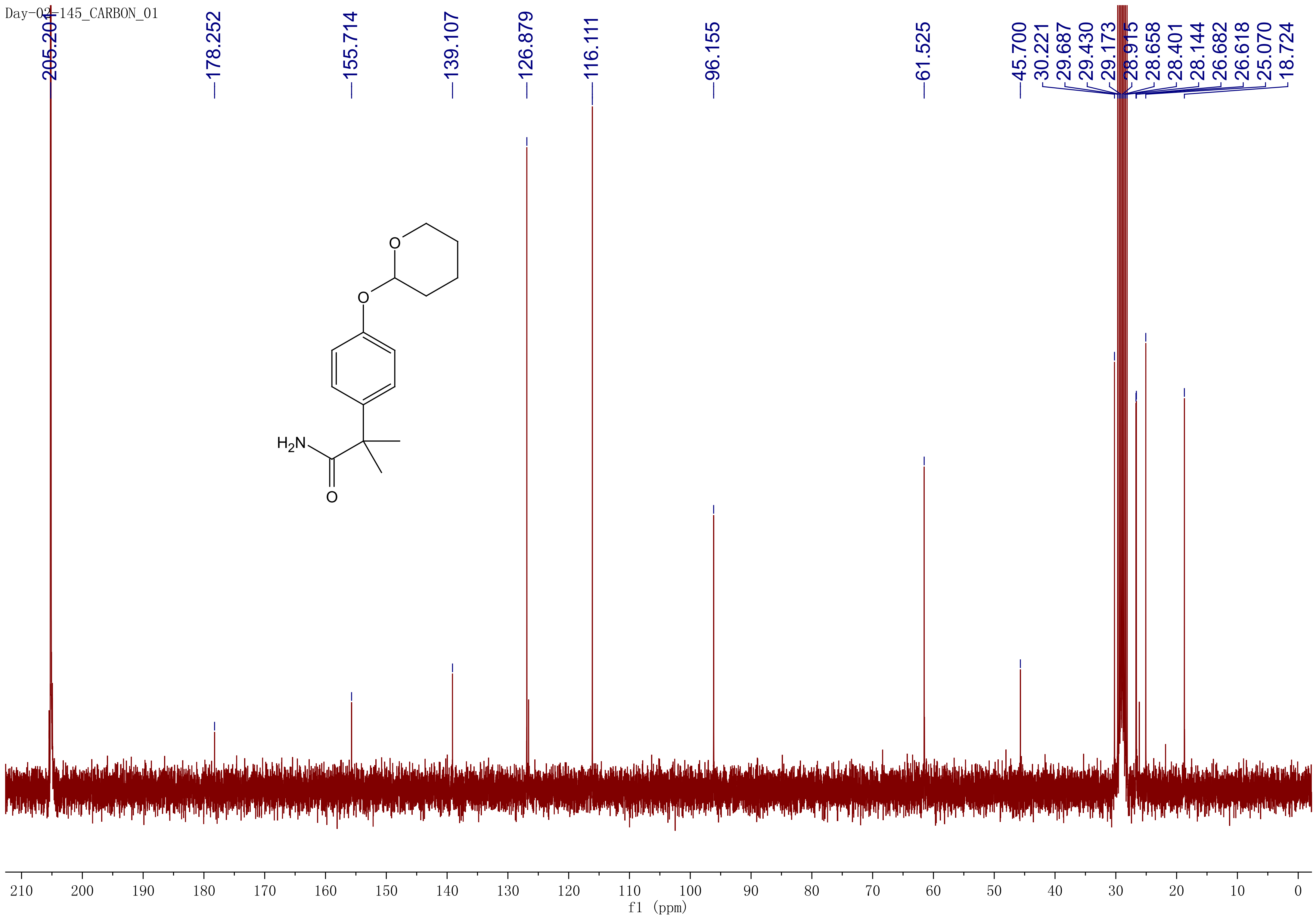
Day-02-145_CARBON_01
205.201
178.252
155.714
139.107
126.879
116.111
96.155
61.525
45.700
30.221
29.687
29.430
29.173
28.915
28.658
28.401
28.144
26.682
26.618
25.070
18.724
O
O
H2N
O
210 200 190 180 170 160 150 140 130 120 110 100 90 80 70 60 50 40 30 20 10 0
f1 (ppm)

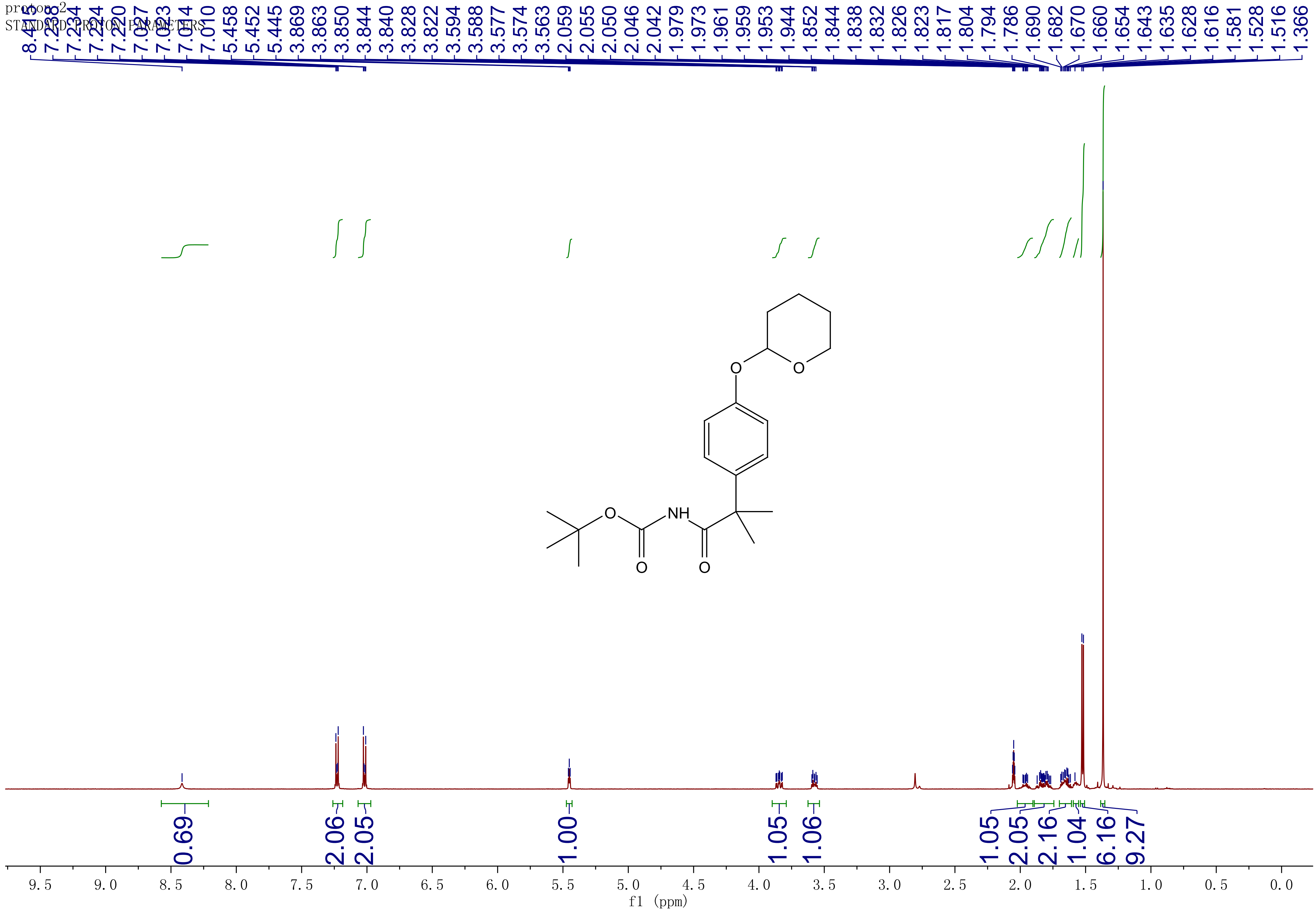
proton-2
STANDARD PROTON PARAMETERS
8.415
7.238
7.234
7.224
7.220
7.027
7.023
7.014
7.010
5.458
5.452
5.445
3.869
3.863
3.850
3.844
3.840
3.828
3.822
3.594
3.588
3.577
3.574
3.563
2.059
2.055
2.050
2.046
2.042
1.979
1.973
1.961
1.959
1.953
1.944
1.852
1.844
1.838
1.832
1.826
1.823
1.817
1.804
1.794
1.786
1.690
1.682
1.670
1.660
1.654
1.643
1.635
1.628
1.616
1.581
1.528
1.516
1.366
O
O
O
O
NH
O
O
0.69
2.06
2.05
1.00
1.05
1.06
1.05
2.05
2.16
1.04
6.16
9.27
9.5
9.0
8.5
8.0
7.5
7.0
6.5
6.0
5.5
5.0
4.5
4.0
3.5
3.0
2.5
2.0
1.5
1.0
0.5
0.0
f1 (ppm)

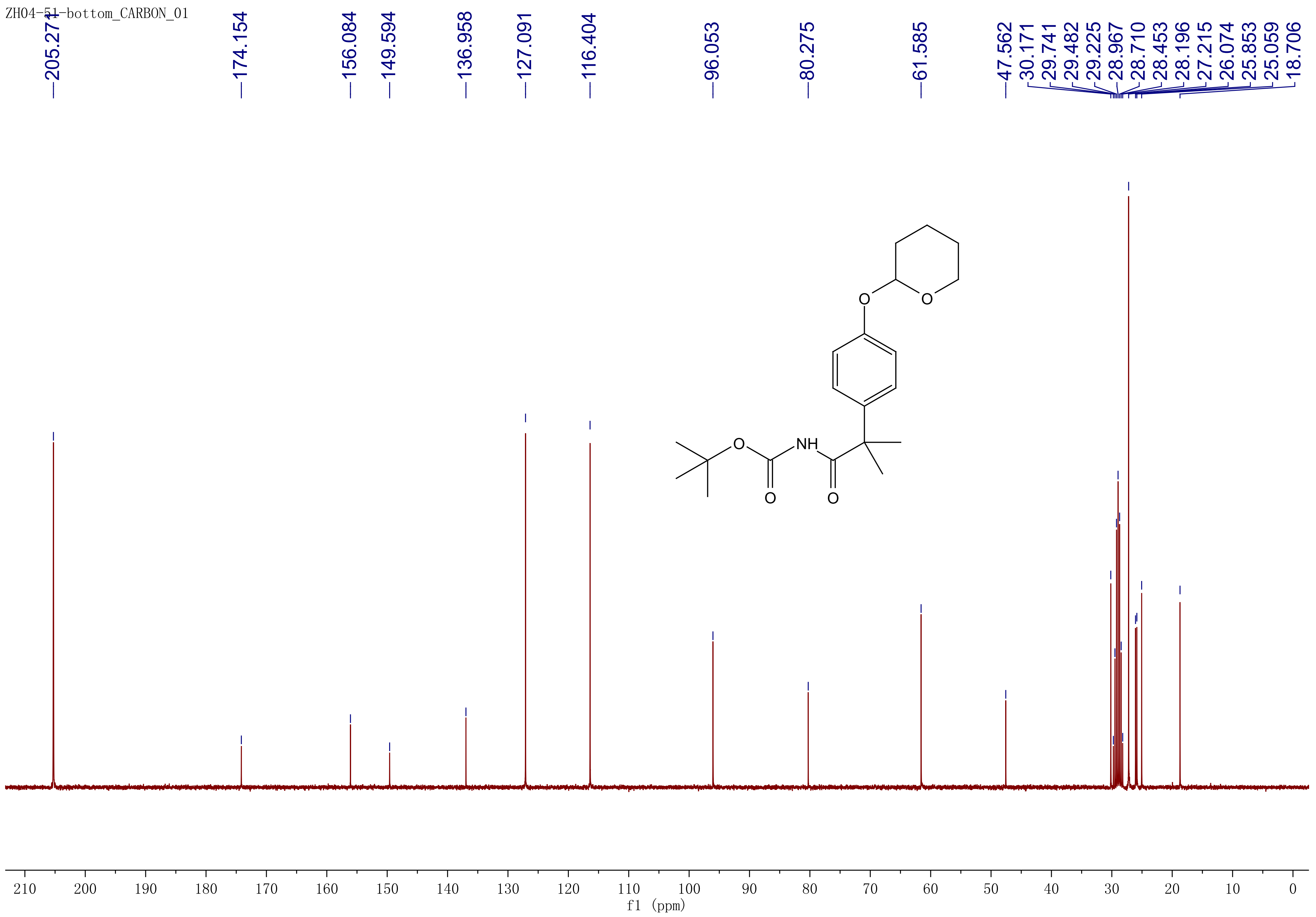
ZH04-51-bottom_CARBON_01
205.271
174.154
156.084
149.594
136.958
127.091
116.404
96.053
80.275
61.585
47.562
30.171
29.741
29.482
29.225
28.967
28.710
28.453
28.196
27.215
26.074
25.853
25.059
18.706
O
O
NH
O
O
O
O
210 200 190 180 170 160 150 140 130 120 110 100 90 80 70 60 50 40 30 20 10 0
f1 (ppm)

ZH04-61

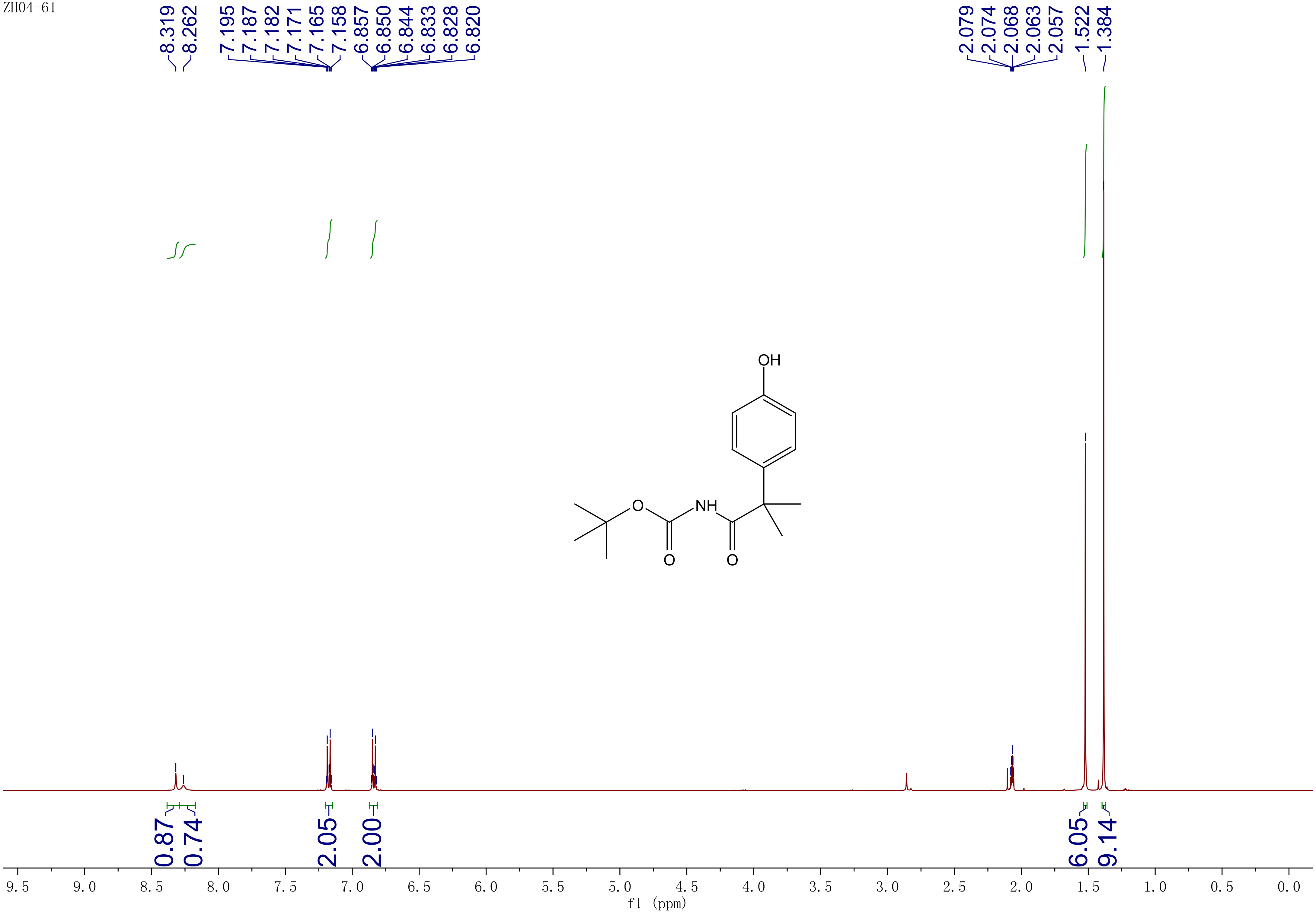

8.319
8.262
7.195
7.187
7.182
7.171
7.165
7.158
6.857
6.850
6.844
6.833
6.828
6.820
2.079
2.074
2.068
2.063
2.057
1.522
1.384
OH
O
NH
O
O
0.87
0.74
2.05
2.00
6.05
9.14
9.5
9.0
8.5
8.0
7.5
7.0
6.5
6.0
5.5
5.0
4.5
4.0
3.5
3.0
2.5
2.0
1.5
1.0
0.5
0.0
f1 (ppm)

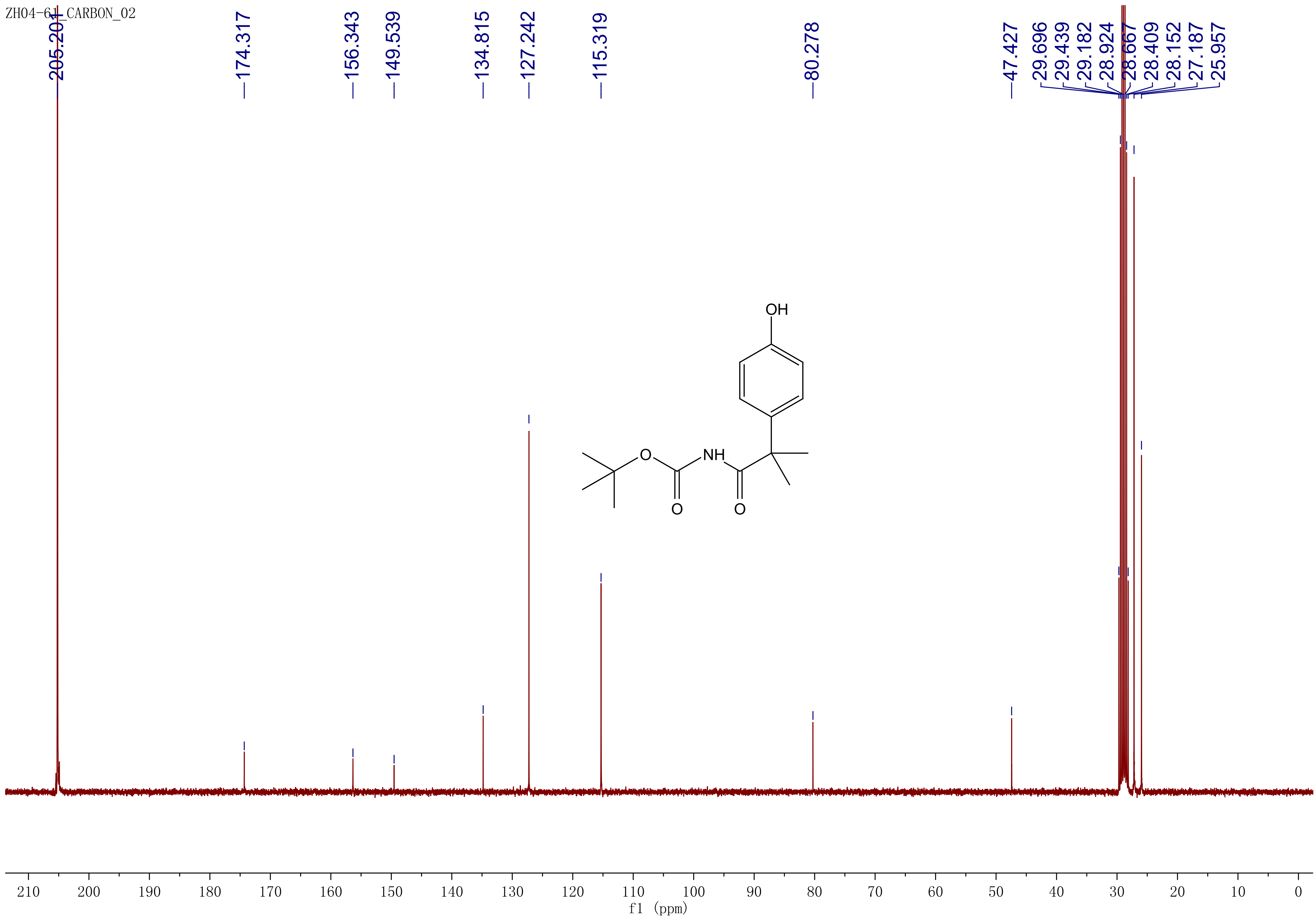
ZH04-61_CARBON_02
205.204
174.317
156.343
149.539
134.815
127.242
115.319
80.278
47.427
29.696
29.439
29.182
28.924
28.667
28.409
28.152
27.187
25.957
OH
O
NH
O
O
210 200 190 180 170 160 150 140 130 120 110 100 90 80 70 60 50 40 30 20 10 0
f1 (ppm)

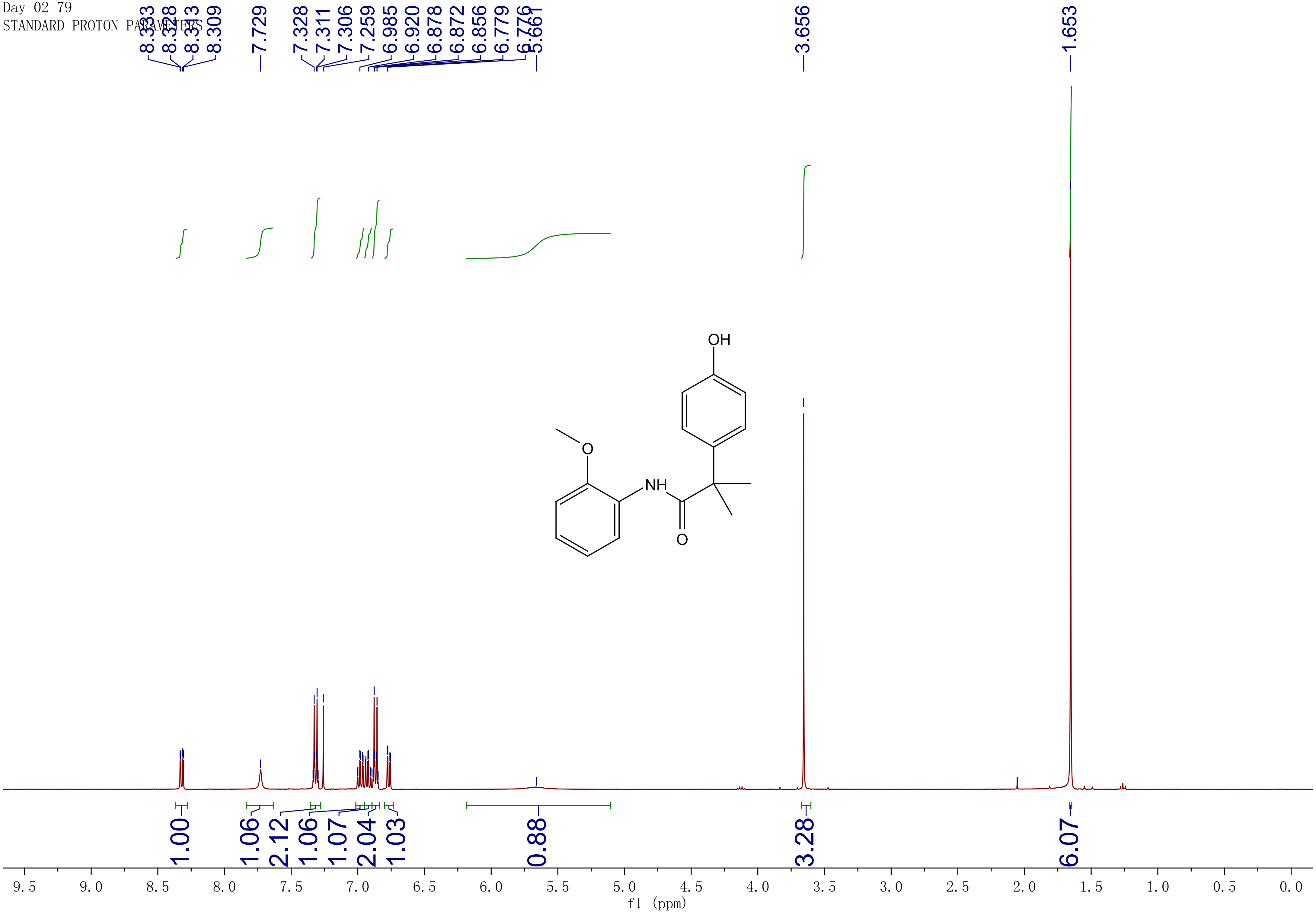

Day-02-79
STANDARD PROTON PARAMETERS
8.333
8.328
8.313
8.309
7.729
7.328
7.311
7.306
7.259
6.985
6.920
6.878
6.872
6.856
6.779
6.776
5.661
3.656
1.653
OH
O
NH
O
1.00
1.06
2.12
1.06
1.07
2.04
1.03
0.88
3.28
6.07
9.5
9.0
8.5
8.0
7.5
7.0
6.5
6.0
5.5
5.0
4.5
4.0
3.5
3.0
2.5
2.0
1.5
1.0
0.5
0.0
f1 (ppm)

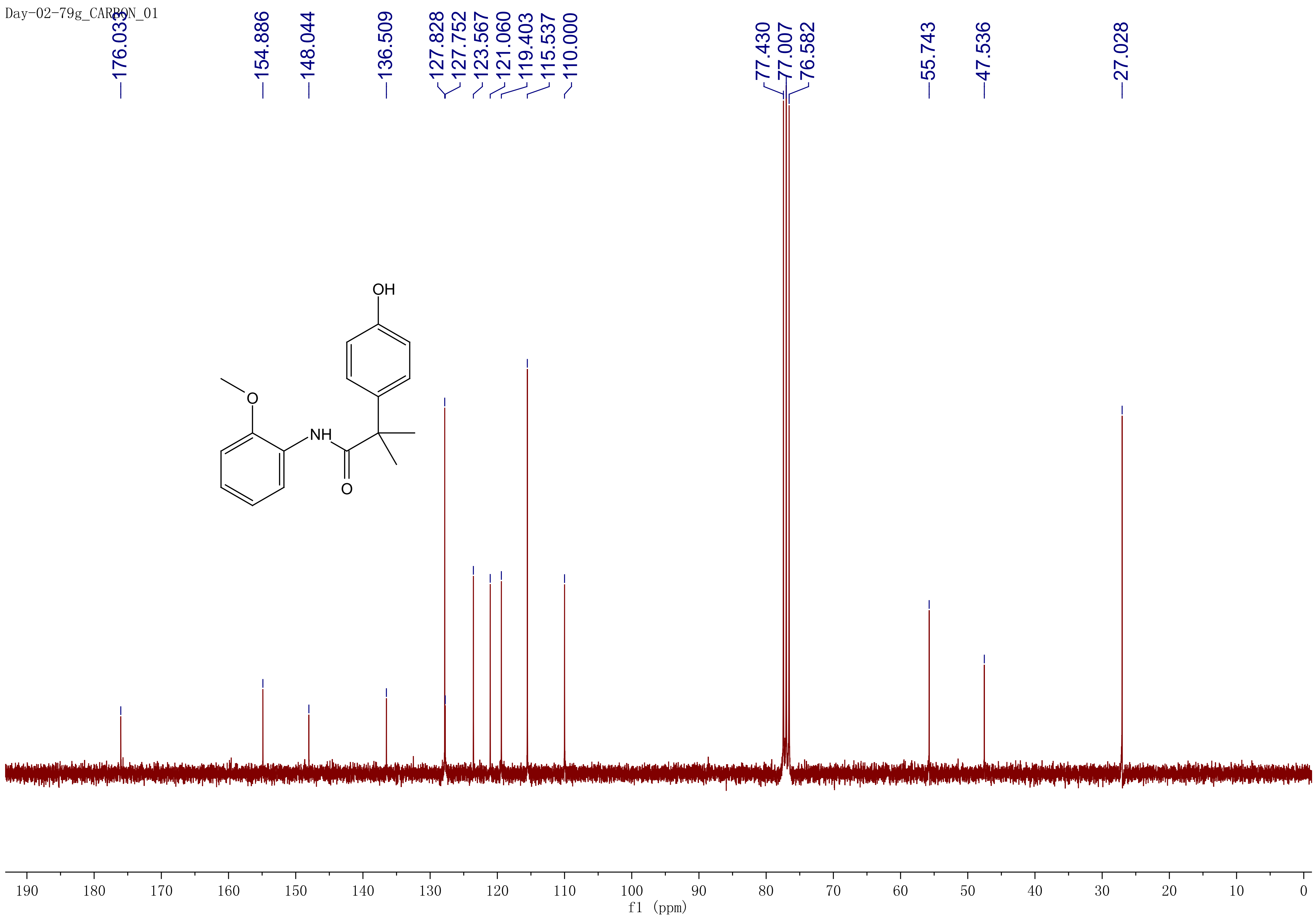
Day-02-79g_CARBON_01
176.033
154.886
148.044
136.509
127.828
127.752
123.567
121.060
119.403
115.537
110.000
77.430
77.007
76.582
55.743
47.536
27.028
OH
O
NH
O
190 180 170 160 150 140 130 120 110 100 90 80 70 60 50 40 30 20 10 0
f1 (ppm)

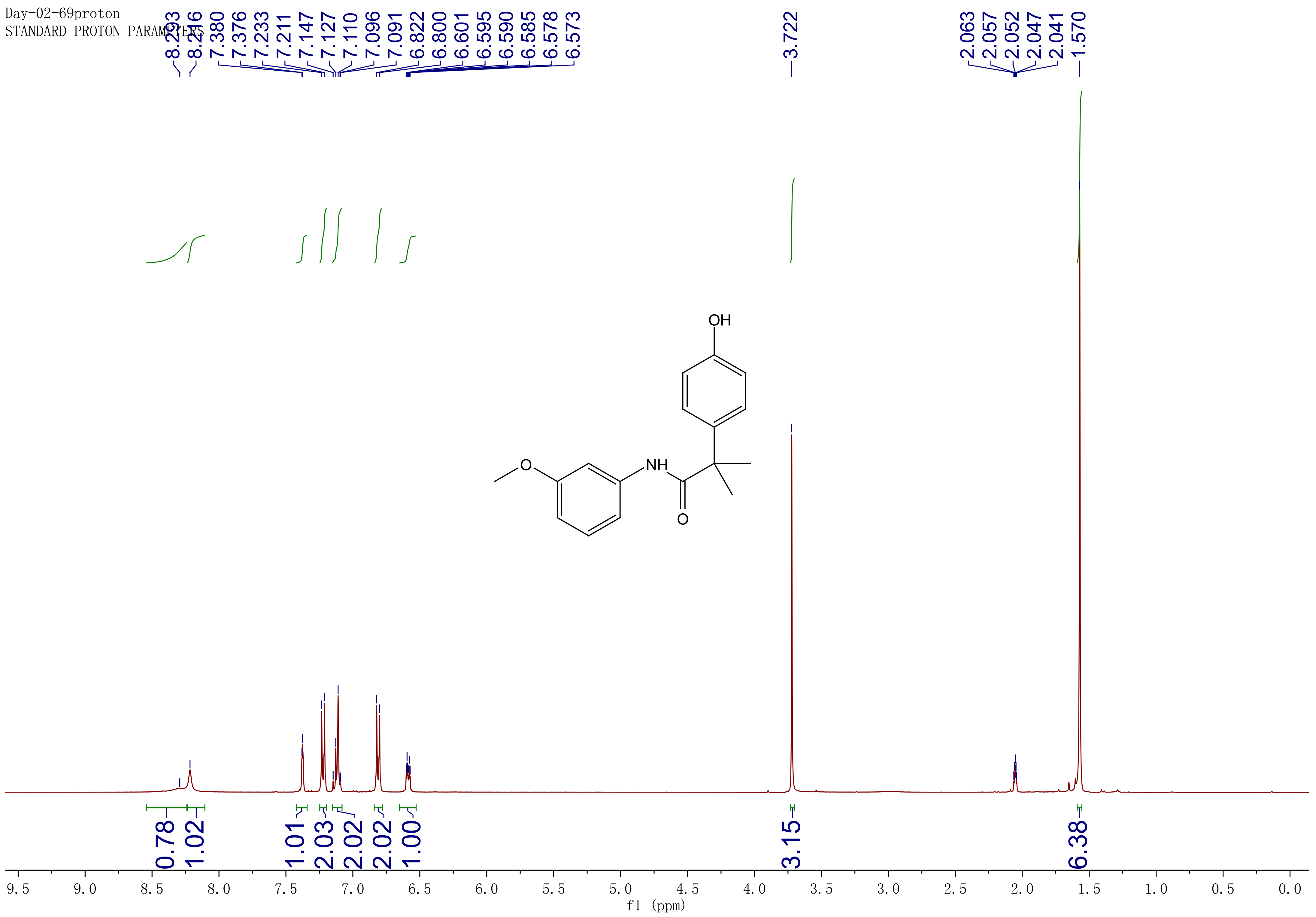

Day-02-69proton
STANDARD PROTON PARAMETERS
8.293
8.216
7.380
7.376
7.233
7.211
7.147
7.127
7.110
7.096
7.091
6.822
6.800
6.601
6.595
6.590
6.585
6.578
6.573
3.722
2.063
2.057
2.052
2.047
2.041
1.570
OH
O
NH
O
0.78
1.02
1.01
2.03
2.02
2.02
1.00
3.15
6.38
9.5 9.0 8.5 8.0 7.5 7.0 6.5 6.0 5.5 5.0 4.5 4.0 3.5 3.0 2.5 2.0 1.5 1.0 0.5 0.0
f1 (ppm)

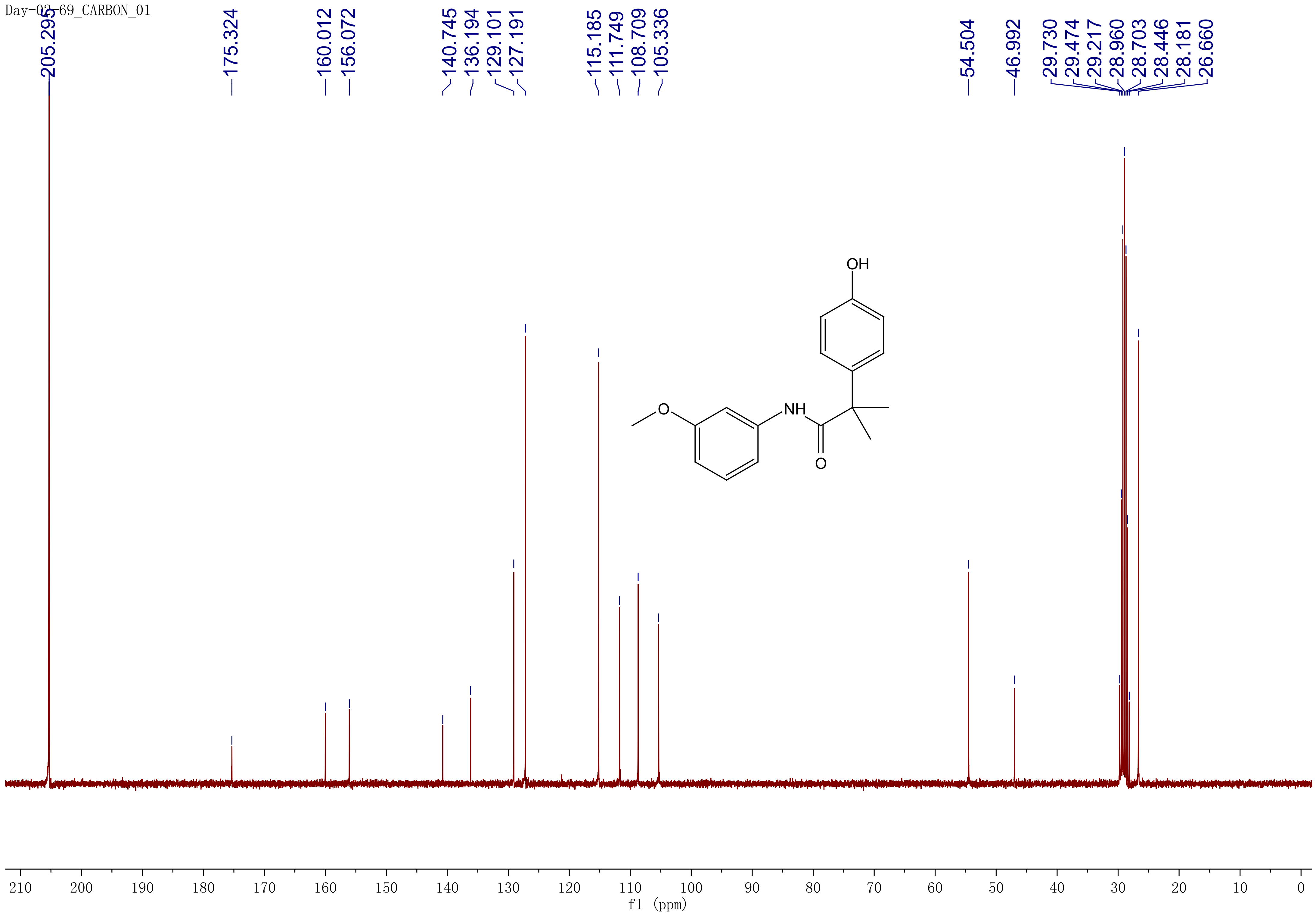
Day-02-69_CARBON_01
205.295
175.324
160.012
156.072
140.745
136.194
129.101
127.191
115.185
111.749
108.709
105.336
54.504
46.992
29.730
29.474
29.217
28.960
28.703
28.446
28.181
26.660
OH
NH
O
O
210 200 190 180 170 160 150 140 130 120 110 100 90 80 70 60 50 40 30 20 10 0
f1 (ppm)

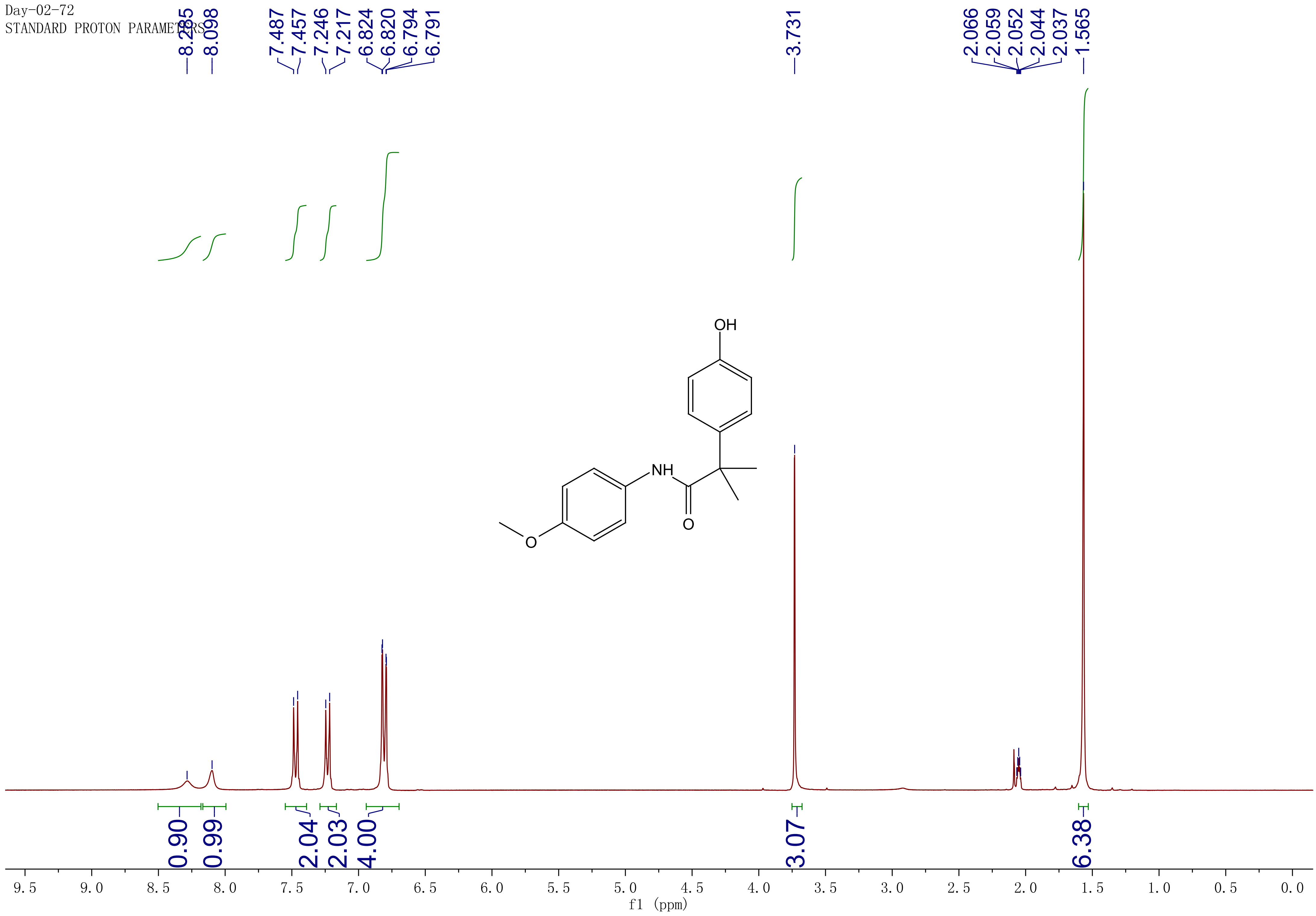

Day-02-72
STANDARD PROTON PARAMETERS
8.285
8.098
7.487
7.457
7.246
7.217
6.824
6.820
6.794
6.791
3.731
2.066
2.059
2.052
2.044
2.037
1.565
OH
NH
O
O
0.90
0.99
2.04
2.03
4.00
3.07
6.38
9.5 9.0 8.5 8.0 7.5 7.0 6.5 6.0 5.5 5.0 4.5 4.0 3.5 3.0 2.5 2.0 1.5 1.0 0.5 0.0
f1 (ppm)

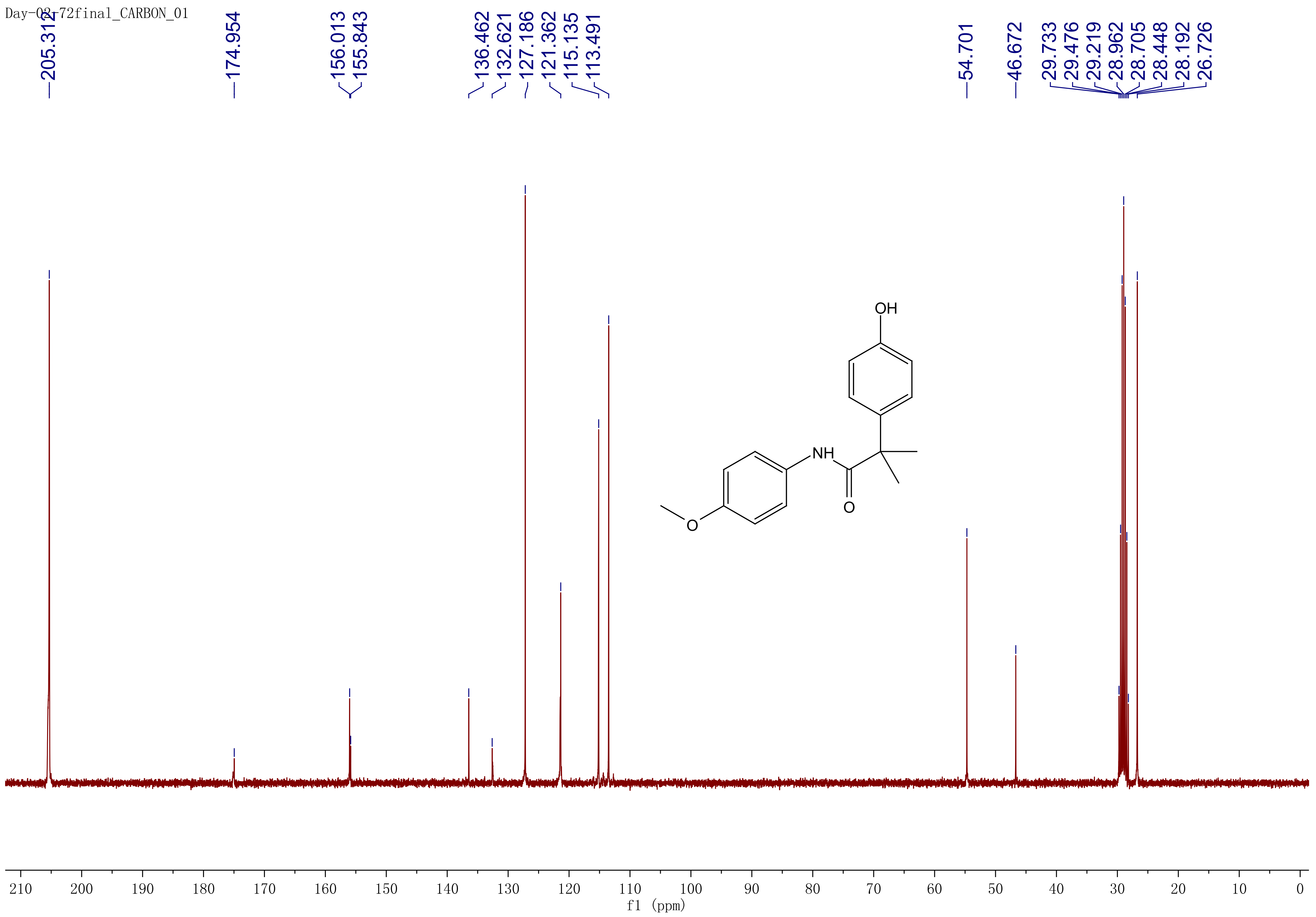
Day-02-72final_CARBON_01
205.312
174.954
156.013
155.843
136.462
132.621
127.186
121.362
115.135
113.491
54.701
46.672
29.733
29.476
29.219
28.962
28.705
28.448
28.192
26.726
OH
NH
O
O
210 200 190 180 170 160 150 140 130 120 110 100 90 80 70 60 50 40 30 20 10 0
f1 (ppm)

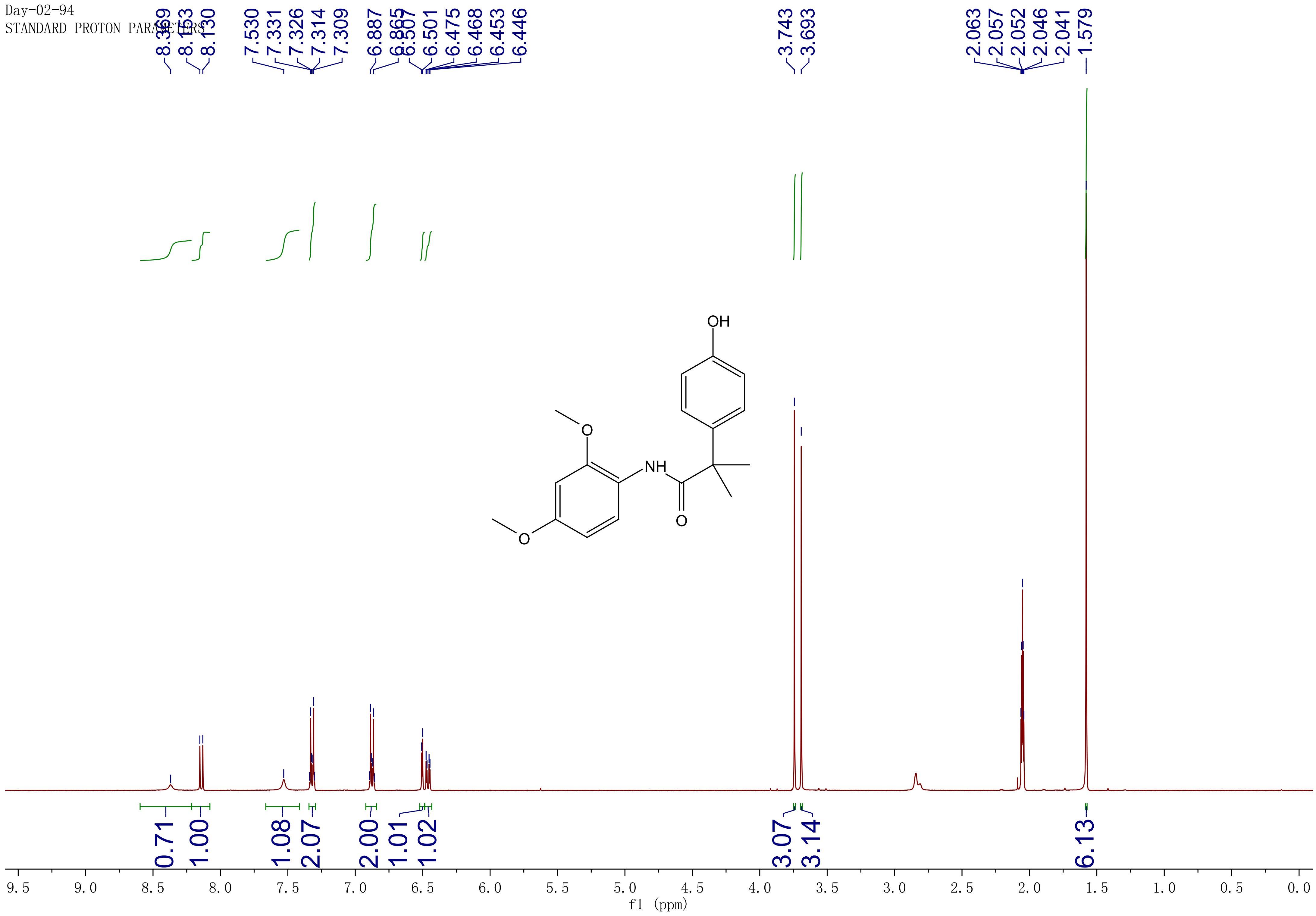
Day-02-94
STANDARD PROTON PARAMETERS
8.369
8.153
8.130
7.530
7.331
7.326
7.314
7.309
6.887
6.865
6.507
6.501
6.475
6.468
6.453
6.446
3.743
3.693
2.063
2.057
2.052
2.046
2.041
1.579
OH
O
NH
O
O
0.71
1.00
1.08
2.07
2.00
1.01
1.02
3.07
3.14
6.13
9.5
9.0
8.5
8.0
7.5
7.0
6.5
6.0
5.5
5.0
4.5
4.0
3.5
3.0
2.5
2.0
1.5
1.0
0.5
0.0
f1 (ppm)

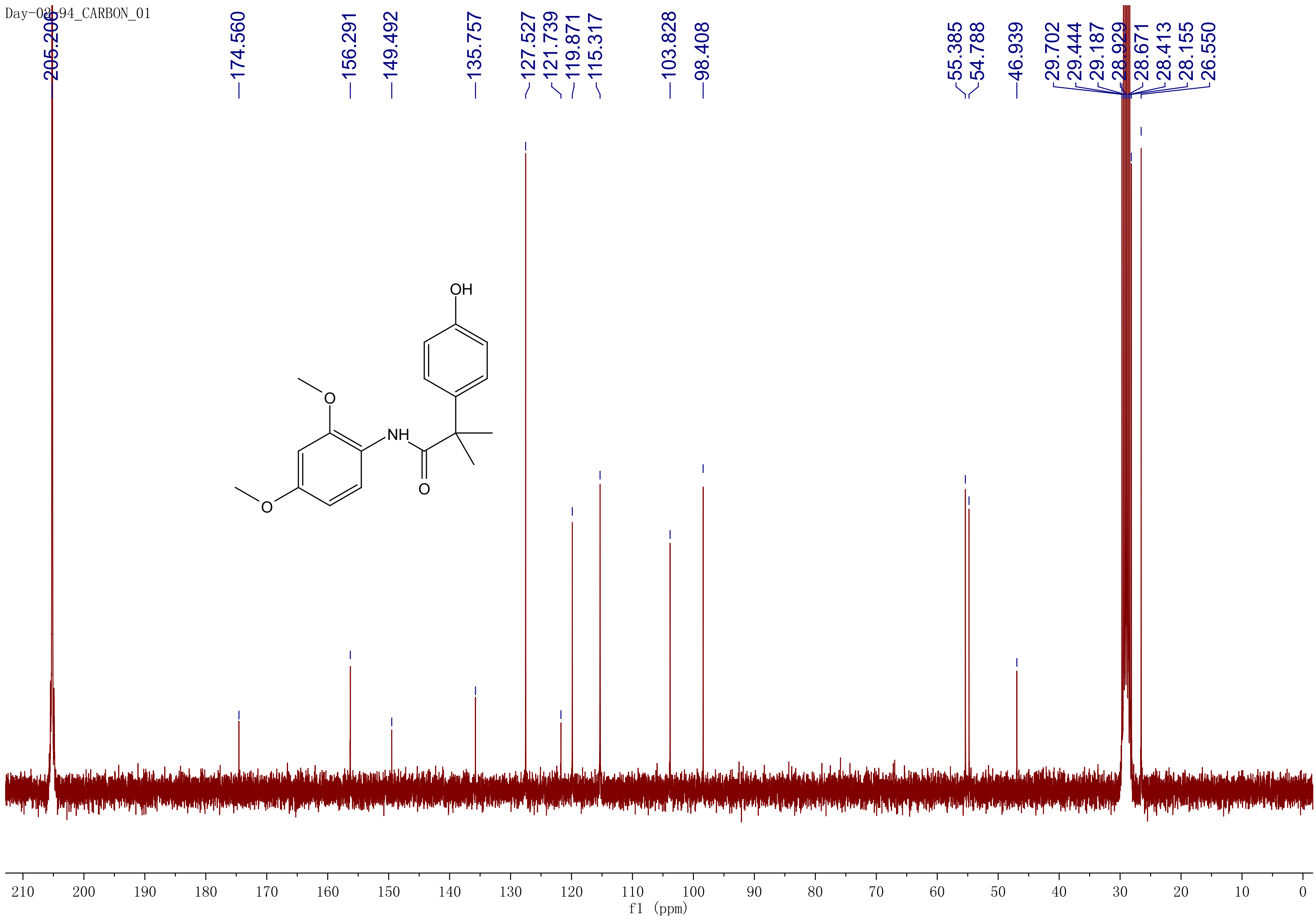
Day-02-94_CARBON_01
205.206
174.560
156.291
149.492
135.757
127.527
121.739
119.871
115.317
103.828
98.408
55.385
54.788
46.939
29.702
29.444
29.187
28.929
28.671
28.413
28.155
26.550
OH
NH
O
O
O
210 200 190 180 170 160 150 140 130 120 110 100 90 80 70 60 50 40 30 20 10 0
f1 (ppm)

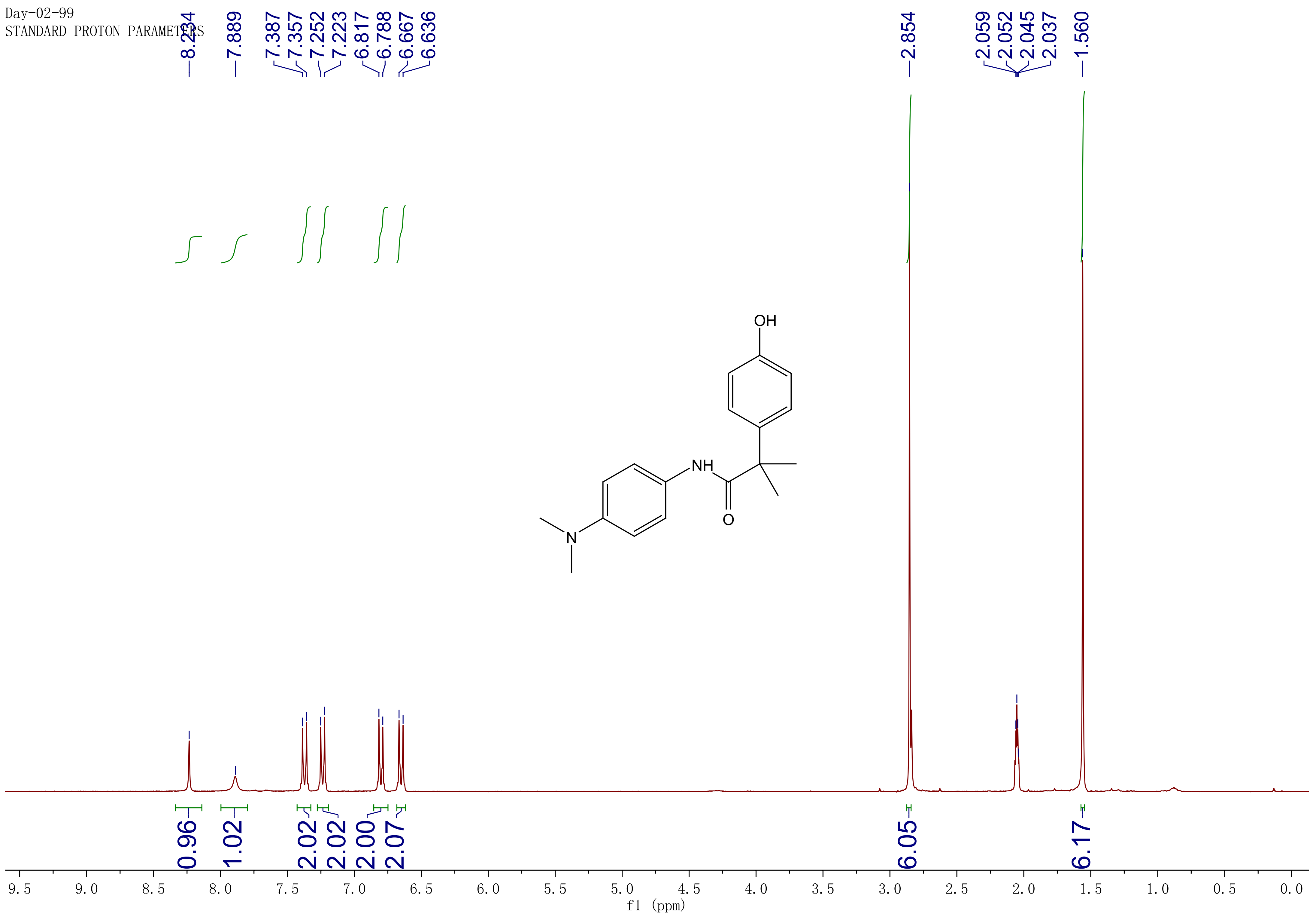
Day-02-99
STANDARD PROTON PARAMETERS
8.234
7.889
7.387
7.357
7.252
7.223
6.817
6.788
6.667
6.636
2.854
2.059
2.052
2.045
2.037
1.560
OH
NH
O
N
0.96
1.02
2.02
2.02
2.00
2.07
6.05
6.17
9.5
9.0
8.5
8.0
7.5
7.0
6.5
6.0
5.5
5.0
4.5
4.0
3.5
3.0
2.5
2.0
1.5
1.0
0.5
0.0
f1 (ppm)

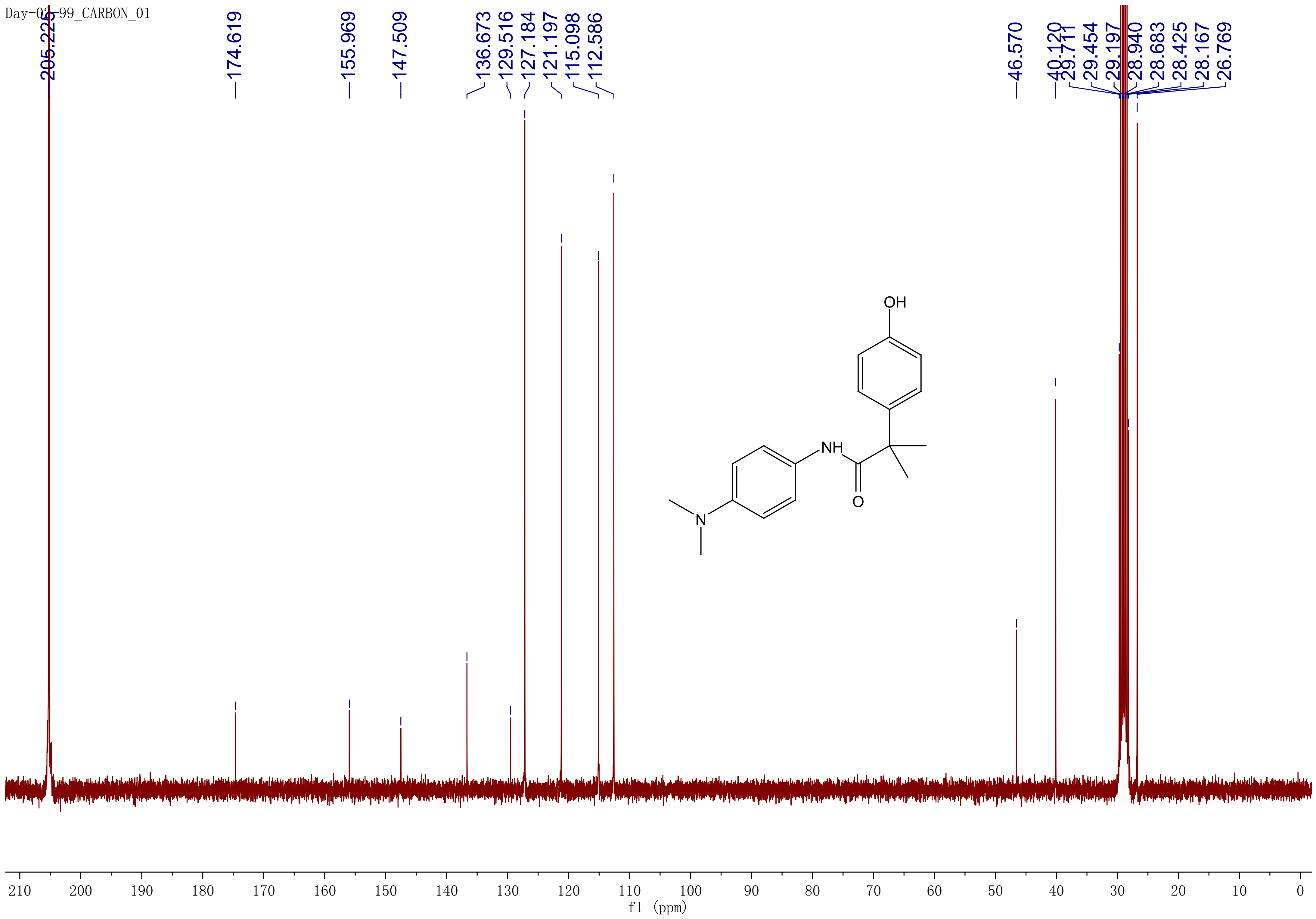
205.225
174.619
155.969
147.509
136.673
129.516
127.184
121.197
115.098
112.586
46.570
40.120
29.711
29.454
29.197
28.940
28.683
28.425
28.167
26.769
OH
NH
O
N
210 200 190 180 170 160 150 140 130 120 110 100 90 80 70 60 50 40 30 20 10 0
f1 (ppm)

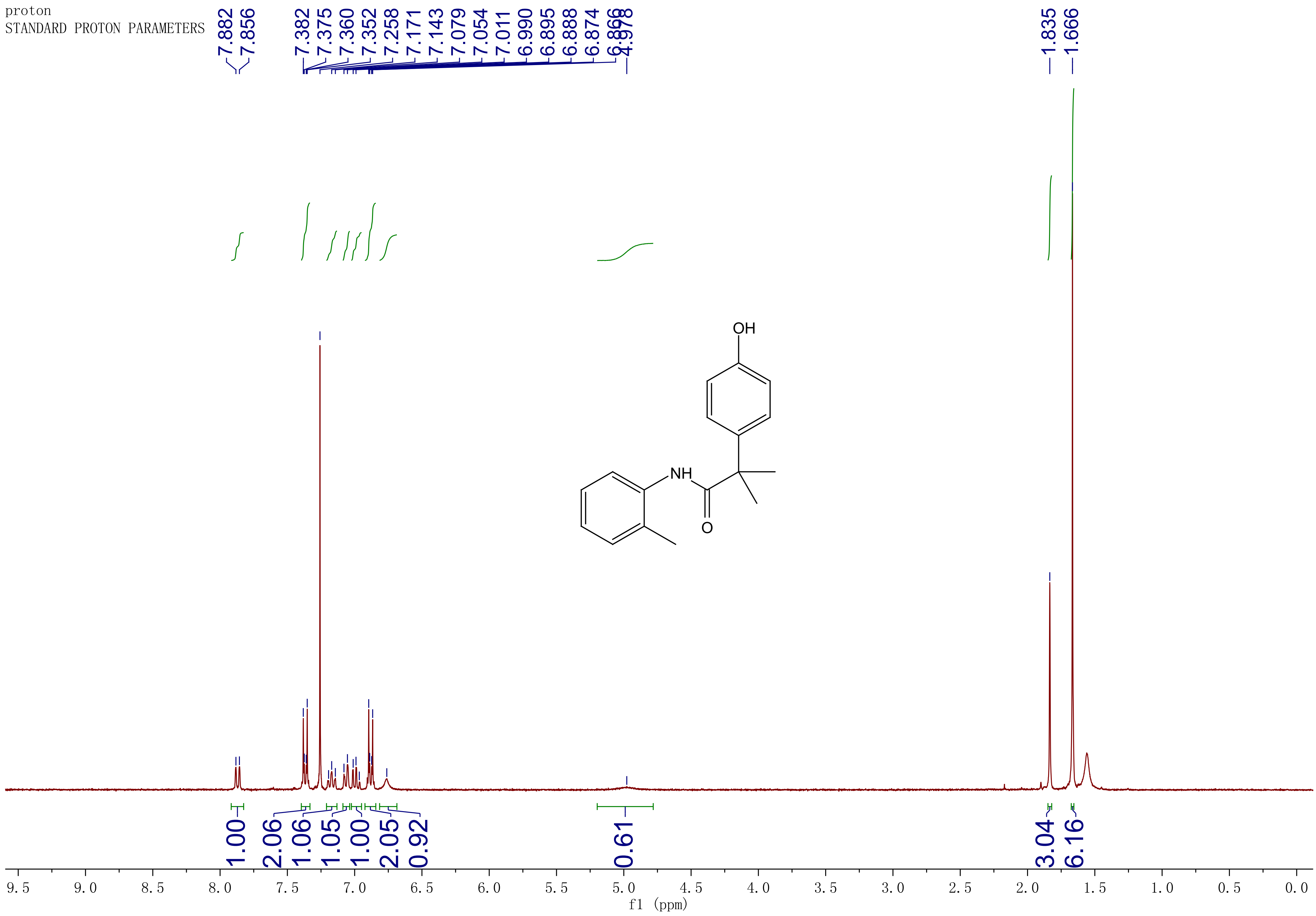
proton
STANDARD PROTON PARAMETERS
7.882
7.856
7.382
7.375
7.360
7.352
7.258
7.171
7.143
7.079
7.054
7.011
6.990
6.895
6.888
6.874
6.866
4.978
1.835
1.666
OH
NH
O
1.00
2.06
1.06
1.05
1.00
2.05
0.92
0.61
3.04
6.16
9.5
9.0
8.5
8.0
7.5
7.0
6.5
6.0
5.5
5.0
4.5
4.0
3.5
3.0
2.5
2.0
1.5
1.0
0.5
0.0
f1 (ppm)

Day-02-78_CARBON_03

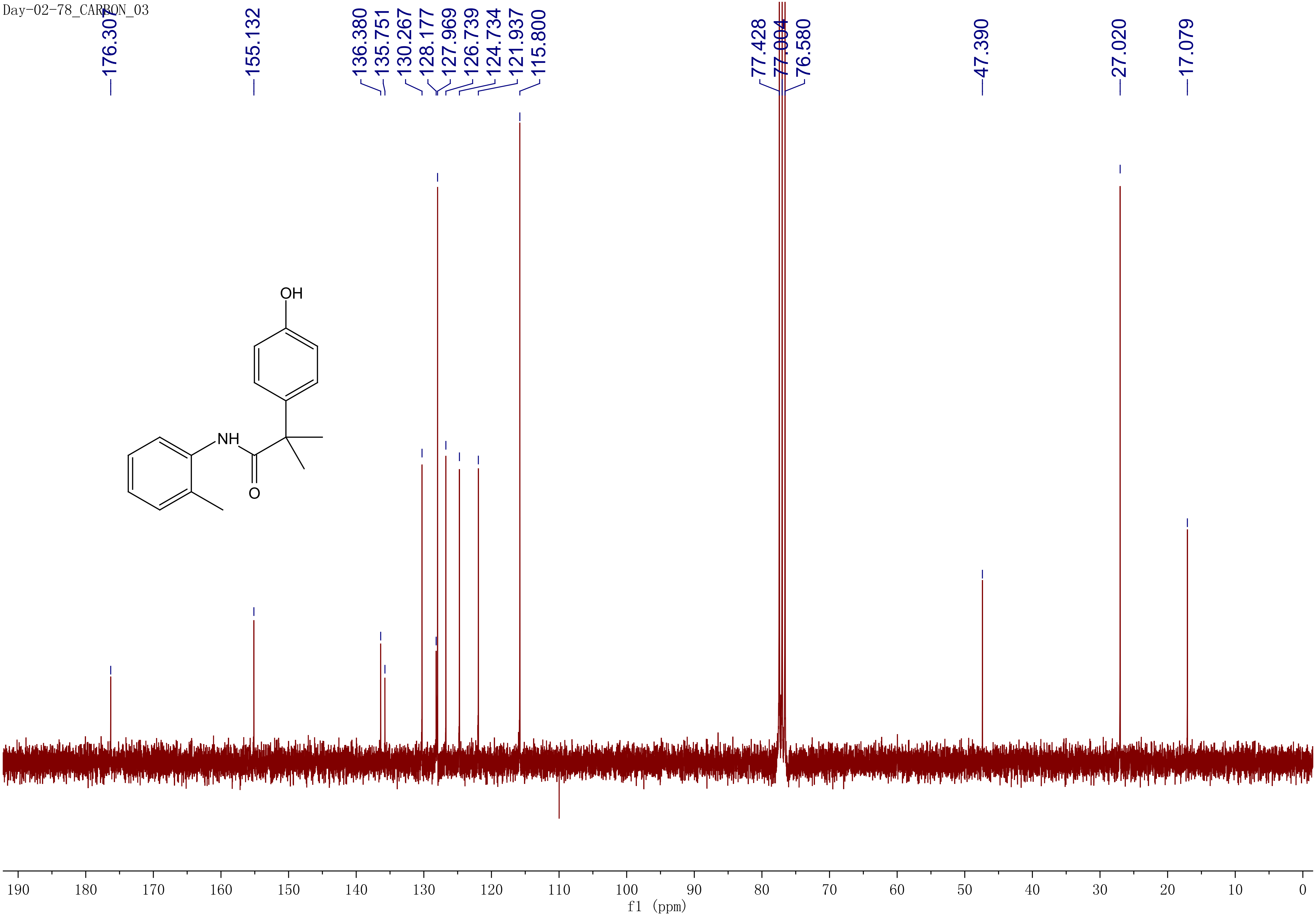

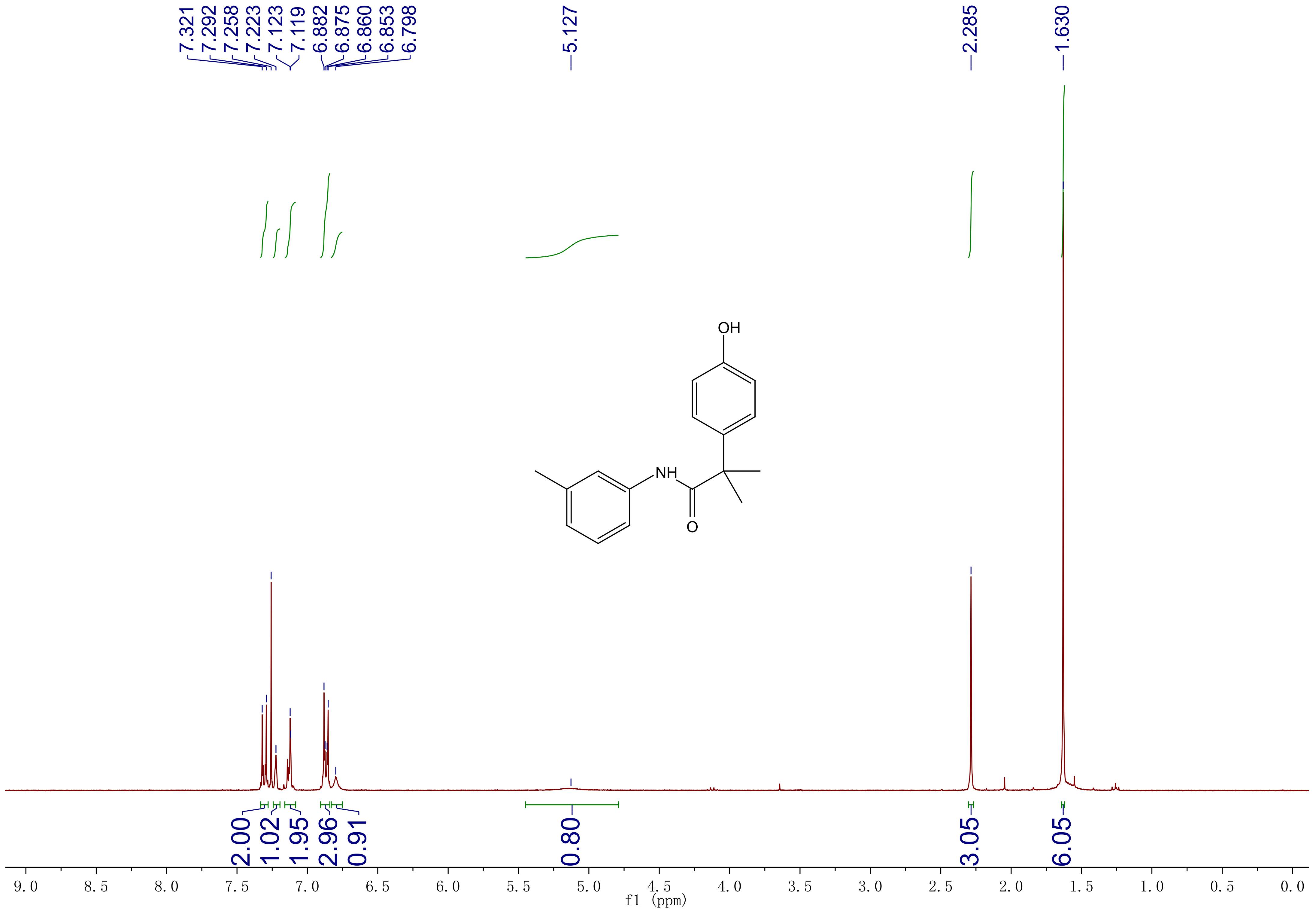
7.321
7.292
7.258
7.223
7.123
7.119
6.882
6.875
6.860
6.853
6.798
5.127
2.285
1.630
OH
NH
O
2.00
1.02
1.95
2.96
0.91
0.80
3.05
6.05
9.0
8.5
8.0
7.5
7.0
6.5
6.0
5.5
5.0
4.5
4.0
3.5
3.0
2.5
2.0
1.5
1.0
0.5
0.0
f1 (ppm)

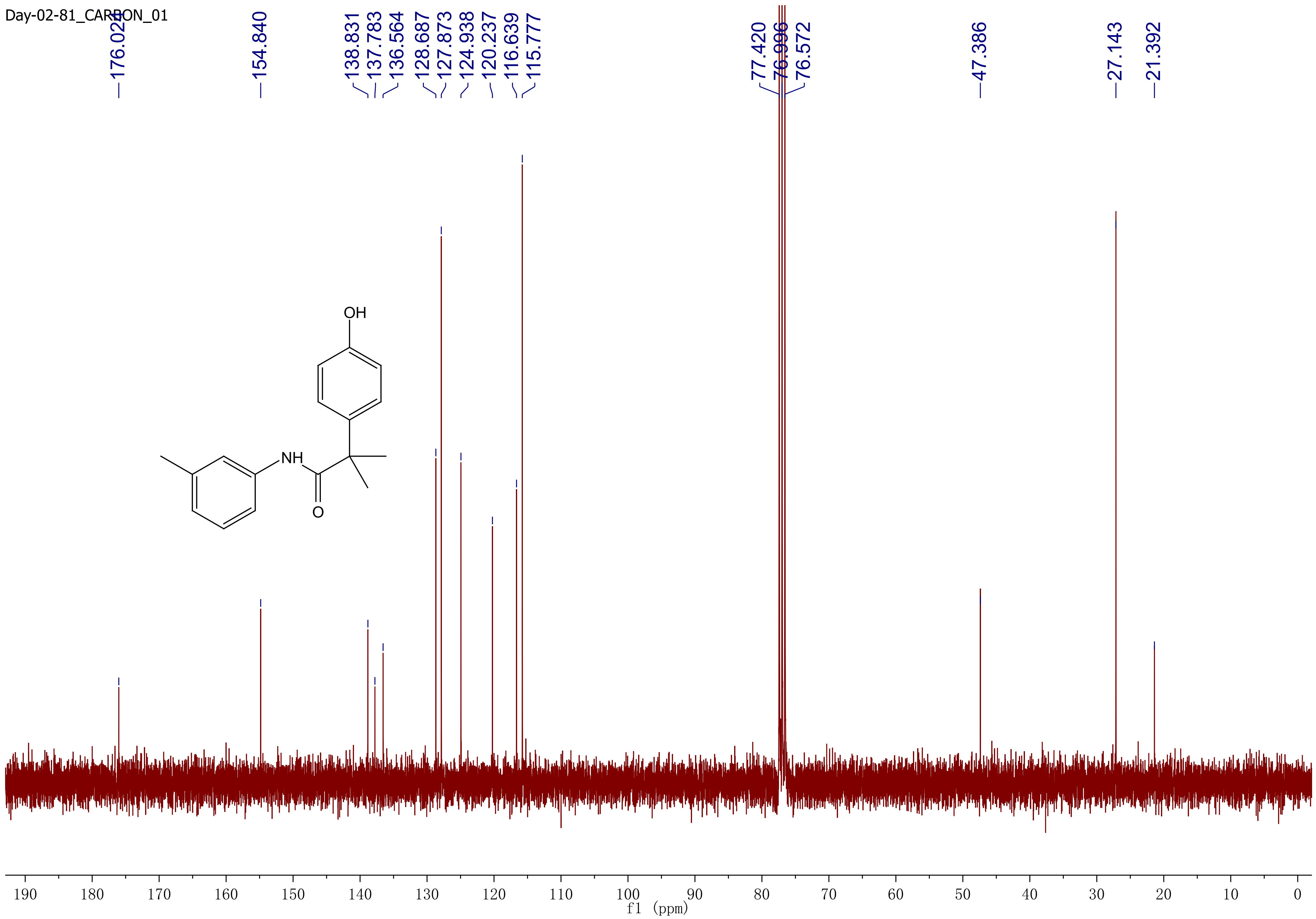
Day-02-81_CARBON_01
176.024
154.840
138.831
137.783
136.564
128.687
127.873
124.938
120.237
116.639
115.777
77.420
76.996
76.572
47.386
27.143
21.392
OH
NH
O
190 180 170 160 150 140 130 120 110 100 90 80 70 60 50 40 30 20 10 0
f1 (ppm)

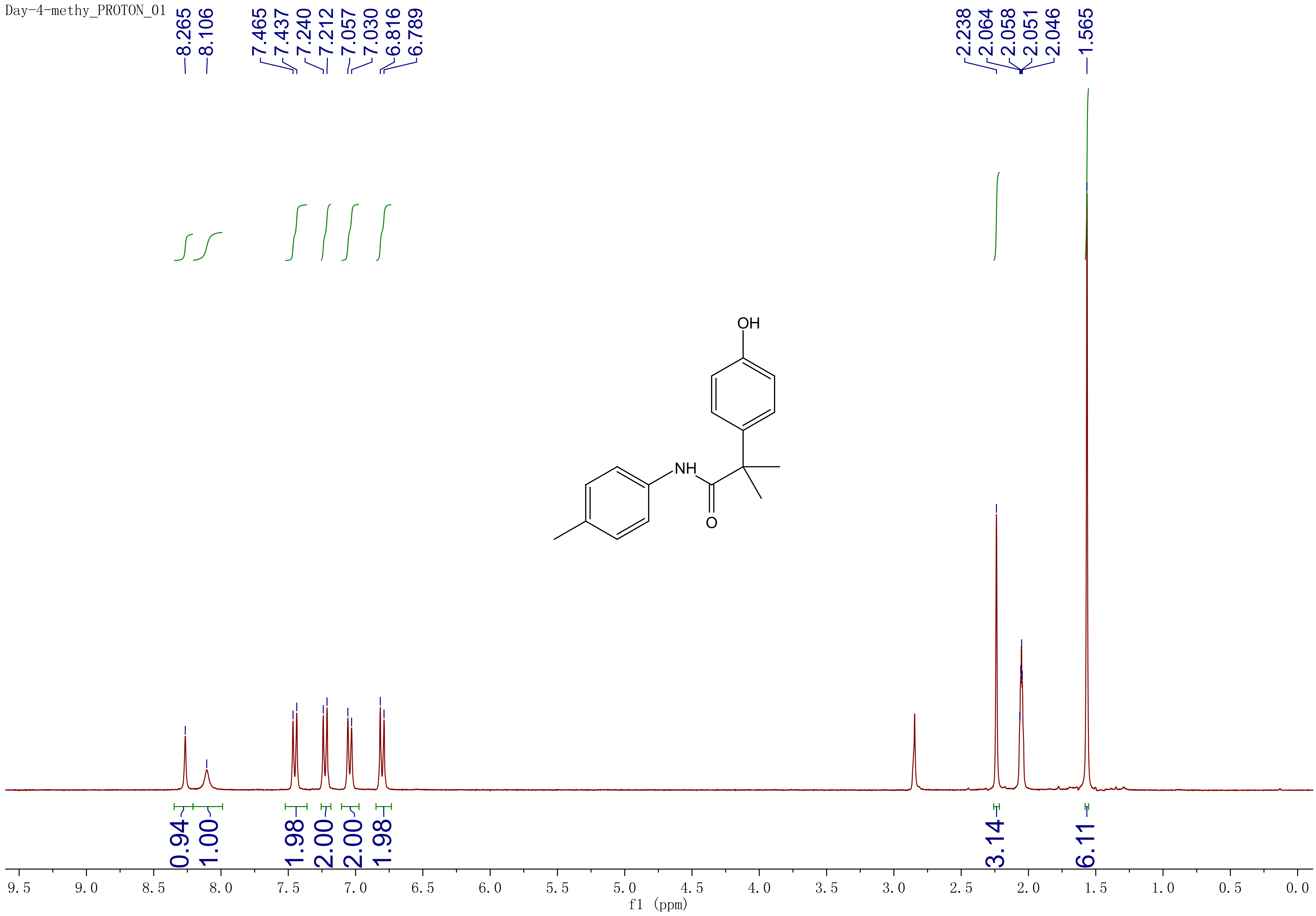
Day-4-methy_PROTON_01
8.265
8.106
7.465
7.437
7.240
7.212
7.057
7.030
6.816
6.789
2.238
2.064
2.058
2.051
2.046
1.565
OH
NH
O
0.94
1.00
1.98
2.00
2.00
1.98
3.14
6.11
9.5
9.0
8.5
8.0
7.5
7.0
6.5
6.0
5.5
5.0
4.5
4.0
3.5
3.0
2.5
2.0
1.5
1.0
0.5
0.0
f1 (ppm)

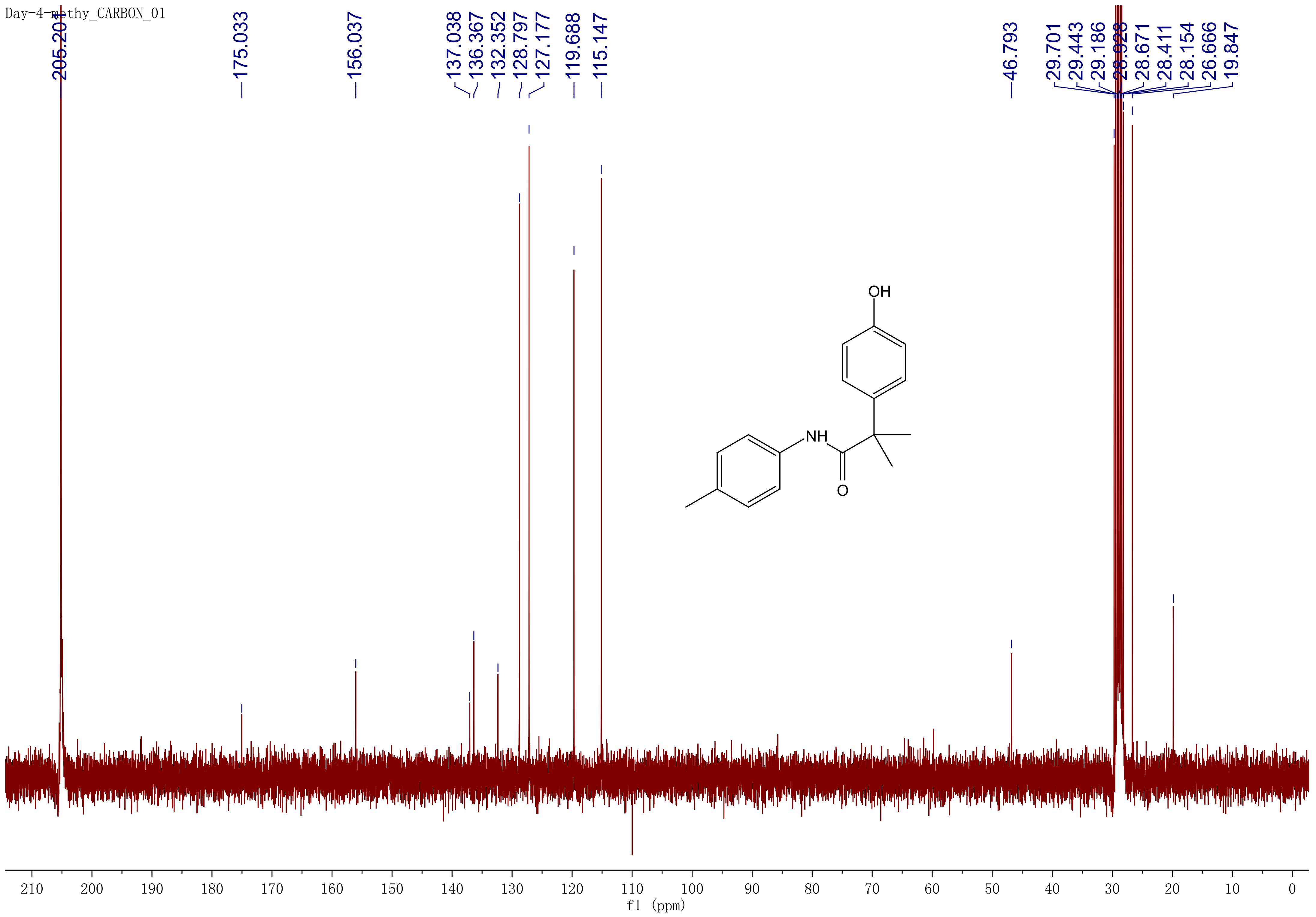

Day-4-methy_CARBON_01
205.201
175.033
156.037
137.038
136.367
132.352
128.797
127.177
119.688
115.147
46.793
29.701
29.443
29.186
28.928
28.671
28.411
28.154
26.666
19.847
OH
NH
O
210 200 190 180 170 160 150 140 130 120 110 100 90 80 70 60 50 40 30 20 10 0
f1 (ppm)

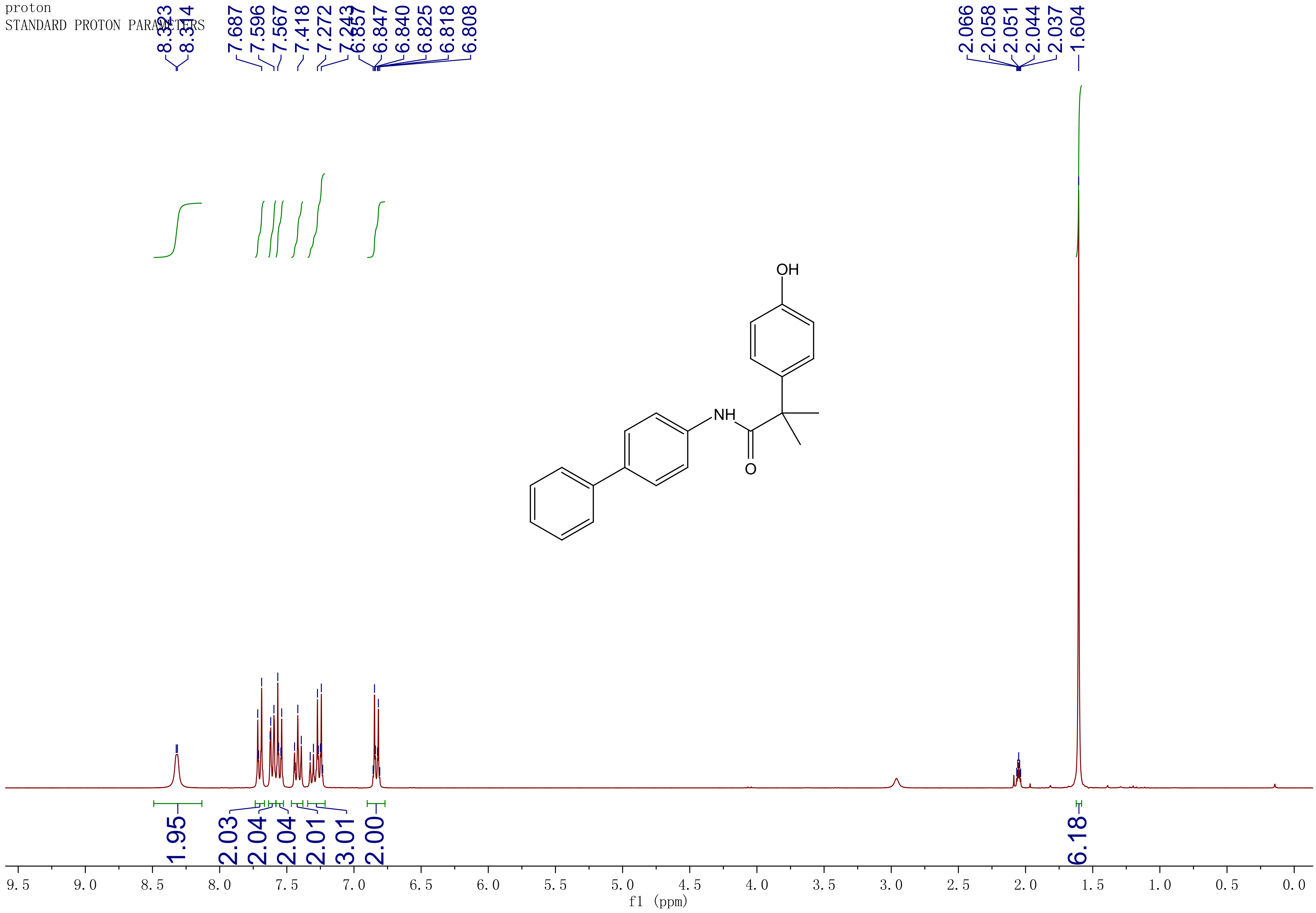

proton
STANDARD PROTON PARAMETERS
8.323
8.314
7.687
7.596
7.567
7.418
7.272
7.243
6.857
6.847
6.840
6.825
6.818
6.808
2.066
2.058
2.051
2.044
2.037
1.604
OH
NH
O
1.95
2.03
2.04
2.04
2.01
3.01
2.00
6.18
9.5 9.0 8.5 8.0 7.5 7.0 6.5 6.0 5.5 5.0 4.5 4.0 3.5 3.0 2.5 2.0 1.5 1.0 0.5 0.0
f1 (ppm)

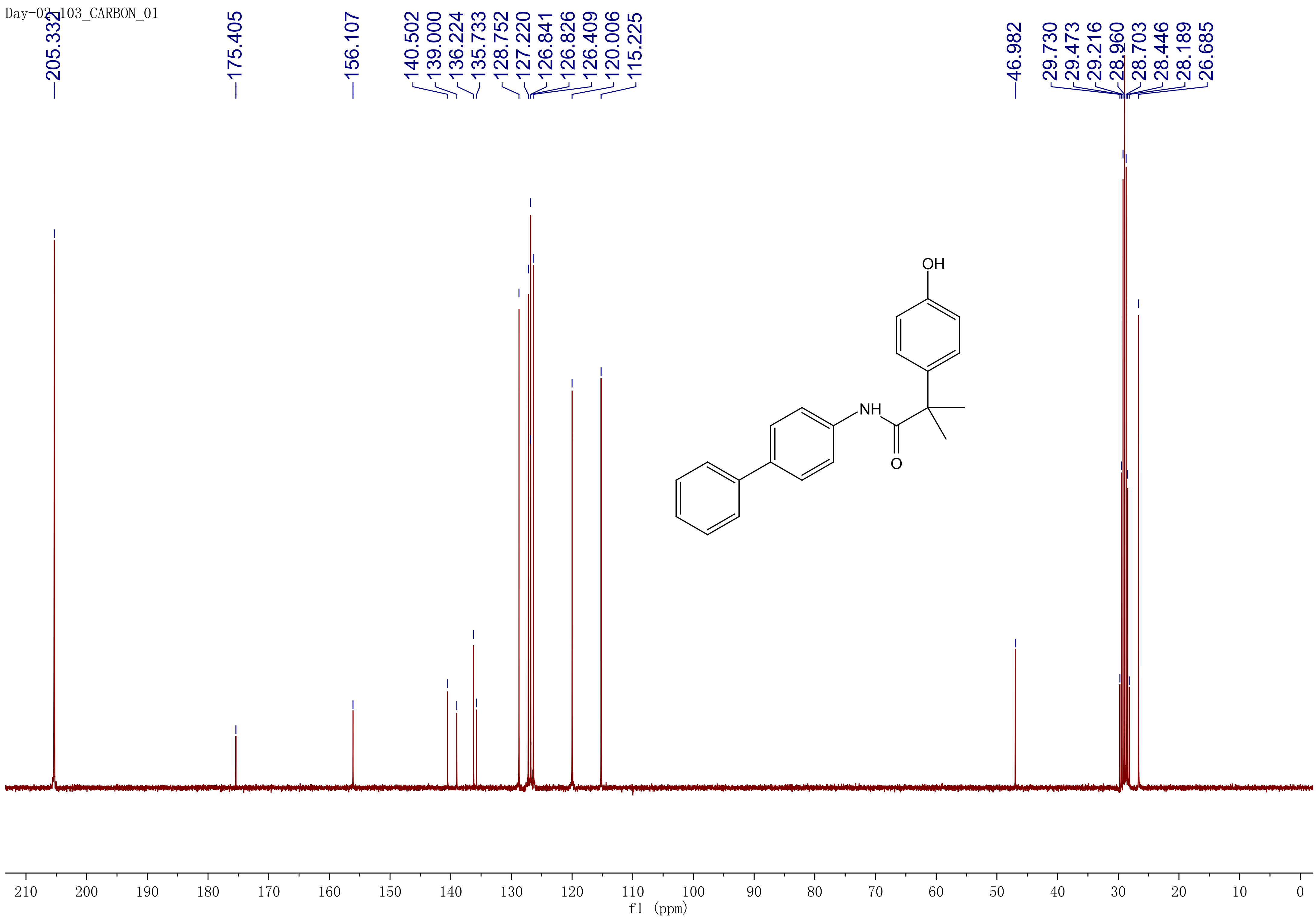

Day-02-103_CARBON_01
205.332
175.405
156.107
140.502
139.000
136.224
135.733
128.752
127.220
126.841
126.826
126.409
120.006
115.225
46.982
29.730
29.473
29.216
28.960
28.703
28.446
28.189
26.685
OH
NH
O
210 200 190 180 170 160 150 140 130 120 110 100 90 80 70 60 50 40 30 20 10 0
f1 (ppm)

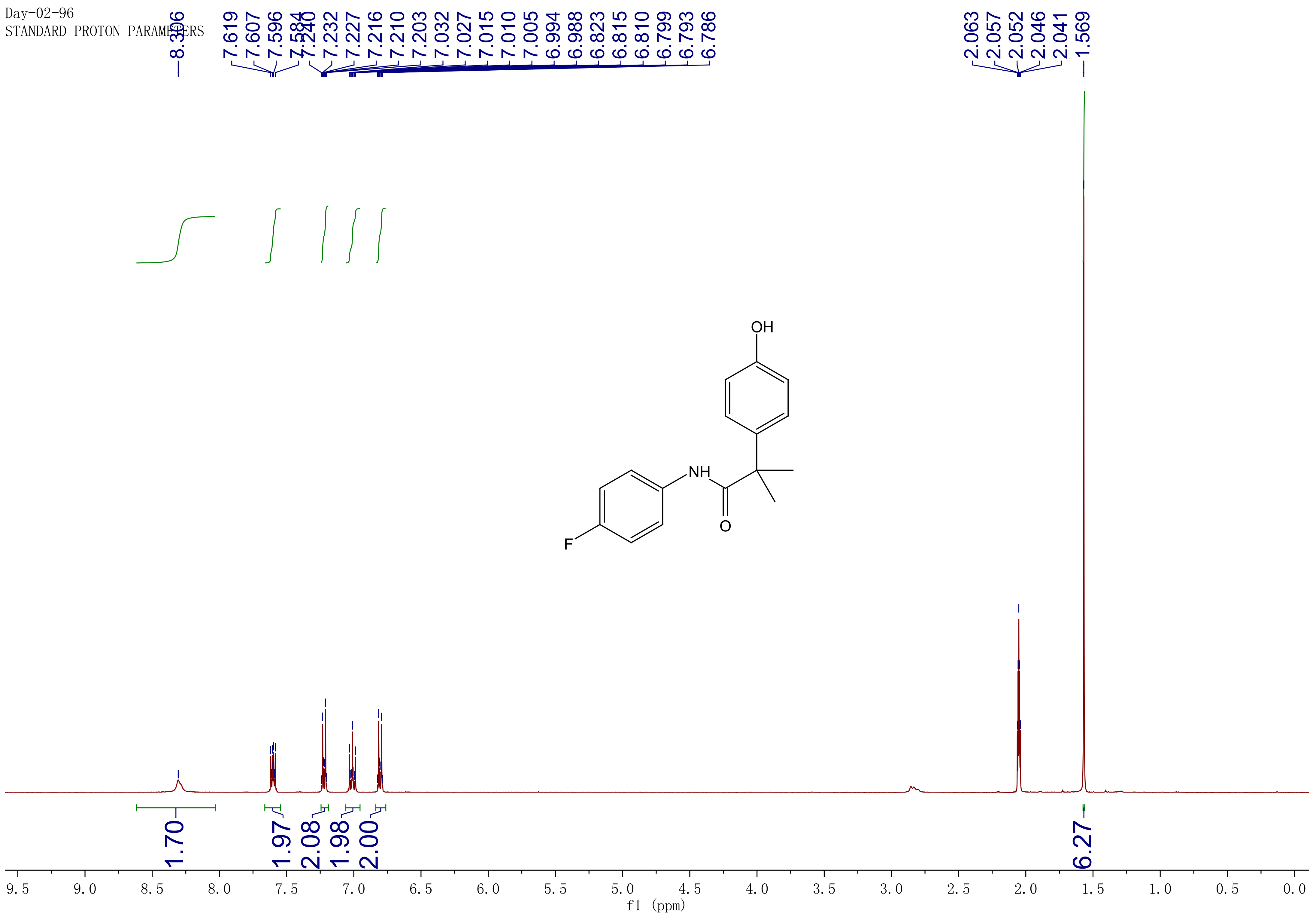

Day-02-96
STANDARD PROTON PARAMETERS
8.306
7.619
7.607
7.596
7.584
7.240
7.232
7.227
7.216
7.210
7.203
7.032
7.027
7.015
7.010
7.005
6.994
6.988
6.823
6.815
6.810
6.799
6.793
6.786
2.063
2.057
2.052
2.046
2.041
1.569
OH
NH
O
F
1.70
1.97
2.08
1.98
2.00
6.27
9.5
9.0
8.5
8.0
7.5
7.0
6.5
6.0
5.5
5.0
4.5
4.0
3.5
3.0
2.5
2.0
1.5
1.0
0.5
0.0
f1 (ppm)

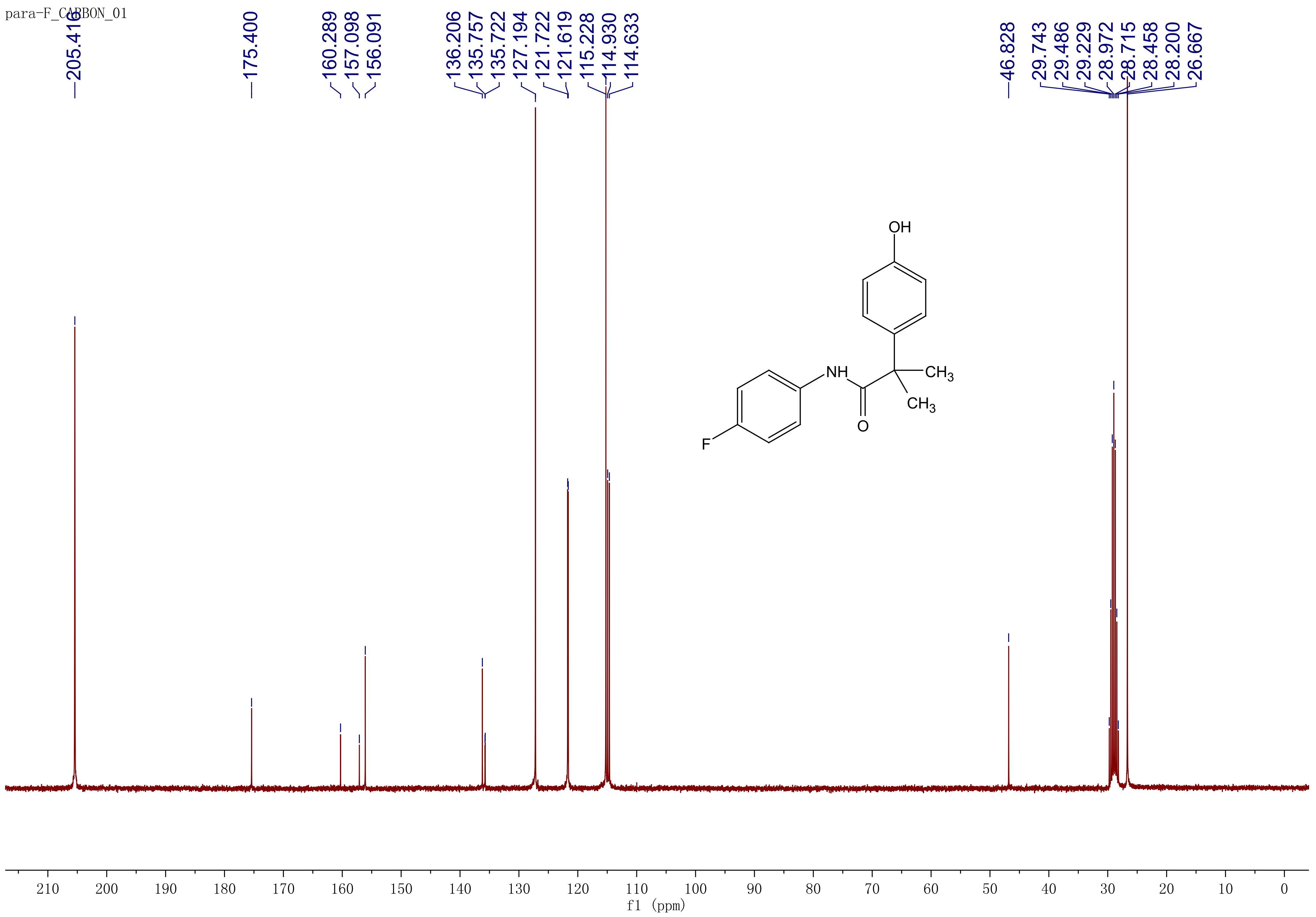

para-F_CARBON_01
205.416
175.400
160.289
157.098
156.091
136.206
135.757
135.722
127.194
121.722
121.619
115.228
114.930
114.633
46.828
29.743
29.486
29.229
28.972
28.715
28.458
28.200
26.667
OH
NH
CH3
CH3
O
F
210 200 190 180 170 160 150 140 130 120 110 100 90 80 70 60 50 40 30 20 10 0
f1 (ppm)

Day-chloro_PROTON_01

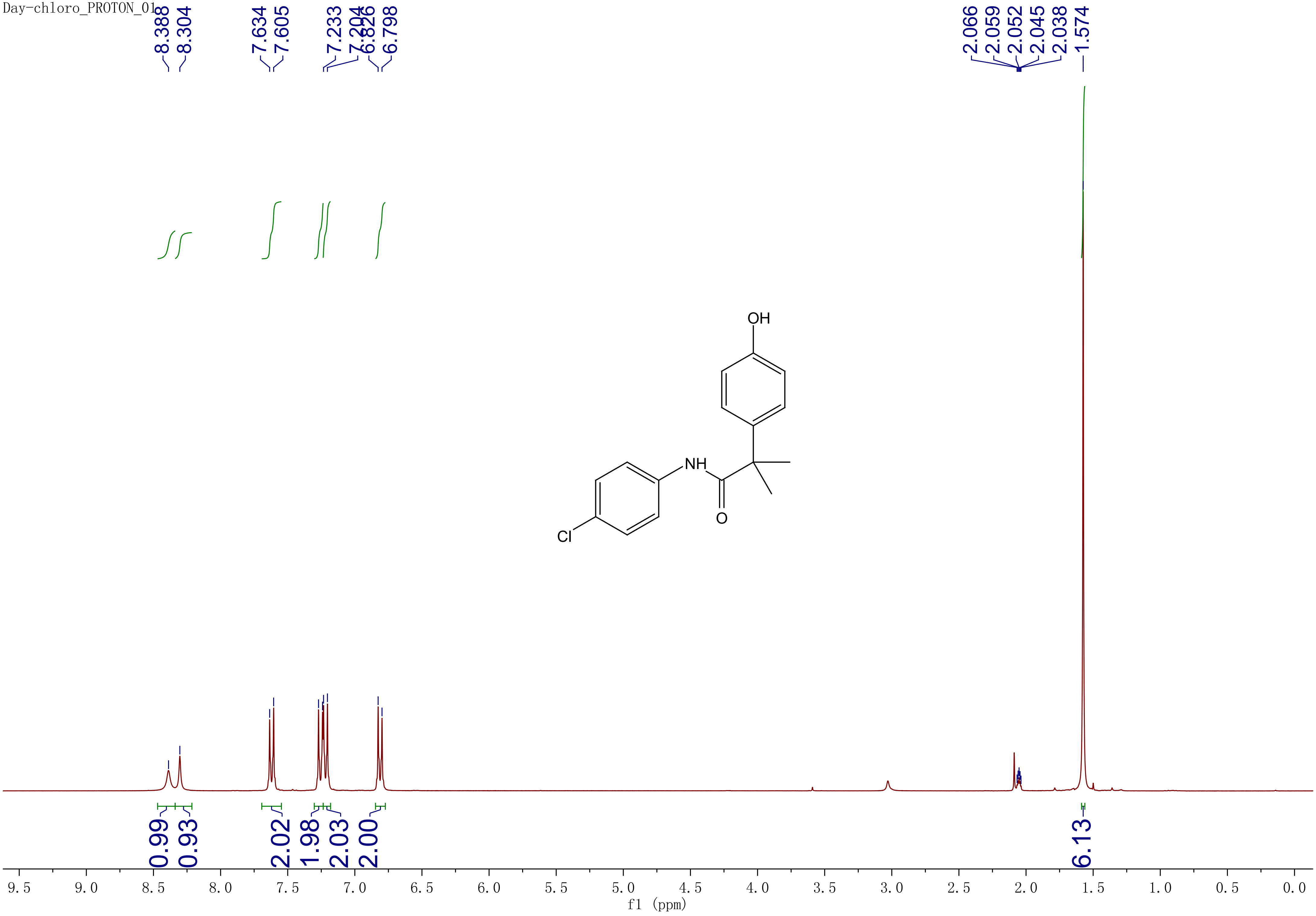

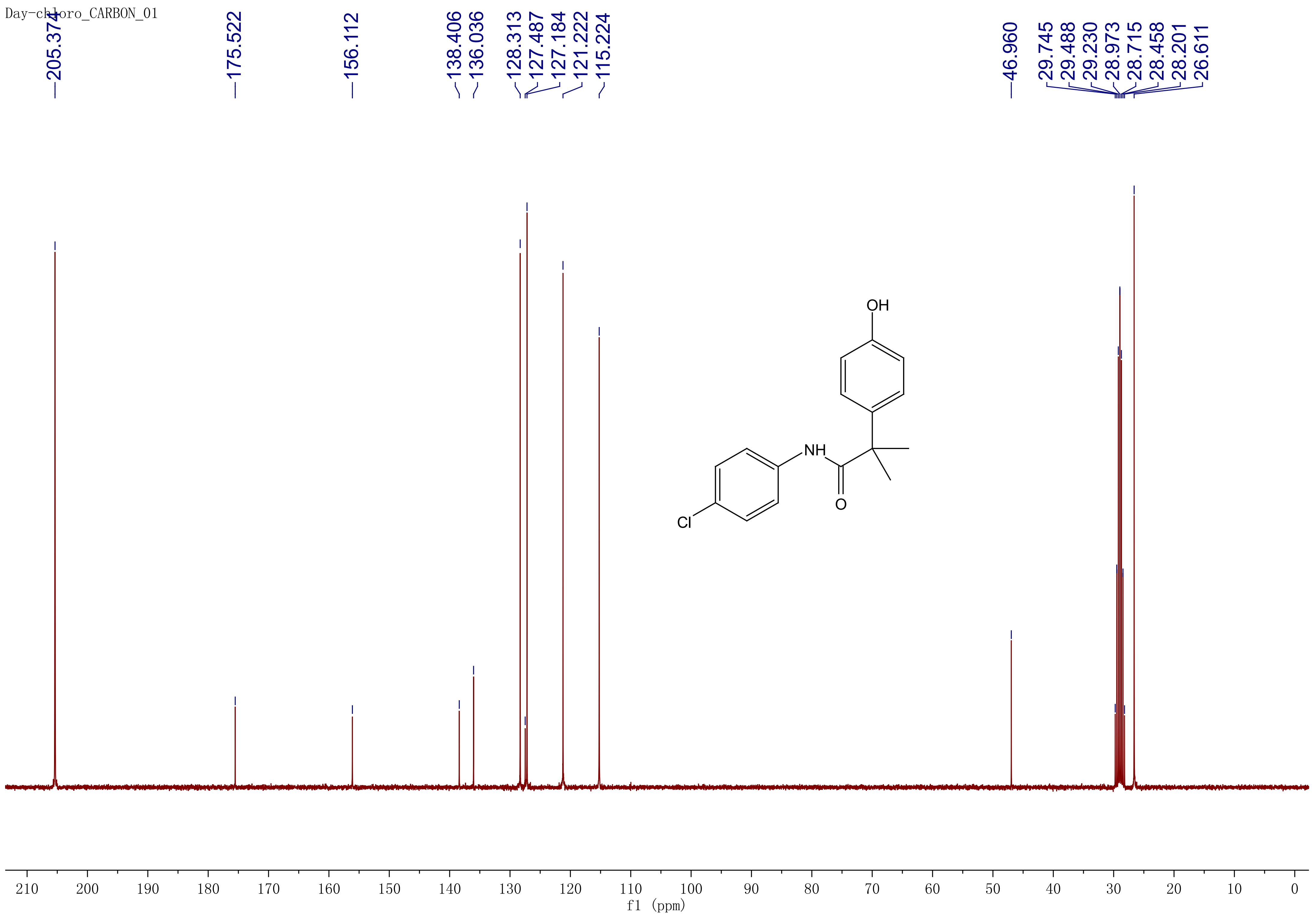
Day-chloro_CARBON_01
205.374
175.522
156.112
138.406
136.036
128.313
127.487
127.184
121.222
115.224
46.960
29.745
29.488
29.230
28.973
28.715
28.458
28.201
26.611
OH
NH
O
Cl
210 200 190 180 170 160 150 140 130 120 110 100 90 80 70 60 50 40 30 20 10 0
f1 (ppm)

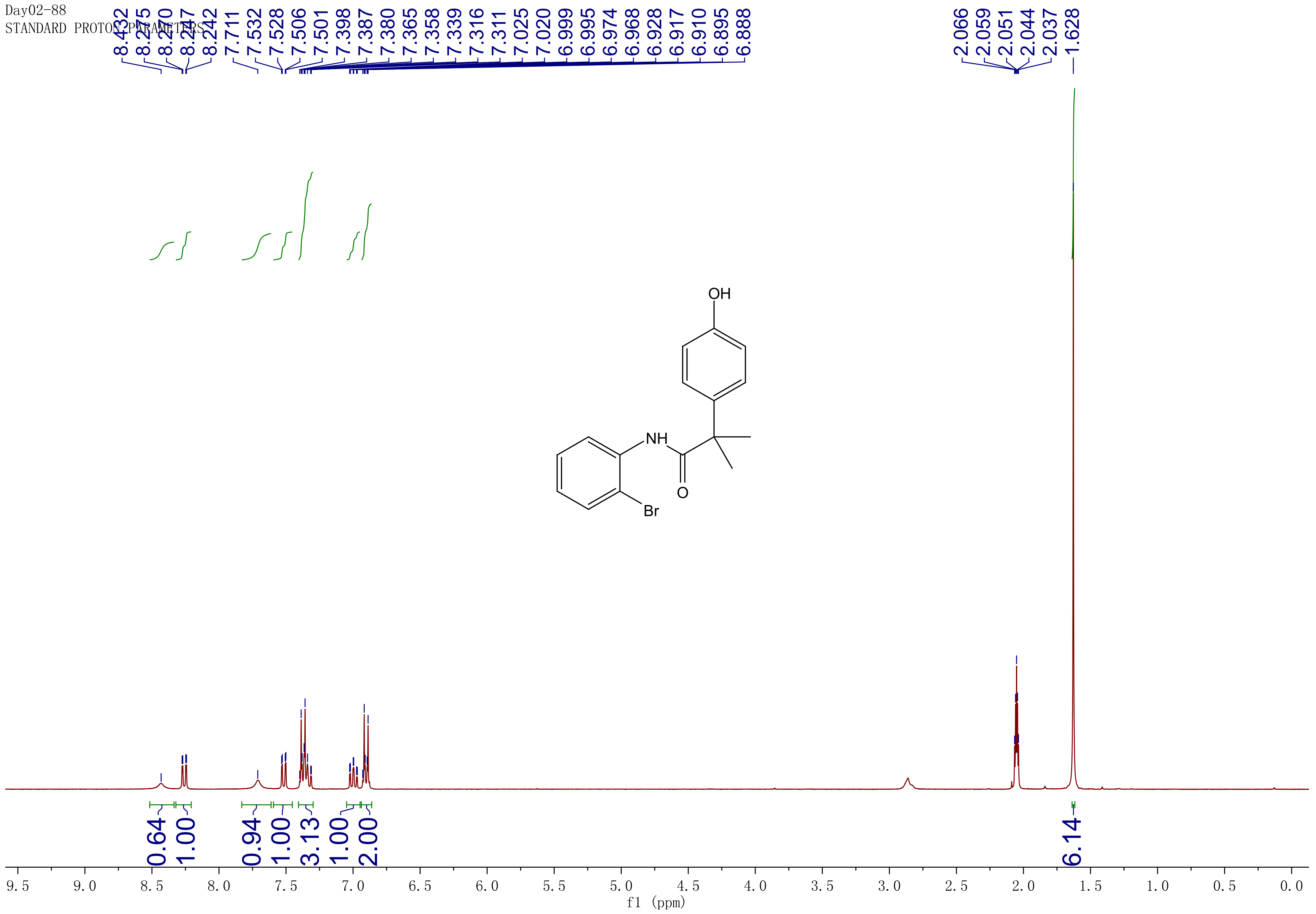
Day02-88
STANDARD PROTON PARAMETERS
8.432
8.275
8.270
8.247
8.242
7.711
7.532
7.528
7.506
7.501
7.398
7.387
7.380
7.365
7.358
7.339
7.316
7.311
7.025
7.020
6.999
6.995
6.974
6.968
6.928
6.917
6.910
6.895
6.888
2.066
2.059
2.051
2.044
2.037
1.628
OH
NH
O
Br
0.64
1.00
0.94
1.00
3.13
1.00
2.00
6.14
9.5
9.0
8.5
8.0
7.5
7.0
6.5
6.0
5.5
5.0
4.5
4.0
3.5
3.0
2.5
2.0
1.5
1.0
0.5
0.0
f1 (ppm)

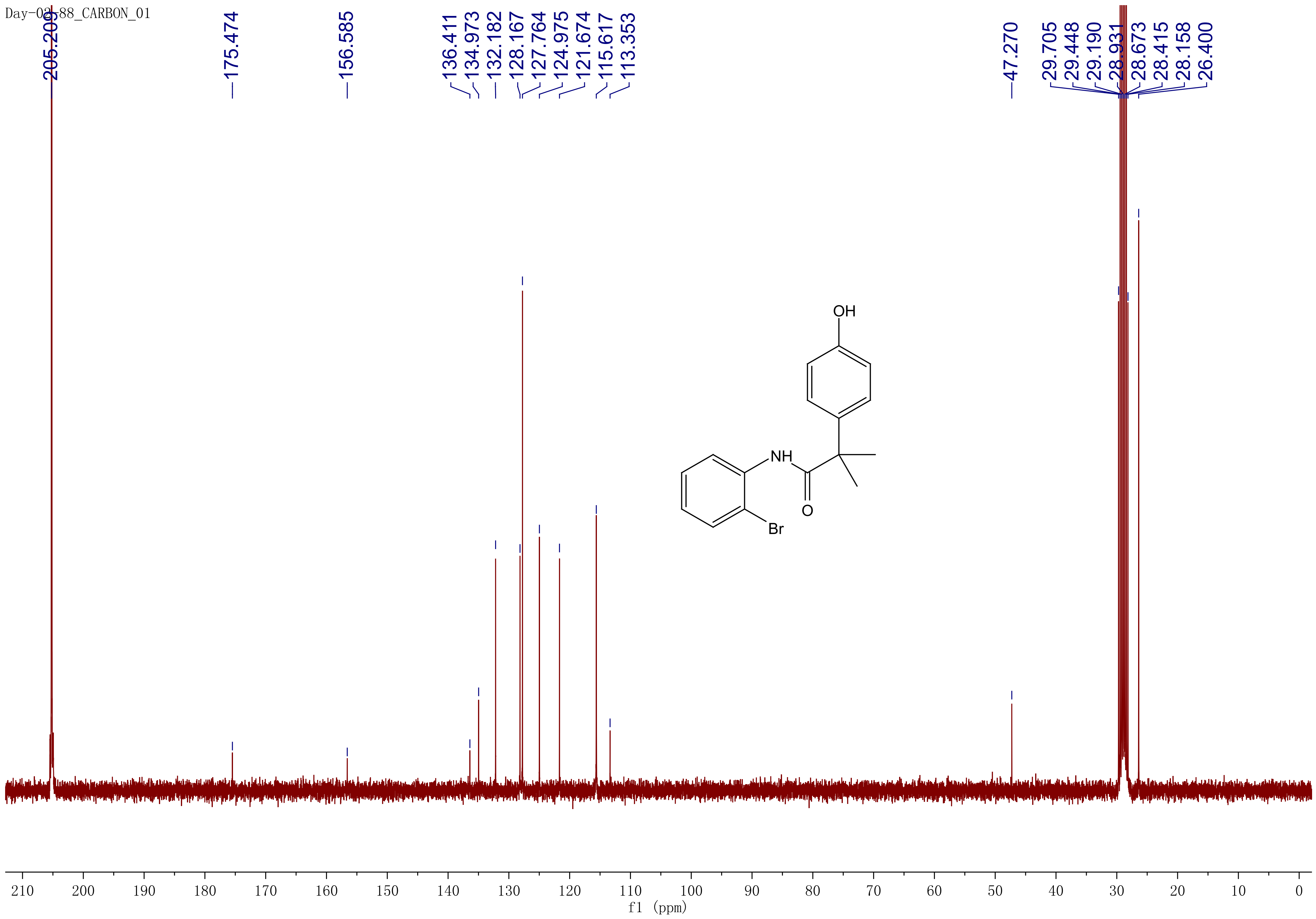

Day-02-88_CARBON_01
205.209
175.474
156.585
136.411
134.973
132.182
128.167
127.764
124.975
121.674
115.617
113.353
47.270
29.705
29.448
29.190
28.931
28.673
28.415
28.158
26.400
OH
NH
O
Br
210 200 190 180 170 160 150 140 130 120 110 100 90 80 70 60 50 40 30 20 10 0
f1 (ppm)

proton
STANDARD PROTON PARAMETERS

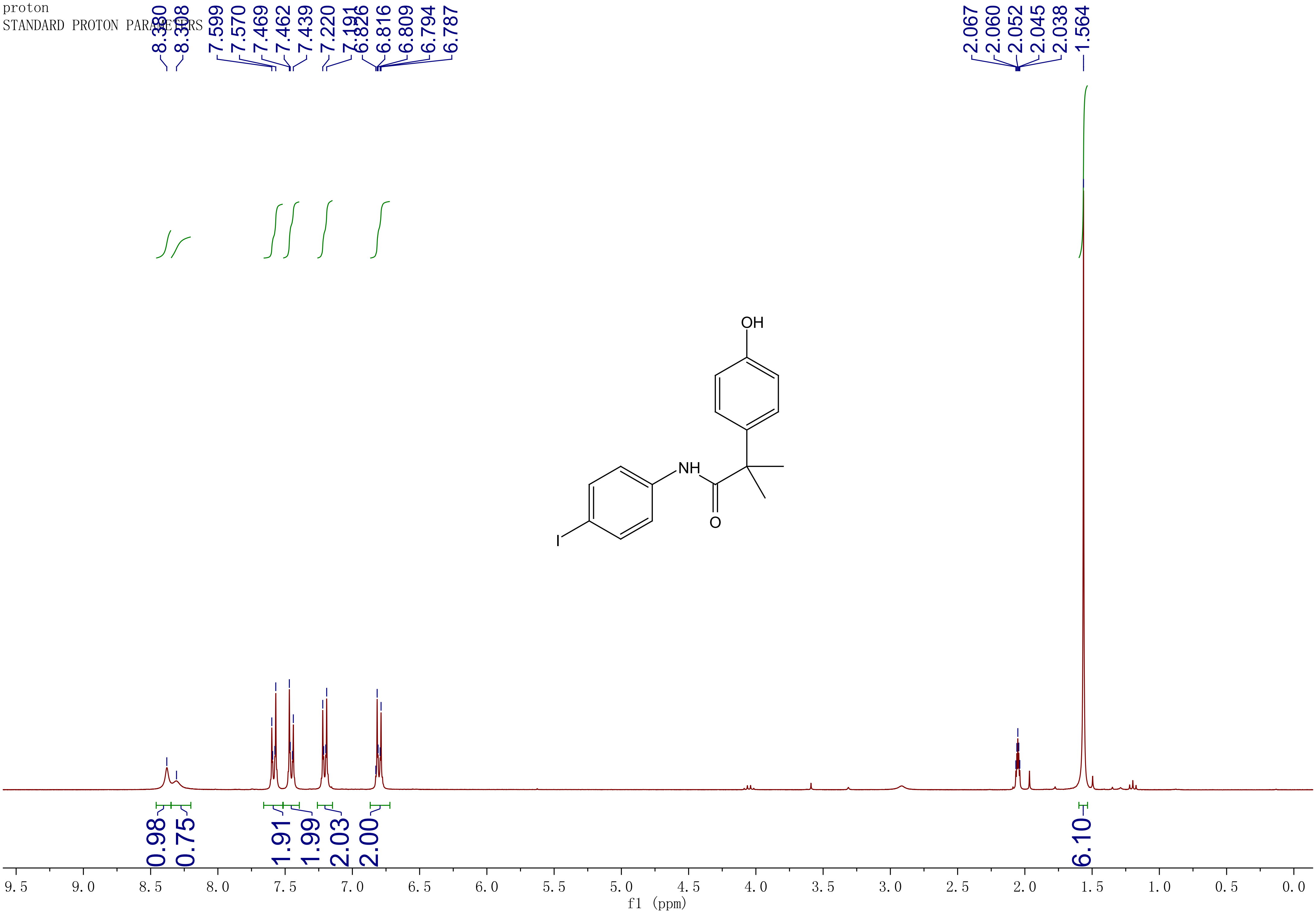

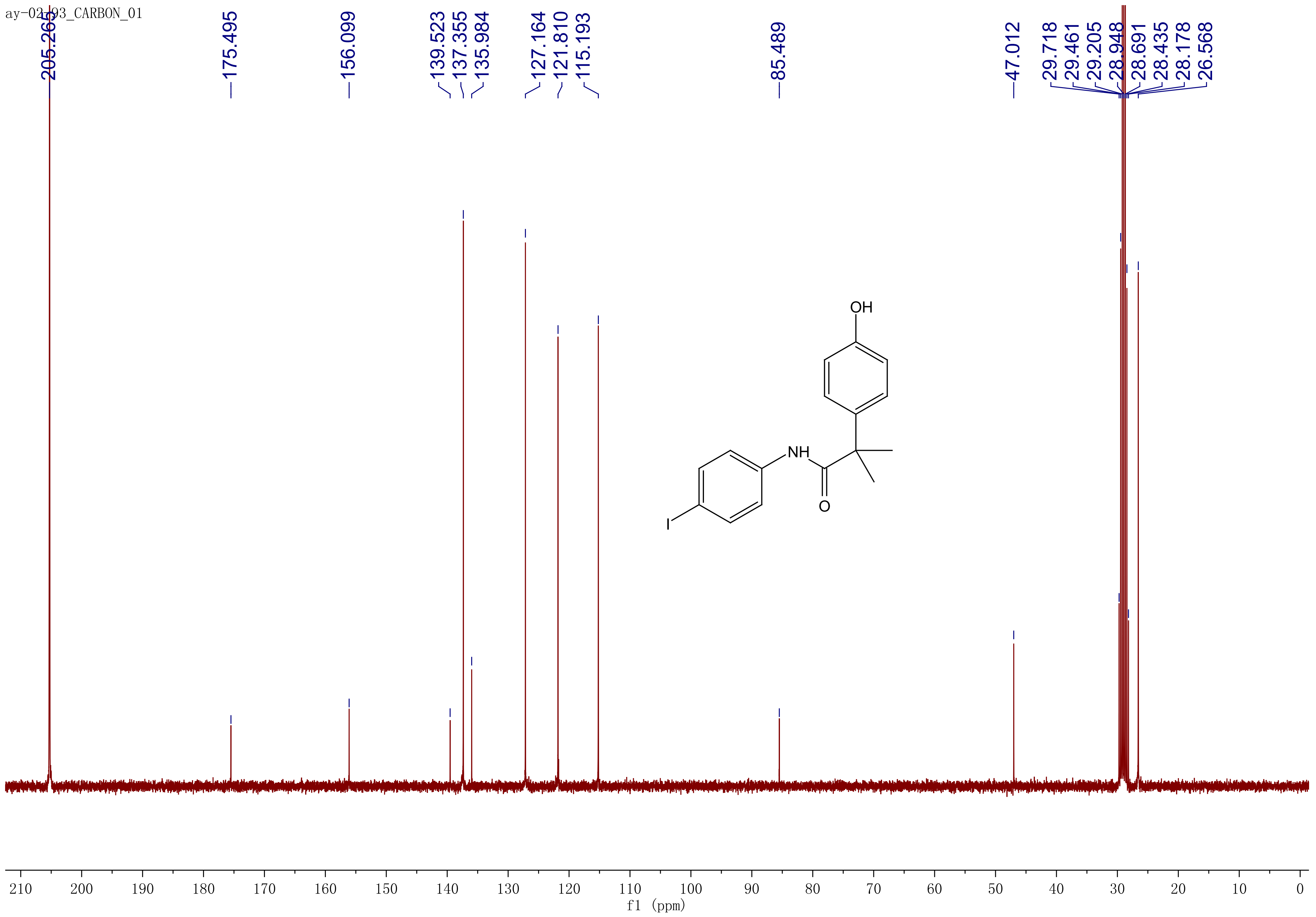

ay-02-93_CARBON_01
205.265
175.495
156.099
139.523
137.355
135.984
127.164
121.810
115.193
85.489
47.012
29.718
29.461
29.205
28.948
28.691
28.435
28.178
26.568
OH
NH
O
I
f1 (ppm)

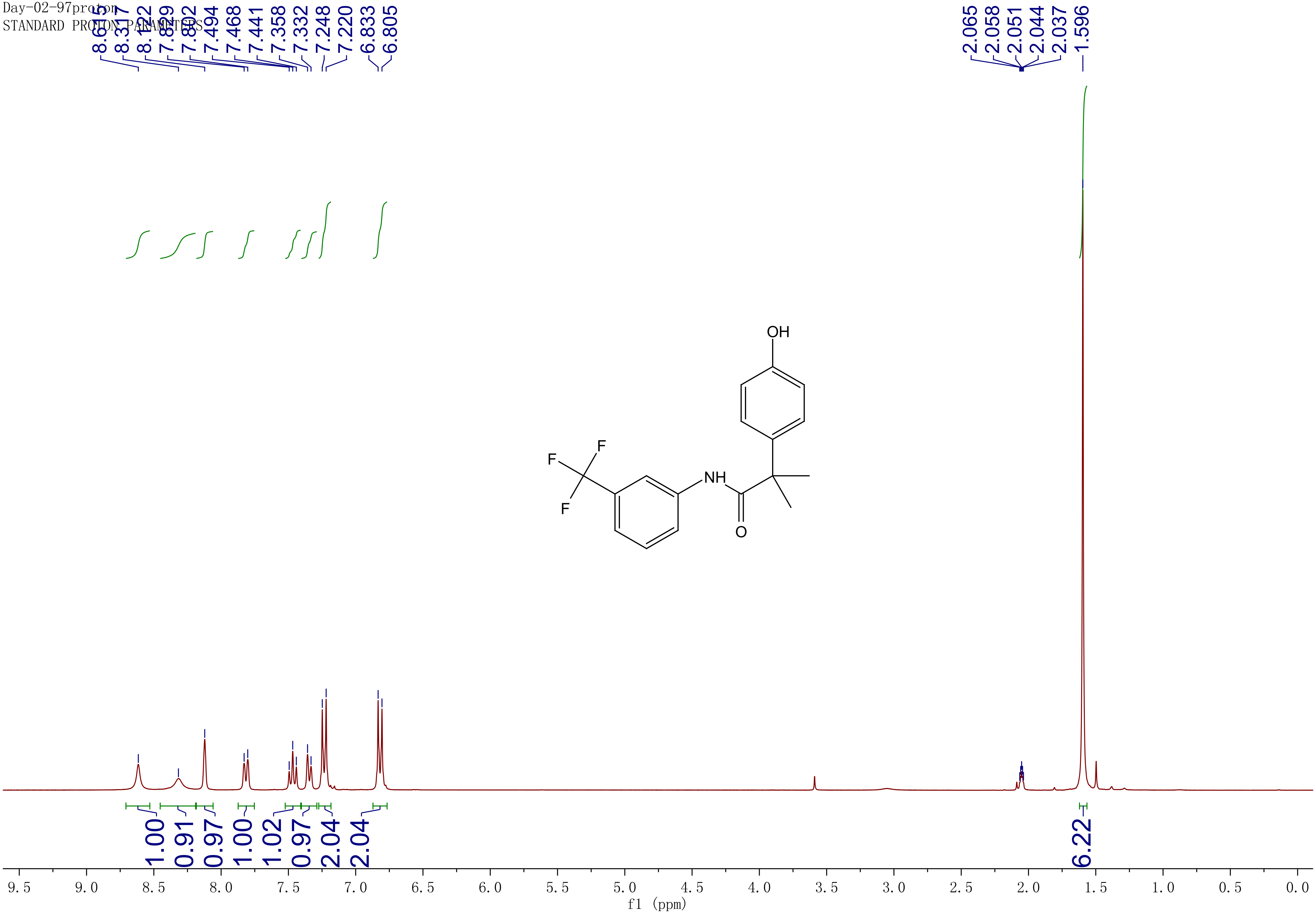
Day-02-97proton
STANDARD PROTON PARAMETERS
8.615
8.317
8.122
7.829
7.802
7.494
7.468
7.441
7.358
7.332
7.248
7.220
6.833
6.805
2.065
2.058
2.051
2.044
2.037
1.596
OH
NH
O
F
F
F
1.00
0.91
0.97
1.00
1.02
0.97
2.04
2.04
6.22
9.5
9.0
8.5
8.0
7.5
7.0
6.5
6.0
5.5
5.0
4.5
4.0
3.5
3.0
2.5
2.0
1.5
1.0
0.5
0.0
f1 (ppm)

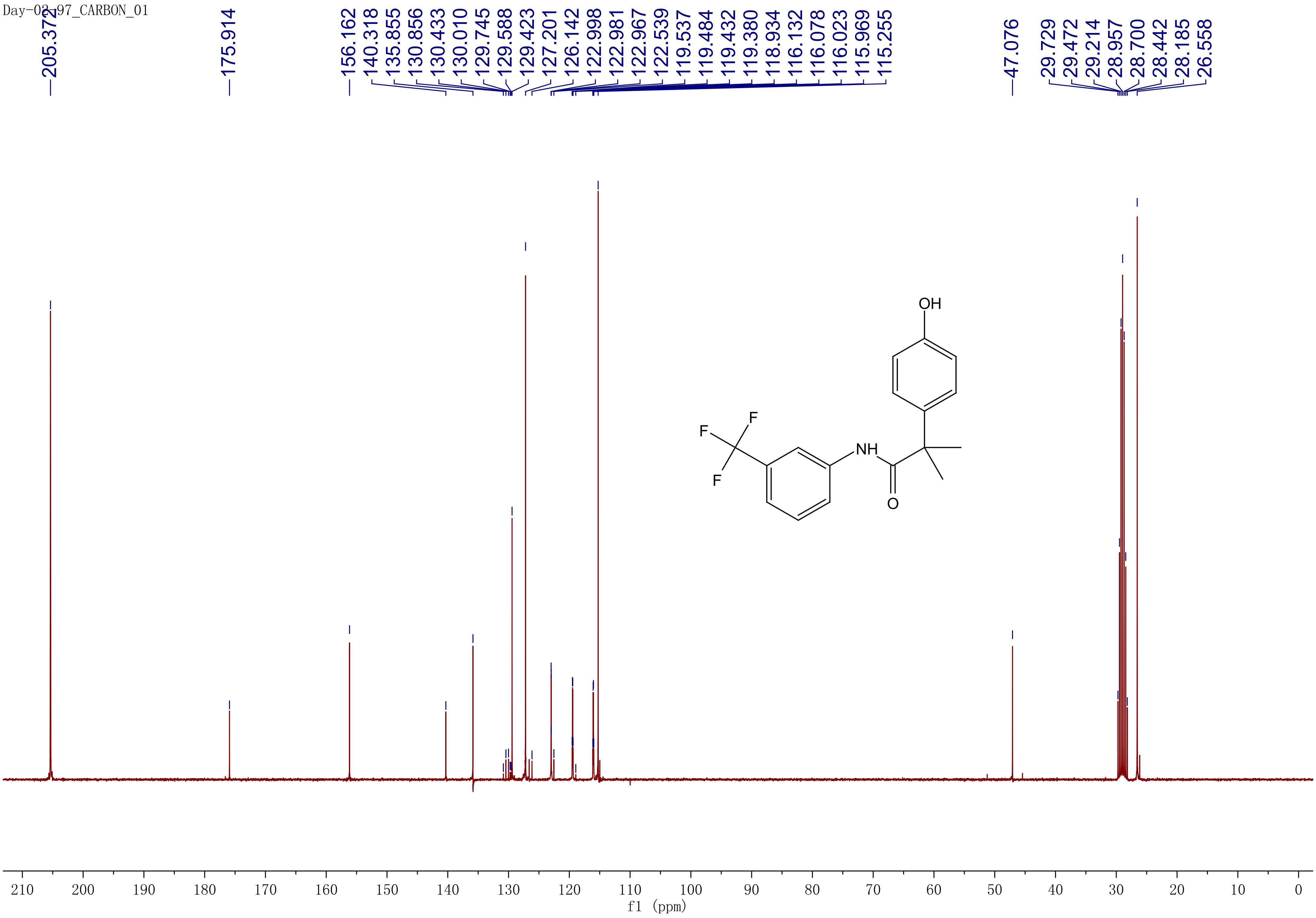
Day-02-97_CARBON_01
205.372
175.914
156.162
140.318
135.855
130.856
130.433
130.010
129.745
129.588
129.423
127.201
126.142
122.998
122.981
122.967
122.539
119.537
119.484
119.432
119.380
118.934
116.132
116.078
116.023
115.969
115.255
47.076
29.729
29.472
29.214
28.957
28.700
28.442
28.185
26.558
OH
F
F
F
NH
O
210 200 190 180 170 160 150 140 130 120 110 100 90 80 70 60 50 40 30 20 10 0
f1 (ppm)

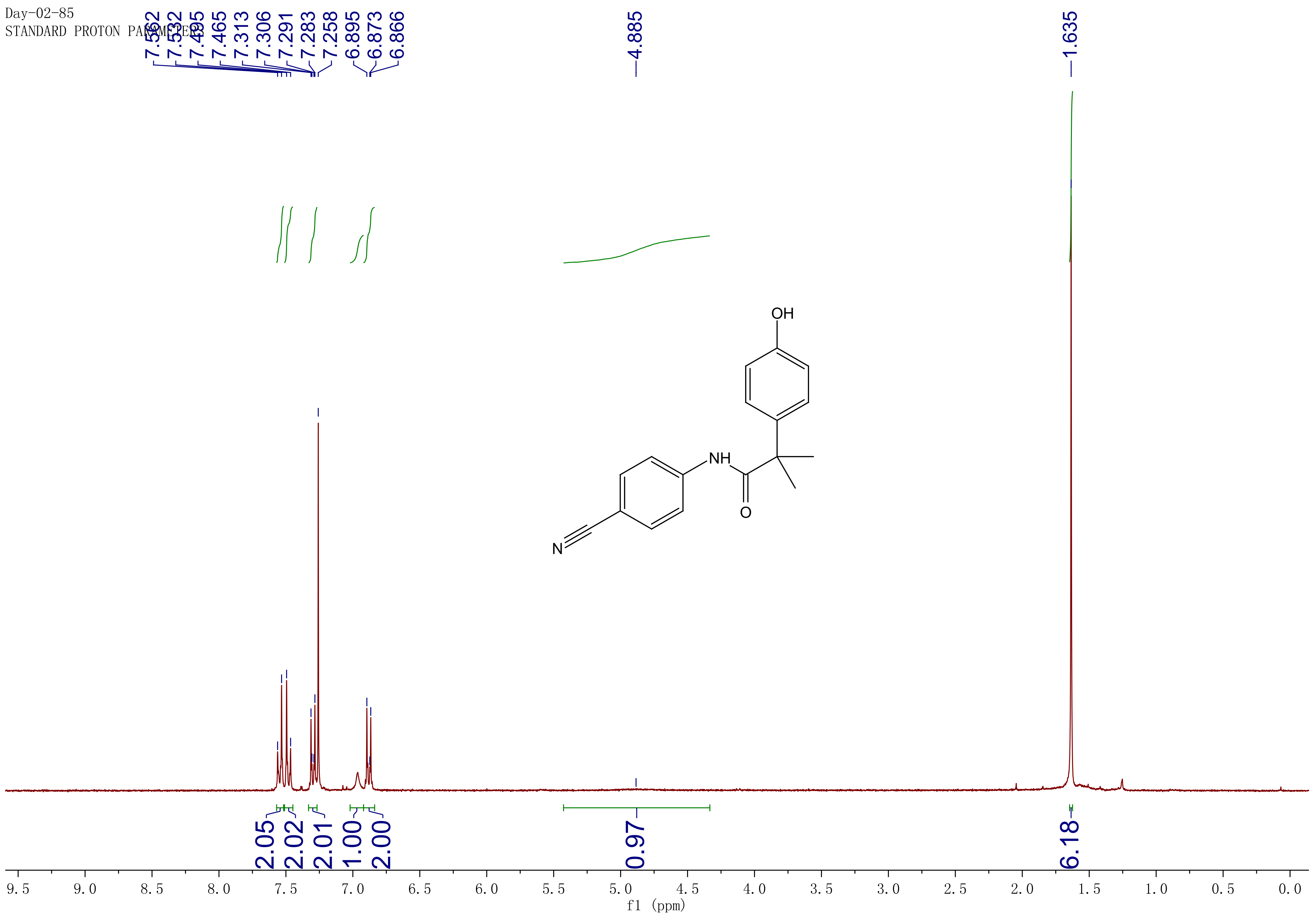

Day-02-85
STANDARD PROTON PARAMETERS
7.562
7.532
7.495
7.465
7.313
7.306
7.291
7.283
7.258
6.895
6.873
6.866
4.885
1.635
OH
NH
O
N
2.05
2.02
2.01
1.00
2.00
0.97
6.18
9.5 9.0 8.5 8.0 7.5 7.0 6.5 6.0 5.5 5.0 4.5 4.0 3.5 3.0 2.5 2.0 1.5 1.0 0.5 0.0
f1 (ppm)

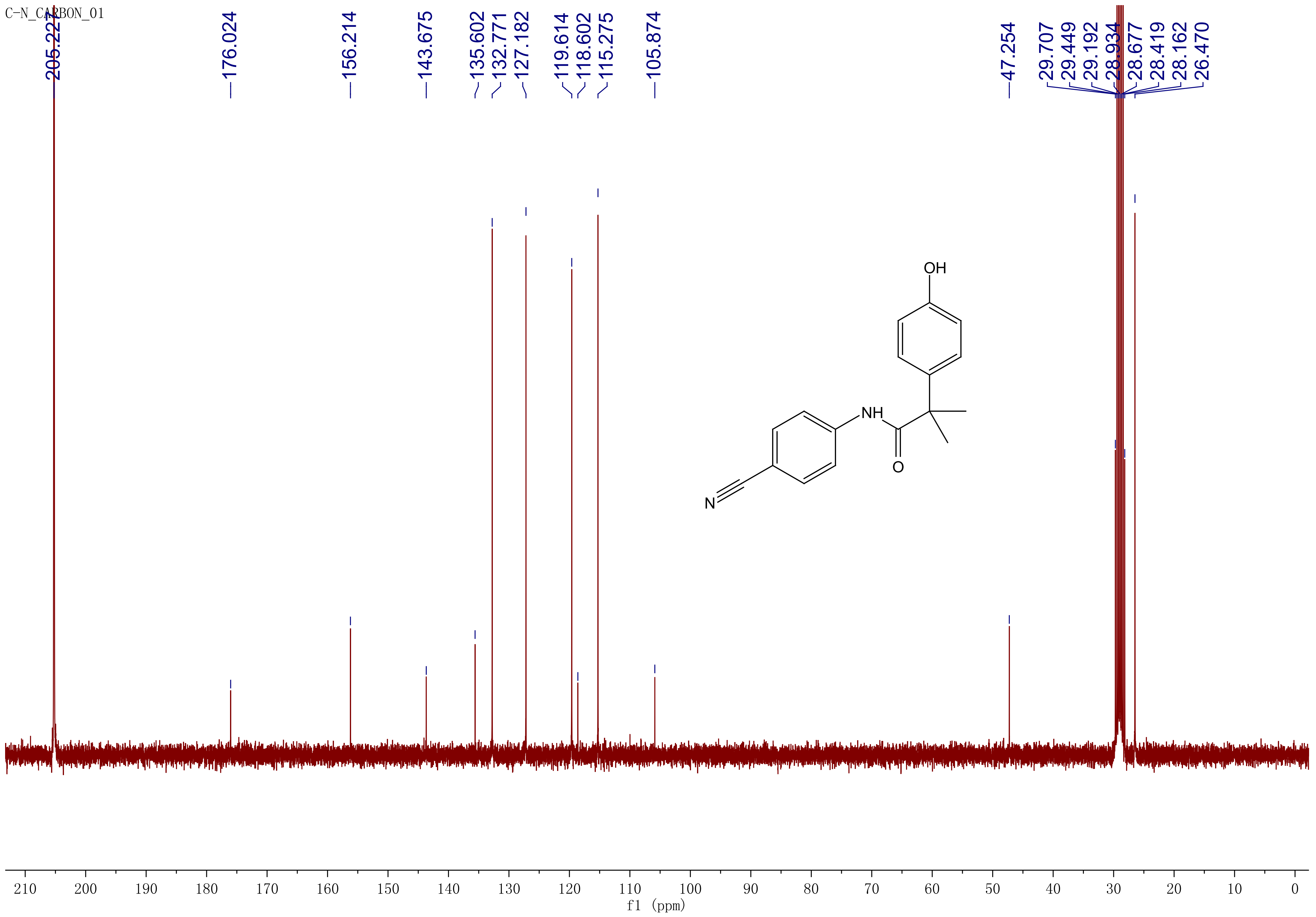
C-N_CARBON_01
205.227
176.024
156.214
143.675
135.602
132.771
127.182
119.614
118.602
115.275
105.874
47.254
29.707
29.449
29.192
28.934
28.677
28.419
28.162
26.470
OH
NH
O
N
210 200 190 180 170 160 150 140 130 120 110 100 90 80 70 60 50 40 30 20 10 0
f1 (ppm)

nitrogood proton
STANDARD PROTON PARAMETERS

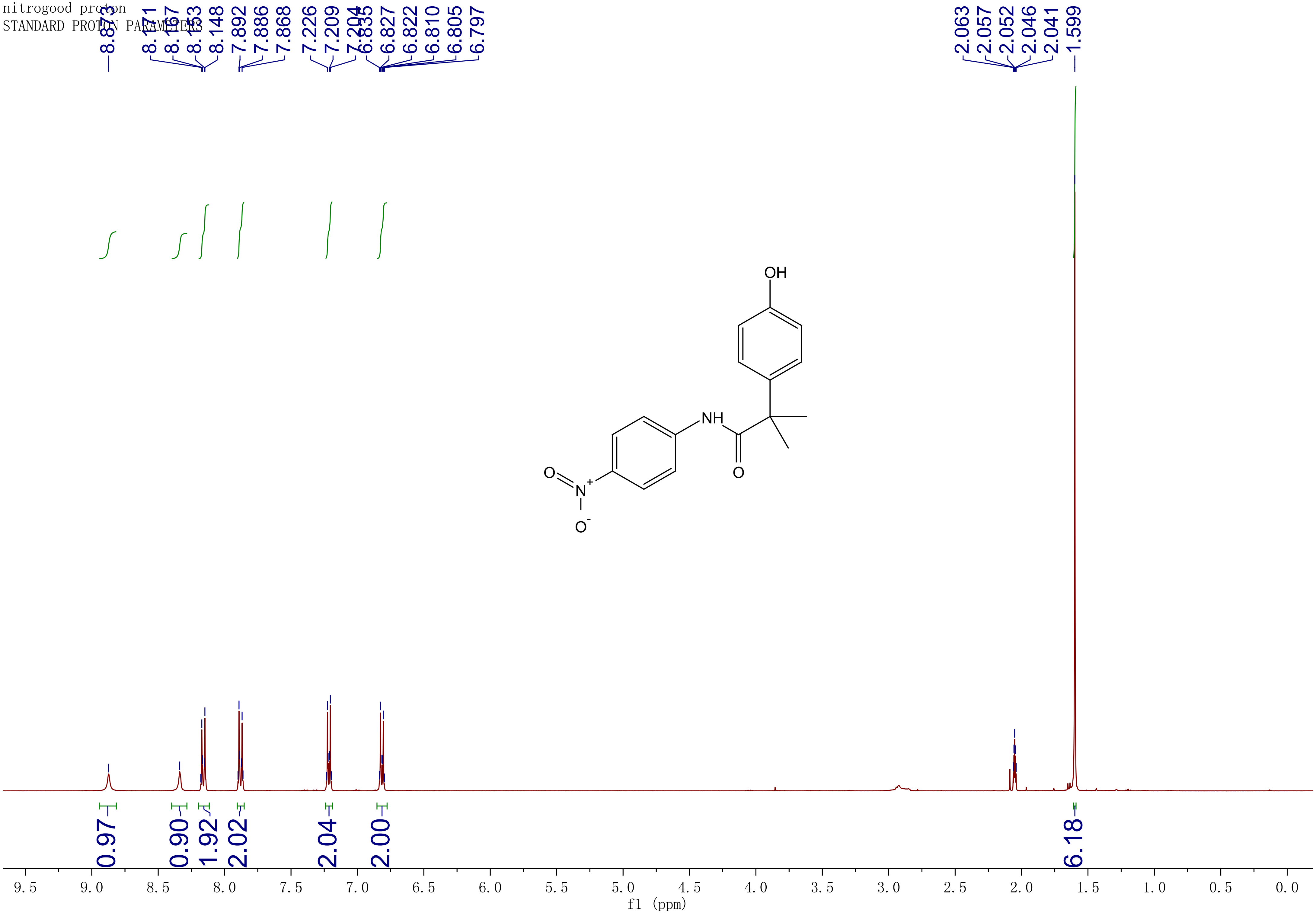

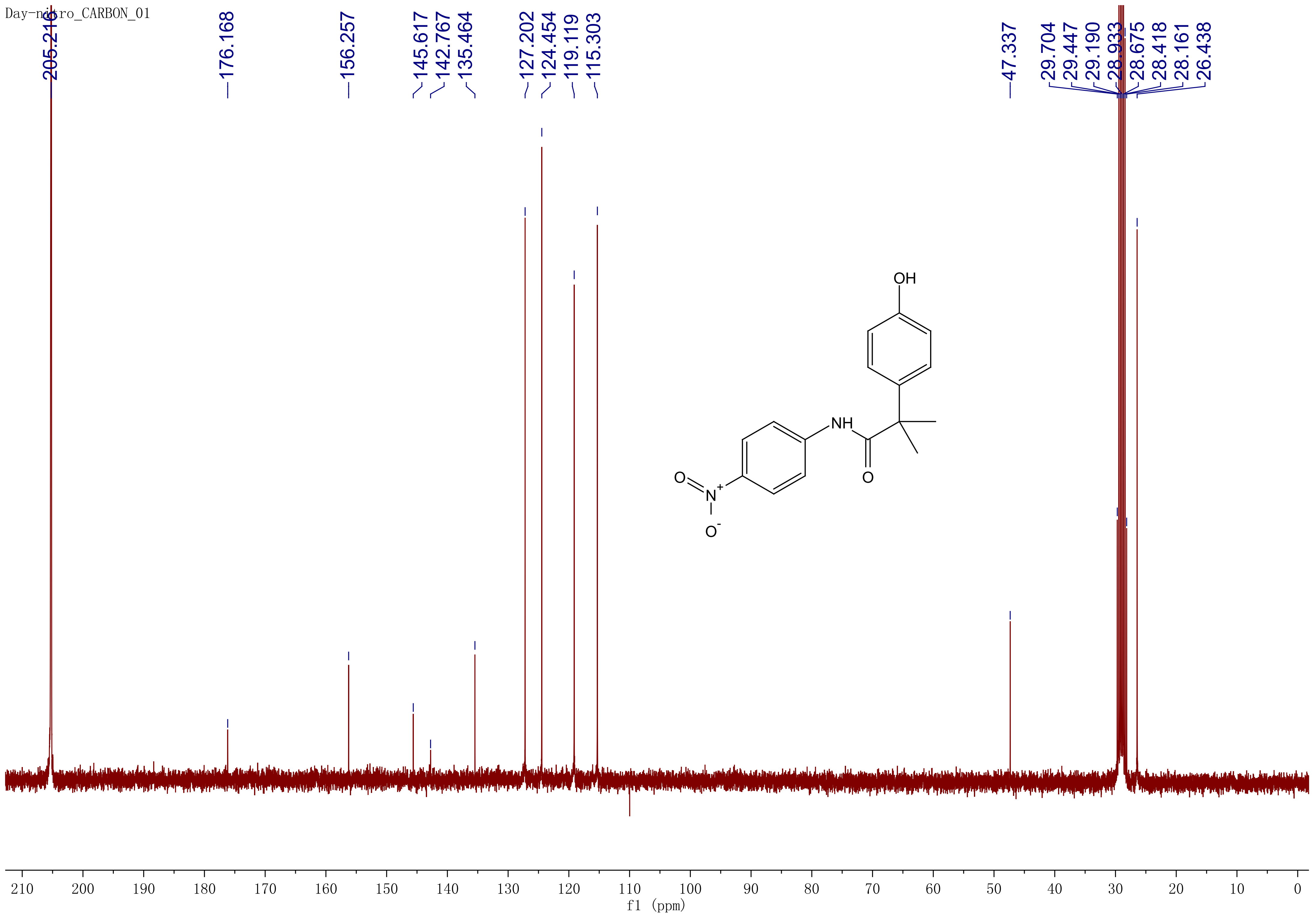

Day-nitro_CARBON_01
205.216
176.168
156.257
145.617
142.767
135.464
127.202
124.454
119.119
115.303
47.337
29.704
29.447
29.190
28.933
28.675
28.418
28.161
26.438
OH
NH
O
O
N+
O-
210 200 190 180 170 160 150 140 130 120 110 100 90 80 70 60 50 40 30 20 10 0
f1 (ppm)

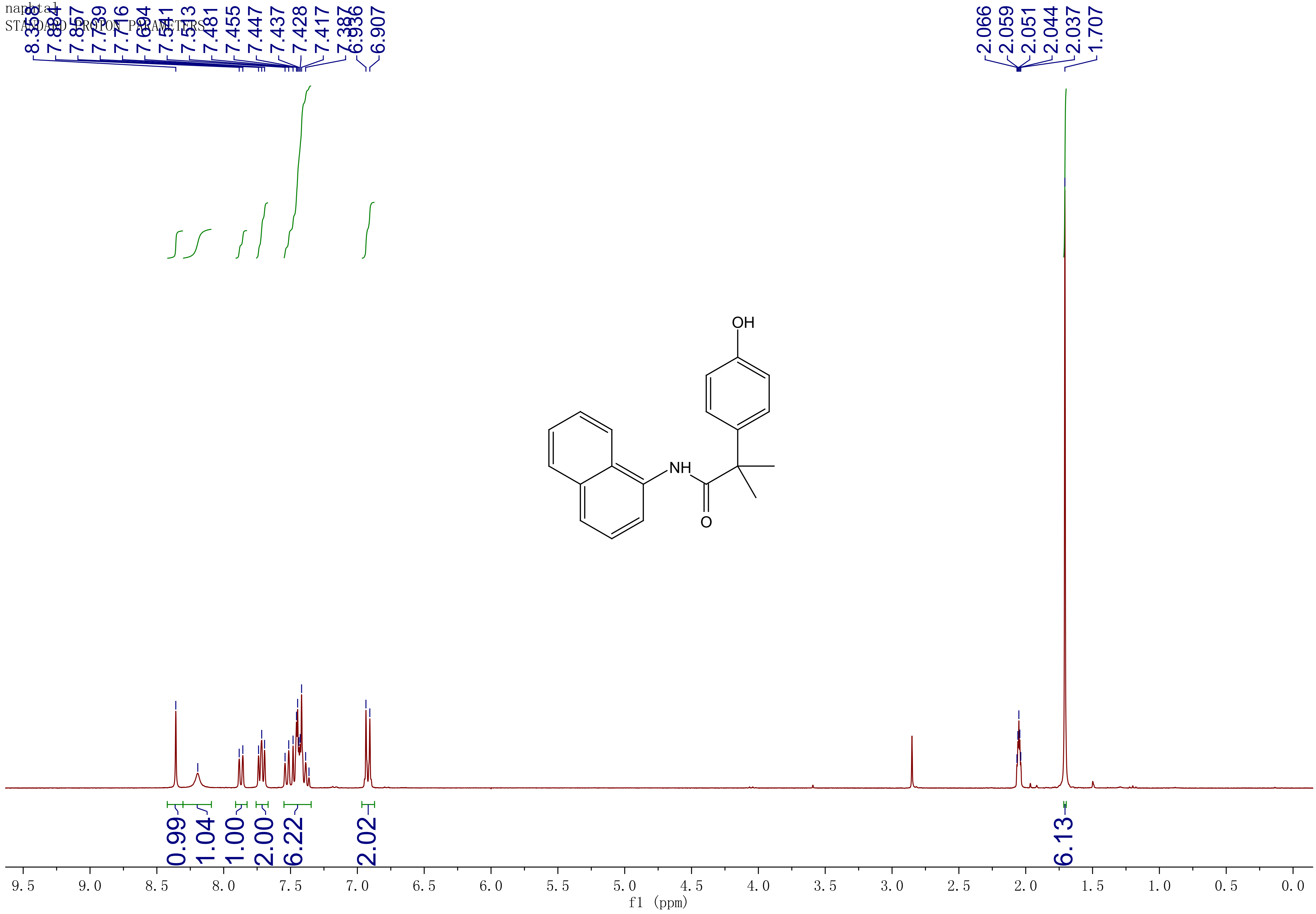
naphtal
STANDARD PROTON PARAMETERS
8.358
7.884
7.857
7.739
7.716
7.694
7.541
7.513
7.481
7.455
7.447
7.437
7.428
7.417
7.387
6.936
6.907
2.066
2.059
2.051
2.044
2.037
1.707
OH
NH
O
0.99
1.04
1.00
2.00
6.22
2.02
6.13
9.5
9.0
8.5
8.0
7.5
7.0
6.5
6.0
5.5
5.0
4.5
4.0
3.5
3.0
2.5
2.0
1.5
1.0
0.5
0.0
f1 (ppm)

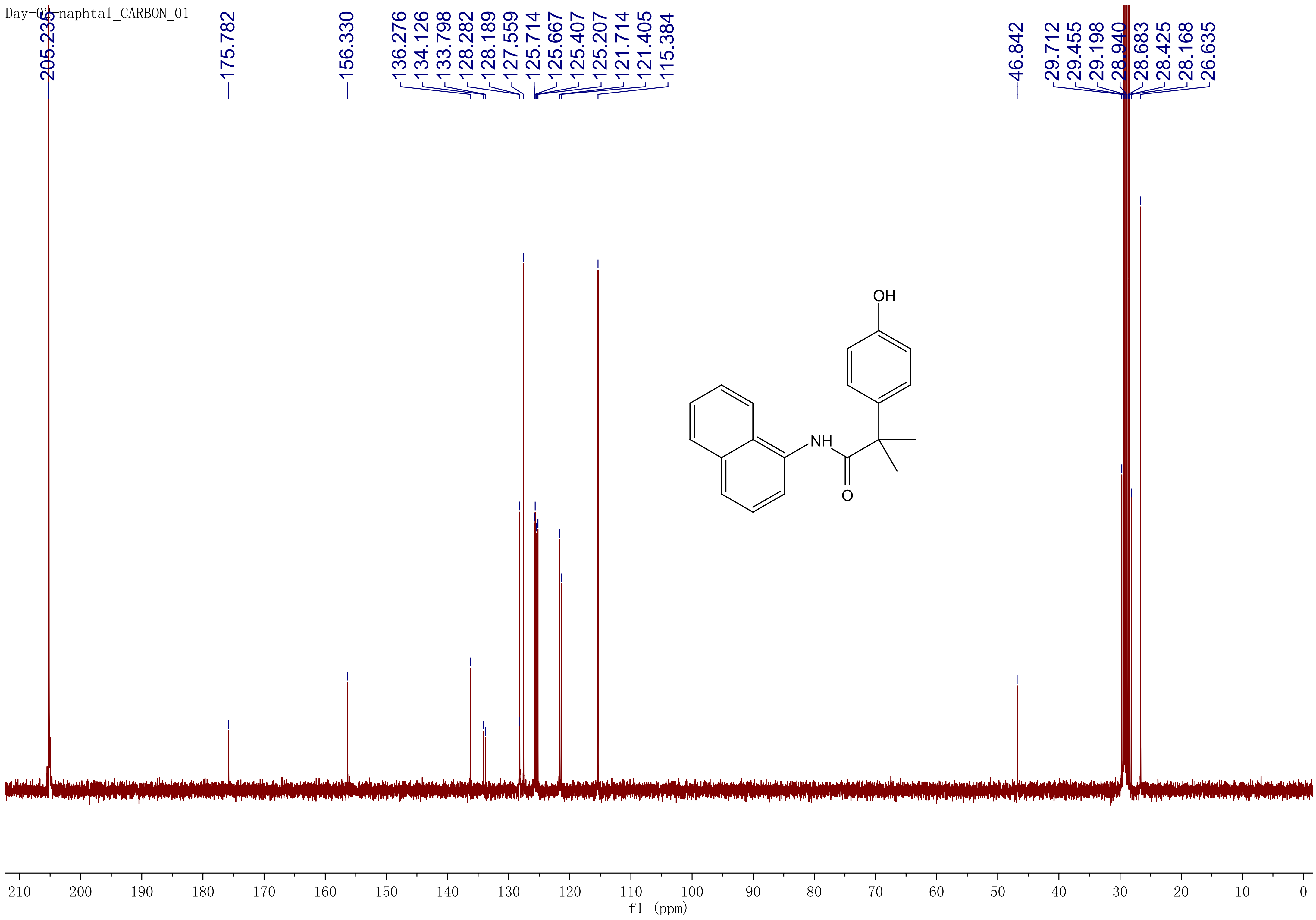

naphtal_CARBON_01
205.235
175.782
156.330
136.276
134.126
133.798
128.282
128.189
127.559
125.714
125.667
125.407
125.207
121.714
121.405
115.384
46.842
29.712
29.455
29.198
28.940
28.683
28.425
28.168
26.635
OH
NH
O
210 200 190 180 170 160 150 140 130 120 110 100 90 80 70 60 50 40 30 20 10 0
f1 (ppm)

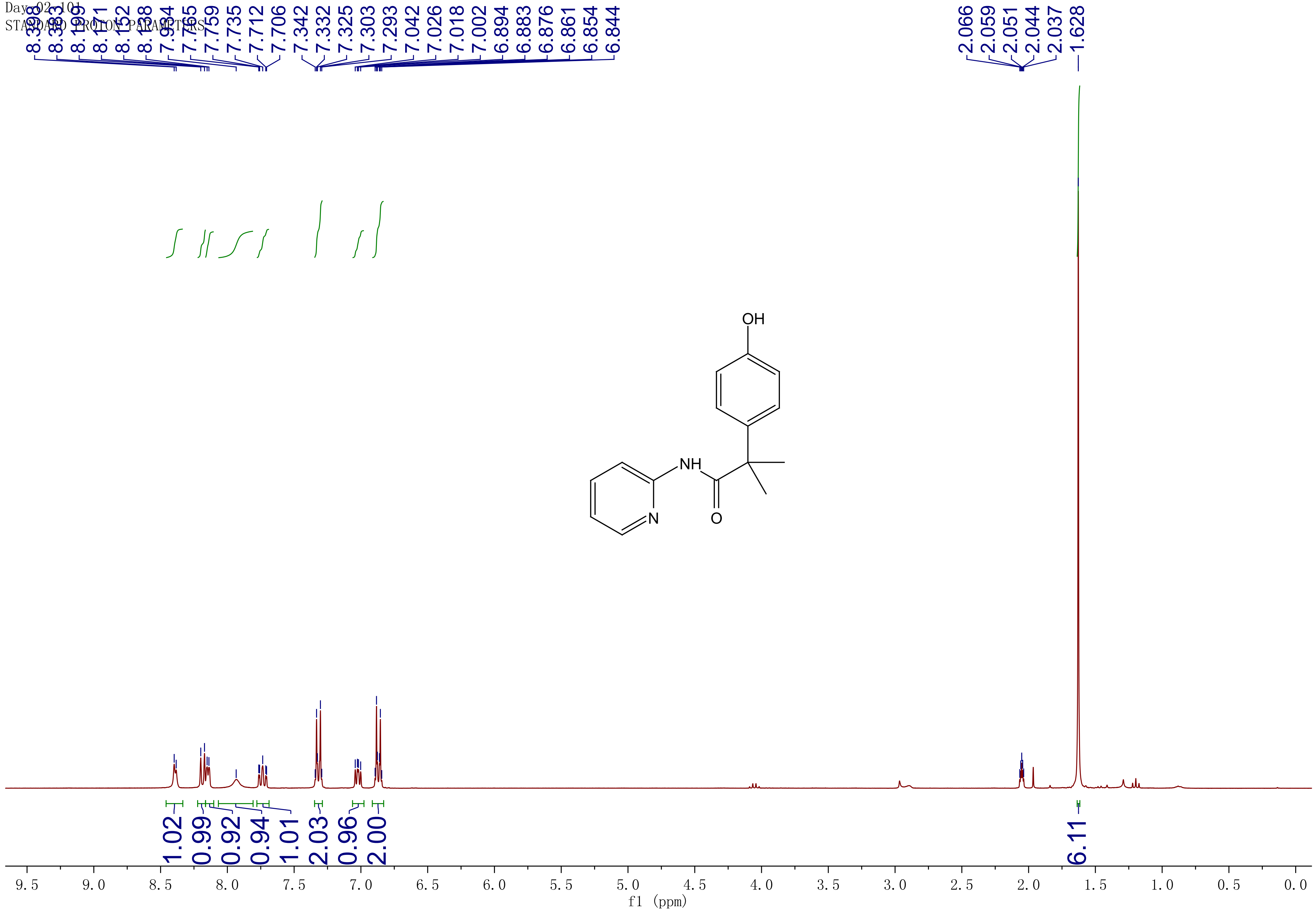
Day-02-101
STANDARD PROTON PARAMETERS
8.398
8.383
8.199
8.171
8.152
8.138
7.934
7.765
7.759
7.735
7.712
7.706
7.342
7.332
7.325
7.303
7.293
7.042
7.026
7.018
7.002
6.894
6.883
6.876
6.861
6.854
6.844
2.066
2.059
2.051
2.044
2.037
1.628
OH
NH
N
O
1.02
0.99
0.92
0.94
1.01
2.03
0.96
2.00
6.11
9.5 9.0 8.5 8.0 7.5 7.0 6.5 6.0 5.5 5.0 4.5 4.0 3.5 3.0 2.5 2.0 1.5 1.0 0.5 0.0
f1 (ppm)

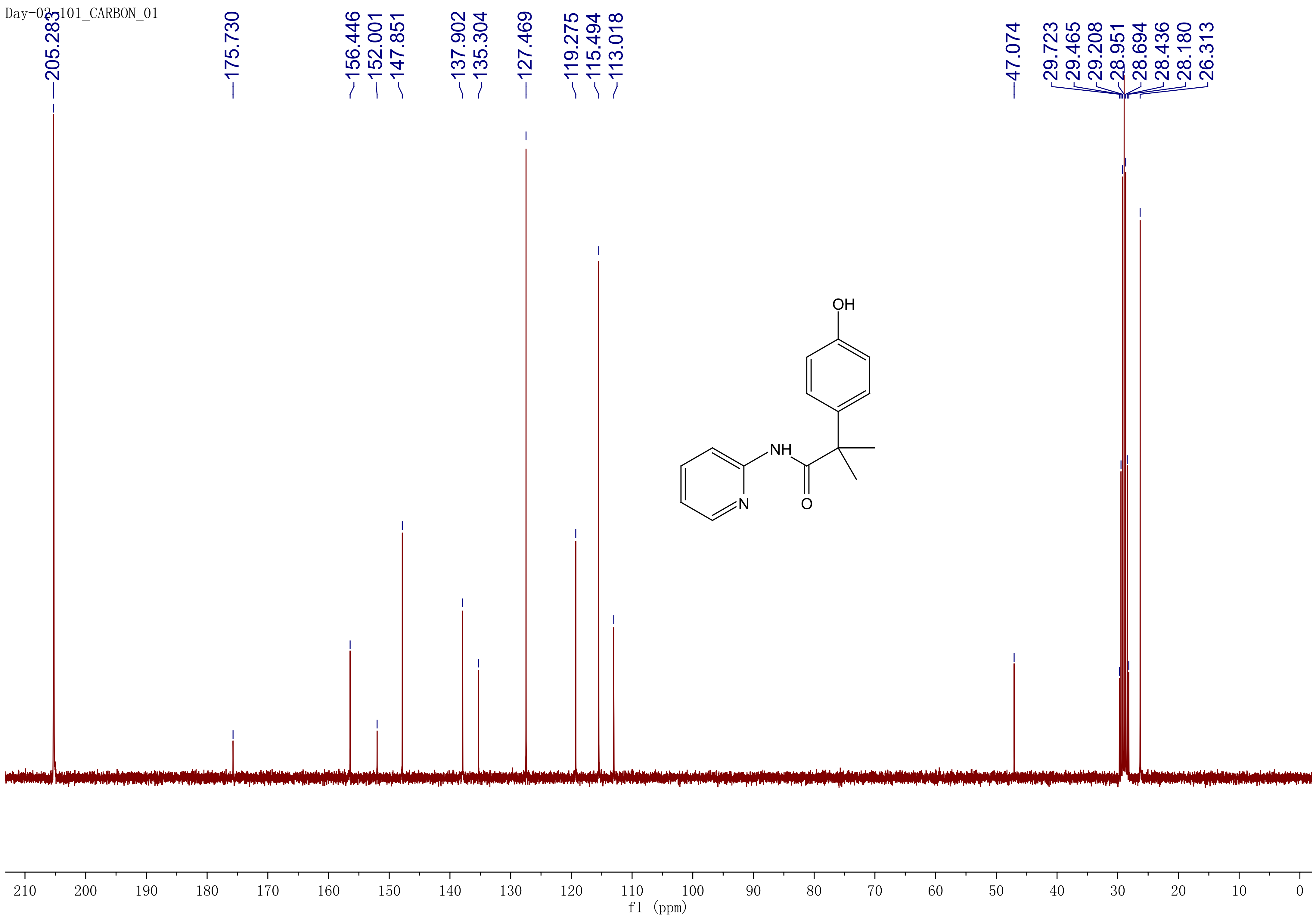
Day-02-101_CARBON_01
205.283
175.730
156.446
152.001
147.851
137.902
135.304
127.469
119.275
115.494
113.018
47.074
29.723
29.465
29.208
28.951
28.694
28.436
28.180
26.313
OH
NH
N
O
210 200 190 180 170 160 150 140 130 120 110 100 90 80 70 60 50 40 30 20 10 0
f1 (ppm)

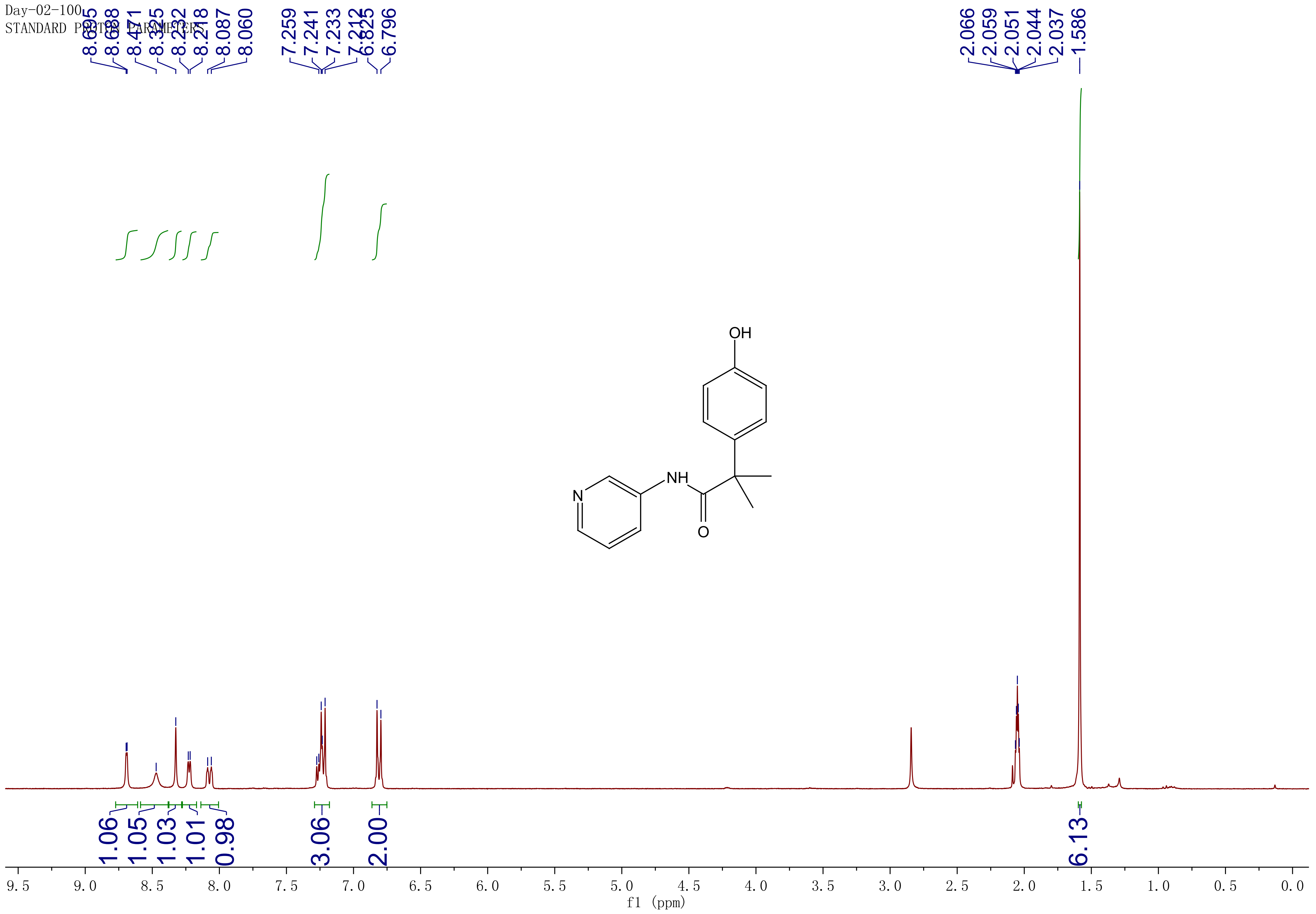

Day-02-100
STANDARD PROTON PARAMETERS
8.695
8.688
8.471
8.325
8.222
8.218
8.087
8.060
7.259
7.241
7.233
7.212
6.825
6.796
2.066
2.059
2.051
2.044
2.037
1.586
OH
NH
N
O
1.06
1.05
1.03
1.01
0.98
3.06
2.00
6.13
9.5
9.0
8.5
8.0
7.5
7.0
6.5
6.0
5.5
5.0
4.5
4.0
3.5
3.0
2.5
2.0
1.5
1.0
0.5
0.0
f1 (ppm)

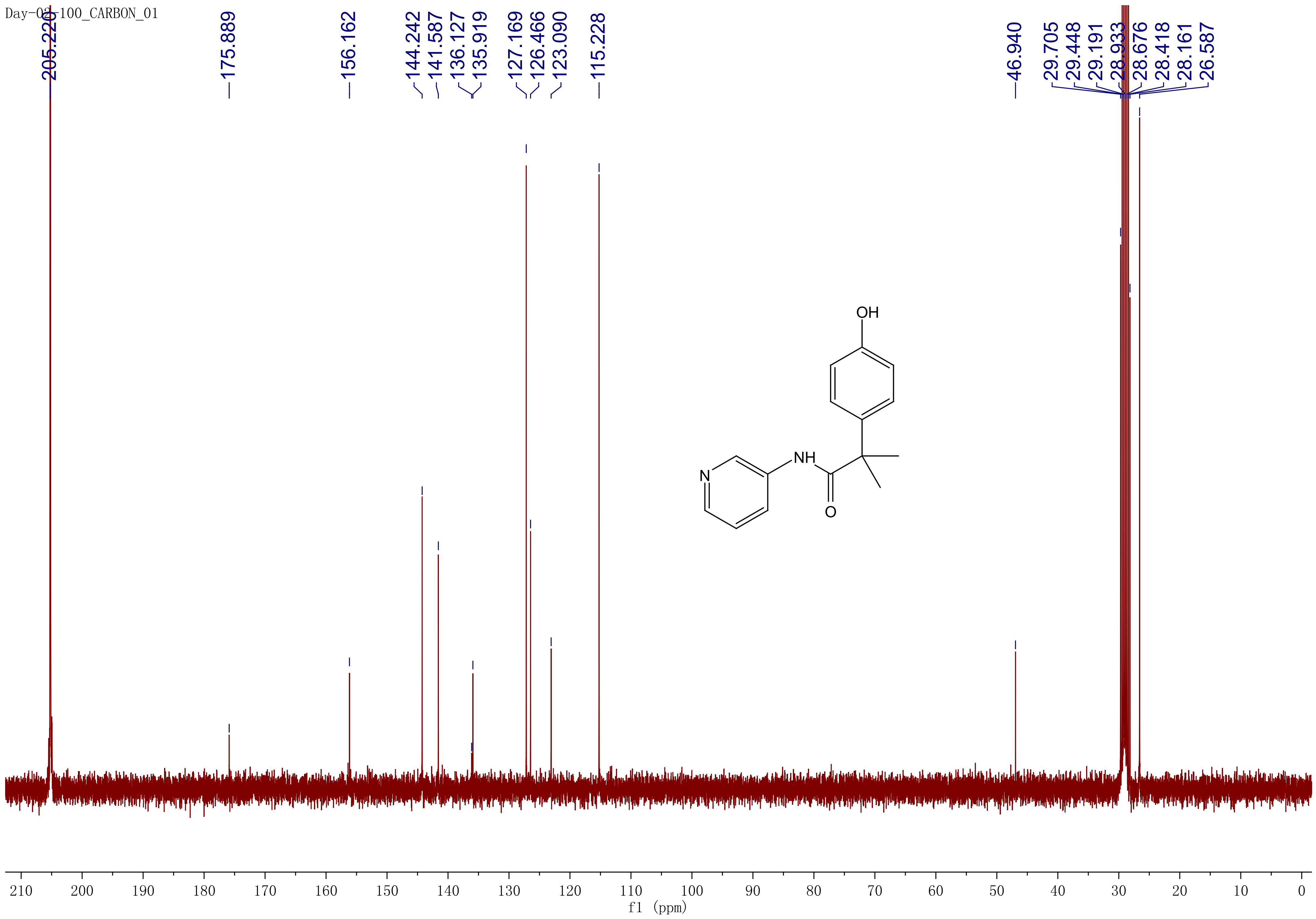
Day-02-100_CARBON_01
205.220
175.889
156.162
144.242
141.587
136.127
135.919
127.169
126.466
123.090
115.228
46.940
29.705
29.448
29.191
28.933
28.676
28.418
28.161
26.587
OH
NH
N
O
210 200 190 180 170 160 150 140 130 120 110 100 90 80 70 60 50 40 30 20 10 0
f1 (ppm)

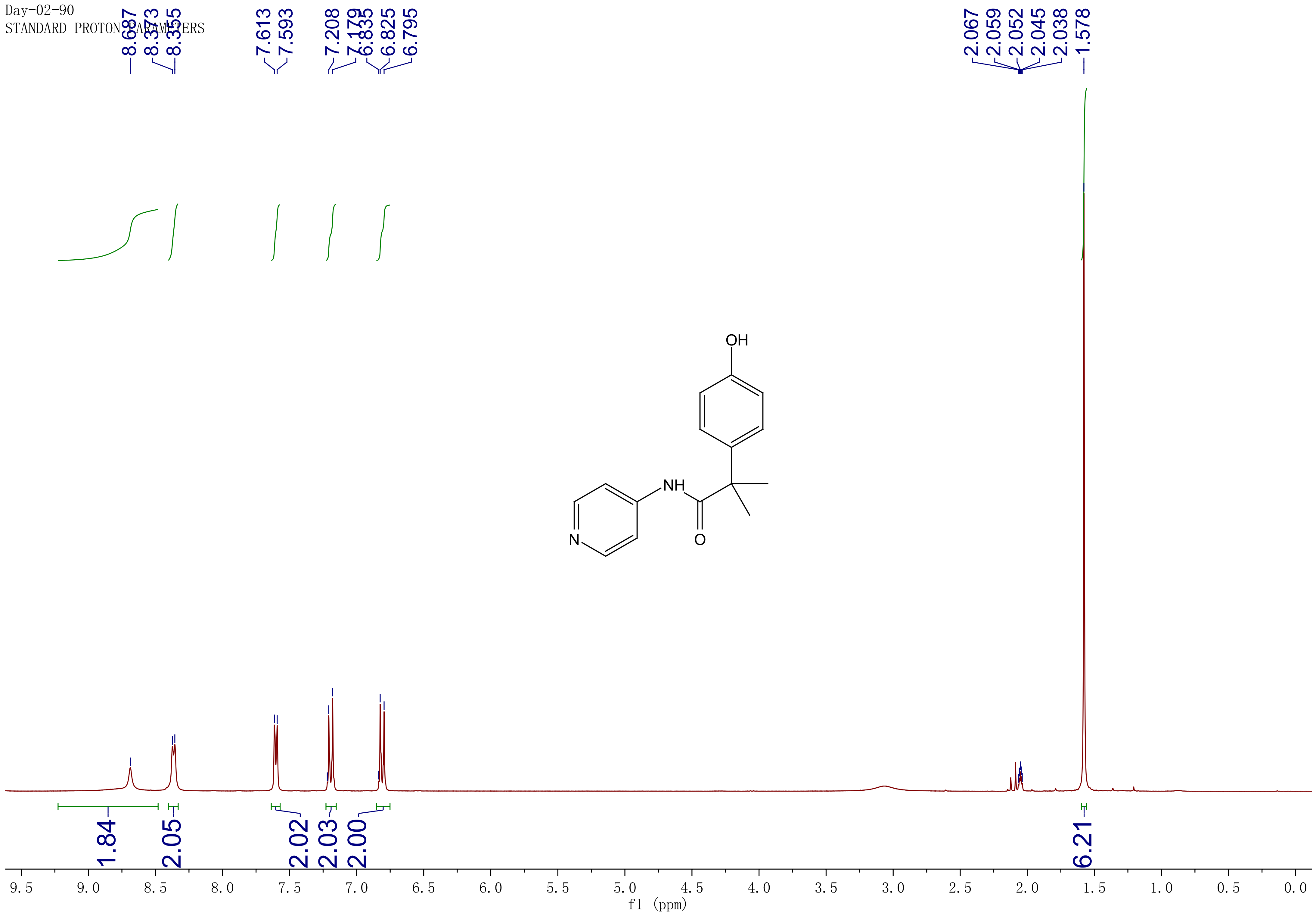
Day-02-90
STANDARD PROTON PARAMETERS
8.687
8.373
8.355
7.613
7.593
7.208
7.179
6.835
6.825
6.795
2.067
2.059
2.052
2.045
2.038
1.578
OH
NH
N
O
1.84
2.05
2.02
2.03
2.00
6.21
9.5
9.0
8.5
8.0
7.5
7.0
6.5
6.0
5.5
5.0
4.5
4.0
3.5
3.0
2.5
2.0
1.5
1.0
0.5
0.0
f1 (ppm)

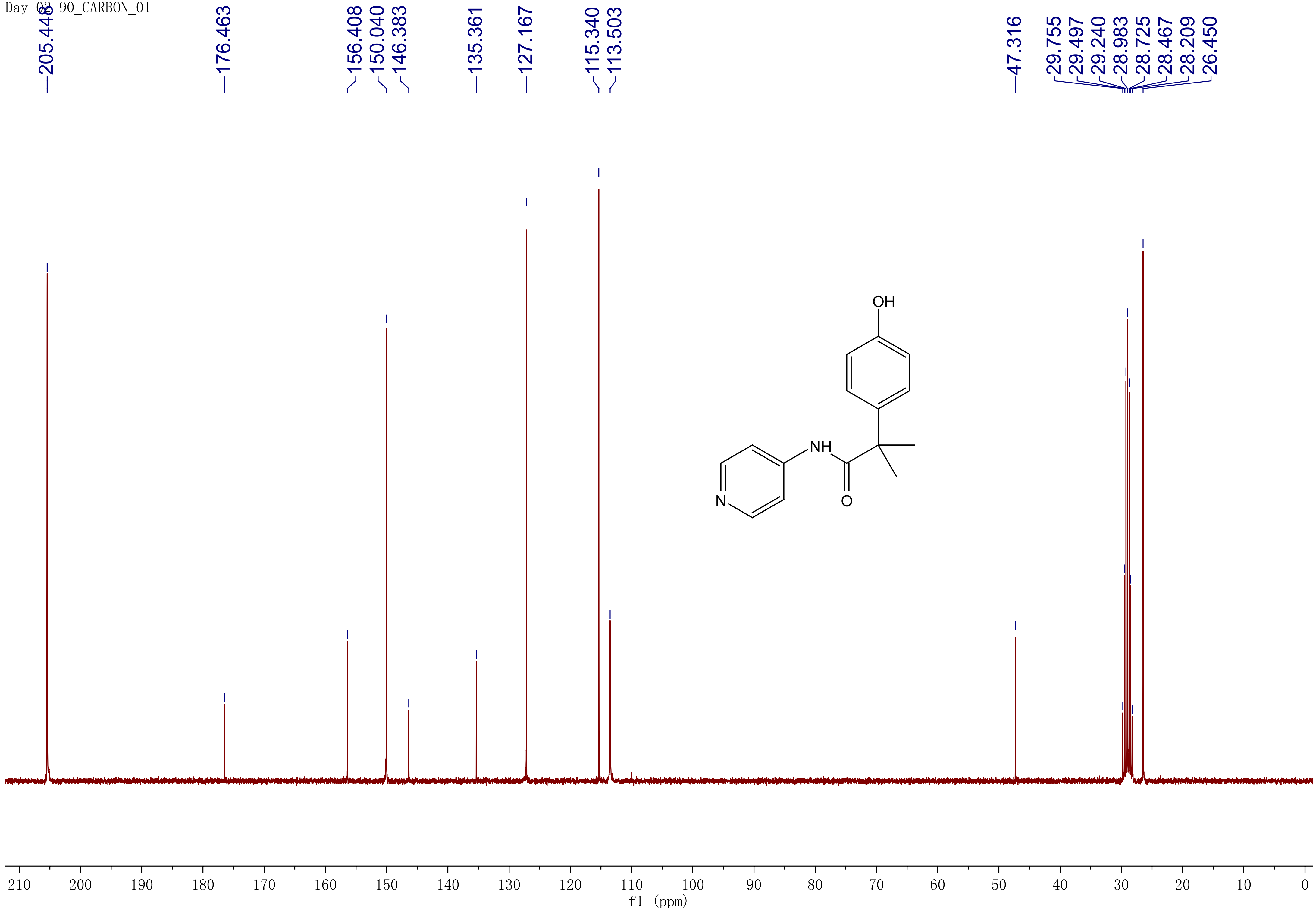
Day-02-90_CARBON_01
205.448
176.463
156.408
150.040
146.383
135.361
127.167
115.340
113.503
47.316
29.755
29.497
29.240
28.983
28.725
28.467
28.209
26.450
OH
NH
N
O
210 200 190 180 170 160 150 140 130 120 110 100 90 80 70 60 50 40 30 20 10 0
f1 (ppm)

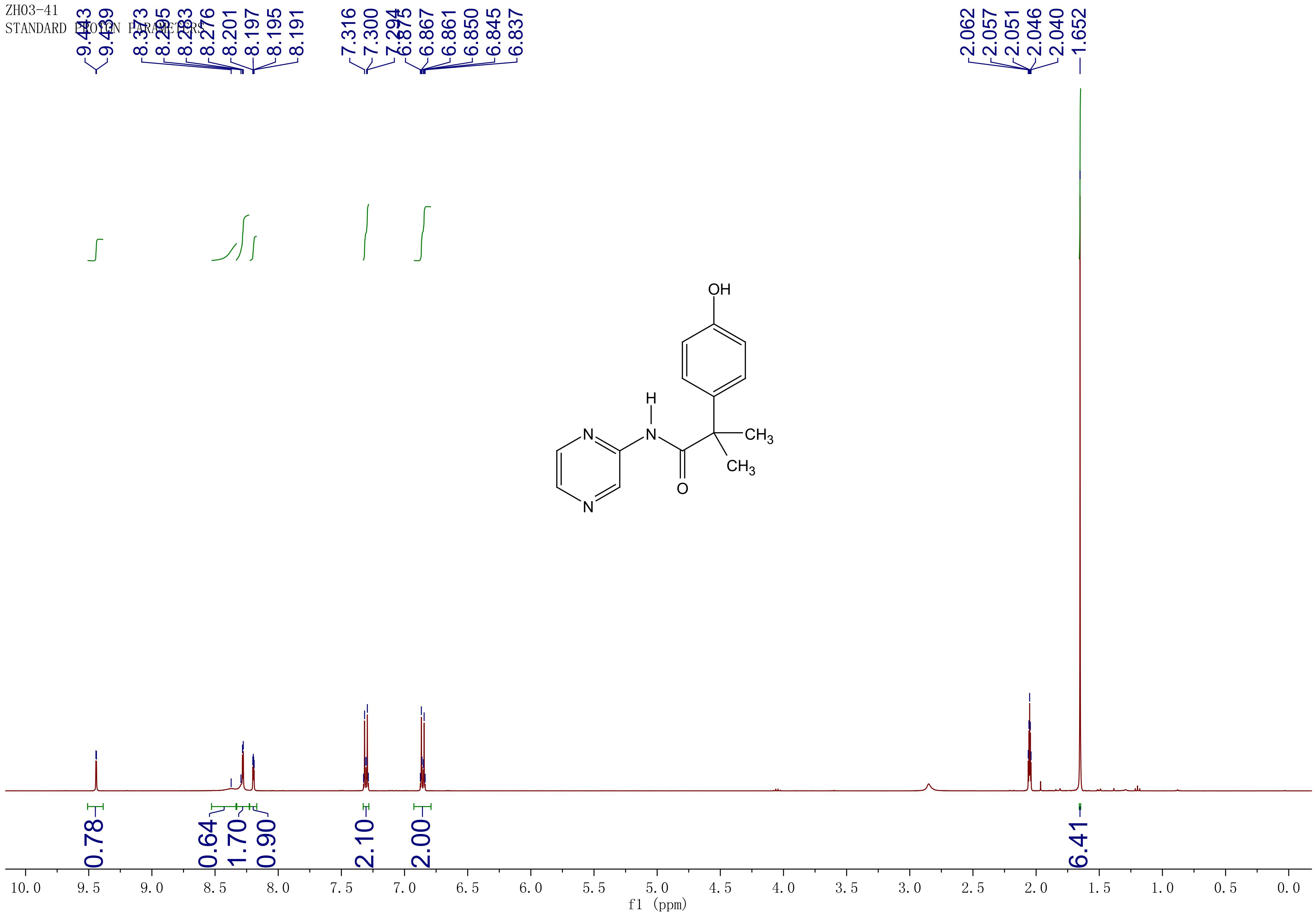
ZH03-41
STANDARD PROTON PARAMETERS
9.443
9.439
8.373
8.295
8.283
8.276
8.201
8.197
8.195
8.191
7.316
7.300
7.294
6.875
6.867
6.861
6.850
6.845
6.837
2.062
2.057
2.051
2.046
2.040
1.652
OH
H
N
N
N
CH3
CH3
O
0.78
0.64
1.70
0.90
2.10
2.00
6.41
10.0
9.5
9.0
8.5
8.0
7.5
7.0
6.5
6.0
5.5
5.0
4.5
4.0
3.5
3.0
2.5
2.0
1.5
1.0
0.5
0.0
f1 (ppm)

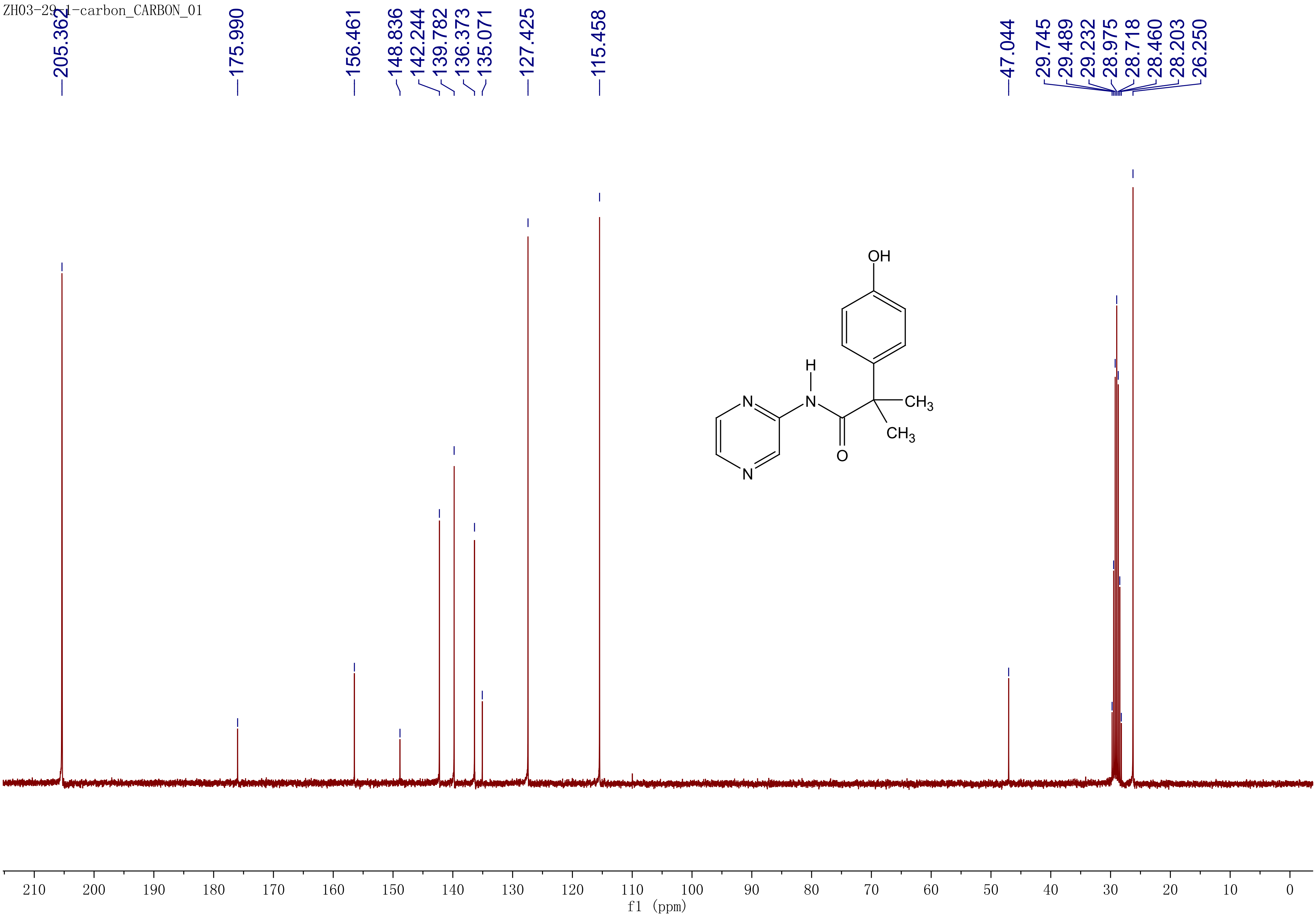
ZH03-29-1-carbon_CARBON_01
205.362
175.990
156.461
148.836
142.244
139.782
136.373
135.071
127.425
115.458
47.044
29.745
29.489
29.232
28.975
28.718
28.460
28.203
26.250
OH
H
N
N
N
O
CH3
CH3
210 200 190 180 170 160 150 140 130 120 110 100 90 80 70 60 50 40 30 20 10 0
f1 (ppm)

Day-02-108
STANDARD PROTON PARAMETERS

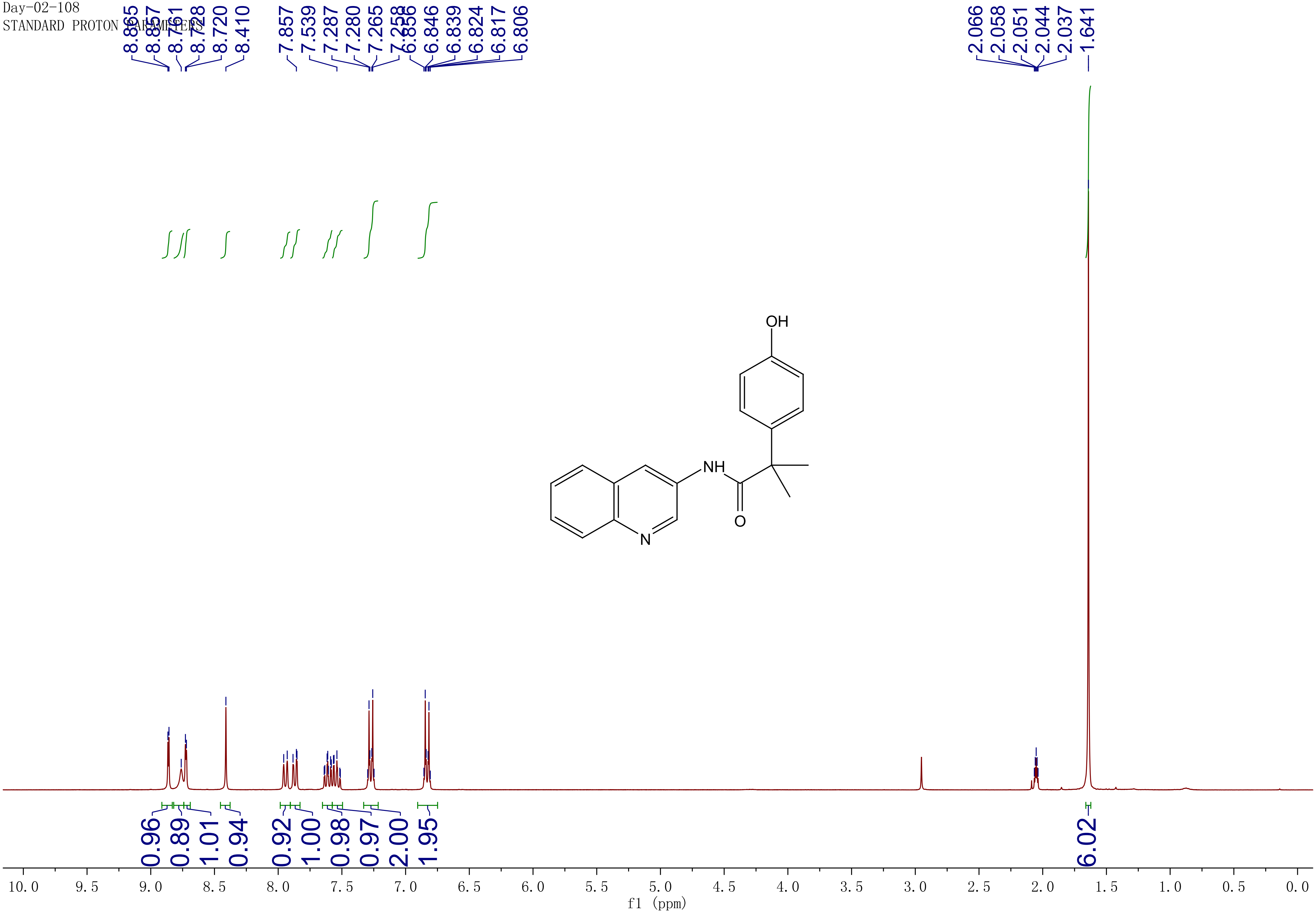

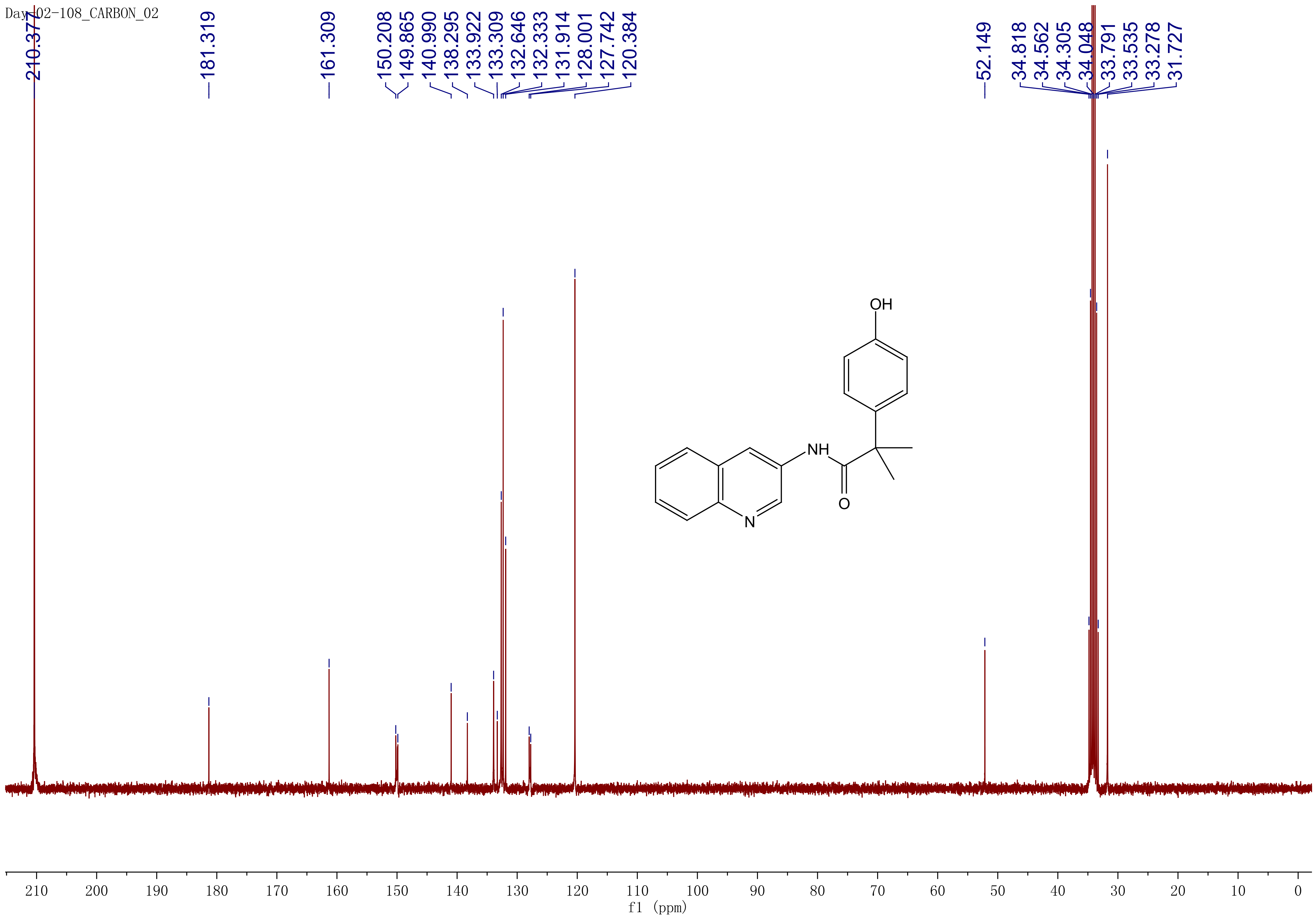
Day-02-108_CARBON_02
210.377
181.319
161.309
150.208
149.865
140.990
138.295
133.922
133.309
132.646
132.333
131.914
128.001
127.742
120.384
52.149
34.818
34.562
34.305
34.048
33.791
33.535
33.278
31.727
OH
NH
O
N
210 200 190 180 170 160 150 140 130 120 110 100 90 80 70 60 50 40 30 20 10 0
f1 (ppm)

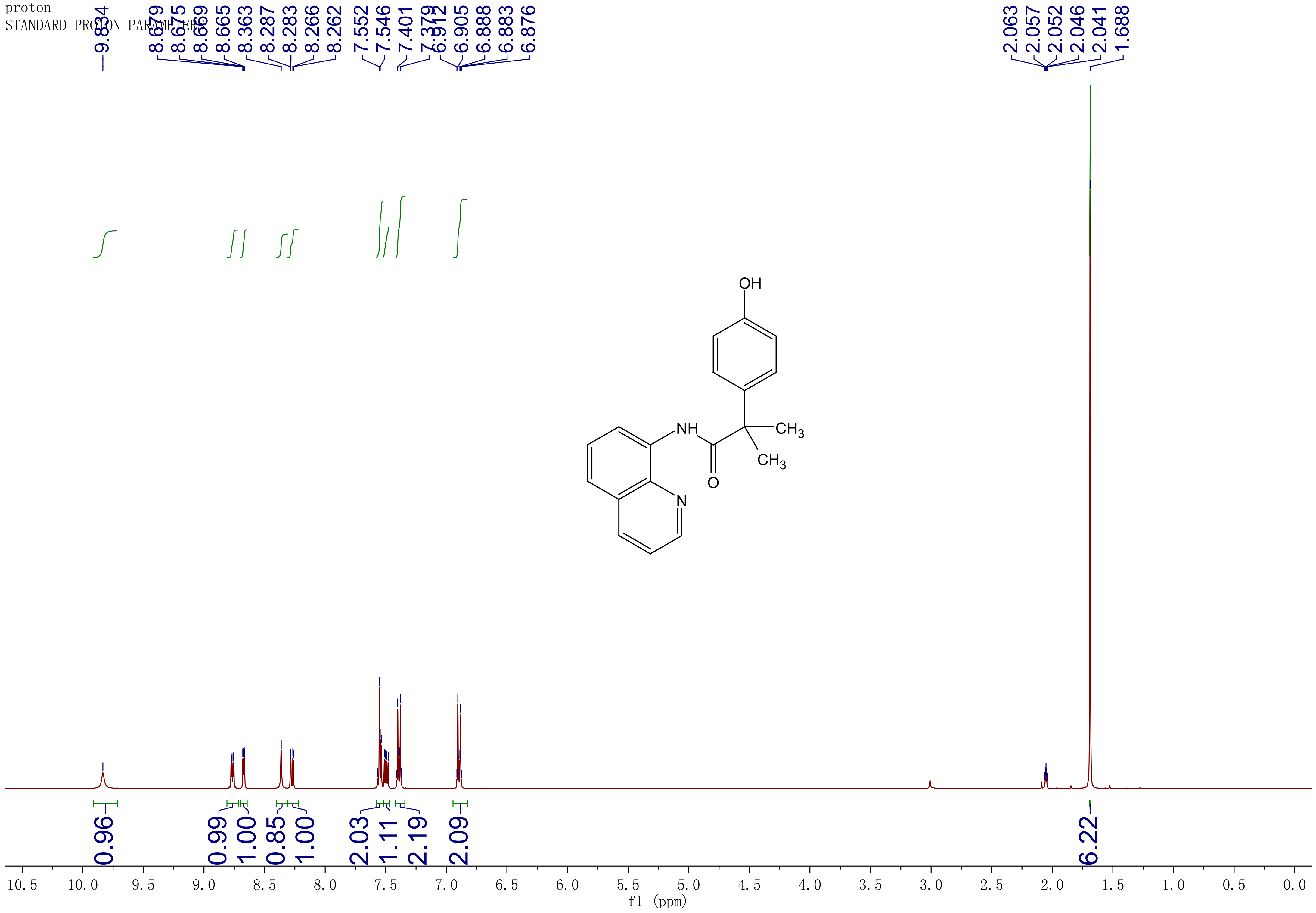
proton
STANDARD PROTON PARAMETERS
9.834
8.679
8.675
8.669
8.665
8.363
8.287
8.283
8.266
8.262
7.552
7.546
7.401
7.379
6.912
6.905
6.888
6.883
6.876
2.063
2.057
2.052
2.046
2.041
1.688
OH
NH
CH3
CH3
O
N
0.96
0.99
1.00
0.85
1.00
2.03
1.11
2.19
2.09
6.22
f1 (ppm)

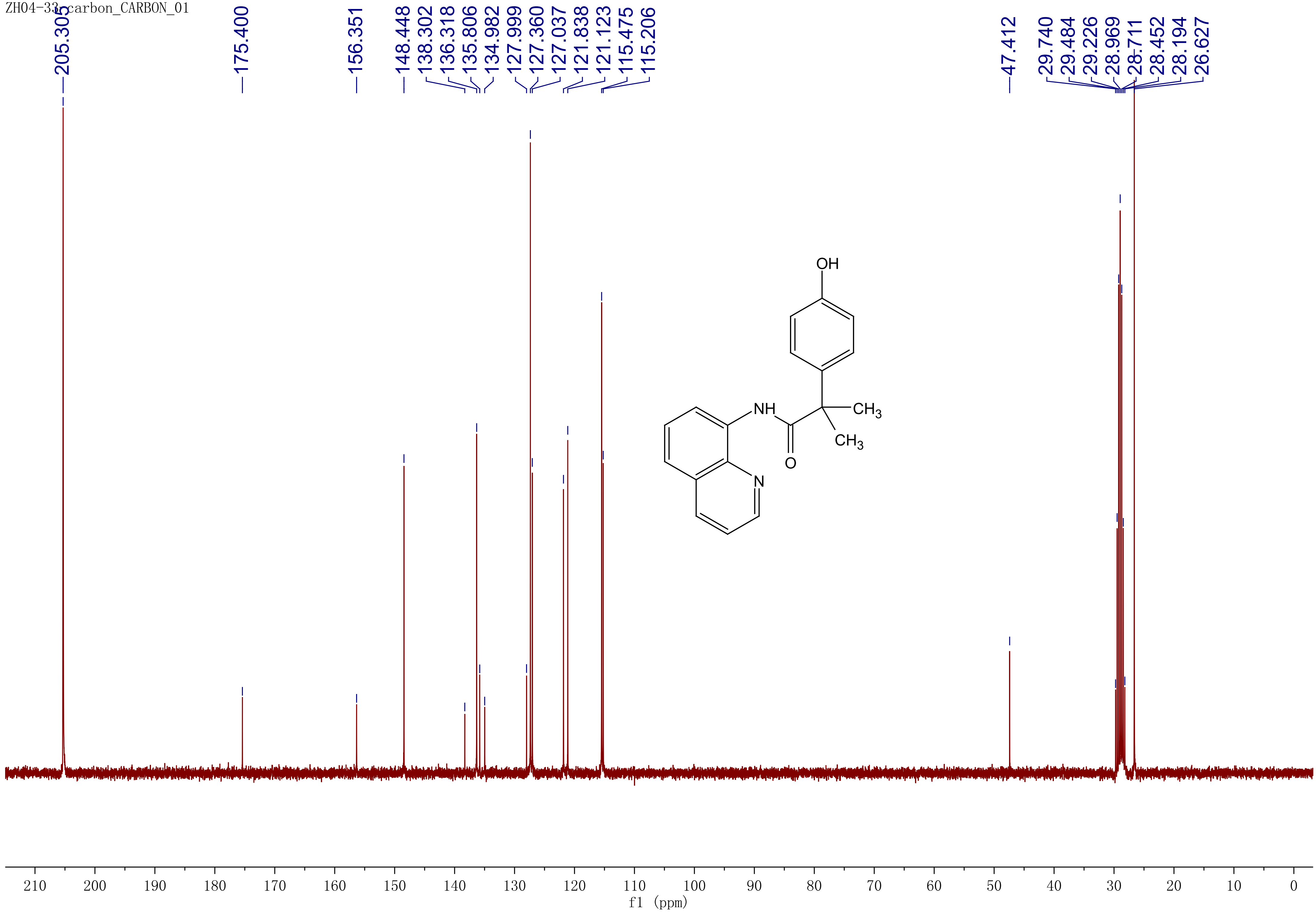
ZH04-33-carbon_CARBON_01
205.305
175.400
156.351
148.448
138.302
136.318
135.806
134.982
127.999
127.360
127.037
121.838
121.123
115.475
115.206
47.412
29.740
29.484
29.226
28.969
28.711
28.452
28.194
26.627
OH
NH
CH3
CH3
O
N
210 200 190 180 170 160 150 140 130 120 110 100 90 80 70 60 50 40 30 20 10 0
f1 (ppm)

Day-02-141
STANDARD PROTON PARAMETERS

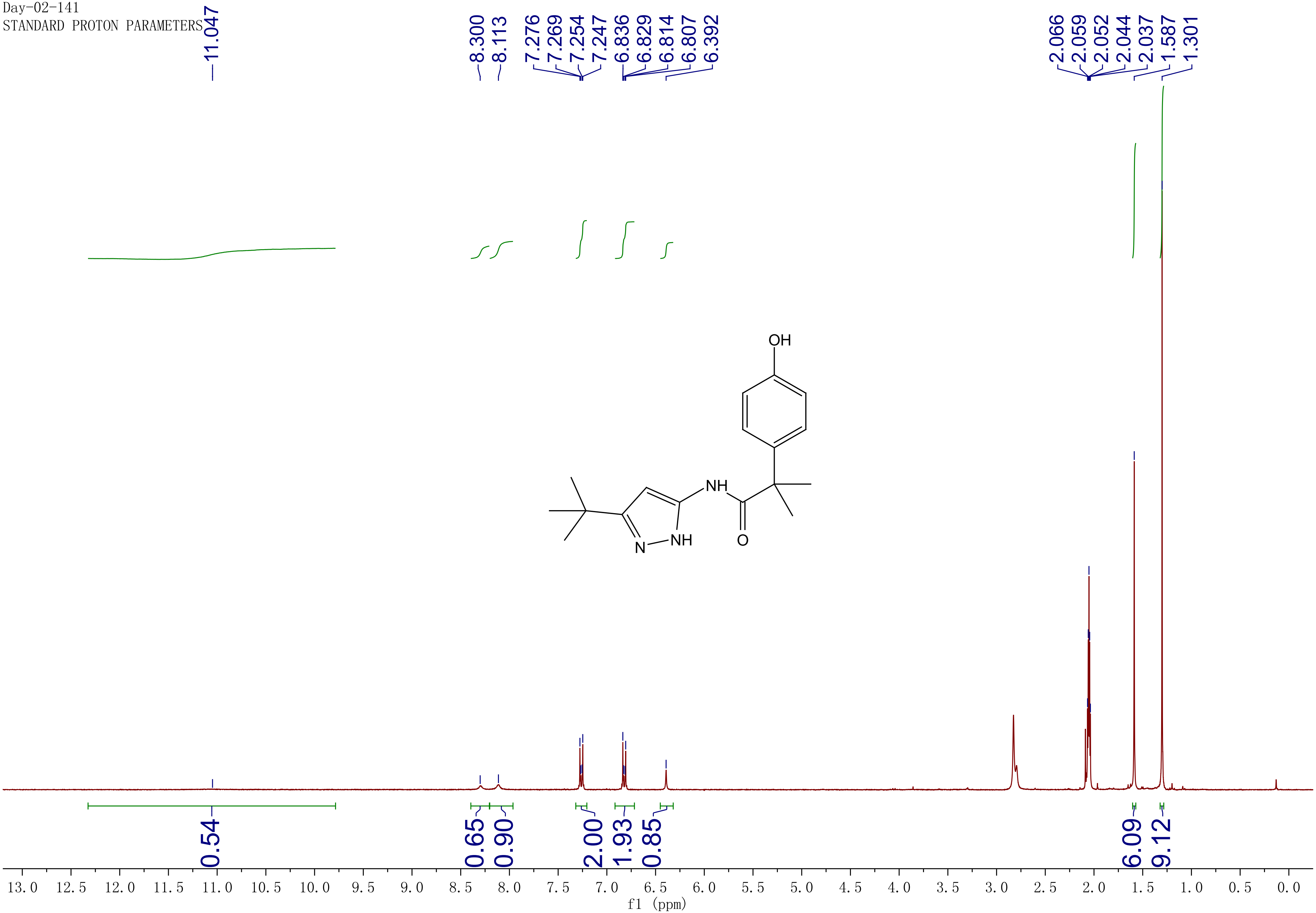

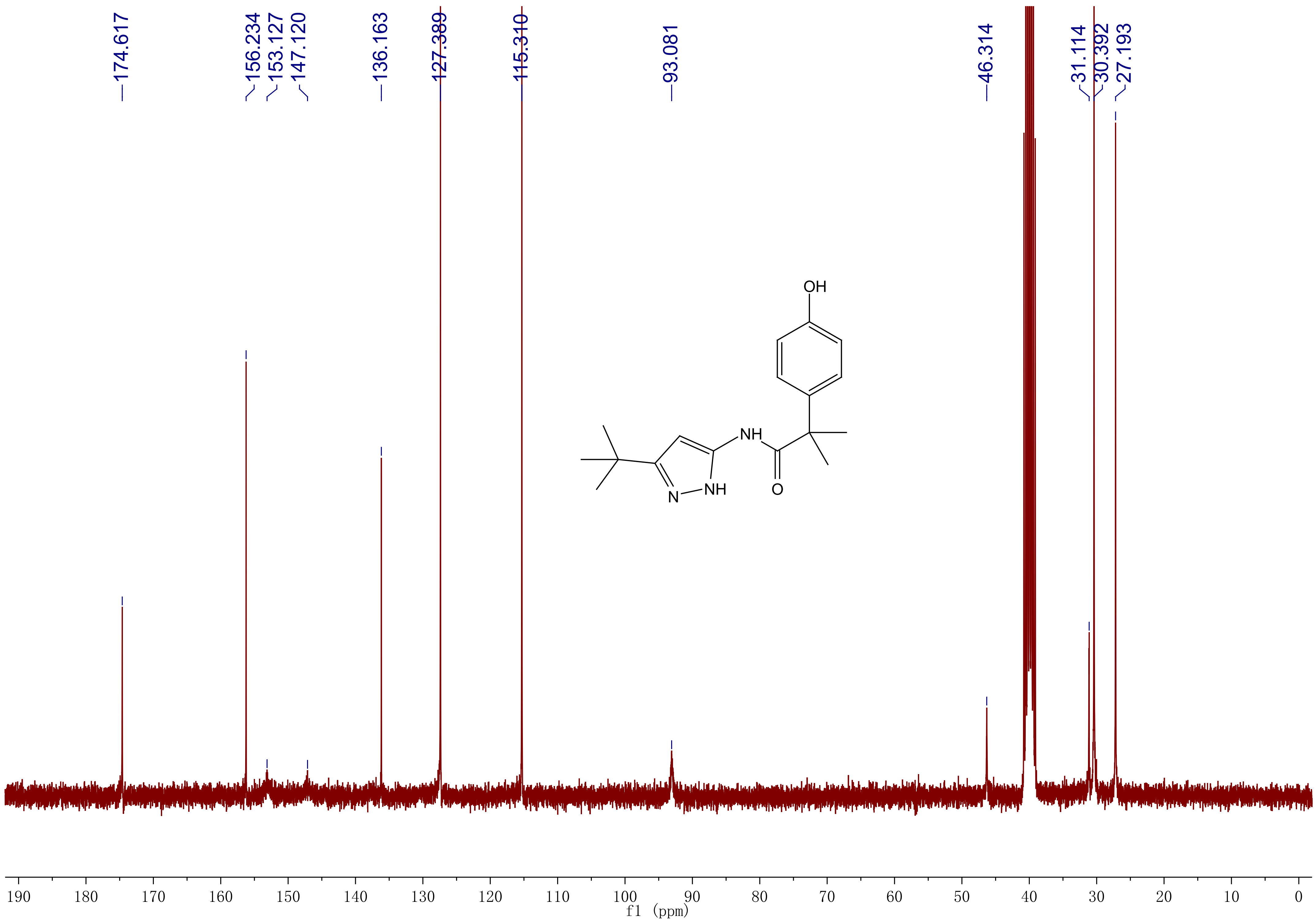

174.617
156.234
153.127
147.120
136.163
127.389
115.310
93.081
46.314
31.114
30.392
27.193
OH
NH
N
NH
O
190 180 170 160 150 140 130 120 110 100 90 80 70 60 50 40 30 20 10 0
f1 (ppm)

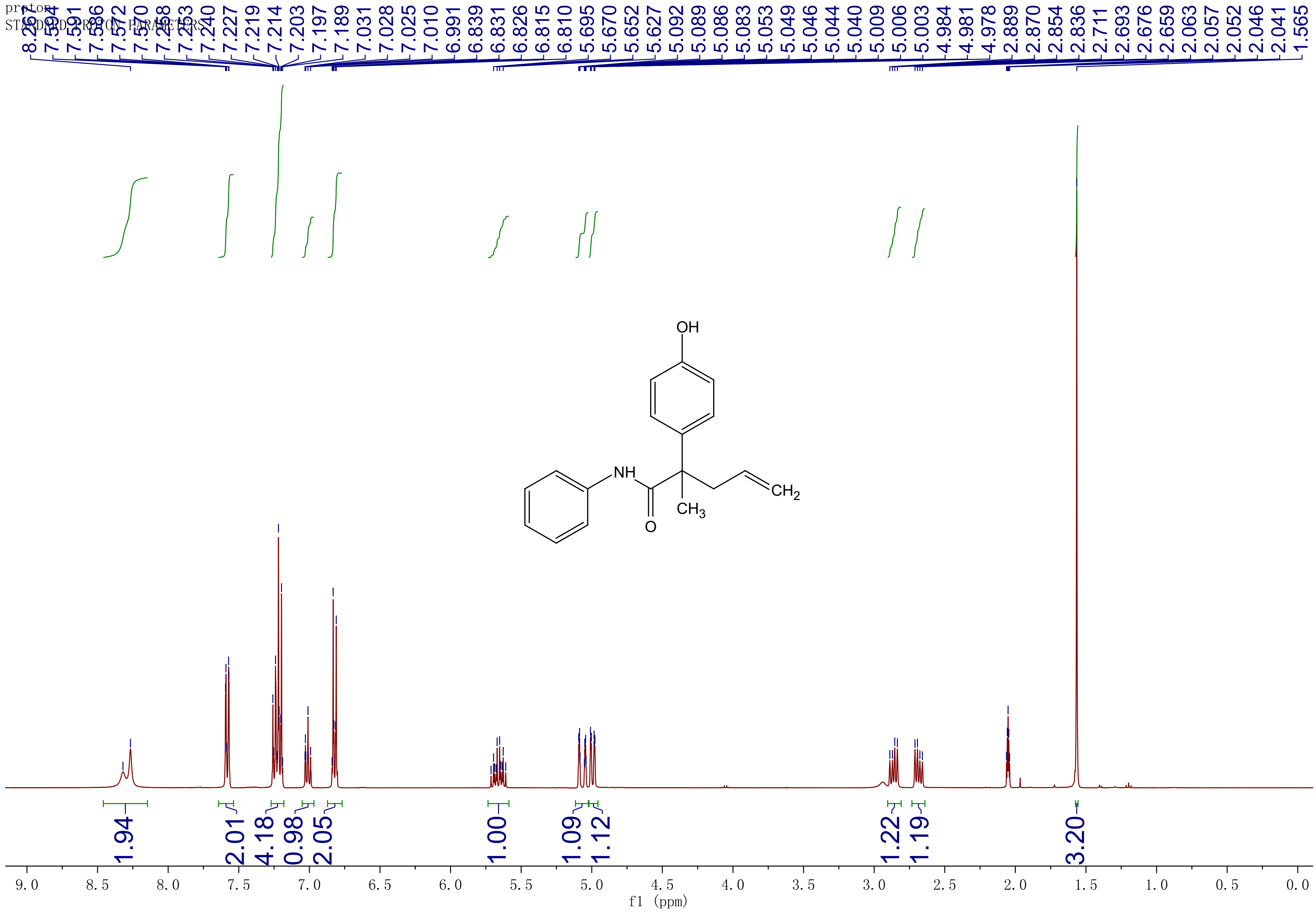
proton
STANDARD PROTON PARAMETERS
7.594
7.591
7.586
7.572
7.570
7.258
7.253
7.240
7.227
7.219
7.214
7.203
7.197
7.189
7.031
7.028
7.025
7.010
6.991
6.839
6.831
6.826
6.815
6.810
5.695
5.670
5.652
5.627
5.092
5.089
5.086
5.083
5.053
5.049
5.046
5.044
5.040
5.009
5.006
5.003
4.984
4.981
4.978
2.889
2.870
2.854
2.836
2.711
2.693
2.676
2.659
2.063
2.057
2.052
2.046
2.041
1.565
OH
NH
O
CH3
CH2
1.94
2.01
4.18
0.98
2.05
1.00
1.09
1.12
1.22
1.19
3.20
9.0
8.5
8.0
7.5
7.0
6.5
6.0
5.5
5.0
4.5
4.0
3.5
3.0
2.5
2.0
1.5
1.0
0.5
0.0
f1 (ppm)

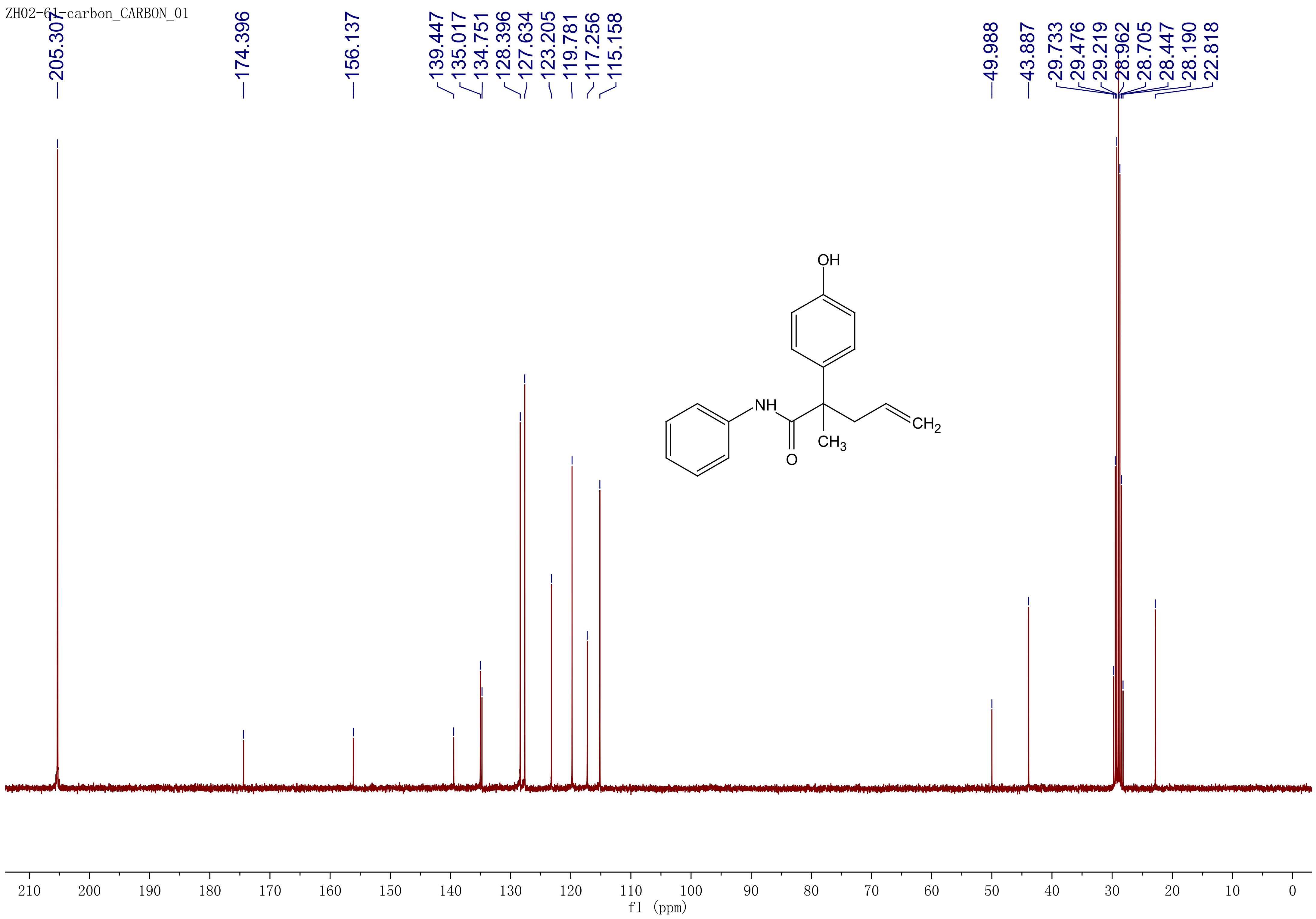
ZH02-61-carbon_CARBON_01
205.307
174.396
156.137
139.447
135.017
134.751
128.396
127.634
123.205
119.781
117.256
115.158
49.988
43.887
29.733
29.476
29.219
28.962
28.705
28.447
28.190
22.818
OH
NH
O
CH3
CH2
210 200 190 180 170 160 150 140 130 120 110 100 90 80 70 60 50 40 30 20 10 0
f1 (ppm)

ZH03-17-1

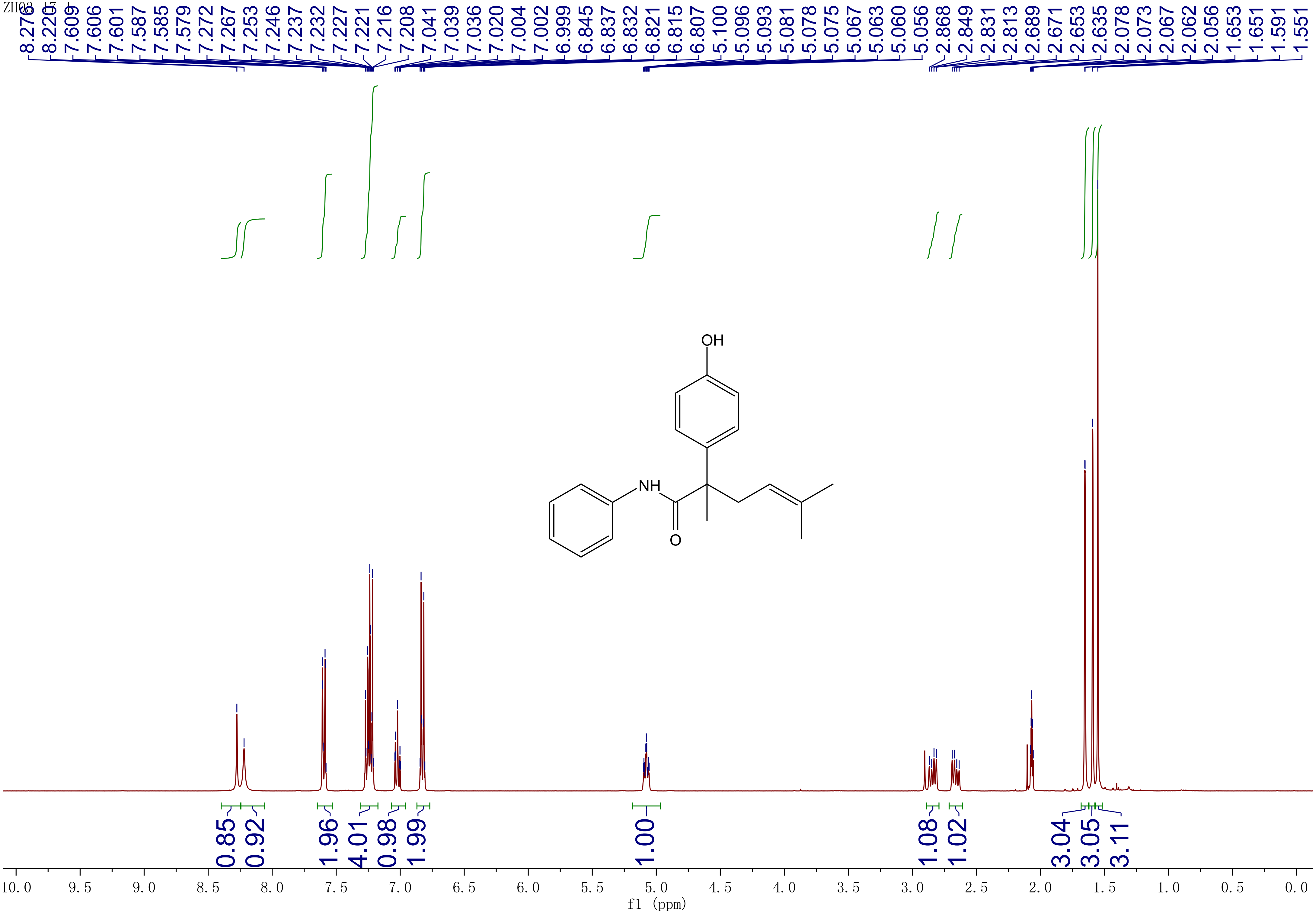

8.276
8.220
7.609
7.606
7.601
7.587
7.585
7.579
7.272
7.267
7.253
7.246
7.237
7.232
7.227
7.221
7.216
7.208
7.041
7.039
7.036
7.020
7.004
7.002
6.999
6.845
6.837
6.832
6.821
6.815
6.807
5.100
5.096
5.093
5.081
5.078
5.075
5.067
5.063
5.060
5.056
2.868
2.849
2.831
2.813
2.689
2.671
2.653
2.635
2.078
2.073
2.067
2.062
2.056
1.653
1.651
1.591
1.551
OH
NH
O
0.85
0.92
1.96
4.01
0.98
1.99
1.00
1.08
1.02
3.04
3.05
3.11
10.0
9.5
9.0
8.5
8.0
7.5
7.0
6.5
6.0
5.5
5.0
4.5
4.0
3.5
3.0
2.5
2.0
1.5
1.0
0.5
0.0
f1 (ppm)

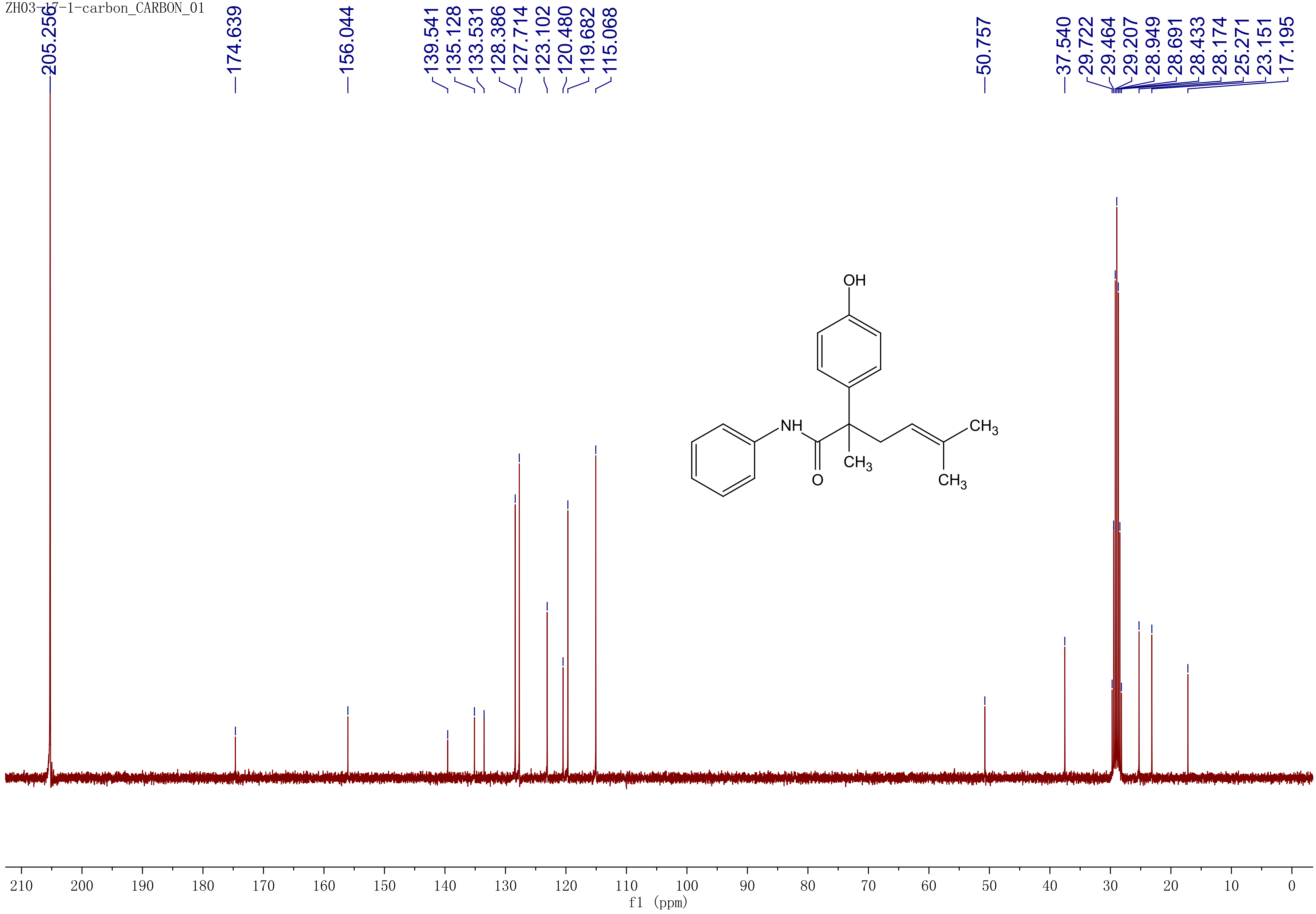

ZH03-17-1-carbon_CARBON_01
205.256
174.639
156.044
139.541
135.128
133.531
128.386
127.714
123.102
120.480
119.682
115.068
50.757
37.540
29.722
29.464
29.207
28.949
28.691
28.433
28.174
25.271
23.151
17.195
OH
NH
O
CH3
CH3
CH3
210 200 190 180 170 160 150 140 130 120 110 100 90 80 70 60 50 40 30 20 10 0
f1 (ppm)

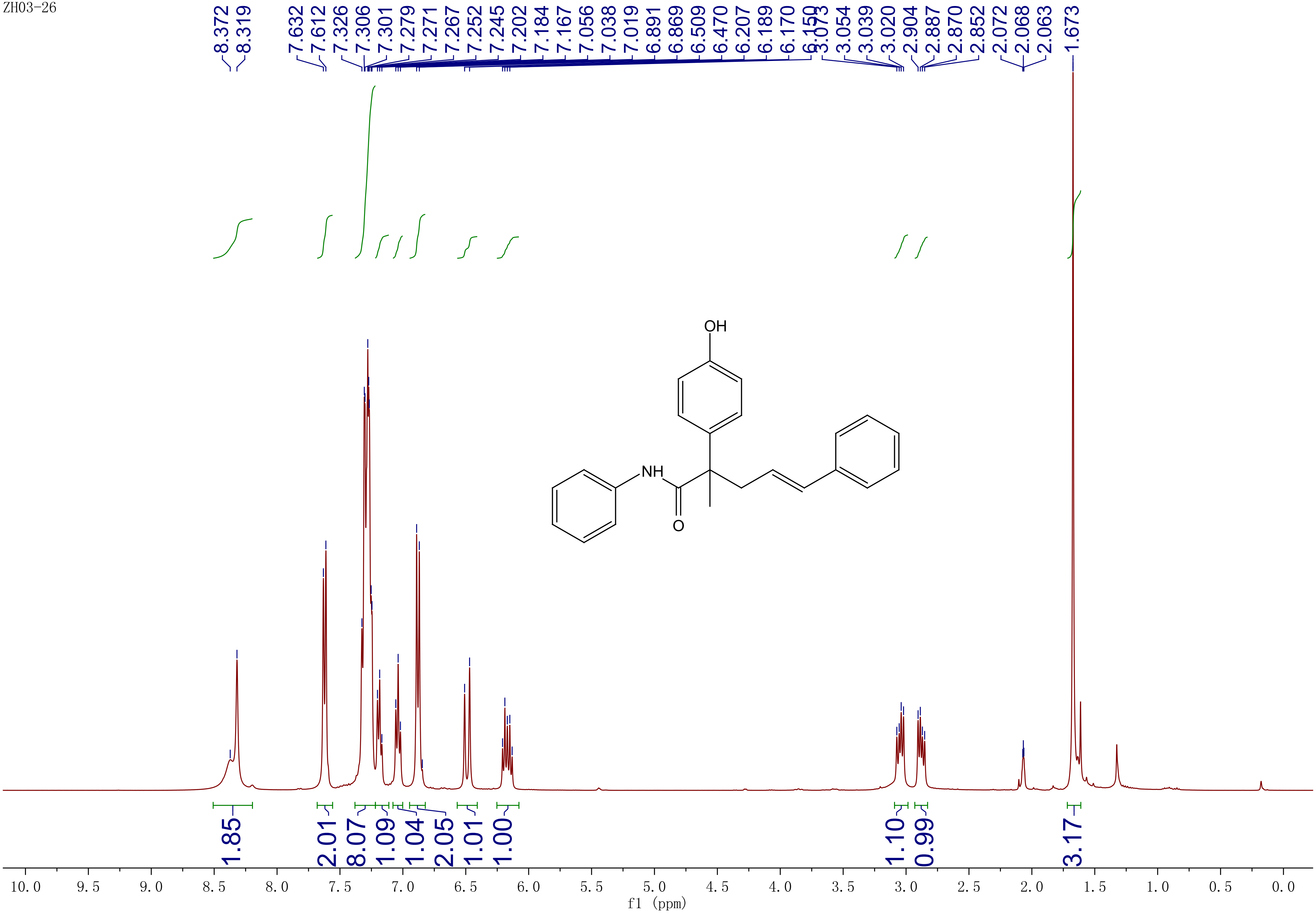
ZH03-26
8.372
8.319
7.632
7.612
7.326
7.306
7.301
7.279
7.271
7.267
7.252
7.245
7.202
7.184
7.167
7.056
7.038
7.019
6.891
6.869
6.509
6.470
6.207
6.189
6.170
6.15
3.073
3.054
3.039
3.020
2.904
2.887
2.870
2.852
2.072
2.068
2.063
1.673
OH
NH
O
1.85
2.01
8.07
1.09
1.04
2.05
1.01
1.00
1.10
0.99
3.17
10.0
9.5
9.0
8.5
8.0
7.5
7.0
6.5
6.0
5.5
5.0
4.5
4.0
3.5
3.0
2.5
2.0
1.5
1.0
0.5
0.0
f1 (ppm)

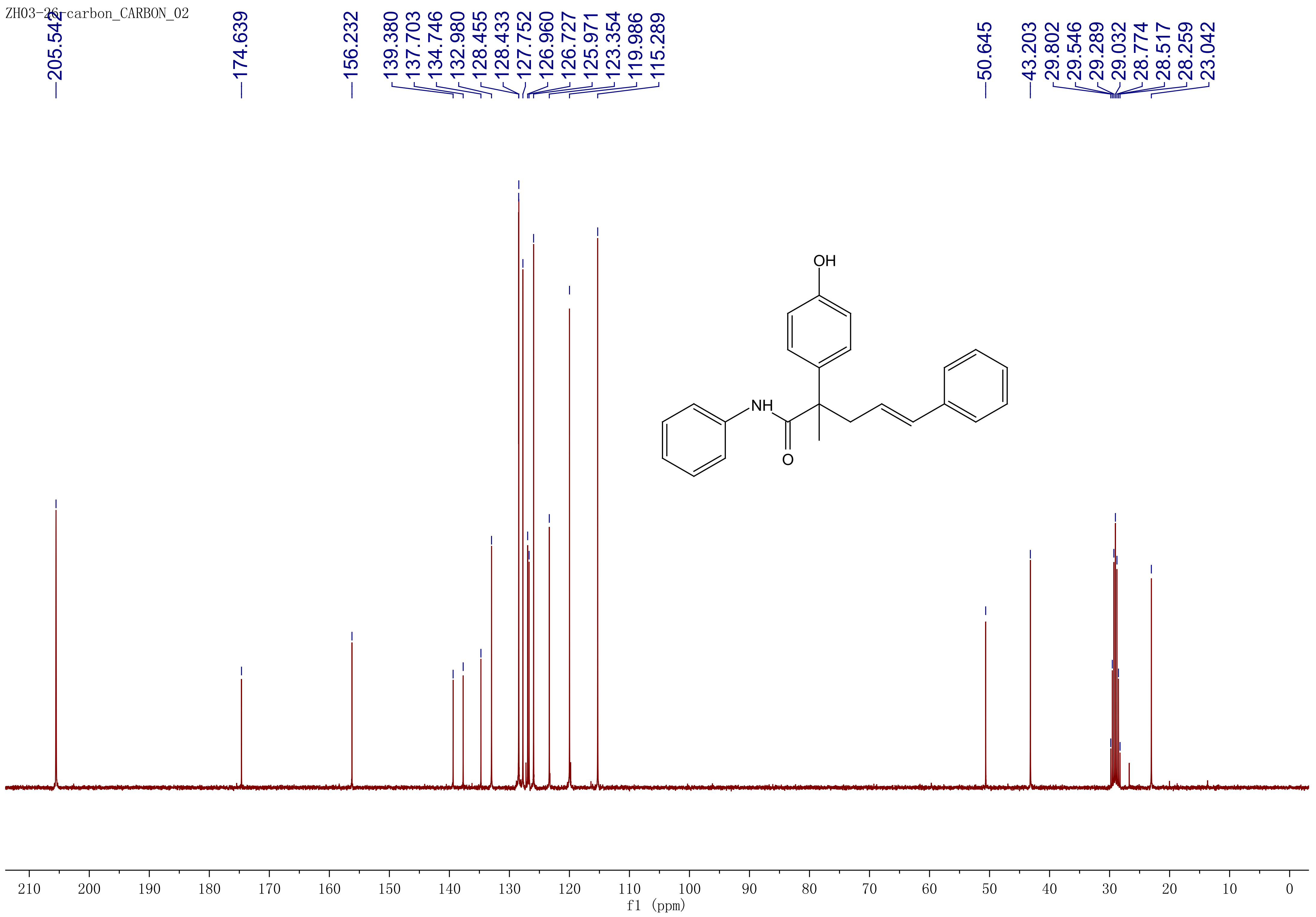
ZH03-26-carbon_CARBON_02
205.542
174.639
156.232
139.380
137.703
134.746
132.980
128.455
128.433
127.752
126.960
126.727
125.971
123.354
119.986
115.289
50.645
43.203
29.802
29.546
29.289
29.032
28.774
28.517
28.259
23.042
OH
NH
O
210 200 190 180 170 160 150 140 130 120 110 100 90 80 70 60 50 40 30 20 10 0
f1 (ppm)

proton
ZH03-17-2

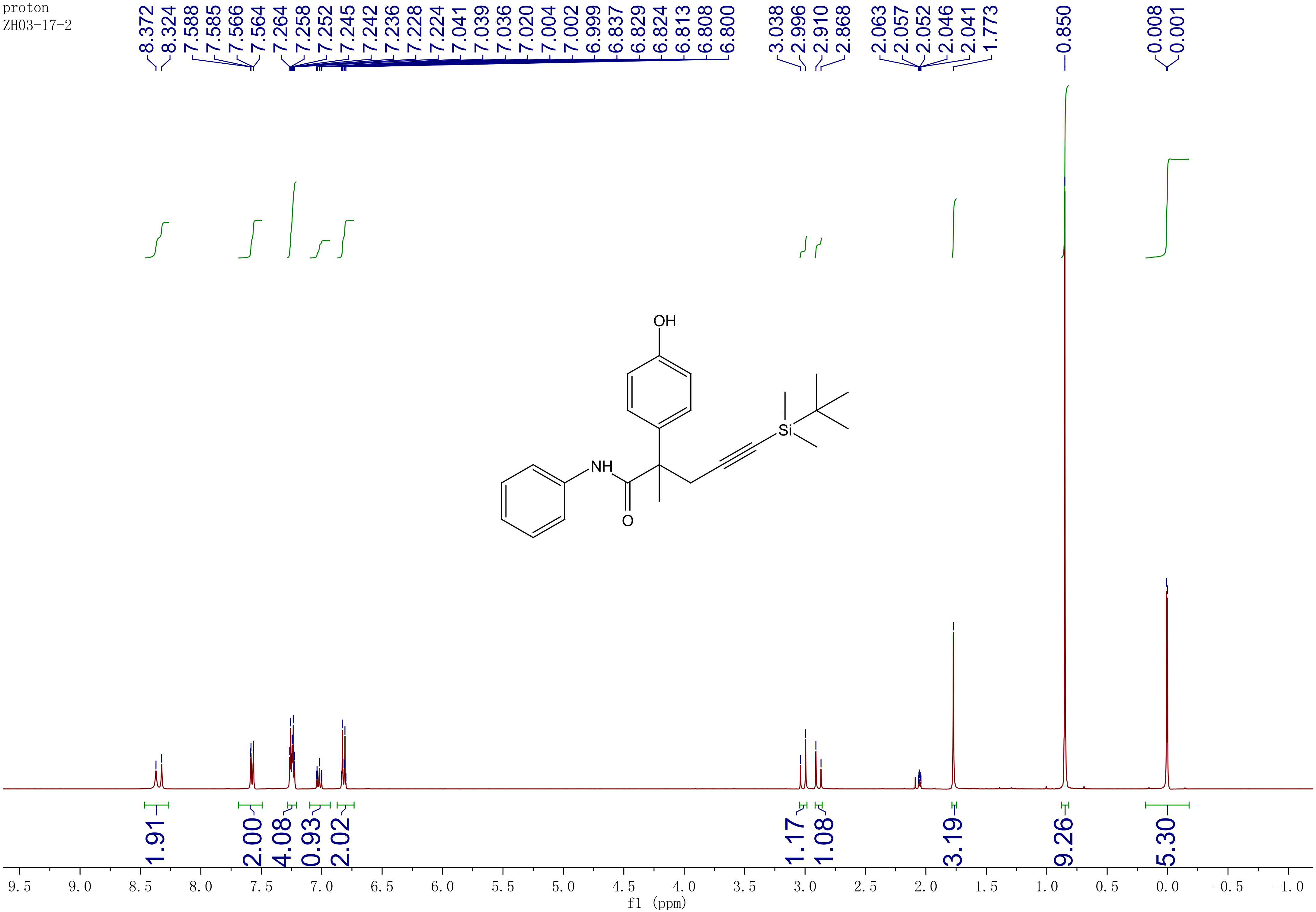

ZH03-17-2-carbon_CARBON_01

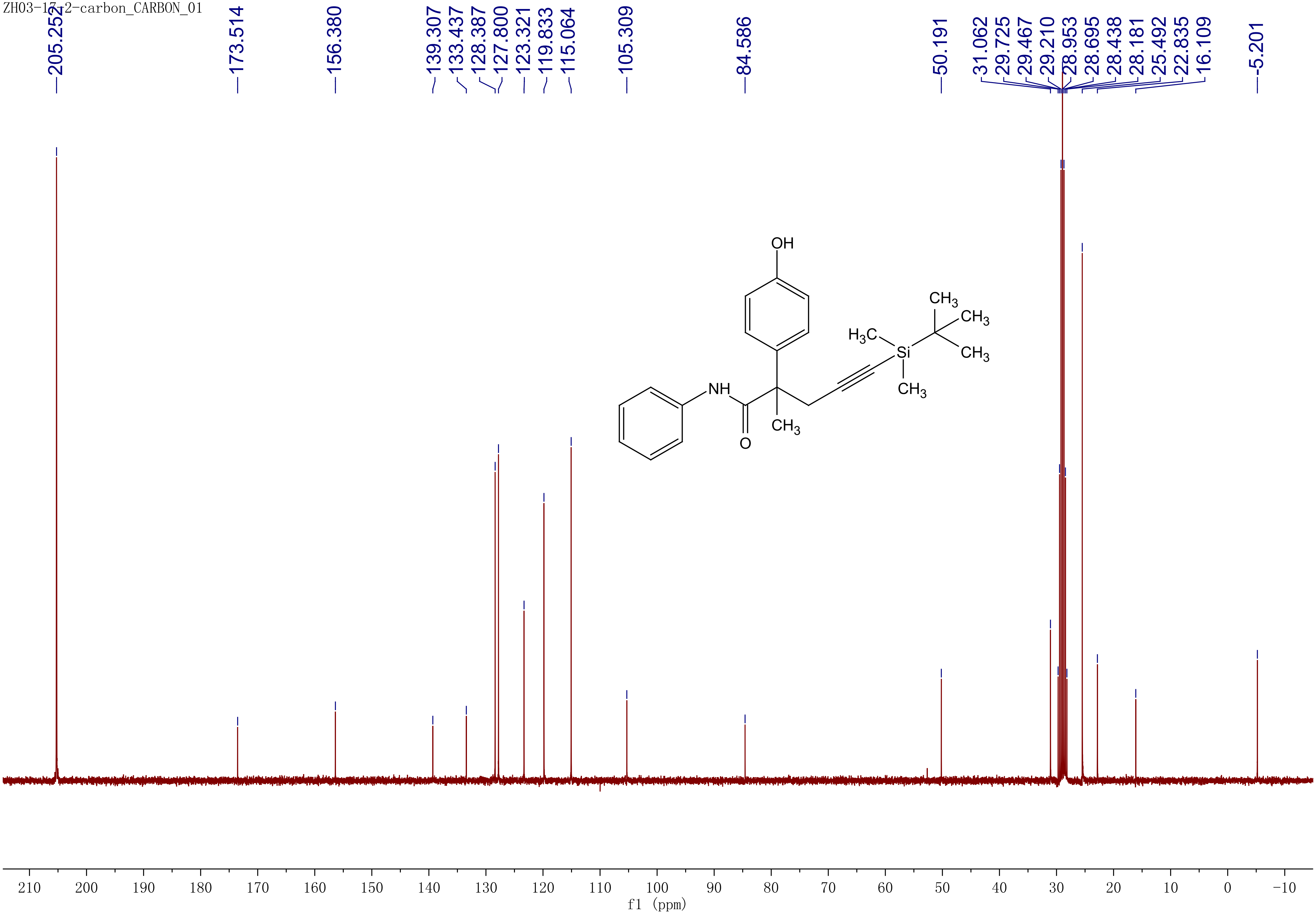

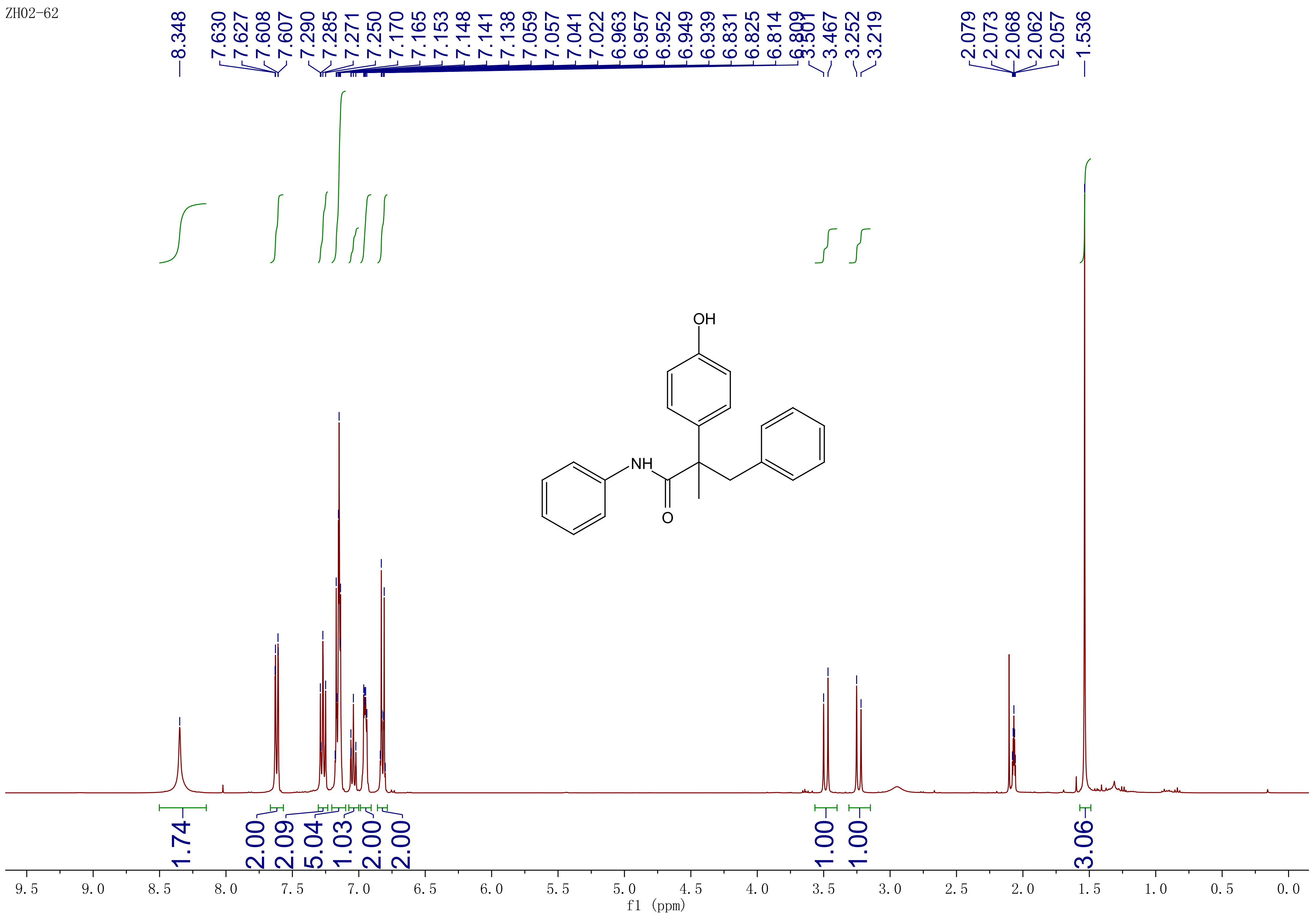
ZH02-62
8.348
7.630
7.627
7.608
7.607
7.290
7.285
7.271
7.250
7.170
7.165
7.153
7.148
7.141
7.138
7.059
7.057
7.041
7.022
6.963
6.957
6.952
6.949
6.939
6.831
6.825
6.814
6.809
3.501
3.467
3.252
3.219
2.079
2.073
2.068
2.062
2.057
1.536
OH
NH
O
1.74
2.00
2.09
5.04
1.03
2.00
2.00
1.00
1.00
3.06
9.5
9.0
8.5
8.0
7.5
7.0
6.5
6.0
5.5
5.0
4.5
4.0
3.5
3.0
2.5
2.0
1.5
1.0
0.5
0.0
f1 (ppm)

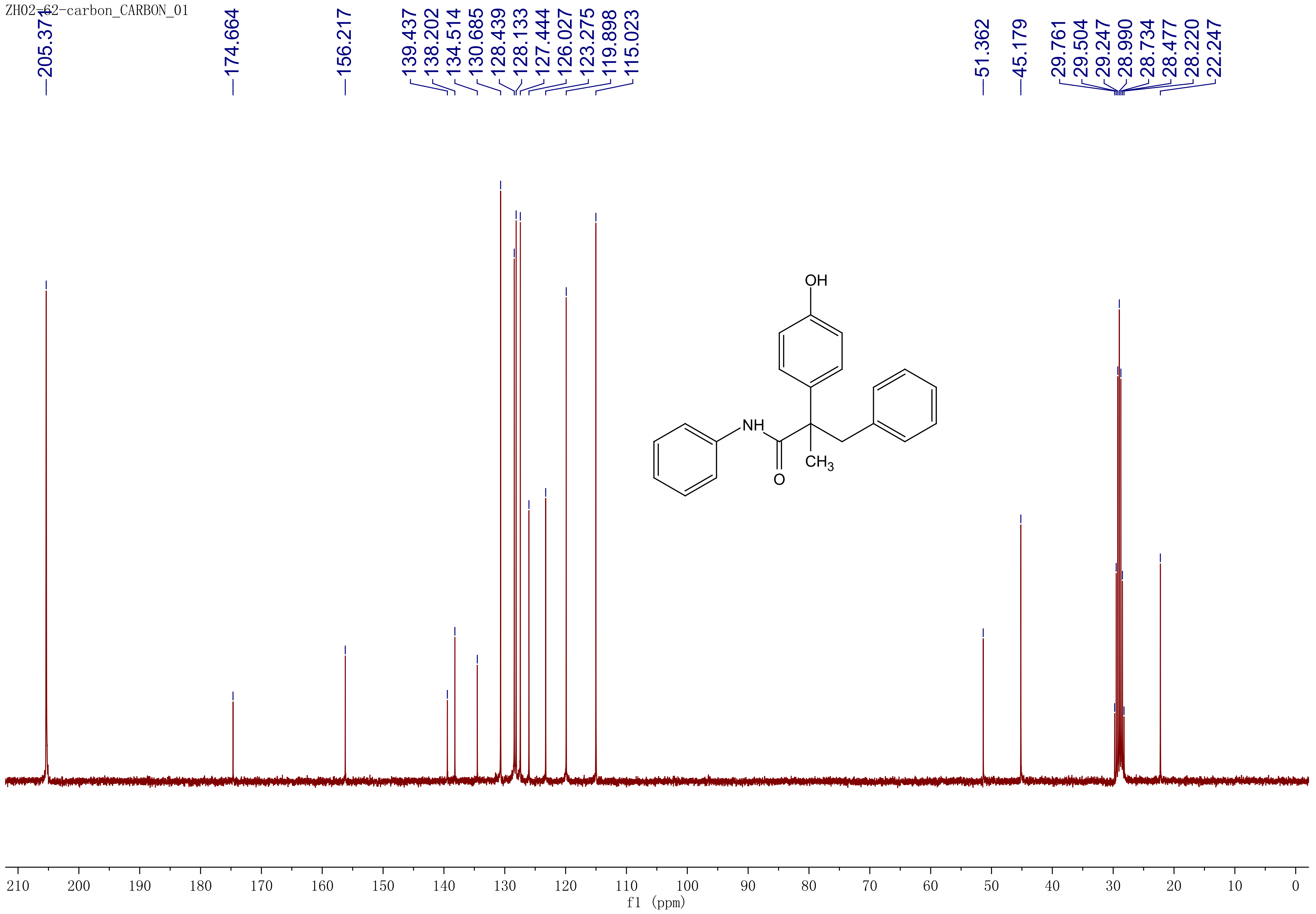
ZH02-62-carbon_CARBON_01
205.37
174.664
156.217
139.437
138.202
134.514
130.685
128.439
128.133
127.444
126.027
123.275
119.898
115.023
51.362
45.179
29.761
29.504
29.247
28.990
28.734
28.477
28.220
22.247
OH
NH
O
CH3
210 200 190 180 170 160 150 140 130 120 110 100 90 80 70 60 50 40 30 20 10 0
f1 (ppm)

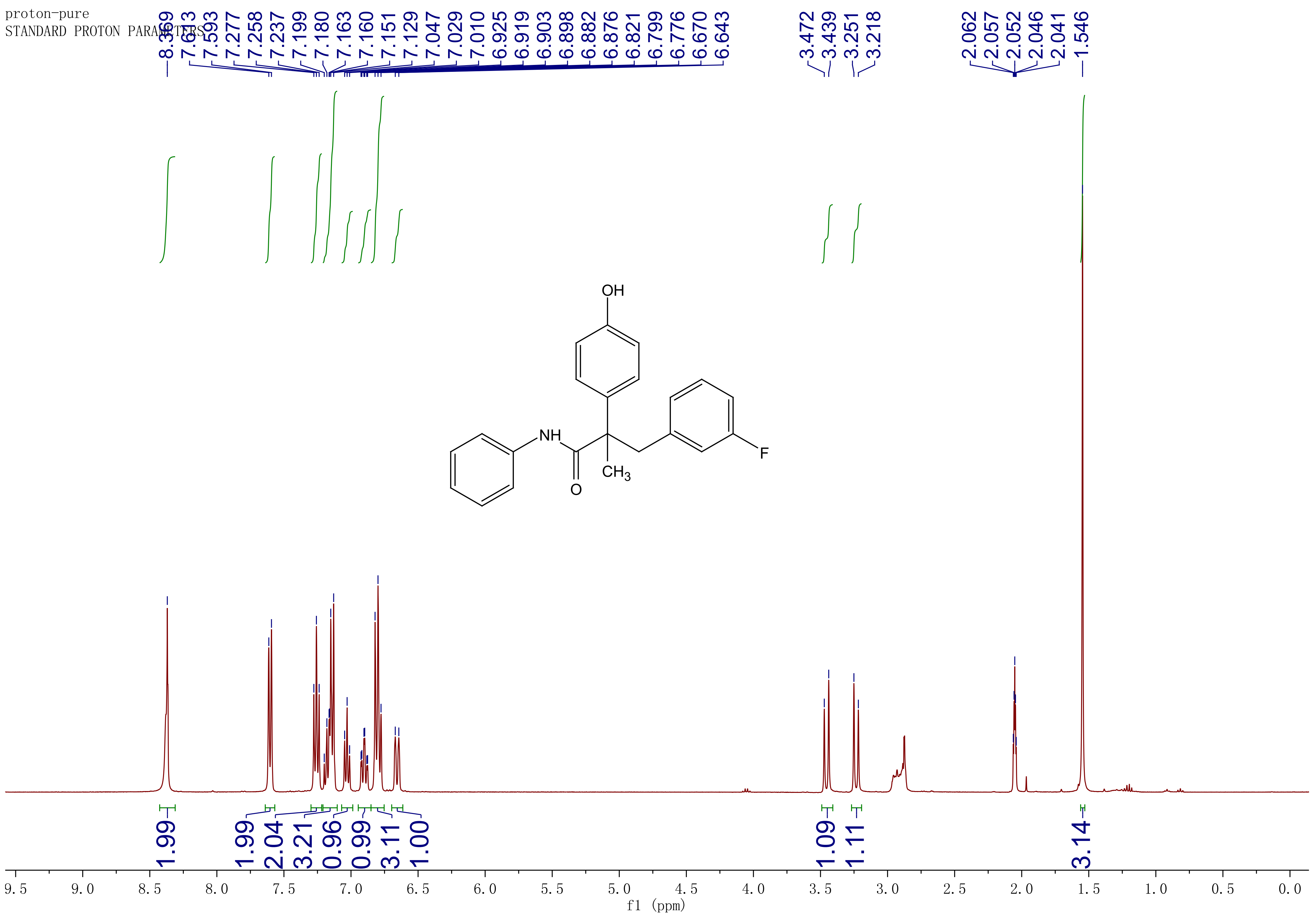

proton-pure
STANDARD PROTON PARAMETERS
8.369
7.613
7.593
7.277
7.258
7.237
7.199
7.180
7.163
7.160
7.151
7.129
7.047
7.029
7.010
6.925
6.919
6.903
6.898
6.882
6.876
6.821
6.799
6.776
6.670
6.643
3.472
3.439
3.251
3.218
2.062
2.057
2.052
2.046
2.041
1.546
OH
NH
O
CH3
F
1.99
1.99
2.04
3.21
0.96
0.99
3.11
1.00
1.09
1.11
3.14
9.5 9.0 8.5 8.0 7.5 7.0 6.5 6.0 5.5 5.0 4.5 4.0 3.5 3.0 2.5 2.0 1.5 1.0 0.5 0.0
f1 (ppm)

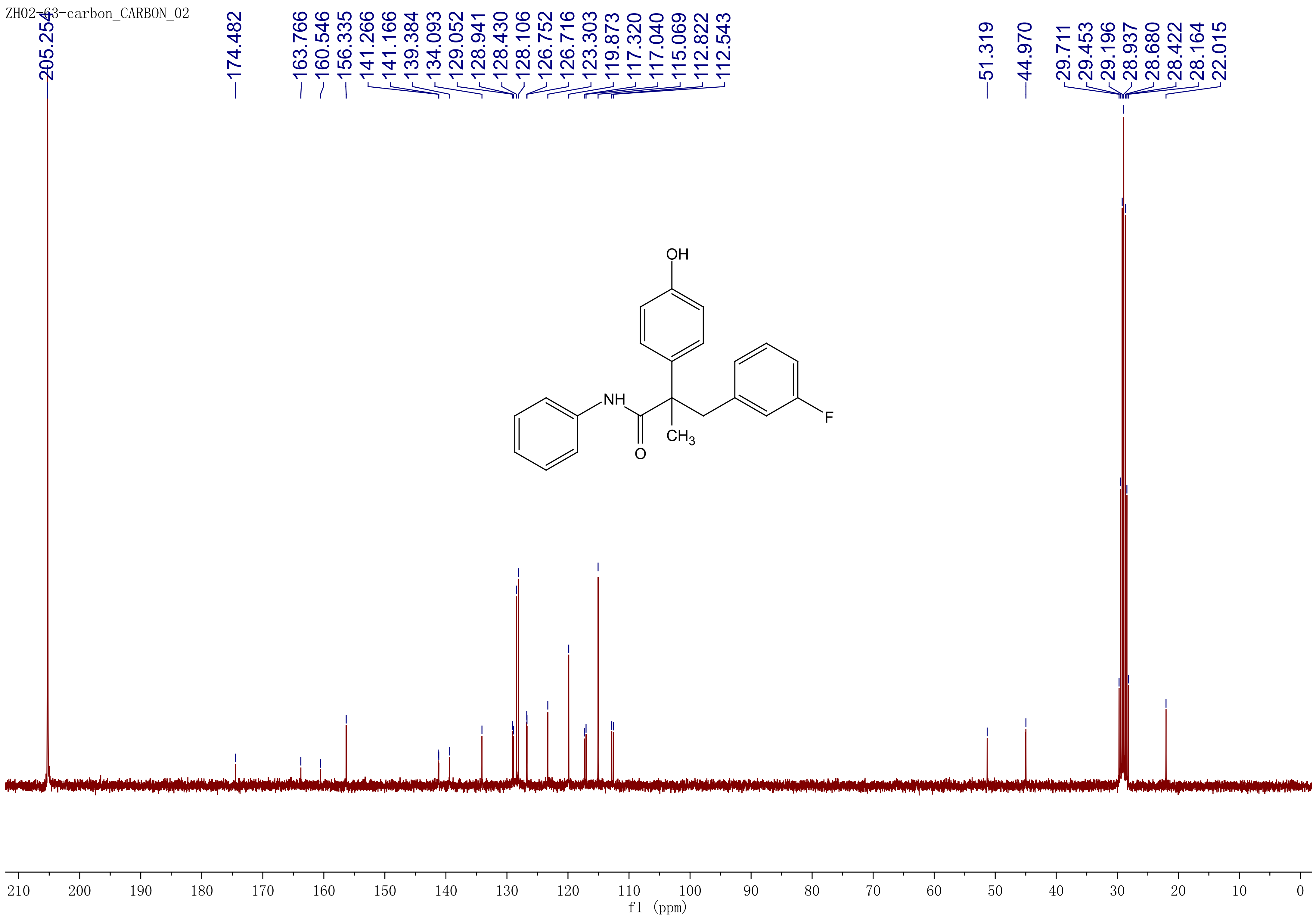
ZH02-63-carbon_CARBON_02
205.254
174.482
163.766
160.546
156.335
141.266
141.166
139.384
134.093
129.052
128.941
128.430
128.106
126.752
126.716
123.303
119.873
117.320
117.040
115.069
112.822
112.543
51.319
44.970
29.711
29.453
29.196
28.937
28.680
28.422
28.164
22.015
OH
NH
O
CH3
F
210 200 190 180 170 160 150 140 130 120 110 100 90 80 70 60 50 40 30 20 10 0
f1 (ppm)

ZH03-13-1

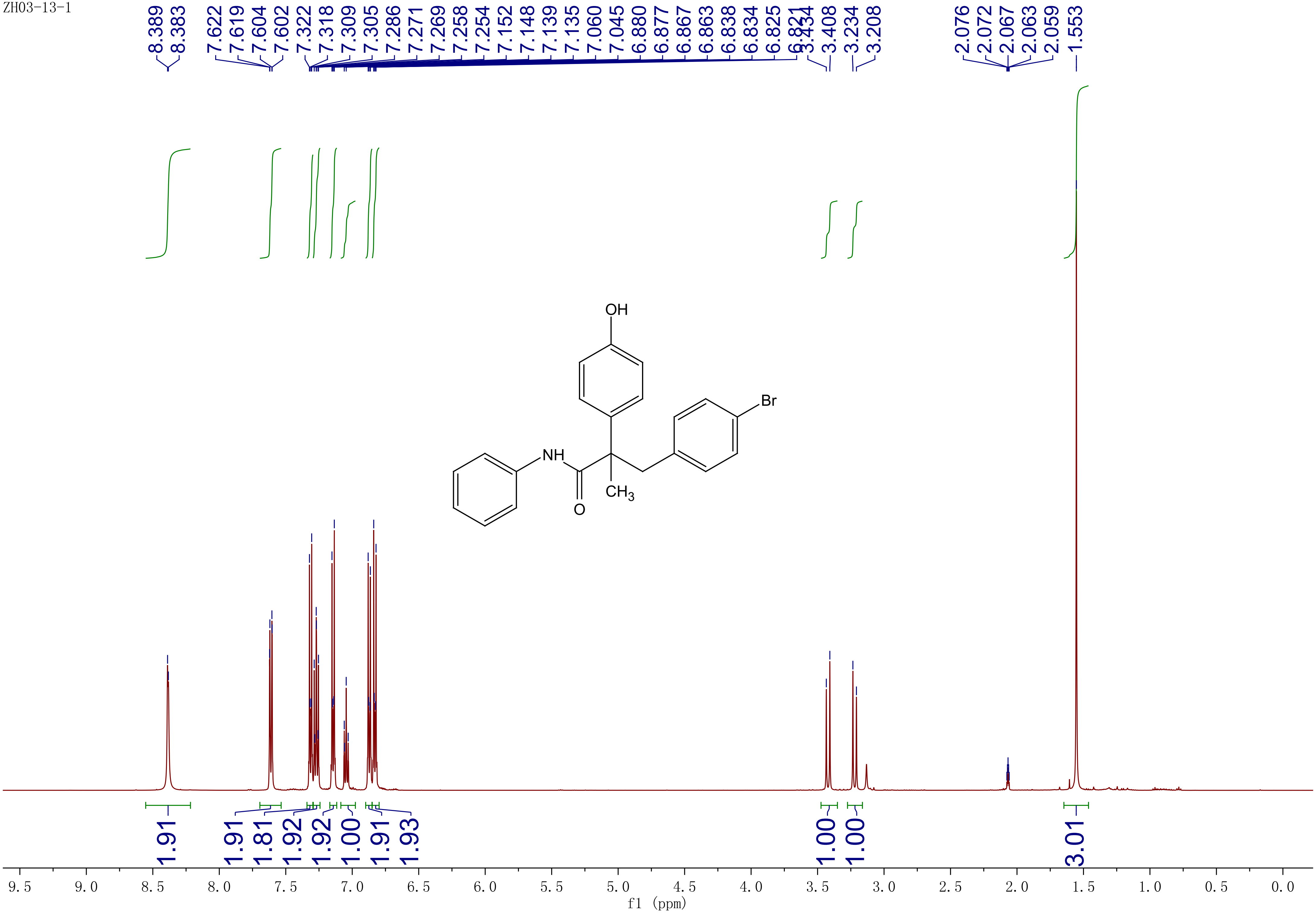
8.389
8.383
7.622
7.619
7.604
7.602
7.322
7.318
7.309
7.305
7.286
7.271
7.269
7.258
7.254
7.152
7.148
7.139
7.135
7.060
7.045
6.880
6.877
6.867
6.863
6.838
6.834
6.825
6.821
3.434
3.408
3.234
3.208
2.076
2.072
2.067
2.063
2.059
1.553
OH
Br
NH
O
$CH_3$
1.91
1.91
1.81
1.92
1.92
1.00
1.91
1.93
1.00
1.00
3.01
9.5
9.0
8.5
8.0
7.5
7.0
6.5
6.0
5.5
5.0
4.5
4.0
3.5
3.0
2.5
2.0
1.5
1.0
0.5
0.0
f1 (ppm)

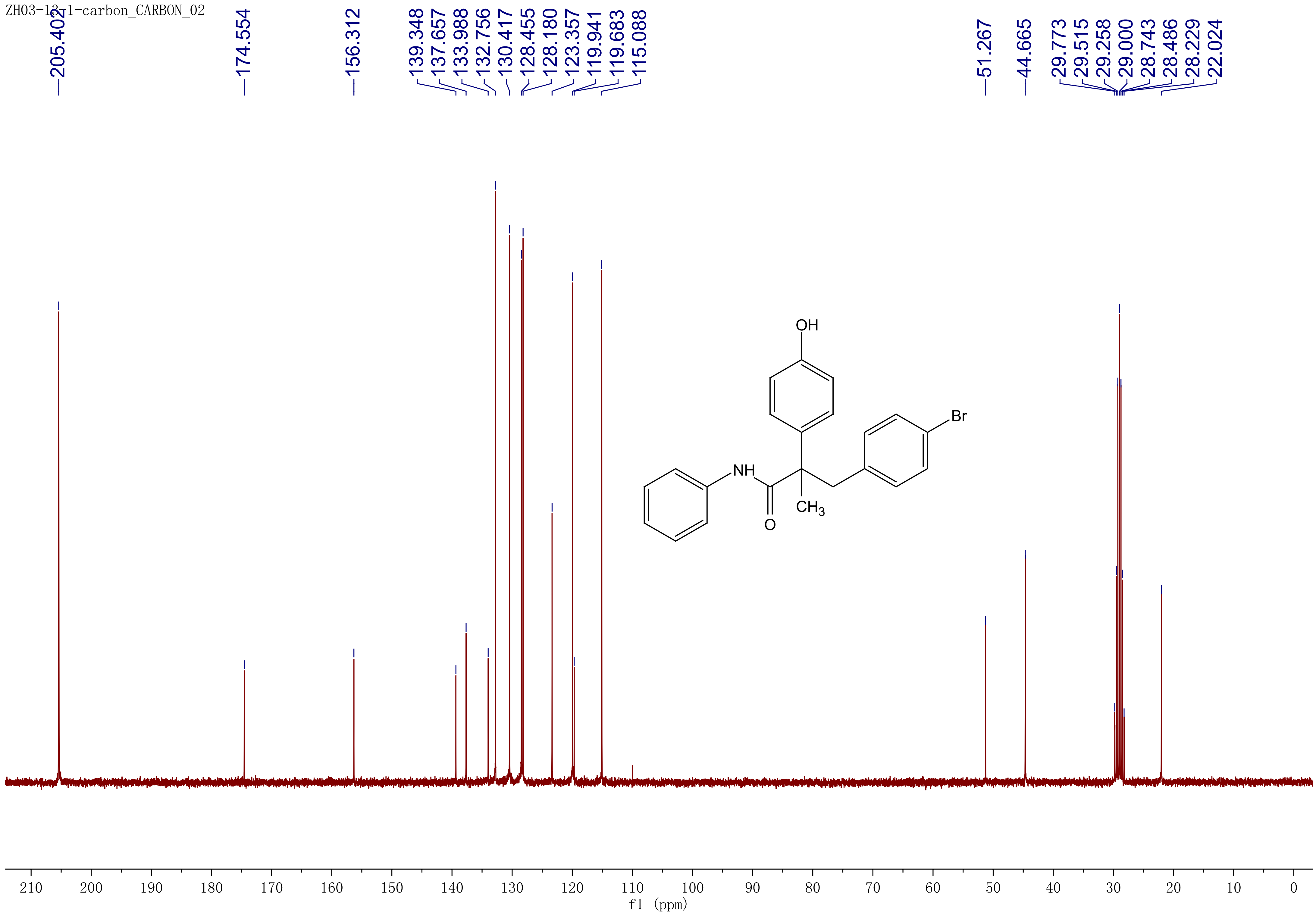
ZH03-13-1-carbon_CARBON_02
205.402
174.554
156.312
139.348
137.657
133.988
132.756
130.417
128.455
128.180
123.357
119.941
119.683
115.088
51.267
44.665
29.773
29.515
29.258
29.000
28.743
28.486
28.229
22.024
OH
Br
NH
O
CH3
210
200
190
180
170
160
150
140
130
120
110
100
90
80
70
60
50
40
30
20
10
0
f1 (ppm)

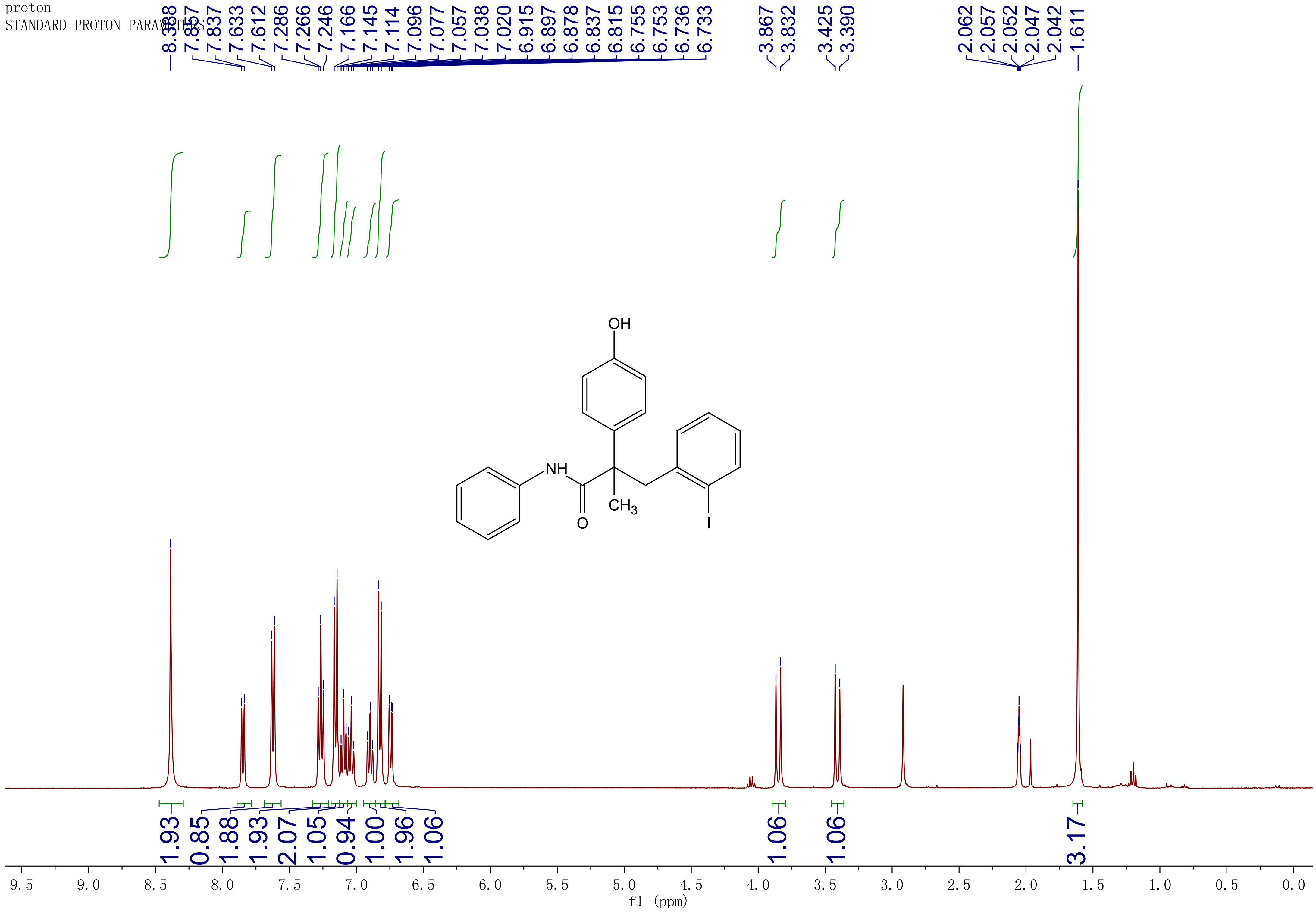
proton
STANDARD PROTON PARAMETERS
8.388
7.857
7.837
7.633
7.612
7.286
7.266
7.246
7.166
7.145
7.114
7.096
7.077
7.057
7.038
7.020
6.915
6.897
6.878
6.837
6.815
6.755
6.753
6.736
6.733
3.867
3.832
3.425
3.390
2.062
2.057
2.052
2.047
2.042
1.611
OH
NH
O
CH3
I
1.93
0.85
1.88
1.93
2.07
1.05
0.94
1.00
1.96
1.06
1.06
1.06
3.17
9.5
9.0
8.5
8.0
7.5
7.0
6.5
6.0
5.5
5.0
4.5
4.0
3.5
3.0
2.5
2.0
1.5
1.0
0.5
0.0
f1 (ppm)

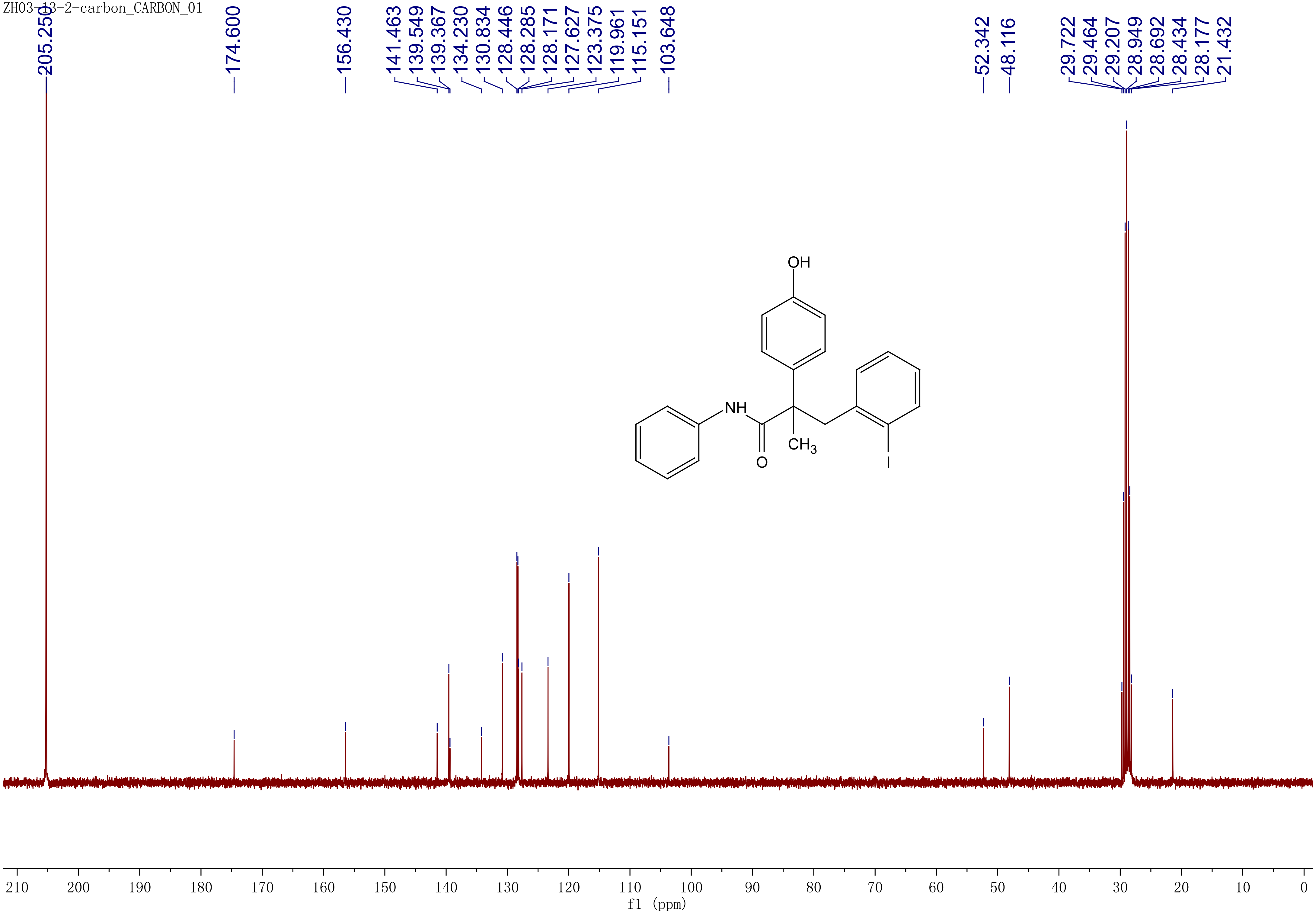
ZH03-13-2-carbon_CARBON_01
205.250
174.600
156.430
141.463
139.549
139.367
134.230
130.834
128.446
128.285
128.171
127.627
123.375
119.961
115.151
103.648
52.342
48.116
29.722
29.464
29.207
28.949
28.692
28.434
28.177
21.432
OH
NH
O
CH3
I
210 200 190 180 170 160 150 140 130 120 110 100 90 80 70 60 50 40 30 20 10 0
f1 (ppm)

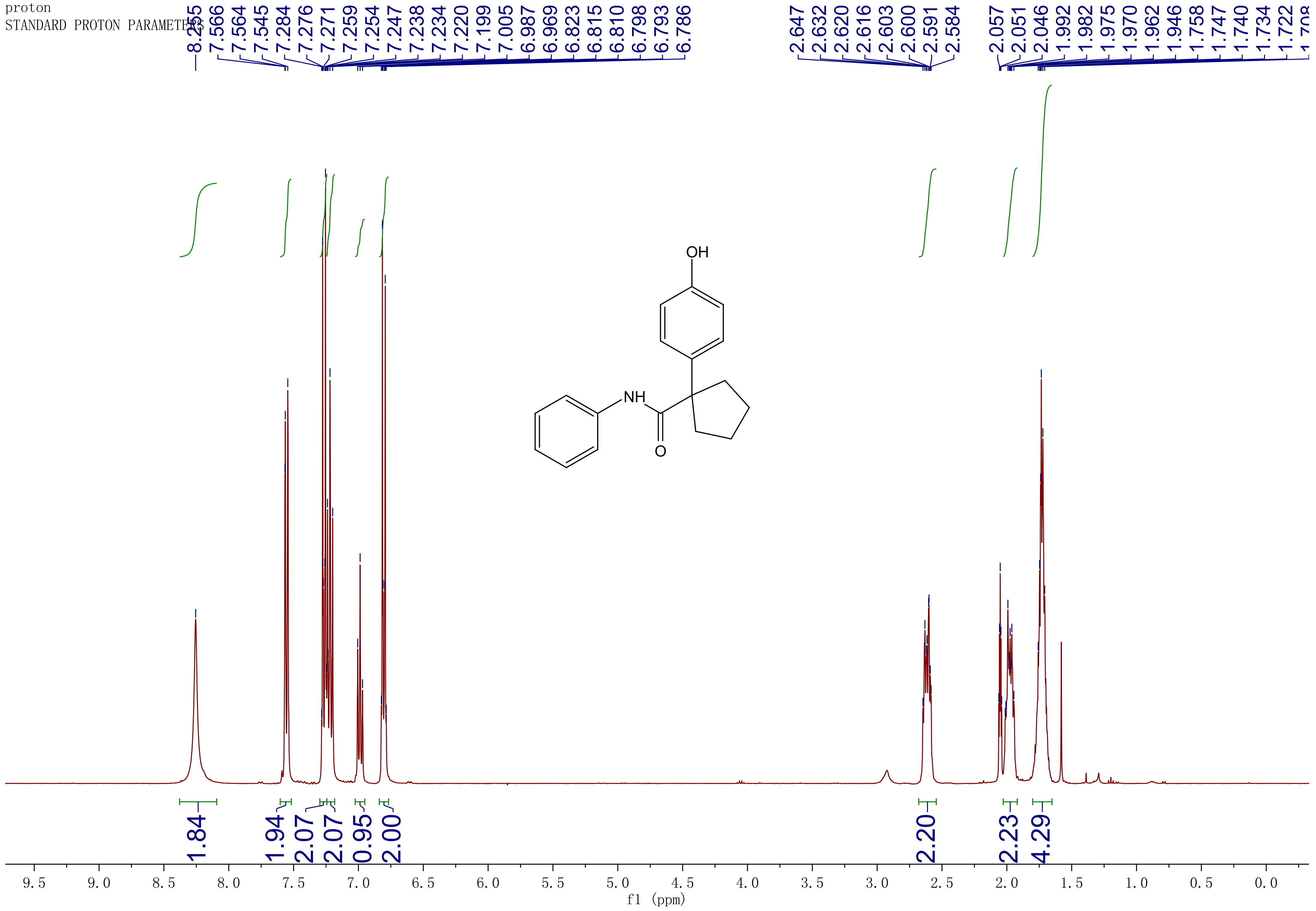
proton
STANDARD PROTON PARAMETERS
8.255
7.566
7.564
7.545
7.284
7.276
7.271
7.259
7.254
7.247
7.238
7.234
7.220
7.199
7.005
6.987
6.969
6.823
6.815
6.810
6.798
6.793
6.786
2.647
2.632
2.620
2.616
2.603
2.600
2.591
2.584
2.057
2.051
2.046
1.992
1.982
1.975
1.970
1.962
1.946
1.758
1.747
1.740
1.734
1.722
OH
NH
O
1.84
1.94
2.07
2.07
0.95
2.00
2.20
2.23
4.29
9.5
9.0
8.5
8.0
7.5
7.0
6.5
6.0
5.5
5.0
4.5
4.0
3.5
3.0
2.5
2.0
1.5
1.0
0.5
0.0
f1 (ppm)

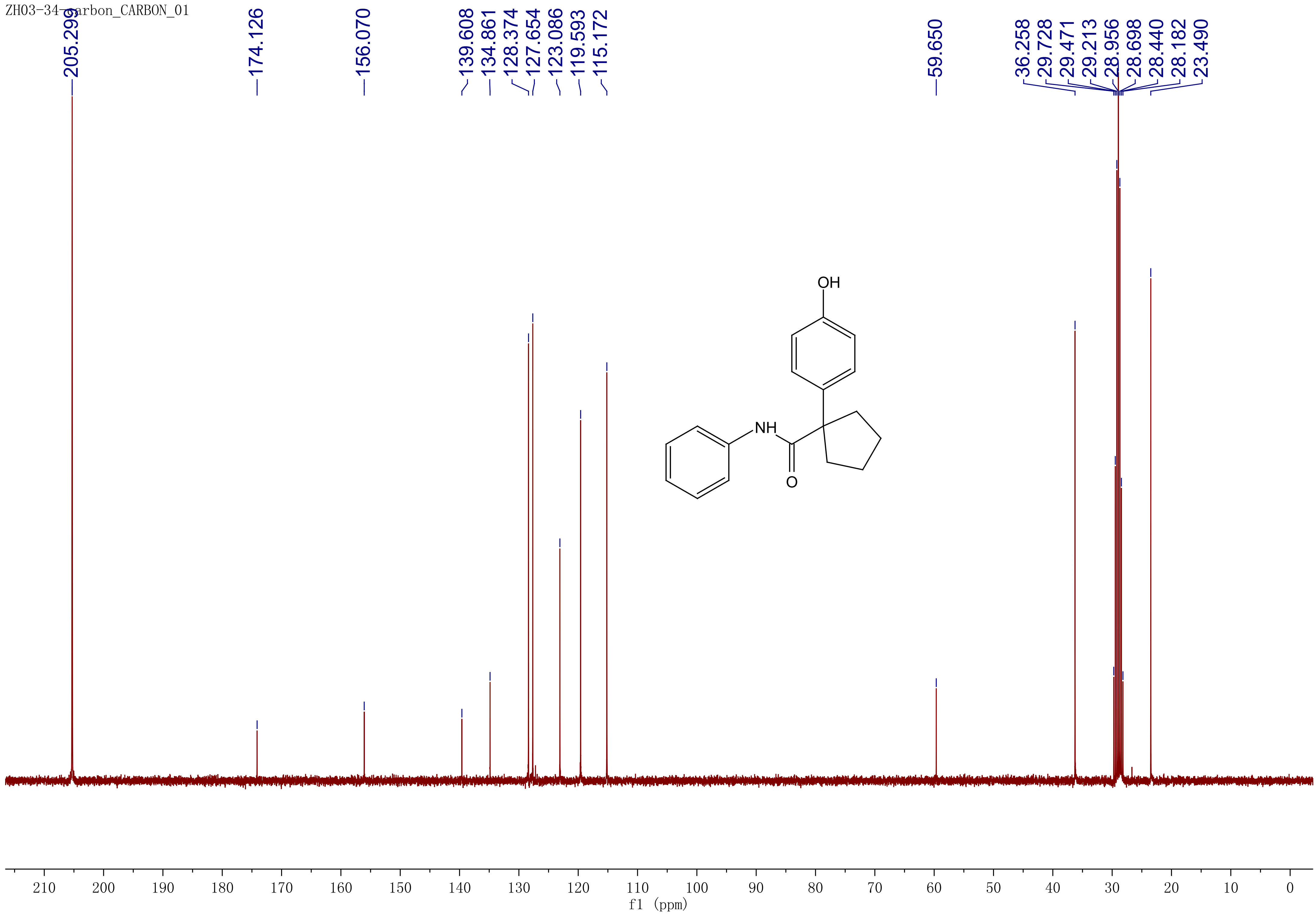
ZH03-34-carbon_CARBON_01
205.299
174.126
156.070
139.608
134.861
128.374
127.654
123.086
119.593
115.172
59.650
36.258
29.728
29.471
29.213
28.956
28.698
28.440
28.182
23.490
OH
NH
O
210 200 190 180 170 160 150 140 130 120 110 100 90 80 70 60 50 40 30 20 10 0
f1 (ppm)

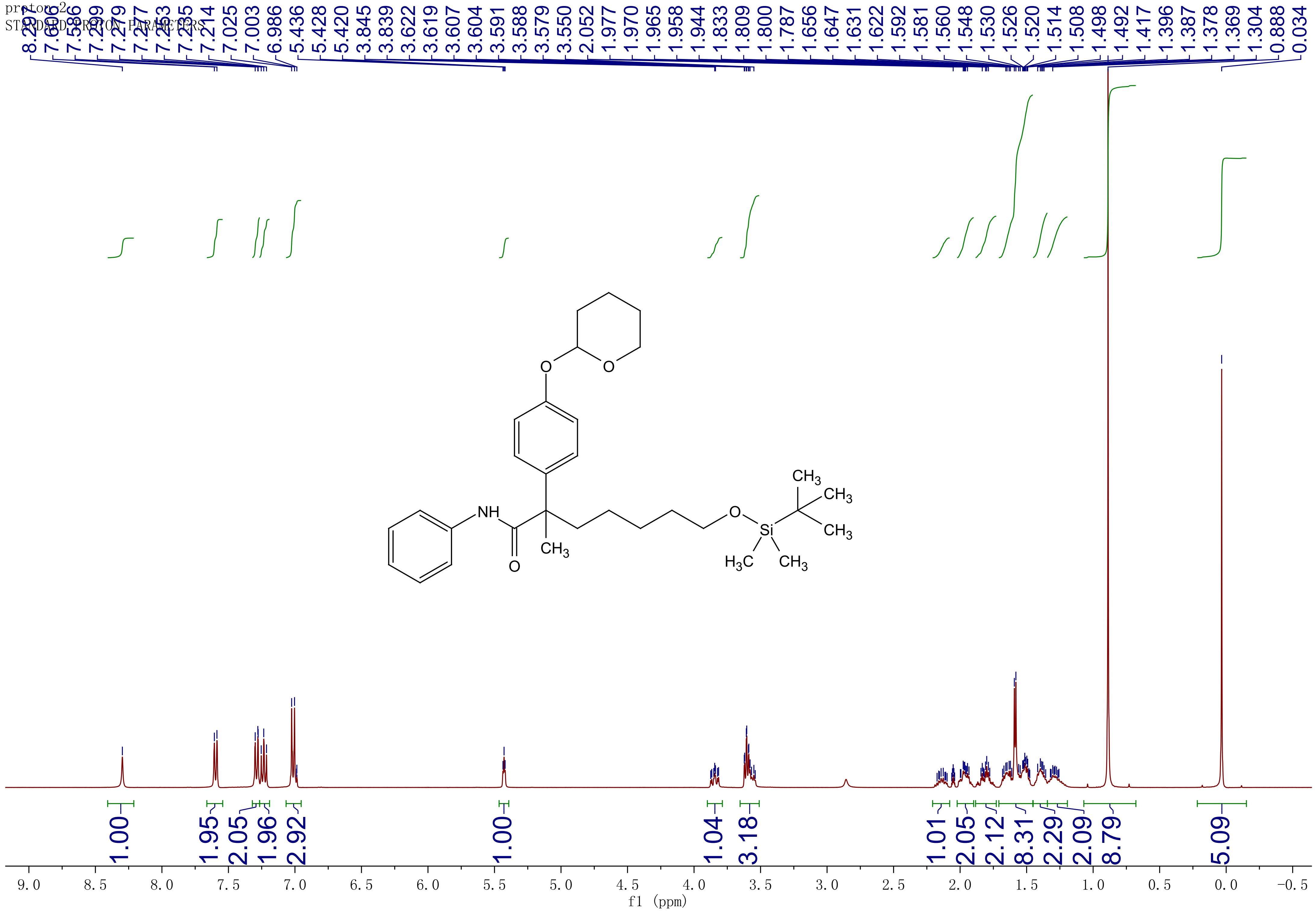

proton-2
STANDARD PROTON PARAMETERS
8.297
7.606
7.586
7.299
7.279
7.277
7.253
7.235
7.214
7.025
7.003
6.986
5.436
5.428
5.420
3.845
3.839
3.622
3.619
3.607
3.604
3.591
3.588
3.579
3.550
2.052
1.977
1.970
1.965
1.958
1.944
1.833
1.809
1.800
1.787
1.656
1.647
1.631
1.622
1.592
1.581
1.560
1.548
1.530
1.526
1.520
1.514
1.508
1.498
1.492
1.417
1.396
1.387
1.378
1.369
1.304
0.888
0.034
NH
O
O
O
CH3
O
Si
CH3
CH3
CH3
H3C
CH3
1.00
1.95
2.05
1.96
2.92
1.00
1.04
3.18
1.01
2.05
2.12
8.31
2.29
2.09
8.79
5.09
9.0
8.5
8.0
7.5
7.0
6.5
6.0
5.5
5.0
4.5
4.0
3.5
3.0
2.5
2.0
1.5
1.0
0.5
0.0
-0.5
f1 (ppm)

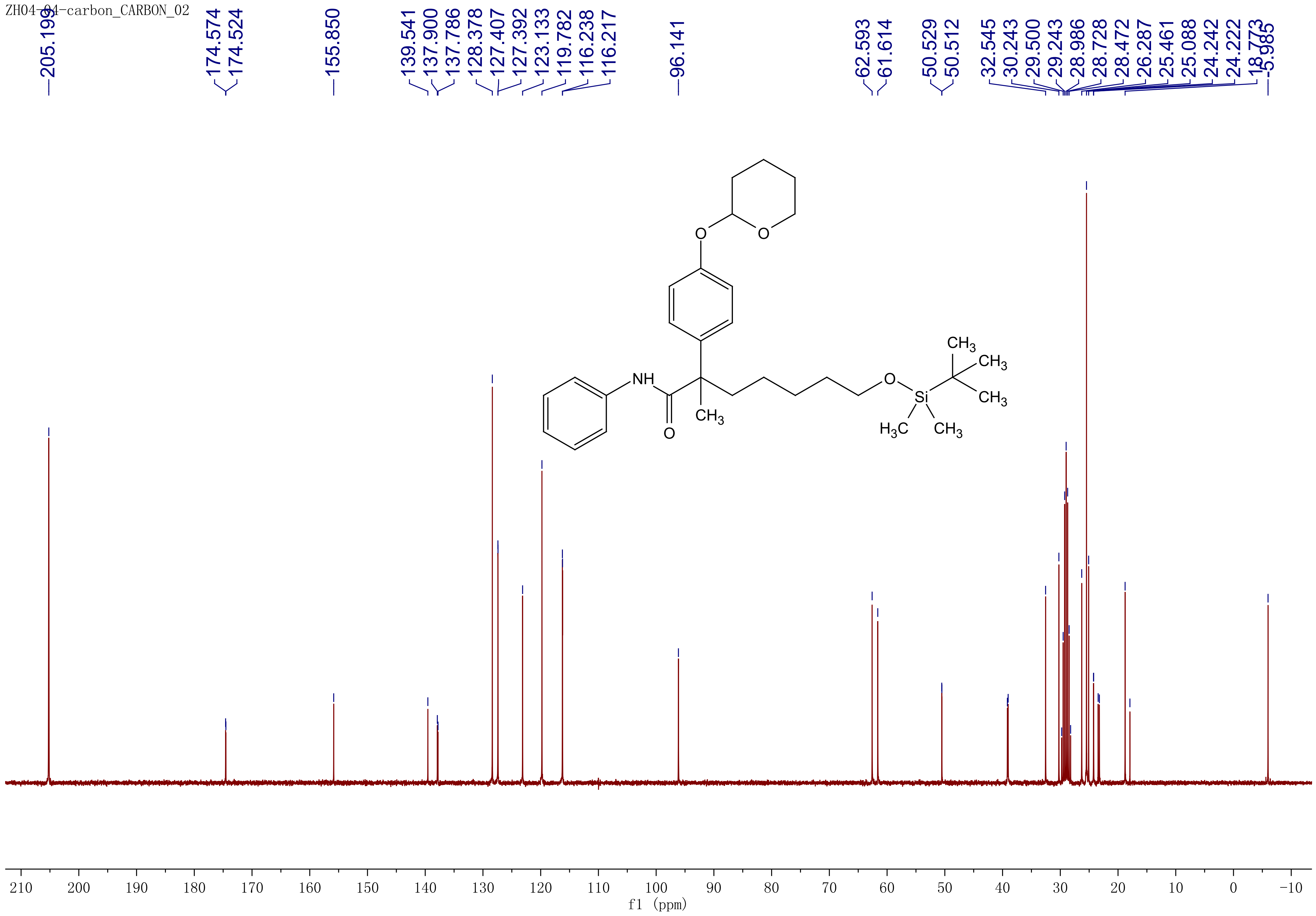

ZH04-04-carbon_CARBON_02
205.199
174.574
174.524
155.850
139.541
137.900
137.786
128.378
127.407
127.392
123.133
119.782
116.238
116.217
96.141
62.593
61.614
50.529
50.512
32.545
30.243
29.500
29.243
28.986
28.728
28.472
26.287
25.461
25.088
24.242
24.222
18.773
-5.985
O
O
NH
O
CH3
O
Si
CH3
CH3
CH3
H3C
CH3
210
200
190
180
170
160
150
140
130
120
110
100
90
80
70
60
50
40
30
20
10
0
-10
f1 (ppm)

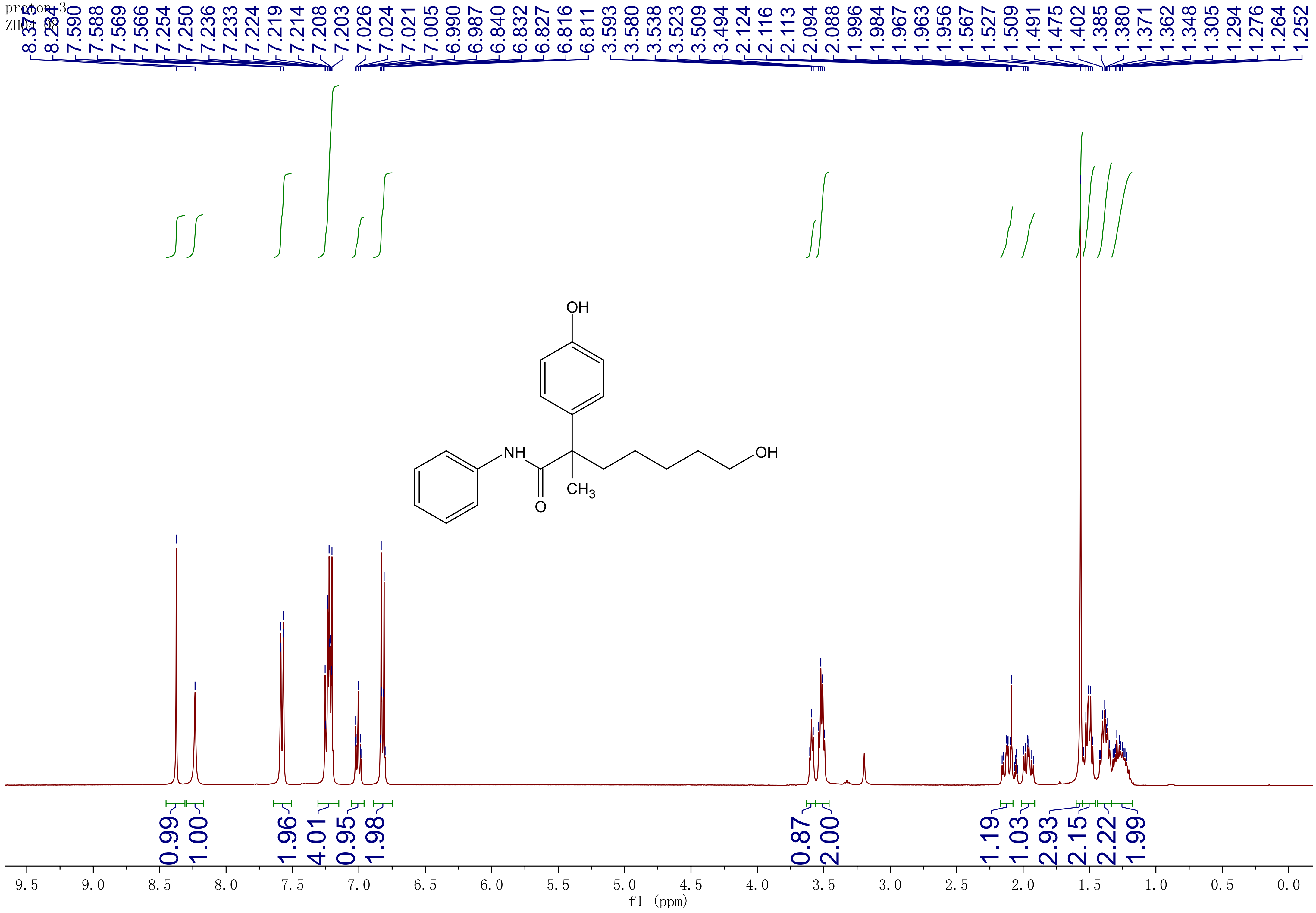
proton-3
ZH04-08
8.375
8.234
7.590
7.588
7.569
7.566
7.254
7.250
7.236
7.233
7.224
7.219
7.214
7.208
7.203
7.026
7.024
7.021
7.005
6.990
6.987
6.840
6.832
6.827
6.816
6.811
3.593
3.580
3.538
3.523
3.509
3.494
2.124
2.116
2.113
2.094
2.088
1.996
1.984
1.967
1.963
1.956
1.567
1.527
1.509
1.491
1.475
1.402
1.385
1.380
1.371
1.362
1.348
1.305
1.294
1.276
1.264
1.252
OH
NH
OH
CH3
O
0.99
1.00
1.96
4.01
0.95
1.98
0.87
2.00
1.19
1.03
2.93
2.15
2.22
1.99
9.5
9.0
8.5
8.0
7.5
7.0
6.5
6.0
5.5
5.0
4.5
4.0
3.5
3.0
2.5
2.0
1.5
1.0
0.5
0.0
f1 (ppm)

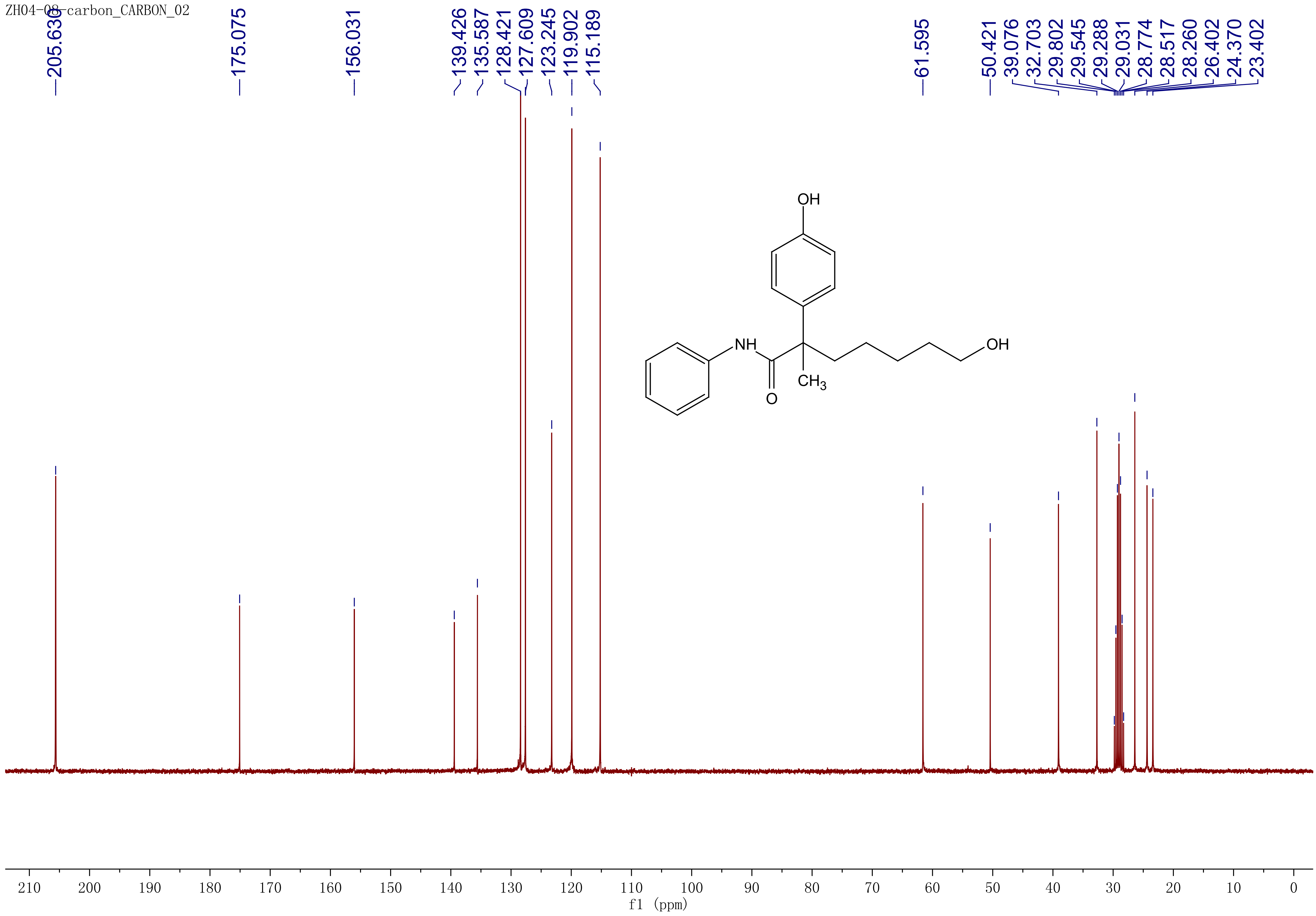
ZH04-08-carbon_CARBON_02
205.630
175.075
156.031
139.426
135.587
128.421
127.609
123.245
119.902
115.189
61.595
50.421
39.076
32.703
29.802
29.545
29.288
29.031
28.774
28.517
28.260
26.402
24.370
23.402
OH
NH
OH
$CH_3$
O
210 200 190 180 170 160 150 140 130 120 110 100 90 80 70 60 50 40 30 20 10 0
f1 (ppm)

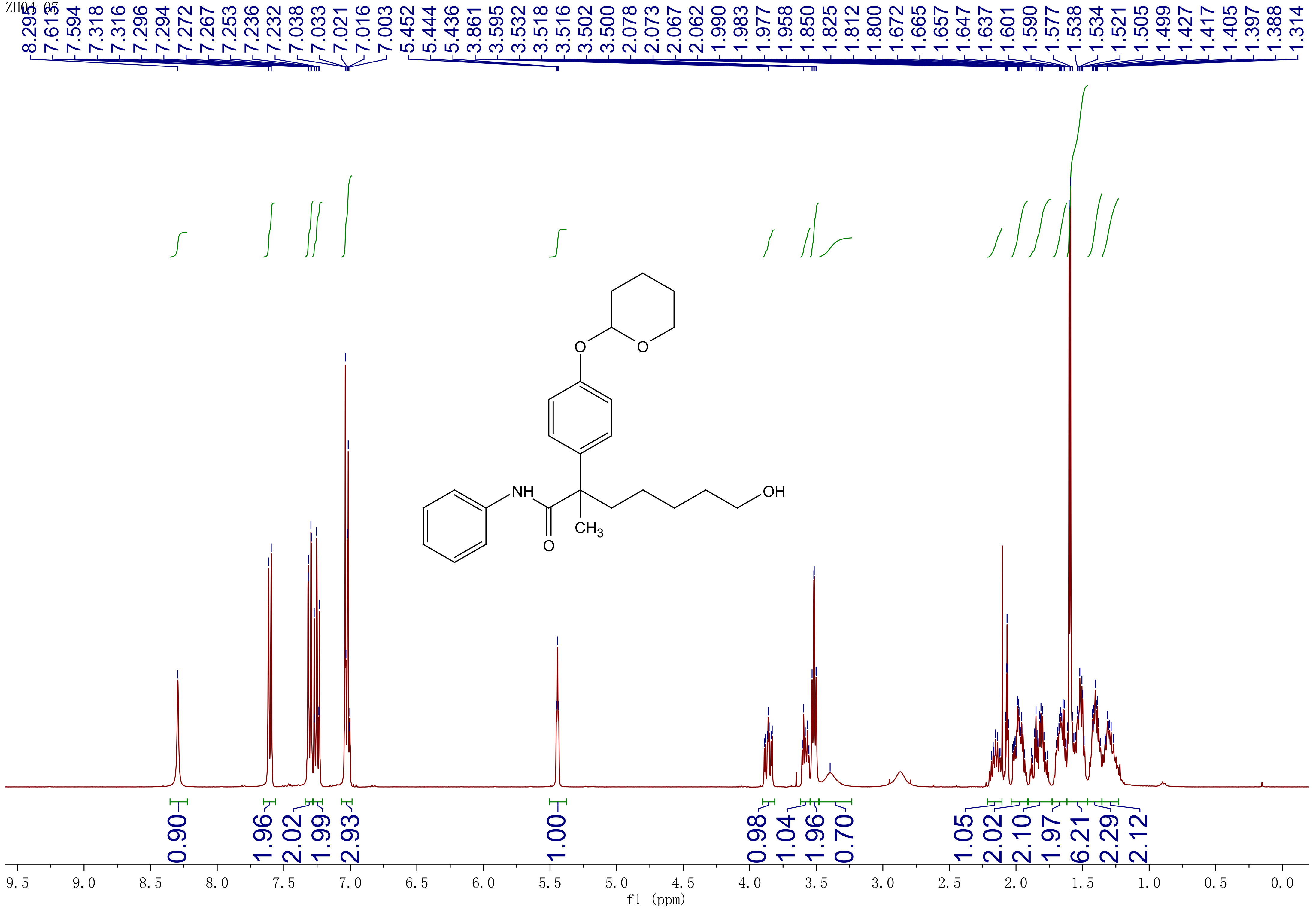
ZH04-07
8.295 7.613 7.594 7.318 7.316 7.296 7.294 7.272 7.267 7.253 7.236 7.232 7.038 7.033 7.021 7.016 7.003 5.452 5.444 5.436 3.861 3.595 3.532 3.518 3.516 3.502 3.500 2.078 2.073 2.067 2.062 1.990 1.983 1.977 1.958 1.850 1.825 1.812 1.800 1.672 1.665 1.657 1.647 1.637 1.601 1.590 1.577 1.538 1.534 1.521 1.505 1.499 1.427 1.417 1.405 1.397 1.388 1.314
O
O
NH
OH
CH3
O
0.90 1.96 2.02 1.99 2.93 1.00 0.98 1.04 1.96 0.70 1.05 2.02 2.10 1.97 6.21 2.29 2.12
9.5 9.0 8.5 8.0 7.5 7.0 6.5 6.0 5.5 5.0 4.5 4.0 3.5 3.0 2.5 2.0 1.5 1.0 0.5 0.0
f1 (ppm)

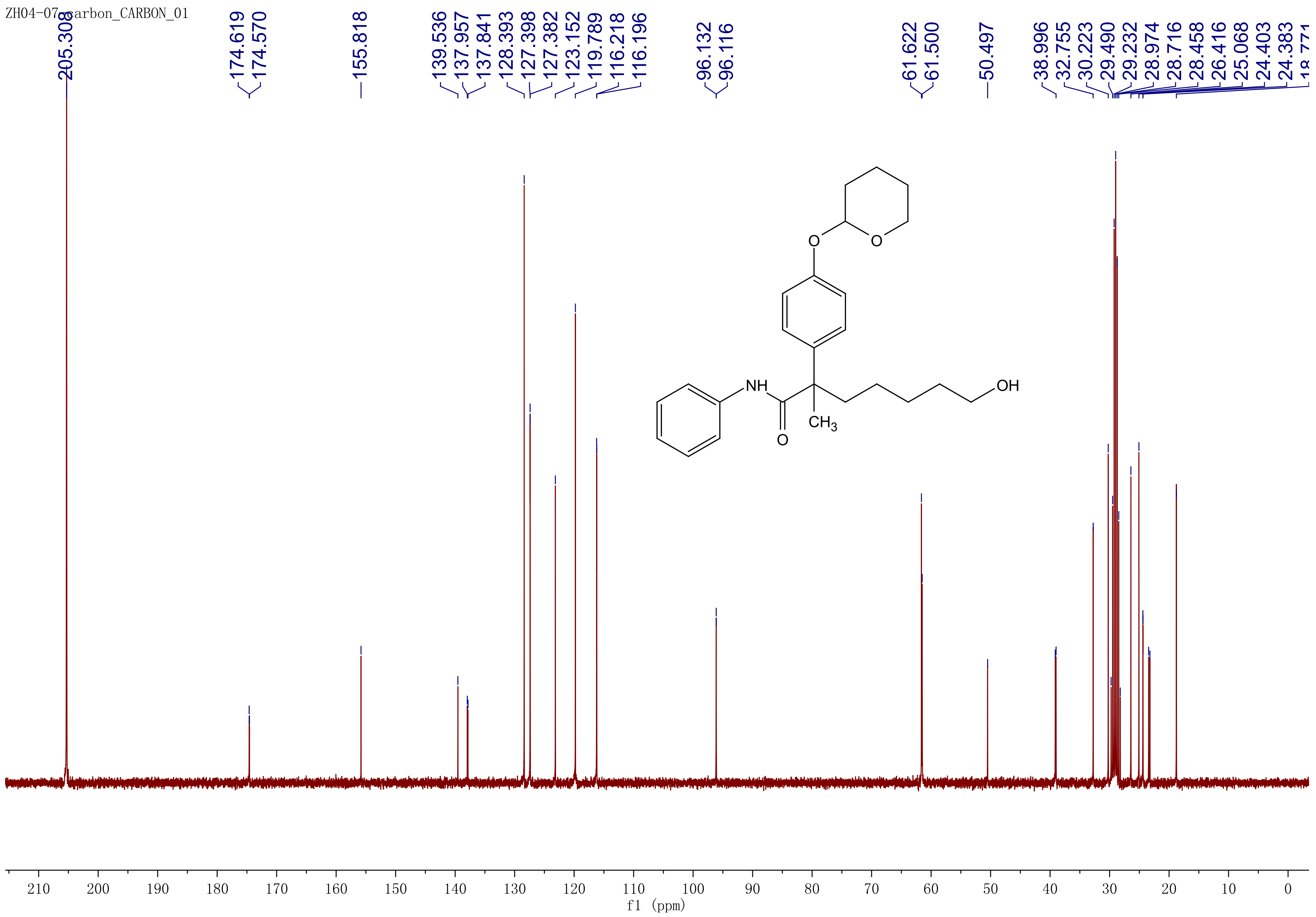
ZH04-07-carbon_CARBON_01
205.308
174.619
174.570
155.818
139.536
137.957
137.841
128.393
127.398
127.382
123.152
119.789
116.218
116.196
96.132
96.116
61.622
61.500
50.497
38.996
32.755
30.223
29.490
29.232
28.974
28.716
28.458
26.416
25.068
24.403
24.383
O
O
NH
OH
$CH_3$
O
210 200 190 180 170 160 150 140 130 120 110 100 90 80 70 60 50 40 30 20 10 0
f1 (ppm)

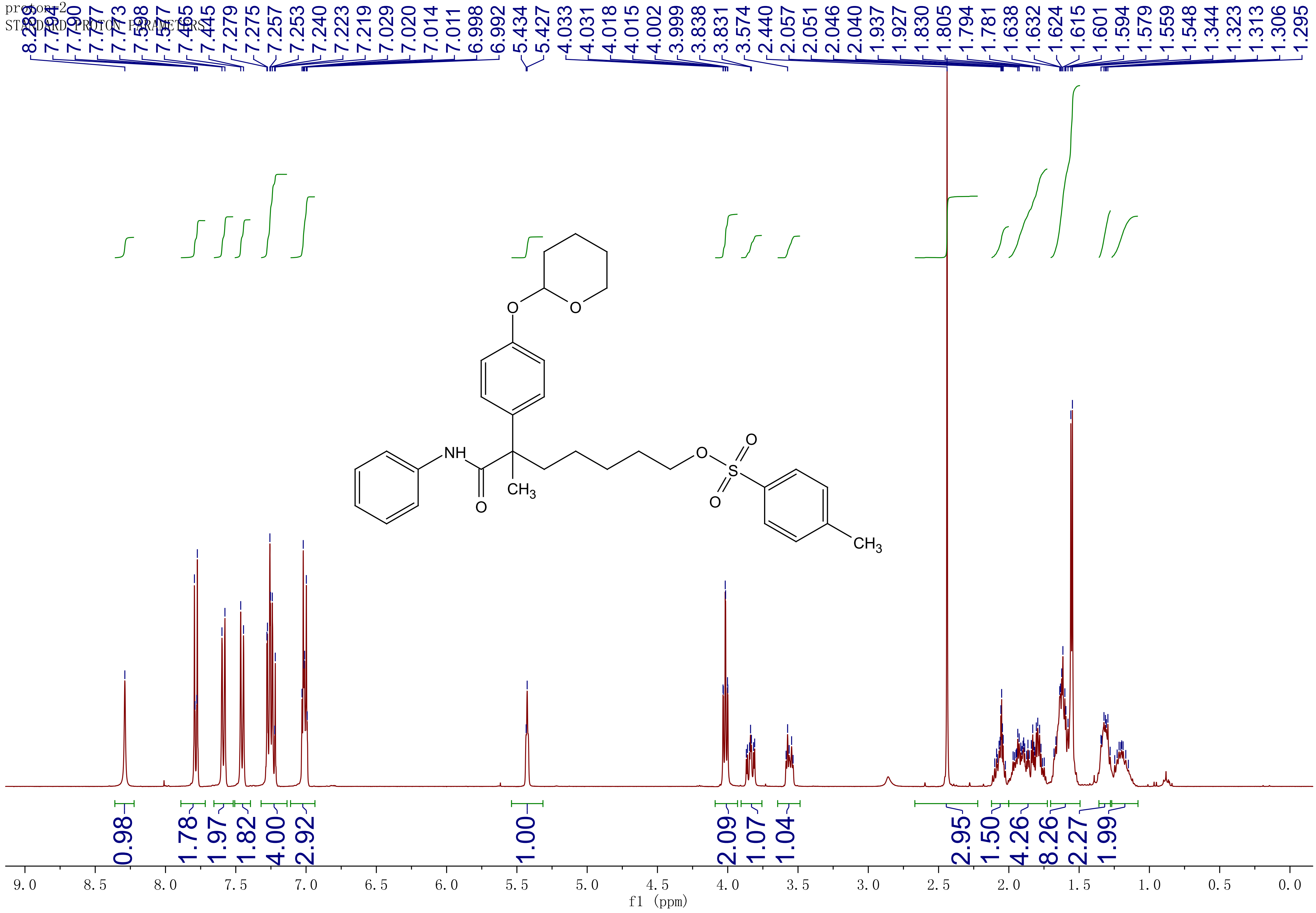
proton-2
STANDARD PROTON PARAMETERS
8.289
7.794
7.790
7.777
7.773
7.598
7.577
7.465
7.445
7.279
7.275
7.257
7.253
7.240
7.223
7.219
7.029
7.020
7.014
7.011
6.998
6.992
5.434
5.427
4.033
4.031
4.018
4.015
4.002
3.999
3.838
3.831
3.574
2.440
2.057
2.051
2.046
2.040
1.937
1.927
1.830
1.805
1.794
1.781
1.638
1.632
1.624
1.615
1.601
1.594
1.579
1.559
1.548
1.344
1.323
1.313
1.306
1.295
O
O
NH
O
CH3
O
S
O
O
CH3
0.98
1.78
1.97
1.82
4.00
2.92
1.00
2.09
1.07
1.04
2.95
1.50
4.26
8.26
2.27
1.99
9.0
8.5
8.0
7.5
7.0
6.5
6.0
5.5
5.0
4.5
4.0
3.5
3.0
2.5
2.0
1.5
1.0
0.5
0.0
f1 (ppm)

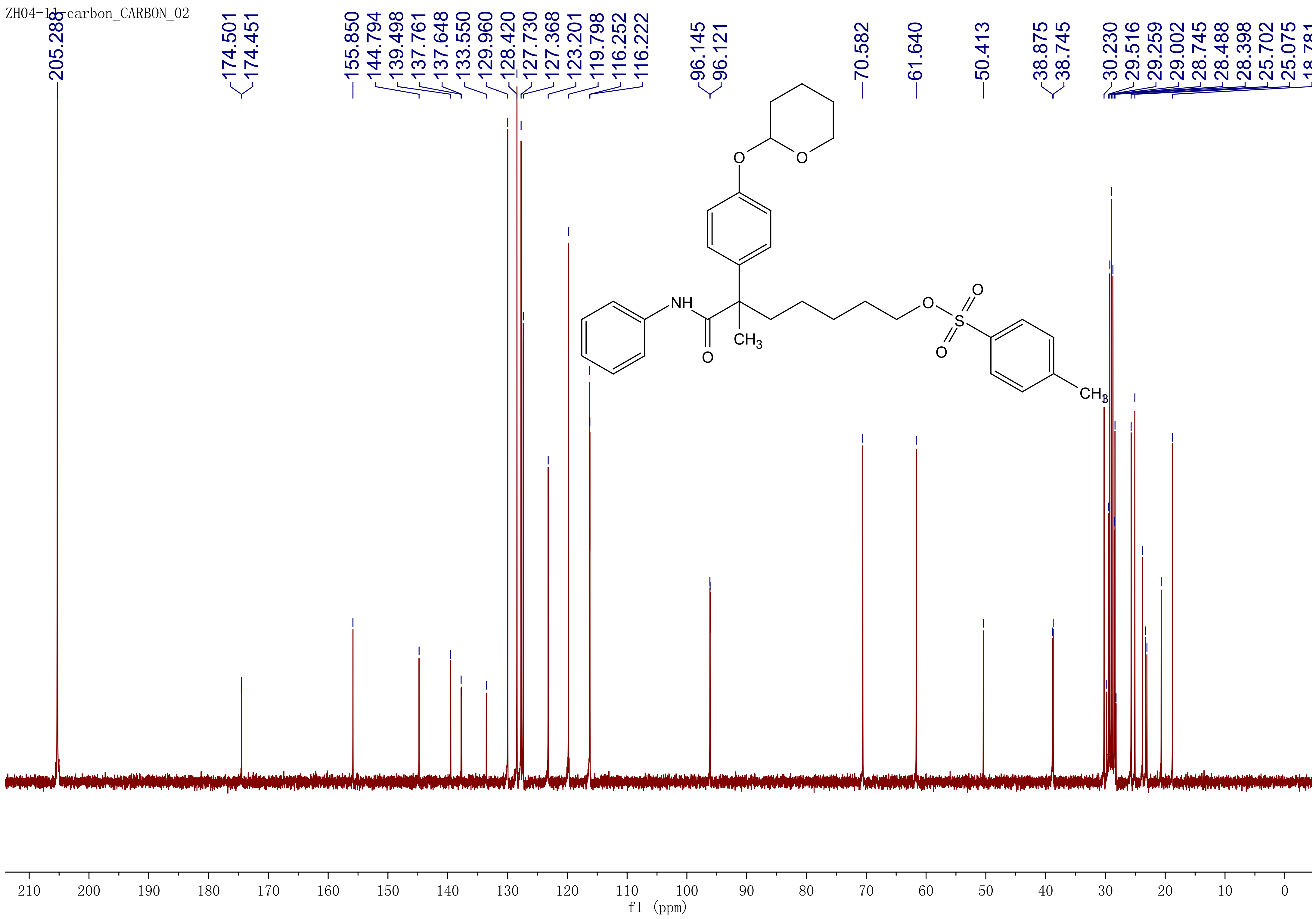
ZH04-11-carbon_CARBON_02
205.288
174.501
174.451
155.850
144.794
139.498
137.761
137.648
133.550
129.960
128.420
127.730
127.368
123.201
119.798
116.252
116.222
96.145
96.121
70.582
61.640
50.413
38.875
38.745
30.230
29.516
29.259
29.002
28.745
28.488
28.398
25.702
25.075
O
O
NH
O
CH3
O
S
O
O
CH3
210
200
190
180
170
160
150
140
130
120
110
100
90
80
70
60
50
40
30
20
10
0
f1 (ppm)

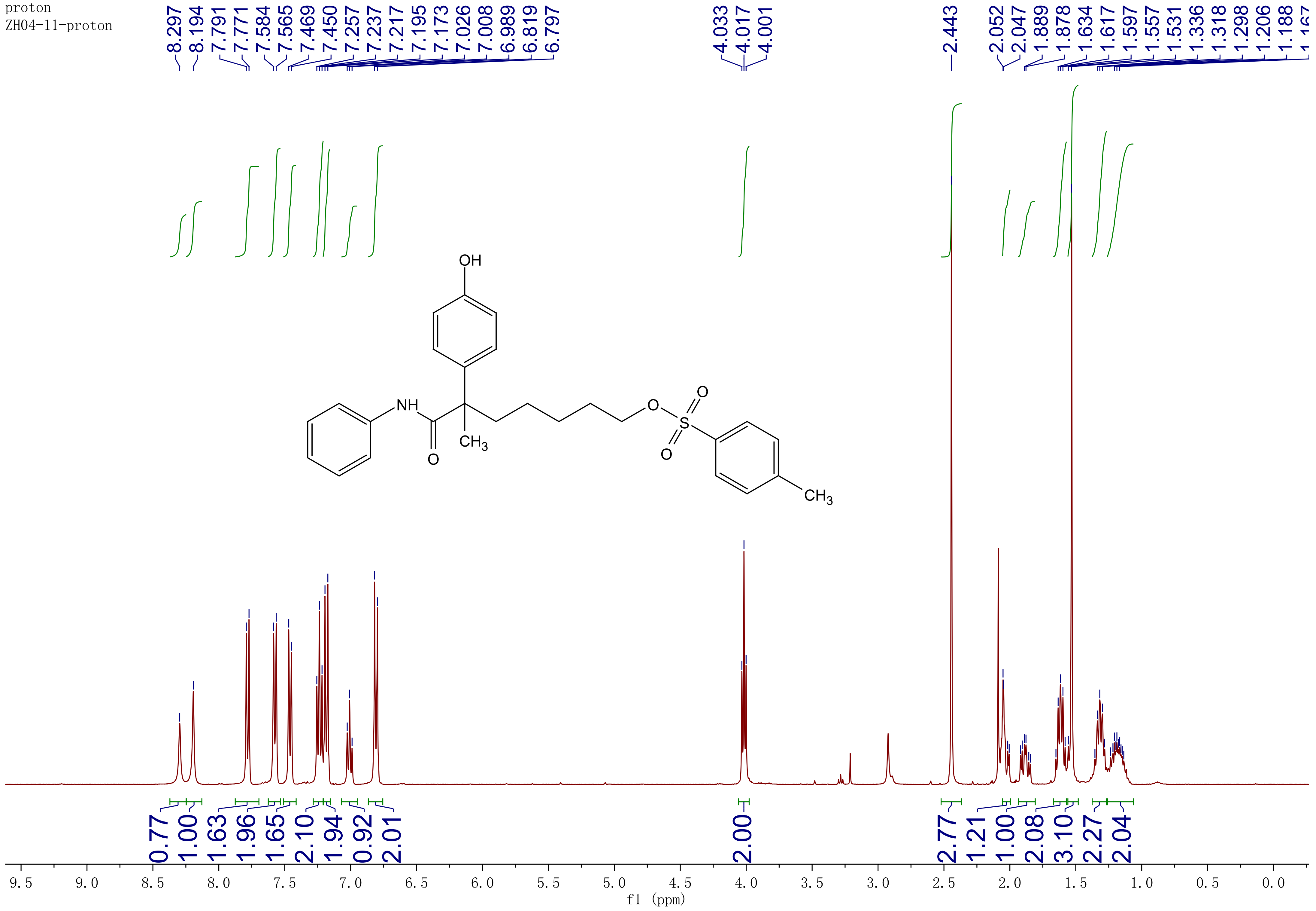

proton
ZH04-11-proton
8.297
8.194
7.791
7.771
7.584
7.565
7.469
7.450
7.257
7.237
7.217
7.195
7.173
7.026
7.008
6.989
6.819
6.797
4.033
4.017
4.001
2.443
2.052
2.047
1.889
1.878
1.634
1.617
1.597
1.557
1.531
1.336
1.318
1.298
1.206
1.188
OH
NH
O
CH3
O
S
O
O
CH3
0.77
1.00
1.63
1.96
1.65
2.10
1.94
0.92
2.01
2.00
2.77
1.21
1.00
2.08
3.10
2.27
2.04
9.5
9.0
8.5
8.0
7.5
7.0
6.5
6.0
5.5
5.0
4.5
4.0
3.5
3.0
2.5
2.0
1.5
1.0
0.5
0.0
f1 (ppm)

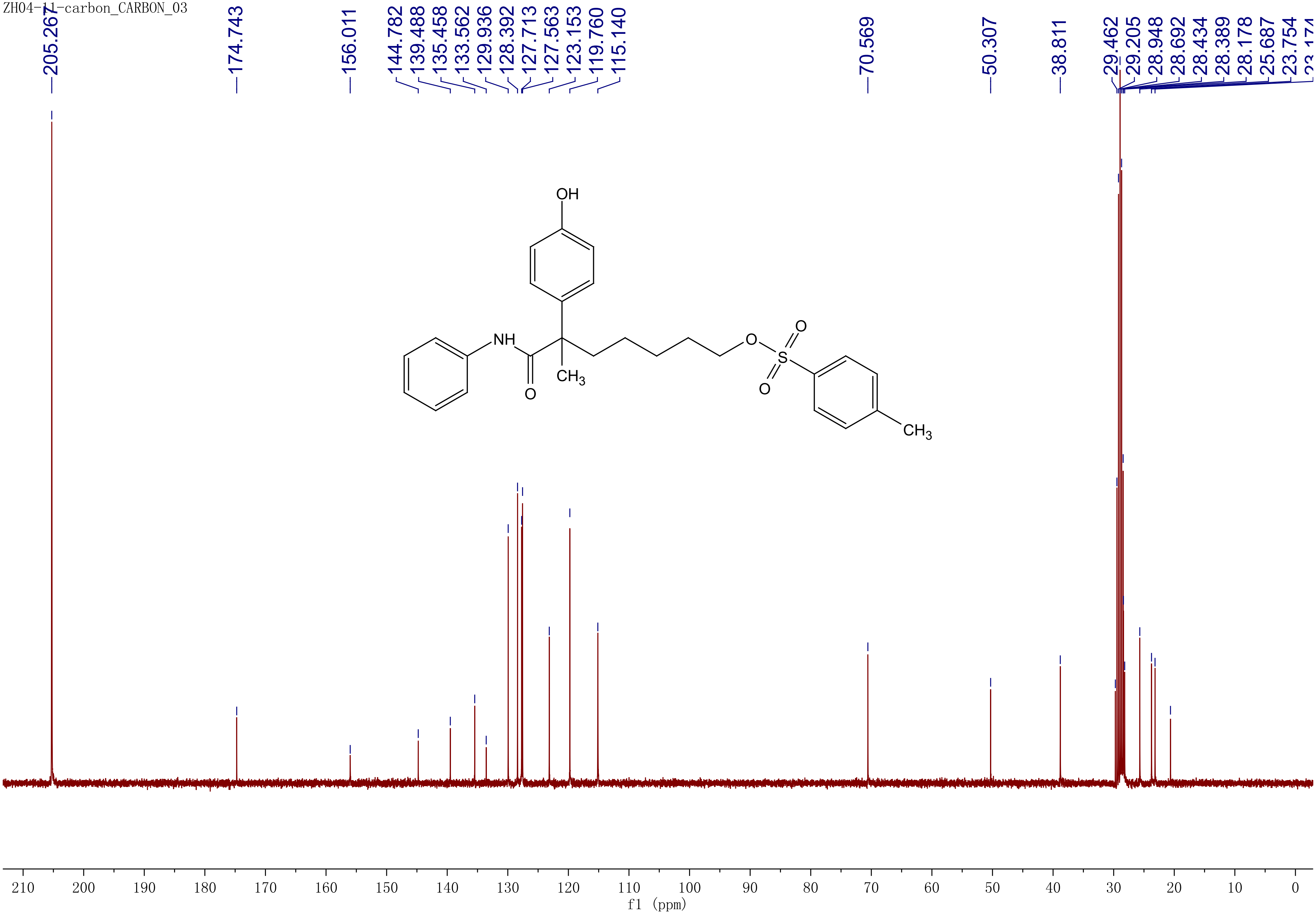

ZH04-11-carbon_CARBON_03
205.267
174.743
156.011
144.782
139.488
135.458
133.562
129.936
128.392
127.713
127.563
123.153
119.760
115.140
70.569
50.307
38.811
29.462
29.205
28.948
28.692
28.434
28.389
28.178
25.687
23.754
OH
NH
O
CH3
O
S
O
O
CH3
210 200 190 180 170 160 150 140 130 120 110 100 90 80 70 60 50 40 30 20 10 0
f1 (ppm)

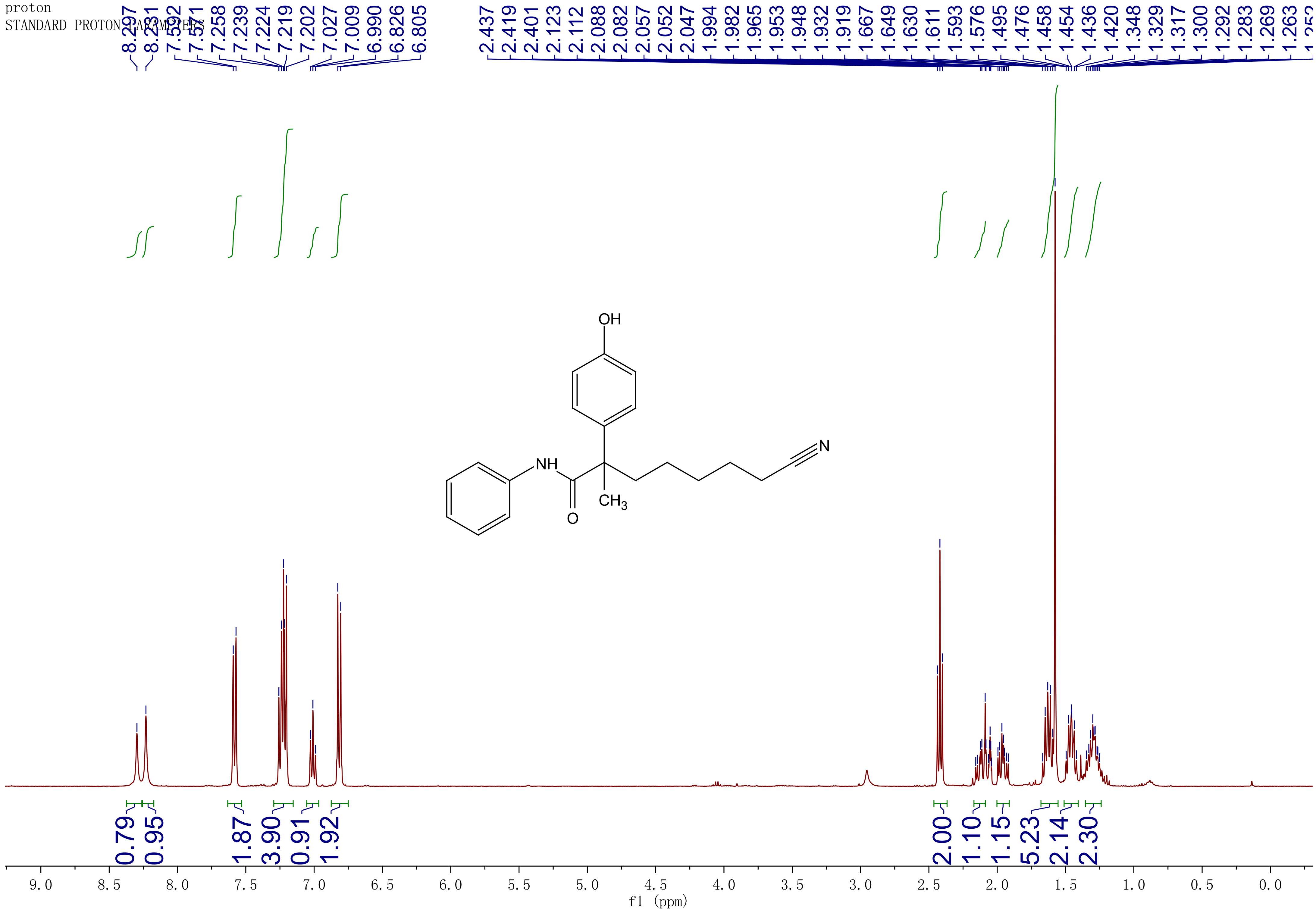
proton
STANDARD PROTON PARAMETERS
8.297
8.231
7.592
7.571
7.258
7.239
7.224
7.219
7.202
7.027
7.009
6.990
6.826
6.805
2.437
2.419
2.401
2.123
2.112
2.088
2.082
2.057
2.052
2.047
1.994
1.982
1.965
1.953
1.948
1.932
1.919
1.667
1.649
1.630
1.611
1.593
1.576
1.495
1.476
1.458
1.454
1.436
1.420
1.348
1.329
1.317
1.300
1.292
1.283
1.269
1.263
OH
NH
O
$CH_3$
N
0.79
0.95
1.87
3.90
0.91
1.92
2.00
1.10
1.15
5.23
2.14
2.30
9.0
8.5
8.0
7.5
7.0
6.5
6.0
5.5
5.0
4.5
4.0
3.5
3.0
2.5
2.0
1.5
1.0
0.5
0.0
f1 (ppm)

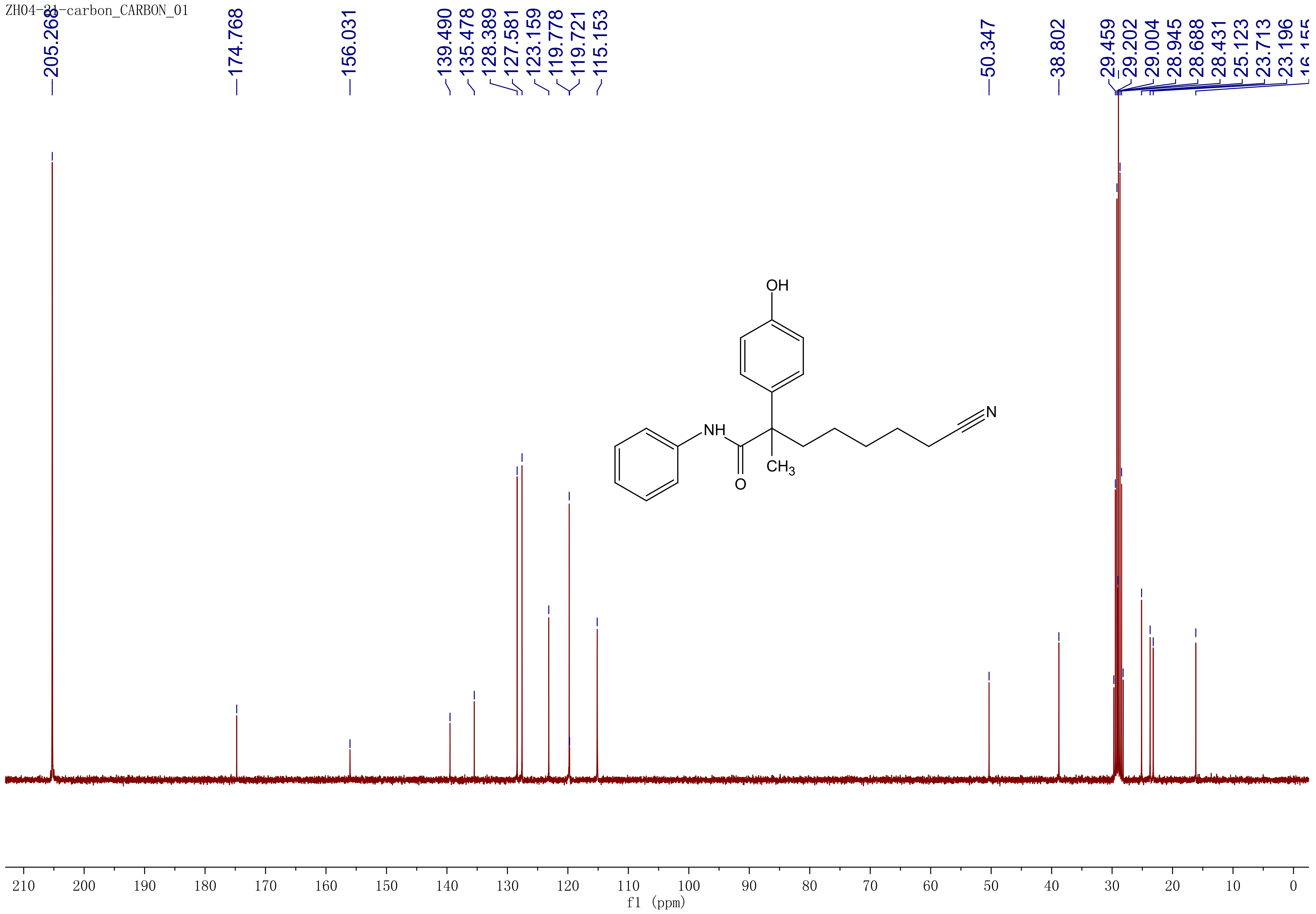

ZH04-21-carbon_CARBON_01
205.268
174.768
156.031
139.490
135.478
128.389
127.581
123.159
119.778
119.721
115.153
50.347
38.802
29.459
29.202
29.004
28.945
28.688
28.431
25.123
23.713
23.196
OH
NH
N
O
CH3
210 200 190 180 170 160 150 140 130 120 110 100 90 80 70 60 50 40 30 20 10 0
f1 (ppm)

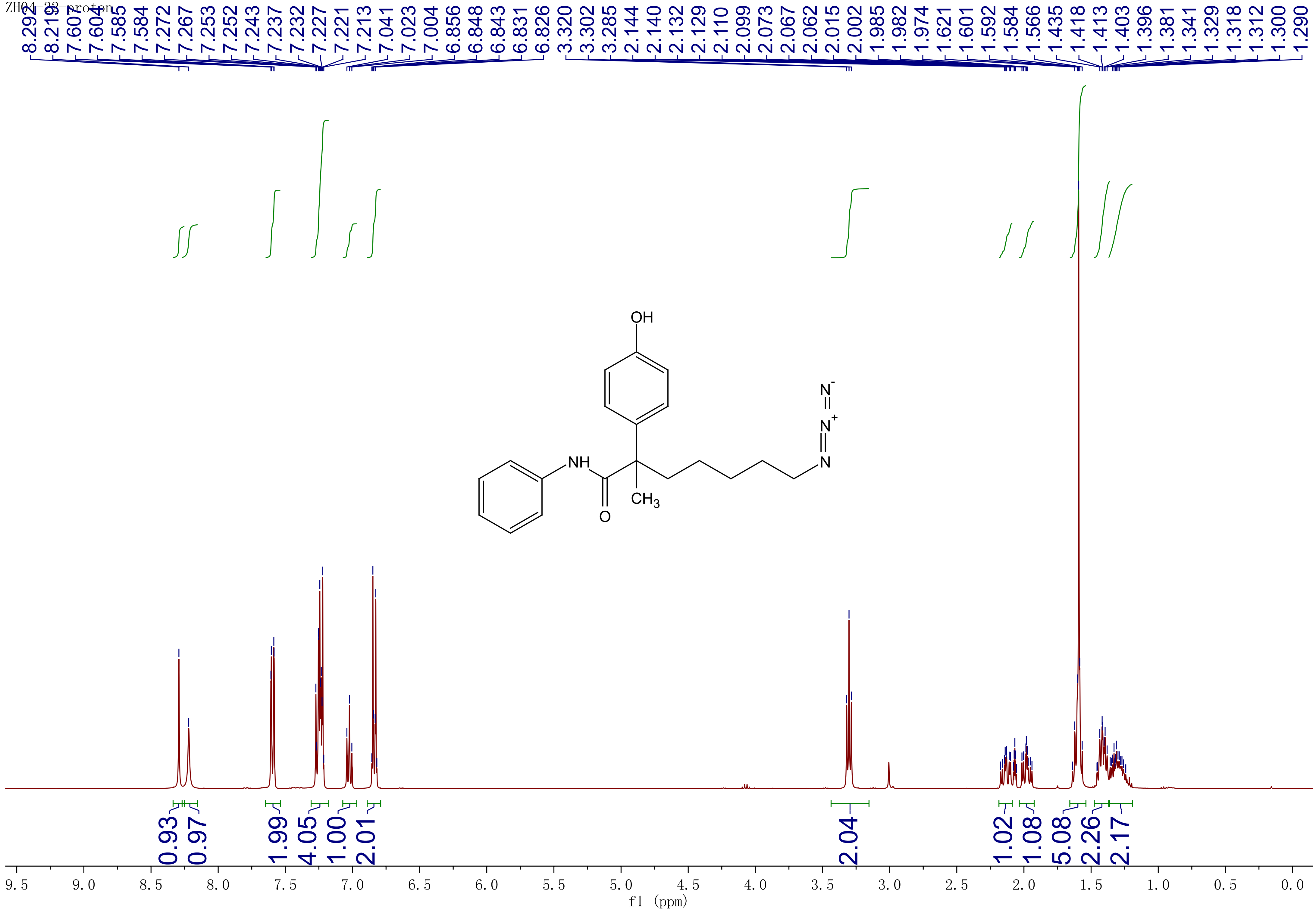

ZH04-22-proton
8.292
8.219
7.607
7.604
7.585
7.584
7.272
7.267
7.253
7.252
7.243
7.237
7.232
7.227
7.221
7.213
7.041
7.023
7.004
6.856
6.848
6.843
6.831
6.826
3.320
3.302
3.285
2.144
2.140
2.132
2.129
2.110
2.099
2.073
2.067
2.062
2.015
2.002
1.985
1.982
1.974
1.621
1.601
1.592
1.584
1.566
1.435
1.418
1.413
1.403
1.396
1.381
1.341
1.329
1.318
1.312
1.300
1.290
OH
NH
O
CH3
N-
N+
N
0.93
0.97
1.99
4.05
1.00
2.01
2.04
1.02
1.08
5.08
2.26
2.17
9.5
9.0
8.5
8.0
7.5
7.0
6.5
6.0
5.5
5.0
4.5
4.0
3.5
3.0
2.5
2.0
1.5
1.0
0.5
0.0
f1 (ppm)

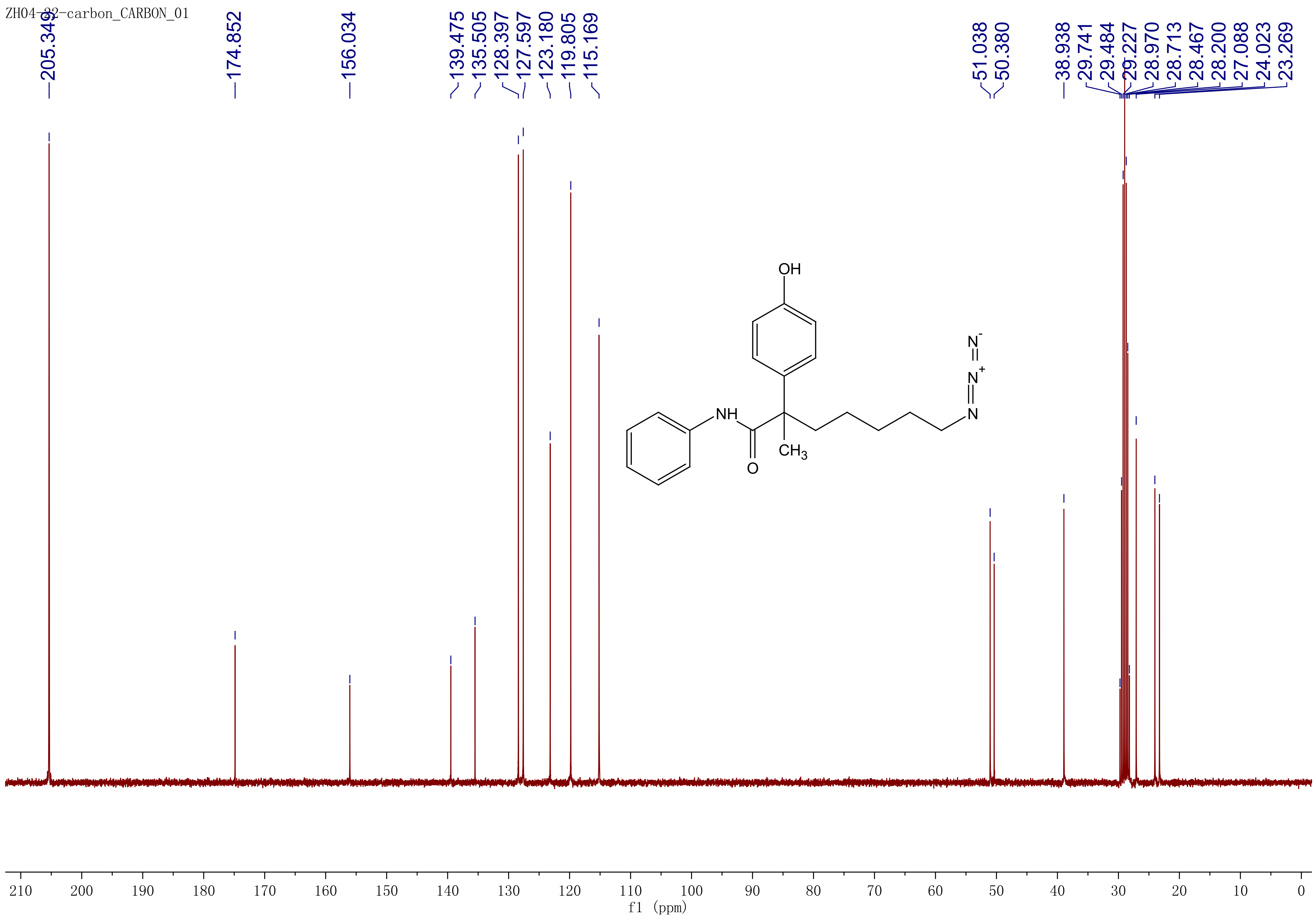

ZH04-82-carbon_CARBON_01
205.34
174.852
156.034
139.475
135.505
128.397
127.597
123.180
119.805
115.169
51.038
50.380
38.938
29.741
29.484
29.227
28.970
28.713
28.467
28.200
27.088
24.023
23.269
OH
NH
O
CH3
N-
N+
N
210
200
190
180
170
160
150
140
130
120
110
100
90
80
70
60
50
40
30
20
10
0
f1 (ppm)

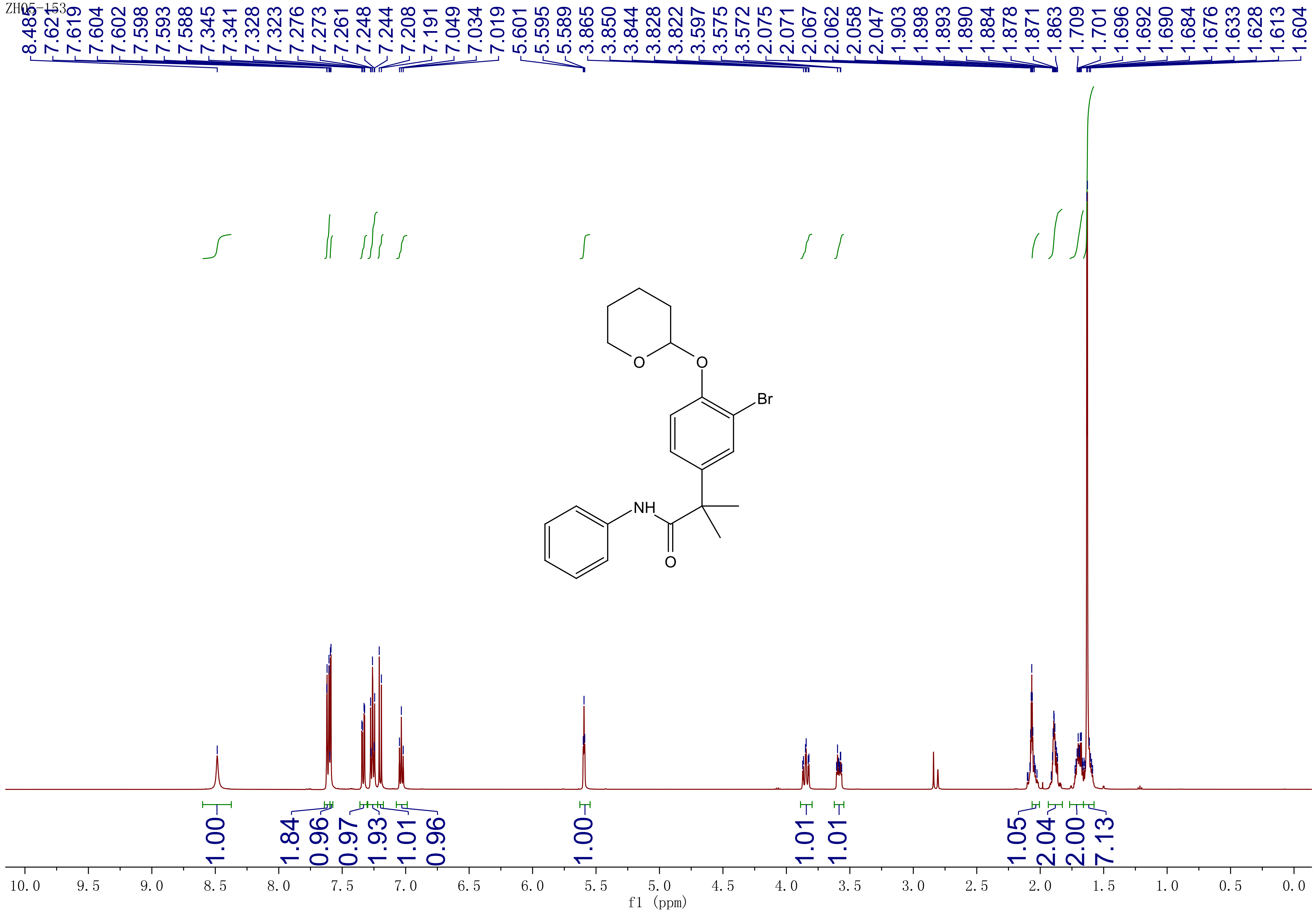
ZH05-153
8.485
7.621
7.619
7.604
7.602
7.598
7.593
7.588
7.345
7.341
7.328
7.323
7.276
7.273
7.261
7.248
7.244
7.208
7.191
7.049
7.034
7.019
5.601
5.595
5.589
3.865
3.850
3.844
3.828
3.822
3.597
3.575
3.572
2.075
2.071
2.067
2.062
2.058
2.047
1.903
1.898
1.893
1.890
1.884
1.878
1.871
1.863
1.709
1.701
1.696
1.692
1.690
1.684
1.676
1.633
1.628
1.613
1.604
O
O
Br
NH
O
1.00
1.84
0.96
0.97
1.93
1.01
0.96
1.00
1.01
1.01
1.05
2.04
2.00
7.13
10.0
9.5
9.0
8.5
8.0
7.5
7.0
6.5
6.0
5.5
5.0
4.5
4.0
3.5
3.0
2.5
2.0
1.5
1.0
0.5
0.0
f1 (ppm)

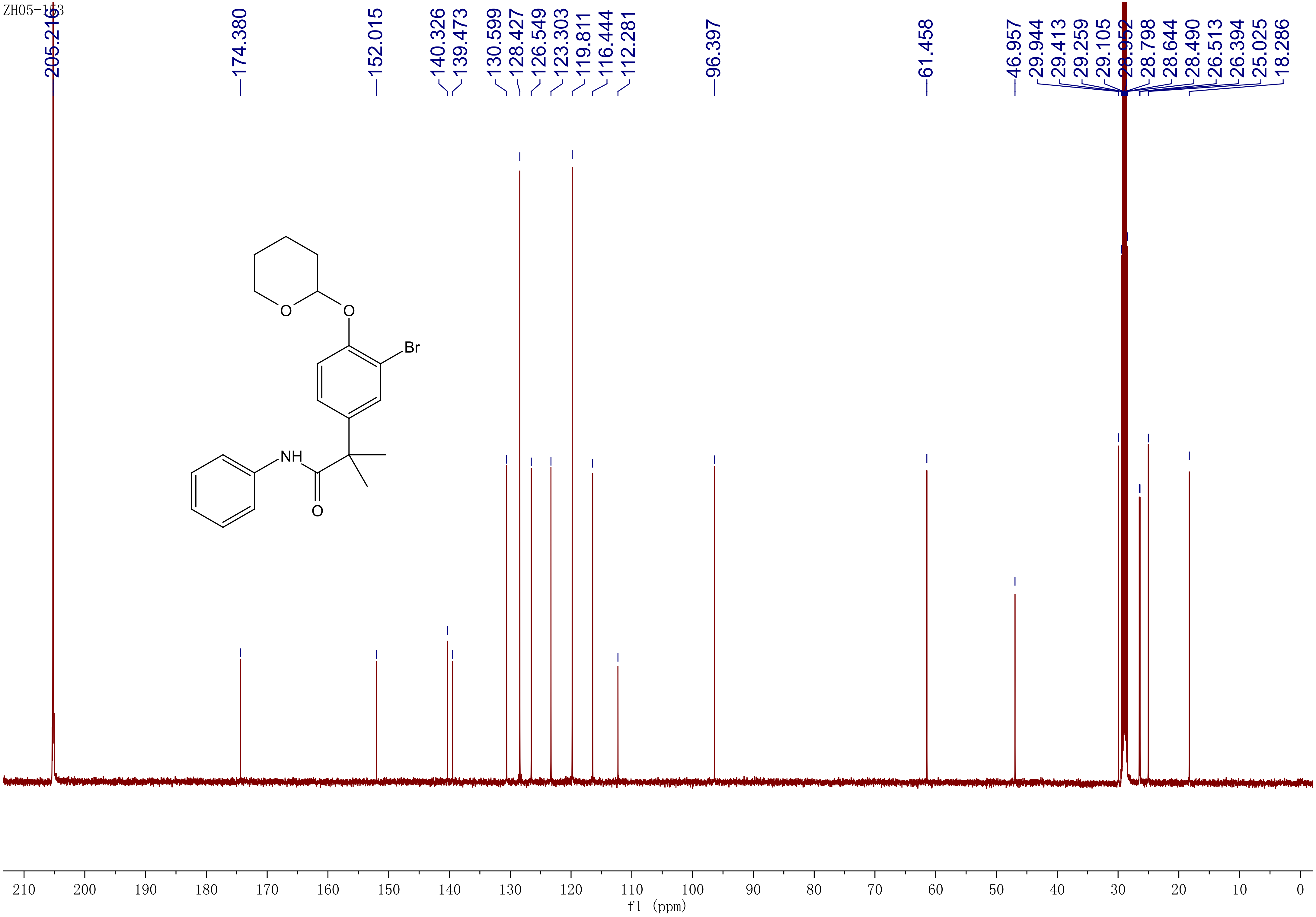
ZH05-153
205.216
174.380
152.015
140.326
139.473
130.599
128.427
126.549
123.303
119.811
116.444
112.281
96.397
61.458
46.957
29.944
29.413
29.259
29.105
28.952
28.798
28.644
28.490
26.513
26.394
25.025
18.286
O
O
Br
NH
O
210 200 190 180 170 160 150 140 130 120 110 100 90 80 70 60 50 40 30 20 10 0
f1 (ppm)

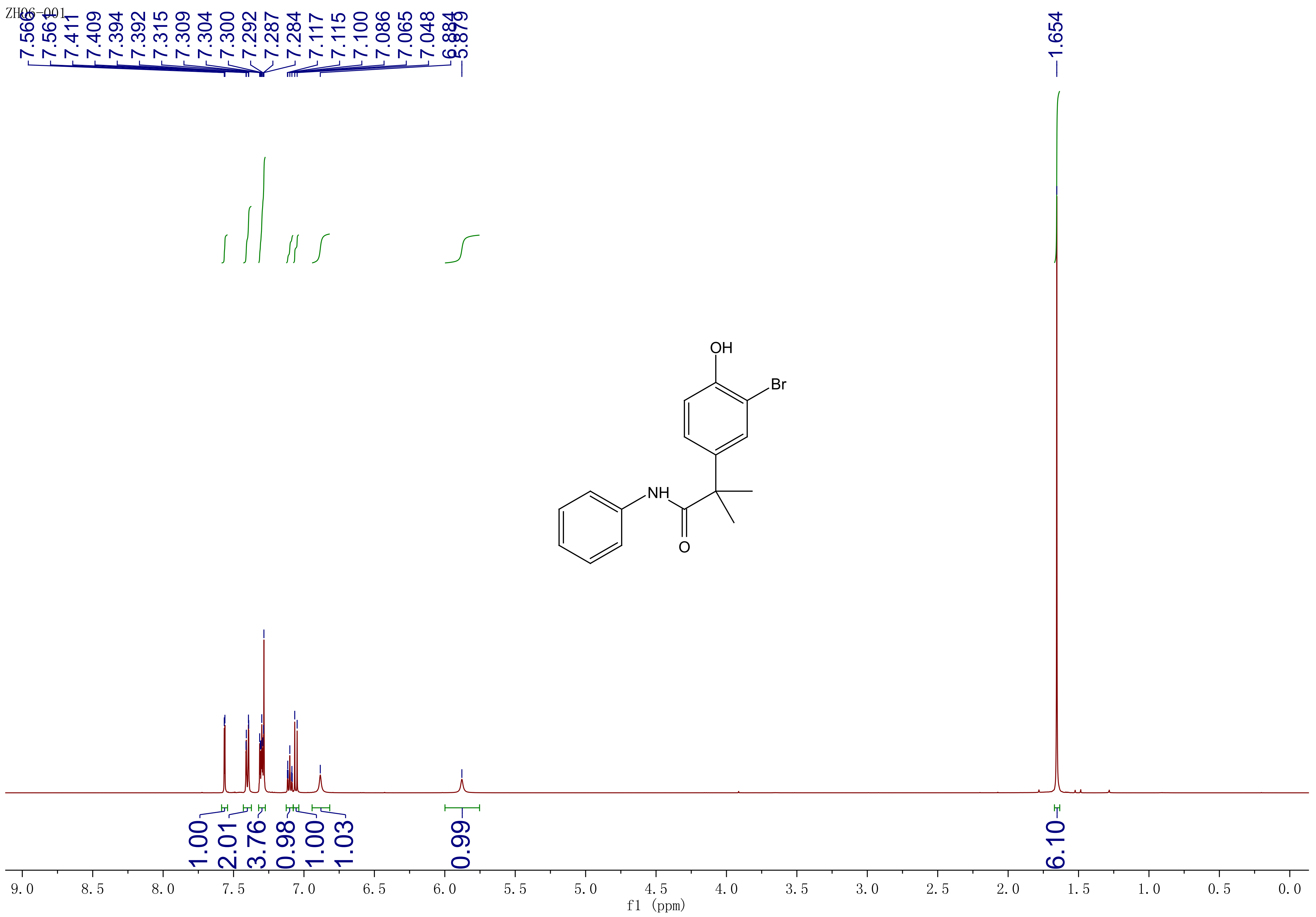

ZH06-001
7.566
7.561
7.411
7.409
7.394
7.392
7.315
7.309
7.304
7.300
7.292
7.287
7.284
7.117
7.115
7.100
7.086
7.065
7.048
6.884
5.879
1.654
OH
Br
NH
O
1.00
2.01
3.76
0.98
1.00
1.03
0.99
6.10
f1 (ppm)

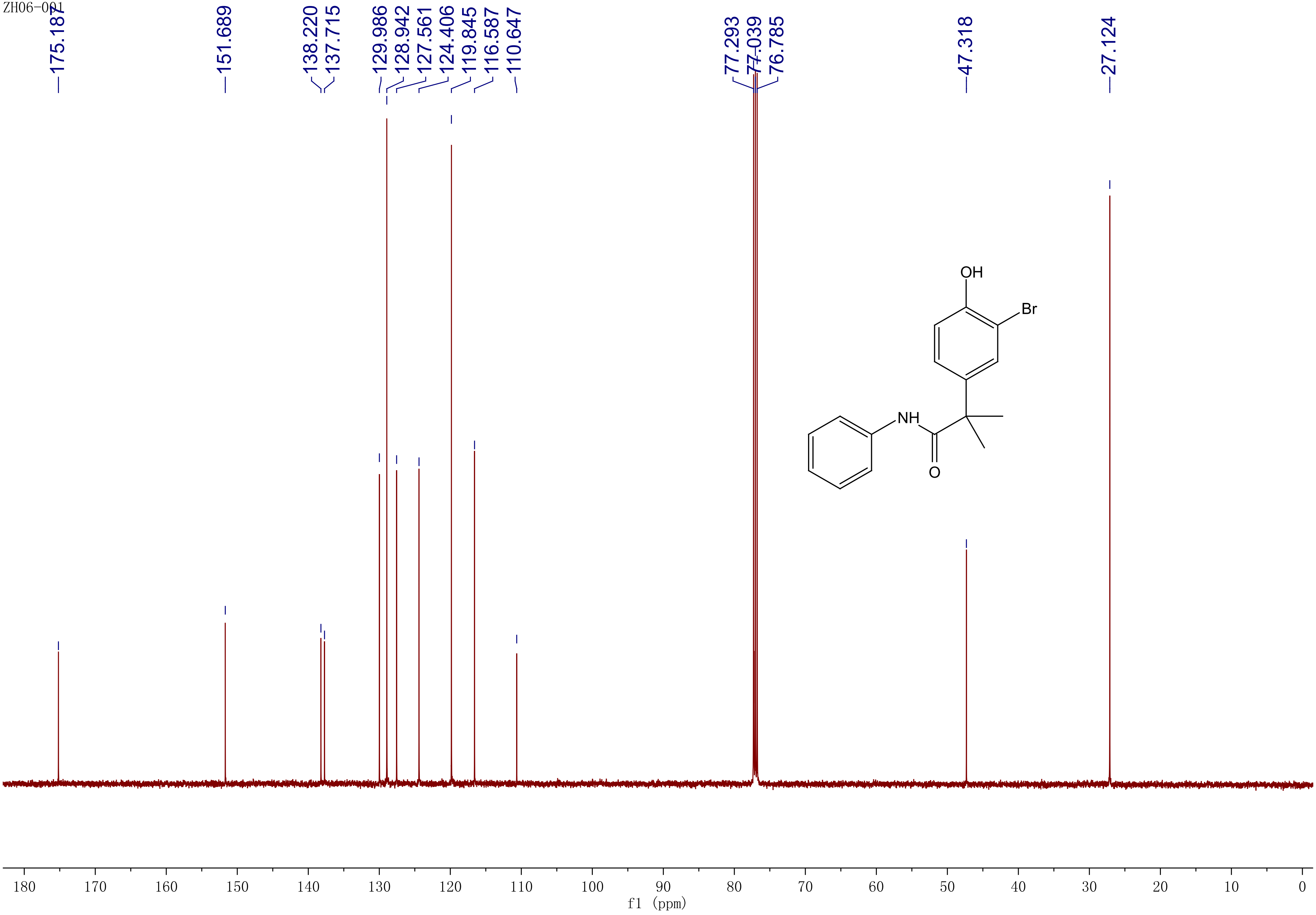
ZH06-001
175.187
151.689
138.220
137.715
129.986
128.942
127.561
124.406
119.845
116.587
110.647
77.293
77.039
76.785
47.318
27.124
OH
Br
NH
O
180 170 160 150 140 130 120 110 100 90 80 70 60 50 40 30 20 10 0
f1 (ppm)

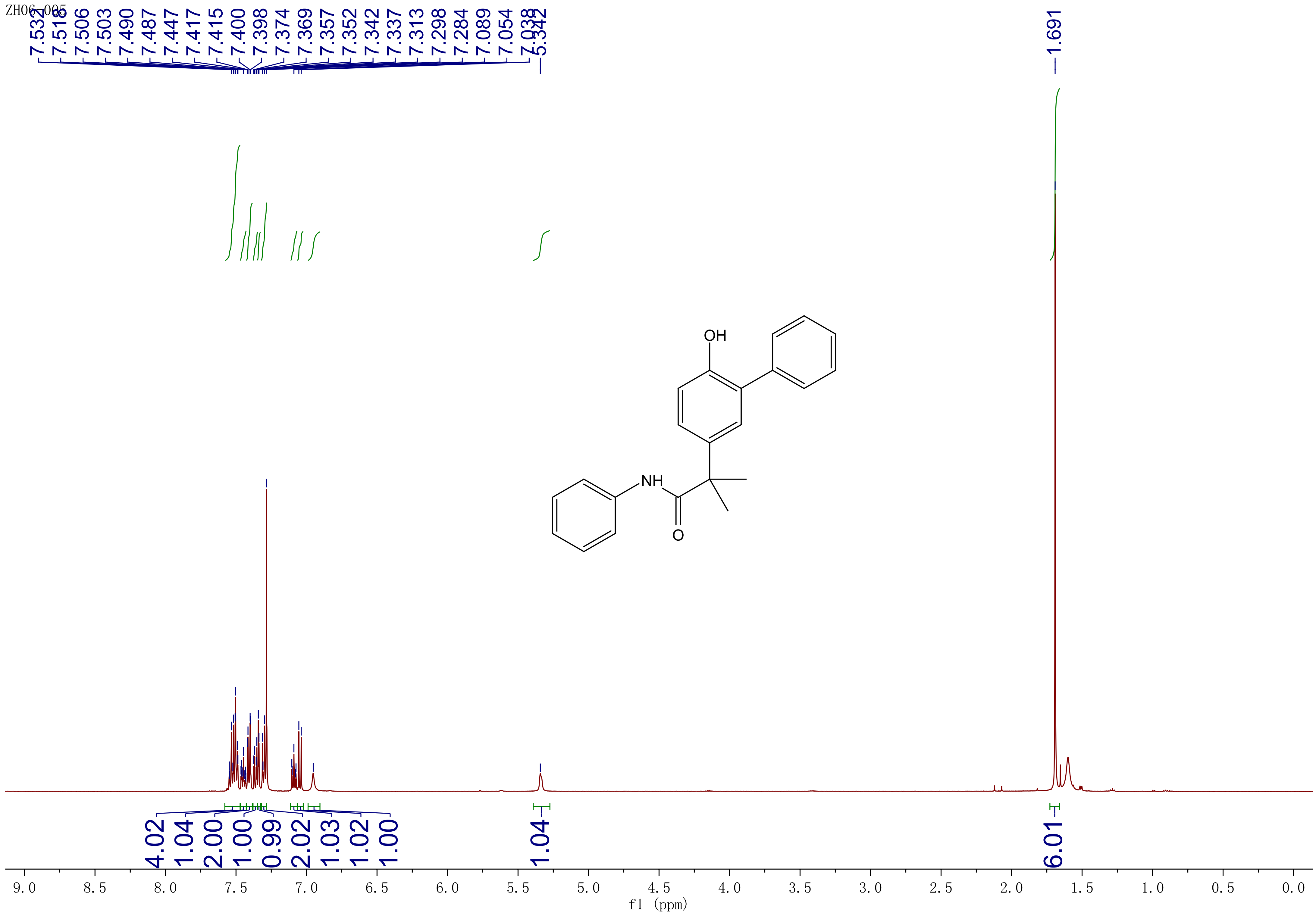
ZH06-005
7.532
7.518
7.506
7.503
7.490
7.487
7.447
7.417
7.415
7.400
7.398
7.374
7.369
7.357
7.352
7.342
7.337
7.313
7.298
7.284
7.089
7.054
7.038
5.342
1.691
OH
NH
O
4.02
1.04
2.00
1.00
0.99
2.02
1.03
1.02
1.00
1.04
6.01
9.0 8.5 8.0 7.5 7.0 6.5 6.0 5.5 5.0 4.5 4.0 3.5 3.0 2.5 2.0 1.5 1.0 0.5 0.0
f1 (ppm)

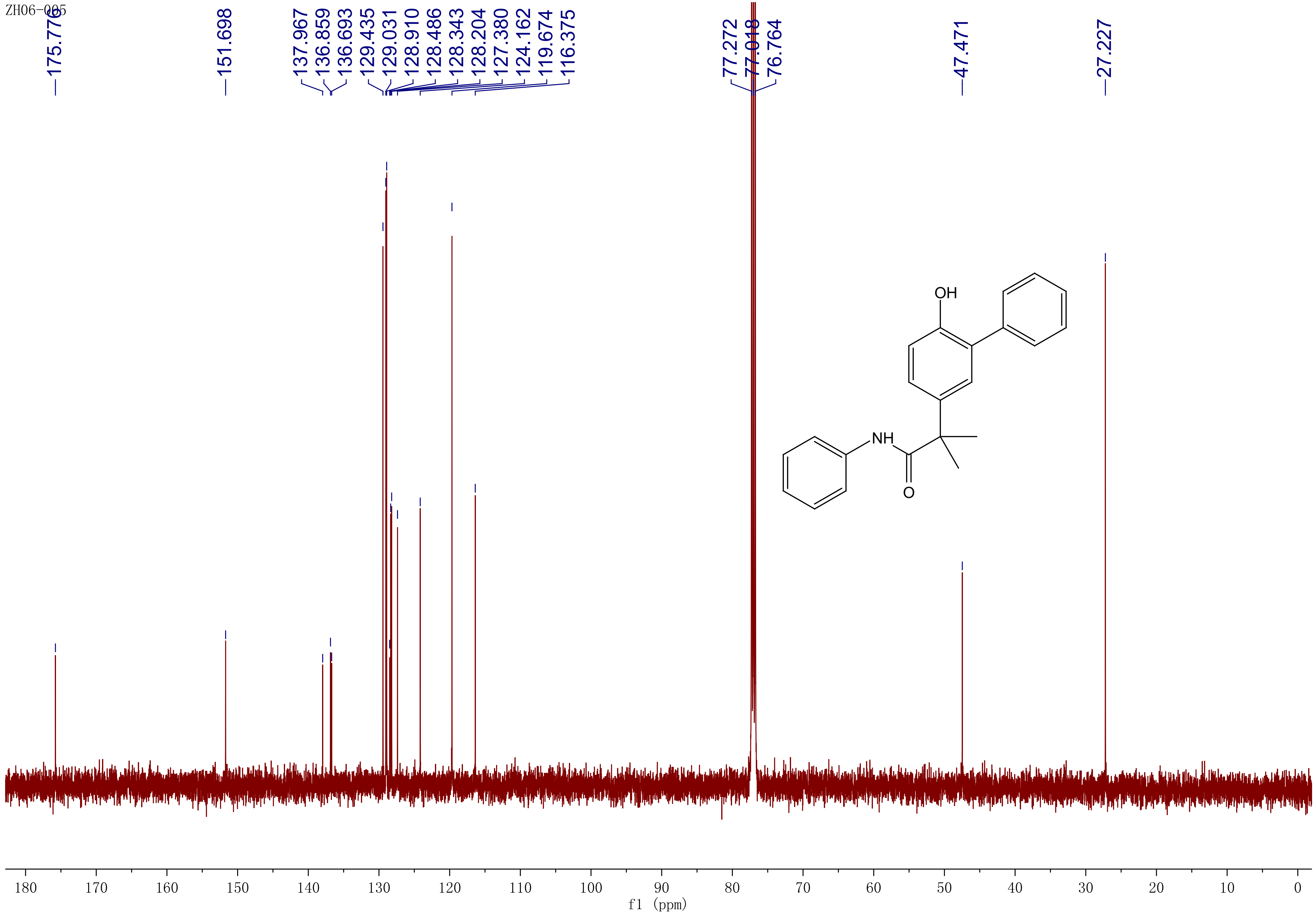
ZH06-005
175.776
151.698
137.967
136.859
136.693
129.435
129.031
128.910
128.486
128.343
128.204
127.380
124.162
119.674
116.375
77.272
77.018
76.764
47.471
27.227
OH
NH
O
180 170 160 150 140 130 120 110 100 90 80 70 60 50 40 30 20 10 0
f1 (ppm)

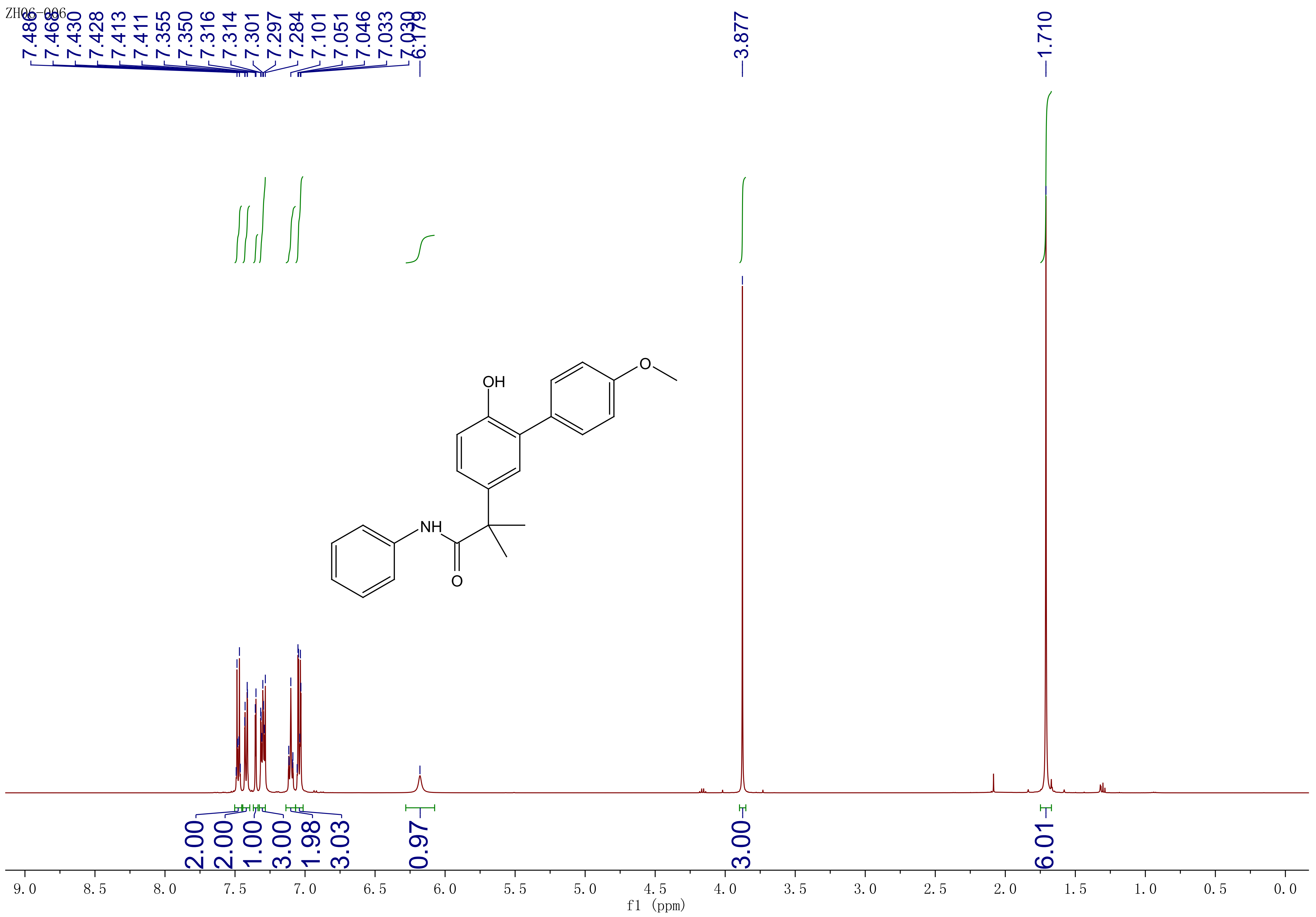

ZH06-006
7.486
7.469
7.430
7.428
7.413
7.411
7.355
7.350
7.316
7.314
7.301
7.297
7.284
7.101
7.051
7.046
7.033
7.030
6.179
3.877
1.710
OH
O
NH
O
2.00
2.00
1.00
3.00
1.98
3.03
0.97
3.00
6.01
9.0
8.5
8.0
7.5
7.0
6.5
6.0
5.5
5.0
4.5
4.0
3.5
3.0
2.5
2.0
1.5
1.0
0.5
0.0
f1 (ppm)

ZH06-006

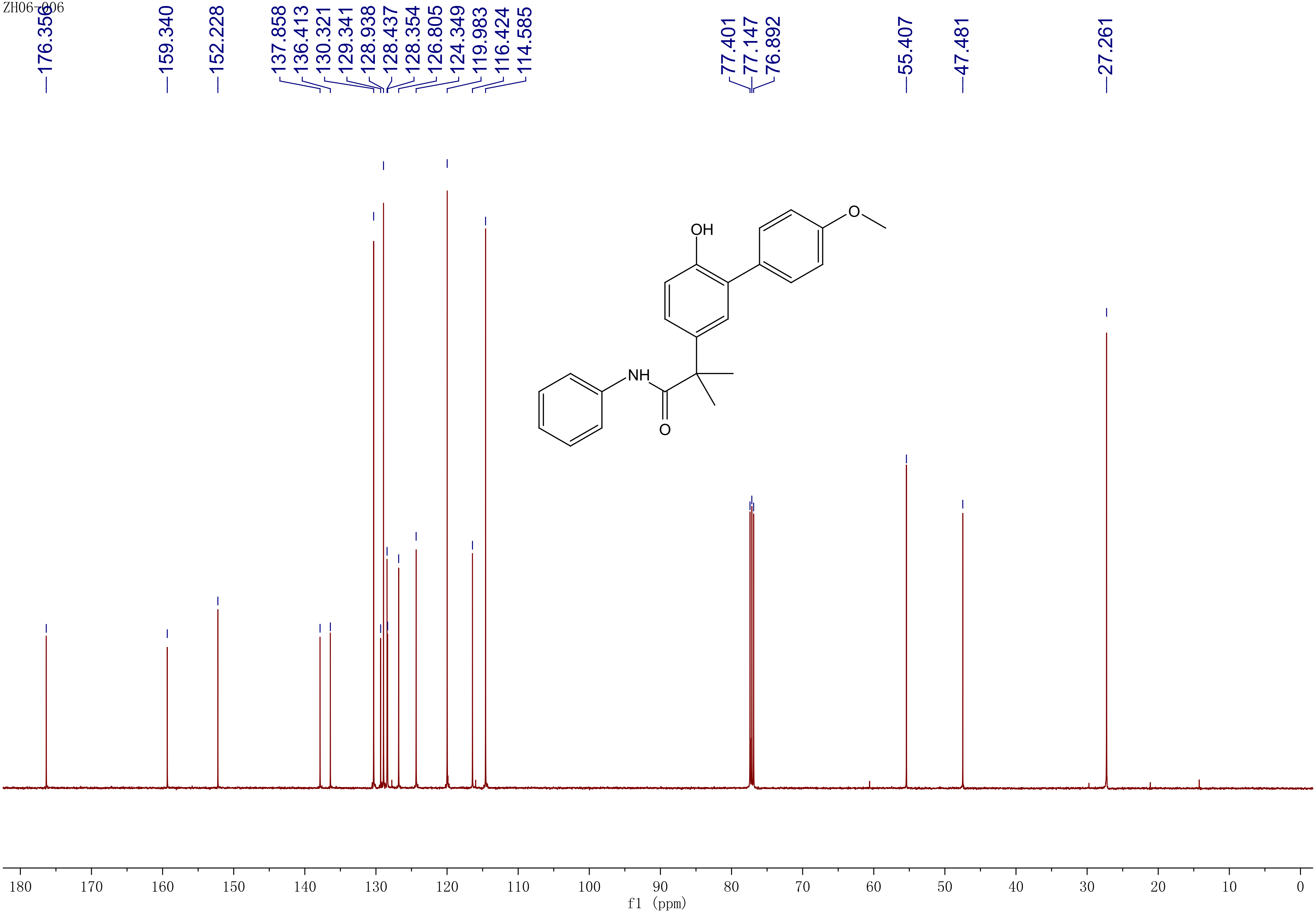
176.356
159.340
152.228
137.858
136.413
130.321
129.341
128.938
128.437
128.354
126.805
124.349
119.983
116.424
114.585
77.401
77.147
76.892
55.407
47.481
27.261
OH
O
NH
O
180 170 160 150 140 130 120 110 100 90 80 70 60 50 40 30 20 10 0
f1 (ppm)

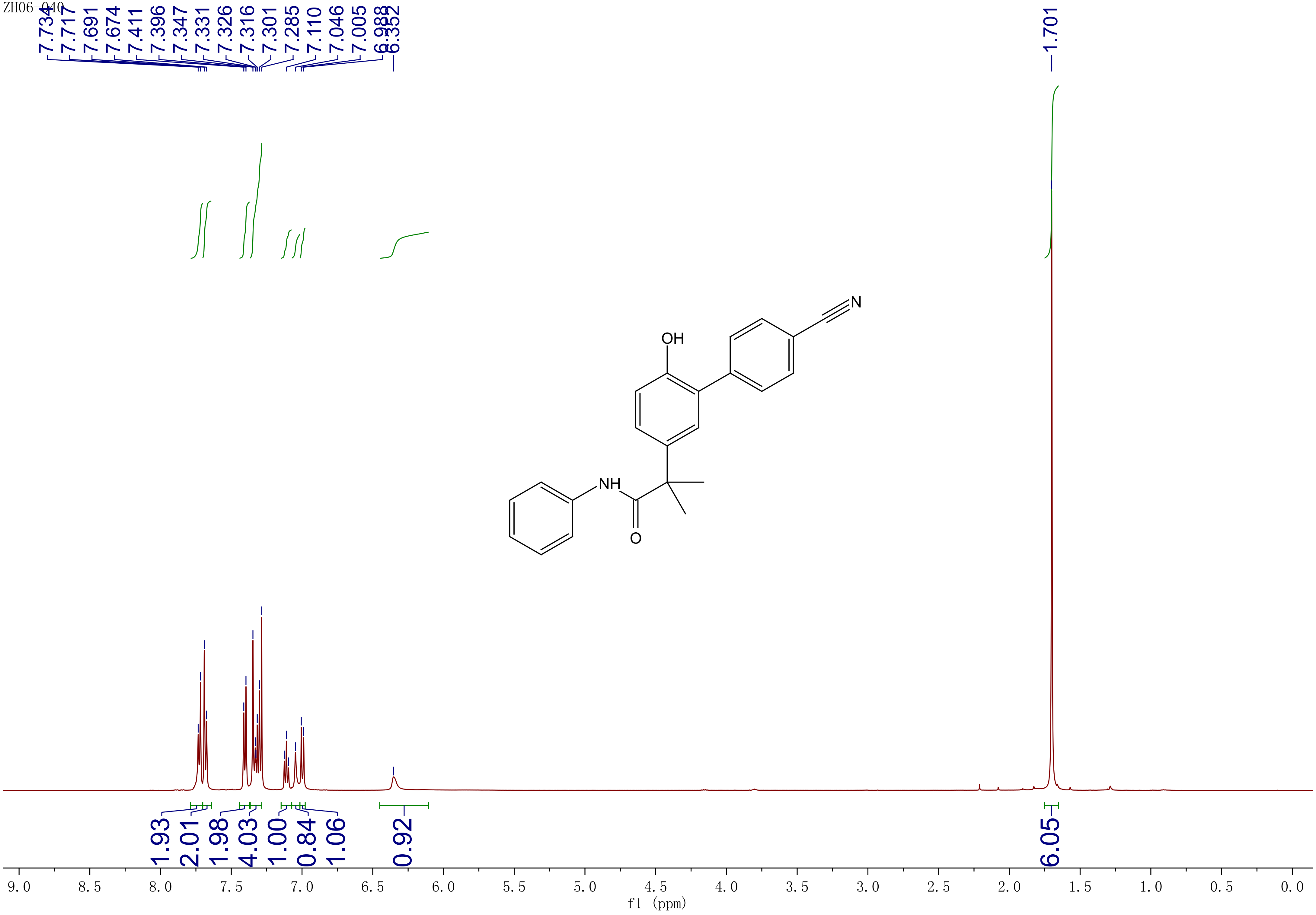

ZH06-040
7.734
7.717
7.691
7.674
7.411
7.396
7.347
7.331
7.326
7.316
7.301
7.285
7.110
7.046
7.005
6.988
6.352
1.701
OH
N
NH
O
1.93
2.01
1.98
4.03
1.00
0.84
1.06
0.92
6.05
9.0
8.5
8.0
7.5
7.0
6.5
6.0
5.5
5.0
4.5
4.0
3.5
3.0
2.5
2.0
1.5
1.0
0.5
0.0
f1 (ppm)

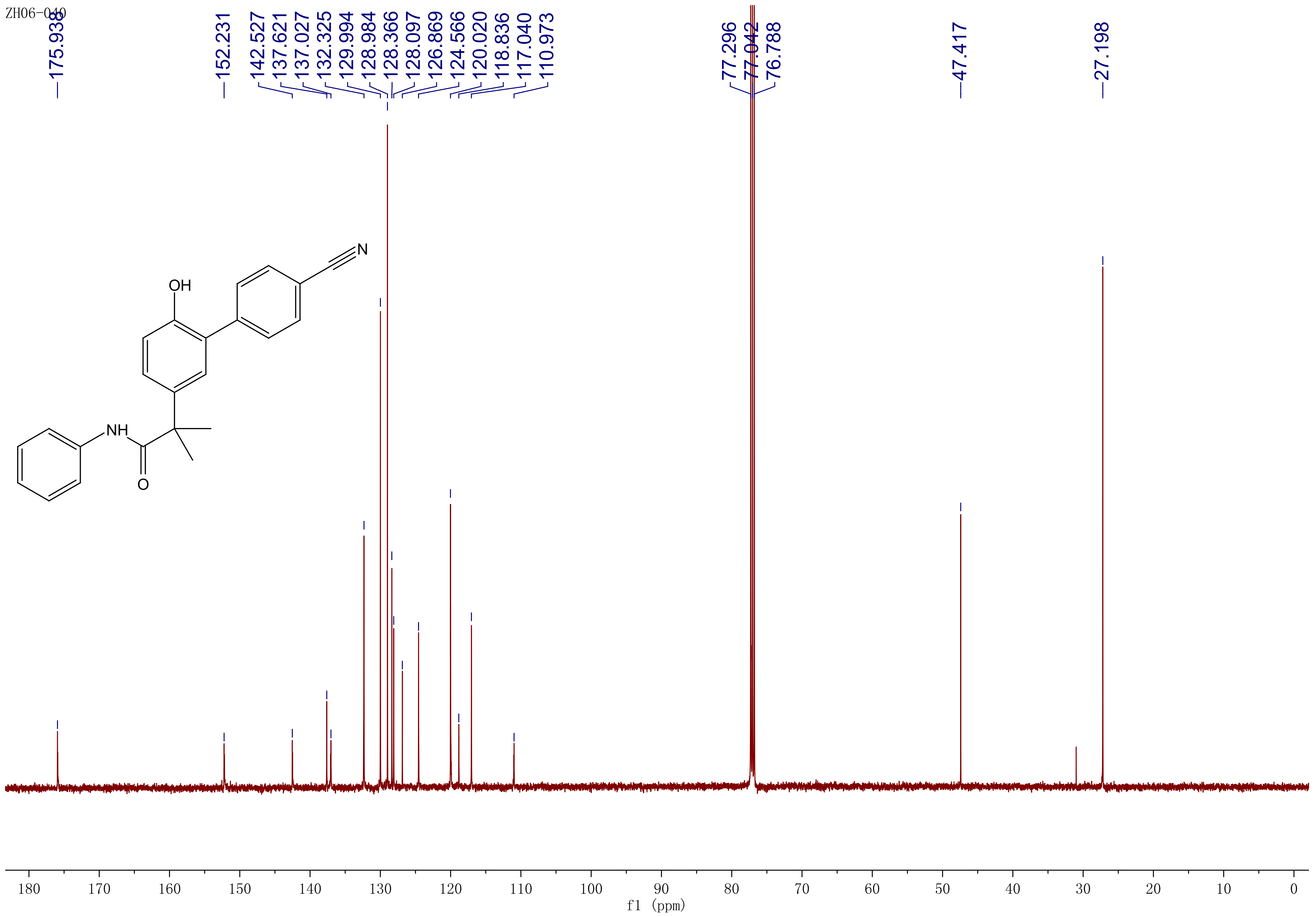
ZH06-040
175.938
152.231
142.527
137.621
137.027
132.325
129.994
128.984
128.366
128.097
126.869
124.566
120.020
118.836
117.040
110.973
77.296
77.042
76.788
47.417
27.198
OH
N
NH
O
180 170 160 150 140 130 120 110 100 90 80 70 60 50 40 30 20 10 0
f1 (ppm)

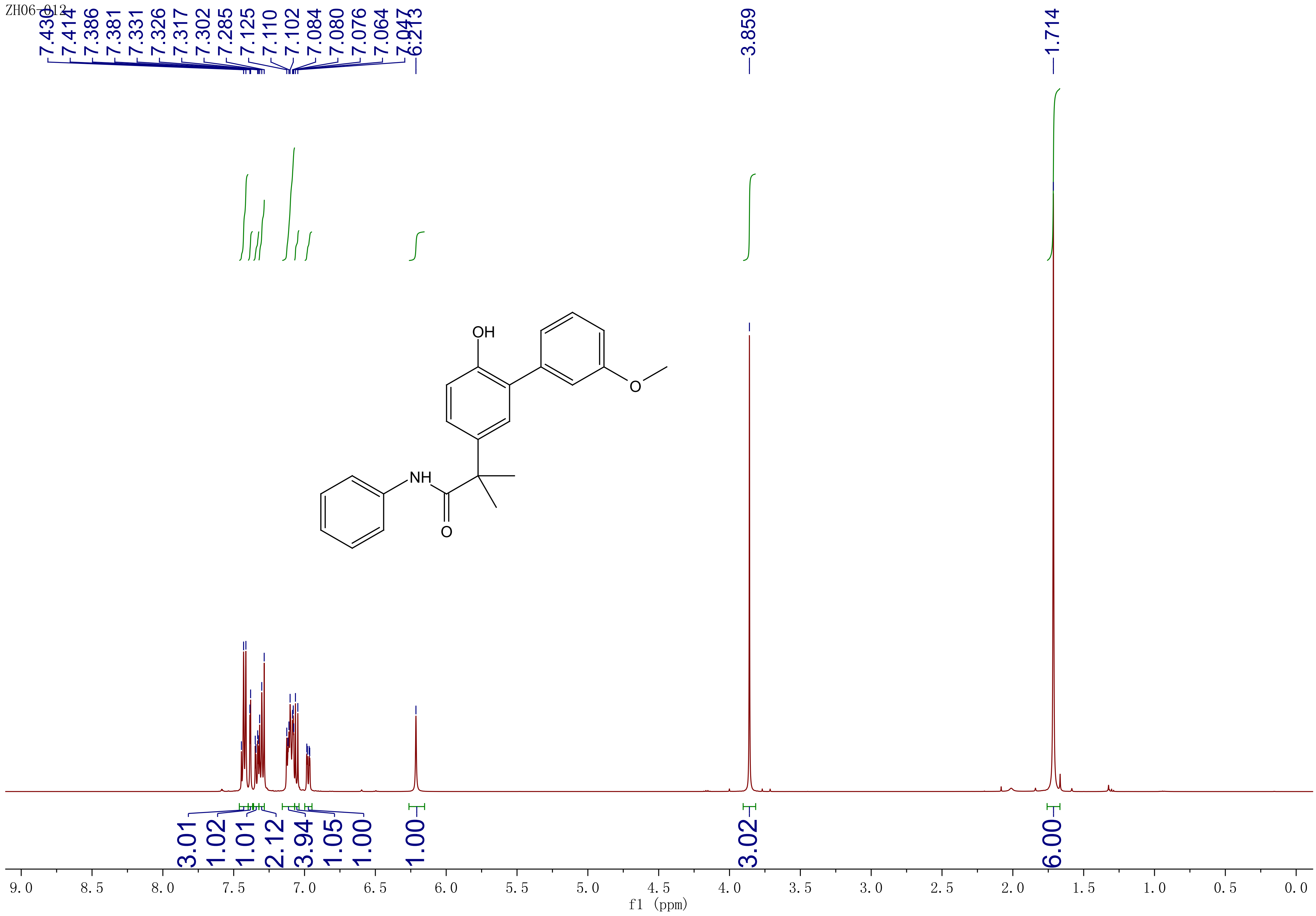

ZH06-012
7.430
7.414
7.386
7.381
7.331
7.326
7.317
7.302
7.285
7.125
7.110
7.102
7.084
7.080
7.076
7.064
7.047
6.213
3.859
1.714
OH
O
NH
O
3.01
1.02
1.01
2.12
3.94
1.05
1.00
1.00
3.02
6.00
9.0
8.5
8.0
7.5
7.0
6.5
6.0
5.5
5.0
4.5
4.0
3.5
3.0
2.5
2.0
1.5
1.0
0.5
0.0
f1 (ppm)

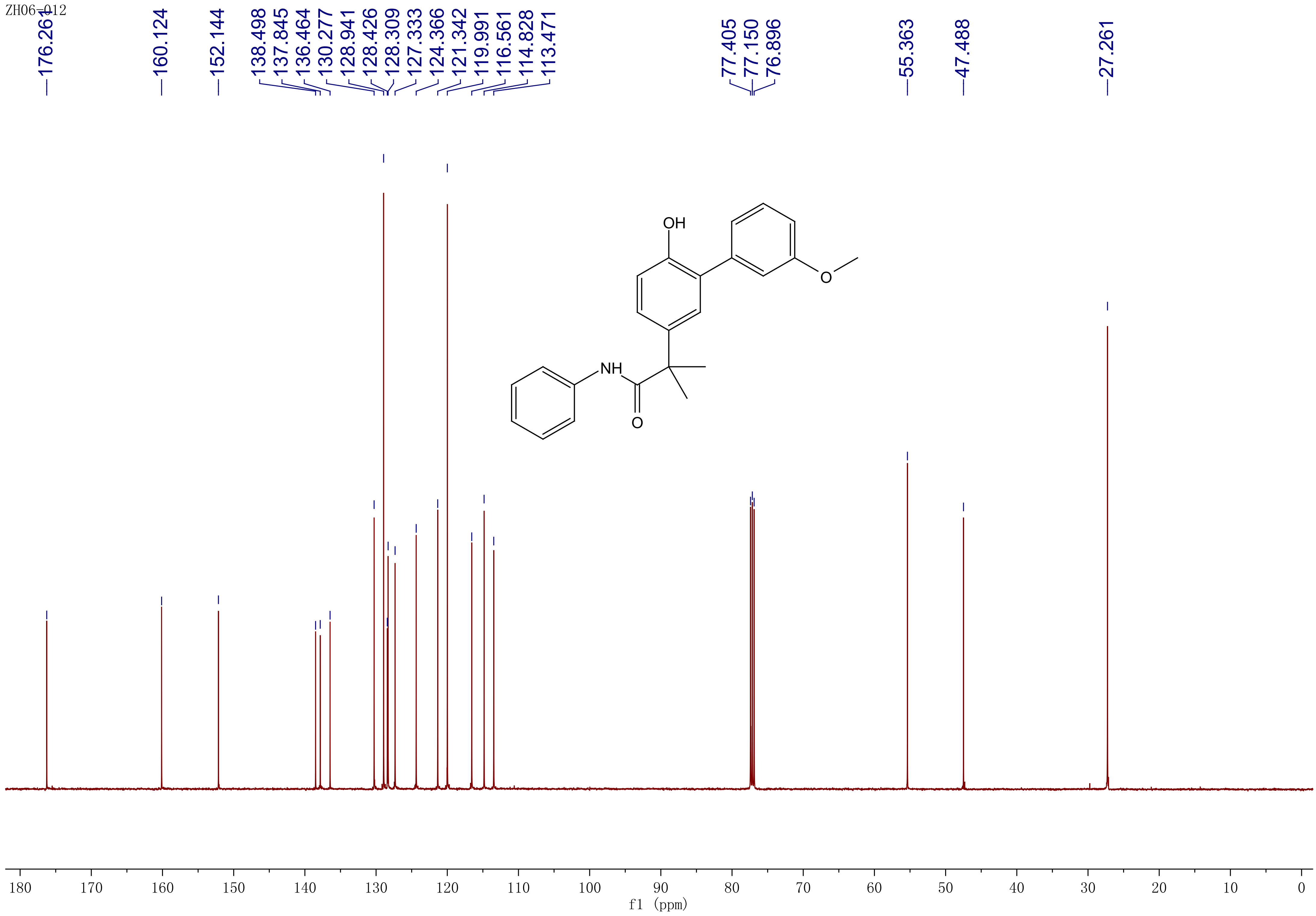
ZH06-012
176.261
160.124
152.144
138.498
137.845
136.464
130.277
128.941
128.426
128.309
127.333
124.366
121.342
119.991
116.561
114.828
113.471
77.405
77.150
76.896
55.363
47.488
27.261
OH
O
NH
O
f1 (ppm)

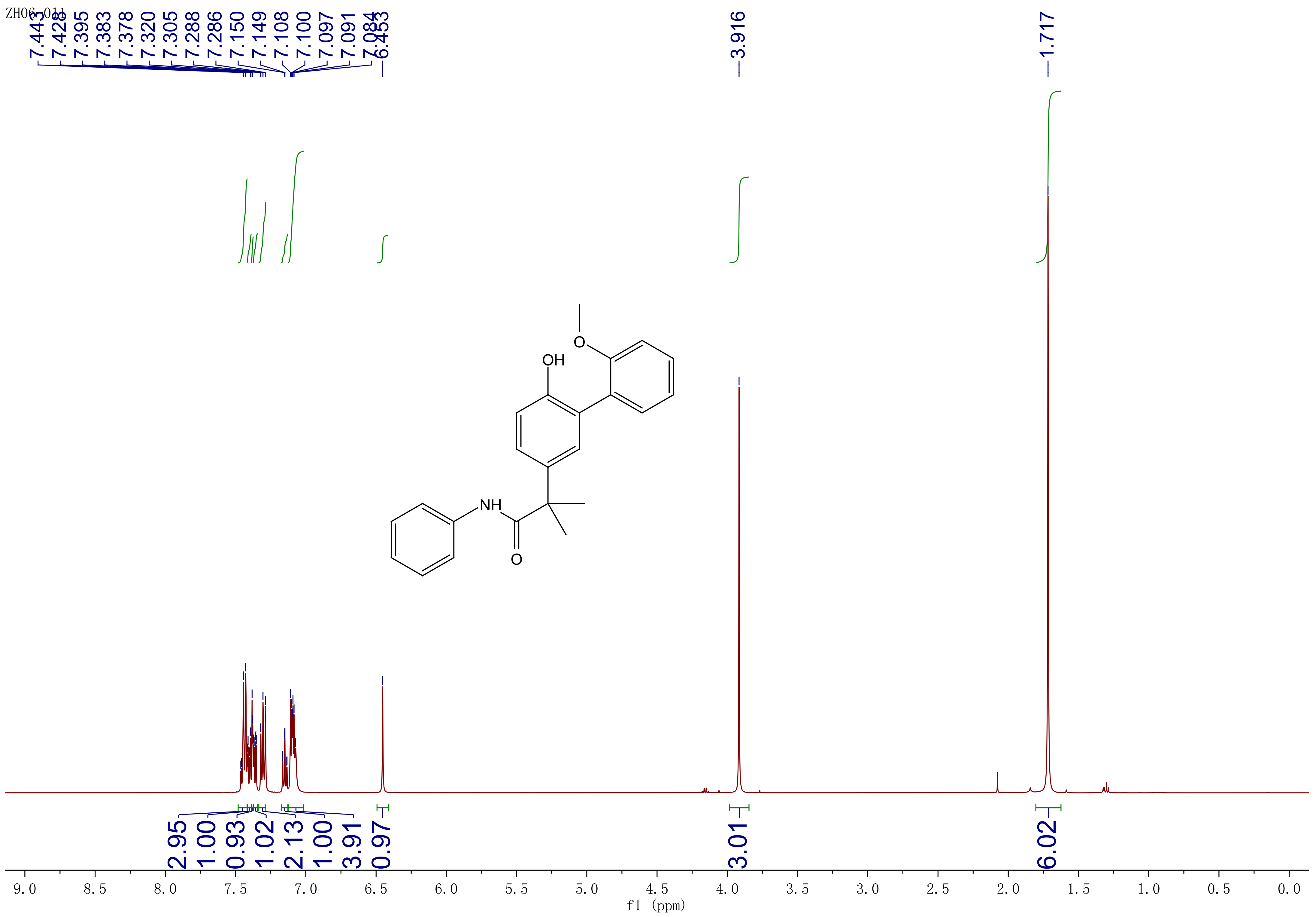

ZH06-011
7.443
7.428
7.395
7.383
7.378
7.320
7.305
7.288
7.286
7.150
7.149
7.108
7.100
7.097
7.091
7.084
6.453
3.916
1.717
OH
O
NH
O
2.95
1.00
0.93
1.02
2.13
1.00
3.91
0.97
3.01
6.02
9.0
8.5
8.0
7.5
7.0
6.5
6.0
5.5
5.0
4.5
4.0
3.5
3.0
2.5
2.0
1.5
1.0
0.5
0.0
f1 (ppm)

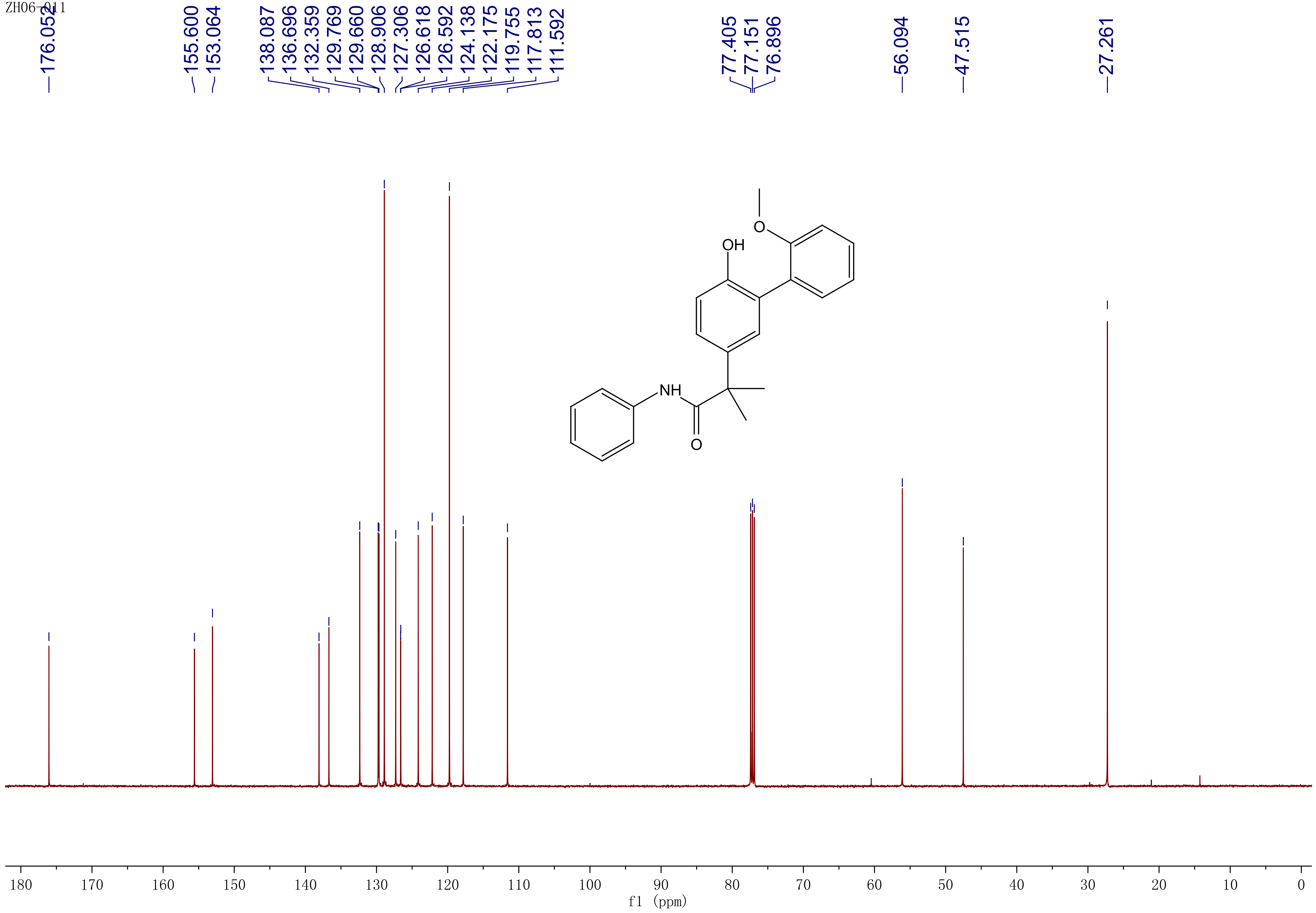
ZH06-011
176.052
155.600
153.064
138.087
136.696
132.359
129.769
129.660
128.906
127.306
126.618
126.592
124.138
122.175
119.755
117.813
111.592
77.405
77.151
76.896
56.094
47.515
27.261
OH
O
NH
O
180 170 160 150 140 130 120 110 100 90 80 70 60 50 40 30 20 10 0
f1 (ppm)

ZH06-038

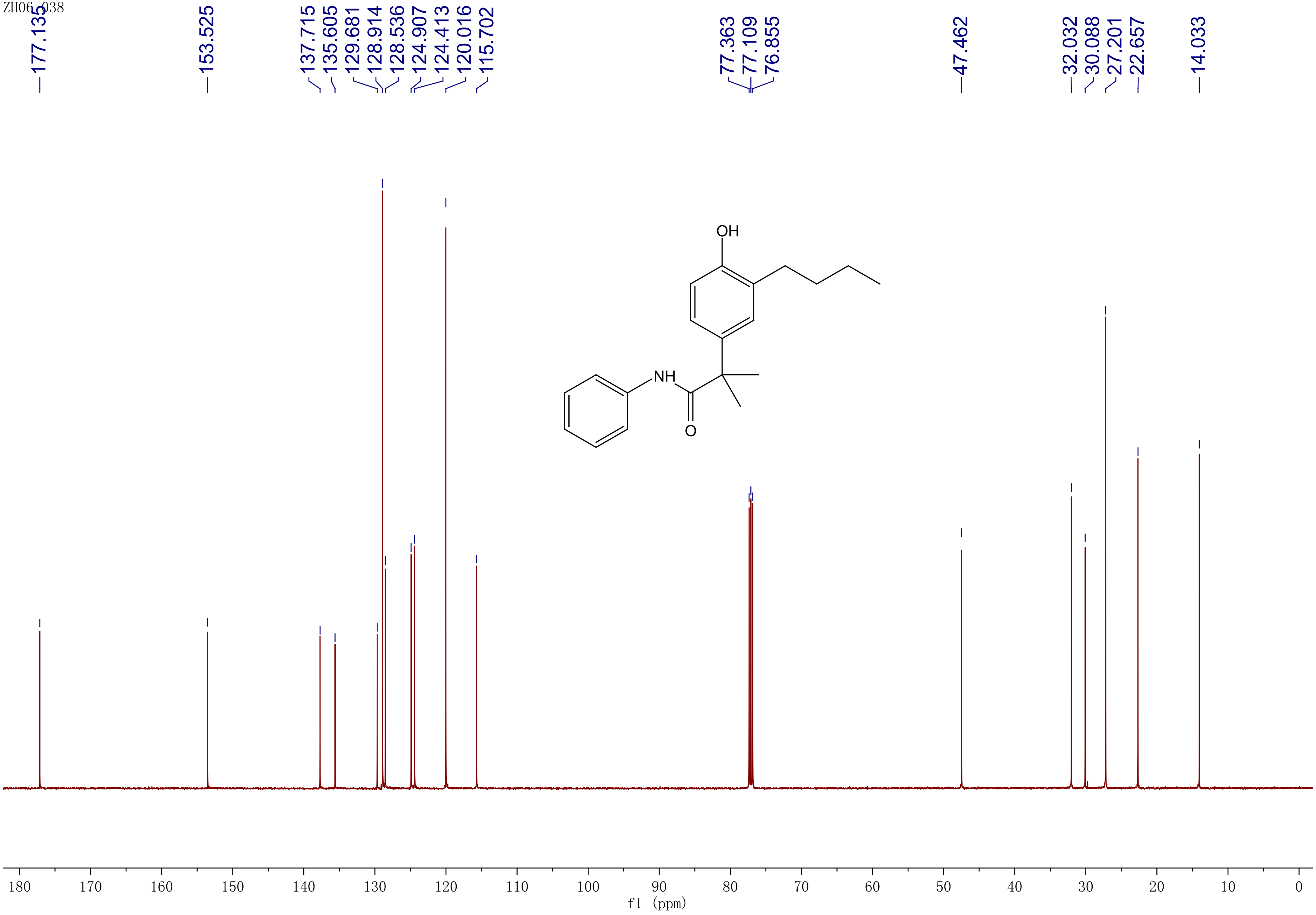
177.135
153.525
137.715
135.605
129.681
128.914
128.536
124.907
124.413
120.016
115.702
77.363
77.109
76.855
47.462
32.032
30.088
27.201
22.657
14.033
OH
NH
O
180 170 160 150 140 130 120 110 100 90 80 70 60 50 40 30 20 10 0
f1 (ppm)

ZH06-038

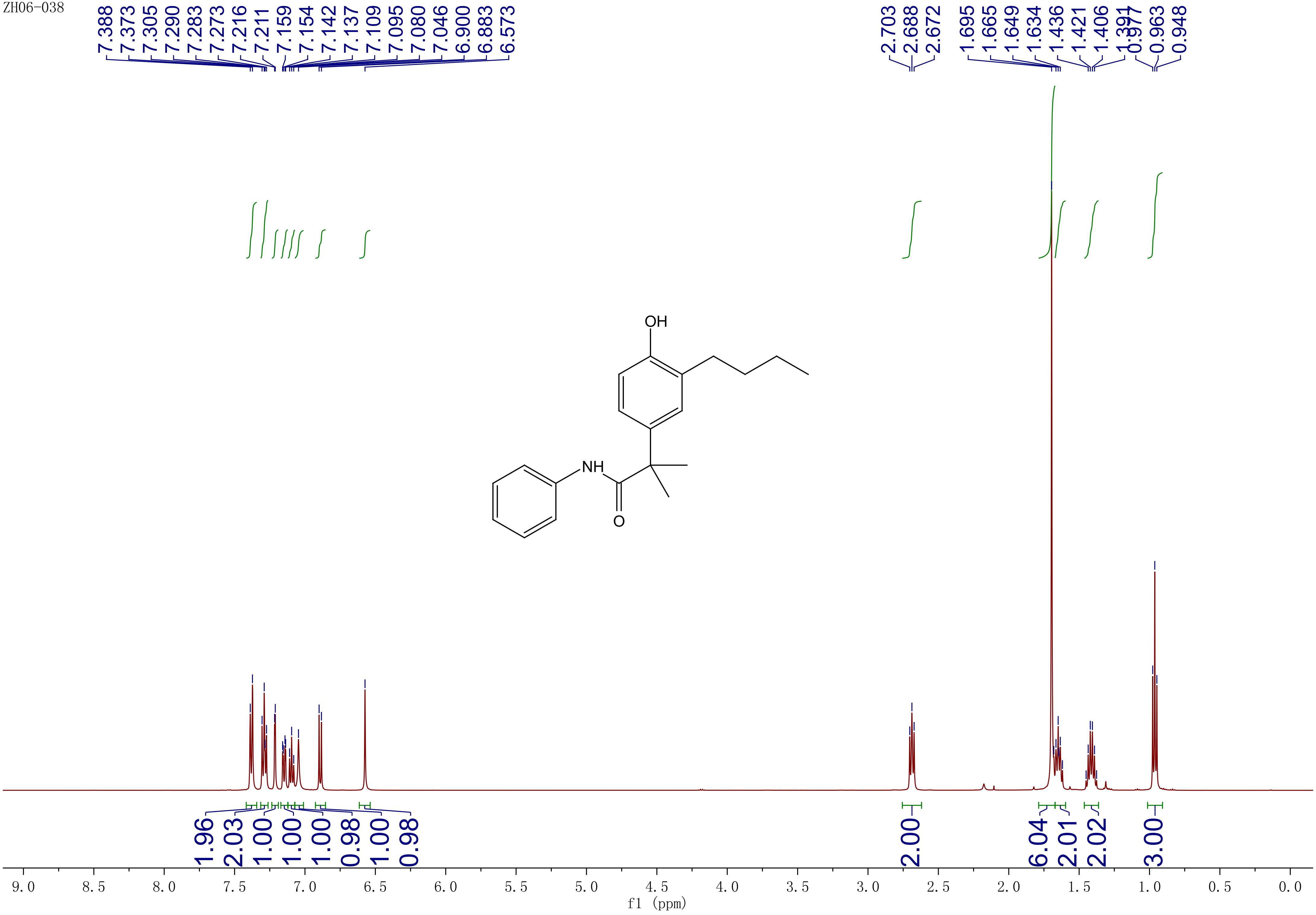

7.388
7.373
7.305
7.290
7.283
7.273
7.216
7.211
7.159
7.154
7.142
7.137
7.109
7.095
7.080
7.046
6.900
6.883
6.573
2.703
2.688
2.672
1.695
1.665
1.649
1.634
1.436
1.421
1.406
1.391
0.977
0.963
0.948
OH
NH
O
1.96
2.03
1.00
1.00
1.00
0.98
1.00
0.98
2.00
6.04
2.01
2.02
3.00
9.0
8.5
8.0
7.5
7.0
6.5
6.0
5.5
5.0
4.5
4.0
3.5
3.0
2.5
2.0
1.5
1.0
0.5
0.0
f1 (ppm)

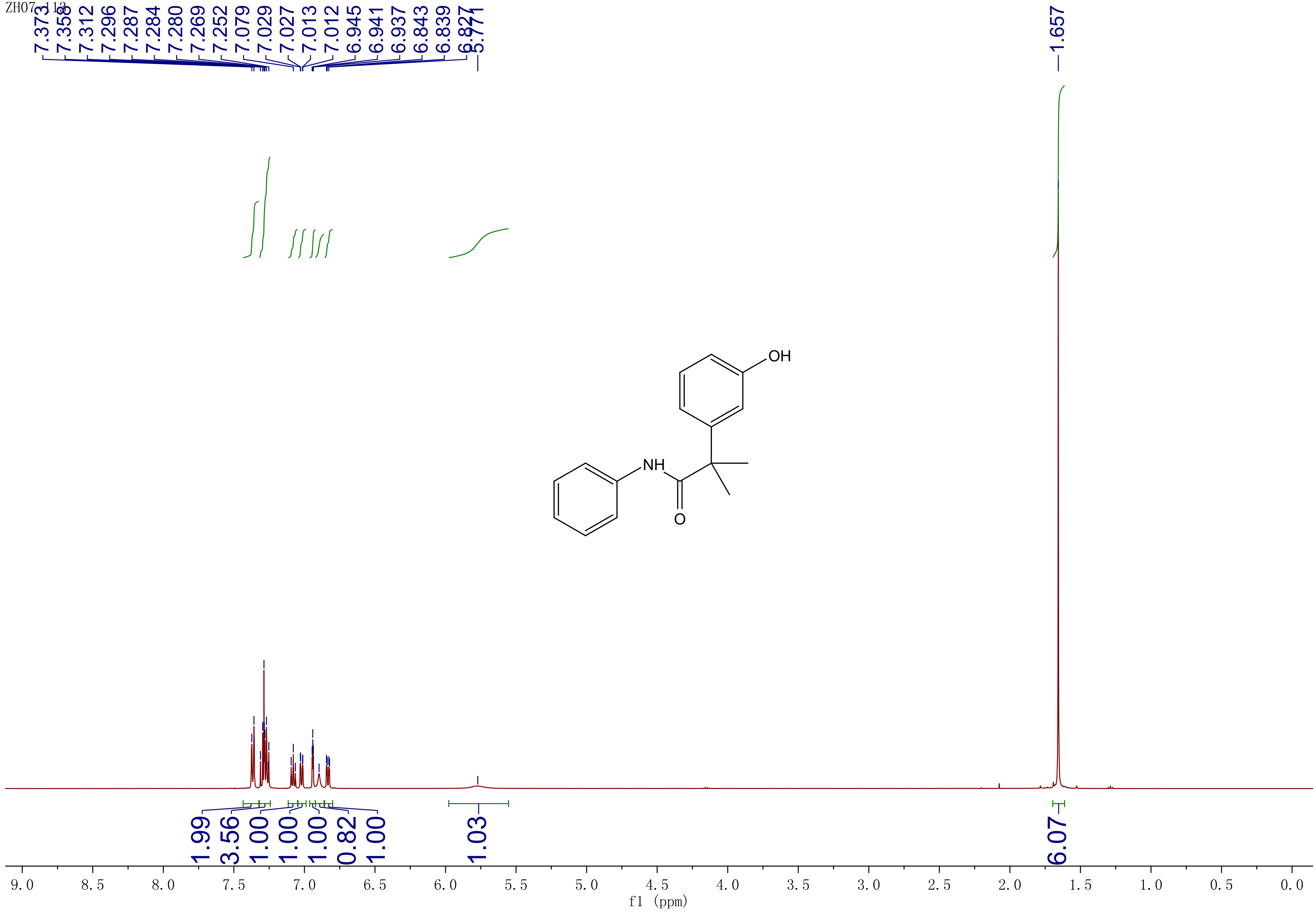
ZH07-113
7.373
7.358
7.312
7.296
7.287
7.284
7.280
7.269
7.252
7.079
7.029
7.027
7.013
7.012
6.945
6.941
6.937
6.843
6.839
6.827
5.771
1.657
OH
NH
O
1.99
3.56
1.00
1.00
1.00
0.82
1.00
1.03
6.07
9.0 8.5 8.0 7.5 7.0 6.5 6.0 5.5 5.0 4.5 4.0 3.5 3.0 2.5 2.0 1.5 1.0 0.5 0.0
f1 (ppm)

ZH07-113

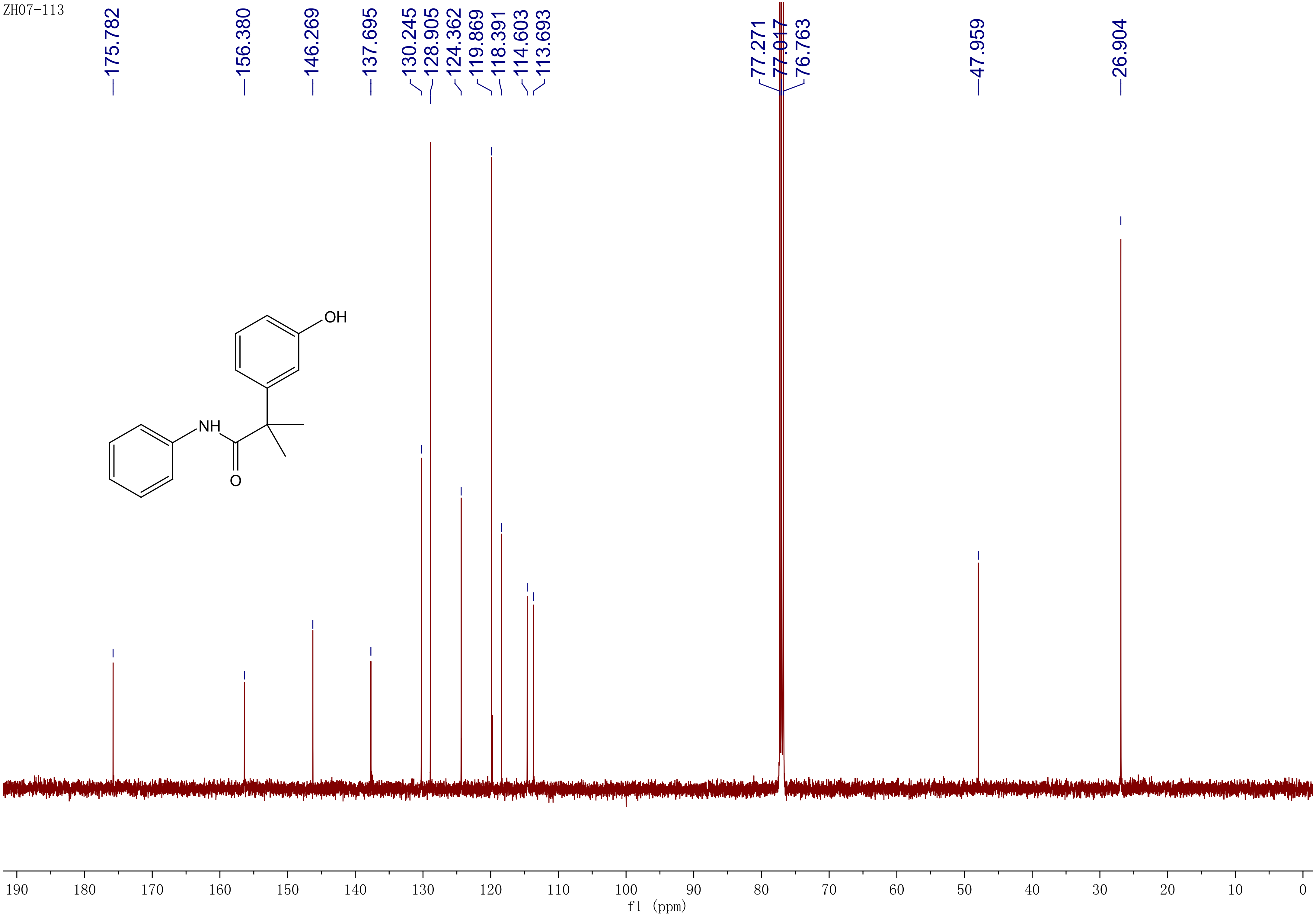
175.782
156.380
146.269
137.695
130.245
128.905
124.362
119.869
118.391
114.603
113.693
77.271
77.017
76.763
47.959
26.904
OH
NH
O
190 180 170 160 150 140 130 120 110 100 90 80 70 60 50 40 30 20 10 0
f1 (ppm)

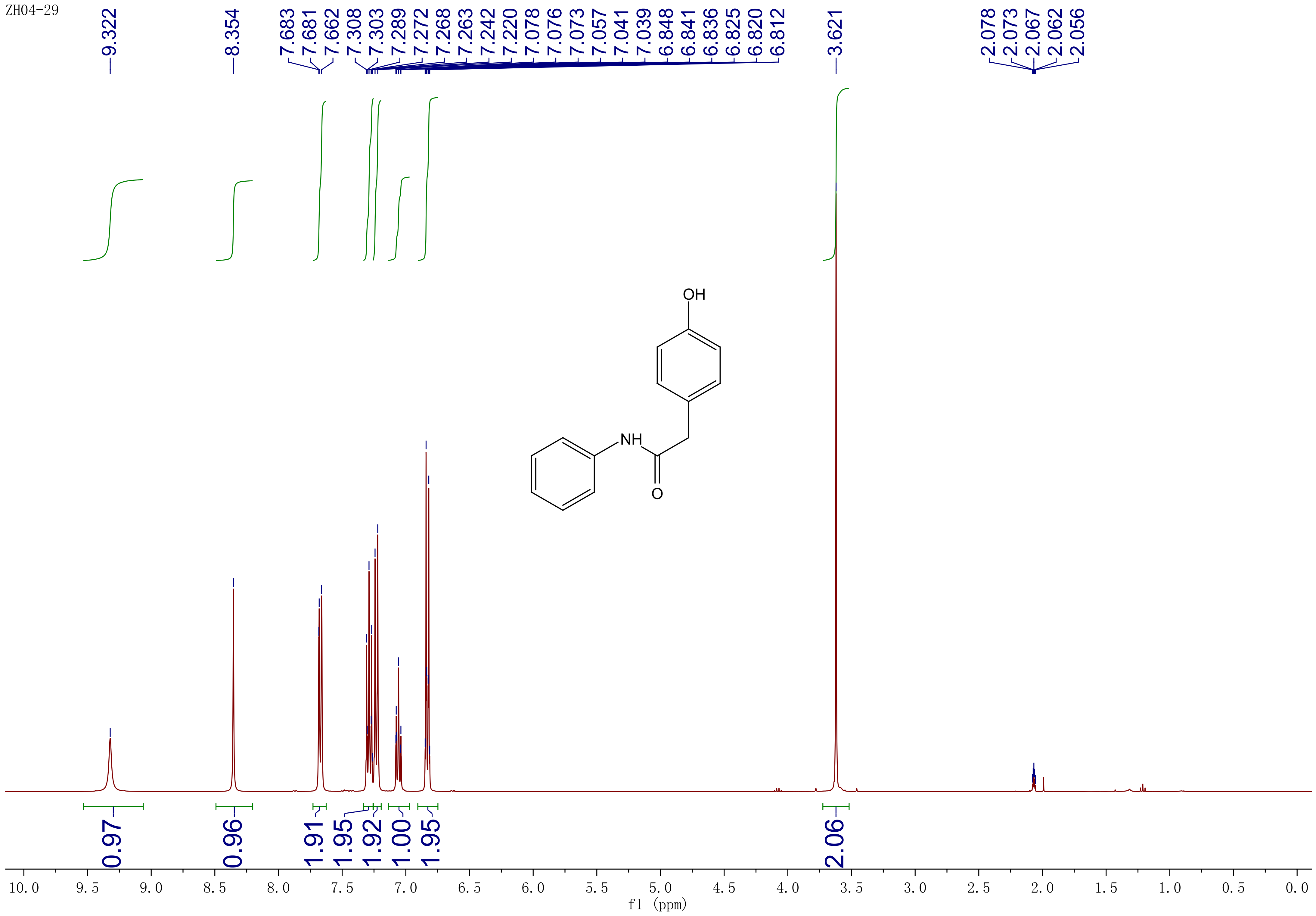
ZH04-29
9.322
8.354
7.683
7.681
7.662
7.308
7.303
7.289
7.272
7.268
7.263
7.242
7.220
7.078
7.076
7.073
7.057
7.041
7.039
6.848
6.841
6.836
6.825
6.820
6.812
3.621
2.078
2.073
2.067
2.062
2.056
OH
NH
O
0.97
0.96
1.91
1.95
1.92
1.00
1.95
2.06
10.0
9.5
9.0
8.5
8.0
7.5
7.0
6.5
6.0
5.5
5.0
4.5
4.0
3.5
3.0
2.5
2.0
1.5
1.0
0.5
0.0
f1 (ppm)

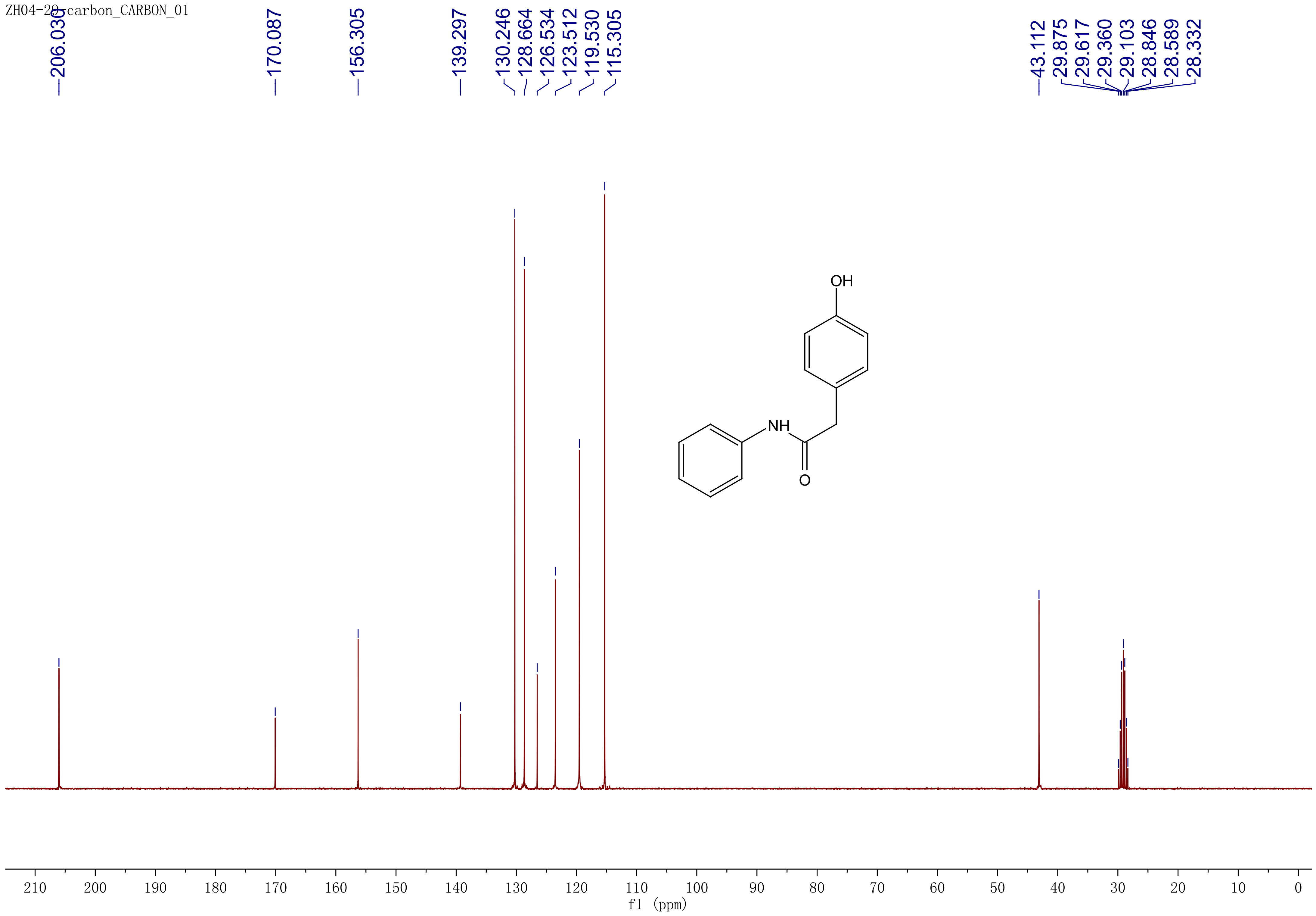
ZH04-29-carbon_CARBON_01
206.030
170.087
156.305
139.297
130.246
128.664
126.534
123.512
119.530
115.305
43.112
29.875
29.617
29.360
29.103
28.846
28.589
28.332
OH
NH
O
210 200 190 180 170 160 150 140 130 120 110 100 90 80 70 60 50 40 30 20 10 0
f1 (ppm)

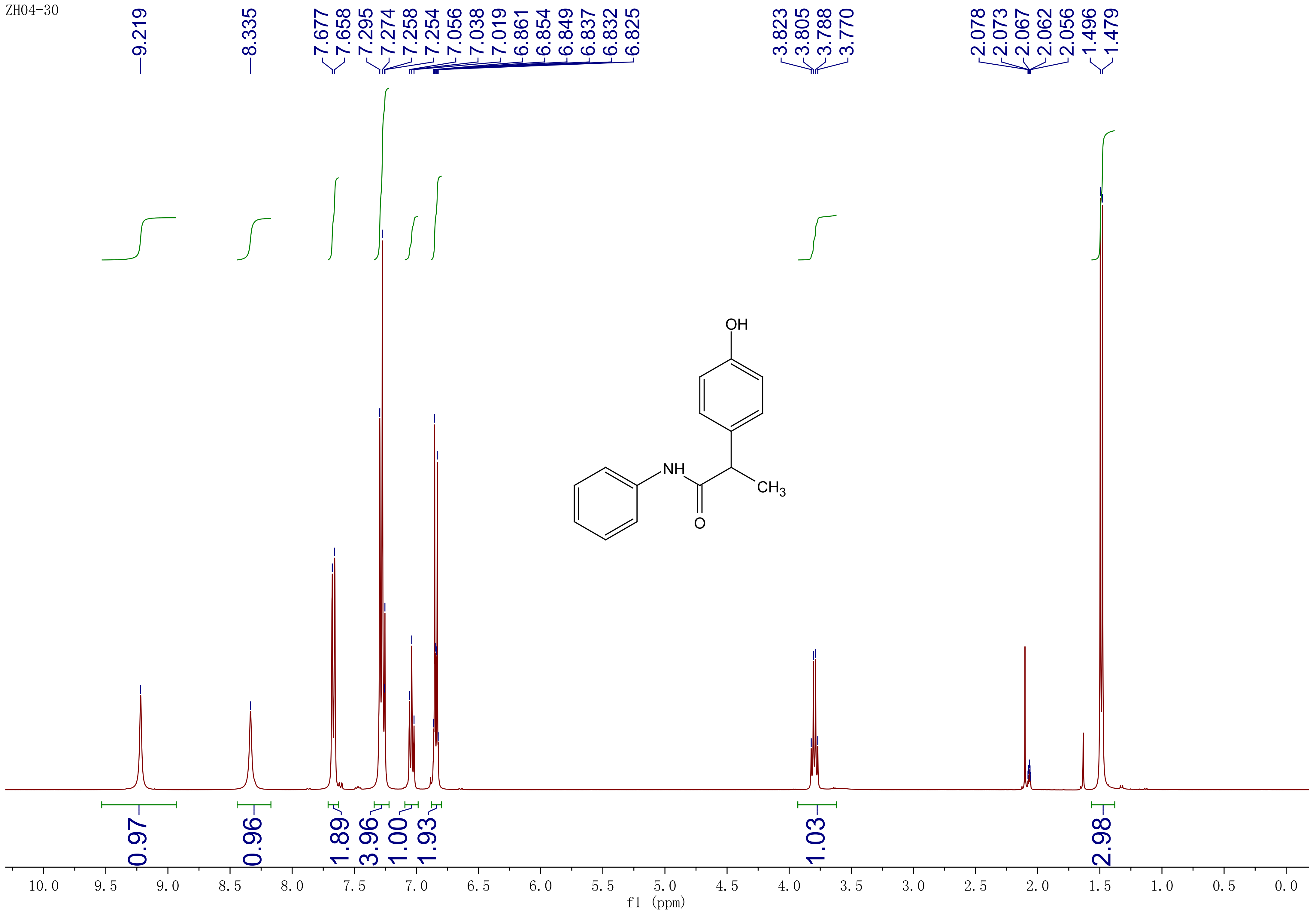

ZH04-30
9.219
8.335
7.677
7.658
7.295
7.274
7.258
7.254
7.056
7.038
7.019
6.861
6.854
6.849
6.837
6.832
6.825
3.823
3.805
3.788
3.770
2.078
2.073
2.067
2.062
2.056
1.496
1.479
OH
NH
CH3
O
0.97
0.96
1.89
3.96
1.00
1.93
1.03
2.98
10.0 9.5 9.0 8.5 8.0 7.5 7.0 6.5 6.0 5.5 5.0 4.5 4.0 3.5 3.0 2.5 2.0 1.5 1.0 0.5 0.0
f1 (ppm)

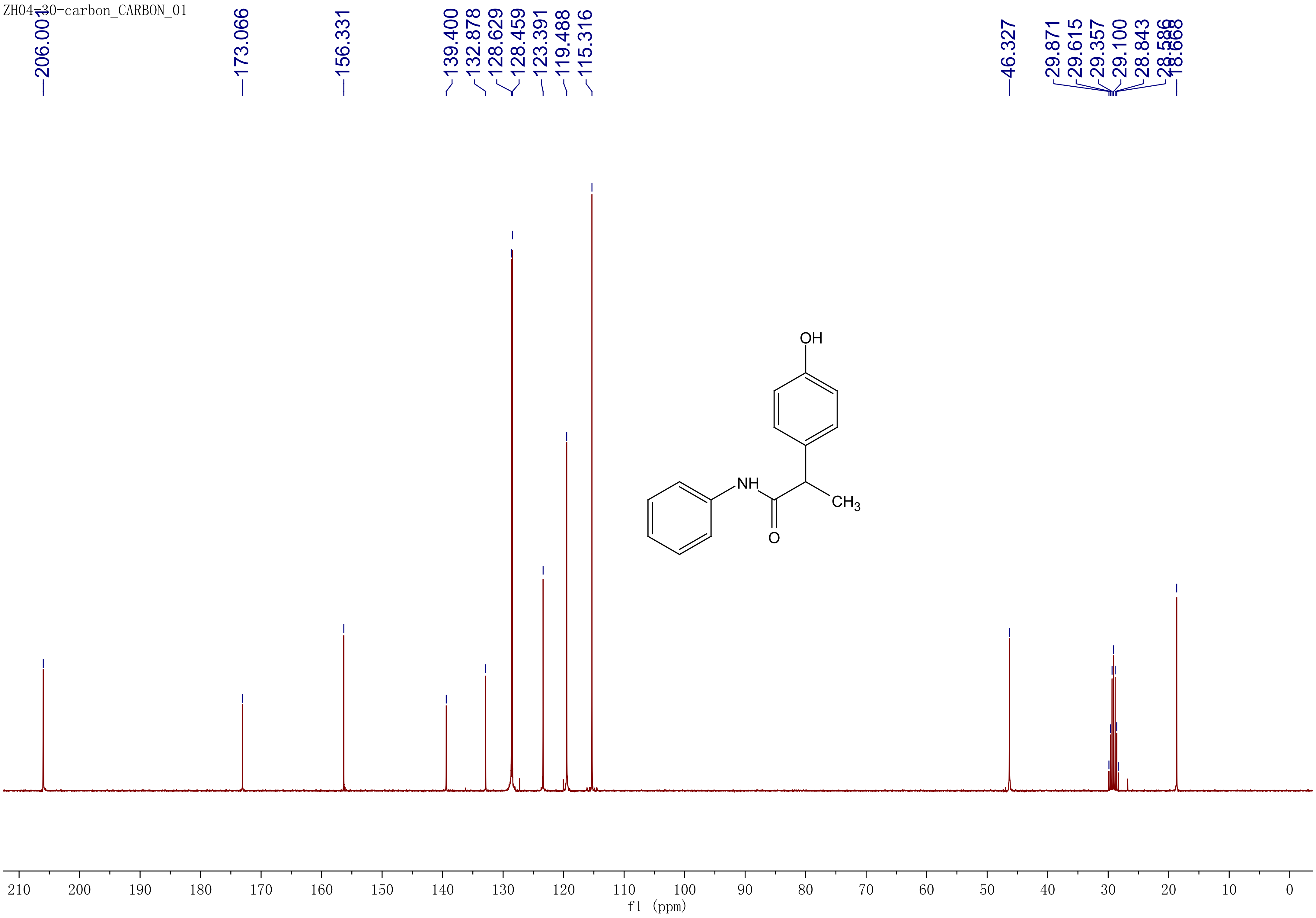
ZH04-30-carbon_CARBON_01
206.001
173.066
156.331
139.400
132.878
128.629
128.459
123.391
119.488
115.316
46.327
29.871
29.615
29.357
29.100
28.843
OH
NH
CH3
O
210
200
190
180
170
160
150
140
130
120
110
100
90
80
70
60
50
40
30
20
10
0
f1 (ppm)

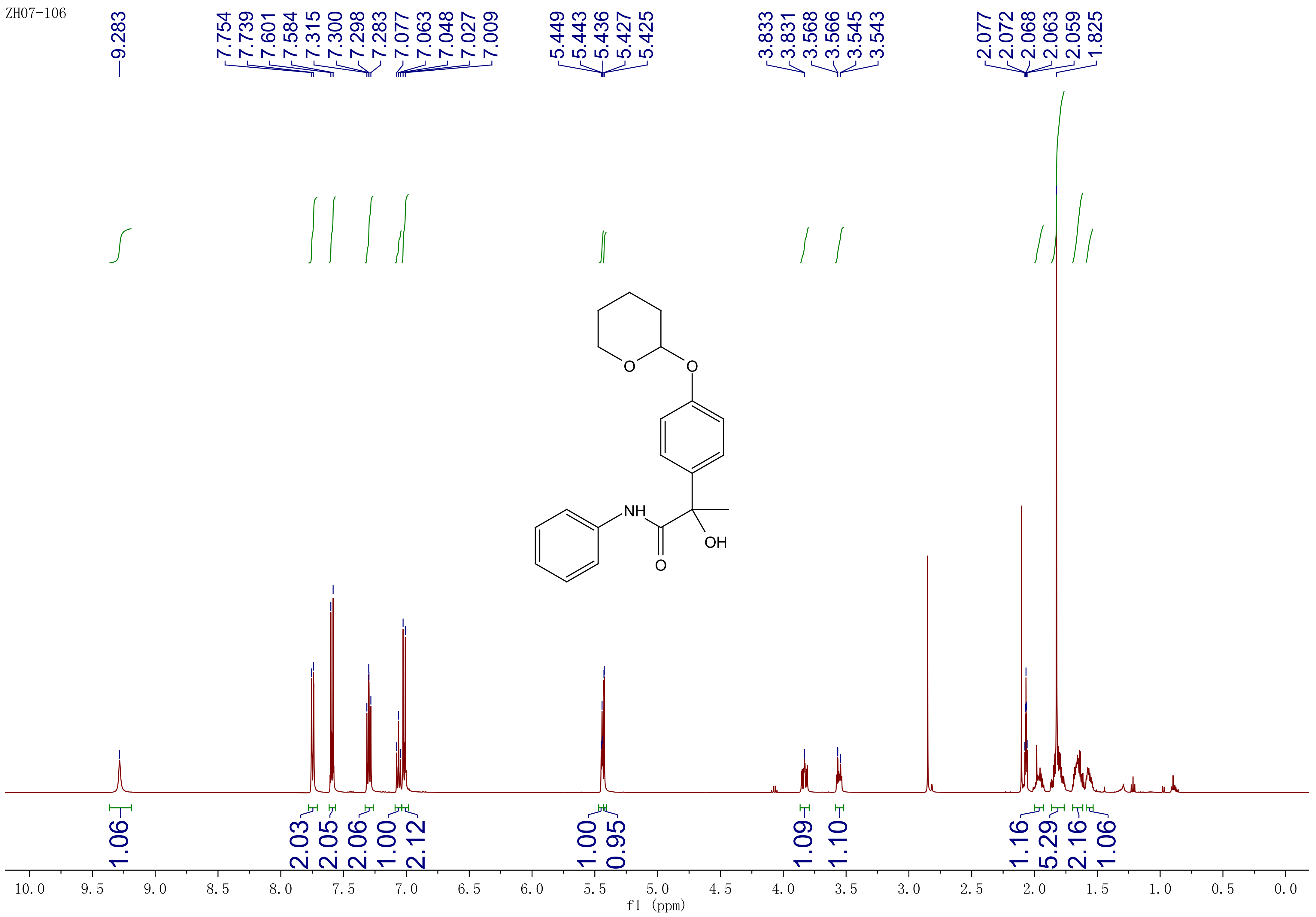
ZH07-106
9.283
7.754
7.739
7.601
7.584
7.315
7.300
7.298
7.283
7.077
7.063
7.048
7.027
7.009
5.449
5.443
5.436
5.427
5.425
3.833
3.831
3.568
3.566
3.545
3.543
2.077
2.072
2.068
2.063
2.059
1.825
O
O
NH
OH
O
1.06
2.03
2.05
2.06
1.00
2.12
1.00
0.95
1.09
1.10
1.16
5.29
2.16
1.06
10.0 9.5 9.0 8.5 8.0 7.5 7.0 6.5 6.0 5.5 5.0 4.5 4.0 3.5 3.0 2.5 2.0 1.5 1.0 0.5 0.0
f1 (ppm)

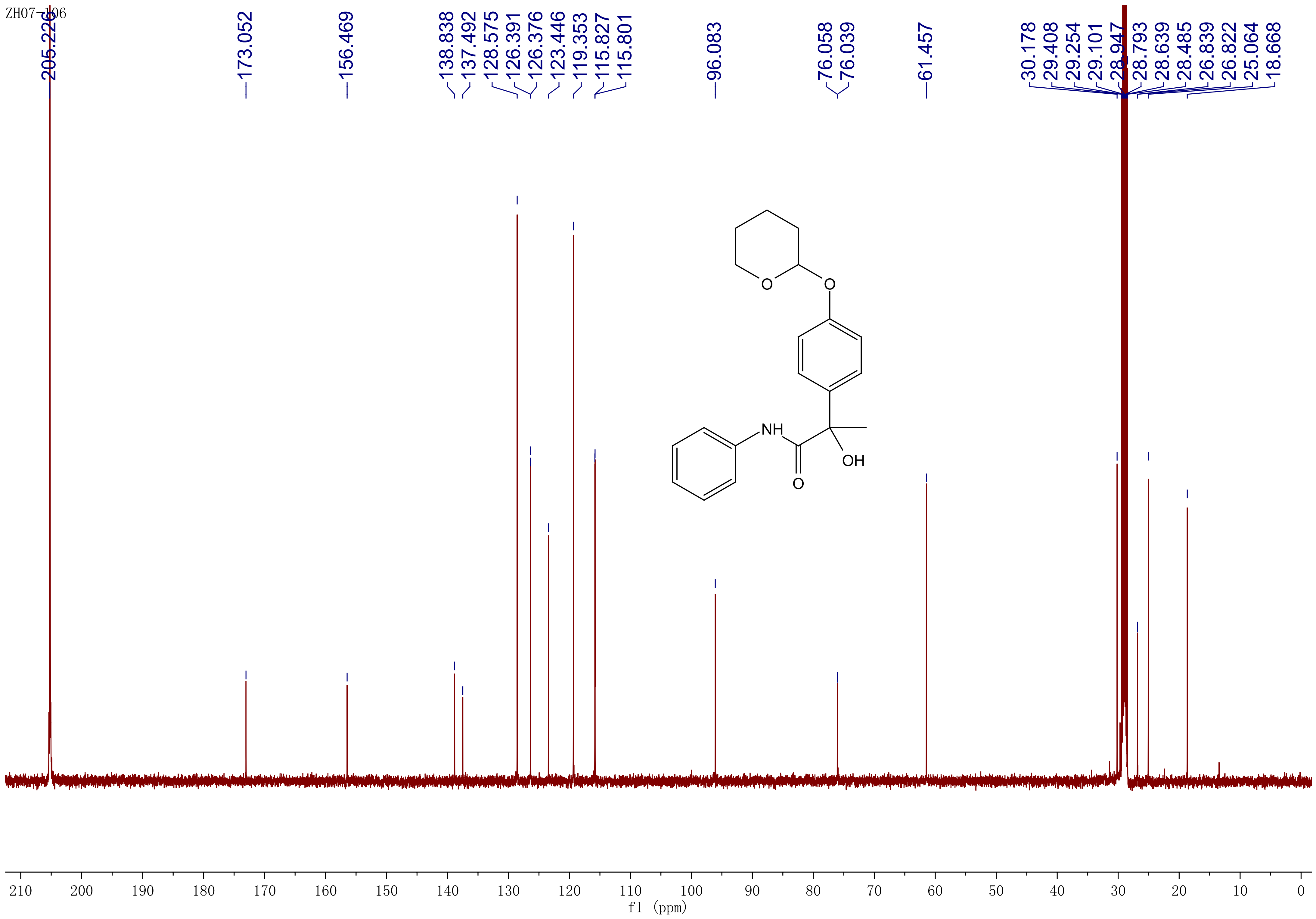
ZH07-106
205.226
173.052
156.469
138.838
137.492
128.575
126.391
126.376
123.446
119.353
115.827
115.801
96.083
76.058
76.039
61.457
30.178
29.408
29.254
29.101
28.947
28.793
28.639
28.485
26.839
26.822
25.064
18.668
NH
O
O
O
OH
f1 (ppm)

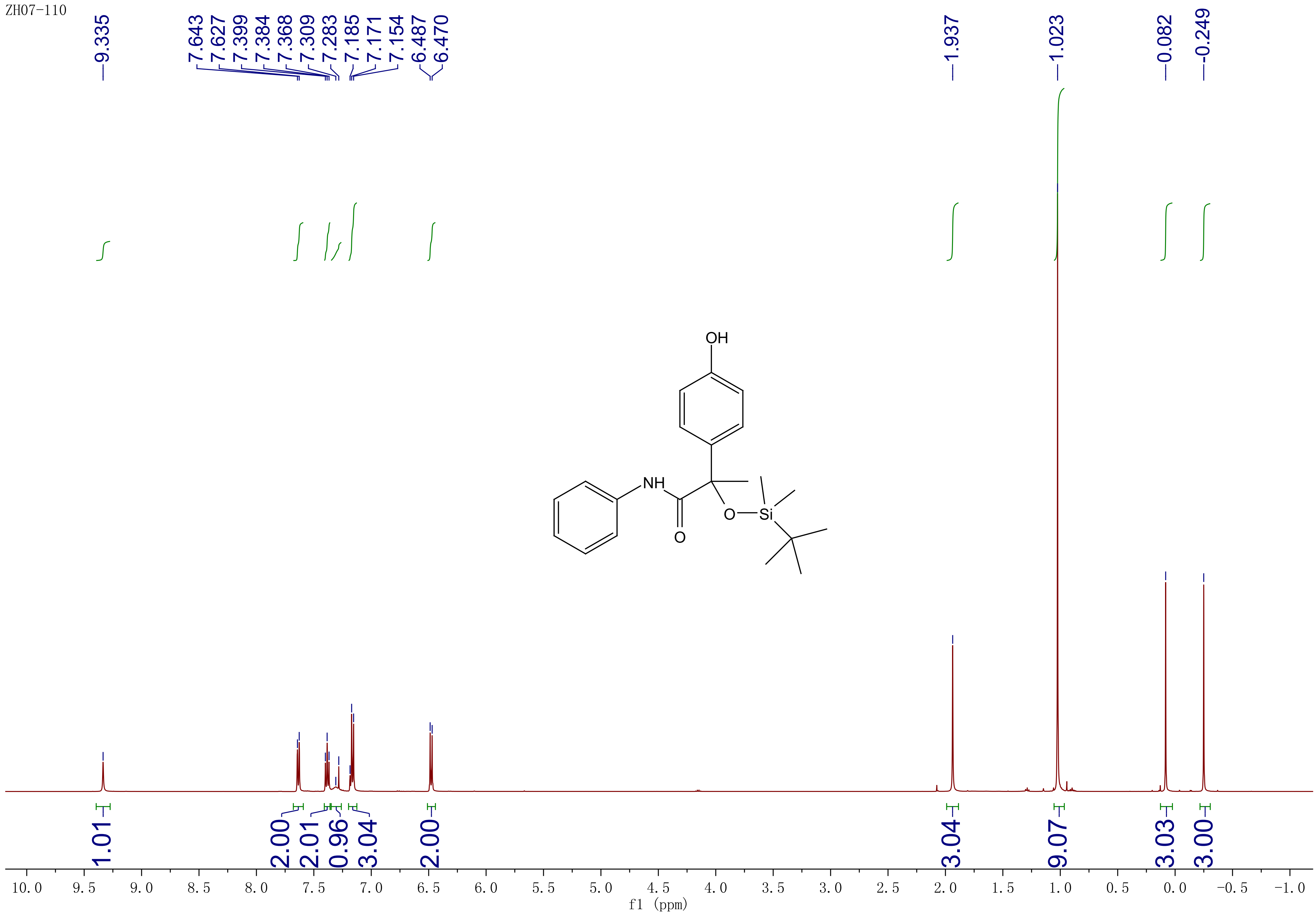
ZH07-110
9.335
7.643
7.627
7.399
7.384
7.368
7.309
7.283
7.185
7.171
7.154
6.487
6.470
1.937
1.023
0.082
-0.249
OH
NH
O
O
Si
1.01
2.00
2.01
0.96
3.04
2.00
3.04
9.07
3.03
3.00
10.0 9.5 9.0 8.5 8.0 7.5 7.0 6.5 6.0 5.5 5.0 4.5 4.0 3.5 3.0 2.5 2.0 1.5 1.0 0.5 0.0 -0.5 -1.0
f1 (ppm)

ZH07-110

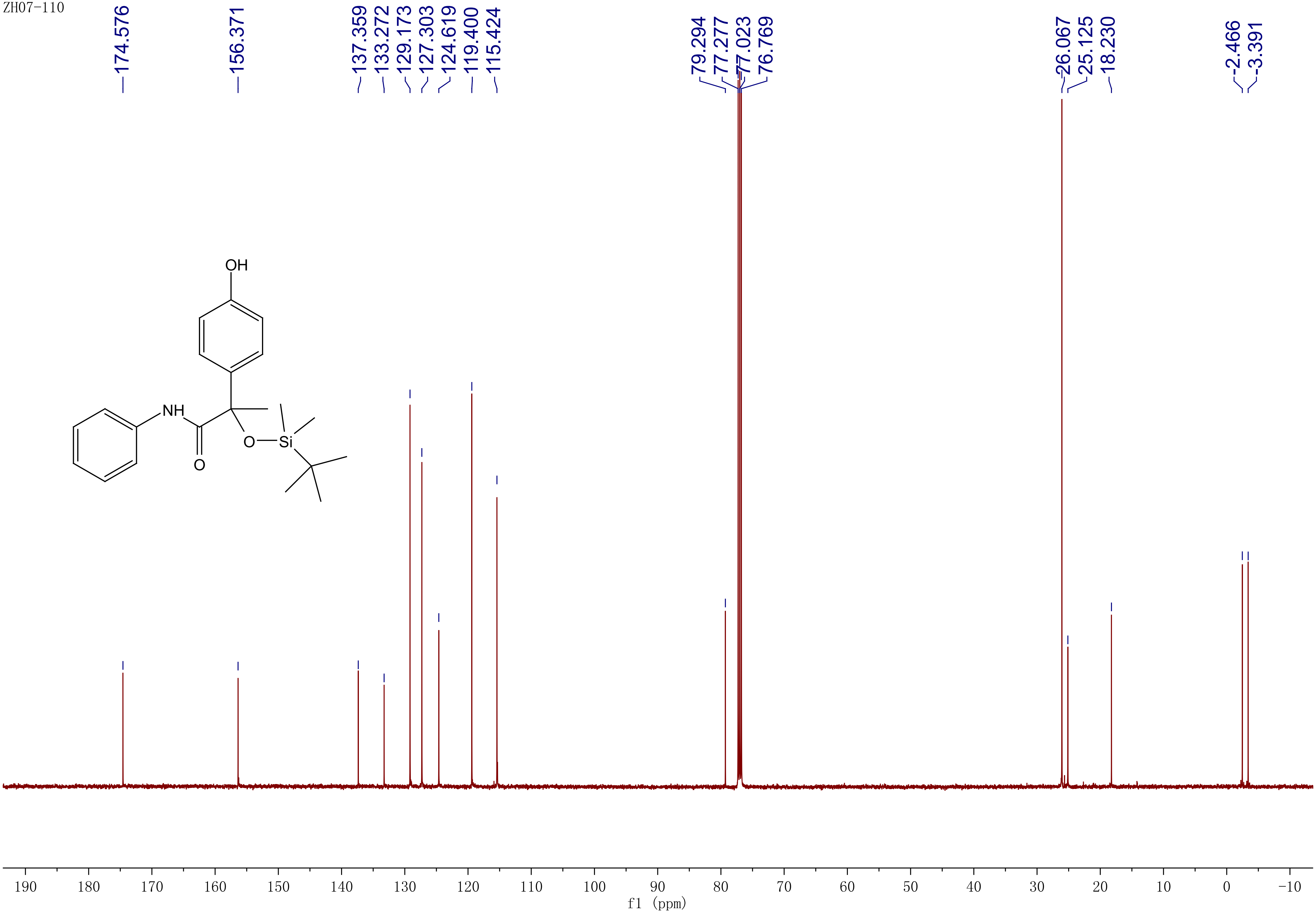
174.576
156.371
137.359
133.272
129.173
127.303
124.619
119.400
115.424
79.294
77.277
77.023
76.769
26.067
25.125
18.230
-2.466
-3.391
OH
NH
O
Si
O
190 180 170 160 150 140 130 120 110 100 90 80 70 60 50 40 30 20 10 0 −10
f1 (ppm)

ZH06-112-top

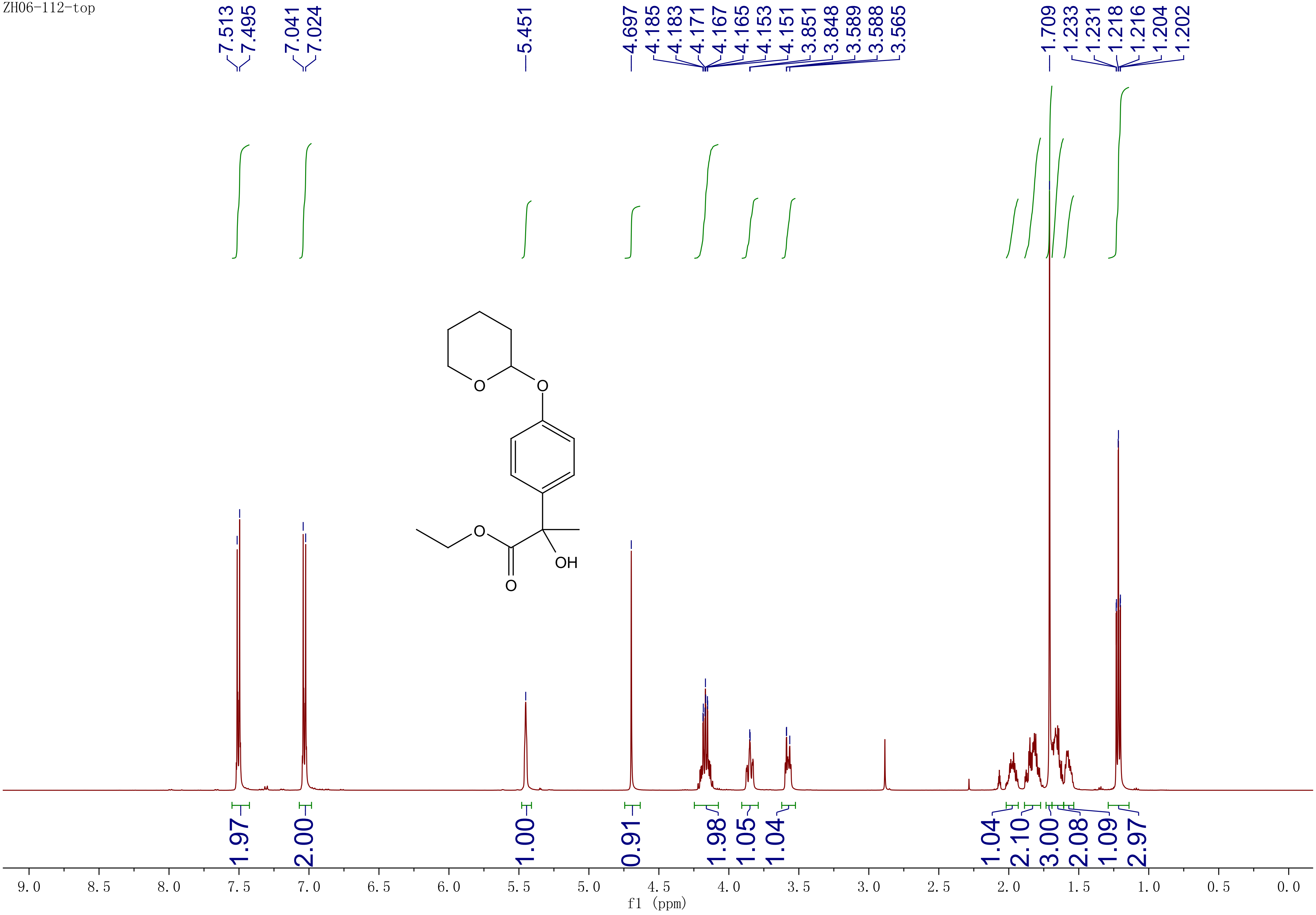
7.513
7.495
7.041
7.024
5.451
4.697
4.185
4.183
4.171
4.167
4.165
4.153
4.151
3.851
3.848
3.589
3.588
3.565
1.709
1.233
1.231
1.218
1.216
1.204
1.202
O
O
O
O
OH
O
1.97
2.00
1.00
0.91
1.98
1.05
1.04
1.04
2.10
3.00
2.08
1.09
2.97
9.0
8.5
8.0
7.5
7.0
6.5
6.0
5.5
5.0
4.5
4.0
3.5
3.0
2.5
2.0
1.5
1.0
0.5
0.0
f1 (ppm)

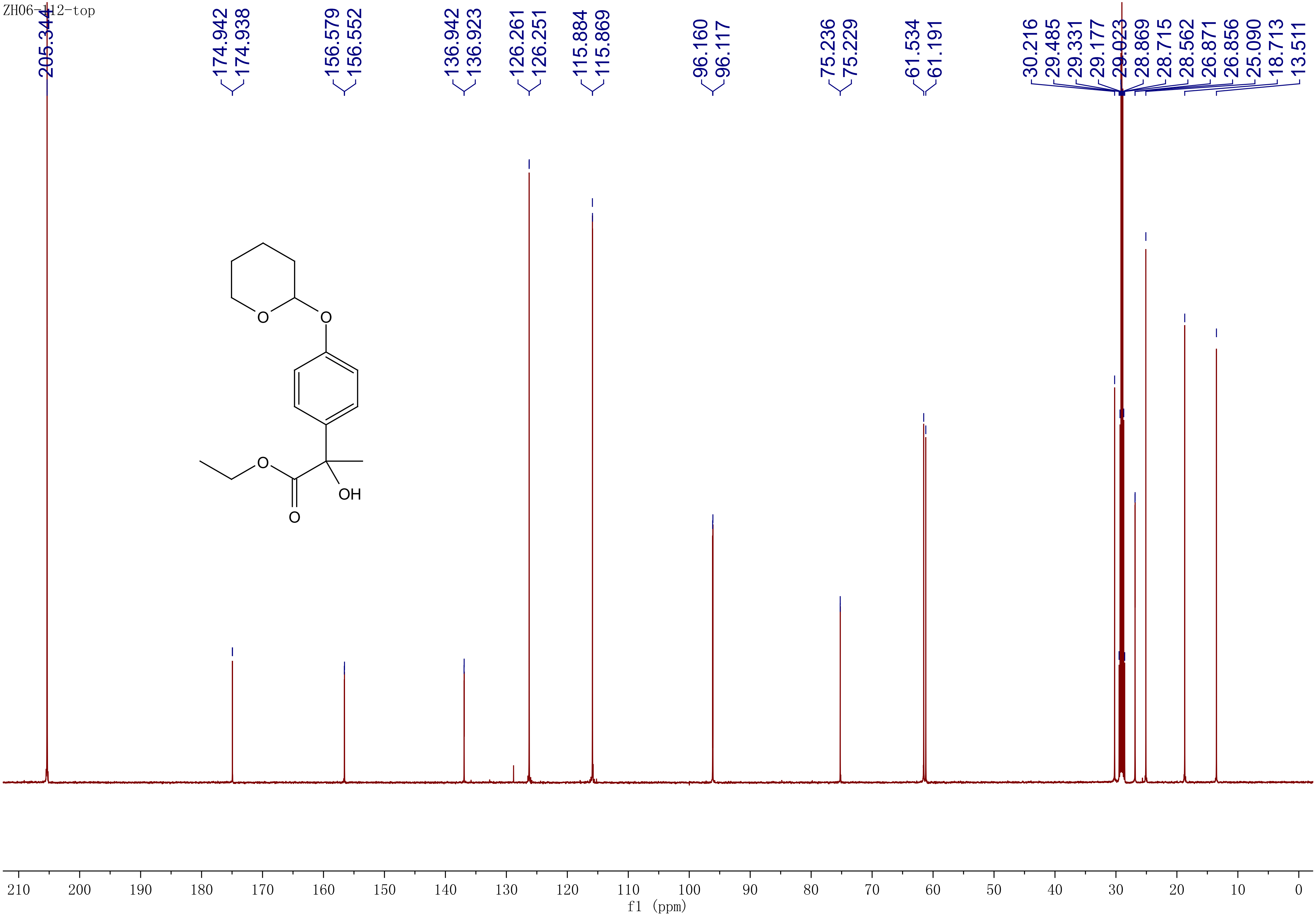
ZH06-112-top
205.344
174.942
174.938
156.579
156.552
136.942
136.923
126.261
126.251
115.884
115.869
96.160
96.117
75.236
75.229
61.534
61.191
30.216
29.485
29.331
29.177
29.023
28.869
28.715
28.562
26.871
26.856
25.090
18.713
13.511
O
O
O
O
OH
210 200 190 180 170 160 150 140 130 120 110 100 90 80 70 60 50 40 30 20 10 0
f1 (ppm)

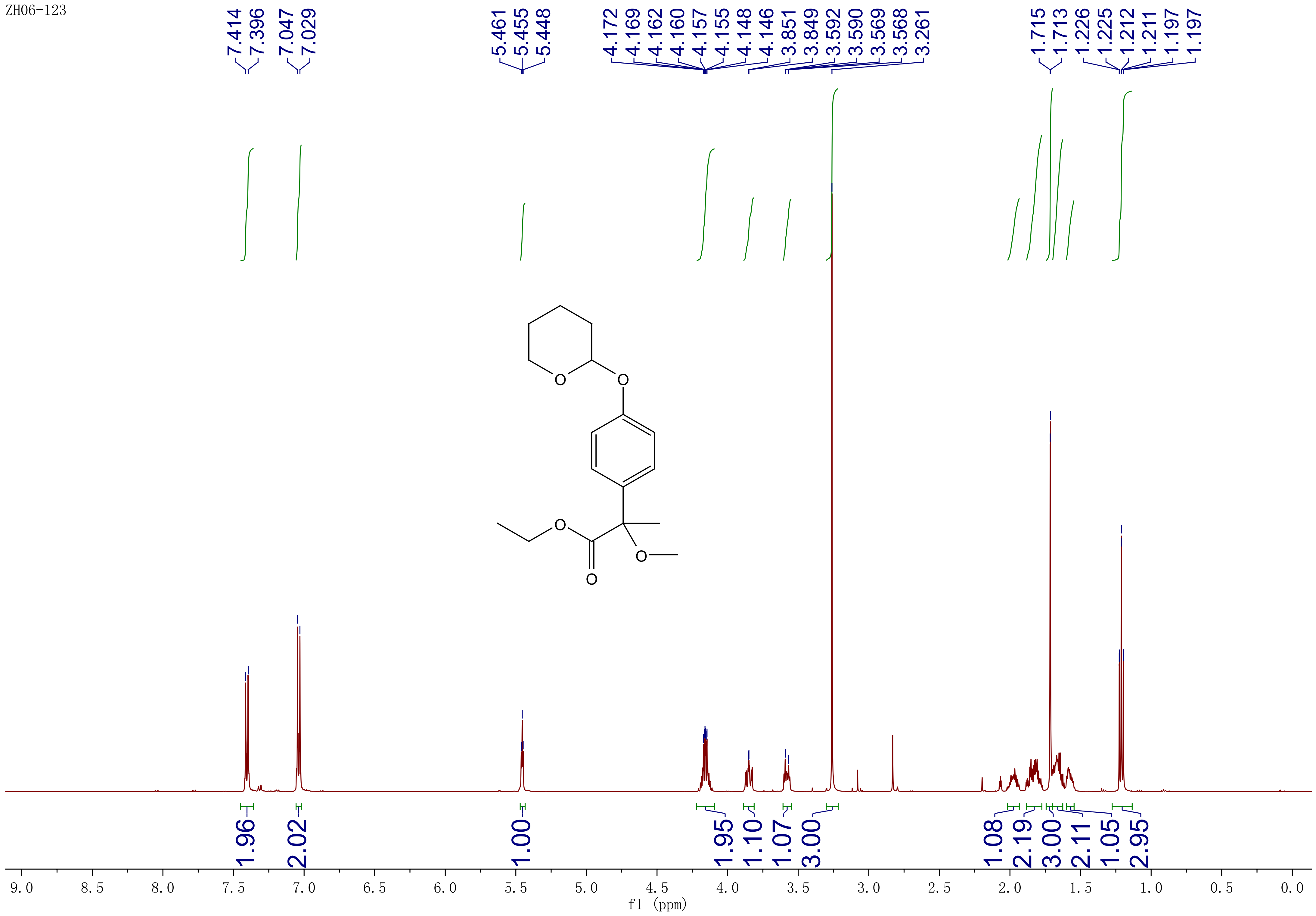
ZH06-123
7.414
7.396
7.047
7.029
5.461
5.455
5.448
4.172
4.169
4.162
4.160
4.157
4.155
4.148
4.146
3.851
3.849
3.592
3.590
3.569
3.568
3.261
1.715
1.713
1.226
1.225
1.212
1.211
1.197
1.197
1.96
2.02
1.00
1.95
1.10
1.07
3.00
1.08
2.19
3.00
2.11
1.05
2.95
9.0
8.5
8.0
7.5
7.0
6.5
6.0
5.5
5.0
4.5
4.0
3.5
3.0
2.5
2.0
1.5
1.0
0.5
0.0
f1 (ppm)
O
O
O
O
O
O

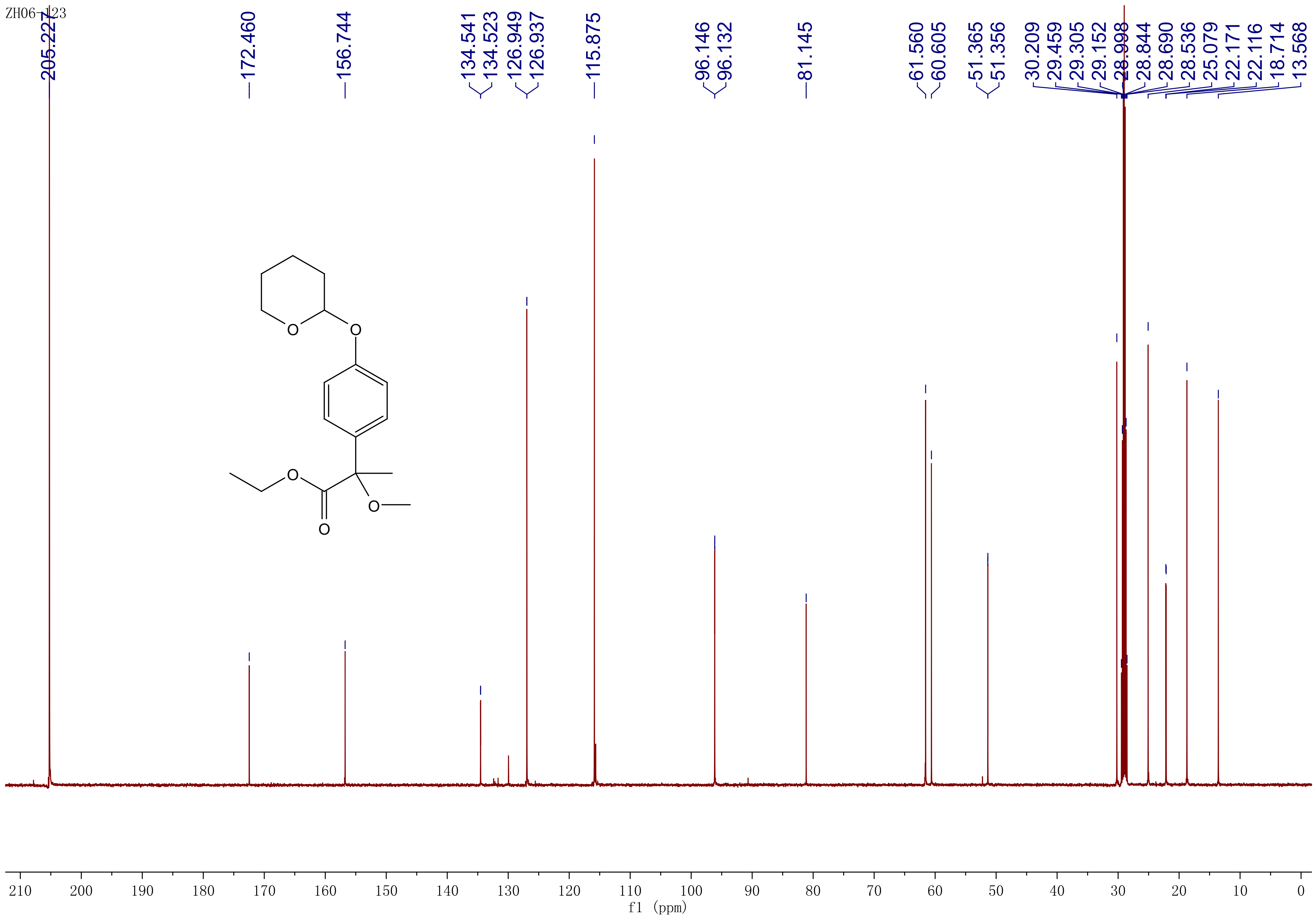
ZH06-123
205.227
172.460
156.744
134.541
134.523
126.949
126.937
115.875
96.146
96.132
81.145
61.560
60.605
51.365
51.356
30.209
29.459
29.305
29.152
28.998
28.844
28.690
28.536
25.079
22.171
22.116
18.714
13.568
O
O
O
O
O
210 200 190 180 170 160 150 140 130 120 110 100 90 80 70 60 50 40 30 20 10 0
f1 (ppm)

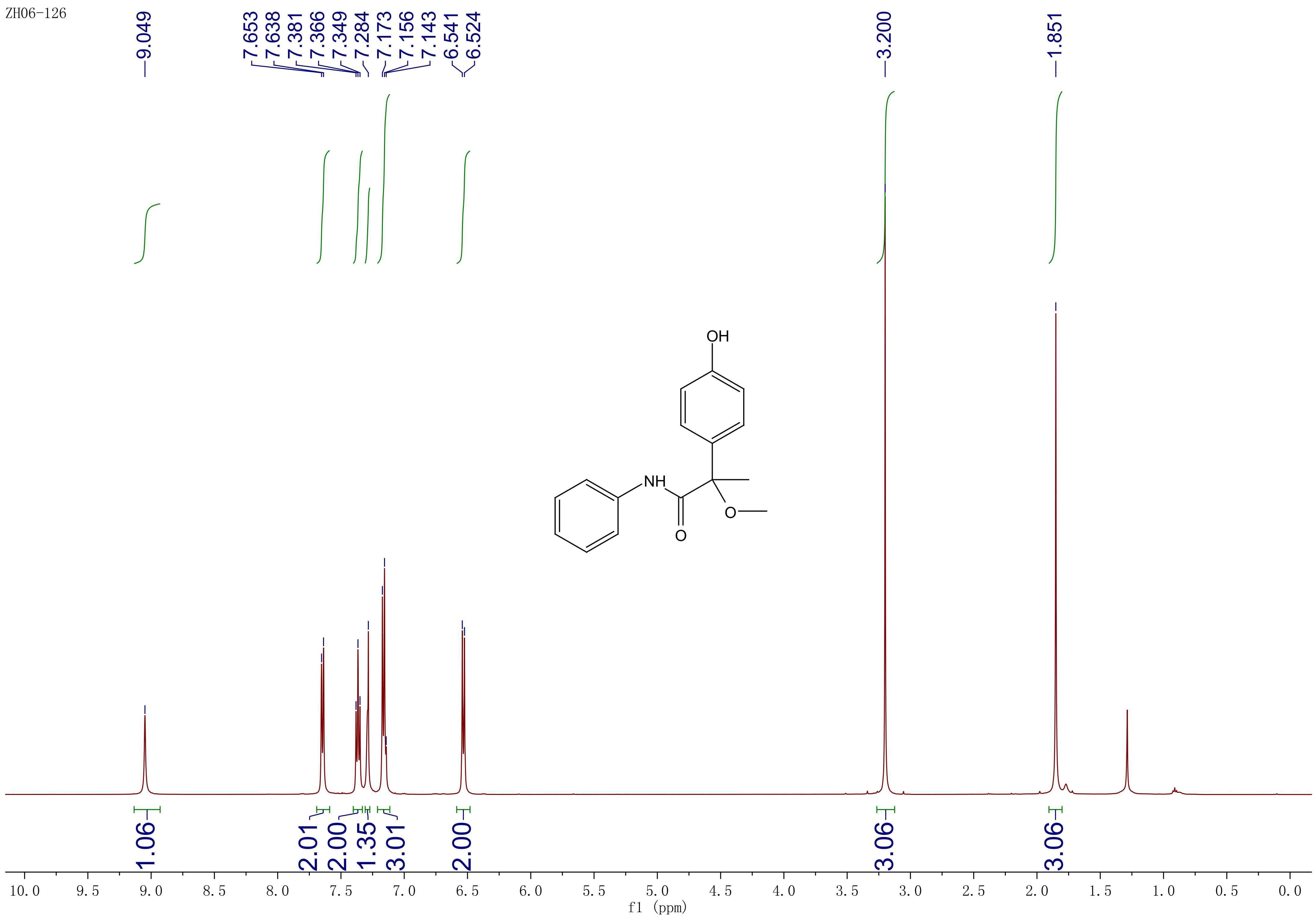

ZH06-126
9.049
7.653
7.638
7.381
7.366
7.349
7.284
7.173
7.156
7.143
6.541
6.524
3.200
1.851
OH
NH
O
O
1.06
2.01
2.00
1.35
3.01
2.00
3.06
3.06
10.0 9.5 9.0 8.5 8.0 7.5 7.0 6.5 6.0 5.5 5.0 4.5 4.0 3.5 3.0 2.5 2.0 1.5 1.0 0.5 0.0
f1 (ppm)

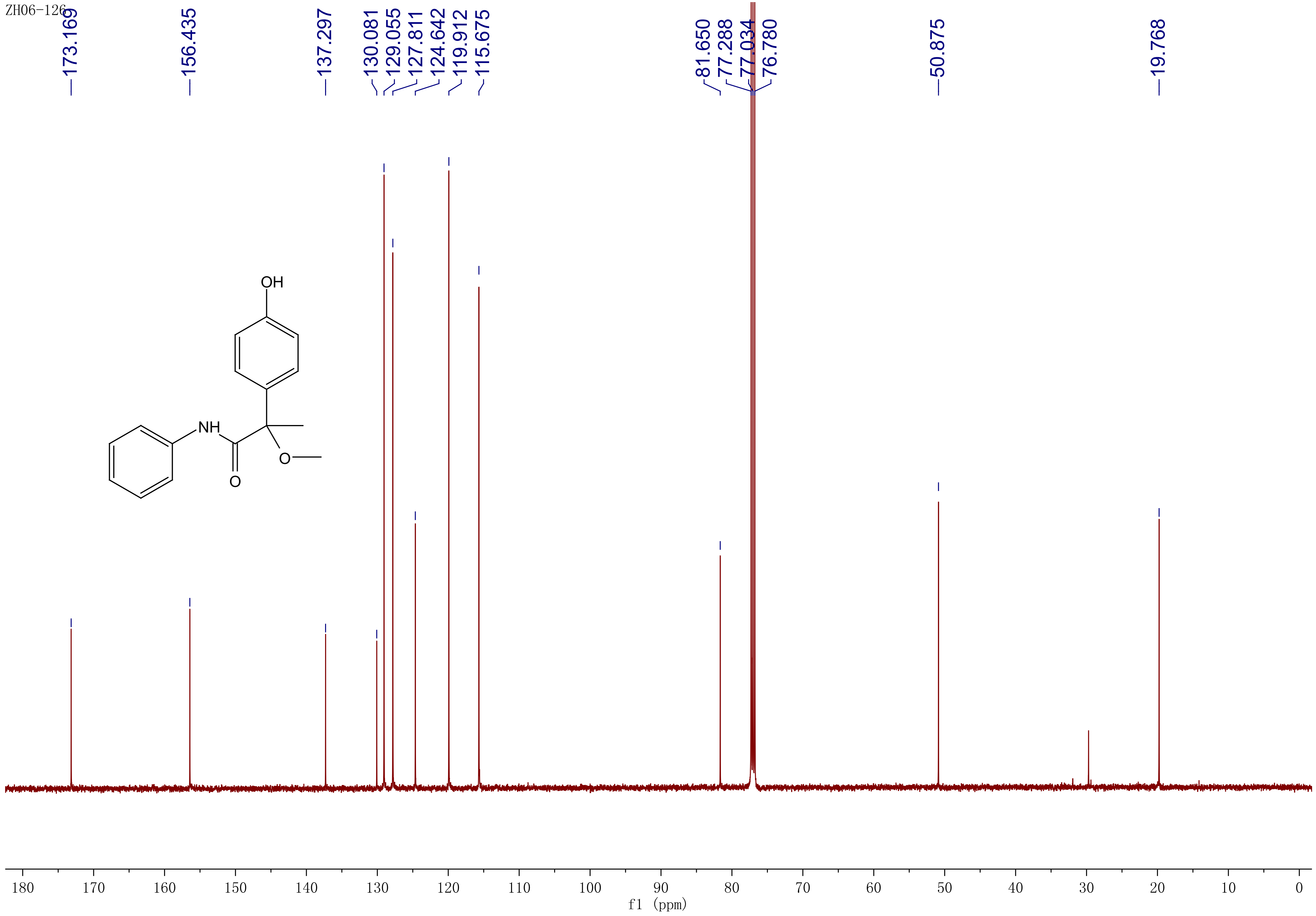
ZH06-126
173.169
156.435
137.297
130.081
129.055
127.811
124.642
119.912
115.675
81.650
77.288
77.034
76.780
50.875
19.768
OH
NH
O
O
180 170 160 150 140 130 120 110 100 90 80 70 60 50 40 30 20 10 0
f1 (ppm)

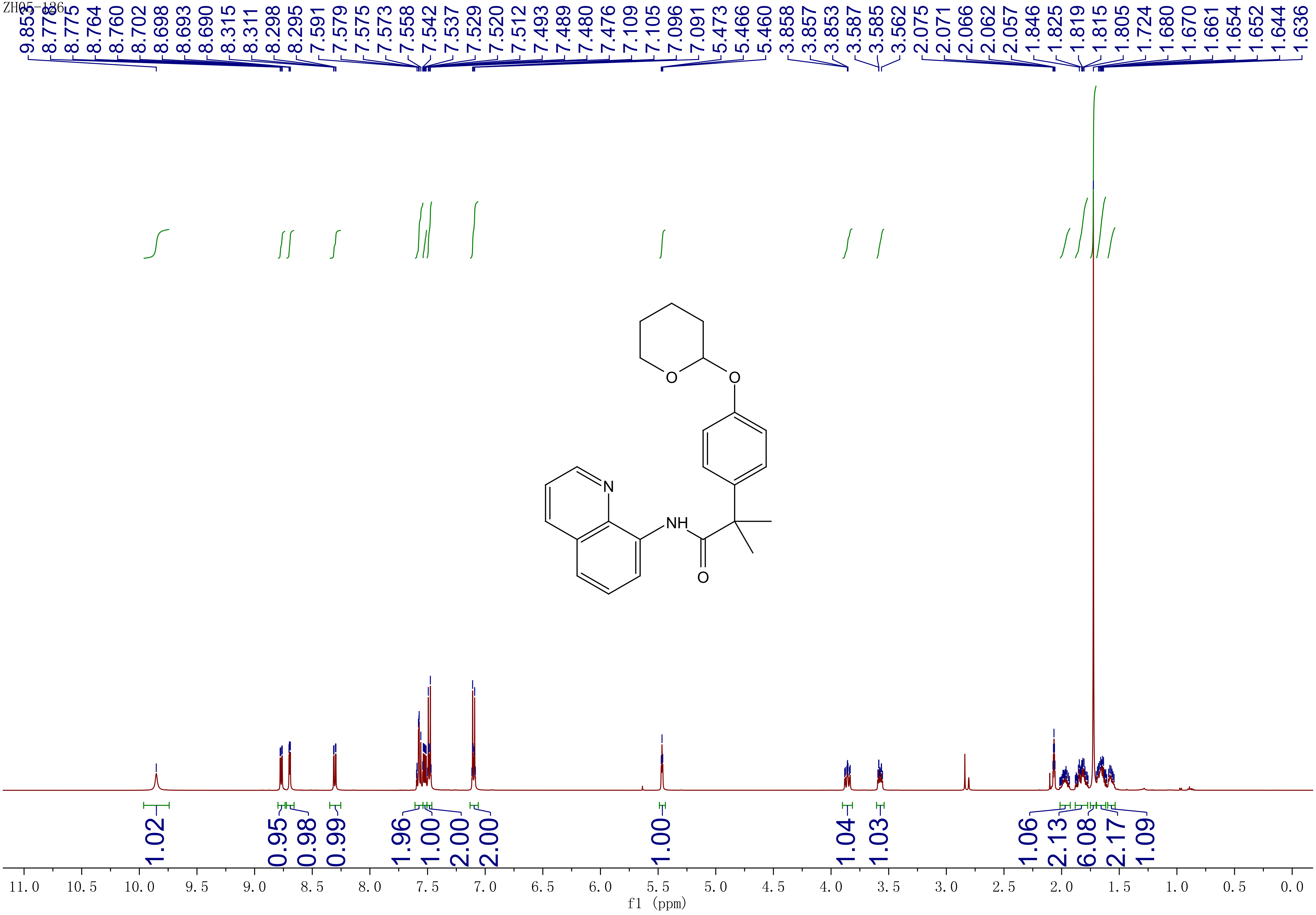
ZH05-126
9.853 8.779 8.775 8.764 8.760 8.702 8.698 8.693 8.690 8.315 8.311 8.298 8.295 7.591 7.579 7.575 7.573 7.558 7.542 7.537 7.529 7.520 7.512 7.493 7.489 7.480 7.476 7.109 7.105 7.096 7.091 5.473 5.466 5.460 3.858 3.857 3.853 3.587 3.585 3.562 2.075 2.071 2.066 2.062 2.057 1.846 1.825 1.819 1.815 1.805 1.724 1.680 1.670 1.661 1.654 1.652 1.644 1.636
N
NH
O
O
O
1.02 0.95 0.98 0.99 1.96 1.00 2.00 2.00 1.00 1.04 1.03 1.06 2.13 6.08 2.17 1.09
11.0 10.5 10.0 9.5 9.0 8.5 8.0 7.5 7.0 6.5 6.0 5.5 5.0 4.5 4.0 3.5 3.0 2.5 2.0 1.5 1.0 0.5 0.0
f1 (ppm)

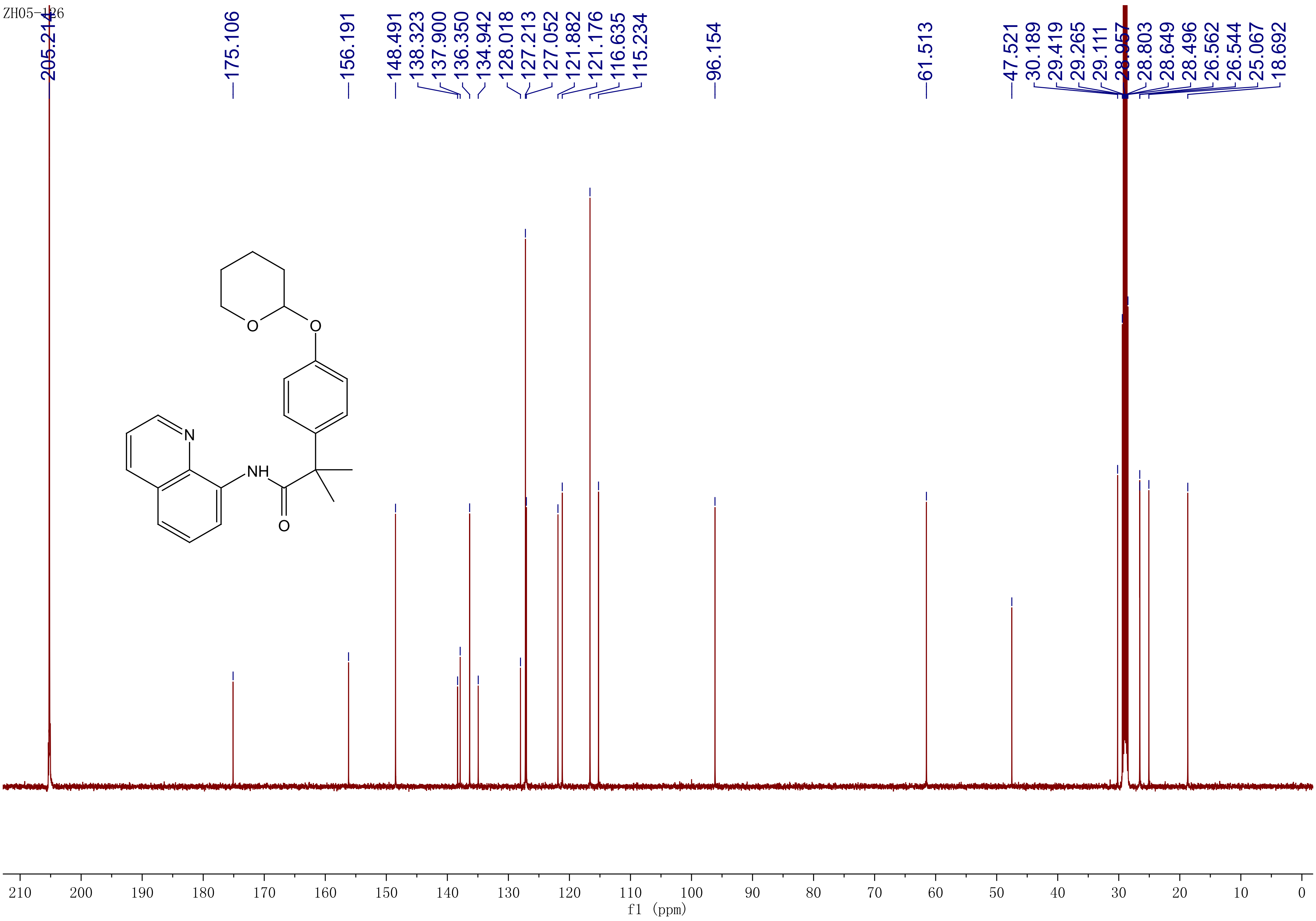
ZH05-126
205.214
175.106
156.191
148.491
138.323
137.900
136.350
134.942
128.018
127.213
127.052
121.882
121.176
116.635
115.234
96.154
61.513
47.521
30.189
29.419
29.265
29.111
28.957
28.803
28.649
28.496
26.562
26.544
25.067
18.692
O
O
N
NH
O
210 200 190 180 170 160 150 140 130 120 110 100 90 80 70 60 50 40 30 20 10 0
f1 (ppm)

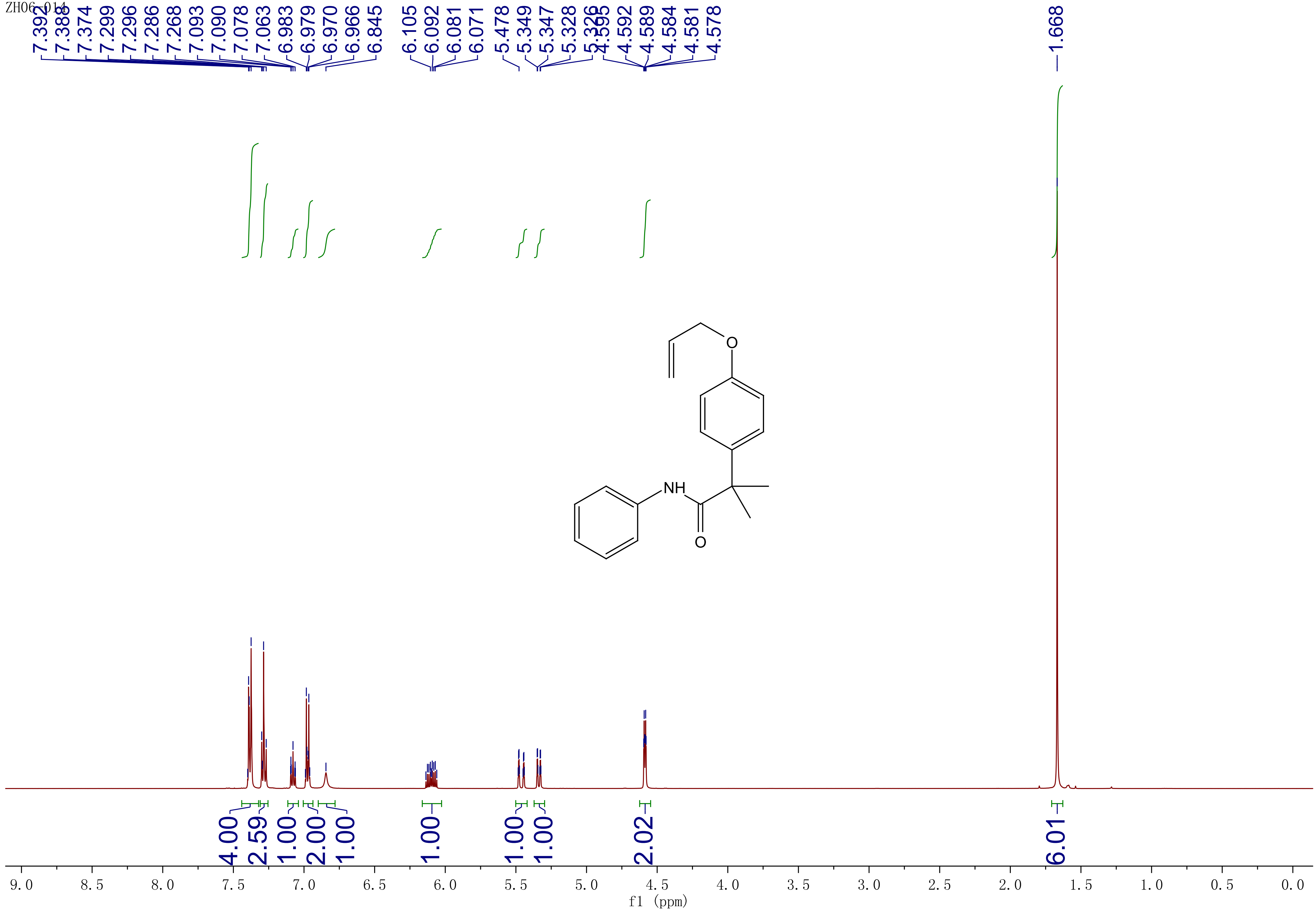
ZH06-014
7.392
7.388
7.374
7.299
7.296
7.286
7.268
7.093
7.090
7.078
7.063
6.983
6.979
6.970
6.966
6.845
6.105
6.092
6.081
6.071
5.478
5.349
5.347
5.328
5.326
4.595
4.592
4.589
4.584
4.581
4.578
1.668
NH
O
O
4.00
2.59
1.00
2.00
1.00
1.00
1.00
1.00
2.02
6.01
9.0
8.5
8.0
7.5
7.0
6.5
6.0
5.5
5.0
4.5
4.0
3.5
3.0
2.5
2.0
1.5
1.0
0.5
0.0
f1 (ppm)

ZH06-014

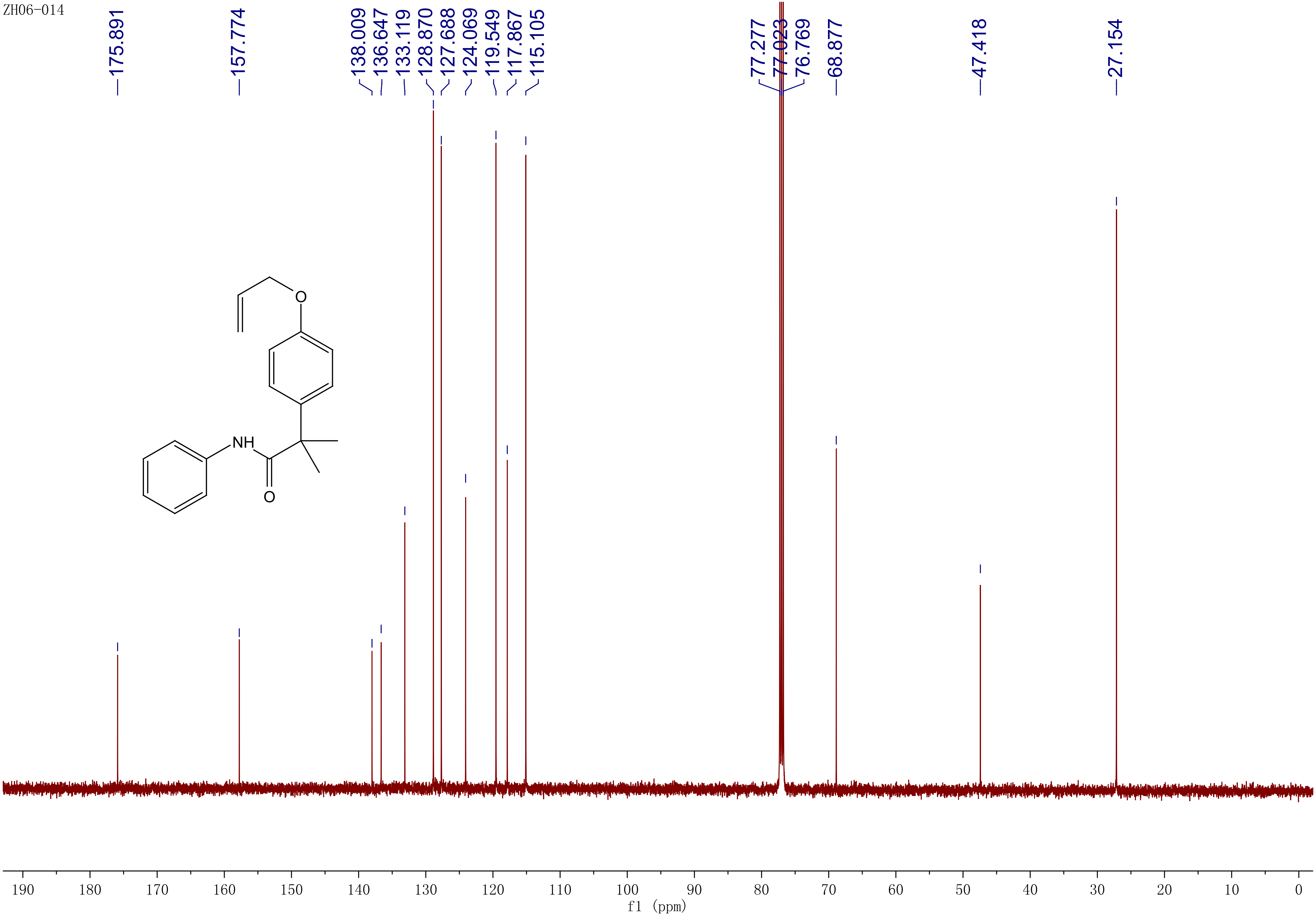
175.891
157.774
138.009
136.647
133.119
128.870
127.688
124.069
119.549
117.867
115.105
77.277
77.023
76.769
68.877
47.418
27.154
O
NH
O
190 180 170 160 150 140 130 120 110 100 90 80 70 60 50 40 30 20 10 0
f1 (ppm)

Compound **20**, gHSQC

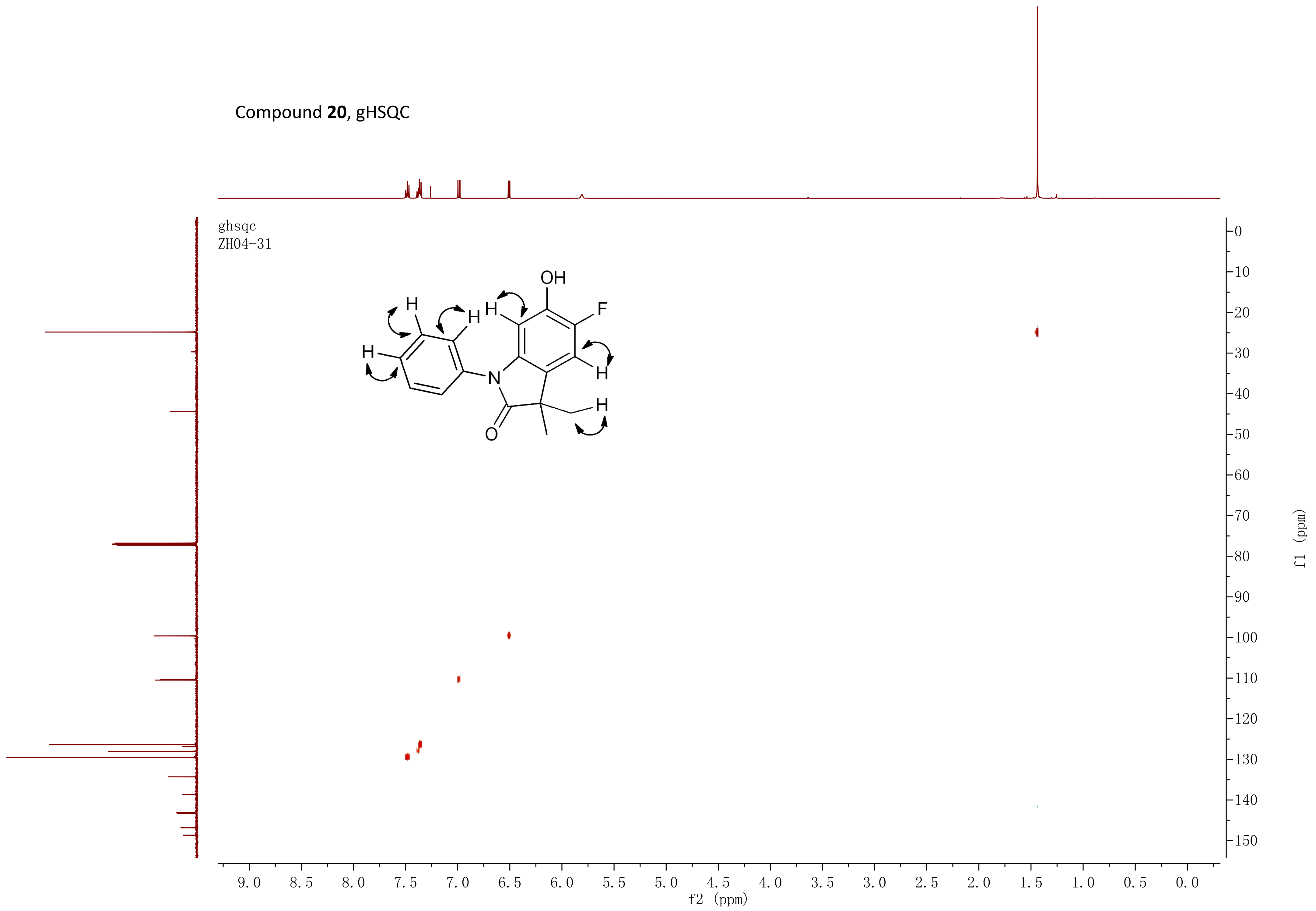

Compound **20**, NOESY

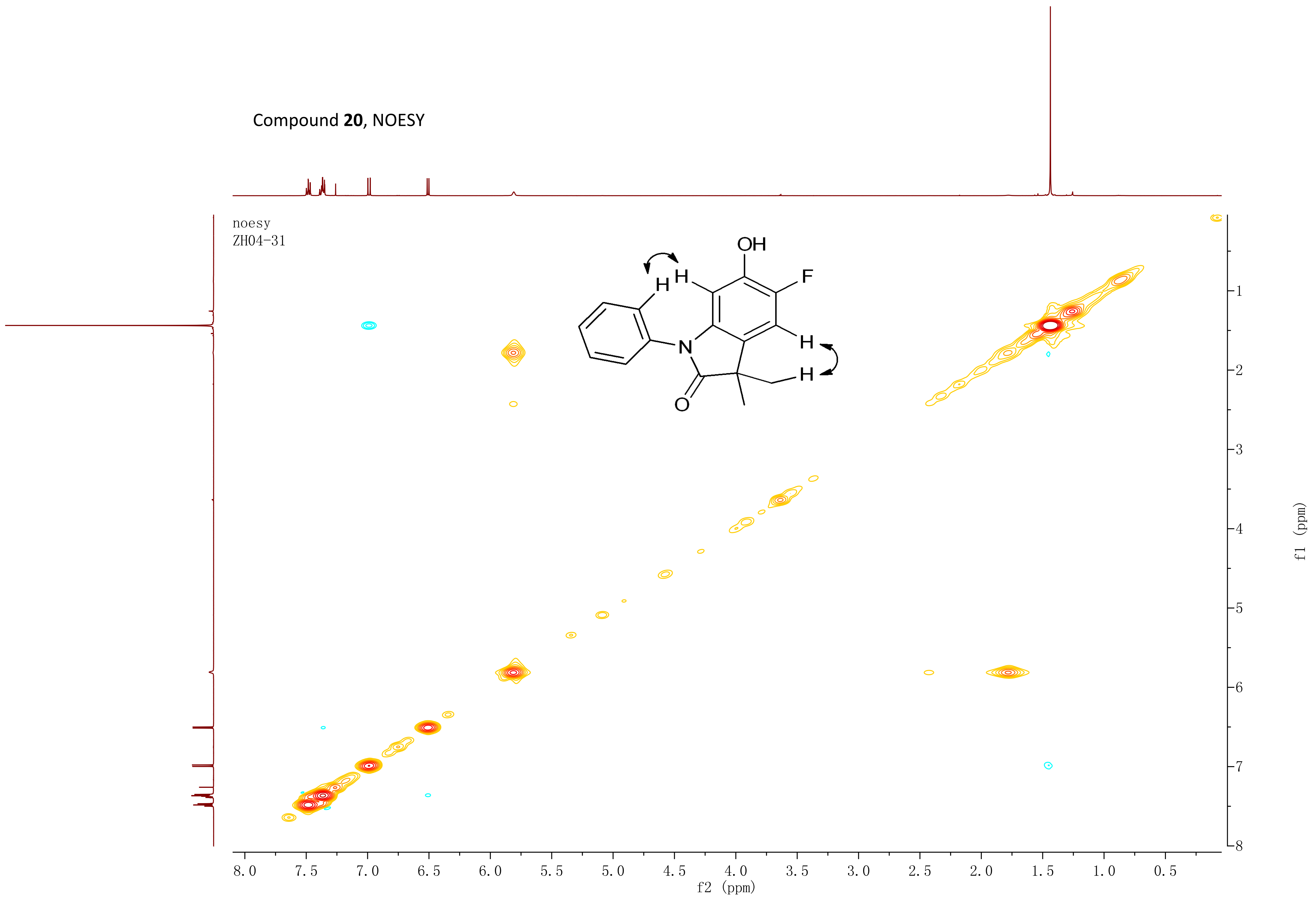

Compound **21**, NOESY

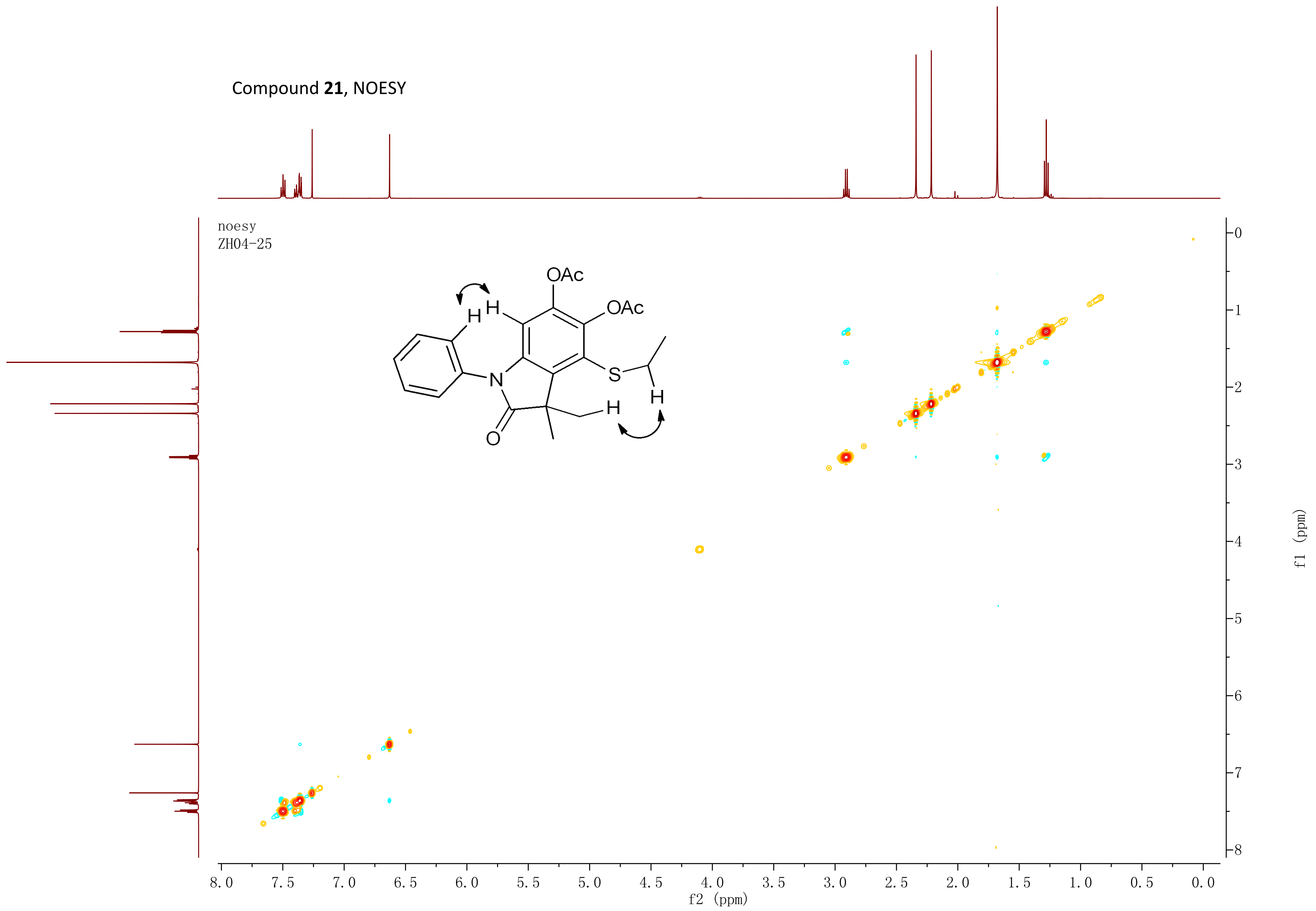

Compound **22**, NOESY

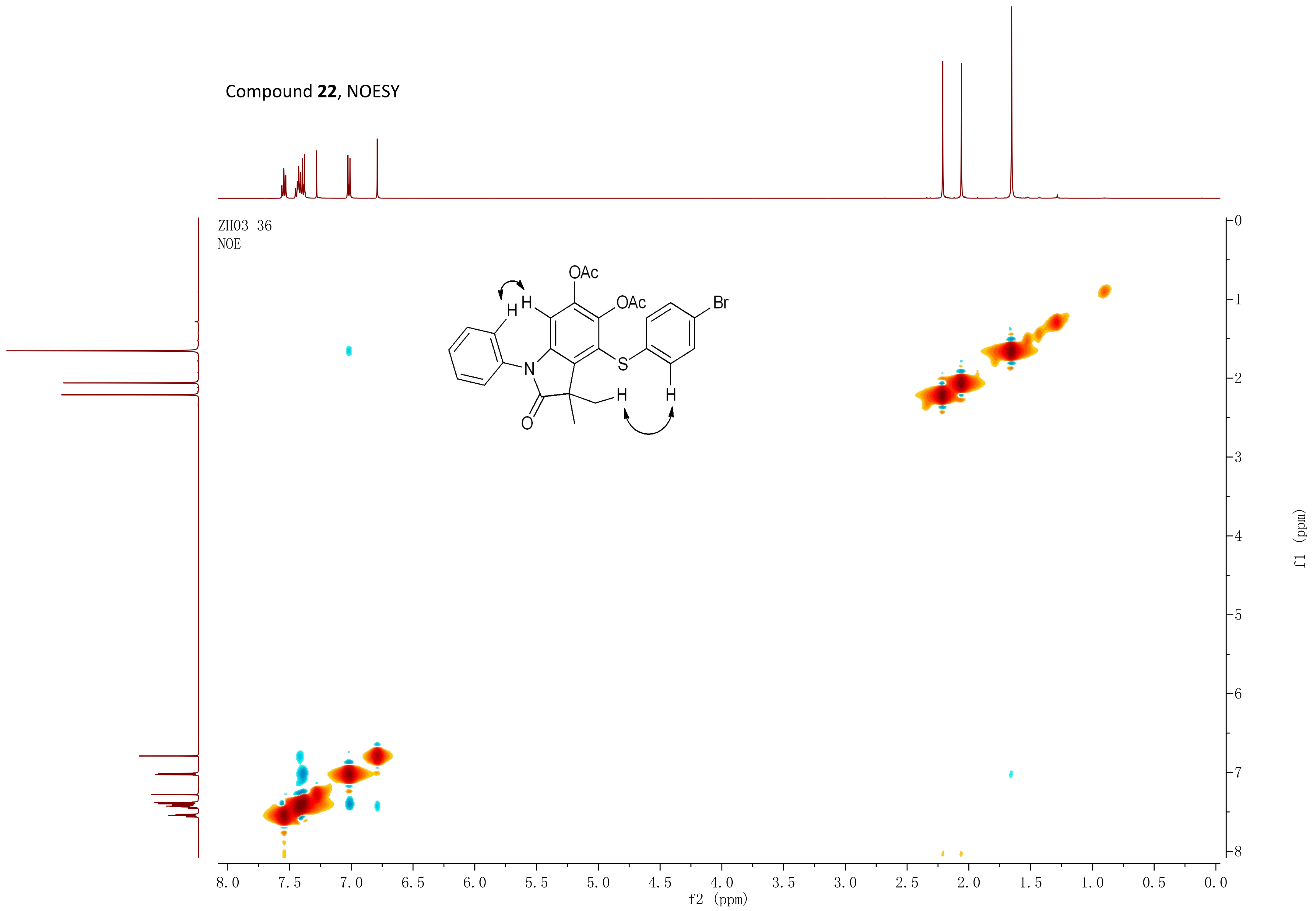

Supplement: Supplementary file 1 [file SC-007-C5SC02395E-s001.pdf]
