# Supplementary material for: A bio-inspired synthesis of oxindoles by catalytic aerobic dual C–H functionalization of phenols
Source: Chem Sci. 2015 Oct 6;7(1):358–69. doi: 10.1039/c5sc02395e (PMC5952266; doi:10.1039/c5sc02395e)
Supplement: Supplementary file 2 [file SC-007-C5SC02395E-s002.pdf]

Electronic Supplementary Material (ESI) for Chemical Science.
This journal is © The Royal Society of Chemistry 2015

A Bio-Inspired Synthesis of Oxindoles by Catalytic Aerobic Dual C-H Functionalization of Phenols

Zheng Huang,† Mohammad S. Askari,‡ Kenneth Virgil N. Esguerra,† Tian-Yang Dai,† Ohhyeon Kwon,† Xavier Ottenwaelder,*,‡ Jean-Philip Lumb*,†

† Department of Chemistry, McGill University, Montreal, QC, H3A 0B8, Canada

‡ Department of Chemistry and Biochemistry, Concordia University, Montreal, QC, H4B 1R6, Canada

Table of Contents

1. General Experimental 3

2. General Procedure for the Oxidation 4

3. Reaction Optimization and Mechanism Investigation 5

a) Complete Optimization Table (Table S1) 5
b) Cyclization of **Q2** (Table S2) 6
c) Cyclization of **Q1** (Table S3) 7
d) Redox Exchange (Table S4) 8
e) Oxidative C-O Coupling (Table S5) 9
f) Cyclization from para-Hydroxyquinone (HQ) Intermediate 10
g) Control Experiments on catechol **C2** and **C3** 11
h) Preparation of the Compounds for Mechanism Study 13

4. Reaction Intermediates SQ1-3 16

a) Mass Spectrometry of Reaction Solutions 16
b) DBEDCu(I) : **Q1-3** Binding and UV-Vis Features of **SQ1-3** 17
c) Synthesis of **SQ3** 18
d) Concentration Profiles During Reaction 18
e) Use of **SQ1-3** as precatalysts 19

5. Synthesis and Characterization of the Compounds in Tables 1-3 and Schemes 6-9 21

a) Reactions in Table 1 21
b) Reactions in Table 2 24
c) Reactions in Table 3 38
d) Reactions in Scheme 6 45
e) Reactions in Scheme 7 48
f) Reactions in Scheme 8 52
g) Reactions in Scheme 9 55

6. Synthesis and Characterization of Substrates and Precursors 61

a) General Procedures 61
b) Synthesis and Characterization of Precursors 62
i. Alkyl Halides 62

ii. Esters 64
c) Synthesis and Characterization of the Phenols 75
i. Substrates in Table 1...75
ii. Substrates in Table 2..79
iii. Substrates in Table 3 94
iv. Substrates in Scheme 6 104
v. Substrates in Scheme 7 110
vi. Substrates in Scheme 8 115

7. X-ray Crystallography..117

1. General Experimental

Chemicals and solvents were purchased from Sigma Aldrich, Alfa Aesar, Strem Chemicals or TCI. Solvents were dried and purified using a PureSolv MD 7 (from Innovative Technology) or MB SPS 800 (from MBraun). We have not observed differences in the reaction outcome using either of these solvent purifiers. The copper(I) salt $[Cu(CH_3CN)_4](PF_6)$, abbreviated $CuPF_6$, was purchased from commercial sources or made via a literature procedure (Kubas, G. J., *Inorg. Synth.* **1990,** *28*, 68-70). $[Cu(CH_3CN)_4](SbF_6)$ was prepared via the same method, but using $HSbF_6$ instead of HPF_6. All copper(I) complexes were stored inside the glovebox. *N*,*N'*-di-*tert*-butylethylenedimaine (DBED) and 1,8-Diazabicycloundec-7-ene (DBU) were distilled over CaH_2 under N_2 prior to use. Molecular sieves (4 Å, powdered and "activated") were purchased from Sigma Aldrich and were flame-dried with a torch in the reaction vessel immediately prior to use. Unless otherwise noted, reactions were performed in flame-dried glassware under a positive pressure of nitrogen using standard synthetic organic, inert atmosphere techniques. All oxidation reactions were set-up in flame-dried, 25 mL Radley tubes with a Teflon-coated stir bar under a nitrogen atmosphere (*Praxair*, N_2 pre-purified). The reaction vessels were then connected to a cylinder of O_2 (Praxair), purged three times with O_2 and then pressurized to +1.0 atm.

Proton nuclear magnetic resonance (^1H NMR) spectra were acquired using Varian 500 MHz, Bruker Ascend 500 MHz, Bruker Ascend 400 MHz, Varian Inova 400 MHz and Varian Mercury 300 MHz spectrometers. Chemical shifts (δ) are reported in parts per million (ppm) and are calibrated to the residual solvent peak. Coupling constants (*J*) are reported in Hz. Multiplicities are reported using the following abbreviations: *s* = singlet; *d* = doublet; *t* = triplet; *q* = quartet; *m* = multiplet (range of multiplet is given). Carbon nuclear magnetic resonance (^{13}C NMR) spectra were acquired using Varian 125 MHz, Bruker Ascend 125 MHz, Bruker Ascend 100 MHz, Varian Inova 100 MHz and Varian Mercury 75 MHz spectrometers. Chemical shifts (δ) are reported in parts per million (ppm) and are calibrated to the residual solvent peak. Fluorine nuclear magnetic resonance (^{19}F NMR) spectra were acquired using Varian Mercury 282 MHz spectrometers. Chemical shifts (δ) are reported in parts per million (ppm). Coupling constants (*J*) are reported in Hz. Multiplicities are reported using the following abbreviations: *s* = singlet; *d* = doublet; *t* = triplet; *q* = quartet; *m* = multiplet (range of multiplet is given). High resolution mass spectra (HRMS) were recorded using a Bruker maXis Impact TOF mass spectrometer by electrospray ionization time of flight reflectron experiments. Fourier-transform infrared (FT-IR) spectra were recorded on a Thermo Scientific Nicolet 6700 FT-IR spectrometer. Analytical thin-layer chromatography was performed on pre-coated 250 mm layer thickness silica gel 60 F_{254} plates (EMD Chemicals Inc.). Visualization was performed by ultraviolet light and/or by staining with potassium permanganate or cerium molybdate. Purifications by column chromatography were performed using either a Biotage Isolera™ One (Snap Ultra, particle size 25 µm, 230-400 mesh), or standard column chromatography using silica gel (40-63 µm, 230-400 mesh).

UV-visible spectra were recorded on a B&W Tek iTrometer equipped with fiber-optic cables connected to a Hellma full-quartz dip-probe having a 1.0 mm pathlength. The probe was immersed in the solution inside a custom-made Schlenk flask. Electrospray ionization mass-spectrometry (ESI-MS) experiments were performed via direct injection on a Micromass Quattro LC.

2. General Procedure for the Oxidation

General Procedure A: ***experiments in Table 1-4 and Scheme 5 on 1.0 mmol scale***. A flame-dried, 25 mL Radley tube equipped with a Teflon-coated stir bar and a rubber septum was charged with the phenol (1.0 mmol, 1.0 equiv) and then purged with a steady stream of N_2 for 5 min, prior to the addition of dry and degassed CH_2Cl_2 (4 mL). In a separate, flame-dried 5 mL microwave vial $[Cu(CH_3CN)_4](PF_6)$ (0.04 mmol, 14.9 mg, 4 mol%) and N,N'-di-*tert*-butylethylenediamine (0.20 mmol, 43.1 μL, 20 mol%) were dissolved in CH_2Cl_2 (1.0 mL, 0.04 M) to afford a homogeneous pink solution. This solution was then added to the Radley tube via syringe to afford a final volume of 5.0 mL and a concentration 0.2 M with respect to phenol. The rubber septum was then rapidly removed and replaced with a Radley cap, which was connected to a tank of O_2 and pressurized to 1 atm. Under a constant pressure of O_2 (1 atm), the reaction was vented 3 times for 10 s to remove N_2. A dramatic color change was observed within 2 min, resulting in a blackish/brown reaction mixture. The reaction mixture was then stirred at room temperature (20-23 ^oC) for 4 h, depressurized by opening to the atmosphere and quenched by the addition of $NaHSO_4$ (20 mL, 10% by weight aqueous solution). The phases were then separated and the aqueous phase was extracted with CH_2Cl_2 (3 × 20 mL). The combined organic fractions were then dried over $MgSO_4$, filtered and concentrated *in vacuo*. The residue was then purified on silica gel using hexanes/ethyl acetate as eluent to afford the oxindoloquinone.

Modification of the General Procedure A (M1): ***addition of DBU (Table 1, Entry 3)***. The following change was made to the standard reaction procedure. Neat 1,8-Diazabicycloundec-7-ene (DBU) (0.30 mmol, 44.8 μL, 30 mol%) was added via syringe to a Radley tube containing phenol, $[Cu(CH_3CN)_4](PF_6)$, DBED and solvent under an atmosphere of N_2 before exposing to O_2. The reaction was then carried out as described in the general procedure.

Modification of the General Procedure A (M2): ***reaction at 45 ^oC (Table 1, Entry 6)***. The following change was made to the standard reaction procedure. Instead of stirring the reaction mixture at room temperature, the reaction vessel was immersed in a 45 ^oC oil bath and stirred at this temperature. The reaction was then carried out as described in the general procedure.

Modification of the General Procedure A (M3): ***modified work-up (Table 2, compound 5 and 18-24)***. The following change was made to the standard reaction procedure. NH_4Cl/NH_3OH (20 mL, 10:1 volume mixture of a saturated aqueous NH_4Cl solution and a 30% aqueous solution of ammonium hydroxide) was used instead of the standard $NaHSO_4$ solution during the work-up. Further analysis was then carried out as described in the general procedure.

3. Reaction Optimization and Mechanism Investigation

a) Complete Optimization Table (Table S1)

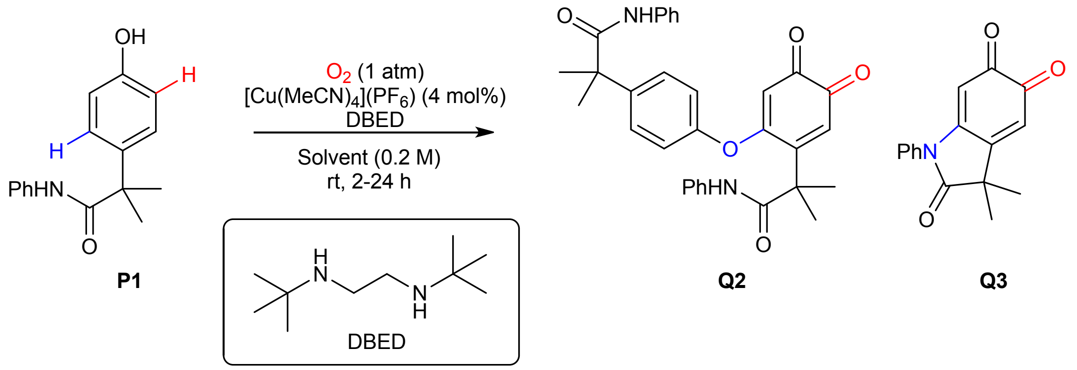

entry[a]	DBED (mol%)	Solvent	time (h)	Conversion (%)	Yield of **2**[b,d] (%)	Yield of **3**[b,d] (%)
1	5	CH_2Cl_2	2	43	42	< 1
2	7.5	CH_2Cl_2	2	64	36	24
3	10	CH_2Cl_2	2	81	4	76
4[c]	5	CH_2Cl_2	2	71	52	12
5[c]	10	CH_2Cl_2	2	67	2	64
6	10	THF	2	41	2	37
7	10	EtOAc	2	36	8	24
8	20	CH_2Cl_2	4	99	< 2	98 (90)
9[e]	20	CH_2Cl_2	4	92	< 2	86
10[f]	20	CH_2Cl_2	24	79	< 2	71
11[c,f]	20	CH_2Cl_2	24	77	< 2	72

[a] Reactions performed with 0.5 mmol of **1**. [b] Product yield determined by ^{1}H-NMR using hexamethylbenzene as an internal standard. [c] Reaction performed with 4 Å mol. sieves (100 mg). [d] Isolated yield in parenthesis using 1 mmol of **1**. [e] Reaction performed using a balloon of O_2. [f] Reaction performed under open flask conditions.

General Procedure: A flame-dried, 25 mL Radley tube equipped with a Teflon-coated stir bar and a rubber septum was charged with, phenol **P1** (0.5 mmol, 1.0 equiv) and hexamethylbenzene (12.2 mg, 0.075 mmol, internal NMR standard). The reaction vessel was then purged with a steady stream of N_2 for 5 min, prior to the addition of dry and degassed solvent (2 mL). In a separate, flame-dried 5 mL microwave vial [Cu(CH$_3$CN)$_4$](PF$_6$) (7.5 mg, 0.02 mmol, 4 mol%) and N,N'-di-*tert*-butylethylenediamine (0.025-0.1 mmol, 5-20 mol%) were dissolved in the indicated solvent (0.5 mL, 0.04 M) to afford a homogeneous pink solution. This solution was then added to the Radley tube via syringe to afford a final volume of 2.5 mL and a concentration in phenol of 0.2 M. The rubber septum was then rapidly removed and replaced with a Radley cap, which was connected to a tank of O_2 and pressurized to 1 atm. Under a constant pressure of O_2 (1 atm), the reaction was vented 3 times for 10 s to remove N_2. A dramatic color change was observed within 2 min, resulting in a blackish/brown reaction mixture. The reaction mixture was then stirred at room temperature (20-23 ^oC) for the indicated amount of time, depressurized by opening to the atmosphere and quenched by the addition of $NaHSO_4$ (10 mL, 10% by weight aqueous solution). The phases were then separated and the aqueous phase was extracted with CH_2Cl_2 (3 × 10 mL). The combined organic fractions were then dried over $MgSO_4$, filtered and concentrated *in vacuo* to afford a blood-red residue which was analyzed directly by ^{1}H-NMR.

Modification of General Procedure: *addition of 4Å Molecular Sieves (Table S1, Entries 4,5 and 11).* The following change was made to the standard reaction procedure. A 25-mL Radkey tube was charged with 4 Å molecular sieves (100 mg). The reaction vessel was then evacuated and flame dried with a torch until the molecular sieves began to "dance" in the reaction vessel. The reaction vessel was then cooled to rt, and the reaction was carried out as described in the general procedure.

Modification of the General Procedure: ***use of an oxygen balloon (Table S1, Entry 9).*** The following change was made to the standard reaction procedure. Instead of replacing the rubber septum with a Radley cap, the nitrogen line was simply removed and replaced with a balloon of O_2. O_2 was then bubbled through the reaction mixture for 5 min, and the flask was then equipped with a newly inflated balloon of O_2. The reaction mixture was then stirred at room temperature (20-23 ^{o}C) for 4h and quenched by the addition of $NaHSO_4$ (10 mL, 10% by weight aqueous solution). Further analysis was then carried out as described in the general procedure.

Modification of the General Procedure: ***open flask conditions (Table S1, Entry 10 and 11).*** The following change was made to the standard reaction procedure. Instead of replacing the rubber septum with a Radley cap, the rubber septum was simply removed, exposing the reaction to the open air. The reaction mixture was then stirred at room temperature (20-23 ^{o}C) for 24h and quenched by the addition of $NaHSO_4$ (10 mL, 10% by weight aqueous solution). Further analysis was then carried out as described in the general procedure.

b) Cyclization of Q2 (Table S2)

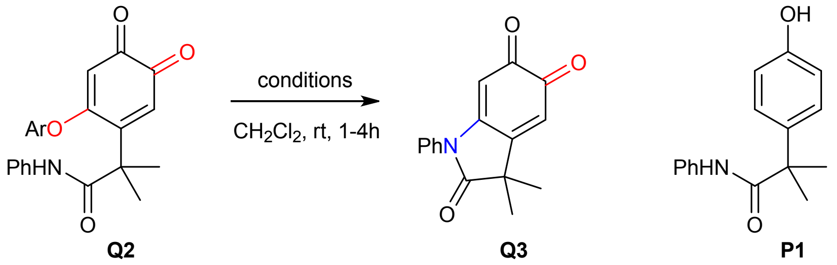

entry[a]	Condition	Time (h)	Conversion (%)	Yield (%)[b] Q3	P1
1	20 mol% DBED	1	35	32	35
2	20 mol% DBED	4	100	> 95	> 95
3	20 mol% DBU	1	100	> 95	> 95
4	20 mol% Cs_2CO_3	1	100	95	92
5	catalytic conditions[c]	4	100	> 95	0
6	20 mol% $TsOH{\cdot}H_2O$	1	0	0	0

[a] Reactions performed with 0.1 mmol of **Q2**. [b] Product yield determined by ^{1}H-NMR using hexamethylbenzene as an internal standard. [c] catalytic conditions: $[Cu(MeCN)_4](PF_6)$ (4 mol%), DBED (20 mol%), DCM (0.2 M), rt, 4h, O_2 (1 atm).

General Procedure (Entry 1-4 and 6): A flame-dried, 25 mL round-bottom flask equipped with a Teflon-coated stir bar and a rubber septum was charged with **Q2** (0.1 mmol, 52.3 mg, 1.0 equiv) and hexamethylbenzene (2.4 mg, 0.015 mmol, internal NMR standard) followed by the addition of dry and degassed CH_2Cl_2 (10 mL) via syringe. The corresponding additive (0.02 mmol, 20 mol%) was then added and the resulting mixture was stirred at room temperature (20-23 ^{o}C) for the indicated amount of time. It was then quenched by the addition of $NaHSO_4$ (10 mL, 10% by weight aqueous solution) and extracted with CH_2Cl_2 (3 × 10 mL). The combined organic fractions were then dried over $MgSO_4$, filtered and concentrated *in vacuo* to afford the crude residue which was analyzed directly by ^{1}H-NMR.

Procedure (Entry 5): A flame-dried, 25 mL Radley tube equipped with a Teflon-coated stir bar and a rubber septum was charged with, **Q2** (0.1 mmol, 52.3 mg, 1.0 equiv) and hexamethylbenzene (2.4 mg, 0.015 mmol, internal NMR standard). The reaction vessel was then purged with a steady stream of N_2 for 5 min. In a separate, flame-dried 5 mL microwave vial $[Cu(CH_3CN)_4](PF_6)$ (1.5 mg, 0.004 mmol, 4 mol%) and N,N'-di-*tert*-butylethylenediamine (DBED) (4.4 μL, 0.02 mmol, 20 mol%) were dissolved in the indicated solvent (0.5 mL) to afford a homogeneous pink solution. This solution was then added to the Radley tube via syringe to afford a homogeneous solution. The rubber septum was then rapidly removed and replaced with a Radley cap, which was connected to a tank of O_2 and pressurized to 1 atm. Under a constant pressure of O_2 (1 atm), the reaction was vented 3 times for 10 s to remove N_2. The reaction mixture was then stirred at room

temperature (20-23 °C) for 4h, depressurized by opening to the atmosphere and quenched by the addition of $NaHSO_4$ (10 mL, 10% by weight aqueous solution). The phases were then separated and the aqueous phase was extracted with CH_2Cl_2 (3 × 10 mL). The combined organic fractions were then dried over $MgSO_4$, filtered and concentrated *in vacuo* to afford the crude residue which was analyzed directly by ^{1}H-NMR.

c) Cyclization of Q1 (Table S3)

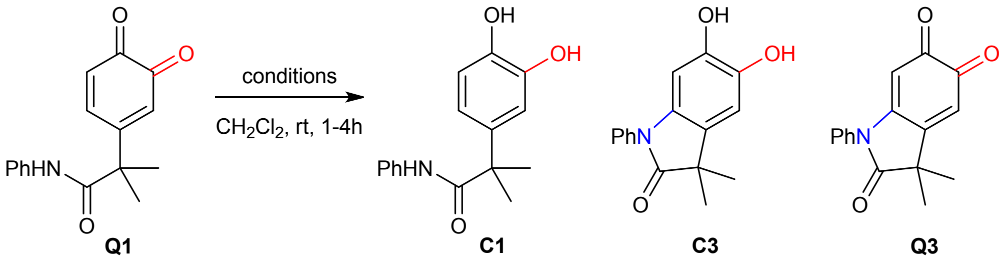

entry[a]	Condition	Conversion (%)	Yield (%)[b] C1	C3	Q3
1	20 mol% DBED	100	25	3	42
2	20 mol% DBU	100	25	7	45
3	20 mol% K_2CO_3	88	0	0	12
4	20 mol% TsOH·H_2O	79	30	0	23
5	catalytic conditions[c]	100	---	---	58
6	$PhI(OAc)_2$ (1.1 eq.), DBED (20 mol%)	100	---	---	53
7[d]	NBu_4Br (5 mol%), $NaIO_4$ (1.1 eq.), DBED (20 mol%)	100	---	---	73

[a] Reactions performed with 0.1 mmol of **Q1**. [b] Product yield determined by ^{1}H-NMR using hexamethylbenzene as an internal standard. [c] catalytic conditions: $[Cu(MeCN)_4](PF_6)$ (4 mol%), DBED (20 mol%), DCM (0.2 M), rt, 4h, O_2 (1 atm). [d] The reaction was running in a mixture of CH_2Cl_2 (10 mL) and water (2 mL).

General Procedure (Entry 1-4): A flame-dried, 25 mL round-bottom flask equipped with a Teflon-coated stir bar and a rubber septum was charged with **Q1** (0.1 mmol, 26.9 mg, 1.0 equiv) and hexamethylbenzene (2.4 mg, 0.015 mmol, internal NMR standard) followed by the addition of dry and degassed CH_2Cl_2 (10 mL) via syringe. The corresponding additive (0.02 mmol, 20 mol%) was then added and the resulting mixture was stirred at room temperature (20-23 °C) for 1h. It was then quenched by the addition of $NaHSO_4$ (10 mL, 10% by weight aqueous solution) and extracted with CH_2Cl_2 (3 × 10 mL). The combined organic fractions were then dried over $MgSO_4$, filtered and concentrated *in vacuo* to afford the crude residue which was analyzed directly by ^{1}H-NMR.

Procedure (Entry 5): A flame-dried, 25 mL Radley tube equipped with a Teflon-coated stir bar and a rubber septum was charged with, **Q1** (0.1 mmol, 26.9 mg, 1.0 equiv) and hexamethylbenzene (2.4 mg, 0.015 mmol, internal NMR standard). The reaction vessel was then purged with a steady stream of N_2 for 5 min. In a separate, flame-dried 5 mL microwave vial $[Cu(CH_3CN)_4](PF_6)$ (1.5 mg, 0.004 mmol, 4 mol%) and N,N'-di-*tert*-butylethylenediamine (DBED) (4.4 µL, 0.02 mmol, 20 mol%) were dissolved in the indicated solvent (0.5 mL) to afford a homogeneous pink solution. This solution was then added to the Radley tube via syringe to afford a homogeneous solution. The rubber septum was then rapidly removed and replaced with a Radley cap, which was connected to a tank of O_2 and pressurized to 1 atm. Under a constant pressure of O_2 (1 atm), the reaction was vented 3 times for 10 s to remove N_2. The reaction mixture was then stirred at room temperature (20-23 °C) for 4h, depressurized by opening to the atmosphere and quenched by the addition of $NaHSO_4$ (10 mL, 10% by weight aqueous solution). The phases were then separated and the aqueous phase was extracted with CH_2Cl_2 (3 × 10 mL). The combined organic fractions were then dried over $MgSO_4$, filtered and concentrated *in vacuo* to afford the crude residue which was analyzed directly by ^{1}H-NMR.

Procedure (Entry 6): A flame-dried, 25 mL round-bottom flask equipped with a Teflon-coated stir bar and a rubber septum was charged with **Q1** (0.1 mmol, 26.9 mg, 1.0 equiv), $PhI(OAc)_2$ (0.11 mmol, 35.4 mg, 1.1 equiv) and hexamethylbenzene

(2.4 mg, 0.015 mmol, internal NMR standard) followed by the addition of dry and degassed CH_2Cl_2 (10 mL) via syringe. N,N'-di-*tert*-butylethylenediamine (DBED) (4.4 μL, 0.02 mmol, 20 mol%) was then added via syringe and the resulting mixture was stirred at room temperature (20-23 °C) for 1h. It was then quenched by the addition of $NaHSO_4$ (10 mL, 10% by weight aqueous solution) and extracted with CH_2Cl_2 (3 × 10 mL). The combined organic fractions were then dried over $MgSO_4$, filtered and concentrated *in vacuo* to afford the crude residue which was analyzed directly by ^{1}H-NMR.

Procedure (Entry 7): A flame-dried, 25 mL round-bottom flask equipped with a Teflon-coated stir bar and a rubber septum was charged with **Q1** (0.1 mmol, 26.9 mg, 1.0 equiv), NBu_4Br (0.005 mmol, 1.6 mg, 5 mol%), $NaIO_4$ (0.11 mmol, 23.5 mg, 1.1 equiv) and hexamethylbenzene (2.4 mg, 0.015 mmol, internal NMR standard) followed by the addition of dry and degassed CH_2Cl_2 (10 mL) and water (2 mL) via syringe. N,N'-di-*tert*-butylethylenediamine (DBED) (4.4 μL, 0.02 mmol, 20 mol%) was then added via syringe and the resulting mixture was stirred at room temperature (20-23 °C) for 1h. It was then quenched by the addition of $NaHSO_4$ (10 mL, 10% by weight aqueous solution) and extracted with CH_2Cl_2 (3 × 10 mL). The combined organic fractions were then dried over $MgSO_4$, filtered and concentrated *in vacuo* to afford the crude residue which was analyzed directly by ^{1}H-NMR.

d) Redox Exchange (Table S4)

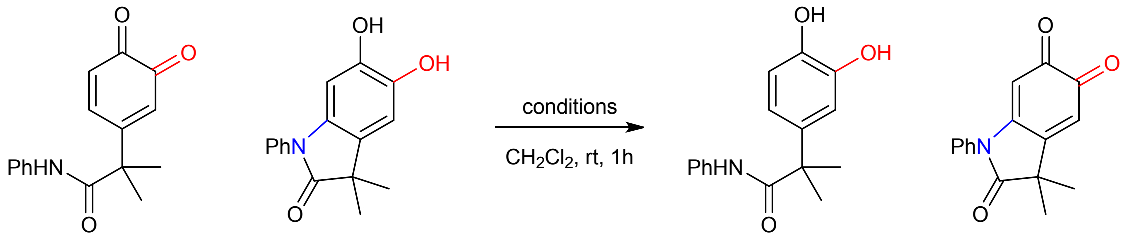

Q1 **C3** **C1** **Q3**

entry[a]	Conditions	Conversion (%) Q1	C3	Yield (%)[b] C1	Q3
1	N_2	100	100	60	76
2	O_2	100	100	54	77
3	20 mol% DBED, N_2	100	100	63	88
4	20 mol% DBED, O_2	100	100	46	94

[a] Reactions performed with 0.1 mmol of **Q1**. [b] Product yield determined by ^{1}H-NMR using hexamethylbenzene as an internal standard.

General Procedure (Entry 1 and 3): A flame-dried, 25 mL round-bottom flask equipped with a Teflon-coated stir bar and a rubber septum was charged with **Q1** (0.1 mmol, 26.9 mg, 1.0 equiv), **C3** (0.1 mmol, 26.9 mg, 1.0 equiv) and hexamethylbenzene (2.4 mg, 0.015 mmol, internal NMR standard) followed by the addition of dry and degassed CH_2Cl_2 (10 mL) via syringe. For entry 3, N,N'-di-*tert*-butylethylenediamine (DBED) (4.4 μL, 0.02 mmol, 20 mol%) was then added via syringe and the resulting mixture was stirred at room temperature (20-23 °C) for 1h. It was then quenched by the addition of $NaHSO_4$ (10 mL, 10% by weight aqueous solution) and extracted with CH_2Cl_2 (3 × 10 mL). The combined organic fractions were then dried over $MgSO_4$, filtered and concentrated *in vacuo* to afford the crude residue which was analyzed directly by ^{1}H-NMR.

General Procedure (Entry 2 and 4): A flame-dried, 25 mL Radley tube equipped with a Teflon-coated stir bar and a rubber septum was charged with, **Q1** (0.1 mmol, 26.9 mg, 1.0 equiv), **C3** (0.1 mmol, 26.9 mg, 1.0 equiv) and hexamethylbenzene (2.4 mg, 0.015 mmol, internal NMR standard). The reaction vessel was then purged with a steady stream of N_2 for 5 min, prior to the addition of dry and degassed CH_2Cl_2 (10 mL) via syringe. For entry 4, N,N'-di-*tert*-butylethylenediamine (DBED) (4.4 μL, 0.02 mmol, 20 mol%) was then added via syringe. The rubber septum was then rapidly removed and replaced with a Radley cap, which was connected to a tank of O_2 and pressurized to 1 atm. Under a constant pressure of O_2 (1

atm), the reaction was vented 3 times for 10 s to remove N_2. The reaction mixture was then stirred at room temperature (20-23 °C) for 1h, depressurized by opening to the atmosphere and quenched by the addition of $NaHSO_4$ (10 mL, 10% by weight aqueous solution). The phases were then separated and the aqueous phase was extracted with CH_2Cl_2 (3 × 10 mL). The combined organic fractions were then dried over $MgSO_4$, filtered and concentrated *in vacuo* to afford the crude residue which was analyzed directly by ^{1}H-NMR.

e) Oxidative C-O Coupling (Table S5)

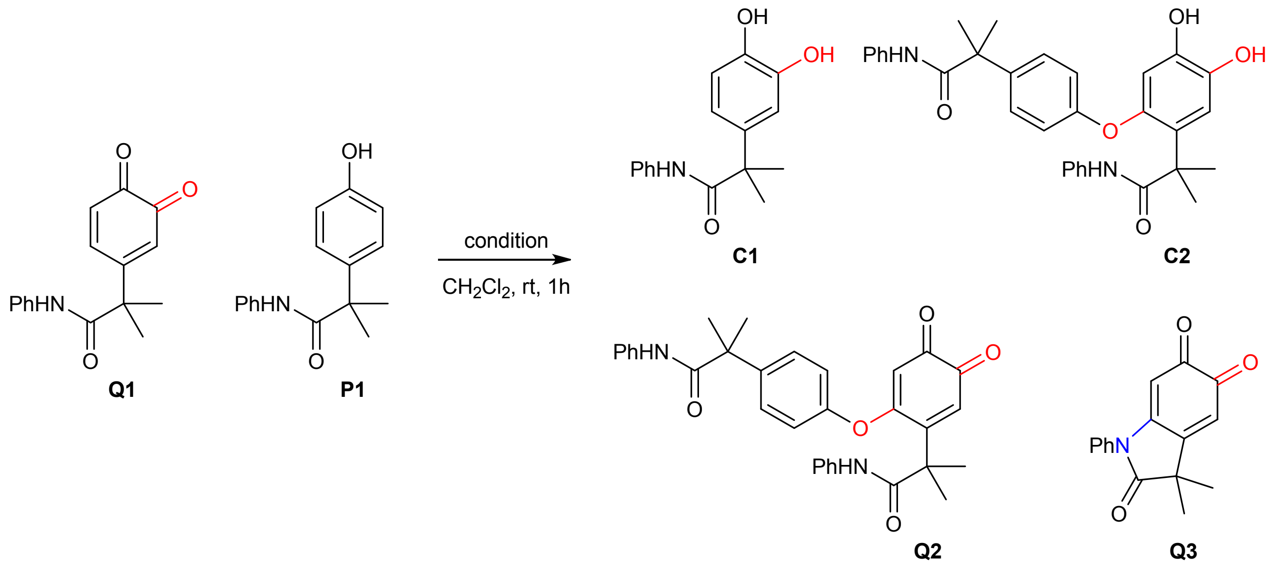

entry[a]	condition	conversion (**Q1**) (%)	conversion (**P1**) (%)	Yield (%)[b] C1	C2	Q2	Q3
1	DBED (20 mol%)	100	4	36	0	0	50
2	[Cu(MeCN)$_4$(PF$_6$)] (1 equiv), DBED (1.2 equiv)	89	16	55	0	13	6
3	[Cu(MeCN)$_4$(PF$_6$)] (1 equiv), DBED (1.2 equiv), O_2 (1 atm)	100	93	0	0	57	24
4	[Cu(MeCN)$_4$(PF$_6$)] (1 equiv), DBED (1.0 equiv), O_2 (1 atm)	100	100	0	0	80	4

[a] Reactions performed with 0.1 mmol of **Q1**. [b] Product yield determined by ^{1}H-NMR using hexamethylbenzene as an internal standard.

Procedure (Entry 1): A flame-dried, 25 mL round-bottom flask equipped with a Teflon-coated stir bar and a rubber septum was charged with **Q1** (0.1 mmol, 26.9 mg, 1.0 equiv), **P1** (0.1 mmol, 25.5 mg, 1.0 equiv) and hexamethylbenzene (2.4 mg, 0.015 mmol, internal NMR standard) followed by the addition of dry and degassed CH_2Cl_2 (10 mL) via syringe. N,N'-di-*tert*-butylethylenediamine (DBED) (4.4 µL, 0.02 mmol, 20 mol%) was then added via syringe and the resulting mixture was stirred at room temperature (20-23 °C) for 1h. It was then quenched by the addition of $NaHSO_4$ (10 mL, 10% by weight aqueous solution) and extracted with CH_2Cl_2 (3 × 10 mL). The combined organic fractions were then dried over $MgSO_4$, filtered and concentrated *in vacuo* to afford the crude residue which was analyzed directly by ^{1}H-NMR.

Procedure (Entry 2): A flame-dried, 25 mL round-bottom flask equipped with a Teflon-coated stir bar and a rubber septum was charged with compound **Q1** (0.1 mmol, 26.9 mg, 1.0 equiv) and then purged with a steady stream of N_2 for 5 min, prior to the addition of dry and degassed CH_2Cl_2 (5 mL) and then cool to -78 °C. In a separate, flame-dried 10 mL microwave vial equipped with a Teflon-coated stir bar and a rubber septum was charged with [Cu(MeCN)$_4$](PF$_6$) (0.1 mmol, 37.3 mg, 1.0 equiv) and then purged with a steady stream of N_2 for 5 min, prior to the addition of dry and degassed CH_2Cl_2 (3 mL). DBED (0.1 mmol, 21.6 µL, 1.0 equiv) was then added to the copper solution dropwise at room temperature, causing dramatic color change from colorless to yellow. The resulting yellow solution was added to the solution of **Q1** in

CH_2Cl_2 at - 78 °C dropwise, and resulting a purple solution immediately. The solution was stirred at - 78 °C for 10 min, warmed to rt, at which temperature a solution of **P1** (0.1 mmol, 25.5 mg, 1.0 equiv) and DBED (0.02 mmol, 4.4 μL, 0.2 equiv) in CH_2Cl_2 (2 mL) was added. The resulting mixture was stirred at room temperature (20-23 °C) for 1h. It was then quenched by the addition of $NaHSO_4$ (10 mL, 10% by weight aqueous solution) and extracted with CH_2Cl_2 (3 × 10 mL). The combined organic fractions were then dried over $MgSO_4$, filtered and concentrated *in vacuo* to afford the crude residue which was analyzed directly by ^{1}H-NMR.

General Procedure (Entry 3 and 4): A flame-dried, 25 mL Radley tube equipped with a Teflon-coated stir bar and a rubber septum was charged with compound **Q1** (0.1 mmol, 26.9 mg, 1.0 equiv) and then purged with a steady stream of N_2 for 5 min, prior to the addition of dry and degassed CH_2Cl_2 (5 mL) and then cool to -78 °C. In a separate, flame-dried 10 mL microwave vial equipped with a Teflon-coated stir bar and a rubber septum was charged with $[Cu(MeCN)_4](PF_6)$ (0.1 mmol, 37.3 mg, 1.0 equiv) and then purged with a steady stream of N_2 for 5 min, prior to the addition of dry and degassed CH_2Cl_2 (3 mL). DBED (0.1 mmol, 21.6 μL, 1.0 equiv) was then added to the copper solution dropwise at room temperature, causing dramatic color change from colorless to yellow. The resulting yellow solution was added to the solution of **Q1** in CH_2Cl_2 at - 78 °C dropwise, and resulting a purple solution immediately. The solution was stirred at - 78 °C for 10 min, warmed to rt, at which temperature a solution of **P** (0.1 mmol, 25.5 mg, 1.0 equiv) [and for entry 3, DBED (0.02 mmol, 4.4 μL, 0.2 equiv)] in CH_2Cl_2 (2 mL) was added. The rubber septum was then rapidly removed and replaced with a Radley cap, which was connected to a tank of O_2 and pressurized to 1 atm. Under a constant pressure of O_2 (1 atm), the reaction was vented 3 times for 10 s to remove N_2. The reaction mixture was then stirred at room temperature (20-23 °C) for 1h, depressurized by opening to the atmosphere and quenched by the addition of $NaHSO_4$ (10 mL, 10% by weight aqueous solution). The phases were then separated and the aqueous phase was extracted with CH_2Cl_2 (3 × 10 mL). The combined organic fractions were then dried over $MgSO_4$, filtered and concentrated *in vacuo* to afford the crude residue which was analyzed directly by ^{1}H-NMR.

f) Cyclization from para-Hydroxyquinone (HQ) Intermediate

Exposure of compound HQ to the standard oxidation conditions:

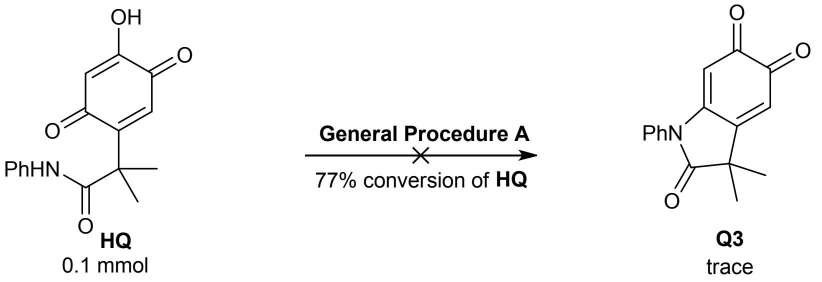

Procedure: A flame-dried, 25 mL Radley tube equipped with a Teflon-coated stir bar and a rubber septum was charged with **HQ** (28.5 mg, 0.1 mmol, 1.0 equiv) and hexamethylbenzene (2.4 mg, 0.015 mmol, internal NMR standard). The reaction vessel was then purged with a steady stream of N_2 for 5 min. In a separate, flame-dried 5 mL microwave vial $[Cu(CH_3CN)_4](PF_6)$ (1.5 mg, 0.004 mmol, 4 mol%) and N,N'-di-*tert*-butylethylenediamine (DBED) (4.4 μL, 0.02 mmol, 20 mol%) were dissolved in the indicated solvent (0.5 mL) to afford a homogeneous pink solution. This solution was then added to the Radley tube via syringe to afford a homogeneous solution. The rubber septum was then rapidly removed and replaced with a Radley cap, which was connected to a tank of O_2 and pressurized to 1 atm. Under a constant pressure of O_2 (1 atm), the reaction was vented 3 times for 10 s to remove N_2. The reaction mixture was then stirred at room temperature (20-23 °C) for 4h, depressurized by opening to the atmosphere and quenched by the addition of $NaHSO_4$ (10 mL, 10% by weight aqueous solution). The phases were then separated and the aqueous phase was extracted with CH_2Cl_2 (3 × 10 mL). The combined organic fractions were then dried over $MgSO_4$, filtered and concentrated *in vacuo* to afford the crude residue which was analyzed directly by ^{1}H-NMR. The crude NMR showed consumption of 77% of starting material **HQ**, and only trace amounts (< 1% NMR yield) of **Q3**.

Conversion of compound HQ with DBED:

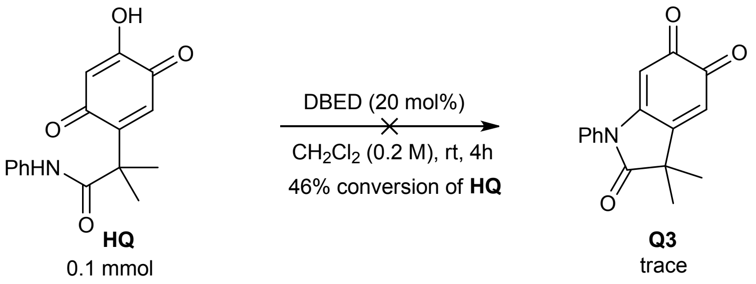

Procedure: A flame-dried 5 mL microwave vial equipped with a Teflon-coated stir bar and a rubber septum was charged with compound **S1** (0.1 mmol, 28.5 mg, 1.0 equiv) and hexamethylbenzene (2.4 mg, 0.015 mmol, internal NMR standard) in dry and degassed CH_2Cl_2 (0.5 mL). N,N'-di-*tert*-butylethylenediamine (4.4 μL, 0.02 mmol, 20 mol%) was added and the resulting mixture was stirred at room temperature (20-23 °C) for 4h. The reaction mixture was then quenched by the addition of $NaHSO_4$ (10 mL, 10% by weight aqueous solution) and extracted with CH_2Cl_2 (3 × 10 mL). The combined organic fractions were then dried over $MgSO_4$, filtered and concentrated *in vacuo* to afford an orange residue which was analyzed directly by ^{1}H-NMR. The crude NMR showed consumption of 46% of starting material **HQ**, and only a trace amount (< 1% NMR yield) of **Q3**.

g) Control Experiments on catechol C2 and C3

Exposure of compound C2 to the standard oxidation conditions:

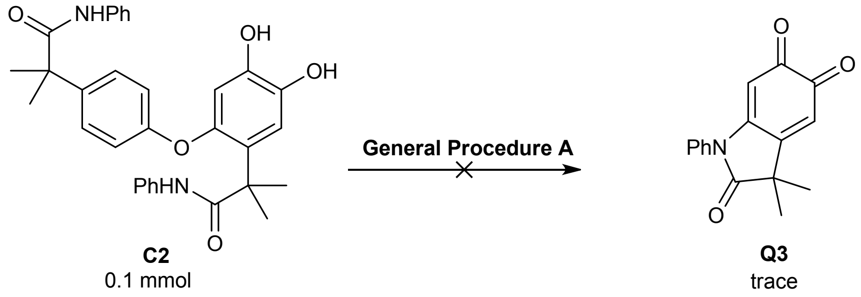

Procedure: A flame-dried, 25 mL Radley tube equipped with a Teflon-coated stir bar and a rubber septum was charged with **C2** (52.5 mg, 0.1 mmol, 1.0 equiv) and hexamethylbenzene (2.4 mg, 0.015 mmol, internal NMR standard). The reaction vessel was then purged with a steady stream of N_2 for 5 min. In a separate, flame-dried 5 mL microwave vial $[Cu(CH_3CN)_4](PF_6)$ (1.5 mg, 0.004 mmol, 4 mol%) and N,N'-di-*tert*-butylethylenediamine (DBED) (4.4 μL, 0.02 mmol, 20 mol%) were dissolved in the indicated solvent (0.5 mL) to afford a homogeneous pink solution. This solution was then added to the Radley tube via syringe to afford a homogeneous solution. The rubber septum was then rapidly removed and replaced with a Radley cap, which was connected to a tank of O_2 and pressurized to 1 atm. Under a constant pressure of O_2 (1 atm), the reaction was vented 3 times for 10 s to remove N_2. The reaction mixture was then stirred at room temperature (20-23 °C) for 4h, depressurized by opening to the atmosphere and quenched by the addition of $NaHSO_4$ (10 mL, 10% by weight aqueous solution). The phases were then separated and the aqueous phase was extracted with CH_2Cl_2 (3 × 10 mL). The combined organic fractions were then dried over $MgSO_4$, filtered and concentrated *in vacuo* to afford the crude residue which was analyzed directly by ^{1}H-NMR. The crude NMR showed only trace amounts (< 1% NMR yield) of **Q3** was formed.

Oxidation of P1 under standard condition in the presence of C2:

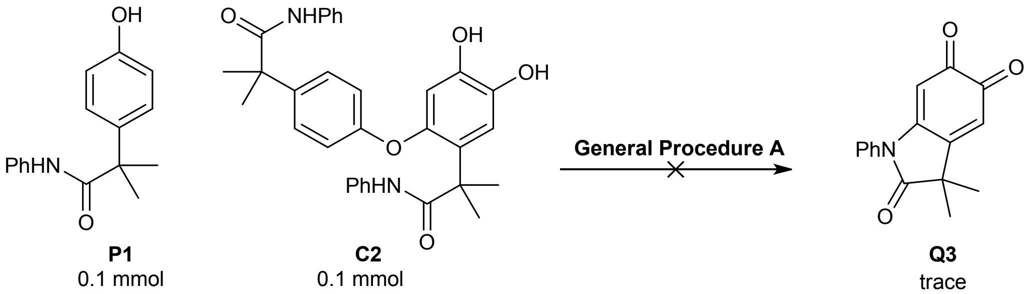

Procedure: A flame-dried, 25 mL Radley tube equipped with a Teflon-coated stir bar and a rubber septum was charged with **P1** (25.5 mg, 0.1 mmol, 1.0 equiv), **C2** (52.5 mg, 0.1 mmol, 1.0 equiv) and hexamethylbenzene (2.4 mg, 0.015 mmol, internal NMR standard). The reaction vessel was then purged with a steady stream of N_2 for 5 min. In a separate, flame-dried 5 mL microwave vial $[Cu(CH_3CN)_4](PF_6)$ (1.5 mg, 0.004 mmol, 4 mol%) and N,N'-di-*tert*-butylethylenediamine (DBED) (4.4 μL, 0.02 mmol, 20 mol%) were dissolved in the indicated solvent (0.5 mL) to afford a homogeneous pink solution. This solution was then added to the Radley tube via syringe to afford a homogeneous solution. The rubber septum was then rapidly removed and replaced with a Radley cap, which was connected to a tank of O_2 and pressurized to 1 atm. Under a constant pressure of O_2 (1 atm), the reaction was vented 3 times for 10 s to remove N_2. The reaction mixture was then stirred at room temperature (20-23 °C) for 4h, depressurized by opening to the atmosphere and quenched by the addition of $NaHSO_4$ (10 mL, 10% by weight aqueous solution). The phases were then separated and the aqueous phase was extracted with CH_2Cl_2 (3 × 10 mL). The combined organic fractions were then dried over $MgSO_4$, filtered and concentrated *in vacuo* to afford the crude residue which was analyzed directly by ^{1}H-NMR. The crude NMR showed only trace amounts (< 1% NMR yield) of **Q3** was formed.

Exposure of compound C3 to the standard oxidation conditions:

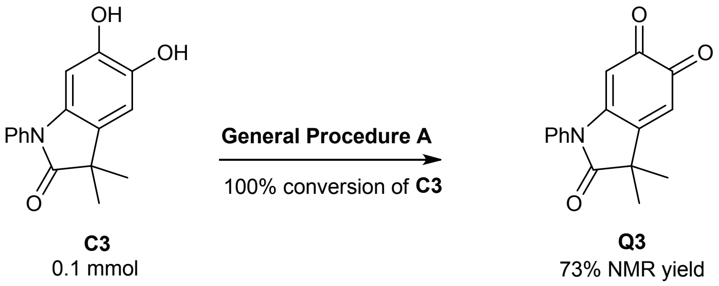

Procedure: A flame-dried, 25 mL Radley tube equipped with a Teflon-coated stir bar and a rubber septum was charged with **C3** (26.9 mg, 0.1 mmol, 1.0 equiv) and hexamethylbenzene (2.4 mg, 0.015 mmol, internal NMR standard). The reaction vessel was then purged with a steady stream of N_2 for 5 min. In a separate, flame-dried 5 mL microwave vial $[Cu(CH_3CN)_4](PF_6)$ (1.5 mg, 0.004 mmol, 4 mol%) and N,N'-di-*tert*-butylethylenediamine (DBED) (4.4 μL, 0.02 mmol, 20 mol%) were dissolved in the indicated solvent (0.5 mL) to afford a homogeneous pink solution. This solution was then added to the Radley tube via syringe to afford a homogeneous solution. The rubber septum was then rapidly removed and replaced with a Radley cap, which was connected to a tank of O_2 and pressurized to 1 atm. Under a constant pressure of O_2 (1 atm), the reaction was vented 3 times for 10 s to remove N_2. The reaction mixture was then stirred at room temperature (20-23 °C) for 4h, depressurized by opening to the atmosphere and quenched by the addition of $NaHSO_4$ (10 mL, 10% by weight aqueous solution). The phases were then separated and the aqueous phase was extracted with CH_2Cl_2 (3 × 10 mL). The combined organic fractions were then dried over $MgSO_4$, filtered and concentrated *in vacuo* to afford the crude residue which was analyzed directly by ^{1}H-NMR. The crude NMR showed 100% conversion of **C3** and 73% NMR yield of **Q3** was formed.

h) Preparation of the Compounds for Mechanism Study

Preparation of catechol C1:

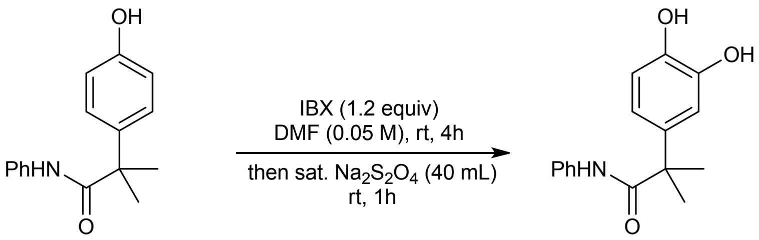

P1
Chemical Formula: $C_{16}H_{17}NO_2$
Molecular Weight: 255.3117

C1
Chemical Formula: $C_{16}H_{17}NO_3$
Molecular Weight: 271.3111

Procedure: The following procedure includes slight modifications to the original procedure of Pettus.[1] A flame-dried, 100 mL round-bottom flask equipped with a Teflon-coated stir bar and a rubber septum was charged with compound **P1** (2 mmol, 510.6 mg, 1.0 equiv) and IBX (2.4 mmol, 672.0 mg, 1.2 equiv) and then purged with a steady stream of N_2 for 5 min, prior to the addition of dry and degassed DMF (40 mL). The resulting suspension was allowed to stir at room temperature for 4h, and quenched with saturated $Na_2S_2O_4$ (40 mL), and stirred for another 1h at room temperature. The mixture was extracted with EtOAc (3 × 50 mL). Combined organic layers were washed with water (150 mL), dried over $MgSO_4$, filtered, and concentrated *in vacuo*. The crude product was purified on silica gel using 40% ethyl acetate in hexanes as eluent to afford **C1** (229.8 mg, 0.85 mmol, 42% yield) as a white solid.

Characterization:

R_f = (ethyl acetate/hexane 1:2): 0.18; **IR** (neat) ν = 3367, 3259, 2966, 1631, 1594, 1520, 1443, 1291, 1258, 1229, 1194, 1119 cm^{-1}; **^{1}H NMR** (500 MHz, acetone-d^6) δ 8.22 (br. s, 1H), 7.87 (d, *J* = 2.0 Hz, 2H), 7.61 (d, *J* = 7.7 Hz, 2H), 7.25 (t, *J* = 8.0 Hz, 2H), 7.02 (t, *J* = 7.4 Hz, 1H), 6.88 (d, *J* = 2.2 Hz, 1H), 6.80 (d, *J* = 8.3 Hz, 1H), 6.75 (dd, *J* = 8.3, 2.2 Hz, 1H), 1.56 (s, 6H); **^{13}C NMR** (126 MHz, acetone-d^6) δ 175.3, 144.9, 143.8, 139.6, 137.2, 128.4, 123.1, 119.6, 117.5, 115.2, 113.5, 47.0, 26.7; **HRMS**: Calcd. for $C_{16}H_{17}NO_3$ $[M+Na]^+$ = 294.1101 m/z, found = 294.1109 m/z.

Preparation of quinone Q1:

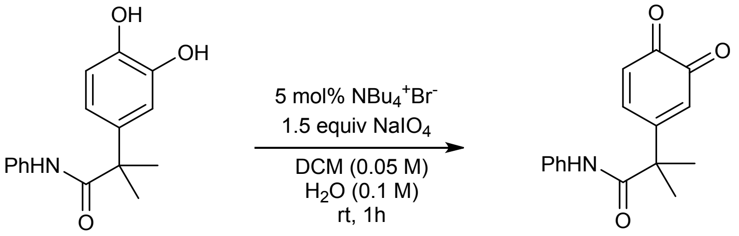

C1
Chemical Formula: $C_{16}H_{17}NO_3$
Molecular Weight: 271.3111

Q1
Chemical Formula: $C_{16}H_{15}NO_3$
Molecular Weight: 269.2952

Procedure: A flame-dried, 25 mL round-bottom flask equipped with a Teflon-coated stir bar and a rubber septum was charged with compound **C1** (0.3 mmol, 81.4 mg, 1.0 equiv), tetrabutylammonium bromide (0.015 mmol, 4.8 mg, 0.05 equiv) and sodium periodate (0.45 mmol, 96.2 mg, 1.5 equiv) and then purged with a steady stream of N_2 for 5 min, prior to the addition of dry and degassed DCM (6 mL) and water (3 mL). The resulting biphasic solution was allowed to stir at room temperature for 1h. Color change was observed within 5 min resulting a brown-red mixture. The mixture was poured on water (10 mL) and extracted with CH_2Cl_2 (3 × 10 mL). Combined organic layers were dried over $MgSO_4$, filtered, and concentrated *in vacuo*. The crude product was directly characterized and used for the next step. Please note that **Q1** is not stable at room temperature. Significant amounts of decomposition are observed after storage on the bench top for 12h. For best results, use immediately following preparation.

[1] Magdziak, D.; Rodriguez, A. A.; Van De Water, R. W.; Pettus, T. R. R. *Org. Lett.* **2002**, *4*, 285-288.

Characterization:

R$_f$ = (ethyl acetate/hexane 1:3): 0.15; **IR** (neat) ν = 3323, 3017, 2977, 2932, 1663, 1598, 1522, 1500, 1439, 1367, 1313, 1215 cm^{-1}; **^{1}H NMR** (500 MHz, $CDCl_3$) δ 7.53 (d, *J* = 7.9 Hz, 2H), 7.46 (br. s, 1H), 7.34 (t, *J* = 7.7 Hz, 2H), 7.16 (t, *J* = 7.3 Hz, 1H), 7.09 (dd, *J* = 10.2, 1.6 Hz, 1H), 6.54 – 6.30 (m, 2H), 1.60 (s, 6H); **^{13}C NMR** (126 MHz, $CDCl_3$) δ 179.8, 179.6, 170.7, 156.8, 140.2, 137.4, 130.3, 129.1, 125.3, 125.1, 120.3, 48.6, 24.0; **HRMS**: Calcd. for $C_{16}H_{15}NO_3$ $[M+H]^+$ = 270.1125 m/z, found = 270.1119 m/z.

Preparation of quinone Q2:

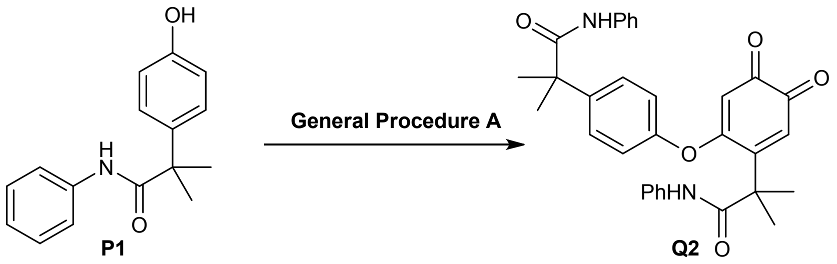

P1
Chemical Formula: $C_{16}H_{17}NO_2$
Molecular Weight: 255.3117

Q2
Chemical Formula: $C_{32}H_{30}N_2O_5$
Molecular Weight: 522.5910

Procedure: The reaction was carried out according to the General Procedure A with the modified amount of N,N'-di-*tert*-butylethylenediamine (DBED) (0.05 mmol, 10.9 μL, 5 mol%) and reaction time (2h).

Amounts of Reagents:

P1 (255.3 mg, 1.0 mmol)

$[Cu(CH_3CN)_4](PF_6)$ (14.9 mg, 0.04 mmol, 4 mol%)

N,N'-di-*tert*-butylethylenediamine (10.9 μL, 0.05 mmol, 5 mol%)

CH_2Cl_2 (5 mL, 0.2 M)

Purification: 30-50% EtOAc in hexanes.

Yield of Product:

Q2: 135.9 mg, 0.26 mmol, 52% yield, dark-red solid.

Characterization:

R$_f$ = (ethyl acetate/hexane 1:2): 0.10; **IR** (neat) ν = 3349, 2976, 1643, 1597, 1562, 1531, 1499, 1437, 1366, 1311, 1234, 1170, 856, 752, 745, 690 cm^{-1}; **^{1}H NMR** (400 MHz, $CDCl_3$) δ 7.68 (s, 1H), 7.51 (d, *J* = 7.8 Hz, 2H), 7.42 (d, *J* = 8.7 Hz, 2H), 7.37 (d, *J* = 7.7 Hz, 2H), 7.27 (d, *J* = 6.6 Hz, 4H), 7.09 (dd, *J* = 13.2, 7.3 Hz, 2H), 6.97 (d, *J* = 8.7 Hz, 2H), 6.78 (s, 1H), 6.46 (s, 1H), 5.55 (s, 1H), 1.70 (s, 6H), 1.63 (s, 6H); **^{13}C NMR** (75 MHz, $CDCl_3$) δ 180.4, 178.2, 174.5, 172.1, 168.5, 154.2, 150.7, 143.9, 137.8, 137.6, 129.0, 128.9, 128.4, 127.3, 124.6, 124.4, 121.3, 120.2, 119.8, 107.2, 47.7, 47.3, 27.2, 25.7; **HRMS**: Calcd. for $C_{32}H_{30}N_2O_5$ $[M+Na]^+$ = 545.2047 m/z, found = 545.2043 m/z.

Preparation of catechol C2:

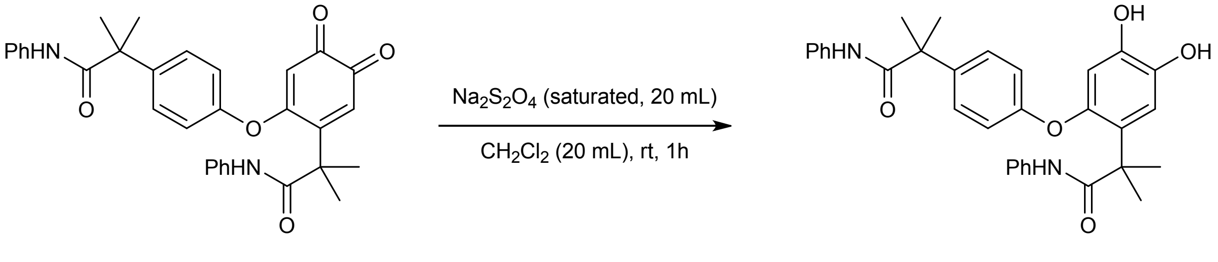

Q2
Chemical Formula: $C_{32}H_{30}N_2O_5$
Molecular Weight: 522.5910

C2
Chemical Formula: $C_{32}H_{32}N_2O_5$
Molecular Weight: 524.6069

Procedure: A flame-dried, 25 mL round-bottom flask equipped with a Teflon-coated stir bar and a rubber septum was charged with compound **Q2** (2.82 mmol, 1.47 g) in CH_2Cl_2 (20 mL). A saturated aqueous solution of $Na_2S_2O_4$ (20 mL) was

then added, and the resulting biphasic mixture was stirred for 1h under N_2. The phases were then separated and the aqueous phase was extracted with CH_2Cl_2 (3 × 20 mL). The combined organic fractions were then dried over $MgSO_4$, filtered and concentrated *in vacuo*. The residue was purified on silica gel using 50% ethyl acetate in hexanes as eluent to afford **C2** (1.46g, 2.79 mmol, 99% yield) as a brown oil.

Characterization:

R_f = (ethyl acetate/hexane 1:1): 0.35; **IR** (neat) ν = 3303, 2970, 1738, 1658, 1596, 1499, 1436, 1365, 1312, 1291, 1228, 1217, 1175, 1144 cm^{-1}; **^{1}H NMR** (500 MHz, $CDCl_3$) δ 7.31 – 7.28 (m, 3H), 7.27 – 7.23 (m, 4H), 7.20 – 7.12 (m, 5H), 7.11 – 6.96 (m, 4H), 6.93 (s, 1H), 6.84 (d, *J* = 8.7 Hz, 2H), 6.49 (s, 1H), 1.56 (s, 6H), 1.52 (s, 6H); **^{13}C NMR** (126 MHz, $CDCl_3$) δ 176.8, 176.3, 156.0, 146.9, 144.0, 140.5, 138.6, 137.7, 137.4, 128.9, 128.7, 127.5, 127.0, 124.7, 124.3, 120.4, 120.2, 118.1, 113.8, 107.6, 47.4, 45.6, 27.2, 26.3; **HRMS**: Calcd. for $C_{32}H_{32}N_2O_5$ $[M+H]^+$ = 525.2384 m/z, found = 525.2377 m/z.

Preparation of *para*-hydroxyquinone HQ:

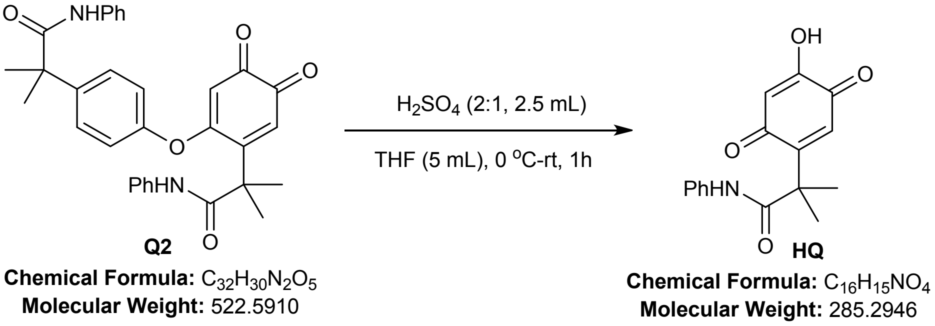

Procedure: A flame-dried, 25 mL round-bottom flask equipped with a Teflon-coated stir bar and a rubber septum was charged with compound **Q2** (0.5 mmol, 261.3 mg, 1.0 equiv) in THF (5 mL). H_2SO_4 (2.5 mL, 2:1 volume ratio of concentrated H_2SO_4 to H_2O) was added dropwise to the solution at 0 °C. The mixture was warmed to room temperature, and stirred for 1h. It was then poured on saturated $NaHCO_3$ solution (100 mL) and CH_2Cl_2 (30 mL). The organic layers were separated and extracted with saturated $NaHCO_3$ solution (2 × 30 mL). The combined red aqueous layers were then washed with CH_2Cl_2 (2 × 30 mL). The aqueous layer was then acidified with concentrated HCl via dropwise addition until the color changed from red to yellow (pH < 2), and extracted with CH_2Cl_2 (3 × 50 mL). The combined organic fractions were then dried over $MgSO_4$, filtered and concentrated *in vacuo*. The crude product was then purified on silica gel using ethyl acetate as eluent to afford **HQ** (62.8 mg, 0.22 mmol, 44% yield) as a yellow solid.

Characterization:

R_f = (ethyl acetate): 0.47; **IR** (neat) ν = 3356, 1653, 1599, 1541, 1500, 1440, 1324, 1206, 1162, 1138, 1031, 895, 874, 752, 689, 654, 511 cm^{-1}; **^{1}H NMR** (400 MHz, $CDCl_3$) δ 7.50 (d, *J* = 7.7 Hz, 2H), 7.38 (br. s, 1H), 7.35 (t, *J* = 7.9 Hz, 2H), 7.14 (t, *J* = 7.4 Hz, 1H), 7.02 (br. s, 1H), 6.80 (s, 1H), 6.11 (s, 1H), 1.62 (s, 6H); **^{13}C NMR** (75 MHz, $CDCl_3$) δ 186.9, 183.6, 173.1, 155.0, 154.2, 137.4, 129.0, 128.3, 124.7, 120.5, 109.1, 46.4, 25.6; **HRMS**: Calcd. for $C_{16}H_{15}NO_4$ $[M-H]^-$ = 284.0928 m/z, found = 284.0926 m/z.

4. Reaction Intermediates SQ1-3

a) Mass Spectrometry of Reaction Solutions

A solution containing **P1** (15.7 mM), $CuPF_6$ (1.4 mM, 9 mol%) and DBED (3.1 mM, 20 mol%) in CH_2Cl_2 was prepared in the glovebox. A portion of the solution was diluted 5-fold and exposed to air, after which 10 µL of acetonitrile was added and the solution was analyzed by ESI-MS. Final concentration of species: [**P1**] = 3.13 mM, [$CuPF_6$] = 0.28 mM, [DBED] = 0.63 mM.

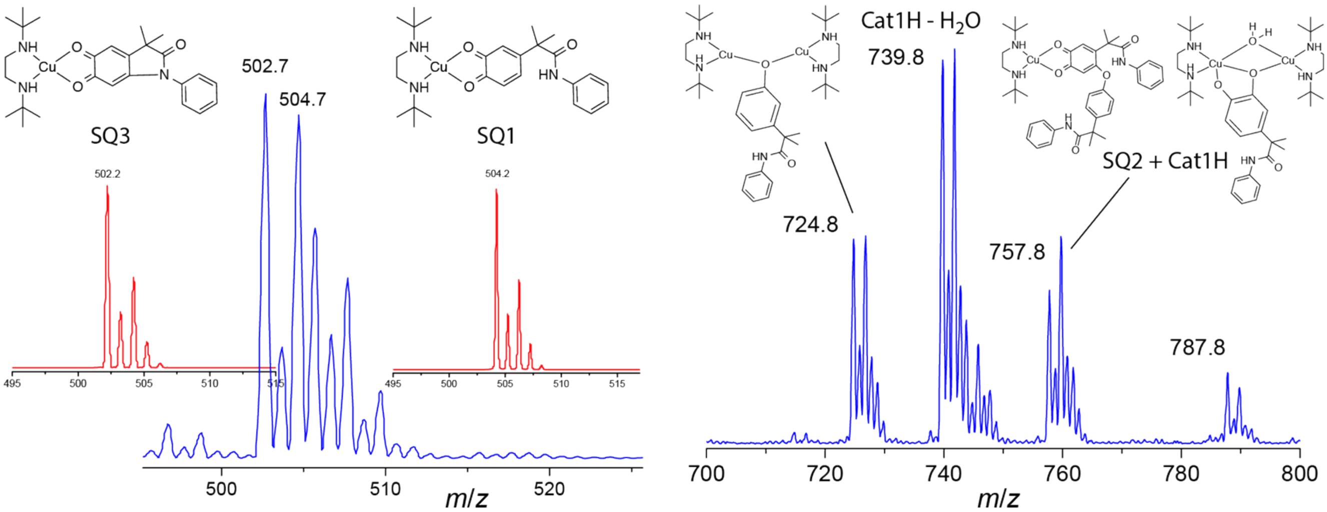

Figure S1. ESI-MS analysis of the above solution after 1 min of oxygenation, showing the signals for **SQ1** and **SQ3** (left) and various species including in the region for **SQ2**.

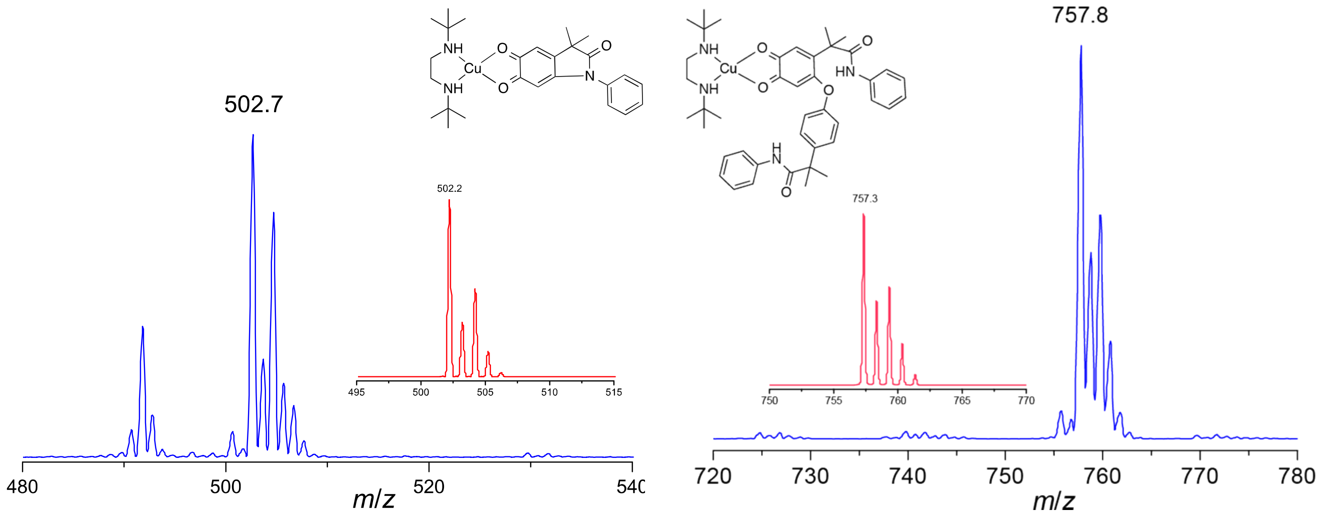

Figure S2. ESI-MS analysis of the above solution after 15 min of oxygenation, showing the signals for **SQ3** (left) and **SQ2** (right).

b) DBEDCu(I) : Q1-3 Binding and UV-Vis Features of SQ1-3

UV-vis titrations were done in CH_2Cl_2 under nitrogen atmosphere (path length = 1.0 cm). The spectra were fit into 1:1 complex:quinone model using multivariate analysis with ReactLab equilibria software.

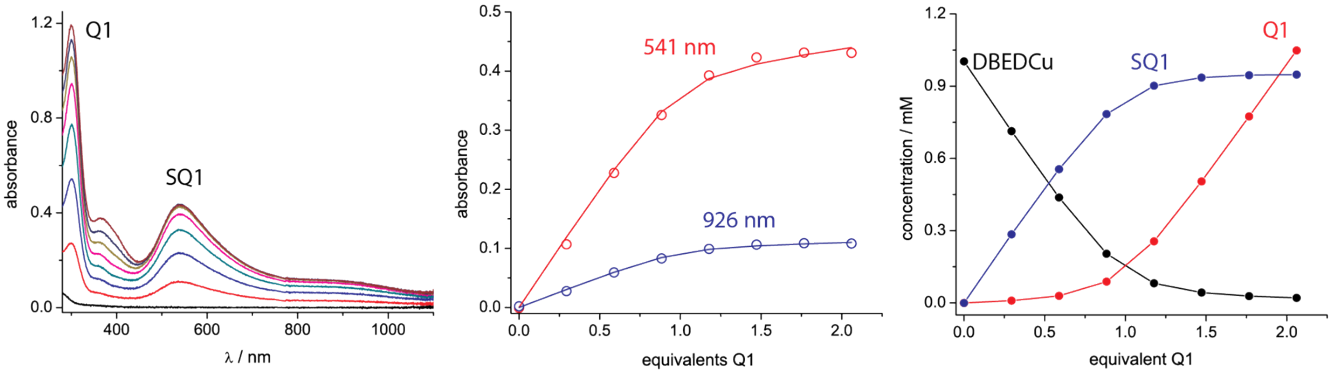

Figure S3. Left: titration of a 1:1 DBED:$CuPF_6$ mixture with **Q1**; [(DBED)$CuPF_6$] = 1.0 mM, [**Q1**]$_{titrant}$ = 59.0 mM. Middle: fitting of absorption maxima using the equilibrium constant for λ_{max} = 541 nm (red) and 926 nm (blue). Right: concentration profiles for [(DBED)Cu]PF_6 (black), **Q1** (red), and **SQ1** (blue).

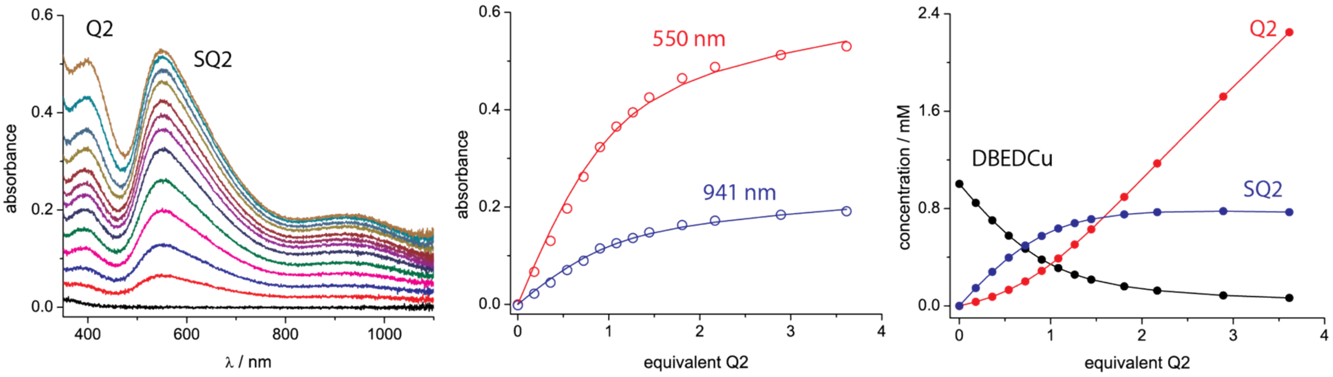

Figure S4. Left: titration of a 1:1 DBED:$CuPF_6$ mixture with **Q2**; [(DBED)$CuPF_6$] = 1.0 mM, [**Q2**] $_{titrant}$ = 18.1 mM. Middle: fitting of absorption maxima using the equilibrium constant for λ_{max} = 550 nm (red) and 941 nm (blue). Right: concentration profiles for [(DBED)Cu]PF_6 (black), **Q2** (red), and **SQ2** (blue).

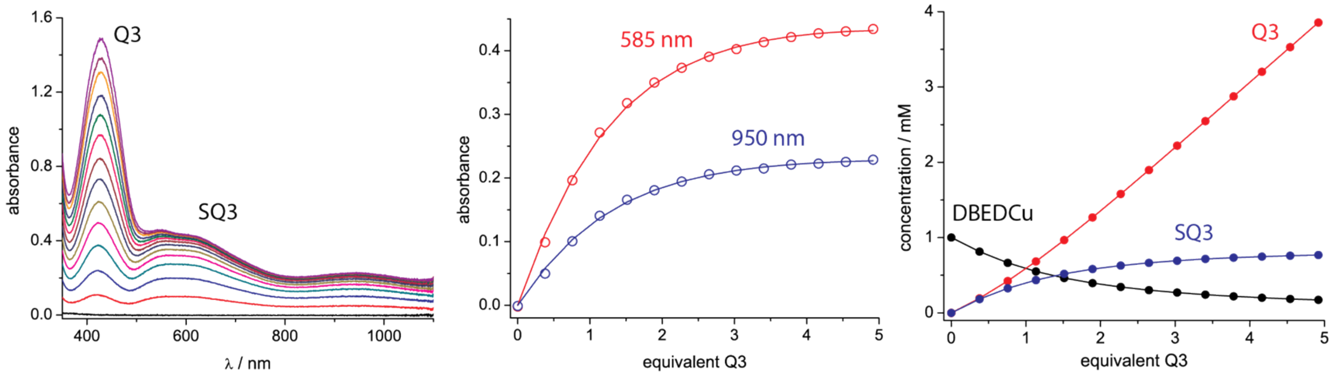

Figure S5. Left: titration of a 1:1 DBED:$CuPF_6$ mixture with **Q3**; [(DBED)$CuPF_6$] = 1.0 mM, [**Q3**] $_{titrant}$ = 75.8 mM. Middle: fitting of absorption maxima using the equilibrium constant for λ_{max} = 585 nm (red) and 950 nm (blue). Right: concentration profiles for [(DBED)Cu]PF_6 (black), **Q3** (red), and **SQ3** (blue).

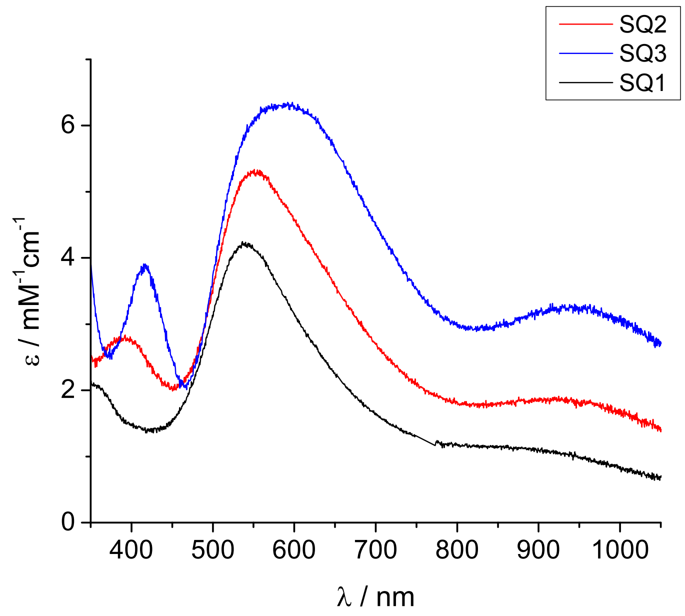

Figure S6. Absorption spectra of **SQ1-3** deduced by the fitting experiments above.

c) Synthesis of SQ3

In a glove box, to a solution of DBED (12.3 mg, 0.071 mmol, 1.1 equiv.) in 2 mL CH_2Cl_2 solid [$(MeCN)_4Cu$](SbF_6) (30 mg, 0.065 mmol, 1 equiv.) was added and the mixture stirred until a homogenous solution was obtained. Solid Q3 (19 mg, 0.071 mmol, 1.1 equiv.) was added and the resulting purple solution was stirred for 20 min. The solution was then added dropwise to 15 mL of stirring pentane upon which purple solid precipitated. The solid was collected, washed with Et_2O and pentane, and dried under vacuum. Yield: 30 mg, 63%. Crystals suitable for X-ray diffraction were grown through layered diffusion of pentane into a concentrated solution of **SQ3** in CH_2Cl_2 at 25 °C.

d) Concentration Profiles During Reaction

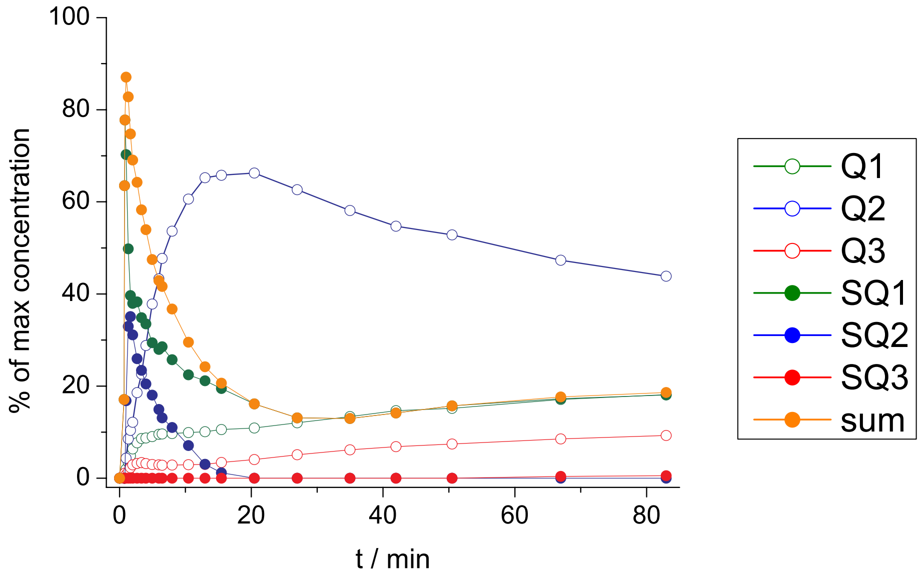

Figure S7. Concentration profile of visible intermediates and products in the oxygenation of **P1** as deduced by fitting UV-vis data. [**P1**] = 15.67 mM, [$CuPF_6$] = 1.25 mM (8 mol%), [DBED] = 1.57 mM (10 mol%), CH_2Cl_2, 25 °C, 1 atm O_2. The y-axis is scaled to the maximum concentration of each species., i.e. $[\textbf{SQ1-3}]_{max}$ = $[CuPF_6]_0$, $[\textbf{Q1}]_{max}$ = $[\textbf{Q3}]_{max}$ = $[\textbf{P1}]_0$ and $[\textbf{Q2}]_{max}$ = 0.5 $[\textbf{P1}]_0$. Thus each point in the graph gives the yield of each species. "sum" represents [**SQ1**] + [**SQ2**] + [**SQ3**].

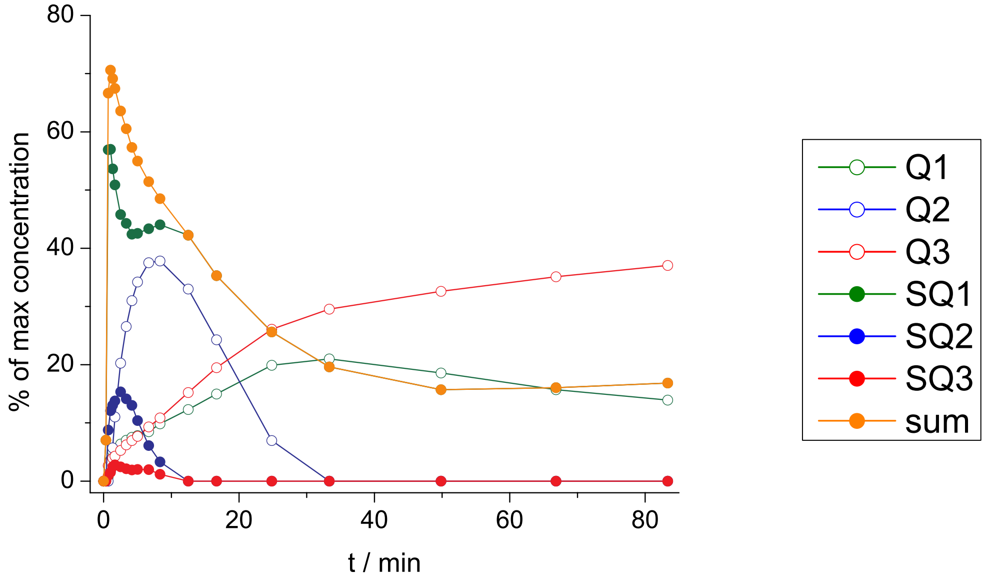

Figure S8. Concentration profile of visible intermediates and products in the oxygenation of **P1** as deduced by fitting UV-vis data. [**P1**] = 15.67 mM, [$CuPF_6$] = 1.42 mM (9 mol%), [DBED] = 3.15 mM (20 mol%), CH_2Cl_2, 25 °C, 1 atm O_2. The y-axis is scaled to the maximum concentration of each species., i.e. [**SQ1-3**]$_{max}$ = [$CuPF_6$]$_0$, [**Q1**]$_{max}$ = [**Q3**]$_{max}$ = [**P1**]$_0$ and [**Q2**]$_{max}$ = 0.5 [**P1**]$_0$. Thus each point in the graph gives the yield of each species. "sum" represents [**SQ1**] + [**SQ2**] + [**SQ3**].

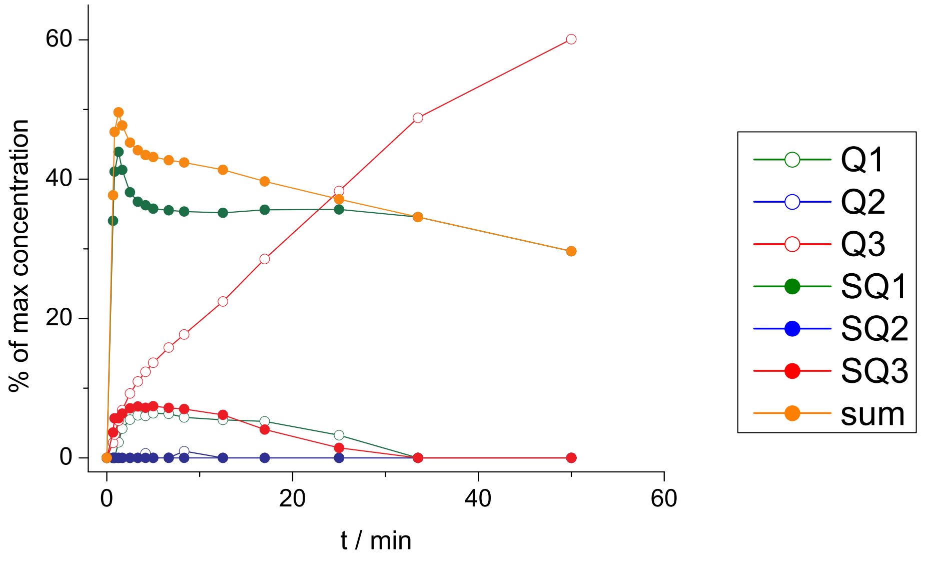

Figure S9. Concentration profile of visible intermediates and products in the oxygenation of **P1** as deduced by fitting UV-vis data. [**P1**] = 15.67 mM, [$CuPF_6$] = 1.37 mM (8.8 mol%), [DBED] = 6.15 mM (40 mol%), CH_2Cl_2, 25 °C, 1 atm O_2. The y-axis is scaled to the maximum concentration of each species., i.e. [**SQ1-3**]$_{max}$ = [$CuPF_6$]$_0$, [**Q1**]$_{max}$ = [**Q3**]$_{max}$ = [**P1**]$_0$ and [**Q2**]$_{max}$ = 0.5 [**P1**]$_0$. Thus each point in the graph gives the yield of each species. "sum" represents [**SQ1**] + [**SQ2**] + [**SQ3**].

e) Use of SQ1-3 as precatalysts

SQ1: To a solution of DBED (4.8 mg, 0.03 mmol) in 1 mL CH_2Cl_2, $CuPF_6$ (10.4 mg, 0.03 mmol) is added and mixture stirred until a homogenous yellow solution is obtained. The solution was cooled to -78 °C under argon and a solution of **Q1** (8 mg, 0.03 mmol) in 0.5 mL of CH_2Cl_2 was added upon which purple semiquinone formed. The mixture was stirred for further 15 min, and then solid ArOH (63 mg, 0.25 mmol) followed by a solution of DBED (5 mg, 0.03 mmol) in 3.5 mL of CH_2Cl_2 were added. The reaction was pressurized with O_2, warmed up to 25 °C and stirred for 4

hours. After completion of reaction, HMB (internal NMR standard) was added and the reaction worked up with 10% $NaHSO_{4(aq)}$. NMR analysis shows >95% yield of **Q3**.

SQ2: To a solution of DBED (4.8 mg, 0.03 mmol) in 1 mL CH_2Cl_2, $CuPF_6$ (10.4 mg, 0.03 mmol) is added and mixture stirred until a homogenous yellow solution is obtained. A solution of **Q2** (14.6 mg, 0.03 mmol) in 0.5 mL of CH_2Cl_2 was added upon which purple semiquinone formed. The mixture was stirred for further 15 min, and then solid ArOH (63 mg, 0.25 mmol) followed by a solution of DBED (5 mg, 0.03 mmol) in 3.5 mL of CH_2Cl_2 were added. The reaction was pressurized with O_2 and stirred for 4 hours. After completion of reaction, HMB (internal NMR standard) was added and the reaction worked up with 10% $NaHSO_4$. NMR analysis shows >95% yield of **Q3**.

SQ3: To a solution of DBED (4.8 mg, 0.03 mmol) in 1 mL CH_2Cl_2, $CuPF_6$ (10.4 mg, 0.03 mmol) is added and mixture stirred until a homogenous yellow solution is obtained. A solution of **Q3** (7.4 mg, 0.03 mmol) in 0.5 mL of CH_2Cl_2 was added upon which purple semiquinone formed. The mixture was stirred for further 15 min, and then solid ArOH (63 mg, 0.25 mmol) followed by a solution of DBED (5 mg, 0.03 mmol) in 3.5 mL of CH_2Cl_2 were added. The reaction was pressurized with O_2 and stirred for 4 hours. After completion of reaction, HMB (internal NMR standard) was added and the reaction worked up with 10% $NaHSO_4$. NMR analysis shows >95% yield of **Q3**.

5. Synthesis and Characterization of the Compounds in Tables 1-3 and Schemes 6-9

a) Reactions in Table 1

Compound Q3:

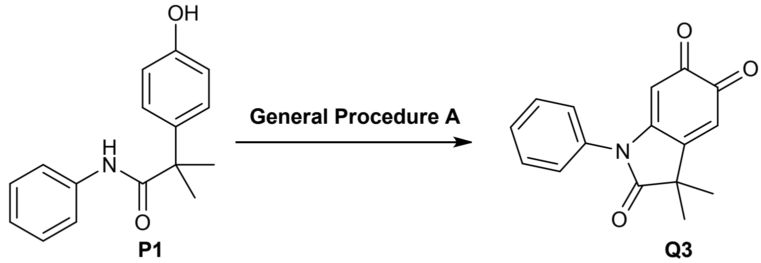

P1 **Chemical Formula:** $C_{16}H_{17}NO_2$ **Molecular Weight:** 255.3117

Q3 **Chemical Formula:** $C_{16}H_{13}NO_3$ **Molecular Weight:** 267.2793

Procedure: The reaction was carried out according to General Procedure A.

Amounts of Reagents:

P1 (255.3 mg, 1.0 mmol)

$[Cu(CH_3CN)_4](PF_6)$ (14.9 mg, 0.04 mmol, 4 mol%)

N,N'-di-*tert*-butylethylenediamine (43.1 μL, 0.20 mmol, 20 mol%)

CH_2Cl_2 (5 mL, 0.2 M)

Purification: 30% EtOAc in hexanes.

Yield of Product:

Q3: 240.3 mg, 0.90 mmol, 90% yield, red solid.

Characterization:

R*f* = (ethyl acetate/hexane 1:2): 0.22; **IR** (neat) ν = 1750, 1681, 1645, 1600, 1588, 1499, 1410, 1240, 1202, 1106, 826, 735, 687, 660 cm^{-1}; **^{1}H NMR** (400 MHz, $CDCl_3$) δ 7.52 (t, *J* = 7.4 Hz, 2H), 7.46 (t, *J* = 7.4 Hz, 1H), 7.27 (d, *J* = 8.6 Hz, 2H), 6.42 (s, 1H), 5.69 (s, 1H), 1.53 (s, 6H); **^{13}C NMR** (75 MHz, $CDCl_3$) δ 179.4, 177.31, 177.25, 155.2, 153.3, 132.1, 129.9, 129.7, 126.7, 123.5, 101.8, 42.3, 24.4; **HRMS**: Calcd. for $C_{16}H_{14}NO_3$ $[M+H]^+$ = 268.0968 m/z, found = 268.0964 m/z. ***The structure was confirmed by X-ray crystallography.***

Large scale synthesis of Q3:

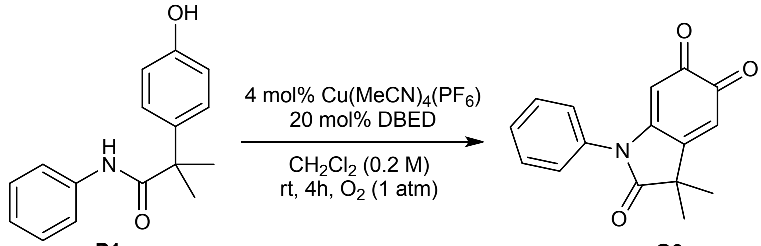

P1 **Chemical Formula:** $C_{16}H_{17}NO_2$ **Molecular Weight:** 255.3117

Q3 **Chemical Formula:** $C_{16}H_{13}NO_3$ **Molecular Weight:** 267.2793

Procedure: A flame-dried, 500 mL round-bottom flask equipped with a Teflon-coated stir bar and a rubber septum was charged with compound **P1** (19.6 mmol, 5.00 g, 1.0 equiv) and then purged with a steady stream of N_2 for 5 min, prior to the addition of dry and degassed CH_2Cl_2 (78 mL). In a separated, flame-dried 50 mL round-bottomed flask $[Cu(CH_3CN)_4](PF_6)$ (292.2 mg, 0.78 mmol, 4 mol%) and N,N'-di-*tert*-butylethylenediamine (845.4 μL, 3.92 mmol, 20 mol%) were dissolved in CH_2Cl_2 (20 mL) to afford a homogeneous pink solution. This solution was then added to the reaction flask via syringe to afford a final volume of 98 mL and a phenol concentration 0.2 M solution. The reaction flask was pressurized with O_2 (1 atm) and stirred at room temperature (20-23 ^oC) for 4 h, then depressurize by opening to the

atmosphere and quenched by the addition of $NaHSO_4$ (200 mL, 10% by weight aqueous solution). The phases were then separated and the aqueous phase was extracted with CH_2Cl_2 (3 × 200 mL). The combined organic fractions were then dried over $MgSO_4$, filtered and concentrated *in vacuo*. The residue was purified on silica gel using 30% ethyl acetate in hexanes as eluent to afford **Q3** (4.94 g, 18.5 mmol, 94% yield) as red solid.

Compound 2a:

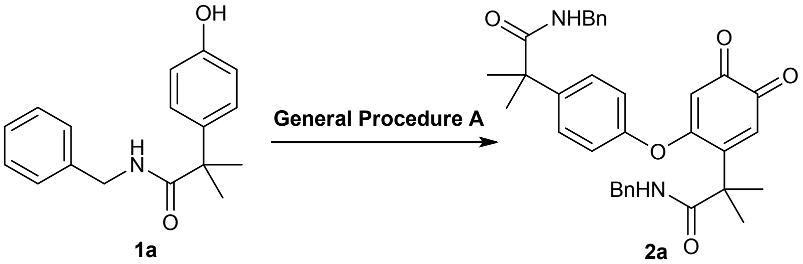

1a
Chemical Formula: $C_{17}H_{19}NO_2$
Molecular Weight: 269.3383

2a
Chemical Formula: $C_{34}H_{34}N_2O_5$
Molecular Weight: 550.6442

Procedure: The reaction was carried out according to the General Procedure A.

Amounts of Reagents:

1a (269.3 mg, 1.0 mmol)

$[Cu(CH_3CN)_4](PF_6)$ (14.9 mg, 0.04 mmol, 4 mol%)

N,N'-di-*tert*-butylethylenediamine (43.1 μL, 0.20 mmol, 20 mol%)

CH_2Cl_2 (5 mL, 0.2 M)

Purification: 30-50% EtOAc in hexanes.

Yield of Product:

2a: 173.5 mg, 0. 32 mmol, 63% yield, red solid.

Characterization:

R*f* = (ethyl acetate/hexane 1:1): 0.17; **IR** (neat) v = 3359, 2975, 2928, 1671, 1649, 1558, 1524, 1502, 1366, 1218, 1172, 1016, 864, 847, 751, 721, 702, 606 cm^{-1}; **^{1}H NMR** (400 MHz, $CDCl_3$) δ 7.40 (d, *J* = 8.6 Hz, 2H), 7.33 – 7.29 (m, 2H), 7.28 – 7.16 (m, 8H), 6.89 (d, *J* = 8.6 Hz, 2H), 6.44 (s, 1H), 6.23 (t, *J* = 5.5 Hz, 1H), 5.61 (t, *J* = 5.3 Hz, 1H), 5.46 (s, 1H), 4.42 (t, *J* = 6.4 Hz, 4H), 1.64 (s, 6H), 1.62 (s, 6H); **^{13}C NMR** (101 MHz, $CDCl_3$) δ 180.3, 178.3, 176.4, 174.2, 168.7, 154.3, 150.7, 144.2, 138.2, 137.9, 128.72, 128.68, 128.4, 127.8, 127.6, 127.54, 127.48, 127.1, 121.0, 107.1, 46.8, 46.5, 43.85, 43.83, 27.2, 25.8; **HRMS**: Calcd. for $C_{34}H_{34}N_2O_5$ $[M+H]^+$ = 551.2540 m/z, found = 551.2544 m/z.

Compound 3a:

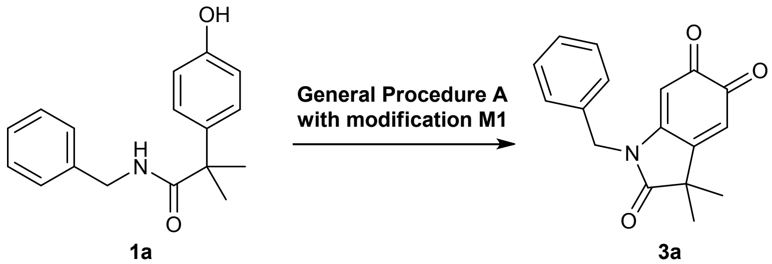

1a
Chemical Formula: $C_{17}H_{19}NO_2$
Molecular Weight: 269.3383

3a
Chemical Formula: $C_{17}H_{15}NO_3$
Molecular Weight: 281.3059

Procedure: The reaction was carried out according to the General Procedure A with modification M1.

Amounts of Reagents:

1a (269.3 mg, 1.0 mmol)

$[Cu(CH_3CN)_4](PF_6)$ (14.9 mg, 0.04 mmol, 4 mol%)

N,N'-di-*tert*-butylethylenediamine (43.1 μL, 0.20 mmol, 20 mol%)

1,8-Diazabicycloundec-7-ene (0.30 mmol, 44.9 μL, 30 mol%)

CH_2Cl_2 (5 mL, 0.2 M)

Purification: 25% EtOAc in hexanes.

Yield of Product:

3a: 172.5 mg, 0.61 mmol, 61% yield, red oil.

Characterization:

R*f* = (ethyl acetate/hexane 1:2): 0.22; **IR** (neat) ν = 3061, 2973, 2931, 2870, 1743, 1685, 1655, 1595, 1496, 1411, 1386, 1325, 1274, 1241, 1202, 1160, 1128, 1079, 992, 904, 825, 748, 725, 697, 658, 617 cm^{-1}; **^{1}H NMR** (500 MHz, $CDCl_3$) δ 7.39 – 7.30 (m, 3H), 7.23 (d, *J* = 6.7 Hz, 2H), 6.39 (s, 1H), 5.83 (s, 1H), 4.85 (s, 2H), 1.51 (s, 6H); **^{13}C NMR** (126 MHz, $CDCl_3$) δ 179.2, 178.0, 176.9, 155.3, 152.3, 133.6, 129.2, 128.6, 127.5, 123.5, 102.0, 44.5, 42.1, 24.4; **HRMS**: Calcd. for $C_{17}H_{15}NO_3$ $[M+H]^+$ = 282.1125 m/z, found = 282.1123 m/z.

Compound 3b:

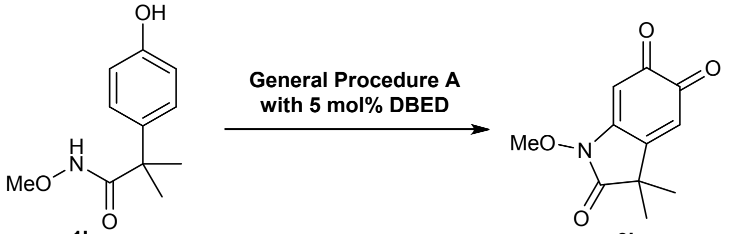

1b **Chemical Formula:** $C_{11}H_{15}NO_3$ **Molecular Weight:** 209.2417

3b **Chemical Formula:** $C_{11}H_{11}NO_4$ **Molecular Weight:** 221.2093

Procedure: The reaction was carried out according to the General Procedure A using N,N'-di-*tert*-butylethylenediamine (DBED) (10.8 μL, 0.05 mmol, 5 mol%) instead of 20 mol%.

Amounts of Reagents:

1b (209.2 mg, 1.0 mmol)

$[Cu(CH_3CN)_4](PF_6)$ (14.9 mg, 0.04 mmol, 4 mol%)

N,N'-di-*tert*-butylethylenediamine (10.8 μL, 0.05 mmol, 5 mol%)

CH_2Cl_2 (5 mL, 0.2 M)

Purification: 30% EtOAc in hexanes.

Yield of Product:

3b: 130.0 mg, 0.59 mmol, 59% yield, red solid.

Characterization:

R*f* = (ethyl acetate/hexane 1:2): 0.32; **IR** (neat) ν = 2964, 2917, 1757, 1687, 1654, 1611, 1392, 1323, 1307, 1269, 1240, 1196, 1171, 1127, 1028, 950, 919, 883, 826, 763, 737, 714, 631 cm^{-1}; **^{1}H NMR** (400 MHz, $CDCl_3$) δ 6.38 (s, 1H), 5.96 (s, 1H), 4.01 (s, 3H), 1.46 (s, 6H); **^{13}C NMR** (75 MHz, $CDCl_3$) δ 179.1, 176.4, 171.7, 151.8, 148.3, 124.4, 99.4, 64.4, 41.1, 24.0; **HRMS**: Calcd. for $C_{11}H_{11}NO_4$ $[M+Na]^+$ = 244.0580 m/z, found = 244.0579 m/z.

Compound 3c:

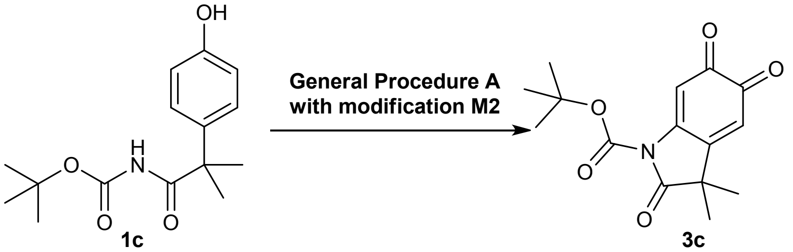

1c **Chemical Formula:** $C_{15}H_{21}NO_4$ **Molecular Weight:** 279.3315

3c **Chemical Formula:** $C_{15}H_{17}NO_5$ **Molecular Weight:** 291.2992

Procedure: The reaction was carried out according to the General Procedure A with modification M2.

<u>Amounts of Reagents</u>:

1c (279.3 mg, 1.0 mmol)

$[Cu(CH_3CN)_4](PF_6)$ (14.9 mg, 0.04 mmol, 4 mol%)

N,N'-di-*tert*-butylethylenediamine (43.1 μL, 0.20 mmol, 20 mol%)

CH_2Cl_2 (5 mL, 0.2 M)

<u>Purification</u>: 30% EtOAc in hexanes.

<u>Yield of Product:</u>

3c: 244.2 mg, 0.84 mmol, 84% yield, red solid.

<u>Characterization:</u>

R_f = (ethyl acetate/hexane 1:3): 0.33; **IR** (neat) ν = 2992, 2981, 1802, 1746, 1689, 1660, 1590, 1371, 1296, 1274, 1242, 1139, 1109, 848, 829, 733, 636 cm^{-1}; **^{1}H NMR** (500 MHz, $CDCl_3$) δ 6.76 (s, 1H), 6.36 (s, 1H), 1.61 (s, 9H), 1.48 (s, 6H); **^{13}C NMR** (126 MHz, $CDCl_3$) δ 178.4, 178.3, 174.8, 154.8, 148.0, 147.5, 123.0, 109.2, 87.2, 42.5, 27.8, 24.8; **HRMS**: Calcd. for $C_{15}H_{17}NO_5$ $[M+Na]^+$ = 314.0999 m/z, found = 314.0993 m/z.

b) Reactions in Table 2

<u>Entry 1, Table 2</u>:

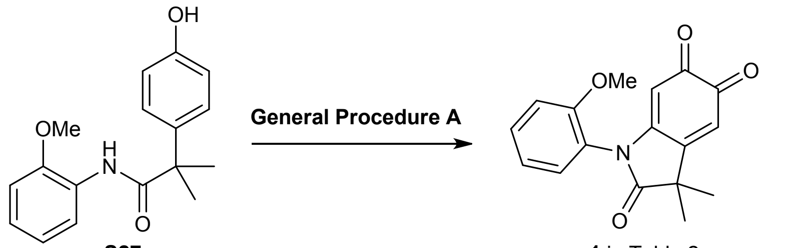

S27 **Chemical Formula:** $C_{17}H_{19}NO_3$ **Molecular Weight:** 285.3377

1 in Table 2 **Chemical Formula:** $C_{17}H_{15}NO_4$ **Molecular Weight:** 297.3053

<u>Procedure</u>: The reaction was carried out according to the General Procedure A.

<u>Amounts of Reagents</u>:

S27 (285.3 mg, 1.0 mmol)

$[Cu(CH_3CN)_4](PF_6)$ (14.9 mg, 0.04 mmol, 4 mol%)

N,N'-di-*tert*-butylethylenediamine (43.1 μL, 0.20 mmol, 20 mol%)

CH_2Cl_2 (5 mL, 0.2 M)

<u>Purification</u>: 40% EtOAc in hexanes.

<u>Yield of Product:</u>

1: 285.4 mg, 0.96 mmol, 96% yield, red solid.

<u>Characterization:</u>

R_f = (ethyl acetate/hexane 1:2): 0.17; **IR** (neat) ν = 3008, 2974, 2933, 1756, 1686, 1651, 1597, 1502, 1462, 1409, 1333, 1251, 1238, 1197, 1119, 1095, 1019, 827, 747, 655 cm^{-1}; **^{1}H NMR** (400 MHz, $CDCl_3$) δ 7.49 (t, *J* = 8.6 Hz, 1H), 7.21 (dd, *J* = 7.6, 1.1 Hz, 1H), 7.09 (t, *J* = 8.5 Hz, 2H), 6.44 (s, 1H), 5.50 (s, 1H), 3.82 (s, 3H), 1.56 (s, 6H).; **^{13}C NMR** (75 MHz, $CDCl_3$) δ 179.7, 177.3, 177.2, 155.7, 154.4, 153.6, 131.7, 128.7, 123.4, 121.3, 120.4, 112.5, 101.9, 55.9, 42.4, 25.1, 23.8; **HRMS**: Calcd. for $C_{17}H_{15}NO_4$ $[M+H]^+$ = 298.1074 m/z, found = 298.1074 m/z.

Entry 2, Table 2:

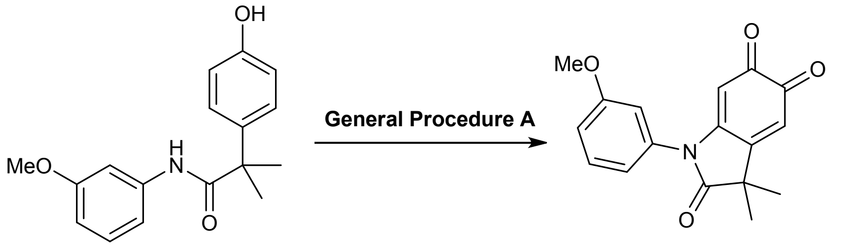

S28
Chemical Formula: $C_{17}H_{19}NO_3$
Molecular Weight: 285.3377

2 in Table 2
Chemical Formula: $C_{17}H_{15}NO_4$
Molecular Weight: 297.3053

Procedure: The reaction was carried out according to the General Procedure A.

Amounts of Reagents:

S28 (285.3 mg, 1.0 mmol)

$[Cu(CH_3CN)_4](PF_6)$ (14.9 mg, 0.04 mmol, 4 mol%)

N,N'-di-*tert*-butylethylenediamine (43.1 μL, 0.20 mmol, 20 mol%)

CH_2Cl_2 (5 mL, 0.2 M)

Purification: 40% EtOAc in hexanes.

Yield of Product:

2: 292.4 mg, 0.98 mmol, 98% yield, red oil.

Characterization:

R*f* = (ethyl acetate/hexane 1:2): 0.21; **IR** (neat) ν = 2974, 2935, 1751, 1685, 1651, 1586, 1490, 1457, 1406, 1331, 1246, 1192, 1166, 1098, 1036, 829, 758, 688 cm^{-1}; **^{1}H NMR** (400 MHz, $CDCl_3$) δ 7.46 (t, *J* = 8.1 Hz, 1H), 7.04 (ddd, *J* = 8.4, 2.4, 0.6 Hz, 1H), 6.87 (ddd, *J* = 7.8, 1.8, 0.8 Hz, 1H), 6.81 (t, *J* = 2.2 Hz, 1H), 6.46 (s, 1H), 5.80 (s, 1H), 3.86 (s, 3H), 1.58 (s, 6H); **^{13}C NMR** (75 MHz, $CDCl_3$) δ 179.3, 177.3, 177.1, 160.7, 155.2, 153.2, 133.1, 130.7, 123.5, 118.6, 115.5, 112.5, 102.2, 55.6, 42.3, 24.5; **HRMS**: Calcd. for $C_{17}H_{15}NO_4$ $[M+H]^+$ = 298.1074 m/z, found = 298.1073 m/z.

Entry 3, Table 2:

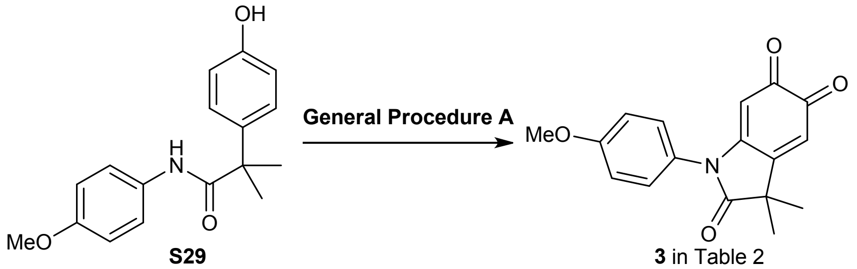

S29
Chemical Formula: $C_{17}H_{19}NO_3$
Molecular Weight: 285.3377

3 in Table 2
Chemical Formula: $C_{17}H_{15}NO_4$
Molecular Weight: 297.3053

Procedure: The reaction was carried out according to the General Procedure A.

Amounts of Reagents:

S29 (285.3 mg, 1.0 mmol)

$[Cu(CH_3CN)_4](PF_6)$ (14.9 mg, 0.04 mmol, 4 mol%)

N,N'-di-*tert*-butylethylenediamine (43.1 μL, 0.20 mmol, 20 mol%)

CH_2Cl_2 (5 mL, 0.2 M)

Purification: 40% EtOAc in hexanes.

Yield of Product:

3: 260.9 mg, 0.88 mmol, 88% yield, red solid.

Characterization:

R*f* = (ethyl acetate/hexane 1:2): 0.16; **IR** (neat) ν = 2970, 2932, 1752, 1679, 1646, 1596, 1516, 1415, 1339, 1312, 1245, 1206, 1096, 1031, 841, 822, 767, 634 cm^{-1}; **^{1}H NMR** (400 MHz, $CDCl_3$) δ 7.19 (d, *J* = 8.9 Hz, 2H), 7.04 (d, *J* = 8.9 Hz, 2H),

6.45 (s, 1H), 5.76 (s, 1H), 3.86 (s, 3H), 1.56 (s, 6H); **^{13}C NMR** (75 MHz, $CDCl_3$) δ 179.4, 177.4, 177.3, 160.3, 155.3, 153.5, 127.8, 124.4, 123.5, 115.2, 101.9, 55.6, 42.2, 24.5; **HRMS**: Calcd. for $C_{17}H_{15}NO_4$ $[M+H]^+$ = 298.1074 m/z, found = 298.1074 m/z.

Entry 4, Table 2:

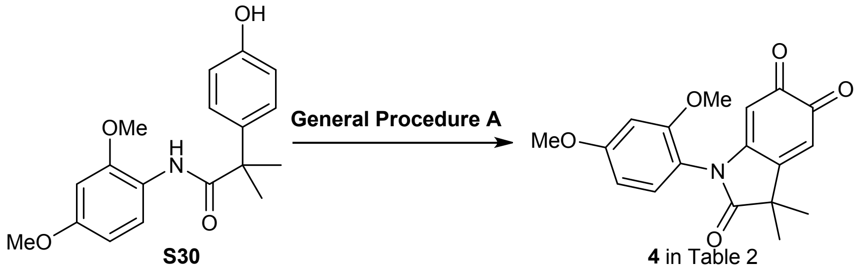

S30
Chemical Formula: $C_{18}H_{21}NO_4$
Molecular Weight: 315.3636

4 in Table 2
Chemical Formula: $C_{18}H_{17}NO_5$
Molecular Weight: 327.3313

Procedure: The reaction was carried out according to the General Procedure A.

Amounts of Reagents:

S30 (315.4 mg, 1.0 mmol)

$[Cu(CH_3CN)_4](PF_6)$ (14.9 mg, 0.04 mmol, 4 mol%)

N,N'-di-*tert*-butylethylenediamine (43.1 μL, 0.20 mmol, 20 mol%)

CH_2Cl_2 (5 mL, 0.2 M)

Purification: 40% EtOAc in hexanes.

Yield of Product:

4: 293.8 mg, 0.90 mmol, 90% yield, red solid.

Characterization:

R_f = (ethyl acetate/hexane 1:2): 0.16; **IR** (neat) v = 3011, 2971, 2917, 2848, 1754, 1688, 1648, 1599, 1510, 1461, 1417, 1386, 1332, 1316, 1287, 1242, 1202, 1152, 1122, 1095, 1023, 944, 910, 827, 748, 728, 634 cm^{-1}; **^{1}H NMR** (400 MHz, $CDCl_3$) δ 7.07 (d, *J* = 8.5 Hz, 1H), 6.56-6.54 (m, 2H), 6.38 (s, 1H), 5.44 (s, 1H), 3.80 (s, 3H), 3.73 (s, 3H), 1.50 (s, 6H); **^{13}C NMR** (75 MHz, $CDCl_3$) δ 179.8, 177.5, 177.3, 162.2, 155.7, 155.4, 154.1, 129.3, 123.3, 113.2, 105.2, 101.7, 99.7, 55.9, 55.6, 42.3, 25.0, 23.6; **HRMS**: Calcd. for $C_{18}H_{17}NO_5$ $[M+Na]^+$ = 350.0999 m/z, found = 350.0998 m/z.

Entry 5, Table 2:

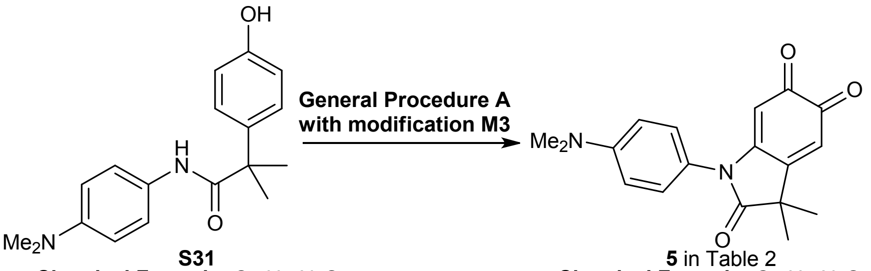

S31
Chemical Formula: $C_{18}H_{22}N_2O_2$
Molecular Weight: 298.3795

5 in Table 2
Chemical Formula: $C_{18}H_{18}N_2O_3$
Molecular Weight: 310.3471

Procedure: The reaction was carried out according to the General Procedure A with modification M3.

Amounts of Reagents:

S31 (298.4 mg, 1.0 mmol)

$[Cu(CH_3CN)_4](PF_6)$ (14.9 mg, 0.04 mmol, 4 mol%)

N,N'-di-*tert*-butylethylenediamine (43.1 μL, 0.20 mmol, 20 mol%)

CH_2Cl_2 (5 mL, 0.2 M)

Purification: 40% EtOAc in hexanes.

Yield of Product:

5: 218.3 mg, 0.70 mmol, 70% yield, dark-red solid.

Characterization:

R_f = (ethyl acetate/hexane 1:1): 0.48; **IR** (neat) v = 2916, 2816, 1750, 1682, 1646, 1599, 1536, 1444, 1417, 1372, 1334, 1241, 1212, 1181, 1152, 1104, 831, 803, 764, 726, 630 cm^{-1}; **^{1}H NMR** (400 MHz, $CDCl_3$) δ 7.08 (d, *J* = 8.9 Hz, 2H), 6.74 (d, *J* = 8.9 Hz, 2H), 6.41 (s, 1H), 5.77 (s, 1H), 3.00 (s, 6H), 1.52 (s, 6H); **^{13}C NMR** (75 MHz, $CDCl_3$) δ 179.7, 177.6, 177.4, 155.5, 154.0, 150.8, 127.1, 123.3, 119.9, 112.4, 101.8, 42.1, 40.3, 24.5; **HRMS**: Calcd. for $C_{18}H_{19}N_2O_3$ $[M+H]^+$ = 311.1390 m/z, found = 311.1385 m/z.

Entry 6, Table 2:

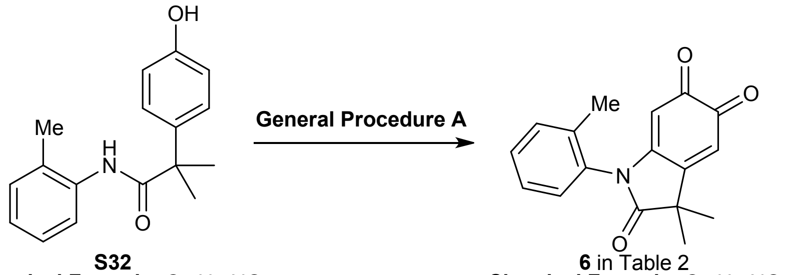

S32
Chemical Formula: $C_{17}H_{19}NO_2$
Molecular Weight: 269.3383

6 in Table 2
Chemical Formula: $C_{17}H_{15}NO_3$
Molecular Weight: 281.3059

Procedure: The reaction was carried out according to the General Procedure A.

Amounts of Reagents:

S32 (269.3 mg, 1.0 mmol)

$[Cu(CH_3CN)_4](PF_6)$ (14.9 mg, 0.04 mmol, 4 mol%)

N,N'-di-*tert*-butylethylenediamine (43.1 μL, 0.20 mmol, 20 mol%)

CH_2Cl_2 (5 mL, 0.2 M)

Purification: 30% EtOAc in hexanes.

Yield of Product:

6: 261.6 mg, 0.93 mmol, 93%, red solid.

Characterization:

R_f = (ethyl acetate/hexane 1:2): 0.28; **IR** (neat) v = 2976, 2932, 1754, 1684, 1650, 1608, 1598, 1494, 1460, 1406, 1388, 1328, 1240, 1191, 1118, 1097, 829, 757, 748, 733, 656 cm^{-1}; **^{1}H NMR** (400 MHz, $CDCl_3$) δ 7.45 – 7.31 (m, 3H), 7.13 (d, *J* = 7.6 Hz, 1H), 6.45 (s, 1H), 5.45 (s, 1H), 2.14 (s, 3H), 1.56 (s, 6H); **^{13}C NMR** (75 MHz, $CDCl_3$) δ 179.4, 177.2, 177.0, 155.2, 153.3, 135.4, 131.8, 130.9, 130.5, 127.7, 127.5, 123.8, 102.0, 42.4, 25.1, 23.9, 17.3; **HRMS**: Calcd. for $C_{17}H_{15}NO_3$ $[M+H]^+$ = 282.1125 m/z, found = 282.1124 m/z.

Entry 7, Table 2:

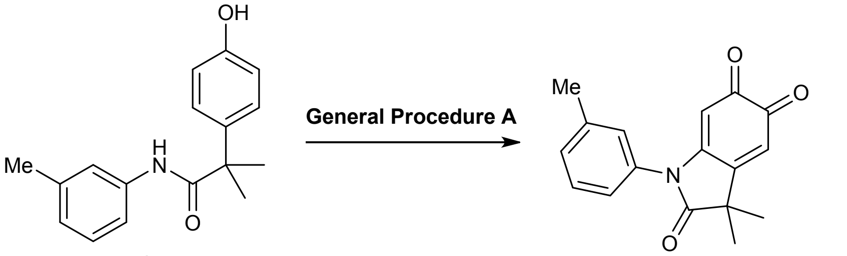

S33
Chemical Formula: $C_{17}H_{19}NO_2$
Molecular Weight: 269.3383

7 in Table 2
Chemical Formula: $C_{17}H_{15}NO_3$
Molecular Weight: 281.3059

Procedure: The reaction was carried out according to the General Procedure A.

Amounts of Reagents:

S33 (269.3 mg, 1.0 mmol)

[Cu(CH_3CN)$_4$](PF_6) (14.9 mg, 0.04 mmol, 4 mol%)

N,N'-di-*tert*-butylethylenediamine (43.1 μL, 0.20 mmol, 20 mol%)

CH_2Cl_2 (5 mL, 0.2 M)

Purification: 30% EtOAc in hexanes.

Yield of Product:

7: 258.1 mg, 0.92 mmol, 92%, red oil.

Characterization:

R*f* = (ethyl acetate/hexane 1:2): 0.29; **IR** (neat) ν = 3012, 2976, 2931, 2871, 1754, 1685, 1650, 1596, 1490, 1459, 1404, 1386, 1330, 1238, 1201, 1175, 1100, 830, 747, 691, 661 cm^{-1}; **^{1}H NMR** (400 MHz, $CDCl_3$) δ 7.41 (t, *J* = 7.7 Hz, 1H), 7.28 (d, *J* = 7.7 Hz, 1H), 7.06 (d, *J* = 8.3 Hz, 2H), 6.43 (s, 1H), 5.73 (s, 1H), 2.40 (s, 3H), 1.54 (s, 6H); **^{13}C NMR** (75 MHz, $CDCl_3$) δ 179.4, 177.4, 177.2, 155.3, 153.3, 140.2, 132.0, 130.6, 129.7, 127.1, 123.6, 123.5, 102.1, 42.3, 24.5, 21.3; **HRMS**: Calcd. for $C_{17}H_{15}NO_3$ [M+H]$^+$ = 282.1125 m/z, found = 282.1124 m/z.

Entry 8, Table 2:

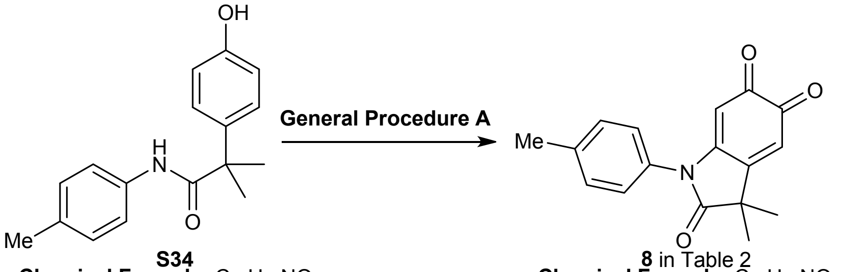

S34
Chemical Formula: $C_{17}H_{19}NO_2$
Molecular Weight: 269.3383

8 in Table 2
Chemical Formula: $C_{17}H_{15}NO_3$
Molecular Weight: 281.3059

Procedure: The reaction was carried out according to the General Procedure A.

Amounts of Reagents:

S34 (269.3 mg, 1.0 mmol)

[Cu(CH_3CN)$_4$](PF_6) (14.9 mg, 0.04 mmol, 4 mol%)

N,N'-di-*tert*-butylethylenediamine (43.1 μL, 0.20 mmol, 20 mol%)

CH_2Cl_2 (5 mL, 0.2 M)

Purification: 30% EtOAc in hexanes.

Yield of Product:

8: 245.3 mg, 0.87 mmol, 87%, red solid.

Characterization:

R*f* = (ethyl acetate/hexane 1:2): 0.27; **IR** (neat) ν = 2975, 2916, 2870, 1764, 1687, 1647, 1597, 1513, 1420, 1386, 1329, 1307, 1269, 1242, 1193, 1183, 1150, 1103, 828, 820, 761, 729, 632 cm^{-1}; **^{1}H NMR** (400 MHz, $CDCl_3$) δ 7.31 (d, *J* = 8.1 Hz, 2H), 7.14 (d, *J* = 8.2 Hz, 2H), 6.41 (s, 1H), 5.71 (s, 1H), 2.39 (s, 3H), 1.53 (s, 6H); **^{13}C NMR** (75 MHz, $CDCl_3$) δ 179.4, 177.34, 177.31, 155.3, 153.4, 140.0, 130.5, 129.4, 126.4, 123.5, 101.9, 42.3, 24.5, 21.3; **HRMS**: Calcd. for $C_{17}H_{15}NO_3$ [M+H]$^+$ = 282.1125 m/z, found = 282.1125 m/z.

Entry 9, Table 2:

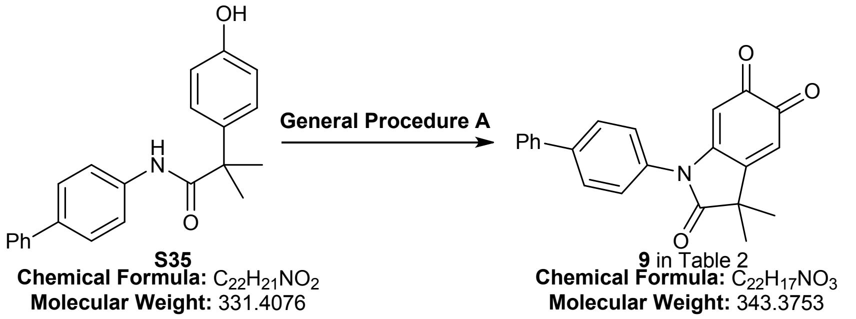

Procedure: The reaction was carried out according to the General Procedure A.

Amounts of Reagents:

S35 (331.4 mg, 1.0 mmol)

$[Cu(CH_3CN)_4](PF_6)$ (14.9 mg, 0.04 mmol, 4 mol%)

N,N'-di-*tert*-butylethylenediamine (43.1 μL, 0.20 mmol, 20 mol%)

CH_2Cl_2 (5 mL, 0.2 M)

Purification: 30% EtOAc in hexanes.

Yield of Product:

9: 319.9 mg, 0.87 mmol, 87%, red solid.

Characterization:

R_f = (ethyl acetate/hexane 1:2): 0.18; **IR** (neat) ν = 3059, 3032, 2967, 2874, 1751, 1681, 1646, 1600, 1520, 1487, 1414, 1387, 1335, 1240, 1206, 1180, 1152, 1108, 1099, 830, 767, 688, 636 cm^{-1}; ^{1}**H NMR** (400 MHz, $CDCl_3$) δ 7.75 (d, *J* = 8.3 Hz, 2H), 7.60 (d, *J* = 7.4 Hz, 2H), 7.48 (t, *J* = 7.5 Hz, 2H), 7.41 (t, *J* = 7.3 Hz, 1H), 7.37 (d, *J* = 8.5 Hz, 2H), 6.47 (s, 1H), 5.87 (s, 1H), 1.59 (s, 6H); ^{13}**C NMR** (75 MHz, $CDCl_3$) δ 179.3, 177.3, 177.2, 155.2, 153.0, 142.9, 139.5, 131.0, 129.0, 128.6, 128.1, 127.2, 126.9, 123.6, 102.2, 42.3, 24.6; **HRMS**: Calcd. for $C_{22}H_{17}NO_3$ $[M+Na]^+$ = 366.1101 m/z, found = 366.1107 m/z.

Entry 10, Table 2:

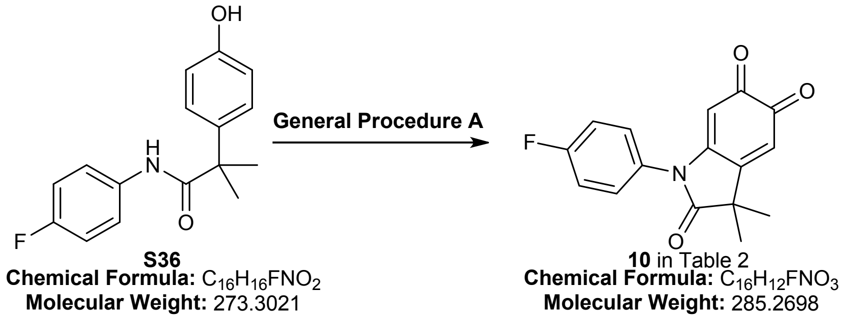

Procedure: The reaction was carried out according to the General Procedure A.

Amounts of Reagents:

S36 (273.3 mg, 1.0 mmol)

$[Cu(CH_3CN)_4](PF_6)$ (14.9 mg, 0.04 mmol, 4 mol%)

N,N'-di-*tert*-butylethylenediamine (43.1 μL, 0.20 mmol, 20 mol%)

CH_2Cl_2 (5 mL, 0.2 M)

Purification: 30% EtOAc in hexanes.

Yield of Product:

10: 268.9 mg, 0.94 mmol, 94%, red solid.

Characterization:

R_f = (ethyl acetate/hexane 1:2): 0.22; **IR** (neat) ν = 3061, 2983, 1754, 1681, 1653, 1596, 1510, 1465, 1410, 1332, 1244, 1219, 1197, 1161, 1153, 1106, 1098, 838, 760, 630 cm^{-1}; ^{1}**H NMR** (400 MHz, $CDCl_3$) δ 7.32 – 7.21 (m, 4H), 6.45 (s, 1H),

5.73 (s, 1H), 1.56 (s, 6H); **^{13}C NMR** (75 MHz, $CDCl_3$) δ 179.2, 177.2, 162.7 (d, J_F = 251.1 Hz), 155.0, 153.1, 128.7 (d, J_F = 9.0 Hz), 127.9 (d, J_F = 3.4 Hz), 123.7, 117.1 (d, J_F = 23.2 Hz), 102.0, 42.3, 24.5; **HRMS**: Calcd. for $C_{16}H_{12}FNO_3$ $[M+Na]^+$ = 308.0693 m/z, found = 308.0694 m/z.

<u>Entry 11, Table 2</u>:

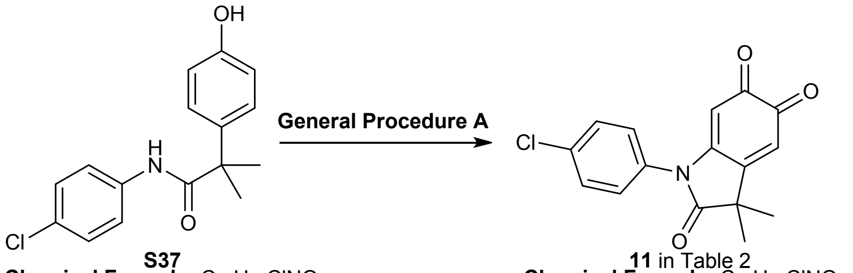

S37 **Chemical Formula:** $C_{16}H_{16}ClNO_2$ **Molecular Weight:** 289.7567

11 in Table 2 **Chemical Formula:** $C_{16}H_{12}ClNO_3$ **Molecular Weight:** 301.7244

<u>Procedure</u>: The reaction was carried out according to the General Procedure A.

<u>Amounts of Reagents</u>:

S37 (289.8 mg, 1.0 mmol)

$[Cu(CH_3CN)_4](PF_6)$ (14.9 mg, 0.04 mmol, 4 mol%)

N,N'-di-*tert*-butylethylenediamine (43.1 μL, 0.20 mmol, 20 mol%)

CH_2Cl_2 (5 mL, 0.2 M)

<u>Purification</u>: 30% EtOAc in hexanes.

<u>Yield of Product</u>:

11: 276.1 mg, 0.92 mmol, 92%, red solid.

<u>Characterization</u>:

R_f = (ethyl acetate/hexane 1:2): 0.31; **IR** (neat) ν = 3052, 2977, 1752, 1681, 1652, 1599, 1496, 1408, 1328, 1239, 1200, 1175, 1101, 1085, 840, 759, 621 cm^{-1}; **^{1}H NMR** (400 MHz, $CDCl_3$) δ 7.56 (d, *J* = 8.8 Hz, 2H), 7.27 (d, *J* = 8.7 Hz, 2H), 6.48 (s, 1H), 5.78 (s, 1H), 1.59 (s, 6H); **^{13}C NMR** (75 MHz, $CDCl_3$) δ 179.1, 177.2, 177.1, 154.9, 152.7, 135.7, 130.5, 130.2, 128.0, 123.7, 102.1, 42.3, 24.5; **HRMS**: Calcd. for $C_{16}H_{12}ClNO_3$ $[M+Na]^+$ = 324.0398 m/z, found = 324.0402 m/z.

<u>Entry 12, Table 2</u>:

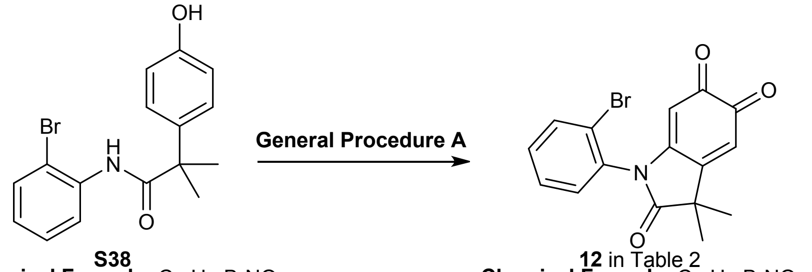

S38 **Chemical Formula:** $C_{16}H_{16}BrNO_2$ **Molecular Weight:** 334.2077

12 in Table 2 **Chemical Formula:** $C_{16}H_{12}BrNO_3$ **Molecular Weight:** 346.1754

<u>Procedure</u>: The reaction was carried out according to the General Procedure A.

<u>Amounts of Reagents</u>:

S38 (334.2 mg, 1.0 mmol)

$[Cu(CH_3CN)_4](PF_6)$ (14.9 mg, 0.04 mmol, 4 mol%)

N,N'-di-*tert*-butylethylenediamine (43.1 μL, 0.20 mmol, 20 mol%)

CH_2Cl_2 (5 mL, 0.2 M)

<u>Purification</u>: 30% EtOAc in hexanes.

<u>Yield of Product</u>:

12: 344.7 mg, 0.99 mmol, 99%, red oil.

Characterization:

R$_f$ = (ethyl acetate/hexane 1:2): 0.24; **IR** (neat) ν = 2975, 2933, 1756, 1686, 1651, 1600, 1477, 1406, 1386, 1332, 1237, 1198, 1152, 1101, 1046, 828, 746, 680, 644 cm^{-1}; **^{1}H NMR** (400 MHz, $CDCl_3$) δ 7.75 (d, *J* = 8.0 Hz, 1H), 7.49 (t, *J* = 7.6 Hz, 1H), 7.40 (dt, *J* = 7.9, 1.5 Hz, 1H), 7.30 (dd, *J* = 7.8, 1.3 Hz, 1H), 6.44 (s, 1H), 5.39 (s, 1H), 1.57 (s, 3H), 1.55 (s, 3H); **^{13}C NMR** (75 MHz, $CDCl_3$) δ 179.3, 177.1, 176.5, 155.1, 152.6, 134.2, 131.9, 131.4, 129.6, 129.2, 123.8, 122.0, 102.3, 42.6, 25.1, 23.7; **HRMS**: Calcd. for $C_{16}H_{12}BrNO_3$ $[M+H]^+$ = 346.0073 m/z, found = 346.0075 m/z.

Entry 13, Table 2:

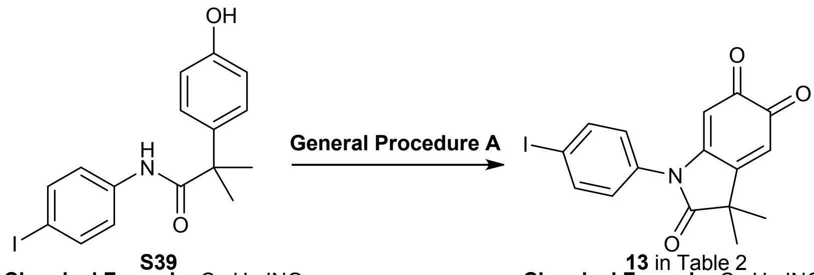

S39 **Chemical Formula:** $C_{16}H_{16}INO_2$ **Molecular Weight:** 381.2082

13 in Table 2 **Chemical Formula:** $C_{16}H_{12}INO_3$ **Molecular Weight:** 393.1759

Procedure: The reaction was carried out according to the General Procedure A.

Amounts of Reagents:

S39 (381.2 mg, 1.0 mmol)

$[Cu(CH_3CN)_4](PF_6)$ (14.9 mg, 0.04 mmol, 4 mol%)

N,N'-di-*tert*-butylethylenediamine (43.1 μL, 0.20 mmol, 20 mol%)

CH_2Cl_2 (5 mL, 0.2 M)

Purification: 30% EtOAc in hexanes.

Yield of Product:

13: 360.7 mg, 0.92 mmol, 92%, red solid.

Characterization:

R$_f$ = (ethyl acetate/hexane 1:2): 0.28; **IR** (neat) ν = 3057, 2974, 1748, 1679, 1647, 1597, 1486, 1409, 1386, 1337, 1246, 1199, 1176, 1102, 1058, 1009, 838, 814, 764, 738, 628 cm^{-1}; **^{1}H NMR** (400 MHz, $CDCl_3$) δ 7.91 (d, *J* = 8.6 Hz, 2H), 7.07 (d, *J* = 8.6 Hz, 2H), 6.46 (s, 1H), 5.77 (s, 1H), 1.57 (s, 6H); **^{13}C NMR** (75 MHz, $CDCl_3$) δ 179.1, 177.2, 176.9, 154.9, 152.5, 139.2, 131.8, 128.4, 123.7, 102.1, 95.4, 42.3, 24.6; **HRMS**: Calcd. for $C_{16}H_{12}INO_3$ $[M+H]^+$ = 393.9935 m/z, found = 393.9933 m/z.

Entry 14, Table 2:

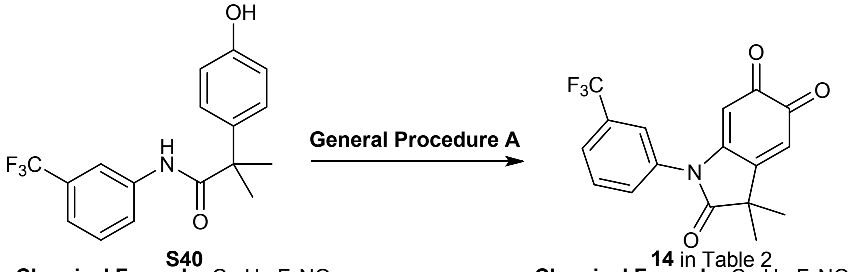

S40 **Chemical Formula:** $C_{17}H_{16}F_3NO_2$ **Molecular Weight:** 323.3096

14 in Table 2 **Chemical Formula:** $C_{17}H_{12}F_3NO_3$ **Molecular Weight:** 335.2773

Procedure: The reaction was carried out according to the General Procedure A.

Amounts of Reagents:

S40 (323.3 mg, 1.0 mmol)

$[Cu(CH_3CN)_4](PF_6)$ (14.9 mg, 0.04 mmol, 4 mol%)

N,N'-di-*tert*-butylethylenediamine (43.1 μL, 0.20 mmol, 20 mol%)

CH_2Cl_2 (5 mL, 0.2 M)

Purification: 30% EtOAc in hexanes.

Yield of Product:

14: 331.9 mg, 0.99 mmol, 99%, red oil.

Characterization:

R*f* = (ethyl acetate/hexane 1:2): 0.24; **IR** (neat) ν = 2979, 1753, 1686, 1654, 1594, 1494, 1454, 1406, 1336, 1322, 1239, 1193, 1170, 1152, 1124, 1104, 1090, 1068, 879, 830, 804, 760, 697, 679, 611 cm^{-1}; **^{1}H NMR** (400 MHz, $CDCl_3$) δ 7.79 – 7.65 (m, 2H), 7.59 (s, 1H), 7.53 (d, *J* = 7.4 Hz, 1H), 6.44 (s, 1H), 5.67 (s, 1H), 1.56 (s, 6H); **^{13}C NMR** (75 MHz, $CDCl_3$) δ 179.0, 177.2, 177.0, 154.9, 152.6, 132.8, 132.5 (q, J_F = 33.4 Hz), 130.7, 130.3, 126.5 (q, J_F = 3.6 Hz), 123.9 (q, J_F = 3.8 Hz), 123.8, 123.2 (q, J_F = 272.8 Hz), 101.9, 42.4, 24.5; **HRMS**: Calcd. for $C_{17}H_{12}F_3NO_3$ $[M+H]^+$ = 336.0842 m/z, found = 336.0844 m/z.

Entry 15, Table 2:

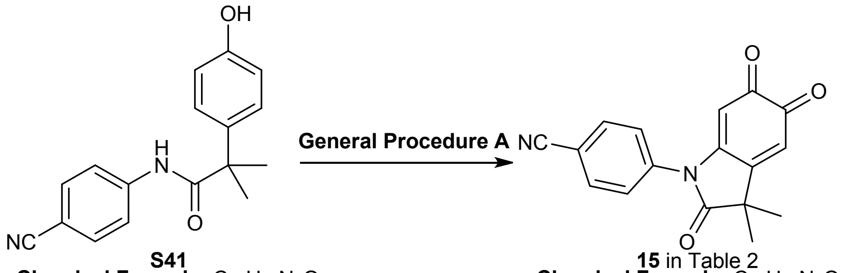

S41 **Chemical Formula:** $C_{17}H_{16}N_2O_2$ **Molecular Weight:** 280.3211

15 in Table 2 **Chemical Formula:** $C_{17}H_{12}N_2O_3$ **Molecular Weight:** 292.2888

Procedure: The reaction was carried out according to the General Procedure A.

Amounts of Reagents:

S41 (280.3 mg, 1.0 mmol)

$[Cu(CH_3CN)_4](PF_6)$ (14.9 mg, 0.04 mmol, 4 mol%)

N,N'-di-*tert*-butylethylenediamine (43.1 μL, 0.20 mmol, 20 mol%)

CH_2Cl_2 (5 mL, 0.2 M)

Purification: 40% EtOAc in hexanes.

Yield of Product:

15: 212.8 mg, 0.73 mmol, 73%, red solid.

Characterization:

R*f* = (ethyl acetate/hexane 1:2): 0.14; **IR** (neat) ν = 3060, 2976, 2228, 1748, 1686, 1653, 1599, 1506, 1412, 1335, 1282, 1246, 1204, 1105, 846, 827, 767, 630 cm^{-1}; **^{1}H NMR** (400 MHz, $CDCl_3$) δ 7.87 (d, *J* = 7.9 Hz, 2H), 7.49 (d, *J* = 8.5 Hz, 2H), 6.48 (s, 1H), 5.80 (s, 1H), 1.58 (s, 6H); **^{13}C NMR** (75 MHz, $CDCl_3$) δ 178.8, 177.1, 176.8, 154.7, 151.9, 136.1, 133.8, 127.7, 123.9, 117.5, 113.6, 102.2, 42.5, 24.6; **HRMS**: Calcd. for $C_{17}H_{12}N_2O_3$ $[M+H]^+$ = 293.0921 m/z, found = 293.0920 m/z.

Entry 16, Table 2:

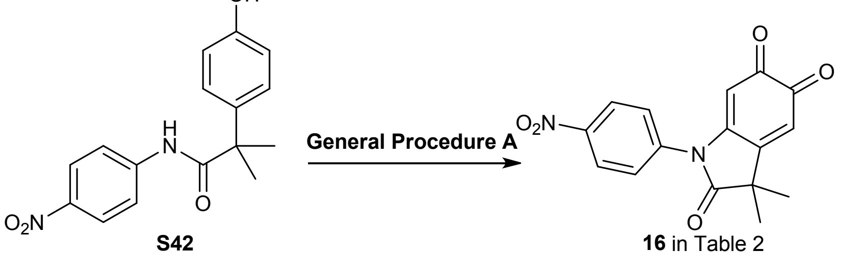

S42 **Chemical Formula:** $C_{16}H_{16}N_2O_4$ **Molecular Weight:** 300.3092

16 in Table 2 **Chemical Formula:** $C_{16}H_{12}N_2O_5$ **Molecular Weight:** 312.2769

Procedure: The reaction was carried out according to the General Procedure A.

Amounts of Reagents:

S42 (300.3 mg, 1.0 mmol)

$[Cu(CH_3CN)_4](PF_6)$ (14.9 mg, 0.04 mmol, 4 mol%)
N,N'-di-*tert*-butylethylenediamine (43.1 μL, 0.20 mmol, 20 mol%)
CH_2Cl_2 (5 mL, 0.2 M)

Purification: 40% EtOAc in hexanes.

Yield of Product:

16: 283.5 mg, 0.91 mmol, 91%, red solid.

Characterization:

R*f* = (ethyl acetate/hexane 1:1): 0.31; **IR** (neat) ν = 3065, 2977, 1751, 1684, 1655, 1600, 1593, 1521, 1496, 1405, 1390, 1349, 1329, 1305, 1267, 1240, 1197, 1172, 1152, 1098, 974, 942, 842, 745, 686 cm^{-1}; **^{1}H NMR** (400 MHz, $CDCl_3$) δ 8.46 (d, *J* = 8.9 Hz, 2H), 7.59 (d, *J* = 8.9 Hz, 2H), 6.52 (s, 1H), 5.86 (s, 1H), 1.62 (s, 6H); **^{13}C NMR** (75 MHz, $CDCl_3$) δ 178.7, 177.1, 176.7, 154.5, 151.7, 147.8, 137.6, 127.8, 125.3, 124.0, 102.4, 42.5, 24.7; **HRMS**: Calcd. for $C_{16}H_{12}N_2O_5$ $[M+Na]^+$ = 335.0638 m/z, found = 335.0650 m/z.

Entry 17, Table 2:

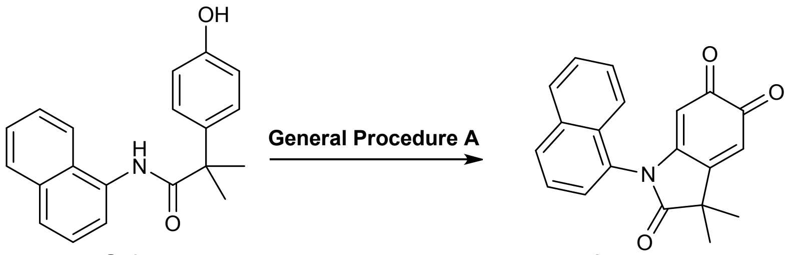

S43
Chemical Formula: $C_{20}H_{19}NO_2$
Molecular Weight: 305.3704

17 in Table 2
Chemical Formula: $C_{20}H_{15}NO_3$
Molecular Weight: 317.3380

Procedure: The reaction was carried out according to the General Procedure A.

Amounts of Reagents:

S43 (305.4 mg, 1.0 mmol)
$[Cu(CH_3CN)_4](PF_6)$ (14.9 mg, 0.04 mmol, 4 mol%)
N,N'-di-*tert*-butylethylenediamine (43.1 μL, 0.20 mmol, 20 mol%)
CH_2Cl_2 (5 mL, 0.2 M)

Purification: 30% EtOAc in hexanes.

Yield of Product:

17: 311.8 mg, 0.98 mmol, 98%, red solid.

Characterization:

R*f* = (ethyl acetate/hexane 1:2): 0.20; **IR** (neat) ν = 3054, 2985, 2934, 1749, 1683, 1648, 1603, 1508, 1464, 1417, 1386, 1329, 1235, 1172, 1122, 1037, 834, 809, 774, 651, 612 cm^{-1}; **^{1}H NMR** (400 MHz, $CDCl_3$) δ 8.07 (d, *J* = 8.3 Hz, 1H), 8.01 (dd, *J* = 7.3, 2.0 Hz, 1H), 7.69 – 7.54 (m, 3H), 7.45 (dd, *J* = 7.3, 0.9 Hz, 2H), 6.55 (s, 1H), 5.40 (s, 1H), 1.74 (s, 3H), 1.68 (s, 3H); **^{13}C NMR** (75 MHz, $CDCl_3$) δ 179.4, 177.6, 177.1, 155.3, 153.8, 134.6, 130.9, 129.1, 128.5, 128.4, 128.0, 127.2, 126.3, 125.5, 123.8, 121.2, 102.7, 42.7, 25.3, 24.3; **HRMS**: Calcd. for $C_{20}H_{15}NO_3$ $[M+H]^+$ = 318.1125 m/z, found = 318.1119 m/z.

Entry 18, Table 2:

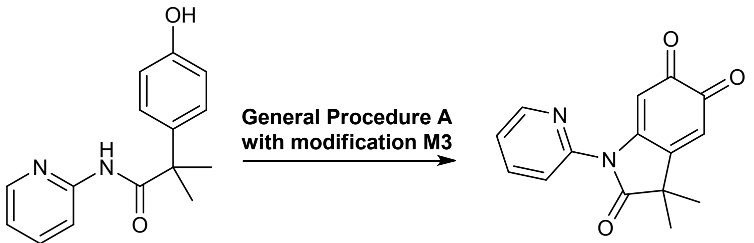

S44 **18** in Table 2
Chemical Formula: $C_{15}H_{16}N_2O_2$ **Chemical Formula:** $C_{15}H_{12}N_2O_3$
Molecular Weight: 256.2997 **Molecular Weight:** 268.2674

Procedure: The reaction was carried out according to the General Procedure A with modification M3.

Amounts of Reagents:

S44 (256.3 mg, 1.0 mmol)

[Cu(CH_3CN)$_4$](PF_6) (14.9 mg, 0.04 mmol, 4 mol%)

N,N'-di-*tert*-butylethylenediamine (43.1 μL, 0.20 mmol, 20 mol%)

CH_2Cl_2 (5 mL, 0.2 M)

Purification: 50% EtOAc in hexanes.

Yield of Product:

18: 220.2 mg, 0.82 mmol, 82%, red solid.

Characterization:

R*f* = (ethyl acetate/hexane 1:1): 0.21; **IR** (neat) ν = 3064, 2972, 2917, 1752, 1681, 1640, 1594, 1573, 1467, 1436, 1388, 1330, 1245, 1182, 1157, 1118, 1091, 841, 787, 762, 746, 680, 639, 617 cm^{-1}; **^{1}H NMR** (400 MHz, $CDCl_3$) δ 8.62 (d, *J* = 3.8 Hz, 1H), 7.93 (td, *J* = 7.9, 1.8 Hz, 1H), 7.57 (d, *J* = 8.1 Hz, 1H), 7.40 (dd, *J* = 7.5, 4.9 Hz, 1H), 6.44 (s, 1H), 6.39 (s, 1H), 1.56 (s, 6H); **^{13}C NMR** (75 MHz, $CDCl_3$) δ 179.0, 178.0, 177.0, 155.3, 150.7, 149.3, 146.9, 138.8, 124.1, 123.3, 121.4, 104.8, 42.4, 24.6; **HRMS**: Calcd. for $C_{15}H_{12}N_2O_3$ $[M+Na]^+$ = 291.0740 m/z, found = 291.0747 m/z.

Entry 19, Table 2:

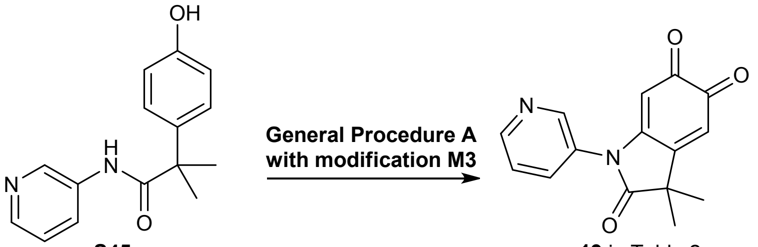

S45 **19** in Table 2
Chemical Formula: $C_{15}H_{16}N_2O_2$ **Chemical Formula:** $C_{15}H_{12}N_2O_3$
Molecular Weight: 256.2997 **Molecular Weight:** 268.2674

Procedure: The reaction was carried out according to the General Procedure A with modification M3.

Amounts of Reagents:

S45 (256.3 mg, 1.0 mmol)

[Cu(CH_3CN)$_4$](PF_6) (14.9 mg, 0.04 mmol, 4 mol%)

N,N'-di-*tert*-butylethylenediamine (43.1 μL, 0.20 mmol, 20 mol%)

CH_2Cl_2 (5 mL, 0.2 M)

Purification: 50%-70% EtOAc in hexanes.

Yield of Product:

19: 187.2 mg, 0.70 mmol, 70%, red solid.

Characterization:

R*f* = (ethyl acetate): 0.44; **IR** (neat) ν = 3041, 2916, 1747, 1679, 1650, 1597, 1577, 1481, 1428, 1406, 1387, 1339, 1204, 1109, 1092, 872, 858, 761, 705, 679 cm^{-1}; **^{1}H NMR** (400 MHz, $CDCl_3$) δ 8.77 (d, *J* = 4.7 Hz, 1H), 8.64 (d, *J* = 2.2 Hz, 1H), 7.71

(d, J = 8.2 Hz, 1H), 7.55 (dd, J = 8.1, 4.8 Hz, 1H), 6.51 (s, 1H), 5.79 (s, 1H), 1.61 (s, 6H); **^{13}C NMR** (126 MHz, $CDCl_3$) δ 178.9, 177.11, 177.08, 154.7, 152.4, 150.8, 147.8, 134.4, 129.2, 124.3, 123.9, 102.2, 42.5, 24.7; **HRMS**: Calcd. for $C_{15}H_{12}N_2O_3$ $[M+H]^+$ = 269.0921 m/z, found = 269.0918 m/z.

Entry 20, Table 2:

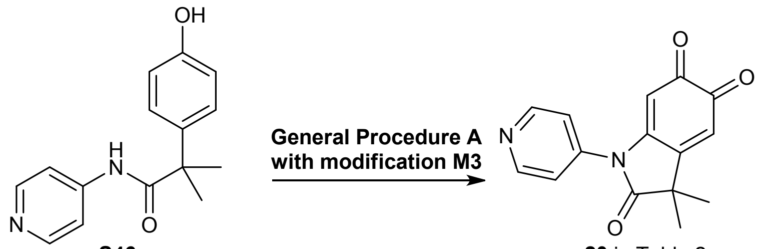

S46
Chemical Formula: $C_{15}H_{16}N_2O_2$
Molecular Weight: 256.2997

20 in Table 2
Chemical Formula: $C_{15}H_{12}N_2O_3$
Molecular Weight: 268.2674

Procedure: The reaction was carried out according to the General Procedure A with modification M3.

Amounts of Reagents:

S46 (256.3 mg, 1.0 mmol)

$[Cu(CH_3CN)_4](PF_6)$ (14.9 mg, 0.04 mmol, 4 mol%)

N,N'-di-*tert*-butylethylenediamine (43.1 μL, 0.20 mmol, 20 mol%)

CH_2Cl_2 (5 mL, 0.2 M)

Purification: EtOAc.

Yield of Product:

20: 237.2 mg, 0.88 mmol, 88%, red solid.

Characterization:

R_f = (ethyl acetate): 0.28; **IR** (neat) ν = 2978, 1756, 1682, 1654, 1604, 1582, 1493, 1462, 1404, 1330, 1242, 1195, 1155, 1102, 824, 761, 660 cm^{-1}; **^{1}H NMR** (400 MHz, $CDCl_3$) δ 8.84 (d, J = 6.0 Hz, 2H), 7.33 (dd, J = 4.7, 1.4 Hz, 2H), 6.48 (s, 1H), 5.88 (s, 1H), 1.58 (s, 6H); **^{13}C NMR** (75 MHz, $CDCl_3$) δ 178.7, 177.2, 176.5, 154.6, 151.7, 151.4, 139.9, 123.9, 120.9, 102.4, 42.4, 24.6; **HRMS**: Calcd. for $C_{15}H_{12}N_2O_3$ $[M+H]^+$ = 269.0921 m/z, found = 269.0919 m/z.

Entry 21, Table 2:

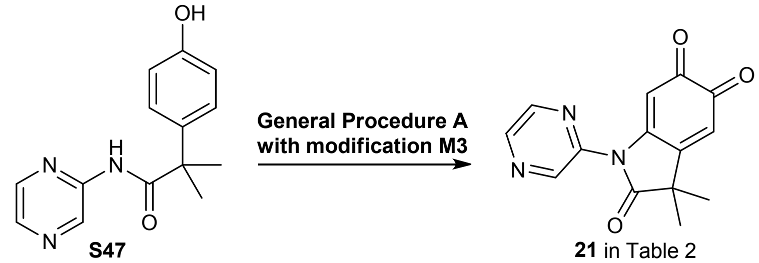

S47
Chemical Formula: $C_{14}H_{15}N_3O_2$
Molecular Weight: 257.2878

21 in Table 2
Chemical Formula: $C_{14}H_{11}N_3O_3$
Molecular Weight: 269.2554

Procedure: The reaction was carried out according to the General Procedure A with modification M3.

Amounts of Reagents:

S47 (257.3 mg, 1.0 mmol)

$[Cu(CH_3CN)_4](PF_6)$ (14.9 mg, 0.04 mmol, 4 mol%)

N,N'-di-*tert*-butylethylenediamine (43.1 μL, 0.20 mmol, 20 mol%)

CH_2Cl_2 (5 mL, 0.2 M)

Purification: 40% EtOAc in hexanes.

Yield of Product:

21: 214.5 mg, 0.80 mmol, 80%, red solid.

Characterization:

R$_f$ = (ethyl acetate/hexane 1:1): 0.38; **IR** (neat) ν = 1753, 1679, 1646, 1597, 1465, 1415, 1387, 1332, 1249, 1199, 1163, 1113, 1050, 1013, 847, 769, 746, 702, 630 cm^{-1}; **^{1}H NMR** (500 MHz, $CDCl_3$) δ 9.01 (d, *J* = 1.0 Hz, 1H), 8.67 (d, *J* = 2.4 Hz, 1H), 8.62 – 8.59 (m, 1H), 6.52 (s, 1H), 6.47 (s, 1H), 1.60 (s, 6H); **^{13}C NMR** (126 MHz, $CDCl_3$) δ 178.6, 177.9, 176.9, 155.0, 149.7, 144.2, 144.1, 143.1, 142.5, 123.5, 105.6, 42.4, 24.7; **HRMS**: Calcd. for $C_{14}H_{11}N_3O_3$ $[M+Na]^+$ = 292.0693 m/z, found = 292.0698 m/z.

Entry 22, Table 2:

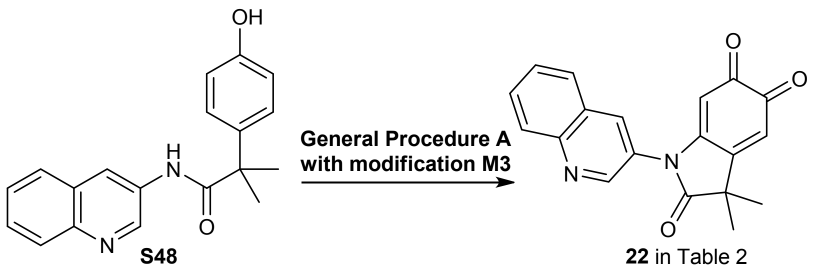

S48
Chemical Formula: $C_{19}H_{18}N_2O_2$
Molecular Weight: 306.3584

22 in Table 2
Chemical Formula: $C_{19}H_{14}N_2O_3$
Molecular Weight: 318.3261

Procedure: The reaction was carried out according to the General Procedure A with modification M3.

Amounts of Reagents:

S48 (306.4 mg, 1.0 mmol)

$[Cu(CH_3CN)_4](PF_6)$ (14.9 mg, 0.04 mmol, 4 mol%)

N,N'-di-*tert*-butylethylenediamine (43.1 μL, 0.20 mmol, 20 mol%)

CH_2Cl_2 (5 mL, 0.2 M)

Purification: 50% EtOAc in hexanes.

Yield of Product:

22: 263.7 mg, 0.83 mmol, 83%, red solid.

Characterization:

R$_f$ = (ethyl acetate/hexane 1:1): 0.20; **IR** (neat) ν = 1746, 1684, 1651, 1596, 1496, 1466, 1408, 1387, 1346, 1323, 1244, 1178, 1128, 1100, 990, 896, 841, 747, 729, 635 cm^{-1}; **^{1}H NMR** (400 MHz, $CDCl_3$) δ 8.84 (d, *J* = 2.2 Hz, 1H), 8.22 (d, *J* = 8.0 Hz, 2H), 7.91 (d, *J* = 8.1 Hz, 1H), 7.87 (t, *J* = 7.6 Hz, 1H), 7.69 (t, *J* = 7.5 Hz, 1H), 6.52 (s, 1H), 5.84 (s, 1H), 1.64 (s, 6H); **^{13}C NMR** (75 MHz, $CDCl_3$) δ 178.9, 177.3, 177.1, 154.8, 152.6, 147.8, 147.4, 134.0, 131.3, 129.6, 128.1, 128.0, 127.4, 125.7, 123.9, 102.1, 42.5, 24.7; **HRMS**: Calcd. for $C_{19}H_{14}N_2O_3$ $[M+Na]^+$ = 341.0897 m/z, found = 341.0899 m/z.

Entry 23, Table 2:

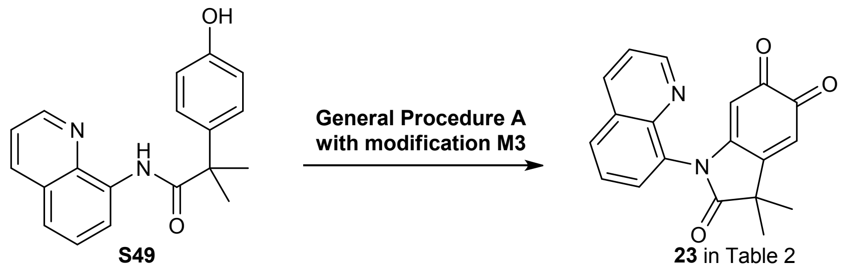

S49
Chemical Formula: $C_{19}H_{18}N_2O_2$
Molecular Weight: 306.3584

23 in Table 2
Chemical Formula: $C_{19}H_{14}N_2O_3$
Molecular Weight: 318.3261

Procedure: The reaction was carried out according to the General Procedure A with modification M3. The amount of N,N'-di-*tert*-butylethylenediamine (64.7 μL, 0.30 mmol, 30 mol%) and reaction time (6h) were also modified from the standard condition.

Amounts of Reagents:

S49 (306.4 mg, 1.0 mmol)

$[Cu(CH_3CN)_4](PF_6)$ (14.9 mg, 0.04 mmol, 4 mol%)
N,N'-di-*tert*-butylethylenediamine (64.7 μL, 0.30 mmol, 30 mol%)
CH_2Cl_2 (5 mL, 0.2 M)
Purification: 50% EtOAc in hexanes.
Yield of Product:
23: 190.9 mg, 0.60 mmol, 60%, red solid.
Characterization:
R*f* = (ethyl acetate/hexane 1:1): 0.15; **IR** (neat) ν = 3051, 3010, 2974, 2931, 1752, 1682, 1649, 1594, 1499, 1412, 1385, 1332, 1236, 1202, 1178, 1155, 1120, 1046, 880, 827, 791, 746, 646 cm^{-1}; **^{1}H NMR** (400 MHz, $CDCl_3$) δ 8.84 (dd, *J* = 4.1, 1.3 Hz, 1H), 8.23 (dd, *J* = 8.3, 1.2 Hz, 1H), 7.99 (dd, *J* = 8.0, 0.8 Hz, 1H), 7.71 (d, *J* = 6.2 Hz, 1H), 7.66 (t, *J* = 7.7 Hz, 1H), 7.46 (dd, *J* = 8.3, 4.2 Hz, 1H), 6.49 (s, 1H), 5.31 (s, 1H), 1.71 (s, 3H), 1.61 (s, 3H); **^{13}C NMR** (75 MHz, $CDCl_3$) δ 179.8, 178.0, 177.4, 156.0, 154.8, 151.5, 142.9, 136.3, 130.6, 129.9, 129.4, 129.3, 126.1, 123.4, 122.4, 102.1, 42.8, 25.3, 23.8; **HRMS**: Calcd. for $C_{19}H_{14}N_2O_3$ $[M+Na]^+$ = 341.0897 m/z, found = 341.0893 m/z.

Entry 24, Table 2:

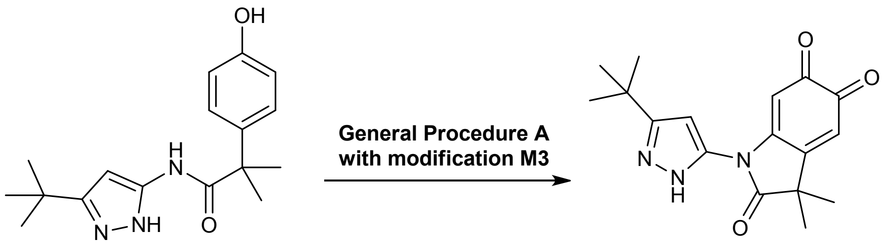

S50
Chemical Formula: $C_{17}H_{23}N_3O_2$
Molecular Weight: 301.3834

24 in Table 2
Chemical Formula: $C_{17}H_{19}N_3O_3$
Molecular Weight: 313.3511

Procedure: The reaction was carried out according to the General Procedure A with modification M3. A mixture of THF (1 mL) and CH_2Cl_2 (4 mL) was also used as solvent instead of CH_2Cl_2 (5 mL).
Amounts of Reagents:
S50 (301.4 mg, 1.0 mmol)
$[Cu(CH_3CN)_4](PF_6)$ (14.9 mg, 0.04 mmol, 4 mol%)
N,N'-di-*tert*-butylethylenediamine (43.1 μL, 0.20 mmol, 20 mol%)
THF (1 mL) and CH_2Cl_2 (4 mL), 0.2 M in mixing solvents
Purification: 50%-70% EtOAc in hexanes.
Yield of Product:
24: 147.3 mg, 0.47 mmol, 47%, red solid. 147.7 mg, 0.49 mmol, 49% **S50** recovered. 92% yield brsm.
Characterization:
R*f* = (ethyl acetate/hexane 1:1): 0.43; **IR** (neat) ν = 3277, 2958, 2868, 1772, 1683, 1638, 1596, 1563, 1473, 1459, 1378, 1314, 1291, 1251, 1185, 1105, 1000, 857, 806, 735, 641 cm^{-1}; **^{1}H NMR** (500 MHz, acetone-*d_6*) δ 12.19 (br. s, 1H), 6.70 (s, 1H), 6.62 (s, 1H), 6.55 (s, 1H), 1.58 (s, 6H), 1.42 (s, 9H); **^{13}C NMR** (126 MHz, acetone-*d_6*) δ 179.6, 178.2, 176.9, 155.4, 153.9, 151.0, 143.2, 123.1, 104.8, 96.3, 41.9, 31.0, 29.4, 23.7; **HRMS**: Calcd. for $C_{17}H_{19}N_3O_3$ $[M+H]^+$ = 314.1499 m/z, found = 314.1503 m/z.

c) Reactions in Table 3

Entry 1, Table 3:

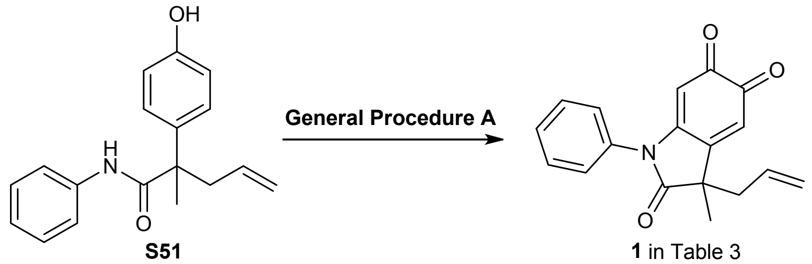

S51
Chemical Formula: $C_{18}H_{19}NO_2$
Molecular Weight: 281.3490

1 in Table 3
Chemical Formula: $C_{18}H_{15}NO_3$
Molecular Weight: 293.3166

Procedure: The reaction was carried out according to the General Procedure A.

Amounts of Reagents:

S51 (281.3 mg, 1.0 mmol)

$[Cu(CH_3CN)_4](PF_6)$ (14.9 mg, 0.04 mmol, 4 mol%)

N,N'-di-*tert*-butylethylenediamine (43.1 μL, 0.20 mmol, 20 mol%)

CH_2Cl_2 (5 mL, 0.2 M)

Purification: 30% EtOAc in hexanes.

Yield of Product:

1: 273.7 mg, 0.93 mmol, 93%, red solid.

Characterization:

R_f = (ethyl acetate/hexane 1:2): 0.25; **IR** (neat) ν = 3062, 2977, 2931, 1750, 1684, 1651, 1599, 1587, 1494, 1453, 1407, 1336, 1247, 1198, 1173, 1098, 1024, 996, 922, 828, 732, 692, 659 cm^{-1}; **^{1}H NMR** (400 MHz, $CDCl_3$) δ 7.57 (t, *J* = 7.3 Hz, 2H), 7.52 (t, *J* = 7.1 Hz, 1H), 7.28 – 7.24 (m, 2H), 6.48 (s, 1H), 5.81 – 5.62 (m, 2H), 5.25 (s, 1H), 5.24 – 5.17 (m, 1H), 2.78 (dd, *J* = 13.5, 8.1 Hz, 1H), 2.57 (dd, *J* = 13.5, 6.9 Hz, 1H), 1.59 (s, 3H); **^{13}C NMR** (75 MHz, $CDCl_3$) δ 179.2, 177.3, 176.3, 153.6, 153.5, 132.1, 130.3, 130.0, 129.8, 126.7, 124.2, 121.5, 101.8, 46.7, 43.2, 22.6; **HRMS**: Calcd. for $C_{18}H_{15}NO_3$ $[M+Na]^+$ = 316.0944 m/z, found = 316.0947 m/z.

Entry 2, Table 3:

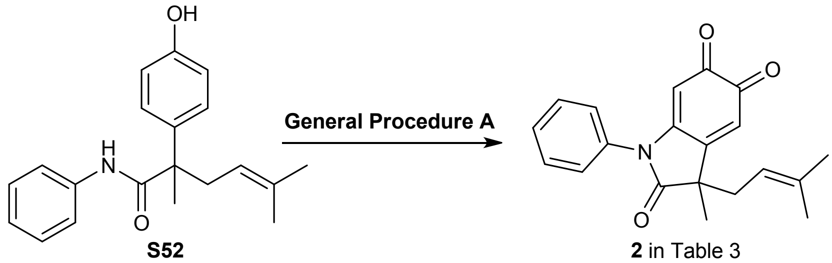

S52
Chemical Formula: $C_{20}H_{23}NO_2$
Molecular Weight: 309.4021

2 in Table 3
Chemical Formula: $C_{20}H_{19}NO_3$
Molecular Weight: 321.3698

Procedure: The reaction was carried out according to the General Procedure A.

Amounts of Reagents:

S52 (309.4 mg, 1.0 mmol)

$[Cu(CH_3CN)_4](PF_6)$ (14.9 mg, 0.04 mmol, 4 mol%)

N,N'-di-*tert*-butylethylenediamine (43.1 μL, 0.20 mmol, 20 mol%)

CH_2Cl_2 (5 mL, 0.2 M)

Purification: 30% EtOAc in hexanes.

Yield of Product:

2: 309.8 mg, 0.96 mmol, 96%, red oil.

Characterization:

R$_f$ = (ethyl acetate/hexane 1:2): 0.38; **IR** (neat) ν = 3062, 2971, 2930, 1754, 1686, 1652, 1600, 1494, 1453, 1407, 1334, 1244, 1197, 1087, 828, 742, 692, 661 cm^{-1}; **^{1}H NMR** (400 MHz, $CDCl_3$) δ 7.52 (t, *J* = 7.4 Hz, 2H), 7.46 (t, *J* = 7.3 Hz, 1H), 7.20 (d, *J* = 7.3 Hz, 2H), 6.44 (s, 1H), 5.66 (s, 1H), 5.04 (dd, *J* = 8.3, 7.2 Hz, 1H), 2.76 (dd, *J* = 13.8, 8.6 Hz, 1H), 2.47 (dd, *J* = 13.8, 7.0 Hz, 1H), 1.69 (s, 3H), 1.59 (s, 3H), 1.53 (s, 3H); **^{13}C NMR** (75 MHz, $CDCl_3$) δ 179.3, 177.3, 176.7, 154.4, 153.9, 138.3, 132.2, 130.0, 129.8, 126.6, 123.9, 116.2, 101.5, 47.1, 38.2, 26.0, 22.2, 18.1; **HRMS**: Calcd. for $C_{20}H_{19}NO_3$ $[M+H]^+$ = 322.1438 m/z, found = 322.1435 m/z.

Entry 3, Table 3:

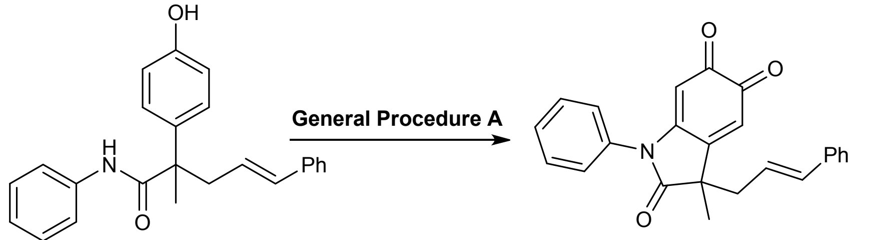

S53
Chemical Formula: $C_{24}H_{23}NO_2$
Molecular Weight: 357.4449

3 in Table 3
Chemical Formula: $C_{24}H_{19}NO_3$
Molecular Weight: 369.4126

Procedure: The reaction was carried out according to the General Procedure A.

Amounts of Reagents:

S53 (357.4 mg, 1.0 mmol)

$[Cu(CH_3CN)_4](PF_6)$ (14.9 mg, 0.04 mmol, 4 mol%)

N,N'-di-*tert*-butylethylenediamine (43.1 μL, 0.20 mmol, 20 mol%)

CH_2Cl_2 (5 mL, 0.2 M)

Purification: 30% EtOAc in hexanes.

Yield of Product:

3: 312.8 mg, 0.85 mmol, 85%, red oil.

Characterization:

R$_f$ = (ethyl acetate/hexane 1:2): 0.31; **IR** (neat) ν = 3058, 3024, 2974, 2932, 1752, 1685, 1652, 1599, 1494, 1449, 1406, 1332, 1245, 1197, 1093, 1020, 969, 828, 739, 690, 660 cm^{-1}; **^{1}H NMR** (500 MHz, $CDCl_3$) δ 7.52 – 7.42 (m, 3H), 7.36 – 7.24 (m, 5H), 7.19 – 7.08 (m, 2H), 6.56 (d, *J* = 13.6 Hz, 2H), 6.09 (ddd, *J* = 15.5, 8.2, 7.2 Hz, 1H), 5.69 (s, 1H), 2.92 (dd, *J* = 13.3, 8.7 Hz, 1H), 2.73 (dd, *J* = 13.3, 6.7 Hz, 1H), 1.64 (s, 3H); **^{13}C NMR** (126 MHz, $CDCl_3$) δ 179.2, 177.3, 176.3, 153.8, 153.6, 136.3, 136.1, 132.0, 130.0, 129.8, 128.8, 128.2, 126.7, 126.3, 124.3, 121.2, 101.9, 47.4, 42.8, 22.4; **HRMS**: Calcd. for $C_{24}H_{19}NO_3$ $[M+H]^+$ = 370.1438 m/z, found = 370.1435 m/z.

Entry 4, Table 3:

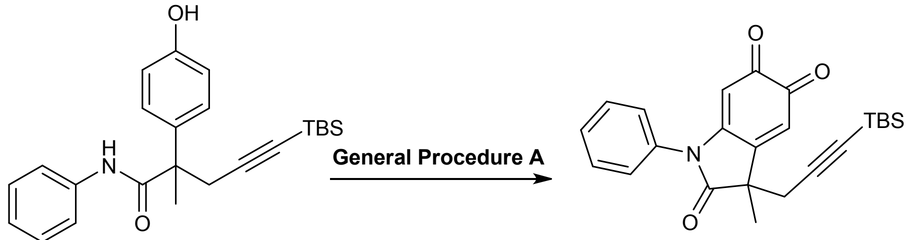

S54
Chemical Formula: $C_{24}H_{31}NO_2Si$
Molecular Weight: 393.5939

4 in Table 3
Chemical Formula: $C_{24}H_{27}NO_3Si$
Molecular Weight: 405.5616

Procedure: The reaction was carried out according to the General Procedure A.

Amounts of Reagents:

S54 (393.6 mg, 1.0 mmol)

[Cu(CH_3CN)$_4$](PF_6) (14.9 mg, 0.04 mmol, 4 mol%)
N,N'-di-*tert*-butylethylenediamine (43.1 μL, 0.20 mmol, 20 mol%)
CH_2Cl_2 (5 mL, 0.2 M)
Purification: 30% EtOAc in hexanes.
Yield of Product:
4: 393.7 mg, 0.97 mmol, 97%, red oil.
Characterization:
R$_f$ = (ethyl acetate/hexane 1:2): 0.41; **IR** (neat) v = 2952, 2928, 2856, 2178, 1754, 1688, 1656, 1603, 1496, 1455, 1408, 1337, 1249, 1199, 1094, 1039, 1017, 825, 810, 775, 743 cm^{-1}; **^{1}H NMR** (400 MHz, $CDCl_3$) δ 7.51 (t, *J* = 7.3 Hz, 2H), 7.46 (t, *J* = 7.3 Hz, 1H), 7.28 (d, *J* = 7.1 Hz, 2H), 6.57 (s, 1H), 5.73 (s, 1H), 2.90 (d, *J* = 16.7 Hz, 1H), 2.74 (d, *J* = 16.7 Hz, 1H), 1.55 (s, 3H), 0.81 (s, 9H), 0.01 (s, 3H), -0.01 (s, 3H); **^{13}C NMR** (75 MHz, $CDCl_3$) δ 179.1, 177.2, 175.8, 153.6, 152.7, 132.1, 129.9, 129.8, 126.6, 124.4, 101.8, 100.1, 87.5, 45.7, 29.8, 25.9, 22.1, 16.4, -4.6, -4.7; **HRMS**: Calcd. for $C_{24}H_{27}NO_3Si$ [M+H]$^+$ = 406.1833 m/z, found = 406.1828 m/z.

Entry 5, Table 3:

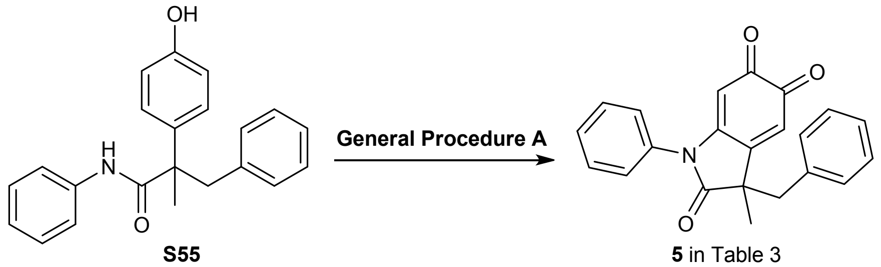

S55
Chemical Formula: $C_{22}H_{21}NO_2$
Molecular Weight: 331.4076

5 in Table 3
Chemical Formula: $C_{22}H_{17}NO_3$
Molecular Weight: 343.3753

Procedure: The reaction was carried out according to the General Procedure A.
Amounts of Reagents:
S55 (331.4 mg, 1.0 mmol)
[Cu(CH_3CN)$_4$](PF_6) (14.9 mg, 0.04 mmol, 4 mol%)
N,N'-di-*tert*-butylethylenediamine (43.1 μL, 0.20 mmol, 20 mol%)
CH_2Cl_2 (5 mL, 0.2 M)
Purification: 30% EtOAc in hexanes.
Yield of Product:
5: 329.9 mg, 0.96 mmol, 96%, red solid.
Characterization:
R$_f$ = (ethyl acetate/hexane 1:2): 0.27; **IR** (neat) v = 3061, 3030, 2973, 2929, 1752, 1686, 1653, 1601, 1494, 1453, 1409, 1343, 1311, 1254, 1200, 1130, 1093, 1072, 1025, 829, 739, 693, 661 cm^{-1}; **^{1}H NMR** (400 MHz, $CDCl_3$) δ 7.50 – 7.35 (m, 3H), 7.35 – 7.17 (m, 3H), 7.08 (d, *J* = 6.9 Hz, 2H), 6.79 (s, 2H), 6.54 (s, 1H), 5.37 (s, 1H), 3.37 (d, *J* = 13.1 Hz, 1H), 3.02 (d, *J* = 13.1 Hz, 1H), 1.67 (s, 3H); **^{13}C NMR** (75 MHz, $CDCl_3$) δ 179.2, 177.1, 176.0, 153.9, 153.6, 134.5, 131.8, 129.8, 129.7, 128.7, 127.9, 126.6, 124.5, 101.4, 48.7, 45.7, 22.8; **HRMS**: Calcd. for $C_{22}H_{17}NO_3$ [M]$^-$ = 343.1214 m/z, found = 343.1213 m/z.

Entry 6, Table 3:

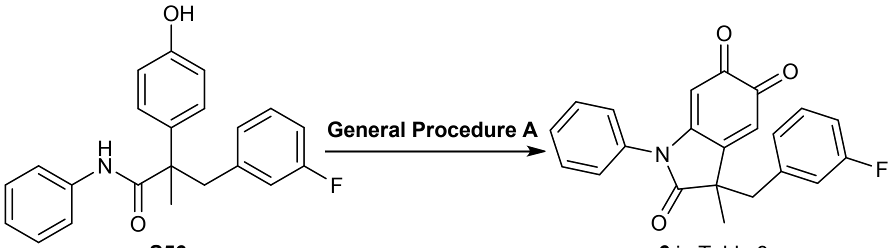

S56
Chemical Formula: $C_{22}H_{20}FNO_2$
Molecular Weight: 349.3981

6 in Table 3
Chemical Formula: $C_{22}H_{16}FNO_3$
Molecular Weight: 361.3657

Procedure: The reaction was carried out according to the General Procedure A.

Amounts of Reagents:

S56 (349.4 mg, 1.0 mmol)

$[Cu(CH_3CN)_4](PF_6)$ (14.9 mg, 0.04 mmol, 4 mol%)

N,N'-di-*tert*-butylethylenediamine (43.1 μL, 0.20 mmol, 20 mol%)

CH_2Cl_2 (5 mL, 0.2 M)

Purification: 30% EtOAc in hexanes.

Yield of Product:

6: 334.8 mg, 0.93 mmol, 93%, red solid.

Characterization:

$\mathbf{R_f}$ = (ethyl acetate/hexane 1:2): 0.29; **IR** (neat) v = 3005, 2922, 1758, 1751, 1689, 1653, 1600, 1589, 1487, 1410, 1342, 1250, 1197, 1146, 1128, 1086, 1026, 954, 834, 144, 694, 661 cm^{-1}; **^{1}H NMR** (400 MHz, $CDCl_3$) δ 7.57 – 7.39 (m, 3H), 7.24 (td, *J* = 7.9, 6.1 Hz, 1H), 7.01 (td, *J* = 8.4, 2.0 Hz, 1H), 6.89 (d, *J* = 7.6 Hz, 3H), 6.84 (d, *J* = 9.5 Hz, 1H), 6.56 (s, 1H), 5.46 (s, 1H), 3.41 (d, *J* = 13.2 Hz, 1H), 3.04 (d, *J* = 13.2 Hz, 1H), 1.70 (s, 3H); **^{13}C NMR** (75 MHz, $CDCl_3$) δ 179.0, 177.0, 175.8, 162.6 (d, J_F = 247.7 Hz), 153.38, 153.36, 136.9 (d, J_F = 7.2 Hz), 131.7, 130.3 (d, J_F = 8.3 Hz), 130.0, 129.9, 126.5, 125.6 (d, J_F = 3.0 Hz), 124.5, 116.7 (d, J_F = 21.4 Hz), 114.9 (d, J_F = 20.9 Hz), 101.7, 48.5, 44.9 (d, J_F = 1.6 Hz), 23.1; **HRMS**: Calcd. for $C_{22}H_{16}FNO_3$ $[M+Na]^+$ = 384.1006 m/z, found = 384.1006 m/z.

Entry 7, Table 3:

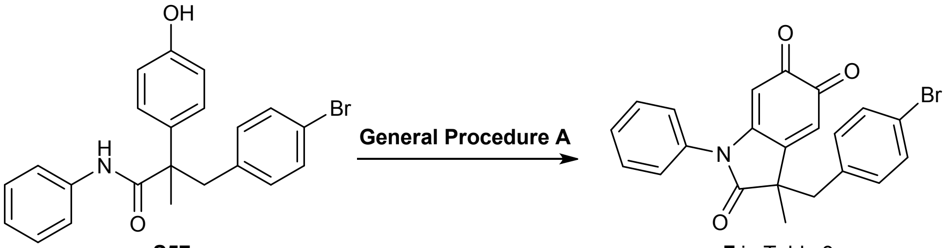

S57
Chemical Formula: $C_{22}H_{20}BrNO_2$
Molecular Weight: 410.3037

7 in Table 3
Chemical Formula: $C_{22}H_{16}BrNO_3$
Molecular Weight: 422.2713

Procedure: The reaction was carried out according to the General Procedure A.

Amounts of Reagents:

S57 (410.3 mg, 1.0 mmol)

$[Cu(CH_3CN)_4](PF_6)$ (14.9 mg, 0.04 mmol, 4 mol%)

N,N'-di-*tert*-butylethylenediamine (43.1 μL, 0.20 mmol, 20 mol%)

CH_2Cl_2 (5 mL, 0.2 M)

Purification: 30% EtOAc in hexanes.

Yield of Product:

7: 344.6 mg, 0.82 mmol, 82%, red solid.

Characterization:

R$_f$ = (ethyl acetate/hexane 1:2): 0.28; **IR** (neat) ν = 3055, 2935, 1749, 1678, 1650, 1600, 1586, 1488, 1406, 1345, 1247, 1199, 1170, 1128, 1091, 1072, 1011, 846, 835, 800, 745, 692, 661 cm^{-1}; **^{1}H NMR** (400 MHz, $CDCl_3$) δ 7.53 – 7.45 (m, 3H), 7.41 (d, *J* = 8.4 Hz, 2H), 7.00 (d, *J* = 8.4 Hz, 2H), 6.86 (d, *J* = 6.4 Hz, 2H), 6.56 (s, 1H), 5.49 (s, 1H), 3.36 (d, *J* = 13.2 Hz, 1H), 3.01 (d, *J* = 13.3 Hz, 1H), 1.70 (s, 3H); **^{13}C NMR** (75 MHz, $CDCl_3$) δ 179.0, 177.0, 175.8, 153.5, 153.3, 133.4, 131.8, 131.7, 131.5, 130.0, 129.9, 126.4, 124.5, 122.1, 101.8, 48.5, 44.8, 23.0; **HRMS**: Calcd. for $C_{22}H_{16}BrNO_3$ $[M+Na]^+$ = 444.0206 m/z, found = 444.0204 m/z.

Entry 8, Table 3:

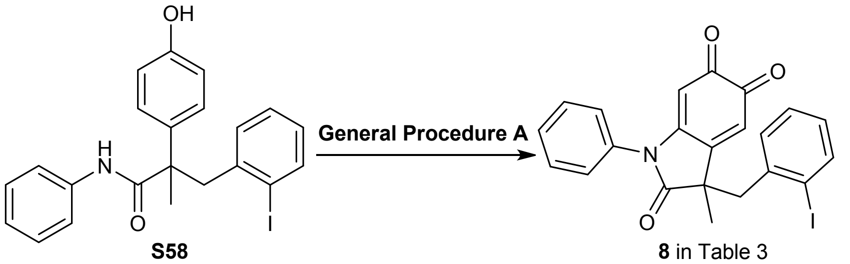

S58 **8** in Table 3

Chemical Formula: $C_{22}H_{20}INO_2$ **Chemical Formula:** $C_{22}H_{16}INO_3$

Molecular Weight: 457.3042 **Molecular Weight:** 469.2718

Procedure: The reaction was carried out according to the General Procedure A.

Amounts of Reagents:

S58 (457.3 mg, 1.0 mmol)

$[Cu(CH_3CN)_4](PF_6)$ (14.9 mg, 0.04 mmol, 4 mol%)

N,N'-di-*tert*-butylethylenediamine (43.1 μL, 0.20 mmol, 20 mol%)

CH_2Cl_2 (5 mL, 0.2 M)

Purification: 30% EtOAc in hexanes.

Yield of Product:

8: 281.1 mg, 0.60 mmol, 60%, red solid.

Characterization:

R$_f$ = (ethyl acetate/hexane 1:2): 0.30; **IR** (neat) ν = 3054, 2976, 1756, 1685, 1646, 1596, 1588, 1497, 1457, 1410, 1340, 1244, 1203, 1135, 1112, 1088, 1015, 898, 830, 784, 742, 688 cm^{-1}; **^{1}H NMR** (400 MHz, $CDCl_3$) δ 7.81 (d, *J* = 7.9 Hz, 1H), 7.55 – 7.45 (m, 3H), 7.32 – 7.21 (m, 2H), 7.10 (d, *J* = 7.1 Hz, 2H), 6.98 (t, *J* = 7.0 Hz, 1H), 6.42 (s, 1H), 5.54 (s, 1H), 3.45 (s, 2H), 1.69 (s, 3H); **^{13}C NMR** (75 MHz, $CDCl_3$) δ 178.8, 177.1, 176.2, 153.6, 151.0, 140.5, 137.7, 132.0, 130.3, 129.9, 129.8, 129.6, 128.5, 126.6, 126.2, 102.9, 101.7, 47.9, 47.4, 22.7; **HRMS**: Calcd. for $C_{22}H_{16}INO_3$ $[M+Na]^+$ = 492.0067 m/z, found = 492.0060 m/z.

Entry 9, Table 3:

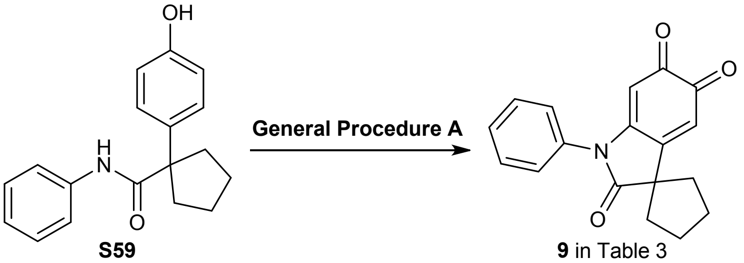

S59 **9** in Table 3

Chemical Formula: $C_{18}H_{19}NO_2$ **Chemical Formula:** $C_{18}H_{15}NO_3$

Molecular Weight: 281.3490 **Molecular Weight:** 293.3166

Procedure: The reaction was carried out according to the General Procedure A.

Amounts of Reagents:

S59 (281.3 mg, 1.0 mmol)

$[Cu(CH_3CN)_4](PF_6)$ (14.9 mg, 0.04 mmol, 4 mol%)
N,N'-di-*tert*-butylethylenediamine (43.1 μL, 0.20 mmol, 20 mol%)
CH_2Cl_2 (5 mL, 0.2 M)
Purification: 40% EtOAc in hexanes.
Yield of Product:
9: 281.8 mg, 0.96 mmol, 96%, red oil.
Characterization:
R_f = (ethyl acetate/hexane 1:2): 0.16; **IR** (neat) ν = 3057, 2950, 2871, 1747, 1682, 1648, 1601, 1589, 1493, 1413, 1330, 1261, 1246, 1203, 1098, 826, 739, 692, 660 cm^{-1}; **^{1}H NMR** (500 MHz, $CDCl_3$) δ 7.55 (t, *J* = 7.4 Hz, 2H), 7.49 (t, *J* = 7.4 Hz, 1H), 7.29 (d, *J* = 7.2 Hz, 2H), 6.44 (s, 1H), 5.76 (s, 1H), 2.44 – 2.37 (m, 2H), 2.15 – 2.07 (m, 2H), 2.05 – 1.95 (m, 4H); **^{13}C NMR** (75 MHz, $CDCl_3$) δ 179.6, 178.0, 177.3, 156.8, 153.8, 132.3, 129.9, 129.7, 126.7, 123.0, 101.6, 51.8, 40.0, 26.9; **HRMS**: Calcd. for $C_{18}H_{15}NO_3$ $[M+Na]^+$ = 316.0944 m/z, found = 316.0954 m/z.

Entry 10, Table 3:

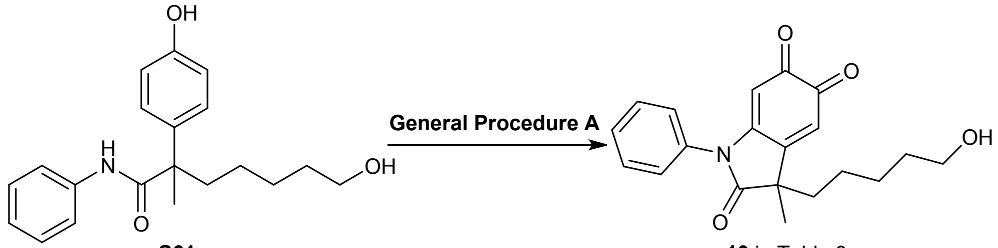

S61
Chemical Formula: $C_{20}H_{25}NO_3$
Molecular Weight: 327.4174

10 in Table 3
Chemical Formula: $C_{20}H_{21}NO_4$
Molecular Weight: 339.3850

Procedure: The reaction was carried out according to the General Procedure A.
Amounts of Reagents:
S61 (327.4 mg, 1.0 mmol)
$[Cu(CH_3CN)_4](PF_6)$ (14.9 mg, 0.04 mmol, 4 mol%)
N,N'-di-*tert*-butylethylenediamine (43.1 μL, 0.20 mmol, 20 mol%)
CH_2Cl_2 (5 mL, 0.2 M)
Purification: 60% EtOAc in hexanes.
Yield of Product:
10: 306.9 mg, 0.90 mmol, 90%, red oil.
Characterization:
R_f = (ethyl acetate/hexane 1:1): 0.12; **IR** (neat) ν = 3490, 3012, 2933, 2858, 1753, 1685, 1651, 1599, 1588, 1494, 1455, 1407, 1333, 1251, 1197, 1101, 830, 742, 692, 662 cm^{-1}; **^{1}H NMR** (400 MHz, $CDCl_3$) δ 7.55 (t, *J* = 7.4 Hz, 2H), 7.49 (t, *J* = 7.3 Hz, 1H), 7.25 (d, *J* = 5.5 Hz, 2H), 6.42 (s, 1H), 5.73 (s, 1H), 3.59 (t, *J* = 6.4 Hz, 2H), 2.13 – 2.01 (m, 1H), 1.85 – 1.74 (m, 1H), 1.73 (br. s, 1H), 1.57 – 1.48 (m, 5H), 1.41 – 1.23 (m, 4H); **^{13}C NMR** (75 MHz, $CDCl_3$) δ 179.2, 177.4, 176.9, 154.4, 153.6, 132.1, 130.0, 129.8, 126.6, 123.8, 101.8, 62.5, 46.6, 39.2, 32.1, 25.8, 24.6, 23.7; **HRMS**: Calcd. for $C_{20}H_{21}NO_4$ $[M+H]^+$ = 340.1543 m/z, found = 340.1549 m/z.

Entry 11, Table 3:

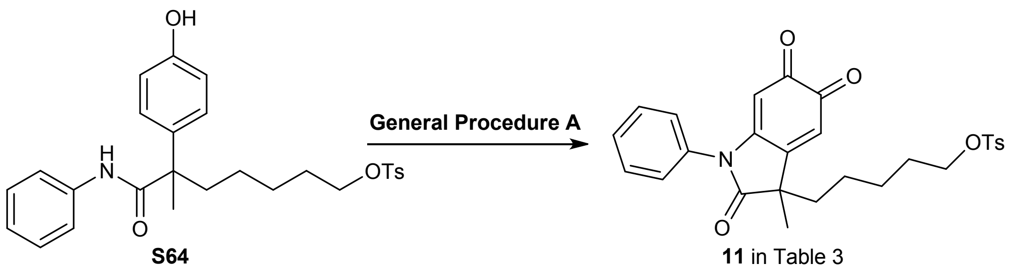

S64
Chemical Formula: $C_{27}H_{31}NO_5S$
Molecular Weight: 481.6037

11 in Table 3
Chemical Formula: $C_{27}H_{27}NO_6S$
Molecular Weight: 493.5714

Procedure: The reaction was carried out according to the General Procedure A.

Amounts of Reagents:

S64 (481.6 mg, 1.0 mmol)

$[Cu(CH_3CN)_4](PF_6)$ (14.9 mg, 0.04 mmol, 4 mol%)

N,N'-di-*tert*-butylethylenediamine (43.1 μL, 0.20 mmol, 20 mol%)

CH_2Cl_2 (5 mL, 0.2 M)

Purification: 30% EtOAc in hexanes.

Yield of Product:

11: 468.8 mg, 0.95 mmol, 95%, red oil.

Characterization:

$\mathbf{R}_f$ = (ethyl acetate/hexane 1:2): 0.35; **IR** (neat) ν = 3064, 3012, 2860, 1754, 1686, 1653, 1601, 1494, 1455, 1407, 1352, 1334, 1255, 1172, 1098, 939, 902, 828, 814, 743, 692, 660, 553 cm^{-1}; **^{1}H NMR** (400 MHz, $CDCl_3$) δ 7.72 (d, *J* = 8.2 Hz, 2H), 7.53 (t, *J* = 7.4 Hz, 2H), 7.48 (t, *J* = 7.2 Hz, 1H), 7.31 (d, *J* = 8.0 Hz, 2H), 7.25 (d, *J* = 8.7 Hz, 2H), 6.40 (s, 1H), 5.71 (s, 1H), 3.95 (t, *J* = 6.2 Hz, 2H), 2.41 (s, 3H), 2.06 – 1.93 (m, 1H), 1.80 – 1.68 (m, 1H), 1.60 (dt, *J* = 12.8, 6.3 Hz, 2H), 1.51 (s, 3H), 1.42 – 1.12 (m, 4H); **^{13}C NMR** (75 MHz, $CDCl_3$) δ 179.2, 177.3, 176.7, 154.2, 153.5, 144.8, 132.9, 132.1, 130.0, 129.9, 129.8, 127.8, 126.7, 123.8, 101.9, 70.1, 46.5, 38.8, 28.4, 25.5, 24.2, 23.7, 21.6; **HRMS**: Calcd. for $C_{27}H_{27}NO_6S$ $[M+H]^+$ = 494.1632 m/z, found = 494.1631 m/z.

Entry 12, Table 3:

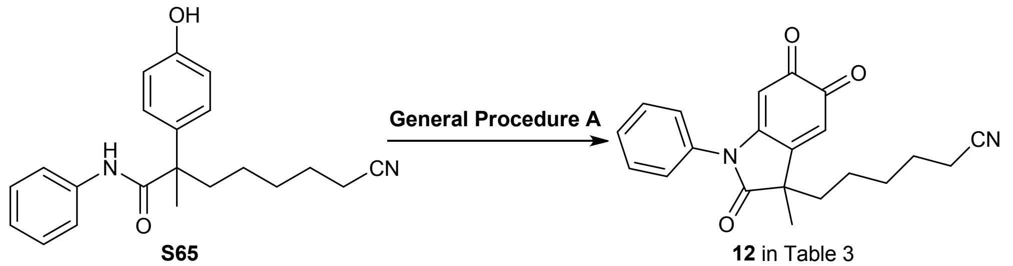

S65
Chemical Formula: $C_{21}H_{24}N_2O_2$
Molecular Weight: 336.4275

12 in Table 3
Chemical Formula: $C_{21}H_{20}N_2O_3$
Molecular Weight: 348.3951

Procedure: The reaction was carried out according to the General Procedure A.

Amounts of Reagents:

S65 (336.4 mg, 1.0 mmol)

$[Cu(CH_3CN)_4](PF_6)$ (14.9 mg, 0.04 mmol, 4 mol%)

N,N'-di-*tert*-butylethylenediamine (43.1 μL, 0.20 mmol, 20 mol%)

CH_2Cl_2 (5 mL, 0.2 M)

Purification: 40% EtOAc in hexanes.

Yield of Product:

12: 262.7 mg, 0.75 mmol, 75%, red oil.

Characterization:

R$_f$ = (ethyl acetate/hexane 1:2): 0.09; **IR** (neat) ν = 3015, 2935, 2860, 2244, 1753, 1686, 1653, 1600, 1494, 1455, 1407, 1334, 1296, 1247, 1197, 1099, 830, 743, 692, 662 cm^{-1}; **^{1}H NMR** (400 MHz, $CDCl_3$) δ 7.52 (t, *J* = 7.4 Hz, 2H), 7.46 (t, *J* = 7.4 Hz, 1H), 7.24 (d, *J* = 7.3 Hz, 2H), 6.40 (s, 1H), 5.69 (s, 1H), 2.29 (t, *J* = 7.0 Hz, 2H), 2.03 (td, *J* = 13.2, 4.5 Hz, 1H), 1.78 (td, *J* = 12.8, 3.4 Hz, 1H), 1.66 – 1.55 (m, 2H), 1.51 (s, 3H), 1.48 – 1.30 (m, 3H), 1.32 – 1.18 (m, 1H); **^{13}C NMR** (75 MHz, $CDCl_3$) δ 179.2, 177.3, 176.7, 154.1, 153.5, 132.0, 130.0, 129.8, 126.7, 123.9, 119.5, 101.9, 46.5, 38.7, 28.6, 24.9, 24.0, 23.7, 17.0; **HRMS**: Calcd. for $C_{21}H_{20}N_2O_3$ $[M+H]^+$ = 349.1547 m/z, found = 349.1544 m/z.

Entry 13, Table 3:

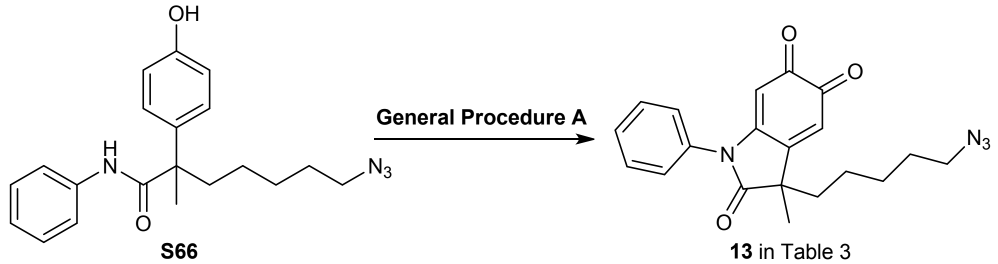

S66
Chemical Formula: $C_{20}H_{24}N_4O_2$
Molecular Weight: 352.4302

13 in Table 3
Chemical Formula: $C_{20}H_{20}N_4O_3$
Molecular Weight: 364.3978

Procedure: The reaction was carried out according to the General Procedure A.

Amounts of Reagents:

S66 (352.4 mg, 1.0 mmol)

$[Cu(CH_3CN)_4](PF_6)$ (14.9 mg, 0.04 mmol, 4 mol%)

N,N'-di-*tert*-butylethylenediamine (43.1 μL, 0.20 mmol, 20 mol%)

CH_2Cl_2 (5 mL, 0.2 M)

Purification: 30% EtOAc in hexanes.

Yield of Product:

13: 341.9 mg, 0.94 mmol, 94%, red oil.

Characterization:

R$_f$ = (ethyl acetate/hexane 1:2): 0.35; **IR** (neat) ν = 3062, 2933, 2859, 2091, 1753, 1686, 1652, 1600, 1494, 1454, 1406, 1332, 1245, 1196, 1101, 829, 742, 692, 661 cm^{-1}; **^{1}H NMR** (400 MHz, $CDCl_3$) δ 7.55 (t, *J* = 7.4 Hz, 2H), 7.49 (t, *J* = 7.3 Hz, 1H), 7.25 (d, *J* = 5.7 Hz, 2H), 6.42 (s, 1H), 5.73 (s, 1H), 3.23 (t, *J* = 6.7 Hz, 2H), 2.12 – 2.00 (m, 1H), 1.85 – 1.72 (m, 1H), 1.62 – 1.48 (m, 5H), 1.44 – 1.31 (m, 3H), 1.31 – 1.20 (m, 1H); **^{13}C NMR** (75 MHz, $CDCl_3$) δ 179.2, 177.3, 176.7, 154.2, 153.5, 132.1, 130.0, 129.9, 126.6, 123.8, 101.9, 51.2, 46.5, 39.0, 28.5, 26.7, 24.4, 23.7; **HRMS**: Calcd. for $C_{20}H_{20}N_4O_3$ $[M+Na]^+$ = 387.1428 m/z, found = 387.1426 m/z.

d) Reactions in Scheme 6

Compound 8:

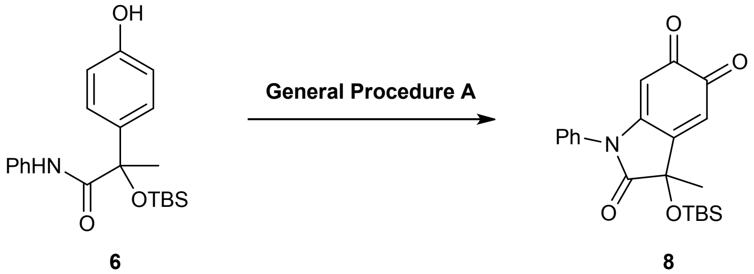

6
Chemical Formula: $C_{21}H_{29}NO_3S_i$
Molecular Weight: 371.5454

8
Chemical Formula: $C_{21}H_{25}NO_4Si$
Molecular Weight: 383.5130

Procedure: The reaction was carried out according to the General Procedure A using 10 mol% $[Cu(MeCN)_4](PF_6)$ and 50

mol% of N,N'-di-*tert*-butylethylenediamine (DBED) for 12h.

Amounts of Reagents:

6 (371.6 mg, 1.0 mmol)

$[Cu(CH_3CN)_4](PF_6)$ (37.3 mg, 0.10 mmol, 10 mol%)

N,N'-di-*tert*-butylethylenediamine (107.8 μL, 0.50 mmol, 50 mol%)

CH_2Cl_2 (5 mL, 0.2 M)

Purification: 25% EtOAc in hexanes.

Yield of Product:

8: 293.1 mg, 0.76 mmol, 76%, red foam.

Characterization:

R_f = (ethyl acetate/hexanes 1:3): 0.47; **IR** (neat) ν = 2926, 2856, 1768, 1688, 1658, 1603, 1494, 1403, 1257, 1228, 1194, 1147, 1132 cm^{-1}; **^{1}H NMR** (500 MHz, $CDCl_3$) δ 7.60 (t, *J* = 7.5 Hz, 2H), 7.54 (t, *J* = 7.3 Hz, 1H), 7.30 (d, *J* = 7.4 Hz, 2H), 6.65 (s, 1H), 5.82 (s, 1H), 1.76 (s, 3H), 0.93 (s, 9H), 0.23 (s, 3H), 0.12 (s, 3H); **^{13}C NMR** (126 MHz, $CDCl_3$) δ 179.5, 177.2, 173.8, 152.3, 151.8, 131.9, 130.1, 130.0, 126.4, 124.6, 102.7, 72.8, 26.9, 25.5, 18.1, -3.18, -3.22; **HRMS**: Calcd. for $C_{21}H_{25}NO_4Si$ $[M+H]^+$ = 384.1626 m/z, found = 384.1621 m/z.

Compound 9:

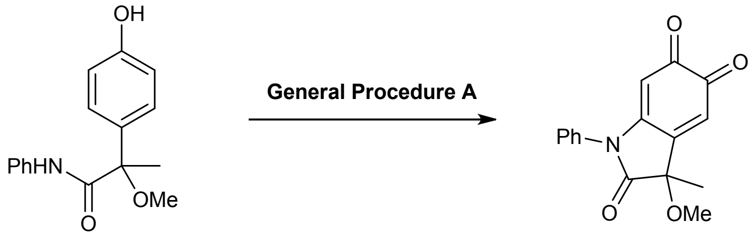

7

Chemical Formula: $C_{16}H_{17}NO_3$
Molecular Weight: 271.3111

9

Chemical Formula: $C_{16}H_{13}NO_4$
Molecular Weight: 283.2787

Procedure: The reaction was carried out according to the General Procedure A using 10 mol% $[Cu(MeCN)_4](PF_6)$ and 50 mol% of N,N'-di-*tert*-butylethylenediamine (DBED) for 12h.

Amounts of Reagents:

7 (271.3 mg, 1.0 mmol)

$[Cu(CH_3CN)_4](PF_6)$ (37.3 mg, 0.10 mmol, 10 mol%)

N,N'-di-*tert*-butylethylenediamine (107.8 μL, 0.50 mmol, 50 mol%)

CH_2Cl_2 (5 mL, 0.2 M)

Purification: 30% EtOAc in hexanes.

Yield of Product:

9: 201.5 mg, 0.71 mmol, 71%, red solid.

Characterization:

R_f = (ethyl acetate/hexanes 1:2): 0.24; **IR** (neat) ν = 1766, 1687, 1652, 1600, 1495, 1442, 1407, 1369, 1329, 1235, 1195, 1174, 1139 cm^{-1}; **^{1}H NMR** (500 MHz, $CDCl_3$) δ 7.60 (t, *J* = 7.4 Hz, 2H), 7.55 (t, *J* = 7.4 Hz, 1H), 7.31 (d, *J* = 7.2 Hz, 2H), 6.68 (s, 1H), 5.83 (s, 1H), 3.41 (s, 3H), 1.77 (s, 3H); **^{13}C NMR** (126 MHz, $CDCl_3$) δ 179.0, 177.1, 172.5, 152.0, 148.9, 131.8, 130.1, 126.5, 125.7, 102.8, 76.7, 53.7, 23.7; **HRMS**: Calcd. for $C_{16}H_{13}NO_4$ $[M+H]^+$ = 284.0917 m/z, found = 284.0915 m/z.

Compound S1:

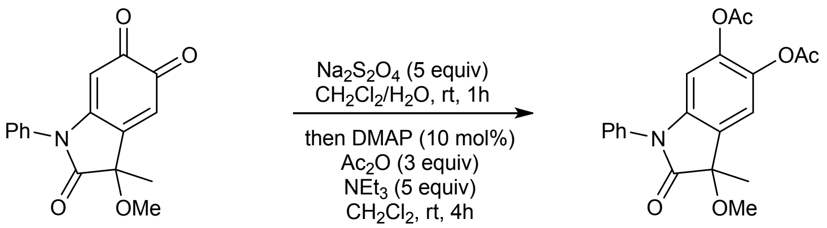

9

Chemical Formula: $C_{16}H_{13}NO_4$
Molecular Weight: 283.2787

S1

Chemical Formula: $C_{20}H_{19}NO_6$
Molecular Weight: 369.3680

Procedure: A 50 mL round-bottom flask equipped with a Teflon-coated stir bar and a rubber septum was charged with **9** (2.58 mmol, 729.8 mg, 1 equiv) and $Na_2S_2O_4$ (12.90 mmol, 2.25 g, 5 equiv) and then purged with a steady stream of N_2 for 5 min, prior to the addition of CH_2Cl_2 (10 mL) and water (10 mL). The mixture was stirred at room temperature for 1h and then poured on water (50 mL). The mixture was extracted with CH_2Cl_2 (3 × 50 mL), and the combined organic layers were dried over $MgSO_4$, filtered and concentrated *in vacuo*. The crude product was directly dissolved in dry and degassed CH_2Cl_2 (10 mL). 4-(N,N-dimethylamino)pyridine (DMAP) (0.26 mmol, 31.8 mg, 10 mol%), NEt_3 (12.90 mmol, 1.79 mL, 5 equiv) and Ac_2O (7.74 mmol, 731.7 µL, 3 equiv) was then added. And the resulting mixture was stirred at room temperature for 4h and then poured on HCl solution (50 mL, 2M). The resulting mixture was extracted with CH_2Cl_2 (3 × 50 mL), and the combined organic layers were dried over $MgSO_4$, filtered and concentrated *in vacuo*. The crude product was purified on silica gel using 30% ethyl acetate in hexanes as eluent to afford **S1** (729.4 mg, 1.97 mmol, 77% yield) as a white solid.

Characterization:

R_f = (EtOAc/Hexane 1:2): 0.26; **IR** (neat) ν = 3062, 2986, 2932, 2826, 1770, 1732, 1624, 1596, 1498, 1486, 1428, 1368, 1172, 1128 cm^{-1}; **^{1}H NMR** (500 MHz, $CDCl_3$) δ 7.53 (t, *J* = 7.8 Hz, 2H), 7.43 (t, *J* = 7.5 Hz, 1H), 7.39 (d, *J* = 7.2 Hz, 2H), 7.25 (s, 1H), 6.70 (s, 1H), 3.19 (s, 3H), 2.31 (s, 3H), 2.26 (s, 3H), 1.68 (s, 3H); **^{13}C NMR** (126 MHz, $CDCl_3$) δ 175.7, 168.4, 168.0, 143.1, 141.5, 138.3, 133.6, 129.9, 128.5, 126.3, 126.2, 119.6, 105.6, 79.4, 53.3, 24.1, 20.60, 20.57; **GC-MS** R_t = 28.06 min, m/z = 369.2, 327.2, 285.1, 254.1, 196.1, 144.1, 77.1.

Compound 10:

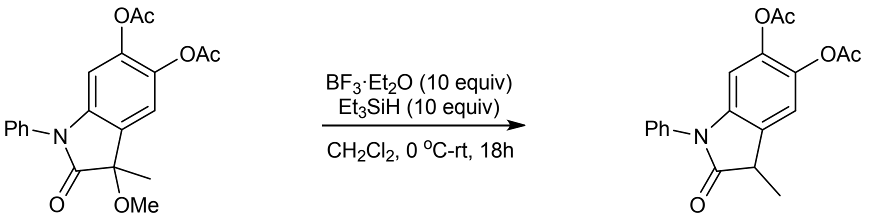

S1

Chemical Formula: $C_{20}H_{19}NO_6$
Molecular Weight: 369.3680

10

Chemical Formula: $C_{19}H_{17}NO_5$
Molecular Weight: 339.3420

Procedure: A flame-dried, 10 mL round-bottom flask equipped with a Teflon-coated stir bar and a rubber septum was charged with **S1** (0.5 mmol, 184.7 mg, 1 equiv) and then purged with a steady stream of N_2 for 5 min, prior to the addition of CH_2Cl_2 (2.5 mL, 0.2 M). The mixture was cooled to 0 °C, at which temperature $BF_3 \cdot Et_2O$ (5.0 mmol, 617.1 µL, 10 equiv) and Et_3SiH (5.0 mmol, 798.6 µL, 10 equiv) was added via syringe dropwise. The mixture was then warmed to room temperature and stirred for 18h before quenching with saturated $NaHCO_3$ (20 mL). The mixture was extracted with CH_2Cl_2 (3 × 20 mL), and the combined organic layers were dried over $MgSO_4$, filtered and concentrated *in vacuo*. The crude product was purified on silica gel using 40% ethyl acetate in hexanes as eluent to afford **10** (139.0 mg, 0.41 mmol, 82% yield) as a pale-yellow foam.

Characterization:

R$_f$ = (EtOAc/Hexane 1:1): 0.51; **IR** (neat) ν = 3059, 2978, 2934, 2873, 1768, 1720, 1626, 1596, 1498, 1486, 1430, 1368, 1326, 1204, 1167, 1113 cm^{-1}; **^{1}H NMR** (500 MHz, $CDCl_3$) δ 7.53 (dd, *J* = 8.0, 7.5 Hz, 2H), 7.45 – 7.36 (m, 3H), 7.16 (d, *J* = 1.1 Hz, 1H), 6.65 (s, 1H), 3.64 (qd, *J* = 7.6, 0.8 Hz, 1H), 2.30 (s, 3H), 2.25 (s, 3H), 1.60 (d, *J* = 7.6 Hz, 3H); **^{13}C NMR** (126 MHz, $CDCl_3$) δ 177.6, 168.7, 168.3, 142.1, 141.7, 137.7, 134.2, 129.8, 128.3, 128.2, 126.5, 119.3, 104.9, 40.6, 20.58, 20.55, 15.4; **GC-MS** R_t = 28.49 min, m/z = 339.2, 297.1, 255.1, 226.1, 77.0.

e) Reactions in Scheme 7

Compound 11:

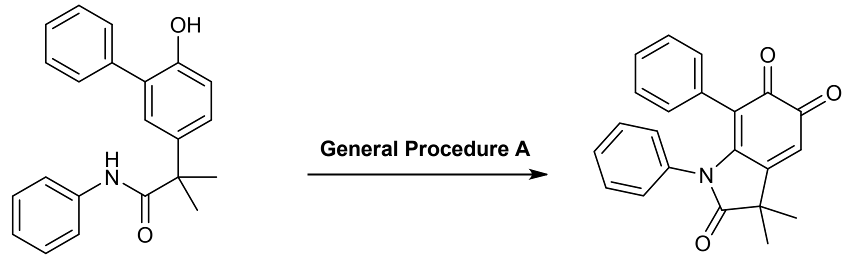

S69
Chemical Formula: $C_{22}H_{21}NO_2$
Molecular Weight: 331.4076

11
Chemical Formula: $C_{22}H_{17}NO_3$
Molecular Weight: 343.3753

Procedure: The reaction was carried out according to the General Procedure A using 30 mol% of N,N'-di-*tert*-butylethylenediamine (DBED).

Amounts of Reagents:

S69 (331.4 mg, 1.0 mmol)

$[Cu(CH_3CN)_4](PF_6)$ (14.9 mg, 0.04 mmol, 4 mol%)

N,N'-di-*tert*-butylethylenediamine (64.7 μL, 0.30 mmol, 30 mol%)

CH_2Cl_2 (5 mL, 0.2 M)

Purification: 5% EtOAc in CH_2Cl_2.

Yield of Product:

11: 302.5 mg, 0.88 mmol, 88%, orange solid.

Characterization:

R$_f$ = (ethyl acetate/CH_2Cl_2 1:20): 0.40; **IR** (neat) ν = 3059, 1751, 1740, 1681, 1651, 1609, 1587, 1495, 1383, 1337, 1302, 1243, 1192, 1160 cm^{-1}; **^{1}H NMR** (500 MHz, $CDCl_3$) δ 7.07 (t, *J* = 7.4 Hz, 1H), 7.03 – 6.95 (m, 3H), 6.91 (t, *J* = 7.5 Hz, 2H), 6.80 (d, *J* = 7.7 Hz, 2H), 6.71 (d, *J* = 7.2 Hz, 2H), 6.53 (s, 1H), 1.61 (s, 6H); **^{13}C NMR** (126 MHz, $CDCl_3$) δ 178.27, 178.25, 177.9, 157.9, 145.9, 133.1, 130.3, 129.7, 128.4, 127.4, 127.3, 127.1, 122.3, 117.4, 42.2, 24.9; **HRMS**: Calcd. for $C_{22}H_{17}NO_3$ $[M+Na]^+$ = 366.1101 m/z, found = 366.1098 m/z.

Compound 12:

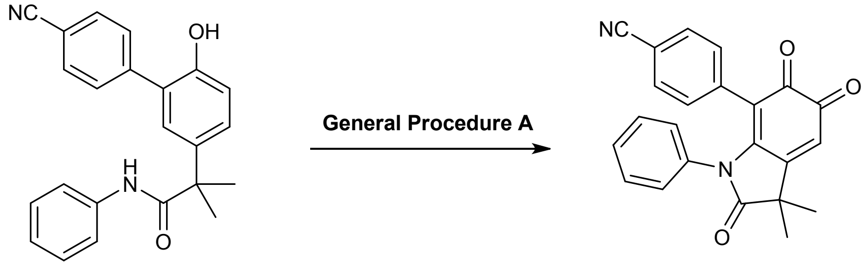

S71
Chemical Formula: $C_{23}H_{20}N_2O_2$
Molecular Weight: 356.4171

12
Chemical Formula: $C_{23}H_{16}N_2O_3$
Molecular Weight: 368.3847

Procedure: The reaction was carried out according to the General Procedure A using 30 mol% of N,N'-di-*tert*-

butylethylenediamine (DBED).

Amounts of Reagents:

S71 (356.4 mg, 1.0 mmol)

$[Cu(CH_3CN)_4](PF_6)$ (14.9 mg, 0.04 mmol, 4 mol%)

N,N'-di-*tert*-butylethylenediamine (64.7 μL, 0.30 mmol, 30 mol%)

CH_2Cl_2 (5 mL, 0.2 M)

Purification: 5% EtOAc in CH_2Cl_2.

Yield of Product:

12: 242.5 mg, 0.66 mmol, 66%, red solid.

Characterization:

R*f* = (ethyl acetate/CH_2Cl_2 1:10): 0.52; **IR** (neat) ν = 3057, 2967, 2233, 1751, 1741, 1683, 1650, 1611, 1595, 1588, 1501, 1394, 1384, 1335, 1301, 1246, 1193, 1160 cm^{-1}; ^{1}**H NMR** (500 MHz, $CDCl_3$) δ 7.22 (d, *J* = 8.3 Hz, 2H), 7.18 (d, *J* = 7.5 Hz, 1H), 7.08 (t, *J* = 7.8 Hz, 2H), 6.86 (d, *J* = 8.3 Hz, 2H), 6.81 (d, *J* = 7.4 Hz, 2H), 6.60 (s, 1H), 1.64 (s, 6H); ^{13}**C NMR** (126 MHz, $CDCl_3$) δ 177.9, 177.6, 176.8, 157.2, 146.9, 134.9, 132.8, 131.0, 130.8, 129.1, 128.8, 127.2, 123.1, 118.5, 115.2, 111.0, 42.1, 24.9; **HRMS**: Calcd. for $C_{23}H_{16}N_2O_3$ $[M+Na]^+$ = 391.1053 m/z, found = 391.1042 m/z.

Compound 13:

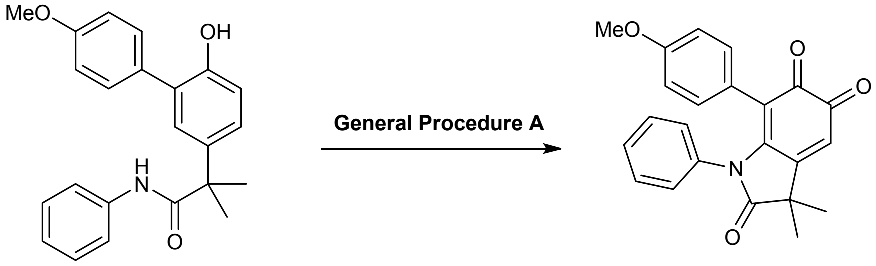

S70
Chemical Formula: $C_{23}H_{23}NO_3$
Molecular Weight: 361.4336

13
Chemical Formula: $C_{23}H_{19}NO_4$
Molecular Weight: 373.4013

Procedure: The reaction was carried out according to the General Procedure A using 30 mol% of N,N'-di-*tert*-butylethylenediamine (DBED).

Amounts of Reagents:

S70 (361.4 mg, 1.0 mmol)

$[Cu(CH_3CN)_4](PF_6)$ (14.9 mg, 0.04 mmol, 4 mol%)

N,N'-di-*tert*-butylethylenediamine (64.7 μL, 0.30 mmol, 30 mol%)

CH_2Cl_2 (5 mL, 0.2 M)

Purification: 5% EtOAc in CH_2Cl_2.

Yield of Product:

13: 326.4 mg, 0.87 mmol, 87%, red solid.

Characterization:

R*f* = (ethyl acetate/hexane 1:1): 0.44; **IR** (neat) ν = 2915, 1739, 1680, 1648, 1589, 1510, 1383, 1329, 1300, 1240, 1194, 1172 cm^{-1}; ^{1}**H NMR** (500 MHz, $CDCl_3$) δ 7.12 (t, *J* = 7.4 Hz, 1H), 7.05 (t, *J* = 7.6 Hz, 2H), 6.81 (d, *J* = 7.7 Hz, 2H), 6.64 (d, *J* = 8.7 Hz, 2H), 6.52 (s, 1H), 6.45 (d, *J* = 8.7 Hz, 2H), 3.69 (s, 3H), 1.62 (s, 6H); ^{13}**C NMR** (126 MHz, $CDCl_3$) δ 178.4, 178.192, 178.186, 158.9, 158.0, 145.4, 133.2, 131.4, 128.3, 128.2, 127.1, 122.0, 121.9, 117.2, 113.0, 55.2, 42.3, 24.9; **HRMS**: Calcd. for $C_{23}H_{19}NO_4$ $[M+Na]^+$ = 396.1206 m/z, found = 396.1220 m/z. **The structure was confirmed by X-ray crystallography.**

Compound 14:

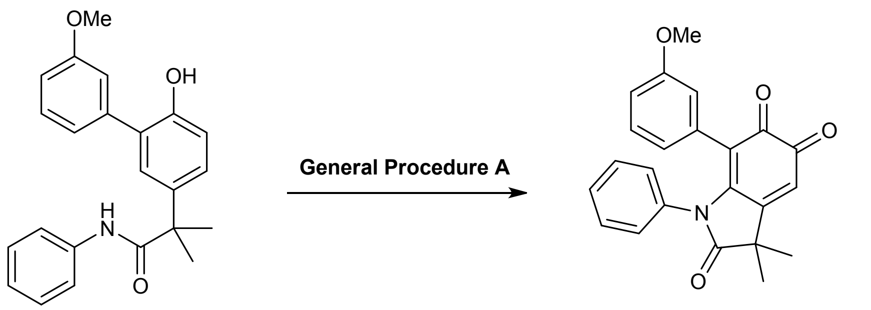

S72
Chemical Formula: $C_{23}H_{23}NO_3$
Molecular Weight: 361.4336

14
Chemical Formula: $C_{23}H_{19}NO_4$
Molecular Weight: 373.4013

Procedure: The reaction was carried out according to the General Procedure A using 30 mol% of N,N'-di-*tert*-butylethylenediamine (DBED).

Amounts of Reagents:

S72 (361.4 mg, 1.0 mmol)

$[Cu(CH_3CN)_4](PF_6)$ (14.9 mg, 0.04 mmol, 4 mol%)

N,N'-di-*tert*-butylethylenediamine (64.7 μL, 0.30 mmol, 30 mol%)

CH_2Cl_2 (5 mL, 0.2 M)

Purification: 5% EtOAc in CH_2Cl_2.

Yield of Product:

14: 341.6 mg, 0.91 mmol, 91%, red solid.

Characterization:

R_f = (ethyl acetate/CH_2Cl_2 1:20): 0.42; **IR** (neat) ν = 3059, 2972, 2936, 2834, 1746, 1683, 1650, 1578, 1486, 1456, 1380, 1332, 1300, 1252, 1224, 1187 cm^{-1}; **^{1}H NMR** (500 MHz, $CDCl_3$) δ 7.09 (t, *J* = 7.2 Hz, 1H), 7.04 (br. s, 2H), 6.86 (t, *J* = 7.9 Hz, 1H), 6.84 (br. s, 2H), 6.53 (ddd, *J* = 8.2, 2.4, 0.5 Hz, 1H), 6.52 (s, 1H), 6.38 (d, *J* = 7.6 Hz, 1H), 6.20 (dd, *J* = 2.2, 1.6 Hz, 1H), 3.61 (s, 3H), 1.61 (s, 6H); **^{13}C NMR** (126 MHz, $CDCl_3$) δ 178.3, 178.2, 177.8, 158.4, 157.8, 146.0, 133.2, 130.9, 128.5, 128.4, 128.3, 127.0, 122.9, 122.4, 117.2, 116.1, 113.4, 55.0, 42.2, 24.9; **HRMS**: Calcd. for $C_{23}H_{19}NO_4$ $[M+Na]^+$ = 396.1206 m/z, found = 396.1204 m/z.

Compound 15:

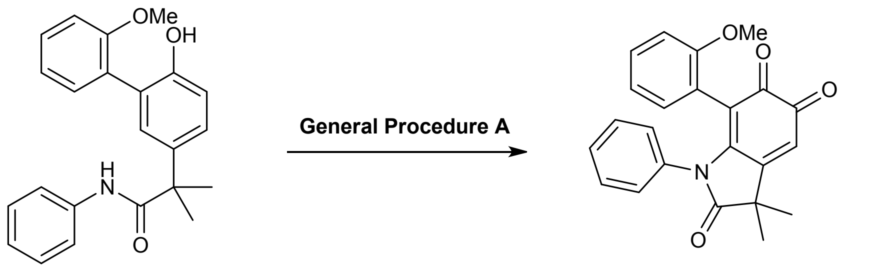

S73
Chemical Formula: $C_{23}H_{23}NO_3$
Molecular Weight: 361.4336

15
Chemical Formula: $C_{23}H_{19}NO_4$
Molecular Weight: 373.4013

Procedure: The reaction was carried out according to the General Procedure A using 30 mol% of N,N'-di-*tert*-butylethylenediamine (DBED).

Amounts of Reagents:

S73 (361.4 mg, 1.0 mmol)

$[Cu(CH_3CN)_4](PF_6)$ (14.9 mg, 0.04 mmol, 4 mol%)

N,N'-di-*tert*-butylethylenediamine (64.7 μL, 0.30 mmol, 30 mol%)

CH_2Cl_2 (5 mL, 0.2 M)

Purification: 5% EtOAc in CH_2Cl_2.

Yield of Product:

15: 347.1 mg, 0.93 mmol, 93%, red solid.

Characterization:

R*f* = (ethyl acetate/CH_2Cl_2 1:20): 0.36; **IR** (neat) ν = 3045, 2977, 2919, 1751, 1742, 1678, 1647, 1606, 1586, 1491, 1454, 1394, 1383, 1331, 1303, 1238, 1192, 1156, 1119 cm^{-1}; **^{1}H NMR** (500 MHz, $CDCl_3$) δ 7.09 (br. t, *J* = 7.0 Hz, 1H), 7.04 (t, *J* = 7.4 Hz, 1H), 7.01 – 6.95 (m, 2H), 6.92 (br. t, *J* = 7.2 Hz, 1H), 6.77 (dd, *J* = 7.5, 1.6 Hz, 1H), 6.69 (br. d, *J* = 7.3 Hz, 1H), 6.65 (td, *J* = 7.4, 0.4 Hz, 1H), 6.51 (s, 1H), 6.32 (d, *J* = 8.3 Hz, 1H), 3.55 (s, 3H), 1.62 (s, 3H), 1.59 (s, 3H); **^{13}C NMR** (126 MHz, $CDCl_3$) δ 178.8, 178.3, 177.4, 157.7, 155.9, 146.5, 132.3, 131.5, 129.7, 128.4, 127.7, 127.5, 127.4, 126.7, 122.1, 119.9, 119.1, 113.7, 109.8, 54.8, 42.3, 25.1, 24.5; **HRMS**: Calcd. for $C_{23}H_{19}NO_4$ $[M+Na]^+$ = 396.1206 m/z, found = 396.1208 m/z.

Compound 16:

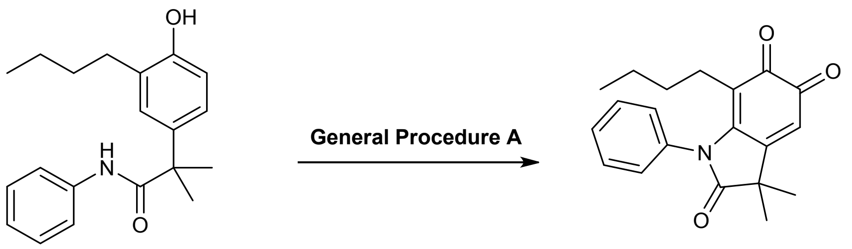

S74
Chemical Formula: $C_{20}H_{25}NO_2$
Molecular Weight: 311.4180

16
Chemical Formula: $C_{20}H_{21}NO_3$
Molecular Weight: 323.3856

Procedure: The reaction was carried out according to the General Procedure A using 30 mol% of N,N'-di-*tert*-butylethylenediamine (DBED).

Amounts of Reagents:

S74 (311.4 mg, 1.0 mmol)

$[Cu(CH_3CN)_4](PF_6)$ (14.9 mg, 0.04 mmol, 4 mol%)

N,N'-di-*tert*-butylethylenediamine (64.7 μL, 0.30 mmol, 30 mol%)

CH_2Cl_2 (5 mL, 0.2 M)

Purification: 5% EtOAc in CH_2Cl_2.

Yield of Product:

16: 263.8 mg, 0.82 mmol, 82%, red solid.

Characterization:

R*f* = (ethyl acetate/CH_2Cl_2 1:20): 0.50; **IR** (neat) ν = 3058, 2960, 2928, 2860, 1751, 1680, 1643, 1588, 1492, 1394, 1384, 1331, 1284, 1235, 1183, 1120 cm^{-1}; **^{1}H NMR** (500 MHz, $CDCl_3$) δ 7.56 – 7.49 (m, 3H), 7.33 – 7.28 (m, 2H), 6.36 (s, 1H), 1.80 – 1.74 (m, 2H), 1.54 (s, 6H), 1.07 – 0.98 (m, 2H), 0.81 (sextet, *J* = 7.3 Hz, 2H), 0.63 (t, *J* = 7.3 Hz, 3H); **^{13}C NMR** (126 MHz, $CDCl_3$) δ 178.73, 178.66, 178.5, 158.2, 146.0, 134.9, 129.9, 129.6, 128.1, 121.2, 118.5, 42.2, 31.4, 24.8, 23.3, 22.7, 13.6; **HRMS**: Calcd. for $C_{20}H_{21}NO_3$ $[M+Na]^+$ = 346.1414 m/z, found = 346.1409 m/z.

Synthesis of Q3 from *meta*-substituted phenol:

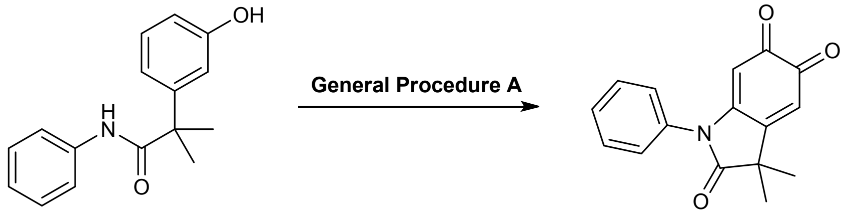

S75
Chemical Formula: $C_{16}H_{17}NO_2$
Molecular Weight: 255.3117

Q3
Chemical Formula: $C_{16}H_{13}NO_3$
Molecular Weight: 267.2793

Procedure: The reaction was carried out according to the General Procedure A.

Amounts of Reagents:

S75 (255.3 mg, 1.0 mmol)

$[Cu(CH_3CN)_4](PF_6)$ (14.9 mg, 0.04 mmol, 4 mol%)

N,N'-di-*tert*-butylethylenediamine (43.1 μL, 0.20 mmol, 20 mol%)

CH_2Cl_2 (5 mL, 0.2 M)

Yield of Product:

Q3: 33.7 mg, 0.13 mmol, 13%, red solid.

f) Reactions in Scheme 8

Compound 18:

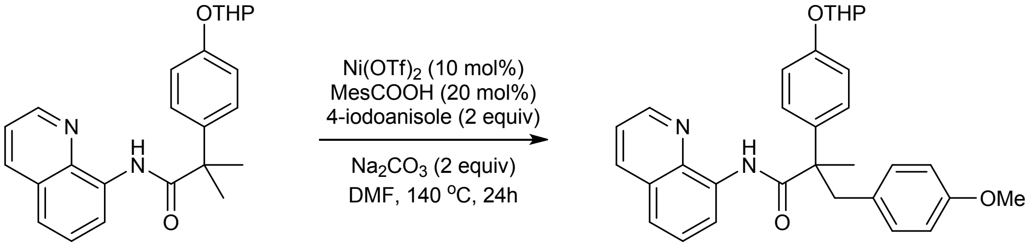

17
Chemical Formula: $C_{24}H_{26}N_2O_3$
Molecular Weight: 390.4748

18
Chemical Formula: $C_{31}H_{32}N_2O_4$
Molecular Weight: 496.5968

Procedure: The following procedure is based on the procedure of Chatani.[2] A flame-dried, 5 mL microwave vial equipped with a Teflon-coated stir bar and a rubber septum was charged with $Ni(OTf)_2$ (35.7 mg, 0.1 mmol, 0.1 equiv), 2,4,6-trimethylbenzoic acid (MesCOOH) (32.8 mg, 0.2 mmol, 0.2 equiv), **17** (390.5 mg, 1.0 mmol, 1.0 equiv), 4-iodoanisole (468.1 mg, 2.0 mmol, 2.0 equiv) and sodium carbonate (212.0 mg, 2.0 mmol, 2.0 equiv) and then purged with a steady stream of N_2 for 5 min, prior to the addition of dry and degassed DMF (2 mL). The mixture was then heated to 140 °C and stirred for 24h, quenched with water (40 mL) and diluted with ethyl acetate (40 mL). The aqueous layer was separated and extracted with ethyl acetate (2 × 40 mL), and the combined organic layers were dried over $MgSO_4$, filtered and concentrated *in vacuo*. The crude product was purified on silica gel using 15% ethyl acetate in hexanes as eluent to afford **18** (150.3 mg, 0.30 mmol, 30% yield) as a white solid. Starting material **17** (131.6 mg, 0.34 mmol) was also recovered from the reaction mixture, resulting 45% yield brsm.

Characterization:

R*f* = (EtOAc/Hexane 1:5): 0.32; **IR** (neat) ν = 3336, 2937, 1674, 1607, 1519, 1510, 1486, 1426, 1384, 1324, 1240, 1201, 1111 cm^{-1}; **^{1}H NMR** (500 MHz, Acetone-d_6) δ 9.94 (br. s, 1H), 8.81 (d, *J* = 6.5 Hz, 1H), 8.69 (d, *J* = 3.9 Hz, 1H), 8.31 (d, *J* = 8.2 Hz, 1H), 7.65 – 7.55 (m, 2H), 7.52 (dd, *J* = 8.2, 4.2 Hz, 1H), 7.42 (dd, *J* = 8.9, 2.4 Hz, 2H), 7.08 (d, *J* = 8.6 Hz, 2H), 6.92 (dd, *J* = 8.6, 3.0 Hz, 2H), 6.71 (d, *J* = 8.5 Hz, 2H), 5.46 (d, *J* = 2.4 Hz, 1H), 3.86 (t, *J* = 10.1 Hz, 1H), 3.71 (s, 3H), 3.61 – 3.51 (m, 2H), 3.31 (d, *J* = 13.5 Hz, 1H), 2.02 – 1.92 (m, 1H), 1.89 – 1.76 (m, 2H), 1.70 – 1.61 (m, 5H), 1.60 – 1.54 (m, 1H); **^{13}C NMR** (126 MHz, Acetone-d_6) δ 174.4, 158.4, 156.2, 148.5, 138.3, 136.5, 136.4, 134.8, 131.5, 129.6, 128.1, 128.0, 127.1, 121.9, 121.3, 116.4, 115.4, 113.0, 96.2, 61.6, 54.4, 52.2, 44.1, 30.2, 25.1, 22.5, 18.7 (***a complex mixture of diastereomers are observed in the ^{13}C NMR, which accounts for the presence of extra ^{13}C signals***); **HRMS**: Calcd. for $C_{31}H_{32}N_2O_4$ $[M+Na]^+$ = 519.2254 m/z, found = 519.2259 m/z.

[2] Aihara, Y.; Chatani, N. *J. Am. Chem. Soc.* **2014**, *136*, 898-901.

Compound S2:

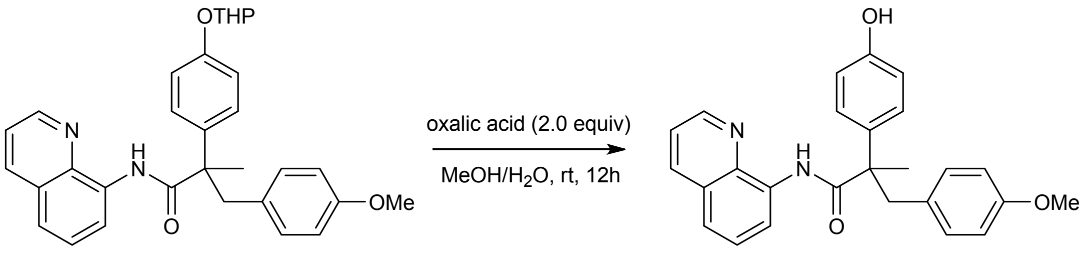

18
Chemical Formula: $C_{31}H_{32}N_2O_4$
Molecular Weight: 496.5968

S2
Chemical Formula: $C_{26}H_{24}N_2O_3$
Molecular Weight: 412.4804

Procedure: In a 50 mL round-bottom flask, **18** (437.9 mg, 0.88 mmol, 1 equiv) was dissolved in methanol and water (10 mL 9:1 by volume, 0.1 M), and oxalic acid (1.76 mmol, 158.4 mg, 2 equiv) was added directly as a solid. After stirring for 12h, the mixture was concentrated *in vacuo*, diluted with ethyl acetate (20 mL) and washed with a saturated aqueous solution of sodium bicarbonate (20 mL). The aqueous layer was extracted with ethyl acetate (2 × 20 mL), and the combined organic layers were dried over $MgSO_4$, filtered and concentrated *in vacuo*. The crude product was purified on silica gel using 50% ethyl acetate in hexanes as eluent to afford **S2** (327.5 mg, 0.79 mmol, 90% yield) as a white solid.

Characterization:

$\mathbf{R_f}$ = (EtOAc/Hexane 1:3): 0.26; **IR** (neat) ν = 3328, 3253, 3041, 2982, 1661, 1613, 1594, 1528, 1512, 1485, 1462, 1423, 1384, 1327, 1272, 1234, 1211, 1177 cm^{-1}; ^{1}**H NMR** (500 MHz, $CDCl_3$) δ 9.91 (br. s, 1H), 8.81 (dd, *J* = 6.7, 2.2 Hz, 1H), 8.70 (dd, *J* = 4.2, 1.7 Hz, 1H), 8.35 (br. s, 1H), 8.33 (dd, *J* = 8.3, 1.6 Hz, 1H), 7.63 – 7.56 (m, 2H), 7.53 (dd, *J* = 8.3, 4.2 Hz, 1H), 7.32 (d, *J* = 8.8 Hz, 2H), 6.90 (d, *J* = 8.7 Hz, 2H), 6.88 (d, *J* = 8.8 Hz, 2H), 6.71 (d, *J* = 8.7 Hz, 2H), 3.72 (s, 3H), 3.50 (d, *J* = 13.5 Hz, 1H), 3.30 (d, *J* = 13.5 Hz, 1H), 1.62 (s, 3H); ^{13}**C NMR** (126 MHz, $CDCl_3$) δ 174.7, 158.3, 156.4, 148.5, 138.3, 136.4, 134.9, 134.3, 131.5, 129.7, 128.2, 128.1, 127.1, 121.9, 121.2, 115.3, 115.3, 112.9, 54.4, 52.1, 44.2, 22.6; **HRMS**: Calcd. for $C_{26}H_{24}N_2O_3$ $[M+Na]^+$ = 435.1679 m/z, found = 435.1687 m/z.

Compound 19:

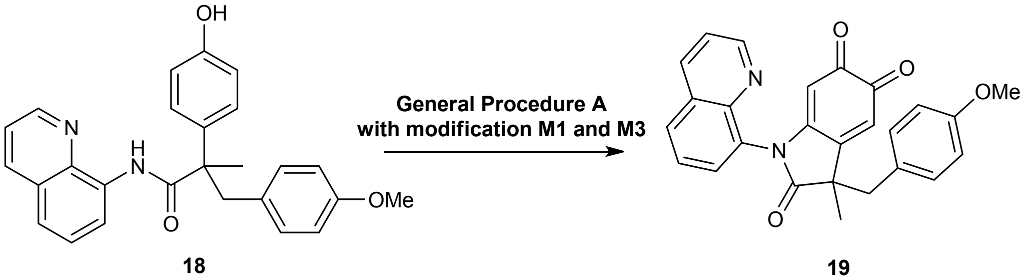

18
Chemical Formula: $C_{26}H_{24}N_2O_3$
Molecular Weight: 412.4804

19
Chemical Formula: $C_{26}H_{20}N_2O_4$
Molecular Weight: 424.4480

Procedure: The reaction was carried out according to the General Procedure A with modification M1 and M3.

Amounts of Reagents:

18 (206.2 mg, 0.5 mmol)

$[Cu(CH_3CN)_4](PF_6)$ (7.5 mg, 0.02 mmol, 4 mol%)

N,N'-di-*tert*-butylethylenediamine (21.6 μL, 0.10 mmol, 20 mol%)

1,8-diazabicyclo[5,4,0]undec-7-ene (DBU) (22.4 μL, 0.15 mmol, 30 mol%)

CH_2Cl_2 (2.5 mL, 0.2 M)

Purification: 20-50% EtOAc in CH_2Cl_2.

Yield of Product:

19: 145.4 mg, 0.34 mmol, 69%, dark-red foam, mixture of two rotatory diastereomers.

Characterization:

R_f = (ethyl acetate/CH_2Cl_2 1:1): 0.81, 0.42; **IR** (neat) ν = 2930, 1753, 1686, 1649, 1600, 1512, 1412, 1341, 1302, 1241, 1177, 1133, 1118 cm^{-1}; **^{1}H NMR** (major diastereomer, 500 MHz, $CDCl_3$) δ 8.85 (dd, *J* = 4.2, 1.6 Hz, 1H), 8.22 (dd, *J* = 8.3, 1.4 Hz, 1H), 7.97 (dd, *J* = 8.3, 1.0 Hz, 1H), 7.58 (dd, *J* = 8.1, 7.5 Hz, 1H), 7.47 (dd, *J* = 8.3, 4.2 Hz, 1H), 7.12 (d, *J* = 8.6 Hz, 2H), 7.03 (dd, *J* = 7.3, 1.1 Hz, 1H), 6.86 (d, *J* = 8.6 Hz, 2H), 6.58 (s, 1H), 5.04 (s, 1H), 3.82 (s, 3H), 3.40 (d, *J* = 13.4 Hz, 1H), 3.05 (d, *J* = 13.4 Hz, 1H), 1.80 (s, 3H); **^{1}H NMR** (minor diastereomer, 500 MHz, $CDCl_3$) δ 8.75 (dd, *J* = 4.1, 1.6 Hz, 1H), 8.24 (dd, *J* = 7.5, 1.4 Hz, 1H), 8.01 (dd, *J* = 7.8, 1.8 Hz, 1H), 7.72 – 7.65 (m, 2H), 7.48 (dd, *J* = 8.2, 4.0 Hz, 1H), 7.16 (d, *J* = 8.6 Hz, 2H), 6.83 (d, *J* = 8.7 Hz, 2H), 6.18 (s, 1H), 5.25 (s, 1H), 3.82 (s, 3H), 3.37 (d, *J* = 13.8 Hz, 1H), 3.23 (d, *J* = 13.8 Hz, 1H), 1.65 (s, 3H); **^{13}C NMR** (mixture of two diastereomers, 126 MHz, $CDCl_3$) δ 179.7, 179.6, 177.3, 177.2, 176.9, 159.2, 159.0, 155.0, 154.74, 154.71, 153.7, 151.6, 151.3, 142.97, 142.93, 136.3, 136.2, 131.9, 131.1, 130.6, 129.87, 129.83, 129.4, 129.3, 129.2, 129.1, 126.9, 126.4, 126.2, 126.1, 124.8, 124.4, 122.44, 122.40, 114.0, 113.8, 102.2, 101.7, 55.3, 55.2, 49.2, 48.1, 44.4, 43.1, 23.2, 22.6; **HRMS**: Calcd. for $C_{26}H_{20}N_2O_4$ $[M+Na]^+$ = 447.1315 m/z, found = 447.1317 m/z.

Compound 21:

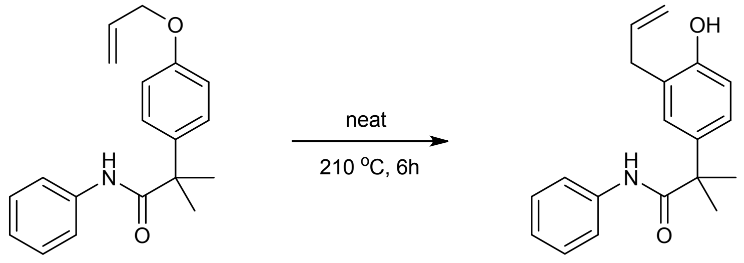

20
Chemical Formula: $C_{19}H_{21}NO_2$
Molecular Weight: 295.3755

21
Chemical Formula: $C_{19}H_{21}NO_2$
Molecular Weight: 295.3755

Procedure: A flame-dried, 5 mL microwave vial equipped with a Teflon-coated stir bar and a rubber septum was charged with compound **20** (1.00 g, 3.39 mmol, 1.0 equiv) was heated to 210 °C in a pre-heated oil bath for 6h under N_2. The mixture was then purified on silica gel using 20% ethyl acetate in hexanes as eluent to afford **21** (741.4 mg, 2.51 mmol, 74% yield) as a white solid.

Characterization:

R_f = (EtOAc/Hexane 1:3): 0.36; **IR** (neat) ν = 3344, 3278, 2975, 1658, 1598, 1512, 1500, 1442, 1384, 1356, 1320, 1256, 1233, 1177, 1144, 1120 cm^{-1}; **^{1}H NMR** (500 MHz, $CDCl_3$) δ 7.39 (d, *J* = 8.3 Hz, 2H), 7.30 (dd, *J* = 8.1, 7.6 Hz, 2H), 7.24 (d, *J* = 2.4 Hz, 1H), 7.20 (dd, *J* = 8.4, 2.5 Hz, 1H), 7.15 – 7.04 (m, 2H), 6.94 (d, *J* = 8.3 Hz, 1H), 6.91 (br. s, 1H), 6.08 (ddt, *J* = 16.7, 10.1, 6.5 Hz, 1H), 5.28 – 5.03 (m, 2H), 3.49 (d, *J* = 6.5 Hz, 2H), 1.70 (s, 6H); **^{13}C NMR** (126 MHz, $CDCl_3$) δ 177.1, 153.9, 137.7, 136.5, 135.8, 128.9, 128.5, 126.7, 125.7, 124.5, 120.2, 116.2, 116.1, 47.5, 34.9, 27.2; **HRMS**: Calcd. for $C_{19}H_{21}NO_2$ $[M+Na]^+$ = 318.1464 m/z, found = 318.1467 m/z.

Compound 22:

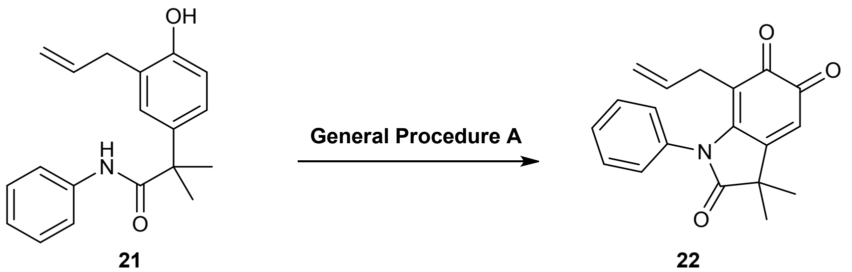

21
Chemical Formula: $C_{19}H_{21}NO_2$
Molecular Weight: 295.3755

22
Chemical Formula: $C_{19}H_{17}NO_3$
Molecular Weight: 307.3432

Procedure: The reaction was carried out according to the General Procedure A using 30 mol% of N,N′-di-*tert*-butylethylenediamine (DBED).

Amounts of Reagents:
21 (295.4 mg, 1.0 mmol)
$[Cu(CH_3CN)_4](PF_6)$ (14.9 mg, 0.04 mmol, 4 mol%)
N,N'-di-*tert*-butylethylenediamine (64.7 μL, 0.30 mmol, 30 mol%)
CH_2Cl_2 (5 mL, 0.2 M)
Purification: 5% EtOAc in CH_2Cl_2.
Yield of Product:
22: 215.4 mg, 0.70 mmol, 70%, red solid.
Characterization:
R*f* = (ethyl acetate/CH_2Cl_2 1:20): 0.62; **IR** (neat) v = 3060, 2974, 1746, 1679, 1648, 1593, 1588, 1495, 1383, 1335, 1246, 1181, 1170, 1130, 1123 cm^{-1}; **^{1}H NMR** (500 MHz, $CDCl_3$) δ 7.54 – 7.45 (m, 3H), 7.28 – 7.20 (m, 2H), 6.40 (s, 1H), 5.37 (ddt, *J* = 16.1, 10.3, 5.9 Hz, 1H), 4.80 (dd, *J* = 10.1, 1.4 Hz, 1H), 4.55 (dd, *J* = 17.1, 1.5 Hz, 1H), 2.58 (d, *J* = 5.8 Hz, 2H), 1.53 (s, 6H); **^{13}C NMR** (126 MHz, $CDCl_3$) δ 178.5, 178.44, 178.35, 158.0, 146.9, 134.5, 134.1, 130.1, 129.6, 128.4, 121.7, 115.8, 114.9, 42.2, 27.2, 24.8; **HRMS**: Calcd. for $C_{19}H_{17}NO_3$ $[M+Na]^+$ = 330.1101 m/z, found = 330.1103 m/z.

g) Reactions in Scheme 9

Compound 23:

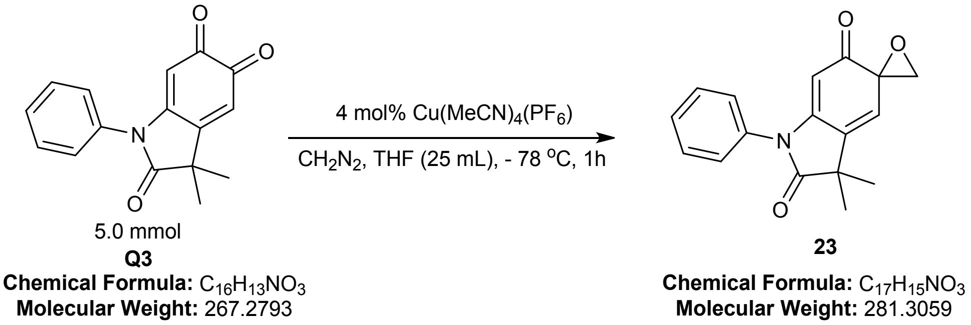

Procedure: The following procedure includes slight modifications to the original procedure of Pettus.[3] A flame-dried, 50 mL round-bottom flask equipped with a Teflon-coated stir bar and a rubber septum was charged with compound **Q3** (5.0 mmol, 1.34 g, 1.0 equiv) and $[Cu(CH_3CN)_4](PF_6)$ (74.5 mg, 0.20 mmol, 4 mol%), and then purged with a steady stream of N_2 for 5 min, prior to the addition of dry and degassed THF (25 mL). The reaction was cooled down to -78 °C, at which temperature diazomethane (0.5 M in Et_2O) was added dropwise until the color changed from dark red to green. The mixture was then stirred at this temperature for 1h, and then quenched by the addition of AcOH (5 mL), and warmed to room temperature. The reaction mixture was then poured onto H_2O (50 mL) and extracted with EtOAc (3 × 50 mL). The combined organic fractions were washed with a saturated aqueous solution of $NaHCO_3$ (50 mL), and then dried over $MgSO_4$. The reaction mixture was then filtered and concentrated *in vacuo*, and the residue was purified on silica gel using 30% ethyl acetate in hexanes as eluent to afford **23** (1.21 g, 4.30 mmol, 86% yield) as a yellow solid.
Characterization:
R*f* = (ethyl acetate/hexane 1:2): 0.21; **IR** (neat) v = 3063, 2976, 1743, 1682, 1652, 1578, 1490, 1412, 1363, 1170, 1109, 901, 850, 739, 664 cm^{-1}; **^{1}H NMR** (400 MHz, $CDCl_3$) δ 7.53 (t, *J* = 7.4 Hz, 2H), 7.46 (t, *J* = 7.4 Hz, 1H), 7.28 (d, *J* = 7.4 Hz, 2H), 6.07 (s, 1H), 5.64 (s, 1H), 3.43 (d, *J* = 7.7 Hz, 1H), 3.16 (d, *J* = 7.7 Hz, 1H), 1.51 (s, 3H), 1.50 (s, 3H); **^{13}C NMR** (75 MHz, $CDCl_3$) δ 191.3, 178.7, 155.9, 141.7, 132.6, 130.3, 129.8, 129.4, 126.7, 99.9, 58.0, 56.0, 41.9, 25.2, 24.9; **HRMS**: Calcd. for $C_{17}H_{15}NO_3$ $[M+Na]^+$ = 304.0944 m/z, found = 304.0953 m/z. **The structure was confirmed by X-ray crystallography.**

[3] Miller, L. A.; Marsini, M. A.; Pettus, T. R. R. *Org. Lett.* **2009**, *11*, 1955-1958.

Compound 24:

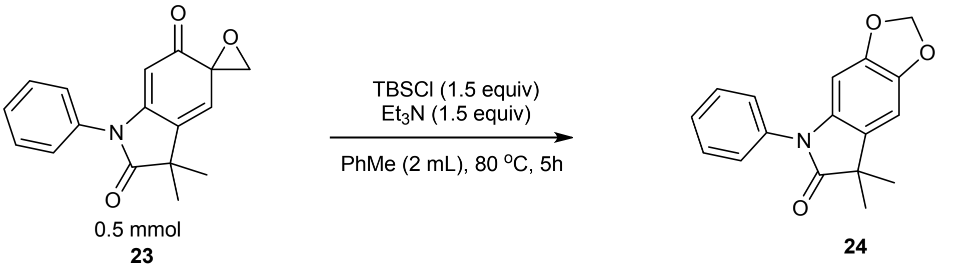

23
Chemical Formula: $C_{17}H_{15}NO_3$
Molecular Weight: 281.3059

24
Chemical Formula: $C_{17}H_{15}NO_3$
Molecular Weight: 281.3059

Procedure: The following procedure is based on the procedure of Gesson.[4] A flame-dried, 5 mL round-bottom flask equipped with a Teflon-coated stir bar and a rubber septum was charged with compound **23** (0.5 mmol, 140.7 mg, 1.0 equiv) and then purged with a steady stream of N_2 for 5 min, prior to the addition of dry and degassed PhMe (2 mL). *tert*-Butyldimethylsilyl chloride (TBSCl) (0.75 mmol, 113.0 mg, 1.5 equiv) was then added as a solid and neat Et_3N (0.75 mmol, 104.5 μL, 1.5 equiv) was then added via syringe, and the mixture was stirred at 80 °C for 5h, before quenching with a saturated aqueous solution of $NaHCO_3$ (10 mL). The mixture was extracted with EtOAc (3 × 10 mL), and the combined organic fractions were dried over $MgSO_4$. The reaction mixture was then filtered and concentrated *in vacuo*, the residue was purified on silica gel using 10% ethyl acetate in hexanes as eluent to afford **24** (61.3 mg, 0.22 mmol, 44% yield) as a yellow solid.

Characterization:

R_f = (ethyl acetate/hexane 1:5): 0.35; **IR** (neat) ν = 3044, 2964, 2925, 2867, 1706, 1626, 1613, 1596, 1500, 1468, 1384, 1237, 1176, 1153, 1037, 932, 892, 857, 746, 721, 696 cm^{-1}; **^{1}H NMR** (400 MHz, $CDCl_3$) δ 7.51 (t, *J* = 7.7 Hz, 2H), 7.44 – 7.33 (m, 3H), 6.79 (s, 1H), 6.45 (s, 1H), 5.91 (s, 2H), 1.45 (s, 6H); **^{13}C NMR** (75 MHz, $CDCl_3$) δ 180.8, 146.8, 143.6, 136.5, 134.7, 129.5, 127.8, 127.6, 126.4, 104.0, 101.0, 93.3, 44.6, 24.9; **HRMS**: Calcd. for $C_{17}H_{15}NO_3$ $[M+H]^+$ = 282.1125 m/z, found = 282.1123 m/z.

Compound 25:

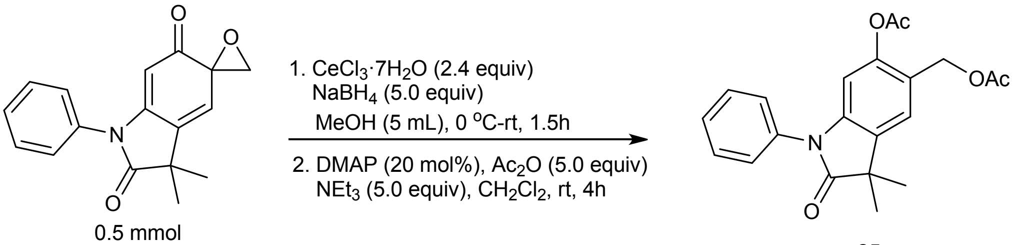

23
Chemical Formula: $C_{17}H_{15}NO_3$
Molecular Weight: 281.3059

25
Chemical Formula: $C_{21}H_{21}NO_5$
Molecular Weight: 367.3951

Procedure: The following procedure includes slight modifications to the original procedure of Waldmann.[5] A flame-dried, 10 mL round-bottom flask equipped with a Teflon-coated stir bar and a rubber septum was charged with compound **23** (0.5 mmol, 140,7 mg, 1.0 equiv), $CeCl_3$ heptahydrate (1.2 mmol, 447.1 mg, 2.4 equiv) and $NaBH_4$ (2.5 mmol, 94.6 mg, 5.0 equiv) and then purged with a steady stream of N_2 for 5 min, prior to the addition of dry and degassed methanol (5 mL) at 0 °C. The resulting slurry was allowed to stir at 0 °C for 1h, and warmed up to room temperature for another 30 min, before quenching with saturated NH_4Cl (10 mL). The mixture was extracted with EtOAc (3 × 10 mL). Combined organic layers were dried over $MgSO_4$, filtered, and concentrated *in vacuo*. The crude product was directly dissolved in DCM (5 mL), and added 4-dimethylaminopyridine (DMAP) (0.1 mmol, 12.2 mg, 0.2 equiv), Et_3N (2.5 mmol, 348.5 μL, 5.0 equiv) and Ac_2O (2.5 mmol, 236.3 μL, 5.0 equiv). The mixture was stirred at room temperature for 4h, before quenching with

[4] Gesson, J.-P.; Mondon, M.; Pokrovska, N. *Synlett* **1997**, *12*, 1395-1396.
[5] Hinterding, K.; Knebel, A.; Herrlich, P.; Waldmann, H. *Bioorg. Med. Chem.* **1998**, *6*, 1153-1162.

water (10 mL). The aqueous layer was extracted with EtOAc (3 × 10 mL), and combined organic layers were dried over $MgSO_4$. It was filtered and concentrated *in vacuo*. The crude product was purified on silica gel using 15% ethyl acetate in hexanes as eluent to afford **25** (100.2 mg, 0.27 mmol, 55% yield) as a brownish oil.

<u>Characterization:</u>

R_f = (ethyl acetate/hexane 1:5): 0.20; **IR** (neat) ν = 3018, 2970, 2930, 1765, 1726, 1625, 1598, 1503, 1487, 1369, 1330, 1222, 1192, 1143, 1069, 1022, 902, 749, 696, 666, 646 cm^{-1}; **^{1}H NMR** (500 MHz, $CDCl_3$) δ 7.53 (t, *J* = 7.8 Hz, 2H), 7.44 – 7.38 (m, 3H), 7.34 (s, 1H), 6.61 (s, 1H), 5.08 (s, 2H), 2.30 (s, 3H), 2.08 (s, 3H), 1.51 (s, 6H); **^{13}C NMR** (126 MHz, $CDCl_3$) δ 180.6, 170.7, 169.4, 149.0, 143.8, 134.2, 133.1, 129.7, 128.2, 126.4, 125.0, 122.3, 104.5, 61.3, 44.1, 24.7, 20.9, 20.8; **HRMS**: Calcd. for $C_{21}H_{21}NO_5$ $[M+Na]^+$ = 390.1312 m/z, found = 390.1312 m/z.

<u>Compound 26</u>:

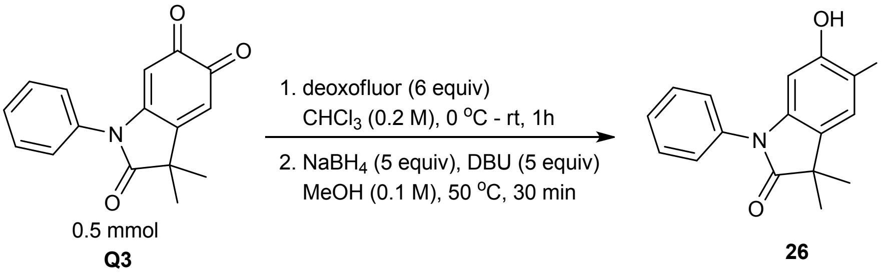

Chemical Formula: $C_{16}H_{13}NO_3$
Molecular Weight: 267.2793

Chemical Formula: $C_{16}H_{14}FNO_2$
Molecular Weight: 271.2863

<u>Procedure</u>: The following procedure includes the procedure of Akai.[6] A flame-dried, 10 mL round-bottom flask equipped with a Teflon-coated stir bar and a rubber septum was charged with compound **Q3** (0.5 mmol, 133.6 mg, 1.0 equiv) and then purged with a steady stream of N_2 for 5 min, prior to the addition of dry and degassed $CHCl_3$ (2.5 mL). The solution was cooled to 0 °C, and deoxofluor (1.33 mL, 50% solution in PhMe) was added dropwise. After the addition, the flask was warmed up to room temperature, and stirred for 1h. The reaction was quenched with water (10 mL), and extracted with CH_2Cl_2 (3 × 10 mL). The combined organic fractions were washed with saturated $NaHCO_3$ (10 mL), and then dried over $MgSO_4$. After filtered and concentrated *in vacuo*, the residue was purified on silica gel using 15% ethyl acetate in hexanes as eluent to afford a mixture of the difluoroketone intermediate, which was directly subjected to the next step without further purification. The mixture was dissolved in dry and degassed MeOH (5 mL). 1,8-Diazabicycloundec-7-ene (DBU) (2.5 mmol, 373.9 µL, 5 equiv) and $NaBH_4$ (2.5 mmol, 94.6 mg, 5 equiv) was then added at room temperature. The mixture was stirred at 50 °C for 30 min, before quenching with saturated NH_4Cl aqueous solution (10 mL). The aqueous layer was extracted with EtOAc (3 × 10 mL), and combined organic layers were dried over $MgSO_4$. It was filtered and concentrated *in vacuo*. The crude product was purified on silica gel using 20% ethyl acetate in hexanes as eluent to afford **26** (59.7 mg, 0.22 mmol, 44% overall yield) as a white solid.

<u>Characterization:</u>

R_f = (ethyl acetate/hexane 1:5): 0.17; **IR** (neat) ν = 3259, 2975, 2917, 2849, 1696, 1631, 1592, 1502, 1490, 1458, 1446, 1385, 1368, 1320, 1274, 1215, 1181, 939, 865, 798, 761, 723, 692, 650, 603, 587 cm^{-1}; **^{1}H NMR** (500 MHz, $CDCl_3$) δ 7.48 (t, *J* = 7.7 Hz, 2H), 7.40 – 7.35 (m, 3H), 6.99 (d, *J* = 9.5 Hz, 1H), 6.51 (d, *J* = 7.2 Hz, 1H), 5.81 (br. s, 1H), 1.44 (s, 6H); **^{13}C NMR** (126 MHz, $CDCl_3$) δ 181.0, 147.8 (d, J_F = 232.7 Hz), 143.2 (d, J_F = 15.6 Hz), 138.6 (d, J_F = 2.2 Hz), 134.3, 129.6, 128.0, 126.9 (d, J_F = 6.4 Hz), 126.4, 110.4 (d, J_F = 21.2 Hz), 99.6, 44.4 (d, J_F = 1.4 Hz), 24.8; **^{19}F NMR** (282 MHz, $CDCl_3$) δ -147.34 – -147.45 (m, 1F); **HRMS**: Calcd. for $C_{16}H_{14}FNO_2$ $[M+Na]^+$ = 294.0901 m/z, found = 294.0897 m/z.

Regio-chemistry was confirmed by NOE.

[6] Nemoto, H.; Nishiyama, T.; Akai, S. *Org. Lett.* **2011**, *13*, 2714-2717.

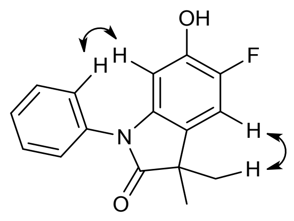

Compound 27:

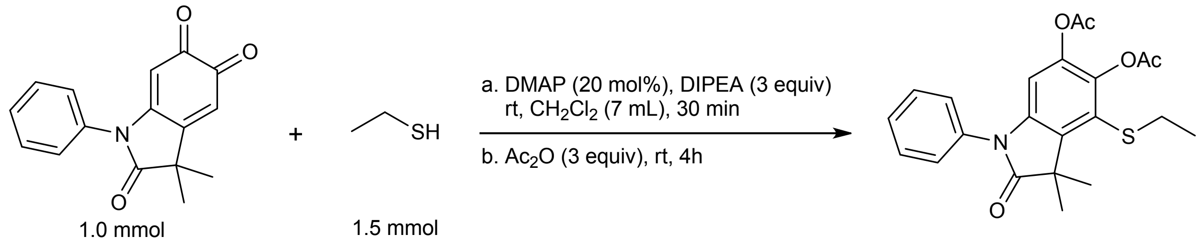

Q3
Chemical Formula: $C_{16}H_{13}NO_3$
Molecular Weight: 267.2793

27
Chemical Formula: $C_{22}H_{23}NO_5S$
Molecular Weight: 413.4867

Procedure: A flame-dried, 50 mL round-bottom flask equipped with a Teflon-coated stir bar and a rubber septum was charged with compound **Q3** (1.0 mmol, 267.3 mg, 1.0 equiv) and N,N-dimethylamino pyridine (DMAP) (0.2 mmol, 24.4 mg, 0.2 equiv), and then purged with a steady stream of N_2 for 5 min, prior to the addition of dry and degassed CH_2Cl_2 (5 mL) and dry N,N-di-isopropylethylamine (DIPEA) (1.0 mmol, 174.2 μL, 1.0 equiv). In a separate, flame-dried 5 mL microwave vial ethanethiol (1.5 mmol, 108.2 μL, 1.5 equiv) and DIPEA (2.0 mmol, 348.4 μL, 2.0 equiv) were dissolved in CH_2Cl_2. (2.0 mL), and added to the solution of quinone via syringe to afford a final volume of 7.0 mL. The reaction was stirred at room temperature for 30 min under N_2, at which point Ac_2O (3.0 mmol, 283.6 μL, 3.0 equiv) was added dropwise. The mixture was then stirred at room temperature for another 4h, then quenched by the addition of $NaHSO_4$ (20 mL, 10% by weight aqueous solution). The phases were then separated and the aqueous phase was extracted with CH_2Cl_2 (3 × 20 mL). The combined organic fractions were then dried over $MgSO_4$, filtered and concentrated *in vacuo*. The residue was purified on silica gel using 15% ethyl acetate in hexanes as eluent to afford **27** (378.3 mg, 0.91 mmol, 91% yield) as a white solid.

Characterization:

R_f = (ethyl acetate/hexane 1:5): 0.19; **IR** (neat) ν = 3018, 2971, 2931, 2870, 1774, 1724, 1607, 1591, 1502, 1453, 1418, 1368, 1194, 1180, 1151, 1092, 1010, 920, 878, 747, 693, 665 cm^{-1}; **^{1}H NMR** (500 MHz, $CDCl_3$) δ 7.50 (t, *J* = 7.7 Hz, 2H), 7.39 (t, *J* = 7.5 Hz, 1H), 7.36 (dd, *J* = 8.4, 1.2 Hz, 2H), 6.63 (s, 1H), 2.91 (q, *J* = 7.4 Hz, 2H), 2.34 (s, 3H), 2.21 (s, 3H), 1.68 (s, 6H), 1.28 (t, *J* = 7.4 Hz, 3H); **^{13}C NMR** (126 MHz, $CDCl_3$) δ 180.4, 168.2, 168.0, 142.3, 141.1, 139.9, 135.6, 134.0, 129.7, 128.3, 127.0, 126.7, 105.1, 46.6, 30.2, 23.4, 20.6, 20.5, 14.9; **HRMS**: Calcd. for $C_{22}H_{23}NO_5S$ $[M+H]^+$ = 414.1370 m/z, found = 414.1366 m/z.

Regio-chemistry was confirmed by NOE.

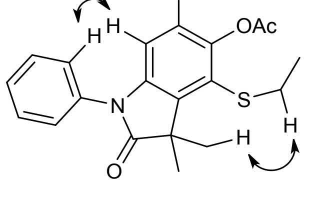

Compound 28:

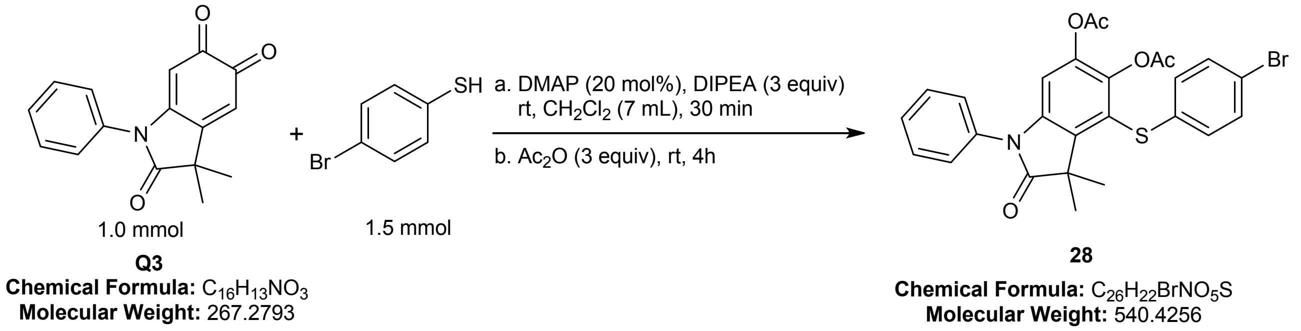

Procedure: A flame-dried, 50 mL round-bottom flask equipped with a Teflon-coated stir bar and a rubber septum was charged with compound **Q3** (1.0 mmol, 267.3 mg, 1.0 equiv) and *N,N*-dimethylamino pyridine (DMAP) (0.2 mmol, 24.4 mg, 0.2 equiv), and then purged with a steady stream of N_2 for 5 min, prior to the addition of dry and degassed CH_2Cl_2 (5 mL) and dry *N,N*-di-isopropylethylamine (DIPEA) (1.0 mmol, 174.2 µL, 1.0 equiv). In a separate, flame-dried 5 mL microwave vial 4-bromobenzenethiol (1.5 mmol, 283.6 mg, 1.5 equiv) and DIPEA (2.0 mmol, 348.4 µL, 2.0 equiv) were dissolved in CH_2Cl_2. (2.0 mL), and added to the solution of quinone via syringe to afford a final volume of 7.0 mL. The reaction was stirred at room temperature for 30 min under N_2, at which point Ac_2O (3.0 mmol, 283.6 µL, 3.0 equiv) was added dropwise. The mixture was then stirred at room temperature for another 4h, then quenched by the addition of $NaHSO_4$ (20 mL, 10% by weight aqueous solution). The phases were then separated and the aqueous phase was extracted with CH_2Cl_2 (3 × 20 mL). The combined organic fractions were then dried over $MgSO_4$, filtered and concentrated *in vacuo*. The residue was purified on silica gel using 15% ethyl acetate in hexanes as eluent to afford **28** (264.5 mg, 0.49 mmol, 49% yield) as a purple solid.

Characterization:

R_f = (ethyl acetate/hexane 1:5): 0.22; **IR** (neat) ν = 3016, 2971, 2931, 2870, 1765, 1720, 1608, 1591, 1502, 1473, 1420, 1369, 1322, 1190, 1152, 1085, 1005, 919, 873, 811, 746, 692, 665, 613, 573 cm^{-1}; **^{1}H NMR** (500 MHz, $CDCl_3$) δ 7.55 (t, *J* = 7.8 Hz, 2H), 7.46 – 7.41 (m, 3H), 7.39 (d, *J* = 8.6 Hz, 2H), 7.02 (d, *J* = 8.6 Hz, 2H), 6.79 (s, 1H), 2.21 (s, 3H), 2.06 (s, 3H), 1.65 (s, 6H); **^{13}C NMR** (126 MHz, $CDCl_3$) δ 180.1, 168.0, 167.8, 143.0, 141.6, 139.5, 136.3, 135.4, 133.8, 132.2, 129.9, 128.6, 128.4, 126.7, 123.6, 119.8, 106.5, 46.7, 23.3, 20.6, 20.0; **HRMS**: Calcd. for $C_{26}H_{22}BrNO_5S$ $[M+H]^+$ = 562.0294 m/z, found = 562.0287 m/z.

Regio-chemistry was confirmed by NOE.

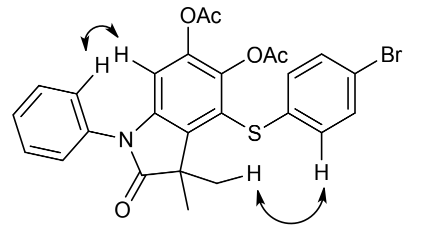

Compound 29:

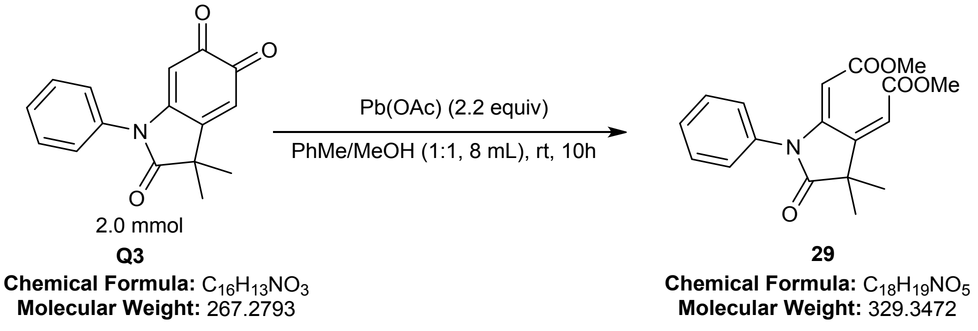

Procedure: The following procedure includes the procedure of Gilheany.[7] A flame-dried, 25 mL round-bottom flask

equipped with a Teflon-coated stir bar and a rubber septum was charged with compound **Q3** (2.0 mmol, 534.6 mg, 1.0 equiv) and then purged with a steady stream of N_2 for 5 min, prior to the addition of dry and degassed PhMe (4 mL) and MeOH (4 mL). $Pb(OAc)_4$ (4.4 mmol, 1.95 g, 2.2 equiv) was then added as a solid. The mixture was stirred at room temperature for 10h, before quenching with saturated $NaHCO_3$ (40 mL). It was extracted with EtOAc (3 × 40 mL), and the combined organic fractions were dried over $MgSO_4$, filtered and concentrated *in vacuo*, the residue was purified on silica gel using 20% ethyl acetate in hexanes as eluent to afford **29** (384.9 mg, 1.17 mmol, 58% yield) as a yellow solid.

Characterization:

R*f* = (ethyl acetate/hexane 1:2): 0.64; **IR** (neat) ν = 2995, 2953, 1741, 1721, 1713, 1641, 1631, 1493, 1453, 1438, 1396, 1355, 1316, 1279, 1214, 1147, 1015, 1002, 906, 860, 816, 756, 724, 708, 581 cm^{-1}; **^{1}H NMR** (500 MHz, $CDCl_3$) δ 7.48 (t, *J* = 7.7 Hz, 2H), 7.40 (t, *J* = 7.5 Hz, 1H), 7.22 (d, *J* = 7.3 Hz, 2H), 6.10 (s, 1H), 5.33 (s, 1H), 3.70 (s, 3H), 3.58 (s, 3H), 1.42 (s, 6H); **^{13}C NMR** (126 MHz, $CDCl_3$) δ 176.3, 166.2, 165.6, 149.1, 147.6, 133.6, 129.9, 129.1, 127.6, 119.8, 100.6, 51.7, 51.3, 47.0, 23.4; **HRMS**: Calcd. for $C_{18}H_{19}NO_5$ $[M+H]^+$ = 330.1336 m/z, found = 330.1331 m/z. **The structure was confirmed by X-ray crystallography.**

Compound 30:

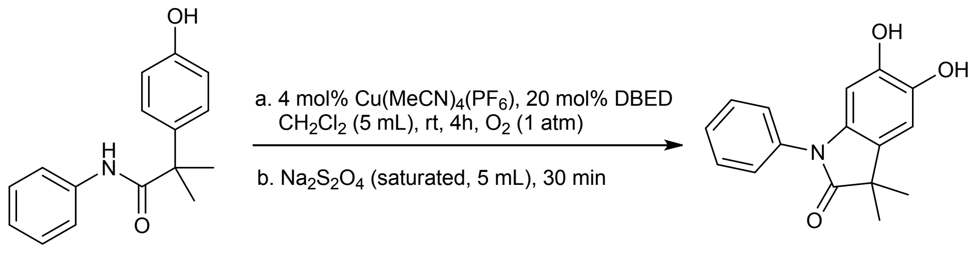

1.0 mmol
P1
Chemical Formula: $C_{16}H_{17}NO_2$
Molecular Weight: 255.3117

30
Chemical Formula: $C_{16}H_{15}NO_3$
Molecular Weight: 269.2952

Procedure: A flame-dried, 25 mL Radley tube equipped with a Teflon-coated stir bar and a rubber septum was charged with compound **P1** (1.0 mmol, 255.3 mg, 1.0 equiv) and then purged with a steady stream of N_2 for 5 min, prior to the addition of dry and degassed CH_2Cl_2 (4 mL). In a separated, flame-dried 5 mL microwave vial $[Cu(CH_3CN)_4](PF_6)$ (14.9 mg, 0.04 mmol, 4 mol%) and N,N'-di-*tert*-butylethylenediamine (43.1 μL, 0.20 mmol, 20 mol%) were dissolved in CH_2Cl_2. (1.0 mL, 0.04 M) to afford a homogeneous pink solution. This solution was then added to the Radley tube via syringe to afford a final volume of 5.0 mL and a phenol concentration 0.2 M solution. The rubber septum was then rapidly removed and replaced with a Radley cap, which was connected to a tank of O_2 and pressurize to 1 atm. Under a constant pressure of O_2 (1 atm), the reaction was vented 3 times for 10 s to remove N_2. A dramatic color change was observed within 2 min, resulting in a blackish/brown reaction mixture. The reaction mixture was then stirred at room temperature for 4 h, and then a saturated aqueous solution of $Na_2S_2O_4$ (5 mL) was added, and the resulting biphasic mixture was stirred for another 30 min under N_2. The phases were then separated and the aqueous phase was extracted with CH_2Cl_2 (3 × 20 mL). The combined organic fractions were then dried over $MgSO_4$, filtered and concentrated *in vacuo*. The residue was purified on silica gel using 40% ethyl acetate in hexanes as eluent to afford **30** (204.5 mg, 0.76 mmol, 76% yield) as a yellow solid.

Characterization:

R*f* = (ethyl acetate/hexane 1:2): 0.19; **IR** (neat) ν = 3555, 3541, 3222, 2972, 1684, 1621, 1491, 1467, 1452, 1334, 1207, 1175, 1154, 939, 881, 798, 751, 693, 648 cm^{-1}; **^{1}H NMR** (400 MHz, $CDCl_3$) δ 7.42 (t, *J* = 6.7 Hz, 2H), 7.38 – 7.27 (m, 3H), 6.77 (br. s, 2H), 6.61 (br. s, 1H), 6.39 (s, 1H), 1.37 (s, 6H); **^{13}C NMR** (75 MHz, $CDCl_3$) δ 182.4, 143.8, 140.6, 134.9, 134.3, 129.5, 128.1, 126.4, 110.3, 98.7, 44.8, 24.8; **HRMS**: Calcd. for $C_{16}H_{15}NO_3$ $[M+Na]^+$ = 292.0944 m/z, found = 292.0941 m/z.

6. Synthesis and Characterization of Substrates and Precursors

[7] Walsh, J. G.; Furlong, P. J.; Byrne, L. A.; Gilheany, D. G. *Tetrahedron* **1999**, *55*, 11519-11536.

a) General Procedures

General Procedure B: ***synthesis of the esters.*** A flame-dried, 25 mL round-bottom flask equipped with a Teflon-coated stir bar and a rubber septum was charged with compound **S9** (5 mmol, 1.32 g, 1.0 equiv) and then purged with a steady stream of N_2 for 5 min, prior to the addition of dry and degassed THF (8 mL). A solution of lithium diisopropylamide (LDA) (2.0 M, 6.0 mmol, 3.0 mL, 1.2 equiv) was then added dropwise at -78 ^oC, and the resulting mixture was stirred at this temperature for 15 min. A separate, flame-dried, 5 mL round-bottom flask equipped with a Teflon-coated stir bar and a rubber septum was charged with the alkyl halide (6.0-7.5 mmol, 1.2-1.5 equiv) and then purged with a steady stream of N_2 for 5 min, prior to the addition of dry and degassed THF (2 mL) to afford a homogeneous solution. This solution was then added to the solution of **S9** in THF dropwise via syringe. The reaction was stirred at -78 ^oC for 4h, and then warmed to room temperature and stirred for another 2h. The mixture was diluted with water (50 mL) and extracted with ethyl acetate (3 × 50 mL). The combined organic layers were dried over $MgSO_4$, filtered and concentrated *in vacuo*. The crude product was then purified on silica gel using hexanes/ethyl acetate as eluent.

General Procedure C: ***amidation of the esters with NaHMDS and THP deprotection.*** The following procedure is based on the procedure of Wang.[8] A flame-dried, 50 mL round-bottom flask equipped with a Teflon-coated stir bar and a rubber septum was charged with the aniline (3.6-4.5 mmol, 1.2-1.5 equiv) and then purged with a steady stream of N_2 for 5 min, prior to the addition of dry and degassed THF (10 mL). Sodium bis(trimethylsilyl)amide (NaHMDS) (6 mmol, 6.0 mL, 1.0 M in THF, 2 equiv) was added dropwise at 0 ^oC and stirred for 20 min. A separate, flame-dried, 5 mL round-bottom flask equipped with a Teflon-coated stir bar and a rubber septum was charged with the ester (3 mmol, 1.0 equiv) and then purged with a steady stream of N_2 for 5 min, prior to the addition of dry and degassed THF (5 mL) to afford a homogeneous solution. This solution was then added to the solution of the aniline and NaHMDS in THF dropwise via syringe at 0 ^oC. The mixture was then warmed to room temperature and stirred for 4h, quenched with saturated ammonium chloride (30 mL) and diluted with ethyl acetate (30 mL). The aqueous layer was separated and extracted with ethyl acetate (3 × 60 mL), and the combined organic layers were dried over $MgSO_4$, filtered and concentrated *in vacuo*. The resulting crude mixture was dissolved in methanol and water (30 mL 9:1 by volume, 0.1 M), and oxalic acid (6 mmol, 540.2 mg, 2 equiv) was added directly as a solid. After stirring for 12h, the mixture was concentrated *in vacuo*, diluted with ethyl acetate (60 mL) and washed with a saturated aqueous solution of sodium bicarbonate (60 mL). The aqueous layer was extracted with ethyl acetate (3 × 60 mL), and the combined organic layers were dried over $MgSO_4$, filtered and concentrated *in vacuo*. The crude product was then purified on silica gel using hexanes/ethyl acetate as eluent to afford the phenol.

Modification of the General Procedure C (M4): ***modified reaction temperature (compound S47).*** The following change was made to the standard reaction procedure. During the amidation step, the reaction was performed at 60 ^oC for 4h. The reaction was then carried out as described in the general procedure.

General Procedure D: ***amidation of the acids with EDC and THP deprotection.*** A flame-dried, 50 mL round-bottom flask equipped with a Teflon-coated stir bar and a rubber septum was charged with the acid (3 mmol, 1.0 equiv) and then purged with a steady stream of N_2 for 5 min, prior to the addition of dry and degassed THF (15 mL, 0.2 M). 1-Ethyl-3-(3-dimethylaminopropyl)carbodiimide (EDC) (4.5 mmol, 698.6 mg, 1.5 equiv) and 1-hydroxybenzotriazole (HOBt) (4.5 mmol, 608.0 mg, 1.5 equiv) were then added as solids, followed by *N*-methylmorpholine (30 mmol, 3.3mL, 10 equiv) and the amine (3.6-6.0 mmol, 1.2-2.0 equiv) via syringe at room temperature. After stirring for 18 h, the mixture was quenched with a 10% aqueous solution of sodium hydroxide (30 mL) and extracted with ethyl acetate (3 × 60 mL). The combined organic layers were dried over $MgSO_4$, filtered and concentrated *in vacuo*. The resulting crude mixture was dissolved in methanol and water (30 mL 9:1 by volume, 0.1 M), and oxalic acid (6 mmol, 540.2 mg, 2 equiv) was added as a solid. After stirring for 12h, the mixture was concentrated *in vacuo*, diluted with ethyl acetate (60 mL) and washed with saturated sodium bicarbonate solution (60 mL). The aqueous layer was extracted with ethyl acetate (3 × 60 mL), and the combined organic layers were dried over $MgSO_4$, filtered and concentrated *in vacuo*. The crude product was then purified on silica

---

[8] Wang, J.; Rosingana, M.; Discordia, R. P.; Soundararajan, N.; Polniaszek, R. *SynLett* **2001**, *9*, 1485-1487.

gel using hexanes/ethyl acetate as eluent to afford the phenol.

General Procedure E: *amidation of the acids with CDI and THP deprotection.* A flame-dried, 50 mL sealed-tube equipped with a Teflon-coated stir bar and a rubber septum was charged with the acid (3 mmol, 1.0 equiv) and then purged with a steady stream of N_2 for 5 min, prior to the addition of dry and degassed THF (15 mL, 0.2 M). N,N'-carbonyldiimidazole (CDI) (3.3 mmol, 535.1 mg, 1.1 equiv) was then added as a solid and the solution was stirred at 35 °C for 4h and then concentrated *in vacuo*. The resulting yellow oil was dissolved in dry and degassed THF (15 mL, 0.2 M) and DBU (3 mmol, 448.6 μL, 1 equiv) and the aniline (3.6 mmol, 1.2 equiv) were added via syringe under N_2 at room temperature. The septum was then replaced with a high-pressure Teflon coated screw cap, and the reaction mixture was stirred at 80 °C for 24h, before cooling to room temperature and quenching with a 10% aqueous solution of sodium hydroxide (30 mL) and extracted with ethyl acetate (3 × 60 mL). The combined organic layers were dried over $MgSO_4$, filtered and concentrated *in vacuo*. The resulting crude mixture was dissolved in methanol and water (30 mL 9:1 by volume, 0.1 M), and oxalic acid (6 mmol, 540.2 mg, 2 equiv) was added as a solid. After stirring for 12h, the mixture was concentrated *in vacuo*, diluted with ethyl acetate (60 mL) and washed with a saturated aqueous solution of sodium bicarbonate (60 mL). The aqueous layer was extracted with ethyl acetate (3 × 60 mL), and the combined organic layers were dried over $MgSO_4$, filtered and concentrated *in vacuo*. The crude product was then purified on silica gel using hexanes/ethyl acetate as eluent to afford the phenol.

General procedure F: *procedure for the Suzuki coupling.* The following procedure is based on the procedure of Liu.[9] A 10 mL round-bottom flask equipped with a Teflon-coated stir bar and a rubber septum was charged with $Pd(OAc)_2$ (1.1 mg, 0.005 mmol, 0.0025 equiv), compound **S68** (668.4 mg, 2 mmol, 1 equiv), the arylboronic acid (3 mmol, 1.5 equiv) and then purged with a steady stream of N_2 for 5 min, prior to the addition of degassed H_2O (4 mL). Diisopropylamine (4 mmol, 560.6 μL, 2 equiv) was then added with a syringe. The mixture was then warmed to 100 °C and stirred for 12h under N_2, quenched with HCl (2 M, 20 mL) and extracted with ethyl acetate (3 × 20 mL). The combined organic layers were dried over $MgSO_4$, filtered and concentrated *in vacuo*. The crude product was purified on silica gel to afford the corresponding 2,4-disubstituted phenol substrate.

b) Synthesis and Characterization of Precursors

i. Alkyl Halides

<u>Compound S3</u>:

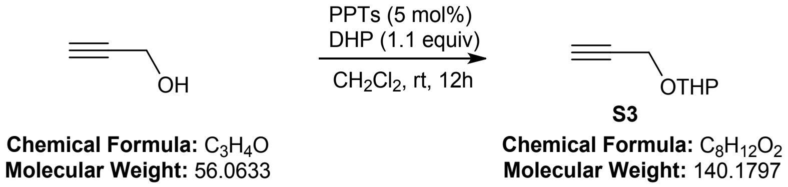

Procedure: A flame-dried, 250 mL round-bottom flask equipped with a Teflon-coated stir bar and a rubber septum was charged with propargyl alcohol (50 mmol, 2.89 mL, 1.0 equiv) and then purged with a steady stream of N_2 for 5 min, prior to the addition of dry and degassed CH_2Cl_2 (100 mL). Pyridinium *p*-toluenesulfonate (PPTs) (2.5 mmol, 628.2 mg, 0.05 equiv) and 3,4-dihydro-2*H*-pyran (DHP) (55 mmol, 5.02 mL, 1.1 equiv) was then added, and the mixture was stirred at room temperature for 12h, before quenching with water (100 mL). The aqueous layer was washed with CH_2Cl_2 (2 × 100 mL). The combined organic layers were dried over $MgSO_4$, filtered and concentrated *in vacuo.* The crude product was purified on silica gel using 10% ethyl acetate in hexanes as eluent to afford **S3** (6.66 g, 47.5 mmol, 95% yield) as a colorless oil.

Characterization:

^{1}H NMR (300 MHz, $CDCl_3$) δ 4.81 (t, *J* = 3.1 Hz, 1H), 4.27 (d, *J* = 2.4 Hz, 1H), 4.24 (d, *J* = 2.4 Hz, 1H), 3.89 – 3.78 (m, 1H), 3.59 – 3.47 (m, 1H), 2.40 (t, *J* = 2.4 Hz, 1H), 1.89 – 1.68 (m, 2H), 1.68 – 1.47 (m, 3H). The characterization data match the

[9] Liu, C.; Zhang, Y.; Liu, N.; Qiu, J. *Green Chem.* **2012**, *14*, 2999–3003.

previous report.[10]

Compound S4:

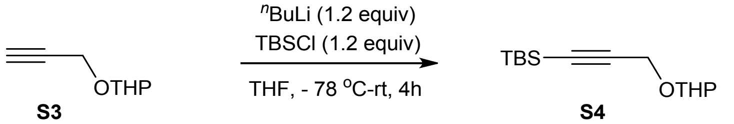

S3

Chemical Formula: $C_8H_{12}O_2$
Molecular Weight: 140.1797

S4

Chemical Formula: $C_{14}H_{26}O_2Si$
Molecular Weight: 254.4405

Procedure: A flame-dried, 250 mL round-bottom flask equipped with a Teflon-coated stir bar and a rubber septum was charged with **S3** (40 mmol, 5.61 g, 1.0 equiv) and then purged with a steady stream of N_2 for 5 min, prior to the addition of dry and degassed THF (50 mL). ^{n}BuLi (48 mmol, 19.2 mL, 2.5 M solution in hexane, 1.2 equiv) was then added dropwise at -78 °C. The resulting solution was stirred at this temperature for 20 min. In a separate, flame-dried, 50 mL round-bottom flask equipped with a Teflon-coated stir bar and a rubber septum was charged with *tert*-butyldimethylsilyl chloride (TBSCl) (48 mmol, 7.23 g, 1.2 equiv) and then purged with a steady stream of N_2 for 5 min, prior to the addition of dry and degassed THF (20 mL) to afford a homogeneous solution. It was then added to the solution of **S3** in THF via syringe dropwise at -78 °C. The mixture was stirred at -78 °C for 2h, and room temperature for another 2h, before quenching with saturated NH_4Cl solution (50 mL). The mixture was extracted with EtOAc (3 × 100 mL), and combined organic layers were dried over $MgSO_4$. It was then filtered and concentrated *in vacuo.* The crude product was purified on silica gel using 5% ethyl acetate in hexanes as eluent to afford **S4** (6.47 g, 25.4 mmol, 64% yield) as a colorless oil.

Characterization:

^{1}H NMR (400 MHz, $CDCl_3$) δ 4.85 (t, *J* = 3.4 Hz, 1H), 4.28 (s, 1H), 4.27 (s, 1H), 3.89 – 3.80 (m, 1H), 3.57 – 3.48 (m, 1H), 1.78 – 1.68 (m, 1H), 1.67 – 1.58 (m, 2H), 1.57 – 1.48 (m, 3H), 0.93 (s, 9H), 0.11 (s, 6H). The characterization data match the previous report.[6]

Compound S5:

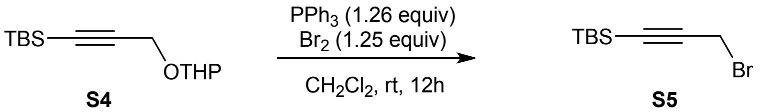

S4

Chemical Formula: $C_{14}H_{26}O_2Si$
Molecular Weight: 254.4405

S5

Chemical Formula: $C_9H_{17}BrSi$
Molecular Weight: 233.2208

Procedure: A flame-dried, 100 mL round-bottom flask equipped with a Teflon-coated stir bar and a rubber septum was charged with PPh_3 (12.6 mmol, 3.30 g, 1.26 equiv) and then purged with a steady stream of N_2 for 5 min, prior to the addition of dry and degassed CH_2Cl_2 (30 mL). Br_2 (12.5 mmol, 1.98 g, 1.25 equiv) was then added dropwise at room temperature. In a separate, flame-dried, 50 mL round-bottom flask equipped with a Teflon-coated stir bar and a rubber septum was charged with compound **S4** (10 mmol, 2.54 g, 1.0 equiv) and then purged with a steady stream of N_2 for 5 min, prior to the addition of dry and degassed CH_2Cl_2 (20 mL) to afford a homogeneous solution. It was then added to the solution of PPh_3 and Br_2 in CH_2Cl_2 via syringe, and the resulting mixture was stirred at room temperature for 12h. It was quenched with water (50 mL), and extracted with CH_2Cl_2 (3 × 50 mL). The combined organic layers were dried over $MgSO_4$, filtered and concentrated *in vacuo.* The crude product was purified on silica gel using hexanes as eluent to afford **S5** (1.81 g, 7.8 mmol, 78% yield) as a colorless oil.

Characterization:

^{1}H NMR (400 MHz, $CDCl_3$) δ 3.92 (s, 2H), 0.94 (s, 9H), 0.11 (s, 6H). The characterization data match the previous report.[11]

---

[10] Allegretti, P. A.; Ferreira, E. M. *Org. Lett.* **2011**, *13*, 5924-5927.
[11] Schelper, M.; Meijere, A. *Eur. J. Org. Chem.* **2005**, 582-592.

Compound S6:

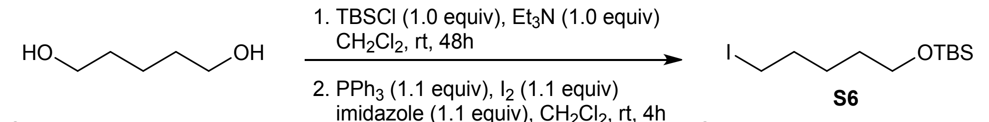

Chemical Formula: $C_5H_{12}O_2$
Molecular Weight: 104.1476

Chemical Formula: $C_{11}H_{25}IOSi$
Molecular Weight: 328.3056

Procedure: A flame-dried, 500 mL round-bottom flask equipped with a Teflon-coated stir bar and a rubber septum was charged with 1,5-pentanediol (100 mmol, 10.42 g, 1.0 equiv) and then purged with a steady stream of N_2 for 5 min, prior to the addition of dry and degassed Et_3N (100 mmol, 13.94 mL, 1.0 equiv) and CH_2Cl_2 (250 mL). In a separate, flame-dried, 100 mL round-bottom flask equipped with a Teflon-coated stir bar and a rubber septum was charged with *tert*-butyldimethylsilyl chloride (TBSCl) (100 mmol, 15.07 g, 1.0 equiv) and then purged with a steady stream of N_2 for 5 min, prior to the addition of dry and degassed CH_2Cl_2 (50 mL) to afford a homogeneous solution. It was then added to the solution of 1.5-pentanediol in CH_2Cl_2 via syringe and the resulting solution was stirred at room temperature for 48h before pouring on water (200 mL). Phases were separated and the aqueous layer was extracted with CH_2Cl_2 (3 × 200 mL). The combined organic layers were dried over $MgSO_4$, filtered and concentrated *in vacuo.* The crude product was directly moved for the next step. A flame-dried, 500 mL round-bottom flask equipped with a Teflon-coated stir bar and a rubber septum was charged with the crude product and then purged with a steady stream of N_2 for 5 min, prior to the addition of dry and degassed CH_2Cl_2 (300 mL). Imidazole (110 mmol, 7.49 g, 1.1 equiv), PPh_3 (110 mmol, 28.85 g, 1.1 equiv) and I_2 (110 mmol, 27.92 g, 1.1 equiv) was then added as solid portion-wise at room temperature. The resulting mixture was stirred at room temperature for 4h, before pouring on hexane (500 mL). The mixture was filtered and concentrated *in vacuo.* The crude product was purified on silica gel using hexanes eluent to afford **S6** (13.16 g, 40.1 mmol, 40% yield) as a colorless oil.

Characterization:

^{1}H NMR (400 MHz, $CDCl_3$) δ 3.60 (t, *J* = 6.2 Hz, 2H), 3.19 (t, *J* = 7.0 Hz, 2H), 1.89 – 1.76 (m, 2H), 1.57 – 1.49 (m, 2H), 1.48 – 1.40 (m, 2H), 0.89 (s, 9H), 0.04 (s, 6H). **^{13}C NMR** (75 MHz, $CDCl_3$) δ 62.8, 33.3, 31.7, 26.9, 26.0, 18.3, 7.0, -5.3. The characterization data matches the literature data.[12]

ii. Esters

Compound S7:

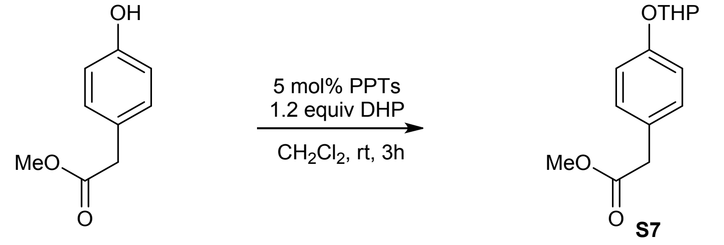

Chemical Formula: $C_9H_{10}O_3$
Molecular Weight: 166.1739

Chemical Formula: $C_{14}H_{18}O_4$
Molecular Weight: 250.2903

Procedure: A flame-dried, 500 mL round-bottom flask equipped with a Teflon-coated stir bar and a rubber septum was charged with pyridinium *p*-toluenesulfonate (PPTs) (25 mmol, 6.28 g, 5 mol%) and methyl 2-(4-hydroxyphenyl)acetate (500 mmol, 83.08 g, 1.0 equiv) and then purged with a steady stream of N_2 for 5 min, prior to the addition of dry and degassed CH_2Cl_2 (250 mL). 3,4-Dihydro-2H-pyran (DHP) (600 mmol, 54.74 mL, 1.2 equiv) was then added via syringe. The mixture was stirred at room temperature for 3h, and then quenched with water (100 mL). The aqueous layer was washed with CH_2Cl_2 (3 × 200 mL), and the combined organic layers were dried over $MgSO_4$, filtered and concentrated *in vacuo*. The crude product was purified on silica gel using 10% ethyl acetate in hexanes as eluent to afford **S7** (124.10 g, 495.8 mmol, 99% yield) as a colorless oil.

---

[12] Ellwood, A. R.; Porter, M. J. *J. Org. Chem.* **2009**, *74*, 7982-7985.

Characterization:

R$_f$ = (EtOAc/Hexane 1:9): 0.30; **IR** (neat) ν =2943, 1613, 1509, 1453, 1355, 1231, 1109, 1036, 919 cm^{-1}; **^{1}H NMR** (300 MHz, Acetone-d_6) δ 7.20 (d, J = 8.6 Hz, 2H), 7.00 (d, J = 8.6 Hz, 2H), 5.42 (t, J = 3.3 Hz, 1H), 3.84 (ddd, J = 12.0, 9.2, 3.2 Hz, 1H), 3.63 (s, 3H), 3.57 (s, 2H), 3.55 (ddd, J = 12.0, 9.2, 3.2 Hz, 1H), 2.03 – 1.90 (m, 1H), 1.90 – 1.74 (m, 2H), 1.73 – 1.49 (m, 3H); **^{13}C NMR** (75 MHz, Acetone-d6) δ 171.6, 156.2, 130.1, 127.5, 116.4, 96.2, 61.5, 51.1, 39.6, 30.2, 25.1, 18.7; **HRMS**: Calcd. for $C_{14}H_{18}O_4$ $[M+Na]^+$ = 273.1097 m/z, found = 273.1106 m/z. This compound was reported previously.[13] However, we were not able to find the characterization data.

Compound S8:

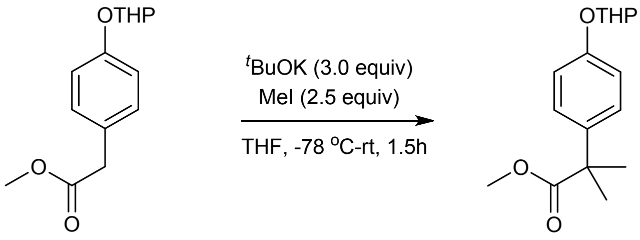

S7
Chemical Formula: $C_{14}H_{18}O_4$
Molecular Weight: 250.2903

S8
Chemical Formula: $C_{16}H_{22}O_4$
Molecular Weight: 278.3435

Procedure: A flame-dried, 500 mL round-bottom flask equipped with a Teflon-coated stir bar and a rubber septum was charged with potassium *tert*-butoxide (^{t}BuOK) (150 mmol, 16.83 g, 3.0 equiv) and then purged with a steady stream of N_2 for 5 min, prior to the addition of dry and degassed THF (200 mL). In a separate, flame-dried, 100 mL round-bottom flask equipped with a Teflon-coated stir bar and a rubber septum was charged with compound **S7** (50 mmol, 12.51 g, 1.0 equiv) and then purged with a steady stream of N_2 for 5 min, prior to the addition of dry and degassed THF (50 mL) to afford a homogeneous solution. It was added to the ^{t}BuOK solution in THF via syringe at -78 °C. After stirring for 20 min at -78 °C, iodomethane (125 mmol, 7.78 mL, 2.5 equiv) was added via syringe dropwise to the solution, and the resulting mixture was stirred at -78 °C for 1h and then warmed to room temperature and stirred for another 30 min before quenching with water (100 mL). It was extracted with ethyl acetate (3 × 100 mL), and the combined organic layers were dried over $MgSO_4$, filtered and concentrated *in vacuo*. The crude product was purified on silica gel using 5% ethyl acetate in hexanes as eluent to afford **S8** (12.03 g, 43.2 mmol, 86% yield) as a pale-yellow oil.

Characterization:

R$_f$ = (EtOAc/Hexane 1:9): 0.40; **IR** (neat) ν = 3376, 2068, 3013, 2974, 2925, 2848, 1600, 1436, 1399, 1215, 1121, 1016, 937, 808, 647, 468 cm^{-1}; **^{1}H NMR** (300 MHz, Acetone-d_6) δ 7.25 (d, J = 8.8 Hz, 2H), 6.99 (d, J = 8.8 Hz, 2H), 5.42 (t, J = 3.3 Hz, 1H), 3.84 (ddd, J = 11.3, 9.2, 3.4 Hz, 1H), 3.60 (s, 3H) , 3.54 (ddd, J = 11.3, 9.2, 3.4 Hz, 1H), 2.02 – 1.89 (m, 1H), 1.89 – 1.74 (m, 2H), 1.72 – 1.55 (m, 3H), 1.52 (s, 6H); **^{13}C NMR** (75 MHz, Acetone-d_6) δ 176.4, 155.9, 137.8, 126.5, 116.2, 96.1, 61.5, 51.3, 45.6, 30.2, 26.15, 16.12, 25.1, 18.7; **HRMS**: Calcd. for $C_{16}H_{22}O_4$ $[M+Na]^+$ = 301.1410 m/z, found = 301.1407 m/z.

Compound S9:

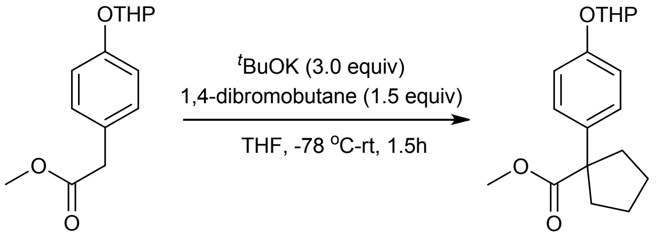

S7
Chemical Formula: $C_{14}H_{18}O_4$
Molecular Weight: 250.2903

S9
Chemical Formula: $C_{18}H_{24}O_4$
Molecular Weight: 304.3808

Procedure: A flame-dried, 50 mL round-bottom flask equipped with a Teflon-coated stir bar and a rubber septum was

[13] Ando, K.; Suzuki, Y. *Tetrahedron Lett.* **2010**, *51*, 2323-2325.

charged with potassium *tert*-butoxide (^tBuOK) (15 mmol, 1.68 g, 3.0 equiv) and then purged with a steady stream of N_2 for 5 min, prior to the addition of dry and degassed THF (20 mL). In a separate, flame-dried, 100 mL round-bottom flask equipped with a Teflon-coated stir bar and a rubber septum was charged with compound **S7** (5 mmol, 1.25 g, 1.0 equiv) and then purged with a steady stream of N_2 for 5 min, prior to the addition of dry and degassed THF (5 mL) to afford a homogeneous solution. It was added to the ^tBuOK solution in THF via syringe at -78 °C. After stirring for 20 min at -78 °C, 1,4-dibromobutane (7.5 mmol, 895.6 μL, 1.5 equiv) was added via syringe dropwise to the solution, and the resulting mixture was stirred at -78 °C for 1h and then warmed to room temperature and stirred for another 30 min before quenching with water (25 mL). It was extracted with ethyl acetate (3 × 25 mL), and the combined organic layers were dried over $MgSO_4$, filtered and concentrated *in vacuo*. The crude product was purified on silica gel using 5% ethyl acetate in hexanes as eluent to afford **S9** (872.2 mg, 2.87 mmol, 57% yield) as a white solid.

Characterization:

R_f = (EtOAc/Hexane 1:10): 0.52; **IR** (neat) ν = 2977, 2948, 2850, 1724, 1608, 1507, 1446, 1232, 1181, 1157, 1110, 1075, 1038, 955, 919, 873, 837, 818, 755, 616, 531 cm^{-1}; **^{1}H NMR** (400 MHz, Acetone-d_6) δ 7.27 (d, *J* = 8.8 Hz, 2H), 6.98 (d, *J* = 8.8 Hz, 2H), 5.41 (t, *J* = 3.2 Hz, 1H), 3.88 – 3.77 (m, 1H), 3.59 – 3.51 (m, 4H), 2.67 – 2.51 (m, 2H), 2.00 – 1.90 (m, 1H), 1.90 – 1.76 (m, 4H), 1.76 – 1.60 (m, 6H), 1.60 – 1.52 (m, 1H); **^{13}C NMR** (75 MHz, Acetone-d_6) δ 175.7, 156.0, 136.3, 127.6, 116.1, 96.1, 61.5, 58.3, 51.5, 36.03, 36.00, 30.2, 25.1, 23.2, 18.7; **HRMS**: Calcd. for $C_{18}H_{24}O_4$ $[M+Na]^+$ = 327.1567 m/z, found = 327.1565 m/z.

Compound S10:

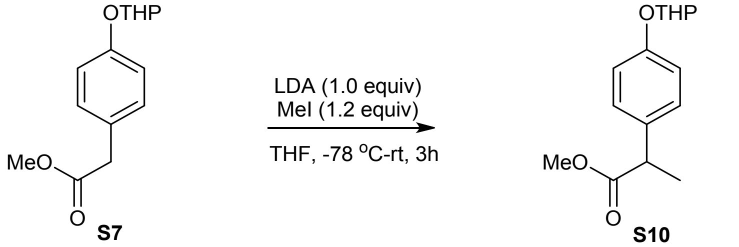

S7
Chemical Formula: $C_{14}H_{18}O_4$
Molecular Weight: 250.2903

S10
Chemical Formula: $C_{15}H_{20}O_4$
Molecular Weight: 264.3169

Procedure: A flame-dried, 25 mL Radley tube equipped with a Teflon-coated stir bar and a rubber septum was charged with compound **S7** (30 mmol, 7.51 g, 1.0 equiv) and then purged with a steady stream of N_2 for 5 min, prior to the addition of dry and degassed THF (60 mL). A solution of lithium diisopropylamide (LDA) (2.0 M, 15 mL, 30 mmol, 1.0 equiv) was then added dropwise at -78 °C. The resulting mixture was stirred at -78 °C for 15 min, at which time iodomethane (36 mmol, 2.24 mL, 1.2 equiv) was added dropwise to the solution. The reaction was stirred at -78 °C for 2h, and then warmed to room temperature and stirred for another 1h. The mixture was diluted with water (100 mL) and extracted with ethyl acetate (3 × 100 mL). The combined organic layers were dried over $MgSO_4$, filtered and concentrated *in vacuo*. The crude product was then purified on silica gel using 5% ethyl acetate in hexanes as eluent to afford **S10** (7.14 g, 27.0 mmol, 90% yield) as a pale-yellow oil.

Characterization:

R_f = (ethyl acetate/hexane 1:5): 0.53; **IR** (neat) ν = 2944, 2874, 1734, 1610, 1509, 1434, 1234, 1202, 1162, 1109, 1036, 1020, 961, 919, 834 cm^{-1}; **^{1}H NMR** (400 MHz, acetone-d_6) δ 7.21 (d, *J* = 8.7 Hz, 2H), 7.00 (d, *J* = 8.7 Hz, 2H), 5.42 (t, *J* = 3.2 Hz, 1H), 3.87 – 3.79 (m, 1H), 3.71 (q, *J* = 7.1 Hz, 1H), 3.60 (s, 3H), 3.59 – 3.52 (m, 1H), 2.01 – 1.90 (m, 1H), 1.88 – 1.73 (m, 2H), 1.70 – 1.59 (m, 2H), 1.59 – 1.50 (m, 1H), 1.41 (d, *J* = 7.1 Hz, 3H); **^{13}C NMR** (75 MHz, acetone-d_6) δ 174.4, 156.3, 133.9, 128.2, 116.5, 96.1, 61.5, 51.1, 44.2, 30.2, 25.1, 18.7, 18.3; **HRMS**: Calcd. for $C_{15}H_{20}O_4$ $[M+Na]^+$ = 287.1254 m/z, found = 287.1248 m/z.

Compound S11:

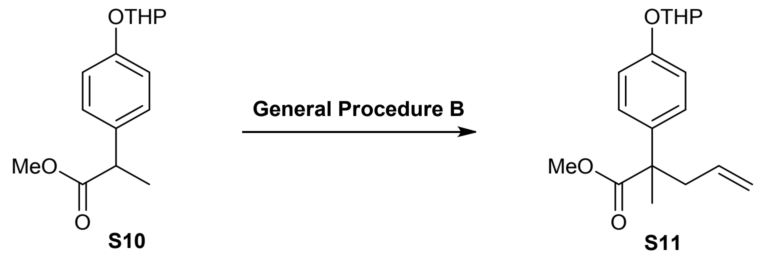

S10
Chemical Formula: $C_{15}H_{20}O_4$
Molecular Weight: 264.3169

S11
Chemical Formula: $C_{18}H_{24}O_4$
Molecular Weight: 304.3808

Procedure: The reaction was carried out according to the General Procedure B.

Amounts of Reagents:

S10 (5 mmol, 1.32 g)

LDA (6 mmol, 3 mL 2.0 M solution)

allyl bromide (7.5 mmol, 649.1 μL)

THF (10 mL, 0.5 M)

Purification: 5% EtOAc in hexanes.

Yield of Product:

S11: 1.41 g, 4.63 mmol, 93% yield, colorless oil.

Characterization:

R_f = (ethyl acetate/hexane 1:10): 0.40; **IR** (neat) ν = 2944, 2874, 1728, 1609, 1510, 1455, 1434, 1238, 1202, 1180, 1109, 1036, 963, 919, 830 cm^{-1}; **^{1}H NMR** (400 MHz, acetone-d_6) δ 7.25 (d, *J* = 8.8 Hz, 2H), 7.01 (d, *J* = 8.8 Hz, 2H), 5.63 (ddt, *J* = 17.3, 10.1, 7.2 Hz, 1H), 5.43 (t, *J* = 3.3 Hz, 1H), 5.08 (ddd, *J* = 17.1, 1.5, 0.6 Hz, 1H), 5.03 (dd, *J* = 10.2, 1.9 Hz, 1H), 3.83 (ddd, *J* = 11.5, 9.4, 3.2 Hz, 1H), 3.61 (s, 3H), 3.59 – 3.53 (m, 1H), 2.80 (dd, *J* = 13.7, 7.1 Hz, 1H), 2.63 (dd, *J* = 13.5, 7.3 Hz, 1H), 2.01 – 1.89 (m, 1H), 1.89 – 1.73 (m, 2H), 1.70 – 1.60 (m, 2H), 1.61 – 1.51 (m, 1H), 1.48 (s, 3H); **^{13}C NMR** (75 MHz, acetone-d_6) δ175.5, 156.0, 136.48, 136.47, 134.5, 126.8, 117.7, 116.2, 96.1, 61.5, 51.3, 49.1, 43.7, 43.6, 30.2, 25.1, 22.30, 22.27, 18.7 (***a complex mixture of diastereomers are observed in the ^{13}C NMR, which accounts for the presence of extra ^{13}C signals***); **HRMS**: Calcd. for $C_{18}H_{24}O_4$ $[M+Na]^+$ = 327.1567 m/z, found = 327.1560 m/z.

Compound S12:

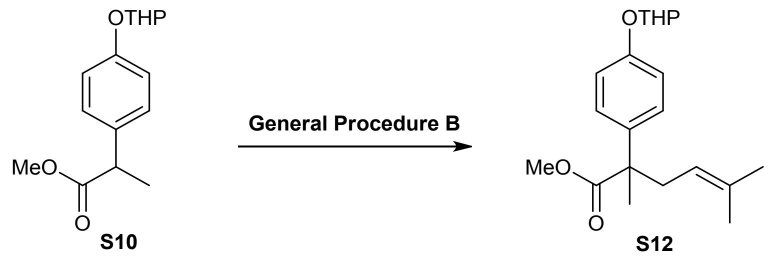

S10
Chemical Formula: $C_{15}H_{20}O_4$
Molecular Weight: 264.3169

S12
Chemical Formula: $C_{20}H_{28}O_4$
Molecular Weight: 332.4339

Procedure: The reaction was carried out according to the General Procedure B.

Amounts of Reagents:

S10 (5 mmol, 1.32 g)

LDA (6 mmol, 3 mL 2.0 M solution)

prenyl bromide (7.5 mmol, 866.4 μL)

THF (10 mL, 0.5 M)

Purification: 5% EtOAc in hexanes.

Yield of Product:

S12: 534.5 mg, 1.61 mmol, 32% yield, pale-yellow oil.

Characterization:

R$_f$ = (ethyl acetate/hexane 1:10): 0.36; **IR** (neat) v = 2944, 2875, 1728, 1609, 1509, 1454, 1434, 1376, 1356, 1237, 1202, 1179, 1109, 1037, 1020, 964, 920, 872, 829 cm^{-1}; **^{1}H NMR** (400 MHz, acetone-*d$_6$*) δ 7.26 (d, *J* = 8.9 Hz, 2H), 7.01 (d, *J* = 8.9 Hz, 2H), 5.44 (t, *J* = 3.3 Hz, 1H), 5.03 (dddd, *J* = 7.4, 6.0, 2.8, 1.4 Hz, 1H), 3.91 – 3.80 (m, 1H), 3.62 (s, 3H), 3.61 – 3.54 (m, 1H), 2.76 (dd, *J* = 14.7, 7.8 Hz, 1H), 2.59 (dd, *J* = 14.1, 7.4 Hz, 1H), 2.03 – 1.90 (m, 1H), 1.91 – 1.75 (m, 2H), 1.73 – 1.63 (m, 5H), 1.62 – 1.55 (m, 4H), 1.47 (s, 3H); **^{13}C NMR** (75 MHz, acetone-*d$_6$*) δ 175.8, 155.9, 136.9, 134.0, 126.9, 120.0, 116.1, 96.1, 61.5, 51.2, 49.7, 37.6, 37.5, 30.2, 25.2, 25.1, 22.40, 22.36, 18.7, 17.2 (***a complex mixture of diastereomers are observed in the ^{13}C NMR, which accounts for the presence of extra ^{13}C signals***); **HRMS**: Calcd. for $C_{20}H_{28}O_4$ $[M+Na]^+$ = 355.1880 m/z, found = 355.1871 m/z.

Compound S13:

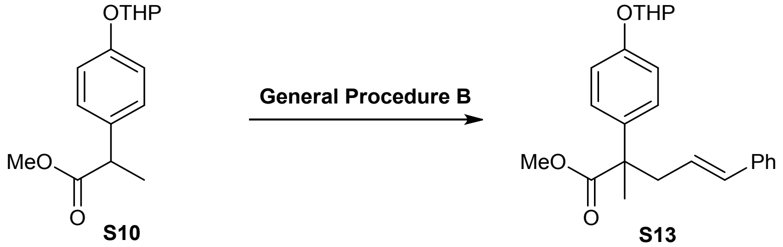

S10
Chemical Formula: $C_{15}H_{20}O_4$
Molecular Weight: 264.3169

S13
Chemical Formula: $C_{24}H_{28}O_4$
Molecular Weight: 380.4767

Procedure: The reaction was carried out according to the General Procedure B.

Amounts of Reagents:

S10 (5 mmol, 1.32 g)

LDA (6 mmol, 3 mL 2.0 M solution)

cinnamyl bromide (7.5 mmol, 1.48 g)

THF (10 mL, 0.5 M)

Purification: 5% EtOAc in hexanes.

Yield of Product:

S13: 1.62 g, 4.26 mmol, 85% yield, colorless oil.

Characterization:

R$_f$ = (ethyl acetate/hexane 1:10): 0.25; **IR** (neat) v = 3027, 2944, 2873, 1727, 1608, 1509, 1452, 1238, 1201, 1179, 1109, 1036, 1021, 962, 920, 872, 829, 739, 693 cm^{-1}; **^{1}H NMR** (400 MHz, acetone-*d$_6$*) δ 7.38 – 7.26 (m, 6H), 7.21 (t, *J* = 7.2 Hz, 1H), 7.09 – 7.02 (m, 2H), 6.50 (d, *J* = 15.8 Hz, 1H), 6.21 – 6.07 (m, 1H), 5.44 (t, *J* = 3.3 Hz, 1H), 3.91 – 3.82 (m, 1H), 3.66 (s, 3H), 3.61 – 3.55 (m, 1H), 2.99 (ddd, *J* = 13.7, 7.3, 1.0 Hz, 1H), 2.81 (dd, *J* = 13.7, 7.4 Hz, 1H), 2.04 – 1.92 (m, 1H), 1.91 – 1.75 (m, 2H), 1.72 – 1.62 (m, 2H), 1.62 – 1.55 (m, 4H); **^{13}C NMR** (75 MHz, acetone-*d$_6$*) δ175.6, 156.0, 137.5, 136.6, 133.3, 128.5, 127.1, 126.9, 126.03, 125.98, 116.3, 96.2, 61.6, 51.4, 49.7, 43.0, 42.9, 30.2, 25.1, 22.52, 22.50, 18.8 (***a complex mixture of diastereomers are observed in the ^{13}C NMR, which accounts for the presence of extra ^{13}C signals***); **HRMS**: Calcd. for $C_{24}H_{28}O_4$ $[M+H]^+$ = 381.2060 m/z, found = 381.2055 m/z.

Compound S14:

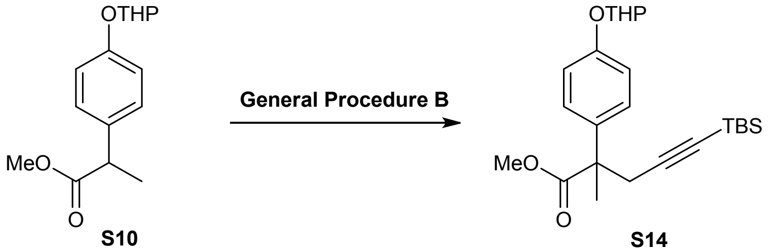

S10
Chemical Formula: $C_{15}H_{20}O_4$
Molecular Weight: 264.3169

S14
Chemical Formula: $C_{24}H_{36}O_4Si$
Molecular Weight: 416.6257

Procedure: The reaction was carried out according to the General Procedure B.

Amounts of Reagents:

S10 (5 mmol, 1.32 g)

LDA (6 mmol, 3 mL 2.0 M solution)

S5 (7.5 mmol, 1.75 g)

THF (10 mL, 0.5 M)

Purification: 5% EtOAc in hexanes.

Yield of Product:

S14: 1.71 g, 4.10 mmol, 82% yield, pale-yellow oil.

Characterization:

R$_f$ = (ethyl acetate/hexane 1:10): 0.28; **IR** (neat) v = 2949, 2855, 2175, 1732, 1610, 1511, 1462, 1241, 1202, 1180, 1109, 1036, 1022, 965, 921, 824, 810, 773, 680 cm^{-1}; **^{1}H NMR** (400 MHz, acetone-d_6) δ 7.28 (d, J = 8.9 Hz, 2H), 7.02 (d, J = 8.9 Hz, 2H), 5.44 (t, J = 3.2 Hz, 1H), 3.85 (ddd, J = 11.3, 9.5, 3.1 Hz, 1H), 3.66 (s, 3H), 3.62 – 3.52 (m, 1H), 2.99 (d, J = 16.6 Hz, 1H), 2.83 (dd, J = 16.6, 1.2 Hz, 1H), 2.03 – 1.91 (m, 1H), 1.91 – 1.75 (m, 2H), 1.74 – 1.62 (m, 5H), 1.61 – 1.50 (m, 1H), 0.91 (s, 9H), 0.07 (d, J = 2.2 Hz, 6H); **^{13}C NMR** (75 MHz, acetone-d_6) δ 174.6, 156.2, 135.2, 126.8, 116.2, 104.7, 96.1, 84.6, 61.5, 51.6, 49.4, 30.54, 30.52, 30.2, 25.5, 25.1, 22.59, 22.56, 18.6, 16.1, -5.22, -5.24 (***a complex mixture of diastereomers are observed in the ^{13}C NMR, which accounts for the presence of extra ^{13}C signals***); **HRMS**: Calcd. for $C_{24}H_{36}O_4Si$ [M+Na]$^+$ = 439.2275 m/z, found = 439.2276 m/z.

Compound S15:

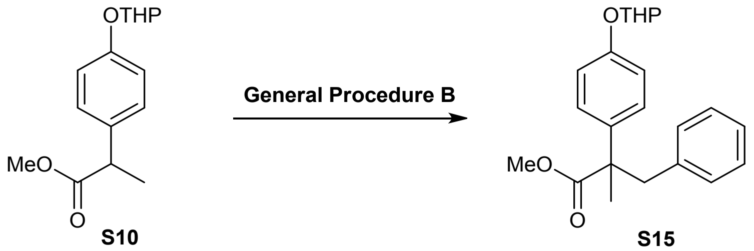

S10
Chemical Formula: $C_{15}H_{20}O_4$
Molecular Weight: 264.3169

S15
Chemical Formula: $C_{22}H_{26}O_4$
Molecular Weight: 354.4394

Procedure: The reaction was carried out according to the General Procedure B.

Amounts of Reagents:

S10 (5 mmol, 1.32 g)

LDA (6 mmol, 3 mL 2.0 M solution)

benzyl bromide (6.0 mmol, 713.6 μL)

THF (10 mL, 0.5 M)

Purification: 5% EtOAc in hexanes.

Yield of Product:

S15: 1.71 g, 4.82 mmol, 96% yield, pale-yellow oil.

Characterization:

R$_f$ = (ethyl acetate/hexane 1:10): 0.39; **IR** (neat) v = 3029, 2945, 2872, 1726, 1608, 1509, 1453, 1239, 1201, 1178, 1110, 1097, 1036, 1021, 962, 920, 872, 743, 701 cm^{-1}; **^{1}H NMR** (400 MHz, acetone-d_6) δ 7.26 (d, J = 8.8 Hz, 2H), 7.23 – 7.14 (m, 3H), 7.02 (d, J = 8.9 Hz, 4H), 5.44 (d, J = 2.3 Hz, 1H), 3.90 – 3.78 (m, 1H), 3.64 (s, 3H), 3.61 – 3.52 (m, 1H), 3.44 (d, J = 13.1 Hz, 1H), 3.15 (d, J = 13.2 Hz, 1H), 2.02 – 1.90 (m, 1H), 1.90 – 1.73 (m, 2H), 1.72 – 1.61 (m, 2H), 1.60 – 1.53 (m, 1H), 1.41 (s, 3H); **^{13}C NMR** (75 MHz, acetone-d_6) δ 175.6, 156.0, 137.8, 136.6, 130.4, 127.73, 127.10, 126.3, 116.1, 96.2, 61.6, 51.3, 50.5, 45.1, 45.0, 30.2, 25.1, 21.94, 21.92, 18.7 (***a complex mixture of diastereomers are observed in the ^{13}C NMR, which accounts for the presence of extra ^{13}C signals***); **HRMS**: Calcd. for $C_{22}H_{26}O_4$ [M+Na]$^+$ = 377.1723 m/z, found = 377.1717 m/z.

Compound S16:

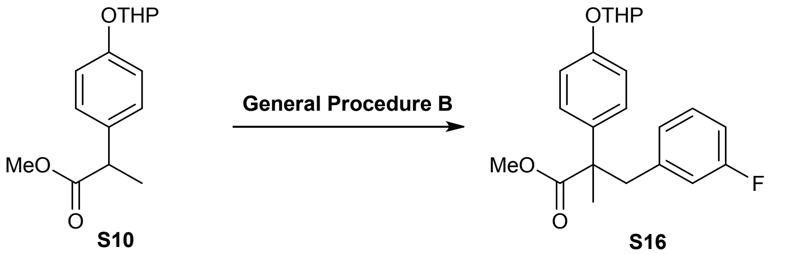

S10
Chemical Formula: $C_{15}H_{20}O_4$
Molecular Weight: 264.3169

S16
Chemical Formula: $C_{22}H_{25}FO_4$
Molecular Weight: 372.4299

Procedure: The reaction was carried out according to the General Procedure B.

Amounts of Reagents:

S10 (5 mmol, 1.32 g)

LDA (6 mmol, 3 mL 2.0 M solution)

3-fluorobenzyl bromide (7.5 mmol, 920.0 μL)

THF (10 mL, 0.5 M)

Purification: 5% EtOAc in hexanes.

Yield of Product:

S16: 1.78 g, 4.78 mmol, 96% yield, pale-yellow oil.

Characterization:

R*f* = (ethyl acetate/hexane 1:10): 0.22; **IR** (neat) ν = 2946, 2873, 1727, 1612, 1586, 1509, 1487, 1446, 1239, 1179, 1110, 1036, 961, 920, 872, 831, 778, 695 cm^{-1}; **^{1}H NMR** (400 MHz, acetone-d_6) δ 7.28 – 7.20 (m, 3H), 7.02 (d, *J* = 8.9 Hz, 2H), 6.95 (td, *J* = 8.4, 2.4 Hz, 1H), 6.86 (d, *J* = 7.7 Hz, 1H), 6.74 (d, *J* = 10.5 Hz, 1H), 5.45 (dd, *J* = 5.0, 3.0 Hz, 1H), 3.89 – 3.79 (m, 1H), 3.65 (s, 3H), 3.61 – 3.52 (m, 1H), 3.43 (d, *J* = 13.2 Hz, 1H), 3.19 (d, *J* = 13.2 Hz, 1H), 2.03 – 1.90 (m, 1H), 1.90 – 1.73 (m, 2H), 1.71 – 1.61 (m, 2H), 1.61 – 1.52 (m, 1H), 1.43 (s, 3H); **^{13}C NMR** (75 MHz, acetone-d_6) δ 175.5, 162.3 (d, J_F = 243.1 Hz), 156.1, 140.6 (d, J_F = 7.5 Hz), 136.2, 129.4 (d, J_F = 8.4 Hz), 127.1, 126.5 (d, J_F = 2.7 Hz), 117.0 (d, J_F = 21.3 Hz), 116.2, 113.0 (d, J_F = 21.1 Hz), 96.1, 61.5, 51.4, 50.4, 44.7, 30.2, 25.1, 21.7, 18.7; **HRMS**: Calcd. for $C_{22}H_{25}FO_4$ $[M+Na]^+$ = 395.1629 m/z, found = 395.1622 m/z.

Compound S17:

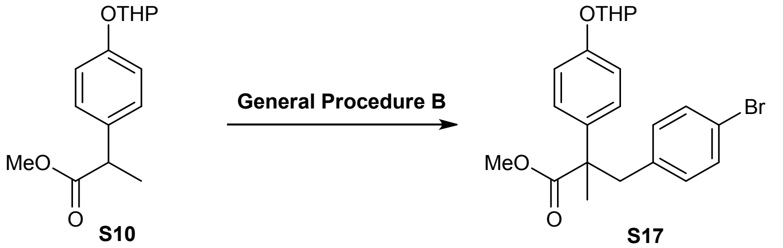

S10
Chemical Formula: $C_{15}H_{20}O_4$
Molecular Weight: 264.3169

S17
Chemical Formula: $C_{22}H_{25}BrO_4$
Molecular Weight: 433.3355

Procedure: The reaction was carried out according to the General Procedure B.

Amounts of Reagents:

S10 (5 mmol, 1.32 g)

LDA (6 mmol, 3 mL 2.0 M solution)

4-bromobenzyl bromide (7.5 mmol, 1.87 g)

THF (10 mL, 0.5 M)

Purification: 5% EtOAc in hexanes.

Yield of Product:

S17: 1.60 g, 3.69 mmol, 74% yield, pale-yellow oil.

Characterization:

R$_f$ = (ethyl acetate/hexane 1:10): 0.21; **IR** (neat) v = 2946, 2871, 1727, 1608, 1509, 1487, 1239, 1201, 1178, 1109, 1072, 1036, 1012, 962, 920, 832 cm^{-1}; **^{1}H NMR** (400 MHz, acetone-*d$_6$*) δ 7.38 (d, *J* = 8.4 Hz, 2H), 7.25 (d, *J* = 8.3 Hz, 2H), 7.03 (d, *J* = 8.9 Hz, 2H), 6.96 (dd, *J* = 8.4, 1.4 Hz, 2H), 5.45 (t, *J* = 3.2 Hz, 1H), 3.94 – 3.79 (m, 1H), 3.66 (s, 3H), 3.62 – 3.54 (m, 1H), 3.40 (d, *J* = 13.2 Hz, 1H), 3.16 (d, *J* = 13.2 Hz, 1H), 2.04 – 1.92 (m, 1H), 1.91 – 1.76 (m, 2H), 1.74 – 1.62 (m, 2H), 1.62 – 1.53 (m, 1H), 1.44 (s, 3H); **^{13}C NMR** (75 MHz, acetone-*d$_6$*) δ 175.5, 156.1, 137.1, 136.2, 132.5, 130.7, 127.1, 120.0, 116.2, 96.2, 61.6, 51.4, 50.4, 44.44, 44.42, 30.2, 25.1, 21.7, 18.7 (***a complex mixture of diastereomers are observed in the ^{13}C NMR, which accounts for the presence of extra ^{13}C signals***); **HRMS**: Calcd. for $C_{22}H_{25}BrO_4$ $[M+Na]^+$ = 455.0828 m/z, found = 455.0823 m/z.

Compound S18:

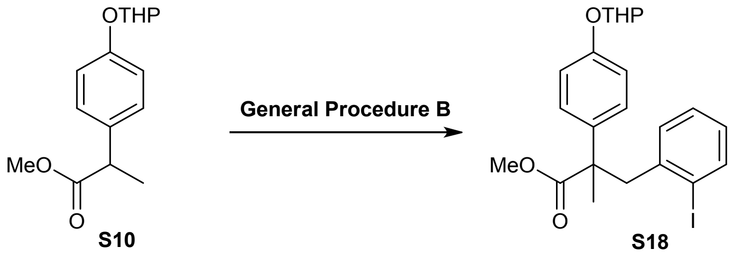

S10
Chemical Formula: $C_{15}H_{20}O_4$
Molecular Weight: 264.3169

S18
Chemical Formula: $C_{22}H_{25}IO_4$
Molecular Weight: 480.3360

Procedure: The reaction was carried out according to the General Procedure B.

Amounts of Reagents:

S10 (5 mmol, 1.32 g)

LDA (6 mmol, 3 mL 2.0 M solution)

2-iodobenzyl bromide (7.5 mmol, 2.23 g)

THF (10 mL, 0.5 M)

Purification: 5% EtOAc in hexanes.

Yield of Product:

S18: 1.42 g, 2.96 mmol, 59% yield, pale-yellow oil.

Characterization:

R$_f$ = (ethyl acetate/hexane 1:10): 0.26; **IR** (neat) v = 2946, 2870, 1727, 1608, 1509, 1456, 1433, 1239, 1201, 1179, 1122, 1110, 1037, 1010, 963, 920, 872, 832, 816, 746, 647 cm^{-1}; **^{1}H NMR** (400 MHz, acetone-*d$_6$*) δ 7.88 (dd, *J* = 7.9, 1.1 Hz, 1H), 7.28 (d, *J* = 8.9 Hz, 2H), 7.18 (t, *J* = 7.5 Hz, 1H), 7.06 (d, *J* = 8.9 Hz, 2H), 6.93 (td, *J* = 7.7, 1.6 Hz, 1H), 6.86 (d, *J* = 7.8 Hz, 1H), 5.46 (t, *J* = 3.2 Hz, 1H), 3.91 – 3.83 (m, 1H), 3.78 (d, *J* = 13.9 Hz, 1H), 3.70 (s, 3H), 3.63 – 3.54 (m, 1H), 3.45 (d, *J* = 13.9 Hz, 1H), 2.05 – 1.92 (m, 1H), 1.91 – 1.77 (m, 2H), 1.73 – 1.62 (m, 2H), 1.62 – 1.56 (m, 1H), 1.52 (s, 3H); **^{13}C NMR** (75 MHz, acetone-*d$_6$*) δ 175.7, 156.2, 141.0, 139.8, 136.2, 130.2, 128.5, 128.0, 127.3, 116.3, 103.5, 96.2, 61.6, 51.7, 51.3, 48.12, 48.10, 30.3, 25.1, 21.14, 21.13, 18.8 (***a complex mixture of diastereomers are observed in the ^{13}C NMR, which accounts for the presence of extra ^{13}C signals***); **HRMS**: Calcd. for $C_{22}H_{25}IO_4$ $[M+Na]^+$ = 503.0690 m/z, found = 503.0677 m/z.

Compound S19:

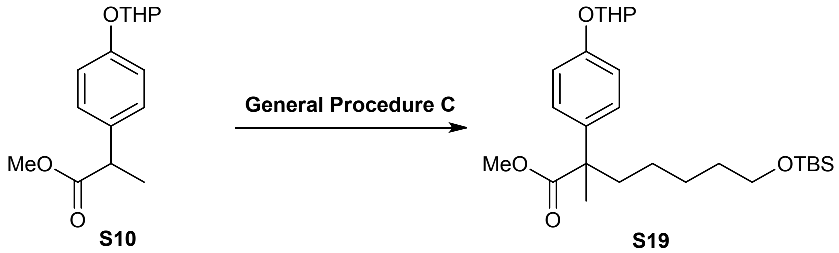

S10
Chemical Formula: $C_{15}H_{20}O_4$
Molecular Weight: 264.3169

S19
Chemical Formula: $C_{26}H_{44}O_5Si$
Molecular Weight: 464.7101

Procedure: The reaction was carried out according to the General Procedure C on 22.4 mmol scale.

Amounts of Reagents:

S10 (22.4 mmol, 5.92 g)

LDA (26.9 mmol, 13.4 mL 2.0 M solution)

S6 (31.2 mmol, 10.24 g)

THF (45 mL, 0.5 M)

Purification: 5% EtOAc in hexanes.

Yield of Product:

S19: 9.79 g, 21.07 mmol, 94% yield, yellow oil.

Characterization:

R_f = (ethyl acetate/hexane 1:10): 0.44; **IR** (neat) ν = 2930, 2856, 1730, 1609, 1510, 1441, 1240, 1202, 1180, 1106, 1038, 1021, 966, 921, 831, 773, 661 cm^{-1}; **^{1}H NMR** (400 MHz, acetone-*d_6*) δ 7.23 (d, *J* = 8.8 Hz, 2H), 7.00 (d, *J* = 8.9 Hz, 2H), 5.41 (t, *J* = 3.2 Hz, 1H), 3.88 – 3.79 (m, 1H), 3.61 (t, *J* = 6.2 Hz, 2H), 3.61 (s, 3H), 3.59 – 3.52 (m, 1H), 2.07 – 1.73 (m, 5H), 1.70 – 1.60 (m, 2H), 1.60 – 1.43 (m, 6H), 1.43 – 1.31 (m, 2H), 1.29 – 1.14 (m, 2H), 0.90 (s, 9H), 0.05 (s, 6H); **^{13}C NMR** (75 MHz, acetone-*d_6*) δ 176.0, 155.8, 137.2, 137.1, 126.8, 116.1, 96.1, 62.6, 61.5, 51.2, 49.4, 39.39, 39.35, 32.5, 30.3, 26.2, 25.5, 25.1, 24.4, 22.52, 22.49, 18.7, 18.0, -6.0 (***a complex mixture of diastereomers are observed in the ^{13}C NMR, which accounts for the presence of extra ^{13}C signals***); **HRMS**: Calcd. for $C_{26}H_{44}O_5Si$ $[M+Na]^+$ = 487.2850 m/z, found = 487.2849 m/z.

Compound S20:

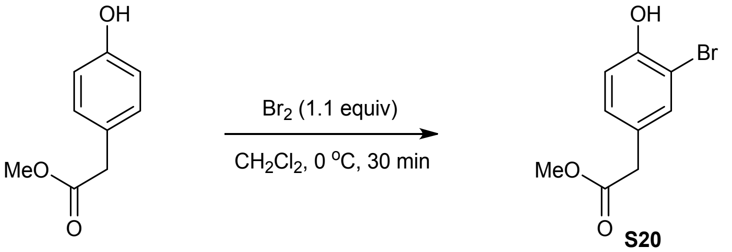

S20

Chemical Formula: $C_9H_{10}O_3$
Molecular Weight: 166.1739

Chemical Formula: $C_9H_9BrO_3$
Molecular Weight: 245.0700

Procedure: A flame-dried, 1 L round-bottom flask equipped with a Teflon-coated stir bar and a rubber septum was charged with 2-(4-hydroxyphenyl)acetate (100 mmol, 16.62 g, 1.0 equiv) and then purged with a steady stream of N_2 for 5 min, prior to the addition of dry and degassed CH_2Cl_2 (500 mL). The reaction was then cooled to 0 °C, Br_2 (110 mmol, 5.67 mL, 1.1 equiv) was then added dropwise via syringe. The mixture was stirred at this temperature for 30 min, and then quenched with saturated $NaHCO_3$ (200 mL). The aqueous layer was washed with CH_2Cl_2 (2 × 200 mL), and the combined organic layers were dried over $MgSO_4$, filtered and concentrated *in vacuo*. The crude product was purified on silica gel using 15% ethyl acetate in hexanes as eluent to afford **S20** (23.33 g, 95.2 mmol, 95% yield) as a white solid.

Characterization:

^{1}H NMR (500 MHz, $CDCl_3$) δ 7.42 (d, *J* = 2.1 Hz, 1H), 7.15 (dd, *J* = 8.3, 2.0 Hz, 1H), 6.98 (d, *J* = 8.3 Hz, 1H), 5.57 (br. s, 1H),

3.72 (s, 3H), 3.56 (s, 2H). The characterization data matches previous report.[14]

Compound S21:

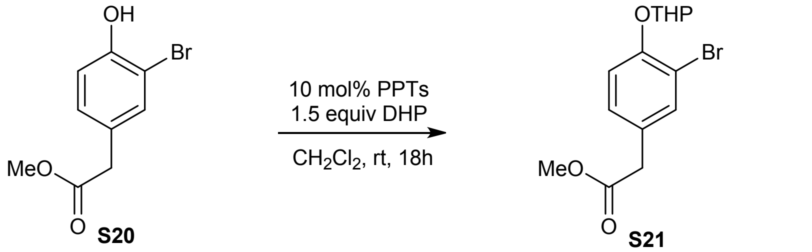

S20
Chemical Formula: $C_9H_9BrO_3$
Molecular Weight: 245.0700

S21
Chemical Formula: $C_{14}H_{17}BrO_4$
Molecular Weight: 329.1864

Procedure: A flame-dried, 500 mL round-bottom flask equipped with a Teflon-coated stir bar and a rubber septum was charged with pyridinium *p*-toluenesulfonate (PPTs) (10 mmol, 2.51 g, 10 mol%) and **S20** (95.2 mmol, 23.33 g, 1.0 equiv) and then purged with a steady stream of N_2 for 5 min, prior to the addition of dry and degassed CH_2Cl_2 (200 mL). 3,4-Dihydro-2H-pyran (DHP) (150 mmol, 13.69 mL, 1.5 equiv) was then added via syringe. The mixture was stirred at room temperature for 18 h, and then quenched with water (100 mL). The aqueous layer was washed with CH_2Cl_2 (2 × 200 mL), and the combined organic layers were dried over $MgSO_4$, filtered and concentrated *in vacuo*. The crude product was purified on silica gel using 10% ethyl acetate in hexanes as eluent to afford **S21** (25.22 g, 76.6 mmol, 80% yield) as a colorless oil.

Characterization:

^{1}H NMR (500 MHz, Acetone-d_6) δ 7.53 (d, *J* = 2.1 Hz, 1H), 7.24 (dd, *J* = 8.5, 2.1 Hz, 1H), 7.18 (d, *J* = 8.5 Hz, 1H), 5.59 (t, *J* = 2.9 Hz, 1H), 3.84 (td, *J* = 11.0, 3.0 Hz, 1H), 3.66 (s, 3H), 3.62 (s, 2H), 3.58 (dtd, *J* = 11.3, 3.8, 1.2 Hz, 1H), 2.11 – 2.02 (m, 1H), 1.95 – 1.82 (m, 2H), 1.75 – 1.64 (m, 2H), 1.65 – 1.57 (m, 1H); **^{13}C NMR** (126 MHz, Acetone-d_6) δ 171.2, 152.3, 133.8, 129.5, 129.3, 116.4, 112.1, 96.4, 61.4, 51.2, 39.0, 29.9, 25.0, 18.3. The characterization data matches previous report.[15]

Compound S22:

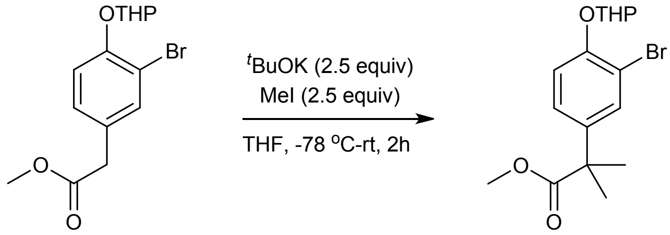

S21
Chemical Formula: $C_{14}H_{17}BrO_4$
Molecular Weight: 329.1864

S22
Chemical Formula: $C_{16}H_{21}BrO_4$
Molecular Weight: 357.2395

Procedure: A flame-dried, 250 mL round-bottom flask equipped with a Teflon-coated stir bar and a rubber septum was charged with potassium *tert*-butoxide (^{t}BuOK) (125 mmol, 14.03 g, 2.5 equiv) and then purged with a steady stream of N_2 for 5 min, prior to the addition of dry and degassed THF (50 mL). In a separate, flame-dried, 100 mL round-bottom flask equipped with a Teflon-coated stir bar and a rubber septum was charged with compound **S21** (50 mmol, 16.46 g, 1.0 equiv) and then purged with a steady stream of N_2 for 5 min, prior to the addition of dry and degassed THF (50 mL) to afford a homogeneous solution. It was added to the ^{t}BuOK solution in THF via syringe at -78 °C. After stirring for 20 min at -78 °C, iodomethane (125 mmol, 7.78 mL, 2.5 equiv) was added via syringe dropwise to the solution, and the resulting mixture was stirred at -78 °C for 1h and then warmed to room temperature and stirred for another 1h before

[14] Sun,H.; Zhu, L.; Yang, H.; Qian, W.; Guo, L.; Zhou, S.; Gao, B.; Li, Z.; Zhou, Y.; Jiang, H.; Chen, K.; Zhen, X.; Liu, H. *Bioorg. Med. Chem.* **2013**, *21*, 856-868.

[15] Bubert, C.; Woo, L. W. L.; Sutcliffe, O. B.; Mahon, M. F.; Chander, S. K.; Purohit, A.; Reed, M. J.; Potter, B. V. L. *ChemMedChem* **2008**, *3*, 1708-1730.

quenching with water (100 mL). It was extracted with ethyl acetate (3 × 100 mL), and the combined organic layers were dried over $MgSO_4$, filtered and concentrated *in vacuo*. The crude product was purified on silica gel using 5% ethyl acetate in hexanes as eluent to afford **S22** (16.38 g, 45.8 mmol, 92% yield) as a pale-yellow oil.

Characterization:

R_f = (EtOAc/Hexane 1:10): 0.40; **IR** (neat) v = 2946, 2876, 1729, 1494, 1387, 1356, 1248, 1202, 1144, 1111 cm^{-1}; **^1H NMR** (500 MHz, Acetone-d_6) δ 7.54 (d, J = 2.4 Hz, 1H), 7.29 (dd, J = 8.7, 2.4 Hz, 1H), 7.19 (d, J = 8.7 Hz, 1H), 5.59 (t, J = 2.9 Hz, 1H), 3.84 (td, J = 11.0, 3.0 Hz, 1H), 3.64 (s, 3H), 3.58 (dtd, J = 11.3, 3.8, 1.2 Hz, 1H), 2.09 – 2.05 (m, 1H), 1.94 – 1.82 (m, 2H), 1.74 – 1.65 (m, 2H), 1.64 – 1.58 (m, 1H), 1.55 (s, 6H); **^{13}C NMR** (126 MHz, Acetone-d_6) δ 176.0, 152.0, 139.5, 130.3, 126.0, 116.3, 112.2, 96.4, 61.4, 51.5, 45.5, 29.9, 25.99, 25.97, 25.0, 18.3; **HRMS**: Calcd. for $C_{16}H_{21}O_4Br$ $[M+Na]^+$ = 379.0515 m/z, found = 379.0514 m/z.

Compound S23:

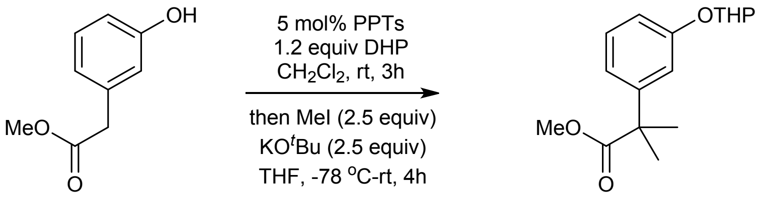

S23

Chemical Formula: $C_9H_{10}O_3$
Molecular Weight: 166.1739

Chemical Formula: $C_{16}H_{22}O_4$
Molecular Weight: 278.3435

Procedure: A flame-dried, 500 mL round-bottom flask equipped with a Teflon-coated stir bar and a rubber septum was charged with pyridinium p-toluenesulfonate (PPTs) (2.5 mmol, 628.2 mg, 5 mol%) and methyl 2-(3-hydroxyphenyl)acetate (50 mmol, 8.31 g, 1.0 equiv) and then purged with a steady stream of N_2 for 5 min, prior to the addition of dry and degassed CH_2Cl_2 (100 mL). 3,4-Dihydro-2H-pyran (DHP) (60 mmol, 5.09 mL, 1.2 equiv) was then added via syringe. The mixture was stirred at room temperature for 3h, and then quenched with water (100 mL). The aqueous layer was washed with CH_2Cl_2 (3 × 100 mL), and the combined organic layers were dried over $MgSO_4$, filtered and concentrated *in vacuo*. The crude product was dissolved in dried and degassed THF (100 mL), and cooled to -78 °C, at which temperature a solution of potassium *tert*-butoxide (^{t}BuOK) (50 mL, 2.5 M in THF) was added dropwise. After stirring for 20 min at -78 °C, iodomethane (125 mmol, 7.78 mL, 2.5 equiv) was added via syringe dropwise to the solution, and the resulting mixture was stirred at -78 °C for 1h and then warmed to room temperature and stirred for another 3h before quenching with water (100 mL). It was extracted with ethyl acetate (3 × 100 mL), and the combined organic layers were dried over $MgSO_4$, filtered and concentrated *in vacuo*. The crude product was purified on silica gel using 10% ethyl acetate in hexanes as eluent to afford **S23** (11.72 g, 42.1 mmol, 84% yield) as a colorless oil.

Characterization:

R_f = (EtOAc/Hexane 1:10): 0.39; **IR** (neat) v = 2945, 2874, 1730, 1606, 1582, 1488, 1433, 1387, 1357, 1253, 1190, 1144, 1107 cm^{-1}; **^1H NMR** (500 MHz, Acetone-d_6) δ 7.25 (t, J = 8.0 Hz, 1H), 7.02 (d, J = 1.8 Hz, 1H), 6.96 (dd, J = 8.0, 1.3 Hz, 2H), 5.44 (t, J = 3.3 Hz, 1H), 3.86 (ddd, J = 11.9, 9.4, 3.2 Hz, 1H), 3.63 (s, 3H), 3.58 (dt, J = 11.8, 4.2 Hz, 1H), 2.02 – 1.94 (m, 1H), 1.89 – 1.77 (m, 2H), 1.71 – 1.63 (m, 2H), 1.61 – 1.56 (m, 1H), 1.54 (s, 6H); **^{13}C NMR** (126 MHz, Acetone-d_6) δ 176.2, 157.4, 146.4, 129.1, 118.6, 114.25, 114.23, 96.3, 61.6, 51.4, 46.3, 30.2, 26.0, 25.1, 18.8; **GC-MS** R_t = 22.02 min, m/z = 278.1, 219.2, 194.2, 135.2, 107.1, 85.1.

c) Synthesis and Characterization of the Phenols

i. Substrates in Table 1

Compound S24:

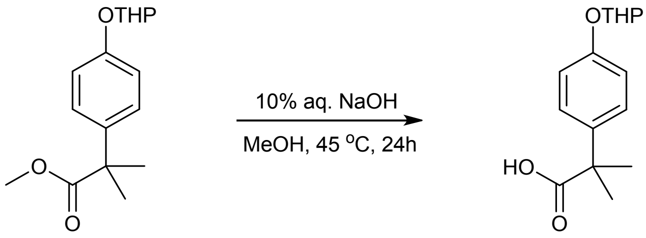

S8 **Chemical Formula:** $C_{16}H_{22}O_4$ **Molecular Weight:** 278.3435

S24 **Chemical Formula:** $C_{15}H_{20}O_4$ **Molecular Weight:** 264.3169

Procedure: A flame-dried, 250 mL round-bottom flask equipped with a Teflon-coated stir bar and a rubber septum was charged with compound **S8** (30 mmol, 8.35 g, 1 equiv) and then added methanol (30 mL) and 10% sodium hydroxide aqueous solution (30 mL). The mixture was stirred at 45 °C for 24h, and concentrated *in vacuo*. The resulted mixture was diluted ethyl acetate (80 mL), and then acidified with 1 M HCl solution to pH =3. The aqueous layer was separated and extracted with ethyl acetate (3 × 50 mL). The combined organic layers were filtered and concentrated *in vacuo* to afford **S24** (6.58 g, 24.89 mmol, 83% yield) as a white solid, which was used directly without further purification.

Characterization:

R_f = (EtOAc/Hexane 1:4): 0.33; **IR** (neat) v = 3063, 2943, 2882, 1692, 1609, 1510, 1467, 1407, 1369, 1282, 1239, 1202, 1170, 1161, 1112, 1050, 1036, 1023, 960, 919, 873, 827, 732, 645 cm^{-1}; **^{1}H NMR** (400 MHz, $CDCl_3$) δ 11.12 (br. s, 1H), 7.31 (d, *J* = 8.8 Hz, 2H), 7.01 (d, *J* = 8.8 Hz, 2H), 5.41 (t, *J* = 3.3 Hz, 1H), 3.90 (ddd, *J* = 12.4, 9.4, 3.1 Hz, 1H), 3.64 – 3.55 (m, 1H), 2.09 – 1.93 (m, 1H), 1.90 – 1.80 (m, 2H), 1.75 – 1.60 (m, 3H), 1.57 (s, 6H); **^{13}C NMR** (75 MHz, Acetone-d_6) δ 178.1, 155.8, 138.2, 126.6, 116.1, 96.1, 61.5, 45.4, 30.2, 26.3, 25.1, 18.7; **HRMS**: Calcd. for $C_{15}H_{20}O_4$ $[M+Na]^+$ = 287.1254 m/z, found = 287.1249 m/z.

Compound P1:

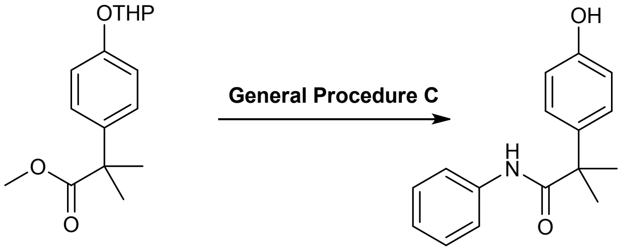

S8 **Chemical Formula:** $C_{16}H_{22}O_4$ **Molecular Weight:** 278.3435

P1 **Chemical Formula:** $C_{16}H_{17}NO_2$ **Molecular Weight:** 255.3117

Procedure: The reaction was carried out according to the General Procedure C on 10 mmol scale.

Amounts of Reagents:

S8 (10 mmol, 2.78 g)

aniline (15 mmol, 1.37 mL)

NaHMDS (20 mL, 1M solution in THF)

THF (50 mL, 0.2 M)

oxalic acid (20 mmol, 1.80 g)

MeOH/H_2O (90 mL/10 mL, 0.1 M)

Purification: 30% EtOAc in hexanes.

Yield of Product:

P1: 2.41 g, 9.44 mmol, 94% yield, white solid.

Characterization:

R$_f$ = (EtOAc/Hexane 3:7): 0.28; **IR** (neat) ν = 3341, 2943, 1655, 1598, 1531, 1508, 1493, 1436, 1393, 1357, 1314, 1038, 1016, 919, 752, 693, 545, 511 cm^{-1}; **^{1}H NMR** (400 MHz, Acetone-*d*$_6$) δ 8.27 (br. s, 1H), 8.19 (br. s, 1H), 7.58 (dd, *J* = 8.7, 1.1 Hz, 2H), 7.28 – 7.19 (m, 4H), 7.00 (t, *J* = 7.4 Hz, 1H), 6.81 (d, *J* = 8.8 Hz, 2H), 1.58 (s, 6H); **^{13}C NMR** (75 MHz, Acetone-*d*$_6$) δ 175.2, 156.1, 139.6, 136.2, 128.4, 127.2, 123.1, 119.6, 115.2, 46.9, 26.7; **HRMS**: Calcd. for $C_{16}H_{17}NO_2$ $[M+H]^+$ = 256.1332 m/z, found = 256.1331 m/z.

Compound 1a:

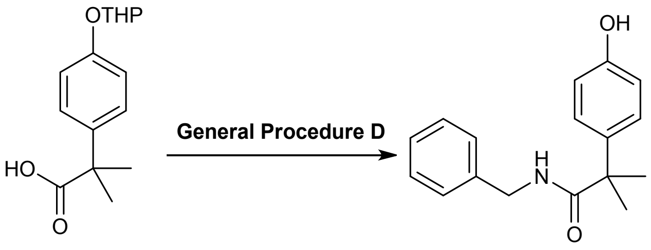

S24
Chemical Formula: $C_{15}H_{20}O_4$
Molecular Weight: 264.3169

1a
Chemical Formula: $C_{17}H_{19}NO_2$
Molecular Weight: 269.3383

Procedure: The reaction was carried out according to the General Procedure D.

Amounts of Reagents:

S24 (3 mmol, 783.0 mg)
benzylamine (6 mmol, 655.4 μL)
EDC (4.5 mmol, 698.6 mg)
HOBt (4.5 mmol, 608.0 mg)
N-methylmorpholine (30 mmol, 3.3 mL)
THF (15 mL, 0.2 M)
oxalic acid (6 mmol, 540.2 mg)
MeOH/H_2O (27 mL/3 mL, 0.1 M)

Purification: 50% EtOAc in hexanes.

Yield of Product:

1a: 622.17 mg, 2.31 mmol, 77% yield, white solid.

Characterization:

R$_f$ = (EtOAc/hexane 1:1): 0.46; **IR** (neat) ν = 3329, 3128, 2972, 1635, 1511, 1454, 1285, 1177, 828, 735, 694 cm^{-1}; **^{1}H NMR** (300 MHz, Acetone-*d*$_6$) δ 8.24 (br. s, 1H), 7.41 – 7.04 (m, 7H), 6.86 (br. s, 1H), 6.77 (d, *J* = 8.6 Hz, 2H), 4.34 (d, *J* = 6.1 Hz, 2H), 1.51 (s, 6H); **^{13}C NMR** (75 MHz, Acentone-*d*$_6$) δ 176.5, 155.9, 140.1, 136.7, 128.1, 127.3 , 127.1, 126.5, 115.0, 45.7, 42.7, 26.7; **HRMS**: calcd. for $C_{17}H_{19}NO_2$ $[M+Na]^+$ = 292.1308 m/z, found = 292.1307 m/z.

Compound 1b:

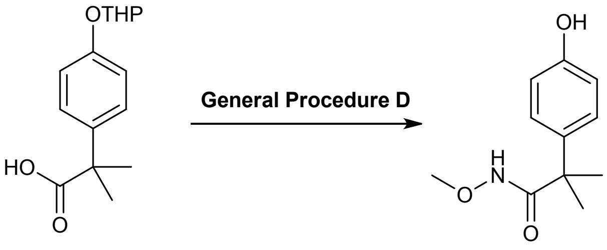

S24
Chemical Formula: $C_{15}H_{20}O_4$
Molecular Weight: 264.3169

1b
Chemical Formula: $C_{11}H_{15}NO_3$
Molecular Weight: 209.2417

Procedure: The reaction was carried out according to the General Procedure D.

Amounts of Reagents:

S24 (3 mmol, 783.0 mg)

O-methylhydroxylamine hydrochloride (3.6 mmol, 300.7 mg)

EDC (4.5 mmol, 698.6 mg)

HOBt (4.5 mmol, 608.0 mg)

N-methylmorpholine (30 mmol, 3.3 mL)

THF (15 mL, 0.2 M)

oxalic acid (6 mmol, 540.2 mg)

MeOH/H_2O (27 mL/3 mL, 0.1 M)

Purification: 50% EtOAc in hexanes.

Yield of Product:

1b: 313.9 mg, 1.50 mmol, 50% yield, white solid.

Characterization:

R*f* = (EtOAc): 0.50; **IR** (neat) ν = 3240, 2977, 2934, 1647, 1605, 1500, 1276, 1005, 889, 870, 556 cm^{-1}; **^{1}H NMR** (300 MHz, Acentone-*d6*) δ 9.62 (br. s, 1H), 8.24 (br. s, 1H), 7.19 (d, *J* = 8.7 Hz, 2H), 6.78 (d, *J* = 8.7 Hz, 2H), 3.58 (s, 3H), 1.48 (s, 6H); **^{13}C NMR** (75 MHz, Acentone-*d6*) δ 174.4, 156.1, 135.7, 127.0, 115.1, 62.7, 44.5, 26.5; **HRMS**: calcd. for $C_{11}H_{15}NO_3$ $[M+H]^+$ = 210.1125 m/z, found = 210.1126 m/z.

Compound S25:

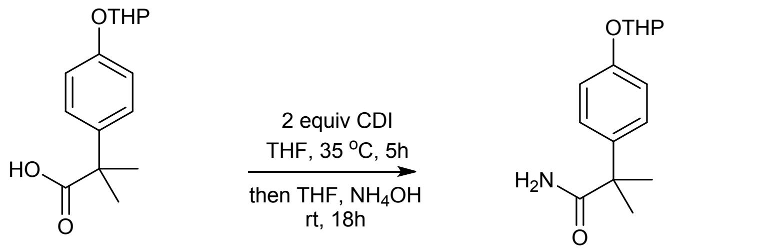

S24
Chemical Formula: $C_{15}H_{20}O_4$
Molecular Weight: 264.3169

S25
Chemical Formula: $C_{15}H_{21}NO_3$
Molecular Weight: 263.3321

Procedure: A flame-dried, 250 mL round-bottom flask equipped with a Teflon-coated stir bar and a rubber septum was charged with compound **S24** (10 mmol, 2.64 g, 1.0 equiv) and then purged with a steady stream of N_2 for 5 min, prior to the addition of dry and degassed THF (100 mL). N,N'-Carbonyldiimidazole (CDI) (20 mmol, 3.24 g, 2 equiv) was then added as solid and the resulting solution was stirred at 35 °C for 5 h and concentrated *in vacuo*. The residue was dissolved in THF (100 mL) and saturated ammonium hydroxide aqueous solution (100 mL) was added and stirred for another 18 h. The mixture was extracted with ethyl acetate (3 × 100 mL), and the combined organic layers were tried with $MgSO_4$, filtered, and concentrated *in vacuo*. The crude product was purification on silica gel using 70% ethyl acetate in hexanes as eluent to afford **S25** (1.37 g, 5.20 mmol, 52% yield) as a white solid.

Characterization:

R*f* = (EtOAc/hexane 1:1): 0.33; **IR** (neat) ν = 3384, 3210, 2954, 1617, 1511, 1396, 1241, 1076, 1027, 835, 560 cm^{-1}; **^{1}H NMR** (300 MHz, Acentone-*d6*) δ 7.30 (d, *J* = 8.4 Hz, 2H), 6.99 (d, *J* = 8.4 Hz, 2H), 6.15 (br. s, 2H), 5.42 (t, *J* = 3.4 Hz, 1H), 3.90 – 3.78 (m, 1H), 3.62 – 3.51 (m, 1H), 2.01 – 1.92 (m, 1H), 1.87 – 1.74 (m, 2H), 1.72 – 1.56 (m, 3H), 1.49 (s, 6H); **^{13}C NMR** (75 MHz, Acentone-*d6*) δ 178.3, 155.7, 139.1, 126.9, 116.1, 96.2, 61.5, 45.7, 30.2, 26.7, 26.6, 25.1, 18.7; **HRMS**: calcd. for $C_{15}H_{21}NO_3$ $[M+Na]^+$ = 286.1414 m/z, found = 286.1414 m/z.

Compound S26:

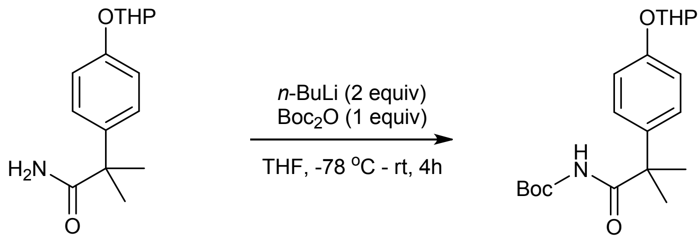

S25
Chemical Formula: $C_{15}H_{21}NO_3$
Molecular Weight: 263.3321

S26
Chemical Formula: $C_{20}H_{29}NO_5$
Molecular Weight: 363.4480

Procedure: A flame-dried, 250 mL round-bottom flask equipped with a Teflon-coated stir bar and a rubber septum was charged with compound **S25** (6 mmol, 1.58 g, 1 equiv) and then purged with a steady stream of N_2 for 5 min, prior to the addition of dry and degassed THF (60 mL). The resulting suspension was cooled down to -78 °C and *n*-Butyl lithium solution (12 mmol, 4.8 mL of 2.5 M in hexane, 2 equiv) was added dropwise. The mixture was warmed up and stirred at room temperature for 20 min. The solution turned clear, and cooled down again to -78 °C. In a separate, flame-dried, 50 mL round-bottom flask equipped with a Teflon-coated stir bar and a rubber septum was charged with di-*tert*-butyl dicarbonate (Boc_2O) (6 mmol, 1.31 g, 1.0 equiv) and then purged with a steady stream of N_2 for 5 min, prior to the addition of dry and degassed THF (20 mL) to afford a homogeneous solution. It was added to the reaction solution dropwise via syringe at -78 °C and then warmed up to room temperature and stirred for 4h. The solution was quenched by saturated NH_4Cl solution (50 mL), and extracted with ethyl acetate (3 × 50 mL). The combined organic layers were dried with $MgSO_4$, filtered, and concentrated *in vacuo*. The crude product was purification on silica gel using 20% ethyl acetate in hexanes as eluent to afford **S26** (1.71 g, 4.7 mmol, 78% yield) as a white solid.

Characterization:

R*f* = (EtOAc/hexane 1:4): 0.29; **IR** (neat) ν = 3321, 2952, 2928, 2878, 1783, 1769, 1495, 1471, 1365, 1244, 1205, 1179, 1139, 1026, 992, 965, 919, 873, 830, 700, 553 cm^{-1}; **^{1}H NMR** (500 MHz, Acentone-*d$_6$*) δ 8.42 (br. s, 1H), 7.23 (d, *J* = 8.8 Hz, 2H), 7.02 (d, *J* = 8.8 Hz, 2H), 5.45 (t, *J* = 3.3 Hz, 1H), 3.90 – 3.79 (m, 1H), 3.58 (dt, *J* = 9.8, 3.9 Hz, 1H), 2.02 – 1.90 (m, 1H), 1.89 – 1.74 (m, 2H), 1.70 – 1.61 (m, 2H), 1.60 – 1.55 (m, 1H), 1.53 (s, 3H), 1.52 (s, 3H), 1.37 (s, 9H); **^{13}C NMR** (75 MHz, Acentone-*d$_6$*) δ 174.2, 156.1, 149.6, 137.0, 127.1, 116.4, 96.1, 80.3, 61.6, 47.6, 30.2, 27.2, 26.1, 25.9, 25.1, 18.7; **HRMS**: calcd. for $C_{20}H_{29}NO_5$ $[M+Na]^+$ = 386.1938 m/z, found = 386.1938 m/z.

Compound 1c:

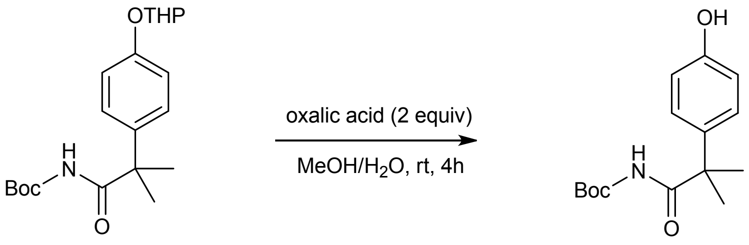

S26
Chemical Formula: $C_{20}H_{29}NO_5$
Molecular Weight: 363.4480

1c
Chemical Formula: $C_{15}H_{21}NO_4$
Molecular Weight: 279.3315

Procedure: A flame-dried, 100 mL round-bottom flask equipped with a Teflon-coated stir bar and a rubber septum was charged with compound **S26** (4.7 mmol, 1.71 g, 1 equiv) and added methanol and water (9:1 by volume, 50 mL). Oxalic acid (9.4 mmol, 846.3 mg, 2 equiv) was then added as solid. After stirring for 4h at room temperature, the mixture was concentrated *in vacuo*, diluted with ethyl acetate (50 mL) and washed with saturated sodium bicarbonate solution (50 mL). The aqueous layer was separated and extracted with ethyl acetate (3 × 50 mL), and the combined organic layers were dried over $MgSO_4$, filtered and concentrated *in vacuo*. The crude product was then purified on silica gel using 40%

ethyl acetate in hexanes as eluent to afford **1c** (1.01 g, 3.62 mmol, 77% yield) as a white solid.

Characterization:

R*f* = (EtOAc/hexane 1:2): 0.24; **IR** (neat) v = 3326, 2974, 1749, 1492, 1368, 1271, 1245, 1136, 996, 963, 916, 828, 748, 545 cm^{-1}; **^{1}H NMR** (400 MHz, Acentone-d_6) δ 8.32 (br. s, 1H), 8.26 (br. s, 1H), 7.18 (d, *J* = 8.8 Hz, 2H), 6.84 (d, *J* = 8.8 Hz, 2H), 1.52 (s, 6H), 1.38 (s, 9H); **^{13}C NMR** (75 MHz, Acentone-d_6) δ 174.3, 156.3, 149.5, 134.8, 127.2, 115.3, 80.3, 47.4, 27.2, 26.0; **HRMS**: calcd. for $C_{15}H_{21}NO_4$ $[M+Na]^+$ = 302.1363 m/z, found = 302.1362 m/z.

ii. Substrates in Table 2

Compound S27:

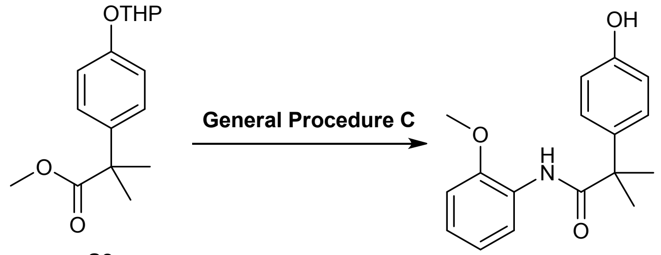

S8 Chemical Formula: $C_{16}H_{22}O_4$ Molecular Weight: 278.3435

S27 Chemical Formula: $C_{17}H_{19}NO_3$ Molecular Weight: 285.3377

Procedure: The reaction was carried out according to the General Procedure C on 2 mmol scale.

Amounts of Reagents:

S8 (2 mmol, 556.7 mg)

2-methoxyaniline (3 mmol, 369.5 mg)

NaHMDS (4 mL, 1M solution in THF)

THF (10 mL, 0.2 M)

oxalic acid (4 mmol, 360.1 mg)

MeOH/H_2O (18 mL/2 mL, 0.1 M)

Purification: 40% EtOAc in hexanes.

Yield of Product:

S27: 439.1 mg, 1.54 mmol, 77% yield, white solid.

Characterization:

R*f* = (EtOAc/Hexane 4:6): 0.43; **IR** (neat) v = 3398, 3254, 1614, 1517, 1452, 1231, 838, 761 cm^{-1}; **^{1}H NMR** (300 MHz, $CDCl_3$) δ 8.32 (dd, *J* = 7.9, 1.8 Hz, 1H), 7.73 (br. s, 1H), 7.32 (d, *J* = 8.7 Hz, 2H), 6.97 (dd, *J* = 7.8, 1.8 Hz, 1H), 6.92 (td, *J* = 7.7, 1.5 Hz, 1H), 6.87 (d, *J* = 8.7 Hz, 2H), 6.77 (dd, *J* = 8.0, 1.5 Hz, 1H), 5.66 (br. s, 1H), 3.66 (s, 3H), 1.65 (s, 6H); **^{13}C NMR** (75 MHz, $CDCl_3$) δ 176.0, 154.9, 148.0, 136.5, 127.8, 127.8, 123.6, 121.1, 119.4, 115.5, 110.0, 55.7, 47.5, 27.0; **HRMS**: calcd. for $C_{17}H_{19}NO_3$ $[M+Na]^+$ = 308.1257 m/z, found = 308.1247 m/z.

Compound S28:

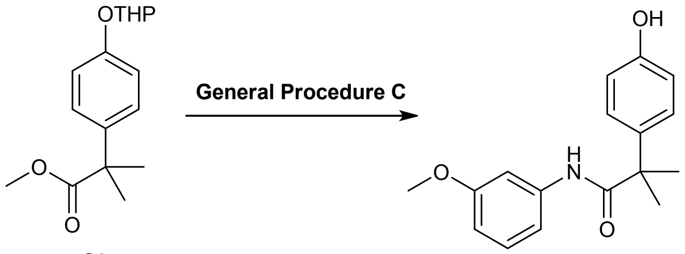

S8 Chemical Formula: $C_{16}H_{22}O_4$ Molecular Weight: 278.3435

S28 Chemical Formula: $C_{17}H_{19}NO_3$ Molecular Weight: 285.3377

Procedure: The reaction was carried out according to the General Procedure C on 2 mmol scale.

Amounts of Reagents:

S8 (2 mmol, 556.7 mg)

3-methoxyaniline (3 mmol, 369.5 mg)

NaHMDS (4 mL, 1M solution in THF)

THF (10 mL, 0.2 M)

oxalic acid (4 mmol, 360.1 mg)

MeOH/H_2O (18 mL/2 mL, 0.1 M)

Purification: 40% EtOAc in hexanes.

Yield of Product:

S28: 473.7 mg, 1.66 mmol, 83% yield, white solid.

Characterization:

R*f* = (EtOAc/Hexane 4:6): 0.42; **IR** (neat) ν = 330, 3234, 1659, 1479, 1450, 1430, 1246, 1162, 1050, 887, 830, 775 cm^{-1}; **^{1}H NMR** (300 MHz, Acetone-d_6) δ 8.29 (br. s, 1H), 8.22 (br. s, 1H), 7.37 (dd, *J* = 2.8, 1.5 Hz, 1H), 7.22 (d, *J* = 8.7 Hz, 2H), 7.14-7.11(m, 2H), 6.81 (d, *J* = 8.7 Hz, 2H), 6.59 (dt, *J* = 7.1, 2.4 Hz, 1H), 3.72 (s, 3H), 1.57 (s, 6H); **^{13}C NMR** (75 MHz, Acetone-d_6) δ 175.3, 160.0, 156.1, 140.7, 136.2, 129.1, 127.2, 115.2, 111.7, 108.7, 105.3, 54.5, 47.0, 26.7; **HRMS**: calcd. for $C_{17}H_{19}NO_3$ $[M+H]^+$ = 286.1438 m/z, found = 286.1437 m/z.

Compound S29:

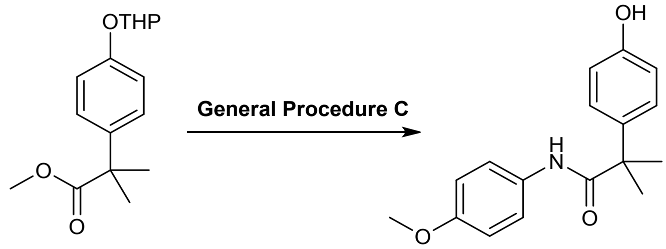

S8
Chemical Formula: $C_{16}H_{22}O_4$
Molecular Weight: 278.3435

S29
Chemical Formula: $C_{17}H_{19}NO_3$
Molecular Weight: 285.3377

Procedure: The reaction was carried out according to the General Procedure C on 2 mmol scale.

Amounts of Reagents:

S8 (2 mmol, 556.7 mg)

4-methoxyaniline (3 mmol, 369.5 mg)

NaHMDS (4 mL, 1M solution in THF)

THF (10 mL, 0.2 M)

oxalic acid (4 mmol, 360.1 mg)

MeOH/H_2O (18 mL/2 mL, 0.1 M)

Purification: 40% EtOAc in hexanes.

Yield of Product:

S29: 486.8 mg, 1.71 mmol, 86% yield, white solid.

Characterization:

R*f* = (EtOAc/Hexane 4:6): 0.32; **IR** (neat) ν = 3234, 2973, 1638, 1508, 1232, 1032, 828, 540 cm^{-1}; **^{1}H NMR** (400 MHz, Acetone-d_6) δ 8.28 (br. s, 1H), 8.10 (br. s, 1H), 7.47 (d, *J* = 9.1 Hz, 2H), 7.23 (d, *J* = 8.9 Hz, 2H), 6.81 (dd, *J* = 8.9, 1.3 Hz, 4H), 3.73 (s, 3H), 1.57 (s, 6H); **^{13}C NMR** (75 MHz, Acetone-d_6) δ 175.0, 156.0, 155.8, 136.5, 132.6, 127.2, 121.4, 115.1, 113.5, 54.7, 46.7, 26.7; **HRMS**: calcd. for $C_{17}H_{19}NO_3$ $[M+H]^+$ = 286.1438 m/z, found = 286.1436 m/z.

Compound S30:

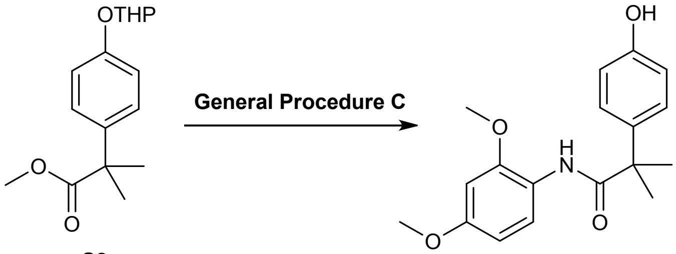

S8
Chemical Formula: $C_{16}H_{22}O_4$
Molecular Weight: 278.3435

S30
Chemical Formula: $C_{18}H_{21}NO_4$
Molecular Weight: 315.3636

Procedure: The reaction was carried out according to the General Procedure C on 2 mmol scale.

Amounts of Reagents:

S8 (2 mmol, 556.7 mg)

2,4-dimethoxyaniline (3 mmol, 459.2 mg)

NaHMDS (4 mL, 1M solution in THF)

THF (10 mL, 0.2 M)

oxalic acid (4 mmol, 360.1 mg)

MeOH/H_2O (18 mL/2 mL, 0.1 M)

Purification: 40% EtOAc in hexanes.

Yield of Product:

S30: 416.3 mg, 1.32 mmol, 66% yield, white solid.

Characterization:

R_f = (EtOAc/Hexane 1:1): 0.47; **IR** (neat) v =3397, 3239, 2367, 2183, 1642, 1615, 1517, 1455, 1281, 1216, 1120, 1029, 839, 792, 668, 726, 638, 537 cm^{-1}; **^{1}H NMR** (400 MHz, Acentone-d_6) δ 8.37 (br. s, 1H), 8.14 (d, J = 8.8 Hz, 1H), 7.53 (br. s, 1H), 7.32 (d, J = 8.7 Hz, 2H), 6.88 (d, J = 8.7 Hz, 2H), 6.50 (d, J = 2.6 Hz, 1H), 6.47-6.45 (m, 1H), 3.74 (s, 3H), 3.69 (s, 3H), 1.58 (s, 6H); **^{13}C NMR** (75 MHz, Acentone-d_6) δ 174.6, 156.3, 149.5, 135.8, 127.5, 121.7, 119.9, 115.3, 103.8, 98.4, 55.4, 54.8, 46.9, 26.6; **HRMS**: calcd. for $C_{18}H_{21}NO_4$ $[M-H]^-$ = 314.1371 m/z, found = 314.1385 m/z.

Compound S31:

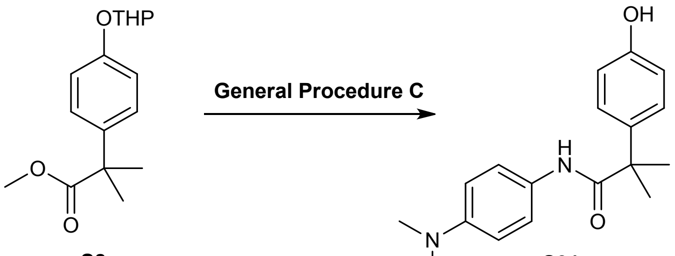

S8
Chemical Formula: $C_{16}H_{22}O_4$
Molecular Weight: 278.3435

S31
Chemical Formula: $C_{18}H_{22}N_2O_2$
Molecular Weight: 298.3795

Procedure: The reaction was carried out according to the General Procedure C on 10 mmol scale.

Amounts of Reagents:

S8 (10 mmol, 2.78 g)

N,N-dimethylbenzene-1,4-diamine (15 mmol, 2.04 g)

NaHMDS (20 mL, 1M solution in THF)

THF (50 mL, 0.2 M)

oxalic acid (20 mmol, 1.80 g)

MeOH/H_2O (90 mL/10 mL, 0.1 M)

Purification: 50% EtOAc in hexanes.

Yield of Product:

S31: 2.06 g, 6.90 mmol, 69% yield, green solid.

Characterization:

R$_f$ = (EtOAc/Hexane 1:1): 0.30; **IR** (neat) ν = 3159, 2931, 2801, 1635, 1502, 1444, 1150, 806, 745, 602, 533 cm^{-1}; **^{1}H NMR** (300 MHz, Acetone-d_6) δ 8.23 (br. s, 1H), 7.89 (br. s, 1H), 7.37 (d, *J* = 9.0 Hz, 2H), 7.24 (d, *J* = 8.7 Hz, 2H), 6.80 (d, *J* = 8.7 Hz, 2H), 6.65 (d, *J* = 9.0 Hz, 2H), 2.85 (s, 6H), 1.56 (s, 6H); **^{13}C NMR** (75 MHz, Acetone-d_6) δ 174.6, 156.0, 147.5, 136.7, 129.5, 127.2, 121.2, 115.1, 112.6, 46.6, 40.1, 26.8; **HRMS**: calcd. for $C_{18}H_{22}N_2O_2$ [M-H]$^-$ = 297.1609 m/z, found = 297.1608 m/z.

Compound S32:

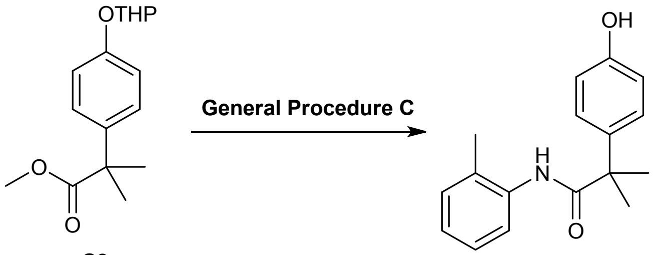

S8
Chemical Formula: $C_{16}H_{22}O_4$
Molecular Weight: 278.3435

S32
Chemical Formula: $C_{17}H_{19}NO_2$
Molecular Weight: 269.3383

Procedure: The reaction was carried out according to the General Procedure C.

Amounts of Reagents:

S8 (3 mmol, 835.0 mg)

o-toluidine (4.5 mmol, 482.2 mg)

NaHMDS (6 mL, 1M solution in THF)

THF (15 mL, 0.2 M)

oxalic acid (6 mmol, 540.2 mg)

MeOH/H_2O (27 mL/3 mL, 0.1 M)

Purification: 40% EtOAc in hexanes.

Yield of Product:

S32: 589.8 mg, 2.19 mmol, 73% yield, white solid.

Characterization:

R$_f$ = (EtOAc/Hexane 4:6): 0.56; **IR** (neat) ν = 3376, 3236, 1616, 1517, 1482, 1277, 1229, 835, 733, 556, 510 cm^{-1}; **^{1}H NMR** (300 MHz, $CDCl_3$) δ 7.87 (d, *J* = 8.1 Hz, 1H), 7.37 (d, *J* = 8.7 Hz, 2H), 7.17 (t, *J* = 7.7 Hz, 1H), 7.06 (d, *J* = 7.5 Hz, 1H), 7.01 (t, *J* = 6.2 Hz, 1H), 6.88 (d, *J* = 8.7 Hz, 2H), 6.76 (br. s, 1H),4.98 (br. s, 1H), 1.83 (s, 3H), 1.67 (s, 6H); **^{13}C NMR** (75 MHz, $CDCl_3$) δ 176.3, 155.1, 136.4, 135.8, 130.3, 128.2, 128.0, 126.7, 124.7, 121.9, 115.8, 47.4, 27.0, 17.1; **HRMS**: calcd. for $C_{17}H_{19}NO_2$ [M+Na]$^+$ = 292.1308 m/z, found = 292.1308 m/z.

Compound S33:

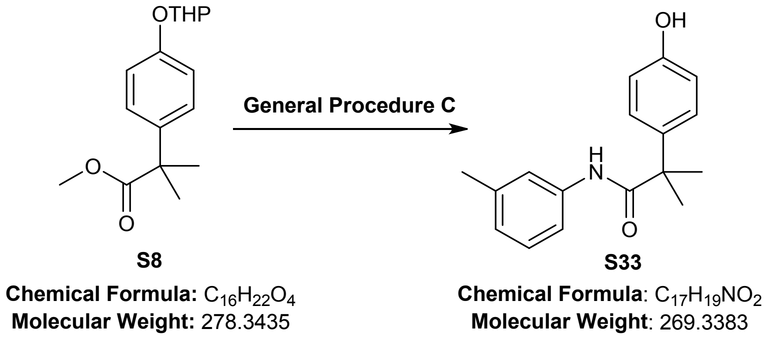

Procedure: The reaction was carried out according to the General Procedure C on 2 mmol scale.

Amounts of Reagents:

S8 (2 mmol, 556.7 mg)

m-toluidine (3 mmol, 321.4 mg)

NaHMDS (4 mL, 1M solution in THF)

THF (10 mL, 0.2 M)

oxalic acid (4 mmol, 360.1 mg)

MeOH/H_2O (18 mL/2 mL, 0.1 M)

Purification: 30% EtOAc in hexanes.

Yield of Product:

S33: 387.6 mg, 1.44 mmol, 72% yield, white solid.

Characterization:

R_f = (EtOAc/Hexane 3:7): 0.43; **IR** (neat) ν = 3158, 2969, 1611, 1513, 1314, 1182, 855, 682, 545, 520. cm^{-1}; **^{1}H NMR** (300 MHz, $CDCl_3$) δ 7.31 (d, *J* = 8.7 Hz, 2H), 7.22 (s, 1H), 7.15 – 7.10 (m, 2H), 6.87 (d, *J* = 8.7 Hz, 3H), 6.80 (br. s, 1H), 5.13 (br. s, 1H), 2.28 (s, 3H), 1.63 (s, 6H); **^{13}C NMR** (75 MHz, $CDCl_3$) δ 176.0, 154.8, 138.8, 137.8, 136.6, 128.7, 127.9, 124.9, 120.2, 116.6, 115.8, 47.4, 27.1, 21.4; **HRMS**: calcd. for $C_{17}H_{19}NO_2$ $[M+Na]^+$ = 292.1308 m/z, found = 292.1309 m/z.

Compound S34:

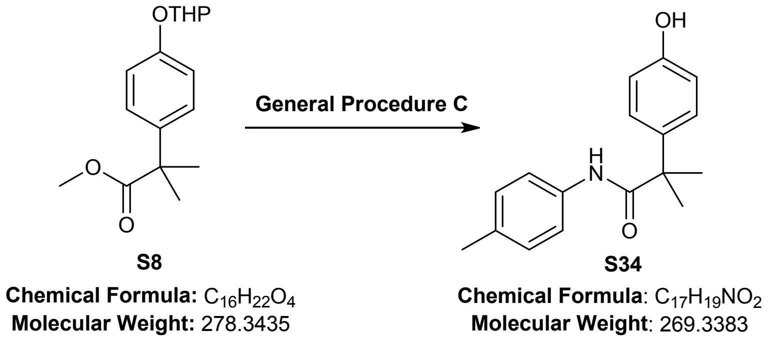

Procedure: The reaction was carried out according to the General Procedure C.

Amounts of Reagents:

S8 (3 mmol, 835.0 mg)

p-toluidine (4.5 mmol, 482.2 mg)

NaHMDS (6 mL, 1M solution in THF)

THF (15 mL, 0.2 M)

oxalic acid (6 mmol, 540.2 mg)

MeOH/H_2O (27 mL/3 mL, 0.1 M)

Purification: 30% EtOAc in hexanes.

Yield of Product:

S34: 670.6 mg, 2.49 mmol, 83% yield, white solid.

Characterization:

R$_f$ = (EtOAc/Hexane 3:7): 0.44; **IR** (neat) ν =3341, 1655, 1588,1508, 1435, 1109, 1017, 1038, 829, 753, 693, 545 cm^{-1}; **^{1}H NMR** (300 MHz, Acetone-d_6) δ 8.27 (br. s, 1H), 8.11 (br. s, 1H), 7.45 (d, *J* = 8.2 Hz, 2H), 7.23 (d, *J* = 8.4 Hz, 2H), 7.04 (d, *J* = 8.1 Hz, 2H), 6.80 (d, *J* = 8.5 Hz, 2H), 2.24 (s, 3H), 1.57 (s, 6H); **^{13}C NMR** (75 MHz, Acetone-d_6) δ 175.0, 156.0, 137.0, 136.4, 132.4, 128.8, 127.2, 119.7, 115.1, 46.8, 26.7, 19.8; **HRMS**: calcd. for $C_{17}H_{19}NO_2$ [M+Na]$^+$ = 292.1308 m/z, found = 292.1313 m/z.

Compound S35:

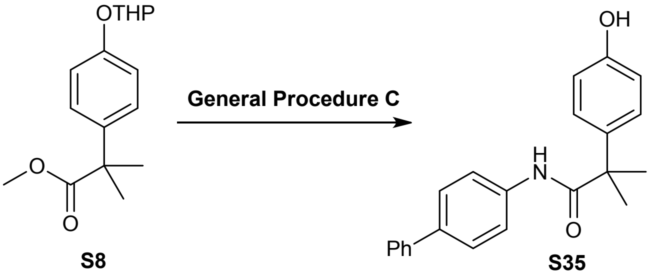

S8
Chemical Formula: $C_{16}H_{22}O_4$
Molecular Weight: 278.3435

S35
Chemical Formula: $C_{22}H_{21}NO_2$
Molecular Weight: 331.4076

Procedure: The reaction was carried out according to the General Procedure C.

Amounts of Reagents:

S8 (3 mmol, 835.0 mg)

4-phenylaniline (4.5 mmol, 760.9 mg)

NaHMDS (6 mL, 1M solution in THF)

THF (15 mL, 0.2 M)

oxalic acid (6 mmol, 540.2 mg)

MeOH/H_2O (27 mL/3 mL, 0.1 M)

Purification: 30% EtOAc in hexanes.

Yield of Product:

S35: 676.1 mg, 2.04 mmol, 68% yield, white solid.

Characterization:

R$_f$ = (EtOAc/Hexane 4:6): 0.51; **IR** (neat) ν = 3159, 2931, 2801, 1635, 1502, 1444, 1150, 806, 745, 602, 533 cm^{-1}; **^{1}H NMR** (300 MHz, Acetone-d_6) δ 8.32 (br. s, 1H), 8.31 (br. s, 1H), 7.70 (d, *J* = 8.7 Hz, 2H), 7.61 (d, *J* = 7.1 Hz, 2H), 7.55 (d, *J* = 8.7 Hz, 2H), 7.42 (t, *J* = 7.5 Hz, 2H), 7.34 – 7.21 (m, 3H), 6.83 (d, *J* = 8.7 Hz, 2H), 1.60 (s, 6H); **^{13}C NMR** (75 MHz, Acetone-d_6) δ 175.4, 156.1, 140.5, 139.0, 136.2, 135.7, 128.8, 127.2, 126.84, 126.83, 126.4, 120.0, 115.2, 47.0, 26.7; **HRMS**: calcd. for $C_{22}H_{21}NO_2$ [M+Na]$^+$ = 354.1464 m/z, found = 354.1468 m/z.

Compound S36:

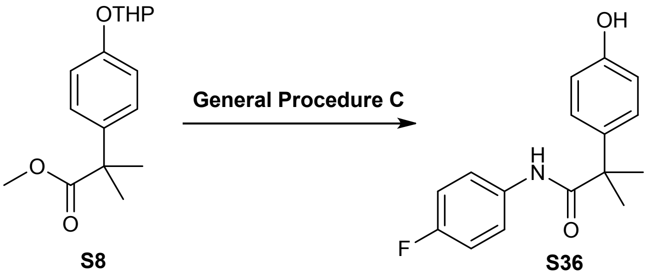

S8
Chemical Formula: $C_{16}H_{22}O_4$
Molecular Weight: 278.3435

S36
Chemical Formula: $C_{16}H_{16}FNO_2$
Molecular Weight: 273.3021

Procedure: The reaction was carried out according to the General Procedure C.

Amounts of Reagents:

S8 (3 mmol, 835.0 mg)

4-fluoroaniline (4.5 mmol, 499.7 mg)

NaHMDS (6 mL, 1M solution in THF)

THF (15 mL, 0.2 M)

oxalic acid (6 mmol, 540.2 mg)

MeOH/H_2O (27 mL/3 mL, 0.1 M)

Purification: 30% EtOAc in hexanes.

Yield of Product:

S36: 557.5 mg, 2.04 mmol, 68% yield, white solid.

Characterization:

R_f = (EtOAc/Hexane 3:7): 0.32; **IR** (neat) v = 3262, 1654, 1612, 1541, 1504, 1402, 1234, 1208, 1143, 827, 516 cm^{-1}; **^{1}H NMR** (400 MHz, Acetone-d_6) δ 8.31 (br. s, 2H), 7.72 – 7.49 (m, 2H), 7.22 (d, J = 8.7 Hz, 2H), 7.01 (t, J = 8.6 Hz, 2H), 6.80 (d, J = 8.7 Hz, 2H), 1.57 (s, 6H); **^{13}C NMR** (75 MHz, Acetone-d_6) δ 175.4, 158.7 (d, J_F = 240.4 Hz), 156.1, 136.2, 135.7 (d, J_F = 2.6 Hz), 127.2, 121.7 (d, J_F = 7.7 Hz), 115.2, 114.8 (d, J_F = 22.3 Hz), 46.8, 26.7; **HRMS**: calcd. for $C_{16}H_{16}FNO_2$ $[M+Na]^+$ = 296.1057 m/z, found = 296.1061 m/z.

Compound S37:

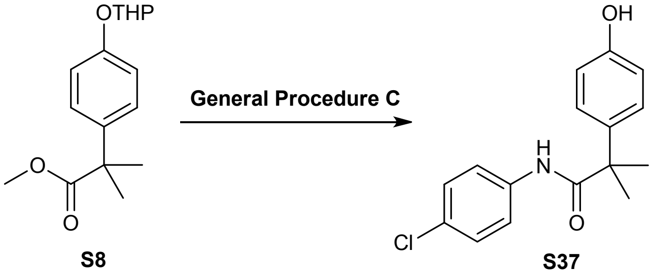

S8
Chemical Formula: $C_{16}H_{22}O_4$
Molecular Weight: 278.3435

S37
Chemical Formula: $C_{16}H_{16}ClNO_2$
Molecular Weight: 289.7567

Procedure: The reaction was carried out according to the General Procedure C.

Amounts of Reagents:

S8 (3 mmol, 835.0 mg)

4-chloroaniline (4.5 mmol, 571.5 mg)

NaHMDS (6 mL, 1M solution in THF)

THF (15 mL, 0.2 M)

oxalic acid (6 mmol, 540.2 mg)

MeOH/H_2O (27 mL/3 mL, 0.1 M)

Purification: 30% EtOAc in hexanes.

Yield of Product:

S37: 754.5 mg, 2.60 mmol, 87% yield, white solid.

Characterization:

R_f = (EtOAc/Hexanes 3:7): 0.47; **IR** (neat) v = 3396, 3305, 1659, 1594, 1492, 1394, 1306, 1182, 1106, 755, 635, 507 cm^{-1}; **^{1}H NMR** (400 MHz, Acetone-d_6) δ 8.39 (br. s, 1H), 8.30 (br. s, 1H), 7.62 (d, J = 8.9 Hz, 2H), 7.26 (d, J = 8.9 Hz, 2H), 7.21 (d, J = 8.7 Hz, 2H), 6.80 (d, J = 8.7 Hz, 2H), 1.57 (s, 6H); **^{13}C NMR** (75 MHz, Acetone-d_6) δ 175.5, 156.1, 138.4, 136.0, 128.3, 127.5, 127.2, 121.2, 115.2, 47.0, 26.6; **HRMS**: calcd. for $C_{16}H_{16}ClNO_2$ $[M-H]^-$ = 288.0797 m/z, found = 288.0802 m/z.

Compound S38:

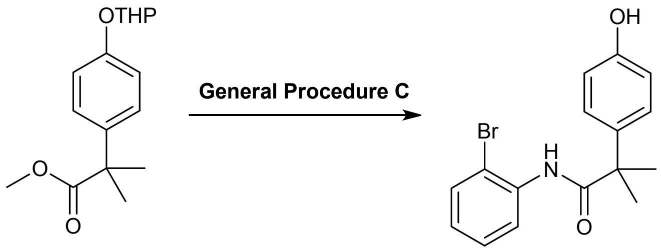

S8
Chemical Formula: $C_{16}H_{22}O_4$
Molecular Weight: 278.3435

S38
Chemical Formula: $C_{16}H_{16}BrNO_2$
Molecular Weight: 334.2077

Procedure: The reaction was carried out according to the General Procedure C.

Amounts of Reagents:

S8 (3 mmol, 835.0 mg)

2-bromoaniline (4.5 mmol, 769.3 mg)

NaHMDS (6 mL, 1M solution in THF)

THF (15 mL, 0.2 M)

oxalic acid (6 mmol, 540.2 mg)

MeOH/H_2O (27 mL/3 mL, 0.1 M)

Purification: 30% EtOAc in hexanes.

Yield of Product:

S38: 872.3 mg, 2.61 mmol, 87% yield, white solid.

Characterization:

R_f = (EtOAc/Hexane 3:7): 0.47; **IR** (neat) v =3260, 1653, 1500, 1447, 1276, 1181, 844, 743, 663, 532 cm^{-1}; **^{1}H NMR** (300 MHz, Acentone-d_6) δ 8.43 (br. s, 1H), 8.26 (dd, *J* = 8.3, 1.6 Hz, 1H), 7.71 (br. s, 1H),7.52 (dd, *J* = 8.0, 1.5 Hz, 1H), 7.41 – 7.25 (m, 3H), 7.00 (ddd, *J* = 8.1, 7.4, 1.6 Hz, 1H), 6.90 (d, *J* = 8.7 Hz, 2H), 1.63 (s, 6H); **^{13}C NMR** (75 MHz, Acentone-d_6) δ 175.5, 156.6, 136.4, 135.0, 132.2, 128.2, 127.8, 125.0, 121.7, 115.6, 113.4, 47.3, 26.4; **HRMS**: calcd. for $C_{16}H_{16}BrNO_2$ $[M-H]^-$ = 332.0292 m/z, found = 332.0281 m/z.

Compound S39:

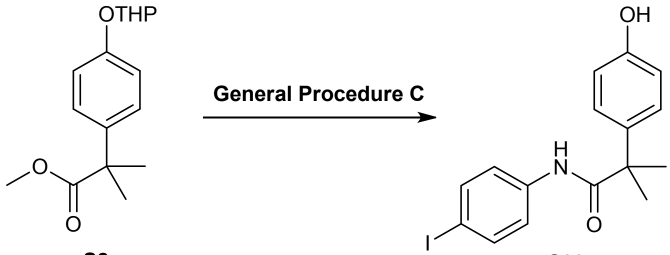

S8
Chemical Formula: $C_{16}H_{22}O_4$
Molecular Weight: 278.3435

S39
Chemical Formula: $C_{16}H_{16}INO_2$
Molecular Weight: 381.2082

Procedure: The reaction was carried out according to the General Procedure C.

Amounts of Reagents:

S8 (3 mmol, 835.0 mg)

4-iodoaniline (4.5 mmol, 985.3 mg)

NaHMDS (6 mL, 1M solution in THF)

THF (15 mL, 0.2 M)

oxalic acid (6 mmol, 540.2 mg)

MeOH/H_2O (27 mL/3 mL, 0.1 M)

Purification: 30% EtOAc in hexanes.

Yield of Product:

S39: 754.4 mg, 1.98 mmol, 66% yield, white solid.

Characterization:

R*f* = (EtOAc/Hexane 1:4): 0.30; **IR** (neat) v = 3368, 2359, 2337, 2170, 1977, 1697, 1652, 1592, 1452, 1391, 1221, 1003, 817, 627, 524, 503 cm^{-1}; **^{1}H NMR** (400 MHz, Acetone-d_6) δ 8.38 (br. s, 1H), 8.31 (br. s, 1H), 7.58 (d, J = 8.8 Hz, 2H), 7.45 (d, J = 8.9 Hz, 2H), 7.21 (d, J = 8.7 Hz, 2H), 6.80 (d, J = 8.7 Hz, 2H), 1.56 (s, 6H); **^{13}C NMR** (75 MHz, Acetone-d_6) δ 175.5, 156.1, 139.5, 137.4, 136.0, 127.2, 121.8, 115.2, 85.5, 47.0, 26.6; **HRMS**: calcd. for $C_{16}H_{16}INO_2$ $[M-H]^-$ = 380.0153 m/z, found = 380.0135 m/z.

Compound S40:

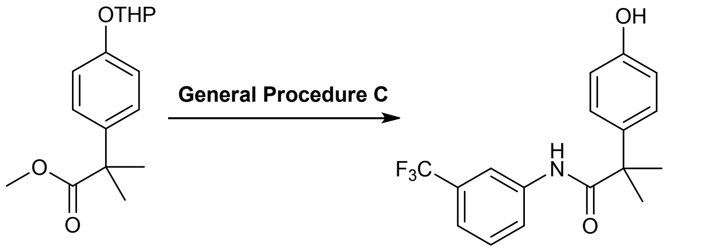

S8
Chemical Formula: $C_{16}H_{22}O_4$
Molecular Weight: 278.3435

S40
Chemical Formula: $C_{17}H_{16}F_3NO_2$
Molecular Weight: 323.3096

Procedure: The reaction was carried out according to the General Procedure C.

Amounts of Reagents:

S8 (3 mmol, 835.0 mg)

3-(trifluoromethyl)aniline (4.5 mmol, 725.0 mg)

NaHMDS (6 mL, 1M solution in THF)

THF (15 mL, 0.2 M)

oxalic acid (6 mmol, 540.2 mg)

MeOH/H_2O (27 mL/3 mL, 0.1 M)

Purification: 30% EtOAc in hexanes.

Yield of Product:

S40: 640.1 mg, 1.98 mmol, 66% yield, white solid.

Characterization:

R*f* = (EtOAc/Hexane 3:7): 0.50; **IR** (neat) v = 3188, 1657, 1616, 1546, 1513, 1498, 1247, 1127, 830, 692, 547 cm^{-1}; **^{1}H NMR** (300 MHz, Acentone-d_6) δ 8.62 (br. s, 1H), 8.32 (br. s, 1H), 8.12 (s, 1H), 7.82 (d, *J* = 8.1 Hz, 1H), 7.47 (t, *J* = 8.0 Hz, 1H), 7.34 (d, *J* = 7.8 Hz, 1H), 7.23 (d, *J* = 8.6 Hz, 2H), 6.82 (d, *J* = 8.6 Hz, 2H), 1.60 (s, 6H); **^{13}C NMR** (75 MHz, Acentone-d_6) δ 175.9, 156.2, 140.3, 135.9, 130.2 (q, J_F = 31.8 Hz), 129.4, 127.2, 124.3 (q, J_F = 271.5 Hz), 123.0 (q, J_F = 1.2 Hz), 119.5 (q, J_F = 4.0 Hz), 116.1 (q, J_F = 4.1 Hz), 115.3, 47.1, 26.6; **HRMS**: calcd. for $C_{17}H_{16}F_3NO_2$ $[M-H]^-$ = 322.1034 m/z, found = 322.1046 m/z.

Compound S41:

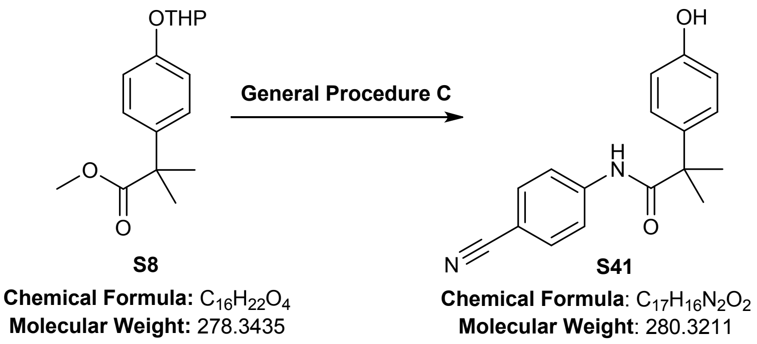

Procedure: The reaction was carried out according to the General Procedure C.

Amounts of Reagents:

S8 (3 mmol, 835.0 mg)

4-aminobenzonitrile (4.5 mmol, 531.2 mg)

NaHMDS (6 mL, 1M solution in THF)

THF (15 mL, 0.2 M)

oxalic acid (6 mmol, 540.2 mg)

MeOH/H_2O (27 mL/3 mL, 0.1 M)

Purification: 40% EtOAc in hexanes.

Yield of Product:

S41: 680.9 mg, 2.43 mmol, 81% yield, white solid.

Characterization:

R*f* = (EtOAc/Hexane 4:6): 0.44; **IR** (neat) ν =3366, 3316, 2230, 1706, 1665, 1589, 1316, 1182, 1176, 1140, 831, 757, 597, 540 cm^{-1}; **^{1}H NMR** (300 MHz, $CDCl_3$) δ 7.55 (d, *J* = 8.9 Hz, 2H), 7.48 (d, *J* = 8.9 Hz, 2H), 7.30 (d, *J* = 8.8 Hz, 2H), 6.96 (br. s, 1H), 6.88 (d, *J* = 8.8 Hz, 2H), 4.88 (br. s, 1H), 1.64 (s, 6H); **^{13}C NMR** (75 MHz, Acetone-d_6) δ 176.0, 156.2, 143.7, 135.6, 132.8, 127.2, 119.6, 118.6, 115.3, 105.9, 47.3, 26.5; **HRMS**: calcd. for $C_{17}H_{16}N_2O_2$ $[M+Na]^+$ = 303.1104 m/z, found = 303.1112 m/z.

Compound S42:

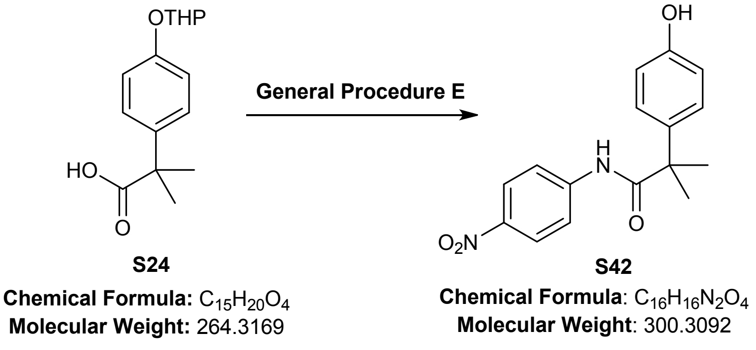

Procedure: The reaction was carried out according to the General Procedure E.

Amounts of Reagents:

S24 (3 mmol, 783.0 mg)

4-nitroaniline (3.6 mmol, 496.9 mg)

CDI (3.3 mmol, 535.1 mg)

DBU (3 mmol, 448.6 μL)

THF (15 mL, 0.2 M)

oxalic acid (6 mmol, 540.2 mg)

MeOH/H_2O (27 mL/3 mL, 0.1 M)

Purification: 50% EtOAc in hexanes.

Yield of Product:

S42: 360.4 mg, 1.20 mmol, 40% yield, white solid.

Characterization:

R*f* = (EtOAc/Hexane 1:1): 0.58; **IR** (neat) ν =3279, 2185, 1675, 1540, 1500, 1341, 1232, 1142, 856, 838, 751, 555 cm^{-1}; **^{1}H NMR** (400 MHz, Acetone-d_6) δ 8.87 (br. s, 1H), 8.34 (br. s, 1H), 8.16 (d, *J* = 9.3 Hz, 2H), 7.88 (d, *J* = 9.2 Hz, 2H), 7.21 (d, *J* = 8.7 Hz, 2H), 6.82 (d, *J* = 8.7 Hz, 2H), 1.60 (s, 6H); **^{13}C NMR** (75 MHz, Acetone-d_6) δ 176.2, 156.3, 145.6, 142.8, 135.5, 127.2, 124.5, 119.1, 115.3, 47.3, 26.4; **HRMS**: calcd. for $C_{16}H_{16}N_2O_4$ $[M-H]^-$ = 299.1037 m/z, found = 299.1038m/z.

Compound S43:

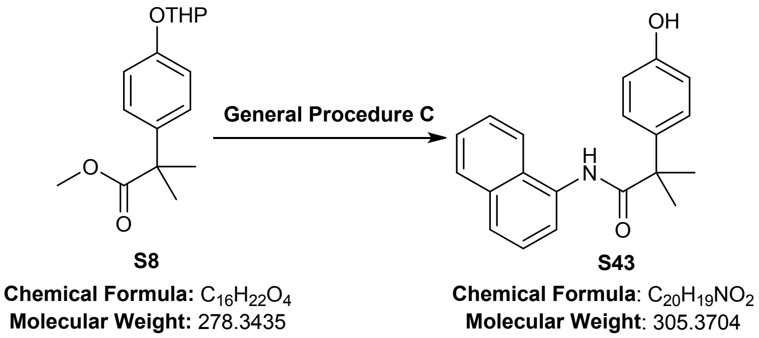

Procedure: The reaction was carried out according to the General Procedure C.

Amounts of Reagents:

S8 (3 mmol, 835.0 mg)

naphthalen-1-amine (4.5 mmol, 643.8 mg)

NaHMDS (6 mL, 1M solution in THF)

THF (15 mL, 0.2 M)

oxalic acid (6 mmol, 540.2 mg)

MeOH/H_2O (27 mL/3 mL, 0.1 M)

Purification: 30% EtOAc in hexanes.

Yield of Product:

S43: 604.6 mg, 1.98 mmol, 66% yield, white solid.

Characterization:

R*f* = (EtOAc/Hexane 3:7): 0.38; **IR** (neat) ν =3402, 3275, 2977, 2182, 1975, 1864, 1513, 1265, 1215, 1149, 834, 794, 561, 534 cm^{-1}; **^{1}H NMR** (300 MHz, Acentone-d_6) δ 8.36 (br. s, 1H), 8.19 (br. s, 1H), 7.87 (d, *J* = 8.1 Hz, 1H), 7.72 (dd, *J* = 7.7, 5.9 Hz, 2H), 7.57– 7.32 (m, 6H), 6.92 (d, *J* = 8.8 Hz, 2H), 1.71 (s, 6H); **^{13}C NMR** (75 MHz, Acentone-d_6) δ 175.8, 156.3, 136.3, 134.1, 133.8, 128.3, 128.2, 127.6, 125.71, 125.67, 125.4, 125.2, 121.7, 121.4, 115.4, 46.8, 26.6; **HRMS**: calcd. for $C_{20}H_{19}NO_2$ $[M-H]^-$ = 304.1343 m/z, found = 304.1337 m/z.

Compound S44:

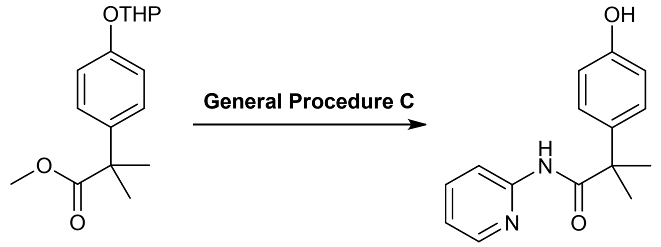

S8

Chemical Formula: $C_{16}H_{22}O_4$
Molecular Weight: 278.3435

S44

Chemical Formula: $C_{15}H_{16}N_2O_2$
Molecular Weight: 256.2997

Procedure: The reaction was carried out according to the General Procedure C.

Amounts of Reagents:

S8 (3 mmol, 835.0 mg)

2-aminopyridine (4.5 mmol, 432.2 mg)

NaHMDS (6 mL, 1M solution in THF)

THF (15 mL, 0.2 M)

oxalic acid (6 mmol, 540.2 mg)

MeOH/H_2O (27 mL/3 mL, 0.1 M)

Purification: 30% EtOAc in hexanes.

Yield of Product:

S44: 653.6 mg, 2.55 mmol, 85% yield, white solid.

Characterization:

R*f* = (EtOAc/hexane 1:1): 0.47; **IR** (neat) v = 3363, 2977, 1689, 1592, 1513, 1448, 1234, 1141, 1094, 1003, 833, 748, 632, 522 cm^{-1}; **^{1}H NMR** (300 MHz, Acentone-*d_6*) δ 8.39 (d, *J* = 4.5 Hz, 1H), 8.18 (d, *J* = 8.5 Hz, 1H), 8.14 (d, *J* = 4.0 Hz, 1H), 7.93 (br. s, 1H), 7.74 (td, *J* = 7.9, 1.9 Hz, 1H), 7.32 (d, *J* = 8.7 Hz, 2H), 7.02 (dd, *J* = 7.3, 4.9 Hz, 1H), 6.87 (d, *J* = 8.6 Hz, 2H), 1.63 (s, 6H); **^{13}C NMR** (75 MHz, Acentone-*d_6*) δ 175.7, 156.4, 152.0, 147.9, 137.9, 135.3, 127.5, 119.3, 115.5, 113.0, 47.1, 26.3; **HRMS**: calcd. for $C_{15}H_{16}N_2O_2$ $[M+H]^+$ = 257.1285 m/z, found = 257.1291 m/z.

Compound S45:

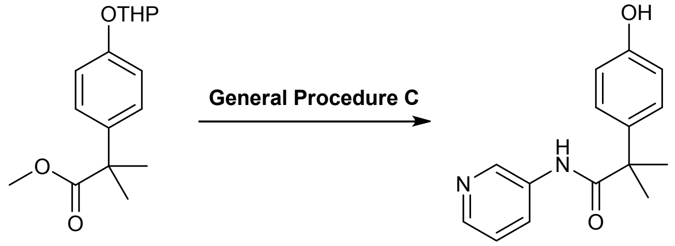

S8

Chemical Formula: $C_{16}H_{22}O_4$
Molecular Weight: 278.3435

S45

Chemical Formula: $C_{15}H_{16}N_2O_2$
Molecular Weight: 256.2997

Procedure: The reaction was carried out according to the General Procedure C.

Amounts of Reagents:

S8 (3 mmol, 835.0 mg)

3-aminopyridine (4.5 mmol, 432.2 mg)

NaHMDS (6 mL, 1M solution in THF)

THF (15 mL, 0.2 M)

oxalic acid (6 mmol, 540.2 mg)

MeOH/H_2O (27 mL/3 mL, 0.1 M)

Purification: EtOAc.

Yield of Product:

S45: 615.1 mg, 2.40 mmol, 80% yield, white solid.

Characterization:

R_f = (EtOAc): 0.40; **IR** (neat) ν = 3306, 1644, 1581, 1400, 1283, 1178, 1132, 1051, 892, 688, 584, 529 cm^{-1}; **^{1}H NMR** (300 MHz, Acentone-d_6) δ 8.69 (d, *J* = 2.1 Hz, 1H), 8.47 (br. s, 1H), 8.32 (s, 1H), 8.22 (d, *J* = 4.2 Hz, 1H), 8.07 (d, *J* = 8.2 Hz, 1H), 7.30 – 7.16 (m, 3H), 6.81 (d, *J* = 8.7 Hz, 2H), 1.59 (s, 6H); **^{13}C NMR** (75 MHz, Acentone-d_6) δ 175.9, 156.2, 144.2, 141.6, 136.1, 135.9, 127.2, 126.5, 123.1, 115.2, 46.9, 26.6; **HRMS**: calcd. for $C_{15}H_{16}N_2O_2$ $[M+H]^+$ = 257.1285 m/z, found = 257.1292 m/z.

Compound S46:

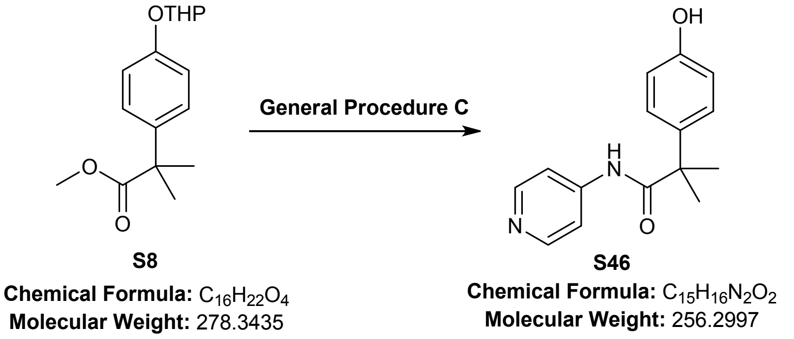

Procedure: The reaction was carried out according to the General Procedure C.

Amounts of Reagents:

S8 (3 mmol, 835.0 mg)

4-aminopyridine (4.5 mmol, 432.2 mg)

NaHMDS (6 mL, 1M solution in THF)

THF (15 mL, 0.2 M)

oxalic acid (6 mmol, 540.2 mg)

MeOH/H_2O (27 mL/3 mL, 0.1 M)

Purification: EtOAc.

Yield of Product:

S46: 622.8 mg, 2.43 mmol, 81% yield, white solid.

Characterization:

R_f = (EtOAc): 0.50; **IR** (neat) ν =3310, 2963, 2737, 1843, 1562m 1500, 1417, 1326, 1253, 1140, 1011, 884, 542 cm^{-1}; **^{1}H NMR** (300 MHz, Acentone-d_6) δ 8.69 (br. s, 2H), 8.36 (d, *J* = 5.6 Hz, 2H), 7.60 (d, *J* = 6.2 Hz, 2H), 7.19 (d, *J* = 8.7 Hz, 2H), 6.81 (d, *J* = 8.7 Hz, 2H), 1.58 (s, 6H); **^{13}C NMR** (75 MHz, Acentone-d_6) δ 176.5, 156.4, 150.0, 146.4, 135.4, 127.2, 115.3, 113.5, 47.3, 26.5; **HRMS**: calcd. for $C_{15}H_{16}N_2O_2$ $[M+H]^+$ = 257.1285 m/z, found = 257.1286 m/z.

Compound S47:

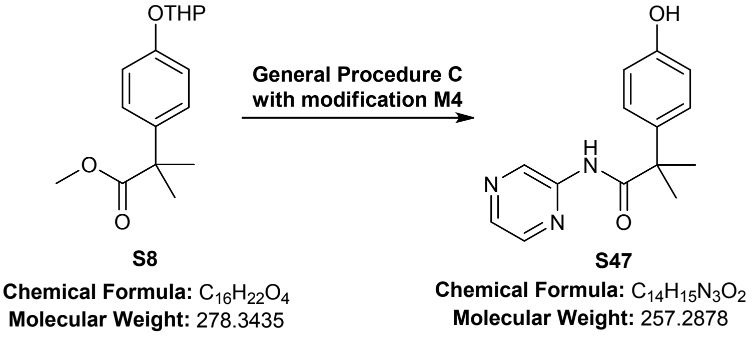

Procedure: The reaction was carried out according to the General Procedure C with modification M4.

Amounts of Reagents:

S8 (3 mmol, 835.0 mg)

2-aminopyrazine (4.5 mmol, 428.0 mg)

NaHMDS (6 mL, 1M solution in THF)

THF (15 mL, 0.2 M)

oxalic acid (6 mmol, 540.2 mg)

MeOH/H_2O (27 mL/3 mL, 0.1 M)

Purification: 50% EtOAc in hexanes.

Yield of Product:

S47: 609.8 mg, 2.37 mmol, 79% yield, white solid.

Characterization:

R_f = (EtOAc/hexane 1:1): 0.25; **IR** (neat) v = 3319, 3244, 2981, 1679, 1668, 1610, 1588, 1515, 1498, 1407, 1293, 1213, 1169, 1151, 1100, 1067, 1014, 836, 816, 676, 549 cm^{-1}; **^{1}H NMR** (400 MHz, Acentone-*d_6*) δ 9.44 (d, *J* = 1.5 Hz, 1H), 8.37 (br. s, 1H), 8.29 (br. s, 1H), 8.28 (d, *J* = 2.5 Hz, 1H), 8.20 (dd, *J* = 2.5, 1.6 Hz, 1H), 7.31 (d, *J* = 8.8 Hz, 2H), 6.86 (d, *J* = 8.8 Hz, 2H), 1.65 (s, 6H); **^{13}C NMR** (75 MHz, Acentone-*d_6*) δ 176.0, 156.5, 148.8, 142.2, 139.8, 136.4, 135.1, 127.4, 115.5, 47.0, 26.3; **HRMS**: calcd. for $C_{14}H_{15}N_3O_2$ $[M+H]^+$ = 258.1237 m/z, found = 258.1234 m/z.

Compound S48:

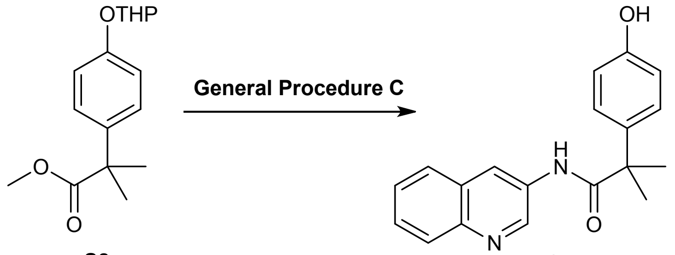

S8
Chemical Formula: $C_{16}H_{22}O_4$
Molecular Weight: 278.3435

S48
Chemical Formula: $C_{19}H_{18}N_2O_2$
Molecular Weight: 306.3584

Procedure: The reaction was carried out according to the General Procedure C.

Amounts of Reagents:

S8 (3 mmol, 835.0 mg)

3-aminoquinolin (4.5 mmol, 648.3 mg)

NaHMDS (6 mL, 1M solution in THF)

THF (15 mL, 0.2 M)

oxalic acid (6 mmol, 540.2 mg)

MeOH/H_2O (27 mL/3 mL, 0.1 M)

Purification: 50% EtOAc in hexanes.

Yield of Product:

S48: 726.1 mg, 2.37 mmol, 79% yield, white solid.

Characterization:

R_f = (EtOAc/hexane 1:1): 0.45; **IR** (neat) v = 3363, 2931, 1679, 1538, 1512, 1490, 1463, 1362, 1252, 1183, 1146, 917, 826, 784, 757, 650, 620 cm^{-1}; **^{1}H NMR** (300 MHz, Acentone-*d_6*) δ 8.86 (d, *J* = 2.5 Hz, 1H), 8.76 (br. s, 1H), 8.72 (d, *J* = 2.3 Hz, 1H), 8.41 (s, 1H), 7.94 (dd, *J* = 8.4, 1.1 Hz, 1H), 7.87 (dd, d, *J* = 8.2, 1.2 Hz, 1H), 7.61 (ddd, *J* = 8.4, 6.9, 1.6 Hz, 1H), 7.54 (ddd, *J* = 7.9, 6.9, 1.4 Hz, 1H), 7.27 (d, *J* = 8.7 Hz, 2H), 6.83 (d, *J* = 8.7 Hz, 2H), 1.64 (s, 6H); **^{13}C NMR** (75 MHz, Acentone-*d_6*) δ 181.3, 161.3, 150.2, 149.9, 141.0, 138.3, 133.9, 133.3, 132.6, 132.3, 131.9, 128.0, 127.7, 120.4, 52.1, 31.7; **HRMS**: calcd. for $C_{19}H_{18}N_2O_2$ $[M+H]^+$ = 307.1411 m/z, found = 307.1450 m/z.

Compound S49:

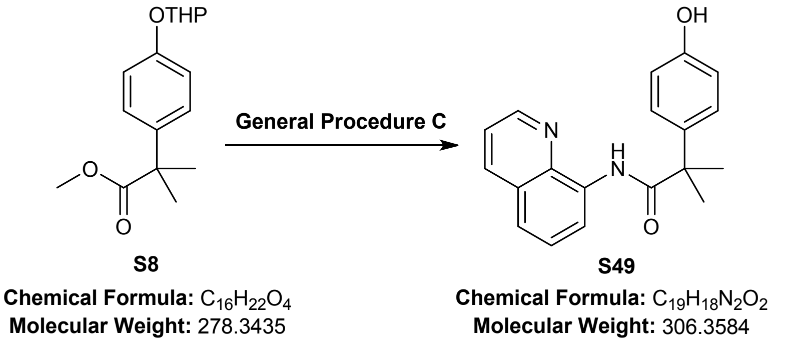

Procedure: The reaction was carried out according to the General Procedure C.

Amounts of Reagents:

S8 (3 mmol, 835.0 mg)

8-aminoquinolin (4.5 mmol, 648.3 mg)

NaHMDS (6 mL, 1M solution in THF)

THF (15 mL, 0.2 M)

oxalic acid (6 mmol, 540.2 mg)

MeOH/H_2O (27 mL/3 mL, 0.1 M)

Purification: 30% EtOAc in hexanes.

Yield of Product:

S49: 790.4 mg, 2.58 mmol, 86% yield, yellow solid.

Characterization:

$\mathbf{R_f}$ = (EtOAc/hexane 1:2): 0.48; **IR** (neat) ν = 3271, 2979, 1648, 1612, 1527, 1513, 1481, 1424, 1327, 1269, 1221, 1178, 1158, 825, 793, 758, 732, 680, 557 cm^{-1}; **^{1}H NMR** (400 MHz, Acentone-d_6) δ 9.83 (br. s, 1H), 8.76 (dd, J = 6.3, 2.6 Hz, 1H), 8.67 (dd, J = 4.2, 1.6 Hz, 1H), 8.36 (br. s, 1H), 8.27 (dd, J = 8.3, 1.5 Hz, 1H), 7.58 – 7.52 (m, 2H), 7.50 (dd, J = 8.3, 4.2 Hz, 1H), 7.39 (d, J = 8.7 Hz, 2H), 6.89 (d, J = 8.7 Hz, 2H), 1.69 (s, 6H); **^{13}C NMR** (75 MHz, Acentone-d_6) δ 175.4, 156.4, 148.4, 138.3, 136.3, 135.8, 135.0, 128.0, 127.4, 127.0, 121.8, 121.1, 115.5, 115.2, 47.4, 26.6; **HRMS**: calcd. for $C_{19}H_{18}N_2O_2$ $[M+Na]^+$ = 329.1260 m/z, found = 329.1259 m/z.

Compound S50:

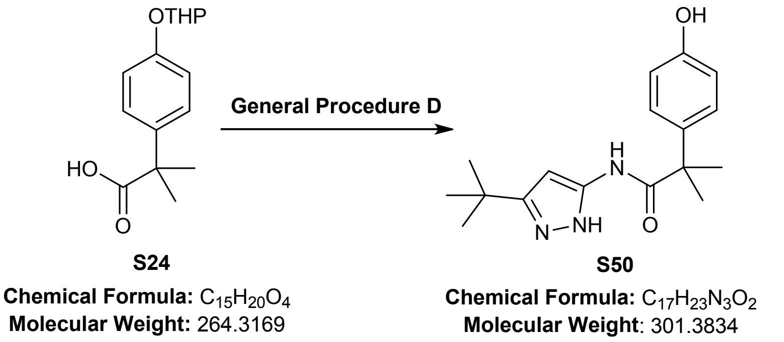

Procedure: The reaction was carried out according to the General Procedure D.

Amounts of Reagents:

S24 (3 mmol, 783.0 mg)

3-(*tert*-butyl)-1*H*-pyrazol-5-amine (3.6 mmol, 500.8 mg)

EDC (4.5 mmol, 698.6 mg)

HOBt (4.5 mmol, 608.0 mg)

N-methylmorpholine (30 mmol, 3.3 mL)
THF (15 mL, 0.2 M)
oxalic acid (6 mmol, 540.2 mg)
MeOH/H_2O (27 mL/3 mL, 0.1 M)
Purification: EtOAc.
Yield of Product:
S50: 397.9 mg, 1.32 mmol, 44% yield, white solid.
Characterization:
R*f* = (EtOAc): 0.28; **IR** (neat) v = 3243, 2968, 1667, 1535, 1513, 1474, 1457, 1362, 1246, 1177, 1148, 1097, 1020, 999, 831, 776, 747, 726, 671, 554 cm^{-1}; **^{1}H NMR** (300 MHz, Acentone-*d*$_6$) δ 11.05 (br. s, 1H), 8.30 (br. s, 1H), 8.11 (br. s, 1H), 7.26 (d, *J* = 8.7 Hz, 2H), 6.82 (d, *J* = 8.7 Hz, 2H), 6.39 (s, 1H), 1.59 (s, 6H), 1.30 (s, 9H); **^{13}C NMR** (75 MHz, DMSO-*d*$_6$) δ 174.6, 156.2, 153.1 (br.), 147.1 (br.), 136.2, 127.4, 115.3, 93.1, 46.3, 31.1, 30.4, 27.2; **HRMS**: calcd. for $C_{17}H_{23}N_3O_2$ $[M+Na]^+$ = 324.1682 m/z, found = 324.1682 m/z.

iii. Substrates in Table 3

Compound S51:

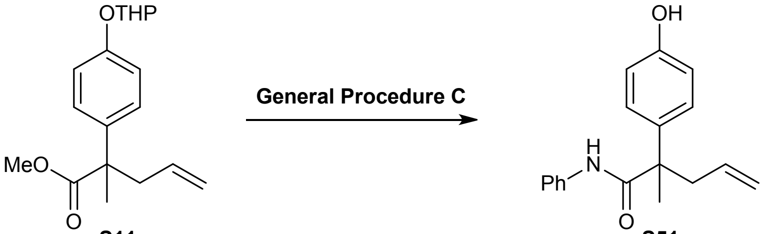

S11
Chemical Formula: $C_{18}H_{24}O_4$
Molecular Weight: 304.3808

S51
Chemical Formula: $C_{18}H_{19}NO_2$
Molecular Weight: 281.3490

Procedure: The reaction was carried out according to the General Procedure C on 2 mmol scale.
Amounts of Reagents:
S11 (2 mmol, 608.4 mg)
aniline (3 mmol, 273.5 μL)
NaHMDS (4 mL, 1M solution in THF)
THF (10 mL, 0.2 M)
oxalic acid (4 mmol, 360.1 mg)
MeOH/H_2O (18 mL/2 mL, 0.1 M)
Purification: 25% EtOAc in hexanes.
Yield of Product:
S51: 540.1 mg, 1.92 mmol, 96% yield, white solid.
Characterization:
R*f* = (ethyl acetate/hexane 1:2): 0.31; **IR** (neat) v = 3308, 3240, 3066, 3020, 2980, 2934, 1644, 1597, 1540, 1513, 1500, 1437, 1364, 1318, 1240, 1229, 1180, 996, 917, 825, 751, 691 cm^{-1}; **^{1}H NMR** (400 MHz, acetone-*d*$_6$) δ 8.32 (br. s, 1H), 8.27 (br. s, 1H), 7.58 (d, *J* = 7.6 Hz, 2H), 7.27 – 7.18 (m, 4H), 7.01 (t, *J* = 7.4 Hz, 1H), 6.82 (d, *J* = 8.8 Hz, 2H), 5.66 (ddt, *J* = 17.3, 10.2, 7.2 Hz, 1H), 5.07 (ddt, *J* = 17.1, 2.4, 1.3 Hz, 1H), 4.99 (d, *J* = 10.2 Hz, 1H), 2.86 (dd, *J* = 13.9, 7.4 Hz, 1H), 2.68 (dd, *J* = 13.9, 7.0 Hz, 1H), 1.57 (s, 3H); **^{13}C NMR** (75 MHz, acetone-*d*$_6$) δ 174.4, 156.1, 139.4, 135.0, 134.8, 128.4, 127.6, 123.2, 119.8, 117.3, 115.2, 50.0, 43.9, 22.8; **HRMS**: Calcd. for $C_{18}H_{19}NO_2$ $[M+Na]^+$ = 304.1308 m/z, found = 304.1307 m/z.

Compound S52:

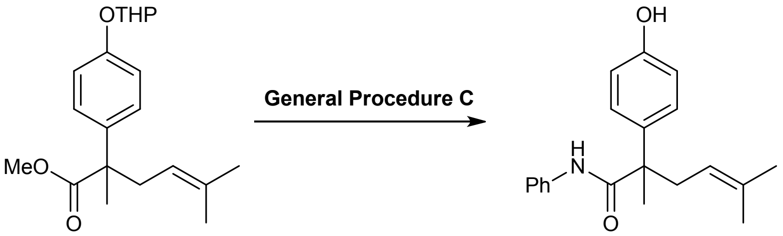

S12
Chemical Formula: $C_{20}H_{28}O_4$
Molecular Weight: 332.4339

S52
Chemical Formula: $C_{20}H_{23}NO_2$
Molecular Weight: 309.4021

Procedure: The reaction was carried out according to the General Procedure C on 1.5 mmol scale.

Amounts of Reagents:

S12 (1.5 mmol, 498.6 mg)

aniline (2.25 mmol, 205.1 μL)

NaHMDS (3 mL, 1M solution in THF)

THF (7.5 mL, 0.2 M)

oxalic acid (3 mmol, 270.1 mg)

MeOH/H_2O (13.5 mL/1.5 mL, 0.1 M)

Purification: 25% EtOAc in hexanes.

Yield of Product:

S52: 421.1 mg, 1.36 mmol, 91% yield, white solid.

Characterization:

R_f = (ethyl acetate/hexane 1:3): 0.43; **IR** (neat) ν = 3302, 3174, 2970, 2928, 1650, 1598, 1538, 1514, 1498, 1439, 1373, 1322, 1264, 1243, 1180, 912, 826, 754, 713, 690 cm^{-1}; **^{1}H NMR** (400 MHz, acetone-d_6) δ 8.28 (br. s, 1H), 8.22 (br. s, 1H), 7.60 (dd, J = 8.6, 1.0 Hz, 2H), 7.31 – 7.17 (m, 4H), 7.02 (t, J = 7.4 Hz, 1H), 6.83 (d, J = 8.7 Hz, 2H), 5.08 (t, J = 7.3 Hz, 1H), 2.84 (dd, J = 14.6, 7.5 Hz, 1H), 2.66 (dd, J = 14.5, 7.2 Hz, 1H), 1.65 (d, J = 0.7 Hz, 3H), 1.59 (s, 3H), 1.55 (s, 3H); **^{13}C NMR** (75 MHz, acetone-d_6) δ 174.6, 156.0, 139.5, 135.1, 133.5, 128.4, 127.7, 123.1, 120.5, 119.7, 115.1, 50.8, 37.5, 25.3, 23.2, 17.2; **HRMS**: Calcd. for $C_{20}H_{23}NO_2$ $[M+Na]^+$ = 332.1621 m/z, found = 332.1622 m/z.

Compound S53:

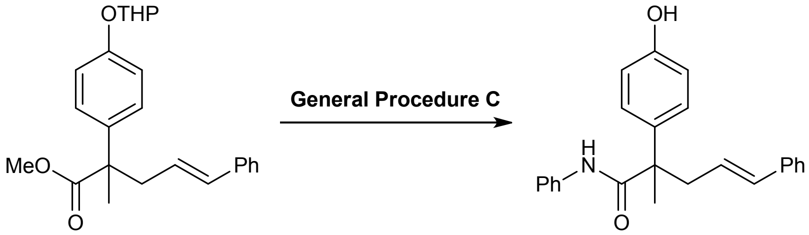

S13
Chemical Formula: $C_{24}H_{28}O_4$
Molecular Weight: 380.4767

S53
Chemical Formula: $C_{24}H_{23}NO_2$
Molecular Weight: 357.4449

Procedure: The reaction was carried out according to the General Procedure C on 2 mmol scale.

Amounts of Reagents:

S13 (2 mmol, 761.0 mg)

aniline (3 mmol, 273.5 μL)

NaHMDS (4 mL, 1M solution in THF)

THF (10 mL, 0.2 M)

oxalic acid (4 mmol, 360.1 mg)

MeOH/H_2O (18 mL/2 mL, 0.1 M)

Purification: 25% EtOAc in hexanes.

Yield of Product:

S53: 562.1 mg, 1.57 mmol, 79% yield, white solid.

Characterization:

R$_f$ = (ethyl acetate/hexane 1:2): 0.46; **IR** (neat) v = 3322, 3249, 1652, 1598, 1540, 1501, 1438, 1319, 1248, 1225, 1179, 1119, 977, 963, 825, 750, 693 cm^{-1}; **^{1}H NMR** (400 MHz, acetone-d_6) δ 8.37 (br. s, 1H), 8.32 (br. s, 1H), 7.62 (d, *J* = 7.9 Hz, 2H), 7.38 – 7.22 (m, 8H), 7.18 (t, *J* = 7.0 Hz, 1H), 7.04 (t, *J* = 7.3 Hz, 1H), 6.87 (t, *J* = 9.1 Hz, 2H), 6.49 (d, *J* = 15.8 Hz, 1H), 6.25 – 6.08 (m, 1H), 3.05 (dd, *J* = 13.8, 7.5 Hz, 1H), 2.88 (dd, *J* = 13.8, 7.1 Hz, 1H), 1.67 (s, 3H); **^{13}C NMR** (75 MHz, acetone-d_6) δ 174.6, 156.2, 139.4, 137.7, 134.7, 133.0, 128.5, 128.4, 127.8, 127.0, 126.7, 126.0, 123.4, 120.0, 115.3, 50.6, 43.2, 23.0; **HRMS**: Calcd. for $C_{24}H_{23}NO_2$ [M+H]$^+$ = 358.1802 m/z, found = 358.1797 m/z.

Compound S54:

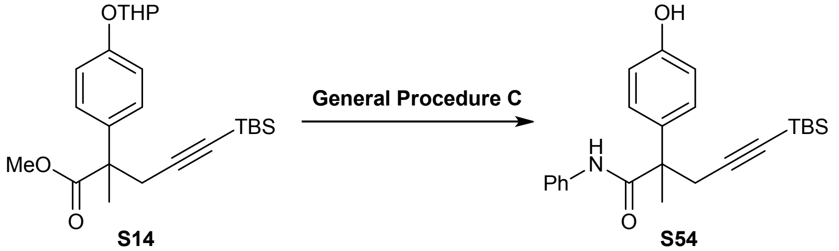

S14
Chemical Formula: $C_{24}H_{36}O_4Si$
Molecular Weight: 416.6257

S54
Chemical Formula: $C_{24}H_{31}NO_2Si$
Molecular Weight: 393.5939

Procedure: The reaction was carried out according to the General Procedure C on 3.3 mmol scale.

Amounts of Reagents:

S14 (3.3 mmol, 1.37 g)

aniline (4.95 mmol, 451.2 μL)

NaHMDS (6.6 mL, 1M solution in THF)

THF (16.5 mL, 0.2 M)

oxalic acid (6.6 mmol, 594.3 mg)

MeOH/H_2O (29.7 mL/3.3 mL, 0.1 M)

Purification: 20% EtOAc in hexanes.

Yield of Product:

S54: 600.7 mg, 1.53 mmol, 46% yield, white solid.

Characterization:

R$_f$ = (ethyl acetate/hexane 1:3): 0.54; **IR** (neat) v = 3384, 3351, 2950, 2929, 2856, 2173, 1653, 1591, 1540, 1516, 1442, 1326, 1247, 1216, 1179, 1041, 824, 774, 747, 692, 680, 598 cm^{-1}; **^{1}H NMR** (400 MHz, acetone-d_6) δ 8.37 (br. s, 1H), 8.32 (br. s, 1H), 7.58 (dd, *J* = 8.6, 1.0 Hz, 2H), 7.29 – 7.21 (m, 4H), 7.02 (t, *J* = 7.4 Hz, 1H), 6.82 (d, *J* = 8.7 Hz, 2H), 3.02 (d, *J* = 17.0 Hz, 1H), 2.89 (d, *J* = 17.0 Hz, 1H), 1.77 (s, 3H), 0.85 (s, 9H), 0.01 (s, 3H), 0.00 (s, 3H); **^{13}C NMR** (75 MHz, acetone-d_6) δ 173.5, 156.4, 139.3, 133.4, 128.4, 127.8, 123.3, 119.8, 115.1, 105.3, 84.6, 50.2, 31.1, 25.5, 22.8, 16.1, -5.2; **HRMS**: Calcd. for $C_{24}H_{31}NO_2Si$ [M+Na]$^+$ = 416.2016 m/z, found = 416.2021 m/z.

Compound S55:

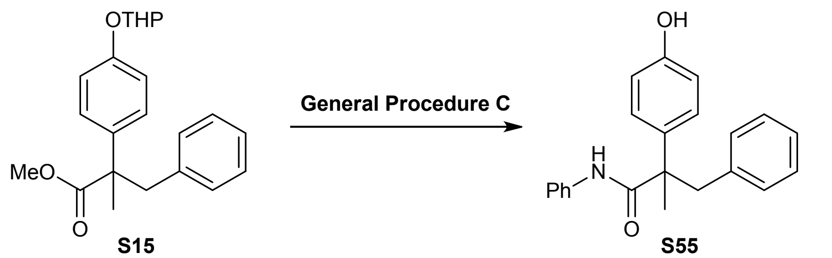

S15 **S55**

Chemical Formula: $C_{22}H_{26}O_4$ **Chemical Formula:** $C_{22}H_{21}NO_2$

Molecular Weight: 354.4394 **Molecular Weight:** 331.4076

Procedure: The reaction was carried out according to the General Procedure C on 2 mmol scale.

Amounts of Reagents:

S15 (2 mmol, 708.5 mg)

aniline (3 mmol, 273.5 μL)

NaHMDS (4 mL, 1M solution in THF)

THF (10 mL, 0.2 M)

oxalic acid (4 mmol, 360.1 mg)

MeOH/H_2O (18 mL/2 mL, 0.1 M)

Purification: 25% EtOAc in hexanes.

Yield of Product:

S55: 578.4 mg, 1.75 mmol, 87% yield, white solid.

Characterization:

$\mathbf{R_f}$ = (ethyl acetate/hexane 1:3): 0.32; **IR** (neat) ν = 3318, 3234, 3025, 2974, 2955, 1643, 1597, 1540, 1512, 1490, 1437, 1371, 1313, 1243, 1180, 1115, 943, 926, 827, 754, 745, 702, 690, 661 cm^{-1}; **^{1}H NMR** (400 MHz, acetone-d_6) δ 8.35 (br. s, 2H), 7.62 (dd, J = 8.5, 0.9 Hz, 2H), 7.27 (t, J = 8.0 Hz, 2H), 7.20 – 7.10 (m, 5H), 7.04 (t, J = 7.4 Hz, 1H), 6.99 – 6.91 (m, 2H), 6.82 (d, J = 8.7 Hz, 2H), 3.48 (d, J = 13.3 Hz, 1H), 3.24 (d, J = 13.3 Hz, 1H), 1.54 (s, 3H); **^{13}C NMR** (75 MHz, acetone-d_6) δ 174.7, 156.2, 139.4, 138.2, 134.5, 130.7, 128.4, 128.1, 127.4, 126.0, 123.3, 119.9, 115.0, 51.4, 45.2, 22.3; **HRMS**: Calcd. for $C_{22}H_{21}NO_2$ $[M+Na]^+$ = 354.1464 m/z, found = 354.1468 m/z.

Compound S56:

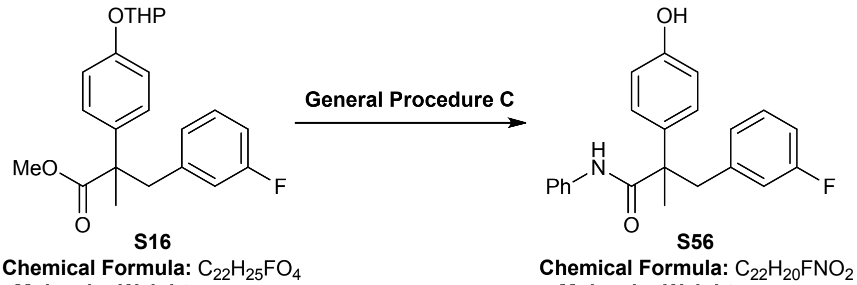

S16 **S56**

Chemical Formula: $C_{22}H_{25}FO_4$ **Chemical Formula:** $C_{22}H_{20}FNO_2$

Molecular Weight: 372.4299 **Molecular Weight:** 349.3981

Procedure: The reaction was carried out according to the General Procedure C on 4 mmol scale.

Amounts of Reagents:

S16 (4 mmol, 1.49 g)

aniline (6 mmol, 546.9 μL)

NaHMDS (8 mL, 1M solution in THF)

THF (20 mL, 0.2 M)

oxalic acid (8 mmol, 720.3 mg)

MeOH/H_2O (36 mL/4 mL, 0.1 M)

Purification: 25% EtOAc in hexanes.

Yield of Product:

S56: 1.02 g, 2.92 mmol, 73% yield, yellow solid.

Characterization:

R_f = (ethyl acetate/hexane 1:3): 0.25; **IR** (neat) ν = 3316, 3233, 3024, 2937, 1644, 1595, 1541, 1512, 1488, 1438, 1370, 1314, 1242, 1181, 1145, 922, 872, 827, 754, 691, 657 cm^{-1}; **^{1}H NMR** (400 MHz, acetone-*d_6*) δ 8.37 (br. s, 2H), 7.60 (d, *J* = 7.9 Hz, 2H), 7.26 (t, *J* = 7.9 Hz, 2H), 7.21 – 7.10 (m, 3H), 7.03 (t, *J* = 7.4 Hz, 1H), 6.90 (td, *J* = 8.6, 2.4 Hz, 1H), 6.80 (t, *J* = 8.9 Hz, 3H), 6.66 (d, *J* = 10.6 Hz, 1H), 3.46 (d, *J* = 13.3 Hz, 1H), 3.23 (d, *J* = 13.3 Hz, 1H), 1.55 (s, 3H); **^{13}C NMR** (75 MHz, acetone-*d_6*) δ 174.5, 162.2 (d, J_F = 242.7 Hz), 156.3, 141.2 (d, J_F = 7.5 Hz), 139.4, 134.1, 129.0 (d, J_F = 8.3 Hz), 128.4, 128.1, 126.7 (d, J_F = 2.7 Hz), 123.3, 119.9, 117.2 (d, J_F = 21.2 Hz), 115.1, 112.7 (d, J_F = 21.1 Hz), 51.3, 45.0, 22.0; **HRMS**: Calcd. for $C_{22}H_{20}FNO_2$ $[M+Na]^+$ = 372.1370 m/z, found = 372.1382 m/z.

Compound S57:

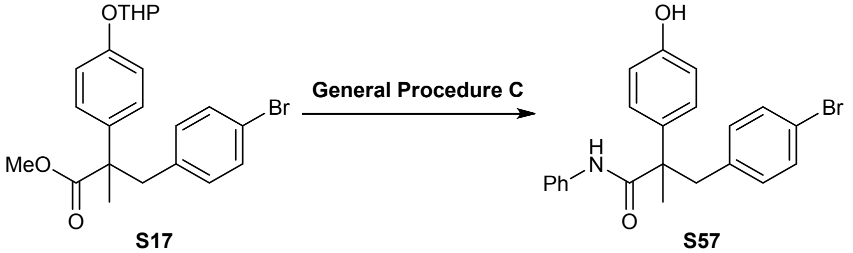

S17
Chemical Formula: $C_{22}H_{25}BrO_4$
Molecular Weight: 433.3355

S57
Chemical Formula: $C_{22}H_{20}BrNO_2$
Molecular Weight: 410.3037

Procedure: The reaction was carried out according to the General Procedure C on 2 mmol scale.

Amounts of Reagents:

S17 (2 mmol, 866.7 mg)

aniline (3 mmol, 273.5 μL)

NaHMDS (4 mL, 1M solution in THF)

THF (10 mL, 0.2 M)

oxalic acid (4 mmol, 360.1 mg)

MeOH/H_2O (18 mL/2 mL, 0.1 M)

Purification: 25% EtOAc in hexanes.

Yield of Product:

S57: 768.6 mg, 1.87 mmol, 94% yield, white solid.

Characterization:

R_f = (ethyl acetate/hexane 1:3): 0.32; **IR** (neat) ν = 3254, 1691, 1665, 1614, 1595, 1513, 1495, 1487, 1439, 1404, 1379, 1260, 1233, 1181, 1111, 1073, 1010, 837, 803, 751, 742, 693, 638 cm^{-1}; **^{1}H NMR** (400 MHz, acetone-*d_6*) ^{1}H NMR (500 MHz, Acetone) δ 8.39 (br. s, 1H), 8.38 (br. s, 1H), 7.61 (dd, *J* = 8.6, 1.0 Hz, 2H), 7.31 (d, *J* = 8.4 Hz, 2H), 7.27 (dd, *J* = 8.4, 7.6 Hz, 2H), 7.14 (d, *J* = 8.7 Hz, 2H), 7.05 (t, *J* = 7.4 Hz, 1H), 6.87 (d, *J* = 8.4 Hz, 2H), 6.83 (d, *J* = 8.7 Hz, 2H), 3.42 (d, *J* = 13.3 Hz, 1H), 3.22 (d, *J* = 13.3 Hz, 1H), 1.55 (s, 3H); **^{13}C NMR** (75 MHz, acetone-*d_6*) δ 174.6, 156.3, 139.4, 137.7, 134.0, 132.8, 130.4, 128.5, 128.2, 123.4, 119.9, 119.7, 115.1, 51.3, 44.7, 22.0; **HRMS**: Calcd. for $C_{22}H_{20}BrNO_2$ $[M+Na]^+$ = 432.0570 m/z, found = 432.0564 m/z.

Compound S58:

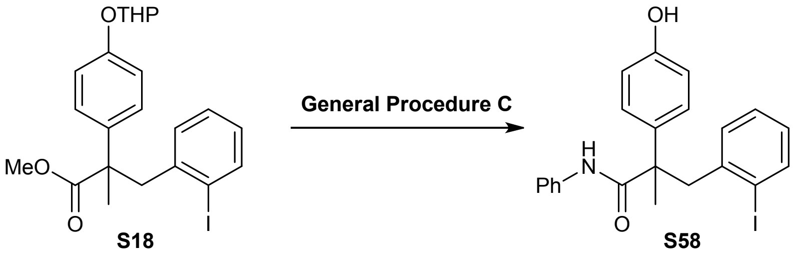

S18
Chemical Formula: $C_{22}H_{25}IO_4$
Molecular Weight: 480.3360

S58
Chemical Formula: $C_{22}H_{20}INO_2$
Molecular Weight: 457.3042

Procedure: The reaction was carried out according to the General Procedure C on 2 mmol scale.

Amounts of Reagents:

S18 (2 mmol, 960.7 mg)

aniline (3 mmol, 273.5 μL)

NaHMDS (4 mL, 1M solution in THF)

THF (10 mL, 0.2 M)

oxalic acid (4 mmol, 360.1 mg)

MeOH/H_2O (18 mL/2 mL, 0.1 M)

Purification: 25% EtOAc in hexanes.

Yield of Product:

S58: 825.9 mg, 1.81 mmol, 90% yield, white solid.

Characterization:

R_f = (ethyl acetate/hexane 1:3): 0.28; **IR** (neat) ν = 3254, 1691, 1665, 1614, 1595, 1513, 1495, 1487, 1439, 1404, 1379, 1260, 1233, 1181, 1111, 1073, 1010, 837, 803, 751, 742, 693, 638 cm^{-1}; **^{1}H NMR** (400 MHz, acetone-d_6) δ 8.39 (br. s, 2H), 7.85 (d, J = 7.9 Hz, 1H), 7.62 (d, J = 8.1 Hz, 2H), 7.27 (t, J = 7.9 Hz, 2H), 7.16 (d, J = 8.6 Hz, 2H), 7.10 (t, J = 7.5 Hz, 1H), 7.04 (t, J = 7.4 Hz, 1H), 6.90 (t, J = 7.6 Hz, 1H), 6.83 (d, J = 8.6 Hz, 2H), 6.74 (dd, J = 7.7, 1.0 Hz, 1H), 3.85 (d, J = 13.9 Hz, 1H), 3.41 (d, J = 13.9 Hz, 1H), 1.61 (s, 3H); **^{13}C NMR** (75 MHz, acetone-d_6) δ 174.6, 156.4, 141.5, 139.5, 139.4, 134.2, 130.8, 128.4, 128.3, 128.2, 127.6, 123.4, 120.0, 115.2, 103.6, 52.3, 48.1, 21.4; **HRMS**: Calcd. for $C_{22}H_{20}INO_2$ $[M+Na]^+$ = 480.0431 m/z, found = 480.0423 m/z.

Compound S59:

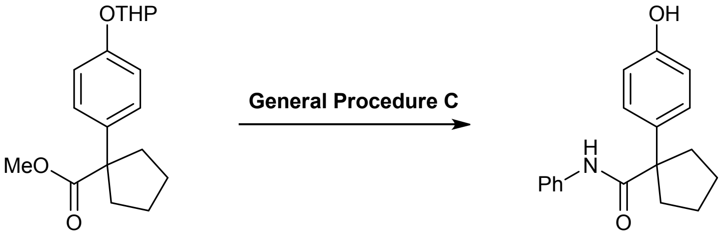

S9
Chemical Formula: $C_{18}H_{24}O_4$
Molecular Weight: 304.3808

S59
Chemical Formula: $C_{18}H_{19}NO_2$
Molecular Weight: 281.3490

Procedure: The reaction was carried out according to the General Procedure C on 2 mmol scale.

Amounts of Reagents:

S9 (2 mmol, 608.8 mg)

aniline (3 mmol, 273.5 μL)

NaHMDS (4 mL, 1M solution in THF)

THF (10 mL, 0.2 M)

oxalic acid (4 mmol, 360.1 mg)

MeOH/H_2O (18 mL/2 mL, 0.1 M)

Purification: 25% EtOAc in hexanes.

Yield of Product:

S59: 552.3 mg, 1.96 mmol, 98% yield, white solid.

Characterization:

R$_f$ = (ethyl acetate/hexane 1:2): 0.47; **IR** (neat) ν = 3396, 3334, 3165, 2946, 2872, 1633, 1597, 1526, 1511, 1496, 1439, 1316, 1230, 697, 828, 740, 687, 629 cm^{-1}; **^{1}H NMR** (400 MHz, acetone-*d$_6$*) δ 8.26 (br. s, 2H), 7.55 (d, *J* = 7.7 Hz, 2H), 7.29 – 7.18 (m, 4H), 6.99 (t, *J* = 7.4 Hz, 1H), 6.80 (d, *J* = 8.7 Hz, 2H), 2.68 – 2.54 (m, 2H), 2.02 – 1.92 (m, 2H), 1.80 – 1.66 (m, 4H); **^{13}C NMR** (75 MHz, acetone-*d$_6$*) δ 174.1, 156.1, 139.6, 134.9, 128.4, 127.7, 123.1, 119.6, 115.2, 59.7, 36.3, 23.5; **HRMS**: Calcd. for $C_{18}H_{19}NO_2$ [M+H]$^+$ = 282.1489 m/z, found = 282.1485 m/z.

Compound S60:

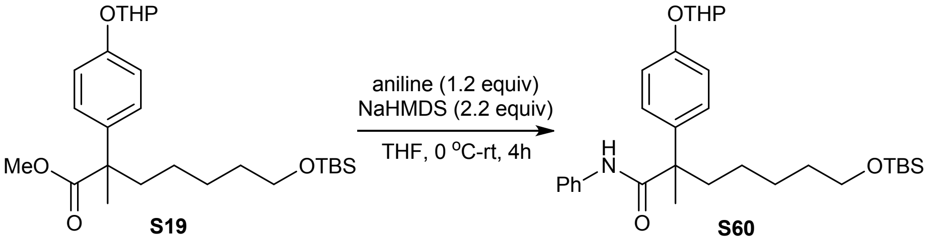

Chemical Formula: $C_{26}H_{44}O_5Si$
Molecular Weight: 464.7101

Chemical Formula: $C_{31}H_{47}NO_4Si$
Molecular Weight: 525.7947

Procedure: A flame-dried, 250 mL round-bottom flask equipped with a Teflon-coated stir bar and a rubber septum was charged with aniline (24.9 mmol, 2.32 mL, 1.2 equiv) and then purged with a steady stream of N_2 for 5 min, prior to the addition of dry and degassed THF (60 mL). Sodium bis(trimethylsilyl)amide (NaHMDS) (45.7 mmol, 45.7 mL of 1 M solution in THF, 2.2 equiv) was added dropwise at 0 °C and stirred for 20 min. In a separate, flame-dried, 5 mL round-bottom flask equipped with a Teflon-coated stir bar and a rubber septum was charged with compound **S19** (20.8 mmol, 9.65 g, 1 equiv) and then purged with a steady stream of N_2 for 5 min, prior to the addition of dry and degassed THF (20 mL) to afford a homogeneous solution. It was then added to the solution of the aniline and NaHMDS in THF dropwise via syringe at 0 °C. The mixture was then warmed to room temperature and stirred for 4h, quenched with saturated ammonium chloride (100 mL) and diluted with ethyl acetate (100 mL). The aqueous layer was separated and extracted with ethyl acetate (3 × 100 mL), and the combined organic layers were dried over $MgSO_4$, filtered and concentrated *in vacuo*. The crude product was then purified on silica gel using 10% ethyl acetate in hexanes as eluent to afford **S60** (9.86 g, 18.75 mmol, 90% yield) as a yellow oil.

Characterization:

R$_f$ = (ethyl acetate/hexane 1:10): 0.25; **IR** (neat) ν = 3405, 3339, 2929, 2855, 1666, 1597, 1509, 1436, 1387, 1358, 1309, 1240, 1202, 1096, 1037, 1021, 964, 920, 831, 774, 750, 691, 661 cm^{-1}; **^{1}H NMR** (400 MHz, acetone-*d$_6$*) δ 8.30 (br. s, 1H), 7.60 (d, *J* = 7.7 Hz, 2H), 7.29 (d, *J* = 7.9 Hz, 2H), 7.23 (t, *J* = 8.0 Hz, 2H), 7.05 – 6.98 (m, 3H), 5.43 (t, *J* = 3.2 Hz, 1H), 3.90 – 3.79 (m, 1H), 3.61 (td, *J* = 6.2, 1.2 Hz, 2H), 3.58 – 3.52 (m, 1H), 2.22 – 2.07 (m, 1H), 2.02 – 1.89 (m, 2H), 1.90 – 1.72 (m, 2H), 1.72 – 1.45 (m, 8H), 1.45 – 1.34 (m, 2H), 1.34 – 1.19 (m, 2H), 0.89 (s, 9H), 0.03 (s, 6H); **^{13}C NMR** (75 MHz, acetone-*d$_6$*) δ 174.6, 174.5, 155.9, 139.5, 137.9, 137.8, 128.4, 127.41, 127.39, 123.1, 119.8, 116.24, 116.22, 96.1, 62.6, 61.6, 50.53, 50.51, 39.2, 39.0, 32.5, 30.2, 26.3, 25.5, 25.1, 24.2, 24.2, 23.4, 23.2, 18.8, 17.9, -6.0 (***a complex mixture of diastereomers are observed in the ^{13}C NMR, which accounts for the presence of extra ^{13}C signals***); **HRMS**: Calcd. for $C_{31}H_{47}NO_4Si$ [M+H]$^+$ = 526.3347 m/z, found = 526.3357 m/z.

Compound S61:

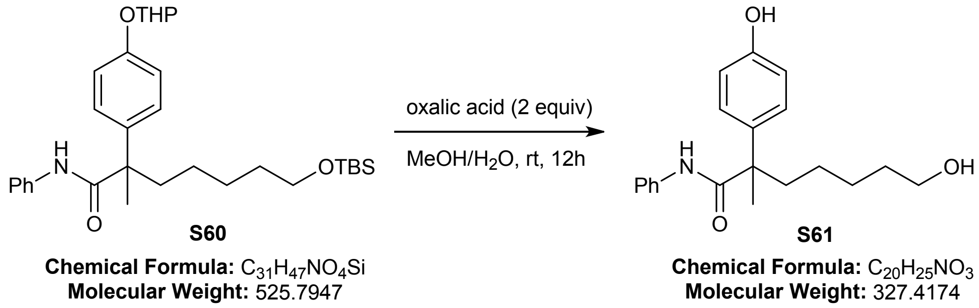

Procedure: A flame-dried, 50 mL round-bottom flask equipped with a Teflon-coated stir bar and a rubber septum was charged with compound **S60** (1.85 mmol, 972.7 mg, 1 equiv) and added methanol and water (9:1 by volume, 20 mL). Oxalic acid (3.7 mmol, 333.2 mg, 2 equiv) was then added as solid. After stirring for 12h, the mixture was concentrated *in vacuo*, diluted with ethyl acetate (40 mL) and washed with saturated sodium bicarbonate solution (40 mL). The aqueous layer was separated and extracted with ethyl acetate (3 × 40 mL), and the combined organic layers were dried over $MgSO_4$, filtered and concentrated *in vacuo*. The crude product was then purified on silica gel using 50% ethyl acetate in hexanes as eluent to afford **S61** (569.4 mg, 1.74 mmol, 94% yield) as a white foam.

Characterization:

$\mathbf{R_f}$ = (ethyl acetate/hexane 1:1): 0.18; **IR** (neat) ν = 3304, 2936, 2861, 1654, 1596, 1512, 1499, 1436, 1374, 1311, 1237, 1179, 1046, 831, 750, 690, 551 cm^{-1}; **^{1}H NMR** (400 MHz, acetone-d_6) δ 8.37 (br. s, 1H), 8.23 (br. s, 1H), 7.58 (dd, *J* = 8.6, 1.0 Hz, 2H), 7.32 – 7.15 (m, 4H), 7.01 (t, *J* = 7.4 Hz, 1H), 6.82 (d, *J* = 8.8 Hz, 2H), 3.59 (t, *J* = 5.0 Hz, 1H), 3.52 (dd, *J* = 11.5, 6.2 Hz, 2H), 2.18 – 2.08 (m, 1H), 2.01 – 1.90 (m, 1H), 1.57 (s, 3H), 1.55 – 1.45 (m, 2H), 1.45 – 1.33 (m, 2H), 1.33 – 1.18 (m, 2H); **^{13}C NMR** (75 MHz, acetone-d_6) δ 175.1, 156.0, 139.4, 135.6, 128.4, 127.6, 123.2, 119.9, 115.2, 61.6, 50.4, 39.1, 32.7, 26.4, 24.4, 23.4; **HRMS**: Calcd. for $C_{20}H_{25}NO_3$ $[M+Na]^+$ = 350.1727 m/z, found = 350.1721 m/z.

Compound S62:

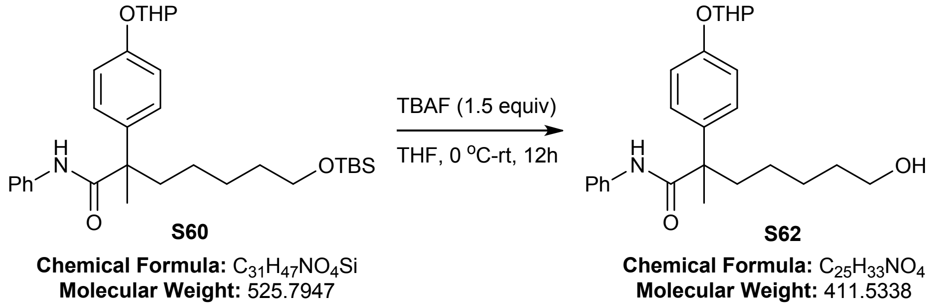

Procedure: A flame-dried, 250 mL round-bottom flask equipped with a Teflon-coated stir bar and a rubber septum was charged with compound **S60** (18.75 mmol, 9.86 g, 1 equiv) and then purged with a steady stream of N_2 for 5 min, prior to the addition of dry and degassed THF (100 mL). Tetra-*n*-butylammonium fluoride trihydrate (TBAF) (28.13 mmol, 8.87 g, 1.5 equiv) was then added portionwise as solid at 0 °C. The resulting solution was warmed up to room temperature and stirred for 12h, diluted with water (100 mL) and extracted with ethyl acetate (3 × 100 mL). The combined organic layers were dried over $MgSO_4$, filtered and concentrated *in vacuo*. The crude product was then purified on silica gel using 40% ethyl acetate in hexanes as eluent to afford **S62** (6.64 g, 16.13 mmol, 86% yield) as a white solid.

Characterization:

$\mathbf{R_f}$ = (ethyl acetate/hexane 1:2): 0.18; **IR** (neat) ν = 3399, 3332, 2937, 2861, 1660, 1598, 1508, 1500, 1437, 1389, 1310, 1238, 1202, 1179, 1109, 1074, 1035, 1022, 962, 920, 872, 830, 752, 692 cm^{-1}; **^{1}H NMR** (400 MHz, acetone-d_6) δ 8.30 (br. s, 1H), 7.60 (d, *J* = 7.7 Hz, 2H), 7.31 (dd, *J* = 8.8, 0.8 Hz, 2H), 7.25 (t, *J* = 7.5 Hz, 2H), 7.06 – 6.99 (m, 3H), 5.44 (t, *J* = 3.2 Hz, 1H), 3.90 – 3.81 (m, 1H), 3.62 – 3.54 (m, 1H), 3.52 (t, *J* = 6.4 Hz, 2H), 3.40 (br. s, 1H), 2.23 – 2.10 (m, 1H), 2.04 – 1.91 (m, 2H), 1.91 – 1.74 (m, 2H), 1.72 – 1.62 (m, 2H), 1.62 – 1.46 (m, 6H), 1.46 – 1.36 (m, 2H), 1.36 – 1.23 (m, 2H); **^{13}C NMR** (75

MHz, acetone-d_6) δ 174.62, 174.57, 155.8, 139.5, 138.0, 137.8, 128.4, 127.40, 127.38, 123.2, 119.8, 116.22, 116.20, 96.13, 96.12, 61.6, 61.5, 50.5, 39.1, 39.0, 32.8, 30.2, 26.4, 25.1, 24.40, 24.38, 23.4, 23.2, 18.8 (***a complex mixture of diastereomers are observed in the ^{13}C NMR, which accounts for the presence of extra ^{13}C signals***); **HRMS**: Calcd. for $C_{25}H_{33}NO_4$ $[M+Na]^+$ = 434.2302 m/z, found = 434.2298 m/z.

Compound S63:

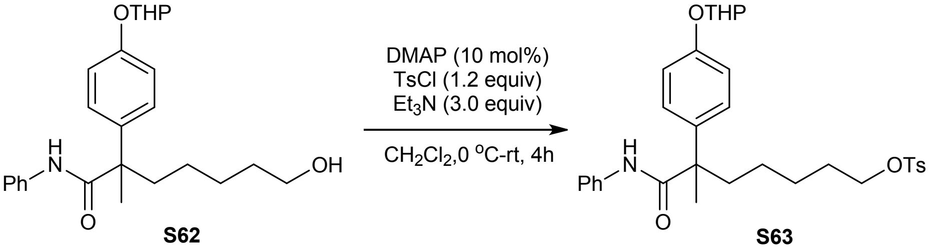

S62
Chemical Formula: $C_{25}H_{33}NO_4$
Molecular Weight: 411.5338

S63
Chemical Formula: $C_{32}H_{39}NO_6S$
Molecular Weight: 565.7202

Procedure: A flame-dried, 250 mL round-bottom flask equipped with a Teflon-coated stir bar and a rubber septum was charged with compound **S62** (5 mmol, 2.06 g, 1 equiv) and 4-(dimethylamino)pyridine (DMAP) (0.5 mmol, 61.1 mg, 10 mol%) and then purged with a steady stream of N_2 for 5 min, prior to the addition of dry and degassed CH_2Cl_2 (50 mL) and Et_3N (15 mmol, 2.09 mL, 3 equiv). In a separate, flame-dried, 50 mL round-bottom flask equipped with a Teflon-coated stir bar and a rubber septum was charged with *p*-toluenesulfonyl chloride (TsCl) (6 mmol, 1.14 g, 1.2 equiv) and then purged with a steady stream of N_2 for 5 min, prior to the addition of dry and degassed CH_2Cl_2 (20 mL) to afford a homogeneous solution. It was added to the reaction solution dropwise via syringe at 0 °C. The resulting solution was then warmed to room temperature and stirred for 4h, before quenching with saturated $NaHCO_3$ (50 mL). The aqueous layer was separated and extracted with CH_2Cl_2 (3 × 50 mL). The combined organic layers were dried over $MgSO_4$, filtered and concentrated *in vacuo*. The crude product was then purified on silica gel using 30% ethyl acetate in hexanes as eluent to afford **S63** (2.60 g, 4.60 mmol, 92% yield) as a white solid.

Characterization:

$\mathbf{R_f}$ = (ethyl acetate/hexane 1:2): 0.70; **IR** (neat) ν = 3337, 2941, 2862, 1662, 1599, 1528, 1511, 1492, 1436, 1358, 1243, 1172, 949, 940, 900, 827, 814, 763, 723, 698, 667, 583, 552 cm^{-1}; **^{1}H NMR** (400 MHz, acetone-d_6) δ 8.29 (br. s, 1H), 7.78 (d, *J* = 8.3 Hz, 2H), 7.59 (d, *J* = 8.5 Hz, 2H), 7.45 (d, *J* = 8.0 Hz, 2H), 7.31 – 7.18 (m, 4H), 7.01 (dt, *J* = 7.4, 2.9 Hz, 3H), 5.43 (t, *J* = 2.8 Hz, 1H), 4.02 (td, *J* = 6.3, 0.9 Hz, 2H), 3.90 – 3.78 (m, 1H), 3.61 – 3.50 (m, 1H), 2.44 (s, 3H), 2.12 – 2.01 (m, 1H), 1.98 – 1.73 (m, 4H), 1.70 – 1.49 (m, 8H), 1.35 – 1.26 (m, 2H), 1.26 – 1.07 (m, 2H); **^{13}C NMR** (75 MHz, acetone-d_6) δ 174.50, 174.45, 155.8, 144.8, 139.5, 137.8, 137.6, 133.6, 130.0, 128.4, 127.7, 127.4, 123.2, 119.8, 116.3, 116.2, 96.14, 96.12, 70.6, 61.6, 50.4, 38.9, 38.7, 30.2, 28.4, 25.7, 25.1, 23.8, 23.3, 23.1, 20.7, 18.8 (***a complex mixture of diastereomers are observed in the ^{13}C NMR, which accounts for the presence of extra ^{13}C signals***); **HRMS**: Calcd. for $C_{32}H_{39}NO_6S$ $[M+Na]^+$ = 588.2390 m/z, found = 588.2385 m/z.

Compound S64:

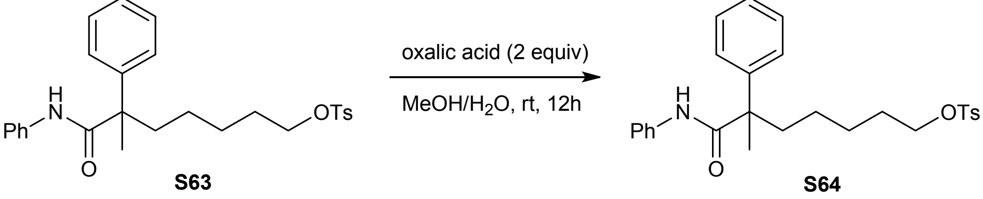

S63
Chemical Formula: $C_{32}H_{39}NO_6S$
Molecular Weight: 565.7202

S64
Chemical Formula: $C_{27}H_{31}NO_5S$
Molecular Weight: 481.6037

Procedure: A flame-dried, 50 mL round-bottom flask equipped with a Teflon-coated stir bar and a rubber septum was

charged with compound **S63** (1.5 mmol, 848.6 mg, 1 equiv) and added methanol and water (9:1 by volume, 15 mL). Oxalic acid (3.0 mmol, 270.1 mg, 2 equiv) was then added as solid. After stirring for 12h, the mixture was concentrated *in vacuo*, diluted with ethyl acetate (30 mL) and washed with saturated sodium bicarbonate solution (30 mL). The aqueous layer was separated and extracted with ethyl acetate (3 × 30 mL), and the combined organic layers were dried over $MgSO_4$, filtered and concentrated *in vacuo*. The crude product was then purified on silica gel using 40% ethyl acetate in hexanes as eluent to afford **S64** (603.4 mg, 1.25 mmol, 84% yield) as a white solid.

Characterization:

R*f* = (ethyl acetate/hexane 1:2): 0.25; **IR** (neat) ν = 3366, 3231, 2951, 2929, 1645, 1597, 1521, 1439, 1358, 1316, 1272, 1228, 1176, 1098, 956, 836, 815, 749, 692, 666, 554 cm^{-1}; **^{1}H NMR** (400 MHz, acetone-*d6*) δ 8.30 (br. s, 1H), 8.19 (br. s, 1H), 7.78 (d, *J* = 8.2 Hz, 2H), 7.57 (d, *J* = 7.9 Hz, 2H), 7.46 (d, *J* = 8.0 Hz, 2H), 7.24 (t, *J* = 7.9 Hz, 2H), 7.18 (d, *J* = 8.7 Hz, 2H), 7.01 (t, *J* = 7.4 Hz, 1H), 6.81 (d, *J* = 8.7 Hz, 2H), 4.02 (t, *J* = 6.4 Hz, 2H), 2.44 (s, 3H), 2.07 – 1.99 (m, 1H), 1.94 – 1.83 (m, 1H), 1.67 – 1.57 (m, 2H), 1.53 (s, 3H), 1.37 – 1.27 (m, 2H), 1.25 – 1.10 (m, 2H); **^{13}C NMR** (75 MHz, acetone-*d6*) δ 174.7, 156.0, 144.8, 139.5, 135.5, 133.6, 129.9, 128.4, 127.7, 127.6, 123.2, 119.8, 115.1, 70.6, 50.3, 38.8, 28.4, 25.7, 23.8, 23.2, 20.6; **HRMS**: Calcd. for $C_{27}H_{31}NO_5S$ $[M+Na]^+$ = 504.1815 m/z, found = 504.1812 m/z.

Compound S65:

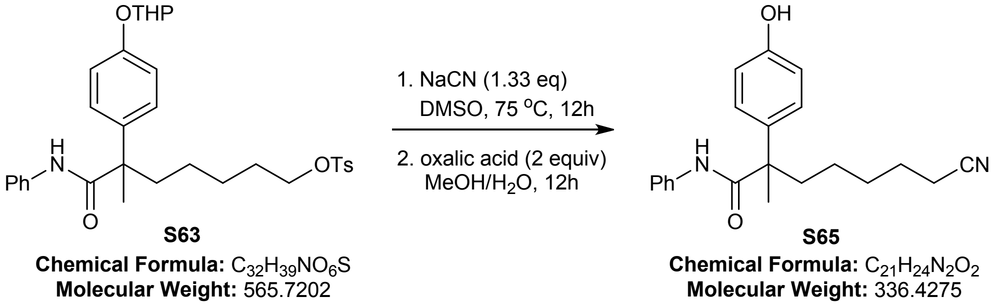

Procedure: A flame-dried, 10 mL round-bottom flask equipped with a Teflon-coated stir bar and a rubber septum was charged with compound **S63** (1.5 mmol, 848.6 mg, 1 equiv) and then purged with a steady stream of N_2 for 5 min, prior to the addition of dry and degassed DMSO (3 mL). Sodium cyanide (2 mmol, 98.0 mg, 1.33 equiv) was then added as solid. The resulting mixture was stirred at 75 °C for 12h, quenched with water (50 mL) and extracted with ethyl acetate (3 × 50 mL). The combined organic layers were dried over $MgSO_4$, filtered and concentrated *in vacuo*. The resulted crude mixture was dissolved in methanol and water (9:1 by volume, 15 mL), and added oxalic acid (3 mmol, 270. 1mg, 2 equiv) as solid. After stirring for 12h, the mixture was concentrated *in vacuo*, diluted with ethyl acetate (30 mL) and washed with saturated sodium bicarbonate solution (30 mL). The aqueous layer was separated and extracted with ethyl acetate (3 × 30 mL), and the combined organic layers were dried over $MgSO_4$, filtered and concentrated *in vacuo*. The crude product was then purified on silica gel using 30% ethyl acetate in hexanes as eluent to afford **S65** (485.6 mg, 1.44 mmol, 96% yield) as a white solid.

Characterization:

R*f* = (ethyl acetate/hexane 1:2): 0.26; **IR** (neat) ν = 3300, 3173, 2984, 2941, 2244, 1649, 1596, 1537, 1512, 1492, 1440, 1320, 1265, 1244, 1180, 908, 826, 814, 761, 696, 553 cm^{-1}; **^{1}H NMR** (400 MHz, acetone-*d6*) δ 8.30 (br. s, 1H), 8.23 (br. s, 1H), 7.58 (d, *J* = 8.2 Hz, 2H), 7.27 – 7.17 (m, 4H), 7.01 (t, *J* = 7.3 Hz, 1H), 6.82 (d, *J* = 8.6 Hz, 2H), 2.42 (t, *J* = 7.1 Hz, 2H), 2.18 – 2.08 (m, 1H), 2.01 – 1.91 (m, 1H), 1.68 – 1.55 (m, 5H), 1.51 – 1.41 (m, 2H), 1.36 – 1.23 (m, 2H); **^{13}C NMR** (75 MHz, acetone-*d6*) δ 174.8, 156.0, 139.5, 135.5, 128.4, 127.6, 123.2, 119.8, 119.7, 115.2, 50.4, 38.8, 29.0, 25.1, 23.7, 23.2, 16.2; **HRMS**: Calcd. for $C_{21}H_{24}N_2O_2$ $[M+Na]^+$ = 359.1730 m/z, found = 359.1728 m/z.

Compound S66:

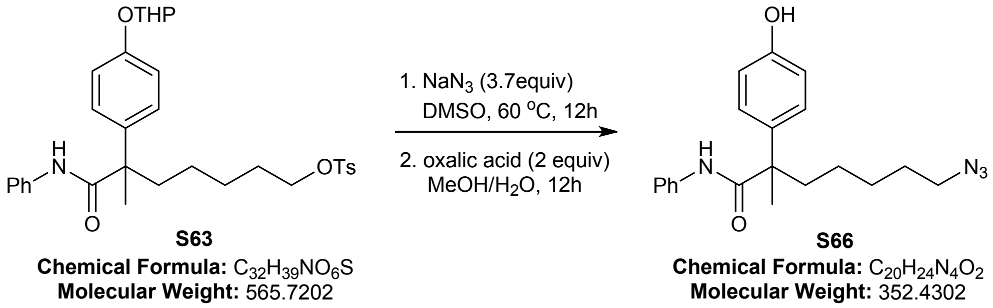

Procedure: A flame-dried, 10 mL round-bottom flask equipped with a Teflon-coated stir bar and a rubber septum was charged with compound **S63** (1.5 mmol, 848.6 mg, 1 equiv) and then purged with a steady stream of N_2 for 5 min, prior to the addition of dry and degassed DMSO (3 mL). Sodium azide (5.6 mmol, 364.1 mg, 3.73 equiv) was then added as solid. The resulting mixture was stirred at 60 °C for 12h, quenched with water (50 mL) and extracted with ethyl acetate (3 × 50 mL). The combined organic layers were dried over $MgSO_4$, filtered and concentrated *in vacuo*. The resulted crude mixture was dissolved in methanol and water (9:1 by volume, 15 mL), and added oxalic acid (3 mmol, 270. 1mg, 2 equiv) as solid. After stirring for 12h, the mixture was concentrated *in vacuo*, diluted with ethyl acetate (30 mL) and washed with saturated sodium bicarbonate solution (30 mL). The aqueous layer was separated and extracted with ethyl acetate (3 × 30 mL), and the combined organic layers were dried over $MgSO_4$, filtered and concentrated *in vacuo*. The crude product was then purified on silica gel using 30% ethyl acetate in hexanes as eluent to afford **S66** (500.4 mg, 1.42 mmol, 95% yield) as a white solid.

Characterization:

R_f = (ethyl acetate/hexane 1:2): 0.56; **IR** (neat) ν = 3294, 3182, 2938, 2862, 2090, 1650, 1596, 1542, 1500, 1438, 1319, 1299, 1246, 1178, 830, 755, 692, 552 cm^{-1}; **^{1}H NMR** (400 MHz, acetone-d_6) δ 8.29 (br. s, 1H), 8.22 (br. s, 1H), 7.60 (dd, *J* = 8.5, 0.9 Hz, 2H), 7.29 – 7.20 (m, 4H), 7.02 (t, *J* = 7.4 Hz, 1H), 6.84 (d, *J* = 8.7 Hz, 2H), 3.30 (t, *J* = 6.9 Hz, 2H), 2.14 (ddd, *J* = 13.2, 11.7, 4.6 Hz, 1H), 1.98 (ddd, *J* = 13.5, 11.5, 5.0 Hz, 1H), 1.65 – 1.55 (m, 5H), 1.48 – 1.37 (m, 2H), 1.37 – 1.20 (m, 2H); **^{13}C NMR** (75 MHz, acetone-d_6) δ 174.9, 156.0, 139.5, 135.5, 128.4, 127.6, 123.2, 119.8, 115.2, 51.0, 50.4, 38.9, 28.5, 27.1, 24.0, 23.3; **HRMS**: Calcd. for $C_{20}H_{24}N_4O_2$ $[M+H]^+$ = 353.1972 m/z, found = 353.1974 m/z.

iv. Substrates in Scheme 6

Compound 4:

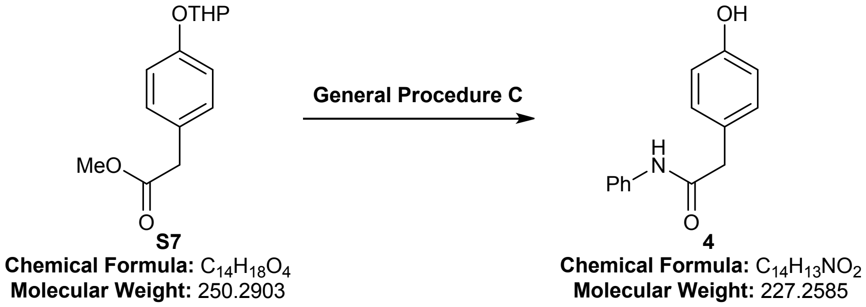

Procedure: The reaction was carried out according to the General Procedure C on 1.6 mmol scale.

Amounts of Reagents:

S7 (1.6 mmol, 400.5 mg)

aniline (2.4 mmol, 218.8 μL)

NaHMDS (3.2 mL, 1M solution in THF)

THF (8 mL, 0.2 M)

oxalic acid (3.2 mmol, 288.1 mg)

MeOH/H_2O (14.4 mL/1.6 mL, 0.1 M)

Purification: 40% EtOAc in hexanes.

Yield of Product:

4: 363.9 mg, 1.60 mmol, 100% yield, white solid.

Characterization:

^{1}H NMR (400 MHz, acetone-d_6) δ 9.32 (br. s, 1H), 8.35 (br. s, 1H), 7.67 (d, J = 7.6 Hz, 2H), 7.32 – 7.20 (m, 4H), 7.06 (t, J = 7.4 Hz, 1H), 6.83 (d, J = 8.6 Hz, 2H), 3.62 (s, 2H); **^{13}C NMR** (75 MHz, acetone-d_6) δ 170.1, 156.3, 139.3, 130.3, 128.7, 126.5, 123.5, 119.5, 115.3, 43.1. The characterization data match previous report.[16]

Compound 5:

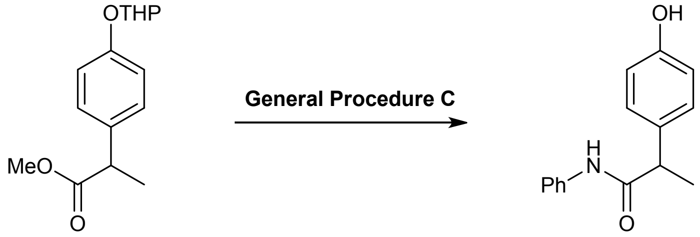

S10
Chemical Formula: $C_{15}H_{20}O_4$
Molecular Weight: 264.3169

5
Chemical Formula: $C_{15}H_{15}NO_2$
Molecular Weight: 241.2851

Procedure: The reaction was carried out according to the General Procedure C on 5.1 mmol scale.

Amounts of Reagents:

S10 (5.1 mmol, 1.35 g)

aniline (6.1 mmol, 556.9 μL)

NaHMDS (10.2 mL, 1M solution in THF)

THF (25.5 mL, 0.2 M)

oxalic acid (10.2 mmol, 918.4 mg)

MeOH/H_2O (45.9 mL/5.1 mL, 0.1 M)

Purification: 30% EtOAc in hexanes.

Yield of Product:

5: 1.04 g, 4.31 mmol, 85% yield, white solid.

Characterization:

R$_f$ = (ethyl acetate/hexane 1:2): 0.24; **IR** (neat) ν = 3347, 3256, 1655, 1595, 1538, 1513, 1441, 1372, 1306, 1231, 1172, 1068, 925, 901, 822, 791, 752, 688, 541 cm^{-1}; **^{1}H NMR** (400 MHz, acetone-d_6) δ 9.11 (br. s, 1H), 8.22 (br. s, 1H), 7.65 (d, J = 7.6 Hz, 2H), 7.27 (t, J = 8.3 Hz, 4H), 7.02 (t, J = 7.4 Hz, 1H), 6.80 (d, J = 8.6 Hz, 2H), 3.76 (q, J = 7.0 Hz, 1H), 1.45 (d, J = 7.0 Hz, 3H); **^{13}C NMR** (75 MHz, acetone-d_6) δ 172.6, 156.3, 139.6, 133.0, 128.5, 128.4, 123.1, 119.2, 115.2, 46.3, 18.7; **HRMS**: Calcd. for $C_{15}H_{15}NO_2$ $[M+Na]^+$ = 264.0995 m/z, found = 264.0990 m/z.

Compound S76:

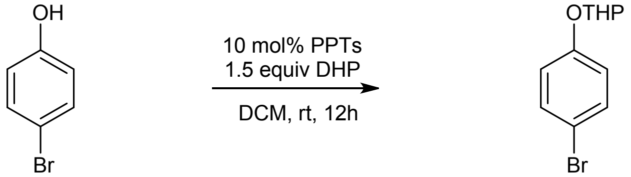

S76

Chemical Formula: C_6H_5BrO
Molecular Weight: 173.0073

Chemical Formula: $C_{11}H_{13}BrO_2$
Molecular Weight: 257.1237

Procedure: A flame-dried, 500 mL round-bottom flask equipped with a Teflon-coated stir bar and a rubber septum was

[16] Iso, Y.; Shindo, H.; Hamana, H. *Tetrahedron* **2000**, *56*, 5353-5361.

charged with pyridinium p-toluenesulfonate (PPTs) (10 mmol, 2.51 g, 10 mol%) and 4-bromophenol (100 mmol, 17.30 g, 1.0 equiv) and then purged with a steady stream of N_2 for 5 min, prior to the addition of dry and degassed CH_2Cl_2 (200 mL). 3,4-Dihydro-2H-pyran (DHP) (150 mmol, 13.69 mL, 1.5 equiv) was then added via syringe. The mixture was stirred at room temperature for 12h, and then quenched with water (100 mL). The aqueous layer was washed with CH_2Cl_2 (3 × 200 mL), and the combined organic layers were dried over $MgSO_4$, filtered and concentrated *in vacuo*. The crude product was purified on silica gel using 5% ethyl acetate in hexanes as eluent to afford **S76** (24.48 g, 95.2 mmol, 95% yield) as a white solid.

Characterization:

^{1}H NMR (500 MHz, $CDCl_3$) δ 7.39 (d, *J* = 9.0 Hz, 2H), 6.96 (d, *J* = 9.0 Hz, 2H), 5.39 (t, *J* = 3.2 Hz, 1H), 3.89 (ddd, *J* = 11.4, 9.8, 3.1 Hz, 1H), 3.62 (dtd, *J* = 11.3, 4.1, 1.3 Hz, 1H), 2.06 – 1.96 (m, 1H), 1.90 – 1.85 (m, 2H), 1.76 – 1.65 (m, 2H), 1.65 – 1.59 (m, 1H). **The characterization data is in accordance with the one that has been reported previously.**[17]

Compound S77:

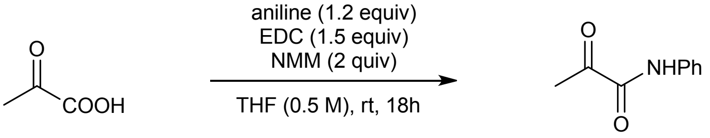

S77

Chemical Formula: $C_3H_4O_3$
Molecular Weight: 88.0621

Chemical Formula: $C_9H_9NO_2$
Molecular Weight: 163.1733

Procedure: A flame-dried, 250 mL round-bottom flask equipped with a Teflon-coated stir bar and a rubber septum was charged with pyruvic acid (50 mmol, 3.52 mL, 1.0 equiv) and then purged with a steady stream of N_2 for 5 min, prior to the addition of dry and degassed THF (100 mL). 1-Ethyl-3-(3-dimethylaminopropyl)carbodiimide (EDC) (75 mmol, 14.38 g, 1.5 equiv) were then added as solids, followed by *N*-methylmorpholine (100 mmol, 11mL, 2 equiv) and aniline (60 mmol, 5.47 mL, 1.2 equiv) via syringe at room temperature. After stirring for 18 h, the mixture was quenched with a 2M HCl (100 mL) and extracted with ethyl acetate (3 × 100 mL). The combined organic layers were dried over $MgSO_4$, filtered and concentrated *in vacuo*. The crude product was purified by silica gel chromatography using hexanes:ethyl acetate mixture (10:1) as the eluent to afford **S77** (1.33 g, 8.15 mmol, 16%) as a white solid.

Characterization:

^{1}H NMR (500 MHz, $CDCl_3$) δ 8.83 (s, 1H), 7.66 (d, *J* = 7.9 Hz, 2H), 7.36 (t, *J* = 7.6 Hz, 2H), 7.17 (t, *J* = 7.2 Hz, 1H), 2.55 (s, 3H). **The characterization data is in accordance with the one that has been reported previously.**[18]

Compound S78:

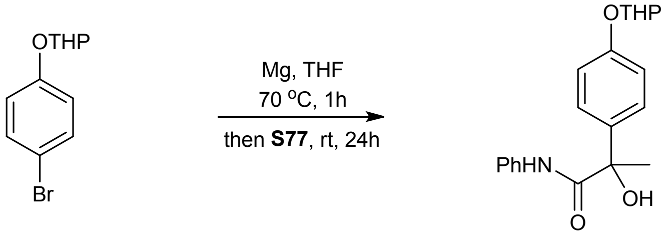

S76 **S78**

Chemical Formula: $C_{11}H_{13}BrO_2$
Molecular Weight: 257.1237

Chemical Formula: $C_{20}H_{23}NO_4$
Molecular Weight: 341.4009

Procedure: A flame-dried, 100 mL round-bottom flask equipped with a Teflon-coated stir bar and a rubber septum was charged with magnesium (30 mmol, 729.3 mg) and **S76** (16.3 mmol, 4.19 g, 2 equiv) and then purged with a steady stream

[17] Dobele, M.; Wiehn, M. S.; Brase, S. *Angew. Chem. Int. Ed.* **2011**, *50*, 11533-11535.
[18] Guin, S.; Rout, S. K.; Gogoi, A.; Ali, W.; Patel, B. K. *Adv. Syn. Catal.* **2014**, *356*, 2559-2565.

of N_2 for 5 min, prior to the addition of dry and degassed THF (20 mL). The resulting mixture was stirred at 70 °C for 1h to produce a brown Grignard solution. In a separate 100 mL round-bottom flask equipped with a Teflon-coated stir bar and a rubber septum was charged with **S77** (8.15 mmol, 1.33 g, 1 equiv) and then purged with a steady stream of N_2 for 5 min, prior to the addition of dry and degassed THF (20 mL). The freshly prepared Grignard reagent was then added to the ethyl pyruvate solution via syringe dropwise at 0 °C. When the addition is complete, the mixture was allowed to stir at room temperature for 24h before the addition of NH_4Cl (saturated solution, 100 mL) and stir for another 30 min. The mixture was extracted with EtOAc (3 × 50 mL), and the combined organic layers were dried over $MgSO_4$, filtered and concentrated *in vacuo*. The crude product was purified on silica gel using 30% ethyl acetate in hexanes as eluent to afford **S78** (1.08 g, 3.16 mmol, 39% yield) as a white solid.

Characterization:

R_f = (EtOAc/Hexane 1:3): 0.25; **IR** (neat) ν = 3358, 2942, 1664, 1600, 1524, 1506, 1441, 1357, 1312, 1236, 1202, 1177, 1108 cm^{-1}; **^{1}H NMR** (500 MHz, Acetone-d_6) δ 9.28 (br. s, 1H), 7.75 (d, *J* = 7.6 Hz, 2H), 7.59 (d, *J* = 8.9 Hz, 2H), 7.30 (dd, *J* = 8.4, 7.6 Hz, 2H), 7.06 (t, *J* = 7.4 Hz, 1H), 7.02 (d, *J* = 8.8 Hz, 2H), 5.44 (t, *J* = 3.3 Hz, 1H), 5.43 (br. d, *J* = 1.2 Hz, 1H), 3.83 (ddd, *J* = 11.4, 9.5, 3.1 Hz, 1H), 3.57 (dtd, *J* = 11.4, 4.3, 0.9 Hz, 1H), 2.00 – 1.93 (m, 1H), 1.86 – 1.76 (m, 5H), 1.70 – 1.62 (m, 2H), 1.59 – 1.53 (m, 1H); **^{13}C NMR** (126 MHz, Acetone-d_6) δ 173.1, 156.5, 138.8, 137.5, 128.6, 126.39, 126.38, 123.4, 119.4, 115.83, 115.80, 96.1, 76.1, 76.0, 61.5, 30.2, 26.84, 26.82, 25.1, 18.7 (***a complex mixture of diastereomers are observed in the ^{13}C NMR, which accounts for the presence of extra ^{13}C signals***); **HRMS**: Calcd. for $C_{20}H_{23}NO_4$ $[M+Na]^+$ = 364.1519 m/z, found = 364.1518 m/z.

Compound 6:

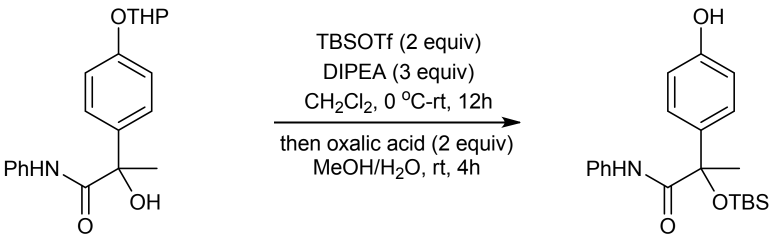

S78 | **6**

Chemical Formula: $C_{20}H_{23}NO_4$
Molecular Weight: 341.4009

Chemical Formula: $C_{21}H_{29}NO_3Si$
Molecular Weight: 371.5454

Procedure: A flame-dried, 100 mL round-bottom flask equipped with a Teflon-coated stir bar and a rubber septum was charged with **S78** (3.16 mmol, 1.08 g, 1 equiv) and then purged with a steady stream of N_2 for 5 min, prior to the addition of dry and degassed CH_2Cl_2 (30 mL). The mixture was then cooled to 0 °C, at which temperature N,N-diisopropylethylamine (DIPEA) (9.48 mmol, 1.65 mL, 3 equiv) and TBSOTf (6.32 mmol, 1.45 mL, 2 equiv) was added. The resulting mixture was stirred at room temperature for 12h and then poured on HCl (2M, 30 mL). The mixture was extracted with CH_2Cl_2 (3 × 50 mL), and the combined organic layers were dried over $MgSO_4$, filtered and concentrated *in vacuo*. The crude product was directly dissolved in $MeOH/H_2O$ (30 mL, 10:1 by volume). Oxalic acid (568.8 mg, 6.32 mmol, 2 equiv) was then added as solid. And the resulting mixture was stirred at room temperature for 4h, before the addition of saturated $NaHCO_3$ solution (30 mL). MeOH was then removed *in vacuo*. The resulting mixture was extracted with EtOAc (3 × 50 mL), and the combined organic layers were dried over $MgSO_4$, filtered and concentrated *in vacuo*. The crude product was purified on silica gel using 30% ethyl acetate in hexanes as eluent to afford **6** (628.1 mg, 1.69 mmol, 53% yield) as a white solid.

Characterization:

R_f = (EtOAc/Hexane 1:3): 0.39; **IR** (neat) ν = 3370, 3204, 2926, 2855, 1661, 1594, 1528, 1517, 1442, 1368, 1281, 1250, 1228, 1178, 1125 cm^{-1}; **^{1}H NMR** (500 MHz, $CDCl_3$) δ 9.34 (br. s, 1H), 7.63 (d, *J* = 7.7 Hz, 2H), 7.38 (t, *J* = 7.9 Hz, 2H), 7.31 (br. s, 1H), 7.17 (t, *J* = 7.7 Hz, 3H), 6.48 (d, *J* = 8.7 Hz, 2H), 1.94 (s, 3H), 1.02 (s, 9H), 0.08 (s, 3H), -0.25 (s, 3H); **^{13}C NMR** (126 MHz, $CDCl_3$) δ 174.6, 156.4, 137.4, 133.3, 129.2, 127.3, 124.6, 119.4, 115.4, 79.3, 26.1, 25.1, 18.2, -2.5, -3.4; **HRMS**: Calcd.

for $C_{21}H_{29}NO_3Si$ [M+Na]$^+$ = 394.1809 m/z, found = 394.1810 m/z.

Compound S79:

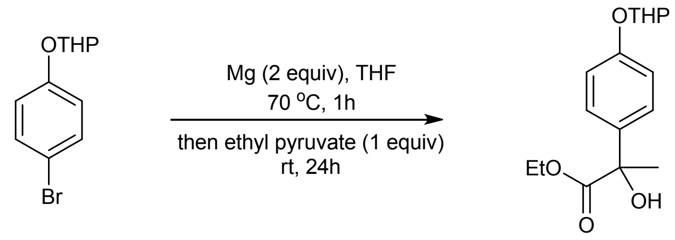

S76

Chemical Formula: $C_{11}H_{13}BrO_2$
Molecular Weight: 257.1237

S79

Chemical Formula: $C_{16}H_{22}O_5$
Molecular Weight: 294.3429

Procedure: A flame-dried, 100 mL round-bottom flask equipped with a Teflon-coated stir bar and a rubber septum was charged with magnesium (100 mmol, 2.43 g, 2 equiv) and **S76** (50 mmol, 12.86 g, 1.0 equiv) and then purged with a steady stream of N_2 for 5 min, prior to the addition of dry and degassed THF (50 mL). The resulting mixture was stirred at 70 °C for 1h to produce a brown Grignard solution. In a separate 250 mL round-bottom flask equipped with a Teflon-coated stir bar and a rubber septum was charged with ethyl pyruvate (50 mmol, 5.56 mL, 1 equiv) and then purged with a steady stream of N_2 for 5 min, prior to the addition of dry and degassed THF (50 mL). The freshly prepared Grignard reagent was then added to the ethyl pyruvate solution via syringe dropwise at 0 °C. When the addition is complete, the mixture was allowed to stir at room temperature for 24h before the addition of NH_4Cl (saturated solution, 100 mL) and stir for another 30 min. The mixture was extracted with EtOAc (3 × 200 mL), and the combined organic layers were dried over $MgSO_4$, filtered and concentrated *in vacuo*. The crude product was purified on silica gel using 20% ethyl acetate in hexanes as eluent to afford **S79** (4.57 g, 15.5 mmol, 31% yield) as a colorless oil.

Characterization:

R*f* = (EtOAc/Hexane 1:2): 0.62; **IR** (neat) v = 3501, 2941, 2873, 1724, 1608, 1507, 1454, 1357, 1234, 1202, 1177, 1147, 1108 cm^{-1}; **^{1}H NMR** (500 MHz, Acetone-d_6) δ 7.50 (d, *J* = 8.9 Hz, 2H), 7.03 (d, *J* = 8.9 Hz, 2H), 5.45 (s, 1H), 4.70 (s, 1H), 4.25 – 4.07 (m, 2H), 3.91 – 3.79 (m, 1H), 3.58 (dt, *J* = 9.6, 4.2 Hz, 1H), 2.02 – 1.93 (m, 1H), 1.89 – 1.77 (m, 2H), 1.71 (s, 3H), 1.69 – 1.61 (m, 2H), 1.61 – 1.54 (m, 1H), 1.22 (t, *J* = 7.1 Hz, 3H); **^{13}C NMR** (126 MHz, Acetone-d_6) δ 174.942, 174.938, 156.58, 156.55, 136.94, 136.92, 126.26, 126.25, 115.88, 115.87, 96.2, 96.1, 75.24, 75.23, 61.5, 61.2, 30.2, 26.87, 26.86, 25.1, 18.7, 13.5 (***a complex mixture of diastereomers are observed in the ^{13}C NMR, which accounts for the presence of extra ^{13}C signals***); **HRMS**: Calcd. for $C_{16}H_{22}O_5$ [M+Na]$^+$ = 317.1359 m/z, found = 317.1357 m/z.

Compound S80:

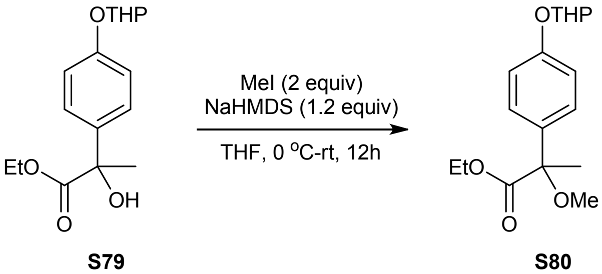

S79

Chemical Formula: $C_{16}H_{22}O_5$
Molecular Weight: 294.3429

S80

Chemical Formula: $C_{17}H_{24}O_5$
Molecular Weight: 308.3695

Procedure: A flame-dried, 100 mL round-bottom flask equipped with a Teflon-coated stir bar and a rubber septum was charged **S79** (15.5 mmol, 4.57 g, 1.0 equiv) and then purged with a steady stream of N_2 for 5 min, prior to the addition of dry and degassed THF (50 mL). The resulting mixture was cooled to 0 °C, at which temperature NaHMDS solution (18.6 mL, 18.6 mmol, 1M in THF, 1.2 equiv) was added followed by MeI (1.93 mL, 31.0 mmol, 2 equiv). The mixture warmed to

room temperature and stir for 12h before the addition of NH_4Cl (saturated solution, 50 mL). The mixture was extracted with EtOAc (3 × 100 mL), and the combined organic layers were dried over $MgSO_4$, filtered and concentrated *in vacuo*. The crude product was purified on silica gel using 20% ethyl acetate in hexanes as eluent to afford **S80** (4.23 g, 13.7 mmol, 88% yield) as a colorless oil.

Characterization:

R_f = (EtOAc/Hexane 1:5): 0.33; **IR** (neat) ν = 2940, 1727, 1608. 1508, 1454, 1370, 1233, 1201, 1176, 1107 cm^{-1}; **^{1}H NMR** (500 MHz, Acetone-d_6) δ 7.40 (d, *J* = 8.9 Hz, 2H), 7.04 (d, *J* = 8.9 Hz, 2H), 5.45 (t, *J* = 3.3 Hz, 1H), 4.22 – 4.09 (m, 2H), 3.85 (ddd, *J* = 11.5, 9.5, 3.2 Hz, 1H), 3.59 (dtd, *J* = 11.4, 4.4, 0.9 Hz, 1H), 3.26 (s, 3H), 2.02 – 1.93 (m, 1H), 1.88 – 1.77 (m, 2H), 1.71 (s, 3H), 1.70 – 1.63 (m, 2H), 1.60 – 1.55 (m, 1H), 1.21 (t, *J* = 7.1 Hz, 3H); **^{13}C NMR** (126 MHz, Acetone-d_6) δ 172.5, 156.7, 134.54, 134.52, 126.95, 126.94, 115.9, 96.15, 96.13, 81.1, 61.6, 60.6, 51.365, 51.356, 30.2, 25.1, 22.2, 22.1, 18.7, 13.6 (***a complex mixture of diastereomers are observed in the ^{13}C NMR, which accounts for the presence of extra ^{13}C signals***); **HRMS**: Calcd. for $C_{17}H_{24}O_5$ $[M+Na]^+$ = 331.1516 m/z, found = 331.1509 m/z.

Compound 7:

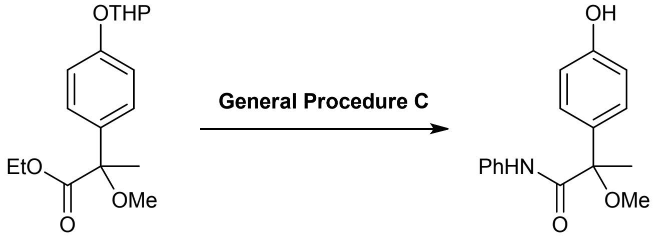

S80　　　**7**

Chemical Formula: $C_{17}H_{24}O_5$
Molecular Weight: 308.3695

Chemical Formula: $C_{16}H_{17}NO_3$
Molecular Weight: 271.3111

Procedure: Followed General Procedure C.

Amounts of Reagents:

S80 (13.72 mmol, 4.23 g)

aniline (20.58 mmol, 1.88 mL)

NaHMDS (30.18 mL, 1M solution in THF)

THF (20 mL, 0.2 M)

oxalic acid (30 mmol, 2.70 g)

MeOH/H_2O (135 mL/15 mL)

Purification: 30% EtOAc in hexanes.

Yield of Product:

7: 3.66 g, 13.4 mmol, 98% yield, white solid.

Characterization:

R_f = (EtOAc/Hexane 1:2): 0.38; **IR** (neat) ν = 3338, 3232, 2928, 1655, 1612, 1595, 1529, 1510, 1442, 1367, 1264, 1236, 1190, 1172, 1137, 1123 cm^{-1}; **^{1}H NMR** (500 MHz, $CDCl_3$) δ 9.05 (br. s, 1H), 7.65 (d, *J* = 7.8 Hz, 2H), 7.37 (t, *J* = 7.9 Hz, 2H), 7.28 (br. s, 1H), 7.16 (t, *J* = 7.5 Hz, 3H), 6.53 (d, *J* = 8.6 Hz, 2H), 3.20 (s, 3H), 1.85 (s, 3H); **^{13}C NMR** (126 MHz, $CDCl_3$) δ 173.2, 156.4, 137.3, 130.1, 129.1, 127.8, 124.6, 119.9, 115.7, 81.6, 50.9, 19.8; **HRMS**: Calcd. for $C_{16}H_{17}NO_3$ $[M+Na]^+$ = 294.1101 m/z, found = 294.1096 m/z.

v. Substrates in Scheme 7

Compound S67:

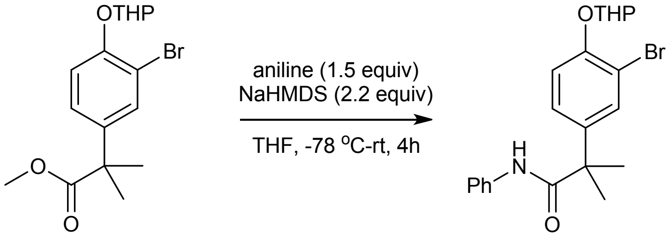

S22
Chemical Formula: $C_{16}H_{21}BrO_4$
Molecular Weight: 357.2395

S67
Chemical Formula: $C_{21}H_{24}BrNO_3$
Molecular Weight: 418.3242

Procedure: The following procedure is based on the procedure of Wang.[19] A flame-dried, 500 mL round-bottom flask equipped with a Teflon-coated stir bar and a rubber septum was charged with aniline (6.84 mL, 75 mmol, 1.5 equiv) and then purged with a steady stream of N_2 for 5 min, prior to the addition of dry and degassed THF (80 mL). Sodium bis(trimethylsilyl)amide (NaHMDS) (110 mmol, 110 mL, 1.0 M in THF, 2.2 equiv) was added dropwise at -78 °C and stirred for 20 min. A separate, flame-dried, 50 mL round-bottom flask equipped with a Teflon-coated stir bar and a rubber septum was charged with **S22** (16.38 g, 45.8 mmol, 1.0 equiv) and then purged with a steady stream of N_2 for 5 min, prior to the addition of dry and degassed THF (20 mL) to afford a homogeneous solution. This solution was then added to the solution of the aniline and NaHMDS in THF dropwise via syringe at -78 °C. The mixture was then warmed to room temperature and stirred for 4h, quenched with saturated ammonium chloride (200 mL) and diluted with ethyl acetate (200 mL). The aqueous layer was separated and extracted with ethyl acetate (2 × 200 mL), and the combined organic layers were dried over $MgSO_4$, filtered and concentrated *in vacuo*. The crude product was purified on silica gel using 10% ethyl acetate in hexanes as eluent to afford **S67** (16.05 g, 38.4 mmol, 84% yield) as a white solid.

Characterization:

R_f = (EtOAc/Hexane 1:5): 0.38; **IR** (neat) ν = 3298, 2943, 1656, 1599, 1533, 1497, 1437, 1314, 1252, 1202, 1111 cm^{-1}; **^{1}H NMR** (500 MHz, Acetone-d_6) δ 8.48 (br. s, 1H), 7.61 (dd, *J* = 8.6, 0.9 Hz, 2H), 7.59 (d, *J* = 2.4 Hz, 1H), 7.33 (dd, *J* = 8.7, 2.4 Hz, 1H), 7.26 (dd, *J* = 8.5, 7.5 Hz, 2H), 7.20 (d, *J* = 8.7 Hz, 1H), 7.03 (t, *J* = 7.4 Hz, 1H), 5.59 (t, *J* = 2.9 Hz, 1H), 3.85 (td, *J* = 11.0, 3.0 Hz, 1H), 3.58 (dtd, *J* = 11.2, 3.8, 1.0 Hz, 1H), 2.12 – 2.00 (m, 1H), 1.94 – 1.83 (m, 2H), 1.77 – 1.66 (m, 2H), 1.66 – 1.58 (m, 7H); **^{13}C NMR** (126 MHz, Acetone-d_6) δ 174.4, 152.0, 140.3, 139.5, 130.6, 128.4, 126.5, 123.3, 119.8, 116.4, 112.3, 96.4, 61.5, 47.0, 29.9, 26.5, 26.4, 25.0, 18.3; **HRMS**: Calcd. for $C_{21}H_{24}BrNO_3$ $[M+Na]^+$ = 440.0832 m/z, found = 440.0825 m/z.

Compound S68:

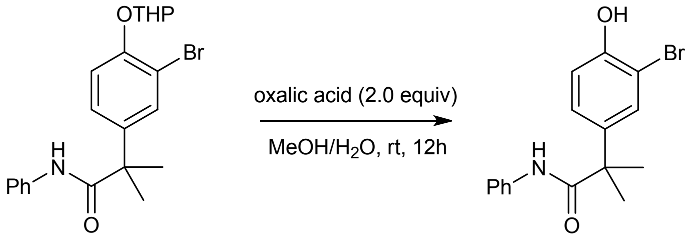

S67
Chemical Formula: $C_{21}H_{24}BrNO_3$
Molecular Weight: 418.3242

S68
Chemical Formula: $C_{16}H_{16}BrNO_2$
Molecular Weight: 334.2077

Procedure: In a 250 mL round-bottom flask, compound **S67** (2.09 g, 5 mmol, 1 equiv) was dissolved in methanol and water (50 mL 9:1 by volume, 0.1 M), and oxalic acid (10 mmol, 900.3 mg, 2 equiv) was added directly as a solid. After stirring for 12h, the mixture was concentrated *in vacuo*, diluted with ethyl acetate (100 mL) and washed with a saturated aqueous solution of sodium bicarbonate (100 mL). The aqueous layer was extracted with ethyl acetate (2 × 100 mL), and the combined organic layers were dried over $MgSO_4$, filtered and concentrated *in vacuo*. The crude product was purified

[19] Wang, J.; Rosingana, M.; Discordia, R. P.; Soundararajan, N.; Polniaszek, R. *SynLett* **2001**, *9*, 1485-1487.

on silica gel using 30% ethyl acetate in hexanes as eluent to afford **S68** (1.64 g, 4.9 mmol, 98% yield) as a white solid.

Characterization:

R*f* = (EtOAc/Hexane 1:3): 0.33; **IR** (neat) ν = 3314, 3159, 2968, 1743, 1632, 1567, 1518, 1501, 1440, 1288, 1248 cm^{-1}; **^{1}H NMR** (500 MHz, $CDCl_3$) δ 7.56 (d, *J* = 2.3 Hz, 1H), 7.40 (dd, *J* = 8.6, 1.0 Hz, 2H), 7.32 – 7.27 (m, 3H), 7.10 (t, *J* = 7.4 Hz, 1H), 7.06 (d, *J* = 8.5 Hz, 1H), 6.88 (br. s, 1H), 5.88 (br. s, 1H), 1.65 (s, 6H); **^{13}C NMR** (126 MHz, $CDCl_3$) δ 175.2, 151.7, 138.2, 137.7, 130.0, 128.9, 127.6, 124.4, 119.8, 116.6, 110.6, 47.3, 27.1; **HRMS**: Calcd. for $C_{16}H_{16}BrNO_2$ $[M+Na]^+$ = 356.0257 m/z, found = 356.0263 m/z.

Compound S69:

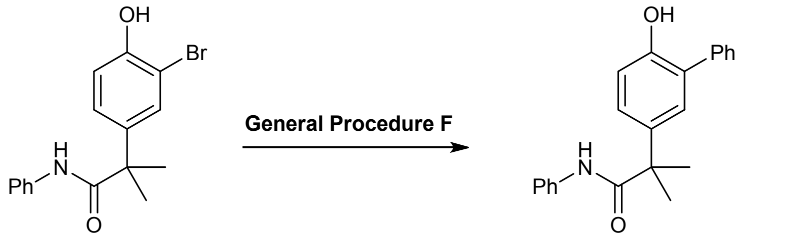

S68
Chemical Formula: $C_{16}H_{16}BrNO_2$
Molecular Weight: 334.2077

S69
Chemical Formula: $C_{22}H_{21}NO_2$
Molecular Weight: 331.4076

Procedure: The reaction was carried out according to the General Procedure F.

Amounts of Reagents:

S68 (668.4 mg, 2 mmol)

Phenylboronic acid (365.8 mg, 3 mmol)

$Pd(OAc)_2$ (1.1 mg, 0.005 mmol)

Diisopropylamine (560.6 µL, 4 mmol)

H_2O (4 mL, 0.5 M)

Purification: 2.5% EtOAc in CH_2Cl_2.

Yield of Product:

S69: 602.2 mg, 1.82 mmol, 91% yield, white solid.

Characterization:

R*f* = (ethyl acetate/CH_2Cl_2 1:40): 0.43; **IR** (neat) ν = 3392, 3142, 2974, 1645, 1596, 1532, 1489, 1438, 1406, 1316, 1271, 1199, 1149, 1128 cm^{-1}; **^{1}H NMR** (500 MHz, $CDCl_3$) δ 7.58 – 7.47 (m, 4H), 7.45 (t, *J* = 7.1 Hz, 1H), 7.41 (dd, *J* = 8.6, 1.0 Hz, 2H), 7.36 (dd, *J* = 8.4, 2.5 Hz, 1H), 7.34 (d, *J* = 2.4 Hz, 1H), 7.32 – 7.27 (m, 2H), 7.09 (t, *J* = 7.4 Hz, 1H), 7.05 (d, *J* = 8.4 Hz, 1H), 6.95 (br. s, 1H), 5.34 (br. s, 1H), 1.69 (s, 6H); **^{13}C NMR** (126 MHz, $CDCl_3$) δ 175.8, 151.7, 138.0, 136.9, 136.7, 129.4, 129.0, 128.9, 128.5, 128.3, 128.2, 127.4, 124.2, 119.7, 116.4, 47.5, 27.2; **HRMS**: Calcd. for $C_{22}H_{21}NO_2$ $[M+Na]^+$ = 354.1464 m/z, found = 354.1473 m/z.

Compound S70:

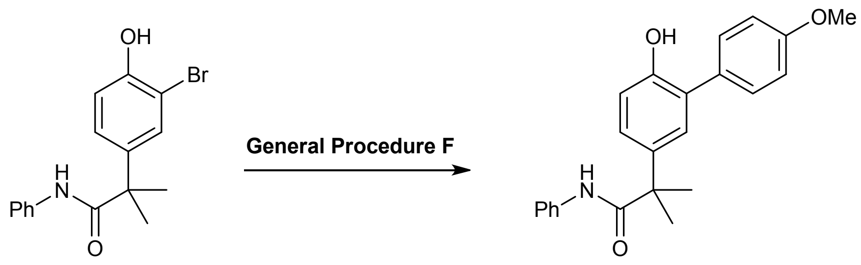

S68
Chemical Formula: $C_{16}H_{16}BrNO_2$
Molecular Weight: 334.2077

S70
Chemical Formula: $C_{23}H_{23}NO_3$
Molecular Weight: 361.4336

Procedure: The reaction was carried out according to the General Procedure F.

Amounts of Reagents:

S68 (668.4 mg, 2 mmol)

4-Methoxyphenylboronic acid (455.9 mg, 3 mmol)

$Pd(OAc)_2$ (1.1 mg, 0.005 mmol)

Diisopropylamine (560.6 µL, 4 mmol)

H_2O (4 mL, 0.5 M)

Purification: 2.5% EtOAc in CH_2Cl_2.

Yield of Product:

S70: 600.7 mg, 1.66 mmol, 83% yield, white solid.

Characterization:

R_f = (ethyl acetate/CH_2Cl_2 1:40): 0.47; **IR** (neat) ν = 3396, 3172, 2970, 2834, 1647, 1598, 1531, 1499, 1438, 1396, 1273, 1239, 1199, 1170, 1127, 1105 cm^{-1}; **^{1}H NMR** (500 MHz, $CDCl_3$) δ 7.48 (d, *J* = 8.8 Hz, 2H), 7.42 (dd, *J* = 8.5, 0.9 Hz, 2H), 7.35 (d, *J* = 2.5 Hz, 1H), 7.33 – 7.28 (m, 3H), 7.14 – 7.07 (m, 2H), 7.04 (dd, *J* = 8.6, 1.9 Hz, 3H), 6.18 (br. s, 1H), 3.88 (s, 3H), 1.71 (s, 6H); **^{13}C NMR** (126 MHz, $CDCl_3$) δ 176.4, 159.3, 152.2, 137.9, 136.4, 130.3, 129.3, 128.9, 128.4, 128.4, 126.8, 124.3, 120.0, 116.4, 114.6, 55.4, 47.5, 27.3; **HRMS**: Calcd. for $C_{23}H_{23}NO_3$ $[M+Na]^+$ = 384.1570 m/z, found = 384.1583 m/z.

Compound S71:

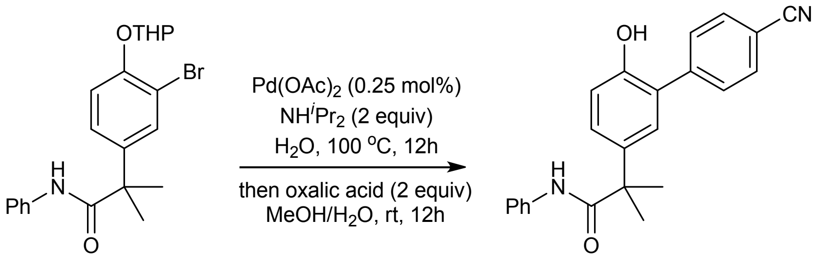

S67 **Chemical Formula:** $C_{21}H_{24}BrNO_3$ **Molecular Weight:** 418.3242

S71 **Chemical Formula:** $C_{23}H_{20}N_2O_2$ **Molecular Weight:** 356.4171

Procedure: The following procedure is based on the procedure of Liu.[20] A 10 mL round-bottom flask equipped with a Teflon-coated stir bar and a rubber septum was charged with $Pd(OAc)_2$ (1.1 mg, 0.005 mmol, 0.0025 equiv), compound **S67** (836.6 mg, 2 mmol, 1 equiv), 4-cyanophenylboronic acid (440.8 mg, 3 mmol, 1.5 equiv) and then purged with a steady stream of N_2 for 5 min, prior to the addition of degassed H_2O (4 mL). Diisopropylamine (4 mmol, 560.6 µL, 2 equiv) was then added with a syringe. The mixture was then warmed to 100 °C and stirred for 12h under N_2, quenched with HCl (2 M, 20 mL) and extracted with ethyl acetate (3 × 20 mL). The combined organic layers were dried over $MgSO_4$, filtered and concentrated *in vacuo*. The crude product was dissolved in methanol and water (20 mL 9:1 by volume, 0.1 M), and oxalic acid (4 mmol, 360.1 mg, 2 equiv) was added directly as a solid. After stirring for 12h, the mixture was concentrated *in vacuo*, diluted with ethyl acetate (20 mL) and washed with a saturated aqueous solution of sodium bicarbonate (20 mL). The aqueous layer was extracted with ethyl acetate (2 × 20 mL), and the combined organic layers were dried over $MgSO_4$, filtered and concentrated *in vacuo*. The crude product was purified on silica gel using 30% ethyl acetate in hexanes as eluent to afford **S71** (495.8 mg, 1.39 mmol, 70% yield) as a white solid.

Characterization:

R_f = (ethyl acetate/hexane 1:2): 0.23; **IR** (neat) ν = 3370, 3266, 2966, 2229, 1648, 1599, 1536, 1496, 1439, 1396, 1314, 1176, 1153, 1133 cm^{-1}; **^{1}H NMR** (500 MHz, $CDCl_3$) δ 7.73 (d, *J* = 8.3 Hz, 2H), 7.68 (d, *J* = 8.4 Hz, 2H), 7.40 (d, *J* = 7.7 Hz, 2H), 7.37 – 7.28 (m, 4H), 7.11 (t, *J* = 7.4 Hz, 1H), 7.05 (br. s, 1H), 7.00 (d, *J* = 8.4 Hz, 1H), 6.35 (br. s, 1H), 1.70 (s, 6H); **^{13}C NMR** (126 MHz, $CDCl_3$) δ 175.9, 152.2, 142.5, 137.6, 137.0, 132.3, 130.0, 129.0, 128.4, 128.1, 126.9, 124.6, 120.0, 118.8, 117.0, 111.0, 47.4, 27.2; **HRMS**: Calcd. for $C_{23}H_{20}N_2O_2$ $[M+Na]^+$ = 379.1417 m/z, found = 379.1424 m/z.

[20] Liu, C.; Zhang, Y.; Liu, N.; Qiu, J. *Green Chem.* **2012**, *14*, 2999–3003.

Compound S72:

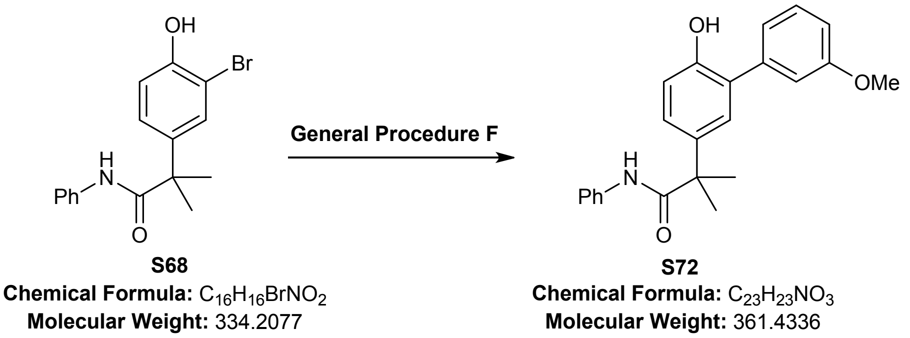

Procedure: The reaction was carried out according to the General Procedure F.

Amounts of Reagents:

S68 (668.4 mg, 2 mmol)

2-Methoxyphenylboronic acid (455.9 mg, 3 mmol)

$Pd(OAc)_2$ (1.1 mg, 0.005 mmol)

Diisopropylamine (560.6 μL, 4 mmol)

H_2O (4 mL, 0.5 M)

Purification: 2.5% EtOAc in CH_2Cl_2.

Yield of Product:

S72: 612.2 mg, 1.69 mmol, 85% yield, white solid.

Characterization:

$\mathbf{R_f}$ = (ethyl acetate/CH_2Cl_2 1:40): 0.33; **IR** (neat) ν = 3387, 3306, 2971, 2935, 2834, 1655, 1596, 1500, 1485, 1436, 1398, 1312, 1275, 1238, 1170, 1145, 1127 cm^{-1}; **^{1}H NMR** (500 MHz, $CDCl_3$) δ 7.43 (t, *J* = 7.9 Hz, 3H), 7.38 (d, *J* = 2.4 Hz, 1H), 7.34 (dd, *J* = 8.5, 2.5 Hz, 1H), 7.32 – 7.28 (m, 2H), 7.16 – 7.07 (m, 4H), 7.06 (d, *J* = 8.5 Hz, 1H), 6.97 (dd, *J* = 8.3, 2.0 Hz, 1H), 6.21 (br. s, 1H), 3.86 (s, 3H), 1.71 (s, 6H); **^{13}C NMR** (126 MHz, $CDCl_3$) δ 176.3, 160.1, 152.1, 138.5, 137.8, 136.5, 130.3, 128.9, 128.4, 128.3, 127.3, 124.4, 121.3, 120.0, 116.6, 114.8, 113.5, 55.4, 47.5, 27.3; **HRMS**: Calcd. for $C_{23}H_{23}NO_3$ $[M+Na]^+$ = 384.1570 m/z, found = 384.1574 m/z.

Compound S73:

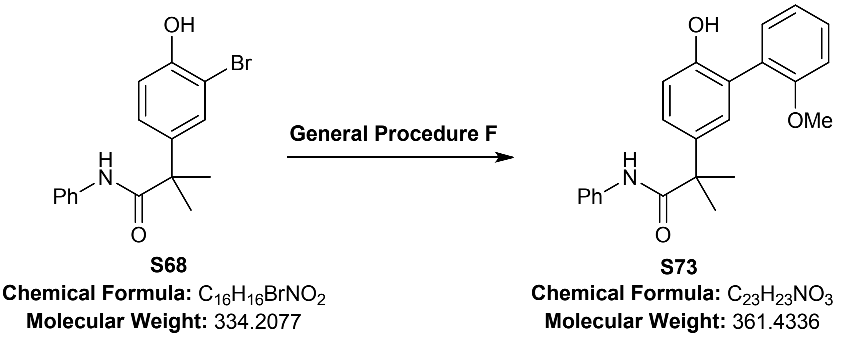

Procedure: The reaction was carried out according to the General Procedure F.

Amounts of Reagents:

S68 (668.4 mg, 2 mmol)

2-Methoxyphenylboronic acid (455.9 mg, 3 mmol)

$Pd(OAc)_2$ (1.1 mg, 0.005 mmol)

Diisopropylamine (560.6 μL, 4 mmol)

H_2O (4 mL, 0.5 M)

Purification: 2.5% EtOAc in CH_2Cl_2.

Yield of Product:

S73: 665.8 mg, 1.84 mmol, 92% yield, white solid.

Characterization:

R$_f$ = (ethyl acetate/CH_2Cl_2 1:40): 0.45; **IR** (neat) ν = 3336, 2982, 2968, 2929, 1662, 1597, 1530, 1489, 1434, 1315, 1273, 1228, 1180, 1144, 1114 cm^{-1}; **^{1}H NMR** (500 MHz, $CDCl_3$) δ 7.48 – 7.42 (m, 3H), 7.41 (dd, *J* = 8.4, 2.5 Hz, 1H), 7.38 (d, *J* = 2.4 Hz, 1H), 7.36 (dd, *J* = 7.6, 1.6 Hz, 1H), 7.33 – 7.29 (m, 2H), 7.15 (td, *J* = 7.5, 0.7 Hz, 1H), 7.13 – 7.01 (m, 4H), 6.45 (br. s, 1H), 3.92 (s, 3H), 1.72 (s, 6H); **^{13}C NMR** (126 MHz, $CDCl_3$) δ 176.1, 155.6, 153.1, 138.1, 136.7, 132.4, 129.8, 129.7, 128.9, 127.3, 126.62, 126.59, 124.1, 122.2, 119.8, 117.8, 111.6, 56.1, 47.5, 27.3; **HRMS**: Calcd. for $C_{23}H_{23}NO_3$ $[M+Na]^+$ = 384.1570 m/z, found = 384.1577 m/z.

Compound S74:

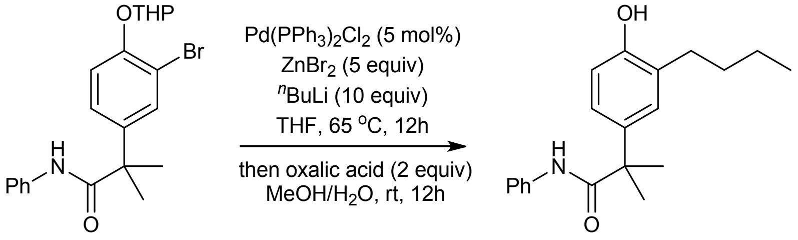

S67
Chemical Formula: $C_{21}H_{24}BrNO_3$
Molecular Weight: 418.3242

S74
Chemical Formula: $C_{20}H_{25}NO_2$
Molecular Weight: 311.4180

Procedure: A 100 mL flame-dried round-bottom flask equipped with a Teflon-coated stir bar and a rubber septum was charged with $Pd(OAc)_2$ (140.4 mg, 0.2 mmol, 0.05 equiv), compound **S67** (1.67 g, 4 mmol, 1 equiv) and then purged with a steady stream of N_2 for 5 min, prior to the addition of dry and degassed THF (20 mL). In a separate 50 mL flame-dried round-bottom flask equipped with a Teflon-coated stir bar and a rubber septum was charged with $ZnBr_2$ (4.5 g, 20 mmol, 5 equiv) and then purged with a steady stream of N_2 for 5 min, prior to the addition of dry and degassed THF (20 mL). It was then cooled to -78 °C, at which temperature ^{n}BuLi (16 mL, 2.5 M in hexane, 40 mmol, 10 equiv) was then added via syringe and the resulting butylzinc solution was stirred at rt for 10 min, prior to the addition to the solution of substrate and catalyst. The mixture was then warmed to 65 °C and stirred for 12h under N_2, quenched with saturated NH_4Cl (40 mL) and extracted with ethyl acetate (3 × 20 mL). The combined organic layers were dried over $MgSO_4$, filtered and concentrated *in vacuo*. The crude product was dissolved in methanol and water (40 mL 9:1 by volume, 0.1 M), and oxalic acid (8 mmol, 720.2 mg, 2 equiv) was added directly as a solid. After stirring for 12h, the mixture was concentrated *in vacuo*, diluted with ethyl acetate (40 mL) and washed with a saturated aqueous solution of sodium bicarbonate (40 mL). The aqueous layer was extracted with ethyl acetate (2 × 40 mL), and the combined organic layers were dried over $MgSO_4$, filtered and concentrated *in vacuo*. The crude product was purified on silica gel using 2.5% ethyl acetate in CH_2Cl_2 as eluent to afford **S74** (342.6 mg, 1.10 mmol, 28% yield) as a yellow solid.

Characterization:

R$_f$ = (ethyl acetate/CH_2Cl_2 1:40): 0.34; **IR** (neat) ν = 3345, 3297, 2974, 2949, 2928, 2868, 1661, 1597, 1526, 1511, 1500, 1442, 1421, 1355, 1318, 1260, 1232, 1178, 1145, 1128 cm^{-1}; **^{1}H NMR** (500 MHz, $CDCl_3$) δ 7.38 (d, *J* = 7.8 Hz, 2H), 7.29 (t, *J* = 7.9 Hz, 2H), 7.21 (d, *J* = 2.3 Hz, 1H), 7.15 (dd, *J* = 8.3, 2.4 Hz, 1H), 7.09 (t, *J* = 7.4 Hz, 1H), 7.05 (br. s, 1H), 6.89 (d, *J* = 8.3 Hz, 1H), 6.57 (br. s, 1H), 2.69 (t, *J* = 7.7 Hz, 2H), 1.70 (s, 6H), 1.65 (quintet, *J* = 7.7 Hz, 2H), 1.41 (sextet, *J* = 7.6 Hz, 2H), 0.96 (t, *J* = 7.4 Hz, 3H); **^{13}C NMR** (126 MHz, $CDCl_3$) δ 177.1, 153.5, 137.7, 135.6, 129.7, 128.9, 128.5, 124.9, 124.4, 120.0, 115.7, 47.5, 32.0, 30.1, 27.2, 22.7, 14.0; **HRMS**: Calcd. for $C_{20}H_{25}NO_2$ $[M+Na]^+$ = 334.1777 m/z, found = 334.1793 m/z.

Compound S75:

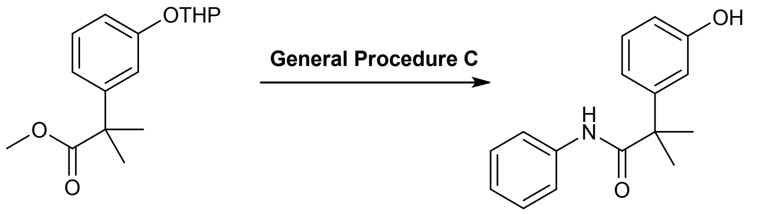

S23
Chemical Formula: $C_{16}H_{22}O_4$
Molecular Weight: 278.3435

S75
Chemical Formula: $C_{16}H_{17}NO_2$
Molecular Weight: 255.3117

Procedure: The reaction was carried out according to the General Procedure C on 10 mmol scale.

Amounts of Reagents:

S23 (10 mmol, 2.78 g)

aniline (15 mmol, 1.37 mL)

NaHMDS (20 mL, 1M solution in THF)

THF (50 mL, 0.2 M)

oxalic acid (20 mmol, 1.80 g)

MeOH/H_2O (90 mL/10 mL, 0.1 M)

Purification: 25% EtOAc in hexanes.

Yield of Product:

S75: 1.85 g, 7.25 mmol, 72% yield, white solid.

Characterization:

R_f = (EtOAc/Hexane 1:3): 0.27; **IR** (neat) ν = 3286, 2969, 1634, 1612, 1585, 1513, 1489, 1445, 1312, 1279, 1262, 1204, 1168, 1144 cm^{-1}; **^{1}H NMR** (500 MHz, $CDCl_3$) δ 7.37 (d, *J* = 7.6 Hz, 2H), 7.32 – 7.24 (m, 3H), 7.08 (t, *J* = 7.4 Hz, 1H), 7.02 (dd, *J* = 7.8, 0.9 Hz, 1H), 6.94 (t, *J* = 2.1 Hz, 1H), 6.90 (br. s, 1H), 6.83 (dd, *J* = 8.1, 2.5 Hz, 1H), 5.77 (br. s, 1H), 1.66 (s, 6H); **^{13}C NMR** (126 MHz, $CDCl_3$) δ 175.8, 156.4, 146.3, 137.7, 130.2, 128.9, 124.4, 119.9, 118.4, 114.6, 113.7, 48.0, 26.9; **GC-MS** R_t = 25.11 min, m/z = 255.1, 136.1, 121.1, 107.0, 93.1, 77.1, 65.1.

vi. Substrates in Scheme 8

Compound 17:

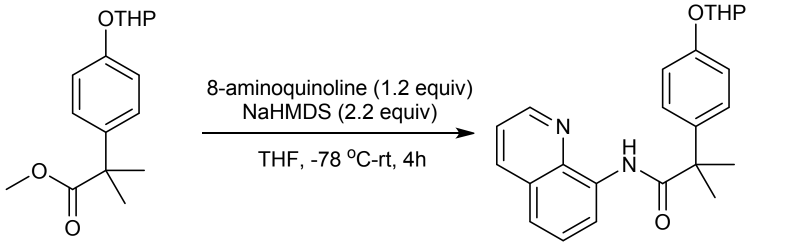

S8
Chemical Formula: $C_{16}H_{22}O_4$
Molecular Weight: 278.3435

17
Chemical Formula: $C_{24}H_{26}N_2O_3$
Molecular Weight: 390.4748

Procedure: The following procedure is based on the procedure of Wang.[21] A flame-dried, 100 mL round-bottom flask equipped with a Teflon-coated stir bar and a rubber septum was charged with 8-aminoquinoline (1.73 g, 12 mmol, 1.2 equiv) and then purged with a steady stream of N_2 for 5 min, prior to the addition of dry and degassed THF (20 mL). Sodium bis(trimethylsilyl)amide (NaHMDS) (22 mmol, 22 mL, 1.0 M in THF, 2.2 equiv) was added dropwise at -78 °C and stirred for 20 min. A separate, flame-dried, 50 mL round-bottom flask equipped with a Teflon-coated stir bar and a rubber septum was charged with **S8** (2.78 g, 10 mmol, 1.0 equiv) and then purged with a steady stream of N_2 for 5 min, prior to the addition of dry and degassed THF (10 mL) to afford a homogeneous solution. This solution was then added to the

[21] Wang, J.; Rosingana, M.; Discordia, R. P.; Soundararajan, N.; Polniaszek, R. *SynLett* **2001**, *9*, 1485-1487.

solution of the aniline and NaHMDS in THF dropwise via syringe at -78 ^oC. The mixture was then warmed to room temperature and stirred for 4h, quenched with saturated ammonium chloride (40 mL) and diluted with ethyl acetate (40 mL). The aqueous layer was separated and extracted with ethyl acetate (2 × 40 mL), and the combined organic layers were dried over $MgSO_4$, filtered and concentrated *in vacuo*. The crude product was purified on silica gel using 15% ethyl acetate in hexanes as eluent to afford **17** (3.48 g, 8.9 mmol, 89% yield) as a slightly brown solid.

Characterization:

R*f* = (EtOAc/Hexane 1:5): 0.42; **IR** (neat) ν = 3367, 3343, 2929, 2877, 1668, 1527, 1509, 1477, 1422, 1385, 1325, 1233, 1179, 1140, 1109 cm^{-1}; **^{1}H NMR** (500 MHz, Acetone-d_6) δ 9.85 (br. s, 1H), 8.77 (dd, *J* = 7.1, 1.9 Hz, 1H), 8.70 (dd, *J* = 4.2, 1.6 Hz, 1H), 8.30 (dd, *J* = 8.3, 1.6 Hz, 1H), 7.61 – 7.54 (m, 2H), 7.52 (dd, *J* = 8.3, 4.2 Hz, 1H), 7.48 (d, *J* = 8.9 Hz, 2H), 7.10 (d, *J* = 8.9 Hz, 2H), 5.47 (t, *J* = 3.3 Hz, 1H), 3.86 (ddd, *J* = 11.4, 9.5, 3.1 Hz, 1H), 3.57 (dtd, *J* = 9.7, 4.2, 0.8 Hz, 1H), 2.01 – 1.93 (m, 1H), 1.88 – 1.77 (m, 2H), 1.72 (s, 6H), 1.70 – 1.62 (m, 2H), 1.60 – 1.54 (m, 1H); **^{13}C NMR** (126 MHz, Acetone-d_6) δ 175.1, 156.2, 148.5, 138.3, 137.9, 136.4, 134.9, 128.0, 127.2, 127.1, 121.9, 121.2, 116.6, 115.2, 96.2, 61.5, 47.5, 30.2, 26.6, 26.5, 25.1, 18.7; **HRMS**: Calcd. for $C_{24}H_{26}N_2O_3$ $[M+Na]^+$ = 413.1836 m/z, found = 413.1843 m/z.

Compound 20:

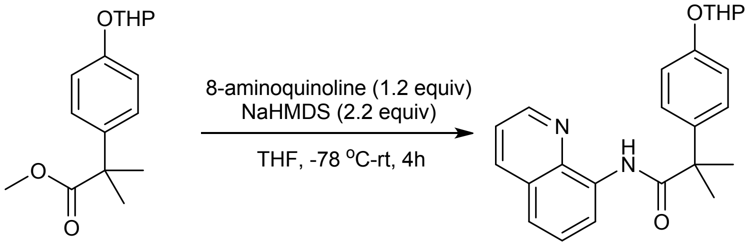

S8

Chemical Formula: $C_{16}H_{22}O_4$
Molecular Weight: 278.3435

20

Chemical Formula: $C_{24}H_{26}N_2O_3$
Molecular Weight: 390.4748

Procedure: A flame-dried, 50 mL round-bottom flask equipped with a Teflon-coated stir bar and a rubber septum was charged with compound **P1** (1.28 g, 5 mmol, 1.0 equiv) and K_2CO_3 (2.07 g, 15 mmol, 3.0 equiv) and then purged with a steady stream of N_2 for 5 min, prior to the addition of dry and degassed DMF (10 mL). Allyl bromide (519.3 μL, 6 mmol, 1.2 equiv) was added dropwise at rt and stirred for 12h. The mixture was then poured on water (40 mL) and extracted with ethyl acetate (3 × 40 mL), and the combined organic layers were washed with water (100 mL) and dried over $MgSO_4$, filtered and concentrated *in vacuo*. The crude product was purified on silica gel using 10% ethyl acetate in hexanes as eluent to afford **20** (1.30 g, 4.40 mmol, 88% yield) as a white solid.

Characterization:

R*f* = (EtOAc/Hexane 1:5): 0.41; **IR** (neat) ν = 3334, 2988, 2893, 1658, 1600, 1536, 1510, 1492, 1436, 1320, 1293, 1241, 1187, 1143 cm^{-1}; **^{1}H NMR** (500 MHz, $CDCl_3$) δ 7.44 – 7.32 (m, 4H), 7.28 (t, *J* = 8.0 Hz, 2H), 7.08 (t, *J* = 7.4 Hz, 1H), 6.97 (d, *J* = 8.8 Hz, 2H), 6.84 (s, 1H), 6.10 (ddt, *J* = 17.2, 10.6, 5.3 Hz, 1H), 5.46 (ddd, *J* = 17.3, 3.1, 1.5 Hz, 1H), 5.34 (ddd, *J* = 10.5, 2.6, 1.3 Hz, 1H), 4.59 (dt, *J* = 5.3, 1.4 Hz, 2H), 1.67 (s, 6H); **^{13}C NMR** (126 MHz, $CDCl_3$) δ 175.9, 157.8, 138.0, 136.6, 133.1, 128.9, 127.7, 124.1 119.5, 117.9, 115.1, 68.9, 47.4, 27.2; **HRMS**: Calcd. for $C_{19}H_{21}NO_2$ $[M+Na]^+$ = 318.1464 m/z, found = 318.1469 m/z.

7. X-ray Crystallography

X-ray diffraction data were collected at 150 K on a Bruker APEX DUO at Mo Kα (λ = 0.71073 Å) or Cu Kα (λ = 1.54178 Å, IμS microfocus source) radiation. The frames were integrated with the Bruker SAINT software package using a narrow-frame algorithm. The data were corrected for absorption effects using the multi-scan method (SADABS). The structure was solved and refined using the Bruker SHELXTL Software Package. The final anisotropic full-matrix least-squares refinement is on F^2. Details are provided in the respective CIF files.

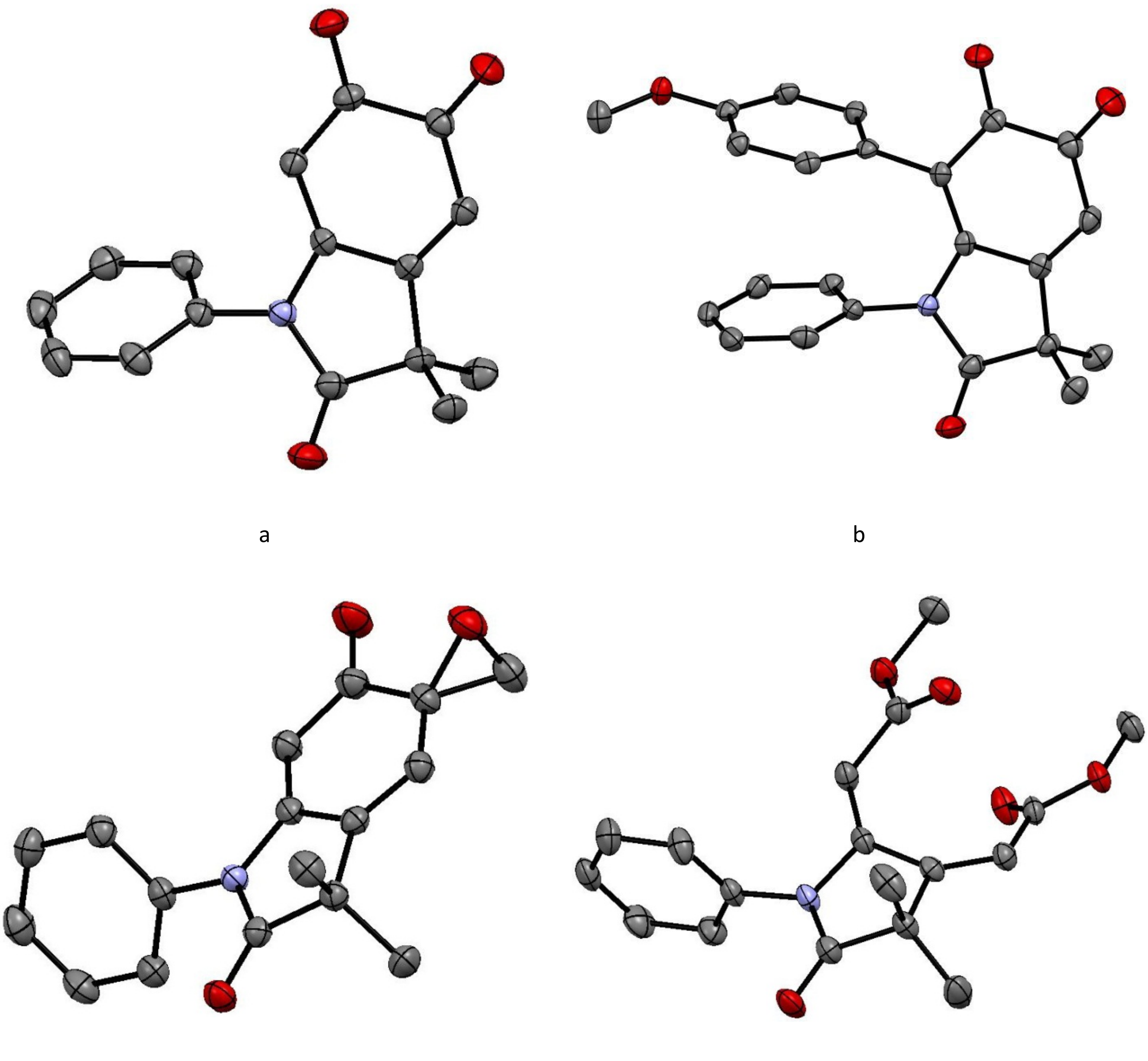

a b

c d

Figure S10. ORTEP representation at 50% ellipsoid probability for the X-ray crystal structures of a) **Compound Q3**, b) **Compound 13**, c) **Compound 23**, and d) **Compound 29**. Hydrogen atoms were removed for clarity.

Table S6. Crystallographic data for compounds in Figure S10 and Figure 2.

**Compound**	**Q3**	**13**	**23**	**29**	**$(SQ3)_2(SbF_6)_2$•2.5CH_2Cl_2**
CCDC deposition number	1406066	1406069	1406067	1406068	1421991
Empirical formula	$C_{16}H_{13}NO_3$	$C_{23}H_{19}NO_4$	$C_{17}H_{15}NO_3$	$C_{18}H_{19}NO_5$	$C_{54.5}H_{79}N_6O_6F_{12}Cl_5Cu_2Sb_2$
Formula weight	267.27	373.39	281.30	329.34	1690.06
T (K)	150(2)	150(2)	150(2)	150(2)	150(2)
Wavelength (Å)	0.71073	0.71073	1.54178	0.71073	0.71073
Crystal system	Orthorhombic	Orthorhombic	Monoclinic	Monoclinic	Triclinic
Space group	Pbca	$P2_12_12_1$	$P2_1/c$	$P2_1$	P-1
Unit cell dimensions					
a (Å)	7.2317(5)	7.6287(9)	5.792(3)	11.1595(11)	13.668(6)
b (Å)	11.2958(7)	11.5801(13)	23.556(9)	6.7253(7)	15.095(6)
c (Å)	32.232(2)	20.615(2)	11.041(5)	11.4275(12)	19.109(8)
α (°)	90	90	90	90	79.735(4)
β (°)	90	90	113.83(3)	103.4130(10)	89.183(4)
γ (°)	90	90	90	90	63.926(4)
V (Å^3)	2633.0(3)	1821.2(4)	1378.0(11)	834.25(15)	3475(2)
Z	8	4	4	2	2
D_{calc}	1.348	1.362	1.356	1.311	1.615
Absorption coefficient	0.094	0.094	0.761	0.096	1.646
F(000)	1120	784	592	348	1698
Crystal size (mm)	0.54 x 0.33 x 0.29	0.61 x 0.16 x 0.13	0.25 x 0.13 x 0.12	0.62 x 0.46 x 0.36	0.07 x 0.16 x 0.79
ϑ (°)	2.53 – 27.47	2.65 – 25.07	3.753 – 68.056	2.30 – 27.55	2.61 – 25.57
Index ranges	*h* = -9→9<br>*k* = -14→14<br>*l* = -41→41	*h* = -9→9<br>*k* = -15→14<br>*l* = -26→26	*h* = -6→6<br>*k* = -27→28<br>*l* = -13→13	*h* = -14→14<br>*k* = -8→8<br>*l* = -14→14	*h* = -17→17<br>*k* = -19→19<br>*l* = -24→24
Reflections collected	28779	21640	19027	9793	37455
Independent reflections	3046	4257	2508	3808	15600
Completeness (ϑ)	99.9%	99.8%	100%	99.7%	97.8%
Data/restraints/parameters	3046/0/183	4257/0/256	2508/0/193	3808/1/221	4257/18/847
Goodness of fit (GOF) on F^2	1.027	1.059	1.070	1.033	1.000
Final R indices [$I > 2\sigma(I)$] (%)	R_1 = 3.71<br>wR_2 = 9.43	R_1 = 3.91<br>wR_2 = 7.65	R_1 = 3.92<br>wR_2 = 10.06	R_1 = 3.34<br>wR_2 = 8.49	R_1 = 5.67<br>wR_2 = 12.13
R indices (all data) (%)	R_1 = 4.37<br>wR_2 = 9.90	R_1 = 5.65<br>wR_2 = 8.29	R_1 = 4.58<br>wR_2 = 10.56	R_1 = 3.59<br>wR_2 = 8.72	R_1 = 12.75<br>wR_2 = 14.84
Largest difference in peak and hole (e Å^{-3})	0.312 and -0.188	0.151 and -0.177	0.420 and -0.215	0.246 and -0.143	1.267 and -1.275
